# Correction: Evolutionary history of Carnivora (Mammalia, Laurasiatheria) inferred from mitochondrial genomes

**DOI:** 10.1371/journal.pone.0249387

**Published:** 2021-03-29

**Authors:** Alexandre Hassanin, Géraldine Veron, Anne Ropiquet, Bettine Jansen van Vuuren, Alexis Lécu, Steven M. Goodman, Jibran Haider, Trung Thanh Nguyen

Figs 2 and 3 in the original article [1] are incorrect. Figs 2A, 2B, 3A, and 3B respectively should have been individual figures, rather than two consolidated figures. The authors have provided correct versions of Fig 2A, 2B, 3A, and 3B as new figures below. Figs 5 and 6 correspond with the originally published Fig 2, and Figs 7 and 8 corresponds with the originally published Fig 3. Please view Figs 5–8 here.

**Fig 5:**
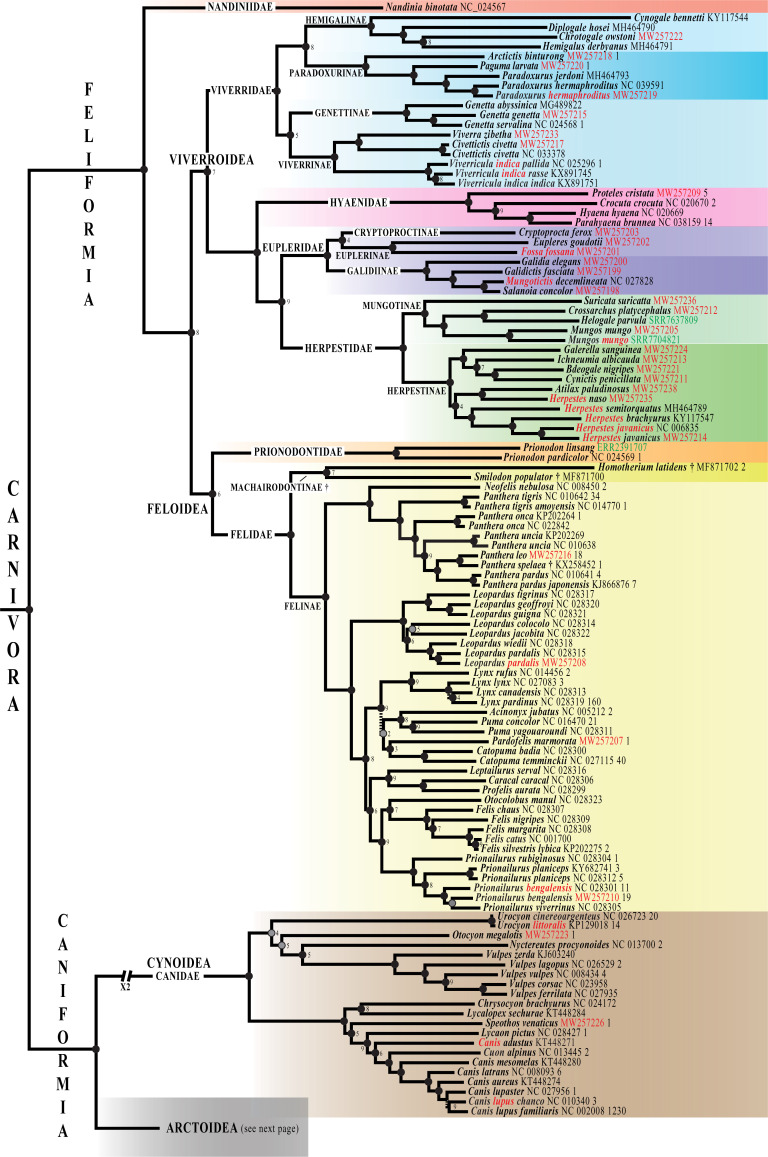
Phylogeny of Carnivora based on mitogenomes. The Bayesian tree was reconstructed using the *mtDNA* dataset (220 taxa and 14,892 bp) and GTR+I+G model. The two outgroup species are not shown. Species names follow the classification of the IUCN [1]; the taxa written in red highlight the taxonomic issues discussed in the main text. The accession numbers of the 42 mitogenomes of Carnivora specially sequenced for this study are indicated in red. The eight mitogenomes here assembled from SRA data are shown in green. The blue circle associated to *Mustela nudipes* MH464792 indicates that the mitogenome was originally misassigned to *Viverra tangalunga*. Fossil species are followed by the symbol “†”. For each terminal taxon, the number of similar mitogenome(s) found in GenBank (pairwise distance < 1%) is indicated after the accession number. Dash branches indicate nodes supported by posterior probability (PP) < 0.95. Black circles indicate nodes that are also monophyletic in the two following trees: SuperTRI bootstrap 50% majority-rule consensus tree; and Bayesian tree obtained from the analysis of the *mtDNA-Tv* dataset and JC69+I+G model. Grey circles show nodes that are not found to be monophyletic with one of the two methods detailed above. White circles indicate nodes that are not monophyletic in both *mtDNA-Tv* and SuperTRI bootstrap consensus trees. No information was provided for the nodes highly supported by the SuperTRI analyses, i.e. which were found monophyletic in all the 10 Bayesian trees reconstructed from the 10 half-overlapping sub-datasets of the *mtDNA* dataset. For nodes less supported by the SuperTRI analyses, the number of Bayesian trees (< 10) showing the nodes is indicated.

**Fig 6:**
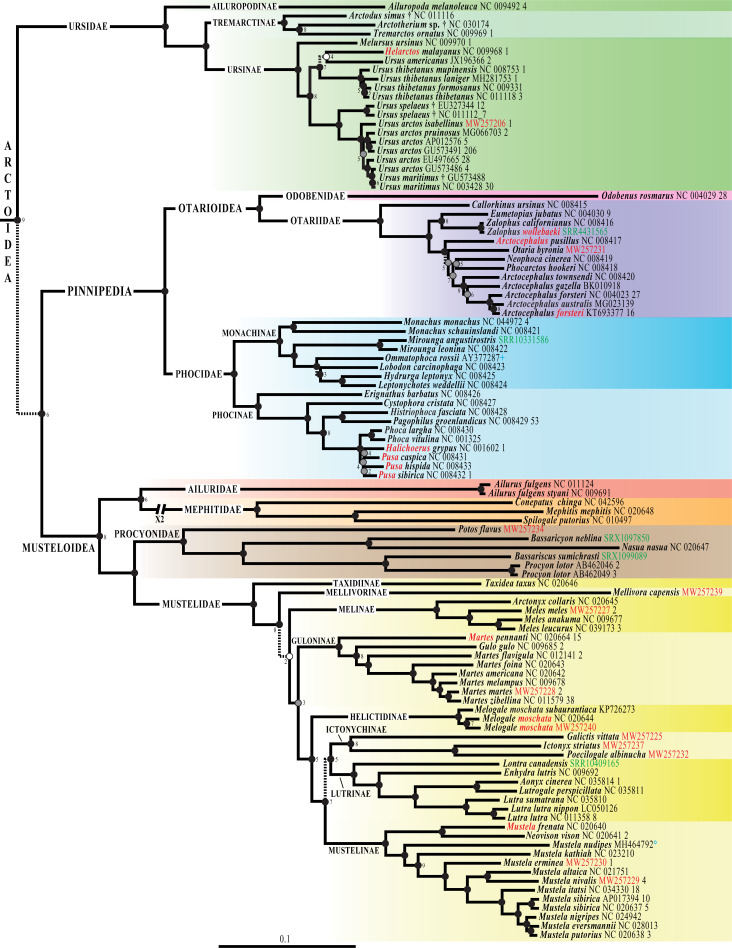
Phylogeny of Carnivora based on mitogenomes. The Bayesian tree was reconstructed using the *mtDNA* dataset (220 taxa and 14,892 bp) and GTR+I+G model. The two outgroup species are not shown. Species names follow the classification of the IUCN [1]; the taxa written in red highlight the taxonomic issues discussed in the main text. The accession numbers of the 42 mitogenomes of Carnivora specially sequenced for this study are indicated in red. The eight mitogenomes here assembled from SRA data are shown in green. The blue circle associated to *Mustela nudipes* MH464792 indicates that the mitogenome was originally misassigned to *Viverra tangalunga*. Fossil species are followed by the symbol “†”. For each terminal taxon, the number of similar mitogenome(s) found in GenBank (pairwise distance < 1%) is indicated after the accession number. Dash branches indicate nodes supported by posterior probability (PP) < 0.95. Black circles indicate nodes that are also monophyletic in the two following trees: SuperTRI bootstrap 50% majority-rule consensus tree; and Bayesian tree obtained from the analysis of the *mtDNA-Tv* dataset and JC69+I+G model. Grey circles show nodes that are not found to be monophyletic with one of the two methods detailed above. White circles indicate nodes that are not monophyletic in both *mtDNA-Tv* and SuperTRI bootstrap consensus trees. No information was provided for the nodes highly supported by the SuperTRI analyses, i.e. which were found monophyletic in all the 10 Bayesian trees reconstructed from the 10 half-overlapping sub-datasets of the *mtDNA* dataset. For nodes less supported by the SuperTRI analyses, the number of Bayesian trees (< 10) showing the nodes is indicated.

**Fig 7:**
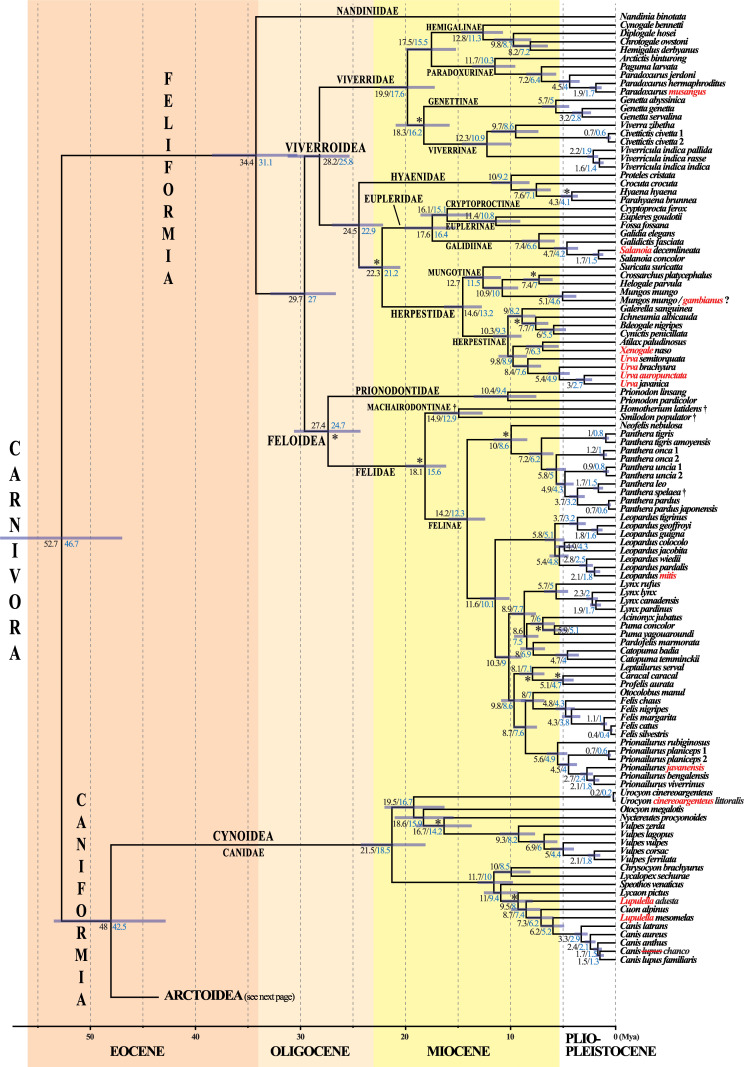
A molecular timescale for carnivoran evolution. The estimates of divergence time were calculated under BEAST v.2.4.7 using the GTR+I+G model on the *mtDNA* dataset. The asterisks show the 21 fossil calibration points used for molecular estimation (see Table 1 for details). The chronogram, mean ages (values in black) and associated 95% confidence intervals (grey bars) were inferred using a uniform prior distribution for fossil calibration points (“U approach”). For comparison, the values in blue are mean ages estimated using a log normal prior distribution for fossil calibration points (“L approach”; see main text for details and discussion). Species names follow the classification of the IUCN [1]; the name of the taxa written in red have been changed following our results which suggest these taxonomic changes (see the discussion for details).

**Fig 8:**
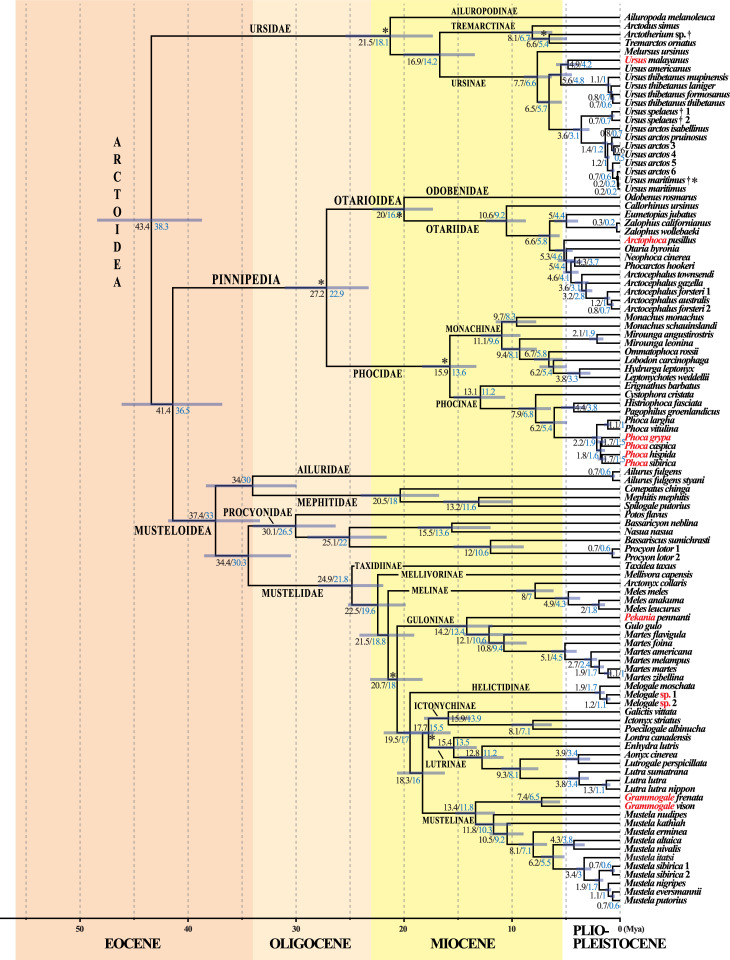
A molecular timescale for carnivoran evolution. The estimates of divergence time were calculated under BEAST v.2.4.7 using the GTR+I+G model on the *mtDNA* dataset. The asterisks show the 21 fossil calibration points used for molecular estimation (see Table 1 for details). The chronogram, mean ages (values in black) and associated 95% confidence intervals (grey bars) were inferred using a uniform prior distribution for fossil calibration points (“U approach”). For comparison, the values in blue are mean ages estimated using a log normal prior distribution for fossil calibration points (“L approach”; see main text for details and discussion). Species names follow the classification of the IUCN [1]; the name of the taxa written in red have been changed following our results which suggest these taxonomic changes (see the discussion for details).
