# Supplementary material for: Evolutionary history of Carnivora (Mammalia, Laurasiatheria) inferred from mitochondrial genomes
Source: PLoS One. 2021 Feb 16;16(2):e0240770. doi: 10.1371/journal.pone.0240770 (PMC7886153; doi:10.1371/journal.pone.0240770)
Supplement: S1 Appendix — (PDF) [file pone.0240770.s001.pdf]

S1 Appendix. Origin of the sequences used in this study (taxon names follow the classification of the IUCN, 2020)

Evolutionary history of Carnivora (Mammalia, Laurasiatheria) inferred from mitochondrial genomes

Alexandre Hassanin[1*], Géraldine Veron[1], Anne Ropiquet[2], Bettine Jansen van Vuuren[3], Alexis Lécu[4], Steven M. Goodman[5], Jibran Haider[1,6,7], Trung Thanh Nguyen[1]

[1] Institut de Systématique, Évolution, Biodiversité (ISYEB), Sorbonne Université, MNHN, CNRS, EPHE, UA, Paris.

[2] Department of Natural Sciences, Faculty of Science and Technology, Middlesex University, United Kingdom.

[3] Centre for Ecological Genomics and Wildlife Conservation, Department of Zoology, University of Johannesburg, South Africa.

[4] Parc zoologique de Paris, Muséum national d'Histoire naturelle, Paris.

[5] Field Museum of Natural History, Chicago, IL, USA.

[6] Department of Wildlife Management, Pir Mehr Ali Shah, Arid Agriculture University Rawalpindi, Pakistan.

[7] Forest Parks & Wildlife Department Gilgit-Baltistan, Pakistan.

* Correspondence: alexandre.hassanin@mnhn.fr

Clade	Family	Subfamily	Genus	Species	subspecies	Voucher	DNA sequencing	Accession number	Number of genomes
OUTGROUP	Tapiridae		*Tapirus*	*terrestris*		T358 = # 4020 Zoo de La Palmyre	Sanger (overlapping PCRs)	MW257204	1
OUTGROUP	Manidae		*Phataginus*	*tricuspis*				NC_026780	1
Feliformia	Felidae	Felinae	*Acinonyx*	*jubatus*				NC_005212	3
Caniformia	Ursidae	Ailuropodinae	*Ailuropoda*	*melanoleuca*				NC_009492	5
Caniformia	Ailuridae		*Ailurus*	*fulgens*				NC_011124	1
Caniformia	Ailuridae		*Ailurus*	*fulgens*	*styani*			NC_009691	1
Caniformia	Mustelidae	Lutrinae	*Aonyx*	*cinerea*				NC_035814	2
Feliformia	Viverridae	Paradoxurinae	*Arctictis*	*binturong*		T605 = MNHN TC28; NMS RL78/97	Sanger (overlapping PCRs)	MW257218	2
Caniformia	Otariidae		*Arctocephalus*	*australis*				MG023139	1
Caniformia	Otariidae		*Arctocephalus*	*forsteri*				KT693377	17
Caniformia	Otariidae		*Arctocephalus*	*forsteri*				NC_004023	28
Caniformia	Otariidae		*Arctocephalus*	*gazella*				BK010918	1
Caniformia	Otariidae		*Arctocephalus*	*pusillus*				NC_008417	1
Caniformia	Otariidae		*Arctocephalus*	*townsendi*				NC_008420	1
Caniformia	Ursidae	Tremarctinae	*Arctodus*	*simus*				NC_011116	1
Caniformia	Mustelidae	Melinae	*Arctonyx*	*collaris*				NC_020645	1
Caniformia	Ursidae	Tremarctinae	*Arctotherium*	sp.				NC_030174	1
Feliformia	Herpestidae	Herpestinae	*Atilax*	*paludinosus*		T606 = MNHN TC59	Sanger (overlapping PCRs)	MW257238	1
Caniformia	Procyonidae		*Bassaricyon*	*neblina*				SRX1097850*	1
Caniformia	Procyonidae		*Bassariscus*	*sumichrasti*				SRX1099089*	1
Feliformia	Herpestidae	Herpestinae	*Bdeogale*	*nigripes*		GLC15, Gabon	Sanger (overlapping PCRs)	MW257221	1
Caniformia	Otariidae		*Callorhinus*	*ursinus*				NC_008415	1
Caniformia	Canidae		*Canis*	*adustus*				KT448271	1
Caniformia	Canidae		*Canis*	*anthus*				NC_027956	2
Caniformia	Canidae		*Canis*	*aureus*				KT448274	1
Caniformia	Canidae		*Canis*	*latrans*				NC_008093	7
Caniformia	Canidae		*Canis*	*lupus*	*chanco*			NC_010340	4
Caniformia	Canidae		*Canis*	*lupus*	*familiaris*			NC_002008	1231
Caniformia	Canidae		*Canis*	*mesomelas*				KT448280	1
Feliformia	Felidae	Felinae	*Caracal*	*caracal*				NC_028306	1
Feliformia	Felidae	Felinae	*Catopuma*	*badia*				NC_028300	1
Feliformia	Felidae	Felinae	*Catopuma*	*temminckii*				NC_027115	41
Feliformia	Viverridae	Hemigalinae	*Chrotogale*	*owstoni*		T607 = MNHN SH4	Sanger (overlapping PCRs)	MW257222	1
Caniformia	Canidae		*Chrysocyon*	*brachyurus*				NC_024172	1
Feliformia	Viverridae	Viverrinae	*Civettictis*	*civetta*		GLC19, Gabon	Sanger (overlapping PCRs)	MW257217	1
Feliformia	Viverridae	Viverrinae	*Civettictis*	*civetta*				NC_033378	1
Caniformia	Mephitidae		*Conepatus*	*chinga*				NC_042596	1
Feliformia	Hyaenidae		*Crocuta*	*crocuta*				NC_020670	3
Feliformia	Herpestidae	Mungotinae	*Crossarchus*	*platycephalus*		C7R66, Cameroon	Sanger (overlapping PCRs)	MW257212	1
Feliformia	Eupleridae	Euplerinae	*Cryptoprocta*	*ferox*		MNHN C13; Univ Montpellier T-832; Studbook # 0008	Sanger (overlapping PCRs)	MW257203	1
Caniformia	Canidae		*Cuon*	*alpinus*				NC_013445	3
Feliformia	Herpestidae	Herpestinae	*Cynictis*	*penicillata*		T375 = SUN-227 Botswana	Sanger (overlapping PCRs)	MW257211	1
Feliformia	Viverridae	Hemigalinae	*Cynogale*	*bennetti*				KY117544	1
Caniformia	Phocidae		*Cystophora*	*cristata*				NC_008427	1
Feliformia	Viverridae	Hemigalinae	*Diplogale*	*hosei*				MH464790	1
Caniformia	Mustelidae	Lutrinae	*Enhydra*	*lutris*				NC_009692	1
Caniformia	Phocidae		*Erignathus*	*barbatus*				NC_008426	1
Caniformia	Otariidae		*Eumetopias*	*jubatus*				NC_004030	10
Feliformia	Eupleridae	Euplerinae	*Eupleres*	*goudotii*		D128; MNHN TC705; RHT062; UADBA 50125 Madagascar	Sanger (overlapping PCRs)	MW257202	1
Feliformia	Felidae	Felinae	*Felis*	*catus*				NC_001700	1
Feliformia	Felidae	Felinae	*Felis*	*chaus*				NC_028307	1
Feliformia	Felidae	Felinae	*Felis*	*margarita*				NC_028308	1
Feliformia	Felidae	Felinae	*Felis*	*nigripes*				NC_028309	1
Feliformia	Felidae	Felinae	*Felis*	*silvestris*	*lybica*			KP202275	3
Feliformia	Eupleridae	Euplerinae	*Fossa*	*fossana*		D350; FMNH 171893 Madagascar	Illumina (shotgun) MRD = 16	MW257201	1
Feliformia	Herpestidae	Herpestinae	*Galerella*	*sanguinea*		T378 = SUN-272 South Africa	Sanger (overlapping PCRs)	MW257224	1
Caniformia	Mustelidae	Galictinae	*Galictis*	*vittata*		T412 = TCCV 1997.137 French Guiana	Sanger (overlapping PCRs)	MW257225	1
Feliformia	Eupleridae	Galidiinae	*Galidia*	*elegans*		D146; FMNH 173102 Madagascar	Illumina (shotgun) MRD = 139	MW257200	1
Feliformia	Eupleridae	Galidiinae	*Galidictis*	*fasciata*		D333; FMNH 156652 Madagascar	Illumina (shotgun) MRD = 124	MW257199	1
Feliformia	Viverridae	Viverrinae	*Genetta*	*abyssinica*				MG489822	1
Feliformia	Viverridae	Viverrinae	*Genetta*	*genetta*		T297 = MNHN SPOT-1479=MNHN-ZM-2004-298, France	Sanger (overlapping PCRs)	MW257215	1
Feliformia	Viverridae	Viverrinae	*Genetta*	*servalina*				NC_024568	2
Caniformia	Mustelidae	Martinae	*Gulo*	*gulo*				NC_009685	3
Caniformia	Phocidae		*Halichoerus*	*grypus*				NC_001602	2
Caniformia	Ursidae	Ursinae	*Helarctos*	*malayanus*				NC_009968	2
Feliformia	Herpestidae	Mungotinae	*Helogale*	*parvula*				SRR7637809*	1
Feliformia	Viverridae	Hemigalinae	*Hemigalus*	*derbyanus*				MH464791	1
Feliformia	Herpestidae	Herpestinae	*Herpestes*	*brachyurus*				KY117547	1
Feliformia	Herpestidae	Herpestinae	*Herpestes*	*javanicus*				NC_006835	1
Feliformia	Herpestidae	Herpestinae	*Herpestes*	*javanicus*		T413 = MNHN VV 1999.510, Thailand	Sanger (overlapping PCRs)	MW257214	1
Feliformia	Herpestidae	Herpestinae	*Herpestes*	*naso*		C7R110 Cameroon	Sanger (overlapping PCRs)	MW257235	1
Feliformia	Herpestidae	Herpestinae	*Herpestes*	*semitorquatus*				MH464789	1
Feliformia	Felidae	Machairodontina	*Homotherium*	*latidens*				MF871702	3
Feliformia	Hyaenidae		*Hyaena*	*hyaena*				NC_020669	1
Caniformia	Phocidae		*Hydrurga*	*leptonyx*				NC_008425	1
Feliformia	Herpestidae	Herpestinae	*Ichneumia*	*albicauda*		T603 = DM1100 TC-157, South Africa	Sanger (overlapping PCRs)	MW257213	1
Caniformia	Mustelidae	Galictinae	*Ictonyx*	*striatus*		T299 = MNHN SPOT-6901 South Africa	Sanger (overlapping PCRs)	MW257237	1
Feliformia	Felidae	Felinae	*Leopardus*	*colocolo*				NC_028314	1
Feliformia	Felidae	Felinae	*Leopardus*	*geoffroyi*				NC_028320	1
Feliformia	Felidae	Felinae	*Leopardus*	*guigna*				NC_028321	1
Feliformia	Felidae	Felinae	*Leopardus*	*jacobita*				NC_028322	1
Feliformia	Felidae	Felinae	*Leopardus*	*pardalis*				NC_028315	1
Feliformia	Felidae	Felinae	*Leopardus*	*pardalis*	*mitis*	T262 = MNHN SPOT-4119 French Guiana	Sanger (overlapping PCRs)	MW257208	1
Feliformia	Felidae	Felinae	*Leopardus*	*tigrinus*				NC_028317	1
Feliformia	Felidae	Felinae	*Leopardus*	*wiedii*				NC_028318	1
Feliformia	Felidae	Felinae	*Leptailurus*	*serval*				NC_028316	1
Caniformia	Phocidae		*Leptonychotes*	*weddellii*				NC_008424	1
Caniformia	Phocidae		*Lobodon*	*carcinophaga*				NC_008423	1
Caniformia	Mustelidae	Lutrinae	*Lontra*	*canadensis*				SRR10409165*	1
Caniformia	Mustelidae	Lutrinae	*Lutra*	*lutra*				LC050126	1
Caniformia	Mustelidae	Lutrinae	*Lutra*	*lutra*				NC_011358	9
Caniformia	Mustelidae	Lutrinae	*Lutra*	*sumatrana*				NC_035810	1
Caniformia	Mustelidae	Lutrinae	*Lutrogale*	*perspicillata*				NC_035811	1
Caniformia	Canidae		*Lycalopex*	*sechurae*				KT448284	1
Caniformia	Canidae		*Lycaon*	*pictus*				NC_028427	2
Feliformia	Felidae	Felinae	*Lynx*	*canadensis*				NC_028313	1
Feliformia	Felidae	Felinae	*Lynx*	*lynx*				NC_027083	4
Feliformia	Felidae	Felinae	*Lynx*	*pardinus*				NC_028319	161
Feliformia	Felidae	Felinae	*Lynx*	*rufus*				NC_014456	3
Caniformia	Mustelidae	Martinae	*Martes*	*americana*				NC_020642	1
Caniformia	Mustelidae	Martinae	*Martes*	*flavigula*				NC_012141	3
Caniformia	Mustelidae	Martinae	*Martes*	*foina*				NC_020643	1
Caniformia	Mustelidae	Martinae	*Martes*	*martes*		T302 = MNHN SPOT-1051 Fance	Sanger (overlapping PCRs)	MW257228	3
Caniformia	Mustelidae	Martinae	*Martes*	*melampus*				NC_009678	1
Caniformia	Mustelidae	Martinae	*Martes*	*pennanti*				NC_020664	16
Caniformia	Mustelidae	Martinae	*Martes*	*zibellina*				NC_011579	39
Caniformia	Mustelidae	Melinae	*Meles*	*anakuma*				NC_009677	1
Caniformia	Mustelidae	Melinae	*Meles*	*leucurus*				NC_039173	4
Caniformia	Mustelidae	Melinae	*Meles*	*meles*		T303 = MNHN SPOT-1533	Sanger (overlapping PCRs)	MW257227	3
Caniformia	Mustelidae	Mellivorinae	*Mellivora*	*capensis*		T370 = SUN-103 Tanzania	Sanger (overlapping PCRs)	MW257239	1
Caniformia	Mustelidae	Helictidinae	*Melogale*	*moschata*	*subaurantiaca*			KP726273	1
Caniformia	Mustelidae	Helictidinae	*Melogale*	*moschata*				NC_020644	1
Caniformia	Mustelidae	Helictidinae	*Melogale*	*moschata*		V7x35A Vietnam	Sanger (overlapping PCRs)	MW257240	1
Caniformia	Ursidae	Ursinae	*Melursus*	*ursinus*				NC_009970	2
Caniformia	Mephitidae		*Mephitis*	*mephitis*				NC_020648	1
Caniformia	Phocidae		*Mirounga*	*angustirostris*				SRR10331586*	1
Caniformia	Phocidae		*Mirounga*	*leonina*				NC_008422	1
Caniformia	Phocidae		*Monachus*	*monachus*				NC_044972	5

Caniformia	Phocidae		*Monachus*	*schauinslandi*				NC_008421	1
Feliformia	Herpestidae	Mungotinae	*Mungos*	*mungo*		MNHN C78, Univ. Montpellier T-1112	Sanger (overlapping PCRs)	MW257205	1
Feliformia	Herpestidae	Mungotinae	*Mungos*	*mungo*				SRR7704821*	1
Feliformia	Eupleridae	Galidiinae	*Mungotictis*	*decemlineata*				NC_027828	1
Caniformia	Mustelidae	Mustelinae	*Mustela*	*altaica*				NC_021751	1
Caniformia	Mustelidae	Mustelinae	*Mustela*	*erminea*		T305 = MNHN SPOT-1142	Sanger (overlapping PCRs)	MW257230	2
Caniformia	Mustelidae	Mustelinae	*Mustela*	*eversmannii*				NC_028013	1
Caniformia	Mustelidae	Mustelinae	*Mustela*	*frenata*				NC_020640	1
Caniformia	Mustelidae	Mustelinae	*Mustela*	*itatsi*				NC_034330	19
Caniformia	Mustelidae	Mustelinae	*Mustela*	*kathiah*				NC_023210	1
Caniformia	Mustelidae	Mustelinae	*Mustela*	*nigripes*				NC_024942	1
Caniformia	Mustelidae	Mustelinae	*Mustela*	*nivalis*		T306 = MNHN SPOT-2728, MNHN-ZM 2004-391, France	Sanger (overlapping PCRs)	MW257229	5
Caniformia	Mustelidae	Mustelinae	*Mustela*	*putorius*				NC_020638	4
Caniformia	Mustelidae	Mustelinae	*Mustela*	*sibirica*				AP017394	11
Caniformia	Mustelidae	Mustelinae	*Mustela*	*sibirica*				NC_020637	6
Feliformia	Nandiniidae		*Nandinia*	*binotata*				NC_024567	1
Caniformia	Procyonidae		*Nasua*	*nasua*				NC_020647	1
Feliformia	Felidae	Pantherinae	*Neofelis*	*nebulosa*				NC_008450	3
Caniformia	Phocidae		*Neophoca*	*cinerea*				NC_008419	1
Caniformia	Mustelidae	Mustelinae	*Neovison*	*vison*				NC_020641	3
Caniformia	Canidae		*Nyctereutes*	*procyonoides*				NC_013700	3
Caniformia	Odobenidae		*Odobenus*	*rosmarus*				NC_004029	29
Caniformia	Phocidae		*Ommatophoca*	*rossii*				RERL	1
Caniformia	Otariidae		*Otaria*	*byronia*		OTAB; Parc zoologique de Paris	Sanger (overlapping PCRs)	MW257231	1
Feliformia	Felidae	Felinae	*Otocolobus*	*manul*				NC_028323	1
Caniformia	Canidae		*Otocyon*	*megalotis*		SAF1 South Africa	Sanger (overlapping PCRs)	MW257223	2
Feliformia	Viverridae	Paradoxurinae	*Paguma*	*larvata*		PDDS11 Laos	Sanger (overlapping PCRs)	MW257220	2
Feliformia	Felidae	Pantherinae	*Panthera*	*leo*		NERO; Parc zoologique de Paris	Ion Torrent (overlapping PCRs) MRD = 211	MW257216	19
Feliformia	Felidae	Pantherinae	*Panthera*	*leo*	*spelaea*			KX258452	2
Feliformia	Felidae	Pantherinae	*Panthera*	*onca*				KP202264	2
Feliformia	Felidae	Pantherinae	*Panthera*	*onca*				NC_022842	1
Feliformia	Felidae	Pantherinae	*Panthera*	*pardus*				NC_010641	5
Feliformia	Felidae	Pantherinae	*Panthera*	*pardus*	*japonensis*			KJ866876	8
Feliformia	Felidae	Pantherinae	*Panthera*	*tigris*				NC_010642	35
Feliformia	Felidae	Pantherinae	*Panthera*	*tigris*	*amoyensis*			NC_014770	2
Feliformia	Felidae	Pantherinae	*Panthera*	*uncia*				KP202269	1
Feliformia	Felidae	Pantherinae	*Panthera*	*uncia*				NC_010638	1
Feliformia	Viverridae	Paradoxurinae	*Paradoxurus*	*hermaphroditus*				NC_039591	1
Feliformia	Viverridae	Paradoxurinae	*Paradoxurus*	*hermaphroditus*		NLNC50 Laos	Sanger (overlapping PCRs)	MW257219	1
Feliformia	Viverridae	Paradoxurinae	*Paradoxurus*	*jerdoni*				MH464793	1
Feliformia	Hyaenidae		*Parahyaena*	*brunnea*				NC_038159	15
Feliformia	Felidae	Pantherinae	*Pardofelis*	*marmorata*		NLN3 Laos	Sanger (overlapping PCRs)	MW257207	2
Caniformia	Phocidae		*Phoca*	*fasciata*				NC_008428	1
Caniformia	Phocidae		*Phoca*	*groenlandica*				NC_008429	54
Caniformia	Phocidae		*Phoca*	*largha*				NC_008430	1
Caniformia	Phocidae		*Phoca*	*vitulina*				NC_001325	1
Caniformia	Otariidae		*Phocarctos*	*hookeri*				NC_008418	1
Caniformia	Mustelidae	Galictinae	*Poecilogale*	*albinucha*		T602 = FMNH 149354 Burundi	Sanger (overlapping PCRs)	MW257232	1
Caniformia	Procyonidae		*Potos*	*flavus*		T414 = TCCV 1995.125	Sanger (overlapping PCRs)	MW257234	1
Feliformia	Felidae	Felinae	*Prionailurus*	*bengalensis*		CKM45 Cambodia	Sanger (overlapping PCRs)	MW257210	20
Feliformia	Felidae	Felinae	*Prionailurus*	*bengalensis*	*javanensis*			NC_028301	12
Feliformia	Felidae	Felinae	*Prionailurus*	*planiceps*				KY682741	4
Feliformia	Felidae	Felinae	*Prionailurus*	*planiceps*				NC_028312	6
Feliformia	Felidae	Felinae	*Prionailurus*	*rubiginosus*				NC_028304	2
Feliformia	Felidae	Felinae	*Prionailurus*	*viverrinus*				NC_028305	1
Feliformia	Prionodontidae		*Prionodon*	*linsang*				ERR2391707*	1
Feliformia	Prionodontidae		*Prionodon*	*pardicolor*				NC_024569	2
Caniformia	Procyonidae		*Procyon*	*lotor*				AB462046	3
Caniformia	Procyonidae		*Procyon*	*lotor*				AB462049	4
Feliformia	Felidae	Felinae	*Profelis*	*aurata*				NC_028299	1
Feliformia	Hyaenidae		*Proteles*	*cristata*		T393 = SUN-1172 South Africa	Sanger (overlapping PCRs)	MW257209	6
Feliformia	Felidae	Felinae	*Puma*	*concolor*				NC_016470	22
Feliformia	Felidae	Felinae	*Puma*	*yagouaroundi*				NC_028311	1
Caniformia	Phocidae		*Pusa*	*caspica*				NC_008431	1
Caniformia	Phocidae		*Pusa*	*hispida*				NC_008433	1
Caniformia	Phocidae		*Pusa*	*sibirica*				NC_008432	2
Feliformia	Eupleridae	Galidiinae	*Salanoia*	*concolor*		D378; MNHN TC-696	Illumina (shotgun) MRD = 31	MW257198	1
Feliformia	Felidae	Machairodontina	*Smilodon*	*populator*				MF871700	1
Caniformia	Canidae		*Speothos*	*venaticus*		C48; MNHN TC-034	Sanger (overlapping PCRs)	MW257226	2
Caniformia	Mephitidae		*Spilogale*	*putorius*				NC_010497	1
Feliformia	Herpestidae	Mungotinae	*Suricata*	*suricatta*		SSM10 South Africa	Sanger (overlapping PCRs)	MW257236	1
Caniformia	Mustelidae	Taxidiinae	*Taxidea*	*taxus*				NC_020646	1
Caniformia	Ursidae	Tremarctinae	*Tremarctos*	*ornatus*				NC_009969	2
Caniformia	Canidae		*Urocyon*	*cinereoargenteus*				NC_026723	21
Caniformia	Canidae		*Urocyon*	*littoralis*	*catalinae*			KP129018	15
Caniformia	Ursidae	Ursinae	*Ursus*	*americanus*				JX196366	3
Caniformia	Ursidae	Ursinae	*Ursus*	*arctos*				AP012576	6
Caniformia	Ursidae	Ursinae	*Ursus*	*arctos*				EU497665	29
Caniformia	Ursidae	Ursinae	*Ursus*	*arctos*				GU573486	5
Caniformia	Ursidae	Ursinae	*Ursus*	*arctos*				GU573491	207
Caniformia	Ursidae	Ursinae	*Ursus*	*arctos*	*isabellinus*	18B5 Pakistan	Illumina (overlapping PCRs) MRD = 204	MW257206	2
Caniformia	Ursidae	Ursinae	*Ursus*	*arctos*	*pruinosus*			MG066703	3
Caniformia	Ursidae	Ursinae	*Ursus*	*maritimus*				GU573488	1
Caniformia	Ursidae	Ursinae	*Ursus*	*maritimus*				NC_003428	31
Caniformia	Ursidae	Ursinae	*Ursus*	*spelaeus*				EU327344	13
Caniformia	Ursidae	Ursinae	*Ursus*	*spelaeus*				NC_011112	8
Caniformia	Ursidae	Ursinae	*Ursus*	*thibetanus*	*formosanus*			NC_009331	1
Caniformia	Ursidae	Ursinae	*Ursus*	*thibetanus*	*laniger*			MH281753	2
Caniformia	Ursidae	Ursinae	*Ursus*	*thibetanus*	*mupinensis*			NC_008753	2
Caniformia	Ursidae	Ursinae	*Ursus*	*thibetanus*	*thibetanus*			NC_011118	4
Feliformia	Viverridae	Viverrinae	*Viverra*	*tangalunga*				MH464792	1
Feliformia	Viverridae	Viverrinae	*Viverra*	*zibetha*		T609 = MNHN SH22	Sanger (overlapping PCRs)	MW257233	1
Feliformia	Viverridae	Viverrinae	*Viverricula*	*indica*	*rasse*			KX891745	1
Feliformia	Viverridae	Viverrinae	*Viverricula*	*indica*	*indica*			KX891751	1
Feliformia	Viverridae	Viverrinae	*Viverricula*	*indica*	*pallida*			NC_025296	2
Caniformia	Canidae		*Vulpes*	*corsac*				NC_023958	1
Caniformia	Canidae		*Vulpes*	*ferrilata*				NC_027935	1
Caniformia	Canidae		*Vulpes*	*lagopus*				NC_026529	3
Caniformia	Canidae		*Vulpes*	*vulpes*				NC_008434	5
Caniformia	Canidae		*Vulpes*	*zerda*				KJ603240	1
Caniformia	Otariidae		*Zalophus*	*californianus*				NC_008416	1
Caniformia	Otariidae		*Zalophus*	*wollebaeki*				SRR4431565*	1
								**TOTAL**	**2442**

* = genomes extracted from SRA data

RERL = AY377132, AY377155, AY377178, AY377201, AY377224, AY377247, AY377269, AY377287, AY377294, AY377316, AY377322, AY377331, AY377349, AY377367, AY377385.

MRD: Mean read depth
