# Supplementary material for: Evolutionary history of Carnivora (Mammalia, Laurasiatheria) inferred from mitochondrial genomes
Source: PLoS One. 2021 Feb 16;16(2):e0240770. doi: 10.1371/journal.pone.0240770 (PMC7886153; doi:10.1371/journal.pone.0240770)
Supplement: S2 Appendix — (PDF) [file pone.0240770.s002.pdf]

S2 Appendix. PCR primers used in this study

Evolutionary history of Carnivora (Mammalia, Laurasiatheria) inferred from mitochondrial genomes

Alexandre Hassanin[1*], Géraldine Veron[1], Anne Ropiquet[2], Bettine Jansen van Vuuren[3],

Alexis Lécu[4], Steven M. Goodman[5], Jibran Haider[1,6,7], Trung Thanh Nguyen[1]

[1] Institut de Systématique, Évolution, Biodiversité (ISYEB), Sorbonne Université, MNHN, CNRS, EPHE, UA, Paris.

[2] Department of Natural Sciences, Faculty of Science and Technology, Middlesex University, United Kingdom.

[3] Centre for Ecological Genomics and Wildlife Conservation, Department of Zoology, University of Johannesburg, South Africa.

[4] Parc zoologique de Paris, Muséum national d'Histoire naturelle, Paris.

[5] Field Museum of Natural History, Chicago, IL, USA.

[6] Department of Wildlife Management, Pir Mehr Ali Shah, Arid Agriculture University Rawalpindi, Pakistan.

[7] Forest Parks & Wildlife Department Gilgit-Baltistan, Pakistan.

* Correspondence: alexandre.hassanin@mnhn.fr

DLU400 [2]<br>DLU400M1: 5'-CGAGCTTAATCACCAWGCCKCGDGAAA-3'<br>DLU405M1 [2]	LPHELA [3]<br>12SL41 [1]<br>12SL200 [2]<br>12SL600 [3]
12SU1230 [1]<br>12SU1230M1 [3]	12SL2226 [1]<br>12SL2226M1 [2]
12SU829 [1]	16SL518 [1]
16SU365 [1]	16SL1056 [1]
16SU946 [1]	N1L64 [1]
Uleu [1]<br>U16S1421 [2]	LMet [2]<br>LMet2 [2]
N1U840 [1]	N2L492 [1]
N2U354M2 [2]<br>IleU [2]	AsnL [1]
TrpU [1]	C1L705 [2]<br>C1L1143 [3]
C1U246 [1]<br>UTyr [2]	C1L1017 [1]<br>LSer [2]
C1U897M1 [2]	C2L15 [1]<br>C2L15M1 [2]
SerU [1]<br>SerUM1 [2]	A8L1 [1]
C2U603 [1]<br>C2U603M3 [2]<br>C2U603M4 [2]	C3L45 [1]<br>C3L45M1: 5'-AGRGCNGANAGRGCYCCTGT-3'<br>C3L168 [2]<br>C3L168M1 [3]<br>C3L168M2 [3]
A6U654M1 [2]<br>A6U654M2 [2]	GlyLM1 [2]
C3U780 [1]<br>C3U780M1 [2]<br>C3U780M3 [3]	N4L27M1 [2]<br>N4L366 [2]<br>N4L366M1 [3]<br>N4L366M2: 5'-CGYTCYGTYTGRTTNCCYCATCGRGT-3'
UArg [2]<br>UArgM1: 5'-TGATTTCGACTCATTAAA-3'	N4L918M1 [1]<br>N4L1071R [2]
N4U681 [2]	Leu2L [1]<br>Leu2LM1 [2]
Ser2U [1]	N5L652 [1]<br>N5L652M1 [3]
N5U501 [1]<br>N5U501M2: 5'-GGNCGRDCHGAYGCHAAYACNGCYGC-3'	N5L1214 [1]
N5U1146 [1]<br>N5U1146M5 [2]	N6RL154 [1]<br>N6RL154M5 [2]<br>CBL1 [3]
N6RU102 [1]	CBL402 [1]
GluMA [2]<br>GluCA: 5'-GACYARTGAYATGAAAAAYCAYCGTTGT-3'<br>CBU162 [1]	LPROMA: 5'-<br>AGAAYWTCAGCTTTGGGDGYTGAHGGTG-3'<br>LTHR [1]<br>L482 [1]<br>L482M1 [3]
U844 [1]<br>CBU1038M1 [3]<br>U1068 [1]<br>UTHR13 [3]	L482 [1]<br>LPHELA [3]

References

1. Hassanin A, Ropiquet A, Couloux A, Cruaud C. Evolution of the mitochondrial genome in mammals living at high altitude: new insights from a study of the tribe Caprini (Bovidae, Antilopinae). J Mol Evol. 2009; 68: 293-310. doi: 10.1007/s00239-009-9208-7.
2. Hassanin A, Delsuc F, Ropiquet A, Hammer C, Jansen van Vuuren B, Matthee C, et al. Pattern and timing of diversification of Cetartiodactyla (Mammalia, Laurasiatheria), as revealed by a comprehensive analysis of mitochondrial genomes. C R Biol. 2012; 335: 32-50. doi: 10.1016/j.crvi.2011.11.002.
3. Hassanin A, Bonillo C, Tshikung D, Pongombo Shongo C, Pourrut X, Kadjo B, et al. Phylogeny of African fruit bats (Chiroptera, Pteropodidae) based on complete mitochondrial genomes. J Zool Syst Evol Res. 2020; 58: 1395-1410. doi: 10.1111/jzs.12373.
