# Supplementary material for: Evolutionary history of Carnivora (Mammalia, Laurasiatheria) inferred from mitochondrial genomes
Source: PLoS One. 2021 Feb 16;16(2):e0240770. doi: 10.1371/journal.pone.0240770 (PMC7886153; doi:10.1371/journal.pone.0240770)
Supplement: S3 Appendix — (PDF) [file pone.0240770.s003.pdf]

S3 Appendix. Base composition

Evolutionary history of Carnivora (Mammalia, Laurasiatheria) inferred from mitochondrial genomes

Alexandre Hassanin[1*], Géraldine Veron[1], Anne Ropiquet[2], Bettine Jansen van Vuuren[3], Alexis Lécu[4], Steven M. Goodman[5], Jibran Haider[1,6,7], Trung Thanh Nguyen[1]

[1] Institut de Systématique, Évolution, Biodiversité (ISYEB), Sorbonne Université, MNHN, CNRS, EPHE, UA, Paris.

[2] Department of Natural Sciences, Faculty of Science and Technology, Middlesex University, United Kingdom.

[3] Centre for Ecological Genomics and Wildlife Conservation, Department of Zoology, University of Johannesburg, South Africa.

[4] Parc zoologique de Paris, Muséum national d'Histoire naturelle, Paris.

[5] Field Museum of Natural History, Chicago, IL, USA.

[6] Department of Wildlife Management, Pir Mehr Ali Shah, Arid Agriculture University Rawalpindi, Pakistan.

[7] Forest Parks & Wildlife Department Gilgit-Baltistan, Pakistan.

* Correspondence: alexandre.hassanin@mnhn.fr

Clade	Family	Taxons	A	C	G	T	A1	C1	G1	T1	A2	C2	G2	T2	A3	C3	G3	T3
Caniformia	Ailuridae	Ailurus_fulgens_NC011124_1	0,3116	0,2543	0,1221	0,3120	0,3296	0,2377	0,1986	0,2341	0,1933	0,2610	0,1202	0,4254	0,4118	0,2641	0,0475	0,2766
Caniformia	Ailuridae	Ailurus_fulgens_styani_NC009691_1	0,3124	0,2543	0,1213	0,3120	0,3305	0,2385	0,1977	0,2333	0,1936	0,2608	0,1191	0,4266	0,4132	0,2635	0,0469	0,2763
Caniformia	Canidae	Canis_adustus_KT448271_1	0,3021	0,2611	0,1323	0,3045	0,3110	0,2359	0,2145	0,2386	0,1955	0,2660	0,1188	0,4198	0,3999	0,2815	0,0635	0,2551
Caniformia	Canidae	Canis_anthus_NC027956_2	0,2983	0,2697	0,1328	0,2992	0,3188	0,2419	0,2086	0,2308	0,1983	0,2624	0,1169	0,4224	0,3780	0,3046	0,0730	0,2444
Caniformia	Canidae	Canis_aureus_KT448274_1	0,3009	0,2677	0,1305	0,3009	0,3191	0,2415	0,2081	0,2314	0,1982	0,2617	0,1172	0,4229	0,3855	0,3000	0,0660	0,2485
Caniformia	Canidae	Canis_latrans_NC008093_7	0,3018	0,2683	0,1298	0,3002	0,3185	0,2427	0,2088	0,2299	0,1975	0,2622	0,1178	0,4227	0,3893	0,2999	0,0628	0,2480
Caniformia	Canidae	Canis_lupus_chanco_NC010340_4	0,3001	0,2685	0,1318	0,2997	0,3184	0,2448	0,2083	0,2285	0,1976	0,2619	0,1179	0,4227	0,3842	0,2988	0,0692	0,2478
Caniformia	Canidae	Canis_lupus_familiaris_NC002008_1231	0,3012	0,2688	0,1302	0,2997	0,3188	0,2427	0,2074	0,2311	0,1975	0,2622	0,1180	0,4224	0,3874	0,3016	0,0653	0,2458
Caniformia	Canidae	Canis_mesomelas_KT448280_1	0,3039	0,2663	0,1287	0,3012	0,3185	0,2429	0,2090	0,2296	0,1956	0,2628	0,1200	0,4217	0,3976	0,2931	0,0570	0,2523
Caniformia	Canidae	Chrysocyon_brachyurus_NC024172_1	0,3075	0,2581	0,1258	0,3086	0,3235	0,2391	0,2030	0,2344	0,1963	0,2624	0,1183	0,4229	0,4027	0,2727	0,0561	0,2685
Caniformia	Canidae	Cuon_alpinus_NC013445_3	0,2994	0,2587	0,1328	0,3091	0,3164	0,2408	0,2092	0,2336	0,1950	0,2611	0,1197	0,4242	0,3868	0,2742	0,0695	0,2695
Caniformia	Canidae	Lycalopex_sechurae_KT448284_1	0,3050	0,2612	0,1280	0,3058	0,3201	0,2423	0,2078	0,2298	0,1958	0,2653	0,1186	0,4203	0,3991	0,2760	0,0575	0,2674
Caniformia	Canidae	Lycaon_pictus_NC028427_2	0,3046	0,2702	0,1265	0,2986	0,3205	0,2466	0,2041	0,2288	0,1969	0,2633	0,1186	0,4213	0,3966	0,3008	0,0569	0,2458
Caniformia	Canidae	Nyctereutes_procyonoides_NC013700_3	0,3044	0,2810	0,1324	0,2822	0,3174	0,2458	0,2097	0,2272	0,1961	0,2652	0,1186	0,4202	0,3996	0,3321	0,0689	0,1994
Caniformia	Canidae	Otocyon_megalotis_SAF1_2	0,2900	0,2858	0,1453	0,2790	0,3151	0,2522	0,2145	0,2183	0,1955	0,2683	0,1197	0,4166	0,3593	0,3369	0,1016	0,2022
Caniformia	Canidae	Speothos_venaticus_C48_2	0,3002	0,2643	0,1308	0,3047	0,3202	0,2452	0,2052	0,2294	0,1972	0,2655	0,1175	0,4199	0,3832	0,2821	0,0697	0,2649
Caniformia	Canidae	Urocyon_cinereoargenteus_NC026723_21	0,2909	0,2795	0,1443	0,2853	0,3149	0,2424	0,2133	0,2294	0,1963	0,2669	0,1189	0,4179	0,3616	0,3291	0,1008	0,2086
Caniformia	Canidae	Urocyon_littoralis_catalinae_KP129018_15	0,2911	0,2796	0,1440	0,2853	0,3152	0,2422	0,2130	0,2297	0,1961	0,2669	0,1191	0,4179	0,3621	0,3296	0,1000	0,2083
Caniformia	Canidae	Vulpes_corsac_NC023958_1	0,2953	0,2728	0,1389	0,2931	0,3174	0,2505	0,2122	0,2199	0,1966	0,2641	0,1191	0,4202	0,3718	0,3038	0,0853	0,2391
Caniformia	Canidae	Vulpes_ferrilata_NC027935_1	0,2927	0,2756	0,1415	0,2902	0,3166	0,2497	0,2130	0,2208	0,1969	0,2655	0,1186	0,4191	0,3646	0,3116	0,0930	0,2308
Caniformia	Canidae	Vulpes_lagopus_NC026529_3	0,2930	0,2779	0,1408	0,2884	0,3182	0,2491	0,2108	0,2219	0,1969	0,2658	0,1186	0,4188	0,3638	0,3188	0,0930	0,2244
Caniformia	Canidae	Vulpes_vulpes_NC008434_5	0,2943	0,2765	0,1389	0,2903	0,3149	0,2508	0,2141	0,2202	0,1966	0,2655	0,1189	0,4191	0,3713	0,3133	0,0839	0,2316
Caniformia	Canidae	Vulpes_zerda_KJ603240_1	0,2878	0,2904	0,1454	0,2764	0,3144	0,2524	0,2149	0,2183	0,1958	0,2647	0,1197	0,4199	0,3532	0,3541	0,1016	0,1911
Caniformia	Mephitidae	Conepatus_chinga_NC042596_1	0,3318	0,2511	0,1173	0,2998	0,3366	0,2297	0,1983	0,2355	0,1966	0,2619	0,1164	0,4252	0,4621	0,2619	0,0372	0,2388
Caniformia	Mephitidae	Mephitis_mephitis_NC020648_1	0,3259	0,2470	0,1214	0,3058	0,3288	0,2316	0,2027	0,2369	0,1969	0,2638	0,1169	0,4224	0,4521	0,2455	0,0444	0,2580
Caniformia	Mephitidae	Spilogale_putorius_NC010497_1	0,3271	0,2441	0,1206	0,3082	0,3260	0,2263	0,2033	0,2444	0,1975	0,2644	0,1166	0,4216	0,4579	0,2416	0,0419	0,2585
Caniformia	Mustelidae	Aonyx_cinerea_NC035814_2	0,2997	0,2992	0,1409	0,2602	0,3169	0,2647	0,2102	0,2083	0,1938	0,2683	0,1191	0,4188	0,3885	0,3646	0,0933	0,1536
Caniformia	Mustelidae	Arctonyx_collaris_NC020645_1	0,3195	0,2670	0,1209	0,2927	0,3238	0,2377	0,2044	0,2341	0,1963	0,2641	0,1164	0,4232	0,4382	0,2991	0,0419	0,2208
Caniformia	Mustelidae	Enhydra_lutris_NC009692_1	0,3119	0,2871	0,1308	0,2702	0,3194	0,2549	0,2088	0,2169	0,1950	0,2663	0,1175	0,4213	0,4213	0,3402	0,0661	0,1725
Caniformia	Mustelidae	Galictis_vittata_T412_1	0,3046	0,2799	0,1381	0,2774	0,3124	0,2497	0,2163	0,2216	0,1958	0,2692	0,1172	0,4178	0,4057	0,3207	0,0808	0,1927
Caniformia	Mustelidae	Gulo_gulo_NC009685_3	0,3049	0,2828	0,1331	0,2791	0,3153	0,2512	0,2107	0,2229	0,1943	0,2651	0,1180	0,4227	0,4052	0,3322	0,0708	0,1918
Caniformia	Mustelidae	Ictonyx_striatus_T299_1	0,3105	0,2806	0,1285	0,2803	0,3224	0,2458	0,2077	0,2241	0,1944	0,2688	0,1178	0,4191	0,4149	0,3274	0,0601	0,1977
Caniformia	Mustelidae	Lontra_canadensis_SRR10409165_1	0,2942	0,2906	0,1479	0,2673	0,3121	0,2583	0,2136	0,2161	0,1936	0,2674	0,1191	0,4199	0,3768	0,3460	0,1111	0,1661
Caniformia	Mustelidae	Lutra_lutra_LC050126_1	0,3072	0,2914	0,1342	0,2672	0,3207	0,2613	0,2061	0,2119	0,1938	0,2674	0,1183	0,4204	0,4071	0,3455	0,0783	0,1691
Caniformia	Mustelidae	Lutra_lutra_NC011358_9	0,3059	0,2933	0,1357	0,2650	0,3194	0,2613	0,2083	0,2111	0,1947	0,2683	0,1178	0,4193	0,4038	0,3505	0,0811	0,1647
Caniformia	Mustelidae	Lutra_sumatrana_NC035810_1	0,3074	0,2918	0,1341	0,2667	0,3205	0,2602	0,2069	0,2124	0,1936	0,2680	0,1180	0,4204	0,4082	0,3471	0,0775	0,1672
Caniformia	Mustelidae	Lutrogale_perspicillata_NC035811_1	0,2945	0,2998	0,1456	0,2601	0,3138	0,2638	0,2124	0,2099	0,1936	0,2677	0,1191	0,4196	0,3760	0,3680	0,1053	0,1508
Caniformia	Mustelidae	Martes_americana_NC020642_1	0,2991	0,2933	0,1400	0,2677	0,3177	0,2569	0,2108	0,2147	0,1947	0,2655	0,1178	0,4221	0,3849	0,3574	0,0914	0,1663
Caniformia	Mustelidae	Martes_flavigula_NC012141_3	0,3074	0,2881	0,1309	0,2736	0,3178	0,2539	0,2083	0,2200	0,1953	0,2664	0,1175	0,4208	0,4092	0,3439	0,0669	0,1800
Caniformia	Mustelidae	Martes_foina_NC020643_1	0,3043	0,2910	0,1350	0,2697	0,3202	0,2547	0,2080	0,2172	0,1941	0,2633	0,1183	0,4243	0,3985	0,3552	0,0786	0,1677
Caniformia	Mustelidae	Martes_martes_T302_3	0,3008	0,2936	0,1394	0,2662	0,3160	0,2555	0,2124	0,2161	0,1927	0,2647	0,1197	0,4229	0,3938	0,3607	0,0861	0,1595
Caniformia	Mustelidae	Martes_melampus_NC009678_1	0,3006	0,2935	0,1389	0,2670	0,3180	0,2535	0,2108	0,2177	0,1930	0,2660	0,1191	0,4218	0,3907	0,3610	0,0869	0,1613
Caniformia	Mustelidae	Martes_pennanti_NC020664_16	0,3011	0,2880	0,1373	0,2736	0,3185	0,2522	0,2097	0,2197	0,1955	0,2655	0,1180	0,4210	0,3893	0,3463	0,0841	0,1802
Caniformia	Mustelidae	Martes_zibellina_NC011579_39	0,2998	0,2928	0,1403	0,2671	0,3169	0,2535	0,2116	0,2180	0,1930	0,2647	0,1194	0,4229	0,3896	0,3602	0,0900	0,1602
Caniformia	Mustelidae	Meles_anakuma_NC009677_1	0,3133	0,2723	0,1242	0,2901	0,3199	0,2422	0,2061	0,2319	0,1952	0,2619	0,1178	0,4252	0,4249	0,3130	0,0489	0,2133
Caniformia	Mustelidae	Meles_leucurus_NC039173_4	0,3145	0,2706	0,1244	0,2906	0,3188	0,2441	0,2072	0,2299	0,1955	0,2605	0,1175	0,4266	0,4291	0,3071	0,0486	0,2152
Caniformia	Mustelidae	Meles_meles_T303_3	0,3166	0,2714	0,1234	0,2886	0,3182	0,2463	0,2083	0,2272	0,1955	0,2608	0,1178	0,4260	0,4360	0,3071	0,0442	0,2127
Caniformia	Mustelidae	Mellivora_capensis_T370_1	0,2835	0,2865	0,1565	0,2734	0,3055	0,2555	0,2219	0,2172	0,1913	0,2680	0,1219	0,4188	0,3538	0,3360	0,1258	0,1844
Caniformia	Mustelidae	Melogale_moschata_KP726273_1	0,3035	0,2841	0,1388	0,2736	0,3152	0,2516	0,2144	0,2188	0,1938	0,2663	0,1189	0,4210	0,4016	0,3344	0,0830	0,1811
Caniformia	Mustelidae	Melogale_moschata_NC020644_1	0,3033	0,2844	0,1388	0,2736	0,3158	0,2519	0,2144	0,2180	0,1941	0,2666	0,1183	0,4210	0,3999	0,3346	0,0836	0,1819
Caniformia	Mustelidae	Melogale_moschata_V0735A_1	0,3008	0,2843	0,1413	0,2737	0,3158	0,2508	0,2144	0,2191	0,1933	0,2660	0,1191	0,4216	0,3932	0,3360	0,0903	0,1805
Caniformia	Mustelidae	Mustela_altaica_NC021751_1	0,3129	0,2726	0,1288	0,2858	0,3210	0,2408	0,2074	0,2308	0,1930	0,2649	0,1186	0,4235	0,4246	0,3121	0,0603	0,2030
Caniformia	Mustelidae	Mustela_erminea_T305_2	0,3212	0,2807	0,1231	0,2750	0,3219	0,2477	0,2061	0,2244	0,1944	0,2666	0,1180	0,4210	0,4474	0,3277	0,0453	0,1797
Caniformia	Mustelidae	Mustela_eversmannii_NC028013_1	0,3131	0,2759	0,1292	0,2818	0,3207	0,2466	0,2080	0,2247	0,1936	0,2677	0,1191	0,4196	0,4249	0,3135	0,0605	0,2011
Caniformia	Mustelidae	Mustela_frenata_NC020640_1	0,3180	0,2726	0,1241	0,2853	0,3227	0,2422	0,2055	0,2297	0,1938	0,2663	0,1178	0,4221	0,4374	0,3094	0,0492	0,2041
Caniformia	Mustelidae	Mustela_itatsi_NC034330_19	0,3175	0,2731	0,1261	0,2834	0,3238	0,2441	0,2052	0,2269	0,1938	0,2674	0,1189	0,4199	0,4349	0,3077	0,0542	0,2033
Caniformia	Mustelidae	Mustela_kathiah_NC023210_1	0,3194	0,2653	0,1260	0,2894	0,3224	0,2383	0,2058	0,2336	0,1941	0,2649	0,1183	0,4227	0,4415	0,2927	0,0539	0,2119
Caniformia	Mustelidae	Mustela_nigripes_NC024942_1	0,3148	0,2758	0,1273	0,2821	0,3235	0,2452	0,2058	0,2255	0,1925	0,2669	0,1200	0,4207	0,4285	0,3152	0,0561	0,2002
Caniformia	Mustelidae	Mustela_nivalis_T306_5	0,3115	0,2760	0,1299	0,2826	0,3196	0,2422	0,2091	0,2291	0,1938	0,2655	0,1183	0,4224	0,4210	0,3205	0,0622	0,1963
Caniformia	Mustelidae	Mustela_putorius_NC020638_4	0,3140	0,2756	0,1279	0,2825	0,3224	0,2449	0,2061	0,2266	0,1930	0,2660	0,1194	0,4216	0,4266	0,3158	0,0583	0,1994
Caniformia	Mustelidae	Mustela_sibirica_AP017394_11	0,3146	0,2734	0,1290	0,2830	0,3221	0,2449	0,2066	0,2263	0,1927	0,2663	0,1200	0,4210	0,4291	0,3088	0,0605	0,2016
Caniformia	Mustelidae	Mustela_sibirica_NC020637_6	0,3148	0,2738	0,1284	0,2830	0,3205	0,2438	0,2083	0,2274	0,1930	0,2658	0,1197	0,4216	0,4310	0,3119	0,0572	0,1999
Caniformia	Mustelidae	Neovison_vison_NC020641_3	0,3230	0,2659	0,1215	0,2896	0,3244	0,2355	0,2061	0,2341	0,1938	0,2688	0,1175	0,4199	0,4507	0,2935	0,0411	0,2147
Caniformia	Mustelidae	Poecilogale_albinucha_T602_1	0,3179	0,2684	0,1232	0,2905	0,3260	0,2383	0,2049	0,2308	0,1941	0,2667	0,1178	0,4214	0,4335	0,3002	0,0469	0,2194
Caniformia	Mustelidae	Taxidea_taxus_NC020646_1	0,3247	0,2857	0,1202	0,2695	0,3249	0,2524	0,2041	0,2186	0,1944	0,2666	0,1189	0,4202	0,4549	0,3380	0,0375	0,1697
Caniformia	Odobenidae	Odobenus_rosmarus_NC004029_29	0,3083	0,2762	0,1366	0,2789	0,3219	0,2477	0,2088	0,2216	0,1944	0,2722	0,1186	0,4149	0,4085	0,3088	0,0825	0,2002
Caniformia	Otariidae	Arctocephalus_australis_MG023139_1	0,3130	0,2836	0,1323	0,2712	0,3242	0,2531	0,2057	0,2170	0,1921	0,2703	0,1185	0,4191	0,4227	0,3272	0,0727	0,1774
Caniformia	Otariidae	Arctocephalus_forsteri_KT693377_17	0,3137	0,2833	0,1317	0,2713	0,3231	0,2542	0,2076	0,2151	0,1921	0,2692	0,1188	0,4199	0,4260	0,3264	0,0688	0,1787
Caniformia	Otariidae	Arctocephalus_forsteri_NC004023_28	0,3142	0,2847	0,1312	0,2700	0,3245	0,2531	0,2059	0,2165	0,1921	0,2701	0,1188	0,4191	0,4260	0,3308	0,0688	0,1743
Caniformia	Otariidae	Arctocephalus_gazella_BK010918_1	0,3147	0,2836	0,1317	0,2701	0,3233	0,2548	0,2079	0,2140	0,1923	0,2701	0,1185	0,4191	0,4285	0,3258	0,0686	0,1771
Caniformia	Otariidae	Arctocephalus_pusillus_NC008417_1	0,3162	0,2841	0,1303	0,2694	0,3245	0,2559	0,2071	0,2126	0,1926	0,2703	0,1182	0,4188	0,4316	0,3261	0,0655	0,1768
Caniformia	Otariidae	Arctocephalus_townsendi_NC008420_1	0,3119	0,2840	0,1337	0,2704	0,3203	0,2551	0,2098	0,2148	0,1932	0,2706	0,1177	0,4185	0,4222	0,3264	0,0736	0,1779
Caniformia	Otariidae	Callorhinus_ursinus_NC008415_1	0,3166	0,2807	0,1295	0,2732	0,3242	0,2526	0,2062	0,2170	0,1929	0,2681	0,1185	0,4205	0,4327	0,3214	0,0638	0,1821
Caniformia	Otariidae	Eumetopias_jubatus_NC004030_10	0,3207	0,2866	0,1247	0,2680	0,3261	0,2565	0,2034	0,2140	0,1932	0,2703	0,1182	0,4183	0,4427	0,3331	0,0525	0,1718
Caniformia	Otariidae	Otaria_byronia_OTAB_1	0,3140	0,2849	0,1324	0,2688	0,3242	0,2553	0,2062	0,2143	0,1923	0,2709	0,1193	0,4174	0,4255	0,3283	0,0716	0,1746
Caniformia	Otariidae	Phocarctos_hookeri_NC008418_1	0,3111	0,2843	0,1354	0,2692	0,3242	0,2498	0,2082	0,2179	0,1929	0,2706	0,1177	0,4188	0,4163	0,3325	0,0802	0,1710
Caniformia	Otariidae	Zalophus_californianus_NC008416_1	0,3191	0,2841	0,1269	0,2698	0,3258	0,2576	0,2051	0,2115	0,1929	0,2709	0,1188	0,4174	0,4387	0,3240	0,0569	0,1805
Caniformia	Otariidae	Zalophus_wollebaeki_SRR4431565_1	0,3192	0,2839	0,1268	0,2702	0,3253	0,2576	0,2054	0,2118	0,1932	0,2709	0,1185	0,4174	0,4392	0,3232	0,0564	0,1813
Caniformia	Phocidae	Cystophora_cristata_NC008427_1	0,3147	0,2905	0,1293	0,2655	0,3232	0,2627	0,2080	0,2061	0,1947	0,2674	0,1186	0,4193	0,4263	0,3413	0,0614	0,1711
Caniformia	Phocidae	Erignathus_barbatus_NC008426_1	0,3203	0,2855	0,1243	0,2699	0,3263	0,2549	0,2055	0,2133	0,1947	0,2666	0,1191	0,4196	0,4399	0,3349	0,0483	0,1769
Caniformia	Phocidae	Halichoerus_grypus_NC001602_2	0,3112	0,2931	0,1326	0,2631	0,3199	0,2605	0,2111	0,2086	0,1955	0,2660	0,1183	0,4202	0,4183	0,3528	0,0683	0,1606
Caniformia	Phocidae	Hydrurga_leptonyx_NC008425_1	0,3276	0,2873	0,1181	0,2670	0,3244	0,2547	0,2052	0,2158	0,1972	0,2658	0,1175	0,4196	0,4613	0,3416	0,0317	0,1655
Caniformia	Phocidae	Leptonychotes_weddellii_NC008424_1	0,3275	0,2896	0,1183	0,2646	0,3246	0,2566	0,2047	0,2141	0,1972	0,2680	0,1175	0,4174	0,4607	0,3444	0,0328	0,1622
Caniformia	Phocidae	Lobodon_carcinophaga_NC008423_1	0,3276	0,2834	0,1181	0,2709	0,3244	0,2544	0,2047	0,2166	0,1975	0,2644	0,1175	0,4207	0,4610	0,3313	0,0322	0,1755
Caniformia	Phocidae	Mirounga_angustirostris_SRR10331586_1	0,3273	0,2769	0,1168	0,2790	0,3263	0,2530	0,2041	0,2166	0,1966	0,2669	0,1164	0,4202	0,4590	0,3108	0,0300	0,2002
Caniformia	Phocidae	Mirounga_leonina_NC008422_1	0,3262	0,2759	0,1181	0,2798	0,3249	0,2516	0,2052	0,2183	0,1950	0,2655	0,1178	0,4218	0,4588	0,3105	0,0314	0,1994
Caniformia	Phocidae	Monachus_monachus_NC044972_5	0,3287	0,2694	0,1171	0,2847	0,3281	0,2531	0,2033	0,2156	0,1975	0,2619	0,1169	0,4236	0,4606	0,2933	0,0311	0,2150
Caniformia	Phocidae	Monachus_schauinslandi_NC008421_1	0,3272	0,2782	0,1178	0,2769	0,3291	0,2544	0,2013	0,2152	0,1977	0,2649	0,1169	0,4204	0,4549	0,3152	0,0350	0,1950
Caniformia	Phocidae	Neophoca_cinerea_NC008419_1	0,3133	0,2854	0,1319	0,2694	0,3233	0,2584	0,2071	0,2112	0,1937	0,2709	0,1171	0,4183	0,4227	0,3270	0,0716	0,1787
Caniformia	Phocidae	Ommatophoca_rossii_AY377287etc_1	0,3273	0,2837	0,1176	0,2715	0,3263	0,2541	0,2036	0,2161	0,1958	0,2661	0,1183	0,4197	0,4597	0,3308	0,0308	0,1786
Caniformia	Phocidae	Phoca_fasciata_NC008428_1	0,3182	0,2851	0,1261	0,2707	0,3232	0,2580	0,2077	0,2111	0,1958	0,2663	0,1178	0,4202	0,4354	0,3310	0,0528	0,1808
Caniformia	Phocidae	Phoca_groenlandica_NC008429_54	0,3170	0,2811	0,1273	0,2746	0,3210	0,2566	0,2099	0,2124	0,1958	0,2638	0,1169	0,4235	0,4343	0,3230	0,0550	0,1877
Caniformia	Phocidae	Phoca_largha_NC008430_1	0,3113	0,2935	0,1326	0,2626	0,3216	0,2619	0,2097	0,2069	0,1950	0,2669	0,1191	0,4191	0,4174	0,3519	0,0689	0,1619
Caniformia	Phocidae	Phoca_vitulina_NC001325_1	0,3115	0,2929	0,1327	0,2629	0,3213	0,2594	0,2097	0,2097	0,1950	0,2663	0,1194	0,4193	0,4182	0,3530	0,0692	0,1597
Caniformia	Phocidae	Pusa_caspica_NC008431_1	0,3104	0,2938	0,1335	0,2623	0,3199	0,2605	0,2113	0,2083	0,1944	0,2672	0,1191	0,4193	0,4168	0,3538	0,0700	0,1594
Caniformia	Phocidae	Pusa_hispida_NC_008433_1	0,3131	0,2924	0,1309	0,2636	0,3210	0,2599	0,2099	0,2091	0,1952	0,2658	0,1189	0,4202	0,4229	0,3516	0,0639	0,1616
Caniformia	Phocidae	Pusa_sibirica_NC008432_2	0,3101	0,2930	0,1339	0,2630	0,3180	0,2588	0,2136	0,2097	0,1950	0,2663	0,1189	0,4199	0,4174	0,3538	0,0694	0,1594
Caniformia	Procyonidae	Bassaricyon_neblina_SRX1097850_1	0,3072	0,2892	0,1453	0,2583	0,3177	0,2544	0,2152	0,2127	0,1966	0,2688	0,1183	0,4163	0,4074	0,3444	0,1025	0,1458
Caniformia	Procyonidae	Bassariscus_sumichrasti_SRX1099089_1	0,3281	0,2734	0,1202	0,2784	0,3321	0,2441	0,2005	0,2233	0,1977	0,2694	0,1183	0,4146	0,4543	0,3066	0,0417	0,1975
Caniformia	Procyonidae	Nasua_nasua_NC020647_1	0,3151	0,2783	0,1368	0,2698	0,3305	0,2444	0,2044	0,2208	0,1988	0,2730	0,1169	0,4113	0,4160	0,3174	0,0891	0,1775
Caniformia	Procyonidae	Potos_flavus_T414_1	0,3149	0,2824	0,1281	0,2746	0,3244	0,2463	0,2074	0,2219	0,1977	0,2730	0,1164	0,4129	0,4227	0,3280	0,0605	0,1888
Caniformia	Procyonidae	Procyon_lotor_AB462046_3	0,3185	0,2663	0,1272	0,2880	0,3244	0,2405	0,2055	0,2297	0,1977	0,2630	0,1172	0,4221	0,4335	0,2955	0,0589	0,2122
Caniformia	Procyonidae	Procyon_lotor_AB462049_4	0,3166	0,2676	0,1288	0,2871	0,3244	0,2416	0,2052	0,2288	0,1980	0,2635	0,1169	0,4216	0,4274	0,2977	0,0642	0,2108
Caniformia	Ursidae	Ailuropoda_melanoleuca_NC009492_5	0,3063	0,2471	0,1364	0,3102	0,3144	0,2463	0,2166	0,2227	0,1955	0,2627	0,1191	0,4227	0,4091	0,2322	0,0736	0,2852
Caniformia	Ursidae	Arctodus_simus_NC011116_1	0,3031	0,2709	0,1379	0,2881	0,3116	0,2527	0,2180	0,2177	0,1944	0,2677	0,1186	0,4193	0,4032	0,2924	0,0772	0,2272
Caniformia	Ursidae	Arctotherium_sp_NC030174_1	0,2994	0,2731	0,1403	0,2872	0,3152	0,2541	0,2141	0,2166	0,1938	0,2669	0,1183	0,4210	0,3891	0,2983	0,0886	0,2241
Caniformia	Ursidae	Helarctos_malayanus_NC009968_2	0,2965	0,2730	0,1425	0,2881	0,3077	0,2549	0,2211	0,2163	0,1947	0,2683	0,1186	0,4185	0,3871	0,2958	0,0878	0,2294
Caniformia	Ursidae	Melursus_ursinus_NC009970_2	0,2927	0,2781	0,1466	0,2826	0,3077	0,2566	0,2211	0,2147	0,1933	0,2694	0,1200	0,4174	0,3771	0,3083	0,0989	0,2158
Caniformia	Ursidae	Tremarctos_ornatus_NC009969_2	0,2977	0,2779	0,1420	0,2824	0,3113	0,2544	0,2172	0,2172	0,1955	0,2674	0,1172	0,4199	0,3863	0,3119	0,0916	0,2102
Caniformia	Ursidae	Ursus_americanus_JX196366_3	0,2956	0,2700	0,1449	0,2896	0,3093	0,2570	0,2208	0,2130	0,1933	0,2683	0,1208	0,4177	0,3842	0,2846	0,0931	0,2380
Caniformia	Ursidae	Ursus_arctos_AP012576_6	0,2933	0,2701	0,1452	0,2913	0,3074	0,2569	0,2208	0,2149	0,1933	0,2660	0,1197	0,4210	0,3793	0,2874	0,0953	0,2380
Caniformia	Ursidae	Ursus_arctos_EU497665_29	0,2931	0,2692	0,1455	0,2922	0,3071	0,2560	0,2211	0,2158	0,1930	0,2660	0,1200	0,4210	0,3791	0,2855	0,0955	0,2399
Caniformia	Ursidae	Ursus_arctos_GU573486_5	0,2939	0,2710	0,1450	0,2901	0,3067	0,2569	0,2212	0,2152	0,1933	0,2663	0,1197	0,4207	0,3816	0,2899	0,0941	0,2344
Caniformia	Ursidae	Ursus_arctos_GU573491_207	0,2936	0,2697	0,1450	0,2918	0,3083	0,2558	0,2199	0,2161	0,1933	0,2663	0,1197	0,4207	0,3793	0,2869	0,0953	0,2385
Caniformia	Ursidae	Ursus_arctos_isabellinus_18B5_2	0,2945	0,2682	0,1443	0,2931	0,3071	0,2544	0,2211	0,2174	0,1930	0,2660	0,1200	0,4210	0,3832	0,2841	0,0919	0,2408
Caniformia	Ursidae	Ursus_arctos_pruinosus_MG066703_3	0,2922	0,2698	0,1464	0,2916	0,3066	0,2558	0,2219	0,2158	0,1930	0,2652	0,1200	0,4218	0,3771	0,2885	0,0973	0,2372
Caniformia	Ursidae	Ursus_maritimus_GU573488_Svalbard	0,2939	0,2699	0,1448	0,2914	0,3066	0,2560	0,2213	0,2161	0,1933	0,2652	0,1197	0,4218	0,3818	0,2885	0,0933	0,2363
Caniformia	Ursidae	Ursus_maritimus_NC003428_31	0,2938	0,2702	0,1449	0,2911	0,3063	0,2563	0,2216	0,2158	0,1936	0,2655	0,1194	0,4216	0,3816	0,2888	0,0936	0,2361
Caniformia	Ursidae	Ursus_spelaeus_EU327344_13	0,2968	0,2677	0,1423	0,2933	0,3096	0,2544	0,2191	0,2169	0,1938	0,2641	0,1194	0,4227	0,3868	0,2846	0,0883	0,2402
Caniformia	Ursidae	Ursus_spelaeus_NC011112_8	0,2975	0,2695	0,1417	0,2913	0,3116	0,2558	0,2172	0,2155	0,1936	0,2647	0,1197	0,4221	0,3874	0,2880	0,0883	0,2363
Caniformia	Ursidae	Ursus_thibetanus_formosanus_NC009331_1	0,2958	0,2704	0,1432	0,2906	0,3063	0,2580	0,2233	0,2124	0,1938	0,2660	0,1197	0,4204	0,3874	0,2871	0,0866	0,2388
Caniformia	Ursidae	Ursus_thibetanus_laniger_MH281753_2	0,2952	0,2704	0,1439	0,2905	0,3063	0,2591	0,2233	0,2113	0,1938	0,2672	0,1197	0,4193	0,3855	0,2849	0,0889	0,2408
Caniformia	Ursidae	Ursus_thibetanus_mupinensis_NC008753_2	0,2970	0,2712	0,1426	0,2893	0,3074	0,2577	0,2222	0,2127	0,1941	0,2672	0,1197	0,4191	0,3893	0,2888	0,0858	0,2361
Caniformia	Ursidae	Ursus_thibetanus_thibetanus_NC011118_4	0,2954	0,2704	0,1436	0,2907	0,3066	0,2574	0,2224	0,2136	0,1938	0,2666	0,1200	0,4196	0,3857	0,2871	0,0883	0,2388
Feliformia	Eupleridae	Cryptoprocta_ferox_CFC13_1	0,3002	0,3062	0,1389	0,2547	0,3135	0,2666	0,2127	0,2072	0,1961	0,2685	0,1191	0,4163	0,3910	0,3835	0,0847	0,1408
Feliformia	Eupleridae	Eupleres_goudotii_D128_1	0,3045	0,2988	0,1341	0,2626	0,3199	0,2577	0,2083	0,2141	0,1944	0,2702	0,1202	0,4152	0,3991	0,3685	0,0739	0,1586
Feliformia	Eupleridae	Fossa_fossana_D350_1	0,3162	0,2943	0,1248	0,2647	0,3207	0,2566	0,2069	0,2158	0,1972	0,2688	0,1172	0,4168	0,4307	0,3574	0,0503	0,1616
Feliformia	Eupleridae	Galidia_elegans_D146_1	0,3016	0,2974	0,1375	0,2635	0,3152	0,2630	0,2124	0,2094	0,1963	0,2680	0,1186	0,4171	0,3932	0,3613	0,0814	0,1641
Feliformia	Eupleridae	Galidictis_fasciata_DM333_1	0,3042	0,3032	0,1346	0,2581	0,3202	0,2644	0,2083	0,2072	0,1958	0,2699	0,1191	0,4152	0,3966	0,3752	0,0764	0,1519
Feliformia	Eupleridae	Mungotictis_decemlineata_NC027828_1	0,3015	0,2976	0,1369	0,2640	0,3174	0,2630	0,2102	0,2094	0,1952	0,2674	0,1197	0,4177	0,3918	0,3624	0,0808	0,1650
Feliformia	Eupleridae	Salanoia_concolor_D378_1	0,3048	0,2996	0,1345	0,2611	0,3196	0,2647	0,2088	0,2069	0,1963	0,2680	0,1189	0,4168	0,3985	0,3660	0,0758	0,1597
Feliformia	Felidae	Acinonyx_jubatus_NC005212_3	0,3158	0,2716	0,1246	0,2880	0,3235	0,2488	0,2072	0,2205	0,1977	0,2630	0,1180	0,4213	0,4263	0,3030	0,0486	0,2222
Feliformia	Felidae	Caracal_caracal_NC028306_1	0,3140	0,2699	0,1274	0,2887	0,3221	0,2499	0,2080	0,2199	0,1983	0,2635	0,1178	0,4204	0,4216	0,2963	0,0564	0,2258
Feliformia	Felidae	Catopuma_badia_NC028300_1	0,3161	0,2733	0,1248	0,2859	0,3232	0,2485	0,2061	0,2222	0,1977	0,2641	0,1183	0,4199	0,4274	0,3071	0,0500	0,2155
Feliformia	Felidae	Catopuma_temminckii_NC027115_41	0,3117	0,2728	0,1296	0,2859	0,3199	0,2485	0,2097	0,2219	0,1988	0,2619	0,1180	0,4213	0,4163	0,3080	0,0611	0,2147
Feliformia	Felidae	Felis_catus_NC001700_2	0,3092	0,2782	0,1316	0,2810	0,3182	0,2508	0,2108	0,2202	0,1972	0,2638	0,1191	0,4199	0,4121	0,3199	0,0650	0,2030
Feliformia	Felidae	Felis_chaus_NC028307_1	0,3105	0,2689	0,1309	0,2897	0,3210	0,2463	0,2091	0,2236	0,1983	0,2630	0,1178	0,4210	0,4121	0,2974	0,0658	0,2247
Feliformia	Felidae	Felis_margarita_NC028308_1	0,3100	0,2766	0,1299	0,2835	0,3194	0,2480	0,2108	0,2219	0,1972	0,2649	0,1180	0,4199	0,4135	0,3169	0,0608	0,2088
Feliformia	Felidae	Felis_nigripes_NC028309_1	0,3092	0,2753	0,1316	0,2839	0,3194	0,2491	0,2099	0,2216	0,1972	0,2624	0,1189	0,4216	0,4110	0,3144	0,0661	0,2086
Feliformia	Felidae	Felis_silvestris_lybica_KP202275_4	0,3105	0,2788	0,1298	0,2809	0,3199	0,2510	0,2097	0,2194	0,1977	0,2638	0,1178	0,4207	0,4138	0,3216	0,0619	0,2027
Feliformia	Felidae	Homotherium_latidens_MF871702_3	0,3095	0,2661	0,1268	0,2976	0,3008	0,2419	0,2265	0,2307	0,1993	0,2669	0,1258	0,4080	0,4285	0,2894	0,0280	0,2541
Feliformia	Felidae	Leopardus_colocolo_NC028314_1	0,3155	0,2781	0,1252	0,2813	0,3244	0,2469	0,2069	0,2219	0,1969	0,2663	0,1191	0,4177	0,4252	0,3210	0,0494	0,2044
Feliformia	Felidae	Leopardus_geoffroyi_NC028320_1	0,3129	0,2801	0,1270	0,2800	0,3230	0,2533	0,2066	0,2172	0,1975	0,2666	0,1186	0,4174	0,4182	0,3205	0,0558	0,2055
Feliformia	Felidae	Leopardus_guigna_NC028321_1	0,3135	0,2814	0,1263	0,2788	0,3238	0,2499	0,2061	0,2202	0,1980	0,2658	0,1180	0,4182	0,4188	0,3285	0,0547	0,1980
Feliformia	Felidae	Leopardus_jacobita_NC028322_1	0,3143	0,2799	0,1260	0,2798	0,3235	0,2499	0,2072	0,2194	0,1966	0,2663	0,1189	0,4182	0,4227	0,3235	0,0519	0,2019
Feliformia	Felidae	Leopardus_pardalis_NC028315_1	0,3137	0,2832	0,1268	0,2763	0,3227	0,2527	0,2083	0,2163	0,1972	0,2655	0,1189	0,4185	0,4213	0,3313	0,0533	0,1941
Feliformia	Felidae	Leopardus_pardalis_T262_1	0,3145	0,2829	0,1260	0,2766	0,3235	0,2508	0,2074	0,2183	0,1963	0,2674	0,1194	0,4168	0,4238	0,3305	0,0511	0,1947

Feliformia	Felidae	Leopardus_tigrinus_NC028317_1	0,3146	0,2806	0,1251	0,2797	0,3241	0,2524	0,2055	0,2180	0,1972	0,2669	0,1189	0,4171	0,4227	0,3224	0,0508	0,2041
Feliformia	Felidae	Leopardus_wiedii_NC028318_1	0,3132	0,2815	0,1274	0,2780	0,3219	0,2505	0,2094	0,2183	0,1977	0,2663	0,1183	0,4177	0,4199	0,3277	0,0544	0,1980
Feliformia	Felidae	Leptailurus_serval_NC028316_1	0,3152	0,2685	0,1251	0,2912	0,3224	0,2449	0,2072	0,2255	0,1986	0,2635	0,1175	0,4204	0,4246	0,2971	0,0505	0,2277
Feliformia	Felidae	Lynx_canadensis_NC028313_1	0,3066	0,2890	0,1336	0,2709	0,3207	0,2547	0,2091	0,2155	0,1980	0,2658	0,1183	0,4179	0,4010	0,3466	0,0733	0,1791
Feliformia	Felidae	Lynx_lynx_NC027083_4	0,3064	0,2913	0,1334	0,2689	0,3202	0,2566	0,2097	0,2136	0,1977	0,2652	0,1183	0,4188	0,4013	0,3521	0,0722	0,1744
Feliformia	Felidae	Lynx_pardinus_NC028319_161	0,3086	0,2864	0,1314	0,2736	0,3205	0,2533	0,2094	0,2169	0,1983	0,2649	0,1180	0,4188	0,4071	0,3410	0,0667	0,1852
Feliformia	Felidae	Lynx_rufus_NC014456_3	0,3079	0,2856	0,1327	0,2739	0,3182	0,2547	0,2108	0,2163	0,1988	0,2638	0,1175	0,4199	0,4066	0,3382	0,0697	0,1855
Feliformia	Felidae	Neofelis_nebulosa_NC008450_3	0,2996	0,2790	0,1379	0,2835	0,3163	0,2516	0,2122	0,2199	0,1966	0,2663	0,1194	0,4177	0,3857	0,3191	0,0822	0,2130
Feliformia	Felidae	Otocolobus_manul_NC028323_1	0,3167	0,2695	0,1236	0,2903	0,3238	0,2416	0,2066	0,2280	0,1983	0,2641	0,1172	0,4204	0,4279	0,3027	0,0469	0,2224
Feliformia	Felidae	Panthera_leo_NERO_19	0,3026	0,2804	0,1341	0,2829	0,3167	0,2510	0,2122	0,2201	0,1961	0,2664	0,1197	0,4178	0,3949	0,3238	0,0705	0,2108
Feliformia	Felidae	Panthera_leo_spelaea_KX258452_2	0,2990	0,2803	0,1379	0,2827	0,3164	0,2531	0,2133	0,2172	0,1956	0,2658	0,1200	0,4186	0,3852	0,3221	0,0803	0,2123
Feliformia	Felidae	Panthera_onca_KP202264_2	0,2994	0,2819	0,1383	0,2805	0,3160	0,2558	0,2127	0,2155	0,1955	0,2669	0,1200	0,4177	0,3866	0,3230	0,0822	0,2083
Feliformia	Felidae	Panthera_onca_NC022842_1	0,3008	0,2829	0,1364	0,2800	0,3166	0,2552	0,2122	0,2161	0,1944	0,2683	0,1208	0,4166	0,3913	0,3252	0,0761	0,2074
Feliformia	Felidae	Panthera_pardus_japonensis_KJ866876_8	0,2996	0,2808	0,1368	0,2828	0,3158	0,2522	0,2133	0,2188	0,1952	0,2658	0,1205	0,4185	0,3880	0,3244	0,0767	0,2111
Feliformia	Felidae	Panthera_pardus_NC010641_5	0,3001	0,2805	0,1366	0,2828	0,3146	0,2519	0,2147	0,2188	0,1950	0,2652	0,1205	0,4193	0,3907	0,3244	0,0747	0,2102
Feliformia	Felidae	Panthera_tigris_amoyensis_NC014770_2	0,2993	0,2810	0,1384	0,2813	0,3174	0,2510	0,2124	0,2191	0,1947	0,2655	0,1205	0,4193	0,3857	0,3266	0,0822	0,2055
Feliformia	Felidae	Panthera_tigris_NC010642_35	0,2992	0,2821	0,1384	0,2804	0,3180	0,2519	0,2111	0,2191	0,1955	0,2660	0,1200	0,4185	0,3841	0,3282	0,0841	0,2036
Feliformia	Felidae	Panthera_uncia_KP202269_1	0,3013	0,2823	0,1357	0,2807	0,3180	0,2549	0,2108	0,2163	0,1958	0,2669	0,1202	0,4171	0,3902	0,3252	0,0761	0,2086
Feliformia	Felidae	Panthera_uncia_NC010638_1	0,3016	0,2813	0,1363	0,2809	0,3188	0,2535	0,2102	0,2174	0,1969	0,2680	0,1194	0,4157	0,3891	0,3224	0,0791	0,2094
Feliformia	Felidae	Pardofelis_marmorata_NLN3_2	0,3081	0,2794	0,1321	0,2805	0,3210	0,2524	0,2088	0,2177	0,1977	0,2663	0,1183	0,4177	0,4054	0,3194	0,0692	0,2061
Feliformia	Felidae	Prionailurus_bengalensis_CKM45_20	0,3129	0,2745	0,1267	0,2859	0,3224	0,2430	0,2088	0,2258	0,1986	0,2647	0,1175	0,4193	0,4177	0,3158	0,0539	0,2127
Feliformia	Felidae	Prionailurus_bengalensis_NC028301_12	0,3154	0,2747	0,1240	0,2859	0,3244	0,2447	0,2066	0,2244	0,1988	0,2655	0,1172	0,4185	0,4229	0,3141	0,0483	0,2147
Feliformia	Felidae	Prionailurus_planiceps_KY682741_4	0,3123	0,2772	0,1274	0,2832	0,3210	0,2472	0,2091	0,2227	0,1988	0,2641	0,1172	0,4199	0,4171	0,3202	0,0558	0,2069
Feliformia	Felidae	Prionailurus_planiceps_NC028312_6	0,3119	0,2779	0,1279	0,2823	0,3207	0,2469	0,2094	0,2230	0,1988	0,2649	0,1172	0,4191	0,4160	0,3219	0,0572	0,2049
Feliformia	Felidae	Prionailurus_rubiginosus_NC028304_2	0,3144	0,2744	0,1260	0,2853	0,3213	0,2466	0,2094	0,2227	0,1988	0,2633	0,1169	0,4210	0,4229	0,3133	0,0517	0,2122
Feliformia	Felidae	Prionailurus_viverrinus_NC028305_1	0,3114	0,2762	0,1285	0,2839	0,3210	0,2460	0,2102	0,2227	0,1980	0,2655	0,1178	0,4188	0,4152	0,3171	0,0575	0,2102
Feliformia	Felidae	Profelis_aurata_NC028299_1	0,3135	0,2666	0,1276	0,2923	0,3244	0,2460	0,2061	0,2236	0,1972	0,2641	0,1183	0,4204	0,4191	0,2896	0,0583	0,2330
Feliformia	Felidae	Puma_concolor_NC016470_22	0,3146	0,2763	0,1268	0,2822	0,3235	0,2472	0,2066	0,2227	0,1988	0,2647	0,1175	0,4191	0,4216	0,3171	0,0564	0,2049
Feliformia	Felidae	Puma_yagouaroundi_NC028311_1	0,3170	0,2712	0,1250	0,2869	0,3252	0,2460	0,2058	0,2230	0,1983	0,2616	0,1175	0,4227	0,4274	0,3060	0,0517	0,2149
Feliformia	Felidae	Smilodon_populator_MF871700_1	0,3153	0,2690	0,1249	0,2908	0,3209	0,2506	0,2066	0,2219	0,1996	0,2665	0,1183	0,4155	0,4253	0,2899	0,0498	0,2351
Feliformia	Herpestidae	Atilax_paludinosus_T606_1	0,3160	0,2860	0,1290	0,2689	0,3213	0,2558	0,2094	0,2136	0,1983	0,2649	0,1175	0,4193	0,4285	0,3374	0,0603	0,1738
Feliformia	Herpestidae	Bdeogale_nigripes_GLC15_1	0,3160	0,2791	0,1288	0,2761	0,3232	0,2510	0,2066	0,2191	0,1969	0,2649	0,1191	0,4191	0,4279	0,3213	0,0605	0,1902
Feliformia	Herpestidae	Crossarchus_platycephalus_C7R66_1	0,3244	0,2946	0,1223	0,2588	0,3263	0,2602	0,2033	0,2102	0,1988	0,2685	0,1178	0,4149	0,4479	0,3549	0,0458	0,1514
Feliformia	Herpestidae	Cynictis_penicillata_T375_1	0,3180	0,2754	0,1268	0,2798	0,3205	0,2485	0,2094	0,2216	0,1988	0,2660	0,1172	0,4179	0,4346	0,3116	0,0539	0,1999
Feliformia	Herpestidae	Galerella_sanguinea_T378_1	0,3154	0,2831	0,1306	0,2709	0,3185	0,2572	0,2113	0,2130	0,1972	0,2672	0,1183	0,4174	0,4304	0,3251	0,0622	0,1823
Feliformia	Herpestidae	Helogale_parvula_SRR7637809_1	0,3194	0,2851	0,1271	0,2684	0,3235	0,2558	0,2058	0,2149	0,1972	0,2666	0,1197	0,4166	0,4374	0,3330	0,0558	0,1738
Feliformia	Herpestidae	Herpestes_brachyurus_KY117547_1	0,3213	0,2738	0,1241	0,2808	0,3246	0,2491	0,2058	0,2205	0,1977	0,2641	0,1172	0,4210	0,4415	0,3083	0,0494	0,2008
Feliformia	Herpestidae	Herpestes_javanicus_NC006835_1	0,3189	0,2739	0,1267	0,2806	0,3227	0,2488	0,2074	0,2211	0,1972	0,2650	0,1180	0,4198	0,4368	0,3079	0,0545	0,2009
Feliformia	Herpestidae	Herpestes_javanicus_T413_1	0,3172	0,2743	0,1280	0,2805	0,3232	0,2485	0,2072	0,2211	0,1972	0,2638	0,1186	0,4204	0,4313	0,3105	0,0583	0,1999
Feliformia	Herpestidae	Herpestes_naso_C07XAR110_1	0,3134	0,2843	0,1311	0,2712	0,3210	0,2535	0,2097	0,2158	0,1975	0,2674	0,1180	0,4171	0,4218	0,3319	0,0655	0,1808
Feliformia	Herpestidae	Herpestes_semitorquatus_MH464789_1	0,3191	0,2884	0,1255	0,2671	0,3244	0,2577	0,2049	0,2130	0,1966	0,2680	0,1183	0,4171	0,4363	0,3394	0,0533	0,1711
Feliformia	Herpestidae	Ichneumia_albicauda_T603_1	0,3183	0,2778	0,1271	0,2768	0,3221	0,2533	0,2080	0,2166	0,1977	0,2672	0,1178	0,4174	0,4351	0,3131	0,0555	0,1963
Feliformia	Herpestidae	Mungos_mungo_MMC7_1	0,3271	0,2821	0,1200	0,2708	0,3277	0,2555	0,2019	0,2149	0,1988	0,2683	0,1178	0,4152	0,4549	0,3227	0,0403	0,1822
Feliformia	Herpestidae	Mungos_mungo_SRR7704821_1	0,3230	0,2821	0,1240	0,2709	0,3257	0,2527	0,2033	0,2183	0,1977	0,2691	0,1186	0,4146	0,4454	0,3246	0,0503	0,1797
Feliformia	Herpestidae	Suricata_suricatta_SSM10_1	0,3193	0,2829	0,1249	0,2730	0,3235	0,2574	0,2038	0,2152	0,1986	0,2683	0,1178	0,4154	0,4357	0,3230	0,0530	0,1883
Feliformia	Hyaenidae	Crocuta_crocuta_NC020670_3	0,3080	0,2989	0,1354	0,2577	0,3185	0,2647	0,2102	0,2066	0,1966	0,2735	0,1194	0,4104	0,4088	0,3585	0,0767	0,1561
Feliformia	Hyaenidae	Hyaena_hyaena_NC020669_1	0,3077	0,2921	0,1352	0,2650	0,3169	0,2630	0,2111	0,2091	0,1966	0,2697	0,1183	0,4154	0,4096	0,3435	0,0764	0,1705
Feliformia	Hyaenidae	Parahyaena_brunnea_NC038159_15	0,3069	0,2974	0,1362	0,2596	0,3160	0,2647	0,2113	0,2080	0,1975	0,2708	0,1172	0,4146	0,4071	0,3569	0,0800	0,1561
Feliformia	Hyaenidae	Proteles_cristata_T393_6	0,3033	0,2992	0,1390	0,2585	0,3130	0,2624	0,2149	0,2097	0,1955	0,2719	0,1191	0,4135	0,4016	0,3632	0,0830	0,1522
Feliformia	Nandiniidae	Nandinia_binotata_NC024567_1	0,3218	0,2901	0,1174	0,2708	0,3207	0,2510	0,2061	0,2222	0,1975	0,2663	0,1169	0,4193	0,4471	0,3530	0,0292	0,1708
Feliformia	Prionodontidae	Prionodon_linsang_ERR2391707_1	0,3130	0,2496	0,1254	0,3120	0,3191	0,2358	0,2097	0,2355	0,1969	0,2627	0,1172	0,4232	0,4229	0,2502	0,0494	0,2774
Feliformia	Prionodontidae	Prionodon_pardicolor_NC024569_2	0,3095	0,2610	0,1277	0,3020	0,3185	0,2402	0,2108	0,2305	0,1947	0,2633	0,1186	0,4235	0,4152	0,2794	0,0536	0,2519
Feliformia	Viverridae	Arctictis_binturong_T605_2	0,3216	0,2428	0,1170	0,3186	0,3210	0,2283	0,2041	0,2466	0,1988	0,2627	0,1161	0,4224	0,4449	0,2374	0,0308	0,2869
Feliformia	Viverridae	Chrotogale_owstoni_T607_1	0,3232	0,2544	0,1151	0,3074	0,3244	0,2294	0,2020	0,2443	0,1977	0,2616	0,1164	0,4243	0,4474	0,2722	0,0269	0,2535
Feliformia	Viverridae	Civettictis_civetta_GLC19_1	0,3088	0,2700	0,1251	0,2961	0,3199	0,2519	0,2055	0,2227	0,1950	0,2647	0,1189	0,4216	0,4116	0,2935	0,0508	0,2441
Feliformia	Viverridae	Civettictis_civetta_NC033378_1	0,3079	0,2708	0,1261	0,2953	0,3194	0,2522	0,2062	0,2223	0,1950	0,2639	0,1189	0,4222	0,4093	0,2965	0,0531	0,2412
Feliformia	Viverridae	Cynogale_bennetti_KY117544_1	0,3251	0,2337	0,1123	0,3290	0,3294	0,2172	0,1988	0,2547	0,1981	0,2595	0,1153	0,4271	0,4477	0,2244	0,0228	0,3052
Feliformia	Viverridae	Diplogale_hosei_MH464790_1	0,3227	0,2481	0,1137	0,3156	0,3238	0,2286	0,2026	0,2451	0,1968	0,2669	0,1170	0,4193	0,4474	0,2487	0,0215	0,2824
Feliformia	Viverridae	Genetta_abyssinica_MG489822_1	0,3134	0,2715	0,1216	0,2936	0,3208	0,2496	0,2033	0,2263	0,1955	0,2612	0,1181	0,4253	0,4238	0,3037	0,0432	0,2292
Feliformia	Viverridae	Genetta_genetta_T297_1	0,3104	0,2689	0,1248	0,2959	0,3194	0,2447	0,2049	0,2311	0,1961	0,2649	0,1178	0,4213	0,4157	0,2971	0,0517	0,2355
Feliformia	Viverridae	Genetta_servalina_NC024568_2	0,3120	0,2664	0,1229	0,2986	0,3196	0,2469	0,2049	0,2286	0,1961	0,2635	0,1172	0,4232	0,4204	0,2888	0,0467	0,2441
Feliformia	Viverridae	Hemigalus_derbyanus_MH464791_1	0,3183	0,2486	0,1200	0,3131	0,3109	0,2240	0,2151	0,2501	0,1982	0,2679	0,1236	0,4104	0,4461	0,2540	0,0213	0,2787
Feliformia	Viverridae	Paguma_larvata_PDDS11_2	0,3207	0,2476	0,1180	0,3137	0,3185	0,2305	0,2055	0,2455	0,1972	0,2644	0,1178	0,4207	0,4463	0,2480	0,0308	0,2749
Feliformia	Viverridae	Paradoxurus_hermaphroditus_NC039591_1	0,3233	0,2346	0,1159	0,3262	0,3224	0,2224	0,2036	0,2516	0,1983	0,2610	0,1169	0,4238	0,4493	0,2202	0,0272	0,3033
Feliformia	Viverridae	Paradoxurus_hermaphroditus_NLNC_1	0,3232	0,2385	0,1153	0,3229	0,3213	0,2258	0,2036	0,2494	0,1983	0,2619	0,1172	0,4227	0,4502	0,2280	0,0253	0,2966
Feliformia	Viverridae	Paradoxurus_jerdoni_MH464793_1	0,3240	0,2376	0,1153	0,3232	0,3207	0,2283	0,2041	0,2469	0,1999	0,2616	0,1158	0,4227	0,4513	0,2230	0,0258	0,2999
Feliformia	Viverridae	Viverra_tangalunga_MH464792_1	0,3174	0,2726	0,1248	0,2852	0,3263	0,2427	0,2019	0,2291	0,1941	0,2685	0,1175	0,4199	0,4318	0,3066	0,0550	0,2066
Feliformia	Viverridae	Viverra_zibetha_T609_1	0,3135	0,2616	0,1212	0,3037	0,3191	0,2388	0,2066	0,2355	0,1952	0,2635	0,1178	0,4235	0,4263	0,2824	0,0392	0,2522
Feliformia	Viverridae	Viverricula_indica_KX891745_1	0,3160	0,2608	0,1176	0,3057	0,3216	0,2413	0,2036	0,2336	0,1972	0,2641	0,1166	0,4221	0,4293	0,2769	0,0325	0,2613
Feliformia	Viverridae	Viverricula_indica_KX891751_1	0,3165	0,2573	0,1168	0,3094	0,3202	0,2372	0,2047	0,2380	0,1972	0,2635	0,1166	0,4227	0,4321	0,2713	0,0292	0,2674
Feliformia	Viverridae	Viverricula_indica_NC025296_2	0,3164	0,2600	0,1172	0,3064	0,3216	0,2391	0,2033	0,2361	0,1969	0,2635	0,1166	0,4229	0,4307	0,2774	0,0317	0,2602
OUTGROUP	Manidae	Phataginus_tricuspis_NC026780	0,3142	0,2460	0,1277	0,3121	0,3235	0,2263	0,2119	0,2383	0,1969	0,2647	0,1166	0,4218	0,4221	0,2470	0,0547	0,2762
OUTGROUP	Tapiridae	Tapirus_terrestris_T358	0,3277	0,2655	0,1146	0,2922	0,3260	0,2474	0,1988	0,2277	0,1972	0,2652	0,1161	0,4216	0,4599	0,2838	0,0289	0,2274
			**0,3167**	**0,2662**	**0,1228**	**0,2943**	**0,3211**	**0,2432**	**0,2059**	**0,2297**	**0,1970**	**0,2654**	**0,1177**	**0,4200**	**0,4319**	**0,2900**	**0,0450**	**0,2331**
			**0,3033**	**0,2337**	**0,1123**	**0,2577**	**0,3109**	**0,2172**	**0,1988**	**0,2066**	**0,1941**	**0,2595**	**0,1153**	**0,4104**	**0,4016**	**0,2202**	**0,0213**	**0,1522**
			**0,3277**	**0,2992**	**0,1390**	**0,3290**	**0,3294**	**0,2647**	**0,2151**	**0,2547**	**0,1999**	**0,2735**	**0,1236**	**0,4271**	**0,4599**	**0,3632**	**0,0830**	**0,3052**
			**0,0244**	**0,0655**	**0,0267**	**0,0712**	**0,0185**	**0,0475**	**0,0162**	**0,0480**	**0,0058**	**0,0140**	**0,0083**	**0,0167**	**0,0583**	**0,1430**	**0,0617**	**0,1530**
