# Supplementary material for: Evolutionary history of Carnivora (Mammalia, Laurasiatheria) inferred from mitochondrial genomes
Source: PLoS One. 2021 Feb 16;16(2):e0240770. doi: 10.1371/journal.pone.0240770 (PMC7886153; doi:10.1371/journal.pone.0240770)
Supplement: S4 Appendix — (PDF) [file pone.0240770.s004.pdf]

S4 Appendix. Distances

Evolutionary history of Carnivora (Mammalia, Laurasiatheria) inferred from mitochondrial genomes

Alexandre Hassanin[1*], Géraldine Veron[1], Anne Ropiquet[2], Bettine Jansen van Vuuren[3],

Alexis Lécu[4], Steven M. Goodman[5], Jibran Haider[1,6,7], Trung Thanh Nguyen[1]

[1] Institut de Systématique, Évolution, Biodiversité (ISYEB), Sorbonne Université, MNHN, CNRS, EPHE, UA, Paris.

[2] Department of Natural Sciences, Faculty of Science and Technology, Middlesex University, United Kingdom.

[3] Centre for Ecological Genomics and Wildlife Conservation, Department of Zoology, University of Johannesburg, South Africa.

[4] Parc zoologique de Paris, Muséum national d'Histoire naturelle, Paris.

[5] Field Museum of Natural History, Chicago, IL, USA.

[6] Department of Wildlife Management, Pir Mehr Ali Shah, Arid Agriculture University Rawalpindi, Pakistan.

[7] Forest Parks & Wildlife Department Gilgit-Baltistan, Pakistan.

* Correspondence: alexandre.hassanin@mnhn.fr

T1	T2	p-distance
Ursus maritimus NC003428 31	Ursus maritimus GU573488 Svalbard	0,00276076
Ursus maritimus GU573488 Svalbard	Ursus arctos GU573486 5	0,00356893
Urocyon littoralis catalinae KP129018 15	Urocyon cinereoargenteus NC026723 21	0,00410691
Zalophus wollebaeki SRR4431565 1	Zalophus californianus NC008416 1	0,00491354
Ursus maritimus NC003428 31	Ursus arctos GU573486 5	0,0057911
Felis silvestris lybica KP202275 4	Felis catus NC001700 2	0,00720442
Ursus maritimus GU573488 Svalbard	Ursus arctos EU497665 29	0,01010033
Ursus thibetanus thibetanus NC011118 4	Ursus thibetanus formosanus NC009331 1	0,01010148
Prionailurus planiceps NC028312 6	Prionailurus planiceps KY682741 4	0,01070563
Ursus arctos GU573491 207	Ursus arctos AP012576 6	0,01077421
Civettictis civetta NC033378 1	Civettictis civetta GLC19 1	0,01117999
Ailurus fulgens styani NC009691 1	Ailurus fulgens NC011124 1	0,01137817
Mustela sibirica NC020637 6	Mustela sibirica AP017394 11	0,01150895
Panthera pardus japonensis KJ866876 8	Panthera pardus NC010641 5	0,01151593
Procyon lotor AB462049 4	Procyon lotor AB462046 3	0,01177737
Mustela putorius NC020638 4	Mustela eversmannii NC028013 1	0,01191277
Ursus maritimus NC003428 31	Ursus arctos EU497665 29	0,01218773
Ursus arctos GU573486 5	Ursus arctos EU497665 29	0,01245759
Ursus spelaeus NC011112 8	Ursus spelaeus EU327344 13	0,01292842
Ursus arctos pruinosus MG066703 3	Ursus arctos GU573491 207	0,01313112
Arctocephalus forsteri KT693377 17	Arctocephalus australis MG023139 1	0,01332436
Ursus thibetanus thibetanus NC011118 4	Ursus thibetanus mupinensis NC008753 2	0,01407421
Ursus arctos pruinosus MG066703 3	Ursus arctos AP012576 6	0,01420912
Panthera uncia NC010638 1	Panthera uncia KP202269 1	0,01568812
Mustela putorius NC020638 4	Mustela nigripes NC024942 1	0,0158837
Ursus thibetanus mupinensis NC008753 2	Ursus thibetanus formosanus NC009331 1	0,01663291
Panthera tigris amoyensis NC014770 2	Panthera tigris NC010642 35	0,01676429
Arctocephalus forsteri NC004023 28	Arctocephalus forsteri KT693377 17	0,01694201
Ursus maritimus GU573488 Svalbard	Ursus arctos GU573491 207	0,0172379
Felis silvestris lybica KP202275 4	Felis margarita NC028308 1	0,01730407
Mustela nigripes NC024942 1	Mustela eversmannii NC028013 1	0,01736438
Ursus maritimus GU573488 Svalbard	Ursus arctos pruinosus MG066703 3	0,0179121
Ursus thibetanus thibetanus NC011118 4	Ursus thibetanus laniger MH281753 2	0,01811571
Ursus arctos pruinosus MG066703 3	Ursus arctos EU497665 29	0,01865284
Ursus thibetanus laniger MH281753 2	Ursus thibetanus formosanus NC009331 1	0,01865414
Ursus arctos GU573491 207	Ursus arctos EU497665 29	0,01878661
Phoca vitulina NC001325 1	Phoca largha NC008430 1	0,01898862
Ursus maritimus GU573488 Svalbard	Ursus arctos AP012576 6	0,01898945
Martes zibellina NC011579 39	Martes martes T302 3	0,01904667
Ursus maritimus NC003428 31	Ursus arctos GU573491 207	0,01919063
Ursus maritimus NC003428 31	Ursus arctos pruinosus MG066703 3	0,01932622
Panthera onca NC022842 1	Panthera onca KP202264 2	0,01952673
Felis margarita NC028308 1	Felis catus NC001700 2	0,01966065
Ursus arctos GU573491 207	Ursus arctos GU573486 5	0,01966281
Ursus arctos pruinosus MG066703 3	Ursus arctos GU573486 5	0,0200677
Ursus arctos EU497665 29	Ursus arctos AP012576 6	0,02026886
Arctocephalus forsteri NC004023 28	Arctocephalus australis MG023139 1	0,02072217
Lutra lutra NC011358 9	Lutra lutra LC050126 1	0,02086278
Ursus maritimus NC003428 31	Ursus arctos AP012576 6	0,02094227
Ursus arctos GU573486 5	Ursus arctos AP012576 6	0,02114523
Ursus maritimus GU573488 Svalbard	Ursus arctos isabellinus 18B5 2	0,02181671
Melogale moschata V0735A 1	Melogale moschata NC020644 1	0,02200784
Ursus thibetanus mupinensis NC008753 2	Ursus thibetanus laniger MH281753 2	0,02255912
Pusa sibirica NC008432 2	Pusa caspica NC008431 1	0,02269055
Ursus arctos isabellinus 18B5 2	Ursus arctos EU497665 29	0,02269207
Ursus arctos isabellinus 18B5 2	Ursus arctos GU573491 207	0,02269207
Ursus arctos isabellinus 18B5 2	Ursus arctos AP012576 6	0,0233666
Ursus arctos pruinosus MG066703 3	Ursus arctos isabellinus 18B5 2	0,02336661
Ursus arctos isabellinus 18B5 2	Ursus arctos GU573486 5	0,02350124
Ursus maritimus NC003428 31	Ursus arctos isabellinus 18B5 2	0,02376944
Pusa sibirica NC008432 2	Pusa hispida NC 008433 1	0,02478088
Viverricula indica KX891751 1	Viverricula indica KX891745 1	0,02545283
Pusa sibirica NC008432 2	Halichoerus grypus NC001602 2	0,02566114
Salanoia concolor D378 1	Mungotictis decemlineata NC027828 1	0,02585336
Canis lupus familiaris NC002008 1231	Canis lupus chanco NC010340 4	0,02613532
Pusa caspica NC008431 1	Halichoerus grypus NC001602 2	0,0266041
Pusa hispida NC 008433 1	Pusa caspica NC008431 1	0,02700287
Panthera leo spelaea KX258452 2	Panthera leo NERO 19	0,02728407
Leopardus guigna NC028321 1	Leopardus geoffroyi NC028320 1	0,0274692
Canis lupus chanco NC010340 4	Canis anthus NC027956 2	0,02760988
Melogale moschata NC020644 1	Melogale moschata KP726273 1	0,02766136
Martes melampus NC009678 1	Martes martes T302 3	0,02772895
Pusa sibirica NC008432 2	Phoca largha NC008430 1	0,02801138
Canis lupus familiaris NC002008 1231	Canis anthus NC027956 2	0,02821613
Mustela sibirica NC020637 6	Mustela putorius NC020638 4	0,02867142
Melogale moschata V0735A 1	Melogale moschata KP726273 1	0,02873679
Mustela sibirica AP017394 11	Mustela nigripes NC024942 1	0,02894064
Mustela sibirica NC020637 6	Mustela nigripes NC024942 1	0,02894064
Paradoxurus hermaphroditus NLNC 1	Paradoxurus hermaphroditus NC039591 1	0,02915629
Martes zibellina NC011579 39	Martes melampus NC009678 1	0,02954438
Mustela sibirica AP017394 11	Mustela putorius NC020638 4	0,02961368
Viverricula indica NC025296 2	Viverricula indica KX891745 1	0,02962831
Pusa hispida NC 008433 1	Halichoerus grypus NC001602 2	0,02983977
Pusa caspica NC008431 1	Phoca largha NC008430 1	0,03009887
Leopardus pardalis T262 1	Leopardus pardalis NC028315 1	0,03016226
Meles leucurus NC039173 4	Meles anakuma NC009677 1	0,03041928
Viverricula indica NC025296 2	Viverricula indica KX891751 1	0,03050338
Mustela sibirica NC020637 6	Mustela eversmannii NC028013 1	0,03062323
Pusa sibirica NC008432 2	Phoca vitulina NC001325 1	0,03070495
Lynx lynx NC027083 4	Lynx canadensis NC028313 1	0,0308376
Mirounga leonina NC008422 1	Mirounga angustirostris SRR10331586 1	0,03110987
Lynx pardinus NC028319 161	Lynx canadensis NC028313 1	0,03124158
Mustela sibirica AP017394 11	Mustela eversmannii NC028013 1	0,03149818
Pusa caspica NC008431 1	Phoca vitulina NC001325 1	0,03158041
Pusa hispida NC 008433 1	Phoca largha NC008430 1	0,03286348
Phoca largha NC008430 1	Halichoerus grypus NC001602 2	0,0330044
Lynx pardinus NC028319 161	Lynx lynx NC027083 4	0,03339618
Prionailurus viverrinus NC028305 1	Prionailurus bengalensis CKM45 20	0,03366672
Pusa hispida NC 008433 1	Phoca vitulina NC001325 1	0,03387352
Phoca vitulina NC001325 1	Halichoerus grypus NC001602 2	0,03388003
Vulpes ferrilata NC027935 1	Vulpes corsac NC023958 1	0,03480778
Canis lupus chanco NC010340 4	Canis aureus KT448274 1	0,03516817

T1	T2	p-distance
Lynx lynx NC027083 4	Gulo gulo NC009685 3	0,1817112
Prionailurus planiceps KY682741 4	Canis lupus chanco NC010340 4	0,18171173
Melogale moschata KP726273 1	Conepatus chinga NC042596 1	0,18171516
Lynx canadensis NC028313 1	Eumetopias jubatus NC004030 10	0,18171641
Lynx pardinus NC028319 161	Eumetopias jubatus NC004030 10	0,18171696
Monachus monachus NC044972 5	Crocuta crocuta NC020670 3	0,18171942
Martes foina NC020643 1	Genetta abyssinica MG489822 1	0,18172349
Mustela kathiah NC023210 1	Crossarchus platycephalus C7R66 1	0,18172356
Meles meles T303 3	Xenogale naso C07XAR110 1	0,18172358
Viverricula indica KX891745 1	Lutra lutra LC050126 1	0,18172398
Mustela itatsi NC034330 19	Arctocephalus gazella BK010918 1	0,18172486
Potos flavus T414 1	Arctocephalus townsendi NC008420 1	0,18172564
Crossarchus platycephalus C7R66 1	Arctocephalus forsteri KT693377 17	0,18172604
Martes americana NC020642 1	Arctocephalus gazella BK010918 1	0,18172607
Nasua nasua NC020647 1	Erignathus barbatus NC008426 1	0,18172666
Genetta servalina NC024568 2	Enhydra lutris NC009692 1	0,18172836
Mustela kathiah NC023210 1	Arctocephalus pusillus NC008417 1	0,18173002
Mirounga leonina NC008422 1	Galidia elegans D146 1	0,18173079
Mustela putorius NC020638 4	Genetta abyssinica MG489822 1	0,18173105
Lutra lutra LC050126 1	Catopuma badia NC028300 1	0,18173154
Mustela nigripes NC024942 1	Canis anthus NC027956 2	0,18173207
Potos flavus T414 1	Arctocephalus pusillus NC008417 1	0,1817327
Martes zibellina NC011579 39	Arctotherium sp NC030174 1	0,18173391
Lutra lutra NC011358 9	Leopardus geoffroyi NC028320 1	0,18173435
Meles anakuma NC009677 1	Felis margarita NC028308 1	0,18173487
Martes americana NC020642 1	Civettictis civetta GLC19 1	0,18173499
Pusa caspica NC008431 1	Paradoxurus hermaphroditus NLNC 1	0,18173501
Neovison vison NC020641 3	Mungos mungo/gambianus SRR77048	0,1817354
Martes melampus NC009678 1	Lynx rufus NC014456 3	0,18173613
Phocarctos hookeri NC008418 1	Neovison vison NC020641 3	0,18173625
Panthera onca KP202264 2	Mustela frenata NC020640 1	0,1817369
Mustela sibirica AP017394 11	Leopardus tigrinus NC028317 1	0,18173741
Paradoxurus jerdoni MH464793 1	Mustela itatsi NC034330 19	0,18173794
Mustela eversmannii NC028013 1	Felis silvestris lybica KP202275 4	0,18173796
Civettictis civetta GLC19 1	Ailurus fulgens styani NC009691 1	0,18173853
Speothos venaticus C48 2	Erignathus barbatus NC008426 1	0,18173887
Vulpes vulpes NC008434 5	Leopardus pardalis T262 1	0,1817394
Vulpes vulpes NC008434 5	Puma yagouaroundi NC028311 1	0,18174011
Mustela itatsi NC034330 19	Catopuma badia NC028300 1	0,18174043
Ursus thibetanus mupinensis NC00875	Lutra sumatrana NC035810 1	0,18174084
Martes americana NC020642 1	Leopardus wiedii NC028318 1	0,18174095
Melogale moschata V0735A 1	Leopardus geoffroyi NC028320 1	0,18174128
Puma concolor NC016470 22	Canis aureus KT448274 1	0,18174152
Prionailurus rubiginosus NC028304 2	Canis anthus NC027956 2	0,18174182
Potos flavus T414 1	Leopardus guigna NC028321 1	0,18174185
Prionailurus viverrinus NC028305 1	Martes americana NC020642 1	0,18174192
Ursus arctos EU497665 29	Ailurus fulgens styani NC009691 1	0,18174219
Puma concolor NC016470 22	Canis latrans NC008093 7	0,18174236
Vulpes vulpes NC008434 5	Prionailurus bengalensis CKM45 20	0,18174319
Ursus arctos GU573491 207	Mustela kathiah NC023210 1	0,18174334
Ursus maritimus NC003428 31	Arctocephalus pusillus NC008417 1	0,18174355
Ursus arctos isabellinus 18B5 2	Mustela eversmannii NC028013 1	0,18174398
Ursus arctos isabellinus 18B5 2	Mustela nigripes NC024942 1	0,18174468
Ursus spelaeus EU327344 13	Eumetopias jubatus NC004030 10	0,18174508
Ursus arctos isabellinus 18B5 2	Melogale moschata NC020644 1	0,18174523
Procyon lotor AB462046 3	Prionailurus rubiginosus NC028304 2	0,18174548
Viverra zibetha T609 1	Ursus arctos isabellinus 18B5 2	0,18174554
Mustela altaica NC021751 1	Leopardus jacobita NC028322 1	0,18174598
Martes flavigula NC012141 3	Civettictis civetta NC033378 1	0,18174653
Paradoxurus jerdoni MH464793 1	Arctodus simus NC011116 1	0,18174711
Ursus thibetanus formosanus NC0093	Martes zibellina NC011579 39	0,1817472
Lutra lutra LC050126 1	Arctocephalus forsteri NC004023 28	0,18174732
Mustela altaica NC021751 1	Catopuma temminckii NC027115 41	0,18174821
Zalophus californianus NC008416 1	Homotherium latidens MF871702 3	0,18174857
Mustela nivalis T306 5	Canis aureus KT448274 1	0,18174887
Panthera uncia NC010638 1	Monachus schauinslandi NC008421 1	0,18174888
Ursus thibetanus formosanus NC0093	Fossa fossana D350 1	0,18174888
Procyon lotor AB462049 4	Prionailurus bengalensis CKM45 20	0,18174908
Neovison vison NC020641 3	Felis silvestris lybica KP202275 4	0,18174954
Prionailurus planiceps NC028312 6	Canis aureus KT448274 1	0,1817497
Ursus arctos GU573486 5	Mustela eversmannii NC028013 1	0,18175013
Ursus arctos GU573486 5	Mustela nigripes NC024942 1	0,18175083
Martes foina NC020643 1	Felis catus NC001700 2	0,18175267
Puma yagouaroundi NC028311 1	Bassariscus sumichrasti SRX1099089 1	0,18175267
Ursus thibetanus formosanus NC0093	Arctocephalus pusillus NC008417 1	0,18175425
Ursus arctos AP012576 6	Meles leucurus NC039173 4	0,18175487
Phoca vitulina NC001325 1	Chrysocyon brachyurus NC024172 1	0,18175505
Martes flavigula NC012141 3	Hemigalus derbyanus MH464791 1	0,18175626
Viverra zibetha T609 1	Ursus thibetanus formosanus NC0093	0,18175647
Ursus spelaeus NC011112 8	Mustela itatsi NC034330 19	0,18175744
Ictonyx striatus T299 1	Arctodus simus NC011116 1	0,18175825
Proteles cristata T393 6	Phoca largha NC008430 1	0,181759
Viverricula indica KX891751 1	Ursus thibetanus laniger MH281753 2	0,1817603
Helarctos malayanus NC009968 2	Genetta servalina NC024568 2	0,1817605
Poecilogale albinucha T602 1	Arctocephalus forsteri NC004023 28	0,18176156
Zalophus californianus NC008416 1	Ursus maritimus NC003428 31	0,18176273
Vulpes ferrilata NC027935 1	Monachus monachus NC044972 5	0,18176518
Ursus thibetanus thibetanus NC01111	Leopardus geoffroyi NC028320 1	0,1817676
Ursus thibetanus mupinensis NC00875	Smilodon populator MF871700 1	0,18176839
Cystophora cristata NC008427 1	Cuon alpinus NC013445 3	0,18177724
Lynx lynx NC027083 4	Canis lupus chanco NC010340 4	0,181778
Puma yagouaroundi NC028311 1	Martes flavigula NC012141 3	0,18177825
Leopardus wiedii NC028318 1	Gulo gulo NC009685 3	0,18177845
Halichoerus grypus NC001602 2	Canis anthus NC027956 2	0,18177937
Gulo gulo NC009685 3	Felis silvestris lybica KP202275 4	0,18177958
Martes martes T302 3	Genetta abyssinica MG489822 1	0,18178163
Martes flavigula NC012141 3	Felis margarita NC028308 1	0,18178202
Puma concolor NC016470 22	Canis lupus chanco NC010340 4	0,18178236
Prionailurus bengalensis CKM45 20	Martes flavigula NC012141 3	0,1817832
Smilodon populator MF871700 1	Ailuropoda melanoleuca NC009492 5	0,18178718
Meles leucurus NC039173 4	Crossarchus platycephalus C7R66 1	0,18178859
Mungos mungo/gambianus SRR77048	Callorhinus ursinus NC008415 1	0,18179014

Prionailurus bengalensis NC028301 12	Prionailurus bengalensis CKM45 20	0,03575401
Canis aureus KT448274 1	Canis anthus NC027956 2	0,03596906
Canis lupus familiaris NC002008 1231	Canis aureus KT448274 1	0,03631193
Leopardus wiedii NC028318 1	Leopardus pardalis T262 1	0,03635629
Martes melampus NC009678 1	Martes americana NC020642 1	0,03647621
Martes martes T302 3	Martes americana NC020642 1	0,03701676
Leopardus wiedii NC028318 1	Leopardus pardalis NC028315 1	0,03723153
Martes zibellina NC011579 39	Martes americana NC020642 1	0,03916818
Prionailurus viverrinus NC028305 1	Prionailurus bengalensis NC028301 12	0,04033174
Canis latrans NC008093 7	Canis aureus KT448274 1	0,04035241
Canis lupus familiaris NC002008 1231	Canis latrans NC008093 7	0,04175084
Canis lupus chanco NC010340 4	Canis latrans NC008093 7	0,04215762
Canis latrans NC008093 7	Canis anthus NC027956 2	0,04268498
Urva javanica T413 1	Urva javanica/auropunctata NC006835 1	0,04478869
Mustela nigripes NC024942 1	Mustela itatsi NC034330 19	0,04509355
Arctocephalus gazella BK010918 1	Arctocephalus forsteri KT693377 17	0,04542396
Leptonychotes weddellii NC008424 1	Hydrurga leptonyx NC008425 1	0,04578814
Mustela putorius NC020638 4	Mustela itatsi NC034330 19	0,0460358
Genetta servalina NC024568 2	Genetta genetta T297 1	0,04646155
Arctocephalus gazella BK010918 1	Arctocephalus forsteri NC004023 28	0,04657369
Mustela itatsi NC034330 19	Mustela eversmannii NC028013 1	0,04664154
Arctocephalus gazella BK010918 1	Arctocephalus australis MG023139 1	0,04676985
Mustela sibirica NC020637 6	Mustela itatsi NC034330 19	0,04691076
Mustela sibirica AP017394 11	Mustela itatsi NC034330 19	0,04704536
Arctocephalus townsendi NC008420 1	Arctocephalus australis MG023139 1	0,04777927
Arctocephalus townsendi NC008420 1	Arctocephalus forsteri KT693377 17	0,04892328
Arctocephalus townsendi NC008420 1	Arctocephalus gazella BK010918 1	0,04892332
Leopardus tigrinus NC028317 1	Leopardus guigna NC028321 1	0,04955228
Lutra sumatrana NC035810 1	Lutra lutra NC011358 9	0,04959957
Arctocephalus townsendi NC008420 1	Arctocephalus forsteri NC004023 28	0,05055553
Ursus spelaeus EU327344 13	Ursus maritimus GU573488 Svalbard	0,05077594
Ursus spelaeus NC011112 8	Ursus maritimus GU573488 Svalbard	0,05111229
Lutra sumatrana NC035810 1	Lutra lutra LC050126 1	0,05128205
Leopardus tigrinus NC028317 1	Leopardus geoffroyi NC028320 1	0,0516394
Ursus spelaeus EU327344 13	Ursus arctos EU497665 29	0,05178591
Ursus spelaeus EU327344 13	Ursus arctos GU573486 5	0,05199051
Ursus spelaeus NC011112 8	Ursus arctos GU573486 5	0,05232691
Ursus spelaeus EU327344 13	Ursus maritimus NC003428 31	0,05259421
Ursus spelaeus NC011112 8	Ursus maritimus NC003428 31	0,05266117
Ursus spelaeus EU327344 13	Ursus arctos GU573491 207	0,05266176
Ursus spelaeus EU327344 13	Ursus arctos pruinosus MG066703 3	0,05273114
Ursus spelaeus NC011112 8	Ursus arctos EU497665 29	0,05279567
Ursus spelaeus NC011112 8	Ursus arctos GU573491 207	0,05279602
Ursus spelaeus EU327344 13	Ursus arctos AP012576 6	0,0531354
Ursus spelaeus NC011112 8	Ursus arctos pruinosus MG066703 3	0,05353892
Ursus spelaeus NC011112 8	Ursus arctos AP012576 6	0,05353913
Ursus spelaeus NC011112 8	Ursus arctos isabellinus 18B5 2	0,05367114
Panthera pardus japonensis KJ866876 8	Panthera leo NERO 19	0,05374338
Ursus spelaeus EU327344 13	Ursus arctos isabellinus 18B5 2	0,05400827
Panthera pardus japonensis KJ866876 8	Panthera leo spelaea KX258452 2	0,05442771
Panthera pardus NC010641 5	Panthera leo NERO 19	0,05516832
Panthera pardus NC010641 5	Panthera leo spelaea KX258452 2	0,05517932
Parahyaena brunnea NC038159 15	Hyaena hyaena NC020669 1	0,05588097
Zalophus wollebaeki SRR4431565 1	Eumetopias jubatus NC004030 10	0,05621317
Zalophus californianus NC008416 1	Eumetopias jubatus NC004030 10	0,05628484
Prionailurus planiceps KY682741 4	Prionailurus bengalensis CKM45 20	0,05682891
Prionailurus planiceps KY682741 4	Prionailurus bengalensis NC028301 12	0,05696202
Prionailurus planiceps NC028312 6	Prionailurus bengalensis NC028301 12	0,05736601
Lutrogale perspicillata NC035811 1	Aonyx cinerea NC035814 2	0,05801191
Prionailurus planiceps NC028312 6	Prionailurus bengalensis CKM45 20	0,05817562
Felis silvestris lybica KP202275 4	Felis chaus NC028307 1	0,0586453
Paradoxurus jerdoni MH464793 1	Paradoxurus hermaphroditus NLNC 1	0,05864925
Salanoia concolor D378 1	Galidictis fasciata DM333 1	0,05911264
Prionailurus rubiginosus NC028304 2	Prionailurus bengalensis NC028301 12	0,05931861
Phoca groenlandica NC008429 54	Phoca fasciata NC008428 1	0,05945728
Prionailurus rubiginosus NC028304 2	Prionailurus bengalensis CKM45 20	0,05965703
Phocarctos hookeri NC008418 1	Arctocephalus australis MG023139 1	0,05975774
Catopuma temminckii NC027115 41	Catopuma badia NC028300 1	0,06005925
Phocarctos hookeri NC008418 1	Arctocephalus forsteri KT693377 17	0,0602961
Felis chaus NC028307 1	Felis catus NC001700 2	0,06039591
Neophoca cinerea NC008419 1	Arctocephalus australis MG023139 1	0,06049798
Prionailurus viverrinus NC028305 1	Prionailurus planiceps KY682741 4	0,06059916
Mungotictis decemlineata NC027828 1	Galidictis fasciata DM333 1	0,06072847
Neophoca cinerea NC008419 1	Arctocephalus forsteri KT693377 17	0,06076716
Felis margarita NC028308 1	Felis chaus NC028307 1	0,06100189
Felis silvestris lybica KP202275 4	Felis nigripes NC028309 1	0,06127121
Paradoxurus jerdoni MH464793 1	Paradoxurus hermaphroditus NC039591 1	0,06134267
Arctocephalus pusillus NC008417 1	Arctocephalus australis MG023139 1	0,06144257
Neophoca cinerea NC008419 1	Arctocephalus townsendi NC008420 1	0,0615074
Neophoca cinerea NC008419 1	Arctocephalus gazella BK010918 1	0,0615747
Mustela nivalis T306 5	Mustela altaica NC021751 1	0,06158713
Meles meles T303 3	Meles leucurus NC039173 4	0,06164614
Prionailurus viverrinus NC028305 1	Prionailurus planiceps NC028312 6	0,06181117
Felis nigripes NC028309 1	Felis chaus NC028307 1	0,06201185
Leopardus wiedii NC028318 1	Leopardus jacobita NC028322 1	0,06214233
Felis nigripes NC028309 1	Felis catus NC001700 2	0,06221384
Arctocephalus pusillus NC008417 1	Arctocephalus forsteri KT693377 17	0,06225014
Lobodon carcinophaga NC008423 1	Leptonychotes weddellii NC008424 1	0,06235204
Prionailurus rubiginosus NC028304 2	Prionailurus planiceps KY682741 4	0,062505
Leopardus pardalis NC028315 1	Leopardus jacobita NC028322 1	0,06268094
Leopardus pardalis T262 1	Leopardus colocolo NC028314 1	0,0626886
Prionailurus viverrinus NC028305 1	Prionailurus rubiginosus NC028304 2	0,06275356
Neophoca cinerea NC008419 1	Arctocephalus forsteri NC004023 28	0,06277481
Phocarctos hookeri NC008418 1	Arctocephalus townsendi NC008420 1	0,06298789
Leopardus pardalis T262 1	Leopardus jacobita NC028322 1	0,06301757
Mungos mungo/gambianus SRR7704821 1	Mungos mungo MMC7 1	0,06302324
Phocarctos hookeri NC008418 1	Neophoca cinerea NC008419 1	0,06305518
Phocarctos hookeri NC008418 1	Arctocephalus gazella BK010918 1	0,06305522
Felis nigripes NC028309 1	Felis margarita NC028308 1	0,06315648
Prionailurus rubiginosus NC028304 2	Prionailurus planiceps NC028312 6	0,06335847
Leopardus pardalis NC028315 1	Leopardus colocolo NC028314 1	0,0634294
Phocarctos hookeri NC008418 1	Arctocephalus forsteri NC004023 28	0,06344817
Arctocephalus pusillus NC008417 1	Arctocephalus gazella BK010918 1	0,06346168

Mustela sibirica AP017394 11	Conepatus chinga NC042596 1	0,18179241
Tremarctos ornatus NC009969 2	Meles leucurus NC039173 4	0,1817939
Martes zibellina NC011579 39	Canis latrans NC008093 7	0,18179442
Lycalopex sechurae KT448284 1	Homotherium latidens MF871702 3	0,18179512
Leopardus colocolo NC028314 1	Gulo gulo NC009685 3	0,18179622
Taxidea taxus NC020646 1	Cynictis penicillata T375 1	0,18179695
Tremarctos ornatus NC009969 2	Neophoca cinerea NC008419 1	0,18179712
Neophoca cinerea NC008419 1	Ailurus fulgens NC011124 1	0,1817973
Mustela altaica NC021751 1	Conepatus chinga NC042596 1	0,18179779
Procyon lotor AB462049 4	Neophoca cinerea NC008419 1	0,1817984
Lycaon pictus NC028427 2	Genetta abyssinica MG489822 1	0,18179874
Mustela putorius NC020638 4	Canis latrans NC008093 7	0,18179874
Puma yagouaroundi NC028311 1	Lutra lutra NC011358 9	0,18179911
Genetta servalina NC024568 2	Conepatus chinga NC042596 1	0,18179999
Viverricula indica KX891745 1	Meles anakuma NC009677 1	0,18180054
Viverra tangalunga MH464792 1	Otaria byronia OTAB 1	0,18180111
Mungos mungo MMC7 1	Bassariscus sumichrasti SRX1099089 1	0,18180121
Ommatophoca rossii AY377287etc 1	Canis lupus chanco NC010340 4	0,18180154
Otocyon megalotis SAF1 2	Mirounga leonina NC008422 1	0,18180194
Viverra zibetha T609 1	Meles meles T303 3	0,18180194
Felis silvestris lybica KP202275 4	Ailurus fulgens styani NC009691 1	0,18180206
Pusa caspica NC008431 1	Paradoxurus jerdoni MH464793 1	0,18180223
Vulpes corsac NC023958 1	Viverricula indica KX891751 1	0,18180329
Vulpes ferrilata NC027935 1	Viverricula indica KX891745 1	0,18180367
Prionailurus bengalensis CKM45 20	Lutra lutra NC011358 9	0,18180379
Phoca groenlandica NC008429 54	Canis anthus NC027956 2	0,18180387
Crossarchus platycephalus C7R66 1	Canis aureus KT448274 1	0,18180425
Paradoxurus hermaphroditus NLNC 1	Mustela putorius NC020638 4	0,18180434
Viverra tangalunga MH464792 1	Paguma larvata PDDS11 2	0,18180473
Mustela itatsi NC034330 19	Leopardus tigrinus NC028317 1	0,18180487
Tremarctos ornatus NC009969 2	Leptailurus serval NC028316 1	0,1818051
Mustela itatsi NC034330 19	Felis silvestris lybica KP202275 4	0,18180536
Mustela kathiah NC023210 1	Felis chaus NC028307 1	0,18180563
Melogale moschata KP726273 1	Felis nigripes NC028309 1	0,18180679
Prionailurus planiceps KY682741 4	Martes americana NC020642 1	0,18180738
Mustela sibirica AP017394 11	Leopardus guigna NC028321 1	0,18180762
Paradoxurus hermaphroditus NLNC 1	Ailurus fulgens NC011124 1	0,18180799
Melogale moschata KP726273 1	Leopardus guigna NC028321 1	0,1818084
Leopardus wiedii NC028318 1	Canis latrans NC008093 7	0,18180846
Otaria byronia OTAB 1	Ailurus fulgens NC011124 1	0,18180847
Viverricula indica KX891745 1	Mustela altaica NC021751 1	0,18180856
Ursus arctos GU573491 207	Mustela sibirica AP017394 11	0,18180859
Otocolobus manul NC028323 1	Canis anthus NC027956 2	0,18181042
Ursus arctos EU497665 29	Mustela kathiah NC023210 1	0,18181103
Ursus maritimus NC003428 31	Mustela eversmannii NC028013 1	0,18181124
Ursus maritimus GU573488 Svalbard	Mustela eversmannii NC028013 1	0,18181133
Ursus maritimus GU573488 Svalbard	Mustela nigripes NC024942 1	0,18181203
Procyon lotor AB462049 4	Leopardus wiedii NC028318 1	0,18181315
Mustela altaica NC021751 1	Felis catus NC001700 2	0,18181334
Phoca largha NC008430 1	Paradoxurus hermaphroditus NLNC 1	0,18181364
Phoca vitulina NC001325 1	Paradoxurus hermaphroditus NLNC 1	0,18181385
Leptailurus serval NC028316 1	Arctocephalus pusillus NC008417 1	0,18181394
Melursus ursinus NC009970 2	Martes melampus NC009678 1	0,18181483
Ursus thibetanus thibetanus NC01111	Callorhinus ursinus NC008415 1	0,18181515
Leopardus pardalis NC028315 1	Canis aureus KT448274 1	0,18181527
Viverricula indica NC025296 2	Mustela nivalis T306 5	0,18181564
Lynx lynx NC027083 4	Lycaon pictus NC028427 2	0,18181656
Zalophus wollebaeki SRR4431565 1	Canis adustus KT448271 1	0,18181705
Lynx rufus NC014456 3	Ictonyx striatus T299 1	0,18181929
Leopardus geoffroyi NC028320 1	Arctodus simus NC011116 1	0,18182045
Prionailurus bengalensis CKM45 20	Arctodus simus NC011116 1	0,18182063
Ursus spelaeus EU327344 13	Mustela itatsi NC034330 19	0,18182534
Ursus spelaeus NC011112 8	Neophoca cinerea NC008419 1	0,18182687
Ursus americanus JX196366 3	Martes flavigula NC012141 3	0,18182817
Mustela eversmannii NC028013 1	Canis lupus familiaris NC002008 1231	0,18183127
Poecilogale albinucha T602 1	Mephitis mephitis NC020648 1	0,18183172
Ommatophoca rossii AY377287etc 1	Ailuropoda melanoleuca NC009492 5	0,18183233
Zalophus wollebaeki SRR4431565 1	Ursus spelaeus EU327344 13	0,18183373
Melogale moschata KP726273 1	Eumetopias jubatus NC004030 10	0,18183407
Prionodon linsang ERR2391707 1	Monachus monachus NC044972 5	0,18183528
Viverricula indica KX891745 1	Martes flavigula NC012141 3	0,18183583
Mustela nivalis T306 5	Canis lupus familiaris NC002008 1231	0,18184085
Spilogale putorius NC010497 1	Homotherium latidens MF871702 3	0,18184203
Martes americana NC020642 1	Civettictis civetta NC033378 1	0,18184219
Procyon lotor AB462049 4	Canis adustus KT448271 1	0,18184271
Mustela sibirica NC020637 6	Civettictis civetta NC033378 1	0,18184395
Leopardus pardalis T262 1	Gulo gulo NC009685 3	0,18184544
Prionailurus rubiginosus NC028304 2	Gulo gulo NC009685 3	0,18184608
Chrotogale owstoni T607 1	Callorhinus ursinus NC008415 1	0,18184812
Prionailurus bengalensis CKM45 20	Gulo gulo NC009685 3	0,18184993
Phoca groenlandica NC008429 54	Panthera pardus NC010641 5	0,1818514
Viverra tangalunga MH464792 1	Diplogale hosei MH464790 1	0,18185355
Martes melampus NC009678 1	Fossa fossana D350 1	0,18185379
Pusa hispida NC 008433 1	Cuon alpinus NC013445 3	0,18185484
Meles meles T303 3	Arctocephalus gazella BK010918 1	0,18185523
Meles meles T303 3	Crossarchus platycephalus C7R66 1	0,18185607
Crossarchus platycephalus C7R66 1	Callorhinus ursinus NC008415 1	0,18185847
Mustela kathiah NC023210 1	Urva semitorquata MH464789 1	0,18185972
Vulpes vulpes NC008434 5	Mirounga leonina NC008422 1	0,1818624
Tremarctos ornatus NC009969 2	Bassariscus sumichrasti SRX1099089 1	0,18186343
Melogale moschata NC020644 1	Chrotogale owstoni T607 1	0,18186432
Mustela sibirica AP017394 11	Canis latrans NC008093 7	0,18186608
Lutra lutra LC050126 1	Leptailurus serval NC028316 1	0,18186678
Felis catus NC001700 2	Enhydra lutris NC009692 1	0,18186827
Felis margarita NC028308 1	Enhydra lutris NC009692 1	0,18186827
Felis silvestris lybica KP202275 4	Arctonyx collaris NC020645 1	0,18186925
Chrotogale owstoni T607 1	Arctodus simus NC011116 1	0,18186995
Prionailurus planiceps KY682741 4	Callorhinus ursinus NC008415 1	0,18187062
Viverricula indica KX891745 1	Mustela putorius NC020638 4	0,18187085
Lutra sumatrana NC035810 1	Catopuma badia NC028300 1	0,1818711
Lynx rufus NC014456 3	Lutra sumatrana NC035810 1	0,18187135
Mustela eversmannii NC028013 1	Genetta servalina NC024568 2	0,18187162
Paradoxurus jerdoni MH464793 1	Mustela nigripes NC024942 1	0,1818721

Leopardus wiedii NC028318 1	Leopardus colocolo NC028314 1	0,06356341
Meles meles T303 3	Meles anakuma NC009677 1	0,06406891
Arctocephalus townsendi NC008420 1	Arctocephalus pusillus NC008417 1	0,06420177
Leopardus jacobita NC028322 1	Leopardus colocolo NC028314 1	0,0644389
Lobodon carcinophaga NC008423 1	Hydrurga leptonyx NC008425 1	0,06450744
Arctocephalus pusillus NC008417 1	Arctocephalus forsteri NC004023 28	0,0645345
Leopardus wiedii NC028318 1	Leopardus tigrinus NC028317 1	0,06470073
Panthera onca NC022842 1	Panthera leo NERO 19	0,06492936
Leopardus pardalis NC028315 1	Leopardus geoffroyi NC028320 1	0,06497004
Panthera pardus NC010641 5	Panthera onca NC022842 1	0,06499299
Leopardus pardalis NC028315 1	Leopardus guigna NC028321 1	0,06510469
Panthera uncia KP202269 1	Panthera leo NERO 19	0,06532627
Leopardus tigrinus NC028317 1	Leopardus pardalis T262 1	0,065374
Leopardus wiedii NC028318 1	Leopardus geoffroyi NC028320 1	0,06550865
Leopardus wiedii NC028318 1	Leopardus guigna NC028321 1	0,06557598
Genetta servalina NC024568 2	Genetta abyssinica MG489822 1	0,06579403
Otaria byronia OTAB 1	Arctocephalus australis MG023139 1	0,06581736
Martes melampus NC009678 1	Martes foina NC020643 1	0,06582569
Phocarctos hookeri NC008418 1	Arctocephalus pusillus NC008417 1	0,06595154
Panthera onca NC022842 1	Panthera leo spelaea KX258452 2	0,06595208
Neophoca cinerea NC008419 1	Arctocephalus pusillus NC008417 1	0,0660188
Leopardus tigrinus NC028317 1	Leopardus pardalis NC028315 1	0,06611459
Leopardus pardalis T262 1	Leopardus guigna NC028321 1	0,06631657
Otaria byronia OTAB 1	Arctocephalus forsteri KT693377 17	0,06662494
Leopardus pardalis T262 1	Leopardus geoffroyi NC028320 1	0,06672053
Panthera uncia KP202269 1	Panthera leo spelaea KX258452 2	0,06675241
Otaria byronia OTAB 1	Arctocephalus gazella BK010918 1	0,06675974
Lynx rufus NC014456 3	Lynx canadensis NC028313 1	0,06692702
Panthera uncia KP202269 1	Panthera onca NC022842 1	0,06693207
Martes martes T302 3	Martes foina NC020643 1	0,06703719
Lynx rufus NC014456 3	Lynx lynx NC027083 4	0,06706168
Panthera uncia KP202269 1	Panthera pardus japonensis KJ866876 8	0,06712901
Panthera uncia KP202269 1	Panthera pardus NC010641 5	0,0673449
Martes foina NC020643 1	Martes americana NC020642 1	0,06750403
Martes zibellina NC011579 39	Martes foina NC020643 1	0,06750407
Leopardus jacobita NC028322 1	Leopardus guigna NC028321 1	0,06759577
Otaria byronia OTAB 1	Arctocephalus forsteri NC004023 28	0,06763686
Panthera pardus japonensis KJ866876 8	Panthera onca NC022842 1	0,06774062
Lynx rufus NC014456 3	Lynx pardinus NC028319 161	0,06827363
Pusa sibirica NC008432 2	Phoca fasciata NC008428 1	0,0683432
Otaria byronia OTAB 1	Arctocephalus pusillus NC008417 1	0,06851067
Otaria byronia OTAB 1	Arctocephalus townsendi NC008420 1	0,06864396
Profelis aurata NC028299 1	Caracal caracal NC028306 1	0,06867762
Pusa caspica NC008431 1	Phoca fasciata NC008428 1	0,06874716
Zalophus wollebaeki SRR4431565 1	Arctocephalus australis MG023139 1	0,0688474
Leopardus jacobita NC028322 1	Leopardus geoffroyi NC028320 1	0,06887498
Leopardus guigna NC028321 1	Leopardus colocolo NC028314 1	0,06888319
Zalophus californianus NC008416 1	Arctocephalus australis MG023139 1	0,06892014
Pusa hispida NC 008433 1	Phoca fasciata NC008428 1	0,06902138
Panthera uncia NC010638 1	Panthera leo NERO 19	0,06909757
Zalophus wollebaeki SRR4431565 1	Arctocephalus gazella BK010918 1	0,06918448
Leopardus tigrinus NC028317 1	Leopardus jacobita NC028322 1	0,06934626
Zalophus californianus NC008416 1	Arctocephalus gazella BK010918 1	0,06939162
Phoca fasciata NC008428 1	Halichoerus grypus NC001602 2	0,06944096
Ommatophoca rossii AY377287etc 1	Leptonychotes weddellii NC008424 1	0,06944294
Eumetopias jubatus NC004030 10	Arctocephalus pusillus NC008417 1	0,06947235
Vulpes vulpes NC008434 5	Vulpes corsac NC023958 1	0,06948091
Zalophus wollebaeki SRR4431565 1	Arctocephalus forsteri KT693377 17	0,06952045
Genetta genetta T297 1	Genetta abyssinica MG489822 1	0,06954803
Zalophus californianus NC008416 1	Arctocephalus forsteri KT693377 17	0,06959324
Zalophus californianus NC008416 1	Arctocephalus pusillus NC008417 1	0,0697974
Leopardus tigrinus NC028317 1	Leopardus colocolo NC028314 1	0,06989313
Leopardus geoffroyi NC028320 1	Leopardus colocolo NC028314 1	0,07002763
Zalophus californianus NC008416 1	Arctocephalus forsteri NC004023 28	0,07007688
Pusa hispida NC 008433 1	Phoca groenlandica NC008429 54	0,0700992
Zalophus wollebaeki SRR4431565 1	Arctocephalus pusillus NC008417 1	0,07012865
Zalophus wollebaeki SRR4431565 1	Arctocephalus forsteri NC004023 28	0,07013857
Pusa sibirica NC008432 2	Phoca groenlandica NC008429 54	0,07016126
Panthera onca KP202264 2	Panthera leo NERO 19	0,07031175
Eumetopias jubatus NC004030 10	Arctocephalus forsteri KT693377 17	0,07041398
Vulpes vulpes NC008434 5	Vulpes ferrilata NC027935 1	0,07042348
Phocarctos hookeri NC008418 1	Otaria byronia OTAB 1	0,0704608
Eumetopias jubatus NC004030 10	Arctocephalus australis MG023139 1	0,07048127
Pusa caspica NC008431 1	Phoca groenlandica NC008429 54	0,07049789
Panthera uncia NC010638 1	Panthera onca NC022842 1	0,07050087
Eumetopias jubatus NC004030 10	Arctocephalus gazella BK010918 1	0,07061574
Phoca groenlandica NC008429 54	Halichoerus grypus NC001602 2	0,07105744
Panthera uncia NC010638 1	Panthera leo spelaea KX258452 2	0,07106335
Urva javanica/auropunctata NC006835 1	Urva brachyura KY117547 1	0,07119344
Canis mesomelas KT448280 1	Canis latrans NC008093 7	0,07119527
Panthera uncia KP202269 1	Panthera onca KP202264 2	0,07130353
Phoca largha NC008430 1	Phoca fasciata NC008428 1	0,07158
Panthera onca KP202264 2	Panthera leo spelaea KX258452 2	0,07167238
Urva javanica T413 1	Urva brachyura KY117547 1	0,07184218
Phoca largha NC008430 1	Phoca groenlandica NC008429 54	0,07198381
Phoca vitulina NC001325 1	Phoca groenlandica NC008429 54	0,07205139
Otaria byronia OTAB 1	Neophoca cinerea NC008419 1	0,07207606
Ursus thibetanus thibetanus NC011118 4	Ursus americanus JX196366 3	0,0722634
Panthera uncia NC010638 1	Panthera pardus japonensis KJ866876 8	0,07231349
Ursus thibetanus mupinensis NC008753 2	Ursus americanus JX196366 3	0,07233164
Phoca vitulina NC001325 1	Phoca fasciata NC008428 1	0,07238831
Panthera uncia NC010638 1	Panthera pardus NC010641 5	0,07239576
Ursus thibetanus formosanus NC009331 1	Ursus americanus JX196366 3	0,0724013
Zalophus californianus NC008416 1	Arctocephalus townsendi NC008420 1	0,07242002
Eumetopias jubatus NC004030 10	Arctocephalus forsteri NC004023 28	0,07265987
Zalophus californianus NC008416 1	Phocarctos hookeri NC008418 1	0,07289109
Ommatophoca rossii AY377287etc 1	Lobodon carcinophaga NC008423 1	0,07298877
Zalophus wollebaeki SRR4431565 1	Arctocephalus townsendi NC008420 1	0,07302011
Zalophus californianus NC008416 1	Neophoca cinerea NC008419 1	0,07309761
Zalophus wollebaeki SRR4431565 1	Neophoca cinerea NC008419 1	0,07329376
Cynictis penicillata T375 1	Bdeogale nigripes GLC15 1	0,07339079
Panthera pardus NC010641 5	Panthera onca KP202264 2	0,07340538
Ursus thibetanus laniger MH281753 2	Ursus americanus JX196366 3	0,07341386

Ursus thibetanus mupinensis NC00875	Arctocephalus forsteri KT693377 17	0,18187225
Mustela nigripes NC024942 1	Caracal caracal NC028306 1	0,18187346
Mustela itatsi NC034330 19	Caracal caracal NC028306 1	0,18187349
Melogale moschata KP726273 1	Felis silvestris lybica KP202275 4	0,18187359
Genetta servalina NC024568 2	Canis latrans NC008093 7	0,1818736
Viverra tangalunga MH464792 1	Genetta genetta T297 1	0,18187498
Ursus arctos EU497665 29	Mustela sibirica AP017394 11	0,18187599
Ursus arctos EU497665 29	Mustela sibirica NC020637 6	0,18187599
Nyctereutes procyonoides NC013700 3	Lynx rufus NC014456 3	0,18187608
Nyctereutes procyonoides NC013700 3	Leopardus pardalis NC028315 1	0,18187666
Poecilogale albinucha T602 1	Paradoxurus jerdoni MH464793 1	0,18187699
Vulpes ferrilata NC027935 1	Monachus schauinslandi NC008421 1	0,18187715
Prionailurus bengalensis NC028301 12	Canis anthus NC027956 2	0,18187726
Prionailurus planiceps NC028312 6	Mustela altaica NC021751 1	0,18188047
Martes pennanti NC020664 16	Leopardus jacobita NC028322 1	0,18188135
Lynx canadensis NC028313 1	Arctodus simus NC011116 1	0,18188262
Leopardus tigrinus NC028317 1	Bassariscus sumichrasti SRX1099089 1	0,18188287
Felis catus NC001700 2	Arctodus simus NC011116 1	0,18188328
Civettictis civetta GLC19 1	Canis aureus KT448274 1	0,18188348
Pardofelis marmorata NLN3 2	Martes foina NC020643 1	0,1818869
Genetta servalina NC024568 2	Canis aureus KT448274 1	0,18188974
Viverra zibetha T609 1	Ursus spelaeus EU327344 13	0,18189429
Canis aureus KT448274 1	Acinonyx jubatus NC005212 3	0,18189622
Felis chaus NC028307 1	Chrysocyon brachyurus NC024172 1	0,1818981
Suricata suricatta SSM10 1	Monachus monachus NC044972 5	0,18190111
Martes flavigula NC012141 3	Atilax paludinosus T606 1	0,18190391
Mustela putorius NC020638 4	Civettictis civetta NC033378 1	0,18191174
Ursus arctos GU573491 207	Homotherium latidens MF871702 3	0,18191196
Neovison vison NC020641 3	Urva javanica/auropunctata NC006835	0,18191251
Martes flavigula NC012141 3	Galidictis fasciata DM333 1	0,1819129
Phoca groenlandica NC008429 54	Neofelis nebulosa NC008450 3	0,18191546
Martes flavigula NC012141 3	Leopardus pardalis NC028315 1	0,18191738
Erignathus barbatus NC008426 1	Tapirus terrestris T358	0,18191811
Nasua nasua NC020647 1	Arctonyx collaris NC020645 1	0,18191884
Viverra tangalunga MH464792 1	Nasua nasua NC020647 1	0,18192171
Zalophus californianus NC008416 1	Acinonyx jubatus NC005212 3	0,18192266
Panthera leo spelaea KX258452 2	Canis adustus KT448271 1	0,18192296
Crossarchus platycephalus C7R66 1	Arctonyx collaris NC020645 1	0,18192354
Martes americana NC020642 1	Crossarchus platycephalus C7R66 1	0,18192491
Mustela putorius NC020638 4	Arctocephalus townsendi NC008420 1	0,18192492
Martes zibellina NC011579 39	Fossa fossana D350 1	0,18192545
Nandinia binotata NC024567 1	Bassaricyon neblina SRX1097850 1	0,18192786
Melogale moschata V0735A 1	Hemigalus derbyanus MH464791 1	0,18192834
Martes flavigula NC012141 3	Acinonyx jubatus NC005212 3	0,18192954
Arctocephalus townsendi NC008420 1	Aonyx cinerea NC035814 2	0,18193185
Genetta servalina NC024568 2	Callorhinus ursinus NC008415 1	0,18193395
Prionailurus bengalensis NC028301 12	Lutra lutra NC011358 9	0,1819341
Mustela nivalis T306 5	Arctocephalus townsendi NC008420 1	0,18193491
Mustela altaica NC021751 1	Arctocephalus townsendi NC008420 1	0,18193571
Prionailurus viverrinus NC028305 1	Ailurus fulgens NC011124 1	0,18193634
Vulpes lagopus NC026529 3	Phoca groenlandica NC008429 54	0,18193793
Mustela eversmannii NC028013 1	Lynx rufus NC014456 3	0,18193883
Panthera onca KP202264 2	Mustela kathiah NC023210 1	0,18193893
Potos flavus T414 1	Galerella sanguinea T378 1	0,18193911
Ursus arctos EU497665 29	Melogale moschata KP726273 1	0,18193956
Leopardus wiedii NC028318 1	Callorhinus ursinus NC008415 1	0,18193966
Potos flavus T414 1	Lynx lynx NC027083 4	0,18194012
Martes foina NC020643 1	Urva semitorquata MH464789 1	0,18194045
Prionailurus viverrinus NC028305 1	Mustela sibirica NC020637 6	0,1819409
Martes americana NC020642 1	Felis catus NC001700 2	0,18194196
Melogale moschata KP726273 1	Catopuma badia NC028300 1	0,18194214
Paradoxurus hermaphroditus NC03959	Conepatus chinga NC042596 1	0,18194225
Viverricula indica KX891751 1	Arctotherium sp NC030174 1	0,18194278
Martes zibellina NC011579 39	Leopardus pardalis NC028315 1	0,18194282
Civettictis civetta GLC19 1	Arctotherium sp NC030174 1	0,18194397
Ursus maritimus NC003428 31	Ailurus fulgens styani NC009691 1	0,18194413
Procyon lotor AB462049 4	Felis silvestris lybica KP202275 4	0,18194616
Catopuma temminckii NC027115 41	Canis latrans NC008093 7	0,18194826
Mustela nigripes NC024942 1	Chrysocyon brachyurus NC024172 1	0,18195003
Otocyon megalotis SAF1 2	Leopardus pardalis NC028315 1	0,1819503
Eumetopias jubatus NC004030 10	Canis adustus KT448271 1	0,18195373
Viverra zibetha T609 1	Ictonyx striatus T299 1	0,18195446
Ursus thibetanus mupinensis NC00875	Puma yagouaroundi NC028311 1	0,18195643
Ictonyx striatus T299 1	Caracal caracal NC028306 1	0,18195663
Leopardus guigna NC028321 1	Ictonyx striatus T299 1	0,18195739
Ursus arctos AP012576 6	Mustela sibirica NC020637 6	0,18195805
Viverra zibetha T609 1	Chrysocyon brachyurus NC024172 1	0,1819589
Ursus thibetanus laniger MH281753 2	Neophoca cinerea NC008419 1	0,18195894
Ursus spelaeus EU327344 13	Ailurus fulgens NC011124 1	0,18195927
Martes martes T302 3	Helarctos malayanus NC009968 2	0,18195984
Paguma larvata PDDS11 2	Canis aureus KT448274 1	0,1819603
Martes zibellina NC011579 39	Leopardus colocolo NC028314 1	0,18196078
Ursus thibetanus thibetanus NC01111	Bassariscus sumichrasti SRX1099089 1	0,18196094
Prionailurus viverrinus NC028305 1	Lycalopex sechurae KT448284 1	0,18196389
Ursus spelaeus NC011112 8	Mustela nivalis T306 5	0,18196473
Panthera leo NERO 19	Mustela frenata NC020640 1	0,18197177
Pusa sibirica NC008432 2	Neofelis nebulosa NC008450 3	0,18197466
Ursus spelaeus EU327344 13	Arctictis binturong T605 2	0,18197684
Phocarctos hookeri NC008418 1	Lutra lutra NC011358 9	0,18198889
Enhydra lutris NC009692 1	Arctocephalus townsendi NC008420 1	0,18198924
Melogale moschata V0735A 1	Arctocephalus forsteri KT693377 17	0,18199356
Ursus arctos GU573491 207	Eumetopias jubatus NC004030 10	0,18199578
Otaria byronia OTAB 1	Enhydra lutris NC009692 1	0,18199587
Halichoerus grypus NC001602 2	Diplogale hosei MH464790 1	0,18199702
Viverricula indica NC025296 2	Lutra lutra NC011358 9	0,18199731
Neophoca cinerea NC008419 1	Aonyx cinerea NC035814 2	0,18199939
Phocarctos hookeri NC008418 1	Martes pennanti NC020664 16	0,18200061
Mustela sibirica NC020637 6	Canis latrans NC008093 7	0,18200082
Mustela altaica NC021751 1	Canis adustus KT448271 1	0,18200095
Neophoca cinerea NC008419 1	Melogale moschata NC020644 1	0,18200108
Meles anakuma NC009677 1	Leopardus pardalis NC028315 1	0,18200189
Meles anakuma NC009677 1	Lynx canadensis NC028313 1	0,18200226
Lutra lutra LC050126 1	Felis margarita NC028308 1	0,18200257

Mustela nigripes NC024942 1	Mustela altaica NC021751 1	0,07349915
Mustela sibirica NC020637 6	Mustela altaica NC021751 1	0,07349931
Ommatophoca rossii AY377287etc 1	Hydrurga leptonyx NC008425 1	0,07365942
Zalophus wollebaeki SRR4431565 1	Phocarctos hookeri NC008418 1	0,07376055
Canis mesomelas KT448280 1	Canis lupus familiaris NC002008 1231	0,0738586
Panthera pardus japonensis KJ866876 8	Panthera onca KP202264 2	0,0740641
Ursus thibetanus laniger MH281753 2	Helarctos malayanus NC009968 2	0,07407861
Mustela putorius NC020638 4	Mustela altaica NC021751 1	0,07410488
Eumetopias jubatus NC004030 10	Arctocephalus townsendi NC008420 1	0,07418384
Xenogale naso C07XAR110 1	Atilax paludinosus T606 1	0,07426609
Canis mesomelas KT448280 1	Canis aureus KT448274 1	0,07434006
Canis mesomelas KT448280 1	Canis lupus chanco NC010340 4	0,07447606
Zalophus californianus NC008416 1	Otaria byronia OTAB 1	0,07457604
Mustela sibirica AP017394 11	Mustela altaica NC021751 1	0,07457617
Puma yagouaroundi NC028311 1	Puma concolor NC016470 22	0,07460275
Neophoca cinerea NC008419 1	Eumetopias jubatus NC004030 10	0,07485698
Paradoxurus hermaphroditus NLNC 1	Paguma larvata PDDS11 2	0,07494445
Panthera uncia NC010638 1	Panthera onca KP202264 2	0,07500673
Ursus thibetanus formosanus NC009331 1	Helarctos malayanus NC009968 2	0,07501946
Mustela itatsi NC034330 19	Mustela altaica NC021751 1	0,07504738
Zalophus wollebaeki SRR4431565 1	Otaria byronia OTAB 1	0,07517616
Ursus americanus JX196366 3	Helarctos malayanus NC009968 2	0,07523122
Ursus thibetanus mupinensis NC008753 2	Helarctos malayanus NC009968 2	0,07541428
Ursus thibetanus thibetanus NC011118 4	Helarctos malayanus NC009968 2	0,07562821
Mustela eversmannii NC028013 1	Mustela altaica NC021751 1	0,07585477
Panthera tigris amoyensis NC014770 2	Panthera onca NC022842 1	0,07608826
Canis mesomelas KT448280 1	Canis anthus NC027956 2	0,07642148
Mustela erminea T305 2	Mustela altaica NC021751 1	0,07659559
Otaria byronia OTAB 1	Eumetopias jubatus NC004030 10	0,07660986
Phocarctos hookeri NC008418 1	Eumetopias jubatus NC004030 10	0,07674295
Mustela nivalis T306 5	Mustela nigripes NC024942 1	0,07686458
Mustela itatsi NC034330 19	Mustela erminea T305 2	0,07719747
Pusa sibirica NC008432 2	Cystophora cristata NC008427 1	0,07743065
Mustela nivalis T306 5	Mustela itatsi NC034330 19	0,07753778
Mustela sibirica AP017394 11	Mustela nivalis T306 5	0,07760496
Mustela sibirica NC020637 6	Mustela nivalis T306 5	0,07780697
Paradoxurus jerdoni MH464793 1	Paguma larvata PDDS11 2	0,07783987
Paradoxurus hermaphroditus NC039591 1	Paguma larvata PDDS11 2	0,07797455
Pusa hispida NC 008433 1	Cystophora cristata NC008427 1	0,07817734
Mustela putorius NC020638 4	Mustela nivalis T306 5	0,07821066
Ursus thibetanus thibetanus NC011118 4	Ursus spelaeus NC011112 8	0,07859395
Ursus thibetanus formosanus NC009331 1	Ursus spelaeus NC011112 8	0,07879325
Ursus thibetanus laniger MH281753 2	Ursus spelaeus NC011112 8	0,07912518
Ursus thibetanus mupinensis NC008753 2	Ursus maritimus GU573488 Svalbard	0,07918775
Ursus thibetanus formosanus NC009331 1	Ursus maritimus GU573488 Svalbard	0,07918979
Pusa caspica NC008431 1	Cystophora cristata NC008427 1	0,07931592
Ursus thibetanus laniger MH281753 2	Ursus maritimus GU573488 Svalbard	0,0793948
Ursus thibetanus thibetanus NC011118 4	Ursus maritimus GU573488 Svalbard	0,07939494
Mustela nivalis T306 5	Mustela eversmannii NC028013 1	0,07942209
Mustela nigripes NC024942 1	Mustela erminea T305 2	0,0795531
Ichneumia albicauda T603 1	Bdeogale nigripes GLC15 1	0,07965931
Ursus thibetanus laniger MH281753 2	Ursus arctos AP012576 6	0,07966777
Ursus thibetanus laniger MH281753 2	Ursus arctos GU573491 207	0,07973158
Mustela nivalis T306 5	Mustela erminea T305 2	0,07975921
Ursus thibetanus formosanus NC009331 1	Ursus arctos GU573491 207	0,07979582
Ursus thibetanus formosanus NC009331 1	Ursus spelaeus EU327344 13	0,07980373
Ursus thibetanus thibetanus NC011118 4	Ursus spelaeus EU327344 13	0,07980682
Panthera tigris amoyensis NC014770 2	Panthera leo NERO 19	0,07981323
Ursus thibetanus thibetanus NC011118 4	Ursus arctos isabellinus 18B5 2	0,07986645
Phoca fasciata NC008428 1	Cystophora cristata NC008427 1	0,07992478
Ursus thibetanus mupinensis NC008753 2	Ursus arctos isabellinus 18B5 2	0,07992862
Ursus thibetanus formosanus NC009331 1	Ursus arctos EU497665 29	0,07993047
Ursus thibetanus laniger MH281753 2	Ursus maritimus NC003428 31	0,08000083
Ursus thibetanus thibetanus NC011118 4	Ursus arctos GU573491 207	0,08000111
Phoca largha NC008430 1	Cystophora cristata NC008427 1	0,08006235
Ursus thibetanus formosanus NC009331 1	Ursus maritimus NC003428 31	0,08006522
Ursus thibetanus formosanus NC009331 1	Ursus arctos isabellinus 18B5 2	0,08006524
Ursus maritimus GU573488 Svalbard	Helarctos malayanus NC009968 2	0,08006654
Ursus thibetanus thibetanus NC011118 4	Ursus arctos EU497665 29	0,08006834
Ursus thibetanus thibetanus NC011118 4	Ursus arctos AP012576 6	0,08007207
Prionailurus rubiginosus NC028304 2	Felis silvestris lybica KP202275 4	0,08012389
Ursus thibetanus mupinensis NC008753 2	Ursus maritimus NC003428 31	0,08013041
Puma concolor NC016470 22	Acinonyx jubatus NC005212 3	0,08013164
Neovison vison NC020641 3	Mustela frenata NC020640 1	0,08016544
Ursus thibetanus mupinensis NC008753 2	Ursus arctos GU573491 207	0,08019779
Ursus thibetanus formosanus NC009331 1	Ursus arctos AP012576 6	0,08020362
Ursus thibetanus mupinensis NC008753 2	Ursus arctos EU497665 29	0,08026513
Ursus thibetanus laniger MH281753 2	Ursus arctos pruinosus MG066703 3	0,08027367
Ursus thibetanus thibetanus NC011118 4	Ursus arctos pruinosus MG066703 3	0,08027389
Panthera tigris amoyensis NC014770 2	Panthera pardus NC010641 5	0,08028013
Ursus thibetanus mupinensis NC008753 2	Ursus arctos GU573486 5	0,08033596
Ursus thibetanus laniger MH281753 2	Ursus arctos EU497665 29	0,08033747
Ursus thibetanus laniger MH281753 2	Ursus arctos isabellinus 18B5 2	0,08033747
Ursus thibetanus formosanus NC009331 1	Ursus arctos pruinosus MG066703 3	0,08033803
Phoca vitulina NC001325 1	Cystophora cristata NC008427 1	0,08039923
Ursus thibetanus thibetanus NC011118 4	Ursus maritimus NC003428 31	0,08040501
Ursus thibetanus laniger MH281753 2	Ursus spelaeus EU327344 13	0,0804722
Ursus arctos isabellinus 18B5 2	Helarctos malayanus NC009968 2	0,08053817
Halichoerus grypus NC001602 2	Cystophora cristata NC008427 1	0,08055259
Panthera tigris amoyensis NC014770 2	Panthera pardus japonensis KJ866876 8	0,08060087
Ursus thibetanus formosanus NC009331 1	Ursus arctos GU573486 5	0,08060736
Ursus thibetanus thibetanus NC011118 4	Ursus arctos GU573486 5	0,08067784
Mustela sibirica NC020637 6	Mustela erminea T305 2	0,08069727
Ursus thibetanus mupinensis NC008753 2	Ursus arctos pruinosus MG066703 3	0,08074021
Ursus thibetanus mupinensis NC008753 2	Ursus arctos AP012576 6	0,08074031
Ichneumia albicauda T603 1	Cynictis penicillata T375 1	0,0808712
Ursus thibetanus laniger MH281753 2	Ursus arctos GU573486 5	0,08094717
Mustela putorius NC020638 4	Mustela erminea T305 2	0,08096648
Mustela sibirica AP017394 11	Mustela erminea T305 2	0,08096648
Prionailurus rubiginosus NC028304 2	Felis margarita NC028308 1	0,0809992
Prionailurus rubiginosus NC028304 2	Felis catus NC001700 2	0,08120119
Ursus thibetanus mupinensis NC008753 2	Ursus spelaeus NC011112 8	0,08135008
Ursus maritimus NC003428 31	Helarctos malayanus NC009968 2	0,08148064

Salanoia concolor D378 1	Martes martes T302 3	0,18200319
Prionailurus viverrinus NC028305 1	Meles anakuma NC009677 1	0,18200387
Prionailurus viverrinus NC028305 1	Lutra lutra LC050126 1	0,18200393
Mungotictis decemlineata NC027828 1	Melogale moschata NC020644 1	0,18200481
Vulpes vulpes NC008434 5	Genetta servalina NC024568 2	0,18200545
Paradoxurus jerdoni MH464793 1	Mustela sibirica NC020637 6	0,1820064
Procyon lotor AB462046 3	Arctotherium sp NC030174 1	0,18200728
Ictonyx striatus T299 1	Fossa fossana D350 1	0,18200774
Mustela putorius NC020638 4	Leptailurus serval NC028316 1	0,18200813
Genetta genetta T297 1	Ailurus fulgens styani NC009691 1	0,18200879
Vulpes vulpes NC008434 5	Leopardus wiedii NC028318 1	0,18200879
Prionailurus planiceps NC028312 6	Martes americana NC020642 1	0,18200916
Vulpes vulpes NC008434 5	Caracal caracal NC028306 1	0,18200937
Martes zibellina NC011579 39	Felis catus NC001700 2	0,18200967
Mustela eversmannii NC028013 1	Leopardus geoffroyi NC028320 1	0,18200973
Ursus arctos pruinosus MG066703 3	Mustela sibirica NC020637 6	0,18201077
Procyon lotor AB462049 4	Arctodus simus NC011116 1	0,18201087
Civettictis civetta GLC19 1	Arctocephalus pusillus NC008417 1	0,18201172
Smilodon populator MF871700 1	Procyon lotor AB462046 3	0,18201184
Ursus arctos GU573486 5	Martes zibellina NC011579 39	0,18201274
Nyctereutes procyonoides NC013700 3	Leopardus geoffroyi NC028320 1	0,18201359
Procyon lotor AB462046 3	Leopardus pardalis NC028315 1	0,18201458
Ursus thibetanus laniger MH281753 2	Martes melampus NC009678 1	0,18201478
Ursus thibetanus formosanus NC00933	Callorhinus ursinus NC008415 1	0,18201482
Phoca vitulina NC001325 1	Paradoxurus jerdoni MH464793 1	0,1820156
Martes foina NC020643 1	Galerella sanguinea T378 1	0,18201599
Hemigalus derbyanus MH464791 1	Enhydra lutris NC009692 1	0,18201673
Zalophus wollebaeki SRR4431565 1	Procyon lotor AB462046 3	0,18201675
Martes zibellina NC011579 39	Helarctos malayanus NC009968 2	0,18201695
Martes martes T302 3	Felis nigripes NC028309 1	0,18201761
Melursus ursinus NC009970 2	Arctocephalus australis MG023139 1	0,18201885
Helarctos malayanus NC009968 2	Fossa fossana D350 1	0,1820192
Helarctos malayanus NC009968 2	Enhydra lutris NC009692 1	0,18201931
Catopuma badia NC028300 1	Arctotherium sp NC030174 1	0,18201956
Panthera onca NC022842 1	Mustela erminea T305 2	0,18202043
Ursus thibetanus laniger MH281753 2	Salanoia concolor D378 1	0,18202083
Ursus thibetanus laniger MH281753 2	Arctocephalus australis MG023139 1	0,18202169
Meles leucurus NC039173 4	Diplogale hosei MH464790 1	0,1820219
Paradoxurus hermaphroditus NC03959	Bassariscus sumichrasti SRX1099089 1	0,18202649
Zalophus californianus NC008416 1	Crossarchus platycephalus C7R66 1	0,18202718
Mirounga angustirostris SRR10331586	Crocuta crocuta NC020670 3	0,18202786
Viverricula indica KX891751 1	Chrysocyon brachyurus NC024172 1	0,18202868
Viverricula indica KX891745 1	Ursus thibetanus laniger MH281753 2	0,18202944
Ursus thibetanus laniger MH281753 2	Smilodon populator MF871700 1	0,18202986
Procyon lotor AB462046 3	Acinonyx jubatus NC005212 3	0,18202987
Panthera leo spelaea KX258452 2	Mirounga angustirostris SRR10331586	0,18203032
Meles meles T303 3	Urva javanica/auropunctata NC006835	0,18203156
Otocolobus manul NC028323 1	Chrysocyon brachyurus NC024172 1	0,18203431
Mustela itatsi NC034330 19	Canis lupus chanco NC010340 4	0,18203844
Catopuma badia NC028300 1	Canis mesomelas KT448280 1	0,18203892
Mustela altaica NC021751 1	Canis lupus familiaris NC002008 1231	0,18203957
Spilogale putorius NC010497 1	Martes melampus NC009678 1	0,18203995
Smilodon populator MF871700 1	Nyctereutes procyonoides NC013700 3	0,18204094
Ursus spelaeus NC011112 8	Acinonyx jubatus NC005212 3	0,18204923
Paradoxurus hermaphroditus NLNC 1	Halichoerus grypus NC001602 2	0,18205103
Viverra zibetha T609 1	Eumetopias jubatus NC004030 10	0,18205345
Felis margarita NC028308 1	Canis lupus chanco NC010340 4	0,18205482
Phocarctos hookeri NC008418 1	Lutra lutra LC050126 1	0,18205689
Genetta abyssinica MG489822 1	Canis latrans NC008093 7	0,18205805
Mustela sibirica NC020637 6	Arctocephalus townsendi NC008420 1	0,18205939
Mustela kathiah NC023210 1	Arctocephalus gazella BK010918 1	0,18205965
Melogale moschata V0735A 1	Fossa fossana D350 1	0,18205975
Salanoia concolor D378 1	Lutra sumatrana NC035810 1	0,18206249
Nasua nasua NC020647 1	Cystophora cristata NC008427 1	0,18206348
Conepatus chinga NC042596 1	Arctodus simus NC011116 1	0,18206374
Urocyon littoralis catalinae KP129018	Pusa caspica NC008431 1	0,18206376
Otaria byronia OTAB 1	Melogale moschata V0735A 1	0,18206546
Otaria byronia OTAB 1	Fossa fossana D350 1	0,18206607
Salanoia concolor D378 1	Arctonyx collaris NC020645 1	0,18206693
Taxidea taxus NC020646 1	Xenogale naso C07XAR110 1	0,18206912
Ursus arctos GU573486 5	Eumetopias jubatus NC004030 10	0,18206984
Enhydra lutris NC009692 1	Catopuma temminckii NC027115 41	0,18206994
Pusa sibirica NC008432 2	Paradoxurus jerdoni MH464793 1	0,18207155
Prionodon linsang ERR2391707 1	Phoca fasciata NC008428 1	0,18207236
Neovison vison NC020641 3	Urva brachyura KY117547 1	0,1820735
Martes melampus NC009678 1	Canis aureus KT448274 1	0,18207416
Prionailurus planiceps KY682741 4	Lutra sumatrana NC035810 1	0,18207425
Lutra sumatrana NC035810 1	Felis margarita NC028308 1	0,18207462
Mustela eversmannii NC028013 1	Felis margarita NC028308 1	0,18207499
Procyon lotor AB462046 3	Otaria byronia OTAB 1	0,18207647
Mustela sibirica AP017394 11	Catopuma badia NC028300 1	0,18207663
Martes zibellina NC011579 39	Felis nigripes NC028309 1	0,18207708
Martes zibellina NC011579 39	Felis chaus NC028307 1	0,18207763
Paradoxurus hermaphroditus NC03959	Canis latrans NC008093 7	0,18207787
Ursus arctos pruinosus MG066703 3	Mustela sibirica AP017394 11	0,18207809
Felis margarita NC028308 1	Canis latrans NC008093 7	0,18207884
Catopuma badia NC028300 1	Ailurus fulgens styani NC009691 1	0,18207905
Puma yagouaroundi NC028311 1	Canis anthus NC027956 2	0,18207949
Zalophus californianus NC008416 1	Genetta abyssinica MG489822 1	0,18207955
Parahyaena brunnea NC038159 15	Mustela erminea T305 2	0,1820799
Ursus arctos isabellinus 18B5 2	Arctocephalus pusillus NC008417 1	0,18208043
Crossarchus platycephalus C7R66 1	Canis lupus chanco NC010340 4	0,1820817
Mustela altaica NC021751 1	Caracal caracal NC028306 1	0,18208265
Melogale moschata NC020644 1	Leopardus tigrinus NC028317 1	0,18208283
Prionailurus rubiginosus NC028304 2	Aonyx cinerea NC035814 2	0,18208371
Ursus thibetanus formosanus NC00933	Arctocephalus forsteri KT693377 17	0,18208484
Lutra lutra LC050126 1	Acinonyx jubatus NC005212 3	0,1820851
Ursus arctos GU573486 5	Ailurus fulgens styani NC009691 1	0,18208525
Arctocephalus forsteri NC004023 28	Ailurus fulgens NC011124 1	0,18208537
Melursus ursinus NC009970 2	Arctocephalus townsendi NC008420 1	0,18208548
Catopuma temminckii NC027115 41	Bassariscus sumichrasti SRX1099089 1	0,18208864
Prionailurus planiceps KY682741 4	Lycaon pictus NC028427 2	0,18209097
Prionailurus planiceps NC028312 6	Lycalopex sechurae KT448284 1	0,18209331

Ursus arctos GU573486 5	Helarctos malayanus NC009968 2	0,08148395
Ursus spelaeus NC011112 8	Ursus americanus JX196366 3	0,08149976
Mustela eversmannii NC028013 1	Mustela erminea T305 2	0,08157222
Ursus thibetanus thibetanus NC011118 4	Melursus ursinus NC009970 2	0,08161756
Ursus spelaeus NC011112 8	Helarctos malayanus NC009968 2	0,08162262
Puma yagouaroundi NC028311 1	Acinonyx jubatus NC005212 3	0,08181482
Prionailurus bengalensis NC028301 12	Felis chaus NC028307 1	0,0818745
Prionailurus rubiginosus NC028304 2	Felis chaus NC028307 1	0,08194183
Ursus spelaeus EU327344 13	Helarctos malayanus NC009968 2	0,08195938
Meles leucurus NC039173 4	Arctonyx collaris NC020645 1	0,08197052
Ursus arctos GU573491 207	Helarctos malayanus NC009968 2	0,08201937
Ursus thibetanus mupinensis NC008753 2	Ursus spelaeus EU327344 13	0,08202407
Puma concolor NC016470 22	Lynx rufus NC014456 3	0,08207649
Prionailurus bengalensis CKM45 20	Felis silvestris lybica KP202275 4	0,08207831
Panthera tigris amoyensis NC014770 2	Panthera leo spelaea KX258452 2	0,08211901
Panthera uncia KP202269 1	Panthera tigris amoyensis NC014770 2	0,08214948
Ursus arctos EU497665 29	Helarctos malayanus NC009968 2	0,08215405
Panthera tigris NC010642 35	Panthera onca NC022842 1	0,08221572
Ursus thibetanus formosanus NC009331 1	Melursus ursinus NC009970 2	0,08236139
Puma concolor NC016470 22	Catopuma badia NC028300 1	0,08248048
Ursus maritimus GU573488 Svalbard	Melursus ursinus NC009970 2	0,0824932
Panthera tigris amoyensis NC014770 2	Panthera onca KP202264 2	0,08254983
Prionailurus bengalensis NC028301 12	Felis silvestris lybica KP202275 4	0,08281713
Ursus thibetanus mupinensis NC008753 2	Melursus ursinus NC009970 2	0,08289701
Ursus arctos AP012576 6	Helarctos malayanus NC009968 2	0,08290111
Prionailurus bengalensis CKM45 20	Felis catus NC001700 2	0,08295362
Ursus arctos GU573491 207	Melursus ursinus NC009970 2	0,08336858
Ursus thibetanus laniger MH281753 2	Melursus ursinus NC009970 2	0,08337571
Ursus arctos EU497665 29	Melursus ursinus NC009970 2	0,08350337
Prionailurus bengalensis NC028301 12	Felis nigripes NC028309 1	0,0836251
Cuon alpinus NC013445 3	Canis latrans NC008093 7	0,08370287
Ursus maritimus NC003428 31	Melursus ursinus NC009970 2	0,0837053
Prionailurus rubiginosus NC028304 2	Otocolobus manul NC028323 1	0,08382709
Phoca groenlandica NC008429 54	Cystophora cristata NC008427 1	0,08383015
Ursus maritimus GU573488 Svalbard	Ursus americanus JX196366 3	0,08385008
Ursus spelaeus EU327344 13	Ursus americanus JX196366 3	0,08392493
Ursus arctos GU573486 5	Melursus ursinus NC009970 2	0,08397768
Prionailurus bengalensis NC028301 12	Leptailurus serval NC028316 1	0,08402909
Ursus arctos pruinosus MG066703 3	Helarctos malayanus NC009968 2	0,08404301
Ursus maritimus NC003428 31	Ursus americanus JX196366 3	0,08405212
Prionailurus bengalensis CKM45 20	Felis margarita NC028308 1	0,08409835
Panthera tigris NC010642 35	Panthera leo NERO 19	0,08419137
Meles meles T303 3	Arctonyx collaris NC020645 1	0,0841914
Prionailurus bengalensis CKM45 20	Felis chaus NC028307 1	0,08450242
Ursus arctos EU497665 29	Ursus americanus JX196366 3	0,08472568
Lynx canadensis NC028313 1	Catopuma badia NC028300 1	0,08476973
Prionailurus bengalensis NC028301 12	Felis catus NC001700 2	0,08476973
Puma yagouaroundi NC028311 1	Catopuma badia NC028300 1	0,08476973
Ursus arctos AP012576 6	Melursus ursinus NC009970 2	0,08478619
Mirounga leonina NC008422 1	Leptonychotes weddellii NC008424 1	0,08483706
Cuon alpinus NC013445 3	Canis lupus familiaris NC002008 1231	0,0848634
Ursus arctos GU573486 5	Ursus americanus JX196366 3	0,08486746
Canis latrans NC008093 7	Canis adustus KT448271 1	0,08491793
Helogale parvula SRR7637809 1	Crossarchus platycephalus C7R66 1	0,08497172
Prionailurus bengalensis NC028301 12	Felis margarita NC028308 1	0,08497172
Vulpes vulpes NC008434 5	Vulpes lagopus NC026529 3	0,08510065
Ursus arctos pruinosus MG066703 3	Melursus ursinus NC009970 2	0,08519012
Meles anakuma NC009677 1	Arctonyx collaris NC020645 1	0,08520089
Profelis aurata NC028299 1	Leptailurus serval NC028316 1	0,08530837
Ursus spelaeus EU327344 13	Melursus ursinus NC009970 2	0,08539883
Ursus arctos GU573491 207	Ursus americanus JX196366 3	0,08539898
Lynx pardinus NC028319 161	Catopuma temminckii NC027115 41	0,08544303
Panthera uncia NC010638 1	Panthera tigris amoyensis NC014770 2	0,08544889
Ursus arctos isabellinus 18B5 2	Ursus americanus JX196366 3	0,08546618
Prionailurus bengalensis NC028301 12	Otocolobus manul NC028323 1	0,08551037
Ursus spelaeus NC011112 8	Melursus ursinus NC009970 2	0,08553299
Lynx rufus NC014456 3	Catopuma badia NC028300 1	0,0855777
Ursus arctos AP012576 6	Ursus americanus JX196366 3	0,08560498
Leptailurus serval NC028316 1	Caracal caracal NC028306 1	0,08564503
Prionailurus planiceps NC028312 6	Felis silvestris lybica KP202275 4	0,08564503
Prionailurus bengalensis CKM45 20	Leptailurus serval NC028316 1	0,08564715
Cuon alpinus NC013445 3	Canis aureus KT448274 1	0,08571012
Lynx lynx NC027083 4	Catopuma badia NC028300 1	0,08571236
Prionailurus rubiginosus NC028304 2	Felis nigripes NC028309 1	0,08571236
Mirounga leonina NC008422 1	Hydrurga leptonyx NC008425 1	0,08571655
Canis aureus KT448274 1	Canis adustus KT448271 1	0,08572612
Cuon alpinus NC013445 3	Canis lupus chanco NC010340 4	0,08575172
Lynx lynx NC027083 4	Catopuma temminckii NC027115 41	0,08577969
Otocolobus manul NC028323 1	Felis silvestris lybica KP202275 4	0,08577969
Panthera tigris NC010642 35	Panthera pardus NC010641 5	0,08580273
Salanoia concolor D378 1	Galidia elegans D146 1	0,08584125
Prionailurus bengalensis CKM45 20	Felis nigripes NC028309 1	0,08584891
Prionailurus planiceps KY682741 4	Felis silvestris lybica KP202275 4	0,08591436
Prionailurus bengalensis CKM45 20	Lynx canadensis NC028313 1	0,08598362
Ursus arctos isabellinus 18B5 2	Melursus ursinus NC009970 2	0,08599506
Prionailurus bengalensis NC028301 12	Lynx canadensis NC028313 1	0,08604902
Puma concolor NC016470 22	Lynx lynx NC027083 4	0,08625101
Puma concolor NC016470 22	Lynx pardinus NC028319 161	0,08625101
Puma yagouaroundi NC028311 1	Lynx canadensis NC028313 1	0,08625101
Ursus arctos pruinosus MG066703 3	Ursus americanus JX196366 3	0,08627883
Lynx rufus NC014456 3	Catopuma temminckii NC027115 41	0,08631834
Prionailurus rubiginosus NC028304 2	Leptailurus serval NC028316 1	0,08631834
Lynx pardinus NC028319 161	Catopuma badia NC028300 1	0,08638567
Panthera tigris NC010642 35	Panthera leo spelaea KX258452 2	0,08642738
Puma concolor NC016470 22	Lynx canadensis NC028313 1	0,08652034
Puma yagouaroundi NC028311 1	Lynx rufus NC014456 3	0,08672233
Prionailurus planiceps NC028312 6	Felis margarita NC028308 1	0,08678966
Panthera tigris NC010642 35	Panthera pardus japonensis KJ866876 8	0,08679568
Melursus ursinus NC009970 2	Helarctos malayanus NC009968 2	0,08680644
Puma concolor NC016470 22	Prionailurus rubiginosus NC028304 2	0,08685699
Galidictis fasciata DM333 1	Galidia elegans D146 1	0,08691847
Prionailurus planiceps KY682741 4	Felis chaus NC028307 1	0,08692432
Catopuma badia NC028300 1	Acinonyx jubatus NC005212 3	0,08693273

Gulo gulo NC009685 3	Arctocephalus townsendi NC008420 1	0,18209347
Ursus spelaeus EU327344 13	Martes martes T302 3	0,1820938
Ursus spelaeus NC011112 8	Martes martes T302 3	0,18209404
Paguma larvata PDDS11 2	Chrysocyon brachyurus NC024172 1	0,18209536
Ursus arctos EU497665 29	Neovison vison NC020641 3	0,1820969
Ursus thibetanus formosanus NC00933	Leopardus wiedii NC028318 1	0,18209861
Ursus thibetanus formosanus NC00933	Puma yagouaroundi NC028311 1	0,18209995
Monachus monachus NC044972 5	Xenogale naso C07XAR110 1	0,18210042
Paradoxurus hermaphroditus NLNC 1	Mephitis mephitis NC020648 1	0,18210228
Ursus spelaeus NC011112 8	Otaria byronia OTAB 1	0,18210228
Meles anakuma NC009677 1	Civettictis civetta NC033378 1	0,18210854
Viverra zibetha T609 1	Martes flavigula NC012141 3	0,1821087
Leopardus pardalis T262 1	Canis lupus familiaris NC002008 1231	0,18210883
Felis nigripes NC028309 1	Canis lupus familiaris NC002008 1231	0,1821108
Felis catus NC001700 2	Canis lupus familiaris NC002008 1231	0,18211085
Lynx rufus NC014456 3	Gulo gulo NC009685 3	0,18211603
Prionailurus bengalensis CKM45 20	Canis lupus familiaris NC002008 1231	0,18211921
Martes flavigula NC012141 3	Felis silvestris lybica KP202275 4	0,18211944
Panthera leo spelaea KX258452 2	Monachus monachus NC044972 5	0,18212049
Meles leucurus NC039173 4	Arctocephalus gazella BK010918 1	0,18212424
Taxidea taxus NC020646 1	Diplogale hosei MH464790 1	0,18212996
Salanoia concolor D378 1	Martes americana NC020642 1	0,18213043
Urocyon cinereoargenteus NC026723 2	Pusa caspica NC008431 1	0,1821311
Otaria byronia OTAB 1	Martes americana NC020642 1	0,1821312
Urocyon cinereoargenteus NC026723 2	Cystophora cristata NC008427 1	0,18213147
Urocyon littoralis catalinae KP129018	Cystophora cristata NC008427 1	0,18213147
Tremarctos ornatus NC009969 2	Arctonyx collaris NC020645 1	0,18213323
Melogale moschata NC020644 1	Arctocephalus forsteri KT693377 17	0,18213478
Enhydra lutris NC009692 1	Caracal caracal NC028306 1	0,1821366
Mustela kathiah NC023210 1	Canis anthus NC027956 2	0,18213665
Felis nigripes NC028309 1	Enhydra lutris NC009692 1	0,18213749
Mustela frenata NC020640 1	Galidictis fasciata DM333 1	0,18213809
Panthera uncia KP202269 1	Mirounga leonina NC008422 1	0,18213867
Meles leucurus NC039173 4	Felis chaus NC028307 1	0,18213883
Lutra lutra NC011358 9	Hemigalus derbyanus MH464791 1	0,18213899
Caracal caracal NC028306 1	Arctonyx collaris NC020645 1	0,18213917
Prionailurus rubiginosus NC028304 2	Callorhinus ursinus NC008415 1	0,18213959
Panthera pardus japonensis KJ866876	Mustela frenata NC020640 1	0,18214074
Viverra tangalunga MH464792 1	Paradoxurus jerdoni MH464793 1	0,18214135
Prionailurus planiceps NC028312 6	Lutra sumatrana NC035810 1	0,18214139
Mustela putorius NC020638 4	Civettictis civetta GLC19 1	0,18214159
Prionailurus planiceps NC028312 6	Mustela itatsi NC034330 19	0,1821418
Mustela putorius NC020638 4	Arctotherium sp NC030174 1	0,18214232
Prionailurus bengalensis CKM45 20	Arctonyx collaris NC020645 1	0,18214235
Mustela itatsi NC034330 19	Felis margarita NC028308 1	0,18214242
Viverra tangalunga MH464792 1	Felis chaus NC028307 1	0,18214284
Martes americana NC020642 1	Felis margarita NC028308 1	0,18214418
Mustela altaica NC021751 1	Genetta abyssinica MG489822 1	0,18214498
Ursus arctos GU573486 5	Callorhinus ursinus NC008415 1	0,18214515
Vulpes lagopus NC026529 3	Mirounga angustirostris SRR10331586	0,18214618
Prionodon linsang ERR2391707 1	Canis aureus KT448274 1	0,18214631
Ursus arctos GU573491 207	Ailurus fulgens styani NC009691 1	0,18214637
Ursus arctos GU573486 5	Melogale moschata KP726273 1	0,18214755
Puma yagouaroundi NC028311 1	Procyon lotor AB462046 3	0,18215056
Martes pennanti NC020664 16	Leopardus wiedii NC028318 1	0,18215083
Martes martes T302 3	Catopuma badia NC028300 1	0,18215102
Ursus thibetanus formosanus NC00933	Enhydra lutris NC009692 1	0,18215208
Prionailurus viverrinus NC028305 1	Mustela nivalis T306 5	0,18215272
Procyon lotor AB462046 3	Catopuma badia NC028300 1	0,18215308
Ursus thibetanus thibetanus NC01111	Arctocephalus australis MG023139 1	0,18215378
Martes foina NC020643 1	Lynx rufus NC014456 3	0,18215595
Lutra sumatrana NC035810 1	Helarctos malayanus NC009968 2	0,18215683
Canis latrans NC008093 7	Arctictis binturong T605 2	0,18215771
Ictonyx striatus T299 1	Felis chaus NC028307 1	0,18215929
Ursus spelaeus EU327344 13	Ailurus fulgens styani NC009691 1	0,18216135
Otaria byronia OTAB 1	Gulo gulo NC009685 3	0,1821617
Ursus thibetanus laniger MH281753 2	Civettictis civetta GLC19 1	0,18216358
Ursus arctos GU573491 207	Neovison vison NC020641 3	0,18216366
Ursus maritimus NC003428 31	Neovison vison NC020641 3	0,18216385
Prionailurus bengalensis CKM45 20	Lycaon pictus NC028427 2	0,18216395
Ursus thibetanus laniger MH281753 2	Otaria byronia OTAB 1	0,18216701
Phoca vitulina NC001325 1	Diplogale hosei MH464790 1	0,18217054
Meles meles T303 3	Canis lupus chanco NC010340 4	0,1821731
Zalophus californianus NC008416 1	Leopardus tigrinus NC028317 1	0,18217544
Pusa caspica NC008431 1	Neofelis nebulosa NC008450 3	0,18217653
Otaria byronia OTAB 1	Homotherium latidens MF871702 3	0,1821782
Ursus arctos AP012576 6	Neovison vison NC020641 3	0,18217838
Civettictis civetta NC033378 1	Ailurus fulgens styani NC009691 1	0,18218194
Prionailurus planiceps NC028312 6	Gulo gulo NC009685 3	0,18218286
Lynx canadensis NC028313 1	Gulo gulo NC009685 3	0,18218324
Nasua nasua NC020647 1	Halichoerus grypus NC001602 2	0,18218367
Ursus americanus JX196366 3	Ailurus fulgens styani NC009691 1	0,18218578
Martes flavigula NC012141 3	Leopardus jacobita NC028322 1	0,18218587
Martes melampus NC009678 1	Genetta abyssinica MG489822 1	0,18218713
Zalophus californianus NC008416 1	Smilodon populator MF871700 1	0,18218988
Mustela eversmannii NC028013 1	Fossa fossana D350 1	0,18219489
Mustela erminea T305 2	Mungos mungo/gambianus SRR77048	0,18219762
Mustela frenata NC020640 1	Xenogale naso C07XAR110 1	0,18219814
Pusa sibirica NC008432 2	Nasua nasua NC020647 1	0,18219846
Taxidea taxus NC020646 1	Urva brachyura KY117547 1	0,18219985
Paradoxurus hermaphroditus NLNC 1	Canis mesomelas KT448280 1	0,18220127
Melogale moschata NC020644 1	Arctocephalus townsendi NC008420 1	0,18220147
Nyctereutes procyonoides NC013700 3	Mustela frenata NC020640 1	0,18220247
Genetta abyssinica MG489822 1	Callorhinus ursinus NC008415 1	0,18220283
Prionailurus planiceps KY682741 4	Lutra lutra NC011358 9	0,18220429
Felis catus NC001700 2	Ailurus fulgens styani NC009691 1	0,18220596
Melogale moschata V0735A 1	Civettictis civetta GLC19 1	0,18220623
Prionailurus viverrinus NC028305 1	Arctonyx collaris NC020645 1	0,18220627
Mustela kathiah NC023210 1	Lynx rufus NC014456 3	0,18220703
Felis chaus NC028307 1	Ailurus fulgens styani NC009691 1	0,18220751
Pardofelis marmorata NLN3 2	Martes melampus NC009678 1	0,18220808
Paradoxurus hermaphroditus NLNC 1	Mustela eversmannii NC028013 1	0,18220809
Pardofelis marmorata NLN3 2	Mustela putorius NC020638 4	0,18220884

Puma concolor NC016470 22	Prionailurus bengalensis NC028301 12	0,08705898
Puma yagouaroundi NC028311 1	Lynx pardinus NC028319 161	0,08712631
Prionailurus viverrinus NC028305 1	Felis silvestris lybica KP202275 4	0,08712815
Lynx canadensis NC028313 1	Catopuma temminckii NC027115 41	0,08719365
Prionailurus planiceps KY682741 4	Felis margarita NC028308 1	0,08719365
Puma yagouaroundi NC028311 1	Lynx lynx NC027083 4	0,08719365
Prionailurus planiceps NC028312 6	Felis chaus NC028307 1	0,08726098
Ursus americanus JX196366 3	Melursus ursinus NC009970 2	0,0872846
Otocolobus manul NC028323 1	Felis chaus NC028307 1	0,08732831
Cuon alpinus NC013445 3	Canis anthus NC027956 2	0,08733851
Otocolobus manul NC028323 1	Felis catus NC001700 2	0,08739564
Prionailurus bengalensis CKM45 20	Lynx rufus NC014456 3	0,08739761
Leptailurus serval NC028316 1	Catopuma badia NC028300 1	0,08746297
Prionailurus planiceps NC028312 6	Felis catus NC001700 2	0,08746297
Puma concolor NC016470 22	Prionailurus bengalensis CKM45 20	0,08746534
Tremarctos ornatus NC009969 2	Arctodus simus NC011116 1	0,08749961
Otocolobus manul NC028323 1	Felis margarita NC028308 1	0,0875303
Prionailurus rubiginosus NC028304 2	Catopuma badia NC028300 1	0,0875303
Prionailurus bengalensis CKM45 20	Otocolobus manul NC028323 1	0,08753251
Canis lupus familiaris NC002008 1231	Canis adustus KT448271 1	0,08755629
Prionailurus bengalensis NC028301 12	Lynx pardinus NC028319 161	0,08766496
Mirounga leonina NC008422 1	Lobodon carcinophaga NC008423 1	0,08766929
Ichneumia albicauda T603 1	Atilax paludinosus T606 1	0,08767136
Prionailurus viverrinus NC028305 1	Felis chaus NC028307 1	0,08773321
Prionailurus bengalensis CKM45 20	Catopuma badia NC028300 1	0,08773467
Panthera uncia KP202269 1	Panthera tigris NC010642 35	0,08773831
Canis lupus chanco NC010340 4	Canis adustus KT448271 1	0,08775297
Prionailurus planiceps NC028312 6	Leptailurus serval NC028316 1	0,08779962
Prionailurus rubiginosus NC028304 2	Lynx rufus NC014456 3	0,08779962
Panthera tigris NC010642 35	Panthera onca KP202264 2	0,08780185
Prionailurus planiceps KY682741 4	Felis catus NC001700 2	0,08786695
Mirounga angustirostris SRR10331586 1	Leptonychotes weddellii NC008424 1	0,08787435
Prionailurus planiceps KY682741 4	Leptailurus serval NC028316 1	0,08793429
Puma yagouaroundi NC028311 1	Catopuma temminckii NC027115 41	0,08806895
Puma yagouaroundi NC028311 1	Prionailurus bengalensis NC028301 12	0,08806895
Puma concolor NC016470 22	Catopuma temminckii NC027115 41	0,08813628
Prionailurus bengalensis CKM45 20	Lynx pardinus NC028319 161	0,08813835
Prionailurus rubiginosus NC028304 2	Lynx canadensis NC028313 1	0,08820361
Urva semitorquata MH464789 1	Urva javanica/auropunctata NC006835 1	0,08823071
Prionailurus bengalensis NC028301 12	Catopuma badia NC028300 1	0,08827094
Puma concolor NC016470 22	Pardofelis marmorata NLN3 2	0,08827094
Ichneumia albicauda T603 1	Xenogale naso C07XAR110 1	0,08827873
Prionailurus rubiginosus NC028304 2	Lynx pardinus NC028319 161	0,08833827
Puma yagouaroundi NC028311 1	Prionailurus rubiginosus NC028304 2	0,08833827
Prionailurus bengalensis CKM45 20	Lynx lynx NC027083 4	0,08840768
Mirounga angustirostris SRR10331586 1	Hydrurga leptonyx NC008425 1	0,08841719
Prionailurus planiceps NC028312 6	Otocolobus manul NC028323 1	0,08847293
Prionailurus viverrinus NC028305 1	Leptailurus serval NC028316 1	0,08847418
Otocolobus manul NC028323 1	Felis nigripes NC028309 1	0,08854026
Prionailurus bengalensis NC028301 12	Lynx lynx NC027083 4	0,08854026
Canis anthus NC027956 2	Canis adustus KT448271 1	0,08859467
Prionailurus viverrinus NC028305 1	Felis catus NC001700 2	0,08860936
Ichneumia albicauda T603 1	Galerella sanguinea T378 1	0,08861506
Prionailurus planiceps KY682741 4	Otocolobus manul NC028323 1	0,08867493
Vulpes lagopus NC026529 3	Vulpes ferrilata NC027935 1	0,08880361
Lynx rufus NC014456 3	Felis silvestris lybica KP202275 4	0,08880959
Canis mesomelas KT448280 1	Canis adustus KT448271 1	0,08884515
Urva semitorquata MH464789 1	Atilax paludinosus T606 1	0,08887692
Urva semitorquata MH464789 1	Urva brachyura KY117547 1	0,08887692
Mirounga angustirostris SRR10331586 1	Lobodon carcinophaga NC008423 1	0,08888899
Prionailurus bengalensis NC028301 12	Lynx rufus NC014456 3	0,08894425
Urva semitorquata MH464789 1	Xenogale naso C07XAR110 1	0,08894425
Prionailurus viverrinus NC028305 1	Felis margarita NC028308 1	0,08894588
Puma yagouaroundi NC028311 1	Prionailurus bengalensis CKM45 20	0,08894661
Lynx lynx NC027083 4	Leopardus pardalis T262 1	0,08901356
Lynx pardinus NC028319 161	Acinonyx jubatus NC005212 3	0,08908793
Prionailurus rubiginosus NC028304 2	Lynx lynx NC027083 4	0,08914624
Lynx lynx NC027083 4	Leopardus pardalis NC028315 1	0,0891481
Lynx rufus NC014456 3	Acinonyx jubatus NC005212 3	0,08915506
Profelis aurata NC028299 1	Prionailurus bengalensis NC028301 12	0,08934823
Leptailurus serval NC028316 1	Felis silvestris lybica KP202275 4	0,08941557
Prionailurus rubiginosus NC028304 2	Catopuma temminckii NC027115 41	0,08941557
Lynx canadensis NC028313 1	Acinonyx jubatus NC005212 3	0,0894248
Prionailurus viverrinus NC028305 1	Lynx canadensis NC028313 1	0,08955292
Vulpes lagopus NC026529 3	Vulpes corsac NC023958 1	0,08961152
Prionailurus bengalensis NC028301 12	Leopardus pardalis T262 1	0,08961865
Prionailurus viverrinus NC028305 1	Otocolobus manul NC028323 1	0,08961871
Lynx lynx NC027083 4	Acinonyx jubatus NC005212 3	0,08962687
Lynx rufus NC014456 3	Felis margarita NC028308 1	0,08968489
Prionailurus planiceps NC028312 6	Felis nigripes NC028309 1	0,08968489
Cynictis penicillata T375 1	Atilax paludinosus T606 1	0,08975222
Prionailurus bengalensis NC028301 12	Catopuma temminckii NC027115 41	0,08975222
Prionailurus planiceps KY682741 4	Felis nigripes NC028309 1	0,08975222
Puma concolor NC016470 22	Felis chaus NC028307 1	0,08981955
Prionailurus bengalensis CKM45 20	Catopuma temminckii NC027115 41	0,08982167
Mungotictis decemlineata NC027828 1	Galidia elegans D146 1	0,08994815
Otocolobus manul NC028323 1	Catopuma badia NC028300 1	0,08995421
Profelis aurata NC028299 1	Prionailurus rubiginosus NC028304 2	0,08995421
Lynx rufus NC014456 3	Felis catus NC001700 2	0,09002154
Urva semitorquata MH464789 1	Urva javanica T413 1	0,09002154
Xenogale naso C07XAR110 1	Bdeogale nigripes GLC15 1	0,09002154
Xenogale naso C07XAR110 1	Cynictis penicillata T375 1	0,09002154
Leptailurus serval NC028316 1	Felis margarita NC028308 1	0,09008887
Tremarctos ornatus NC009969 2	Arctotherium sp NC030174 1	0,09012173
Prionailurus planiceps KY682741 4	Catopuma badia NC028300 1	0,0901562
Arctotherium sp NC030174 1	Arctodus simus NC011116 1	0,09022675
Prionailurus bengalensis NC028301 12	Caracal caracal NC028306 1	0,09029087
Catopuma temminckii NC027115 41	Acinonyx jubatus NC005212 3	0,09036648
Lynx canadensis NC028313 1	Leptailurus serval NC028316 1	0,09042553
Pardofelis marmorata NLN3 2	Lynx lynx NC027083 4	0,09042553
Puma concolor NC016470 22	Leptailurus serval NC028316 1	0,09042553
Prionailurus rubiginosus NC028304 2	Leopardus pardalis T262 1	0,09042646
Lynx canadensis NC028313 1	Leopardus pardalis T262 1	0,09042745

Viverricula indica KX891751 1	Mustela itatsi NC034330 19	0,18220885
Vulpes vulpes NC008434 5	Homotherium latidens MF871702 3	0,18220969
Paradoxurus hermaphroditus NLNC 1	Mustela altaica NC021751 1	0,18220988
Prionailurus viverrinus NC028305 1	Mustela sibirica AP017394 11	0,18221012
Viverra zibetha T609 1	Canis anthus NC027956 2	0,18221012
Puma concolor NC016470 22	Melogale moschata V0735A 1	0,18221036
Martes americana NC020642 1	Catopuma temminckii NC027115 41	0,1822104
Vulpes vulpes NC008434 5	Felis chaus NC028307 1	0,18221056
Prionailurus viverrinus NC028305 1	Lutra sumatrana NC035810 1	0,18221074
Vulpes vulpes NC008434 5	Puma concolor NC016470 22	0,18221092
Prionailurus rubiginosus NC028304 2	Melogale moschata V0735A 1	0,18221121
Vulpes zerda KJ603240 1	Leopardus wiedii NC028318 1	0,1822121
Potos flavus T414 1	Leopardus jacobita NC028322 1	0,18221237
Prionodon pardicolor NC024569 2	Mustela erminea T305 2	0,18221256
Paradoxurus hermaphroditus NC03959	Ailurus fulgens styani NC009691 1	0,18221262
Ursus arctos GU573491 207	Martes americana NC020642 1	0,1822136
Genetta abyssinica MG489822 1	Canis aureus KT448274 1	0,1822138
Viverra zibetha T609 1	Martes martes T302 3	0,18221439
Ursus thibetanus laniger MH281753 2	Callorhinus ursinus NC008415 1	0,18221697
Otocolobus manul NC028323 1	Mustela altaica NC021751 1	0,18221705
Paguma larvata PDDS11 2	Mustela nivalis T306 5	0,1822175
Poecilogale albinucha T602 1	Otaria byronia OTAB 1	0,18221907
Zalophus wollebaeki SRR4431565 1	Ursus maritimus GU573488 Svalbard	0,1822194
Ichneumia albicauda T603 1	Arctodus simus NC011116 1	0,18221942
Mustela altaica NC021751 1	Catopuma badia NC028300 1	0,18221962
Melogale moschata KP726273 1	Helarctos malayanus NC009968 2	0,18222022
Mustela altaica NC021751 1	Leopardus geoffroyi NC028320 1	0,18222085
Puma concolor NC016470 22	Arctodus simus NC011116 1	0,18222088
Ursus spelaeus NC011112 8	Martes pennanti NC020664 16	0,18222088
Leopardus jacobita NC028322 1	Arctodus simus NC011116 1	0,18222128
Lynx canadensis NC028313 1	Lycaon pictus NC028427 2	0,18222181
Melursus ursinus NC009970 2	Enhydra lutris NC009692 1	0,18222189
Melursus ursinus NC009970 2	Arctocephalus gazella BK010918 1	0,18222272
Cynictis penicillata T375 1	Canis lupus chanco NC010340 4	0,18222405
Taxidea taxus NC020646 1	Panthera onca NC022842 1	0,18222605
Puma yagouaroundi NC028311 1	Lycalopex sechurae KT448284 1	0,18222724
Ursus thibetanus laniger MH281753 2	Martes martes T302 3	0,18222886
Arctocephalus pusillus NC008417 1	Acinonyx jubatus NC005212 3	0,18223338
Arctodus simus NC011116 1	Arctictis binturong T605 2	0,18223353
Panthera tigris amoyensis NC014770 2	Canis adustus KT448271 1	0,18223517
Ursus thibetanus thibetanus NC011118	Arctocephalus forsteri NC004023 28	0,18223558
Viverricula indica KX891751 1	Mephitis mephitis NC020648 1	0,18223575
Ursus thibetanus thibetanus NC011118	Puma yagouaroundi NC028311 1	0,18223655
Leptailurus serval NC028316 1	Canis mesomelas KT448280 1	0,18223684
Paradoxurus jerdoni MH464793 1	Mephitis mephitis NC020648 1	0,18223709
Pusa caspica NC008431 1	Cuon alpinus NC013445 3	0,18223852
Helarctos malayanus NC009968 2	Catopuma badia NC028300 1	0,18223967
Mustela erminea T305 2	Urva javanica/auropunctata NC006835	0,18224104
Panthera leo NERO 19	Mustela kathiah NC023210 1	0,18224199
Zalophus wollebaeki SRR4431565 1	Acinonyx jubatus NC005212 3	0,1822442
Lynx lynx NC027083 4	Canis lupus familiaris NC002008 1231	0,18224439
Lynx pardinus NC028319 161	Canis lupus familiaris NC002008 1231	0,18224479
Martes zibellina NC011579 39	Genetta abyssinica MG489822 1	0,18224601
Ursus americanus JX196366 3	Arctocephalus forsteri NC004023 28	0,18224798
Viverricula indica KX891751 1	Ursus americanus JX196366 3	0,18225121
Puma concolor NC016470 22	Martes flavigula NC012141 3	0,18225195
Ursus americanus JX196366 3	Otaria byronia OTAB 1	0,18225199
Ursus americanus JX196366 3	Ailurus fulgens NC011124 1	0,18225312
Leopardus tigrinus NC028317 1	Eumetopias jubatus NC004030 10	0,18225679
Nasua nasua NC020647 1	Mustela putorius NC020638 4	0,18225868
Lycaon pictus NC028427 2	Civettictis civetta NC033378 1	0,18225904
Leopardus colocolo NC028314 1	Canis lupus chanco NC010340 4	0,18225996
Ommatophoca rossii AY377287etc 1	Cryptoprocta ferox CFC13 1	0,18226214
Lontra canadensis SRR10409165 1	Ailurus fulgens NC011124 1	0,18226305
Neophoca cinerea NC008419 1	Martes americana NC020642 1	0,18226376
Martes zibellina NC011579 39	Galidictis fasciata DM333 1	0,18226551
Tremarctos ornatus NC009969 2	Arctocephalus gazella BK010918 1	0,1822674
Profelis aurata NC028299 1	Lutra lutra LC050126 1	0,18227082
Ursus arctos isabellinus 18B5 2	Taxidea taxus NC020646 1	0,18227169
Mephitis mephitis NC020648 1	Enhydra lutris NC009692 1	0,18227358
Ursus thibetanus mupinensis NC00875	Callorhinus ursinus NC008415 1	0,18227394
Panthera uncia KP202269 1	Mustela frenata NC020640 1	0,18227534
Panthera pardus japonensis KJ866876	Mustela kathiah NC023210 1	0,18227619
Mustela itatsi NC034330 19	Canis aureus KT448274 1	0,18227677
Potos flavus T414 1	Lynx pardinus NC028319 161	0,1822771
Mustela nigripes NC024942 1	Arctotherium sp NC030174 1	0,18227734
Vulpes corsac NC023958 1	Prionailurus rubiginosus NC028304 2	0,18227845
Martes americana NC020642 1	Felis nigripes NC028309 1	0,18227872
Mustela itatsi NC034330 19	Leopardus guigna NC028321 1	0,18227947
Vulpes zerda KJ603240 1	Pusa hispida NC 008433 1	0,18228057
Viverra zibetha T609 1	Procyon lotor AB462046 3	0,18228133
Ursus arctos isabellinus 18B5 2	Mustela putorius NC020638 4	0,18228294
Ursus arctos GU573491 207	Mustela nigripes NC024942 1	0,18228319
Prionailurus planiceps NC028312 6	Aonyx cinerea NC035814 2	0,18228485
Procyon lotor AB462046 3	Leopardus pardalis T262 1	0,18228486
Leopardus wiedii NC028318 1	Aonyx cinerea NC035814 2	0,18228634
Martes martes T302 3	Canis aureus KT448274 1	0,18228666
Puma yagouaroundi NC028311 1	Mustela nivalis T306 5	0,1822869
Ursus arctos GU573486 5	Martes americana NC020642 1	0,18228698
Ursus thibetanus thibetanus NC011118	Arctocephalus townsendi NC008420 1	0,18228756
Otocolobus manul NC028323 1	Martes martes T302 3	0,18228763
Ursus arctos GU573486 5	Mustela putorius NC020638 4	0,18228903
Pusa caspica NC008431 1	Arctictis binturong T605 2	0,18228963
Ursus thibetanus laniger MH281753 2	Arctocephalus townsendi NC008420 1	0,1822903
Zalophus californianus NC008416 1	Melogale moschata KP726273 1	0,18229178
Zalophus wollebaeki SRR4431565 1	Viverra zibetha T609 1	0,1822923
Mustela itatsi NC034330 19	Acinonyx jubatus NC005212 3	0,18229257
Prionailurus planiceps NC028312 6	Lycaon pictus NC028427 2	0,18229294
Martes melampus NC009678 1	Leopardus colocolo NC028314 1	0,18229322
Prionailurus bengalensis CKM45 20	Bassariscus sumichrasti SRX1099089 1	0,18229398
Ursus spelaeus EU327344 13	Meles anakuma NC009677 1	0,18229465
Mustela kathiah NC023210 1	Helarctos malayanus NC009968 2	0,18229596
Potos flavus T414 1	Arctocephalus forsteri NC004023 28	0,18229817

Pardofelis marmorata NLN3 2	Catopuma badia NC028300 1	0,09049286
Prionailurus bengalensis NC028301 12	Leopardus pardalis NC028315 1	0,09056117
Prionailurus rubiginosus NC028304 2	Leopardus wiedii NC028318 1	0,09056121
Prionailurus bengalensis NC028301 12	Leopardus wiedii NC028318 1	0,09056132
Prionailurus bengalensis NC028301 12	Leopardus colocolo NC028314 1	0,09057286
Cuon alpinus NC013445 3	Canis mesomelas KT448280 1	0,09059604
Prionailurus rubiginosus NC028304 2	Leopardus colocolo NC028314 1	0,09063948
Bdeogale nigripes GLC15 1	Atilax paludinosus T606 1	0,09069486
Lynx pardinus NC028319 161	Felis silvestris lybica KP202275 4	0,09076219
Prionailurus planiceps KY682741 4	Lynx canadensis NC028313 1	0,09076219
Leptailurus serval NC028316 1	Felis catus NC001700 2	0,09082952
Leptailurus serval NC028316 1	Felis chaus NC028307 1	0,09082952
Prionailurus planiceps NC028312 6	Lynx canadensis NC028313 1	0,09082952
Prionailurus rubiginosus NC028304 2	Caracal caracal NC028306 1	0,09082952
Prionailurus viverrinus NC028305 1	Felis nigripes NC028309 1	0,09083121
Prionailurus viverrinus NC028305 1	Lynx pardinus NC028319 161	0,09089933
Panthera uncia NC010638 1	Panthera tigris NC010642 35	0,09090304
Prionailurus planiceps NC028312 6	Catopuma badia NC028300 1	0,09096418
Puma yagouaroundi NC028311 1	Otocolobus manul NC028323 1	0,09096418
Lynx lynx NC027083 4	Leopardus wiedii NC028318 1	0,09103357
Profelis aurata NC028299 1	Prionailurus bengalensis CKM45 20	0,09103382
Otocolobus manul NC028323 1	Leptailurus serval NC028316 1	0,09109885
Puma yagouaroundi NC028311 1	Felis silvestris lybica KP202275 4	0,09109885
Prionailurus rubiginosus NC028304 2	Acinonyx jubatus NC005212 3	0,09110758
Prionailurus bengalensis NC028301 12	Acinonyx jubatus NC005212 3	0,09110779
Lynx pardinus NC028319 161	Leptailurus serval NC028316 1	0,09116618
Prionailurus planiceps NC028312 6	Lynx rufus NC014456 3	0,09116618
Leopardus pardalis NC028315 1	Felis silvestris lybica KP202275 4	0,09116718
Lynx canadensis NC028313 1	Felis silvestris lybica KP202275 4	0,09123351
Prionailurus planiceps KY682741 4	Lynx lynx NC027083 4	0,09130084
Prionailurus rubiginosus NC028304 2	Leopardus pardalis NC028315 1	0,09130177
Leptailurus serval NC028316 1	Catopuma temminckii NC027115 41	0,09136817
Profelis aurata NC028299 1	Catopuma badia NC028300 1	0,09136817
Puma yagouaroundi NC028311 1	Leptailurus serval NC028316 1	0,0914355
Lynx pardinus NC028319 161	Leopardus pardalis T262 1	0,09143721
Parahyaena brunnea NC038159 15	Crocuta crocuta NC020670 3	0,09149743
Prionailurus planiceps NC028312 6	Lynx lynx NC027083 4	0,09150283
Profelis aurata NC028299 1	Prionailurus viverrinus NC028305 1	0,09150372
Prionailurus bengalensis NC028301 12	Leopardus jacobita NC028322 1	0,09150407
Prionailurus rubiginosus NC028304 2	Leopardus geoffroyi NC028320 1	0,09150545
Leptailurus serval NC028316 1	Leopardus wiedii NC028318 1	0,09157097
Prionailurus viverrinus NC028305 1	Lynx lynx NC027083 4	0,09157287
Prionailurus rubiginosus NC028304 2	Leopardus jacobita NC028322 1	0,09163869
Hemigalus derbyanus MH464791 1	Chrotogale owstoni T607 1	0,09163979
Felis silvestris lybica KP202275 4	Catopuma badia NC028300 1	0,09170482
Prionailurus bengalensis CKM45 20	Caracal caracal NC028306 1	0,09170718
Prionailurus rubiginosus NC028304 2	Leopardus guigna NC028321 1	0,09170756
Felis silvestris lybica KP202275 4	Catopuma temminckii NC027115 41	0,09177215
Pardofelis marmorata NLN3 2	Catopuma temminckii NC027115 41	0,09177215
Prionailurus planiceps KY682741 4	Lynx pardinus NC028319 161	0,09177215
Profelis aurata NC028299 1	Felis chaus NC028307 1	0,09177215
Puma concolor NC016470 22	Felis silvestris lybica KP202275 4	0,09177215
Felis chaus NC028307 1	Caracal caracal NC028306 1	0,09183948
Pardofelis marmorata NLN3 2	Lynx pardinus NC028319 161	0,09183948
Prionailurus viverrinus NC028305 1	Catopuma badia NC028300 1	0,09184115
Prionailurus planiceps NC028312 6	Lynx pardinus NC028319 161	0,09190682
Puma concolor NC016470 22	Prionailurus planiceps KY682741 4	0,09190682
Puma yagouaroundi NC028311 1	Felis margarita NC028308 1	0,09190682
Puma concolor NC016470 22	Prionailurus viverrinus NC028305 1	0,09190849
Xenogale naso C07XAR110 1	Urva javanica/auropunctata NC006835 1	0,09193436
Pardofelis marmorata NLN3 2	Lynx canadensis NC028313 1	0,09204148
Lynx pardinus NC028319 161	Felis catus NC001700 2	0,09210881
Lynx pardinus NC028319 161	Felis margarita NC028308 1	0,09210881
Urva javanica/auropunctata NC006835 1	Atilax paludinosus T606 1	0,09213674
Puma concolor NC016470 22	Prionailurus planiceps NC028312 6	0,09217614
Puma yagouaroundi NC028311 1	Felis chaus NC028307 1	0,09217614
Prionailurus viverrinus NC028305 1	Lynx rufus NC014456 3	0,09217859
Galerella sanguinea T378 1	Bdeogale nigripes GLC15 1	0,09218321
Felis silvestris lybica KP202275 4	Caracal caracal NC028306 1	0,0923108
Lynx lynx NC027083 4	Felis silvestris lybica KP202275 4	0,0923108
Prionailurus planiceps KY682741 4	Lynx rufus NC014456 3	0,0923108
Puma yagouaroundi NC028311 1	Prionailurus planiceps KY682741 4	0,0923108
Lynx pardinus NC028319 161	Leopardus pardalis NC028315 1	0,09231241
Galerella sanguinea T378 1	Cynictis penicillata T375 1	0,09231783
Profelis aurata NC028299 1	Catopuma temminckii NC027115 41	0,09237813
Leopardus pardalis T262 1	Catopuma badia NC028300 1	0,09237938
Lynx canadensis NC028313 1	Leopardus pardalis NC028315 1	0,09238002
Urva javanica/auropunctata NC006835 1	Cynictis penicillata T375 1	0,09240516
Hyaena hyaena NC020669 1	Crocuta crocuta NC020670 3	0,09244063
Felis margarita NC028308 1	Catopuma temminckii NC027115 41	0,09244546
Puma concolor NC016470 22	Felis margarita NC028308 1	0,09244546
Leopardus pardalis NC028315 1	Felis margarita NC028308 1	0,09244627
Prionailurus planiceps KY682741 4	Leopardus pardalis T262 1	0,09244666
Felis catus NC001700 2	Catopuma temminckii NC027115 41	0,09251279
Felis chaus NC028307 1	Catopuma badia NC028300 1	0,09251279
Felis nigripes NC028309 1	Catopuma badia NC028300 1	0,09258012
Lynx canadensis NC028313 1	Felis catus NC001700 2	0,09258012
Lynx canadensis NC028313 1	Felis chaus NC028307 1	0,09258012
Lynx lynx NC027083 4	Leptailurus serval NC028316 1	0,09258012
Lynx rufus NC014456 3	Leptailurus serval NC028316 1	0,09258012
Prionailurus planiceps KY682741 4	Catopuma temminckii NC027115 41	0,09258012
Puma concolor NC016470 22	Felis nigripes NC028309 1	0,09258012
Prionailurus rubiginosus NC028304 2	Leopardus tigrinus NC028317 1	0,09258125
Viverra zibetha T609 1	Civettictis civetta GLC19 1	0,09258636
Otocolobus manul NC028323 1	Catopuma temminckii NC027115 41	0,09264746
Prionailurus planiceps NC028312 6	Catopuma temminckii NC027115 41	0,09264746
Puma yagouaroundi NC028311 1	Prionailurus planiceps NC028312 6	0,09264746
Otocolobus manul NC028323 1	Leopardus pardalis T262 1	0,09264813
Lynx rufus NC014456 3	Leopardus pardalis NC028315 1	0,09264899
Lynx canadensis NC028313 1	Felis margarita NC028308 1	0,09271479
Lynx pardinus NC028319 161	Felis chaus NC028307 1	0,09271479
Prionailurus viverrinus NC028305 1	Catopuma temminckii NC027115 41	0,09271641
Lynx pardinus NC028319 161	Leopardus colocolo NC028314 1	0,09272693

Viverra zibetha T609 1	Ursus spelaeus NC011112 8	0,18229833
Ursus arctos AP012576 6	Mustela nigripes NC024942 1	0,18229842
Ursus thibetanus laniger MH281753 2	Genetta servalina NC024568 2	0,18229868
Zalophus californianus NC008416 1	Ursus arctos EU497665 29	0,18230158
Ursus thibetanus thibetanus NC01111	Catopuma temminckii NC027115 41	0,18230562
Zalophus californianus NC008416 1	Leopardus pardalis T262 1	0,18231107
Prionailurus planiceps KY682741 4	Canis lupus familiaris NC002008 1231	0,18231241
Vulpes zerda KJ603240 1	Civettictis civetta NC033378 1	0,18231572
Tremarctos ornatus NC009969 2	Canis adustus KT448271 1	0,18231912
Martes flavigula NC012141 3	Leopardus tigrinus NC028317 1	0,18232088
Spilogale putorius NC010497 1	Martes martes T302 3	0,18232316
Mungos mungo MMC7 1	Enhydra lutris NC009692 1	0,18232538
Meles anakuma NC009677 1	Arctocephalus australis MG023139 1	0,18232584
Mungos mungo MMC7 1	Arctonyx collaris NC020645 1	0,18232796
Mustela kathiah NC023210 1	Arctocephalus forsteri KT693377 17	0,18232812
Callorhinus ursinus NC008415 1	Bassaricyon neblina SRX1097850 1	0,18233131
Vulpes corsac NC023958 1	Pusa caspica NC008431 1	0,1823319
Neophoca cinerea NC008419 1	Fossa fossana D350 1	0,18233253
Melogale moschata KP726273 1	Galidictis fasciata DM333 1	0,18233316
Lutra lutra LC050126 1	Genetta abyssinica MG489822 1	0,18233329
Vulpes corsac NC023958 1	Mirounga leonina NC008422 1	0,18233383
Mungotictis decemlineata NC027828 1	Meles meles T303 3	0,18233384
Martes pennanti NC020664 16	Crossarchus platycephalus C7R66 1	0,18233545
Mustela erminea T305 2	Galidictis fasciata DM333 1	0,18233714
Vulpes vulpes NC008434 5	Hemigalus derbyanus MH464791 1	0,1823374
Martes zibellina NC011579 39	Genetta servalina NC024568 2	0,18234079
Otocolobus manul NC028323 1	Meles anakuma NC009677 1	0,18234088
Meles anakuma NC009677 1	Civettictis civetta GLC19 1	0,18234123
Parahyaena brunnea NC038159 15	Ommatophoca rossii AY377287etc 1	0,18234135
Ictonyx striatus T299 1	Callorhinus ursinus NC008415 1	0,1823418
Paradoxurus jerdoni MH464793 1	Mustela eversmannii NC028013 1	0,18234262
Mustela kathiah NC023210 1	Caracal caracal NC028306 1	0,18234381
Ursus thibetanus mupinensis NC00875	Enhydra lutris NC009692 1	0,18234394
Lutra sumatrana NC035810 1	Felis nigripes NC028309 1	0,18234397
Mustela nigripes NC024942 1	Felis catus NC001700 2	0,18234412
Mustela eversmannii NC028013 1	Arctotherium sp NC030174 1	0,18234417
Leopardus geoffroyi NC028320 1	Callorhinus ursinus NC008415 1	0,1823443
Prionailurus planiceps NC028312 6	Melogale moschata KP726273 1	0,18234511
Melogale moschata V0735A 1	Felis nigripes NC028309 1	0,18234575
Neovison vison NC020641 3	Ichneumia albicauda T603 1	0,18234611
Ursus arctos pruinosus MG066703 3	Meles leucurus NC039173 4	0,18234624
Lynx lynx NC027083 4	Canis latrans NC008093 7	0,18234636
Viverra zibetha T609 1	Martes pennanti NC020664 16	0,18234658
Smilodon populator MF871700 1	Canis aureus KT448274 1	0,18234697
Martes americana NC020642 1	Leopardus guigna NC028321 1	0,1823473
Martes zibellina NC011579 39	Leopardus jacobita NC028322 1	0,18234752
Ursus arctos isabellinus 18B5 2	Martes americana NC020642 1	0,1823485
Tremarctos ornatus NC009969 2	Prionailurus bengalensis CKM45 20	0,18234852
Mustela nigripes NC024942 1	Lycaon pictus NC028427 2	0,18234853
Vulpes vulpes NC008434 5	Catopuma badia NC028300 1	0,18234886
Lynx canadensis NC028313 1	Aonyx cinerea NC035814 2	0,18234922
Prionailurus bengalensis NC028301 12	Neophoca cinerea NC008419 1	0,18234932
Nyctereutes procyonoides NC013700 3	Leptailurus serval NC028316 1	0,18234967
Martes zibellina NC011579 39	Canis aureus KT448274 1	0,18235061
Ursus arctos GU573491 207	Melogale moschata NC020644 1	0,18235146
Martes melampus NC009678 1	Helarctos malayanus NC009968 2	0,18235177
Phoca groenlandica NC008429 54	Panthera uncia KP202269 1	0,18235216
Prionailurus planiceps KY682741 4	Melogale moschata NC020644 1	0,18235232
Leopardus tigrinus NC028317 1	Arctocephalus pusillus NC008417 1	0,18235271
Catopuma temminckii NC027115 41	Canis aureus KT448274 1	0,18235393
Zalophus wollebaeki SRR4431565 1	Ursus maritimus NC003428 31	0,18235406
Martes martes T302 3	Leptailurus serval NC028316 1	0,18235511
Lutra lutra NC011358 9	Arctocephalus forsteri NC004023 28	0,18235566
Pusa hispida NC 008433 1	Otocyon megalotis SAF1 2	0,18235604
Lynx pardinus NC028319 161	Lycaon pictus NC028427 2	0,18235736
Arctictis binturong T605 2	Ailurus fulgens NC011124 1	0,1823599
Ursus arctos GU573486 5	Martes martes T302 3	0,18236028
Ursus arctos AP012576 6	Mustela sibirica AP017394 11	0,18236203
Felis margarita NC028308 1	Canis aureus KT448274 1	0,1823622
Ursus arctos AP012576 6	Mustela kathiah NC023210 1	0,18236229
Mustela kathiah NC023210 1	Ailuropoda melanoleuca NC009492 5	0,18236251
Ursus arctos AP012576 6	Ailurus fulgens styani NC009691 1	0,18236256
Proteles cristata T393 6	Mirounga angustirostris SRR10331586	0,18236361
Diplogale hosei MH464790 1	Bassariscus sumichrasti SRX1099089 1	0,18236551
Mustela nivalis T306 5	Mephitis mephitis NC020648 1	0,18236566
Ursus spelaeus EU327344 13	Neophoca cinerea NC008419 1	0,18236603
Zalophus californianus NC008416 1	Procyon lotor AB462049 4	0,18236788
Canis lupus chanco NC010340 4	Atilax paludinosus T606 1	0,18236811
Ursus thibetanus laniger MH281753 2	Leopardus pardalis NC028315 1	0,18236823
Viverricula indica NC025296 2	Ursus thibetanus laniger MH281753 2	0,18236825
Leopardus pardalis NC028315 1	Canis lupus chanco NC010340 4	0,18237093
Ursus thibetanus laniger MH281753 2	Caracal caracal NC028306 1	0,18237117
Ursus thibetanus mupinensis NC00875	Acinonyx jubatus NC005212 3	0,18237536
Phoca vitulina NC001325 1	Canis mesomelas KT448280 1	0,18237616
Zalophus californianus NC008416 1	Prionailurus bengalensis NC028301 12	0,18237759
Neophoca cinerea NC008419 1	Canis adustus KT448271 1	0,18237978
Mustela nigripes NC024942 1	Civettictis civetta NC033378 1	0,18238309
Viverricula indica KX891745 1	Cuon alpinus NC013445 3	0,18239017
Felis catus NC001700 2	Canis lupus chanco NC010340 4	0,18239054
Canis adustus KT448271 1	Arctocephalus forsteri KT693377 17	0,18239364
Ursus thibetanus mupinensis NC00875	Homotherium latidens MF871702 3	0,18239452
Mungos mungo MMC7 1	Meles leucurus NC039173 4	0,18239507
Melogale moschata KP726273 1	Arctocephalus forsteri KT693377 17	0,18239731
Martes melampus NC009678 1	Canis latrans NC008093 7	0,18239802
Mustela itatsi NC034330 19	Urva semitorquata MH464789 1	0,18239848
Ursus arctos isabellinus 18B5 2	Martes flavigula NC012141 3	0,18239935
Prionodon linsang ERR2391707 1	Meles meles T303 3	0,18239968
Martes americana NC020642 1	Galidictis fasciata DM333 1	0,18239997
Canis latrans NC008093 7	Atilax paludinosus T606 1	0,18240042
Salanoia concolor D378 1	Aonyx cinerea NC035814 2	0,18240345
Leopardus jacobita NC028322 1	Ailurus fulgens styani NC009691 1	0,18240692
Mustela altaica NC021751 1	Arctocephalus gazella BK010918 1	0,18240903
Lutra sumatrana NC035810 1	Catopuma temminckii NC027115 41	0,18241039

Prionailurus viverrinus NC028305 1	Leopardus pardalis T262 1	0,09277587
Pardofelis marmorata NLN3 2	Lynx rufus NC014456 3	0,09278212
Leopardus pardalis NC028315 1	Felis catus NC001700 2	0,09278303
Prionailurus viverrinus NC028305 1	Caracal caracal NC028306 1	0,09278335
Lynx canadensis NC028313 1	Leopardus wiedii NC028318 1	0,09278409
Felis margarita NC028308 1	Catopuma badia NC028300 1	0,09284945
Profelis aurata NC028299 1	Felis nigripes NC028309 1	0,09284945
Puma concolor NC016470 22	Otocolobus manul NC028323 1	0,09284945
Lynx rufus NC014456 3	Leopardus pardalis T262 1	0,09285109
Prionailurus bengalensis CKM45 20	Leopardus jacobita NC028322 1	0,09285315
Prionailurus bengalensis CKM45 20	Acinonyx jubatus NC005212 3	0,09286079
Felis catus NC001700 2	Caracal caracal NC028306 1	0,09291678
Felis catus NC001700 2	Catopuma badia NC028300 1	0,09291678
Prionailurus bengalensis NC028301 12	Leopardus tigrinus NC028317 1	0,09291799
Lynx pardinus NC028319 161	Leopardus guigna NC028321 1	0,09291822
Prionailurus bengalensis CKM45 20	Leopardus pardalis NC028315 1	0,0929202
Leptailurus serval NC028316 1	Felis nigripes NC028309 1	0,09298411
Leopardus pardalis NC028315 1	Felis chaus NC028307 1	0,09298442
Galerella sanguinea T378 1	Atilax paludinosus T606 1	0,09299108
Prionailurus bengalensis NC028301 12	Pardofelis marmorata NLN3 2	0,09305144
Prionailurus rubiginosus NC028304 2	Pardofelis marmorata NLN3 2	0,09305144
Profelis aurata NC028299 1	Prionailurus planiceps KY682741 4	0,09305144
Puma concolor NC016470 22	Felis catus NC001700 2	0,09305144
Puma concolor NC016470 22	Profelis aurata NC028299 1	0,09305144
Leptailurus serval NC028316 1	Leopardus pardalis T262 1	0,09305214
Lynx rufus NC014456 3	Leopardus jacobita NC028322 1	0,09305337
Urva semitorquata MH464789 1	Galerella sanguinea T378 1	0,09305834
Catopuma badia NC028300 1	Caracal caracal NC028306 1	0,09311877
Profelis aurata NC028299 1	Felis silvestris lybica KP202275 4	0,09311877
Profelis aurata NC028299 1	Prionailurus planiceps NC028312 6	0,09311877
Urva semitorquata MH464789 1	Cynictis penicillata T375 1	0,09311877
Leptailurus serval NC028316 1	Leopardus pardalis NC028315 1	0,09311936
Felis nigripes NC028309 1	Catopuma temminckii NC027115 41	0,0931861
Otocolobus manul NC028323 1	Caracal caracal NC028306 1	0,0931861
Ichneumia albicauda T603 1	Urva semitorquata MH464789 1	0,0931931
Prionailurus planiceps NC028312 6	Acinonyx jubatus NC005212 3	0,09319531
Viverra zibetha T609 1	Civettictis civetta NC033378 1	0,09321253
Prionailurus planiceps KY682741 4	Caracal caracal NC028306 1	0,09325343
Puma yagouaroundi NC028311 1	Felis catus NC001700 2	0,09325343
Prionailurus bengalensis CKM45 20	Leopardus pardalis T262 1	0,09325701
Prionailurus planiceps KY682741 4	Acinonyx jubatus NC005212 3	0,09326264
Lynx lynx NC027083 4	Leopardus guigna NC028321 1	0,0933217
Puma concolor NC016470 22	Leopardus pardalis T262 1	0,09332199
Prionailurus planiceps NC028312 6	Leopardus pardalis T262 1	0,09338939
Prionailurus bengalensis CKM45 20	Leopardus wiedii NC028318 1	0,09339172
Monachus schauinslandi NC008421 1	Lobodon carcinophaga NC008423 1	0,09339806
Leopardus pardalis T262 1	Felis silvestris lybica KP202275 4	0,09345662
Puma yagouaroundi NC028311 1	Felis nigripes NC028309 1	0,09352276
Puma yagouaroundi NC028311 1	Pardofelis marmorata NLN3 2	0,09352276
Prionailurus viverrinus NC028305 1	Leopardus pardalis NC028315 1	0,09358378
Felis chaus NC028307 1	Catopuma temminckii NC027115 41	0,09359009
Lynx canadensis NC028313 1	Felis nigripes NC028309 1	0,09359009
Prionailurus bengalensis CKM45 20	Pardofelis marmorata NLN3 2	0,09359237
Leopardus colocolo NC028314 1	Catopuma badia NC028300 1	0,09360321
Otocolobus manul NC028323 1	Leopardus wiedii NC028318 1	0,09365823
Xenogale naso C07XAR110 1	Galerella sanguinea T378 1	0,09366501
Profelis aurata NC028299 1	Felis catus NC001700 2	0,09372475
Leopardus pardalis T262 1	Felis chaus NC028307 1	0,0937251
Lynx pardinus NC028319 161	Leopardus wiedii NC028318 1	0,09372648
Prionailurus bengalensis NC028301 12	Leopardus guigna NC028321 1	0,09372765
Prionailurus bengalensis CKM45 20	Leopardus colocolo NC028314 1	0,09374026
Lynx lynx NC027083 4	Felis catus NC001700 2	0,09379208
Otocolobus manul NC028323 1	Lynx pardinus NC028319 161	0,09379208
Profelis aurata NC028299 1	Otocolobus manul NC028323 1	0,09379208
Xenogale naso C07XAR110 1	Urva brachyura KY117547 1	0,09379208
Ichneumia albicauda T603 1	Urva javanica/auropunctata NC006835 1	0,09382721
Monachus schauinslandi NC008421 1	Leptonychotes weddellii NC008424 1	0,0938646
Leptailurus serval NC028316 1	Acinonyx jubatus NC005212 3	0,09386808
Monachus monachus NC044972 5	Leptonychotes weddellii NC008424 1	0,09388591
Monachus monachus NC044972 5	Lobodon carcinophaga NC008423 1	0,09388975
Monachus monachus NC044972 5	Hydrurga leptonyx NC008425 1	0,09389012
Leopardus pardalis NC028315 1	Catopuma badia NC028300 1	0,09392797
Lynx lynx NC027083 4	Felis chaus NC028307 1	0,09399407
Lynx lynx NC027083 4	Felis margarita NC028308 1	0,09399407
Prionailurus planiceps NC028312 6	Caracal caracal NC028306 1	0,09399407
Otocolobus manul NC028323 1	Leopardus colocolo NC028314 1	0,09400662
Prionailurus planiceps KY682741 4	Leopardus colocolo NC028314 1	0,09400681
Puma concolor NC016470 22	Leopardus pardalis NC028315 1	0,09406251
Puma concolor NC016470 22	Leopardus jacobita NC028322 1	0,09406263
Prionailurus planiceps NC028312 6	Leopardus jacobita NC028322 1	0,0940631
Prionailurus bengalensis NC028301 12	Leopardus geoffroyi NC028320 1	0,09406418
Felis margarita NC028308 1	Caracal caracal NC028306 1	0,09412874
Zalophus wollebaeki SRR4431565 1	Callorhinus ursinus NC008415 1	0,09415544
Zalophus californianus NC008416 1	Callorhinus ursinus NC008415 1	0,09416326
Puma yagouaroundi NC028311 1	Profelis aurata NC028299 1	0,09419607
Hemigalus derbyanus MH464791 1	Diplogale hosei MH464790 1	0,0942509
Lynx rufus NC014456 3	Felis chaus NC028307 1	0,0942634
Prionailurus planiceps NC028312 6	Leopardus pardalis NC028315 1	0,09426478
Prionailurus planiceps KY682741 4	Leopardus jacobita NC028322 1	0,09426499
Prionailurus planiceps NC028312 6	Pardofelis marmorata NLN3 2	0,09433073
Urva javanica T413 1	Cynictis penicillata T375 1	0,09433073
Urva semitorquata MH464789 1	Bdeogale nigripes GLC15 1	0,09433073
Otocolobus manul NC028323 1	Leopardus pardalis NC028315 1	0,09433138
Leopardus wiedii NC028318 1	Catopuma badia NC028300 1	0,09433203
Ichneumia albicauda T603 1	Urva brachyura KY117547 1	0,09433827
Lynx canadensis NC028313 1	Leopardus guigna NC028321 1	0,09439921
Prionailurus planiceps KY682741 4	Leopardus pardalis NC028315 1	0,09439933
Leptailurus serval NC028316 1	Leopardus colocolo NC028314 1	0,09441056
Prionailurus planiceps NC028312 6	Leopardus colocolo NC028314 1	0,09447823
Lynx rufus NC014456 3	Felis nigripes NC028309 1	0,09453272
Xenogale naso C07XAR110 1	Urva javanica T413 1	0,09453272
Leopardus colocolo NC028314 1	Felis silvestris lybica KP202275 4	0,09454498
Prionailurus planiceps KY682741 4	Pardofelis marmorata NLN3 2	0,09460005

Vulpes zerda KJ603240 1	Civettictis civetta GLC19 1	0,18241039
Potos flavus T414 1	Genetta genetta T297 1	0,18241058
Viverra tangalunga MH464792 1	Paradoxurus hermaphroditus NLNC 1	0,1824109
Prionailurus bengalensis CKM45 20	Meles anakuma NC009677 1	0,18241166
Paguma larvata PDDS11 2	Conepatus chinga NC042596 1	0,18241224
Paradoxurus jerdoni MH464793 1	Melogale moschata NC020644 1	0,18241225
Vulpes vulpes NC008434 5	Felis silvestris lybica KP202275 4	0,18241264
Prionailurus planiceps KY682741 4	Melogale moschata KP726273 1	0,18241267
Vulpes lagopus NC026529 3	Leopardus pardalis T262 1	0,1824128
Bassariscus sumichrasti SRX1099089 1	Arctotherium sp NC030174 1	0,18241283
Mustela erminea T305 2	Cryptoprocta ferox CFC13 1	0,18241306
Melogale moschata V0735A 1	Leopardus jacobita NC028322 1	0,18241309
Nasua nasua NC020647 1	Mirounga angustirostris SRR10331586	0,18241349
Puma yagouaroundi NC028311 1	Martes zibellina NC011579 39	0,182414
Otaria byronia OTAB 1	Mustela nivalis T306 5	0,18241531
Ursus maritimus GU573488 Svalbard	Ailurus fulgens NC011124 1	0,18241547
Procyon lotor AB462046 3	Civettictis civetta GLC19 1	0,18241557
Felis nigripes NC028309 1	Canis latrans NC008093 7	0,18241563
Ommatophoca rossii AY377287etc 1	Canis anthus NC027956 2	0,18241706
Ursus maritimus NC003428 31	Mustela itatsi NC034330 19	0,18241791
Viverricula indica NC025296 2	Procyon lotor AB462049 4	0,18241794
Ursus arctos pruinosus MG066703 3	Mustela nigripes NC024942 1	0,18241826
Martes martes T302 3	Leopardus tigrinus NC028317 1	0,18241972
Procyon lotor AB462046 3	Otocolobus manul NC028323 1	0,18242045
Lutra lutra NC011358 9	Acinonyx jubatus NC005212 3	0,18242183
Profelis aurata NC028299 1	Bassariscus sumichrasti SRX1099089 1	0,18242733
Prionailurus bengalensis CKM45 20	Arctocephalus pusillus NC008417 1	0,18242802
Potos flavus T414 1	Acinonyx jubatus NC005212 3	0,18242891
Ursus thibetanus formosanus NC00933	Bassariscus sumichrasti SRX1099089 1	0,18242976
Mustela putorius NC020638 4	Helarctos malayanus NC009968 2	0,18242979
Gulo gulo NC009685 3	Fossa fossana D350 1	0,18243566
Zalophus californianus NC008416 1	Ursus arctos isabellinus 18B5 2	0,18243623
Crossarchus platycephalus C7R66 1	Arctocephalus forsteri NC004023 28	0,18243626
Felis catus NC001700 2	Chrysocyon brachyurus NC024172 1	0,18243709
Profelis aurata NC028299 1	Lycalopex sechurae KT448284 1	0,18243839
Ursus thibetanus laniger MH281753 2	Leopardus geoffroyi NC028320 1	0,18244006
Viverricula indica KX891751 1	Martes flavigula NC012141 3	0,18244138
Ursus americanus JX196366 3	Arctocephalus australis MG023139 1	0,18244319
Zalophus californianus NC008416 1	Viverra zibetha T609 1	0,18244334
Mephitis mephitis NC020648 1	Ictonyx striatus T299 1	0,1824452
Ursus spelaeus NC011112 8	Arctictis binturong T605 2	0,18244818
Gulo gulo NC009685 3	Civettictis civetta GLC19 1	0,18244846
Ursus thibetanus laniger MH281753 2	Arctictis binturong T605 2	0,18244955
Ursus spelaeus NC011112 8	Arctocephalus forsteri NC004023 28	0,18245155
Profelis aurata NC028299 1	Canis lupus chanco NC010340 4	0,18245256
Nyctereutes procyonoides NC013700 3	Halichoerus grypus NC001602 2	0,18245462
Mephitis mephitis NC020648 1	Martes flavigula NC012141 3	0,18245631
Prionailurus viverrinus NC028305 1	Martes flavigula NC012141 3	0,18245639
Leopardus colocolo NC028314 1	Chrysocyon brachyurus NC024172 1	0,18245743
Lutra sumatrana NC035810 1	Crossarchus platycephalus C7R66 1	0,18245988
Enhydra lutris NC009692 1	Crossarchus platycephalus C7R66 1	0,18246041
Meles anakuma NC009677 1	Crossarchus platycephalus C7R66 1	0,18246113
Nasua nasua NC020647 1	Melogale moschata V0735A 1	0,18246216
Mustela sibirica NC020637 6	Chrotogale owstoni T607 1	0,1824629
Mustela sibirica AP017394 11	Chrotogale owstoni T607 1	0,18246296
Viverra tangalunga MH464792 1	Arctocephalus australis MG023139 1	0,18246424
Mustela kathiah NC023210 1	Mungos mungo MMC7 1	0,18246476
Mustela sibirica AP017394 11	Arctocephalus gazella BK010918 1	0,18246488
Mustela putorius NC020638 4	Arctocephalus gazella BK010918 1	0,18246534
Mustela frenata NC020640 1	Urva brachyura KY117547 1	0,18246557
Mustela nigripes NC024942 1	Arctocephalus gazella BK010918 1	0,18246561
Mustela sibirica NC020637 6	Conepatus chinga NC042596 1	0,18246619
Crossarchus platycephalus C7R66 1	Canis anthus NC027956 2	0,18246722
Ursus maritimus GU573488 Svalbard	Eumetopias jubatus NC004030 10	0,18246736
Pusa sibirica NC008424 2	Canis anthus NC027956 2	0,18246765
Salanoia concolor D378 1	Meles leucurus NC039173 4	0,18246798
Lutrogale perspicillata NC035811 1	Ailurus fulgens NC011124 1	0,18246897
Homotherium latidens MF871702 3	Arctotherium sp NC030174 1	0,18246937
Martes martes T302 3	Urva semitorquata MH464789 1	0,18247019
Viverricula indica KX891745 1	Lutra sumatrana NC035810 1	0,1824719
Martes zibellina NC011579 39	Canis adustus KT448271 1	0,1824737
Poecilogale albinucha T602 1	Arctocephalus australis MG023139 1	0,18247506
Ursus arctos EU497665 29	Callorhinus ursinus NC008415 1	0,18247525
Viverra zibetha T609 1	Conepatus chinga NC042596 1	0,18247542
Viverricula indica KX891745 1	Mustela eversmannii NC028013 1	0,18247664
Vulpes lagopus NC026529 3	Viverra zibetha T609 1	0,18247679
Viverricula indica KX891751 1	Conepatus chinga NC042596 1	0,18247731
Martes melampus NC009678 1	Leopardus tigrinus NC028317 1	0,18247767
Viverra tangalunga MH464792 1	Leopardus tigrinus NC028317 1	0,18247767
Meles anakuma NC009677 1	Catopuma badia NC028300 1	0,18247774
Viverra tangalunga MH464792 1	Leopardus jacobita NC028322 1	0,18247776
Mustela kathiah NC023210 1	Felis nigripes NC028309 1	0,18247782
Leopardus jacobita NC028322 1	Callorhinus ursinus NC008415 1	0,1824782
Mustela nigripes NC024942 1	Civettictis civetta GLC19 1	0,18247867
Prionailurus viverrinus NC028305 1	Mustela eversmannii NC028013 1	0,18247955
Martes zibellina NC011579 39	Lynx rufus NC014456 3	0,18247992
Martes americana NC020642 1	Catopuma badia NC028300 1	0,18248007
Phoca vitulina NC001325 1	Nasua nasua NC020647 1	0,18248023
Tremarctos ornatus NC009969 2	Prionailurus viverrinus NC028305 1	0,18248026
Ursus arctos EU497665 29	Meles leucurus NC039173 4	0,1824809
Paradoxurus hermaphroditus NLNC 1	Canis anthus NC027956 2	0,18248124
Leopardus pardalis NC028315 1	Canis latrans NC008093 7	0,18248148
Lynx pardinus NC028319 161	Canis latrans NC008093 7	0,1824818
Mungotictis decemlineata NC027828 1	Martes foina NC020643 1	0,18248278
Ursus maritimus GU573488 Svalbard	Martes americana NC020642 1	0,18248306
Viverricula indica KX891751 1	Procyon lotor AB462046 3	0,18248358
Parahyaena brunnea NC038159 15	Mustela frenata NC020640 1	0,18248373
Lynx lynx NC027083 4	Aonyx cinerea NC035814 2	0,18248394
Ursus arctos EU497665 29	Arctocephalus pusillus NC008417 1	0,18248442
Ictonyx striatus T299 1	Arctocephalus pusillus NC008417 1	0,18248455
Mustela kathiah NC023210 1	Lycaon pictus NC028427 2	0,18248467
Ursus maritimus NC003428 31	Mustela putorius NC020638 4	0,18248481
Ursus maritimus GU573488 Svalbard	Mustela putorius NC020638 4	0,18248489

Puma yagouaroundi NC028311 1	Caracal caracal NC028306 1	0,09460005
Puma concolor NC016470 22	Leopardus wiedii NC028318 1	0,09460129
Lynx canadensis NC028313 1	Leopardus colocolo NC028314 1	0,09461269
Lycaon pictus NC028427 2	Canis aureus KT448274 1	0,09461787
Profelis aurata NC028299 1	Lynx rufus NC014456 3	0,09466738
Leopardus pardalis T262 1	Felis nigripes NC028309 1	0,09466834
Prionailurus planiceps NC028312 6	Leopardus wiedii NC028318 1	0,09466885
Leopardus colocolo NC028314 1	Felis chaus NC028307 1	0,09467871
Lynx lynx NC027083 4	Leopardus colocolo NC028314 1	0,09468006
Prionailurus viverrinus NC028305 1	Leopardus jacobita NC028322 1	0,09472834
Urva brachyura KY117547 1	Atilax paludinosus T606 1	0,09473471
Puma yagouaroundi NC028311 1	Prionailurus viverrinus NC028305 1	0,0947363
Lynx lynx NC027083 4	Leopardus jacobita NC028322 1	0,09473707
Puma concolor NC016470 22	Caracal caracal NC028306 1	0,09480204
Profelis aurata NC028299 1	Leopardus colocolo NC028314 1	0,09481375
Ommatophoca rossii AY377287etc 1	Mirounga leonina NC008422 1	0,09485417
Prionailurus planiceps KY682741 4	Leopardus wiedii NC028318 1	0,09487079
Leopardus jacobita NC028322 1	Catopuma badia NC028300 1	0,09493808
Prionailurus viverrinus NC028305 1	Leopardus colocolo NC028314 1	0,09494034
Eumetopias jubatus NC004030 10	Callorhinus ursinus NC008415 1	0,09505272
Lynx pardinus NC028319 161	Felis nigripes NC028309 1	0,09507137
Profelis aurata NC028299 1	Felis margarita NC028308 1	0,09507137
Leopardus wiedii NC028318 1	Felis chaus NC028307 1	0,09507187
Leopardus pardalis NC028315 1	Felis nigripes NC028309 1	0,09507215
Lynx pardinus NC028319 161	Leopardus geoffroyi NC028320 1	0,09507285
Lynx rufus NC014456 3	Leopardus wiedii NC028318 1	0,09507309
Prionailurus viverrinus NC028305 1	Pardofelis marmorata NLN3 2	0,09507327
Prionailurus viverrinus NC028305 1	Leopardus wiedii NC028318 1	0,0951323
Leopardus wiedii NC028318 1	Felis silvestris lybica KP202275 4	0,09513995
Monachus schauinslandi NC008421 1	Hydrurga leptonyx NC008425 1	0,09514814
Leopardus jacobita NC028322 1	Felis silvestris lybica KP202275 4	0,09520745
Pardofelis marmorata NLN3 2	Acinonyx jubatus NC005212 3	0,09521503
Pardofelis marmorata NLN3 2	Leptailurus serval NC028316 1	0,09527336
Leopardus pardalis T262 1	Felis margarita NC028308 1	0,09527431
Lynx lynx NC027083 4	Leopardus geoffroyi NC028320 1	0,09527432
Leptailurus serval NC028316 1	Leopardus jacobita NC028322 1	0,0952744
Prionailurus bengalensis CKM45 20	Leopardus guigna NC028321 1	0,09527881
Mustela kathiah NC023210 1	Mustela erminea T305 2	0,09530219
Leptailurus serval NC028316 1	Leopardus guigna NC028321 1	0,09534413
Otocolobus manul NC028323 1	Acinonyx jubatus NC005212 3	0,09541678
Lynx rufus NC014456 3	Leopardus colocolo NC028314 1	0,09541902
Leopardus wiedii NC028318 1	Felis nigripes NC028309 1	0,0954763
Leopardus pardalis T262 1	Catopuma temminckii NC027115 41	0,09547643
Lynx canadensis NC028313 1	Leopardus jacobita NC028322 1	0,09547763
Prionailurus bengalensis CKM45 20	Leopardus geoffroyi NC028320 1	0,0954807
Prionailurus viverrinus NC028305 1	Acinonyx jubatus NC005212 3	0,09548614
Prionailurus bengalensis CKM45 20	Leopardus tigrinus NC028317 1	0,09554644
Urva javanica/auropunctata NC006835 1	Bdeogale nigripes GLC15 1	0,09557028
Catopuma temminckii NC027115 41	Caracal caracal NC028306 1	0,09561002
Otocolobus manul NC028323 1	Lynx canadensis NC028313 1	0,09561002
Otocolobus manul NC028323 1	Leopardus jacobita NC028322 1	0,09561086
Lynx lynx NC027083 4	Felis nigripes NC028309 1	0,09574468
Urva javanica T413 1	Atilax paludinosus T606 1	0,09574468
Leopardus pardalis T262 1	Felis catus NC001700 2	0,0957458
Felis silvestris lybica KP202275 4	Acinonyx jubatus NC005212 3	0,09575397
Leopardus colocolo NC028314 1	Catopuma temminckii NC027115 41	0,09575728
Lycaon pictus NC028427 2	Canis latrans NC008093 7	0,09580901
Urva brachyura KY117547 1	Cynictis penicillata T375 1	0,09581202
Leopardus jacobita NC028322 1	Felis chaus NC028307 1	0,09581263
Leopardus pardalis NC028315 1	Catopuma temminckii NC027115 41	0,09581303
Leptailurus serval NC028316 1	Leopardus tigrinus NC028317 1	0,09588033
Lynx lynx NC027083 4	Leopardus tigrinus NC028317 1	0,09588175
Pardofelis marmorata NLN3 2	Leopardus pardalis T262 1	0,09594809
Puma concolor NC016470 22	Leopardus colocolo NC028314 1	0,0959591
Lynx pardinus NC028319 161	Leopardus jacobita NC028322 1	0,09601603
Prionailurus planiceps NC028312 6	Leopardus guigna NC028321 1	0,09601646
Felis nigripes NC028309 1	Caracal caracal NC028306 1	0,09608134
Lynx canadensis NC028313 1	Leopardus geoffroyi NC028320 1	0,09608249
Lynx rufus NC014456 3	Leopardus guigna NC028321 1	0,0960829
Otocolobus manul NC028323 1	Lynx lynx NC027083 4	0,09614867
Otocolobus manul NC028323 1	Lynx rufus NC014456 3	0,09614867
Leopardus jacobita NC028322 1	Felis margarita NC028308 1	0,09614987
Ommatophoca rossii AY377287etc 1	Mirounga angustirostris SRR10331586 1	0,09616695
Lynx canadensis NC028313 1	Caracal caracal NC028306 1	0,096216
Mungos mungo MMC7 1	Crossarchus platycephalus C7R66 1	0,09621926
Urva brachyura KY117547 1	Bdeogale nigripes GLC15 1	0,09628333
Leopardus colocolo NC028314 1	Felis margarita NC028308 1	0,09629553
Pardofelis marmorata NLN3 2	Felis silvestris lybica KP202275 4	0,09635066
Leopardus wiedii NC028318 1	Felis margarita NC028308 1	0,09635166
Prionailurus planiceps KY682741 4	Leopardus geoffroyi NC028320 1	0,09635298
Leopardus guigna NC028321 1	Felis chaus NC028307 1	0,09635396
Ichneumia albicauda T603 1	Urva javanica T413 1	0,09642597
Profelis aurata NC028299 1	Acinonyx jubatus NC005212 3	0,09642658
Prionailurus planiceps NC028312 6	Leopardus geoffroyi NC028320 1	0,09648761
Leptailurus serval NC028316 1	Leopardus geoffroyi NC028320 1	0,09648872
Urva javanica T413 1	Bdeogale nigripes GLC15 1	0,09655266
Leopardus jacobita NC028322 1	Felis catus NC001700 2	0,09655396
Lynx canadensis NC028313 1	Leopardus tigrinus NC028317 1	0,09655495
Felis chaus NC028307 1	Acinonyx jubatus NC005212 3	0,09656108
Leopardus geoffroyi NC028320 1	Felis silvestris lybica KP202275 4	0,09662228
Leopardus colocolo NC028314 1	Felis catus NC001700 2	0,09663232
Leopardus wiedii NC028318 1	Catopuma temminckii NC027115 41	0,09668843
Prionailurus planiceps KY682741 4	Leopardus guigna NC028321 1	0,09668979
Urva javanica/auropunctata NC006835 1	Galerella sanguinea T378 1	0,09672454
Leopardus wiedii NC028318 1	Felis catus NC001700 2	0,0967558
Lynx rufus NC014456 3	Leopardus tigrinus NC028317 1	0,09682401
Leopardus jacobita NC028322 1	Catopuma temminckii NC027115 41	0,09689055
Leopardus tigrinus NC028317 1	Catopuma badia NC028300 1	0,09689064
Puma concolor NC016470 22	Leopardus guigna NC028321 1	0,09689175
Leopardus guigna NC028321 1	Felis silvestris lybica KP202275 4	0,09689177
Leopardus geoffroyi NC028320 1	Felis chaus NC028307 1	0,09695985
Lycaon pictus NC028427 2	Canis lupus chanco NC010340 4	0,09697928
Lynx pardinus NC028319 161	Caracal caracal NC028306 1	0,09702397

Ursus arctos EU497665 29	Mustela nigripes NC024942 1	0,18248555
Zalophus wollebaeki SRR4431565 1	Crossarchus platycephalus C7R66 1	0,18248627
Ursus thibetanus mupinensis NC00875	Canis adustus KT448271 1	0,18248646
Prionailurus rubiginosus NC028304 2	Martes pennanti NC020664 16	0,18248665
Prionailurus viverrinus NC028305 1	Mustela altaica NC021751 1	0,18248679
Ursus arctos isabellinus 18B5 2	Genetta servalina NC024568 2	0,18248689
Melogale moschata NC020644 1	Leptailurus serval NC028316 1	0,18248741
Panthera onca NC022842 1	Mustela frenata NC020640 1	0,18248895
Phoca groenlandica NC008429 54	Hyaena hyaena NC020669 1	0,18248987
Melursus ursinus NC009970 2	Arctocephalus forsteri KT693377 17	0,18249035
Ursus arctos GU573486 5	Mustela itatsi NC034330 19	0,18249153
Neovison vison NC020641 3	Catopuma temminckii NC027115 41	0,1824946
Ursus spelaeus NC011112 8	Lutra sumatrana NC035810 1	0,1824955
Ursus thibetanus thibetanus NC01111	Neophoca cinerea NC008419 1	0,18249561
Ictonyx striatus T299 1	Catopuma badia NC028300 1	0,18249567
Zalophus californianus NC008416 1	Mungos mungo/gambianus SRR77048	0,18249807
Viverra zibetha T609 1	Ursus thibetanus laniger MH281753 2	0,18250069
Neovison vison NC020641 3	Canis mesomelas KT448280 1	0,18250266
Paguma larvata PDDS11 2	Mephitis mephitis NC020648 1	0,18250544
Ursus spelaeus NC011112 8	Puma yagouaroundi NC028311 1	0,18250866
Ursus thibetanus laniger MH281753 2	Arctocephalus forsteri NC004023 28	0,18250933
Ursus thibetanus formosanus NC0093	Arctictis binturong T605 2	0,18251316
Vulpes vulpes NC008434 5	Smilodon populator MF871700 1	0,1825134
Profelis aurata NC028299 1	Canis lupus familiaris NC002008 1231	0,18251553
Lycalopex sechurae KT448284 1	Acinonyx jubatus NC005212 3	0,18251613
Mustela eversmannii NC028013 1	Civettictis civetta NC033378 1	0,1825175
Monachus monachus NC044972 5	Galidia elegans D146 1	0,18251766
Helarctos malayanus NC009968 2	Arctictis binturong T605 2	0,18251859
Prionailurus planiceps KY682741 4	Gulo gulo NC009685 3	0,18251975
Panthera tigris NC010642 35	Monachus monachus NC044972 5	0,18252075
Leopardus geoffroyi NC028320 1	Gulo gulo NC009685 3	0,18252137
Viverra zibetha T609 1	Cuon alpinus NC013445 3	0,18252137
Paradoxurus jerdoni MH464793 1	Cuon alpinus NC013445 3	0,18252525
Ursus thibetanus mupinensis NC00875	Civettictis civetta NC033378 1	0,18252623
Leopardus wiedii NC028318 1	Eumetopias jubatus NC004030 10	0,18252712
Meles anakuma NC009677 1	Arctocephalus townsendi NC008420 1	0,18252726
Smilodon populator MF871700 1	Mustela nigripes NC024942 1	0,18252847
Meles meles T303 3	Eupleres goudotii D128 1	0,18252926
Prionailurus bengalensis NC028301 12	Cuon alpinus NC013445 3	0,1825303
Ommatophoca rossii AY377287etc 1	Urva brachyura KY117547 1	0,18253075
Melogale moschata KP726273 1	Eupleres goudotii D128 1	0,18253112
Viverricula indica KX891745 1	Lutra lutra NC011358 9	0,18253158
Neophoca cinerea NC008419 1	Mustela putorius NC020638 4	0,18253447
Potos flavus T414 1	Atilax paludinosus T606 1	0,18253559
Martes pennanti NC020664 16	Arctocephalus townsendi NC008420 1	0,18253767
Viverra zibetha T609 1	Callorhinus ursinus NC008415 1	0,18253891
Enhydra lutris NC009692 1	Arctotherium sp NC030174 1	0,18254046
Lynx rufus NC014456 3	Lutra lutra NC011358 9	0,18254155
Ursus arctos GU573491 207	Taxidea taxus NC020646 1	0,18254164
Mustela nivalis T306 5	Fossa fossana D350 1	0,18254189
Melogale moschata V0735A 1	Genetta servalina NC024568 2	0,18254246
Mustela altaica NC021751 1	Mungos mungo MMC7 1	0,18254253
Meles anakuma NC009677 1	Felis nigripes NC028309 1	0,18254273
Profelis aurata NC028299 1	Meles leucurus NC039173 4	0,18254308
Tremarctos ornatus NC009969 2	Mustela nivalis T306 5	0,18254457
Taxidea taxus NC020646 1	Profelis aurata NC028299 1	0,18254519
Meles anakuma NC009677 1	Leopardus guigna NC028321 1	0,1825453
Mustela sibirica NC020637 6	Felis catus NC001700 2	0,18254599
Mustela sibirica NC020637 6	Felis nigripes NC028309 1	0,18254612
Zalophus wollebaeki SRR4431565 1	Melogale moschata KP726273 1	0,18254772
Mustela kathiah NC023210 1	Catopuma temminckii NC027115 41	0,18254907
Zalophus wollebaeki SRR4431565 1	Genetta abyssinica MG489822 1	0,1825491
Leopardus tigrinus NC028317 1	Canis latrans NC008093 7	0,18254918
Martes zibellina NC011579 39	Leopardus wiedii NC028318 1	0,18254931
Felis silvestris lybica KP202275 4	Canis anthus NC027956 2	0,18254951
Martes zibellina NC011579 39	Leopardus geoffroyi NC028320 1	0,18254972
Felis catus NC001700 2	Canis latrans NC008093 7	0,18254974
Ursus arctos EU497665 29	Ailurus fulgens NC011124 1	0,18255013
Vulpes vulpes NC008434 5	Mirounga angustirostris SRR10331586	0,18255024
Ursus arctos GU573491 207	Ailurus fulgens NC011124 1	0,18255031
Lynx pardinus NC028319 161	Arctocephalus pusillus NC008417 1	0,18255176
Phoca fasciata NC008428 1	Otocyon megalotis SAF1 2	0,18255188
Ursus spelaeus NC011112 8	Canis adustus KT448271 1	0,18255351
Procyon lotor AB462049 4	Felis chaus NC028307 1	0,18255378
Felis catus NC001700 2	Canis aureus KT448274 1	0,18255389
Melogale moschata NC020644 1	Felis margarita NC028308 1	0,1825548
Genetta abyssinica MG489822 1	Enhydra lutris NC009692 1	0,18255518
Pardofelis marmorata NLN3 2	Martes martes T302 3	0,18255591
Felis nigripes NC028309 1	Bassariscus sumichrasti SRX1099089 1	0,18255706
Ursus thibetanus thibetanus NC01111	Lutra lutra LC050126 1	0,18255751
Phoca vitulina NC001325 1	Otocyon megalotis SAF1 2	0,18255776
Poecilogale albinucha T602 1	Leopardus geoffroyi NC028320 1	0,18255854
Ailurus fulgens styani NC009691 1	Acinonyx jubatus NC005212 3	0,18255895
Zalophus wollebaeki SRR4431565 1	Poecilogale albinucha T602 1	0,18256092
Ictonyx striatus T299 1	Felis silvestris lybica KP202275 4	0,18256168
Ursus spelaeus NC011112 8	Fossa fossana D350 1	0,1825652
Lycaon pictus NC028427 2	Felis chaus NC028307 1	0,18256536
Canis latrans NC008093 7	Acinonyx jubatus NC005212 3	0,18256575
Martes melampus NC009678 1	Canis lupus chanco NC010340 4	0,18256746
Viverricula indica KX891751 1	Ursus spelaeus NC011112 8	0,18256769
Ursus spelaeus NC011112 8	Civettictis civetta GLC19 1	0,18256772
Viverricula indica KX891745 1	Ursus spelaeus NC011112 8	0,18256782
Zalophus wollebaeki SRR4431565 1	Homotherium latidens MF871702 3	0,18257047
Ursus thibetanus laniger MH281753 2	Leopardus wiedii NC028318 1	0,1825708
Zalophus californianus NC008416 1	Ursus arctos GU573491 207	0,18257082
Ursus thibetanus laniger MH281753 2	Felis chaus NC028307 1	0,18257217
Leopardus pardalis T262 1	Canis lupus chanco NC010340 4	0,18257399
Taxidea taxus NC020646 1	Canis lupus familiaris NC002008 1231	0,18257563
Ursus thibetanus thibetanus NC01111	Catopuma badia NC028300 1	0,18257613
Zalophus californianus NC008416 1	Lynx pardinus NC028319 161	0,18257695
Leopardus tigrinus NC028317 1	Canis lupus familiaris NC002008 1231	0,1825802
Lynx pardinus NC028319 161	Canis lupus chanco NC010340 4	0,18258397
Leopardus pardalis NC028315 1	Gulo gulo NC009685 3	0,18258633

Leopardus guigna NC028321 1	Catopuma temminckii NC027115 41	0,09702678
Leopardus colocolo NC028314 1	Felis nigripes NC028309 1	0,09703608
Urva brachyura KY117547 1	Galerella sanguinea T378 1	0,09709843
Profelis aurata NC028299 1	Lynx pardinus NC028319 161	0,09715863
Leopardus tigrinus NC028317 1	Felis silvestris lybica KP202275 4	0,09715997
Felis catus NC001700 2	Acinonyx jubatus NC005212 3	0,09716795
Leopardus jacobita NC028322 1	Felis nigripes NC028309 1	0,09729457
Leopardus geoffroyi NC028320 1	Felis margarita NC028308 1	0,0972957
Leopardus guigna NC028321 1	Felis margarita NC028308 1	0,09729588
Caracal caracal NC028306 1	Acinonyx jubatus NC005212 3	0,09730216
Leopardus tigrinus NC028317 1	Felis chaus NC028307 1	0,09736114
Martes melampus NC009678 1	Martes flavigula NC012141 3	0,09748234
Pardofelis marmorata NLN3 2	Leopardus pardalis NC028315 1	0,09749668
Lynx pardinus NC028319 161	Leopardus tigrinus NC028317 1	0,0974974
Leopardus jacobita NC028322 1	Acinonyx jubatus NC005212 3	0,0975062
Puma yagouaroundi NC028311 1	Leopardus colocolo NC028314 1	0,09750777
Leopardus tigrinus NC028317 1	Catopuma temminckii NC027115 41	0,09756381
Leopardus geoffroyi NC028320 1	Felis catus NC001700 2	0,09756496
Callorhinus ursinus NC008415 1	Arctocephalus townsendi NC008420 1	0,0975812
Callorhinus ursinus NC008415 1	Arctocephalus australis MG023139 1	0,09758128
Cuon alpinus NC013445 3	Canis adustus KT448271 1	0,09758973
Lynx rufus NC014456 3	Leopardus geoffroyi NC028320 1	0,09763146
Lycaon pictus NC028427 2	Canis lupus familiaris NC002008 1231	0,0976458
Lynx lynx NC027083 4	Caracal caracal NC028306 1	0,09769728
Profelis aurata NC028299 1	Lynx canadensis NC028313 1	0,09769728
Profelis aurata NC028299 1	Leopardus wiedii NC028318 1	0,09769782
Puma yagouaroundi NC028311 1	Leopardus wiedii NC028318 1	0,09769815
Leopardus pardalis NC028315 1	Acinonyx jubatus NC005212 3	0,09770803
Prionailurus viverrinus NC028305 1	Leopardus guigna NC028321 1	0,09775803
Leopardus guigna NC028321 1	Felis nigripes NC028309 1	0,09776705
Leopardus guigna NC028321 1	Felis catus NC001700 2	0,0977671
Poecilogale albinucha T602 1	Ictonyx striatus T299 1	0,09780671
Pardofelis marmorata NLN3 2	Felis catus NC001700 2	0,09783194
Profelis aurata NC028299 1	Leopardus pardalis T262 1	0,09783247
Puma yagouaroundi NC028311 1	Leopardus pardalis T262 1	0,09783287
Puma concolor NC016470 22	Leopardus geoffroyi NC028320 1	0,09783433
Felis margarita NC028308 1	Acinonyx jubatus NC005212 3	0,09784111
Martes martes T302 3	Martes flavigula NC012141 3	0,09789252
Prionailurus viverrinus NC028305 1	Leopardus geoffroyi NC028320 1	0,09789268
Profelis aurata NC028299 1	Lynx lynx NC027083 4	0,09789927
Puma yagouaroundi NC028311 1	Leopardus jacobita NC028322 1	0,09790031
Leopardus wiedii NC028318 1	Acinonyx jubatus NC005212 3	0,09790977
Profelis aurata NC028299 1	Pardofelis marmorata NLN3 2	0,0979666
Leopardus pardalis T262 1	Caracal caracal NC028306 1	0,09796734
Leopardus geoffroyi NC028320 1	Catopuma temminckii NC027115 41	0,09796939
Felis nigripes NC028309 1	Acinonyx jubatus NC005212 3	0,09797562
Vulpes zerda KJ603240 1	Vulpes vulpes NC008434 5	0,09802733
Prionailurus planiceps NC028312 6	Leopardus tigrinus NC028317 1	0,0980356
Leopardus colocolo NC028314 1	Caracal caracal NC028306 1	0,09804616
Profelis aurata NC028299 1	Leopardus jacobita NC028322 1	0,09810183
Leopardus tigrinus NC028317 1	Felis catus NC001700 2	0,09810252
Pardofelis marmorata NLN3 2	Leopardus wiedii NC028318 1	0,09810285
Callorhinus ursinus NC008415 1	Arctocephalus pusillus NC008417 1	0,09812235
Profelis aurata NC028299 1	Leopardus pardalis NC028315 1	0,098169
Leopardus guigna NC028321 1	Catopuma badia NC028300 1	0,09817158
Pardofelis marmorata NLN3 2	Felis margarita NC028308 1	0,09823593
Lutra sumatrana NC035810 1	Aonyx cinerea NC035814 2	0,09825695
Leopardus pardalis NC028315 1	Caracal caracal NC028306 1	0,09830397
Puma yagouaroundi NC028311 1	Leopardus pardalis NC028315 1	0,09830415
Puma concolor NC016470 22	Leopardus tigrinus NC028317 1	0,09830461
Callorhinus ursinus NC008415 1	Arctocephalus forsteri KT693377 17	0,09838884
Martes zibellina NC011579 39	Martes flavigula NC012141 3	0,09842528
Leopardus wiedii NC028318 1	Caracal caracal NC028306 1	0,09843876
Leopardus tigrinus NC028317 1	Felis margarita NC028308 1	0,09843904
Leopardus pardalis T262 1	Acinonyx jubatus NC005212 3	0,09844881
Pardofelis marmorata NLN3 2	Felis chaus NC028307 1	0,09857258
Prionailurus planiceps KY682741 4	Leopardus tigrinus NC028317 1	0,09857418
Lycaon pictus NC028427 2	Canis anthus NC027956 2	0,09863661
Lynx rufus NC014456 3	Caracal caracal NC028306 1	0,09863991
Pusa hispida NC 008433 1	Erignathus barbatus NC008426 1	0,09864585
Prionailurus viverrinus NC028305 1	Leopardus tigrinus NC028317 1	0,0987006
Pardofelis marmorata NLN3 2	Leopardus colocolo NC028314 1	0,09878714
Pardofelis marmorata NLN3 2	Leopardus jacobita NC028322 1	0,09884363
Lycaon pictus NC028427 2	Canis adustus KT448271 1	0,0988751
Leopardus geoffroyi NC028320 1	Catopuma badia NC028300 1	0,09891215
Callorhinus ursinus NC008415 1	Arctocephalus gazella BK010918 1	0,09892737
Monachus monachus NC044972 5	Mirounga leonina NC008422 1	0,09893482
Pardofelis marmorata NLN3 2	Felis nigripes NC028309 1	0,09897657
Martes flavigula NC012141 3	Martes americana NC020642 1	0,09903072
Leopardus geoffroyi NC028320 1	Felis nigripes NC028309 1	0,09918103
Otocolobus manul NC028323 1	Leopardus geoffroyi NC028320 1	0,09918181
Phoca fasciata NC008428 1	Erignathus barbatus NC008426 1	0,09918231
Pardofelis marmorata NLN3 2	Otocolobus manul NC028323 1	0,09924589
Urva javanica T413 1	Galerella sanguinea T378 1	0,09925439
Puma yagouaroundi NC028311 1	Leopardus guigna NC028321 1	0,09931631
Lutra lutra LC050126 1	Aonyx cinerea NC035814 2	0,09940103
Lutra lutra NC011358 9	Aonyx cinerea NC035814 2	0,09940103
Leopardus colocolo NC028314 1	Acinonyx jubatus NC005212 3	0,09940334
Pusa sibirica NC008432 2	Erignathus barbatus NC008426 1	0,09944788
Monachus schauinslandi NC008421 1	Monachus monachus NC044972 5	0,09947958
Pardofelis marmorata NLN3 2	Caracal caracal NC028306 1	0,09951521
Otocolobus manul NC028323 1	Leopardus guigna NC028321 1	0,09951856
Lutrogale perspicillata NC035811 1	Lutra sumatrana NC035810 1	0,09967002
Vulpes zerda KJ603240 1	Vulpes corsac NC023958 1	0,09971049
Leopardus jacobita NC028322 1	Caracal caracal NC028306 1	0,09971819
Mungos mungo/gambianus SRR7704821 1	Crossarchus platycephalus C7R66 1	0,09978454
Callorhinus ursinus NC008415 1	Arctocephalus forsteri NC004023 28	0,09983991
Lycaon pictus NC028427 2	Canis mesomelas KT448280 1	0,09997846
Vulpes zerda KJ603240 1	Vulpes ferrilata NC027935 1	0,10011446
Panthera onca NC022842 1	Neofelis nebulosa NC008450 3	0,10014136
Puma yagouaroundi NC028311 1	Leopardus tigrinus NC028317 1	0,10018969
Lycalopex sechurae KT448284 1	Canis aureus KT448274 1	0,100202
Leptonychotes weddellii NC008424 1	Erignathus barbatus NC008426 1	0,10039052

Leopardus jacobita NC028322 1	Gulo gulo NC009685 3	0,18258667
Panthera pardus NC010641 5	Ommatophoca rossii AY377287etc 1	0,18258736
Paradoxurus jerdoni MH464793 1	Halichoerus grypus NC001602 2	0,18258961
Lynx lynx NC027083 4	Eumetopias jubatus NC004030 10	0,1825927
Lutra lutra LC050126 1	Crossarchus platycephalus C7R66 1	0,18259364
Tremarctos ornatus NC009969 2	Hemigalus derbyanus MH464791 1	0,18259607
Mustela nigripes NC024942 1	Arctocephalus townsendi NC008420 1	0,18259867
Melogale moschata KP726273 1	Arctocephalus australis MG023139 1	0,1825992
Salanoia concolor D378 1	Lutra lutra LC050126 1	0,18260066
Salanoia concolor D378 1	Melogale moschata V0735A 1	0,18260212
Procyon lotor AB462046 3	Chrotogale owstoni T607 1	0,18260217
Suricata suricatta SSM10 1	Mustela erminea T305 2	0,18260224
Tremarctos ornatus NC009969 2	Ailurus fulgens styani NC009691 1	0,18260336
Genetta abyssinica MG489822 1	Conepatus chinga NC042596 1	0,18260346
Ursus thibetanus thibetanus NC011118	Civettictis civetta NC033378 1	0,1826048
Ursus thibetanus thibetanus NC011118	Homotherium latidens MF871702 3	0,18260612
Ursus thibetanus laniger MH281753 2	Canis adustus KT448271 1	0,18260621
Tremarctos ornatus NC009969 2	Procyon lotor AB462046 3	0,18260624
Lutra lutra NC011358 9	Catopuma badia NC028300 1	0,18260637
Canis lupus chanco NC010340 4	Acinonyx jubatus NC005212 3	0,18260679
Mustela sibirica AP017394 11	Canis anthus NC027956 2	0,18260691
Mustela itatsi NC034330 19	Canis latrans NC008093 7	0,18260761
Otocolobus manul NC028323 1	Lutra lutra LC050126 1	0,18260761
Mungotictis decemlineata NC027828 1	Martes martes T302 3	0,18260968
Melogale moschata KP726273 1	Civettictis civetta GLC19 1	0,18261042
Ursus thibetanus thibetanus NC011118	Smilodon populator MF871700 1	0,18261074
Felis margarita NC028308 1	Ailurus fulgens NC011124 1	0,18261082
Ursus thibetanus formosanus NC00933	Smilodon populator MF871700 1	0,18261106
Mustela eversmannii NC028013 1	Leopardus jacobita NC028322 1	0,18261309
Tremarctos ornatus NC009969 2	Lynx rufus NC014456 3	0,18261331
Prionodon pardicolor NC024569 2	Meles meles T303 3	0,1826134
Paradoxurus jerdoni MH464793 1	Mustela altaica NC021751 1	0,18261355
Lutra sumatrana NC035810 1	Leopardus guigna NC028321 1	0,18261366
Civettictis civetta GLC19 1	Ailurus fulgens NC011124 1	0,18261372
Potos flavus T414 1	Felis silvestris lybica KP202275 4	0,18261379
Genetta servalina NC024568 2	Ailurus fulgens NC011124 1	0,1826138
Paguma larvata PDDS11 2	Melogale moschata NC020644 1	0,18261452
Prionailurus bengalensis CKM45 20	Callorhinus ursinus NC008415 1	0,18261483
Vulpes lagopus NC026529 3	Leopardus wiedii NC028318 1	0,18261498
Mustela eversmannii NC028013 1	Catopuma badia NC028300 1	0,18261534
Melogale moschata KP726273 1	Catopuma temminckii NC027115 41	0,18261538
Mustela sibirica NC020637 6	Leopardus guigna NC028321 1	0,18261559
Vulpes zerda KJ603240 1	Leopardus pardalis NC028315 1	0,1826161
Zalophus wollebaeki SRR4431565 1	Potos flavus T414 1	0,18261641
Martes zibellina NC011579 39	Leopardus guigna NC028321 1	0,18261707
Ursus arctos isabellinus 18B5 2	Ailurus fulgens styani NC009691 1	0,18261799
Procyon lotor AB462046 3	Genetta servalina NC024568 2	0,18261828
Mustela sibirica NC020637 6	Canis aureus KT448274 1	0,18261933
Ursus arctos EU497665 29	Mustela eversmannii NC028013 1	0,18261951
Ursus thibetanus mupinensis NC00875	Bassariscus sumichrasti SRX1099089 1	0,18262248
Ursus thibetanus laniger MH281753 2	Martes zibellina NC011579 39	0,18262312
Otocyon megalotis SAF1 2	Mirounga angustirostris SRR10331586	0,18262325
Poecilogale albinucha T602 1	Lynx pardinus NC028319 161	0,18262348
Felis silvestris lybica KP202275 4	Bassariscus sumichrasti SRX1099089 1	0,18262373
Profelis aurata NC028299 1	Canis aureus KT448274 1	0,18262509
Ursus spelaeus EU327344 13	Martes pennanti NC020664 16	0,18262509
Martes foina NC020643 1	Ichneumia albicauda T603 1	0,18262511
Viverra tangalunga MH464792 1	Arctocephalus forsteri NC004023 28	0,18262555
Puma yagouaroundi NC028311 1	Arctodus simus NC011116 1	0,18262565
Ursus thibetanus thibetanus NC011118	Melogale moschata V0735A 1	0,18262567
Lynx canadensis NC028313 1	Ictonyx striatus T299 1	0,18262675
Mirounga angustirostris SRR10331586	Hyaena hyaena NC020669 1	0,18262705
Lycaon pictus NC028427 2	Leopardus pardalis T262 1	0,18262771
Ursus spelaeus NC011112 8	Arctocephalus forsteri KT693377 17	0,18262972
Ursus thibetanus mupinensis NC00875	Caracal caracal NC028306 1	0,18262985
Ursus thibetanus formosanus NC00933	Genetta servalina NC024568 2	0,18263279
Ursus thibetanus formosanus NC00933	Leopardus pardalis NC028315 1	0,18263704
Ursus spelaeus EU327344 13	Arctocephalus pusillus NC008417 1	0,18263739
Ictonyx striatus T299 1	Genetta abyssinica MG489822 1	0,18263792
Procyon lotor AB462049 4	Mephitis mephitis NC020648 1	0,18263876
Leopardus tigrinus NC028317 1	Canis aureus KT448274 1	0,18263912
Paradoxurus hermaphroditus NC03959	Canis lupus chanco NC010340 4	0,18264587
Zalophus californianus NC008416 1	Leopardus geoffroyi NC028320 1	0,18264821
Leopardus geoffroyi NC028320 1	Canis lupus familiaris NC002008 1231	0,18265268
Smilodon populator MF871700 1	Mustela sibirica NC020637 6	0,18265955
Meles anakuma NC009677 1	Arctocephalus forsteri KT693377 17	0,1826628
Taxidea taxus NC020646 1	Prionodon linsang ERR2391707 1	0,18266524
Melogale moschata KP726273 1	Chrotogale owstoni T607 1	0,18266664
Neophoca cinerea NC008419 1	Melogale moschata KP726273 1	0,18266791
Mustela frenata NC020640 1	Mungos mungo/gambianus SRR77048	0,18266828
Ursus arctos GU573491 207	Martes flavigula NC012141 3	0,18266925
Paradoxurus jerdoni MH464793 1	Callorhinus ursinus NC008415 1	0,18266942
Neophoca cinerea NC008419 1	Mustela itatsi NC034330 19	0,18266952
Melogale moschata V0735A 1	Galidictis fasciata DM333 1	0,18266954
Tremarctos ornatus NC009969 2	Fossa fossana D350 1	0,18266961
Tremarctos ornatus NC009969 2	Arctocephalus australis MG023139 1	0,18266979
Mungos mungo MMC7 1	Arctocephalus gazella BK010918 1	0,18267035
Potos flavus T414 1	Urva semitorquata MH464789 1	0,18267047
Melogale moschata KP726273 1	Canis adustus KT448271 1	0,18267076
Ommatophoca rossii AY377287etc 1	Nyctereutes procyonoides NC013700 3	0,18267088
Vulpes ferrilata NC027935 1	Nandinia binotata NC024567 1	0,18267088
Phocarctos hookeri NC008418 1	Ailurus fulgens styani NC009691 1	0,1826714
Mustela frenata NC020640 1	Ichneumia albicauda T603 1	0,18267186
Otaria byronia OTAB 1	Aonyx cinerea NC035814 2	0,18267293
Smilodon populator MF871700 1	Mustela nivalis T306 5	0,18267404
Helarctos malayanus NC009968 2	Civettictis civetta NC033378 1	0,18267477
Nyctereutes procyonoides NC013700 3	Mustela erminea T305 2	0,18267477
Potos flavus T414 1	Paradoxurus hermaphroditus NLNC 1	0,18267524
Lutra lutra LC050126 1	Felis catus NC001700 2	0,18267593
Lutra lutra LC050126 1	Felis nigripes NC028309 1	0,18267603
Panthera onca KP202264 2	Meles meles T303 3	0,18267608
Poecilogale albinucha T602 1	Arctocephalus townsendi NC008420 1	0,18267651
Lutra lutra NC011358 9	Leopardus jacobita NC028322 1	0,18267672

Profelis aurata NC028299 1	Leopardus guigna NC028321 1	0,10039424
Mustela frenata NC020640 1	Mustela erminea T305 2	0,10041729
Ommatophoca rossii AY377287etc 1	Monachus schauinslandi NC008421 1	0,10042308
Leopardus tigrinus NC028317 1	Acinonyx jubatus NC005212 3	0,10046874
Lycalopex sechurae KT448284 1	Canis adustus KT448271 1	0,10055067
Otocolobus manul NC028323 1	Leopardus tigrinus NC028317 1	0,1005934
Leopardus tigrinus NC028317 1	Felis nigripes NC028309 1	0,10059365
Neophoca cinerea NC008419 1	Callorhinus ursinus NC008415 1	0,10060958
Leopardus guigna NC028321 1	Caracal caracal NC028306 1	0,10066324
Pusa caspica NC008431 1	Erignathus barbatus NC008426 1	0,10079451
Viverricula indica KX891745 1	Viverra zibetha T609 1	0,10086863
Puma yagouaroundi NC028311 1	Leopardus geoffroyi NC028320 1	0,10099961
Halichoerus grypus NC001602 2	Erignathus barbatus NC008426 1	0,10109231
Leopardus geoffroyi NC028320 1	Acinonyx jubatus NC005212 3	0,10114397
Lutrogale perspicillata NC035811 1	Lutra lutra NC011358 9	0,10115061
Ommatophoca rossii AY377287etc 1	Monachus monachus NC044972 5	0,10120999
Vulpes zerda KJ603240 1	Vulpes lagopus NC026529 3	0,10125901
Mungos mungo/gambianus SRR7704821 1	Helogale parvula SRR7637809 1	0,10133316
Viverricula indica KX891745 1	Civettictis civetta GLC19 1	0,10154198
Mustela kathiah NC023210 1	Mustela itatsi NC034330 19	0,10156145
Lobodon carcinophaga NC008423 1	Erignathus barbatus NC008426 1	0,10167675
Mustela putorius NC020638 4	Mustela kathiah NC023210 1	0,10169606
Panthera leo NERO 19	Neofelis nebulosa NC008450 3	0,10171083
Lycalopex sechurae KT448284 1	Canis latrans NC008093 7	0,1017345
Monachus schauinslandi NC008421 1	Mirounga leonina NC008422 1	0,10174274
Lutrogale perspicillata NC035811 1	Lutra lutra LC050126 1	0,10182328
Profelis aurata NC028299 1	Leopardus geoffroyi NC028320 1	0,10187548
Viverricula indica NC025296 2	Viverra zibetha T609 1	0,10188091
Leopardus guigna NC028321 1	Acinonyx jubatus NC005212 3	0,10188476
Chrysocyon brachyurus NC024172 1	Canis latrans NC008093 7	0,10194567
Phoca largha NC008430 1	Erignathus barbatus NC008426 1	0,10194675
Monachus monachus NC044972 5	Mirounga angustirostris SRR10331586 1	0,10211045
Viverricula indica KX891751 1	Viverra zibetha T609 1	0,102148
Phocarctos hookeri NC008418 1	Callorhinus ursinus NC008415 1	0,10215741
Phoca vitulina NC001325 1	Erignathus barbatus NC008426 1	0,10221624
Mustela nigripes NC024942 1	Mustela kathiah NC023210 1	0,10223448
Martes foina NC020643 1	Martes flavigula NC012141 3	0,10226534
Leopardus geoffroyi NC028320 1	Caracal caracal NC028306 1	0,10227919
Hydrurga leptonyx NC008425 1	Erignathus barbatus NC008426 1	0,10228273
Viverricula indica KX891745 1	Civettictis civetta NC033378 1	0,10237245
Diplogale hosei MH464790 1	Chrotogale owstoni T607 1	0,10244611
Lycaon pictus NC028427 2	Cuon alpinus NC013445 3	0,10249942
Lycalopex sechurae KT448284 1	Canis lupus chanco NC010340 4	0,10251277
Pardofelis marmorata NLN3 2	Leopardus guigna NC028321 1	0,1025472
Chrysocyon brachyurus NC024172 1	Canis lupus familiaris NC002008 1231	0,10257038
Phoca groenlandica NC008429 54	Erignathus barbatus NC008426 1	0,10261643
Panthera leo spelaea KX258452 2	Neofelis nebulosa NC008450 3	0,10267487
Leopardus tigrinus NC028317 1	Caracal caracal NC028306 1	0,10274804
Pardofelis marmorata NLN3 2	Leopardus geoffroyi NC028320 1	0,10295115
Paguma larvata PDDS11 2	Arctictis binturong T605 2	0,1029666
Erignathus barbatus NC008426 1	Cystophora cristata NC008427 1	0,10315109
Profelis aurata NC028299 1	Leopardus tigrinus NC028317 1	0,10315179
Viverricula indica KX891751 1	Civettictis civetta GLC19 1	0,10315803
Chrysocyon brachyurus NC024172 1	Canis aureus KT448274 1	0,10319321
Pusa hispida NC 008433 1	Leptonychotes weddellii NC008424 1	0,10335752
Otaria byronia OTAB 1	Callorhinus ursinus NC008415 1	0,10337169
Mustela kathiah NC023210 1	Mustela eversmannii NC028013 1	0,10337865
Mungos mungo MMC7 1	Helogale parvula SRR7637809 1	0,10342269
Mustela kathiah NC023210 1	Mustela altaica NC021751 1	0,10345068
Lycalopex sechurae KT448284 1	Canis lupus familiaris NC002008 1231	0,10352097
Viverra tangalunga MH464792 1	Mustela erminea T305 2	0,10358056
Pardofelis marmorata NLN3 2	Leopardus tigrinus NC028317 1	0,10362406
Lycalopex sechurae KT448284 1	Canis mesomelas KT448280 1	0,10368806
Viverricula indica NC025296 2	Civettictis civetta GLC19 1	0,10369947
Proteles cristata T393 6	Parahyaena brunnea NC038159 15	0,10384741
Mustela nivalis T306 5	Mustela kathiah NC023210 1	0,10385464
Panthera pardus NC010641 5	Neofelis nebulosa NC008450 3	0,10386203
Panthera pardus japonensis KJ866876 8	Neofelis nebulosa NC008450 3	0,10390835
Mustela sibirica NC020637 6	Mustela kathiah NC023210 1	0,10391708
Viverricula indica KX891751 1	Civettictis civetta NC033378 1	0,10392103
Monachus schauinslandi NC008421 1	Mirounga angustirostris SRR10331586 1	0,10397393
Phoca fasciata NC008428 1	Leptonychotes weddellii NC008424 1	0,10403089
Lycalopex sechurae KT448284 1	Chrysocyon brachyurus NC024172 1	0,10404101
Mustela sibirica AP017394 11	Mustela kathiah NC023210 1	0,10405169
Neovison vison NC020641 3	Mustela erminea T305 2	0,10405549
Chrysocyon brachyurus NC024172 1	Canis adustus KT448271 1	0,10408285
Pusa sibirica NC008432 2	Leptonychotes weddellii NC008424 1	0,10409372
Panthera uncia KP202269 1	Neofelis nebulosa NC008450 3	0,10417773
Viverricula indica NC025296 2	Civettictis civetta NC033378 1	0,10419373
Phoca fasciata NC008428 1	Lobodon carcinophaga NC008423 1	0,10430798
Martes martes T302 3	Gulo gulo NC009685 3	0,10434999
Panthera onca NC022842 1	Leopardus pardalis NC028315 1	0,10443665
Panthera onca KP202264 2	Neofelis nebulosa NC008450 3	0,10451434
Paradoxurus hermaphroditus NLNC 1	Arctictis binturong T605 2	0,10458227
Pusa caspica NC008431 1	Leptonychotes weddellii NC008424 1	0,1046997
Phoca largha NC008430 1	Leptonychotes weddellii NC008424 1	0,10484253
Proteles cristata T393 6	Hyaena hyaena NC020669 1	0,10499319
Mustela kathiah NC023210 1	Mustela frenata NC020640 1	0,10526316
Lycalopex sechurae KT448284 1	Canis anthus NC027956 2	0,10533241
Panthera tigris amoyensis NC014770 2	Neofelis nebulosa NC008450 3	0,10539205
Panthera pardus NC010641 5	Leopardus pardalis NC028315 1	0,1053974
Chrysocyon brachyurus NC024172 1	Canis lupus chanco NC010340 4	0,10547578
Leptonychotes weddellii NC008424 1	Halichoerus grypus NC001602 2	0,10553713
Phoca vitulina NC001325 1	Leptonychotes weddellii NC008424 1	0,10558342
Phoca largha NC008430 1	Hydrurga leptonyx NC008425 1	0,10559332
Panthera pardus japonensis KJ866876 8	Leopardus pardalis NC028315 1	0,1056432
Pusa hispida NC 008433 1	Hydrurga leptonyx NC008425 1	0,10564939
Chrysocyon brachyurus NC024172 1	Canis anthus NC027956 2	0,10571618
Paradoxurus hermaphroditus NC039591 1	Arctictis binturong T605 2	0,10572654
Martes zibellina NC011579 39	Gulo gulo NC009685 3	0,10582522
Pusa sibirica NC008432 2	Hydrurga leptonyx NC008425 1	0,10585425
Panthera onca NC022842 1	Caracal caracal NC028306 1	0,10598418
Panthera onca NC022842 1	Leopardus pardalis T262 1	0,1059855

Eupleres goudotii D128 1	Arctodus simus NC011116 1	0,18267722
Paradoxurus jerdoni MH464793 1	Meles leucurus NC039173 4	0,18267736
Felis nigripes NC028309 1	Ailurus fulgens styani NC009691 1	0,18267751
Ursus spelaeus NC011112 8	Gulo gulo NC009685 3	0,18267816
Martes flavigula NC012141 3	Canis lupus chanco NC010340 4	0,18267858
Ursus thibetanus mupinensis NC00875	Martes pennanti NC020664 16	0,18267891
Urocyon cinereoargenteus NC026723 2	Phoca groenlandica NC008429 54	0,18267924
Ursus arctos GU573491 207	Martes zibellina NC011579 39	0,18267988
Viverricula indica KX891751 1	Viverra tangalunga MH464792 1	0,18267989
Prionailurus viverrinus NC028305 1	Callorhinus ursinus NC008415 1	0,18268029
Procyon lotor AB462049 4	Arctotherium sp NC030174 1	0,18268044
Lutra sumatrana NC035810 1	Felis catus NC001700 2	0,18268053
Viverra tangalunga MH464792 1	Profelis aurata NC028299 1	0,18268158
Mungos mungo MMC7 1	Martes foina NC020643 1	0,1826818
Viverricula indica NC025296 2	Mustela putorius NC020638 4	0,1826819
Vulpes vulpes NC008434 5	Phoca largha NC008430 1	0,18268195
Puma yagouaroundi NC028311 1	Melogale moschata KP726273 1	0,18268245
Urocyon littoralis catalinae KP129018	Phoca largha NC008430 1	0,18268274
Puma concolor NC016470 22	Martes zibellina NC011579 39	0,18268299
Urocyon cinereoargenteus NC026723 2	Phoca vitulina NC001325 1	0,18268305
Viverricula indica KX891751 1	Mustela altaica NC021751 1	0,18268345
Paradoxurus hermaphroditus NC03959	Ailurus fulgens NC011124 1	0,18268381
Urocyon cinereoargenteus NC026723 2	Pusa hispida NC 008433 1	0,18268393
Ursus thibetanus mupinensis NC00875	Neophoca cinerea NC008419 1	0,18268438
Ursus arctos GU573486 5	Martes melampus NC009678 1	0,18268481
Ursus maritimus NC003428 31	Martes americana NC020642 1	0,18268499
Prionailurus rubiginosus NC028304 2	Nyctereutes procyonoides NC013700 3	0,18268518
Procyon lotor AB462049 4	Paguma larvata PDDS11 2	0,18268579
Viverra tangalunga MH464792 1	Lycaon pictus NC028427 2	0,18268603
Ursus arctos pruinosus MG066703 3	Mustela kathiah NC023210 1	0,18268639
Procyon lotor AB462046 3	Lynx pardinus NC028319 161	0,18268679
Procyon lotor AB462049 4	Leopardus pardalis NC028315 1	0,18268746
Procyon lotor AB462049 4	Leopardus pardalis T262 1	0,18268837
Ursus thibetanus formosanus NC00933	Homotherium latidens MF871702 3	0,18268858
Ursus thibetanus formosanus NC00933	Martes pennanti NC020664 16	0,18268873
Puma yagouaroundi NC028311 1	Melogale moschata NC020644 1	0,18268876
Melogale moschata NC020644 1	Catopuma badia NC028300 1	0,18268934
Melogale moschata NC020644 1	Felis chaus NC028307 1	0,18268996
Prionailurus viverrinus NC028305 1	Melogale moschata NC020644 1	0,18269005
Melursus ursinus NC009970 2	Callorhinus ursinus NC008415 1	0,18269038
Mustela nivalis T306 5	Leopardus tigrinus NC028317 1	0,1826904
Mustela putorius NC020638 4	Chrysocyon brachyurus NC024172 1	0,1826905
Mustela frenata NC020640 1	Lycalopex sechurae KT448284 1	0,18269107
Melursus ursinus NC009970 2	Martes zibellina NC011579 39	0,18269154
Meles meles T303 3	Arctictis binturong T605 2	0,18269233
Phoca largha NC008430 1	Otocyon megalotis SAF1 2	0,18269244
Martes foina NC020643 1	Genetta servalina NC024568 2	0,18269342
Ailurus fulgens NC011124 1	Acinonyx jubatus NC005212 3	0,18269375
Prionailurus bengalensis CKM45 20	Canis latrans NC008093 7	0,18269403
Ursus thibetanus formosanus NC00933	Neophoca cinerea NC008419 1	0,18269497
Viverra zibetha T609 1	Lycaon pictus NC028427 2	0,1826961
Paradoxurus jerdoni MH464793 1	Lycaon pictus NC028427 2	0,18269917
Ursus spelaeus EU327344 13	Arctonyx collaris NC020645 1	0,18270054
Ursus spelaeus EU327344 13	Aonyx cinerea NC035814 2	0,18270145
Zalophus californianus NC008416 1	Mungos mungo MMC7 1	0,18270177
Viverricula indica KX891751 1	Ursus spelaeus EU327344 13	0,18270238
Leopardus colocolo NC028314 1	Canis latrans NC008093 7	0,18270254
Zalophus wollebaeki SRR4431565 1	Melursus ursinus NC009970 2	0,18270339
Fossa fossana D350 1	Arctocephalus forsteri NC004023 28	0,182704
Lutra lutra NC011358 9	Canis adustus KT448271 1	0,1827078
Zalophus californianus NC008416 1	Mustela nivalis T306 5	0,18270813
Zalophus californianus NC008416 1	Leopardus pardalis NC028315 1	0,1827133
Leopardus pardalis NC028315 1	Canis lupus familiaris NC002008 1231	0,18271501
Civettictis civetta NC033378 1	Canis anthus NC027956 2	0,18271546
Zalophus californianus NC008416 1	Melursus ursinus NC009970 2	0,18271723
Ursus thibetanus thibetanus NC011118	Arctictis binturong T605 2	0,18271793
Paguma larvata PDDS11 2	Eumetopias jubatus NC004030 10	0,18272261
Pardofelis marmorata NLN3 2	Martes flavigula NC012141 3	0,1827262
Viverricula indica NC025296 2	Spilogale putorius NC010497 1	0,18272634
Felis nigripes NC028309 1	Canis lupus chanco NC010340 4	0,18272766
Leopardus pardalis NC028315 1	Eumetopias jubatus NC004030 10	0,18272862
Neovison vison NC020641 3	Cuon alpinus NC013445 3	0,18273026
Mustela sibirica AP017394 11	Arctocephalus forsteri KT693377 17	0,18273319
Diplogale hosei MH464790 1	Ailurus fulgens NC011124 1	0,18273354
Viverricula indica KX891751 1	Enhydra lutris NC009692 1	0,1827351
Phocarctos hookeri NC008418 1	Mustela kathiah NC023210 1	0,18273588
Prionodon linsang ERR2391707 1	Cystophora cristata NC008427 1	0,18273634
Pusa sibirica NC008432 2	Prionodon linsang ERR2391707 1	0,18273634
Neophoca cinerea NC008419 1	Mustela nigripes NC024942 1	0,18273677
Ursus arctos isabellinus 18B5 2	Eumetopias jubatus NC004030 10	0,18273678
Pusa caspica NC008431 1	Nasua nasua NC020647 1	0,18273689
Melogale moschata V0735A 1	Arctocephalus pusillus NC008417 1	0,18273698
Ursus arctos EU497665 29	Martes flavigula NC012141 3	0,18273708
Procyon lotor AB462046 3	Arctocephalus gazella BK010918 1	0,18273841
Phocarctos hookeri NC008418 1	Aonyx cinerea NC035814 2	0,18273894
Melogale moschata NC020644 1	Arctocephalus australis MG023139 1	0,18274051
Mungos mungo/gambianus SRR77048	Arctocephalus pusillus NC008417 1	0,18274051
Salanoia concolor D378 1	Potos flavus T414 1	0,18274198
Viverricula indica KX891745 1	Melogale moschata KP726273 1	0,18274395
Prionailurus viverrinus NC028305 1	Lutra lutra NC011358 9	0,18274468
Lutra lutra LC050126 1	Leopardus guigna NC028321 1	0,18274477
Melogale moschata NC020644 1	Galidictis fasciata DM333 1	0,18274489
Vulpes vulpes NC008434 5	Viverra zibetha T609 1	0,1827457
Melogale moschata KP726273 1	Genetta abyssinica MG489822 1	0,18274593
Ursus spelaeus EU327344 13	Gulo gulo NC009685 3	0,18274614
Vulpes ferrilata NC027935 1	Viverricula indica KX891751 1	0,18274632
Pardofelis marmorata NLN3 2	Mustela frenata NC020640 1	0,18274634
Taxidea taxus NC020646 1	Ailuropoda melanoleuca NC009492 5	0,18274674
Puma concolor NC016470 22	Mustela eversmannii NC028013 1	0,18274775
Lutra sumatrana NC035810 1	Leptailurus serval NC028316 1	0,18274781
Tremarctos ornatus NC009969 2	Prionailurus planiceps KY682741 4	0,18274842
Lynx pardinus NC028319 161	Arctocephalus forsteri KT693377 17	0,18274949
Mustela nivalis T306 5	Canis latrans NC008093 7	0,18275037

Mustela itatsi NC034330 19	Mustela frenata NC020640 1	0,1060035
Crossarchus platycephalus C7R66 1	Atilax paludinosus T606 1	0,10604633
Prionailurus bengalensis NC028301 12	Panthera onca NC022842 1	0,10611864
Phoca vitulina NC001325 1	Hydrurga leptonyx NC008425 1	0,10626661
Pusa caspica NC008431 1	Hydrurga leptonyx NC008425 1	0,10632539
Panthera uncia NC010638 1	Neofelis nebulosa NC008450 3	0,10633267
Mirounga leonina NC008422 1	Erignathus barbatus NC008426 1	0,10638298
Panthera leo NERO 19	Leopardus pardalis NC028315 1	0,10647979
Panthera onca NC022842 1	Leopardus wiedii NC028318 1	0,10652421
Pusa hispida NC 008433 1	Lobodon carcinophaga NC008423 1	0,10652503
Chrysocyon brachyurus NC024172 1	Canis mesomelas KT448280 1	0,10655015
Speothos venaticus C48 2	Canis adustus KT448271 1	0,10676356
Martes melampus NC009678 1	Gulo gulo NC009685 3	0,10676717
Phoca fasciata NC008428 1	Hydrurga leptonyx NC008425 1	0,1070014
Prionailurus rubiginosus NC028304 2	Panthera onca NC022842 1	0,10706139
Pusa sibirica NC008432 2	Lobodon carcinophaga NC008423 1	0,10706609
Panthera tigris NC010642 35	Neofelis nebulosa NC008450 3	0,10707556
Prionailurus planiceps KY682741 4	Panthera onca NC022842 1	0,10719581
Panthera pardus NC010641 5	Leopardus pardalis T262 1	0,10721596
Paradoxurus jerdoni MH464793 1	Arctictis binturong T605 2	0,10727572
Panthera pardus NC010641 5	Lynx lynx NC027083 4	0,10728251
Martes americana NC020642 1	Gulo gulo NC009685 3	0,10730594
Speothos venaticus C48 2	Canis latrans NC008093 7	0,10745759
Martes flavigula NC012141 3	Gulo gulo NC009685 3	0,10747208
Prionailurus bengalensis NC028301 12	Panthera pardus japonensis KJ866876 8	0,10752761
Mungos mungo MMC7 1	Urva semitorquata MH464789 1	0,10753009
Panthera pardus NC010641 5	Leopardus wiedii NC028318 1	0,10755217
Mustela frenata NC020640 1	Mustela altaica NC021751 1	0,10755652
Panthera pardus japonensis KJ866876 8	Leopardus wiedii NC028318 1	0,10766329
Prionailurus bengalensis NC028301 12	Panthera pardus NC010641 5	0,10768496
Prionailurus bengalensis NC028301 12	Panthera leo NERO 19	0,1076889
Mustela nivalis T306 5	Mustela frenata NC020640 1	0,10769139
Mungos mungo MMC7 1	Cynictis penicillata T375 1	0,10773081
Phoca groenlandica NC008429 54	Leptonychotes weddellii NC008424 1	0,10773432
Pusa caspica NC008431 1	Lobodon carcinophaga NC008423 1	0,10773937
Mustela nigripes NC024942 1	Mustela frenata NC020640 1	0,1077534
Panthera leo NERO 19	Leopardus pardalis T262 1	0,10775919
Lobodon carcinophaga NC008423 1	Halichoerus grypus NC001602 2	0,10776845
Urva semitorquata MH464789 1	Crossarchus platycephalus C7R66 1	0,10779693
Proteles cristata T393 6	Crocuta crocuta NC020670 3	0,10782142
Panthera leo NERO 19	Lynx lynx NC027083 4	0,10782518
Panthera onca NC022842 1	Felis silvestris lybica KP202275 4	0,1078692
Panthera onca NC022842 1	Leopardus colocolo NC028314 1	0,10788245
Hydrurga leptonyx NC008425 1	Halichoerus grypus NC001602 2	0,10790405
Phoca largha NC008430 1	Lobodon carcinophaga NC008423 1	0,10794977
Prionailurus rubiginosus NC028304 2	Panthera pardus NC010641 5	0,10795502
Mungos mungo MMC7 1	Xenogale naso C07XAR110 1	0,10800053
Prionailurus bengalensis NC028301 12	Panthera onca KP202264 2	0,10806625
Puma concolor NC016470 22	Panthera onca NC022842 1	0,10807144
Leptonychotes weddellii NC008424 1	Cystophora cristata NC008427 1	0,10813358
Hydrurga leptonyx NC008425 1	Cystophora cristata NC008427 1	0,10814243
Panthera leo NERO 19	Lynx rufus NC014456 3	0,10816077
Panthera pardus japonensis KJ866876 8	Leopardus pardalis T262 1	0,10826935
Prionailurus planiceps NC028312 6	Panthera onca NC022842 1	0,10827315
Phoca vitulina NC001325 1	Lobodon carcinophaga NC008423 1	0,10828641
Panthera pardus japonensis KJ866876 8	Lynx lynx NC027083 4	0,10833558
Prionailurus rubiginosus NC028304 2	Panthera pardus japonensis KJ866876 8	0,10833558
Panthera onca KP202264 2	Leopardus pardalis NC028315 1	0,10833695
Prionailurus bengalensis CKM45 20	Panthera onca NC022842 1	0,10834292
Lycaon pictus NC028427 2	Lycalopex sechurae KT448284 1	0,10835931
Prionailurus rubiginosus NC028304 2	Panthera onca KP202264 2	0,10840291
Panthera uncia KP202269 1	Leopardus pardalis NC028315 1	0,10840404
Lobodon carcinophaga NC008423 1	Cystophora cristata NC008427 1	0,10847886
Panthera onca NC022842 1	Felis chaus NC028307 1	0,10854263
Galerella sanguinea T378 1	Crossarchus platycephalus C7R66 1	0,1085448
Panthera onca KP202264 2	Leopardus pardalis T262 1	0,10860634
Mungos mungo MMC7 1	Atilax paludinosus T606 1	0,10860679
Panthera pardus NC010641 5	Felis silvestris lybica KP202275 4	0,10862828
Prionailurus bengalensis CKM45 20	Panthera leo NERO 19	0,10863402
Xenogale naso C07XAR110 1	Crossarchus platycephalus C7R66 1	0,10867223
Prionodon pardicolor NC024569 2	Prionodon linsang ERR2391707 1	0,10868199
Mustela sibirica AP017394 11	Mustela frenata NC020640 1	0,10869565
Mustela sibirica NC020637 6	Mustela frenata NC020640 1	0,10869565
Neovison vison NC020641 3	Mustela kathiah NC023210 1	0,10869961
Panthera pardus NC010641 5	Lynx pardinus NC028319 161	0,10876375
Panthera leo NERO 19	Leopardus wiedii NC028318 1	0,10876903
Panthera onca KP202264 2	Caracal caracal NC028306 1	0,10880689
Panthera onca NC022842 1	Leptailurus serval NC028316 1	0,10887926
Prionailurus viverrinus NC028305 1	Panthera onca NC022842 1	0,10888122
Neovison vison NC020641 3	Mustela itatsi NC034330 19	0,10890142
Phoca groenlandica NC008429 54	Hydrurga leptonyx NC008425 1	0,10895478
Panthera onca KP202264 2	Leopardus wiedii NC028318 1	0,10901026
Profelis aurata NC028299 1	Panthera onca NC022842 1	0,10901394
Lutra sumatrana NC035810 1	Enhydra lutris NC009692 1	0,10902483
Prionailurus bengalensis NC028301 12	Panthera leo spelaea KX258452 2	0,10905714
Mungos mungo MMC7 1	Galerella sanguinea T378 1	0,10908611
Phoca groenlandica NC008429 54	Lobodon carcinophaga NC008423 1	0,10908939
Monachus monachus NC044972 5	Erignathus barbatus NC008426 1	0,10917401
Panthera leo NERO 19	Felis silvestris lybica KP202275 4	0,10923751
Puma concolor NC016470 22	Panthera leo NERO 19	0,10923778
Panthera pardus japonensis KJ866876 8	Felis silvestris lybica KP202275 4	0,10927821
Panthera onca NC022842 1	Lynx lynx NC027083 4	0,10928298
Panthera onca NC022842 1	Lynx rufus NC014456 3	0,10928316
Panthera pardus japonensis KJ866876 8	Lynx pardinus NC028319 161	0,10934554
Prionailurus planiceps KY682741 4	Panthera onca KP202264 2	0,10934554
Panthera leo NERO 19	Leopardus tigrinus NC028317 1	0,10937535
Pardofelis marmorata NLN3 2	Panthera onca NC022842 1	0,10941792
Lutra lutra LC050126 1	Enhydra lutris NC009692 1	0,10942863
Mustela frenata NC020640 1	Mustela eversmannii NC028013 1	0,10943599
Panthera pardus NC010641 5	Leopardus colocolo NC028314 1	0,10944887
Cynictis penicillata T375 1	Crossarchus platycephalus C7R66 1	0,1094802
Mungos mungo/gambianus SRR7704821 1	Urva semitorquata MH464789 1	0,10954753
Panthera onca KP202264 2	Felis silvestris lybica KP202275 4	0,10954753

Callorhinus ursinus NC008415 1	Ailuropoda melanoleuca NC009492 5	0,18275067
Panthera leo NERO 19	Ommatophoca rossii AY377287etc 1	0,18275078
Urocyon littoralis catalinae KP129018	Pusa hispida NC 008433 1	0,18275119
Taxidea taxus NC020646 1	Panthera pardus japonensis KJ866876	0,18275173
Genetta servalina NC024568 2	Arctotherium sp NC030174 1	0,18275228
Ursus arctos EU497665 29	Martes americana NC020642 1	0,18275245
Ursus arctos isabellinus 18B5 2	Ailurus fulgens NC011124 1	0,18275259
Prionailurus bengalensis CKM45 20	Melogale moschata KP726273 1	0,18275329
Poecilogale albinucha T602 1	Arctocephalus pusillus NC008417 1	0,18275343
Procyon lotor AB462046 3	Lynx canadensis NC028313 1	0,18275352
Procyon lotor AB462046 3	Felis catus NC001700 2	0,18275462
Procyon lotor AB462046 3	Prionailurus planiceps KY682741 4	0,18275513
Ursus maritimus NC003428 31	Melogale moschata NC020644 1	0,18275549
Paguma larvata PDDS11 2	Lycalopex sechurae KT448284 1	0,1827558
Prionailurus planiceps NC028312 6	Melogale moschata NC020644 1	0,18275604
Ursus spelaeus EU327344 13	Callorhinus ursinus NC008415 1	0,18275747
Mustela nivalis T306 5	Felis silvestris lybica KP202275 4	0,18275806
Ursus thibetanus thibetanus NC01111	Martes pennanti NC020664 16	0,18275945
Ursus arctos pruinosus MG066703 3	Melogale moschata NC020644 1	0,18276201
Ursus thibetanus formosanus NC0093	Salanoia concolor D378 1	0,18276222
Ursus spelaeus NC011112 8	Arctocephalus townsendi NC008420 1	0,18276373
Leopardus colocolo NC028314 1	Ailurus fulgens styani NC009691 1	0,18276595
Mustela nigripes NC024942 1	Helarctos malayanus NC009968 2	0,18276688
Ursus arctos AP012576 6	Melogale moschata NC020644 1	0,18276694
Helarctos malayanus NC009968 2	Arctonyx collaris NC020645 1	0,18276717
Phoca groenlandica NC008429 54	Panthera leo spelaea KX258452 2	0,18276995
Lynx rufus NC014456 3	Canis aureus KT448274 1	0,1827703
Paradoxurus hermaphroditus NLNC 1	Ailuropoda melanoleuca NC009492 5	0,18277086
Felis margarita NC028308 1	Chrysocyon brachyurus NC024172 1	0,18277419
Ursus thibetanus thibetanus NC01111	Felis chaus NC028307 1	0,18277423
Ursus spelaeus NC011112 8	Poecilogale albinucha T602 1	0,18277439
Leopardus colocolo NC028314 1	Aonyx cinerea NC035814 2	0,18277515
Ursus thibetanus formosanus NC0093	Leopardus geoffroyi NC028320 1	0,1827759
Lontra canadensis SRR10409165 1	Homotherium latidens MF871702 3	0,18277723
Martes flavigula NC012141 3	Canis anthus NC027956 2	0,18277819
Meles anakuma NC009677 1	Diplogale hosei MH464790 1	0,18277884
Canis adustus KT448271 1	Arctocephalus townsendi NC008420 1	0,18278666
Panthera pardus NC010641 5	Mustela frenata NC020640 1	0,18278714
Viverricula indica KX891745 1	Ursus americanus JX196366 3	0,18278953
Gulo gulo NC009685 3	Catopuma badia NC028300 1	0,18279015
Prionodon pardicolor NC024569 2	Monachus monachus NC044972 5	0,18279034
Procyon lotor AB462046 3	Civettictis civetta NC033378 1	0,18279332
Prionailurus bengalensis NC028301 12	Eumetopias jubatus NC004030 10	0,18279417
Cystophora cristata NC008427 1	Cynogale bennetti KY117544 1	0,18279484
Leopardus pardalis T262 1	Eumetopias jubatus NC004030 10	0,18279594
Genetta abyssinica MG489822 1	Ailurus fulgens NC011124 1	0,18279737
Prionailurus bengalensis CKM45 20	Eumetopias jubatus NC004030 10	0,18279877
Mustela kathiah NC023210 1	Arctocephalus australis MG023139 1	0,18279949
Phocarctos hookeri NC008418 1	Meles leucurus NC039173 4	0,18280022
Melogale moschata V0735A 1	Arctocephalus townsendi NC008420 1	0,18280058
Melogale moschata V0735A 1	Arctocephalus australis MG023139 1	0,18280138
Mungotictis decemlineata NC027828 1	Martes melampus NC009678 1	0,18280214
Mungotictis decemlineata NC027828 1	Lutra lutra LC050126 1	0,1828029
Mustela frenata NC020640 1	Cynictis penicillata T375 1	0,18280321
Mustela erminea T305 2	Helogale parvula SRR7637809 1	0,18280409
Melogale moschata NC020644 1	Urva semitorquata MH464789 1	0,18280838
Panthera uncia KP202269 1	Ommatophoca rossii AY377287etc 1	0,18280976
Potos flavus T414 1	Galidictis fasciata DM333 1	0,18281001
Paradoxurus jerdoni MH464793 1	Meles anakuma NC009677 1	0,1828104
Ursus arctos GU573486 5	Martes flavigula NC012141 3	0,18281052
Puma yagouaroundi NC028311 1	Callorhinus ursinus NC008415 1	0,18281242
Viverricula indica NC025296 2	Lutra sumatrana NC035810 1	0,18281278
Catopuma badia NC028300 1	Callorhinus ursinus NC008415 1	0,18281294
Meles anakuma NC009677 1	Felis chaus NC028307 1	0,18281294
Ursus arctos isabellinus 18B5 2	Martes melampus NC009678 1	0,18281299
Urva semitorquata MH464789 1	Arctodus simus NC011116 1	0,18281345
Ursus thibetanus mupinensis NC00875	Arctocephalus townsendi NC008420 1	0,18281388
Martes melampus NC009678 1	Caracal caracal NC028306 1	0,1828142
Xenogale naso C07XAR110 1	Arctodus simus NC011116 1	0,18281433
Mustela sibirica AP017394 11	Felis catus NC001700 2	0,18281527
Melogale moschata KP726273 1	Felis margarita NC028308 1	0,1828167
Phoca largha NC008430 1	Nasua nasua NC020647 1	0,18281682
Phoca largha NC008430 1	Canis anthus NC027956 2	0,18281701
Vulpes lagopus NC026529 3	Prionailurus bengalensis NC028301 12	0,18281785
Martes americana NC020642 1	Leopardus jacobita NC028322 1	0,18281852
Ursus arctos isabellinus 18B5 2	Mustela frenata NC020640 1	0,18281935
Procyon lotor AB462049 4	Lynx lynx NC027083 4	0,1828202
Arctotherium sp NC030174 1	Aonyx cinerea NC035814 2	0,18282112
Nyctereutes procyonoides NC013700 3	Leopardus guigna NC028321 1	0,18282163
Ursus maritimus GU573488 Svalbard	Mustela itatsi NC034330 19	0,18282203
Procyon lotor AB462046 3	Felis margarita NC028308 1	0,18282263
Meles anakuma NC009677 1	Arctictis binturong T605 2	0,18282512
Zalophus wollebaeki SRR4431565 1	Ursus arctos isabellinus 18B5 2	0,18282537
Nandinia binotata NC024567 1	Mephitis mephitis NC020648 1	0,18282574
Ursus arctos GU573486 5	Mustela frenata NC020640 1	0,18282618
Felis nigripes NC028309 1	Arctodus simus NC011116 1	0,18282624
Canis aureus KT448274 1	Atilax paludinosus T606 1	0,18282741
Pardofelis marmorata NLN3 2	Canis adustus KT448271 1	0,18282828
Lycaon pictus NC028427 2	Civettictis civetta GLC19 1	0,18282959
Lycaon pictus NC028427 2	Leopardus wiedii NC028318 1	0,18282999
Martes foina NC020643 1	Felis chaus NC028307 1	0,18283057
Ursus thibetanus thibetanus NC01111	Salanoia concolor D378 1	0,18283176
Smilodon populator MF871700 1	Otocyon megalotis SAF1 2	0,18283202
Leptailurus serval NC028316 1	Ictonyx striatus T299 1	0,18283226
Zalophus californianus NC008416 1	Potos flavus T414 1	0,1828327
Mustela eversmannii NC028013 1	Helarctos malayanus NC009968 2	0,18283351
Neovison vison NC020641 3	Lycalopex sechurae KT448284 1	0,18283612
Ursus spelaeus EU327344 13	Civettictis civetta GLC19 1	0,18283708
Ursus thibetanus laniger MH281753 2	Felis margarita NC028308 1	0,18284084
Viverra zibetha T609 1	Mephitis mephitis NC020648 1	0,18284129
Melursus ursinus NC009970 2	Leopardus pardalis T262 1	0,18284228
Ursus thibetanus thibetanus NC01111	Caracal caracal NC028306 1	0,182843
Helarctos malayanus NC009968 2	Catopuma temminckii NC027115 41	0,18284477

Panthera onca NC022842 1	Catopuma badia NC028300 1	0,10955283
Prionailurus viverrinus NC028305 1	Panthera pardus japonensis KJ866876 8	0,10961661
Panthera onca NC022842 1	Felis catus NC001700 2	0,10961982
Lycalopex sechurae KT448284 1	Cuon alpinus NC013445 3	0,10962098
Panthera pardus NC010641 5	Caracal caracal NC028306 1	0,10963762
Panthera pardus NC010641 5	Leptailurus serval NC028316 1	0,10963784
Mustela putorius NC020638 4	Mustela frenata NC020640 1	0,10963791
Speothos venaticus C48 2	Canis lupus chanco NC010340 4	0,10964075
Prionailurus rubiginosus NC028304 2	Panthera leo NERO 19	0,10964191
Puma concolor NC016470 22	Panthera uncia KP202269 1	0,10968219
Panthera leo NERO 19	Leopardus geoffroyi NC028320 1	0,10971253
Panthera pardus japonensis KJ866876 8	Leopardus colocolo NC028314 1	0,10976216
Panthera leo NERO 19	Lynx pardinus NC028319 161	0,10977777
Panthera leo spelaea KX258452 2	Lynx lynx NC027083 4	0,10979509
Ommatophoca rossii AY377287etc 1	Erignathus barbatus NC008426 1	0,10980333
Puma concolor NC016470 22	Panthera leo spelaea KX258452 2	0,10986503
Panthera uncia KP202269 1	Felis chaus NC028307 1	0,10988419
Panthera pardus japonensis KJ866876 8	Leopardus jacobita NC028322 1	0,10988542
Prionailurus bengalensis CKM45 20	Panthera pardus japonensis KJ866876 8	0,10988647
Panthera onca NC022842 1	Felis margarita NC028308 1	0,1098893
Puma yagouaroundi NC028311 1	Panthera onca NC022842 1	0,10988963
Neovison vison NC020641 3	Mustela nigripes NC024942 1	0,10991083
Ichneumia albicauda T603 1	Crossarchus platycephalus C7R66 1	0,10995947
Prionailurus viverrinus NC028305 1	Panthera pardus NC010641 5	0,10997698
Panthera leo NERO 19	Lynx canadensis NC028313 1	0,10998005
Panthera uncia KP202269 1	Leopardus pardalis T262 1	0,1100202
Panthera onca NC022842 1	Lynx canadensis NC028313 1	0,1100237
Mungos mungo/gambianus SRR7704821 1	Galerella sanguinea T378 1	0,11002706
Panthera pardus japonensis KJ866876 8	Caracal caracal NC028306 1	0,11008618
Panthera tigris amoyensis NC014770 2	Leopardus wiedii NC028318 1	0,11009104
Panthera onca NC022842 1	Leopardus geoffroyi NC028320 1	0,11009316
Speothos venaticus C48 2	Canis aureus KT448274 1	0,11014017
Panthera pardus japonensis KJ866876 8	Leptailurus serval NC028316 1	0,11015351
Puma concolor NC016470 22	Panthera onca KP202264 2	0,11015351
Prionailurus bengalensis NC028301 12	Panthera tigris NC010642 35	0,11015692
Panthera onca KP202264 2	Leopardus colocolo NC028314 1	0,11016662
Panthera leo NERO 19	Leptailurus serval NC028316 1	0,11018062
Prionailurus bengalensis CKM45 20	Panthera leo spelaea KX258452 2	0,11020471
Crossarchus platycephalus C7R66 1	Bdeogale nigripes GLC15 1	0,11022084
Panthera uncia KP202269 1	Felis silvestris lybica KP202275 4	0,11022084
Prionailurus bengalensis NC028301 12	Panthera tigris amoyensis NC014770 2	0,11022431
Prionailurus bengalensis CKM45 20	Panthera pardus NC010641 5	0,1102468
Martes foina NC020643 1	Gulo gulo NC009685 3	0,11027119
Mungos mungo/gambianus SRR7704821 1	Atilax paludinosus T606 1	0,11028817
Lycaon pictus NC028427 2	Chrysocyon brachyurus NC024172 1	0,11029693
Panthera leo spelaea KX258452 2	Leopardus pardalis NC028315 1	0,11033605
Prionailurus bengalensis CKM45 20	Panthera onca KP202264 2	0,11035782
Speothos venaticus C48 2	Canis mesomelas KT448280 1	0,11035958
Panthera tigris amoyensis NC014770 2	Leopardus pardalis NC028315 1	0,11036018
Panthera leo NERO 19	Caracal caracal NC028306 1	0,11038265
Panthera uncia KP202269 1	Catopuma badia NC028300 1	0,11042284
Prionailurus bengalensis NC028301 12	Panthera uncia KP202269 1	0,11042284
Prionailurus planiceps NC028312 6	Panthera onca KP202264 2	0,11042284
Panthera onca NC022842 1	Leopardus tigrinus NC028317 1	0,11042975
Panthera leo spelaea KX258452 2	Felis silvestris lybica KP202275 4	0,11047108
Panthera pardus japonensis KJ866876 8	Lynx rufus NC014456 3	0,11049017
Suricata suricatta SSM10 1	Crossarchus platycephalus C7R66 1	0,11049017
Panthera onca NC022842 1	Lynx pardinus NC028319 161	0,11049511
Panthera pardus NC010641 5	Felis catus NC001700 2	0,11051366
Speothos venaticus C48 2	Canis anthus NC027956 2	0,11055449
Panthera uncia NC010638 1	Leopardus pardalis NC028315 1	0,11055862
Prionailurus viverrinus NC028305 1	Panthera onca KP202264 2	0,11055956
Neovison vison NC020641 3	Mustela eversmannii NC028013 1	0,11058377
Fossa fossana D350 1	Eupleres goudotii D128 1	0,11062647
Panthera pardus NC010641 5	Leopardus jacobita NC028322 1	0,11065066
Prionailurus viverrinus NC028305 1	Panthera leo NERO 19	0,11065485
Panthera leo spelaea KX258452 2	Leopardus pardalis T262 1	0,11067311
Prionailurus rubiginosus NC028304 2	Panthera leo spelaea KX258452 2	0,11067355
Panthera onca KP202264 2	Felis chaus NC028307 1	0,11069217
Panthera pardus japonensis KJ866876 8	Lynx canadensis NC028313 1	0,11069217
Prionailurus bengalensis CKM45 20	Panthera tigris amoyensis NC014770 2	0,11070446
Panthera pardus NC010641 5	Lynx canadensis NC028313 1	0,11071689
Panthera tigris amoyensis NC014770 2	Lynx lynx NC027083 4	0,11076232
Panthera uncia KP202269 1	Leopardus colocolo NC028314 1	0,11077237
Neovison vison NC020641 3	Mustela putorius NC020638 4	0,11078572
Panthera leo NERO 19	Felis catus NC001700 2	0,11078604
Panthera tigris NC010642 35	Leopardus wiedii NC028318 1	0,1108319
Panthera tigris amoyensis NC014770 2	Leopardus colocolo NC028314 1	0,11084359
Panthera leo spelaea KX258452 2	Leopardus wiedii NC028318 1	0,11087529
Puma yagouaroundi NC028311 1	Panthera onca KP202264 2	0,11089416
Mungos mungo MMC7 1	Ichneumia albicauda T603 1	0,11097189
Panthera onca KP202264 2	Lynx rufus NC014456 3	0,11102882
Panthera pardus japonensis KJ866876 8	Felis catus NC001700 2	0,11102882
Suricata suricatta SSM10 1	Mungos mungo/gambianus SRR7704821 1	0,11102882
Puma concolor NC016470 22	Panthera tigris amoyensis NC014770 2	0,1110329
Panthera leo NERO 19	Leopardus guigna NC028321 1	0,11105954
Panthera onca KP202264 2	Lynx lynx NC027083 4	0,11109615
Panthera onca KP202264 2	Lynx pardinus NC028319 161	0,11109615
Urva javanica T413 1	Crossarchus platycephalus C7R66 1	0,11109615
Panthera onca NC022842 1	Otocolobus manul NC028323 1	0,11110169
Mirounga angustirostris SRR10331586 1	Erignathus barbatus NC008426 1	0,11110552
Panthera pardus NC010641 5	Lynx rufus NC014456 3	0,11111905
Martes pennanti NC020664 16	Martes americana NC020642 1	0,11111986
Neovison vison NC020641 3	Mustela sibirica NC020637 6	0,11112238
Panthera leo NERO 19	Felis margarita NC028308 1	0,11112276
Panthera leo NERO 19	Leopardus jacobita NC028322 1	0,11112624
Mungos mungo/gambianus SRR7704821 1	Xenogale naso C07XAR110 1	0,11116348
Panthera onca KP202264 2	Leptailurus serval NC028316 1	0,11116348
Panthera onca KP202264 2	Lynx canadensis NC028313 1	0,11116348
Panthera onca NC022842 1	Felis nigripes NC028309 1	0,1111685
Lutra lutra NC011358 9	Enhydra lutris NC009692 1	0,11117841
Speothos venaticus C48 2	Canis lupus familiaris NC002008 1231	0,11118237
Panthera onca KP202264 2	Catopuma badia NC028300 1	0,11123081

Paradoxurus hermaphroditus NC03959	Canis lupus familiaris NC002008 1231	0,18284746
Puma concolor NC016470 22	Canis lupus familiaris NC002008 1231	0,18285151
Phoca largha NC008430 1	Canis mesomelas KT448280 1	0,18285312
Otocolobus manul NC028323 1	Martes flavigula NC012141 3	0,1828554
Ursus americanus JX196366 3	Mustela frenata NC020640 1	0,18285623
Genetta abyssinica MG489822 1	Canis lupus chanco NC010340 4	0,18285641
Ommatophoca rossii AY377287etc 1	Urva javanica T413 1	0,18285787
Ommatophoca rossii AY377287etc 1	Bdeogale nigripes GLC15 1	0,18286008
Spilogale putorius NC010497 1	Paguma larvata PDDS11 2	0,18286014
Prionailurus rubiginosus NC028304 2	Eumetopias jubatus NC004030 10	0,18286163
Cuon alpinus NC013445 3	Caracal caracal NC028306 1	0,18286608
Mustela itatsi NC034330 19	Chrotogale owstoni T607 1	0,18286675
Viverra tangalunga MH464792 1	Arctocephalus townsendi NC008420 1	0,18286745
Urva semitorquata MH464789 1	Arctonyx collaris NC020645 1	0,18286823
Callorhinus ursinus NC008415 1	Atilax paludinosus T606 1	0,18286873
Ommatophoca rossii AY377287etc 1	Helogale parvula SRR7637809 1	0,18287092
Ursus maritimus NC003428 31	Eumetopias jubatus NC004030 10	0,18287133
Canis adustus KT448271 1	Arctocephalus australis MG023139 1	0,18287134
Ursus thibetanus formosanus NC0093	Civettictis civetta NC033378 1	0,18287307
Ichneumia albicauda T603 1	Bassariscus sumichrasti SRX1099089 1	0,18287624
Potos flavus T414 1	Paradoxurus hermaphroditus NC03959	0,18287684
Procyon lotor AB462049 4	Arctocephalus pusillus NC008417 1	0,18287998
Lutra sumatrana NC035810 1	Canis adustus KT448271 1	0,18288086
Neovison vison NC020641 3	Xenogale naso C07XAR110 1	0,18288104
Viverricula indica KX891751 1	Mustela sibirica AP017394 11	0,18288116
Profelis aurata NC028299 1	Ailurus fulgens styani NC009691 1	0,18288182
Phoca fasciata NC008428 1	Nasua nasua NC020647 1	0,18288183
Ursus maritimus GU573488 Svalbard	Martes zibellina NC011579 39	0,18288191
Mustela eversmannii NC028013 1	Civettictis civetta GLC19 1	0,18288209
Mustela putorius NC020638 4	Leopardus tigrinus NC028317 1	0,18288223
Lutra sumatrana NC035810 1	Leopardus tigrinus NC028317 1	0,1828828
Mustela putorius NC020638 4	Caracal caracal NC028306 1	0,18288326
Mustela nivalis T306 5	Arctocephalus pusillus NC008417 1	0,18288344
Mustela itatsi NC034330 19	Felis chaus NC028307 1	0,18288393
Vulpes vulpes NC008434 5	Felis margarita NC028308 1	0,1828842
Mustela kathiah NC023210 1	Genetta genetta T297 1	0,18288437
Prionailurus bengalensis NC028301 12	Melogale moschata KP726273 1	0,18288441
Viverra zibetha T609 1	Melogale moschata NC020644 1	0,18288484
Urocyon littoralis catalinae KP129018	Phoca vitulina NC001325 1	0,18288507
Prionailurus planiceps KY682741 4	Martes zibellina NC011579 39	0,18288518
Melogale moschata V0735A 1	Leopardus guigna NC028321 1	0,18288596
Salanoia concolor D378 1	Martes foina NC020643 1	0,18288648
Ursus arctos EU497665 29	Mustela frenata NC020640 1	0,18288687
Mustela sibirica NC020637 6	Lycaon pictus NC028427 2	0,18288709
Procyon lotor AB462049 4	Genetta servalina NC024568 2	0,18288711
Poecilogale albinucha T602 1	Paradoxurus hermaphroditus NLNC 1	0,18288717
Poecilogale albinucha T602 1	Conepatus chinga NC042596 1	0,18288727
Procyon lotor AB462049 4	Lynx pardinus NC028319 161	0,18288846
Ursus arctos GU573491 207	Mustela eversmannii NC028013 1	0,18288849
Ursus maritimus GU573488 Svalbard	Melogale moschata NC020644 1	0,18289019
Zalophus wollebaeki SRR4431565 1	Procyon lotor AB462049 4	0,18289158
Ursus thibetanus formosanus NC0093	Arctocephalus australis MG023139 1	0,18289268
Neovison vison NC020641 3	Felis catus NC001700 2	0,18289432
Pardofelis marmorata NLN3 2	Ictonyx striatus T299 1	0,182898
Lutra sumatrana NC035810 1	Acinonyx jubatus NC005212 3	0,18289863
Ursus thibetanus mupinensis NC00875	Catopuma temminckii NC027115 41	0,18290065
Viverricula indica NC025296 2	Ailuropoda melanoleuca NC009492 5	0,18290082
Prionodon pardicolor NC024569 2	Phoca vitulina NC001325 1	0,18290094
Prionodon pardicolor NC024569 2	Phoca largha NC008430 1	0,18290104
Prionodon pardicolor NC024569 2	Neovison vison NC020641 3	0,18290168
Speothos venaticus C48 2	Homotherium latidens MF871702 3	0,18290244
Paradoxurus hermaphroditus NLNC 1	Chrysocyon brachyurus NC024172 1	0,18290298
Ursus arctos AP012576 6	Mustela eversmannii NC028013 1	0,1829036
Smilodon populator MF871700 1	Canis latrans NC008093 7	0,18290445
Ursus thibetanus thibetanus NC01111	Otaria byronia OTAB 1	0,18290497
Mungos mungo/gambianus SRR77048	Canis lupus familiaris NC002008 1231	0,18290538
Leopardus jacobita NC028322 1	Chrysocyon brachyurus NC024172 1	0,18290882
Lycalopex sechurae KT448284 1	Felis silvestris lybica KP202275 4	0,18291202
Leopardus wiedii NC028318 1	Canis mesomelas KT448280 1	0,18291207
Ursus americanus JX196366 3	Arctocephalus townsendi NC008420 1	0,18291368
Leopardus geoffroyi NC028320 1	Chrysocyon brachyurus NC024172 1	0,18291484
Melogale moschata V0735A 1	Civettictis civetta NC033378 1	0,18291493
Conepatus chinga NC042596 1	Callorhinus ursinus NC008415 1	0,18292023
Mustela nivalis T306 5	Canis lupus chanco NC010340 4	0,1829212
Puma yagouaroundi NC028311 1	Gulo gulo NC009685 3	0,18292354
Galictis vittata T412 1	Eumetopias jubatus NC004030 10	0,18292646
Mustela kathiah NC023210 1	Arctocephalus townsendi NC008420 1	0,18293363
Phocarctos hookeri NC008418 1	Meles meles T303 3	0,18293469
Prionailurus bengalensis CKM45 20	Canis lupus chanco NC010340 4	0,18293765
Vulpes corsac NC023958 1	Pusa sibirica NC008432 2	0,18293779
Ursus spelaeus EU327344 13	Canis adustus KT448271 1	0,18293914
Galerella sanguinea T378 1	Canis latrans NC008093 7	0,18293931
Crossarchus platycephalus C7R66 1	Arctocephalus gazella BK010918 1	0,18293951
Smilodon populator MF871700 1	Lycalopex sechurae KT448284 1	0,18293983
Urocyon littoralis catalinae KP129018	Erignathus barbatus NC008426 1	0,18293983
Prionodon linsang ERR2391707 1	Canis latrans NC008093 7	0,18294281
Lutra lutra NC011358 9	Leptailurus serval NC028316 1	0,18294398
Leopardus wiedii NC028318 1	Ailurus fulgens styani NC009691 1	0,18294539
Poecilogale albinucha T602 1	Callorhinus ursinus NC008415 1	0,18294744
Felis chaus NC028307 1	Arctonyx collaris NC020645 1	0,18294786
Prionailurus rubiginosus NC028304 2	Ailurus fulgens styani NC009691 1	0,18294805
Otocolobus manul NC028323 1	Ailurus fulgens styani NC009691 1	0,18294901
Paradoxurus hermaphroditus NLNC 1	Mustela sibirica NC020637 6	0,1829491
Ursus maritimus NC003428 31	Martes zibellina NC011579 39	0,18294924
Cynictis penicillata T375 1	Arctodus simus NC011116 1	0,18294932
Viverricula indica KX891745 1	Tremarctos ornatus NC009969 2	0,18295008
Leopardus guigna NC028321 1	Callorhinus ursinus NC008415 1	0,18295041
Potos flavus T414 1	Felis nigripes NC028309 1	0,18295047
Potos flavus T414 1	Catopuma temminckii NC027115 41	0,18295068
Pardofelis marmorata NLN3 2	Martes americana NC020642 1	0,18295148
Poecilogale albinucha T602 1	Neophoca cinerea NC008419 1	0,18295169
Vulpes lagopus NC026529 3	Phoca vitulina NC001325 1	0,18295169
Ursus arctos GU573486 5	Taxidea taxus NC020646 1	0,18295191

Panthera uncia KP202269 1	Caracal caracal NC028306 1	0,11123081
Profelis aurata NC028299 1	Panthera onca KP202264 2	0,11123081
Panthera onca KP202264 2	Leopardus geoffroyi NC028320 1	0,11123274
Puma concolor NC016470 22	Panthera pardus NC010641 5	0,11125449
Panthera leo spelaea KX258452 2	Lynx rufus NC014456 3	0,11127779
Panthera leo spelaea KX258452 2	Lynx pardinus NC028319 161	0,11127786
Panthera onca KP202264 2	Felis catus NC001700 2	0,11129814
Panthera onca KP202264 2	Felis margarita NC028308 1	0,11129814
Profelis aurata NC028299 1	Panthera uncia KP202269 1	0,11129814
Panthera uncia KP202269 1	Leopardus wiedii NC028318 1	0,11129949
Panthera onca NC022842 1	Leopardus jacobita NC028322 1	0,11130507
Prionailurus planiceps KY682741 4	Panthera pardus NC010641 5	0,11132249
Panthera leo NERO 19	Leopardus colocolo NC028314 1	0,11133867
Panthera leo spelaea KX258452 2	Leopardus colocolo NC028314 1	0,11135845
Panthera pardus japonensis KJ866876 8	Felis nigripes NC028309 1	0,11136547
Prionailurus rubiginosus NC028304 2	Panthera tigris amoyensis NC014770 2	0,11136896
Panthera pardus NC010641 5	Felis margarita NC028308 1	0,11138899
Panthera leo spelaea KX258452 2	Felis catus NC001700 2	0,11141415
Prionailurus planiceps KY682741 4	Panthera pardus japonensis KJ866876 8	0,11150014
Prionailurus planiceps KY682741 4	Panthera uncia KP202269 1	0,11150014
Speothos venaticus C48 2	Lycaon pictus NC028427 2	0,11150153
Panthera leo NERO 19	Catopuma badia NC028300 1	0,1115277
Prionailurus planiceps KY682741 4	Panthera leo NERO 19	0,11152811
Suricata suricatta SSM10 1	Mungos mungo MMC7 1	0,11156958
Panthera pardus NC010641 5	Felis nigripes NC028309 1	0,11159129
Puma yagouaroundi NC028311 1	Panthera leo NERO 19	0,11159487
Neovison vison NC020641 3	Mustela nivalis T306 5	0,11159886
Panthera uncia KP202269 1	Felis catus NC001700 2	0,1116348
Panthera tigris amoyensis NC014770 2	Leopardus pardalis T262 1	0,11163961
Viverra tangalunga MH464792 1	Mustela nigripes NC024942 1	0,11165702
Martes pennanti NC020664 16	Martes melampus NC009678 1	0,11165722
Neovison vison NC020641 3	Mustela sibirica AP017394 11	0,11166085
Cuon alpinus NC013445 3	Chrysocyon brachyurus NC024172 1	0,11166325
Panthera pardus japonensis KJ866876 8	Felis margarita NC028308 1	0,11170213
Panthera uncia KP202269 1	Felis margarita NC028308 1	0,11170213
Puma concolor NC016470 22	Panthera pardus japonensis KJ866876 8	0,11170213
Prionailurus viverrinus NC028305 1	Panthera leo spelaea KX258452 2	0,11175341
Panthera tigris NC010642 35	Leopardus pardalis NC028315 1	0,11177424
Panthera onca NC022842 1	Leopardus guigna NC028321 1	0,11177659
Panthera pardus NC010641 5	Felis chaus NC028307 1	0,11179215
Panthera leo spelaea KX258452 2	Leopardus jacobita NC028322 1	0,11181859
Prionailurus planiceps KY682741 4	Panthera leo spelaea KX258452 2	0,11181927
Prionailurus rubiginosus NC028304 2	Panthera uncia KP202269 1	0,11183679
Profelis aurata NC028299 1	Panthera pardus japonensis KJ866876 8	0,11183679
Panthera tigris NC010642 35	Leopardus geoffroyi NC028320 1	0,11184244
Martes zibellina NC011579 39	Martes pennanti NC020664 16	0,11185908
Panthera pardus NC010641 5	Leopardus tigrinus NC028317 1	0,11186281
Martes pennanti NC020664 16	Martes martes T302 3	0,11186752
Panthera uncia KP202269 1	Felis nigripes NC028309 1	0,11190412
Panthera pardus NC010641 5	Leopardus geoffroyi NC028320 1	0,111931
Neovison vison NC020641 3	Mustela altaica NC021751 1	0,11193526
Panthera leo spelaea KX258452 2	Felis margarita NC028308 1	0,11195268
Panthera leo spelaea KX258452 2	Leptailurus serval NC028316 1	0,11195453
Mungos mungo/gambianus SRR7704821 1	Cynictis penicillata T375 1	0,11197145
Panthera uncia KP202269 1	Lynx rufus NC014456 3	0,11197145
Panthera pardus japonensis KJ866876 8	Leopardus tigrinus NC028317 1	0,11197266
Puma concolor NC016470 22	Panthera tigris NC010642 35	0,11197564
Viverra tangalunga MH464792 1	Mustela itatsi NC034330 19	0,11199354
Prionailurus planiceps NC028312 6	Panthera pardus NC010641 5	0,11199606
Urva javanica/auropunctata NC006835 1	Crossarchus platycephalus C7R66 1	0,1120077
Panthera leo spelaea KX258452 2	Leopardus tigrinus NC028317 1	0,11202134
Profelis aurata NC028299 1	Panthera pardus NC010641 5	0,11206265
Panthera pardus japonensis KJ866876 8	Leopardus geoffroyi NC028320 1	0,11210819
Neofelis nebulosa NC008450 3	Felis chaus NC028307 1	0,11212622
Panthera pardus NC010641 5	Leopardus guigna NC028321 1	0,11213311
Pardofelis marmorata NLN3 2	Panthera uncia KP202269 1	0,11217345
Prionailurus planiceps NC028312 6	Panthera pardus japonensis KJ866876 8	0,11217345
Panthera uncia NC010638 1	Leopardus pardalis T262 1	0,11217474
Panthera tigris NC010642 35	Lynx lynx NC027083 4	0,11217622
Panthera tigris amoyensis NC014770 2	Catopuma badia NC028300 1	0,11217672
Prionailurus rubiginosus NC028304 2	Panthera tigris NC010642 35	0,11217696
Procyon lotor AB462046 3	Bassariscus sumichrasti SRX1099089 1	0,1121879
Puma yagouaroundi NC028311 1	Panthera pardus NC010641 5	0,11219721
Pardofelis marmorata NLN3 2	Panthera onca KP202264 2	0,11224078
Prionailurus planiceps NC028312 6	Panthera uncia KP202269 1	0,11224078
Prionailurus bengalensis CKM45 20	Panthera tigris NC010642 35	0,11225326
Panthera tigris NC010642 35	Leopardus colocolo NC028314 1	0,11225782
Panthera onca KP202264 2	Leopardus tigrinus NC028317 1	0,1123098
Panthera uncia KP202269 1	Leopardus guigna NC028321 1	0,1123099
Prionailurus bengalensis CKM45 20	Panthera uncia KP202269 1	0,11231069
Panthera tigris amoyensis NC014770 2	Leptailurus serval NC028316 1	0,11231118
Panthera pardus NC010641 5	Catopuma badia NC028300 1	0,11233193
Prionailurus planiceps NC028312 6	Panthera leo spelaea KX258452 2	0,11235831
Urva brachyura KY117547 1	Crossarchus platycephalus C7R66 1	0,11237544
Panthera tigris NC010642 35	Leopardus pardalis T262 1	0,11238039
Panthera tigris amoyensis NC014770 2	Leopardus geoffroyi NC028320 1	0,11238101
Viverra tangalunga MH464792 1	Mustela kathiah NC023210 1	0,11239737
Viverra tangalunga MH464792 1	Mustela putorius NC020638 4	0,11239737
Panthera leo NERO 19	Catopuma temminckii NC027115 41	0,11240295
Panthera uncia KP202269 1	Leopardus geoffroyi NC028320 1	0,1124445
Phoca fasciata NC008428 1	Mirounga leonina NC008422 1	0,11244747
Prionailurus planiceps NC028312 6	Panthera leo NERO 19	0,11247139
Panthera tigris amoyensis NC014770 2	Lynx pardinus NC028319 161	0,11251323
Panthera tigris NC010642 35	Lynx pardinus NC028319 161	0,11251325
Panthera onca NC022842 1	Catopuma temminckii NC027115 41	0,1125152
Panthera leo spelaea KX258452 2	Caracal caracal NC028306 1	0,11255978
Panthera leo spelaea KX258452 2	Leopardus geoffroyi NC028320 1	0,11256035
Speothos venaticus C48 2	Chrysocyon brachyurus NC024172 1	0,11258615
Panthera uncia NC010638 1	Felis silvestris lybica KP202275 4	0,11264476
Mungos mungo/gambianus SRR7704821 1	Ichneumia albicauda T603 1	0,11265392
Panthera leo spelaea KX258452 2	Lynx canadensis NC028313 1	0,11269173
Panthera uncia KP202269 1	Leopardus jacobita NC028322 1	0,11271349
Panthera tigris amoyensis NC014770 2	Leopardus jacobita NC028322 1	0,1127845

Phoca vitulina NC001325 1	Canis anthus NC027956 2	0,18295202
Prionailurus planiceps NC028312 6	Martes zibellina NC011579 39	0,18295239
Phoca largha NC008430 1	Nyctereutes procyonoides NC013700 3	0,18295321
Prionailurus planiceps KY682741 4	Canis anthus NC027956 2	0,18295398
Taxidea taxus NC020646 1	Galerella sanguinea T378 1	0,18295404
Halichoerus grypus NC001602 2	Canis mesomelas KT448280 1	0,18295439
Prionailurus viverrinus NC028305 1	Nyctereutes procyonoides NC013700 3	0,18295519
Mustela kathiah NC023210 1	Arctocephalus forsteri NC004023 28	0,18295549
Melogale moschata NC020644 1	Genetta genetta T297 1	0,18295597
Ursus arctos GU573486 5	Arctocephalus gazella BK010918 1	0,18295726
Prionailurus bengalensis NC028301 12	Melogale moschata NC020644 1	0,18295783
Procyon lotor AB462046 3	Felis chaus NC028307 1	0,18295822
Procyon lotor AB462049 4	Otocolobus manul NC028323 1	0,18295878
Felis nigripes NC028309 1	Canis aureus KT448274 1	0,18295935
Viverricula indica KX891745 1	Martes foina NC020643 1	0,18295941
Zalophus wollebaeki SRR4431565 1	Ursus arctos GU573491 207	0,18296012
Leopardus jacobita NC028322 1	Aonyx cinerea NC035814 2	0,18296014
Prionodon pardicolor NC024569 2	Mustela nivalis T306 5	0,18296179
Ursus thibetanus mupinensis NC00875	Lynx canadensis NC028313 1	0,18296212
Ursus thibetanus mupinensis NC00875	Lynx pardinus NC028319 161	0,18296295
Melursus ursinus NC009970 2	Meles leucurus NC039173 4	0,18296428
Martes foina NC020643 1	Felis nigripes NC028309 1	0,18296535
Puma yagouaroundi NC028311 1	Ictonyx striatus T299 1	0,18296574
Ursus thibetanus mupinensis NC00875	Felis chaus NC028307 1	0,18296586
Meles anakuma NC009677 1	Helarctos malayanus NC009968 2	0,18296731
Viverricula indica KX891745 1	Ailuropoda melanoleuca NC009492 5	0,18296738
Ursus thibetanus mupinensis NC00875	Leopardus guigna NC028321 1	0,18296829
Zalophus wollebaeki SRR4431565 1	Leopardus tigrinus NC028317 1	0,18296967
Ursus arctos isabellinus 18B5 2	Canis adustus KT448271 1	0,18296985
Zalophus californianus NC008416 1	Lutrogale perspicillata NC035811 1	0,1829721
Leopardus pardalis T262 1	Canis mesomelas KT448280 1	0,1829744
Ursus thibetanus thibetanus NC01111	Poecilogale albinucha T602 1	0,18297587
Panthera uncia NC010638 1	Canis adustus KT448271 1	0,18297675
Lynx lynx NC027083 4	Lycalopex sechurae KT448284 1	0,18297765
Leopardus tigrinus NC028317 1	Canis lupus chanco NC010340 4	0,18297786
Canis adustus KT448271 1	Arctocephalus pusillus NC008417 1	0,18298182
Zalophus californianus NC008416 1	Catopuma badia NC028300 1	0,18298271
Zalophus californianus NC008416 1	Otocolobus manul NC028323 1	0,18298309
Phoca groenlandica NC008429 54	Cuon alpinus NC013445 3	0,18299051
Ursus americanus JX196366 3	Melogale moschata NC020644 1	0,18299167
Panthera pardus japonensis KJ866876	Ommatophoca rossii AY377287etc 1	0,18299194
Leopardus guigna NC028321 1	Gulo gulo NC009685 3	0,1829924
Crossarchus platycephalus C7R66 1	Tapirus terrestris T358	0,1829949
Phocarctos hookeri NC008418 1	Homotherium latidens MF871702 3	0,18299563
Chrotogale owstoni T607 1	Arctocephalus australis MG023139 1	0,18299593
Lutra lutra NC011358 9	Crossarchus platycephalus C7R66 1	0,18299697
Otocyon megalotis SAF1 2	Halichoerus grypus NC001602 2	0,18299834
Ursus spelaeus EU327344 13	Genetta abyssinica MG489822 1	0,18299887
Enhydra lutris NC009692 1	Arctocephalus forsteri KT693377 17	0,1829994
Mustela sibirica NC020637 6	Arctocephalus gazella BK010918 1	0,18300338
Martes pennanti NC020664 16	Conepatus chinga NC042596 1	0,18300672
Smilodon populator MF871700 1	Canis mesomelas KT448280 1	0,183007
Nasua nasua NC020647 1	Mirounga leonina NC008422 1	0,18300702
Mungos mungo/gambianus SRR77048	Canis latrans NC008093 7	0,18300703
Nyctereutes procyonoides NC013700 3	Hemigalus derbyanus MH464791 1	0,1830073
Viverra tangalunga MH464792 1	Tremarctos ornatus NC009969 2	0,18300962
Prionailurus planiceps NC028312 6	Lutra lutra NC011358 9	0,18301228
Felis nigripes NC028309 1	Arctonyx collaris NC020645 1	0,1830142
Ursus maritimus GU573488 Svalbard	Martes melampus NC009678 1	0,18301529
Urocyon littoralis catalinae KP129018	Phoca groenlandica NC008429 54	0,18301596
Ursus arctos isabellinus 18B5 2	Enhydra lutris NC009692 1	0,18301733
Mustela nigripes NC024942 1	Felis nigripes NC028309 1	0,18301758
Profelis aurata NC028299 1	Mustela kathiah NC023210 1	0,18301778
Mustela itatsi NC034330 19	Arctotherium sp NC030174 1	0,18301795
Puma concolor NC016470 22	Melogale moschata KP726273 1	0,18301825
Prionailurus planiceps NC028312 6	Arctocephalus forsteri KT693377 17	0,18301839
Lutrogale perspicillata NC035811 1	Arctodus simus NC011116 1	0,18301864
Vulpes lagopus NC026529 3	Phoca largha NC008430 1	0,18301888
Urocyon cinereoargenteus NC026723 2	Phoca largha NC008430 1	0,18301944
Vulpes zerda KJ603240 1	Prionailurus rubiginosus NC028304 2	0,18301994
Vulpes vulpes NC008434 5	Ommatophoca rossii AY377287etc 1	0,18302131
Ursus maritimus NC003428 31	Mustela frenata NC020640 1	0,18302153
Ursus maritimus GU573488 Svalbard	Mustela frenata NC020640 1	0,18302155
Vulpes lagopus NC026529 3	Catopuma badia NC028300 1	0,18302198
Hemigalus derbyanus MH464791 1	Ailuropoda melanoleuca NC009492 5	0,1830222
Zalophus wollebaeki SRR4431565 1	Mungos mungo/gambianus SRR77048	0,18302356
Procyon lotor AB462049 4	Felis margarita NC028308 1	0,18302415
Smilodon populator MF871700 1	Ictonyx striatus T299 1	0,18302432
Neovison vison NC020641 3	Cynogale bennetti KY117544 1	0,18302451
Catopuma badia NC028300 1	Arctocephalus pusillus NC008417 1	0,18302488
Viverra zibetha T609 1	Ursus arctos GU573491 207	0,18302499
Martes pennanti NC020664 16	Leopardus pardalis NC028315 1	0,18302543
Pardofelis marmorata NLN3 2	Canis aureus KT448274 1	0,18302554
Melogale moschata NC020644 1	Felis catus NC001700 2	0,18302605
Prionailurus rubiginosus NC028304 2	Arctotherium sp NC030174 1	0,18302669
Otocyon megalotis SAF1 2	Leopardus wiedii NC028318 1	0,18302777
Ursus arctos GU573486 5	Ailurus fulgens NC011124 1	0,18302785
Speothos venaticus C48 2	Pusa hispida NC 008433 1	0,18302818
Smilodon populator MF871700 1	Meles meles T303 3	0,18302833
Viverra zibetha T609 1	Lycalopex sechurae KT448284 1	0,18303017
Mungos mungo MMC7 1	Canis aureus KT448274 1	0,1830323
Viverricula indica KX891751 1	Ictonyx striatus T299 1	0,18303299
Ursus arctos AP012576 6	Ailurus fulgens NC011124 1	0,18303579
Vulpes vulpes NC008434 5	Leopardus colocolo NC028314 1	0,18303721
Paradoxurus hermaphroditus NC03959	Chrysocyon brachyurus NC024172 1	0,18303734
Lycalopex sechurae KT448284 1	Arctictis binturong T605 2	0,18303968
Eumetopias jubatus NC004030 10	Chrotogale owstoni T607 1	0,18303972
Ursus spelaeus NC011112 8	Mustela altaica NC021751 1	0,1830404
Martes pennanti NC020664 16	Acinonyx jubatus NC005212 3	0,18304209
Puma concolor NC016470 22	Lycalopex sechurae KT448284 1	0,18304281
Ursus spelaeus NC011112 8	Leopardus pardalis NC028315 1	0,183043
Vulpes vulpes NC008434 5	Genetta abyssinica MG489822 1	0,18304594
Zalophus californianus NC008416 1	Prionailurus viverrinus NC028305 1	0,18304637

Procyon lotor AB462049 4	Bassariscus sumichrasti SRX1099089 1	0,11279359
Prionailurus bengalensis CKM45 20	Neofelis nebulosa NC008450 3	0,11280221
Panthera pardus NC010641 5	Catopuma temminckii NC027115 41	0,11280426
Puma yagouaroundi NC028311 1	Panthera leo spelaea KX258452 2	0,11282917
Panthera pardus japonensis KJ866876 8	Felis chaus NC028307 1	0,11284675
Puma yagouaroundi NC028311 1	Panthera pardus japonensis KJ866876 8	0,11284675
Panthera onca KP202264 2	Leopardus guigna NC028321 1	0,11284865
Panthera tigris amoyensis NC014770 2	Felis chaus NC028307 1	0,11284973
Profelis aurata NC028299 1	Panthera leo NERO 19	0,11287371
Pardofelis marmorata NLN3 2	Panthera leo NERO 19	0,11287486
Panthera pardus japonensis KJ866876 8	Catopuma badia NC028300 1	0,11291409
Prionailurus viverrinus NC028305 1	Panthera tigris amoyensis NC014770 2	0,11291893
Panthera uncia KP202269 1	Leptailurus serval NC028316 1	0,11298142
Panthera uncia NC010638 1	Caracal caracal NC028306 1	0,11298142
Panthera uncia KP202269 1	Lynx lynx NC027083 4	0,11304875
Suricata suricatta SSM10 1	Helogale parvula SRR7637809 1	0,11304875
Panthera tigris amoyensis NC014770 2	Lynx canadensis NC028313 1	0,11305147
Panthera tigris amoyensis NC014770 2	Felis silvestris lybica KP202275 4	0,11305166
Prionailurus bengalensis NC028301 12	Neofelis nebulosa NC008450 3	0,11306931
Neofelis nebulosa NC008450 3	Leopardus pardalis T262 1	0,11306951
Panthera uncia KP202269 1	Catopuma temminckii NC027115 41	0,11311608
Puma yagouaroundi NC028311 1	Panthera tigris amoyensis NC014770 2	0,11312006
Panthera tigris amoyensis NC014770 2	Leopardus tigrinus NC028317 1	0,11312076
Panthera leo NERO 19	Felis chaus NC028307 1	0,11314273
Panthera pardus japonensis KJ866876 8	Leopardus guigna NC028321 1	0,11318552
Panthera tigris NC010642 35	Leopardus jacobita NC028322 1	0,11318865
Neofelis nebulosa NC008450 3	Felis silvestris lybica KP202275 4	0,11320306
Prionailurus rubiginosus NC028304 2	Neofelis nebulosa NC008450 3	0,11327135
Viverra tangalunga MH464792 1	Mustela nivalis T306 5	0,11327769
Panthera tigris amoyensis NC014770 2	Lynx rufus NC014456 3	0,11332152
Pusa hispida NC 008433 1	Mirounga leonina NC008422 1	0,11332264
Phoca fasciata NC008428 1	Mirounga angustirostris SRR10331586 1	0,11333221
Panthera leo spelaea KX258452 2	Catopuma badia NC028300 1	0,11336846
Panthera leo spelaea KX258452 2	Leopardus guigna NC028321 1	0,11336875
Puma yagouaroundi NC028311 1	Panthera uncia KP202269 1	0,1133854
Prionailurus viverrinus NC028305 1	Panthera uncia KP202269 1	0,11338738
Viverra tangalunga MH464792 1	Mustela sibirica NC020637 6	0,11340692
Puma concolor NC016470 22	Panthera uncia NC010638 1	0,11345273
Mungos mungo/gambianus SRR7704821 1	Bdeogale nigripes GLC15 1	0,11352006
Panthera uncia NC010638 1	Catopuma badia NC028300 1	0,11352006
Panthera uncia NC010638 1	Felis chaus NC028307 1	0,11352006
Mungos mungo MMC7 1	Bdeogale nigripes GLC15 1	0,1135216
Phoca groenlandica NC008429 54	Mirounga leonina NC008422 1	0,11352498
Prionailurus viverrinus NC028305 1	Panthera tigris NC010642 35	0,11352515
Viverra tangalunga MH464792 1	Mustela eversmannii NC028013 1	0,11354153
Viverra tangalunga MH464792 1	Mustela sibirica AP017394 11	0,11354153
Viverra tangalunga MH464792 1	Mustela altaica NC021751 1	0,11354671
Panthera onca KP202264 2	Otocolobus manul NC028323 1	0,11358739
Prionailurus planiceps KY682741 4	Panthera tigris amoyensis NC014770 2	0,11359039
Panthera tigris NC010642 35	Leopardus tigrinus NC028317 1	0,11359233
Monachus schauinslandi NC008421 1	Erignathus barbatus NC008426 1	0,11366242
Speothos venaticus C48 2	Cuon alpinus NC013445 3	0,113674
Panthera onca KP202264 2	Felis nigripes NC028309 1	0,11372206
Neofelis nebulosa NC008450 3	Leopardus pardalis NC028315 1	0,11374263
Panthera tigris NC010642 35	Catopuma badia NC028300 1	0,11379293
Neofelis nebulosa NC008450 3	Leptailurus serval NC028316 1	0,11381019
Panthera uncia KP202269 1	Otocolobus manul NC028323 1	0,11385672
Prionailurus bengalensis NC028301 12	Panthera uncia NC010638 1	0,11385672
Profelis aurata NC028299 1	Panthera uncia NC010638 1	0,11385672
Panthera onca KP202264 2	Leopardus jacobita NC028322 1	0,11385837
Panthera tigris NC010642 35	Leptailurus serval NC028316 1	0,11385994
Panthera pardus japonensis KJ866876 8	Catopuma temminckii NC027115 41	0,11392405
Panthera uncia KP202269 1	Leopardus tigrinus NC028317 1	0,11399291
Pardofelis marmorata NLN3 2	Panthera leo spelaea KX258452 2	0,11403951
Urva semitorquata MH464789 1	Helogale parvula SRR7637809 1	0,11405871
Panthera uncia NC010638 1	Leopardus wiedii NC028318 1	0,11406003
Phoca fasciata NC008428 1	Monachus schauinslandi NC008421 1	0,11407153
Panthera leo spelaea KX258452 2	Felis chaus NC028307 1	0,11410853
Profelis aurata NC028299 1	Panthera tigris amoyensis NC014770 2	0,11412909
Panthera tigris NC010642 35	Felis chaus NC028307 1	0,11412919
Panthera uncia NC010638 1	Leopardus colocolo NC028314 1	0,11413946
Neofelis nebulosa NC008450 3	Felis nigripes NC028309 1	0,1141459
Panthera uncia KP202269 1	Lynx canadensis NC028313 1	0,11419337
Suricata suricatta SSM10 1	Urva semitorquata MH464789 1	0,11419337
Panthera tigris NC010642 35	Catopuma temminckii NC027115 41	0,1141964
Pardofelis marmorata NLN3 2	Panthera pardus NC010641 5	0,11421785
Panthera uncia NC010638 1	Felis catus NC001700 2	0,1142607
Pardofelis marmorata NLN3 2	Panthera pardus japonensis KJ866876 8	0,1142607
Panthera tigris NC010642 35	Lynx canadensis NC028313 1	0,11426342
Profelis aurata NC028299 1	Panthera tigris NC010642 35	0,11426375
Prionailurus planiceps KY682741 4	Neofelis nebulosa NC008450 3	0,11428094
Panthera leo NERO 19	Acinonyx jubatus NC005212 3	0,11429977
Panthera tigris NC010642 35	Acinonyx jubatus NC005212 3	0,1143425
Panthera onca NC022842 1	Acinonyx jubatus NC005212 3	0,11434437
Panthera leo NERO 19	Felis nigripes NC028309 1	0,11435611
Panthera uncia NC010638 1	Leopardus geoffroyi NC028320 1	0,11439718
Panthera tigris amoyensis NC014770 2	Caracal caracal NC028306 1	0,11439892
Panthera tigris NC010642 35	Lynx rufus NC014456 3	0,11439908
Profelis aurata NC028299 1	Panthera leo spelaea KX258452 2	0,11444615
Prionailurus bengalensis CKM45 20	Panthera uncia NC010638 1	0,11446535
Suricata suricatta SSM10 1	Cynictis penicillata T375 1	0,11453003
Panthera uncia KP202269 1	Lynx pardinus NC028319 161	0,11459736
Pusa sibirica NC008432 2	Mirounga leonina NC008422 1	0,11459736
Helogale parvula SRR7637809 1	Galerella sanguinea T378 1	0,11460569
Panthera tigris amoyensis NC014770 2	Felis catus NC001700 2	0,11466763
Panthera tigris amoyensis NC014770 2	Acinonyx jubatus NC005212 3	0,11467905
Prionailurus planiceps NC028312 6	Panthera tigris amoyensis NC014770 2	0,11473505
Enhydra lutris NC009692 1	Aonyx cinerea NC035814 2	0,11474527
Prionailurus viverrinus NC028305 1	Neofelis nebulosa NC008450 3	0,11475382
Martes pennanti NC020664 16	Martes foina NC020643 1	0,11475861
Panthera tigris amoyensis NC014770 2	Catopuma temminckii NC027115 41	0,11480219
Puma yagouaroundi NC028311 1	Panthera tigris NC010642 35	0,11480318
Neofelis nebulosa NC008450 3	Leopardus wiedii NC028318 1	0,1148205

Zalophus californianus NC008416 1	Genetta servalina NC024568 2	0,18304656
Ursus thibetanus mupinensis NC00875	Arctocephalus forsteri NC004023 28	0,1830474
Zalophus californianus NC008416 1	Prionailurus rubiginosus NC028304 2	0,18304974
Ursus americanus JX196366 3	Martes zibellina NC011579 39	0,18304986
Melursus ursinus NC009970 2	Homotherium latidens MF871702 3	0,18305728
Prionailurus viverrinus NC028305 1	Gulo gulo NC009685 3	0,18306012
Paradoxurus jerdoni MH464793 1	Ommatophoca rossii AY377287etc 1	0,18306021
Mustela erminea T305 2	Cuon alpinus NC013445 3	0,18306324
Otocolobus manul NC028323 1	Eumetopias jubatus NC004030 10	0,18306413
Phoca fasciata NC008428 1	Cynogale bennetti KY117544 1	0,18306819
Lutra lutra LC050126 1	Eupleres goudotii D128 1	0,18306939
Martes americana NC020642 1	Fossa fossana D350 1	0,1830698
Mustela itatsi NC034330 19	Fossa fossana D350 1	0,18307105
Neophoca cinerea NC008419 1	Melogale moschata V0735A 1	0,18307182
Vulpes ferrilata NC027935 1	Pusa sibirica NC008432 2	0,18307284
Vulpes zerda KJ603240 1	Pusa caspica NC008431 1	0,18307428
Lycaon pictus NC028427 2	Homotherium latidens MF871702 3	0,18307607
Martes melampus NC009678 1	Galerella sanguinea T378 1	0,18307671
Vulpes corsac NC023958 1	Salanoia concolor D378 1	0,18307701
Prionodon linsang ERR2391707 1	Mustela erminea T305 2	0,18307723
Tremarctos ornatus NC009969 2	Aonyx cinerea NC035814 2	0,18307737
Smilodon populator MF871700 1	Mustela itatsi NC034330 19	0,18307841
Viverra tangalunga MH464792 1	Canis latrans NC008093 7	0,18307891
Lutra lutra NC011358 9	Felis silvestris lybica KP202275 4	0,18307985
Ursus maritimus NC003428 31	Taxidea taxus NC020646 1	0,18308035
Mustela nivalis T306 5	Chrotogale owstoni T607 1	0,1830806
Meles anakuma NC009677 1	Felis catus NC001700 2	0,1830814
Profelis aurata NC028299 1	Meles meles T303 3	0,18308225
Lynx canadensis NC028313 1	Lutra sumatrana NC035810 1	0,18308318
Mustela sibirica NC020637 6	Leopardus tigrinus NC028317 1	0,18308425
Prionailurus planiceps KY682741 4	Arctocephalus australis MG023139 1	0,18308547
Prionailurus planiceps KY682741 4	Arctocephalus forsteri KT693377 17	0,18308549
Martes zibellina NC011579 39	Catopuma badia NC028300 1	0,1830865
Melogale moschata KP726273 1	Felis chaus NC028307 1	0,1830866
Vulpes lagopus NC026529 3	Prionailurus rubiginosus NC028304 2	0,18308662
Vulpes ferrilata NC027935 1	Prionailurus rubiginosus NC028304 2	0,18308678
Prionailurus viverrinus NC028305 1	Melogale moschata KP726273 1	0,18308735
Martes zibellina NC011579 39	Leptailurus serval NC028316 1	0,18308806
Neophoca cinerea NC008419 1	Lynx pardinus NC028319 161	0,18308832
Viverra zibetha T609 1	Procyon lotor AB462049 4	0,18308896
Poecilogale albinucha T602 1	Paguma larvata PDDS11 2	0,18309063
Ursus arctos pruinosus MG066703 3	Mustela eversmannii NC028013 1	0,18309088
Tremarctos ornatus NC009969 2	Genetta abyssinica MG489822 1	0,18309177
Viverra zibetha T609 1	Poecilogale albinucha T602 1	0,18309203
Felis margarita NC028308 1	Aonyx cinerea NC035814 2	0,18309264
Ursus maritimus GU573488 Svalbard	Genetta servalina NC024568 2	0,18309316
Leopardus geoffroyi NC028320 1	Aonyx cinerea NC035814 2	0,18309353
Procyon lotor AB462046 3	Prionailurus viverrinus NC028305 1	0,18309355
Zalophus wollebaeki SRR4431565 1	Ursus arctos EU497665 29	0,18309484
Ursus thibetanus laniger MH281753 2	Melogale moschata V0735A 1	0,18309535
Mustela eversmannii NC028013 1	Genetta abyssinica MG489822 1	0,18309782
Leopardus tigrinus NC028317 1	Ictonyx striatus T299 1	0,18309994
Ursus spelaeus EU327344 13	Arctocephalus townsendi NC008420 1	0,18310066
Ursus spelaeus NC011112 8	Arctocephalus australis MG023139 1	0,18310113
Lycaon pictus NC028427 2	Arctodus simus NC011116 1	0,18310165
Viverra tangalunga MH464792 1	Leopardus colocolo NC028314 1	0,18310316
Melogale moschata V0735A 1	Arctocephalus forsteri NC004023 28	0,18310584
Mungos mungo MMC7 1	Canis lupus familiaris NC002008 1231	0,18310601
Paradoxurus jerdoni MH464793 1	Ailuropoda melanoleuca NC009492 5	0,18310736
Canis aureus KT448274 1	Arctodus simus NC011116 1	0,18310744
Poecilogale albinucha T602 1	Genetta abyssinica MG489822 1	0,18310821
Ursus spelaeus EU327344 13	Mustela nivalis T306 5	0,18310969
Leopardus tigrinus NC028317 1	Chrysocyon brachyurus NC024172 1	0,18311064
Prionailurus planiceps KY682741 4	Chrysocyon brachyurus NC024172 1	0,18311153
Vulpes zerda KJ603240 1	Monachus monachus NC044972 5	0,18311438
Zalophus californianus NC008416 1	Lynx canadensis NC028313 1	0,18311453
Melursus ursinus NC009970 2	Arctocephalus forsteri NC004023 28	0,18311928
Prionodon linsang ERR2391707 1	Canis lupus chanco NC010340 4	0,18311946
Panthera leo NERO 19	Mustela erminea T305 2	0,18311988
Gulo gulo NC009685 3	Genetta servalina NC024568 2	0,18312028
Viverra zibetha T609 1	Ursus americanus JX196366 3	0,18312599
Martes flavigula NC012141 3	Galerella sanguinea T378 1	0,18312626
Martes flavigula NC012141 3	Felis nigripes NC028309 1	0,1831301
Paradoxurus hermaphroditus NLNC 1	Cuon alpinus NC013445 3	0,18313068
Civettictis civetta NC033378 1	Arctocephalus pusillus NC008417 1	0,18313271
Nasua nasua NC020647 1	Aonyx cinerea NC035814 2	0,18313684
Melogale moschata KP726273 1	Urva semitorquata MH464789 1	0,18313755
Mustela sibirica AP017394 11	Urva javanica T413 1	0,18313988
Ursus maritimus NC003428 31	Martes flavigula NC012141 3	0,18314086
Mungos mungo/gambianus SRR77048	Arctocephalus gazella BK010918 1	0,18314137
Nyctereutes procyonoides NC013700 3	Fossa fossana D350 1	0,18314138
Vulpes zerda KJ603240 1	Nandinia binotata NC024567 1	0,18314311
Meles meles T303 3	Canis anthus NC027956 2	0,18314321
Martes melampus NC009678 1	Genetta servalina NC024568 2	0,18314458
Mustela putorius NC020638 4	Canis anthus NC027956 2	0,18314573
Pardofelis marmorata NLN3 2	Enhydra lutris NC009692 1	0,18314801
Lutra lutra LC050126 1	Leopardus tigrinus NC028317 1	0,18314849
Meles meles T303 3	Genetta genetta T297 1	0,18315075
Ursus thibetanus mupinensis NC00875	Arctocephalus australis MG023139 1	0,18315142
Meles anakuma NC009677 1	Catopuma temminckii NC027115 41	0,1831516
Otocolobus manul NC028323 1	Lutra sumatrana NC035810 1	0,18315205
Mustela eversmannii NC028013 1	Leptailurus serval NC028316 1	0,1831529
Leopardus wiedii NC028318 1	Arctocephalus australis MG023139 1	0,18315481
Martes americana NC020642 1	Caracal caracal NC028306 1	0,18315499
Martes americana NC020642 1	Leopardus geoffroyi NC028320 1	0,18315527
Viverricula indica KX891745 1	Procyon lotor AB462049 4	0,18315598
Ursus maritimus NC003428 31	Ailurus fulgens NC011124 1	0,1831561
Martes martes T302 3	Ichneumia albicauda T603 1	0,18315691
Caracal caracal NC028306 1	Canis latrans NC008093 7	0,1831571
Procyon lotor AB462049 4	Lynx canadensis NC028313 1	0,18315735
Vulpes zerda KJ603240 1	Prionailurus bengalensis CKM45 20	0,18315792
Tremarctos ornatus NC009969 2	Arctocephalus forsteri NC004023 28	0,18315816
Poecilogale albinucha T602 1	Lynx lynx NC027083 4	0,18315905

Panthera onca KP202264 2	Catopuma temminckii NC027115 41	0,11486668
Panthera tigris NC010642 35	Leopardus guigna NC028321 1	0,11493972
Panthera onca KP202264 2	Acinonyx jubatus NC005212 3	0,11494492
Neofelis nebulosa NC008450 3	Lynx lynx NC027083 4	0,11495237
Neofelis nebulosa NC008450 3	Lynx canadensis NC028313 1	0,11495264
Neofelis nebulosa NC008450 3	Felis catus NC001700 2	0,11495375
Pusa caspica NC008431 1	Mirounga leonina NC008422 1	0,11500134
Panthera tigris amoyensis NC014770 2	Otocolobus manul NC028323 1	0,11500478
Panthera uncia NC010638 1	Leopardus guigna NC028321 1	0,11507058
Ichneumia albicauda T603 1	Helogale parvula SRR7637809 1	0,11507766
Speothos venaticus C48 2	Lycalopex sechurae KT448284 1	0,11507893
Pusa hispida NC 008433 1	Mirounga angustirostris SRR10331586 1	0,11508188
Panthera pardus NC010641 5	Otocolobus manul NC028323 1	0,11509279
Panthera leo spelaea KX258452 2	Felis nigripes NC028309 1	0,11511973
Panthera uncia NC010638 1	Felis margarita NC028308 1	0,115136
Prionailurus planiceps KY682741 4	Panthera uncia NC010638 1	0,115136
Pusa sibirica NC008432 2	Mirounga angustirostris SRR10331586 1	0,11527928
Neofelis nebulosa NC008450 3	Felis margarita NC028308 1	0,11529092
Viverra tangalunga MH464792 1	Mustela frenata NC020640 1	0,11529142
Mungos mungo MMC7 1	Urva javanica/auropunctata NC006835 1	0,11530624
Panthera leo spelaea KX258452 2	Catopuma temminckii NC027115 41	0,11532141
Phoca largha NC008430 1	Mirounga leonina NC008422 1	0,11534639
Urva javanica/auropunctata NC006835 1	Helogale parvula SRR7637809 1	0,11537123
Martes pennanti NC020664 16	Martes flavigula NC012141 3	0,11539831
Panthera uncia NC010638 1	Felis nigripes NC028309 1	0,11540534
Prionailurus rubiginosus NC028304 2	Panthera uncia NC010638 1	0,11540534
Suricata suricatta SSM10 1	Bdeogale nigripes GLC15 1	0,11540534
Mungos mungo MMC7 1	Urva javanica T413 1	0,11540655
Panthera tigris amoyensis NC014770 2	Leopardus guigna NC028321 1	0,11541098
Panthera pardus japonensis KJ866876 8	Acinonyx jubatus NC005212 3	0,11541616
Panthera pardus japonensis KJ866876 8	Otocolobus manul NC028323 1	0,11547267
Panthera uncia NC010638 1	Leopardus jacobita NC028322 1	0,11547399
Phoca groenlandica NC008429 54	Mirounga angustirostris SRR10331586 1	0,1154874
Mirounga leonina NC008422 1	Halichoerus grypus NC001602 2	0,11550284
Panthera pardus NC010641 5	Acinonyx jubatus NC005212 3	0,11550811
Panthera uncia NC010638 1	Lynx rufus NC014456 3	0,11560733
Pusa hispida NC 008433 1	Monachus schauinslandi NC008421 1	0,11562131
Mungos mungo/gambianus SRR7704821 1	Urva javanica T413 1	0,11567466
Prionailurus planiceps NC028312 6	Panthera uncia NC010638 1	0,11567466
Panthera tigris amoyensis NC014770 2	Felis margarita NC028308 1	0,1156777
Prionailurus planiceps NC028312 6	Neofelis nebulosa NC008450 3	0,11569503
Neofelis nebulosa NC008450 3	Catopuma temminckii NC027115 41	0,11569517
Panthera leo NERO 19	Otocolobus manul NC028323 1	0,11570224
Phoca vitulina NC001325 1	Mirounga leonina NC008422 1	0,11575067
Monachus monachus NC044972 5	Cystophora cristata NC008427 1	0,11577519
Phoca fasciata NC008428 1	Monachus monachus NC044972 5	0,11577892
Panthera tigris NC010642 35	Felis silvestris lybica KP202275 4	0,11581217
Prionailurus planiceps KY682741 4	Panthera tigris NC010642 35	0,11581236
Mungos mungo/gambianus SRR7704821 1	Urva javanica/auropunctata NC006835 1	0,11584351
Mirounga leonina NC008422 1	Cystophora cristata NC008427 1	0,11587665
Panthera uncia NC010638 1	Leptailurus serval NC028316 1	0,11587665
Puma concolor NC016470 22	Neofelis nebulosa NC008450 3	0,1159646
Neofelis nebulosa NC008450 3	Catopuma badia NC028300 1	0,11603248
Neofelis nebulosa NC008450 3	Leopardus geoffroyi NC028320 1	0,11603481
Neofelis nebulosa NC008450 3	Leopardus guigna NC028321 1	0,11603494
Phoca groenlandica NC008429 54	Monachus monachus NC044972 5	0,11604822
Suricata suricatta SSM10 1	Atilax paludinosus T606 1	0,11607864
Panthera tigris NC010642 35	Caracal caracal NC028306 1	0,11608211
Smilodon populator MF871700 1	Prionailurus bengalensis CKM45 20	0,11612315
Prionailurus planiceps NC028312 6	Panthera tigris NC010642 35	0,1161491
Helogale parvula SRR7637809 1	Atilax paludinosus T606 1	0,11621331
Panthera tigris NC010642 35	Otocolobus manul NC028323 1	0,11621692
Phoca groenlandica NC008429 54	Monachus schauinslandi NC008421 1	0,11622678
Pusa caspica NC008431 1	Monachus schauinslandi NC008421 1	0,1162906
Pusa hispida NC 008433 1	Monachus monachus NC044972 5	0,11631893
Panthera tigris amoyensis NC014770 2	Felis nigripes NC028309 1	0,11635078
Suricata suricatta SSM10 1	Ichneumia albicauda T603 1	0,11635794
Prionailurus viverrinus NC028305 1	Panthera uncia NC010638 1	0,11641726
Panthera uncia NC010638 1	Catopuma temminckii NC027115 41	0,11648263
Pardofelis marmorata NLN3 2	Panthera uncia NC010638 1	0,11648263
Puma yagouaroundi NC028311 1	Panthera uncia NC010638 1	0,11648263
Suricata suricatta SSM10 1	Galerella sanguinea T378 1	0,11649118
Pusa sibirica NC008432 2	Monachus schauinslandi NC008421 1	0,11649283
Xenogale naso C07XAR110 1	Helogale parvula SRR7637809 1	0,11654996
Mungos mungo MMC7 1	Urva brachyura KY117547 1	0,11655141
Pardofelis marmorata NLN3 2	Panthera tigris amoyensis NC014770 2	0,11655321
Monachus schauinslandi NC008421 1	Cystophora cristata NC008427 1	0,116559
Helogale parvula SRR7637809 1	Cynictis penicillata T375 1	0,11661729
Suricata suricatta SSM10 1	Xenogale naso C07XAR110 1	0,11661729
Smilodon populator MF871700 1	Leptailurus serval NC028316 1	0,11665657
Panthera leo spelaea KX258452 2	Otocolobus manul NC028323 1	0,11666876
Viverricula indica KX891751 1	Genetta servalina NC024568 2	0,11669248
Pusa caspica NC008431 1	Mirounga angustirostris SRR10331586 1	0,11669339
Phoca fasciata NC008428 1	Ommatophoca rossii AY377287etc 1	0,11682773
Viverra tangalunga MH464792 1	Neovison vison NC020641 3	0,1168437
Phoca largha NC008430 1	Monachus schauinslandi NC008421 1	0,11690599
Pusa sibirica NC008432 2	Monachus monachus NC044972 5	0,11692128
Panthera uncia NC010638 1	Lynx lynx NC027083 4	0,11695395
Panthera tigris NC010642 35	Felis margarita NC028308 1	0,11695696
Smilodon populator MF871700 1	Puma concolor NC016470 22	0,11707385
Pusa hispida NC 008433 1	Ommatophoca rossii AY377287etc 1	0,11708092
Panthera uncia NC010638 1	Leopardus tigrinus NC028317 1	0,1170901
Viverricula indica NC025296 2	Genetta servalina NC024568 2	0,11709905
Panthera uncia KP202269 1	Acinonyx jubatus NC005212 3	0,11709958
Neofelis nebulosa NC008450 3	Lynx pardinus NC028319 161	0,11710787
Pardofelis marmorata NLN3 2	Panthera tigris NC010642 35	0,11722677
Ommatophoca rossii AY377287etc 1	Cystophora cristata NC008427 1	0,11724197
Smilodon populator MF871700 1	Prionailurus bengalensis NC028301 12	0,11726377
Panthera uncia NC010638 1	Otocolobus manul NC028323 1	0,1172906
Panthera tigris NC010642 35	Felis catus NC001700 2	0,11729348
Panthera tigris NC010642 35	Felis nigripes NC028309 1	0,11729363
Smilodon populator MF871700 1	Leopardus wiedii NC028318 1	0,11742043
Neofelis nebulosa NC008450 3	Lynx rufus NC014456 3	0,11744461

Pardofelis marmorata NLN3 2	Mustela nivalis T306 5	0,18316138
Poecilogale albinucha T602 1	Genetta genetta T297 1	0,18316151
Lynx rufus NC014456 3	Lycaon pictus NC028427 2	0,18316559
Zalophus wollebaeki SRR4431565 1	Genetta servalina NC024568 2	0,18316562
Lycaon pictus NC028427 2	Leopardus pardalis NC028315 1	0,18316574
Mustela itatsi NC034330 19	Arctictis binturong T605 2	0,18316637
Ursus thibetanus mupinensis NC00875	Felis margarita NC028308 1	0,18316688
Mustela nivalis T306 5	Canis adustus KT448271 1	0,18316799
Leopardus colocolo NC028314 1	Ailurus fulgens NC011124 1	0,18317017
Viverra tangalunga MH464792 1	Ursus thibetanus thibetanus NC01111	0,18317163
Viverra tangalunga MH464792 1	Ursus spelaeus NC011112 8	0,18317193
Helarctos malayanus NC009968 2	Bassariscus sumichrasti SRX1099089 1	0,18317291
Ursus arctos AP012576 6	Arctocephalus pusillus NC008417 1	0,18317367
Ursus spelaeus NC011112 8	Bassariscus sumichrasti SRX1099089 1	0,18317384
Viverricula indica KX891745 1	Ursus spelaeus EU327344 13	0,18317389
Cynictis penicillata T375 1	Canis lupus familiaris NC002008 1231	0,18317546
Mephitis mephitis NC020648 1	Genetta servalina NC024568 2	0,18317696
Viverra tangalunga MH464792 1	Canis lupus familiaris NC002008 1231	0,1831786
Prionailurus bengalensis NC028301 12	Chrysocyon brachyurus NC024172 1	0,18317957
Ursus thibetanus laniger MH281753 2	Catopuma temminckii NC027115 41	0,18318063
Ursus americanus JX196366 3	Fossa fossana D350 1	0,18318065
Ursus americanus JX196366 3	Martes melampus NC009678 1	0,18318318
Ursus spelaeus EU327344 13	Acinonyx jubatus NC005212 3	0,18318862
Ursus thibetanus thibetanus NC01111	Canis adustus KT448271 1	0,18318906
Viverra tangalunga MH464792 1	Canis lupus chanco NC010340 4	0,18318947
Gulo gulo NC009685 3	Felis margarita NC028308 1	0,1831941
Ursus thibetanus thibetanus NC01111	Canis aureus KT448274 1	0,1831945
Taxidea taxus NC020646 1	Panthera pardus NC010641 5	0,18319871
Mungos mungo MMC7 1	Lutra sumatrana NC035810 1	0,18320246
Smilodon populator MF871700 1	Martes martes T302 3	0,18320335
Meles anakuma NC009677 1	Urva semitorquata MH464789 1	0,18320386
Smilodon populator MF871700 1	Martes melampus NC009678 1	0,18320499
Vulpes ferrilata NC027935 1	Pusa caspica NC008431 1	0,1832076
Mungotictis decemlineata NC027828 1	Lutra sumatrana NC035810 1	0,18320778
Urocyon littoralis catalinae KP129018 1	Pusa sibirica NC008432 2	0,18320845
Fossa fossana D350 1	Arctocephalus gazella BK010918 1	0,18320848
Vulpes zerda KJ603240 1	Pusa sibirica NC008432 2	0,18320869
Homotherium latidens MF871702 3	Cuon alpinus NC013445 3	0,18320926
Cynictis penicillata T375 1	Canis anthus NC027956 2	0,18320951
Viverricula indica KX891745 1	Martes melampus NC009678 1	0,1832104
Taxidea taxus NC020646 1	Bdeogale nigripes GLC15 1	0,18321276
Mustela sibirica NC020637 6	Canis anthus NC027956 2	0,18321294
Meles anakuma NC009677 1	Leopardus pardalis T262 1	0,18321404
Mustela eversmannii NC028013 1	Canis aureus KT448274 1	0,18321511
Paguma larvata PDDS11 2	Melogale moschata KP726273 1	0,18321519
Pardofelis marmorata NLN3 2	Arctonyx collaris NC020645 1	0,18321574
Prionailurus planiceps NC028312 6	Callorhinus ursinus NC008415 1	0,18321714
Ursus maritimus NC003428 31	Martes melampus NC009678 1	0,18321727
Meles anakuma NC009677 1	Genetta genetta T297 1	0,18321794
Leopardus colocolo NC028314 1	Eumetopias jubatus NC004030 10	0,18321848
Mustela itatsi NC034330 19	Felis catus NC001700 2	0,18321946
Viverra tangalunga MH464792 1	Paradoxurus hermaphroditus NC03959	0,18321948
Ursus maritimus GU573488 Svalbard	Arctocephalus gazella BK010918 1	0,18321994
Vulpes corsac NC023958 1	Leopardus pardalis T262 1	0,18322036
Prionailurus planiceps NC028312 6	Arctocephalus australis MG023139 1	0,18322037
Vulpes vulpes NC008434 5	Lynx lynx NC027083 4	0,18322068
Vulpes zerda KJ603240 1	Lynx rufus NC014456 3	0,18322127
Martes americana NC020642 1	Canis aureus KT448274 1	0,18322161
Phocarctos hookeri NC008418 1	Lynx pardinus NC028319 161	0,18322274
Meles meles T303 3	Canis aureus KT448274 1	0,18322466
Procyon lotor AB462049 4	Felis catus NC001700 2	0,18322562
Ursus arctos isabellinus 18B5 2	Mustela itatsi NC034330 19	0,18322614
Procyon lotor AB462046 3	Prionailurus planiceps NC028312 6	0,18322667
Lutra sumatrana NC035810 1	Genetta abyssinica MG489822 1	0,18322757
Proteles cristata T393 6	Mustela erminea T305 2	0,18322876
Prionailurus viverrinus NC028305 1	Martes pennanti NC020664 16	0,1832289
Ursus arctos GU573486 5	Neophoca cinerea NC008419 1	0,1832298
Mustela nivalis T306 5	Catopuma badia NC028300 1	0,1832315
Procyon lotor AB462046 3	Leopardus colocolo NC028314 1	0,18323188
Ursus thibetanus mupinensis NC00875	Lynx rufus NC014456 3	0,18323217
Ursus arctos GU573486 5	Melogale moschata NC020644 1	0,18323296
Halichoerus grypus NC001602 2	Cuon alpinus NC013445 3	0,18323359
Mungos mungo MMC7 1	Eumetopias jubatus NC004030 10	0,18323499
Viverra zibetha T609 1	Canis mesomelas KT448280 1	0,18323824
Lycaon pictus NC028427 2	Caracal caracal NC028306 1	0,18323873
Ursus spelaeus EU327344 13	Genetta servalina NC024568 2	0,18324117
Canis lupus familiaris NC002008 1231	Atilax paludinosus T606 1	0,18324228
Ursus thibetanus formosanus NC0093	Poecilogale albinucha T602 1	0,1832429
Mustela nivalis T306 5	Helarctos malayanus NC009968 2	0,18324366
Ursus thibetanus thibetanus NC01111	Lynx canadensis NC028313 1	0,1832442
Genetta abyssinica MG489822 1	Chrysocyon brachyurus NC024172 1	0,18325031
Zalophus californianus NC008416 1	Prionailurus planiceps KY682741 4	0,1832512
Canis aureus KT448274 1	Arctictis binturong T605 2	0,18325263
Smilodon populator MF871700 1	Poecilogale albinucha T602 1	0,18325293
Lycalopex sechurae KT448284 1	Caracal caracal NC028306 1	0,18325298
Panthera uncia NC010638 1	Monachus monachus NC044972 5	0,18325315
Viverricula indica NC025296 2	Gulo gulo NC009685 3	0,18325543
Spilogale putorius NC010497 1	Martes zibellina NC011579 39	0,18325827
Panthera pardus NC010641 5	Mustela kathiah NC023210 1	0,18325838
Prionailurus planiceps NC028312 6	Canis lupus chanco NC010340 4	0,18326117
Ommatophoca rossii AY377287etc 1	Bassaricyon neblina SRX1097850 1	0,18326406
Viverricula indica KX891751 1	Cuon alpinus NC013445 3	0,18326534
Viverricula indica NC025296 2	Cuon alpinus NC013445 3	0,1832664
Potos flavus T414 1	Chrotogale owstoni T607 1	0,18326731
Mungos mungo/gambianus SRR77048	Enhydra lutris NC009692 1	0,18326814
Viverricula indica NC025296 2	Tapirus terrestris T358	0,18326962
Meles anakuma NC009677 1	Fossa fossana D350 1	0,18326992
Meles leucurus NC039173 4	Urva semitorquata MH464789 1	0,18327121
Prionodon pardicolor NC024569 2	Halichoerus grypus NC001602 2	0,1832722
Prionailurus bengalensis CKM45 20	Tapirus terrestris T358	0,18327302
Salanoia concolor D378 1	Lutra lutra NC011358 9	0,18327309
Mustela nigripes NC024942 1	Mungos mungo MMC7 1	0,1832732
Fossa fossana D350 1	Arctocephalus townsendi NC008420 1	0,18327372

Puma yagouaroundi NC028311 1	Neofelis nebulosa NC008450 3	0,11744683
Hemigalus derbyanus MH464791 1	Cynogale bennetti KY117544 1	0,11748588
Helogale parvula SRR7637809 1	Bdeogale nigripes GLC15 1	0,11749259
Lutrogale perspicillata NC035811 1	Enhydra lutris NC009692 1	0,11757115
Neofelis nebulosa NC008450 3	Leopardus jacobita NC028322 1	0,11758284
Pusa caspica NC008431 1	Monachus monachus NC044972 5	0,11766217
Mirounga angustirostris SRR10331586 1	Cystophora cristata NC008427 1	0,11770387
Profelis aurata NC028299 1	Neofelis nebulosa NC008450 3	0,11778325
Panthera leo spelaea KX258452 2	Acinonyx jubatus NC005212 3	0,11782512
Phoca vitulina NC001325 1	Monachus schauinslandi NC008421 1	0,11784873
Smilodon populator MF871700 1	Profelis aurata NC028299 1	0,11790703
Smilodon populator MF871700 1	Caracal caracal NC028306 1	0,11795842
Phoca vitulina NC001325 1	Mirounga angustirostris SRR10331586 1	0,1180492
Phoca largha NC008430 1	Monachus monachus NC044972 5	0,11807487
Phoca groenlandica NC008429 54	Ommatophoca rossii AY377287etc 1	0,11813055
Smilodon populator MF871700 1	Prionailurus rubiginosus NC028304 2	0,11813725
Mungos mungo/gambianus SRR7704821 1	Urva brachyura KY117547 1	0,1181659
Viverricula indica KX891745 1	Genetta servalina NC024568 2	0,11817386
Phoca largha NC008430 1	Mirounga angustirostris SRR10331586 1	0,11818356
Otocolobus manul NC028323 1	Neofelis nebulosa NC008450 3	0,11818799
Smilodon populator MF871700 1	Leopardus pardalis NC028315 1	0,11826344
Phoca vitulina NC001325 1	Monachus monachus NC044972 5	0,11834411
Paradoxurus hermaphroditus NLNC 1	Hemigalus derbyanus MH464791 1	0,11834554
Panthera uncia NC010638 1	Lynx canadensis NC028313 1	0,11843523
Viverra zibetha T609 1	Genetta servalina NC024568 2	0,11851054
Neofelis nebulosa NC008450 3	Leopardus tigrinus NC028317 1	0,11852452
Monachus monachus NC044972 5	Halichoerus grypus NC001602 2	0,1186348
Panthera uncia NC010638 1	Lynx pardinus NC028319 161	0,11870455
Phoca vitulina NC001325 1	Ommatophoca rossii AY377287etc 1	0,11870546
Smilodon populator MF871700 1	Leopardus geoffroyi NC028320 1	0,11876641
Genetta servalina NC024568 2	Civettictis civetta GLC19 1	0,11877988
Mirounga angustirostris SRR10331586 1	Halichoerus grypus NC001602 2	0,11881244
Smilodon populator MF871700 1	Leopardus colocolo NC028314 1	0,11885156
Neofelis nebulosa NC008450 3	Caracal caracal NC028306 1	0,11886086
Paradoxurus jerdoni MH464793 1	Hemigalus derbyanus MH464791 1	0,1188942
Urva brachyura KY117547 1	Helogale parvula SRR7637809 1	0,11890654
Phoca largha NC008430 1	Ommatophoca rossii AY377287etc 1	0,11893807
Suricata suricatta SSM10 1	Urva javanica/auropunctata NC006835 1	0,11894092
Pusa caspica NC008431 1	Ommatophoca rossii AY377287etc 1	0,11906636
Urva javanica T413 1	Helogale parvula SRR7637809 1	0,11910854
Neofelis nebulosa NC008450 3	Leopardus colocolo NC028314 1	0,11914361
Monachus schauinslandi NC008421 1	Halichoerus grypus NC001602 2	0,11915152
Pardofelis marmorata NLN3 2	Neofelis nebulosa NC008450 3	0,11919705
Smilodon populator MF871700 1	Felis nigripes NC028309 1	0,11925392
Smilodon populator MF871700 1	Leopardus pardalis T262 1	0,11945143
Smilodon populator MF871700 1	Felis chaus NC028307 1	0,11948252
Genetta servalina NC024568 2	Civettictis civetta NC033378 1	0,1195465
Viverra zibetha T609 1	Genetta abyssinica MG489822 1	0,11958573
Pusa sibirica NC008432 2	Ommatophoca rossii AY377287etc 1	0,11959648
Paradoxurus hermaphroditus NC039591 1	Hemigalus derbyanus MH464791 1	0,11960472
Mustela erminea T305 2	Lutra lutra LC050126 1	0,11972979
Smilodon populator MF871700 1	Otocolobus manul NC028323 1	0,11973116
Smilodon populator MF871700 1	Prionailurus viverrinus NC028305 1	0,11978362
Viverricula indica NC025296 2	Genetta abyssinica MG489822 1	0,11979228
Ommatophoca rossii AY377287etc 1	Halichoerus grypus NC001602 2	0,11984851
Viverricula indica KX891751 1	Paradoxurus hermaphroditus NLNC 1	0,11985725
Smilodon populator MF871700 1	Felis margarita NC028308 1	0,1199811
Mustela erminea T305 2	Lutra sumatrana NC035810 1	0,11999912
Viverricula indica KX891745 1	Genetta abyssinica MG489822 1	0,120056
Smilodon populator MF871700 1	Acinonyx jubatus NC005212 3	0,12014836
Smilodon populator MF871700 1	Felis silvestris lybica KP202275 4	0,12018841
Galidictis fasciata DM333 1	Fossa fossana D350 1	0,12025534
Smilodon populator MF871700 1	Catopuma badia NC028300 1	0,12027574
Martes pennanti NC020664 16	Gulo gulo NC009685 3	0,12037399
Smilodon populator MF871700 1	Lynx pardinus NC028319 161	0,12039947
Suricata suricatta SSM10 1	Urva javanica T413 1	0,12045516
Smilodon populator MF871700 1	Leopardus jacobita NC028322 1	0,12047716
Smilodon populator MF871700 1	Leopardus tigrinus NC028317 1	0,12053693
Smilodon populator MF871700 1	Catopuma temminckii NC027115 41	0,12062305
Smilodon populator MF871700 1	Homotherium latidens MF871702 3	0,12064341
Cynogale bennetti KY117544 1	Chrotogale owstoni T607 1	0,12075836
Viverricula indica KX891751 1	Genetta abyssinica MG489822 1	0,12080223
Smilodon populator MF871700 1	Puma yagouaroundi NC028311 1	0,12099345
Smilodon populator MF871700 1	Leopardus guigna NC028321 1	0,12099367
Paguma larvata PDDS11 2	Genetta servalina NC024568 2	0,12100195
Prionailurus bengalensis CKM45 20	Homotherium latidens MF871702 3	0,12120473
Panthera uncia NC010638 1	Acinonyx jubatus NC005212 3	0,1212071
Mustela erminea T305 2	Enhydra lutris NC009692 1	0,12121085
Hemigalus derbyanus MH464791 1	Arctictis binturong T605 2	0,12128204
Smilodon populator MF871700 1	Felis catus NC001700 2	0,12128611
Smilodon populator MF871700 1	Prionailurus planiceps KY682741 4	0,12139397
Viverricula indica KX891745 1	Paradoxurus hermaphroditus NLNC 1	0,12140597
Genetta abyssinica MG489822 1	Civettictis civetta GLC19 1	0,1214995
Suricata suricatta SSM10 1	Urva brachyura KY117547 1	0,12166712
Smilodon populator MF871700 1	Lynx canadensis NC028313 1	0,1217572
Smilodon populator MF871700 1	Prionailurus planiceps NC028312 6	0,12176458
Smilodon populator MF871700 1	Lynx lynx NC027083 4	0,12180305
Mustela erminea T305 2	Martes melampus NC009678 1	0,12188325
Mustela erminea T305 2	Lutra lutra NC011358 9	0,12188339
Mustela erminea T305 2	Martes zibellina NC011579 39	0,12189041
Salanoia concolor D378 1	Fossa fossana D350 1	0,12207311
Paguma larvata PDDS11 2	Hemigalus derbyanus MH464791 1	0,12209646
Viverricula indica NC025296 2	Paradoxurus hermaphroditus NLNC 1	0,1223493
Viverricula indica NC025296 2	Hemigalus derbyanus MH464791 1	0,1223601
Viverra zibetha T609 1	Paguma larvata PDDS11 2	0,122416
Mustela erminea T305 2	Martes martes T302 3	0,12256348
Viverra zibetha T609 1	Hemigalus derbyanus MH464791 1	0,12260202
Viverricula indica KX891751 1	Genetta genetta T297 1	0,12261823
Genetta abyssinica MG489822 1	Civettictis civetta NC033378 1	0,12262067
Diplogale hosei MH464790 1	Cynogale bennetti KY117544 1	0,12265089
Viverricula indica KX891751 1	Paradoxurus jerdoni MH464793 1	0,12275267
Smilodon populator MF871700 1	Lynx rufus NC014456 3	0,12286318
Smilodon populator MF871700 1	Pardofelis marmorata NLN3 2	0,12296986

Neophoca cinerea NC008419 1	Mustela kathiah NC023210 1	0,18327531
Salanoia concolor D378 1	Enhydra lutris NC009692 1	0,18327598
Vulpes ferrilata NC027935 1	Mirounga leonina NC008422 1	0,18327691
Vulpes vulpes NC008434 5	Mustela frenata NC020640 1	0,1832771
Urocyon cinereoargenteus NC026723 2	Mirounga leonina NC008422 1	0,18327774
Martes foina NC020643 1	Canis adustus KT448271 1	0,18327782
Tremarctos ornatus NC009969 2	Mustela nigripes NC024942 1	0,18327878
Tremarctos ornatus NC009969 2	Mustela itatsi NC034330 19	0,1832791
Gulo gulo NC009685 3	Canis adustus KT448271 1	0,18327919
Tremarctos ornatus NC009969 2	Procyon lotor AB462049 4	0,18327935
Ommatophoca rossii AY377287etc 1	Cynictis penicillata T375 1	0,18328106
Galerella sanguinea T378 1	Arctonyx collaris NC020645 1	0,18328203
Leopardus tigrinus NC028317 1	Ailurus fulgens styani NC009691 1	0,18328214
Proteles cristata T393 6	Halichoerus grypus NC001602 2	0,1832841
Mustela nivalis T306 5	Conepatus chinga NC042596 1	0,18328455
Vulpes zerda KJ603240 1	Phoca fasciata NC008428 1	0,18328506
Lycaon pictus NC028427 2	Crossarchus platycephalus C7R66 1	0,18328583
Mustela altaica NC021751 1	Canis latrans NC008093 7	0,18328595
Mustela itatsi NC034330 19	Civettictis civetta GLC19 1	0,18328699
Mustela sibirica AP017394 11	Caracal caracal NC028306 1	0,18328726
Phoca groenlandica NC008429 54	Galidia elegans D146 1	0,18328755
Melogale moschata KP726273 1	Felis catus NC001700 2	0,1832877
Martes martes T302 3	Galerella sanguinea T378 1	0,18328826
Leopardus wiedii NC028318 1	Arctocephalus forsteri KT693377 17	0,18328932
Ursus arctos EU497665 29	Mustela putorius NC020638 4	0,18329304
Gulo gulo NC009685 3	Civettictis civetta NC033378 1	0,1832933
Panthera onca NC022842 1	Meles meles T303 3	0,18329337
Ursus arctos EU497665 29	Melogale moschata NC020644 1	0,18329424
Puma yagouaroundi NC028311 1	Martes pennanti NC020664 16	0,18329498
Paradoxurus hermaphroditus NLNC 1	Mustela nivalis T306 5	0,18329524
Otocolobus manul NC028323 1	Arctocephalus pusillus NC008417 1	0,1832954
Profelis aurata NC028299 1	Procyon lotor AB462046 3	0,18329556
Prionailurus bengalensis CKM45 20	Poecilogale albinucha T602 1	0,18329911
Otocyon megalotis SAF1 2	Monachus schauinslandi NC008421 1	0,18329942
Mustela sibirica AP017394 11	Arctocephalus forsteri NC004023 28	0,18330021
Zalophus wollebaeki SRR4431565 1	Catopuma badia NC028300 1	0,18330471
Ursus spelaeus EU327344 13	Fossa fossana D350 1	0,18330623
Smilodon populator MF871700 1	Gulo gulo NC009685 3	0,18330854
Mephitis mephitis NC020648 1	Arctodus simus NC011116 1	0,18330984
Viverricula indica NC025296 2	Ursus spelaeus NC011112 8	0,18331039
Ursus spelaeus EU327344 13	Poecilogale albinucha T602 1	0,18331194
Ommatophoca rossii AY377287etc 1	Ichneumia albicauda T603 1	0,18331313
Ursus thibetanus mupinensis NC00875	Arctictis binturong T605 2	0,18331537
Odobenus rosmarus NC004029 29	Arctodus simus NC011116 1	0,18331544
Ursus spelaeus NC011112 8	Catopuma temminckii NC027115 41	0,18331583
Ursus thibetanus laniger MH281753 2	Catopuma badia NC028300 1	0,18331617
Leopardus pardalis NC028315 1	Canis mesomelas KT448280 1	0,18331653
Lynx pardinus NC028319 161	Lycalopex sechurae KT448284 1	0,18331753
Zalophus californianus NC008416 1	Paguma larvata PDDS11 2	0,18331887
Ursus americanus JX196366 3	Melogale moschata KP726273 1	0,18332018
Ursus americanus JX196366 3	Enhydra lutris NC009692 1	0,18332197
Zalophus californianus NC008416 1	Prionailurus bengalensis CKM45 20	0,18332367
Prionodon linsang ERR2391707 1	Halichoerus grypus NC001602 2	0,18332441
Civettictis civetta NC033378 1	Ailurus fulgens NC011124 1	0,18332656
Zalophus californianus NC008416 1	Ursus arctos AP012576 6	0,18332773
Ursus americanus JX196366 3	Mustela kathiah NC023210 1	0,18332855
Ursus americanus JX196366 3	Mustela nigripes NC024942 1	0,18332949
Martes flavigula NC012141 3	Canis adustus KT448271 1	0,18333042
Chrotogale owstoni T607 1	Arctocephalus forsteri KT693377 17	0,18333286
Mephitis mephitis NC020648 1	Gulo gulo NC009685 3	0,18333688
Arctocephalus townsendi NC008420 1	Ailurus fulgens NC011124 1	0,18333775
Mustela sibirica AP017394 11	Arctocephalus australis MG023139 1	0,1833391
Lutra sumatrana NC035810 1	Eupleres goudotii D128 1	0,18333957
Leopardus jacobita NC028322 1	Tapirus terrestris T358	0,18334091
Neophoca cinerea NC008419 1	Mustela sibirica NC020637 6	0,18334195
Neophoca cinerea NC008419 1	Mustela sibirica AP017394 11	0,18334198
Viverra zibetha T609 1	Enhydra lutris NC009692 1	0,18334287
Ursus maritimus GU573488 Svalbard	Martes flavigula NC012141 3	0,18334296
Potos flavus T414 1	Helogale parvula SRR7637809 1	0,18334357
Potos flavus T414 1	Mungos mungo/gambianus SRR77048	0,18334371
Paguma larvata PDDS11 2	Callorhinus ursinus NC008415 1	0,18334451
Urocyon littoralis catalinae KP129018	Mirounga leonina NC008422 1	0,183345
Melogale moschata NC020644 1	Crossarchus platycephalus C7R66 1	0,1833452
Mungotictis decemlineata NC027828 1	Arctonyx collaris NC020645 1	0,18334679
Lutrogale perspicillata NC035811 1	Arctocephalus townsendi NC008420 1	0,18334684
Vulpes corsac NC023958 1	Mustela erminea T305 2	0,18334821
Civettictis civetta GLC19 1	Callorhinus ursinus NC008415 1	0,18334864
Meles meles T303 3	Ichneumia albicauda T603 1	0,18334897
Ursus maritimus GU573488 Svalbard	Taxidea taxus NC020646 1	0,18334961
Prionailurus planiceps KY682741 4	Meles anakuma NC009677 1	0,18335018
Ursus arctos GU573491 207	Callorhinus ursinus NC008415 1	0,18335034
Mustela nivalis T306 5	Arctocephalus gazella BK010918 1	0,18335082
Vulpes corsac NC023958 1	Smilodon populator MF871700 1	0,18335204
Mustela kathiah NC023210 1	Diplogale hosei MH464790 1	0,18335231
Viverricula indica KX891751 1	Mustela sibirica NC020637 6	0,18335268
Paradoxurus hermaphroditus NLNC 1	Lycalopex sechurae KT448284 1	0,18335281
Pardofelis marmorata NLN3 2	Lutra sumatrana NC035810 1	0,1833536
Ictonyx striatus T299 1	Arctocephalus forsteri KT693377 17	0,18335432
Tremarctos ornatus NC009969 2	Poecilogale albinucha T602 1	0,18335482
Mustela itatsi NC034330 19	Leptailurus serval NC028316 1	0,18335505
Vulpes vulpes NC008434 5	Felis nigripes NC028309 1	0,18335524
Lynx pardinus NC028319 161	Arctocephalus australis MG023139 1	0,18335563
Vulpes vulpes NC008434 5	Prionailurus planiceps NC028312 6	0,18335563
Mustela eversmannii NC028013 1	Leopardus guigna NC028321 1	0,18335631
Mustela erminea T305 2	Hyaena hyaena NC020669 1	0,1833566
Lynx rufus NC014456 3	Canis latrans NC008093 7	0,18335724
Ursus arctos isabellinus 18B5 2	Neophoca cinerea NC008419 1	0,18335828
Paradoxurus hermaphroditus NC03959	Monachus schauinslandi NC008421 1	0,18335883
Viverra zibetha T609 1	Arctocephalus pusillus NC008417 1	0,18335907
Lynx rufus NC014456 3	Arctotherium sp NC030174 1	0,1833621
Phoca largha NC008430 1	Paradoxurus jerdoni MH464793 1	0,1833622
Poecilogale albinucha T602 1	Leptailurus serval NC028316 1	0,18336242
Paradoxurus jerdoni MH464793 1	Mustela nivalis T306 5	0,18336244

Viverricula indica KX891751 1	Paguma larvata PDDS11 2	0,12302202
Neofelis nebulosa NC008450 3	Acinonyx jubatus NC005212 3	0,12304734
Viverricula indica KX891751 1	Paradoxurus hermaphroditus NC039591 1	0,12315669
Viverricula indica KX891745 1	Genetta genetta T297 1	0,12329158
Mungotictis decemlineata NC027828 1	Fossa fossana D350 1	0,12335231
Leptailurus serval NC028316 1	Homotherium latidens MF871702 3	0,12337658
Viverricula indica KX891745 1	Paguma larvata PDDS11 2	0,12342604
Genetta genetta T297 1	Civettictis civetta GLC19 1	0,12342636
Prionailurus bengalensis NC028301 12	Homotherium latidens MF871702 3	0,12355223
Mustela erminea T305 2	Martes americana NC020642 1	0,12357269
Mustela erminea T305 2	Martes flavigula NC012141 3	0,1236658
Viverricula indica NC025296 2	Genetta genetta T297 1	0,12376572
Mustela erminea T305 2	Martes foina NC020643 1	0,12378042
Viverricula indica NC025296 2	Paradoxurus jerdoni MH464793 1	0,1238306
Viverricula indica NC025296 2	Paguma larvata PDDS11 2	0,12389894
Viverricula indica KX891745 1	Paradoxurus jerdoni MH464793 1	0,12396472
Viverra zibetha T609 1	Arctictis binturong T605 2	0,12397692
Genetta genetta T297 1	Civettictis civetta NC033378 1	0,12399235
Fossa fossana D350 1	Cryptoprocta ferox CFC13 1	0,12409792
Smilodon populator MF871700 1	Panthera leo NERO 19	0,12421898
Paradoxurus jerdoni MH464793 1	Chrotogale owstoni T607 1	0,12422005
Hemigalus derbyanus MH464791 1	Civettictis civetta GLC19 1	0,12425752
Viverricula indica KX891745 1	Paradoxurus hermaphroditus NC039591 1	0,12436873
Lynx rufus NC014456 3	Homotherium latidens MF871702 3	0,12439425
Viverricula indica KX891745 1	Arctictis binturong T605 2	0,12458292
Viverra zibetha T609 1	Paradoxurus hermaphroditus NLNC 1	0,12463807
Viverricula indica KX891751 1	Arctictis binturong T605 2	0,12465006
Viverra zibetha T609 1	Genetta genetta T297 1	0,12470578
Paguma larvata PDDS11 2	Chrotogale owstoni T607 1	0,12475952
Lynx pardinus NC028319 161	Homotherium latidens MF871702 3	0,12479014
Salanoia concolor D378 1	Eupleres goudotii D128 1	0,12490153
Mustela erminea T305 2	Martes pennanti NC020664 16	0,12492132
Viverricula indica KX891745 1	Hemigalus derbyanus MH464791 1	0,12518542
Smilodon populator MF871700 1	Panthera pardus NC010641 5	0,12521553
Hemigalus derbyanus MH464791 1	Genetta servalina NC024568 2	0,12530643
Viverricula indica NC025296 2	Paradoxurus hermaphroditus NC039591 1	0,12531167
Smilodon populator MF871700 1	Panthera pardus japonensis KJ866876 8	0,12533127
Paradoxurus hermaphroditus NLNC 1	Chrotogale owstoni T607 1	0,12536396
Smilodon populator MF871700 1	Panthera tigris NC010642 35	0,12545127
Otocolobus manul NC028323 1	Homotherium latidens MF871702 3	0,12550452
Hemigalus derbyanus MH464791 1	Civettictis civetta NC033378 1	0,12550922
Viverra zibetha T609 1	Paradoxurus jerdoni MH464793 1	0,12551343
Leopardus tigrinus NC028317 1	Homotherium latidens MF871702 3	0,12556565
Leopardus jacobita NC028322 1	Homotherium latidens MF871702 3	0,12562697
Smilodon populator MF871700 1	Panthera onca NC022842 1	0,12563597
Paradoxurus hermaphroditus NLNC 1	Diplogale hosei MH464790 1	0,12564659
Lutra lutra LC050126 1	Lontra canadensis SRR10409165 1	0,12571506
Leopardus pardalis T262 1	Homotherium latidens MF871702 3	0,12572984
Smilodon populator MF871700 1	Panthera uncia KP202269 1	0,12576264
Paguma larvata PDDS11 2	Diplogale hosei MH464790 1	0,12577026
Leopardus geoffroyi NC028320 1	Homotherium latidens MF871702 3	0,12579259
Smilodon populator MF871700 1	Panthera tigris amoyensis NC014770 2	0,12582147
Leopardus guigna NC028321 1	Homotherium latidens MF871702 3	0,12583199
Mungotictis decemlineata NC027828 1	Eupleres goudotii D128 1	0,12584354
Prionailurus viverrinus NC028305 1	Homotherium latidens MF871702 3	0,12586959
Lontra canadensis SRR10409165 1	Enhydra lutris NC009692 1	0,12591696
Prionailurus rubiginosus NC028304 2	Homotherium latidens MF871702 3	0,12591739
Lutra sumatrana NC035810 1	Lontra canadensis SRR10409165 1	0,12598425
Lynx canadensis NC028313 1	Homotherium latidens MF871702 3	0,12598515
Genetta servalina NC024568 2	Arctictis binturong T605 2	0,12599693
Smilodon populator MF871700 1	Panthera onca KP202264 2	0,12607536
Leopardus colocolo NC028314 1	Homotherium latidens MF871702 3	0,12611377
Viverricula indica KX891751 1	Hemigalus derbyanus MH464791 1	0,12611414
Mustela frenata NC020640 1	Lutra sumatrana NC035810 1	0,12612361
Paradoxurus jerdoni MH464793 1	Diplogale hosei MH464790 1	0,12613006
Viverricula indica NC025296 2	Arctictis binturong T605 2	0,12613279
Paguma larvata PDDS11 2	Genetta abyssinica MG489822 1	0,12613352
Smilodon populator MF871700 1	Panthera leo spelaea KX258452 2	0,12613575
Paradoxurus hermaphroditus NC039591 1	Chrotogale owstoni T607 1	0,12617245
Puma yagouaroundi NC028311 1	Homotherium latidens MF871702 3	0,1262117
Chrotogale owstoni T607 1	Arctictis binturong T605 2	0,12631948
Homotherium latidens MF871702 3	Felis chaus NC028307 1	0,12637964
Prionailurus planiceps KY682741 4	Homotherium latidens MF871702 3	0,12638649
Paradoxurus jerdoni MH464793 1	Genetta servalina NC024568 2	0,12638879
Smilodon populator MF871700 1	Neofelis nebulosa NC008450 3	0,12639903
Mustela erminea T305 2	Melogale moschata KP726273 1	0,12639928
Homotherium latidens MF871702 3	Catopuma badia NC028300 1	0,12644921
Galidictis fasciata DM333 1	Cryptoprocta ferox CFC13 1	0,12645467
Viverra zibetha T609 1	Paradoxurus hermaphroditus NC039591 1	0,12645613
Prionailurus planiceps NC028312 6	Homotherium latidens MF871702 3	0,12645705
Hemigalus derbyanus MH464791 1	Genetta abyssinica MG489822 1	0,12650274
Lutra lutra NC011358 9	Lontra canadensis SRR10409165 1	0,12658994
Galidictis fasciata DM333 1	Eupleres goudotii D128 1	0,12665293
Melogale moschata NC020644 1	Martes melampus NC009678 1	0,1266631
Leopardus pardalis NC028315 1	Homotherium latidens MF871702 3	0,12666376
Paradoxurus hermaphroditus NLNC 1	Genetta servalina NC024568 2	0,12686014
Civettictis civetta GLC19 1	Arctictis binturong T605 2	0,12687282
Viverricula indica NC025296 2	Diplogale hosei MH464790 1	0,12692247
Lynx lynx NC027083 4	Homotherium latidens MF871702 3	0,12695476
Mustela frenata NC020640 1	Lutra lutra LC050126 1	0,1269985
Melogale moschata NC020644 1	Martes foina NC020643 1	0,12700573
Paradoxurus hermaphroditus NLNC 1	Civettictis civetta GLC19 1	0,12706216
Melogale moschata NC020644 1	Martes zibellina NC011579 39	0,127067
Melogale moschata NC020644 1	Martes martes T302 3	0,12707634
Paradoxurus hermaphroditus NC039591 1	Diplogale hosei MH464790 1	0,1271435
Leopardus wiedii NC028318 1	Homotherium latidens MF871702 3	0,12715741
Paguma larvata PDDS11 2	Genetta genetta T297 1	0,12719727
Panthera leo NERO 19	Homotherium latidens MF871702 3	0,1272129
Civettictis civetta NC033378 1	Arctictis binturong T605 2	0,12730283
Neovison vison NC020641 3	Lutra sumatrana NC035810 1	0,12733865
Puma concolor NC016470 22	Homotherium latidens MF871702 3	0,12734319
Salanoia concolor D378 1	Cryptoprocta ferox CFC13 1	0,12746438
Melogale moschata V0735A 1	Martes melampus NC009678 1	0,12746483

Prionailurus viverrinus NC028305 1	Poecilogale albinucha T602 1	0,18336253
Ursus maritimus GU573488 Svalbard	Martes martes T302 3	0,18336421
Otocyon megalotis SAF1 2	Lynx rufus NC014456 3	0,18336447
Mustela nivalis T306 5	Felis margarita NC028308 1	0,18336448
Zalophus wollebaeki SRR4431565 1	Mustela nivalis T306 5	0,18336704
Zalophus wollebaeki SRR4431565 1	Civettictis civetta GLC19 1	0,18337004
Prionodon pardicolor NC024569 2	Monachus schauinslandi NC008421 1	0,18337084
Ursus arctos GU573486 5	Mustela nivalis T306 5	0,18337165
Paradoxurus hermaphroditus NC03959	Canis aureus KT448274 1	0,18337211
Otaria byronia OTAB 1	Melursus ursinus NC009970 2	0,18337287
Lycalopex sechurae KT448284 1	Leopardus wiedii NC028318 1	0,18337293
Zalophus wollebaeki SRR4431565 1	Leopardus geoffroyi NC028320 1	0,18337423
Smilodon populator MF871700 1	Martes flavigula NC012141 3	0,18337491
Leptailurus serval NC028316 1	Chrysocyon brachyurus NC024172 1	0,18337627
Smilodon populator MF871700 1	Procyon lotor AB462049 4	0,18337631
Ursus arctos pruinosus MG066703 3	Neovison vison NC020641 3	0,18337645
Mustela erminea T305 2	Lycalopex sechurae KT448284 1	0,18337719
Ursus thibetanus formosanus NC0093	Caracal caracal NC028306 1	0,18337955
Leopardus pardalis T262 1	Helarctos malayanus NC009968 2	0,18337989
Zalophus californianus NC008416 1	Viverricula indica KX891745 1	0,18338315
Ursus thibetanus thibetanus NC01111	Leopardus guigna NC028321 1	0,18338403
Ursus spelaeus NC011112 8	Smilodon populator MF871700 1	0,1833865
Melogale moschata KP726273 1	Civettictis civetta NC033378 1	0,18338677
Zalophus californianus NC008416 1	Leopardus wiedii NC028318 1	0,18338735
Martes martes T302 3	Canis lupus chanco NC010340 4	0,18338796
Smilodon populator MF871700 1	Canis anthus NC027956 2	0,18339039
Ursus americanus JX196366 3	Martes foina NC020643 1	0,18340215
Urva semitorquata MH464789 1	Enhydra lutris NC009692 1	0,18340428
Paguma larvata PDDS11 2	Lutra lutra LC050126 1	0,18340577
Mustela putorius NC020638 4	Mungos mungo MMC7 1	0,1834074
Viverra tangalunga MH464792 1	Urva semitorquata MH464789 1	0,1834088
Mungotictis decemlineata NC027828 1	Melogale moschata KP726273 1	0,18341066
Taxidea taxus NC020646 1	Paradoxurus hermaphroditus NC03959	0,18341076
Vulpes lagopus NC026529 3	Nandinia binotata NC024567 1	0,18341099
Ursus americanus JX196366 3	Arctictis binturong T605 2	0,18341121
Otaria byronia OTAB 1	Crossarchus platycephalus C7R66 1	0,18341209
Vulpes zerda KJ603240 1	Mirounga leonina NC008422 1	0,18341234
Leopardus pardalis T262 1	Tapirus terrestris T358	0,18341322
Urva semitorquata MH464789 1	Bassariscus sumichrasti SRX1099089 1	0,18341334
Xenogale naso C07XAR110 1	Canis adustus KT448271 1	0,18341444
Vulpes vulpes NC008434 5	Mustela erminea T305 2	0,18341509
Lutrogale perspicillata NC035811 1	Arctocephalus gazella BK010918 1	0,18341669
Ursus arctos isabellinus 18B5 2	Callorhinus ursinus NC008415 1	0,18341716
Leopardus jacobita NC028322 1	Ailurus fulgens NC011124 1	0,18341726
Prionailurus planiceps NC028312 6	Meles anakuma NC009677 1	0,18341726
Felis silvestris lybica KP202275 4	Ailurus fulgens NC011124 1	0,18341839
Civettictis civetta GLC19 1	Arctocephalus gazella BK010918 1	0,18341915
Mustela sibirica NC020637 6	Arctotherium sp NC030174 1	0,1834193
Ursus arctos EU497665 29	Martes melampus NC009678 1	0,18341941
Felis chaus NC028307 1	Ailurus fulgens NC011124 1	0,18341979
Ursus arctos isabellinus 18B5 2	Martes zibellina NC011579 39	0,18342046
Mustela sibirica NC020637 6	Leptailurus serval NC028316 1	0,18342218
Prionodon pardicolor NC024569 2	Meles anakuma NC009677 1	0,18342221
Ursus americanus JX196366 3	Genetta abyssinica MG489822 1	0,18342228
Vulpes corsac NC023958 1	Phoca vitulina NC001325 1	0,18342268
Vulpes ferrilata NC027935 1	Leopardus pardalis T262 1	0,18342288
Martes zibellina NC011579 39	Catopuma temminckii NC027115 41	0,18342294
Vulpes vulpes NC008434 5	Leopardus tigrinus NC028317 1	0,183423
Vulpes zerda KJ603240 1	Lynx pardinus NC028319 161	0,18342347
Melogale moschata KP726273 1	Leptailurus serval NC028316 1	0,18342349
Vulpes vulpes NC008434 5	Otocolobus manul NC028323 1	0,18342361
Lycaon pictus NC028427 2	Cynictis penicillata T375 1	0,18342362
Ictonyx striatus T299 1	Arctocephalus gazella BK010918 1	0,18342367
Salanoia concolor D378 1	Neovison vison NC020641 3	0,18342391
Zalophus wollebaeki SRR4431565 1	Smilodon populator MF871700 1	0,18342425
Mustela sibirica AP017394 11	Lycaon pictus NC028427 2	0,18342565
Neovison vison NC020641 3	Canis anthus NC027956 2	0,18342596
Mustela altaica NC021751 1	Arctotherium sp NC030174 1	0,18342607
Ursus maritimus GU573488 Svalbard	Arctonyx collaris NC020645 1	0,18342699
Ursus arctos isabellinus 18B5 2	Martes martes T302 3	0,18342759
Lynx rufus NC014456 3	Bassariscus sumichrasti SRX1099089 1	0,18342841
Viverricula indica KX891751 1	Mustela nivalis T306 5	0,18342899
Panthera tigris NC010642 35	Callorhinus ursinus NC008415 1	0,18342926
Prionailurus planiceps NC028312 6	Martes pennanti NC020664 16	0,18342933
Puma yagouaroundi NC028311 1	Procyon lotor AB462049 4	0,18342941
Procyon lotor AB462046 3	Caracal caracal NC028306 1	0,18342957
Prionailurus bengalensis NC028301 12	Arctocephalus pusillus NC008417 1	0,18342969
Martes pennanti NC020664 16	Catopuma temminckii NC027115 41	0,18343002
Leopardus geoffroyi NC028320 1	Arctocephalus pusillus NC008417 1	0,18343076
Ursus arctos pruinosus MG066703 3	Ailurus fulgens styani NC009691 1	0,18343247
Ursus thibetanus thibetanus NC01111	Lutra lutra NC011358 9	0,18343258
Ursus maritimus GU573488 Svalbard	Mustela nivalis T306 5	0,18343273
Prionailurus bengalensis CKM45 20	Melogale moschata NC020644 1	0,18343298
Suricata suricatta SSM10 1	Ommatophoca rossii AY377287etc 1	0,18343307
Lycalopex sechurae KT448284 1	Genetta servalina NC024568 2	0,18343489
Zalophus wollebaeki SRR4431565 1	Paguma larvata PDDS11 2	0,18343645
Canis anthus NC027956 2	Arctictis binturong T605 2	0,18343689
Lycalopex sechurae KT448284 1	Leopardus pardalis T262 1	0,18343751
Zalophus wollebaeki SRR4431565 1	Lynx pardinus NC028319 161	0,18343858
Leopardus geoffroyi NC028320 1	Canis aureus KT448274 1	0,1834392
Zalophus wollebaeki SRR4431565 1	Otocolobus manul NC028323 1	0,18343925
Speothos venaticus C48 2	Mirounga angustirostris SRR10331586	0,18343961
Zalophus wollebaeki SRR4431565 1	Prionailurus bengalensis NC028301 12	0,18344009
Poecilogale albinucha T602 1	Arctictis binturong T605 2	0,18344118
Martes melampus NC009678 1	Canis lupus familiaris NC002008 1231	0,18344155
Smilodon populator MF871700 1	Mustela altaica NC021751 1	0,18344155
Ursus thibetanus formosanus NC0093	Otaria byronia OTAB 1	0,18344177
Mungos mungo MMC7 1	Canis lupus chanco NC010340 4	0,18344186
Civettictis civetta GLC19 1	Chrysocyon brachyurus NC024172 1	0,18344362
Martes zibellina NC011579 39	Canis lupus familiaris NC002008 1231	0,18344437
Helarctos malayanus NC009968 2	Genetta genetta T297 1	0,18344526
Ursus thibetanus formosanus NC0093	Felis chaus NC028307 1	0,18344583
Paguma larvata PDDS11 2	Helarctos malayanus NC009968 2	0,1834476

Panthera onca NC022842 1	Homotherium latidens MF871702 3	0,12757204
Homotherium latidens MF871702 3	Felis margarita NC028308 1	0,1275928
Melogale moschata KP726273 1	Martes melampus NC009678 1	0,12759943
Melogale moschata V0735A 1	Martes foina NC020643 1	0,12760527
Mustela erminea T305 2	Aonyx cinerea NC035814 2	0,12761182
Paradoxurus jerdoni MH464793 1	Genetta abyssinica MG489822 1	0,1276215
Mustela erminea T305 2	Melogale moschata NC020644 1	0,12767817
Panthera onca KP202264 2	Homotherium latidens MF871702 3	0,12767838
Homotherium latidens MF871702 3	Acinonyx jubatus NC005212 3	0,12771998
Profelis aurata NC028299 1	Homotherium latidens MF871702 3	0,12772791
Homotherium latidens MF871702 3	Felis silvestris lybica KP202275 4	0,12776919
Homotherium latidens MF871702 3	Caracal caracal NC028306 1	0,12779881
Paguma larvata PDDS11 2	Civettictis civetta GLC19 1	0,12780285
Mustela nigripes NC024942 1	Enhydra lutris NC009692 1	0,12780699
Melogale moschata KP726273 1	Martes foina NC020643 1	0,12780729
Melogale moschata V0735A 1	Martes martes T302 3	0,12781055
Mustela putorius NC020638 4	Enhydra lutris NC009692 1	0,12787428
Melogale moschata NC020644 1	Martes americana NC020642 1	0,12787451
Martes flavigula NC012141 3	Lutra sumatrana NC035810 1	0,12797642
Melogale moschata V0735A 1	Martes zibellina NC011579 39	0,12800322
Mustela frenata NC020640 1	Enhydra lutris NC009692 1	0,128143
Melogale moschata KP726273 1	Martes martes T302 3	0,12821439
Melogale moschata KP726273 1	Lutra sumatrana NC035810 1	0,12827243
Mustela itatsi NC034330 19	Enhydra lutris NC009692 1	0,12827787
Panthera uncia KP202269 1	Homotherium latidens MF871702 3	0,12831178
Galidia elegans D146 1	Fossa fossana D350 1	0,12833452
Mustela frenata NC020640 1	Lutra lutra NC011358 9	0,12834451
Mustela altaica NC021751 1	Martes melampus NC009678 1	0,12835054
Diplogale hosei MH464790 1	Civettictis civetta GLC19 1	0,12844037
Melogale moschata KP726273 1	Martes zibellina NC011579 39	0,12847432
Mustela nigripes NC024942 1	Martes melampus NC009678 1	0,12847936
Diplogale hosei MH464790 1	Arctictis binturong T605 2	0,12849426
Mustela erminea T305 2	Ictonyx striatus T299 1	0,12856328
Paguma larvata PDDS11 2	Civettictis civetta NC033378 1	0,12857017
Homotherium latidens MF871702 3	Felis nigripes NC028309 1	0,12859359
Paradoxurus hermaphroditus NC039591 1	Genetta servalina NC024568 2	0,12861086
Smilodon populator MF871700 1	Panthera uncia NC010638 1	0,12866794
Eupleres goudotii D128 1	Cryptoprocta ferox CFC13 1	0,12867826
Mustela sibirica NC020637 6	Enhydra lutris NC009692 1	0,12868191
Mustela sibirica AP017394 11	Enhydra lutris NC009692 1	0,128682
Viverricula indica KX891751 1	Diplogale hosei MH464790 1	0,12868801
Homotherium latidens MF871702 3	Felis catus NC001700 2	0,12868854
Neovison vison NC020641 3	Enhydra lutris NC009692 1	0,12869066
Neovison vison NC020641 3	Melogale moschata NC020644 1	0,1286916
Mungotictis decemlineata NC027828 1	Cryptoprocta ferox CFC13 1	0,1288107
Mustela altaica NC021751 1	Enhydra lutris NC009692 1	0,12882262
Neovison vison NC020641 3	Martes flavigula NC012141 3	0,12884991
Melogale moschata KP726273 1	Enhydra lutris NC009692 1	0,12887812
Mustela sibirica NC020637 6	Martes melampus NC009678 1	0,12888326
Neovison vison NC020641 3	Lutra lutra LC050126 1	0,12888668
Mustela altaica NC021751 1	Lutra lutra LC050126 1	0,12888914
Mustela altaica NC021751 1	Martes zibellina NC011579 39	0,12889431
Melogale moschata V0735A 1	Martes americana NC020642 1	0,12894543
Melogale moschata NC020644 1	Enhydra lutris NC009692 1	0,12895215
Martes martes T302 3	Lutra sumatrana NC035810 1	0,1289542
Genetta abyssinica MG489822 1	Arctictis binturong T605 2	0,12907046
Paradoxurus hermaphroditus NLNC 1	Civettictis civetta NC033378 1	0,12910819
Mustela nigripes NC024942 1	Martes flavigula NC012141 3	0,12912056
Martes melampus NC009678 1	Lutra sumatrana NC035810 1	0,12921461
Melogale moschata KP726273 1	Martes americana NC020642 1	0,12921461
Paradoxurus hermaphroditus NLNC 1	Genetta genetta T297 1	0,129217
Ursus thibetanus thibetanus NC011118 4	Arctodus simus NC011116 1	0,12922782
Martes flavigula NC012141 3	Lutra lutra LC050126 1	0,12925565
Viverricula indica NC025296 2	Chrotogale owstoni T607 1	0,12927376
Mustela sibirica AP017394 11	Martes melampus NC009678 1	0,12928711
Mustela eversmannii NC028013 1	Enhydra lutris NC009692 1	0,12928778
Panthera pardus japonensis KJ866876 8	Homotherium latidens MF871702 3	0,12930225
Homotherium latidens MF871702 3	Catopuma temminckii NC027115 41	0,1293024
Ursus thibetanus mupinensis NC008753 2	Arctodus simus NC011116 1	0,12935331
Mustela putorius NC020638 4	Martes melampus NC009678 1	0,12935434
Mustela sibirica AP017394 11	Martes martes T302 3	0,12936427
Mustela frenata NC020640 1	Martes flavigula NC012141 3	0,12938687
Neovison vison NC020641 3	Arctonyx collaris NC020645 1	0,12942721
Poecilogale albinucha T602 1	Mustela erminea T305 2	0,12943478
Martes flavigula NC012141 3	Lutra lutra NC011358 9	0,12945858
Paradoxurus hermaphroditus NLNC 1	Genetta abyssinica MG489822 1	0,12953131
Viverra zibetha T609 1	Chrotogale owstoni T607 1	0,12954254
Mustela erminea T305 2	Melogale moschata V0735A 1	0,12956285
Viverricula indica KX891745 1	Diplogale hosei MH464790 1	0,12956387
Mustela altaica NC021751 1	Martes foina NC020643 1	0,12957297
Mustela frenata NC020640 1	Melogale moschata KP726273 1	0,12962995
Mustela frenata NC020640 1	Melogale moschata NC020644 1	0,1296304
Mustela sibirica NC020637 6	Martes martes T302 3	0,12963346
Mustela erminea T305 2	Gulo gulo NC009685 3	0,12965974
Civettictis civetta GLC19 1	Chrotogale owstoni T607 1	0,12967919
Paradoxurus jerdoni MH464793 1	Civettictis civetta GLC19 1	0,12968823
Mustela nigripes NC024942 1	Lutra sumatrana NC035810 1	0,12969095
Mustela altaica NC021751 1	Martes americana NC020642 1	0,12970167
Melogale moschata KP726273 1	Lutra lutra LC050126 1	0,12975301
Pardofelis marmorata NLN3 2	Homotherium latidens MF871702 3	0,12975444
Martes pennanti NC020664 16	Enhydra lutris NC009692 1	0,12976302
Mustela nivalis T306 5	Enhydra lutris NC009692 1	0,12976517
Neovison vison NC020641 3	Melogale moschata KP726273 1	0,12976831
Mustela nigripes NC024942 1	Martes americana NC020642 1	0,12983128
Paradoxurus hermaphroditus NC039591 1	Civettictis civetta GLC19 1	0,12989025
Mustela nigripes NC024942 1	Martes martes T302 3	0,12990256
Mustela altaica NC021751 1	Martes martes T302 3	0,12990861
Viverra zibetha T609 1	Diplogale hosei MH464790 1	0,12994885
Neovison vison NC020641 3	Lutra lutra NC011358 9	0,12996332
Viverricula indica KX891745 1	Chrotogale owstoni T607 1	0,13001248
Melogale moschata NC020644 1	Lutra sumatrana NC035810 1	0,13002895
Ursus thibetanus formosanus NC009331 1	Arctodus simus NC011116 1	0,13003102
Ursus thibetanus laniger MH281753 2	Arctodus simus NC011116 1	0,13003547

Ursus thibetanus formosanus NC0093	Catopuma temminckii NC027115 41	0,1834486
Lutra lutra LC050126 1	Civettictis civetta NC033378 1	0,18344863
Canis mesomelas KT448280 1	Arctictis binturong T605 2	0,18344928
Ursus thibetanus formosanus NC0093	Arctocephalus forsteri NC004023 28	0,18345103
Zalophus californianus NC008416 1	Felis margarita NC028308 1	0,18345331
Panthera tigris amoyensis NC014770 2	Ommatophoca rossii AY377287etc 1	0,18345398
Zalophus californianus NC008416 1	Viverricula indica NC025296 2	0,18345445
Melogale moschata NC020644 1	Canis adustus KT448271 1	0,18345526
Mustela altaica NC021751 1	Canis lupus chanco NC010340 4	0,18345779
Lutrogale perspicillata NC035811 1	Eumetopias jubatus NC004030 10	0,18345849
Martes flavigula NC012141 3	Lycaon pictus NC028427 2	0,18345886
Ursus americanus JX196366 3	Lutra sumatrana NC035810 1	0,18345976
Ommatophoca rossii AY377287etc 1	Canis mesomelas KT448280 1	0,18346362
Ursus americanus JX196366 3	Martes americana NC020642 1	0,18346362
Eumetopias jubatus NC004030 10	Catopuma badia NC028300 1	0,18346725
Mustela altaica NC021751 1	Diplogale hosei MH464790 1	0,18346737
Genetta servalina NC024568 2	Eumetopias jubatus NC004030 10	0,18346745
Mustela sibirica NC020637 6	Arctocephalus forsteri KT693377 17	0,18347375
Mungotictis decemlineata NC027828 1	Lutra lutra NC011358 9	0,18347533
Cynogale bennetti KY117544 1	Arctonyx collaris NC020645 1	0,18347631
Viverra tangalunga MH464792 1	Phocarctos hookeri NC008418 1	0,18347658
Prionodon linsang ERR2391707 1	Mirounga leonina NC008422 1	0,18347697
Mungotictis decemlineata NC027828 1	Martes americana NC020642 1	0,18347737
Mustela kathiah NC023210 1	Cynogale bennetti KY117544 1	0,18347737
Vulpes vulpes NC008434 5	Crossarchus platycephalus C7R66 1	0,1834778
Ursus arctos EU497665 29	Eumetopias jubatus NC004030 10	0,18347789
Ursus spelaeus NC011112 8	Genetta abyssinica MG489822 1	0,1834784
Urocyon cinereoargenteus NC026723 2	Erignathus barbatus NC008426 1	0,18347853
Tremarctos ornatus NC009969 2	Meles anakuma NC009677 1	0,18347889
Viverra zibetha T609 1	Lutra sumatrana NC035810 1	0,18348303
Viverricula indica NC025296 2	Martes melampus NC009678 1	0,18348365
Zalophus wollebaeki SRR4431565 1	Chrotogale owstoni T607 1	0,18348433
Ursus arctos EU497665 29	Taxidea taxus NC020646 1	0,18348449
Mustela nivalis T306 5	Mungos mungo MMC7 1	0,18348522
Felis catus NC001700 2	Arctonyx collaris NC020645 1	0,18348543
Martes pennanti NC020664 16	Galidictis fasciata DM333 1	0,18348643
Neophoca cinerea NC008419 1	Mustela nivalis T306 5	0,18348692
Vulpes lagopus NC026529 3	Viverricula indica KX891751 1	0,18348692
Panthera uncia KP202269 1	Mustela kathiah NC023210 1	0,18348761
Speothos venaticus C48 2	Pusa caspica NC008431 1	0,18348819
Mustela putorius NC020638 4	Felis catus NC001700 2	0,18348862
Homotherium latidens MF871702 3	Arctocephalus australis MG023139 1	0,18348868
Potos flavus T414 1	Lynx canadensis NC028313 1	0,18348894
Ursus maritimus NC003428 31	Arctocephalus gazella BK010918 1	0,18348926
Mustela sibirica AP017394 11	Leptailurus serval NC028316 1	0,18348947
Prionailurus viverrinus NC028305 1	Mustela itatsi NC034330 19	0,18348964
Vulpes vulpes NC008434 5	Felis catus NC001700 2	0,18348974
Viverricula indica KX891745 1	Melogale moschata NC020644 1	0,1834898
Vulpes corsac NC023958 1	Phoca largha NC008430 1	0,1834898
Procyon lotor AB462049 4	Otaria byronia OTAB 1	0,18348986
Vulpes vulpes NC008434 5	Lynx canadensis NC028313 1	0,18349001
Paguma larvata PDDS11 2	Mustela altaica NC021751 1	0,18349098
Vulpes zerda KJ603240 1	Catopuma temminckii NC027115 41	0,18349135
Ursus thibetanus mupinensis NC00875	Salanoia concolor D378 1	0,18349147
Martes americana NC020642 1	Leopardus tigrinus NC028317 1	0,18349177
Catopuma temminckii NC027115 41	Ailurus fulgens styani NC009691 1	0,18349214
Lynx rufus NC014456 3	Arctocephalus pusillus NC008417 1	0,18349403
Smilodon populator MF871700 1	Neovison vison NC020641 3	0,18349424
Ursus maritimus NC003428 31	Arctonyx collaris NC020645 1	0,18349427
Procyon lotor AB462049 4	Lynx rufus NC014456 3	0,18349454
Homotherium latidens MF871702 3	Galictis vittata T412 1	0,1834947
Procyon lotor AB462046 3	Lynx rufus NC014456 3	0,18349482
Martes pennanti NC020664 16	Genetta genetta T297 1	0,18349509
Viverricula indica KX891745 1	Mustela nivalis T306 5	0,18349591
Puma concolor NC016470 22	Procyon lotor AB462046 3	0,1834961
Zalophus wollebaeki SRR4431565 1	Mungos mungo MMC7 1	0,18349639
Puma concolor NC016470 22	Martes pennanti NC020664 16	0,18349656
Prionailurus planiceps KY682741 4	Martes pennanti NC020664 16	0,18349679
Mustela nivalis T306 5	Genetta servalina NC024568 2	0,18349688
Mustela altaica NC021751 1	Leopardus tigrinus NC028317 1	0,18349689
Martes pennanti NC020664 16	Leopardus pardalis T262 1	0,18349692
Prionailurus rubiginosus NC028304 2	Poecilogale albinucha T602 1	0,18349725
Ursus thibetanus formosanus NC0093	Arctocephalus townsendi NC008420 1	0,18349788
Ursus thibetanus laniger MH281753 2	Lutra lutra LC050126 1	0,18349804
Mustela sibirica AP017394 11	Chrysocyon brachyurus NC024172 1	0,18349835
Procyon lotor AB462049 4	Catopuma badia NC028300 1	0,18349899
Martes martes T302 3	Caracal caracal NC028306 1	0,18349943
Lynx canadensis NC028313 1	Canis aureus KT448274 1	0,18350047
Viverra tangalunga MH464792 1	Arctictis binturong T605 2	0,18350284
Caracal caracal NC028306 1	Bassariscus sumichrasti SRX1099089 1	0,18350303
Zalophus wollebaeki SRR4431565 1	Viverricula indica KX891745 1	0,18350396
Melursus ursinus NC009970 2	Aonyx cinerea NC035814 2	0,18350542
Viverricula indica NC025296 2	Martes foina NC020643 1	0,18350565
Civettictis civetta GLC19 1	Ailuropoda melanoleuca NC009492 5	0,18350625
Zalophus wollebaeki SRR4431565 1	Viverricula indica NC025296 2	0,18350799
Ursus arctos AP012576 6	Mustela putorius NC020638 4	0,18350998
Vulpes ferrilata NC027935 1	Homotherium latidens MF871702 3	0,18351102
Mustela nivalis T306 5	Arctictis binturong T605 2	0,18351169
Gulo gulo NC009685 3	Crossarchus platycephalus C7R66 1	0,18351234
Martes zibellina NC011579 39	Canis lupus chanco NC010340 4	0,18351234
Ursus spelaeus NC011112 8	Leopardus wiedii NC028318 1	0,18351461
Ursus thibetanus thibetanus NC01111	Prionailurus rubiginosus NC028304 2	0,18351586
Ursus spelaeus NC011112 8	Caracal caracal NC028306 1	0,18351622
Ursus spelaeus NC011112 8	Leopardus geoffroyi NC028320 1	0,18351859
Ictonyx striatus T299 1	Arctictis binturong T605 2	0,18351939
Martes flavigula NC012141 3	Urva semitorquata MH464789 1	0,18352148
Leopardus wiedii NC028318 1	Arctocephalus forsteri NC004023 28	0,18352176
Zalophus californianus NC008416 1	Leptailurus serval NC028316 1	0,18352214
Hemigalus derbyanus MH464791 1	Galictis vittata T412 1	0,183523
Urocyon littoralis catalinae KP129018	Halichoerus grypus NC001602 2	0,18352823
Martes flavigula NC012141 3	Catopuma badia NC028300 1	0,18353017
Viverricula indica KX891751 1	Spilogale putorius NC010497 1	0,18353374
Martes flavigula NC012141 3	Felis catus NC001700 2	0,18353474

Martes americana NC020642 1	Lutra sumatrana NC035810 1	0,13008951
Melogale moschata KP726273 1	Lutra lutra NC011358 9	0,13008951
Hemigalus derbyanus MH464791 1	Genetta genetta T297 1	0,1301342
Melogale moschata V0735A 1	Enhydra lutris NC009692 1	0,1301568
Melogale moschata NC020644 1	Lutra lutra LC050126 1	0,13016334
Diplogale hosei MH464790 1	Civettictis civetta NC033378 1	0,13016795
Martes zibellina NC011579 39	Lutra sumatrana NC035810 1	0,13022411
Mustela itatsi NC034330 19	Lutra lutra LC050126 1	0,13029635
Mustela nigripes NC024942 1	Lutra lutra LC050126 1	0,13029657
Melogale moschata NC020644 1	Lutra lutra NC011358 9	0,13029791
Martes martes T302 3	Lutra lutra LC050126 1	0,13030045
Mustela nigripes NC024942 1	Martes zibellina NC011579 39	0,13030288
Panthera leo spelaea KX258452 2	Homotherium latidens MF871702 3	0,13035417
Mustela frenata NC020640 1	Martes melampus NC009678 1	0,13036345
Mustela kathiah NC023210 1	Enhydra lutris NC009692 1	0,13036384
Mustela sibirica NC020637 6	Lutra lutra LC050126 1	0,13036391
Martes foina NC020643 1	Arctonyx collaris NC020645 1	0,13036565
Martes martes T302 3	Lutra lutra NC011358 9	0,13036741
Melogale moschata NC020644 1	Martes flavigula NC012141 3	0,13040665
Martes pennanti NC020664 16	Lutra sumatrana NC035810 1	0,13043644
Mustela sibirica NC020637 6	Martes americana NC020642 1	0,13043694
Mustela putorius NC020638 4	Martes martes T302 3	0,1304411
Mustela altaica NC021751 1	Martes pennanti NC020664 16	0,13044785
Mustela itatsi NC034330 19	Martes melampus NC009678 1	0,13049845
Mustela sibirica NC020637 6	Lutra sumatrana NC035810 1	0,13049857
Mustela eversmannii NC028013 1	Martes melampus NC009678 1	0,13049865
Mustela erminea T305 2	Arctonyx collaris NC020645 1	0,13049898
Mustela sibirica AP017394 11	Martes americana NC020642 1	0,13050409
Mustela putorius NC020638 4	Martes americana NC020642 1	0,13050434
Mustela sibirica AP017394 11	Martes zibellina NC011579 39	0,13050456
Mustela nivalis T306 5	Martes melampus NC009678 1	0,13050474
Mustela sibirica AP017394 11	Melogale moschata NC020644 1	0,13050538
Spilogale putorius NC010497 1	Mephitis mephitis NC020648 1	0,13052028
Panthera pardus NC010641 5	Homotherium latidens MF871702 3	0,1305383
Panthera uncia NC010638 1	Homotherium latidens MF871702 3	0,13054007
Mustela kathiah NC023210 1	Lutra lutra LC050126 1	0,13056527
Mustela erminea T305 2	Meles leucurus NC039173 4	0,13056844
Mustela nivalis T306 5	Lutra sumatrana NC035810 1	0,13057204
Genetta servalina NC024568 2	Chrotogale owstoni T607 1	0,13062046
Mustela erminea T305 2	Lontra canadensis SRR10409165 1	0,13063218
Mustela putorius NC020638 4	Lutra sumatrana NC035810 1	0,13063313
Mustela sibirica NC020637 6	Martes zibellina NC011579 39	0,13063931
Mustela erminea T305 2	Lutrogale perspicillata NC035811 1	0,13064127
Mustela nivalis T306 5	Martes martes T302 3	0,13064897
Martes melampus NC009678 1	Lutra lutra NC011358 9	0,13069521
Paradoxurus jerdoni MH464793 1	Genetta genetta T297 1	0,13069853
Mustela itatsi NC034330 19	Martes zibellina NC011579 39	0,13070695
Mustela nigripes NC024942 1	Martes foina NC020643 1	0,13071197
Martes flavigula NC012141 3	Enhydra lutris NC009692 1	0,13073057
Mustela sibirica AP017394 11	Martes flavigula NC012141 3	0,13073692
Mustela altaica NC021751 1	Martes flavigula NC012141 3	0,13074307
Martes pennanti NC020664 16	Lutra lutra NC011358 9	0,13077308
Martes pennanti NC020664 16	Lutra lutra LC050126 1	0,13077313
Mustela frenata NC020640 1	Martes americana NC020642 1	0,1307738
Mustela nivalis T306 5	Martes zibellina NC011579 39	0,13077931
Paradoxurus hermaphroditus NC039591 1	Genetta abyssinica MG489822 1	0,13083141
Melogale moschata V0735A 1	Lutra sumatrana NC035810 1	0,1308971
Mustela altaica NC021751 1	Lutra lutra NC011358 9	0,13090822
Mustela sibirica NC020637 6	Melogale moschata NC020644 1	0,13090906
Martes melampus NC009678 1	Lutra lutra LC050126 1	0,13103169
Mustela kathiah NC023210 1	Lutra lutra NC011358 9	0,13103634
Mustela altaica NC021751 1	Lutra sumatrana NC035810 1	0,13104282
Neovison vison NC020641 3	Martes zibellina NC011579 39	0,1310477
Civettictis civetta NC033378 1	Chrotogale owstoni T607 1	0,13112278
Paradoxurus jerdoni MH464793 1	Civettictis civetta NC033378 1	0,13112836
Melogale moschata KP726273 1	Martes flavigula NC012141 3	0,13114116
Mustela nivalis T306 5	Martes flavigula NC012141 3	0,13114785
Viverricula indica KX891751 1	Chrotogale owstoni T607 1	0,1311568
Vulpes corsac NC023958 1	Canis adustus KT448271 1	0,13122536
Vulpes vulpes NC008434 5	Canis adustus KT448271 1	0,13123612
Mustela itatsi NC034330 19	Martes martes T302 3	0,13124572
Mustela putorius NC020638 4	Lutra lutra LC050126 1	0,13130607
Mustela kathiah NC023210 1	Martes martes T302 3	0,13131237
Mustela eversmannii NC028013 1	Lutra sumatrana NC035810 1	0,13137364
Neovison vison NC020641 3	Martes americana NC020642 1	0,13138391
Mustela sibirica NC020637 6	Martes foina NC020643 1	0,131385
Paradoxurus hermaphroditus NC039591 1	Civettictis civetta NC033378 1	0,13139741
Martes americana NC020642 1	Lutra lutra NC011358 9	0,1314355
Mustela frenata NC020640 1	Meles leucurus NC039173 4	0,13144304
Mustela sibirica AP017394 11	Melogale moschata KP726273 1	0,13144651
Mustela eversmannii NC028013 1	Martes martes T302 3	0,13145077
Viverra tangalunga MH464792 1	Enhydra lutris NC009692 1	0,13150829
Ursus thibetanus thibetanus NC011118 4	Arctotherium sp NC030174 1	0,13151428
Mustela putorius NC020638 4	Martes zibellina NC011579 39	0,13151442
Mustela nivalis T306 5	Martes foina NC020643 1	0,13152562
Paradoxurus jerdoni MH464793 1	Cynogale bennetti KY117544 1	0,13154703
Mustela itatsi NC034330 19	Lutra sumatrana NC035810 1	0,1315752
Mustela sibirica AP017394 11	Lutra lutra LC050126 1	0,13157545
Mustela eversmannii NC028013 1	Lutra lutra LC050126 1	0,13157547
Mustela sibirica NC020637 6	Melogale moschata KP726273 1	0,13158108
Mustela kathiah NC023210 1	Melogale moschata KP726273 1	0,13158123
Mustela nivalis T306 5	Lutra lutra LC050126 1	0,13158162
Mustela nigripes NC024942 1	Melogale moschata NC020644 1	0,13158195
Mustela itatsi NC034330 19	Martes flavigula NC012141 3	0,13161078
Neofelis nebulosa NC008450 3	Homotherium latidens MF871702 3	0,13164064
Neovison vison NC020641 3	Martes melampus NC009678 1	0,13164599
Mustela frenata NC020640 1	Melogale moschata V0735A 1	0,13164954
Mustela frenata NC020640 1	Martes pennanti NC020664 16	0,13165163
Mustela sibirica AP017394 11	Martes foina NC020643 1	0,13165416
Mustela putorius NC020638 4	Martes flavigula NC012141 3	0,13167915
Mustela eversmannii NC028013 1	Martes flavigula NC012141 3	0,13167989
Martes zibellina NC011579 39	Lutra lutra NC011358 9	0,13170469
Mustela eversmannii NC028013 1	Martes americana NC020642 1	0,13171569

Leptailurus serval NC028316 1	Eumetopias jubatus NC004030 10	0,18353651
Nasua nasua NC020647 1	Mustela eversmannii NC028013 1	0,18353735
Lutra lutra LC050126 1	Canis latrans NC008093 7	0,18353933
Mustela nigripes NC024942 1	Chrotogale owstoni T607 1	0,1835404
Cynictis penicillata T375 1	Arctonyx collaris NC020645 1	0,18354322
Lutra lutra LC050126 1	Galidictis fasciata DM333 1	0,18354398
Phocarctos hookeri NC008418 1	Mustela itatsi NC034330 19	0,18354401
Urocyon cinereoargenteus NC026723 2	Pusa sibirica NC008432 2	0,1835451
Potos flavus T414 1	Phocarctos hookeri NC008418 1	0,18354556
Mungotictis decemlineata NC027828 1	Meles leucurus NC039173 4	0,18354572
Bassariscus sumichrasti SRX1099089 1	Atilax paludinosus T606 1	0,18354715
Mirounga angustirostris SRR10331586	Tapirus terrestris T358	0,18354937
Mephitis mephitis NC020648 1	Lutra lutra NC011358 9	0,18354952
Paguma larvata PDDS11 2	Melogale moschata V0735A 1	0,18355167
Puma concolor NC016470 22	Ailurus fulgens styani NC009691 1	0,18355353
Otaria byronia OTAB 1	Chrotogale owstoni T607 1	0,1835537
Ursus arctos GU573491 207	Martes melampus NC009678 1	0,18355386
Mustela sibirica AP017394 11	Arctotherium sp NC030174 1	0,18355396
Vulpes zerda KJ603240 1	Viverricula indica KX891745 1	0,18355508
Phocarctos hookeri NC008418 1	Mustela altaica NC021751 1	0,18355519
Enhydra lutris NC009692 1	Arctictis binturong T605 2	0,1835555
Mustela itatsi NC034330 19	Felis nigripes NC028309 1	0,18355623
Martes foina NC020643 1	Atilax paludinosus T606 1	0,1835565
Vulpes vulpes NC008434 5	Prionailurus planiceps KY682741 4	0,1835577
Lynx lynx NC027083 4	Canis anthus NC027956 2	0,18355811
Lynx pardinus NC028319 161	Canis anthus NC027956 2	0,18355881
Taxidea taxus NC020646 1	Canis aureus KT448274 1	0,18355928
Felis catus NC001700 2	Canis anthus NC027956 2	0,18355951
Ursus maritimus GU573488 Svalbard	Neophoca cinerea NC008419 1	0,18356012
Nyctereutes procyonoides NC013700 3	Leopardus tigrinus NC028317 1	0,18356027
Viverricula indica KX891751 1	Procyon lotor AB462049 4	0,18356046
Catopuma badia NC028300 1	Ailurus fulgens NC011124 1	0,18356061
Ursus arctos AP012576 6	Eumetopias jubatus NC004030 10	0,18356113
Ursus arctos GU573491 207	Martes martes T302 3	0,18356253
Ursus arctos EU497665 29	Mustela itatsi NC034330 19	0,18356293
Felis catus NC001700 2	Aonyx cinerea NC035814 2	0,18356353
Pardofelis marmorata NLN3 2	Bassariscus sumichrasti SRX1099089 1	0,18356416
Proteles cristata T393 6	Mirounga leonina NC008422 1	0,18356453
Prionailurus bengalensis NC028301 12	Poecilogale albinucha T602 1	0,18356495
Prionailurus bengalensis CKM45 20	Neophoca cinerea NC008419 1	0,1835651
Ursus thibetanus formosanus NC0093	Melogale moschata V0735A 1	0,18356554
Ursus arctos AP012576 6	Callorhinus ursinus NC008415 1	0,18356794
Ursus arctos GU573486 5	Arctonyx collaris NC020645 1	0,1835683
Procyon lotor AB462046 3	Genetta abyssinica MG489822 1	0,18356913
Prionailurus bengalensis CKM45 20	Canis anthus NC027956 2	0,18356977
Mustela altaica NC021751 1	Canis aureus KT448274 1	0,18357231
Melogale moschata KP726273 1	Acinonyx jubatus NC005212 3	0,18357389
Ursus thibetanus mupinensis NC00875	Catopuma badia NC028300 1	0,18357484
Proteles cristata T393 6	Monachus schauinslandi NC008421 1	0,18357907
Zalophus californianus NC008416 1	Lynx lynx NC027083 4	0,18358552
Martes flavigula NC012141 3	Urva javanica T413 1	0,18358573
Urocyon cinereoargenteus NC026723 2	Monachus monachus NC044972 5	0,18358582
Ursus thibetanus mupinensis NC00875	Leopardus colocolo NC028314 1	0,18358919
Vulpes corsac NC023958 1	Hemigalus derbyanus MH464791 1	0,1835918
Mustela nigripes NC024942 1	Cuon alpinus NC013445 3	0,18359384
Puma yagouaroundi NC028311 1	Canis mesomelas KT448280 1	0,18359488
Nandinia binotata NC024567 1	Cuon alpinus NC013445 3	0,18359743
Lynx rufus NC014456 3	Lycalopex sechurae KT448284 1	0,18359767
Ursus thibetanus thibetanus NC01111	Leopardus colocolo NC028314 1	0,18360074
Lutra sumatrana NC035810 1	Atilax paludinosus T606 1	0,18360773
Mustela eversmannii NC028013 1	Arctocephalus townsendi NC008420 1	0,18360789
Pusa hispida NC 008433 1	Cynogale bennetti KY117544 1	0,18360831
Melogale moschata KP726273 1	Arctocephalus townsendi NC008420 1	0,18360832
Mustela sibirica AP017394 11	Urva semitorquata MH464789 1	0,18360975
Ursus arctos isabellinus 18B5 2	Genetta abyssinica MG489822 1	0,18361087
Mungos mungo MMC7 1	Arctocephalus forsteri KT693377 17	0,18361169
Salanoia concolor D378 1	Martes zibellina NC011579 39	0,18361184
Crossarchus platycephalus C7R66 1	Canis latrans NC008093 7	0,18361202
Lutra lutra LC050126 1	Civettictis civetta GLC19 1	0,183613
Mustela erminea T305 2	Bdeogale nigripes GLC15 1	0,18361318
Canis mesomelas KT448280 1	Acinonyx jubatus NC005212 3	0,18361357
Mustela nigripes NC024942 1	Diplogale hosei MH464790 1	0,1836147
Viverra zibetha T609 1	Arctocephalus australis MG023139 1	0,18361846
Viverra zibetha T609 1	Arctocephalus forsteri KT693377 17	0,18361862
Paradoxurus jerdoni MH464793 1	Melogale moschata KP726273 1	0,18361935
Bdeogale nigripes GLC15 1	Arctodus simus NC011116 1	0,18362242
Mustela eversmannii NC028013 1	Leopardus tigrinus NC028317 1	0,18362296
Viverricula indica NC025296 2	Mustela eversmannii NC028013 1	0,18362422
Vulpes lagopus NC026529 3	Lynx pardinus NC028319 161	0,18362483
Melogale moschata V0735A 1	Catopuma badia NC028300 1	0,18362561
Martes pennanti NC020664 16	Civettictis civetta GLC19 1	0,18362643
Ursus thibetanus mupinensis NC00875	Galidictis fasciata DM333 1	0,1836267
Ursus arctos GU573491 207	Mustela frenata NC020640 1	0,18362746
Galidictis fasciata DM333 1	Arctodus simus NC011116 1	0,18362804
Lynx lynx NC027083 4	Arctocephalus pusillus NC008417 1	0,18362805
Nyctereutes procyonoides NC013700 3	Leopardus jacobita NC028322 1	0,18362808
Caracal caracal NC028306 1	Canis anthus NC027956 2	0,1836282
Puma yagouaroundi NC028311 1	Nyctereutes procyonoides NC013700 3	0,18362913
Ursus arctos GU573491 207	Mustela putorius NC020638 4	0,18362941
Viverra tangalunga MH464792 1	Ursus thibetanus mupinensis NC00875	0,18363078
Mustela erminea T305 2	Crocuta crocuta NC020670 3	0,1836312
Viverricula indica NC025296 2	Martes martes T302 3	0,18363132
Prionailurus planiceps KY682741 4	Poecilogale albinucha T602 1	0,18363157
Ursus maritimus NC003428 31	Genetta servalina NC024568 2	0,18363188
Procyon lotor AB462046 3	Leptailurus serval NC028316 1	0,18363212
Prionailurus bengalensis NC028301 12	Aonyx cinerea NC035814 2	0,18363288
Ursus thibetanus laniger MH281753 2	Martes pennanti NC020664 16	0,18363313
Ursus maritimus NC003428 31	Mustela nivalis T306 5	0,18363474
Panthera onca NC022842 1	Mustela nigripes NC024942 1	0,18363498
Profelis aurata NC028299 1	Arctodus simus NC011116 1	0,18363568
Genetta abyssinica MG489822 1	Canis anthus NC027956 2	0,18363577
Ursus arctos pruinosus MG066703 3	Arctocephalus pusillus NC008417 1	0,18363616
Meles anakuma NC009677 1	Arctocephalus forsteri NC004023 28	0,18363644

Genetta genetta T297 1	Arctictis binturong T605 2	0,13172136
Melogale moschata V0735A 1	Martes flavigula NC012141 3	0,13174751
Galidia elegans D146 1	Cryptoprocta ferox CFC13 1	0,13177334
Mustela sibirica AP017394 11	Lutra sumatrana NC035810 1	0,13177739
Mustela nigripes NC024942 1	Melogale moschata KP726273 1	0,13178295
Helarctos malayanus NC009968 2	Arctodus simus NC011116 1	0,13178392
Ursus americanus JX196366 3	Arctodus simus NC011116 1	0,13179861
Mustela erminea T305 2	Meles meles T303 3	0,13184702
Melogale moschata NC020644 1	Martes pennanti NC020664 16	0,13185538
Poecilogale albinucha T602 1	Mustela frenata NC020640 1	0,13185722
Panthera tigris amoyensis NC014770 2	Homotherium latidens MF871702 3	0,131863
Mustela sibirica NC020637 6	Martes flavigula NC012141 3	0,13188124
Lontra canadensis SRR10409165 1	Aonyx cinerea NC035814 2	0,13190658
Martes zibellina NC011579 39	Lutra lutra LC050126 1	0,13190658
Melogale moschata KP726273 1	Martes pennanti NC020664 16	0,13191652
Mustela kathiah NC023210 1	Martes zibellina NC011579 39	0,13191818
Melogale moschata V0735A 1	Lutra lutra NC011358 9	0,13197389
Viverra tangalunga MH464792 1	Lutra lutra LC050126 1	0,13197911
Mustela eversmannii NC028013 1	Melogale moschata NC020644 1	0,1319861
Genetta abyssinica MG489822 1	Diplogale hosei MH464790 1	0,13199119
Mustela itatsi NC034330 19	Martes foina NC020643 1	0,13199161
Lutrogale perspicillata NC035811 1	Lontra canadensis SRR10409165 1	0,13204089
Mustela kathiah NC023210 1	Martes melampus NC009678 1	0,13204582
Martes foina NC020643 1	Lutra sumatrana NC035810 1	0,13204759
Melogale moschata KP726273 1	Aonyx cinerea NC035814 2	0,13210848
Viverra tangalunga MH464792 1	Lutra sumatrana NC035810 1	0,13211386
Mustela eversmannii NC028013 1	Martes zibellina NC011579 39	0,13212001
Mustela nigripes NC024942 1	Martes pennanti NC020664 16	0,13212316
Paradoxurus hermaphroditus NC039591 1	Genetta genetta T297 1	0,1321798
Mustela erminea T305 2	Meles anakuma NC009677 1	0,13218115
Mustela itatsi NC034330 19	Martes pennanti NC020664 16	0,13219021
Vulpes ferrilata NC027935 1	Canis adustus KT448271 1	0,13219309
Martes americana NC020642 1	Lutra lutra LC050126 1	0,13224308
Mustela itatsi NC034330 19	Lutra lutra NC011358 9	0,1323154
Martes foina NC020643 1	Enhydra lutris NC009692 1	0,13231719
Mustela putorius NC020638 4	Melogale moschata NC020644 1	0,13232239
Mustela nivalis T306 5	Martes americana NC020642 1	0,13232686
Neovison vison NC020641 3	Martes pennanti NC020664 16	0,13232824
Neovison vison NC020641 3	Martes foina NC020643 1	0,13233246
Martes flavigula NC012141 3	Arctonyx collaris NC020645 1	0,13234426
Lutra lutra LC050126 1	Gulo gulo NC009685 3	0,13234568
Ursus thibetanus formosanus NC009331 1	Arctotherium sp NC030174 1	0,13238472
Martes martes T302 3	Enhydra lutris NC009692 1	0,13238701
Lutra sumatrana NC035810 1	Ictonyx striatus T299 1	0,13239047
Mustela altaica NC021751 1	Gulo gulo NC009685 3	0,13242607
Martes melampus NC009678 1	Lontra canadensis SRR10409165 1	0,13244498
Mustela nivalis T306 5	Martes pennanti NC020664 16	0,13246696
Mustela kathiah NC023210 1	Martes flavigula NC012141 3	0,13248588
Mustela eversmannii NC028013 1	Melogale moschata KP726273 1	0,1325233
Mustela frenata NC020640 1	Martes martes T302 3	0,13252456
Neovison vison NC020641 3	Martes martes T302 3	0,13252814
Meles leucurus NC039173 4	Martes flavigula NC012141 3	0,13254637
Meles leucurus NC039173 4	Martes foina NC020643 1	0,13258635
Meles meles T303 3	Martes foina NC020643 1	0,13258649
Ursus thibetanus laniger MH281753 2	Arctotherium sp NC030174 1	0,13259166
Lutra lutra LC050126 1	Ictonyx striatus T299 1	0,1325925
Neovison vison NC020641 3	Melogale moschata V0735A 1	0,13259549
Mustela itatsi NC034330 19	Ictonyx striatus T299 1	0,1325998
Galidia elegans D146 1	Eupleres goudotii D128 1	0,13271156
Panthera tigris NC010642 35	Homotherium latidens MF871702 3	0,13271758
Mustela kathiah NC023210 1	Lutra sumatrana NC035810 1	0,13271903
Mustela itatsi NC034330 19	Martes americana NC020642 1	0,13272557
Lutra lutra NC011358 9	Ictonyx striatus T299 1	0,13272676
Mustela eversmannii NC028013 1	Martes pennanti NC020664 16	0,13272899
Lutra lutra NC011358 9	Gulo gulo NC009685 3	0,13275014
Melogale moschata V0735A 1	Lutra lutra LC050126 1	0,13278148
Ursus thibetanus mupinensis NC008753 2	Arctotherium sp NC030174 1	0,13278426
Mustela frenata NC020640 1	Lontra canadensis SRR10409165 1	0,13278617
Mustela frenata NC020640 1	Ictonyx striatus T299 1	0,13280191
Lutra sumatrana NC035810 1	Gulo gulo NC009685 3	0,13281669
Martes melampus NC009678 1	Enhydra lutris NC009692 1	0,13284878
Mustela kathiah NC023210 1	Melogale moschata NC020644 1	0,13286068
Poecilogale albinucha T602 1	Neovison vison NC020641 3	0,13287169
Melogale moschata V0735A 1	Gulo gulo NC009685 3	0,13288745
Mustela sibirica NC020637 6	Lutra lutra NC011358 9	0,13292144
Vulpes lagopus NC026529 3	Canis adustus KT448271 1	0,13292418
Martes martes T302 3	Lontra canadensis SRR10409165 1	0,13292475
Mustela nivalis T306 5	Lutra lutra NC011358 9	0,13292773
Mustela itatsi NC034330 19	Melogale moschata NC020644 1	0,13292824
Mustela putorius NC020638 4	Martes pennanti NC020664 16	0,13293079
Ursus spelaeus EU327344 13	Arctodus simus NC011116 1	0,13293335
Mustela putorius NC020638 4	Gulo gulo NC009685 3	0,13295896
Genetta abyssinica MG489822 1	Chrotogale owstoni T607 1	0,13296601
Urva semitorquata MH464789 1	Fossa fossana D350 1	0,13297872
Mustela nigripes NC024942 1	Lutra lutra NC011358 9	0,13298868
Martes foina NC020643 1	Lutra lutra NC011358 9	0,13298978
Mustela frenata NC020640 1	Martes zibellina NC011579 39	0,13299528
Mustela putorius NC020638 4	Martes foina NC020643 1	0,13313502
Taxidea taxus NC020646 1	Mustela erminea T305 2	0,13313967
Mustela kathiah NC023210 1	Gulo gulo NC009685 3	0,13316113
Genetta servalina NC024568 2	Diplogale hosei MH464790 1	0,1331832
Martes foina NC020643 1	Lutra lutra LC050126 1	0,13319179
Mustela putorius NC020638 4	Melogale moschata KP726273 1	0,13319644
Mustela nigripes NC024942 1	Ictonyx striatus T299 1	0,13320555
Mustela frenata NC020640 1	Gulo gulo NC009685 3	0,13322721
Paradoxurus hermaphroditus NLNC 1	Cynogale bennetti KY117544 1	0,13323186
Martes zibellina NC011579 39	Lontra canadensis SRR10409165 1	0,13325258
Melogale moschata NC020644 1	Gulo gulo NC009685 3	0,13329695
Martes foina NC020643 1	Aonyx cinerea NC035814 2	0,13332634
Mustela sibirica AP017394 11	Martes pennanti NC020664 16	0,13333616
Mustela nigripes NC024942 1	Gulo gulo NC009685 3	0,1333627
Neovison vison NC020641 3	Gulo gulo NC009685 3	0,13336611
Martes zibellina NC011579 39	Enhydra lutris NC009692 1	0,13338718

Genetta abyssinica MG489822 1	Arctocephalus pusillus NC008417 1	0,18363692
Viverra zibetha T609 1	Ursus arctos pruinosus MG066703 3	0,18363759
Ursus thibetanus formosanus NC0093	Galidictis fasciata DM333 1	0,18363824
Viverra tangalunga MH464792 1	Ursus thibetanus formosanus NC0093	0,18364003
Ursus spelaeus EU327344 13	Lutra sumatrana NC035810 1	0,18364075
Zalophus wollebaeki SRR4431565 1	Felis margarita NC028308 1	0,18364088
Zalophus wollebaeki SRR4431565 1	Prionailurus rubiginosus NC028304 2	0,1836412
Melursus ursinus NC009970 2	Arctonyx collaris NC020645 1	0,18364172
Ursus arctos AP012576 6	Neophoca cinerea NC008419 1	0,18364343
Martes americana NC020642 1	Leopardus colocolo NC028314 1	0,18364401
Mustela altaica NC021751 1	Helarctos malayanus NC009968 2	0,18364654
Ursus spelaeus EU327344 13	Catopuma temminckii NC027115 41	0,1836483
Ursus thibetanus laniger MH281753 2	Felis silvestris lybica KP202275 4	0,18364839
Ursus thibetanus thibetanus NC01111	Lynx pardinus NC028319 161	0,18364894
Mustela nigripes NC024942 1	Lycalopex sechurae KT448284 1	0,18364908
Ursus thibetanus thibetanus NC01111	Felis margarita NC028308 1	0,1836495
Prionailurus planiceps KY682741 4	Helarctos malayanus NC009968 2	0,18364966
Ursus spelaeus EU327344 13	Otaria byronia OTAB 1	0,18365183
Smilodon populator MF871700 1	Lycaon pictus NC028427 2	0,18365617
Civettictis civetta NC033378 1	Arctocephalus gazella BK010918 1	0,1836596
Mustela kathiah NC023210 1	Canis mesomelas KT448280 1	0,18366022
Taxidea taxus NC020646 1	Panthera leo NERO 19	0,18366088
Urocyon cinereoargenteus NC026723 2	Halichoerus grypus NC001602 2	0,18366295
Mustela itatsi NC034330 19	Civettictis civetta NC033378 1	0,18366313
Homotherium latidens MF871702 3	Ailuropoda melanoleuca NC009492 5	0,18366578
Ursus americanus JX196366 3	Mustela putorius NC020638 4	0,18366614
Ursus americanus JX196366 3	Martes martes T302 3	0,18366674
Hyaena hyaena NC020669 1	Canis adustus KT448271 1	0,18366948
Proteles cristata T393 6	Monachus monachus NC044972 5	0,18367018
Gulo gulo NC009685 3	Genetta abyssinica MG489822 1	0,1836758
Urva semitorquata MH464789 1	Callorhinus ursinus NC008415 1	0,18367633
Viverra tangalunga MH464792 1	Mungos mungo MMC7 1	0,18367766
Mungos mungo MMC7 1	Arctocephalus australis MG023139 1	0,18367887
Procyon lotor AB462049 4	Chrotogale owstoni T607 1	0,18367961
Ursus arctos pruinosus MG066703 3	Martes flavigula NC012141 3	0,18368044
Mustela nivalis T306 5	Canis mesomelas KT448280 1	0,18368137
Mungos mungo MMC7 1	Martes pennanti NC020664 16	0,18368308
Mephitis mephitis NC020648 1	Lutra lutra LC050126 1	0,18368571
Meles anakuma NC009677 1	Leopardus jacobita NC028322 1	0,18368576
Genetta servalina NC024568 2	Arctocephalus forsteri KT693377 17	0,1836862
Meles anakuma NC009677 1	Caracal caracal NC028306 1	0,18368742
Felis catus NC001700 2	Ailurus fulgens NC011124 1	0,18368761
Felis nigripes NC028309 1	Ailurus fulgens NC011124 1	0,18368779
Martes americana NC020642 1	Genetta abyssinica MG489822 1	0,18368803
Vulpes corsac NC023958 1	Phoca groenlandica NC008429 54	0,1836881
Lutra lutra LC050126 1	Canis aureus KT448274 1	0,18368821
Otocolobus manul NC028323 1	Callorhinus ursinus NC008415 1	0,18368825
Vulpes lagopus NC026529 3	Viverricula indica KX891745 1	0,18368879
Viverricula indica KX891751 1	Mustela putorius NC020638 4	0,18368919
Ursus maritimus NC003428 31	Arctocephalus townsendi NC008420 1	0,18368922
Ursus arctos isabellinus 18B5 2	Arctocephalus forsteri KT693377 17	0,18368962
Profelis aurata NC028299 1	Martes melampus NC009678 1	0,1836898
Otocolobus manul NC028323 1	Ailurus fulgens NC011124 1	0,18368992
Mustela sibirica AP017394 11	Felis nigripes NC028309 1	0,18369073
Potos flavus T414 1	Felis margarita NC028308 1	0,18369134
Prionailurus rubiginosus NC028304 2	Arctocephalus australis MG023139 1	0,18369153
Vulpes lagopus NC026529 3	Felis chaus NC028307 1	0,18369198
Melogale moschata V0735A 1	Felis silvestris lybica KP202275 4	0,18369208
Mustela putorius NC020638 4	Genetta genetta T297 1	0,18369228
Lynx lynx NC027083 4	Lontra canadensis SRR10409165 1	0,18369263
Mustela itatsi NC034330 19	Diplogale hosei MH464790 1	0,18369313
Otocolobus manul NC028323 1	Martes americana NC020642 1	0,18369336
Leopardus geoffroyi NC028320 1	Ailurus fulgens styani NC009691 1	0,18369354
Viverra zibetha T609 1	Arctotherium sp NC030174 1	0,18369411
Conepatus chinga NC042596 1	Arctocephalus pusillus NC008417 1	0,18369432
Neofelis nebulosa NC008450 3	Monachus monachus NC044972 5	0,18369474
Tremarctos ornatus NC009969 2	Leopardus guigna NC028321 1	0,18369494
Lynx canadensis NC028313 1	Arctocephalus pusillus NC008417 1	0,18369557
Salanoia concolor D378 1	Ictonyx striatus T299 1	0,18369594
Zalophus wollebaeki SRR4431565 1	Conepatus chinga NC042596 1	0,18369675
Ursus arctos pruinosus MG066703 3	Mustela putorius NC020638 4	0,1836971
Lynx lynx NC027083 4	Arctotherium sp NC030174 1	0,18369821
Viverra zibetha T609 1	Ursus arctos EU497665 29	0,18369852
Ursus arctos GU573491 207	Civettictis civetta GLC19 1	0,18369874
Smilodon populator MF871700 1	Otaria byronia OTAB 1	0,18369959
Paradoxurus hermaphroditus NLNC 1	Arctodus simus NC011116 1	0,18370028
Leptailurus serval NC028316 1	Arctotherium sp NC030174 1	0,18370049
Ursus maritimus NC003428 31	Martes martes T302 3	0,18370089
Ursus arctos isabellinus 18B5 2	Mustela nivalis T306 5	0,18370189
Ursus spelaeus NC011112 8	Melogale moschata V0735A 1	0,18370332
Ursus thibetanus mupinensis NC00875	Felis silvestris lybica KP202275 4	0,18370515
Ursus arctos AP012576 6	Enhydra lutris NC009692 1	0,18370681
Lycaon pictus NC028427 2	Felis nigripes NC028309 1	0,18370773
Homotherium latidens MF871702 3	Arctocephalus forsteri KT693377 17	0,18370834
Zalophus wollebaeki SRR4431565 1	Leopardus pardalis T262 1	0,18371132
Chrotogale owstoni T607 1	Canis lupus familiaris NC002008 1231	0,18371506
Mungos mungo MMC7 1	Martes flavigula NC012141 3	0,18371637
Lynx rufus NC014456 3	Chrysocyon brachyurus NC024172 1	0,18371689
Paradoxurus jerdoni MH464793 1	Helarctos malayanus NC009968 2	0,18371716
Hemigalus derbyanus MH464791 1	Cuon alpinus NC013445 3	0,18371937
Ursus thibetanus laniger MH281753 2	Leopardus guigna NC028321 1	0,18371968
Melogale moschata NC020644 1	Arctocephalus forsteri NC004023 28	0,1837202
Leopardus colocolo NC028314 1	Ictonyx striatus T299 1	0,18372218
Bassariscus sumichrasti SRX1099089 1	Arctictis binturong T605 2	0,18372227
Mustela nigripes NC024942 1	Canis mesomelas KT448280 1	0,18372458
Lynx canadensis NC028313 1	Canis lupus chanco NC010340 4	0,18372838
Canis lupus chanco NC010340 4	Arctonyx collaris NC020645 1	0,18372849
Viverricula indica KX891751 1	Eumetopias jubatus NC004030 10	0,18373166
Ursus americanus JX196366 3	Meles anakuma NC009677 1	0,18373221
Galerella sanguinea T378 1	Eumetopias jubatus NC004030 10	0,18373354
Mustela sibirica AP017394 11	Mungos mungo MMC7 1	0,18374369
Neophoca cinerea NC008419 1	Meles anakuma NC009677 1	0,18374377
Ictonyx striatus T299 1	Canis adustus KT448271 1	0,18374714

Melogale moschata NC020644 1	Aonyx cinerea NC035814 2	0,1333936
Mustela sibirica AP017394 11	Melogale moschata V0735A 1	0,13339858
Mustela frenata NC020640 1	Martes foina NC020643 1	0,13340488
Mustela nivalis T306 5	Melogale moschata KP726273 1	0,13347074
Mustela sibirica NC020637 6	Martes pennanti NC020664 16	0,13347076
Mustela putorius NC020638 4	Ictonyx striatus T299 1	0,13347496
Melogale moschata KP726273 1	Gulo gulo NC009685 3	0,1334926
Mustela itatsi NC034330 19	Melogale moschata KP726273 1	0,13353303
Neovison vison NC020641 3	Meles meles T303 3	0,13353457
Mustela eversmannii NC028013 1	Martes foina NC020643 1	0,1335386
Martes melampus NC009678 1	Arctonyx collaris NC020645 1	0,13358907
Mustela eversmannii NC028013 1	Lutra lutra NC011358 9	0,13359454
Vulpes zerda KJ603240 1	Canis adustus KT448271 1	0,13359468
Meles anakuma NC009677 1	Martes foina NC020643 1	0,13359585
Martes martes T302 3	Arctonyx collaris NC020645 1	0,13359624
Mustela sibirica AP017394 11	Ictonyx striatus T299 1	0,13360958
Mustela nivalis T306 5	Gulo gulo NC009685 3	0,13363804
Mustela kathiah NC023210 1	Martes americana NC020642 1	0,13366774
Ictonyx striatus T299 1	Enhydra lutris NC009692 1	0,13367
Paradoxurus hermaphroditus NC039591 1	Cynogale bennetti KY117544 1	0,13370234
Mustela putorius NC020638 4	Lutra lutra NC011358 9	0,13372895
Martes americana NC020642 1	Enhydra lutris NC009692 1	0,13379097
Martes americana NC020642 1	Lontra canadensis SRR10409165 1	0,13379097
Meles meles T303 3	Martes melampus NC009678 1	0,13379097
Galerella sanguinea T378 1	Fossa fossana D350 1	0,13379608
Mustela sibirica NC020637 6	Ictonyx striatus T299 1	0,13381153
Mungos mungo/gambianus SRR7704821 1	Fossa fossana D350 1	0,13385403
Meles leucurus NC039173 4	Martes melampus NC009678 1	0,13385826
Mustela sibirica AP017394 11	Lutra lutra NC011358 9	0,13386375
Mustela frenata NC020640 1	Arctonyx collaris NC020645 1	0,13386425
Taxidea taxus NC020646 1	Martes melampus NC009678 1	0,13387421
Martes flavigula NC012141 3	Aonyx cinerea NC035814 2	0,13390464
Viverra tangalunga MH464792 1	Lutra lutra NC011358 9	0,13393091
Mustela sibirica NC020637 6	Melogale moschata V0735A 1	0,13393697
Neovison vison NC020641 3	Meles leucurus NC039173 4	0,13393815
Viverra tangalunga MH464792 1	Martes melampus NC009678 1	0,1339804
Mustela kathiah NC023210 1	Martes foina NC020643 1	0,13401072
Melogale moschata KP726273 1	Lutrogale perspicillata NC035811 1	0,13405952
Meles leucurus NC039173 4	Martes americana NC020642 1	0,13406016
Neovison vison NC020641 3	Ictonyx striatus T299 1	0,13408539
Mustela sibirica NC020637 6	Gulo gulo NC009685 3	0,13410349
Ursus arctos isabellinus 18B5 2	Arctodus simus NC011116 1	0,13413239
Meles meles T303 3	Martes martes T302 3	0,1341349
Helarctos malayanus NC009968 2	Arctotherium sp NC030174 1	0,13413744
Mustela nigripes NC024942 1	Melogale moschata V0735A 1	0,1341389
Viverra tangalunga MH464792 1	Martes pennanti NC020664 16	0,13414204
Mustela altaica NC021751 1	Melogale moschata NC020644 1	0,13421257
Mustela eversmannii NC028013 1	Ictonyx striatus T299 1	0,13421537
Taxidea taxus NC020646 1	Martes martes T302 3	0,13421924
Mustela eversmannii NC028013 1	Gulo gulo NC009685 3	0,13423836
Martes americana NC020642 1	Aonyx cinerea NC035814 2	0,13426207
Ursus spelaeus NC011112 8	Arctodus simus NC011116 1	0,13428001
Mustela eversmannii NC028013 1	Melogale moschata V0735A 1	0,13434076
Ursus spelaeus NC011112 8	Arctotherium sp NC030174 1	0,13434395
Mustela nivalis T306 5	Melogale moschata NC020644 1	0,13434753
Martes martes T302 3	Ictonyx striatus T299 1	0,13441914
Gulo gulo NC009685 3	Enhydra lutris NC009692 1	0,13443163
Martes zibellina NC011579 39	Lutrogale perspicillata NC035811 1	0,13446392
Martes zibellina NC011579 39	Arctonyx collaris NC020645 1	0,13446397
Melursus ursinus NC009970 2	Arctodus simus NC011116 1	0,13447976
Nyctereutes procyonoides NC013700 3	Canis adustus KT448271 1	0,13451122
Ursus thibetanus laniger MH281753 2	Tremarctos ornatus NC009969 2	0,13451646
Xenogale naso C07XAR110 1	Fossa fossana D350 1	0,13452734
Mustela putorius NC020638 4	Melogale moschata V0735A 1	0,13454276
Ursus spelaeus EU327344 13	Arctotherium sp NC030174 1	0,13454629
Meles meles T303 3	Martes zibellina NC011579 39	0,13459855
Melogale moschata V0735A 1	Aonyx cinerea NC035814 2	0,13459855
Meles meles T303 3	Martes pennanti NC020664 16	0,13460831
Martes melampus NC009678 1	Ictonyx striatus T299 1	0,13461104
Mustela sibirica AP017394 11	Gulo gulo NC009685 3	0,13464235
Meles meles T303 3	Martes americana NC020642 1	0,13466586
Mustela frenata NC020640 1	Meles anakuma NC009677 1	0,13467154
Martes zibellina NC011579 39	Ictonyx striatus T299 1	0,1346783
Meles meles T303 3	Gulo gulo NC009685 3	0,13469809
Vulpes zerda KJ603240 1	Nyctereutes procyonoides NC013700 3	0,13472027
Genetta genetta T297 1	Chrotogale owstoni T607 1	0,13472949
Martes foina NC020643 1	Lontra canadensis SRR10409165 1	0,13473929
Mustela nivalis T306 5	Ictonyx striatus T299 1	0,13475996
Poecilogale albinucha T602 1	Martes flavigula NC012141 3	0,13477819
Martes americana NC020642 1	Arctonyx collaris NC020645 1	0,13480046
Martes melampus NC009678 1	Aonyx cinerea NC035814 2	0,13480046
Mustela kathiah NC023210 1	Melogale moschata V0735A 1	0,13481216
Taxidea taxus NC020646 1	Martes zibellina NC011579 39	0,13482466
Ursus arctos isabellinus 18B5 2	Arctotherium sp NC030174 1	0,13487013
Mustela itatsi NC034330 19	Lontra canadensis SRR10409165 1	0,13487262
Meles leucurus NC039173 4	Martes pennanti NC020664 16	0,13487746
Mustela kathiah NC023210 1	Martes pennanti NC020664 16	0,13488159
Mustela altaica NC021751 1	Melogale moschata KP726273 1	0,13488415
Ursus thibetanus thibetanus NC011118 4	Tremarctos ornatus NC009969 2	0,13493207
Prionailurus bengalensis NC028301 12	Nandinia binotata NC024567 1	0,13494131
Martes martes T302 3	Aonyx cinerea NC035814 2	0,13494311
Viverra tangalunga MH464792 1	Martes martes T302 3	0,13494757
Viverra tangalunga MH464792 1	Martes foina NC020643 1	0,1349531
Meles meles T303 3	Martes flavigula NC012141 3	0,13496967
Fossa fossana D350 1	Atilax paludinosus T606 1	0,13499865
Ursus arctos GU573491 207	Arctodus simus NC011116 1	0,13500774
Meles leucurus NC039173 4	Martes martes T302 3	0,13500971
Melogale moschata V0735A 1	Martes pennanti NC020664 16	0,13501239
Martes flavigula NC012141 3	Lontra canadensis SRR10409165 1	0,13504808
Poecilogale albinucha T602 1	Lutra lutra LC050137 1	0,13508137
Viverra tangalunga MH464792 1	Martes americana NC020642 1	0,13514858
Taxidea taxus NC020646 1	Martes foina NC020643 1	0,13516802
Nyctereutes procyonoides NC013700 3	Canis latrans NC008093 7	0,13519154

Mungos mungo/gambianus SRR77048	Canis anthus NC027956 2	0,18374762
Mustela putorius NC020638 4	Diplogale hosei MH464790 1	0,1837482
Tremarctos ornatus NC009969 2	Ailurus fulgens NC011124 1	0,18374845
Lutra sumatrana NC035810 1	Galerella sanguinea T378 1	0,18375221
Lutrogale perspicillata NC035811 1	Arctocephalus pusillus NC008417 1	0,18375231
Smilodon populator MF871700 1	Lutra lutra NC011358 9	0,1837528
Felis chaus NC028307 1	Enhydra lutris NC009692 1	0,18375304
Leopardus wiedii NC028318 1	Ailurus fulgens NC011124 1	0,18375368
Prionailurus planiceps KY682741 4	Arctonyx collaris NC020645 1	0,18375421
Viverricula indica KX891751 1	Melogale moschata KP726273 1	0,1837543
Leopardus geoffroyi NC028320 1	Conepatus chinga NC042596 1	0,18375468
Pardofelis marmorata NLN3 2	Mustela kathiah NC023210 1	0,18375666
Viverricula indica KX891751 1	Mustela nigripes NC024942 1	0,18375696
Speothos venaticus C48 2	Pusa sibirica NC008432 2	0,1837576
Prionodon linsang ERR2391707 1	Phoca largha NC008430 1	0,18375811
Taxidea taxus NC020646 1	Nyctereutes procyonoides NC013700 3	0,18375835
Mustela sibirica NC020637 6	Caracal caracal NC028306 1	0,18375865
Vulpes vulpes NC008434 5	Panthera uncia KP202269 1	0,18375869
Tremarctos ornatus NC009969 2	Prionailurus planiceps NC028312 6	0,18375902
Nyctereutes procyonoides NC013700 3	Civettictis civetta GLC19 1	0,18375993
Melogale moschata V0735A 1	Leptailurus serval NC028316 1	0,18376043
Lontra canadensis SRR10409165 1	Arctodus simus NC011116 1	0,1837606
Vulpes ferrilata NC027935 1	Prionailurus bengalensis NC028301 12	0,18376064
Martes americana NC020642 1	Felis chaus NC028307 1	0,18376069
Salanoia concolor D378 1	Arctodus simus NC011116 1	0,18376078
Leopardus tigrinus NC028317 1	Arctocephalus australis MG023139 1	0,18376099
Leopardus wiedii NC028318 1	Canis anthus NC027956 2	0,18376103
Martes zibellina NC011579 39	Leopardus tigrinus NC028317 1	0,18376149
Catopuma temminckii NC027115 41	Ailurus fulgens NC011124 1	0,18376164
Ursus arctos pruinosus MG066703 3	Martes americana NC020642 1	0,18376252
Taxidea taxus NC020646 1	Panthera onca KP202264 2	0,18376279
Vulpes vulpes NC008434 5	Leopardus geoffroyi NC028320 1	0,18376333
Profelis aurata NC028299 1	Canis latrans NC008093 7	0,18376334
Ursus arctos GU573491 207	Arctonyx collaris NC020645 1	0,18376344
Ursus arctos GU573486 5	Arctocephalus forsteri KT693377 17	0,18376404
Mustela nivalis T306 5	Arctotherium sp NC030174 1	0,18376428
Hemigalus derbyanus MH464791 1	Callorhinus ursinus NC008415 1	0,18376474
Hemigalus derbyanus MH464791 1	Eumetopias jubatus NC004030 10	0,1837649
Martes pennanti NC020664 16	Lynx rufus NC014456 3	0,18376555
Otaria byronia OTAB 1	Lynx pardinus NC028319 161	0,1837659
Ursus thibetanus formosanus NC00933	Lutra lutra LC050126 1	0,18376701
Galerella sanguinea T378 1	Arctodus simus NC011116 1	0,18376845
Mustela sibirica NC020637 6	Arctocephalus forsteri NC004023 28	0,18377231
Ursus thibetanus mupinensis NC00875	Puma concolor NC016470 22	0,18377335
Ictonyx striatus T299 1	Genetta servalina NC024568 2	0,18377346
Mustela sibirica AP017394 11	Melursus ursinus NC009970 2	0,18377428
Viverricula indica KX891751 1	Ailuropoda melanoleuca NC009492 5	0,18377532
Puma concolor NC016470 22	Lycaon pictus NC028427 2	0,18377547
Mustela frenata NC020640 1	Melursus ursinus NC009970 2	0,18377551
Taxidea taxus NC020646 1	Parahyaena brunnea NC038159 15	0,18377846
Lynx lynx NC027083 4	Helarctos malayanus NC009968 2	0,18378115
Galictis vittata T412 1	Arctodus simus NC011116 1	0,1837814
Ursus thibetanus thibetanus NC01111E	Genetta genetta T297 1	0,18378179
Melogale moschata KP726273 1	Arctocephalus forsteri NC004023 28	0,18378228
Ursus thibetanus formosanus NC00933	Felis margarita NC028308 1	0,18378228
Melogale moschata NC020644 1	Acinonyx jubatus NC005212 3	0,18378247
Paguma larvata PDDS11 2	Martes flavigula NC012141 3	0,18378666
Zalophus californianus NC008416 1	Lynx rufus NC014456 3	0,18378931
Martes martes T302 3	Canis lupus familiaris NC002008 1231	0,18379037
Civettictis civetta NC033378 1	Callorhinus ursinus NC008415 1	0,18379162
Lynx canadensis NC028313 1	Canis lupus familiaris NC002008 1231	0,18379357
Nyctereutes procyonoides NC013700 3	Civettictis civetta NC033378 1	0,18379702
Eumetopias jubatus NC004030 10	Bassaricyon neblina SRX1097850 1	0,18379724
Lycalopex sechurae KT448284 1	Felis nigripes NC028309 1	0,18379788
Ursus americanus JX196366 3	Mustela eversmannii NC028013 1	0,1838004
Vulpes zerda KJ603240 1	Halichoerus grypus NC001602 2	0,18380322
Leopardus geoffroyi NC028320 1	Eumetopias jubatus NC004030 10	0,18380617
Paradoxurus hermaphroditus NLNC 1	Enhydra lutris NC009692 1	0,18380849
Viverra zibetha T609 1	Lutra lutra LC050126 1	0,18381284
Mustela frenata NC020640 1	Eupleres goudotii D128 1	0,18381436
Phocarctos hookeri NC008418 1	Ailurus fulgens NC011124 1	0,18381596
Meles leucurus NC039173 4	Canis latrans NC008093 7	0,18381631
Vulpes lagopus NC026529 3	Salanoia concolor D378 1	0,18381743
Lutrogale perspicillata NC035811 1	Homotherium latidens MF871702 3	0,18381789
Lutra lutra LC050126 1	Catopuma temminckii NC027115 41	0,18381937
Martes martes T302 3	Cynictis penicillata T375 1	0,18381937
Neovison vison NC020641 3	Galerella sanguinea T378 1	0,18382365
Taxidea taxus NC020646 1	Eupleres goudotii D128 1	0,18382415
Melogale moschata KP726273 1	Genetta genetta T297 1	0,18382473
Pardofelis marmorata NLN3 2	Mustela eversmannii NC028013 1	0,18382494
Paradoxurus hermaphroditus NC03959	Mustela nigripes NC024942 1	0,18382537
Tremarctos ornatus NC009969 2	Civettictis civetta GLC19 1	0,18382569
Civettictis civetta GLC19 1	Canis anthus NC027956 2	0,18382576
Tremarctos ornatus NC009969 2	Lynx canadensis NC028313 1	0,18382595
Genetta servalina NC024568 2	Canis anthus NC027956 2	0,18382604
Vulpes corsac NC023958 1	Felis nigripes NC028309 1	0,18382648
Vulpes corsac NC023958 1	Felis silvestris lybica KP202275 4	0,18382674
Prionailurus planiceps KY682741 4	Melogale moschata V0735A 1	0,18382688
Vulpes corsac NC023958 1	Felis margarita NC028308 1	0,18382695
Melogale moschata V0735A 1	Leopardus tigrinus NC028317 1	0,18382703
Mustela itatsi NC034330 19	Genetta genetta T297 1	0,18382731
Tremarctos ornatus NC009969 2	Felis silvestris lybica KP202275 4	0,18382826
Otocolobus manul NC028323 1	Martes zibellina NC011579 39	0,18382849
Ursus arctos GU573491 207	Neophoca cinerea NC008419 1	0,1838294
Viverricula indica KX891745 1	Martes martes T302 3	0,1838294
Mirounga angustirostris SRR10331586	Galidia elegans D146 1	0,18383028
Ursus arctos GU573486 5	Arctocephalus townsendi NC008420 1	0,1838305
Viverricula indica KX891751 1	Ursus arctos isabellinus 18B5 2	0,18383281
Viverra zibetha T609 1	Ursus maritimus GU573488 Svalbard	0,18383315
Ursus arctos isabellinus 18B5 2	Civettictis civetta GLC19 1	0,18383329
Mustela altaica NC021751 1	Felis nigripes NC028309 1	0,18383341
Ursus maritimus NC003428 31	Civettictis civetta GLC19 1	0,18383358
Prionailurus bengalensis CKM45 20	Phocarctos hookeri NC008418 1	0,18383402

Mustela kathiah NC023210 1	Arctonyx collaris NC020645 1	0,13521031
Mustela kathiah NC023210 1	Meles leucurus NC039173 4	0,13521215
Poecilogale albinucha T602 1	Mustela kathiah NC023210 1	0,13522188
Mustela altaica NC021751 1	Ictonyx striatus T299 1	0,135231
Mustela itatsi NC034330 19	Gulo gulo NC009685 3	0,13524754
Ursus thibetanus formosanus NC009331 1	Tremarctos ornatus NC009969 2	0,1352638
Gulo gulo NC009685 3	Arctonyx collaris NC020645 1	0,13530296
Taxidea taxus NC020646 1	Martes flavigula NC012141 3	0,13532342
Vulpes lagopus NC026529 3	Nyctereutes procyonoides NC013700 3	0,13539353
Fossa fossana D350 1	Crossarchus platycephalus C7R66 1	0,13540263
Martes zibellina NC011579 39	Aonyx cinerea NC035814 2	0,13540615
Meles leucurus NC039173 4	Martes zibellina NC011579 39	0,13540615
Prionailurus bengalensis CKM45 20	Nandinia binotata NC024567 1	0,13541621
Poecilogale albinucha T602 1	Mustela nigripes NC024942 1	0,1354242
Viverra tangalunga MH464792 1	Ictonyx striatus T299 1	0,13542733
Viverra tangalunga MH464792 1	Martes flavigula NC012141 3	0,13544641
Viverra tangalunga MH464792 1	Martes zibellina NC011579 39	0,13548553
Ursus americanus JX196366 3	Arctotherium sp NC030174 1	0,13550007
Vulpes vulpes NC008434 5	Canis aureus KT448274 1	0,13550073
Martes americana NC020642 1	Lutrogale perspicillata NC035811 1	0,13554056
Melogale moschata KP726273 1	Lontra canadensis SRR10409165 1	0,13560805
Neovison vison NC020641 3	Meles anakuma NC009677 1	0,13561836
Mustela nigripes NC024942 1	Arctonyx collaris NC020645 1	0,13568184
Mustela frenata NC020640 1	Meles meles T303 3	0,13568288
Mustela frenata NC020640 1	Aonyx cinerea NC035814 2	0,13568802
Lontra canadensis SRR10409165 1	Gulo gulo NC009685 3	0,13571578
Martes melampus NC009678 1	Lutrogale perspicillata NC035811 1	0,13574247
Mustela nigripes NC024942 1	Lontra canadensis SRR10409165 1	0,13574773
Martes americana NC020642 1	Ictonyx striatus T299 1	0,13575539
Vulpes corsac NC023958 1	Canis lupus familiaris NC002008 1231	0,13575663
Vulpes corsac NC023958 1	Canis aureus KT448274 1	0,13577823
Meles anakuma NC009677 1	Martes flavigula NC012141 3	0,13577826
Martes flavigula NC012141 3	Lutrogale perspicillata NC035811 1	0,13579077
Taxidea taxus NC020646 1	Gulo gulo NC009685 3	0,13579646
Meles anakuma NC009677 1	Martes americana NC020642 1	0,13580994
Martes foina NC020643 1	Lutrogale perspicillata NC035811 1	0,13581601
Vulpes vulpes NC008434 5	Canis lupus familiaris NC002008 1231	0,13582353
Ursus maritimus GU573488 Svalbard	Arctotherium sp NC030174 1	0,13588031
Mustela sibirica NC020637 6	Meles meles T303 3	0,13588408
Lutra lutra LC050126 1	Arctonyx collaris NC020645 1	0,13594455
Meles anakuma NC009677 1	Martes melampus NC009678 1	0,13594455
Meles meles T303 3	Lutra sumatrana NC035810 1	0,13594455
Mustela putorius NC020638 4	Lontra canadensis SRR10409165 1	0,13594963
Mustela sibirica NC020637 6	Lontra canadensis SRR10409165 1	0,13601688
Ursus arctos EU497665 29	Arctodus simus NC011116 1	0,13601777
Ursus maritimus GU573488 Svalbard	Arctodus simus NC011116 1	0,13601777
Melogale moschata NC020644 1	Meles leucurus NC039173 4	0,13601899
Poecilogale albinucha T602 1	Enhydra lutris NC009692 1	0,1360234
Poecilogale albinucha T602 1	Lutra lutra NC011358 9	0,1360237
Vulpes ferrilata NC027935 1	Canis lupus familiaris NC002008 1231	0,13602592
Melogale moschata NC020644 1	Ictonyx striatus T299 1	0,13603204
Taxidea taxus NC020646 1	Martes americana NC020642 1	0,13603741
Ursus thibetanus mupinensis NC008753 2	Tremarctos ornatus NC009969 2	0,13606757
Galidictis fasciata DM333 1	Crossarchus platycephalus C7R66 1	0,13607852
Melogale moschata NC020644 1	Lutrogale perspicillata NC035811 1	0,13608463
Mustela itatsi NC034330 19	Meles leucurus NC039173 4	0,13608702
Ursus arctos GU573486 5	Arctotherium sp NC030174 1	0,13608782
Ursus arctos GU573486 5	Arctodus simus NC011116 1	0,13609089
Mustela altaica NC021751 1	Aonyx cinerea NC035814 2	0,13609684
Mustela kathiah NC023210 1	Ictonyx striatus T299 1	0,13610005
Otocyon megalotis SAF1 2	Canis latrans NC008093 7	0,13614045
Nandinia binotata NC024567 1	Catopuma badia NC028300 1	0,13615158
Mustela eversmannii NC028013 1	Lontra canadensis SRR10409165 1	0,13615172
Poecilogale albinucha T602 1	Mustela altaica NC021751 1	0,13617051
Paguma larvata PDDS11 2	Cynogale bennetti KY117544 1	0,13619801
Meles anakuma NC009677 1	Martes zibellina NC011579 39	0,13621375
Ursus maritimus NC003428 31	Arctotherium sp NC030174 1	0,13621698
Mustela itatsi NC034330 19	Melogale moschata V0735A 1	0,13622557
Poecilogale albinucha T602 1	Lutra sumatrana NC035810 1	0,13622564
Mustela sibirica NC020637 6	Aonyx cinerea NC035814 2	0,136226
Ursus arctos EU497665 29	Arctotherium sp NC030174 1	0,1362844
Melogale moschata NC020644 1	Meles anakuma NC009677 1	0,13628815
Meles anakuma NC009677 1	Martes martes T302 3	0,13628855
Mustela putorius NC020638 4	Aonyx cinerea NC035814 2	0,13629305
Mustela nivalis T306 5	Aonyx cinerea NC035814 2	0,13629866
Martes foina NC020643 1	Ictonyx striatus T299 1	0,13630426
Vulpes vulpes NC008434 5	Canis latrans NC008093 7	0,13633609
Vulpes zerda KJ603240 1	Canis latrans NC008093 7	0,13633609
Martes martes T302 3	Lutrogale perspicillata NC035811 1	0,13635615
Ictonyx striatus T299 1	Arctonyx collaris NC020645 1	0,13636389
Urva brachyura KY117547 1	Fossa fossana D350 1	0,13641261
Lutra lutra NC011358 9	Arctonyx collaris NC020645 1	0,13641565
Mustela sibirica AP017394 11	Meles meles T303 3	0,13642254
Viverricula indica KX891745 1	Prionailurus bengalensis NC028301 12	0,13642465
Poecilogale albinucha T602 1	Mustela sibirica NC020637 6	0,13643372
Vulpes lagopus NC026529 3	Otocyon megalotis SAF1 2	0,13647892
Mungos mungo MMC7 1	Fossa fossana D350 1	0,1364826
Ursus maritimus NC003428 31	Arctodus simus NC011116 1	0,13648912
Mustela nivalis T306 5	Melogale moschata V0735A 1	0,13649999
Otocyon megalotis SAF1 2	Canis adustus KT448271 1	0,13652189
Lutra sumatrana NC035810 1	Arctonyx collaris NC020645 1	0,13655023
Melogale moschata KP726273 1	Arctonyx collaris NC020645 1	0,13655023
Mustela sibirica AP017394 11	Lontra canadensis SRR10409165 1	0,13655534
Mustela itatsi NC034330 19	Arctonyx collaris NC020645 1	0,13655666
Melogale moschata NC020644 1	Arctonyx collaris NC020645 1	0,13655764
Mustela eversmannii NC028013 1	Aonyx cinerea NC035814 2	0,13656223
Ursus arctos pruinosus MG066703 3	Arctodus simus NC011116 1	0,13656498
Taxidea taxus NC020646 1	Lutra sumatrana NC035810 1	0,13656858
Vulpes lagopus NC026529 3	Canis aureus KT448274 1	0,13658413
Vulpes corsac NC023958 1	Canis latrans NC008093 7	0,1366054
Urocyon cinereoargenteus NC026723 21	Canis adustus KT448271 1	0,13661675
Ursus arctos GU573491 207	Arctotherium sp NC030174 1	0,13662091
Nyctereutes procyonoides NC013700 3	Canis mesomelas KT448280 1	0,13662739

Ictonyx striatus T299 1	Galidictis fasciata DM333 1	0,18383595
Ursus arctos GU573491 207	Poecilogale albinucha T602 1	0,18383726
Zalophus californianus NC008416 1	Chrotogale owstoni T607 1	0,18383755
Mustela sibirica AP017394 11	Arctictis binturong T605 2	0,18383886
Ursus thibetanus mupinensis NC00875	Leopardus jacobita NC028322 1	0,18384007
Enhydra lutris NC009692 1	Arctocephalus forsteri NC004023 28	0,1838402
Ursus thibetanus mupinensis NC00875	Poecilogale albinucha T602 1	0,18384106
Ursus thibetanus thibetanus NC01111	Galidictis fasciata DM333 1	0,18384194
Mustela putorius NC020638 4	Arctocephalus forsteri NC004023 28	0,1838426
Zalophus wollebaeki SRR4431565 1	Leopardus pardalis NC028315 1	0,18384425
Ursus thibetanus mupinensis NC00875	Prionailurus bengalensis CKM45 20	0,18384472
Mustela itatsi NC034330 19	Helarctos malayanus NC009968 2	0,18384531
Phocarctos hookeri NC008418 1	Acinonyx jubatus NC005212 3	0,18384618
Viverra zibetha T609 1	Ursus arctos AP012576 6	0,18384714
Nyctereutes procyonoides NC013700 3	Leopardus colocolo NC028314 1	0,18384872
Meles anakuma NC009677 1	Urva javanica/auropunctata NC006835	0,18384932
Canis lupus familiaris NC002008 1231	Arctonyx collaris NC020645 1	0,18385102
Zalophus wollebaeki SRR4431565 1	Ursus arctos AP012576 6	0,1838516
Prionailurus planiceps NC028312 6	Chrysocyon brachyurus NC024172 1	0,18385237
Ursus thibetanus formosanus NC00933	Leopardus guigna NC028321 1	0,18385343
Urva javanica/auropunctata NC006835	Bassariscus sumichrasti SRX1099089 1	0,18385643
Prionailurus planiceps NC028312 6	Canis lupus familiaris NC002008 1231	0,18386132
Eumetopias jubatus NC004030 10	Conepatus chinga NC042596 1	0,18386377
Gulo gulo NC009685 3	Felis catus NC001700 2	0,18386824
Martes pennanti NC020664 16	Canis lupus chanco NC010340 4	0,18386908
Gulo gulo NC009685 3	Felis chaus NC028307 1	0,18386926
Enhydra lutris NC009692 1	Arctocephalus australis MG023139 1	0,18387483
Mustela putorius NC020638 4	Chrotogale owstoni T607 1	0,1838765
Meles meles T303 3	Atilax paludinosus T606 1	0,18387757
Viverricula indica KX891751 1	Lutra lutra LC050126 1	0,18387817
Melogale moschata V0735A 1	Urva semitorquata MH464789 1	0,18387829
Lutra sumatrana NC035810 1	Galidictis fasciata DM333 1	0,18388048
Pusa caspica NC008431 1	Prionodon linsang ERR2391707 1	0,18388095
Mustela kathiah NC023210 1	Cynictis penicillata T375 1	0,18388107
Mephitis mephitis NC020648 1	Canis adustus KT448271 1	0,18388262
Mustela altaica NC021751 1	Chrotogale owstoni T607 1	0,18388273
Cynictis penicillata T375 1	Canis latrans NC008093 7	0,1838828
Ursus thibetanus formosanus NC00933	Canis adustus KT448271 1	0,18388285
Civettictis civetta NC033378 1	Chrysocyon brachyurus NC024172 1	0,18388312
Procyon lotor AB462049 4	Arctocephalus gazella BK010918 1	0,18388316
Vulpes ferrilata NC027935 1	Mustela frenata NC020640 1	0,18388402
Mustela eversmannii NC028013 1	Canis latrans NC008093 7	0,18388566
Eupleres goudotii D128 1	Arctotherium sp NC030174 1	0,1838868
Lutra lutra NC011358 9	Felis margarita NC028308 1	0,18388775
Fossa fossana D350 1	Arctotherium sp NC030174 1	0,18388815
Prionodon pardicolor NC024569 2	Martes melampus NC009678 1	0,1838882
Martes melampus NC009678 1	Lycaon pictus NC028427 2	0,18388888
Cynictis penicillata T375 1	Canis aureus KT448274 1	0,18388893
Prionodon pardicolor NC024569 2	Mustela frenata NC020640 1	0,1838918
Paradoxurus hermaphroditus NC03959	Mustela putorius NC020638 4	0,18389224
Mustela eversmannii NC028013 1	Felis catus NC001700 2	0,18389271
Ursus arctos GU573491 207	Enhydra lutris NC009692 1	0,18389286
Martes foina NC020643 1	Eupleres goudotii D128 1	0,18389361
Vulpes corsac NC023958 1	Felis chaus NC028307 1	0,18389387
Vulpes lagopus NC026529 3	Felis silvestris lybica KP202275 4	0,18389413
Martes foina NC020643 1	Canis latrans NC008093 7	0,18389463
Mustela altaica NC021751 1	Arctocephalus pusillus NC008417 1	0,18389481
Prionailurus bengalensis NC028301 12	Arctocephalus gazella BK010918 1	0,18389492
Prionodon pardicolor NC024569 2	Mustela sibirica NC020637 6	0,18389492
Galerella sanguinea T378 1	Canis aureus KT448274 1	0,18389544
Nyctereutes procyonoides NC013700 3	Lynx pardinus NC028319 161	0,18389656
Mustela putorius NC020638 4	Lycaon pictus NC028427 2	0,18389703
Mustela itatsi NC034330 19	Lycaon pictus NC028427 2	0,18389794
Paguma larvata PDDS11 2	Arctotherium sp NC030174 1	0,18389837
Poecilogale albinucha T602 1	Felis silvestris lybica KP202275 4	0,18390022
Leopardus geoffroyi NC028320 1	Canis latrans NC008093 7	0,18390065
Mustela eversmannii NC028013 1	Chrysocyon brachyurus NC024172 1	0,18390203
Leopardus guigna NC028321 1	Arctocephalus pusillus NC008417 1	0,1839021
Leptailurus serval NC028316 1	Aonyx cinerea NC035814 2	0,18390225
Mustela sibirica NC020637 6	Chrysocyon brachyurus NC024172 1	0,1839027
Zalophus wollebaeki SRR4431565 1	Lutrogale perspicillata NC035811 1	0,18390299
Zalophus wollebaeki SRR4431565 1	Prionailurus planiceps KY682741 4	0,18391018
Smilodon populator MF871700 1	Mustela putorius NC020638 4	0,1839136
Ursus spelaeus NC011112 8	Genetta servalina NC024568 2	0,18391456
Zalophus wollebaeki SRR4431565 1	Prionailurus bengalensis CKM45 20	0,18391486
Ursus spelaeus EU327344 13	Bassariscus sumichrasti SRX1099089 1	0,18391524
Meles leucurus NC039173 4	Urva javanica/auropunctata NC006835	0,18391682
Ursus thibetanus thibetanus NC01111	Lynx lynx NC027083 4	0,18391715
Ursus spelaeus NC011112 8	Leopardus pardalis T262 1	0,1839186
Poecilogale albinucha T602 1	Leopardus colocolo NC028314 1	0,18392004
Ursus spelaeus NC011112 8	Profelis aurata NC028299 1	0,1839202
Mephitis mephitis NC020648 1	Martes foina NC020643 1	0,18392031
Prionailurus rubiginosus NC028304 2	Helarctos malayanus NC009968 2	0,18392038
Vulpes ferrilata NC027935 1	Hemigalus derbyanus MH464791 1	0,18392418
Zalophus californianus NC008416 1	Puma yagouaroundi NC028311 1	0,18392563
Neovison vison NC020641 3	Ailuropoda melanoleuca NC009492 5	0,18392633
Prionailurus bengalensis CKM45 20	Chrysocyon brachyurus NC024172 1	0,183927
Otocyon megalotis SAF1 2	Monachus monachus NC044972 5	0,18392803
Speothos venaticus C48 2	Monachus monachus NC044972 5	0,18393047
Taxidea taxus NC020646 1	Canis lupus chanco NC010340 4	0,18393441
Viverricula indica NC025296 2	Ursus americanus JX196366 3	0,18393643
Vulpes corsac NC023958 1	Halichoerus grypus NC001602 2	0,18393701
Genetta abyssinica MG489822 1	Eumetopias jubatus NC004030 10	0,18393962
Mungos mungo MMC7 1	Lutra lutra LC050126 1	0,18394022
Prionailurus viverrinus NC028305 1	Eumetopias jubatus NC004030 10	0,18394296
Mustela itatsi NC034330 19	Arctocephalus forsteri KT693377 17	0,18394552
Meles anakuma NC009677 1	Urva javanica T413 1	0,18394573
Phocarctos hookeri NC008418 1	Mustela putorius NC020638 4	0,18394788
Potos flavus T414 1	Cynictis penicillata T375 1	0,18394865
Spilogale putorius NC010497 1	Ictonyx striatus T299 1	0,18394914
Urva semitorquata MH464789 1	Canis latrans NC008093 7	0,18394929
Vulpes lagopus NC026529 3	Fossa fossana D350 1	0,18394952
Vulpes corsac NC023958 1	Nandinia binotata NC024567 1	0,18394953

Mustela sibirica AP017394 11	Aonyx cinerea NC035814 2	0,13662961
Martes flavigula NC012141 3	Ictonyx striatus T299 1	0,13667524
Melogale moschata V0735A 1	Lutrogale perspicillata NC035811 1	0,13668418
Mustela sibirica NC020637 6	Meles leucurus NC039173 4	0,13669224
Vulpes zerda KJ603240 1	Canis aureus KT448274 1	0,13669686
Poecilogale albinucha T602 1	Mustela sibirica AP017394 11	0,13670279
Enhydra lutris NC009692 1	Arctonyx collaris NC020645 1	0,13675214
Meles leucurus NC039173 4	Lutra sumatrana NC035810 1	0,13675214
Mustela nigripes NC024942 1	Meles meles T303 3	0,13675921
Mustela itatsi NC034330 19	Meles meles T303 3	0,13675953
Mustela nigripes NC024942 1	Meles leucurus NC039173 4	0,13675971
Mustela nivalis T306 5	Lontra canadensis SRR10409165 1	0,13676293
Ursus arctos AP012576 6	Arctodus simus NC011116 1	0,136765
Taxidea taxus NC020646 1	Martes pennanti NC020664 16	0,13676673
Viverra tangalunga MH464792 1	Poecilogale albinucha T602 1	0,13677077
Poecilogale albinucha T602 1	Mustela itatsi NC034330 19	0,13683763
Tremarctos ornatus NC009969 2	Helarctos malayanus NC009968 2	0,13687734
Nandinia binotata NC024567 1	Felis silvestris lybica KP202275 4	0,13689408
Mustela nigripes NC024942 1	Aonyx cinerea NC035814 2	0,13689893
Viverra zibetha T609 1	Catopuma badia NC028300 1	0,13690002
Martes pennanti NC020664 16	Ictonyx striatus T299 1	0,13691074
Urocyon littoralis catalinae KP129018 15	Canis adustus KT448271 1	0,13693956
Mustela sibirica AP017394 11	Arctonyx collaris NC020645 1	0,13696063
Mustela sibirica AP017394 11	Meles leucurus NC039173 4	0,13696145
Poecilogale albinucha T602 1	Mustela putorius NC020638 4	0,13697219
Salanoia concolor D378 1	Crossarchus platycephalus C7R66 1	0,13702086
Meles meles T303 3	Lutra lutra NC011358 9	0,13702133
Ursus arctos pruinosus MG066703 3	Arctotherium sp NC030174 1	0,13703083
Mustela kathiah NC023210 1	Aonyx cinerea NC035814 2	0,13703389
Viverra zibetha T609 1	Prionailurus bengalensis CKM45 20	0,13703421
Vulpes lagopus NC026529 3	Canis latrans NC008093 7	0,13707669
Tremarctos ornatus NC009969 2	Melursus ursinus NC009970 2	0,13708828
Mustela putorius NC020638 4	Meles leucurus NC039173 4	0,13709617
Poecilogale albinucha T602 1	Arctonyx collaris NC020645 1	0,13709891
Ursus arctos AP012576 6	Arctotherium sp NC030174 1	0,13710232
Nyctereutes procyonoides NC013700 3	Canis aureus KT448274 1	0,13711531
Vulpes ferrilata NC027935 1	Canis latrans NC008093 7	0,13714401
Vulpes vulpes NC008434 5	Nyctereutes procyonoides NC013700 3	0,13714401
Fossa fossana D350 1	Cynictis penicillata T375 1	0,13715324
Melogale moschata KP726273 1	Meles leucurus NC039173 4	0,13715594
Viverra tangalunga MH464792 1	Melogale moschata NC020644 1	0,13716854
Genetta genetta T297 1	Diplogale hosei MH464790 1	0,13717189
Mustela nivalis T306 5	Lutrogale perspicillata NC035811 1	0,13717356
Mustela sibirica NC020637 6	Arctonyx collaris NC020645 1	0,13722976
Ichneumia albicauda T603 1	Fossa fossana D350 1	0,13723078
Ictonyx striatus T299 1	Aonyx cinerea NC035814 2	0,13723585
Mustela altaica NC021751 1	Lontra canadensis SRR10409165 1	0,13730121
Viverra tangalunga MH464792 1	Aonyx cinerea NC035814 2	0,13730311
Prionailurus rubiginosus NC028304 2	Nandinia binotata NC024567 1	0,13736521
Vulpes zerda KJ603240 1	Canis lupus familiaris NC002008 1231	0,13737629
Nandinia binotata NC024567 1	Lynx lynx NC027083 4	0,13743198
Melogale moschata NC020644 1	Meles meles T303 3	0,13743249
Viverra tangalunga MH464792 1	Melogale moschata KP726273 1	0,13743691
Viverricula indica NC025296 2	Prionailurus bengalensis NC028301 12	0,13743792
Urva semitorquata MH464789 1	Galidictis fasciata DM333 1	0,13749282
Vulpes ferrilata NC027935 1	Canis aureus KT448274 1	0,13752396
Meles leucurus NC039173 4	Gulo gulo NC009685 3	0,13752571
Viverra zibetha T609 1	Cynogale bennetti KY117544 1	0,13754736
Fossa fossana D350 1	Bdeogale nigripes GLC15 1	0,13755724
Leopardus colocolo NC028314 1	Civettictis civetta GLC19 1	0,13758628
Helogale parvula SRR7637809 1	Fossa fossana D350 1	0,13762456
Meles meles T303 3	Lutra lutra LC050126 1	0,13762702
Melogale moschata KP726273 1	Meles meles T303 3	0,13762702
Mustela putorius NC020638 4	Arctonyx collaris NC020645 1	0,13763358
Mustela kathiah NC023210 1	Meles meles T303 3	0,13763449
Civettictis civetta GLC19 1	Catopuma badia NC028300 1	0,13763981
Vulpes lagopus NC026529 3	Canis lupus familiaris NC002008 1231	0,13764131
Taxidea taxus NC020646 1	Lutra lutra LC050126 1	0,13764514
Lontra canadensis SRR10409165 1	Ictonyx striatus T299 1	0,13764893
Poecilogale albinucha T602 1	Mustela nivalis T306 5	0,13765147
Melogale moschata KP726273 1	Meles anakuma NC009677 1	0,13769433
Martes pennanti NC020664 16	Arctonyx collaris NC020645 1	0,13770416
Viverra zibetha T609 1	Prionailurus rubiginosus NC028304 2	0,13770434
Martes pennanti NC020664 16	Aonyx cinerea NC035814 2	0,13770546
Poecilogale albinucha T602 1	Mustela eversmannii NC028013 1	0,13771249
Melogale moschata V0735A 1	Meles leucurus NC039173 4	0,13776162
Mustela itatsi NC034330 19	Meles anakuma NC009677 1	0,13776776
Mustela eversmannii NC028013 1	Arctonyx collaris NC020645 1	0,13776839
Mustela eversmannii NC028013 1	Meles leucurus NC039173 4	0,13776913
Viverra tangalunga MH464792 1	Gulo gulo NC009685 3	0,13780534
Nyctereutes procyonoides NC013700 3	Canis lupus familiaris NC002008 1231	0,13784593
Vulpes vulpes NC008434 5	Canis lupus chanco NC010340 4	0,13785121
Poecilogale albinucha T602 1	Martes martes T302 3	0,1378521
Ursus arctos isabellinus 18B5 2	Tremarctos ornatus NC009969 2	0,13789116
Melogale moschata V0735A 1	Arctonyx collaris NC020645 1	0,13789622
Salanoia concolor D378 1	Urva semitorquata MH464789 1	0,1378964
Viverra zibetha T609 1	Prionailurus viverrinus NC028305 1	0,13790634
Taxidea taxus NC020646 1	Enhydra lutris NC009692 1	0,13791415
Lobodon carcinophaga NC008423 1	Eumetopias jubatus NC004030 10	0,13793579
Ictonyx striatus T299 1	Gulo gulo NC009685 3	0,13794485
Suricata suricatta SSM10 1	Fossa fossana D350 1	0,13796122
Melogale moschata V0735A 1	Meles anakuma NC009677 1	0,13796352
Mustela kathiah NC023210 1	Meles anakuma NC009677 1	0,13796929
Mustela sibirica NC020637 6	Meles anakuma NC009677 1	0,1379697
Cryptoprocta ferox CFC13 1	Crossarchus platycephalus C7R66 1	0,13796997
Nandinia binotata NC024567 1	Felis margarita NC028308 1	0,13797162
Meles anakuma NC009677 1	Martes pennanti NC020664 16	0,1379737
Mustela itatsi NC034330 19	Aonyx cinerea NC035814 2	0,13797617
Mustela nivalis T306 5	Meles leucurus NC039173 4	0,13797709
Mungotictis decemlineata NC027828 1	Crossarchus platycephalus C7R66 1	0,13803066
Salanoia concolor D378 1	Xenogale naso C07XAR110 1	0,13803151
Nandinia binotata NC024567 1	Leopardus pardalis T262 1	0,13804035
Viverra zibetha T609 1	Prionailurus bengalensis NC028301 12	0,13804089

Nyctereutes procyonoides NC013700 3	Meles meles T303 3	0,18395033
Nasua nasua NC020647 1	Mustela nivalis T306 5	0,18395118
Mustela sibirica AP017394 11	Diplogale hosei MH464790 1	0,18395254
Ichneumia albicauda T603 1	Canis latrans NC008093 7	0,18395276
Ursus arctos pruinosus MG066703 3	Taxidea taxus NC020646 1	0,18395574
Tremarctos ornatus NC009969 2	Genetta servalina NC024568 2	0,18395665
Salanoia concolor D378 1	Martes pennanti NC020664 16	0,18395704
Ommatophoca rossii AY377287etc 1	Xenogale naso C07XAR110 1	0,18395722
Ursus maritimus GU573488 Svalbard	Arctocephalus townsendi NC008420 1	0,18395858
Mungos mungo/gambianus SRR77048	Martes foina NC020643 1	0,18395969
Ursus thibetanus mupinensis NC00875	Melogale moschata V0735A 1	0,18395998
Mustela nigripes NC024942 1	Felis chaus NC028307 1	0,18396127
Vulpes ferrilata NC027935 1	Phoca largha NC008430 1	0,18396175
Tremarctos ornatus NC009969 2	Leopardus tigrinus NC028317 1	0,18396187
Prionodon pardicolor NC024569 2	Mustela nigripes NC024942 1	0,1839626
Leopardus geoffroyi NC028320 1	Arctocephalus forsteri KT693377 17	0,18396373
Vulpes vulpes NC008434 5	Catopuma temminckii NC027115 41	0,18396413
Prionailurus planiceps KY682741 4	Nyctereutes procyonoides NC013700 3	0,18396452
Urocyon cinereoargenteus NC026723 2	Mirounga angustirostris SRR10331586	0,18396494
Lynx pardinus NC028319 161	Aonyx cinerea NC035814 2	0,18396619
Viverra zibetha T609 1	Ursus maritimus NC003428 31	0,18396786
Ursus thibetanus mupinensis NC00875	Otaria byronia OTAB 1	0,18396938
Speothos venaticus C48 2	Phoca groenlandica NC008429 54	0,18396978
Taxidea taxus NC020646 1	Lycaon pictus NC028427 2	0,18396987
Panthera uncia NC010638 1	Mirounga angustirostris SRR10331586	0,18397008
Lutra lutra LC050126 1	Helarctos malayanus NC009968 2	0,18397038
Smilodon populator MF871700 1	Melursus ursinus NC009970 2	0,18397042
Mephitis mephitis NC020648 1	Homotherium latidens MF871702 3	0,18397157
Smilodon populator MF871700 1	Mustela sibirica AP017394 11	0,18397179
Mustela nigripes NC024942 1	Arctictis binturong T605 2	0,18397419
Panthera tigris NC010642 35	Ommatophoca rossii AY377287etc 1	0,18397497
Mustela sibirica NC020637 6	Helarctos malayanus NC009968 2	0,1839765
Zalophus wollebaeki SRR4431565 1	Lynx canadensis NC028313 1	0,18397653
Mustela itatsi NC034330 19	Arctocephalus forsteri NC004023 28	0,18397672
Arctocephalus gazella BK010918 1	Acinonyx jubatus NC005212 3	0,18397807
Panthera onca NC022842 1	Melogale moschata NC020644 1	0,18397978
Ursus thibetanus thibetanus NC01111	Aonyx cinerea NC035814 2	0,18398072
Neophoca cinerea NC008419 1	Acinonyx jubatus NC005212 3	0,18398114
Viverricula indica NC025296 2	Ursus spelaeus EU327344 13	0,1839837
Ursus spelaeus NC011112 8	Lynx lynx NC027083 4	0,18398428
Ursus thibetanus thibetanus NC01111	Paguma larvata PDDS11 2	0,18398435
Ursus spelaeus EU327344 13	Puma yagouaroundi NC028311 1	0,18398497
Martes pennanti NC020664 16	Leopardus colocolo NC028314 1	0,18398653
Urocyon littoralis catalinae KP129018	Monachus monachus NC044972 5	0,18398985
Zalophus californianus NC008416 1	Civettictis civetta GLC19 1	0,18399192
Zalophus californianus NC008416 1	Prionailurus planiceps NC028312 6	0,18399213
Leptailurus serval NC028316 1	Gulo gulo NC009685 3	0,18400167
Ursus americanus JX196366 3	Mustela itatsi NC034330 19	0,18400295
Martes pennanti NC020664 16	Civettictis civetta NC033378 1	0,184003
Panthera pardus NC010641 5	Mustela erminea T305 2	0,1840038
Lycalopex sechurae KT448284 1	Felis chaus NC028307 1	0,18400384
Otocyon megalotis SAF1 2	Homotherium latidens MF871702 3	0,18400723
Chrotogale owstoni T607 1	Arctocephalus gazella BK010918 1	0,18400736
Viverricula indica KX891751 1	Tapirus terrestris T358	0,18400805
Prionailurus rubiginosus NC028304 2	Tapirus terrestris T358	0,18400949
Urva brachyura KY117547 1	Callorhinus ursinus NC008415 1	0,18401153
Callorhinus ursinus NC008415 1	Bdeogale nigripes GLC15 1	0,18401241
Meles meles T303 3	Urva brachyura KY117547 1	0,18401301
Leptailurus serval NC028316 1	Tapirus terrestris T358	0,1840148
Mustela nivalis T306 5	Diplogale hosei MH464790 1	0,18401505
Mustela frenata NC020640 1	Bdeogale nigripes GLC15 1	0,18401565
Mustela itatsi NC034330 19	Urva javanica T413 1	0,18401597
Vulpes lagopus NC026529 3	Crossarchus platycephalus C7R66 1	0,18401684
Xenogale naso C07XAR110 1	Bassariscus sumichrasti SRX1099089 1	0,18401927
Catopuma badia NC028300 1	Tapirus terrestris T358	0,18402037
Mustela frenata NC020640 1	Mungotictis decemlineata NC027828 1	0,18402253
Leopardus tigrinus NC028317 1	Ailurus fulgens NC011124 1	0,18402311
Viverra zibetha T609 1	Arctocephalus gazella BK010918 1	0,1840236
Martes americana NC020642 1	Genetta servalina NC024568 2	0,18402386
Tremarctos ornatus NC009969 2	Galerella sanguinea T378 1	0,18402502
Ursus arctos isabellinus 18B5 2	Arctocephalus australis MG023139 1	0,18402626
Ursus maritimus GU573488 Svalbard	Arctocephalus forsteri KT693377 17	0,18402655
Potos flavus T414 1	Felis chaus NC028307 1	0,18402763
Vulpes lagopus NC026529 3	Felis nigripes NC028309 1	0,18402861
Ictonyx striatus T299 1	Arctocephalus townsendi NC008420 1	0,18402864
Ictonyx striatus T299 1	Chrotogale owstoni T607 1	0,18402889
Viverricula indica KX891745 1	Martes pennanti NC020664 16	0,18402895
Vulpes lagopus NC026529 3	Felis margarita NC028308 1	0,18402909
Viverra zibetha T609 1	Nyctereutes procyonoides NC013700 3	0,18402965
Lynx pardinus NC028319 161	Lontra canadensis SRR10409165 1	0,18403007
Phocarctos hookeri NC008418 1	Lynx canadensis NC028313 1	0,18403022
Felis margarita NC028308 1	Canis anthus NC027956 2	0,1840314
Ursus arctos pruinosus MG066703 3	Mustela frenata NC020640 1	0,18403167
Genetta servalina NC024568 2	Arctocephalus pusillus NC008417 1	0,18403199
Mustela altaica NC021751 1	Genetta servalina NC024568 2	0,18403204
Otocolobus manul NC028323 1	Nyctereutes procyonoides NC013700 3	0,18403284
Neophoca cinerea NC008419 1	Leopardus wiedii NC028318 1	0,18403311
Mustela altaica NC021751 1	Felis chaus NC028307 1	0,18403517
Catopuma temminckii NC027115 41	Canis anthus NC027956 2	0,18403532
Lynx pardinus NC028319 161	Arctotherium sp NC030174 1	0,18403554
Panthera onca NC022842 1	Enhydra lutris NC009692 1	0,18403587
Prionailurus planiceps NC028312 6	Arctotherium sp NC030174 1	0,18403643
Martes pennanti NC020664 16	Leopardus guigna NC028321 1	0,18403718
Otaria byronia OTAB 1	Leopardus pardalis NC028315 1	0,18403731
Otocyon megalotis SAF1 2	Lynx lynx NC027083 4	0,18403809
Ursus arctos pruinosus MG066703 3	Ailurus fulgens NC011124 1	0,18403839
Procyon lotor AB462046 3	Catopuma temminckii NC027115 41	0,18403855
Catopuma temminckii NC027115 41	Arctotherium sp NC030174 1	0,18403912
Martes foina NC020643 1	Genetta genetta T297 1	0,18403971
Zalophus wollebaeki SRR4431565 1	Prionailurus viverrinus NC028305 1	0,18404201
Ursus arctos GU573486 5	Genetta servalina NC024568 2	0,18404207
Cryptoprocta ferox CFC13 1	Canis adustus KT448271 1	0,18404241
Mustela sibirica NC020637 6	Melursus ursinus NC009970 2	0,18404366

Neovison vison NC020641 3	Lontra canadensis SRR10409165 1	0,13804905
Nandinia binotata NC024567 1	Genetta abyssinica MG489822 1	0,1380865
Vulpes corsac NC023958 1	Nyctereutes procyonoides NC013700 3	0,13808659
Meles anakuma NC009677 1	Lutra sumatrana NC035810 1	0,13809812
Mungotictis decemlineata NC027828 1	Xenogale naso C07XAR110 1	0,13809863
Mustela sibirica AP017394 11	Meles anakuma NC009677 1	0,13810435
Mustela putorius NC020638 4	Meles meles T303 3	0,13810529
Viverra zibetha T609 1	Leopardus pardalis T262 1	0,13810714
Prionailurus bengalensis NC028301 12	Genetta servalina NC024568 2	0,13810901
Viverricula indica NC025296 2	Leopardus pardalis T262 1	0,13811094
Otocyon megalotis SAF1 2	Canis aureus KT448274 1	0,13814998
Leopardus colocolo NC028314 1	Civettictis civetta NC033378 1	0,13815473
Puma yagouaroundi NC028311 1	Nandinia binotata NC024567 1	0,13817321
Viverricula indica KX891745 1	Prionailurus bengalensis CKM45 20	0,13817899
Ursus americanus JX196366 3	Tremarctos ornatus NC009969 2	0,13817912
Mustela nivalis T306 5	Arctonyx collaris NC020645 1	0,13817915
Vulpes zerda KJ603240 1	Canis lupus chanco NC010340 4	0,13818333
Ursus spelaeus NC011112 8	Tremarctos ornatus NC009969 2	0,13822268
Melursus ursinus NC009970 2	Arctotherium sp NC030174 1	0,13824809
Otocyon megalotis SAF1 2	Canis lupus chanco NC010340 4	0,1382491
Mustela kathiah NC023210 1	Lontra canadensis SRR10409165 1	0,13830461
Melogale moschata NC020644 1	Lontra canadensis SRR10409165 1	0,13830645
Viverra zibetha T609 1	Leopardus wiedii NC028318 1	0,1383094
Viverricula indica NC025296 2	Prionailurus rubiginosus NC028304 2	0,13831353
Mustela altaica NC021751 1	Melogale moschata V0735A 1	0,13831708
Civettictis civetta NC033378 1	Catopuma badia NC028300 1	0,13834637
Viverricula indica KX891745 1	Cynogale bennetti KY117544 1	0,13835551
Meles leucurus NC039173 4	Lutra lutra LC050126 1	0,13836733
Viverra zibetha T609 1	Leopardus pardalis NC028315 1	0,13837638
Poecilogale albinucha T602 1	Martes melampus NC009678 1	0,13837896
Viverricula indica NC025296 2	Leptailurus serval NC028316 1	0,13838063
Hydrurga leptonyx NC008425 1	Eumetopias jubatus NC004030 10	0,1384066
Meles meles T303 3	Ictonyx striatus T299 1	0,13844998
Nyctereutes procyonoides NC013700 3	Canis lupus chanco NC010340 4	0,13845478
Gulo gulo NC009685 3	Aonyx cinerea NC035814 2	0,13847303
Vulpes vulpes NC008434 5	Otocyon megalotis SAF1 2	0,13849859
Mungotictis decemlineata NC027828 1	Urva semitorquata MH464789 1	0,13850221
Mustela altaica NC021751 1	Lutrogale perspicillata NC035811 1	0,13851926
Taxidea taxus NC020646 1	Meles meles T303 3	0,13852146
Vulpes ferrilata NC027935 1	Nyctereutes procyonoides NC013700 3	0,13855787
Ursus arctos GU573491 207	Tremarctos ornatus NC009969 2	0,13856503
Meles meles T303 3	Enhydra lutris NC009692 1	0,13856922
Viverricula indica NC025296 2	Catopuma badia NC028300 1	0,13858709
Taxidea taxus NC020646 1	Lutra lutra NC011358 9	0,13858773
Otocyon megalotis SAF1 2	Canis lupus familiaris NC002008 1231	0,13859136
Viverricula indica NC025296 2	Leopardus colocolo NC028314 1	0,13859873
Martes pennanti NC020664 16	Lontra canadensis SRR10409165 1	0,13864699
Mustela nigripes NC024942 1	Lutrogale perspicillata NC035811 1	0,13864934
Melogale moschata KP726273 1	Ictonyx striatus T299 1	0,13864996
Viverricula indica KX891745 1	Catopuma badia NC028300 1	0,13865082
Neovison vison NC020641 3	Aonyx cinerea NC035814 2	0,13865368
Viverricula indica KX891751 1	Cynogale bennetti KY117544 1	0,13869165
Urva semitorquata MH464789 1	Eupleres goudotii D128 1	0,13870285
Meles leucurus NC039173 4	Lutra lutra NC011358 9	0,13870382
Nandinia binotata NC024567 1	Leopardus pardalis NC028315 1	0,1387136
Viverricula indica KX891751 1	Prionailurus bengalensis NC028301 12	0,1387136
Salanoia concolor D378 1	Galerella sanguinea T378 1	0,13871466
Viverricula indica NC025296 2	Prionailurus bengalensis CKM45 20	0,1387209
Lynx pardinus NC028319 161	Genetta abyssinica MG489822 1	0,13874216
Vulpes zerda KJ603240 1	Otocyon megalotis SAF1 2	0,13876989
Taxidea taxus NC020646 1	Mustela nigripes NC024942 1	0,13879202
Vulpes ferrilata NC027935 1	Canis lupus chanco NC010340 4	0,13879335
Vulpes corsac NC023958 1	Canis lupus chanco NC010340 4	0,1387935
Mustela erminea T305 2	Galictis vittata T412 1	0,13879997
Ursus spelaeus EU327344 13	Tremarctos ornatus NC009969 2	0,13882367
Puma concolor NC016470 22	Civettictis civetta GLC19 1	0,13884956
Mustela itatsi NC034330 19	Galictis vittata T412 1	0,13886641
Leptonychotes weddellii NC008424 1	Callorhinus ursinus NC008415 1	0,13890003
Galidictis fasciata DM333 1	Atilax paludinosus T606 1	0,13890673
Taxidea taxus NC020646 1	Mustela putorius NC020638 4	0,13892661
Prionailurus bengalensis NC028301 12	Civettictis civetta NC033378 1	0,13895039
Nandinia binotata NC024567 1	Felis catus NC001700 2	0,13898143
Otocolobus manul NC028323 1	Nandinia binotata NC024567 1	0,13898169
Nandinia binotata NC024567 1	Leopardus wiedii NC028318 1	0,13898325
Viverricula indica KX891745 1	Prionailurus rubiginosus NC028304 2	0,13898361
Melogale moschata V0735A 1	Ictonyx striatus T299 1	0,13898632
Martes pennanti NC020664 16	Lutrogale perspicillata NC035811 1	0,13904867
Nandinia binotata NC024567 1	Leopardus geoffroyi NC028320 1	0,13905029
Lynx rufus NC014456 3	Civettictis civetta GLC19 1	0,13905102
Mustela frenata NC020640 1	Lutrogale perspicillata NC035811 1	0,1390532
Taxidea taxus NC020646 1	Mustela frenata NC020640 1	0,1390615
Viverricula indica KX891745 1	Leopardus colocolo NC028314 1	0,13906658
Vulpes vulpes NC008434 5	Canis anthus NC027956 2	0,13909648
Otocyon megalotis SAF1 2	Nyctereutes procyonoides NC013700 3	0,1391038
Vulpes ferrilata NC027935 1	Otocyon megalotis SAF1 2	0,13910519
Poecilogale albinucha T602 1	Meles meles T303 3	0,13911711
Prionailurus bengalensis NC028301 12	Civettictis civetta GLC19 1	0,13911901
Mustela altaica NC021751 1	Meles meles T303 3	0,13912131
Meles leucurus NC039173 4	Ictonyx striatus T299 1	0,13912311
Civettictis civetta GLC19 1	Acinonyx jubatus NC005212 3	0,13913117
Taxidea taxus NC020646 1	Melogale moschata NC020644 1	0,13913245
Zalophus californianus NC008416 1	Hydrurga leptonyx NC008425 1	0,13913828
Meles anakuma NC009677 1	Gulo gulo NC009685 3	0,13914113
Meles leucurus NC039173 4	Enhydra lutris NC009692 1	0,13917491
Mungos mungo/gambianus SRR7704821 1	Galidictis fasciata DM333 1	0,13917677
Mustela nivalis T306 5	Meles meles T303 3	0,13918862
Taxidea taxus NC020646 1	Mustela sibirica NC020637 6	0,13919741
Leptonychotes weddellii NC008424 1	Eumetopias jubatus NC004030 10	0,13920502
Hydrurga leptonyx NC008425 1	Callorhinus ursinus NC008415 1	0,13924709
Prionailurus planiceps KY682741 4	Nandinia binotata NC024567 1	0,13925055
Melogale moschata V0735A 1	Meles meles T303 3	0,13930951
Prionailurus planiceps NC028312 6	Nandinia binotata NC024567 1	0,1393179
Prionailurus viverrinus NC028305 1	Nandinia binotata NC024567 1	0,13932091

Mustela sibirica AP017394 11	Helarctos malayanus NC009968 2	0,18404375
Mustela kathiah NC023210 1	Melursus ursinus NC009970 2	0,18404472
Lutra lutra NC011358 9	Genetta abyssinica MG489822 1	0,18404493
Mustela nigripes NC024942 1	Melursus ursinus NC009970 2	0,18404518
Meles meles T303 3	Canis mesomelas KT448280 1	0,18404958
Ursus thibetanus laniger MH281753 2	Poecilogale albinucha T602 1	0,18405248
Arctotherium sp NC030174 1	Acinonyx jubatus NC005212 3	0,18405281
Ursus thibetanus thibetanus NC01111	Felis silvestris lybica KP202275 4	0,18405309
Profelis aurata NC028299 1	Chrysocyon brachyurus NC024172 1	0,1840539
Ursus thibetanus thibetanus NC01111	Leopardus tigrinus NC028317 1	0,18405411
Ursus thibetanus formosanus NC0093	Catopuma badia NC028300 1	0,18405516
Helarctos malayanus NC009968 2	Caracal caracal NC028306 1	0,18405554
Smilodon populator MF871700 1	Eumetopias jubatus NC004030 10	0,18405771
Leopardus geoffroyi NC028320 1	Canis lupus chanco NC010340 4	0,18406075
Lycalopex sechurae KT448284 1	Felis margarita NC028308 1	0,18406557
Lycalopex sechurae KT448284 1	Felis catus NC001700 2	0,18406782
Ursus americanus JX196366 3	Genetta servalina NC024568 2	0,18406843
Mungos mungo MMC7 1	Meles anakuma NC009677 1	0,18407708
Lutra sumatrana NC035810 1	Urva semitorquata MH464789 1	0,18407811
Mungos mungo/gambianus SRR77048	Meles meles T303 3	0,18407902
Phocarctos hookeri NC008418 1	Crossarchus platycephalus C7R66 1	0,18408145
Meles leucurus NC039173 4	Cynogale bennetti KY117544 1	0,18408345
Lutra lutra NC011358 9	Civettictis civetta GLC19 1	0,18408389
Puma yagouaroundi NC028311 1	Tapirus terrestris T358	0,18408391
Lutra lutra NC011358 9	Galerella sanguinea T378 1	0,18408583
Galerella sanguinea T378 1	Enhydra lutris NC009692 1	0,18408762
Mustela itatsi NC034330 19	Canis anthus NC027956 2	0,18408903
Neovison vison NC020641 3	Eupleres goudotii D128 1	0,18409033
Viverricula indica KX891745 1	Melogale moschata V0735A 1	0,18409036
Prionailurus planiceps NC028312 6	Arctonyx collaris NC020645 1	0,18409064
Viverra zibetha T609 1	Melogale moschata V0735A 1	0,18409075
Panthera uncia KP202269 1	Enhydra lutris NC009692 1	0,18409199
Lutra lutra NC011358 9	Cryptoprocta ferox CFC13 1	0,18409206
Acinonyx jubatus NC005212 3	Tapirus terrestris T358	0,18409236
Ursus thibetanus mupinensis NC00875	Lutra lutra LC050126 1	0,1840928
Panthera uncia NC010638 1	Mustela frenata NC020640 1	0,18409322
Urva semitorquata MH464789 1	Canis aureus KT448274 1	0,18409325
Ursus spelaeus EU327344 13	Civettictis civetta NC033378 1	0,18409339
Ursus spelaeus NC011112 8	Civettictis civetta NC033378 1	0,18409362
Ursus spelaeus EU327344 13	Smilodon populator MF871700 1	0,18409364
Urva javanica T413 1	Arctodus simus NC011116 1	0,18409394
Viverricula indica NC025296 2	Melogale moschata KP726273 1	0,18409401
Ursus maritimus GU573488 Svalbard	Genetta abyssinica MG489822 1	0,18409446
Prionodon linsang ERR2391707 1	Phoca vitulina NC001325 1	0,18409482
Ursus maritimus GU573488 Svalbard	Enhydra lutris NC009692 1	0,18409494
Mustela eversmannii NC028013 1	Caracal caracal NC028306 1	0,18409537
Panthera uncia KP202269 1	Mustela erminea T305 2	0,18409599
Vulpes vulpes NC008434 5	Prionailurus viverrinus NC028305 1	0,18409625
Vulpes vulpes NC008434 5	Profelis aurata NC028299 1	0,18409649
Mustela nigripes NC024942 1	Genetta genetta T297 1	0,18409671
Tremarctos ornatus NC009969 2	Caracal caracal NC028306 1	0,18409762
Lynx canadensis NC028313 1	Canis latrans NC008093 7	0,1840979
Mustela eversmannii NC028013 1	Lycaon pictus NC028427 2	0,18409854
Procyon lotor AB462049 4	Civettictis civetta GLC19 1	0,18409862
Ursus arctos EU497665 29	Genetta abyssinica MG489822 1	0,18409908
Prionailurus bengalensis CKM45 20	Arctocephalus australis MG023139 1	0,18409963
Caracal caracal NC028306 1	Arctocephalus pusillus NC008417 1	0,18410306
Melogale moschata KP726273 1	Homotherium latidens MF871702 3	0,18410321
Leopardus pardalis NC028315 1	Arctotherium sp NC030174 1	0,1841033
Panthera onca KP202264 2	Melogale moschata NC020644 1	0,18410382
Ursus arctos AP012576 6	Genetta abyssinica MG489822 1	0,18410455
Otaria byronia OTAB 1	Leopardus wiedii NC028318 1	0,18410546
Ursus arctos EU497665 29	Mustela nivalis T306 5	0,18410616
Ursus thibetanus mupinensis NC00875	Lynx lynx NC027083 4	0,18410668
Procyon lotor AB462046 3	Leopardus geoffroyi NC028320 1	0,18410669
Prionailurus viverrinus NC028305 1	Canis aureus KT448274 1	0,18410738
Mephitis mephitis NC020648 1	Melogale moschata NC020644 1	0,18410814
Ursus thibetanus laniger MH281753 2	Galidictis fasciata DM333 1	0,18410841
Ursus thibetanus mupinensis NC00875	Leopardus tigrinus NC028317 1	0,18410935
Zalophus wollebaeki SRR4431565 1	Paradoxurus jerdoni MH464793 1	0,18410985
Helarctos malayanus NC009968 2	Chrotogale owstoni T607 1	0,18411289
Canis anthus NC027956 2	Acinonyx jubatus NC005212 3	0,18411414
Gulo gulo NC009685 3	Chrotogale owstoni T607 1	0,1841161
Leopardus colocolo NC028314 1	Canis anthus NC027956 2	0,18411645
Ursus thibetanus thibetanus NC01111	Lynx rufus NC014456 3	0,18411988
Ursus thibetanus thibetanus NC01111	Puma concolor NC016470 22	0,18412179
Ursus thibetanus laniger MH281753 2	Lycaon pictus NC028427 2	0,18412428
Ursus thibetanus thibetanus NC01111	Prionailurus bengalensis CKM45 20	0,18412529
Prionailurus bengalensis NC028301 12	Canis mesomelas KT448280 1	0,18412608
Lynx pardinus NC028319 161	Arctocephalus forsteri NC004023 28	0,18412659
Zalophus californianus NC008416 1	Leopardus jacobita NC028322 1	0,18412973
Leopardus geoffroyi NC028320 1	Arctocephalus forsteri NC004023 28	0,18413179
Ommatophoca rossii AY377287etc 1	Lycaon pictus NC028427 2	0,18413502
Ommatophoca rossii AY377287etc 1	Hyaena hyaena NC020669 1	0,18413597
Felis chaus NC028307 1	Canis lupus chanco NC010340 4	0,184136
Ursus thibetanus laniger MH281753 2	Acinonyx jubatus NC005212 3	0,1841369
Genetta abyssinica MG489822 1	Aonyx cinerea NC035814 2	0,184137
Ursus thibetanus thibetanus NC01111	Acinonyx jubatus NC005212 3	0,18413751
Ursus americanus JX196366 3	Chrotogale owstoni T607 1	0,18413781
Mungos mungo/gambianus SRR77048	Lutra lutra NC011358 9	0,18414137
Lutra lutra LC050126 1	Urva semitorquata MH464789 1	0,18414342
Mustela sibirica NC020637 6	Arctocephalus australis MG023139 1	0,18414705
Ursus arctos isabellinus 18B5 2	Gulo gulo NC009685 3	0,18414718
Mustela itatsi NC034330 19	Arctocephalus australis MG023139 1	0,18414746
Mustela kathiah NC023210 1	Atilax paludinosus T606 1	0,18414947
Viverra tangalunga MH464792 1	Smilodon populator MF871700 1	0,18415229
Vulpes lagopus NC026529 3	Mungotictis decemlineata NC027828 1	0,1841538
Martes martes T302 3	Bdeogale nigripes GLC15 1	0,18415396
Tremarctos ornatus NC009969 2	Mustela putorius NC020638 4	0,18415417
Vulpes ferrilata NC027935 1	Salanoia concolor D378 1	0,18415469
Salanoia concolor D378 1	Canis latrans NC008093 7	0,18415543
Lutra lutra LC050126 1	Felis chaus NC028307 1	0,18415664
Crossarchus platycephalus C7R66 1	Arctotherium sp NC030174 1	0,18415761

Leptailurus serval NC028316 1	Genetta servalina NC024568 2	0,13932119
Otocolobus manul NC028323 1	Civettictis civetta GLC19 1	0,13932155
Mustela sibirica NC020637 6	Lutrogale perspicillata NC035811 1	0,13932224
Prionailurus bengalensis CKM45 20	Genetta servalina NC024568 2	0,13932443
Taxidea taxus NC020646 1	Mustela sibirica AP017394 11	0,13933185
Mustela nigripes NC024942 1	Galictis vittata T412 1	0,13933748
Leptailurus serval NC028316 1	Genetta abyssinica MG489822 1	0,13934301
Civettictis civetta NC033378 1	Acinonyx jubatus NC005212 3	0,13936523
Viverricula indica NC025296 2	Cynogale bennetti KY117544 1	0,13936636
Homotherium latidens MF871702 3	Genetta servalina NC024568 2	0,13938498
Viverra zibetha T609 1	Prionailurus planiceps NC028312 6	0,13938744
Viverra zibetha T609 1	Prionailurus planiceps KY682741 4	0,13938755
Mustela frenata NC020640 1	Galictis vittata T412 1	0,13940494
Taxidea taxus NC020646 1	Ictonyx striatus T299 1	0,13941048
Vulpes corsac NC023958 1	Otocyon megalotis SAF1 2	0,13944145
Ursus arctos AP012576 6	Tremarctos ornatus NC009969 2	0,13944745
Viverra tangalunga MH464792 1	Arctonyx collaris NC020645 1	0,13945049
Viverricula indica KX891745 1	Leptailurus serval NC028316 1	0,13945496
Viverricula indica KX891745 1	Puma yagouaroundi NC028311 1	0,13945498
Leopardus pardalis T262 1	Civettictis civetta GLC19 1	0,13945517
Leptailurus serval NC028316 1	Civettictis civetta GLC19 1	0,13945593
Viverra zibetha T609 1	Leopardus colocolo NC028314 1	0,13947031
Vulpes zerda KJ603240 1	Canis anthus NC027956 2	0,13950044
Leopardus colocolo NC028314 1	Hemigalus derbyanus MH464791 1	0,13950756
Viverra tangalunga MH464792 1	Lontra canadensis SRR10409165 1	0,13951649
Mustela putorius NC020638 4	Meles anakuma NC009677 1	0,13951774
Viverra tangalunga MH464792 1	Melogale moschata V0735A 1	0,1395238
Viverricula indica NC025296 2	Profelis aurata NC028299 1	0,13952531
Viverricula indica KX891745 1	Acinonyx jubatus NC005212 3	0,13953419
Prionailurus rubiginosus NC028304 2	Civettictis civetta NC033378 1	0,1395568
Vulpes corsac NC023958 1	Canis anthus NC027956 2	0,13956776
Taxidea taxus NC020646 1	Meles leucurus NC039173 4	0,13959832
Meles anakuma NC009677 1	Enhydra lutris NC009692 1	0,13964601
Meles anakuma NC009677 1	Lutra lutra LC050126 1	0,13964601
Mustela nigripes NC024942 1	Meles anakuma NC009677 1	0,13965227
Viverra zibetha T609 1	Otocolobus manul NC028323 1	0,13965756
Mustela altaica NC021751 1	Arctonyx collaris NC020645 1	0,13965966
Viverricula indica NC025296 2	Otocolobus manul NC028323 1	0,13966055
Leopardus wiedii NC028318 1	Hemigalus derbyanus MH464791 1	0,13966361
Viverricula indica NC025296 2	Acinonyx jubatus NC005212 3	0,13967206
Genetta abyssinica MG489822 1	Catopuma badia NC028300 1	0,13967754
Leopardus pardalis T262 1	Civettictis civetta NC033378 1	0,13968723
Puma concolor NC016470 22	Genetta abyssinica MG489822 1	0,13968895
Urva javanica T413 1	Fossa fossana D350 1	0,13971183
Ursus arctos GU573486 5	Tremarctos ornatus NC009969 2	0,13971609
Nandinia binotata NC024567 1	Lynx canadensis NC028313 1	0,13972144
Mustela eversmannii NC028013 1	Lutrogale perspicillata NC035811 1	0,13972594
Prionailurus bengalensis NC028301 12	Genetta genetta T297 1	0,13972606
Prionailurus bengalensis CKM45 20	Civettictis civetta GLC19 1	0,13972843
Poecilogale albinucha T602 1	Martes foina NC020643 1	0,13973249
Ursus maritimus GU573488 Svalbard	Tremarctos ornatus NC009969 2	0,13977756
Mustela eversmannii NC028013 1	Meles meles T303 3	0,13978781
Viverricula indica KX891745 1	Prionailurus viverrinus NC028305 1	0,13979137
Viverra zibetha T609 1	Profelis aurata NC028299 1	0,13979222
Prionailurus rubiginosus NC028304 2	Civettictis civetta GLC19 1	0,13979258
Mustela sibirica AP017394 11	Lutrogale perspicillata NC035811 1	0,13979317
Lutra lutra LC050126 1	Galictis vittata T412 1	0,13979743
Taxidea taxus NC020646 1	Arctonyx collaris NC020645 1	0,13979749
Poecilogale albinucha T602 1	Melogale moschata NC020644 1	0,13979934
Viverra tangalunga MH464792 1	Taxidea taxus NC020646 1	0,13980152
Viverra zibetha T609 1	Acinonyx jubatus NC005212 3	0,13980375
Otocyon megalotis SAF1 2	Canis anthus NC027956 2	0,13984437
Otocyon megalotis SAF1 2	Canis mesomelas KT448280 1	0,13984574
Nandinia binotata NC024567 1	Leopardus jacobita NC028322 1	0,13985869
Leopardus wiedii NC028318 1	Genetta servalina NC024568 2	0,13985872
Prionailurus rubiginosus NC028304 2	Genetta servalina NC024568 2	0,13985987
Nandinia binotata NC024567 1	Acinonyx jubatus NC005212 3	0,13987075
Puma concolor NC016470 22	Civettictis civetta NC033378 1	0,1398941
Ursus arctos EU497665 29	Tremarctos ornatus NC009969 2	0,13991249
Ursus arctos pruinosus MG066703 3	Tremarctos ornatus NC009969 2	0,13991882
Prionailurus bengalensis NC028301 12	Hemigalus derbyanus MH464791 1	0,13991892
Nandinia binotata NC024567 1	Catopuma temminckii NC027115 41	0,13992265
Mungotictis decemlineata NC027828 1	Galerella sanguinea T378 1	0,13992617
Mustela putorius NC020638 4	Lutrogale perspicillata NC035811 1	0,13992797
Viverricula indica NC025296 2	Leopardus pardalis NC028315 1	0,13992892
Leptailurus serval NC028316 1	Hemigalus derbyanus MH464791 1	0,13995668
Vulpes ferrilata NC027935 1	Canis anthus NC027956 2	0,13997172
Melogale moschata V0735A 1	Lontra canadensis SRR10409165 1	0,13998251
Nandinia binotata NC024567 1	Lynx pardinus NC028319 161	0,13999099
Viverricula indica KX891745 1	Otocolobus manul NC028323 1	0,13999403
Poecilogale albinucha T602 1	Martes zibellina NC011579 39	0,13999431
Urva javanica/auropunctata NC006835 1	Fossa fossana D350 1	0,14001818
Vulpes lagopus NC026529 3	Canis anthus NC027956 2	0,14003906
Nandinia binotata NC024567 1	Genetta servalina NC024568 2	0,14005573
Viverricula indica KX891751 1	Otocolobus manul NC028323 1	0,14006092
Viverricula indica NC025296 2	Puma yagouaroundi NC028311 1	0,14006442
Zalophus wollebaeki SRR4431565 1	Hydrurga leptonyx NC008425 1	0,14006908
Taxidea taxus NC020646 1	Melogale moschata KP726273 1	0,14007445
Prionailurus bengalensis CKM45 20	Civettictis civetta NC033378 1	0,14009885
Galidictis fasciata DM333 1	Cynictis penicillata T375 1	0,14012004
Viverricula indica KX891745 1	Nandinia binotata NC024567 1	0,14012234
Puma concolor NC016470 22	Nandinia binotata NC024567 1	0,14012583
Galidictis fasciata DM333 1	Galerella sanguinea T378 1	0,14012697
Viverricula indica NC025296 2	Nandinia binotata NC024567 1	0,14012726
Poecilogale albinucha T602 1	Melogale moschata KP726273 1	0,14012836
Viverricula indica NC025296 2	Leopardus wiedii NC028318 1	0,14013118
Lutra sumatrana NC035810 1	Galictis vittata T412 1	0,14013416
Vulpes lagopus NC026529 3	Canis lupus chanco NC010340 4	0,14014085
Otocolobus manul NC028323 1	Civettictis civetta NC033378 1	0,14016007
Prionailurus bengalensis NC028301 12	Genetta abyssinica MG489822 1	0,14016214
Viverra tangalunga MH464792 1	Meles meles T303 3	0,14019212
Taxidea taxus NC020646 1	Mustela itatsi NC034330 19	0,14020552
Felis chaus NC028307 1	Civettictis civetta NC033378 1	0,14022918

Lutra lutra NC011358 9	Leopardus guigna NC028321 1	0,18415843
Ursus arctos GU573491 207	Genetta abyssinica MG489822 1	0,18415913
Caracal caracal NC028306 1	Ailurus fulgens styani NC009691 1	0,18416077
Potos flavus T414 1	Canis adustus KT448271 1	0,1841612
Ursus maritimus NC003428 31	Arctocephalus forsteri KT693377 17	0,18416125
Ursus arctos EU497665 29	Martes zibellina NC011579 39	0,18416131
Lutra sumatrana NC035810 1	Caracal caracal NC028306 1	0,18416201
Ursus maritimus NC003428 31	Enhydra lutris NC009692 1	0,18416226
Mustela sibirica AP017394 11	Genetta genetta T297 1	0,18416356
Vulpes lagopus NC026529 3	Leopardus pardalis NC028315 1	0,18416364
Vulpes lagopus NC026529 3	Leptailurus serval NC028316 1	0,18416429
Leopardus pardalis NC028315 1	Arctocephalus forsteri KT693377 17	0,18416436
Prionailurus viverrinus NC028305 1	Melogale moschata V0735A 1	0,18416476
Nyctereutes procyonoides NC013700 3	Lynx lynx NC027083 4	0,18416518
Neophoca cinerea NC008419 1	Leopardus pardalis NC028315 1	0,18416721
Vulpes corsac NC023958 1	Catopuma badia NC028300 1	0,18416741
Paradoxurus jerdoni MH464793 1	Lycalopex sechurae KT448284 1	0,18416744
Ursus arctos GU573486 5	Arctocephalus australis MG023139 1	0,18416792
Otaria byronia OTAB 1	Lynx canadensis NC028313 1	0,18416874
Catopuma badia NC028300 1	Aonyx cinerea NC035814 2	0,18416962
Ursus maritimus GU573488 Svalbard	Civettictis civetta GLC19 1	0,18417026
Chrotogale owstoni T607 1	Ailuropoda melanoleuca NC009492 5	0,18417141
Speothos venaticus C48 2	Phoca fasciata NC008428 1	0,18417148
Viverricula indica KX891745 1	Speothos venaticus C48 2	0,18417355
Felis chaus NC028307 1	Arctodus simus NC011116 1	0,18417367
Speothos venaticus C48 2	Phoca largha NC008430 1	0,18417408
Ursus spelaeus EU327344 13	Melogale moschata V0735A 1	0,18417469
Ursus thibetanus mupinensis NC00875	Genetta genetta T297 1	0,18417469
Viverra zibetha T609 1	Ursus arctos GU573486 5	0,184176
Ursus arctos GU573486 5	Civettictis civetta GLC19 1	0,18417655
Ursus thibetanus mupinensis NC00875	Paguma larvata PDDS11 2	0,18417674
Ursus thibetanus mupinensis NC00875	Prionailurus rubiginosus NC028304 2	0,18417804
Ursus arctos AP012576 6	Arctocephalus gazella BK010918 1	0,18417846
Ursus spelaeus EU327344 13	Arctocephalus forsteri KT693377 17	0,18417868
Ursus arctos AP012576 6	Mustela frenata NC020640 1	0,18417892
Vulpes corsac NC023958 1	Acinonyx jubatus NC005212 3	0,18417922
Zalophus wollebaeki SRR4431565 1	Lynx rufus NC014456 3	0,18417932
Vulpes ferrilata NC027935 1	Acinonyx jubatus NC005212 3	0,18417965
Phoca groenlandica NC008429 54	Crocuta crocuta NC020670 3	0,18417975
Taxidea taxus NC020646 1	Canis mesomelas KT448280 1	0,18417986
Canis adustus KT448271 1	Arctocephalus forsteri NC004023 28	0,18418212
Ursus americanus JX196366 3	Gulo gulo NC009685 3	0,18418235
Viverra tangalunga MH464792 1	Ursus spelaeus EU327344 13	0,18418238
Ursus arctos AP012576 6	Arctonyx collaris NC020645 1	0,18418242
Zalophus californianus NC008416 1	Eupleres goudotii D128 1	0,18418311
Leptailurus serval NC028316 1	Ailuropoda melanoleuca NC009492 5	0,1841833
Melogale moschata V0735A 1	Genetta abyssinica MG489822 1	0,18418387
Martes flavigula NC012141 3	Conepatus chinga NC042596 1	0,18418416
Zalophus californianus NC008416 1	Conepatus chinga NC042596 1	0,18418449
Spilogale putorius NC010497 1	Callorhinus ursinus NC008415 1	0,18418576
Ursus thibetanus laniger MH281753 2	Lynx rufus NC014456 3	0,18418585
Monachus schauinslandi NC008421 1	Crocuta crocuta NC020670 3	0,18418677
Mustela kathiah NC023210 1	Urva javanica/auropunctata NC006835	0,18419212
Arctocephalus forsteri NC004023 28	Aonyx cinerea NC035814 2	0,18419388
Otaria byronia OTAB 1	Canis adustus KT448271 1	0,18419626
Ommatophoca rossii AY377287etc 1	Lycalopex sechurae KT448284 1	0,18419635
Leopardus guigna NC028321 1	Canis lupus familiaris NC002008 1231	0,18420166
Pardofelis marmorata NLN3 2	Gulo gulo NC009685 3	0,18420386
Ursus spelaeus EU327344 13	Arctocephalus forsteri NC004023 28	0,18420438
Ursus thibetanus laniger MH281753 2	Leopardus colocolo NC028314 1	0,18420564
Prionailurus planiceps KY682741 4	Eumetopias jubatus NC004030 10	0,18420869
Puma concolor NC016470 22	Eumetopias jubatus NC004030 10	0,18420929
Mungos mungo/gambianus SRR77048	Lutra lutra LC050126 1	0,18420936
Meles leucurus NC039173 4	Eupleres goudotii D128 1	0,18421182
Felis margarita NC028308 1	Canis mesomelas KT448280 1	0,18421191
Ursus maritimus NC003428 31	Gulo gulo NC009685 3	0,1842147
Meles meles T303 3	Cynictis penicillata T375 1	0,18421502
Mustela eversmannii NC028013 1	Arctocephalus gazella BK010918 1	0,18421566
Neophoca cinerea NC008419 1	Chrotogale owstoni T607 1	0,1842158
Urva javanica/auropunctata NC006835	Canis lupus chanco NC010340 4	0,18421602
Lutra lutra NC011358 9	Galidictis fasciata DM333 1	0,18421644
Lutra lutra LC050126 1	Galidia elegans D146 1	0,18421736
Canis anthus NC027956 2	Atilax paludinosus T606 1	0,18421851
Otocolobus manul NC028323 1	Lutra lutra NC011358 9	0,18422352
Profelis aurata NC028299 1	Arctonyx collaris NC020645 1	0,1842268
Mustela nivalis T306 5	Atilax paludinosus T606 1	0,18422703
Pardofelis marmorata NLN3 2	Callorhinus ursinus NC008415 1	0,18422741
Smilodon populator MF871700 1	Callorhinus ursinus NC008415 1	0,18422782
Melogale moschata NC020644 1	Homotherium latidens MF871702 3	0,18422796
Tremarctos ornatus NC009969 2	Otaria byronia OTAB 1	0,18422821
Ursus arctos GU573491 207	Arctocephalus forsteri KT693377 17	0,18422846
Phoca groenlandica NC008429 54	Nasua nasua NC020647 1	0,18422873
Paguma larvata PDDS11 2	Canis anthus NC027956 2	0,18423107
Panthera pardus japonensis KJ866876	Mustela erminea T305 2	0,18423121
Melogale moschata KP726273 1	Caracal caracal NC028306 1	0,1842313
Potos flavus T414 1	Ichneumia albicauda T603 1	0,18423183
Ursus maritimus NC003428 31	Phocarctos hookeri NC008418 1	0,18423235
Leopardus geoffroyi NC028320 1	Arctocephalus australis MG023139 1	0,18423316
Ursus maritimus NC003428 31	Neophoca cinerea NC008419 1	0,18423337
Vulpes corsac NC023958 1	Mirounga angustirostris SRR10331586	0,1842337
Potos flavus T414 1	Ailuropoda melanoleuca NC009492 5	0,18423538
Ursus arctos EU497665 29	Martes martes T302 3	0,18423602
Felis margarita NC028308 1	Arctocephalus pusillus NC008417 1	0,18423672
Ursus arctos EU497665 29	Civettictis civetta GLC19 1	0,1842377
Profelis aurata NC028299 1	Procyon lotor AB462049 4	0,1842379
Paradoxurus hermaphroditus NC03959	Arctodus simus NC011116 1	0,1842387
Melogale moschata V0735A 1	Helarctos malayanus NC009968 2	0,18424009
Melogale moschata V0735A 1	Canis adustus KT448271 1	0,18424013
Panthera onca NC022842 1	Mustela putorius NC020638 4	0,18424074
Felis margarita NC028308 1	Bassariscus sumichrasti SRX1099089 1	0,18424107
Ursus maritimus GU573488 Svalbard	Poecilogale albinucha T602 1	0,18424131
Helarctos malayanus NC009968 2	Crossarchus platycephalus C7R66 1	0,18424244
Chrotogale owstoni T607 1	Canis lupus chanco NC010340 4	0,18424296

Eupleres goudotii D128 1	Crossarchus platycephalus C7R66 1	0,14025235
Nandinia binotata NC024567 1	Civettictis civetta GLC19 1	0,14025782
Mustela eversmannii NC028013 1	Meles anakuma NC009677 1	0,14025818
Nandinia binotata NC024567 1	Leptailurus serval NC028316 1	0,14026086
Felis margarita NC028308 1	Civettictis civetta GLC19 1	0,14026375
Meles anakuma NC009677 1	Ictonyx striatus T299 1	0,14026727
Mustela sibirica NC020637 6	Galictis vittata T412 1	0,14027967
Smilodon populator MF871700 1	Civettictis civetta GLC19 1	0,14032175
Viverra zibetha T609 1	Caracal caracal NC028306 1	0,14033066
Prionailurus planiceps KY682741 4	Civettictis civetta GLC19 1	0,14033087
Prionailurus viverrinus NC028305 1	Genetta servalina NC024568 2	0,14033094
Felis silvestris lybica KP202275 4	Civettictis civetta GLC19 1	0,14033099
Galictis vittata T412 1	Enhydra lutris NC009692 1	0,14033593
Mustela itatsi NC034330 19	Lutrogale perspicillata NC035811 1	0,14039923
Prionailurus planiceps KY682741 4	Civettictis civetta NC033378 1	0,14043316
Lynx rufus NC014456 3	Civettictis civetta NC033378 1	0,14043441
Otocyon megalotis SAF1 2	Lycalopex sechurae KT448284 1	0,1404365
Vulpes vulpes NC008434 5	Canis mesomelas KT448280 1	0,14044291
Galidia elegans D146 1	Crossarchus platycephalus C7R66 1	0,14045416
Mustela altaica NC021751 1	Meles leucurus NC039173 4	0,14046748
Galictis vittata T412 1	Aonyx cinerea NC035814 2	0,1404704
Vulpes corsac NC023958 1	Canis mesomelas KT448280 1	0,14050972
Leopardus pardalis NC028315 1	Genetta abyssinica MG489822 1	0,14051503
Meles anakuma NC009677 1	Lutra lutra NC011358 9	0,1405209
Salanoia concolor D378 1	Cynictis penicillata T375 1	0,14052373
Viverricula indica KX891745 1	Leopardus pardalis NC028315 1	0,14053087
Viverricula indica KX891751 1	Prionailurus rubiginosus NC028304 2	0,14053181
Viverricula indica NC025296 2	Leopardus jacobita NC028322 1	0,14053543
Nyctereutes procyonoides NC013700 3	Canis anthus NC027956 2	0,14057766
Salanoia concolor D378 1	Atilax paludinosus T606 1	0,14058983
Mungotictis decemlineata NC027828 1	Bdeogale nigripes GLC15 1	0,14059038
Viverricula indica KX891745 1	Leopardus pardalis T262 1	0,14059833
Mustela putorius NC020638 4	Galictis vittata T412 1	0,14061619
Smilodon populator MF871700 1	Civettictis civetta NC033378 1	0,14064857
Ursus maritimus NC003428 31	Tremarctos ornatus NC009969 2	0,14065324
Leptonychotes weddellii NC008424 1	Arctocephalus pusillus NC008417 1	0,14065702
Nandinia binotata NC024567 1	Lynx rufus NC014456 3	0,14066437
Nandinia binotata NC024567 1	Leopardus guigna NC028321 1	0,14066631
Viverra zibetha T609 1	Leptailurus serval NC028316 1	0,14066721
Prionailurus planiceps NC028312 6	Civettictis civetta GLC19 1	0,1406675
Nyctereutes procyonoides NC013700 3	Lycalopex sechurae KT448284 1	0,14068627
Cynogale bennetti KY117544 1	Civettictis civetta GLC19 1	0,14071362
Eupleres goudotii D128 1	Cynictis penicillata T375 1	0,14072175
Salanoia concolor D378 1	Mungos mungo/gambianus SRR7704821 1	0,14072509
Mungotictis decemlineata NC027828 1	Cynictis penicillata T375 1	0,14072566
Poecilogale albinucha T602 1	Meles leucurus NC039173 4	0,14073238
Otocolobus manul NC028323 1	Genetta servalina NC024568 2	0,14073573
Viverricula indica KX891745 1	Catopuma temminckii NC027115 41	0,14073846
Lutra lutra NC011358 9	Galictis vittata T412 1	0,1407398
Taxidea taxus NC020646 1	Neovison vison NC020641 3	0,14074993
Nandinia binotata NC024567 1	Fossa fossana D350 1	0,14078912
Leopardus wiedii NC028318 1	Civettictis civetta GLC19 1	0,14080222
Salanoia concolor D378 1	Ichneumia albicauda T603 1	0,14080308
Leopardus colocolo NC028314 1	Genetta servalina NC024568 2	0,14081778
Civettictis civetta GLC19 1	Catopuma temminckii NC027115 41	0,14087218
Viverricula indica NC025296 2	Puma concolor NC016470 22	0,14087228
Nandinia binotata NC024567 1	Leopardus colocolo NC028314 1	0,14088166
Hemigalus derbyanus MH464791 1	Catopuma badia NC028300 1	0,14090793
Mungotictis decemlineata NC027828 1	Atilax paludinosus T606 1	0,14092635
Xenogale naso C07XAR110 1	Galidictis fasciata DM333 1	0,140927
Viverricula indica KX891751 1	Nandinia binotata NC024567 1	0,14093027
Leopardus pardalis T262 1	Genetta servalina NC024568 2	0,14093597
Lynx pardinus NC028319 161	Genetta servalina NC024568 2	0,14093645
Leopardus pardalis NC028315 1	Civettictis civetta GLC19 1	0,14093649
Mustela eversmannii NC028013 1	Galictis vittata T412 1	0,14095265
Felis silvestris lybica KP202275 4	Civettictis civetta NC033378 1	0,14096937
Otocolobus manul NC028323 1	Genetta abyssinica MG489822 1	0,14097464
Gulo gulo NC009685 3	Galictis vittata T412 1	0,14097911
Prionailurus rubiginosus NC028304 2	Genetta abyssinica MG489822 1	0,14098673
Nyctereutes procyonoides NC013700 3	Chrysocyon brachyurus NC024172 1	0,14099646
Viverra zibetha T609 1	Puma concolor NC016470 22	0,14100361
Felis nigripes NC028309 1	Civettictis civetta GLC19 1	0,14100444
Leopardus wiedii NC028318 1	Genetta abyssinica MG489822 1	0,14100587
Viverricula indica KX891751 1	Prionailurus bengalensis CKM45 20	0,14100657
Viverricula indica NC025296 2	Felis nigripes NC028309 1	0,14100678
Taxidea taxus NC020646 1	Mustela eversmannii NC028013 1	0,14101312
Leopardus wiedii NC028318 1	Civettictis civetta NC033378 1	0,1410345
Leptailurus serval NC028316 1	Civettictis civetta NC033378 1	0,14103824
Prionailurus planiceps NC028312 6	Civettictis civetta NC033378 1	0,14103933
Leopardus jacobita NC028322 1	Hemigalus derbyanus MH464791 1	0,14105934
Lobodon carcinophaga NC008423 1	Callorhinus ursinus NC008415 1	0,14106432
Fossa fossana D350 1	Civettictis civetta GLC19 1	0,14106551
Paguma larvata PDDS11 2	Leptailurus serval NC028316 1	0,14107093
Viverricula indica KX891745 1	Profelis aurata NC028299 1	0,14107139
Viverricula indica NC025296 2	Prionailurus viverrinus NC028305 1	0,14107412
Mirounga leonina NC008422 1	Eumetopias jubatus NC004030 10	0,14109118
Poecilogale albinucha T602 1	Gulo gulo NC009685 3	0,14110492
Smilodon populator MF871700 1	Nandinia binotata NC024567 1	0,14110847
Mungos mungo/gambianus SRR7704821 1	Eupleres goudotii D128 1	0,14112648
Vulpes ferrilata NC027935 1	Canis mesomelas KT448280 1	0,14112729
Mungotictis decemlineata NC027828 1	Mungos mungo/gambianus SRR7704821 1	0,141129
Viverricula indica NC025296 2	Fossa fossana D350 1	0,14113729
Mustela kathiah NC023210 1	Lutrogale perspicillata NC035811 1	0,14113902
Civettictis civetta GLC19 1	Caracal caracal NC028306 1	0,14113948
Viverricula indica KX891745 1	Leopardus geoffroyi NC028320 1	0,14114265
Taxidea taxus NC020646 1	Melogale moschata V0735A 1	0,14115159
Eumetopias jubatus NC004030 10	Erignathus barbatus NC008426 1	0,1411608
Mungos mungo MMC7 1	Galidictis fasciata DM333 1	0,14119992
Viverricula indica KX891751 1	Leopardus pardalis T262 1	0,14120351
Lynx pardinus NC028319 161	Civettictis civetta GLC19 1	0,14120577
Mustela sibirica AP017394 11	Galictis vittata T412 1	0,14122182
Leopardus pardalis NC028315 1	Civettictis civetta NC033378 1	0,14123614
Lynx pardinus NC028319 161	Civettictis civetta NC033378 1	0,14124246

Paguma larvata PDDS11 2	Ictonyx striatus T299 1	0,18424471
Ictonyx striatus T299 1	Felis catus NC001700 2	0,18424517
Ursus arctos AP012576 6	Martes americana NC020642 1	0,18424977
Fossa fossana D350 1	Canis lupus familiaris NC002008 1231	0,18425021
Taxidea taxus NC020646 1	Lycalopex sechurae KT448284 1	0,18425098
Ursus arctos AP012576 6	Genetta servalina NC024568 2	0,18425129
Prionodon pardicolor NC024569 2	Chrysocyon brachyurus NC024172 1	0,18425159
Procyon lotor AB462049 4	Acinonyx jubatus NC005212 3	0,18425186
Ursus thibetanus laniger MH281753 2	Paguma larvata PDDS11 2	0,18425432
Felis nigripes NC028309 1	Chrysocyon brachyurus NC024172 1	0,18425545
Spilogale putorius NC010497 1	Chrotogale owstoni T607 1	0,18425621
Chrysocyon brachyurus NC024172 1	Caracal caracal NC028306 1	0,18425633
Puma yagouaroundi NC028311 1	Chrysocyon brachyurus NC024172 1	0,18425678
Prionailurus bengalensis CKM45 20	Helarctos malayanus NC009968 2	0,18426031
Leopardus tigrinus NC028317 1	Gulo gulo NC009685 3	0,18426995
Phoca vitulina NC001325 1	Cuon alpinus NC013445 3	0,18427168
Ursus spelaeus NC011112 8	Leopardus colocolo NC028314 1	0,18427393
Martes melampus NC009678 1	Canis mesomelas KT448280 1	0,18427597
Homotherium latidens MF871702 3	Helarctos malayanus NC009968 2	0,18428141
Paguma larvata PDDS11 2	Enhydra lutris NC009692 1	0,18428242
Mustela sibirica NC020637 6	Urva semitorquata MH464789 1	0,18428311
Viverra zibetha T609 1	Lutra lutra NC011358 9	0,18428354
Mungos mungo MMC7 1	Arctocephalus townsendi NC008420 1	0,18428402
Neophoca cinerea NC008419 1	Mustela eversmannii NC028013 1	0,1842847
Mustela kathiah NC023210 1	Urva javanica T413 1	0,1842849
Mungotictis decemlineata NC027828 1	Martes zibellina NC011579 39	0,18428549
Tremarctos ornatus NC009969 2	Arctocephalus townsendi NC008420 1	0,18428561
Smilodon populator MF871700 1	Martes zibellina NC011579 39	0,18428932
Melogale moschata NC020644 1	Eupleres goudotii D128 1	0,18428999
Potos flavus T414 1	Mungotictis decemlineata NC027828 1	0,18429004
Lutra lutra NC011358 9	Leopardus tigrinus NC028317 1	0,18429323
Puma concolor NC016470 22	Ailurus fulgens NC011124 1	0,18429443
Vulpes corsac NC023958 1	Viverra zibetha T609 1	0,18429458
Prionodon pardicolor NC024569 2	Melogale moschata KP726273 1	0,1842957
Ursus arctos GU573491 207	Arctocephalus australis MG023139 1	0,18429577
Ursus arctos GU573491 207	Martes pennanti NC020664 16	0,18429588
Atilax paludinosus T606 1	Arctodus simus NC011116 1	0,18429594
Neophoca cinerea NC008419 1	Galerella sanguinea T378 1	0,18429594
Pardofelis marmorata NLN3 2	Mustela sibirica AP017394 11	0,18429618
Ursus arctos EU497665 29	Fossa fossana D350 1	0,18429668
Ursus arctos isabellinus 18B5 2	Arctocephalus gazella BK010918 1	0,18429686
Poecilogale albinucha T602 1	Phocarctos hookeri NC008418 1	0,18429694
Profelis aurata NC028299 1	Lutra sumatrana NC035810 1	0,18429719
Ursus arctos GU573491 207	Arctocephalus gazella BK010918 1	0,18429722
Lynx lynx NC027083 4	Arctocephalus australis MG023139 1	0,18429804
Tremarctos ornatus NC009969 2	Leopardus wiedii NC028318 1	0,1842986
Pardofelis marmorata NLN3 2	Martes zibellina NC011579 39	0,18429866
Vulpes zerda KJ603240 1	Viverricula indica NC025296 2	0,18429874
Ursus maritimus NC003428 31	Genetta abyssinica MG489822 1	0,18429932
Felis nigripes NC028309 1	Canis anthus NC027956 2	0,18430063
Leopardus wiedii NC028318 1	Arctocephalus gazella BK010918 1	0,18430093
Prionailurus planiceps KY682741 4	Canis latrans NC008093 7	0,18430144
Zalophus wollebaeki SRR4431565 1	Bassaricyon neblina SRX1097850 1	0,18430148
Procyon lotor AB462049 4	Prionailurus planiceps KY682741 4	0,18430361
Procyon lotor AB462049 4	Prionailurus planiceps NC028312 6	0,18430382
Enhydra lutris NC009692 1	Ailuropoda melanoleuca NC009492 5	0,18430491
Martes pennanti NC020664 16	Catopuma badia NC028300 1	0,18430503
Puma concolor NC016470 22	Aonyx cinerea NC035814 2	0,18430515
Ichneumia albicauda T603 1	Canis aureus KT448274 1	0,18430628
Martes pennanti NC020664 16	Leopardus geoffroyi NC028320 1	0,1843065
Felis catus NC001700 2	Bassariscus sumichrasti SRX1099089 1	0,18430707
Pardofelis marmorata NLN3 2	Arctodus simus NC011116 1	0,18430752
Ursus arctos GU573486 5	Bassariscus sumichrasti SRX1099089 1	0,18431024
Mustela putorius NC020638 4	Arctictis binturong T605 2	0,18431038
Ursus thibetanus mupinensis NC00875	Felis catus NC001700 2	0,18431102
Felis chaus NC028307 1	Canis aureus KT448274 1	0,1843126
Mustela putorius NC020638 4	Melursus ursinus NC009970 2	0,18431424
Diplogale hosei MH464790 1	Conepatus chinga NC042596 1	0,18431467
Zalophus wollebaeki SRR4431565 1	Leptailurus serval NC028316 1	0,18431503
Ursus arctos GU573486 5	Poecilogale albinucha T602 1	0,18431541
Ursus arctos AP012576 6	Civettictis civetta GLC19 1	0,18431824
Ursus spelaeus EU327344 13	Mustela altaica NC021751 1	0,18431975
Ursus thibetanus formosanus NC00933	Lynx pardinus NC028319 161	0,18431985
Ursus spelaeus NC011112 8	Paguma larvata PDDS11 2	0,18432103
Ursus spelaeus NC011112 8	Prionailurus rubiginosus NC028304 2	0,18432385
Panthera onca NC022842 1	Ictonyx striatus T299 1	0,18432407
Smilodon populator MF871700 1	Melogale moschata KP726273 1	0,18432523
Zalophus californianus NC008416 1	Caracal caracal NC028306 1	0,1843297
Leopardus guigna NC028321 1	Canis lupus chanco NC010340 4	0,18433321
Genetta abyssinica MG489822 1	Canis mesomelas KT448280 1	0,18433474
Otocolobus manul NC028323 1	Canis mesomelas KT448280 1	0,18433514
Helarctos malayanus NC009968 2	Acinonyx jubatus NC005212 3	0,1843403
Mustela kathiah NC023210 1	Cuon alpinus NC01345 3	0,18434083
Neofelis nebulosa NC008450 3	Monachus schauinslandi NC008421 1	0,18434455
Martes melampus NC009678 1	Chrotogale owstoni T607 1	0,18434478
Viverricula indica KX891745 1	Spilogale putorius NC010497 1	0,18434589
Felis silvestris lybica KP202275 4	Canis mesomelas KT448280 1	0,18434609
Martes melampus NC009678 1	Canis anthus NC027956 2	0,18435143
Zalophus wollebaeki SRR4431565 1	Civettictis civetta NC033378 1	0,18435179
Civettictis civetta NC033378 1	Ailuropoda melanoleuca NC009492 5	0,1843521
Mustela nigripes NC024942 1	Cynictis penicillata T375 1	0,18435256
Neophoca cinerea NC008419 1	Mungos mungo MMC7 1	0,18435423
Viverricula indica KX891751 1	Martes melampus NC009678 1	0,18435536
Ursus thibetanus laniger MH281753 2	Civettictis civetta NC033378 1	0,18435562
Lutra lutra LC050126 1	Galerella sanguinea T378 1	0,1843558
Tremarctos ornatus NC009969 2	Mustela eversmannii NC028013 1	0,18435606
Tremarctos ornatus NC009969 2	Phocarctos hookeri NC008418 1	0,18435609
Paradoxurus jerdoni MH464793 1	Lutra sumatrana NC035810 1	0,18435645
Lutra lutra LC050126 1	Caracal caracal NC028306 1	0,18435839
Galerella sanguinea T378 1	Callorhinus ursinus NC008415 1	0,18435898
Conepatus chinga NC042596 1	Civettictis civetta GLC19 1	0,18435919
Ommatophoca rossii AY377287etc 1	Cynogale bennetti KY117544 1	0,18435986
Vulpes vulpes NC008434 5	Paguma larvata PDDS11 2	0,1843611

Poecilogale albinucha T602 1	Martes pennanti NC020664 16	0,14128296
Salanoia concolor D378 1	Bdeogale nigripes GLC15 1	0,14133121
Poecilogale albinucha T602 1	Martes americana NC020642 1	0,14134029
Viverra zibetha T609 1	Puma yagouaroundi NC028311 1	0,14134055
Felis chaus NC028307 1	Civettictis civetta GLC19 1	0,14134172
Mustela nivalis T306 5	Meles anakuma NC009677 1	0,14134187
Viverricula indica NC025296 2	Caracal caracal NC028306 1	0,14134349
Taxidea taxus NC020646 1	Mustela altaica NC021751 1	0,14135858
Pusa hispida NC 008433 1	Callorhinus ursinus NC008415 1	0,14140247
Cynogale bennetti KY117544 1	Civettictis civetta NC033378 1	0,14141513
Nandinia binotata NC024567 1	Civettictis civetta NC033378 1	0,14143682
Galidictis fasciata DM333 1	Bdeogale nigripes GLC15 1	0,14146614
Vulpes zerda KJ603240 1	Canis mesomelas KT448280 1	0,14146812
Leopardus pardalis NC028315 1	Genetta servalina NC024568 2	0,14147449
Viverricula indica KX891745 1	Felis nigripes NC028309 1	0,14147478
Leopardus geoffroyi NC028320 1	Civettictis civetta GLC19 1	0,14147913
Neovison vison NC020641 3	Lutrogale perspicillata NC035811 1	0,14148016
Viverricula indica NC025296 2	Leopardus geoffroyi NC028320 1	0,14148323
Zalophus californianus NC008416 1	Lobodon carcinophaga NC008423 1	0,14149468
Neovison vison NC020641 3	Galictis vittata T412 1	0,14149676
Homotherium latidens MF871702 3	Civettictis civetta GLC19 1	0,14150123
Puma yagouaroundi NC028311 1	Hemigalus derbyanus MH464791 1	0,1415251
Viverra tangalunga MH464792 1	Meles anakuma NC009677 1	0,1415365
Viverricula indica KX891745 1	Leopardus jacobita NC028322 1	0,14154156
Felis catus NC001700 2	Civettictis civetta GLC19 1	0,14154291
Viverricula indica NC025296 2	Leopardus tigrinus NC028317 1	0,14154534
Felis margarita NC028308 1	Civettictis civetta NC033378 1	0,14157574
Cynogale bennetti KY117544 1	Arctictis binturong T605 2	0,14159773
Leopardus geoffroyi NC028320 1	Hemigalus derbyanus MH464791 1	0,14159901
Homotherium latidens MF871702 3	Hemigalus derbyanus MH464791 1	0,14160495
Nandinia binotata NC024567 1	Caracal caracal NC028306 1	0,14160763
Taxidea taxus NC020646 1	Mustela kathiah NC023210 1	0,14161819
Monachus schauinslandi NC008421 1	Eumetopias jubatus NC004030 10	0,14164017
Leopardus geoffroyi NC028320 1	Civettictis civetta NC033378 1	0,14164411
Leopardus geoffroyi NC028320 1	Genetta abyssinica MG489822 1	0,14166701
Hydrurga leptonyx NC008425 1	Arctocephalus gazella BK010918 1	0,14167391
Puma yagouaroundi NC028311 1	Civettictis civetta GLC19 1	0,14167778
Viverra tangalunga MH464792 1	Lutrogale perspicillata NC035811 1	0,14167798
Lynx canadensis NC028313 1	Genetta abyssinica MG489822 1	0,14168176
Genetta servalina NC024568 2	Acinonyx jubatus NC005212 3	0,14169019
Otocyon megalotis SAF1 2	Cuon alpinus NC013445 3	0,14169435
Fossa fossana D350 1	Civettictis civetta NC033378 1	0,14170752
Puma yagouaroundi NC028311 1	Civettictis civetta NC033378 1	0,14171173
Leopardus pardalis NC028315 1	Hemigalus derbyanus MH464791 1	0,14172229
Phoca fasciata NC008428 1	Callorhinus ursinus NC008415 1	0,14173488
Lynx lynx NC027083 4	Genetta abyssinica MG489822 1	0,14174782
Martes martes T302 3	Galictis vittata T412 1	0,14175929
Meles meles T303 3	Aonyx cinerea NC035814 2	0,14179958
Puma yagouaroundi NC028311 1	Genetta abyssinica MG489822 1	0,14180616
Nandinia binotata NC024567 1	Felis nigripes NC028309 1	0,14180954
Lutrogale perspicillata NC035811 1	Ictonyx striatus T299 1	0,14181145
Poecilogale albinucha T602 1	Aonyx cinerea NC035814 2	0,14181168
Mungotictis decemlineata NC027828 1	Ichneumia albicauda T603 1	0,14181282
Viverricula indica KX891751 1	Acinonyx jubatus NC005212 3	0,14182326
Leopardus colocolo NC028314 1	Genetta abyssinica MG489822 1	0,14182504
Mustela altaica NC021751 1	Galictis vittata T412 1	0,14183344
Leptonychotes weddellii NC008424 1	Arctocephalus forsteri KT693377 17	0,1418643
Salanoia concolor D378 1	Helogale parvula SRR7637809 1	0,14186925
Viverra tangalunga MH464792 1	Meles leucurus NC039173 4	0,14187498
Pardofelis marmorata NLN3 2	Nandinia binotata NC024567 1	0,14187682
Viverra zibetha T609 1	Lynx pardinus NC028319 161	0,14187843
Viverricula indica KX891751 1	Leptailurus serval NC028316 1	0,14187866
Otocyon megalotis SAF1 2	Chrysocyon brachyurus NC024172 1	0,14188126
Genetta abyssinica MG489822 1	Acinonyx jubatus NC005212 3	0,14188913
Genetta abyssinica MG489822 1	Fossa fossana D350 1	0,14193068
Pusa sibirica NC008432 2	Callorhinus ursinus NC008415 1	0,14193182
Genetta abyssinica MG489822 1	Felis nigripes NC028309 1	0,14193276
Smilodon populator MF871700 1	Genetta abyssinica MG489822 1	0,14193991
Poecilogale albinucha T602 1	Meles anakuma NC009677 1	0,14194375
Viverricula indica KX891745 1	Puma concolor NC016470 22	0,14194606
Leopardus pardalis T262 1	Genetta abyssinica MG489822 1	0,14194977
Viverra zibetha T609 1	Catopuma temminckii NC027115 41	0,14195053
Viverra zibetha T609 1	Leopardus geoffroyi NC028320 1	0,14195089
Viverricula indica NC025296 2	Catopuma temminckii NC027115 41	0,14195387
Zalophus wollebaeki SRR4431565 1	Lobodon carcinophaga NC008423 1	0,14195402
Lutrogale perspicillata NC035811 1	Gulo gulo NC009685 3	0,14197484
Civettictis civetta NC033378 1	Caracal caracal NC028306 1	0,14197776
Prionailurus bengalensis CKM45 20	Genetta abyssinica MG489822 1	0,14200956
Viverricula indica KX891745 1	Leopardus tigrinus NC028317 1	0,14201276
Prionailurus rubiginosus NC028304 2	Genetta genetta T297 1	0,14201573
Viverricula indica KX891751 1	Catopuma badia NC028300 1	0,14201808
Taxidea taxus NC020646 1	Meles anakuma NC009677 1	0,14201827
Ictonyx striatus T299 1	Galictis vittata T412 1	0,14203928
Viverricula indica KX891745 1	Leopardus wiedii NC028318 1	0,14208004
Viverra zibetha T609 1	Leopardus jacobita NC028322 1	0,14208062
Prionailurus bengalensis NC028301 12	Paguma larvata PDDS11 2	0,14208063
Viverricula indica KX891745 1	Caracal caracal NC028306 1	0,14208128
Martes melampus NC009678 1	Galictis vittata T412 1	0,14208999
Viverricula indica NC025296 2	Homotherium latidens MF871702 3	0,1421099
Leopardus pardalis T262 1	Hemigalus derbyanus MH464791 1	0,14213343
Viverra zibetha T609 1	Lynx rufus NC014456 3	0,14214772
Genetta servalina NC024568 2	Felis chaus NC028307 1	0,14214976
Prionailurus viverrinus NC028305 1	Genetta genetta T297 1	0,14215058
Zalophus californianus NC008416 1	Mirounga leonina NC008422 1	0,14215723
Mustela kathiah NC023210 1	Galictis vittata T412 1	0,14216417
Felis nigripes NC028309 1	Civettictis civetta NC033378 1	0,14217849
Hemigalus derbyanus MH464791 1	Acinonyx jubatus NC005212 3	0,14218308
Civettictis civetta NC033378 1	Catopuma temminckii NC027115 41	0,14218412
Leptonychotes weddellii NC008424 1	Arctocephalus australis MG023139 1	0,14220087
Hydrurga leptonyx NC008425 1	Arctocephalus forsteri KT693377 17	0,14221221
Viverricula indica KX891751 1	Leopardus pardalis NC028315 1	0,14221349
Zalophus wollebaeki SRR4431565 1	Leptonychotes weddellii NC008424 1	0,14221364
Ichneumia albicauda T603 1	Galidictis fasciata DM333 1	0,14221482

Lutra lutra LC050126 1	Cryptoprocta ferox CFC13 1	0,18436207
Panthera onca KP202264 2	Callorhinus ursinus NC008415 1	0,18436219
Panthera onca KP202264 2	Mustela nigripes NC024942 1	0,18436417
Lycaon pictus NC028427 2	Galerella sanguinea T378 1	0,18436457
Mustela sibirica NC020637 6	Felis chaus NC028307 1	0,18436517
Prionailurus bengalensis NC028301 12	Arctocephalus australis MG023139 1	0,1843652
Vulpes lagopus NC026529 3	Pardofelis marmorata NLN3 2	0,18436535
Lynx canadensis NC028313 1	Arctocephalus australis MG023139 1	0,18436547
Mustela sibirica NC020637 6	Genetta genetta T297 1	0,18436554
Vulpes vulpes NC008434 5	Neovison vison NC020641 3	0,18436563
Lynx rufus NC014456 3	Arctocephalus australis MG023139 1	0,18436569
Otocolobus manul NC028323 1	Melogale moschata KP726273 1	0,184366
Prionailurus planiceps KY682741 4	Arctocephalus gazella BK010918 1	0,18436612
Tremarctos ornatus NC009969 2	Catopuma temminckii NC027115 41	0,18436639
Prionodon pardicolor NC024569 2	Mustela itatsi NC034330 19	0,18436672
Ursus maritimus GU573488 Svalbard	Phocarctos hookeri NC008418 1	0,18436709
Nyctereutes procyonoides NC013700 3	Lynx canadensis NC028313 1	0,18436773
Leopardus pardalis NC028315 1	Arctocephalus gazella BK010918 1	0,18436776
Vulpes corsac NC023958 1	Prionailurus bengalensis CKM45 20	0,18436931
Ursus arctos EU497665 29	Arctonyx collaris NC020645 1	0,18436982
Viverricula indica NC025296 2	Otocyon megalotis SAF1 2	0,18437064
Ursus arctos GU573486 5	Enhydra lutris NC009692 1	0,1843708
Ursus arctos AP012576 6	Taxidea taxus NC020646 1	0,18437089
Panthera onca KP202264 2	Neovison vison NC020641 3	0,18437146
Poecilogale albinucha T602 1	Leopardus wiedii NC028318 1	0,1843724
Ursus arctos GU573491 207	Genetta servalina NC024568 2	0,18437245
Panthera tigris amoyensis NC014770 2	Mustela kathiah NC023210 1	0,18437277
Otocolobus manul NC028323 1	Melogale moschata NC020644 1	0,18437289
Panthera onca KP202264 2	Mustela nivalis T306 5	0,18437344
Odobenus rosmarus NC004029 29	Lutra sumatrana NC035810 1	0,18437557
Melursus ursinus NC009970 2	Melogale moschata KP726273 1	0,18437579
Ursus thibetanus formosanus NC00933	Chrotogale owstoni T607 1	0,18437648
Panthera leo spelaea KX258452 2	Mustela kathiah NC023210 1	0,18437657
Ursus arctos GU573486 5	Phocarctos hookeri NC008418 1	0,18437676
Ictonyx striatus T299 1	Felis margarita NC028308 1	0,18438035
Urva semitorquata MH464789 1	Canis lupus chanco NC010340 4	0,18438154
Paradoxurus hermaphroditus NLNC 1	Lycaon pictus NC028427 2	0,18438262
Mustela nigripes NC024942 1	Arctocephalus forsteri NC004023 28	0,18438405
Paradoxurus hermaphroditus NC03959	Ailuropoda melanoleuca NC009492 5	0,18438685
Ursus thibetanus laniger MH281753 2	Leopardus jacobita NC028322 1	0,18438943
Ursus thibetanus laniger MH281753 2	Leopardus tigrinus NC028317 1	0,18438944
Potos flavus T414 1	Diplogale hosei MH464790 1	0,18439053
Chrotogale owstoni T607 1	Tapirus terrestris T358	0,18439388
Melursus ursinus NC009970 2	Catopuma badia NC028300 1	0,18439448
Martes flavigula NC012141 3	Bdeogale nigripes GLC15 1	0,18439636
Otocolobus manul NC028323 1	Lycalopex sechurae KT448284 1	0,18439645
Lycaon pictus NC028427 2	Leopardus colocolo NC028314 1	0,1843989
Ursus thibetanus thibetanus NC01111	Ictonyx striatus T299 1	0,18440016
Ursus americanus JX196366 3	Leopardus pardalis NC028315 1	0,18440701
Lycalopex sechurae KT448284 1	Leopardus colocolo NC028314 1	0,18440759
Prionailurus planiceps KY682741 4	Canis mesomelas KT448280 1	0,18440869
Ictonyx striatus T299 1	Hemigalus derbyanus MH464791 1	0,18440963
Canis lupus chanco NC010340 4	Arctodus simus NC011116 1	0,1844101
Ursus americanus JX196366 3	Catopuma temminckii NC027115 41	0,1844115
Fossa fossana D350 1	Tapirus terrestris T358	0,18441154
Lutra sumatrana NC035810 1	Chrotogale owstoni T607 1	0,18441154
Martes melampus NC009678 1	Urva semitorquata MH464789 1	0,18441294
Mungos mungo/gambianus SRR77048	Meles leucurus NC039173 4	0,18441543
Meles leucurus NC039173 4	Urva javanica T413 1	0,18441668
Ictonyx striatus T299 1	Civettictis civetta NC033378 1	0,18441711
Viverra tangalunga MH464792 1	Atilax paludinosus T606 1	0,18441913
Mustela itatsi NC034330 19	Atilax paludinosus T606 1	0,18441929
Mustela nigripes NC024942 1	Urva javanica T413 1	0,18441974
Leopardus pardalis T262 1	Conepatus chinga NC042596 1	0,1844216
Poecilogale albinucha T602 1	Chrotogale owstoni T607 1	0,18442225
Lutrogale perspicillata NC035811 1	Crossarchus platycephalus C7R66 1	0,18442358
Bassaricyon neblina SRX1097850 1	Arctocephalus pusillus NC008417 1	0,18442394
Arctocephalus australis MG023139 1	Aonyx cinerea NC035814 2	0,18442409
Martes martes T302 3	Xenogale naso C07XAR110 1	0,18442424
Urva javanica T413 1	Bassariscus sumichrasti SRX1099089 1	0,18442659
Felis chaus NC028307 1	Callorhinus ursinus NC008415 1	0,18442838
Felis silvestris lybica KP202275 4	Callorhinus ursinus NC008415 1	0,18442877
Catopuma temminckii NC027115 41	Callorhinus ursinus NC008415 1	0,18442887
Vulpes zerda KJ603240 1	Phoca groenlandica NC008429 54	0,18442969
Ursus arctos isabellinus 18B5 2	Melogale moschata V0735A 1	0,18443067
Potos flavus T414 1	Pardofelis marmorata NLN3 2	0,18443197
Cryptoprocta ferox CFC13 1	Callorhinus ursinus NC008415 1	0,18443242
Potos flavus T414 1	Caracal caracal NC028306 1	0,18443254
Lynx lynx NC027083 4	Arctocephalus forsteri KT693377 17	0,18443258
Prionailurus bengalensis NC028301 12	Melogale moschata V0735A 1	0,18443312
Vulpes corsac NC023958 1	Prionailurus bengalensis NC028301 12	0,18443374
Mungotictis decemlineata NC027828 1	Arctodus simus NC011116 1	0,18443391
Vulpes zerda KJ603240 1	Phoca vitulina NC001325 1	0,18443534
Ursus arctos EU497665 29	Neophoca cinerea NC008419 1	0,18443562
Tremarctos ornatus NC009969 2	Leopardus geoffroyi NC028320 1	0,18443574
Urocyon littoralis catalinae KP129018 1	Mirounga angustirostris SRR10331586	0,18443623
Ursus arctos isabellinus 18B5 2	Arctonyx collaris NC020645 1	0,1844369
Procyon lotor AB462049 4	Felis nigripes NC028309 1	0,1844378
Prionailurus planiceps KY682741 4	Arctocephalus pusillus NC008417 1	0,18443801
Procyon lotor AB462046 3	Felis nigripes NC028309 1	0,18443826
Smilodon populator MF871700 1	Phocarctos hookeri NC008418 1	0,18443848
Panthera onca NC022842 1	Lutra lutra LC050126 1	0,18443927
Puma yagouaroundi NC028311 1	Arctocephalus pusillus NC008417 1	0,1844399
Martes pennanti NC020664 16	Felis silvestris lybica KP202275 4	0,18443991
Poecilogale albinucha T602 1	Arctotherium sp NC030174 1	0,1844402
Prionailurus planiceps KY682741 4	Arctotherium sp NC030174 1	0,18444031
Leopardus guigna NC028321 1	Aonyx cinerea NC035814 2	0,18444046
Taxidea taxus NC020646 1	Chrysocyon brachyurus NC024172 1	0,18444195
Viverra tangalunga MH464792 1	Chrysocyon brachyurus NC024172 1	0,18444243
Panthera tigris amoyensis NC014770 2	Mustela erminea T305 2	0,18444288
Ursus arctos isabellinus 18B5 2	Profelis aurata NC028299 1	0,18444538
Ursus arctos isabellinus 18B5 2	Martes foina NC020643 1	0,18444586
Viverricula indica KX891745 1	Ictonyx striatus T299 1	0,18444626

Viverricula indica KX891751 1	Leopardus colocolo NC028314 1	0,14223126
Homotherium latidens MF871702 3	Civettictis civetta NC033378 1	0,14224084
Vulpes vulpes NC008434 5	Lycalopex sechurae KT448284 1	0,1422603
Urocyon cinereoargenteus NC026723 21	Canis latrans NC008093 7	0,14226082
Lynx lynx NC027083 4	Civettictis civetta GLC19 1	0,14228275
Zalophus californianus NC008416 1	Leptonychotes weddellii NC008424 1	0,14229339
Nandinia binotata NC024567 1	Crossarchus platycephalus C7R66 1	0,14233774
Phoca groenlandica NC008429 54	Callorhinus ursinus NC008415 1	0,14234082
Leptonychotes weddellii NC008424 1	Arctocephalus gazella BK010918 1	0,14240336
Vulpes lagopus NC026529 3	Canis mesomelas KT448280 1	0,14240423
Meles leucurus NC039173 4	Aonyx cinerea NC035814 2	0,14240527
Viverricula indica KX891751 1	Leopardus wiedii NC028318 1	0,1424159
Hemigalus derbyanus MH464791 1	Caracal caracal NC028306 1	0,14241596
Viverricula indica KX891745 1	Felis silvestris lybica KP202275 4	0,14241751
Genetta servalina NC024568 2	Felis margarita NC028308 1	0,14241862
Prionailurus bengalensis CKM45 20	Paguma larvata PDDS11 2	0,14242075
Leopardus guigna NC028321 1	Hemigalus derbyanus MH464791 1	0,14242192
Prionailurus bengalensis CKM45 20	Genetta genetta T297 1	0,1424228
Taxidea taxus NC020646 1	Mustela nivalis T306 5	0,1424356
Prionailurus bengalensis CKM45 20	Hemigalus derbyanus MH464791 1	0,14243667
Felis catus NC001700 2	Civettictis civetta NC033378 1	0,14245139
Cystophora cristata NC008427 1	Callorhinus ursinus NC008415 1	0,14246973
Arctonyx collaris NC020645 1	Aonyx cinerea NC035814 2	0,14247258
Eupleres goudotii D128 1	Atilax paludinosus T606 1	0,1424731
Nandinia binotata NC024567 1	Genetta genetta T297 1	0,1424804
Poecilogale albinucha T602 1	Melogale moschata V0735A 1	0,14248399
Genetta servalina NC024568 2	Catopuma badia NC028300 1	0,14248897
Prionailurus bengalensis NC028301 12	Arctictis binturong T605 2	0,14249776
Arctictis binturong T605 2	Acinonyx jubatus NC005212 3	0,14250831
Urva javanica/auropunctata NC006835 1	Galidictis fasciata DM333 1	0,14251265
Pusa caspica NC008431 1	Callorhinus ursinus NC008415 1	0,14253761
Zalophus wollebaeki SRR4431565 1	Mirounga leonina NC008422 1	0,14254895
Galerella sanguinea T378 1	Cryptoprocta ferox CFC13 1	0,14255834
Urocyon littoralis catalinae KP129018 15	Canis latrans NC008093 7	0,14259745
Viverra zibetha T609 1	Smilodon populator MF871700 1	0,14261273
Vulpes zerda KJ603240 1	Lycalopex sechurae KT448284 1	0,14261304
Leopardus pardalis T262 1	Fossa fossana D350 1	0,14261954
Profelis aurata NC028299 1	Civettictis civetta GLC19 1	0,14262098
Melogale moschata NC020644 1	Galictis vittata T412 1	0,14263058
Viverricula indica KX891745 1	Smilodon populator MF871700 1	0,14263469
Prionailurus rubiginosus NC028304 2	Hemigalus derbyanus MH464791 1	0,14264055
Hydrurga leptonyx NC008425 1	Arctocephalus pusillus NC008417 1	0,14268832
Lynx lynx NC027083 4	Civettictis civetta NC033378 1	0,14272478
Ichneumia albicauda T603 1	Eupleres goudotii D128 1	0,14275366
Viverricula indica KX891745 1	Felis chaus NC028307 1	0,14275476
Puma concolor NC016470 22	Genetta servalina NC024568 2	0,14275508
Prionailurus viverrinus NC028305 1	Genetta abyssinica MG489822 1	0,14276293
Mungotictis decemlineata NC027828 1	Mungos mungo MMC7 1	0,14281464
Lobodon carcinophaga NC008423 1	Arctocephalus forsteri KT693377 17	0,14281777
Viverra zibetha T609 1	Felis chaus NC028307 1	0,14282237
Genetta servalina NC024568 2	Felis silvestris lybica KP202275 4	0,14282241
Genetta servalina NC024568 2	Felis nigripes NC028309 1	0,14282268
Salanoia concolor D378 1	Urva javanica/auropunctata NC006835 1	0,14284892
Genetta servalina NC024568 2	Fossa fossana D350 1	0,14288312
Viverricula indica KX891751 1	Prionailurus viverrinus NC028305 1	0,14288813
Viverricula indica NC025296 2	Felis chaus NC028307 1	0,14289233
Martes zibellina NC011579 39	Galictis vittata T412 1	0,14289321
Leopardus tigrinus NC028317 1	Genetta abyssinica MG489822 1	0,14289418
Martes foina NC020643 1	Galictis vittata T412 1	0,14289618
Prionodon pardicolor NC024569 2	Leopardus pardalis NC028315 1	0,1429023
Leptailurus serval NC028316 1	Genetta genetta T297 1	0,1429586
Paguma larvata PDDS11 2	Catopuma badia NC028300 1	0,14296192
Leopardus jacobita NC028322 1	Genetta abyssinica MG489822 1	0,14296772
Catopuma badia NC028300 1	Arctictis binturong T605 2	0,14297596
Homotherium latidens MF871702 3	Genetta abyssinica MG489822 1	0,14301282
Salanoia concolor D378 1	Mungos mungo MMC7 1	0,14301716
Viverricula indica KX891751 1	Leopardus jacobita NC028322 1	0,14302216
Viverricula indica KX891751 1	Puma yagouaroundi NC028311 1	0,14302331
Viverricula indica KX891745 1	Prionailurus planiceps KY682741 4	0,14302337
Prionailurus planiceps NC028312 6	Genetta servalina NC024568 2	0,14302437
Prionailurus planiceps KY682741 4	Genetta servalina NC024568 2	0,14302443
Prionailurus planiceps NC028312 6	Genetta abyssinica MG489822 1	0,14303313
Paguma larvata PDDS11 2	Leopardus colocolo NC028314 1	0,14303915
Lynx rufus NC014456 3	Genetta abyssinica MG489822 1	0,1430475
Genetta servalina NC024568 2	Cynogale bennetti KY117544 1	0,1430711
Viverra zibetha T609 1	Lynx lynx NC027083 4	0,14309011
Paradoxurus hermaphroditus NLNC 1	Leptailurus serval NC028316 1	0,14309014
Leopardus jacobita NC028322 1	Genetta servalina NC024568 2	0,14309125
Viverricula indica NC025296 2	Leopardus guigna NC028321 1	0,14309955
Prionodon pardicolor NC024569 2	Leopardus pardalis T262 1	0,14310439
Smilodon populator MF871700 1	Genetta servalina NC024568 2	0,14311649
Leopardus tigrinus NC028317 1	Hemigalus derbyanus MH464791 1	0,1431292
Meles anakuma NC009677 1	Aonyx cinerea NC035814 2	0,14314556
Nandinia binotata NC024567 1	Mungotictis decemlineata NC027828 1	0,1431485
Urva semitorquata MH464789 1	Cryptoprocta ferox CFC13 1	0,14315437
Paguma larvata PDDS11 2	Leopardus pardalis T262 1	0,14315623
Prionailurus viverrinus NC028305 1	Civettictis civetta GLC19 1	0,14315987
Prionailurus planiceps NC028312 6	Genetta genetta T297 1	0,14316054
Viverricula indica NC025296 2	Felis silvestris lybica KP202275 4	0,14316173
Viverricula indica KX891751 1	Leopardus geoffroyi NC028320 1	0,14316286
Genetta abyssinica MG489822 1	Caracal caracal NC028306 1	0,14317612
Mungotictis decemlineata NC027828 1	Urva javanica/auropunctata NC006835 1	0,14318494
Prionailurus viverrinus NC028305 1	Civettictis civetta NC033378 1	0,14319517
Xenogale naso C07XAR110 1	Eupleres goudotii D128 1	0,1432136
Monachus monachus NC044972 5	Eumetopias jubatus NC004030 10	0,14321844
Monachus schauinslandi NC008421 1	Callorhinus ursinus NC008415 1	0,14321876
Nandinia binotata NC024567 1	Leopardus tigrinus NC028317 1	0,14322557
Puma yagouaroundi NC028311 1	Genetta servalina NC024568 2	0,14322665
Prionailurus planiceps KY682741 4	Genetta abyssinica MG489822 1	0,14324124
Martes flavigula NC012141 3	Galictis vittata T412 1	0,14327352
Erignathus barbatus NC008426 1	Callorhinus ursinus NC008415 1	0,14327678
Mungotictis decemlineata NC027828 1	Helogale parvula SRR7637809 1	0,14328319
Nandinia binotata NC024567 1	Galidictis fasciata DM333 1	0,14328367

Prionailurus viverrinus NC028305 1	Lycaon pictus NC028427 2	0,18444899
Martes foina NC020643 1	Canis aureus KT448274 1	0,18445064
Otocolobus manul NC028323 1	Lycaon pictus NC028427 2	0,18445121
Ursus thibetanus formosanus NC0093	Lynx canadensis NC028313 1	0,18445365
Ursus thibetanus mupinensis NC00875	Canis aureus KT448274 1	0,18445846
Mungos mungo/gambianus SRR77048	Martes flavigula NC012141 3	0,18445939
Zalophus californianus NC008416 1	Felis silvestris lybica KP202275 4	0,18446319
Spilogale putorius NC010497 1	Arctodus simus NC011116 1	0,18446869
Galidictis fasciata DM333 1	Eumetopias jubatus NC004030 10	0,18446918
Gulo gulo NC009685 3	Felis nigripes NC028309 1	0,18447483
Mirounga leonina NC008422 1	Tapirus terrestris T358	0,18447854
Lutra lutra LC050126 1	Urva javanica T413 1	0,18447903
Civettictis civetta NC033378 1	Aonyx cinerea NC035814 2	0,18447979
Ursus arctos EU497665 29	Canis adustus KT448271 1	0,18448125
Ursus maritimus GU573488 Svalbard	Gulo gulo NC009685 3	0,1844843
Mustela nigripes NC024942 1	Arctocephalus forsteri KT693377 17	0,1844845
Urva javanica T413 1	Arctonyx collaris NC020645 1	0,18448536
Phocarctos hookeri NC008418 1	Fossa fossana D350 1	0,18448575
Mustela itatsi NC034330 19	Xenogale naso C07XAR110 1	0,184487
Vulpes zerda KJ603240 1	Crossarchus platycephalus C7R66 1	0,18448958
Martes pennanti NC020664 16	Urva semitorquata MH464789 1	0,18449025
Galidictis fasciata DM333 1	Arctonyx collaris NC020645 1	0,18449053
Arctocephalus forsteri KT693377 17	Aonyx cinerea NC035814 2	0,1844915
Lutra lutra NC011358 9	Catopuma temminckii NC027115 41	0,18449228
Pardofelis marmorata NLN3 2	Lutra lutra LC050126 1	0,18449438
Mustela sibirica NC020637 6	Diplogale hosei MH464790 1	0,18449485
Panthera onca KP202264 2	Lutra lutra NC011358 9	0,18449587
Monachus schauinslandi NC008421 1	Tapirus terrestris T358	0,18449678
Prionailurus rubiginosus NC028304 2	Ailurus fulgens NC011124 1	0,18449703
Ursus arctos pruinosus MG066703 3	Martes zibellina NC011579 39	0,18449801
Ursus maritimus NC003428 31	Martes pennanti NC020664 16	0,1844981
Lycaon pictus NC028427 2	Fossa fossana D350 1	0,18449835
Mustela sibirica AP017394 11	Ichneumia albicauda T603 1	0,184499
Paradoxurus hermaphroditus NC03959	Mustela itatsi NC034330 19	0,1844992
Vulpes ferrilata NC027935 1	Phoca vitulina NC001325 1	0,18450065
Leopardus pardalis NC028315 1	Arctocephalus australis MG023139 1	0,18450125
Leopardus pardalis T262 1	Arctocephalus australis MG023139 1	0,18450157
Martes zibellina NC011579 39	Caracal caracal NC028306 1	0,18450217
Prionailurus planiceps NC028312 6	Lontra canadensis SRR10409165 1	0,18450221
Pusa hispida NC 008433 1	Paradoxurus hermaphroditus NC03959	0,18450238
Felis silvestris lybica KP202275 4	Arctocephalus pusillus NC008417 1	0,18450569
Ursus arctos EU497665 29	Genetta servalina NC024568 2	0,18450719
Paradoxurus hermaphroditus NC03959	Canis mesomelas KT448280 1	0,18450768
Otocolobus manul NC028323 1	Martes pennanti NC020664 16	0,18450789
Nandinia binotata NC024567 1	Ailuropoda melanoleuca NC009492 5	0,18450814
Mustela nivalis T306 5	Felis catus NC001700 2	0,18450883
Otocyon megalotis SAF1 2	Leopardus jacobita NC028322 1	0,18450962
Otocyon megalotis SAF1 2	Catopuma badia NC028300 1	0,18450968
Otocyon megalotis SAF1 2	Leopardus geoffroyi NC028320 1	0,18451089
Lycaon pictus NC028427 2	Genetta servalina NC024568 2	0,18451367
Ursus thibetanus mupinensis NC00875	Prionailurus planiceps KY682741 4	0,18451372
Ictonyx striatus T299 1	Civettictis civetta GLC19 1	0,18451409
Zalophus wollebaeki SRR4431565 1	Leopardus wiedii NC028318 1	0,18451816
Mustela erminea T305 2	Ailuropoda melanoleuca NC009492 5	0,18451992
Ursus thibetanus formosanus NC0093	Genetta genetta T297 1	0,18452001
Genetta servalina NC024568 2	Chrysocyon brachyurus NC024172 1	0,18452069
Ursus thibetanus laniger MH281753 2	Felis catus NC001700 2	0,18452361
Homotherium latidens MF871702 3	Arctocephalus townsendi NC008420 1	0,18452364
Leopardus pardalis T262 1	Galictis vittata T412 1	0,18452455
Ursus spelaeus NC011112 8	Prionailurus bengalensis NC028301 12	0,18452646
Otaria byronia OTAB 1	Acinonyx jubatus NC005212 3	0,1845268
Smilodon populator MF871700 1	Arctocephalus forsteri KT693377 17	0,18452908
Hemigalus derbyanus MH464791 1	Canis mesomelas KT448280 1	0,1845292
Zalophus californianus NC008416 1	Felis nigripes NC028309 1	0,18453008
Ursus thibetanus formosanus NC0093	Acinonyx jubatus NC005212 3	0,1845392
Spilogale putorius NC010497 1	Martes pennanti NC020664 16	0,18454242
Ursus americanus JX196366 3	Mustela altaica NC021751 1	0,18454449
Mungos mungo/gambianus SRR77048	Arctonyx collaris NC020645 1	0,18455043
Mungos mungo/gambianus SRR77048	Martes zibellina NC011579 39	0,18455151
Mustela nigripes NC024942 1	Urva semitorquata MH464789 1	0,18455282
Mungos mungo/gambianus SRR77048	Arctocephalus australis MG023139 1	0,18455333
Phocarctos hookeri NC008418 1	Mustela sibirica AP017394 11	0,18455346
Mungos mungo MMC7 1	Canis anthus NC027956 2	0,18455476
Mungos mungo MMC7 1	Canis latrans NC008093 7	0,18455495
Mungotictis decemlineata NC027828 1	Meles anakuma NC009677 1	0,18455714
Ursus arctos GU573486 5	Gulo gulo NC009685 3	0,18455768
Leopardus wiedii NC028318 1	Tapirus terrestris T358	0,18455797
Prionailurus bengalensis NC028301 12	Conepatus chinga NC042596 1	0,18455897
Meles meles T303 3	Galerella sanguinea T378 1	0,1845599
Profelis aurata NC028299 1	Lutra lutra NC011358 9	0,18456006
Genetta servalina NC024568 2	Arctocephalus townsendi NC008420 1	0,18456072
Leopardus pardalis NC028315 1	Ailurus fulgens styani NC009691 1	0,18456098
Poecilogale albinucha T602 1	Canis adustus KT448271 1	0,18456307
Ursus thibetanus mupinensis NC00875	Lutra lutra NC011358 9	0,1845635
Melursus ursinus NC009970 2	Hemigalus derbyanus MH464791 1	0,1845638
Ursus arctos GU573491 207	Arctocephalus townsendi NC008420 1	0,18456455
Neovison vison NC020641 3	Cynictis penicillata T375 1	0,18456502
Ursus maritimus GU573488 Svalbard	Arctocephalus australis MG023139 1	0,18456519
Mungos mungo/gambianus SRR77048	Arctodus simus NC011116 1	0,18456542
Profelis aurata NC028299 1	Potos flavus T414 1	0,18456687
Ursus arctos AP012576 6	Martes flavigula NC012141 3	0,18456693
Vulpes ferrilata NC027935 1	Leopardus pardalis NC028315 1	0,18456757
Tremarctos ornatus NC009969 2	Leopardus pardalis NC028315 1	0,1845677
Vulpes lagopus NC026529 3	Puma concolor NC016470 22	0,1845679
Prionodon pardicolor NC024569 2	Mustela sibirica AP017394 11	0,18456812
Leopardus pardalis T262 1	Arctocephalus forsteri KT693377 17	0,18456876
Paradoxurus hermaphroditus NC03959	Canis anthus NC027956 2	0,18456894
Viverra tangalunga MH464792 1	Prionodon pardicolor NC024569 2	0,18456957
Neophoca cinerea NC008419 1	Lynx rufus NC014456 3	0,18456958
Ursus arctos GU573491 207	Meles anakuma NC009677 1	0,18457028
Puma concolor NC016470 22	Canis anthus NC027956 2	0,18457042
Viverricula indica NC025296 2	Melogale moschata NC020644 1	0,18457055
Prionodon pardicolor NC024569 2	Canis latrans NC008093 7	0,18457076

Viverricula indica KX891751 1	Fossa fossana D350 1	0,14328668
Mungos mungo/gambianus SRR7704821 1	Cryptoprocta ferox CFC13 1	0,1432893
Galerella sanguinea T378 1	Eupleres goudotii D128 1	0,143291
Salanoia concolor D378 1	Civettictis civetta GLC19 1	0,14329141
Viverra zibetha T609 1	Felis silvestris lybica KP202275 4	0,14329307
Zalophus californianus NC008416 1	Pusa hispida NC 008433 1	0,14331394
Puma concolor NC016470 22	Hemigalus derbyanus MH464791 1	0,14331591
Salanoia concolor D378 1	Civettictis civetta NC033378 1	0,14332667
Leopardus guigna NC028321 1	Civettictis civetta NC033378 1	0,14332956
Mirounga leonina NC008422 1	Callorhinus ursinus NC008415 1	0,14334252
Homotherium latidens MF871702 3	Genetta genetta T297 1	0,1433488
Viverricula indica KX891745 1	Fossa fossana D350 1	0,14335407
Panthera uncia KP202269 1	Nandinia binotata NC024567 1	0,14335856
Nandinia binotata NC024567 1	Felis chaus NC028307 1	0,14335868
Viverricula indica KX891745 1	Prionailurus planiceps NC028312 6	0,14336
Genetta abyssinica MG489822 1	Felis chaus NC028307 1	0,14336026
Lynx canadensis NC028313 1	Genetta servalina NC024568 2	0,1433603
Genetta servalina NC024568 2	Caracal caracal NC028306 1	0,14336154
Prionodon pardicolor NC024569 2	Leopardus wiedii NC028318 1	0,14337368
Genetta abyssinica MG489822 1	Cynogale bennetti KY117544 1	0,1433793
Catopuma temminckii NC027115 41	Arctictis binturong T605 2	0,14337988
Pusa hispida NC 008433 1	Eumetopias jubatus NC004030 10	0,14339508
Otaria byronia OTAB 1	Leptonychotes weddellii NC008424 1	0,14341682
Viverra zibetha T609 1	Pardofelis marmorata NLN3 2	0,14342773
Viverricula indica KX891751 1	Profelis aurata NC028299 1	0,14342777
Viverricula indica KX891745 1	Felis margarita NC028308 1	0,14342779
Leopardus wiedii NC028318 1	Genetta genetta T297 1	0,1434292
Lobodon carcinophaga NC008423 1	Arctocephalus pusillus NC008417 1	0,14342937
Paradoxurus hermaphroditus NLNC 1	Catopuma badia NC028300 1	0,14343436
Urocyon cinereoargenteus NC026723 21	Canis lupus familiaris NC002008 1231	0,14343607
Prionailurus bengalensis CKM45 20	Arctictis binturong T605 2	0,14344384
Salanoia concolor D378 1	Nandinia binotata NC024567 1	0,14348531
Helogale parvula SRR7637809 1	Galidictis fasciata DM333 1	0,14348558
Mungos mungo MMC7 1	Eupleres goudotii D128 1	0,14348562
Mungotictis decemlineata NC027828 1	Urva javanica T413 1	0,14348608
Genetta genetta T297 1	Fossa fossana D350 1	0,14349014
Viverricula indica KX891751 1	Leopardus tigrinus NC028317 1	0,14349343
Prionailurus bengalensis NC028301 12	Paradoxurus hermaphroditus NLNC 1	0,14349374
Lynx pardinus NC028319 161	Genetta genetta T297 1	0,1434963
Panthera onca NC022842 1	Nandinia binotata NC024567 1	0,14350189
Vulpes corsac NC023958 1	Cuon alpinus NC013445 3	0,14350547
Vulpes vulpes NC008434 5	Cuon alpinus NC013445 3	0,14350575
Viverra tangalunga MH464792 1	Galictis vittata T412 1	0,14351076
Salanoia concolor D378 1	Genetta abyssinica MG489822 1	0,14352354
Phoca fasciata NC008428 1	Eumetopias jubatus NC004030 10	0,14352648
Hydrurga leptonyx NC008425 1	Arctocephalus australis MG023139 1	0,14355886
Leopardus wiedii NC028318 1	Fossa fossana D350 1	0,14356245
Leopardus jacobita NC028322 1	Civettictis civetta GLC19 1	0,14356329
Melogale moschata KP726273 1	Galictis vittata T412 1	0,14356592
Lutrogale perspicillata NC035811 1	Arctonyx collaris NC020645 1	0,14361493
Paguma larvata PDDS11 2	Leopardus pardalis NC028315 1	0,14362757
Viverricula indica KX891751 1	Felis nigripes NC028309 1	0,14362913
Leopardus tigrinus NC028317 1	Civettictis civetta GLC19 1	0,14363045
Leopardus guigna NC028321 1	Civettictis civetta GLC19 1	0,14363407
Viverra zibetha T609 1	Leopardus guigna NC028321 1	0,14363441
Genetta abyssinica MG489822 1	Felis silvestris lybica KP202275 4	0,14364217
Taxidea taxus NC020646 1	Poecilogale albinucha T602 1	0,14364518
Genetta abyssinica MG489822 1	Felis margarita NC028308 1	0,14364547
Monachus monachus NC044972 5	Callorhinus ursinus NC008415 1	0,14364867
Hemigalus derbyanus MH464791 1	Felis nigripes NC028309 1	0,14367661
Prionailurus rubiginosus NC028304 2	Fossa fossana D350 1	0,14369465
Profelis aurata NC028299 1	Nandinia binotata NC024567 1	0,14369518
Prionailurus rubiginosus NC028304 2	Paguma larvata PDDS11 2	0,1436968
Lynx lynx NC027083 4	Genetta servalina NC024568 2	0,1436969
Hydrurga leptonyx NC008425 1	Arctocephalus forsteri NC004023 28	0,14374806
Prionailurus planiceps NC028312 6	Hemigalus derbyanus MH464791 1	0,14375238
Helogale parvula SRR7637809 1	Eupleres goudotii D128 1	0,14375271
Vulpes vulpes NC008434 5	Chrysocyon brachyurus NC024172 1	0,14375703
Parahyaena brunnea NC038159 15	Fossa fossana D350 1	0,14376573
Felis margarita NC028308 1	Arctictis binturong T605 2	0,14377691
Prionodon pardicolor NC024569 2	Leopardus jacobita NC028322 1	0,14377786
Mustela nivalis T306 5	Galictis vittata T412 1	0,1437858
Leopardus guigna NC028321 1	Genetta abyssinica MG489822 1	0,14378671
Profelis aurata NC028299 1	Civettictis civetta NC033378 1	0,14379783
Vulpes corsac NC023958 1	Lycalopex sechurae KT448284 1	0,14382173
Mirounga leonina NC008422 1	Arctocephalus pusillus NC008417 1	0,14382233
Viverra zibetha T609 1	Fossa fossana D350 1	0,14382561
Hemigalus derbyanus MH464791 1	Felis margarita NC028308 1	0,14383145
Leopardus pardalis NC028315 1	Fossa fossana D350 1	0,14383157
Halichoerus grypus NC001602 2	Callorhinus ursinus NC008415 1	0,14385632
Homotherium latidens MF871702 3	Chrotogale owstoni T607 1	0,14386177
Phoca vitulina NC001325 1	Callorhinus ursinus NC008415 1	0,14389476
Panthera uncia NC010638 1	Nandinia binotata NC024567 1	0,14389709
Martes americana NC020642 1	Galictis vittata T412 1	0,14390272
Leptailurus serval NC028316 1	Arctictis binturong T605 2	0,14391187
Leopardus colocolo NC028314 1	Fossa fossana D350 1	0,14391445
Viverricula indica NC025296 2	Smilodon populator MF871700 1	0,14396206
Viverra zibetha T609 1	Leopardus tigrinus NC028317 1	0,14396594
Viverra zibetha T609 1	Felis nigripes NC028309 1	0,1439666
Lynx rufus NC014456 3	Genetta servalina NC024568 2	0,14396665
Viverricula indica KX891745 1	Leopardus guigna NC028321 1	0,14397095
Viverricula indica KX891751 1	Catopuma temminckii NC027115 41	0,14397104
Vulpes zerda KJ603240 1	Cuon alpinus NC013445 3	0,14397703
Pusa caspica NC008431 1	Eumetopias jubatus NC004030 10	0,14399338
Panthera leo NERO 19	Nandinia binotata NC024567 1	0,14399496
Urocyon cinereoargenteus NC026723 21	Canis anthus NC027956 2	0,1440113
Vulpes lagopus NC026529 3	Urocyon cinereoargenteus NC026723 21	0,1440113
Mirounga leonina NC008422 1	Arctocephalus forsteri KT693377 17	0,14401746
Vulpes lagopus NC026529 3	Lycalopex sechurae KT448284 1	0,14401762
Nyctereutes procyonoides NC013700 3	Lycaon pictus NC028427 2	0,1440179
Nandinia binotata NC024567 1	Urva semitorquata MH464789 1	0,144021
Profelis aurata NC028299 1	Hemigalus derbyanus MH464791 1	0,14402145
Halichoerus grypus NC001602 2	Eumetopias jubatus NC004030 10	0,14402184

Prionailurus bengalensis CKM45 20	Arctocephalus gazella BK010918 1	0,1845722
Lycaon pictus NC028427 2	Arctonyx collaris NC020645 1	0,18457289
Prionailurus viverrinus NC028305 1	Neophoca cinerea NC008419 1	0,18457311
Panthera tigris amoyensis NC014770 2	Callorhinus ursinus NC008415 1	0,18457378
Panthera onca NC022842 1	Lutra lutra NC011358 9	0,18457399
Procyon lotor AB462049 4	Prionailurus viverrinus NC028305 1	0,1845745
Otaria byronia OTAB 1	Leopardus pardalis T262 1	0,18457656
Speothos venaticus C48 2	Phoca vitulina NC001325 1	0,18457852
Prionailurus bengalensis CKM45 20	Arctotherium sp NC030174 1	0,18457896
Ursus thibetanus thibetanus NC01111	Crossarchus platycephalus C7R66 1	0,18457998
Felis chaus NC028307 1	Bassariscus sumichrasti SRX1099089 1	0,18458004
Paradoxurus jerdoni MH464793 1	Ictonyx striatus T299 1	0,18458183
Zalophus wollebaeki SRR4431565 1	Lynx lynx NC027083 4	0,18458231
Melogale moschata NC020644 1	Arctictis binturong T605 2	0,18458256
Ursus spelaeus EU327344 13	Arctocephalus australis MG023139 1	0,18458268
Zalophus californianus NC008416 1	Bassaricyon neblina SRX1097850 1	0,18458317
Zalophus wollebaeki SRR4431565 1	Felis silvestris lybica KP202275 4	0,18458362
Zalophus wollebaeki SRR4431565 1	Prionailurus planiceps NC028312 6	0,18458369
Ursus thibetanus formosanus NC0093	Puma concolor NC016470 22	0,18459097
Ursus spelaeus NC011112 8	Felis silvestris lybica KP202275 4	0,18459162
Lutra lutra NC011358 9	Civettictis civetta NC033378 1	0,18459302
Prionailurus planiceps KY682741 4	Arctocephalus forsteri NC004023 28	0,18459862
Viverricula indica KX891751 1	Gulo gulo NC009685 3	0,18459952
Ursus thibetanus laniger MH281753 2	Ictonyx striatus T299 1	0,18460065
Lynx canadensis NC028313 1	Lycalopex sechurae KT448284 1	0,1846008
Smilodon populator MF871700 1	Arctocephalus australis MG023139 1	0,18460242
Felis chaus NC028307 1	Canis lupus familiaris NC002008 1231	0,18460336
Phoca largha NC008430 1	Cuon alpinus NC013445 3	0,18460783
Ursus thibetanus laniger MH281753 2	Canis aureus KT448274 1	0,18460897
Lutra lutra NC011358 9	Urva semitorquata MH464789 1	0,18461406
Speothos venaticus C48 2	Halichoerus grypus NC001602 2	0,18461408
Lutra lutra NC011358 9	Canis latrans NC008093 7	0,18461561
Lutra lutra NC011358 9	Eupleres goudotii D128 1	0,18461753
Melogale moschata KP726273 1	Crossarchus platycephalus C7R66 1	0,18461765
Mustela putorius NC020638 4	Arctocephalus forsteri KT693377 17	0,18461874
Zalophus californianus NC008416 1	Leopardus colocolo NC028314 1	0,18461934
Mustela kathiah NC023210 1	Mungos mungo/gambianus SRR77048	0,18461992
Melogale moschata V0735A 1	Chrotogale owstoni T607 1	0,18462023
Mustela kathiah NC023210 1	Urva brachyura KY117547 1	0,18462104
Mustela sibirica NC020637 6	Urva javanica T413 1	0,1846212
Viverra tangalunga MH464792 1	Xenogale naso C07XAR110 1	0,18462157
Felis catus NC001700 2	Canis mesomelas KT448280 1	0,18462203
Vulpes corsac NC023958 1	Crossarchus platycephalus C7R66 1	0,18462253
Paradoxurus hermaphroditus NLNC 1	Callorhinus ursinus NC008415 1	0,18462257
Meles meles T303 3	Galidia elegans D146 1	0,18462282
Procyon lotor AB462046 3	Arctocephalus forsteri KT693377 17	0,18462297
Catopuma temminckii NC027115 41	Canis mesomelas KT448280 1	0,18462315
Phoca groenlandica NC008429 54	Tapirus terrestris T358	0,18462369
Spilogale putorius NC010497 1	Arctictis binturong T605 2	0,18462451
Tremarctos ornatus NC009969 2	Mustela sibirica AP017394 11	0,18462466
Lutrogale perspicillata NC035811 1	Callorhinus ursinus NC008415 1	0,18462496
Mungotictis decemlineata NC027828 1	Aonyx cinerea NC035814 2	0,18462513
Phocarctos hookeri NC008418 1	Lutrogale perspicillata NC035811 1	0,18462521
Lutra lutra LC050126 1	Genetta genetta T297 1	0,18462534
Vulpes vulpes NC008434 5	Viverra tangalunga MH464792 1	0,18462618
Galidictis fasciata DM333 1	Arctocephalus pusillus NC008417 1	0,18462801
Otaria byronia OTAB 1	Mungos mungo MMC7 1	0,18462847
Genetta servalina NC024568 2	Arctocephalus australis MG023139 1	0,18462867
Ursus arctos pruinosus MG066703 3	Eumetopias jubatus NC004030 10	0,1846302
Hemigalus derbyanus MH464791 1	Arctocephalus pusillus NC008417 1	0,18463089
Mungotictis decemlineata NC027828 1	Martes pennanti NC020664 16	0,18463093
Phocarctos hookeri NC008418 1	Mustela nivalis T306 5	0,18463103
Ursus maritimus GU573488 Svalbard	Canis adustus KT448271 1	0,18463105
Viverricula indica KX891751 1	Mustela eversmannii NC028013 1	0,18463163
Cuon alpinus NC013445 3	Acinonyx jubatus NC005212 3	0,18463245
Ursus arctos GU573491 207	Melogale moschata V0735A 1	0,18463278
Potos flavus T414 1	Felis catus NC001700 2	0,1846337
Ursus arctos EU497665 29	Arctocephalus gazella BK010918 1	0,18463403
Speothos venaticus C48 2	Nandinia binotata NC024567 1	0,1846341
Prionailurus planiceps NC028312 6	Melogale moschata V0735A 1	0,1846347
Vulpes lagopus NC026529 3	Prionailurus viverrinus NC028305 1	0,18463503
Profelis aurata NC028299 1	Mustela erminea T305 2	0,18463594
Leopardus guigna NC028321 1	Ailurus fulgens styani NC009691 1	0,18463625
Viverricula indica NC025296 2	Tremarctos ornatus NC009969 2	0,18463692
Viverricula indica KX891745 1	Arctocephalus pusillus NC008417 1	0,18463811
Phocarctos hookeri NC008418 1	Leopardus tigrinus NC028317 1	0,18463875
Neophoca cinerea NC008419 1	Leopardus pardalis T262 1	0,18463902
Otaria byronia OTAB 1	Lynx lynx NC027083 4	0,1846396
Poecilogale albinucha T602 1	Lynx canadensis NC028313 1	0,18464085
Prionodon pardicolor NC024569 2	Mustela altaica NC021751 1	0,18464106
Ursus arctos isabellinus 18B5 2	Otaria byronia OTAB 1	0,18464394
Smilodon populator MF871700 1	Neophoca cinerea NC008419 1	0,18464421
Mustela erminea T305 2	Chrysocyon brachyurus NC024172 1	0,18464449
Ursus maritimus NC003428 31	Poecilogale albinucha T602 1	0,18464538
Ursus arctos isabellinus 18B5 2	Poecilogale albinucha T602 1	0,18464558
Lycalopex sechurae KT448284 1	Ailurus fulgens styani NC009691 1	0,18464607
Lycaon pictus NC028427 2	Leopardus jacobita NC028322 1	0,18464866
Chrotogale owstoni T607 1	Canis aureus KT448274 1	0,1846493
Ursus thibetanus mupinensis NC00875	Prionailurus bengalensis NC028301 12	0,18464975
Arctocephalus australis MG023139 1	Acinonyx jubatus NC005212 3	0,18465066
Odobenus rosmarus NC004029 29	Arctotherium sp NC030174 1	0,18465124
Zalophus wollebaeki SRR4431565 1	Caracal caracal NC028306 1	0,18465129
Melogale moschata V0735A 1	Acinonyx jubatus NC005212 3	0,18465154
Viverra tangalunga MH464792 1	Ursus thibetanus laniger MH281753 2	0,18465196
Mustela putorius NC020638 4	Ailuropoda melanoleuca NC009492 5	0,1846524
Vulpes zerda KJ603240 1	Leopardus colocolo NC028314 1	0,18465483
Tremarctos ornatus NC009969 2	Odobenus rosmarus NC004029 29	0,18465541
Ursus thibetanus formosanus NC0093	Lynx rufus NC014456 3	0,1846564
Mephitis mephitis NC020648 1	Martes martes T302 3	0,18465707
Ursus thibetanus laniger MH281753 2	Genetta genetta T297 1	0,18465725
Poecilogale albinucha T602 1	Acinonyx jubatus NC005212 3	0,18465772
Ursus thibetanus formosanus NC0093	Leopardus tigrinus NC028317 1	0,18465781
Lynx lynx NC027083 4	Chrysocyon brachyurus NC024172 1	0,18465982

Urva brachyura KY117547 1	Galidictis fasciata DM333 1	0,14402558
Vulpes corsac NC023958 1	Chrysocyon brachyurus NC024172 1	0,14402673
Prionailurus planiceps KY682741 4	Genetta genetta T297 1	0,14403594
Leopardus geoffroyi NC028320 1	Genetta servalina NC024568 2	0,14403845
Urocyon littoralis catalinae KP129018 15	Canis lupus familiaris NC002008 1231	0,14404221
Eupleres goudotii D128 1	Bdeogale nigripes GLC15 1	0,14408854
Galidia elegans D146 1	Atilax paludinosus T606 1	0,14409043
Viverricula indica KX891745 1	Felis catus NC001700 2	0,14410082
Viverricula indica KX891751 1	Felis chaus NC028307 1	0,14410108
Viverricula indica NC025296 2	Felis catus NC001700 2	0,1441043
Paradoxurus jerdoni MH464793 1	Homotherium latidens MF871702 3	0,14410508
Zalophus californianus NC008416 1	Erignathus barbatus NC008426 1	0,14410993
Leopardus tigrinus NC028317 1	Civettictis civetta NC033378 1	0,14413436
Urocyon littoralis catalinae KP129018 15	Canis anthus NC027956 2	0,14414597
Mungos mungo/gambianus SRR7704821 1	Galidia elegans D146 1	0,14415851
Prionailurus bengalensis NC028301 12	Fossa fossana D350 1	0,14416611
Prionailurus planiceps KY682741 4	Paguma larvata PDDS11 2	0,14416794
Paguma larvata PDDS11 2	Otocolobus manul NC028323 1	0,14416867
Leopardus pardalis NC028315 1	Genetta genetta T297 1	0,14416958
Meles leucurus NC039173 4	Galictis vittata T412 1	0,14417131
Viverricula indica NC025296 2	Felis margarita NC028308 1	0,14417189
Paguma larvata PDDS11 2	Acinonyx jubatus NC005212 3	0,14417949
Prionodon pardicolor NC024569 2	Leopardus geoffroyi NC028320 1	0,14418179
Martes pennanti NC020664 16	Galictis vittata T412 1	0,14418246
Prionodon pardicolor NC024569 2	Leopardus colocolo NC028314 1	0,14419755
Profelis aurata NC028299 1	Genetta abyssinica MG489822 1	0,14419869
Lobodon carcinophaga NC008423 1	Arctocephalus forsteri NC004023 28	0,1442188
Lynx rufus NC014456 3	Fossa fossana D350 1	0,14423311
Paguma larvata PDDS11 2	Leopardus wiedii NC028318 1	0,14423393
Prionailurus planiceps NC028312 6	Paguma larvata PDDS11 2	0,14423518
Mustela altaica NC021751 1	Meles anakuma NC009677 1	0,14423583
Viverra zibetha T609 1	Felis margarita NC028308 1	0,14423592
Genetta servalina NC024568 2	Felis catus NC001700 2	0,1442364
Urocyon cinereoargenteus NC026723 21	Canis lupus chanco NC010340 4	0,14423761
Leopardus guigna NC028321 1	Genetta servalina NC024568 2	0,1442405
Smilodon populator MF871700 1	Hemigalus derbyanus MH464791 1	0,14426425
Vulpes lagopus NC026529 3	Urocyon littoralis catalinae KP129018 15	0,14428061
Meles meles T303 3	Lutrogale perspicillata NC035811 1	0,14428809
Lobodon carcinophaga NC008423 1	Arctocephalus gazella BK010918 1	0,14429949
Hyaena hyaena NC020669 1	Fossa fossana D350 1	0,14430372
Lynx canadensis NC028313 1	Genetta genetta T297 1	0,14430419
Viverricula indica NC025296 2	Lynx pardinus NC028319 161	0,14430653
Zalophus wollebaeki SRR4431565 1	Pusa hispida NC 008433 1	0,14431332
Prionailurus planiceps KY682741 4	Hemigalus derbyanus MH464791 1	0,14431819
Leopardus jacobita NC028322 1	Civettictis civetta NC033378 1	0,1443366
Mirounga leonina NC008422 1	Arctocephalus gazella BK010918 1	0,14435446
Zalophus wollebaeki SRR4431565 1	Erignathus barbatus NC008426 1	0,14436781
Mungos mungo MMC7 1	Genetta abyssinica MG489822 1	0,14439161
Hemigalus derbyanus MH464791 1	Felis silvestris lybica KP202275 4	0,14440638
Leptonychotes weddellii NC008424 1	Arctocephalus forsteri NC004023 28	0,1444129
Vulpes ferrilata NC027935 1	Lycalopex sechurae KT448284 1	0,14442685
Salanoia concolor D378 1	Urva brachyura KY117547 1	0,14442918
Phoca largha NC008430 1	Callorhinus ursinus NC008415 1	0,14443353
Hydrurga leptonyx NC008425 1	Arctocephalus townsendi NC008420 1	0,14443387
Viverricula indica KX891751 1	Prionailurus planiceps NC028312 6	0,14443691
Viverricula indica KX891751 1	Felis silvestris lybica KP202275 4	0,14443716
Urocyon cinereoargenteus NC026723 21	Canis aureus KT448274 1	0,14443775
Pardofelis marmorata NLN3 2	Genetta servalina NC024568 2	0,14443852
Lynx rufus NC014456 3	Genetta genetta T297 1	0,14443922
Prionailurus rubiginosus NC028304 2	Arctictis binturong T605 2	0,14445071
Mirounga angustirostris SRR10331586 1	Eumetopias jubatus NC004030 10	0,14446919
Lynx rufus NC014456 3	Hemigalus derbyanus MH464791 1	0,14447008
Viverricula indica NC025296 2	Prionailurus planiceps KY682741 4	0,14450833
Prionailurus viverrinus NC028305 1	Arctictis binturong T605 2	0,14451677
Puma concolor NC016470 22	Arctictis binturong T605 2	0,1445179
Vulpes vulpes NC008434 5	Urocyon cinereoargenteus NC026723 21	0,14454992
Viverricula indica KX891751 1	Homotherium latidens MF871702 3	0,14456677
Lobodon carcinophaga NC008423 1	Arctocephalus australis MG023139 1	0,14456847
Panthera onca KP202264 2	Civettictis civetta GLC19 1	0,14457355
Urocyon littoralis catalinae KP129018 15	Canis lupus chanco NC010340 4	0,14457425
Otocolobus manul NC028323 1	Genetta genetta T297 1	0,14457476
Viverricula indica NC025296 2	Prionailurus planiceps NC028312 6	0,14457567
Genetta genetta T297 1	Catopuma badia NC028300 1	0,14457674
Prionodon pardicolor NC024569 2	Leptailurus serval NC028316 1	0,14458272
Felis chaus NC028307 1	Arctictis binturong T605 2	0,14458512
Pardofelis marmorata NLN3 2	Civettictis civetta NC033378 1	0,14460325
Urva brachyura KY117547 1	Hemigalus derbyanus MH464791 1	0,14460854
Vulpes zerda KJ603240 1	Lycaon pictus NC028427 2	0,14462306
Urva javanica T413 1	Galidictis fasciata DM333 1	0,14463136
Lynx canadensis NC028313 1	Civettictis civetta GLC19 1	0,14463963
Pardofelis marmorata NLN3 2	Civettictis civetta GLC19 1	0,14464039
Viverricula indica NC025296 2	Lynx canadensis NC028313 1	0,14464302
Salanoia concolor D378 1	Leopardus jacobita NC028322 1	0,14464507
Viverricula indica KX891745 1	Homotherium latidens MF871702 3	0,14466055
Pusa sibirica NC008432 2	Eumetopias jubatus NC004030 10	0,14466701
Urva semitorquata MH464789 1	Galidia elegans D146 1	0,14469638
Mungotictis decemlineata NC027828 1	Civettictis civetta GLC19 1	0,14470515
Viverricula indica KX891745 1	Lynx lynx NC027083 4	0,14470607
Viverra zibetha T609 1	Lynx canadensis NC028313 1	0,14470622
Viverricula indica KX891751 1	Caracal caracal NC028306 1	0,14470693
Leopardus tigrinus NC028317 1	Genetta servalina NC024568 2	0,14470728
Monachus schauinslandi NC008421 1	Arctocephalus pusillus NC008417 1	0,14470908
Taxidea taxus NC020646 1	Lontra canadensis SRR10409165 1	0,14471093
Vulpes lagopus NC026529 3	Cuon alpinus NC013445 3	0,14471334
Lynx lynx NC027083 4	Hemigalus derbyanus MH464791 1	0,14474517
Paradoxurus hermaphroditus NC039591 1	Leptailurus serval NC028316 1	0,14477314
Vulpes vulpes NC008434 5	Urocyon littoralis catalinae KP129018 15	0,14481923
Lynx pardinus NC028319 161	Fossa fossana D350 1	0,14483899
Panthera onca KP202264 2	Nandinia binotata NC024567 1	0,14483993
Viverricula indica KX891751 1	Prionailurus planiceps KY682741 4	0,14484096
Panthera leo NERO 19	Civettictis civetta NC033378 1	0,14484195
Otaria byronia OTAB 1	Hydrurga leptonyx NC008425 1	0,14484303
Zalophus californianus NC008416 1	Pusa caspica NC008431 1	0,14485417

Ursus thibetanus thibetanus NC011118	Leopardus jacobita NC028322 1	0,18465991
Canis mesomelas KT448280 1	Arctonyx collaris NC020645 1	0,18466166
Ursus americanus JX196366 3	Melogale moschata V0735A 1	0,18466672
Ictonyx striatus T299 1	Arctocephalus forsteri NC004023 28	0,18466896
Salanoia concolor D378 1	Eumetopias jubatus NC004030 10	0,18467097
Martes flavigula NC012141 3	Chrysocyon brachyurus NC024172 1	0,18467224
Smilodon populator MF871700 1	Mustela eversmannii NC028013 1	0,1846723
Mephitis mephitis NC020648 1	Genetta abyssinica MG489822 1	0,18467356
Paradoxurus jerdoni MH464793 1	Eumetopias jubatus NC004030 10	0,18467483
Gulo gulo NC009685 3	Catopuma temminckii NC027115 41	0,18467724
Prionailurus bengalensis CKM45 20	Canis mesomelas KT448280 1	0,18468358
Meles meles T303 3	Helogale parvula SRR7637809 1	0,18468522
Puma yagouaroundi NC028311 1	Cuon alpinus NC013445 3	0,18468538
Ursus thibetanus thibetanus NC011118	Canis lupus familiaris NC002008 1231	0,18468565
Martes zibellina NC011579 39	Urva semitorquata MH464789 1	0,18468659
Mustela putorius NC020638 4	Urva semitorquata MH464789 1	0,18468715
Martes zibellina NC011579 39	Urva javanica T413 1	0,18468732
Mustela putorius NC020638 4	Xenogale naso C07XAR110 1	0,18468833
Phocarctos hookeri NC008418 1	Mustela nigripes NC024942 1	0,18468888
Procyon lotor AB462046 3	Arctocephalus australis MG023139 1	0,18469058
Salanoia concolor D378 1	Callorhinus ursinus NC008415 1	0,18469131
Martes martes T302 3	Urva javanica T413 1	0,18469185
Martes martes T302 3	Atilax paludinosus T606 1	0,18469276
Viverra tangalunga MH464792 1	Salanoia concolor D378 1	0,18469322
Mustela eversmannii NC028013 1	Canis anthus NC027956 2	0,18469395
Martes zibellina NC011579 39	Galerella sanguinea T378 1	0,18469627
Urva brachyura KY117547 1	Bassariscus sumichrasti SRX1099089 1	0,18469667
Paradoxurus hermaphroditus NLNC 1	Melogale moschata KP726273 1	0,18469681
Mustela altaica NC021751 1	Arctocephalus forsteri KT693377 17	0,18469696
Mustela nivalis T306 5	Mungos mungo/gambianus SRR77048	0,18469732
Ursus arctos AP012576 6	Gulo gulo NC009685 3	0,18469818
Ursus arctos isabellinus 18B5 2	Arctocephalus townsendi NC008420 1	0,18469895
Ursus arctos isabellinus 18B5 2	Martes pennanti NC020664 16	0,1846998
Paradoxurus hermaphroditus NC03959	Mustela eversmannii NC028013 1	0,18469998
Profelis aurata NC028299 1	Ailurus fulgens NC011124 1	0,18470016
Pardofelis marmorata NLN3 2	Mustela sibirica NC020637 6	0,18470021
Paradoxurus hermaphroditus NC03959	Mustela sibirica AP017394 11	0,18470033
Lycalopex sechurae KT448284 1	Arctodus simus NC011116 1	0,18470043
Viverricula indica KX891751 1	Tremarctos ornatus NC009969 2	0,18470202
Genetta genetta T297 1	Ailurus fulgens NC011124 1	0,18470214
Vulpes ferrilata NC027935 1	Felis chaus NC028307 1	0,18470226
Neophoca cinerea NC008419 1	Civettictis civetta GLC19 1	0,1847025
Melogale moschata V0735A 1	Catopuma temminckii NC027115 41	0,18470278
Fossa fossana D350 1	Canis aureus KT448274 1	0,18470374
Leopardus pardalis T262 1	Canis anthus NC027956 2	0,18470392
Ursus arctos pruinosus MG066703 3	Callorhinus ursinus NC008415 1	0,18470411
Vulpes zerda KJ603240 1	Leopardus tigrinus NC028317 1	0,18470411
Lontra canadensis SRR10409165 1	Leopardus geoffroyi NC028320 1	0,18470521
Prionailurus bengalensis NC028301 12	Phocarctos hookeri NC008418 1	0,18470567
Ursus arctos GU573486 5	Martes pennanti NC020664 16	0,18470639
Procyon lotor AB462046 3	Paradoxurus jerdoni MH464793 1	0,18470782
Ichneumia albicauda T603 1	Arctotherium sp NC030174 1	0,18471029
Otaria byronia OTAB 1	Leopardus tigrinus NC028317 1	0,18471171
Pardofelis marmorata NLN3 2	Neovison vison NC020641 3	0,18471199
Melogale moschata V0735A 1	Homotherium latidens MF871702 3	0,18471213
Ursus americanus JX196366 3	Canis adustus KT448271 1	0,18471366
Ursus thibetanus laniger MH281753 2	Mungotictis decemlineata NC027828 1	0,1847143
Mustela sibirica NC020637 6	Arctictis binturong T605 2	0,1847145
Prionodon pardicolor NC024569 2	Martes foina NC020643 1	0,18471561
Speothos venaticus C48 2	Puma yagouaroundi NC028311 1	0,18471648
Mustela altaica NC021751 1	Arctictis binturong T605 2	0,18471652
Taxidea taxus NC020646 1	Hyaena hyaena NC020669 1	0,18471658
Mephitis mephitis NC020648 1	Martes americana NC020642 1	0,18471667
Urva javanica T413 1	Canis lupus chanco NC010340 4	0,1847194
Ursus spelaeus EU327344 13	Lynx lynx NC027083 4	0,18472105
Ursus spelaeus EU327344 13	Leopardus wiedii NC028318 1	0,18472141
Ursus spelaeus EU327344 13	Caracal caracal NC028306 1	0,18472314
Meles leucurus NC039173 4	Canis lupus familiaris NC002008 1231	0,18472353
Ursus spelaeus NC011112 8	Lynx pardinus NC028319 161	0,18472625
Procyon lotor AB462049 4	Genetta abyssinica MG489822 1	0,18472679
Ursus arctos AP012576 6	Poecilogale albinucha T602 1	0,1847274
Leopardus colocolo NC028314 1	Arctocephalus pusillus NC008417 1	0,18472861
Vulpes lagopus NC026529 3	Hemigalus derbyanus MH464791 1	0,18472911
Ursus thibetanus thibetanus NC011118	Lycaon pictus NC028427 2	0,1847304
Leopardus guigna NC028321 1	Canis aureus KT448274 1	0,18473119
Lycaon pictus NC028427 2	Arctictis binturong T605 2	0,18473236
Zalophus californianus NC008416 1	Ursus arctos pruinosus MG066703 3	0,18473265
Zalophus californianus NC008416 1	Catopuma temminckii NC027115 41	0,18473357
Ictonyx striatus T299 1	Acinonyx jubatus NC005212 3	0,18473448
Prionailurus planiceps NC028312 6	Arctocephalus forsteri NC004023 28	0,1847349
Spilogale putorius NC010497 1	Melogale moschata NC020644 1	0,18473583
Lynx rufus NC014456 3	Canis lupus familiaris NC002008 1231	0,18473674
Civettictis civetta GLC19 1	Canis mesomelas KT448280 1	0,18474174
Zalophus californianus NC008416 1	Hemigalus derbyanus MH464791 1	0,18474601
Smilodon populator MF871700 1	Martes pennanti NC020664 16	0,18474773
Puma yagouaroundi NC028311 1	Eumetopias jubatus NC004030 10	0,18474795
Mungos mungo/gambianus SRR77048	Martes melampus NC009678 1	0,1847485
Martes melampus NC009678 1	Cynictis penicillata T375 1	0,18475115
Viverricula indica KX891751 1	Lutra lutra NC011358 9	0,18475308
Mustela sibirica NC020637 6	Mungos mungo MMC7 1	0,18475381
Mustela nigripes NC024942 1	Mungos mungo/gambianus SRR77048	0,18475462
Neophoca cinerea NC008419 1	Crossarchus platycephalus C7R66 1	0,18475577
Prionodon linsang ERR2391707 1	Meles leucurus NC039173 4	0,18475579
Meles leucurus NC039173 4	Galidictis fasciata DM333 1	0,18475732
Gulo gulo NC009685 3	Acinonyx jubatus NC005212 3	0,18475801
Salanoia concolor D378 1	Meles anakuma NC009677 1	0,18475842
Eupleres goudotii D128 1	Arctocephalus pusillus NC008417 1	0,18475884
Leopardus jacobita NC028322 1	Conepatus chinga NC042596 1	0,18475926
Vulpes corsac NC023958 1	Mungotictis decemlineata NC027828 1	0,18476011
Panthera onca KP202264 2	Meles leucurus NC039173 4	0,18476288
Pardofelis marmorata NLN3 2	Meles leucurus NC039173 4	0,1847629
Civettictis civetta GLC19 1	Arctocephalus forsteri KT693377 17	0,18476437
Meles leucurus NC039173 4	Ichneumia albicauda T603 1	0,18476543

Viverricula indica KX891751 1	Smilodon populator MF871700 1	0,14485513
Poecilogale albinucha T602 1	Galictis vittata T412 1	0,1448562
Taxidea taxus NC020646 1	Aonyx cinerea NC035814 2	0,1448563
Nandinia binotata NC024567 1	Hemigalus derbyanus MH464791 1	0,14486863
Hemigalus derbyanus MH464791 1	Felis catus NC001700 2	0,14488131
Phocarctos hookeri NC008418 1	Leptonychotes weddellii NC008424 1	0,14489262
Leptonychotes weddellii NC008424 1	Arctocephalus townsendi NC008420 1	0,14489335
Paguma larvata PDDS11 2	Nandinia binotata NC024567 1	0,14490277
Cryptoprocta ferox CFC13 1	Atilax paludinosus T606 1	0,14490487
Lutrogale perspicillata NC035811 1	Galictis vittata T412 1	0,14491133
Zalophus californianus NC008416 1	Mirounga angustirostris SRR10331586 1	0,14492962
Mungotictis decemlineata NC027828 1	Civettictis civetta NC033378 1	0,14494228
Urva brachyura KY117547 1	Eupleres goudotii D128 1	0,14496379
Xenogale naso C07XAR110 1	Galidia elegans D146 1	0,14496599
Vulpes lagopus NC026529 3	Chrysocyon brachyurus NC024172 1	0,14496891
Prionailurus viverrinus NC028305 1	Hemigalus derbyanus MH464791 1	0,14497469
Viverricula indica KX891745 1	Lynx pardinus NC028319 161	0,14497574
Prionailurus bengalensis CKM45 20	Fossa fossana D350 1	0,1449776
Otaria byronia OTAB 1	Lobodon carcinophaga NC008423 1	0,14497906
Felis silvestris lybica KP202275 4	Arctictis binturong T605 2	0,14498901
Otocolobus manul NC028323 1	Arctictis binturong T605 2	0,14498961
Phoca groenlandica NC008429 54	Eumetopias jubatus NC004030 10	0,14500916
Prionailurus bengalensis CKM45 20	Diplogale hosei MH464790 1	0,14501067
Felis silvestris lybica KP202275 4	Chrotogale owstoni T607 1	0,14503402
Salanoia concolor D378 1	Urva javanica T413 1	0,14503498
Urocyon littoralis catalinae KP129018 15	Canis aureus KT448274 1	0,145045
Puma concolor NC016470 22	Genetta genetta T297 1	0,14504558
Viverricula indica KX891751 1	Leopardus guigna NC028321 1	0,14504841
Zalophus wollebaeki SRR4431565 1	Mirounga angustirostris SRR10331586 1	0,14505184
Genetta genetta T297 1	Acinonyx jubatus NC005212 3	0,14505844
Leopardus colocolo NC028314 1	Arctictis binturong T605 2	0,14507246
Vulpes corsac NC023958 1	Lycaon pictus NC028427 2	0,14510004
Poecilogale albinucha T602 1	Lontra canadensis SRR10409165 1	0,14510882
Viverricula indica KX891751 1	Puma concolor NC016470 22	0,1451104
Viverra zibetha T609 1	Felis catus NC001700 2	0,14511108
Leopardus pardalis T262 1	Genetta genetta T297 1	0,14511238
Prionodon pardicolor NC024569 2	Leopardus tigrinus NC028317 1	0,14511961
Caracal caracal NC028306 1	Arctictis binturong T605 2	0,14512402
Genetta abyssinica MG489822 1	Catopuma temminckii NC027115 41	0,14513697
Prionailurus rubiginosus NC028304 2	Chrotogale owstoni T607 1	0,14516659
Viverricula indica KX891751 1	Felis catus NC001700 2	0,14517777
Viverricula indica NC025296 2	Lynx lynx NC027083 4	0,14518183
Genetta abyssinica MG489822 1	Galidictis fasciata DM333 1	0,1452069
Mirounga leonina NC008422 1	Arctocephalus australis MG023139 1	0,14522928
Vulpes vulpes NC008434 5	Speothos venaticus C48 2	0,14522965
Leptailurus serval NC028316 1	Chrotogale owstoni T607 1	0,14523394
Helogale parvula SRR7637809 1	Galidia elegans D146 1	0,1452354
Helogale parvula SRR7637809 1	Cryptoprocta ferox CFC13 1	0,14524172
Leopardus pardalis T262 1	Crossarchus platycephalus C7R66 1	0,14524616
Smilodon populator MF871700 1	Paguma larvata PDDS11 2	0,14524819
Paguma larvata PDDS11 2	Catopuma temminckii NC027115 41	0,1452515
Vulpes ferrilata NC027935 1	Cuon alpinus NC013445 3	0,14525643
Leopardus colocolo NC028314 1	Genetta genetta T297 1	0,14526361
Neophoca cinerea NC008419 1	Leptonychotes weddellii NC008424 1	0,14529774
Lynx pardinus NC028319 161	Hemigalus derbyanus MH464791 1	0,14529835
Prionailurus bengalensis NC028301 12	Paradoxurus hermaphroditus NC039591 1	0,14531149
Genetta servalina NC024568 2	Catopuma temminckii NC027115 41	0,14531739
Panthera tigris amoyensis NC014770 2	Nandinia binotata NC024567 1	0,14531846
Zalophus californianus NC008416 1	Pusa sibirica NC008432 2	0,14532101
Viverra zibetha T609 1	Panthera onca NC022842 1	0,14532128
Leopardus pardalis T262 1	Arctictis binturong T605 2	0,14532425
Profelis aurata NC028299 1	Arctictis binturong T605 2	0,14532608
Zalophus californianus NC008416 1	Phoca fasciata NC008428 1	0,145329
Zalophus californianus NC008416 1	Phoca groenlandica NC008429 54	0,14533128
Eumetopias jubatus NC004030 10	Cystophora cristata NC008427 1	0,1453387
Hemigalus derbyanus MH464791 1	Felis chaus NC028307 1	0,14535107
Panthera uncia KP202269 1	Civettictis civetta NC033378 1	0,14535172
Panthera onca KP202264 2	Civettictis civetta NC033378 1	0,14535187
Lynx canadensis NC028313 1	Hemigalus derbyanus MH464791 1	0,14535764
Suricata suricatta SSM10 1	Eupleres goudotii D128 1	0,14536817
Felis margarita NC028308 1	Chrotogale owstoni T607 1	0,14537059
Suricata suricatta SSM10 1	Galidictis fasciata DM333 1	0,14537123
Vulpes zerda KJ603240 1	Chrysocyon brachyurus NC024172 1	0,14537141
Paradoxurus hermaphroditus NLNC 1	Leopardus wiedii NC028318 1	0,145377
Neophoca cinerea NC008419 1	Lobodon carcinophaga NC008423 1	0,1453775
Viverricula indica KX891751 1	Felis margarita NC028308 1	0,14538009
Viverra zibetha T609 1	Homotherium latidens MF871702 3	0,14538069
Panthera onca NC022842 1	Civettictis civetta GLC19 1	0,14538911
Lynx canadensis NC028313 1	Civettictis civetta NC033378 1	0,14541876
Phoca fasciata NC008428 1	Arctocephalus gazella BK010918 1	0,14544053
Paguma larvata PDDS11 2	Lynx pardinus NC028319 161	0,14544697
Viverricula indica KX891745 1	Pardofelis marmorata NLN3 2	0,14544748
Leopardus wiedii NC028318 1	Crossarchus platycephalus C7R66 1	0,14544815
Genetta genetta T297 1	Felis nigripes NC028309 1	0,14545026
Prionodon pardicolor NC024569 2	Leopardus guigna NC028321 1	0,14546144
Paradoxurus hermaphroditus NLNC 1	Leopardus colocolo NC028314 1	0,14546224
Viverra zibetha T609 1	Panthera leo NERO 19	0,14547808
Panthera leo NERO 19	Civettictis civetta GLC19 1	0,14547834
Mirounga leonina NC008422 1	Arctocephalus townsendi NC008420 1	0,14549774
Meles leucurus NC039173 4	Lutrogale perspicillata NC035811 1	0,1454992
Galidia elegans D146 1	Bdeogale nigripes GLC15 1	0,14550515
Poecilogale albinucha T602 1	Lutrogale perspicillata NC035811 1	0,1455116
Lynx lynx NC027083 4	Genetta genetta T297 1	0,14551622
Melogale moschata V0735A 1	Galictis vittata T412 1	0,14551763
Zalophus wollebaeki SRR4431565 1	Phoca groenlandica NC008429 54	0,1455214
Diplogale hosei MH464790 1	Acinonyx jubatus NC005212 3	0,14552206
Lynx pardinus NC028319 161	Arctictis binturong T605 2	0,14552785
Smilodon populator MF871700 1	Fossa fossana D350 1	0,14554466
Zalophus californianus NC008416 1	Monachus monachus NC044972 5	0,1455531
Erignathus barbatus NC008426 1	Arctocephalus gazella BK010918 1	0,14556825
Lontra canadensis SRR10409165 1	Arctonyx collaris NC020645 1	0,14556834
Zalophus californianus NC008416 1	Halichoerus grypus NC001602 2	0,1455687
Prionailurus planiceps KY682741 4	Fossa fossana D350 1	0,14558005

Vulpes ferrilata NC027935 1	Phoca groenlandica NC008429 54	0,18476564
Martes zibellina NC011579 39	Ichneumia albicauda T603 1	0,18476641
Martes foina NC020643 1	Urva javanica T413 1	0,18476836
Prionailurus rubiginosus NC028304 2	Arctocephalus forsteri KT693377 17	0,1847688
Cynogale bennetti KY117544 1	Bassariscus sumichrasti SRX1099089 1	0,18476933
Vulpes ferrilata NC027935 1	Leopardus wiedii NC028318 1	0,18476962
Speothos venaticus C48 2	Mustela frenata NC020640 1	0,18476996
Phocarctos hookeri NC008418 1	Lynx lynx NC027083 4	0,18477069
Leopardus pardalis NC028315 1	Canis anthus NC027956 2	0,18477076
Prionodon pardicolor NC024569 2	Martes martes T302 3	0,18477137
Prionailurus planiceps KY682741 4	Lontra canadensis SRR10409165 1	0,18477157
Prionailurus planiceps NC028312 6	Canis anthus NC027956 2	0,18477227
Ursus thibetanus mupinensis NC00875	Chrotogale owstoni T607 1	0,18477266
Leopardus geoffroyi NC028320 1	Arctocephalus gazella BK010918 1	0,18477276
Prionailurus planiceps NC028312 6	Canis latrans NC008093 7	0,18477277
Profelis aurata NC028299 1	Canis anthus NC027956 2	0,18477298
Panthera tigris amoyensis NC014770 2	Meles meles T303 3	0,18477331
Felis chaus NC028307 1	Canis latrans NC008093 7	0,1847735
Phocarctos hookeri NC008418 1	Leopardus geoffroyi NC028320 1	0,18477395
Martes pennanti NC020664 16	Ichneumia albicauda T603 1	0,18477444
Ursus arctos GU573491 207	Mustela itatsi NC034330 19	0,18477461
Zalophus wollebaeki SRR4431565 1	Urva semitorquata MH464789 1	0,18477537
Ursus arctos GU573491 207	Aonyx cinerea NC035814 2	0,18477552
Procyon lotor AB462049 4	Caracal caracal NC028306 1	0,18477565
Ursus arctos isabellinus 18B5 2	Bassariscus sumichrasti SRX1099089 1	0,18477571
Procyon lotor AB462046 3	Leopardus tigrinus NC028317 1	0,18477593
Paradoxurus hermaphroditus NC03959	Lycalopex sechurae KT448284 1	0,18477611
Ursus thibetanus formosanus NC00933	Lutra lutra NC011358 9	0,18477646
Procyon lotor AB462046 3	Leopardus jacobita NC028322 1	0,18477683
Martes pennanti NC020664 16	Helarctos malayanus NC009968 2	0,18477774
Ursus maritimus NC003428 31	Otaria byronia OTAB 1	0,18477805
Otocyon megalotis SAF1 2	Lynx canadensis NC028313 1	0,18477872
Taxidea taxus NC020646 1	Cryptoprocta ferox CFC13 1	0,18478246
Speothos venaticus C48 2	Monachus schauinslandi NC008421 1	0,18478259
Meles leucurus NC039173 4	Ailuropoda melanoleuca NC009492 5	0,18478346
Mustela eversmannii NC028013 1	Melursus ursinus NC009970 2	0,18478511
Ursus thibetanus formosanus NC00933	Aonyx cinerea NC035814 2	0,18478581
Martes pennanti NC020664 16	Arctictis binturong T605 2	0,18478604
Prionodon pardicolor NC024569 2	Lycaon pictus NC028427 2	0,18478608
Viverricula indica NC025296 2	Ictonyx striatus T299 1	0,1847866
Zalophus californianus NC008416 1	Bdeogale nigripes GLC15 1	0,18478814
Zalophus californianus NC008416 1	Urva semitorquata MH464789 1	0,18478855
Ursus spelaeus EU327344 13	Leopardus pardalis T262 1	0,18478879
Profelis aurata NC028299 1	Lycaon pictus NC028427 2	0,184789
Ursus spelaeus EU327344 13	Leopardus pardalis NC028315 1	0,18478902
Mephitis mephitis NC020648 1	Martes pennanti NC020664 16	0,18478964
Ursus thibetanus formosanus NC00933	Felis silvestris lybica KP202275 4	0,18479216
Lynx lynx NC027083 4	Galictis vittata T412 1	0,18479261
Martes americana NC020642 1	Canis lupus chanco NC010340 4	0,18480036
Lutra sumatrana NC035810 1	Civettictis civetta NC033378 1	0,18480089
Martes foina NC020643 1	Canis lupus familiaris NC002008 1231	0,18480395
Martes flavigula NC012141 3	Arctictis binturong T605 2	0,18480635
Zalophus californianus NC008416 1	Galictis vittata T412 1	0,18480822
Lycaon pictus NC028427 2	Gulo gulo NC009685 3	0,18480875
Tremarctos ornatus NC009969 2	Civettictis civetta NC033378 1	0,18480924
Prionodon pardicolor NC024569 2	Gulo gulo NC009685 3	0,18480933
Ursus thibetanus formosanus NC00933	Canis aureus KT448274 1	0,18481119
Genetta genetta T297 1	Canis lupus chanco NC010340 4	0,18481244
Vulpes ferrilata NC027935 1	Halichoerus grypus NC001602 2	0,18481302
Leptailurus serval NC028316 1	Cuon alpinus NC013445 3	0,18481453
Prionodon pardicolor NC024569 2	Lycalopex sechurae KT448284 1	0,18481472
Prionailurus planiceps NC028312 6	Eumetopias jubatus NC004030 10	0,18481481
Mungos mungo MMC7 1	Lutra lutra NC011358 9	0,1848149
Ursus americanus JX196366 3	Mustela nivalis T306 5	0,18481548
Lutra lutra NC011358 9	Atilax paludinosus T606 1	0,18481693
Phocarctos hookeri NC008418 1	Enhydra lutris NC009692 1	0,18481885
Mustela eversmannii NC028013 1	Chrotogale owstoni T607 1	0,18481933
Mungos mungo/gambianus SRR77048	Tapirus terrestris T358	0,18481997
Martes zibellina NC011579 39	Atilax paludinosus T606 1	0,18482149
Mustela itatsi NC034330 19	Mungos mungo MMC7 1	0,18482201
Mustela sibirica AP017394 11	Urva brachyura KY117547 1	0,18482251
Mustela nigripes NC024942 1	Xenogale naso C07XAR110 1	0,18482333
Mungotictis decemlineata NC027828 1	Melogale moschata V0735A 1	0,18482438
Smilodon populator MF871700 1	Arctocephalus forsteri NC004023 28	0,1848283
Mungos mungo/gambianus SRR77048	Bassariscus sumichrasti SRX1099089 1	0,18482891
Martes martes T302 3	Chrotogale owstoni T607 1	0,18482935
Pardofelis marmorata NLN3 2	Lutra lutra NC011358 9	0,18483111
Viverricula indica KX891745 1	Martes zibellina NC011579 39	0,1848318
Otocyon megalotis SAF1 2	Crossarchus platycephalus C7R66 1	0,18483211
Panthera uncia NC010638 1	Mirounga leonina NC008422 1	0,18483213
Nasua nasua NC020647 1	Ictonyx striatus T299 1	0,18483216
Pusa sibirica NC008432 2	Paradoxurus hermaphroditus NC03959	0,18483232
Ursus thibetanus thibetanus NC011118	Canis lupus chanco NC010340 4	0,18483256
Viverra zibetha T609 1	Tremarctos ornatus NC009969 2	0,18483384
Mustela kathiah NC023210 1	Galerella sanguinea T378 1	0,1848343
Suricata suricatta SSM10 1	Arctodus simus NC011116 1	0,18483479
Martes martes T302 3	Canis anthus NC027956 2	0,18483497
Parahyaena brunnea NC038159 15	Enhydra lutris NC009692 1	0,18483518
Mustela sibirica NC020637 6	Ichneumia albicauda T603 1	0,18483575
Mustela sibirica AP017394 11	Felis chaus NC028307 1	0,18483645
Lontra canadensis SRR10409165 1	Arctotherium sp NC030174 1	0,18483646
Vulpes lagopus NC026529 3	Felis catus NC001700 2	0,18483655
Viverra tangalunga MH464792 1	Ichneumia albicauda T603 1	0,18483664
Viverricula indica KX891751 1	Melogale moschata NC020644 1	0,18483686
Canis aureus KT448274 1	Arctonyx collaris NC020645 1	0,18483689
Puma yagouaroundi NC028311 1	Melogale moschata V0735A 1	0,18483737
Melogale moschata V0735A 1	Felis chaus NC028307 1	0,18483779
Zalophus californianus NC008416 1	Civettictis civetta NC033378 1	0,18483901
Tremarctos ornatus NC009969 2	Catopuma badia NC028300 1	0,18483903
Vulpes zerda KJ603240 1	Phoca largha NC008430 1	0,18483914
Mustela altaica NC021751 1	Galidictis fasciata DM333 1	0,18483944
Prionailurus viverrinus NC028305 1	Canis latrans NC008093 7	0,18483952
Prionailurus planiceps KY682741 4	Neophoca cinerea NC008419 1	0,1848399

Pusa hispida NC 008433 1	Arctocephalus gazella BK010918 1	0,14558049
Zalophus wollebaeki SRR4431565 1	Phoca fasciata NC008428 1	0,14558671
Viverricula indica NC025296 2	Panthera onca NC022842 1	0,14559415
Genetta abyssinica MG489822 1	Felis catus NC001700 2	0,14562331
Panthera onca NC022842 1	Civettictis civetta NC033378 1	0,14562543
Neophoca cinerea NC008419 1	Erignathus barbatus NC008426 1	0,14563526
Monachus schauinslandi NC008421 1	Arctocephalus gazella BK010918 1	0,14564575
Prionailurus bengalensis NC028301 12	Paradoxurus jerdoni MH464793 1	0,14564845
Chrotogale owstoni T607 1	Acinonyx jubatus NC005212 3	0,14564922
Prionailurus viverrinus NC028305 1	Fossa fossana D350 1	0,1456506
Profelis aurata NC028299 1	Genetta servalina NC024568 2	0,14565116
Genetta genetta T297 1	Felis margarita NC028308 1	0,14565191
Mungotictis decemlineata NC027828 1	Lynx rufus NC014456 3	0,14565192
Salanoia concolor D378 1	Prionailurus rubiginosus NC028304 2	0,14565362
Salanoia concolor D378 1	Leopardus pardalis T262 1	0,14565593
Zalophus wollebaeki SRR4431565 1	Monachus schauinslandi NC008421 1	0,14565732
Puma yagouaroundi NC028311 1	Arctictis binturong T605 2	0,14566284
Paguma larvata PDDS11 2	Homotherium latidens MF871702 3	0,14566858
Urocyon cinereoargenteus NC026723 21	Nyctereutes procyonoides NC013700 3	0,14569448
Urocyon littoralis catalinae KP129018 15	Nyctereutes procyonoides NC013700 3	0,14569448
Prionailurus bengalensis NC028301 12	Chrotogale owstoni T607 1	0,14570451
Urocyon cinereoargenteus NC026723 21	Otocyon megalotis SAF1 2	0,14570458
Erignathus barbatus NC008426 1	Arctocephalus pusillus NC008417 1	0,1457071
Prionodon linsang ERR2391707 1	Paguma larvata PDDS11 2	0,14571044
Fossa fossana D350 1	Catopuma badia NC028300 1	0,14571244
Neophoca cinerea NC008419 1	Hydrurga leptonyx NC008425 1	0,14571366
Lynx lynx NC027083 4	Fossa fossana D350 1	0,1457141
Prionailurus viverrinus NC028305 1	Paguma larvata PDDS11 2	0,14571629
Zalophus wollebaeki SRR4431565 1	Pusa caspica NC008431 1	0,14571884
Leopardus pardalis T262 1	Eupleres goudotii D128 1	0,14572006
Puma concolor NC016470 22	Prionodon pardicolor NC024569 2	0,1457267
Nandinia binotata NC024567 1	Homotherium latidens MF871702 3	0,14575015
Phoca largha NC008430 1	Eumetopias jubatus NC004030 10	0,14575571
Erignathus barbatus NC008426 1	Arctocephalus townsendi NC008420 1	0,14576897
Viverricula indica KX891745 1	Lynx canadensis NC028313 1	0,14578351
Viverricula indica KX891745 1	Lynx rufus NC014456 3	0,14578371
Leopardus colocolo NC028314 1	Chrotogale owstoni T607 1	0,14578809
Prionodon pardicolor NC024569 2	Prionailurus rubiginosus NC028304 2	0,14579414
Zalophus californianus NC008416 1	Cystophora cristata NC008427 1	0,14579636
Urva javanica/auropunctata NC006835 1	Genetta servalina NC024568 2	0,14580975
Urocyon littoralis catalinae KP129018 15	Otocyon megalotis SAF1 2	0,14583924
Vulpes vulpes NC008434 5	Lycaon pictus NC028427 2	0,14584059
Otocyon megalotis SAF1 2	Lycaon pictus NC028427 2	0,14584135
Galidia elegans D146 1	Galerella sanguinea T378 1	0,14584158
Mungos mungo MMC7 1	Galidia elegans D146 1	0,14584433
Viverricula indica KX891751 1	Prionodon linsang ERR2391707 1	0,14584546
Phocarctos hookeri NC008418 1	Hydrurga leptonyx NC008425 1	0,14584802
Pusa hispida NC 008433 1	Neophoca cinerea NC008419 1	0,14584851
Leopardus pardalis NC028315 1	Crossarchus platycephalus C7R66 1	0,14585188
Salanoia concolor D378 1	Prionailurus bengalensis NC028301 12	0,14585604
Leopardus pardalis NC028315 1	Arctictis binturong T605 2	0,14586291
Prionodon pardicolor NC024569 2	Acinonyx jubatus NC005212 3	0,14587334
Prionailurus bengalensis NC028301 12	Diplogale hosei MH464790 1	0,14589873
Nandinia binotata NC024567 1	Xenogale naso C07XAR110 1	0,14590627
Nasua nasua NC020647 1	Bassaricyon neblina SRX1097850 1	0,14591631
Mungotictis decemlineata NC027828 1	Catopuma badia NC028300 1	0,14591983
Viverricula indica NC025296 2	Pardofelis marmorata NLN3 2	0,14592259
Salanoia concolor D378 1	Leopardus wiedii NC028318 1	0,14592519
Prionailurus planiceps NC028312 6	Arctictis binturong T605 2	0,14593196
Zalophus californianus NC008416 1	Monachus schauinslandi NC008421 1	0,14593881
Mirounga leonina NC008422 1	Arctocephalus forsteri NC004023 28	0,14595531
Genetta abyssinica MG489822 1	Crossarchus platycephalus C7R66 1	0,14595619
Phoca vitulina NC001325 1	Eumetopias jubatus NC004030 10	0,14595763
Xenogale naso C07XAR110 1	Cryptoprocta ferox CFC13 1	0,14598207
Cryptoprocta ferox CFC13 1	Bdeogale nigripes GLC15 1	0,14598235
Prionailurus rubiginosus NC028304 2	Crossarchus platycephalus C7R66 1	0,14598449
Panthera pardus japonensis KJ866876 8	Nandinia binotata NC024567 1	0,14598487
Salanoia concolor D378 1	Catopuma badia NC028300 1	0,14598714
Genetta genetta T297 1	Caracal caracal NC028306 1	0,14598903
Mungotictis decemlineata NC027828 1	Leopardus jacobita NC028322 1	0,14599133
Lynx pardinus NC028319 161	Cryptoprocta ferox CFC13 1	0,14599334
Felis catus NC001700 2	Arctictis binturong T605 2	0,14599897
Prionailurus planiceps KY682741 4	Arctictis binturong T605 2	0,14599925
Zalophus wollebaeki SRR4431565 1	Monachus monachus NC044972 5	0,14601305
Mungotictis decemlineata NC027828 1	Genetta abyssinica MG489822 1	0,1460361
Meles meles T303 3	Lontra canadensis SRR10409165 1	0,14603944
Puma yagouaroundi NC028311 1	Chrotogale owstoni T607 1	0,14604063
Spilogale putorius NC010497 1	Conepatus chinga NC042596 1	0,14604205
Viverra zibetha T609 1	Nandinia binotata NC024567 1	0,14604792
Lobodon carcinophaga NC008423 1	Arctocephalus townsendi NC008420 1	0,14604945
Genetta genetta T297 1	Felis silvestris lybica KP202275 4	0,14605559
Viverricula indica NC025296 2	Lynx rufus NC014456 3	0,14605726
Prionodon pardicolor NC024569 2	Prionailurus planiceps KY682741 4	0,14606321
Hemigalus derbyanus MH464791 1	Catopuma temminckii NC027115 41	0,146082
Panthera pardus NC010641 5	Nandinia binotata NC024567 1	0,14608727
Suricata suricatta SSM10 1	Salanoia concolor D378 1	0,14611141
Phoca fasciata NC008428 1	Arctocephalus pusillus NC008417 1	0,14611794
Pardofelis marmorata NLN3 2	Genetta abyssinica MG489822 1	0,14611822
Prionailurus planiceps NC028312 6	Fossa fossana D350 1	0,1461187
Fossa fossana D350 1	Caracal caracal NC028306 1	0,14611898
Panthera uncia KP202269 1	Civettictis civetta GLC19 1	0,14612234
Meles meles T303 3	Galictis vittata T412 1	0,14612359
Salanoia concolor D378 1	Leopardus pardalis NC028315 1	0,14612705
Nandinia binotata NC024567 1	Arctictis binturong T605 2	0,14612938
Panthera leo spelaea KX258452 2	Civettictis civetta NC033378 1	0,1461571
Nandinia binotata NC024567 1	Cynictis penicillata T375 1	0,14617559
Suricata suricatta SSM10 1	Mungotictis decemlineata NC027828 1	0,1461786
Mirounga angustirostris SRR10331586 1	Callorhinus ursinus NC008415 1	0,14618157
Prionodon linsang ERR2391707 1	Paradoxurus hermaphroditus NC039591 1	0,14618184
Zalophus wollebaeki SRR4431565 1	Pusa sibirica NC008432 2	0,14618574
Leptailurus serval NC028316 1	Fossa fossana D350 1	0,14618638
Salanoia concolor D378 1	Lynx lynx NC027083 4	0,1461902
Salanoia concolor D378 1	Lynx canadensis NC028313 1	0,14619069

Prionailurus planiceps NC028312 6	Nyctereutes procyonoides NC013700 3	0,18484008
Viverricula indica NC025296 2	Martes pennanti NC020664 16	0,18484031
Canis latrans NC008093 7	Arctodus simus NC011116 1	0,18484117
Panthera onca KP202264 2	Aonyx cinerea NC035814 2	0,18484168
Viverra tangalunga MH464792 1	Ursus maritimus GU573488 Svalbard	0,18484189
Ursus arctos pruinosus MG066703 3	Martes martes T302 3	0,18484227
Leopardus guigna NC028321 1	Canis latrans NC008093 7	0,184843
Otaria byronia OTAB 1	Lynx rufus NC014456 3	0,18484308
Phoca groenlandica NC008429 54	Panthera uncia NC010638 1	0,1848436
Mephitis mephitis NC020648 1	Melogale moschata KP726273 1	0,18484381
Otaria byronia OTAB 1	Ictonyx striatus T299 1	0,1848443
Viverricula indica KX891751 1	Martes foina NC020643 1	0,1848447
Profelis aurata NC028299 1	Arctotherium sp NC030174 1	0,18484497
Mustela altaica NC021751 1	Ichneumia albicauda T603 1	0,18484581
Viverra tangalunga MH464792 1	Panthera onca NC022842 1	0,18484662
Ursus arctos GU573491 207	Mustela nivalis T306 5	0,18484668
Otaria byronia OTAB 1	Leopardus geoffroyi NC028320 1	0,18484733
Procyon lotor AB462049 4	Leopardus colocolo NC028314 1	0,18484762
Viverra tangalunga MH464792 1	Ursus arctos GU573486 5	0,18484822
Otocolobus manul NC028323 1	Otaria byronia OTAB 1	0,18484873
Ursus arctos AP012576 6	Martes pennanti NC020664 16	0,18485048
Ursus arctos AP012576 6	Martes zibellina NC011579 39	0,18485071
Meles meles T303 3	Ailuropoda melanoleuca NC009492 5	0,18485099
Ursus arctos GU573486 5	Otaria byronia OTAB 1	0,18485151
Ursus thibetanus thibetanus NC01111	Mungotictis decemlineata NC027828 1	0,18485159
Paguma larvata PDDS11 2	Lycaon pictus NC028427 2	0,18485251
Paguma larvata PDDS11 2	Canis mesomelas KT448280 1	0,18485554
Ursus spelaeus EU327344 13	Prionailurus bengalensis NC028301 12	0,18485765
Ursus thibetanus formosanus NC00993	Prionailurus planiceps KY682741 4	0,18485951
Ursus thibetanus thibetanus NC01111	Felis catus NC001700 2	0,18486083
Leopardus pardalis NC028315 1	Helarctos malayanus NC009968 2	0,18486106
Ursus thibetanus thibetanus NC01111	Prionailurus planiceps KY682741 4	0,18486223
Melursus ursinus NC009970 2	Leopardus pardalis NC028315 1	0,18486241
Mustela altaica NC021751 1	Arctocephalus forsteri NC004023 28	0,18486436
Martes flavigula NC012141 3	Cynictis penicillata T375 1	0,18486547
Genetta abyssinica MG489822 1	Arctotherium sp NC030174 1	0,18486556
Zalophus californianus NC008416 1	Paradoxurus jerdoni MH464793 1	0,18486875
Ursus americanus JX196366 3	Mustela sibirica NC020637 6	0,18487534
Prionodon pardicolor NC024569 2	Martes flavigula NC012141 3	0,18487597
Lynx rufus NC014456 3	Canis lupus chanco NC010340 4	0,18487817
Leopardus geoffroyi NC028320 1	Canis mesomelas KT448280 1	0,18487985
Procyon lotor AB462049 4	Civettictis civetta NC033378 1	0,18488082
Phocarctos hookeri NC008418 1	Meles anakuma NC009677 1	0,18488774
Mustela kathiah NC023210 1	Eupleres goudotii D128 1	0,18488812
Mustela eversmannii NC028013 1	Mungos mungo MMC7 1	0,18488836
Xenogale naso C07XAR110 1	Arctonyx collaris NC020645 1	0,18488947
Potos flavus T414 1	Canis aureus KT448274 1	0,18489002
Phocarctos hookeri NC008418 1	Mustela sibirica NC020637 6	0,18489014
Martes americana NC020642 1	Canis latrans NC008093 7	0,18489149
Ursus spelaeus NC011112 8	Spilogale putorius NC010497 1	0,18489237
Tremarctos ornatus NC009969 2	Mustela sibirica NC020637 6	0,18489408
Helogale parvula SRR7637809 1	Bassariscus sumichrasti SRX1099089 1	0,18489517
Otaria byronia OTAB 1	Mungos mungo/gambianus SRR77048	0,18489638
Viverra tangalunga MH464792 1	Canis anthus NC027956 2	0,18489729
Viverricula indica KX891745 1	Martes americana NC020642 1	0,18489829
Viverricula indica NC025296 2	Callorhinus ursinus NC008415 1	0,18489856
Prionailurus bengalensis CKM45 20	Conepatus chinga NC042596 1	0,18489955
Diplogale hosei MH464790 1	Arctodus simus NC011116 1	0,1848996
Panthera onca KP202264 2	Lutra lutra LC050126 1	0,18489987
Felis margarita NC028308 1	Callorhinus ursinus NC008415 1	0,18490005
Lutra sumatrana NC035810 1	Genetta genetta T297 1	0,18490031
Puma concolor NC016470 22	Callorhinus ursinus NC008415 1	0,18490046
Vulpes lagopus NC026529 3	Martes martes T302 3	0,18490149
Ursus maritimus NC003428 31	Arctocephalus australis MG023139 1	0,18490186
Melogale moschata V0735A 1	Genetta genetta T297 1	0,18490197
Ursus maritimus NC003428 31	Canis adustus KT448271 1	0,18490236
Viverricula indica NC025296 2	Martes americana NC020642 1	0,18490256
Mustela putorius NC020638 4	Felis nigripes NC028309 1	0,18490277
Ictonyx striatus T299 1	Arctocephalus australis MG023139 1	0,18490279
Mungos mungo/gambianus SRR77048	Ictonyx striatus T299 1	0,18490279
Martes foina NC020643 1	Bdeogale nigripes GLC15 1	0,18490306
Viverricula indica NC025296 2	Martes zibellina NC011579 39	0,18490316
Martes foina NC020643 1	Cynictis penicillata T375 1	0,18490322
Phocarctos hookeri NC008418 1	Canis adustus KT448271 1	0,18490399
Ursus arctos GU573486 5	Canis adustus KT448271 1	0,18490507
Leopardus tigrinus NC028317 1	Arctocephalus forsteri KT693377 17	0,1849056
Leopardus geoffroyi NC028320 1	Ailurus fulgens NC011124 1	0,18490584
Vulpes lagopus NC026529 3	Catopuma temminckii NC027115 41	0,18490671
Nyctereutes procyonoides NC013700 3	Felis nigripes NC028309 1	0,18490687
Nyctereutes procyonoides NC013700 3	Felis silvestris lybica KP202275 4	0,18490708
Vulpes ferrilata NC027935 1	Prionailurus bengalensis CKM45 20	0,18490836
Paradoxurus jerdoni MH464793 1	Arctocephalus pusillus NC008417 1	0,18490925
Prionodon pardicolor NC024569 2	Melogale moschata NC020644 1	0,18490931
Ursus maritimus GU573488 Svalbard	Bassariscus sumichrasti SRX1099089 1	0,18490994
Poecilogale albinucha T602 1	Lynx rufus NC014456 3	0,18490998
Leopardus geoffroyi NC028320 1	Canis anthus NC027956 2	0,1849101
Poecilogale albinucha T602 1	Felis chaus NC028307 1	0,18491028
Poecilogale albinucha T602 1	Felis margarita NC028308 1	0,18491054
Martes pennanti NC020664 16	Leopardus tigrinus NC028317 1	0,18491146
Ursus thibetanus laniger MH281753 2	Lutra lutra NC011358 9	0,18491149
Ursus arctos isabellinus 18B5 2	Mustela altaica NC021751 1	0,18491155
Puma yagouaroundi NC028311 1	Poecilogale albinucha T602 1	0,18491165
Viverricula indica NC025296 2	Arctocephalus pusillus NC008417 1	0,18491188
Mustela altaica NC021751 1	Lycaon pictus NC028427 2	0,18491374
Mustela nivalis T306 5	Felis chaus NC028307 1	0,18491381
Ursus spelaeus NC011112 8	Lutra lutra LC050126 1	0,18491443
Prionailurus bengalensis CKM45 20	Lutrogale perspicillata NC035811 1	0,18491448
Leopardus colocolo NC028314 1	Conepatus chinga NC042596 1	0,18491578
Ursus thibetanus formosanus NC00993	Mungotictis decemlineata NC027828 1	0,1849165
Smilodon populator MF871700 1	Martes americana NC020642 1	0,1849169
Ursus arctos GU573486 5	Mustela altaica NC021751 1	0,18491846
Otocolobus manul NC028323 1	Ictonyx striatus T299 1	0,18491974
Lutra lutra LC050126 1	Canis lupus familiaris NC002008 1231	0,18492028

Prionailurus rubiginosus NC028304 2	Mungotictis decemlineata NC027828 1	0,14619209
Leopardus wiedii NC028318 1	Arctictis binturong T605 2	0,14619979
Galidictis fasciata DM333 1	Civettictis civetta NC033378 1	0,14622369
Vulpes corsac NC023958 1	Speothos venaticus C48 2	0,14623991
Speothos venaticus C48 2	Nyctereutes procyonoides NC013700 3	0,14624061
Pusa sibirica NC008432 2	Neophoca cinerea NC008419 1	0,14624238
Lynx canadensis NC028313 1	Fossa fossana D350 1	0,14625289
Galidictis fasciata DM333 1	Civettictis civetta GLC19 1	0,14625446
Viverricula indica KX891751 1	Lynx pardinus NC028319 161	0,1462547
Paguma larvata PDDS11 2	Lynx rufus NC014456 3	0,14625499
Pusa hispida NC 008433 1	Otaria byronia OTAB 1	0,1462553
Leopardus pardalis T262 1	Galidictis fasciata DM333 1	0,14626247
Zalophus californianus NC008416 1	Phoca largha NC008430 1	0,14627492
Homotherium latidens MF871702 3	Arctictis binturong T605 2	0,14628518
Hemigalus derbyanus MH464791 1	Cynictis penicillata T375 1	0,14629336
Mungotictis decemlineata NC027828 1	Urva brachyura KY117547 1	0,14631434
Viverricula indica NC025296 2	Panthera onca KP202264 2	0,14632687
Zalophus californianus NC008416 1	Phoca vitulina NC001325 1	0,14634247
Otaria byronia OTAB 1	Halichoerus grypus NC001602 2	0,14635183
Panthera uncia NC010638 1	Civettictis civetta NC033378 1	0,14636201
Zalophus wollebaeki SRR4431565 1	Halichoerus grypus NC001602 2	0,14636606
Meles anakuma NC009677 1	Lutrogale perspicillata NC035811 1	0,14637418
Nandinia binotata NC024567 1	Atilax paludinosus T606 1	0,14637759
Nandinia binotata NC024567 1	Eupleres goudotii D128 1	0,14637816
Prionodon linsang ERR2391707 1	Paradoxurus hermaphroditus NLNC 1	0,1463837
Cynictis penicillata T375 1	Cryptoprocta ferox CFC13 1	0,14638621
Mungos mungo MMC7 1	Cryptoprocta ferox CFC13 1	0,14638977
Puma yagouaroundi NC028311 1	Paguma larvata PDDS11 2	0,14639044
Zalophus wollebaeki SRR4431565 1	Cystophora cristata NC008427 1	0,14639112
Puma yagouaroundi NC028311 1	Prionodon pardicolor NC024569 2	0,1464045
Ommatophoca rossii AY377287etc 1	Eumetopias jubatus NC004030 10	0,14642513
Vulpes zerda KJ603240 1	Speothos venaticus C48 2	0,14644141
Smilodon populator MF871700 1	Chrotogale owstoni T607 1	0,14644431
Vulpes ferrilata NC027935 1	Lycaon pictus NC028427 2	0,14644693
Felis catus NC001700 2	Chrotogale owstoni T607 1	0,14644824
Viverricula indica KX891745 1	Mungos mungo MMC7 1	0,14645158
Prionailurus planiceps NC028312 6	Paradoxurus hermaphroditus NLNC 1	0,14645632
Lontra canadensis SRR10409165 1	Galictis vittata T412 1	0,14645985
Mungotictis decemlineata NC027828 1	Leopardus wiedii NC028318 1	0,14646322
Mungotictis decemlineata NC027828 1	Leopardus pardalis T262 1	0,14646323
Paradoxurus hermaphroditus NLNC 1	Acinonyx jubatus NC005212 3	0,14646775
Leopardus pardalis T262 1	Cryptoprocta ferox CFC13 1	0,14646864
Fossa fossana D350 1	Acinonyx jubatus NC005212 3	0,14647007
Urva javanica/auropunctata NC006835 1	Hemigalus derbyanus MH464791 1	0,14649999
Erignathus barbatus NC008426 1	Arctocephalus forsteri NC004023 28	0,14650041
Mungos mungo MMC7 1	Genetta servalina NC024568 2	0,14651959
Viverra zibetha T609 1	Panthera onca KP202264 2	0,14652567
Mungotictis decemlineata NC027828 1	Lynx lynx NC027083 4	0,14652641
Leopardus jacobita NC028322 1	Genetta genetta T297 1	0,14652686
Mungotictis decemlineata NC027828 1	Leopardus pardalis NC028315 1	0,14653054
Paradoxurus jerdoni MH464793 1	Catopuma badia NC028300 1	0,146532
Urocyon cinereoargenteus NC026723 21	Canis mesomelas KT448280 1	0,14654979
Hemigalus derbyanus MH464791 1	Fossa fossana D350 1	0,14657161
Erignathus barbatus NC008426 1	Arctocephalus forsteri KT693377 17	0,14657786
Galidia elegans D146 1	Cynictis penicillata T375 1	0,14658298
Chrotogale owstoni T607 1	Catopuma badia NC028300 1	0,14658435
Paradoxurus hermaphroditus NLNC 1	Leopardus pardalis T262 1	0,14658876
Viverricula indica NC025296 2	Mungos mungo MMC7 1	0,14659093
Viverricula indica KX891751 1	Lynx lynx NC027083 4	0,14659104
Leopardus geoffroyi NC028320 1	Fossa fossana D350 1	0,14659131
Mungos mungo MMC7 1	Leopardus wiedii NC028318 1	0,14659147
Prionailurus bengalensis CKM45 20	Crossarchus platycephalus C7R66 1	0,14659411
Lynx canadensis NC028313 1	Galidictis fasciata DM333 1	0,1465949
Panthera tigris NC010642 35	Nandinia binotata NC024567 1	0,14659782
Prionodon pardicolor NC024569 2	Catopuma badia NC028300 1	0,14660624
Paradoxurus hermaphroditus NLNC 1	Homotherium latidens MF871702 3	0,1466265
Nandinia binotata NC024567 1	Chrotogale owstoni T607 1	0,14663649
Leptailurus serval NC028316 1	Diplogale hosei MH464790 1	0,14664018
Meles leucurus NC039173 4	Lontra canadensis SRR10409165 1	0,14664513
Puma concolor NC016470 22	Chrotogale owstoni T607 1	0,14664809
Vulpes lagopus NC026529 3	Lycaon pictus NC028427 2	0,14664856
Viverricula indica KX891745 1	Prionodon linsang ERR2391707 1	0,14665341
Viverricula indica KX891745 1	Crossarchus platycephalus C7R66 1	0,14665417
Otocolobus manul NC028323 1	Hemigalus derbyanus MH464791 1	0,14665812
Genetta servalina NC024568 2	Galerella sanguinea T378 1	0,14666523
Urva javanica/auropunctata NC006835 1	Galidia elegans D146 1	0,14668588
Crossarchus platycephalus C7R66 1	Civettictis civetta NC033378 1	0,14668643
Viverricula indica NC025296 2	Urva javanica/auropunctata NC006835 1	0,14669038
Vulpes corsac NC023958 1	Urocyon cinereoargenteus NC026723 21	0,14670438
Prionailurus viverrinus NC028305 1	Chrotogale owstoni T607 1	0,14671588
Viverra zibetha T609 1	Panthera leo spelaea KX258452 2	0,14672557
Paguma larvata PDDS11 2	Leopardus jacobita NC028322 1	0,14672559
Paguma larvata PDDS11 2	Lynx lynx NC027083 4	0,14672588
Viverricula indica KX891751 1	Lynx rufus NC014456 3	0,14672604
Puma concolor NC016470 22	Paguma larvata PDDS11 2	0,14672682
Leopardus jacobita NC028322 1	Fossa fossana D350 1	0,14672723
Phoca largha NC008430 1	Arctocephalus pusillus NC008417 1	0,14672753
Salanoia concolor D378 1	Lynx pardinus NC028319 161	0,14672993
Paguma larvata PDDS11 2	Leopardus geoffroyi NC028320 1	0,14673258
Monachus monachus NC044972 5	Arctocephalus pusillus NC008417 1	0,1467586
Mephitis mephitis NC020648 1	Conepatus chinga NC042596 1	0,1467644
Otaria byronia OTAB 1	Erignathus barbatus NC008426 1	0,14678413
Viverricula indica KX891745 1	Bdeogale nigripes GLC15 1	0,14678846
Salanoia concolor D378 1	Genetta servalina NC024568 2	0,14679296
Prionailurus planiceps KY682741 4	Paradoxurus hermaphroditus NLNC 1	0,1467931
Pusa hispida NC 008433 1	Arctocephalus pusillus NC008417 1	0,14679444
Galictis vittata T412 1	Arctonyx collaris NC020645 1	0,14679673
Mungotictis decemlineata NC027828 1	Lynx pardinus NC028319 161	0,14679691
Salanoia concolor D378 1	Lynx rufus NC014456 3	0,146797
Lynx pardinus NC028319 161	Galidictis fasciata DM333 1	0,1467973
Prionailurus bengalensis NC028301 12	Mungotictis decemlineata NC027828 1	0,14679848
Nyctereutes procyonoides NC013700 3	Cuon alpinus NC013445 3	0,14680761
Monachus monachus NC044972 5	Arctocephalus gazella BK010918 1	0,14681751

Genetta abyssinica MG489822 1	Arctocephalus forsteri KT693377 17	0,18492243
Nyctereutes procyonoides NC013700 3	Acinonyx jubatus NC005212 3	0,18492399
Ursus spelaeus EU327344 13	Prionailurus rubiginosus NC028304 2	0,18492477
Ursus thibetanus laniger MH281753 2	Lynx lynx NC027083 4	0,18492568
Ursus spelaeus EU327344 13	Leopardus geoffroyi NC028320 1	0,18492787
Genetta servalina NC024568 2	Canis mesomelas KT448280 1	0,18492845
Leopardus wiedii NC028318 1	Galictis vittata T412 1	0,18492858
Lycalopex sechurae KT448284 1	Leopardus pardalis NC028315 1	0,18493047
Smilodon populator MF871700 1	Melogale moschata NC020644 1	0,18493663
Martes foina NC020643 1	Hemigalus derbyanus MH464791 1	0,18493782
Genetta abyssinica MG489822 1	Ailuropoda melanoleuca NC009492 5	0,18494469
Taxidea taxus NC020646 1	Cynogale bennetti KY117544 1	0,18494968
Leopardus guigna NC028321 1	Eumetopias jubatus NC004030 10	0,18495145
Tremarctos ornatus NC009969 2	Crossarchus platycephalus C7R66 1	0,18495195
Mustela nigripes NC024942 1	Arctocephalus australis MG023139 1	0,18495591
Crossarchus platycephalus C7R66 1	Arctocephalus townsendi NC008420 1	0,1849571
Mustela sibirica NC020637 6	Urva brachyura KY117547 1	0,1849573
Mungos mungo/gambianus SRR77048	Arctocephalus forsteri KT693377 17	0,1849575
Mustela nivalis T306 5	Lycalopex sechurae KT448284 1	0,18495791
Potos flavus T414 1	Urva javanica T413 1	0,18495858
Leopardus tigrinus NC028317 1	Conepatus chinga NC042596 1	0,1849609
Galidia elegans D146 1	Aonyx cinerea NC035814 2	0,18496166
Leopardus tigrinus NC028317 1	Tapirus terrestris T358	0,18496294
Taxidea taxus NC020646 1	Helogale parvula SRR7637809 1	0,18496361
Genetta genetta T297 1	Callorhinus ursinus NC008415 1	0,18496622
Civettictis civetta GLC19 1	Arctocephalus australis MG023139 1	0,18496625
Ictonyx striatus T299 1	Crossarchus platycephalus C7R66 1	0,18496798
Vulpes lagopus NC026529 3	Genetta servalina NC024568 2	0,18496822
Ursus arctos pruinosus MG066703 3	Martes melampus NC009678 1	0,18496825
Martes foina NC020643 1	Chrotogale owstoni T607 1	0,18496908
Ichneumia albicauda T603 1	Arctonyx collaris NC020645 1	0,18496941
Viverra tangalunga MH464792 1	Panthera uncia KP202269 1	0,18496957
Mustela nigripes NC024942 1	Cryptoprocta ferox CFC13 1	0,18496987
Lycaon pictus NC028427 2	Urva semitorquata MH464789 1	0,18497002
Viverra zibetha T609 1	Phocarctos hookeri NC008418 1	0,18497039
Vulpes corsac NC023958 1	Lynx rufus NC014456 3	0,18497117
Lynx canadensis NC028313 1	Arctocephalus forsteri KT693377 17	0,18497141
Vulpes lagopus NC026529 3	Prionailurus planiceps KY682741 4	0,18497184
Vulpes corsac NC023958 1	Neovison vison NC020641 3	0,18497217
Lynx canadensis NC028313 1	Lontra canadensis SRR10409165 1	0,18497218
Vulpes zerda KJ603240 1	Lynx canadensis NC028313 1	0,18497227
Lycaon pictus NC028427 2	Urva javanica T413 1	0,18497232
Leopardus guigna NC028321 1	Arctocephalus australis MG023139 1	0,18497369
Poecilogale albinucha T602 1	Paradoxurus hermaphroditus NC03959	0,18497425
Zalophus wollebaeki SRR4431565 1	Eupleres goudotii D128 1	0,18497503
Neovison vison NC020641 3	Helogale parvula SRR7637809 1	0,1849751
Ursus arctos pruinosus MG066703 3	Arctonyx collaris NC020645 1	0,18497598
Ursus arctos GU573486 5	Fossa fossana D350 1	0,18497618
Procyon lotor AB462049 4	Leopardus tigrinus NC028317 1	0,18497755
Prionailurus planiceps KY682741 4	Lutrogale perspicillata NC035811 1	0,1849781
Poecilogale albinucha T602 1	Leopardus jacobita NC028322 1	0,18497857
Panthera tigris NC010642 35	Mustela kathiah NC023210 1	0,18497859
Melogale moschata NC020644 1	Caracal caracal NC028306 1	0,18497877
Melogale moschata NC020644 1	Catopuma temminckii NC027115 41	0,18497927
Leopardus tigrinus NC028317 1	Arctotherium sp NC030174 1	0,18497938
Caracal caracal NC028306 1	Aonyx cinerea NC035814 2	0,1849796
Tremarctos ornatus NC009969 2	Ictonyx striatus T299 1	0,18497968
Mustela nivalis T306 5	Felis nigripes NC028309 1	0,18498006
Ursus maritimus GU573488 Svalbard	Otaria byronia OTAB 1	0,18498017
Otocyon megalotis SAF1 2	Leopardus tigrinus NC028317 1	0,18498047
Otocyon megalotis SAF1 2	Felis silvestris lybica KP202275 4	0,18498062
Otaria byronia OTAB 1	Catopuma badia NC028300 1	0,18498135
Procyon lotor AB462049 4	Leopardus geoffroyi NC028320 1	0,1849817
Procyon lotor AB462046 3	Leopardus guigna NC028321 1	0,18498175
Melursus ursinus NC009970 2	Lutra sumatrana NC035810 1	0,18498503
Ursus thibetanus thibetanus NC01111	Chrotogale owstoni T607 1	0,1849851
Ursus thibetanus thibetanus NC01111	Phocarctos hookeri NC008418 1	0,184986
Arctocephalus townsendi NC008420 1	Acinonyx jubatus NC005212 3	0,1849865
Melursus ursinus NC009970 2	Bassariscus sumichrasti SRX1099089 1	0,18498732
Prionodon pardicolor NC024569 2	Ommatophoca rossii AY377287etc 1	0,18498835
Ursus arctos GU573486 5	Genetta abyssinica MG489822 1	0,1849889
Mustela nigripes NC024942 1	Ailuropoda melanoleuca NC009492 5	0,18498957
Ursus thibetanus formosanus NC0093	Paradoxurus jerdoni MH464793 1	0,18499041
Tremarctos ornatus NC009969 2	Acinonyx jubatus NC005212 3	0,18499057
Ichneumia albicauda T603 1	Canis lupus chanco NC010340 4	0,18499108
Ursus arctos AP012576 6	Mustela itatsi NC034330 19	0,18499193
Ursus spelaeus NC011112 8	Lynx canadensis NC028313 1	0,18499495
Ursus thibetanus formosanus NC0093	Prionailurus rubiginosus NC028304 2	0,18499543
Mustela itatsi NC034330 19	Urva javanica/auropunctata NC006835	0,18499574
Ursus spelaeus NC011112 8	Felis margarita NC028308 1	0,18499619
Catopuma badia NC028300 1	Ailuropoda melanoleuca NC009492 5	0,18499793
Ursus thibetanus thibetanus NC01111	Prionailurus bengalensis NC028301 12	0,18499808
Ursus thibetanus laniger MH281753 2	Prionailurus bengalensis CKM45 20	0,18500021
Lontra canadensis SRR10409165 1	Eumetopias jubatus NC004030 10	0,18500115
Genetta genetta T297 1	Canis lupus familiaris NC002008 1231	0,18500364
Conepatus chinga NC042596 1	Civettictis civetta NC033378 1	0,18500584
Ursus arctos AP012576 6	Canis adustus KT448271 1	0,1850066
Leopardus pardalis NC028315 1	Arctocephalus forsteri NC004023 28	0,18500842
Otocolobus manul NC028323 1	Gulo gulo NC009685 3	0,18501112
Ursus thibetanus formosanus NC0093	Leopardus colocolo NC028314 1	0,18501283
Ursus spelaeus EU327344 13	Ictonyx striatus T299 1	0,18501517
Puma concolor NC016470 22	Cuon alpinus NC013445 3	0,18501569
Martes melampus NC009678 1	Urva javanica T413 1	0,18501979
Paradoxurus jerdoni MH464793 1	Enhydra lutris NC009692 1	0,18502012
Lontra canadensis SRR10409165 1	Callorhinus ursinus NC008415 1	0,18502194
Lontra canadensis SRR10409165 1	Fossa fossana D350 1	0,18502256
Fossa fossana D350 1	Canis latrans NC008093 7	0,18502611
Martes zibellina NC011579 39	Canis anthus NC027956 2	0,18502651
Urva javanica/auropunctata NC00683	Canis lupus familiaris NC002008 1231	0,18502653
Mungotictis decemlineata NC027828 1	Enhydra lutris NC009692 1	0,18502682
Galidictis fasciata DM333 1	Enhydra lutris NC009692 1	0,18502723
Urva javanica T413 1	Canis latrans NC008093 7	0,1850275
Bassaricyon neblina SRX1097850 1	Arctocephalus forsteri KT693377 17	0,18502782

Smilodon populator MF871700 1	Paradoxurus hermaphroditus NLNC 1	0,14682744
Hemigalus derbyanus MH464791 1	Galerella sanguinea T378 1	0,14683121
Pusa sibirica NC008432 2	Phocarctos hookeri NC008418 1	0,14684698
Pusa caspica NC008431 1	Neophoca cinerea NC008419 1	0,14684808
Pusa caspica NC008431 1	Arctocephalus gazella BK010918 1	0,14684971
Pusa caspica NC008431 1	Otaria byronia OTAB 1	0,14685027
Phoca groenlandica NC008429 54	Arctocephalus gazella BK010918 1	0,14685443
Pusa hispida NC 008433 1	Phocarctos hookeri NC008418 1	0,14685816
Prionailurus bengalensis NC028301 12	Urva semitorquata MH464789 1	0,14686024
Paradoxurus jerdoni MH464793 1	Leptailurus serval NC028316 1	0,14686082
Panthera pardus japonensis KJ866876 8	Civettictis civetta GLC19 1	0,14686309
Prionailurus bengalensis CKM45 20	Paradoxurus hermaphroditus NLNC 1	0,14686385
Hyaena hyaena NC020669 1	Crossarchus platycephalus C7R66 1	0,14686422
Leopardus wiedii NC028318 1	Galidictis fasciata DM333 1	0,14686853
Prionodon pardicolor NC024569 2	Prionailurus bengalensis NC028301 12	0,14687221
Prionodon linsang ERR2391707 1	Hemigalus derbyanus MH464791 1	0,14688964
Felis nigripes NC028309 1	Diplogale hosei MH464790 1	0,14690214
Vulpes corsac NC023958 1	Urocyon littoralis catalinae KP129018 15	0,14690635
Leopardus wiedii NC028318 1	Chrotogale owstoni T607 1	0,14691566
Civettictis civetta GLC19 1	Bdeogale nigripes GLC15 1	0,14692384
Panthera leo spelaea KX258452 2	Civettictis civetta GLC19 1	0,14692786
Profelis aurata NC028299 1	Paradoxurus hermaphroditus NLNC 1	0,14692843
Phoca largha NC008430 1	Otaria byronia OTAB 1	0,14692855
Mungotictis decemlineata NC027828 1	Lynx canadensis NC028313 1	0,14693122
Profelis aurata NC028299 1	Genetta genetta T297 1	0,1469319
Urva javanica/auropunctata NC006835 1	Genetta abyssinica MG489822 1	0,14693446
Prionodon pardicolor NC024569 2	Prionailurus planiceps NC028312 6	0,14693889
Viverricula indica KX891745 1	Urva javanica/auropunctata NC006835 1	0,14695629
Viverricula indica NC025296 2	Panthera leo NERO 19	0,14696306
Neophoca cinerea NC008419 1	Mirounga leonina NC008422 1	0,14698146
Leopardus tigrinus NC028317 1	Chrotogale owstoni T607 1	0,14698327
Chrotogale owstoni T607 1	Caracal caracal NC028306 1	0,14698416
Urva javanica T413 1	Hemigalus derbyanus MH464791 1	0,14698455
Prionailurus bengalensis CKM45 20	Chrotogale owstoni T607 1	0,14698827
Vulpes ferrilata NC027935 1	Chrysocyon brachyurus NC024172 1	0,14698903
Paradoxurus hermaphroditus NLNC 1	Nandinia binotata NC024567 1	0,14698973
Prionailurus rubiginosus NC028304 2	Paradoxurus hermaphroditus NLNC 1	0,14699526
Prionailurus bengalensis NC028301 12	Atilax paludinosus T606 1	0,14699562
Panthera leo spelaea KX258452 2	Nandinia binotata NC024567 1	0,14699739
Parahyaena brunnea NC038159 15	Galidictis fasciata DM333 1	0,1469982
Otaria byronia OTAB 1	Monachus schauinslandi NC008421 1	0,1470001
Genetta genetta T297 1	Catopuma temminckii NC027115 41	0,14700113
Leopardus pardalis NC028315 1	Chrotogale owstoni T607 1	0,1470501
Urva javanica T413 1	Eupleres goudotii D128 1	0,14705113
Pusa caspica NC008431 1	Arctocephalus pusillus NC008417 1	0,14705333
Mirounga angustirostris SRR10331586 1	Arctocephalus forsteri KT693377 17	0,14705826
Genetta servalina NC024568 2	Crossarchus platycephalus C7R66 1	0,14705867
Crossarchus platycephalus C7R66 1	Civettictis civetta GLC19 1	0,14705887
Paradoxurus hermaphroditus NLNC 1	Leopardus pardalis NC028315 1	0,14706002
Ichneumia albicauda T603 1	Hemigalus derbyanus MH464791 1	0,1470623
Salanoia concolor D378 1	Catopuma temminckii NC027115 41	0,14706428
Puma yagouaroundi NC028311 1	Genetta genetta T297 1	0,14706603
Genetta genetta T297 1	Felis chaus NC028307 1	0,14706641
Nandinia binotata NC024567 1	Galidia elegans D146 1	0,14712073
Otaria byronia OTAB 1	Mirounga leonina NC008422 1	0,14712246
Phoca fasciata NC008428 1	Arctocephalus forsteri KT693377 17	0,14712352
Phocarctos hookeri NC008418 1	Lobodon carcinophaga NC008423 1	0,14712791
Viverricula indica KX891751 1	Pardofelis marmorata NLN3 2	0,14713046
Parahyaena brunnea NC038159 15	Crossarchus platycephalus C7R66 1	0,1471331
Paradoxurus hermaphroditus NC039591 1	Acinonyx jubatus NC005212 3	0,14714043
Leopardus jacobita NC028322 1	Arctictis binturong T605 2	0,14714296
Lynx rufus NC014456 3	Arctictis binturong T605 2	0,14714403
Leopardus colocolo NC028314 1	Cryptoprocta ferox CFC13 1	0,14715593
Phocarctos hookeri NC008418 1	Mirounga leonina NC008422 1	0,14718257
Erignathus barbatus NC008426 1	Arctocephalus australis MG023139 1	0,14718381
Nandinia binotata NC024567 1	Mungos mungo MMC7 1	0,14718725
Ichneumia albicauda T603 1	Galidia elegans D146 1	0,14719021
Viverricula indica KX891751 1	Mungos mungo MMC7 1	0,1471919
Diplogale hosei MH464790 1	Catopuma badia NC028300 1	0,14719735
Viverricula indica NC025296 2	Crossarchus platycephalus C7R66 1	0,1471986
Panthera uncia NC010638 1	Civettictis civetta GLC19 1	0,1471996
Ichneumia albicauda T603 1	Cryptoprocta ferox CFC13 1	0,14720519
Paradoxurus hermaphroditus NC039591 1	Catopuma badia NC028300 1	0,14720584
Zalophus wollebaeki SRR4431565 1	Phoca largha NC008430 1	0,14720683
Lynx lynx NC027083 4	Arctictis binturong T605 2	0,1472111
Monachus monachus NC044972 5	Arctocephalus australis MG023139 1	0,14722142
Panthera pardus NC010641 5	Civettictis civetta GLC19 1	0,14723113
Vulpes ferrilata NC027935 1	Urocyon cinereoargenteus NC026723 21	0,14724298
Vulpes ferrilata NC027935 1	Urocyon littoralis catalinae KP129018 15	0,14724298
Leopardus pardalis T262 1	Chrotogale owstoni T607 1	0,14725168
Pusa sibirica NC008432 2	Arctocephalus pusillus NC008417 1	0,14725518
Pusa hispida NC 008433 1	Arctocephalus forsteri KT693377 17	0,14726293
Neophoca cinerea NC008419 1	Monachus schauinslandi NC008421 1	0,14726315
Prionailurus rubiginosus NC028304 2	Paradoxurus hermaphroditus NC039591 1	0,14726427
Puma concolor NC016470 22	Atilax paludinosus T606 1	0,14726442
Leopardus tigrinus NC028317 1	Fossa fossana D350 1	0,14726591
Meles anakuma NC009677 1	Galictis vittata T412 1	0,14726742
Leopardus jacobita NC028322 1	Galidictis fasciata DM333 1	0,14727227
Zalophus wollebaeki SRR4431565 1	Phoca vitulina NC001325 1	0,14727433
Leopardus colocolo NC028314 1	Crossarchus platycephalus C7R66 1	0,14728287
Smilodon populator MF871700 1	Prionodon linsang ERR2391707 1	0,14728402
Monachus schauinslandi NC008421 1	Arctocephalus australis MG023139 1	0,14732872
Lynx lynx NC027083 4	Crossarchus platycephalus C7R66 1	0,14732942
Phoca largha NC008430 1	Neophoca cinerea NC008419 1	0,14733028
Paguma larvata PDDS11 2	Leopardus tigrinus NC028317 1	0,14733167
Viverricula indica KX891751 1	Lynx canadensis NC028313 1	0,1473318
Mungos mungo/gambianus SRR7704821 1	Leopardus wiedii NC028318 1	0,14733239
Leopardus wiedii NC028318 1	Atilax paludinosus T606 1	0,14733356
Mirounga angustirostris SRR10331586 1	Arctocephalus pusillus NC008417 1	0,14733461
Galerella sanguinea T378 1	Civettictis civetta GLC19 1	0,14733876
Leopardus pardalis NC028315 1	Cryptoprocta ferox CFC13 1	0,14734375
Leopardus wiedii NC028318 1	Cryptoprocta ferox CFC13 1	0,1473438
Taxidea taxus NC020646 1	Lutrogale perspicillata NC035811 1	0,14734517

Procyon lotor AB462049 4	Fossa fossana D350 1	0,1850279
Leopardus pardalis T262 1	Ailurus fulgens styani NC009691 1	0,18503268
Lynx rufus NC014456 3	Ailurus fulgens styani NC009691 1	0,18503268
Canis anthus NC027956 2	Arctonyx collaris NC020645 1	0,18503296
Leopardus geoffroyi NC028320 1	Tapirus terrestris T358	0,18503439
Felis catus NC001700 2	Callorhinus ursinus NC008415 1	0,18503477
Lynx rufus NC014456 3	Canis mesomelas KT448280 1	0,18503478
Mustela altaica NC021751 1	Cynictis penicillata T375 1	0,18503487
Panthera uncia KP202269 1	Mustela nigripes NC024942 1	0,18503746
Mustela eversmannii NC028013 1	Felis nigripes NC028309 1	0,18503751
Mustela putorius NC020638 4	Felis chaus NC028307 1	0,18503845
Vulpes ferrilata NC027935 1	Lynx rufus NC014456 3	0,18503879
Melogale moschata V0735A 1	Felis margarita NC028308 1	0,18503903
Mustela kathiah NC023210 1	Ichneumia albicauda T603 1	0,1850394
Vulpes corsac NC023958 1	Leptailurus serval NC028316 1	0,18503949
Vulpes corsac NC023958 1	Puma yagouaroundi NC028311 1	0,18503952
Prionailurus planiceps NC028312 6	Arctocephalus gazella BK010918 1	0,18503958
Parahyaena brunnea NC038159 15	Mustela putorius NC020638 4	0,185040 7
Ursus maritimus GU573488 Svalbard	Meles anakuma NC009677 1	0,18504187
Lynx canadensis NC028313 1	Lutrogale perspicillata NC035811 1	0,18504331
Panthera onca NC022842 1	Meles leucurus NC039173 4	0,18504348
Felis nigripes NC028309 1	Arctocephalus pusillus NC008417 1	0,18504454
Nasua nasua NC020647 1	Galictis vittata T412 1	0,18504474
Viverricula indica KX891745 1	Ursus arctos isabellinus 18B5 2	0,18504474
Prionailurus rubiginosus NC028304 2	Arctocephalus pusillus NC008417 1	0,18504529
Pardofelis marmorata NLN3 2	Mustela altaica NC021751 1	0,1850455
Prionailurus planiceps NC028312 6	Poecilogale albinucha T602 1	0,18504579
Puma concolor NC016470 22	Poecilogale albinucha T602 1	0,18504597
Poecilogale albinucha T602 1	Leopardus tigrinus NC028317 1	0,18504651
Puma yagouaroundi NC028311 1	Arctotherium sp NC030174 1	0,18504708
Prionailurus planiceps KY682741 4	Otaria byronia OTAB 1	0,18504749
Panthera leo spelaea KX258452 2	Mustela frenata NC020640 1	0,18504755
Panthera tigris NC010642 35	Mustela erminea T305 2	0,18504868
Prionailurus rubiginosus NC028304 2	Otaria byronia OTAB 1	0,18504894
Viverricula indica NC025296 2	Speothos venaticus C48 2	0,18505019
Ursus spelaeus EU327344 13	Chrotogale owstoni T607 1	0,18505244
Galerella sanguinea T378 1	Canis lupus chanco NC010340 4	0,18505344
Viverra zibetha T609 1	Ailuropoda melanoleuca NC009492 5	0,1850547
Lycalopex sechurae KT448284 1	Ailurus fulgens NC011124 1	0,18505719
Parahyaena brunnea NC038159 15	Neovison vison NC020641 3	0,18505904
Smilodon populator MF871700 1	Chrysocyon brachyurus NC024172 1	0,18505959
Ursus spelaeus EU327344 13	Profelis aurata NC028299 1	0,18506017
Ursus thibetanus laniger MH281753 2	Lynx pardinus NC028319 161	0,18506169
Ursus thibetanus laniger MH281753 2	Paradoxurus jerdoni MH464793 1	0,18506193
Mephitis mephitis NC020648 1	Civettictis civetta GLC19 1	0,18506277
Prionailurus bengalensis NC028301 12	Galictis vittata T412 1	0,18506357
Ursus spelaeus NC011112 8	Leopardus jacobita NC028322 1	0,18506393
Neophoca cinerea NC008419 1	Genetta abyssinica MG489822 1	0,18506396
Ursus thibetanus thibetanus NC01111[illegible]	Leptailurus serval NC028316 1	0,18506567
Prionailurus rubiginosus NC028304 2	Melursus ursinus NC009970 2	0,18506601
Melursus ursinus NC009970 2	Catopuma temminckii NC027115 41	0,18506704
Panthera leo NERO 19	Enhydra lutris NC009692 1	0,18506758
Meles leucurus NC039173 4	Canis lupus chanco NC010340 4	0,18507174
Canis adustus KT448271 1	Arctocephalus gazella BK010918 1	0,18507276
Otaria byronia OTAB 1	Hemigalus derbyanus MH464791 1	0,18507475
Melursus ursinus NC009970 2	Genetta abyssinica MG489822 1	0,18507686
Leopardus tigrinus NC028317 1	Arctocephalus forsteri NC004023 28	0,18507741
Meles leucurus NC039173 4	Canis adustus KT448271 1	0,18508148
Paguma larvata PDDS11 2	Cuon alpinus NC013445 3	0,18508242
Mungos mungo/gambianus SRR77048	Lutra sumatrana NC035810 1	0,18508676
Cuon alpinus NC013445 3	Catopuma temminckii NC027115 41	0,18508795
Puma concolor NC016470 22	Canis mesomelas KT448280 1	0,18508834
Phoca groenlandica NC008429 54	Cynogale bennetti KY117544 1	0,18508871
Eupleres goudotii D128 1	Arctonyx collaris NC020645 1	0,18508998
Mungos mungo MMC7 1	Melogale moschata KP726273 1	0,18509033
Martes zibellina NC011579 39	Xenogale naso C07XAR110 1	0,18509096
Mungos mungo/gambianus SRR77048	Arctocephalus townsendi NC008420 1	0,18509121
Ursus arctos GU573491 207	Smilodon populator MF871700 1	0,18509339
Viverra tangalunga MH464792 1	Cynictis penicillata T375 1	0,18509354
Potos flavus T414 1	Eupleres goudotii D128 1	0,18509416
Leopardus guigna NC028321 1	Cuon alpinus NC013445 3	0,18509448
Cuon alpinus NC013445 3	Arctictis binturong T605 2	0,18509515
Procyon lotor AB462046 3	Crossarchus platycephalus C7R66 1	0,18509527
Cynogale bennetti KY117544 1	Ailurus fulgens styani NC009691 1	0,18509686
Viverricula indica KX891751 1	Lutra sumatrana NC035810 1	0,18509801
Lutra lutra NC011358 9	Felis catus NC001700 2	0,18509987
Lutra sumatrana NC035810 1	Civettictis civetta GLC19 1	0,18509988
Pardofelis marmorata NLN3 2	Meles meles T303 3	0,1851
Lutra lutra NC011358 9	Felis nigripes NC028309 1	0,18510008
Mustela nivalis T306 5	Eupleres goudotii D128 1	0,18510048
Felis silvestris lybica KP202275 4	Conepatus chinga NC042596 1	0,18510069
Prionailurus planiceps KY682741 4	Ailurus fulgens styani NC009691 1	0,18510205
Panthera uncia NC010638 1	Mustela kathiah NC023210 1	0,18510365
Zalophus wollebaeki SRR4431565 1	Hemigalus derbyanus MH464791 1	0,18510386
Ursus maritimus GU573488 Svalbard	Martes pennanti NC020664 16	0,18510412
Potos flavus T414 1	Panthera uncia KP202269 1	0,18510452
Panthera onca KP202264 2	Mustela putorius NC020638 4	0,18510464
Salanoia concolor D378 1	Mustela nivalis T306 5	0,18510561
Martes pennanti NC020664 16	Canis anthus NC027956 2	0,1851061
Prionodon pardicolor NC024569 2	Mustela putorius NC020638 4	0,18510704
Genetta servalina NC024568 2	Aonyx cinerea NC035814 2	0,18510759
Lynx canadensis NC028313 1	Canis anthus NC027956 2	0,18510759
Leopardus tigrinus NC028317 1	Canis anthus NC027956 2	0,18510772
Profelis aurata NC028299 1	Martes zibellina NC011579 39	0,18510799
Leopardus colocolo NC028314 1	Cuon alpinus NC013445 3	0,1851083
Lontra canadensis SRR10409165 1	Leopardus jacobita NC028322 1	0,18510874
Prionailurus bengalensis NC028301 12	Lontra canadensis SRR10409165 1	0,18510877
Vulpes corsac NC023958 1	Catopuma temminckii NC027115 41	0,18510933
Ursus thibetanus mupinensis NC00875	Procyon lotor AB462049 4	0,18511134
Ursus arctos GU573491 207	Bassariscus sumichrasti SRX1099089 1	0,18511184
Ursus maritimus GU573488 Svalbard	Aonyx cinerea NC035814 2	0,18511233
Ursus maritimus GU573488 Svalbard	Mustela altaica NC021751 1	0,18511386
Leopardus tigrinus NC028317 1	Aonyx cinerea NC035814 2	0,18511471

Leopardus geoffroyi NC028320 1	Arctictis binturong T605 2	0,14735284
Homotherium latidens MF871702 3	Fossa fossana D350 1	0,14735974
Neophoca cinerea NC008419 1	Halichoerus grypus NC001602 2	0,14735983
Hemigalus derbyanus MH464791 1	Bdeogale nigripes GLC15 1	0,14736557
Cystophora cristata NC008427 1	Arctocephalus australis MG023139 1	0,14738685
Phoca fasciata NC008428 1	Arctocephalus australis MG023139 1	0,14739268
Leopardus geoffroyi NC028320 1	Chrotogale owstoni T607 1	0,14739378
Viverricula indica NC025296 2	Prionodon linsang ERR2391707 1	0,14739522
Urva brachyura KY117547 1	Cryptoprocta ferox CFC13 1	0,14739648
Urocyon cinereoargenteus NC026723 21	Chrysocyon brachyurus NC024172 1	0,14739726
Fossa fossana D350 1	Felis silvestris lybica KP202275 4	0,14739811
Paradoxurus hermaphroditus NC039591 1	Caracal caracal NC028306 1	0,14739935
Paguma larvata PDDS11 2	Caracal caracal NC028306 1	0,14740051
Lynx lynx NC027083 4	Galidictis fasciata DM333 1	0,14740245
Prionailurus bengalensis CKM45 20	Mungotictis decemlineata NC027828 1	0,14740738
Monachus monachus NC044972 5	Arctocephalus townsendi NC008420 1	0,14742284
Otocolobus manul NC028323 1	Chrotogale owstoni T607 1	0,14745571
Profelis aurata NC028299 1	Fossa fossana D350 1	0,14746583
Panthera pardus NC010641 5	Civettictis civetta NC033378 1	0,14746608
Paradoxurus hermaphroditus NLNC 1	Caracal caracal NC028306 1	0,14746697
Leopardus wiedii NC028318 1	Urva semitorquata MH464789 1	0,14746758
Leopardus wiedii NC028318 1	Eupleres goudotii D128 1	0,14747071
Prionailurus rubiginosus NC028304 2	Galidictis fasciata DM333 1	0,14747204
Paradoxurus hermaphroditus NC039591 1	Leopardus colocolo NC028314 1	0,14748697
Panthera pardus japonensis KJ866876 8	Civettictis civetta NC033378 1	0,14750208
Genetta genetta T297 1	Cynogale bennetti KY117544 1	0,14751644
Homotherium latidens MF871702 3	Diplogale hosei MH464790 1	0,14751984
Leopardus geoffroyi NC028320 1	Diplogale hosei MH464790 1	0,14752185
Mungos mungo/gambianus SRR7704821 1	Genetta abyssinica MG489822 1	0,14753237
Prionailurus rubiginosus NC028304 2	Helogale parvula SRR7637809 1	0,14753354
Ommatophoca rossii AY377287etc 1	Callorhinus ursinus NC008415 1	0,14753412
Profelis aurata NC028299 1	Paguma larvata PDDS11 2	0,14753531
Viverricula indica NC025296 2	Panthera pardus japonensis KJ866876 8	0,14753872
Leopardus geoffroyi NC028320 1	Genetta genetta T297 1	0,1475407
Leopardus colocolo NC028314 1	Eupleres goudotii D128 1	0,14755276
Urva javanica/auropunctata NC006835 1	Eupleres goudotii D128 1	0,14755946
Halichoerus grypus NC001602 2	Arctocephalus pusillus NC008417 1	0,14756487
Viverra zibetha T609 1	Panthera pardus NC010641 5	0,14756581
Smilodon populator MF871700 1	Genetta genetta T297 1	0,14756808
Paguma larvata PDDS11 2	Fossa fossana D350 1	0,14759561
Prionodon linsang ERR2391707 1	Prionailurus bengalensis NC028301 12	0,14759591
Viverra zibetha T609 1	Crossarchus platycephalus C7R66 1	0,14759706
Mungos mungo MMC7 1	Civettictis civetta GLC19 1	0,14759754
Pusa hispida NC 008433 1	Arctocephalus australis MG023139 1	0,14759956
Paguma larvata PDDS11 2	Felis chaus NC028307 1	0,14760272
Viverra zibetha T609 1	Panthera pardus japonensis KJ866876 8	0,14760309
Salanoia concolor D378 1	Felis silvestris lybica KP202275 4	0,14760543
Mungos mungo MMC7 1	Hemigalus derbyanus MH464791 1	0,14760782
Paguma larvata PDDS11 2	Leopardus guigna NC028321 1	0,14760803
Phocarctos hookeri NC008418 1	Erignathus barbatus NC008426 1	0,14765431
Smilodon populator MF871700 1	Prionodon pardicolor NC024569 2	0,14765522
Nandinia binotata NC024567 1	Bdeogale nigripes GLC15 1	0,14765689
Pusa sibirica NC008432 2	Otaria byronia OTAB 1	0,14765838
Felis chaus NC028307 1	Chrotogale owstoni T607 1	0,1476604
Prionodon linsang ERR2391707 1	Paradoxurus jerdoni MH464793 1	0,14766292
Urva semitorquata MH464789 1	Civettictis civetta GLC19 1	0,14766474
Prionailurus rubiginosus NC028304 2	Urva semitorquata MH464789 1	0,14766808
Genetta servalina NC024568 2	Galidictis fasciata DM333 1	0,14766861
Prionailurus rubiginosus NC028304 2	Atilax paludinosus T606 1	0,14766891
Prionailurus bengalensis CKM45 20	Paradoxurus jerdoni MH464793 1	0,1476721
Salanoia concolor D378 1	Puma yagouaroundi NC028311 1	0,14767423
Prionailurus viverrinus NC028305 1	Mungotictis decemlineata NC027828 1	0,1476763
Lynx lynx NC027083 4	Cryptoprocta ferox CFC13 1	0,14767656
Genetta abyssinica MG489822 1	Galerella sanguinea T378 1	0,14768323
Mungos mungo/gambianus SRR7704821 1	Hemigalus derbyanus MH464791 1	0,14768752
Paradoxurus hermaphroditus NC039591 1	Homotherium latidens MF871702 3	0,14768879
Civettictis civetta NC033378 1	Bdeogale nigripes GLC15 1	0,14769574
Pusa caspica NC008431 1	Arctocephalus townsendi NC008420 1	0,14772345
Leopardus jacobita NC028322 1	Chrotogale owstoni T607 1	0,14772387
Otaria byronia OTAB 1	Cystophora cristata NC008427 1	0,147726
Viverricula indica KX891745 1	Mungos mungo/gambianus SRR7704821 1	0,14773154
Urva semitorquata MH464789 1	Genetta servalina NC024568 2	0,14773174
Lynx rufus NC014456 3	Crossarchus platycephalus C7R66 1	0,14773388
Leopardus colocolo NC028314 1	Diplogale hosei MH464790 1	0,14773466
Salanoia concolor D378 1	Prionailurus bengalensis CKM45 20	0,14774442
Urocyon cinereoargenteus NC026723 21	Lycalopex sechurae KT448284 1	0,14776009
Neophoca cinerea NC008419 1	Monachus monachus NC044972 5	0,14776282
Halichoerus grypus NC001602 2	Arctocephalus gazella BK010918 1	0,14776565
Urocyon littoralis catalinae KP129018 15	Canis mesomelas KT448280 1	0,14776957
Phocarctos hookeri NC008418 1	Phoca fasciata NC008428 1	0,14779598
Puma yagouaroundi NC028311 1	Fossa fossana D350 1	0,14780205
Viverricula indica NC025296 2	Bdeogale nigripes GLC15 1	0,14780258
Mungotictis decemlineata NC027828 1	Genetta servalina NC024568 2	0,14780271
Profelis aurata NC028299 1	Paradoxurus hermaphroditus NC039591 1	0,14780337
Phoca vitulina NC001325 1	Otaria byronia OTAB 1	0,14780432
Prionailurus bengalensis NC028301 12	Eupleres goudotii D128 1	0,14780457
Leopardus pardalis T262 1	Atilax paludinosus T606 1	0,14780478
Viverra zibetha T609 1	Panthera uncia KP202269 1	0,14780517
Smilodon populator MF871700 1	Crossarchus platycephalus C7R66 1	0,14780684
Mungotictis decemlineata NC027828 1	Leopardus tigrinus NC028317 1	0,14780955
Viverricula indica KX891745 1	Panthera onca NC022842 1	0,14781277
Lynx canadensis NC028313 1	Arctictis binturong T605 2	0,14781725
Salanoia concolor D378 1	Acinonyx jubatus NC005212 3	0,14782362
Leopardus pardalis NC028315 1	Diplogale hosei MH464790 1	0,14783034
Civettictis civetta NC033378 1	Atilax paludinosus T606 1	0,14783192
Galerella sanguinea T378 1	Civettictis civetta NC033378 1	0,14784102
Fossa fossana D350 1	Chrotogale owstoni T607 1	0,14785017
Vulpes lagopus NC026529 3	Speothos venaticus C48 2	0,14785568
Nandinia binotata NC024567 1	Mungos mungo/gambianus SRR7704821 1	0,14785887
Viverra zibetha T609 1	Xenogale naso C07XAR110 1	0,14786601
Paradoxurus hermaphroditus NC039591 1	Leopardus wiedii NC028318 1	0,14786761
Mungos mungo/gambianus SRR7704821 1	Leopardus geoffroyi NC028320 1	0,14787172
Parahyaena brunnea NC038159 15	Mungotictis decemlineata NC027828 1	0,14787361

Panthera onca NC022842 1	Melogale moschata KP726273 1	0,18511599
Civettictis civetta NC033378 1	Canis mesomelas KT448280 1	0,18511713
Otocolobus manul NC028323 1	Arctodus simus NC011116 1	0,18511726
Speothos venaticus C48 2	Leopardus wiedii NC028318 1	0,18511763
Ursus arctos pruinosus MG066703 3	Aonyx cinerea NC035814 2	0,18511838
Ursus arctos AP012576 6	Arctocephalus forsteri KT693377 17	0,18511991
Lycaon pictus NC028427 2	Genetta genetta T297 1	0,18512097
Zalophus wollebaeki SRR4431565 1	Felis catus NC001700 2	0,18512191
Panthera onca NC022842 1	Aonyx cinerea NC035814 2	0,18512195
Ursus maritimus NC003428 31	Leopardus geoffroyi NC028320 1	0,18512213
Panthera uncia KP202269 1	Canis aureus KT448274 1	0,18512261
Ursus thibetanus laniger MH281753 2	Aonyx cinerea NC035814 2	0,1851227
Vulpes corsac NC023958 1	Leopardus colocolo NC028314 1	0,18512425
Speothos venaticus C48 2	Hemigalus derbyanus MH464791 1	0,18512693
Ursus arctos isabellinus 18B5 2	Arctictis binturong T605 2	0,18512829
Mustela nivalis T306 5	Melursus ursinus NC009970 2	0,18512908
Ursus spelaeus NC011112 8	Felis catus NC001700 2	0,18512999
Galictis vittata T412 1	Catopuma badia NC028300 1	0,18513136
Ursus spelaeus NC011112 8	Puma concolor NC016470 22	0,18513167
Zalophus californianus NC008416 1	Felis catus NC001700 2	0,1851362
Leopardus guigna NC028321 1	Chrysocyon brachyurus NC024172 1	0,18513751
Ursus thibetanus formosanus NC00933	Ictonyx striatus T299 1	0,18513834
Salanoia concolor D378 1	Martes flavigula NC012141 3	0,18514508
Ursus maritimus NC003428 31	Smilodon populator MF871700 1	0,18515015
Leopardus colocolo NC028314 1	Helarctos malayanus NC009968 2	0,18515031
Cystophora cristata NC008427 1	Tapirus terrestris T358	0,18515246
Viverricula indica KX891745 1	Tapirus terrestris T358	0,18515339
Mustela sibirica AP017394 11	Canis mesomelas KT448280 1	0,18515378
Enhydra lutris NC009692 1	Atilax paludinosus T606 1	0,18515587
Ursus arctos GU573491 207	Gulo gulo NC009685 3	0,1851574
Martes americana NC020642 1	Chrotogale owstoni T607 1	0,18515943
Vulpes vulpes NC008434 5	Fossa fossana D350 1	0,18516101
Nyctereutes procyonoides NC013700 3	Helogale parvula SRR7637809 1	0,18516181
Conepatus chinga NC042596 1	Arctocephalus gazella BK010918 1	0,18516321
Vulpes vulpes NC008434 5	Salanoia concolor D378 1	0,18516409
Prionailurus rubiginosus NC028304 2	Conepatus chinga NC042596 1	0,18516453
Leopardus pardalis NC028315 1	Ailurus fulgens NC011124 1	0,18516725
Leopardus guigna NC028321 1	Tapirus terrestris T358	0,18516952
Lutra sumatrana NC035810 1	Cryptoprocta ferox CFC13 1	0,18516962
Prionodon linsang ERR2391707 1	Phoca groenlandica NC008429 54	0,18516971
Vulpes ferrilata NC027935 1	Genetta servalina NC024568 2	0,18517075
Parahyaena brunnea NC038159 15	Lutra lutra LC050126 1	0,18517125
Mustela frenata NC020640 1	Hyaena hyaena NC020669 1	0,18517159
Galerella sanguinea T378 1	Aonyx cinerea NC035814 2	0,18517187
Otocyon megalotis SAF1 2	Mustela frenata NC020640 1	0,18517233
Martes pennanti NC020664 16	Canis latrans NC008093 7	0,18517257
Lutra sumatrana NC035810 1	Felis chaus NC028307 1	0,18517266
Paradoxurus hermaphroditus NLNC 1	Melogale moschata NC020644 1	0,18517275
Caracal caracal NC028306 1	Arctocephalus australis MG023139 1	0,18517312
Prionailurus bengalensis NC028301 12	Arctocephalus forsteri KT693377 17	0,18517326
Otocolobus manul NC028323 1	Arctocephalus gazella BK010918 1	0,18517406
Ursus thibetanus thibetanus NC01111	Conepatus chinga NC042596 1	0,18517427
Vulpes ferrilata NC027935 1	Leptailurus serval NC028316 1	0,18517441
Civettictis civetta GLC19 1	Aonyx cinerea NC035814 2	0,18517473
Vulpes zerda KJ603240 1	Leopardus jacobita NC028322 1	0,18517563
Prionailurus planiceps KY682741 4	Phocarctos hookeri NC008418 1	0,18517628
Prionailurus planiceps NC028312 6	Neophoca cinerea NC008419 1	0,18517658
Zalophus wollebaeki SRR4431565 1	Salanoia concolor D378 1	0,18517663
Vulpes ferrilata NC027935 1	Catopuma temminckii NC027115 41	0,18517664
Paguma larvata PDDS11 2	Arctocephalus pusillus NC008417 1	0,18517794
Neophoca cinerea NC008419 1	Leopardus tigrinus NC028317 1	0,18517801
Chrotogale owstoni T607 1	Arctocephalus forsteri NC004023 28	0,18517803
Zalophus wollebaeki SRR4431565 1	Bdeogale nigripes GLC15 1	0,18517825
Fossa fossana D350 1	Chrysocyon brachyurus NC024172 1	0,18517841
Viverra tangalunga MH464792 1	Ursus maritimus NC003428 31	0,18517849
Ursus thibetanus mupinensis NC00875	Procyon lotor AB462046 3	0,18517919
Ursus maritimus NC003428 31	Bassariscus sumichrasti SRX1099089 1	0,18517922
Ursus arctos EU497665 29	Bassariscus sumichrasti SRX1099089 1	0,18517926
Ursus maritimus NC003428 31	Aonyx cinerea NC035814 2	0,18517961
Ursus arctos EU497665 29	Aonyx cinerea NC035814 2	0,18517962
Panthera tigris amoyensis NC014770 2	Mustela frenata NC020640 1	0,18517986
Pardofelis marmorata NLN3 2	Melogale moschata NC020644 1	0,18518002
Procyon lotor AB462049 4	Leptailurus serval NC028316 1	0,18518035
Prionailurus bengalensis NC028301 12	Lutrogale perspicillata NC035811 1	0,18518119
Melogale moschata NC020644 1	Chrysocyon brachyurus NC024172 1	0,18518393
Ursus thibetanus formosanus NC00933	Crossarchus platycephalus C7R66 1	0,18518451
Prionailurus bengalensis CKM45 20	Aonyx cinerea NC035814 2	0,18518463
Poecilogale albinucha T602 1	Leopardus guigna NC028321 1	0,18518512
Chrysocyon brachyurus NC024172 1	Ailurus fulgens styani NC009691 1	0,18518537
Ursus thibetanus mupinensis NC00875	Prionailurus planiceps NC028312 6	0,18518704
Ursus arctos AP012576 6	Arctocephalus australis MG023139 1	0,18518728
Profelis aurata NC028299 1	Martes foina NC020643 1	0,18518756
Vulpes lagopus NC026529 3	Acinonyx jubatus NC005212 3	0,18518983
Zalophus wollebaeki SRR4431565 1	Puma yagouaroundi NC028311 1	0,18519022
Melursus ursinus NC009970 2	Melogale moschata NC020644 1	0,18519206
Ursus spelaeus NC011112 8	Phocarctos hookeri NC008418 1	0,18519248
Ursus arctos GU573486 5	Martes foina NC020643 1	0,18519276
Ursus thibetanus formosanus NC00933	Paguma larvata PDDS11 2	0,18519332
Ursus spelaeus EU327344 13	Lynx pardinus NC028319 161	0,18519349
Lutra lutra LC050126 1	Canis lupus chanco NC010340 4	0,18519357
Ursus spelaeus EU327344 13	Felis silvestris lybica KP202275 4	0,18519358
Ichneumia albicauda T603 1	Helarctos malayanus NC009968 2	0,18519519
Martes foina NC020643 1	Arctictis binturong T605 2	0,18519618
Leopardus wiedii NC028318 1	Helarctos malayanus NC009968 2	0,18519835
Ursus spelaeus NC011112 8	Leopardus tigrinus NC028317 1	0,18519862
Lycalopex sechurae KT448284 1	Civettictis civetta GLC19 1	0,1851996
Mustela nivalis T306 5	Arctocephalus forsteri NC004023 28	0,18520199
Speothos venaticus C48 2	Acinonyx jubatus NC005212 3	0,1852022
Helarctos malayanus NC009968 2	Canis aureus KT448274 1	0,1852047
Ursus americanus JX196366 3	Martes pennanti NC020664 16	0,18520536
Panthera pardus NC010641 5	Enhydra lutris NC009692 1	0,18521154
Ursus americanus JX196366 3	Mustela sibirica AP017394 11	0,18521206
Martes flavigula NC012141 3	Caracal caracal NC028306 1	0,18521425

Genetta genetta T297 1	Felis catus NC001700 2	0,14787397
Leopardus pardalis NC028315 1	Eupleres goudotii D128 1	0,14787473
Viverra zibetha T609 1	Panthera tigris amoyensis NC014770 2	0,14787816
Viverricula indica NC025296 2	Ichneumia albicauda T603 1	0,14787966
Viverricula indica NC025296 2	Panthera pardus NC010641 5	0,14790572
Urva javanica T413 1	Galidia elegans D146 1	0,14792962
Speothos venaticus C48 2	Otocyon megalotis SAF1 2	0,14793015
Helogale parvula SRR7637809 1	Civettictis civetta GLC19 1	0,14793423
Crossarchus platycephalus C7R66 1	Catopuma badia NC028300 1	0,14793457
Monachus schauinslandi NC008421 1	Arctocephalus forsteri KT693377 17	0,14793481
Suricata suricatta SSM10 1	Cryptoprocta ferox CFC13 1	0,14793487
Pusa hispida NC 008433 1	Arctocephalus townsendi NC008420 1	0,14793597
Prionailurus bengalensis NC028301 12	Crossarchus platycephalus C7R66 1	0,14793736
Prionailurus bengalensis NC028301 12	Helogale parvula SRR7637809 1	0,14793761
Prionailurus rubiginosus NC028304 2	Paradoxurus jerdoni MH464793 1	0,14793786
Lynx rufus NC014456 3	Galidictis fasciata DM333 1	0,14794226
Prionodon pardicolor NC024569 2	Lynx rufus NC014456 3	0,14794807
Leopardus tigrinus NC028317 1	Diplogale hosei MH464790 1	0,14796215
Urocyon littoralis catalinae KP129018 15	Lycalopex sechurae KT448284 1	0,14796257
Leopardus pardalis T262 1	Diplogale hosei MH464790 1	0,14796972
Pusa caspica NC008431 1	Arctocephalus forsteri KT693377 17	0,14799349
Pusa sibirica NC008432 2	Arctocephalus gazella BK010918 1	0,14799434
Profelis aurata NC028299 1	Chrotogale owstoni T607 1	0,14799514
Prionodon linsang ERR2391707 1	Prionailurus rubiginosus NC028304 2	0,14799981
Mungos mungo/gambianus SRR7704821 1	Civettictis civetta GLC19 1	0,14800146
Phocarctos hookeri NC008418 1	Phoca largha NC008430 1	0,14800207
Puma concolor NC016470 22	Paradoxurus hermaphroditus NLNC 1	0,14800529
Viverricula indica NC025296 2	Mungos mungo/gambianus SRR7704821 1	0,1480055
Paradoxurus hermaphroditus NLNC 1	Otocolobus manul NC028323 1	0,14800583
Phoca vitulina NC001325 1	Arctocephalus pusillus NC008417 1	0,1480072
Prionailurus bengalensis CKM45 20	Paradoxurus hermaphroditus NC039591 1	0,14800827
Viverricula indica NC025296 2	Mungotictis decemlineata NC027828 1	0,14800969
Mungos mungo MMC7 1	Leopardus colocolo NC028314 1	0,14801934
Salanoia concolor D378 1	Leopardus colocolo NC028314 1	0,14802343
Pusa caspica NC008431 1	Phocarctos hookeri NC008418 1	0,14805889
Cystophora cristata NC008427 1	Arctocephalus forsteri KT693377 17	0,14806011
Felis silvestris lybica KP202275 4	Diplogale hosei MH464790 1	0,14806266
Urocyon littoralis catalinae KP129018 15	Chrysocyon brachyurus NC024172 1	0,14807063
Phoca largha NC008430 1	Arctocephalus gazella BK010918 1	0,14807242
Mungos mungo MMC7 1	Leopardus pardalis NC028315 1	0,14807278
Paguma larvata PDDS11 2	Felis silvestris lybica KP202275 4	0,14807342
Monachus monachus NC044972 5	Arctocephalus forsteri KT693377 17	0,14809698
Panthera leo NERO 19	Genetta servalina NC024568 2	0,14810477
Pusa sibirica NC008432 2	Arctocephalus forsteri KT693377 17	0,14812823
Smilodon populator MF871700 1	Paradoxurus hermaphroditus NC039591 1	0,14812848
Chrotogale owstoni T607 1	Catopuma temminckii NC027115 41	0,14813526
Mirounga angustirostris SRR10331586 1	Arctocephalus australis MG023139 1	0,1481355
Phoca groenlandica NC008429 54	Arctocephalus pusillus NC008417 1	0,14813836
Prionailurus planiceps NC028312 6	Atilax paludinosus T606 1	0,14813979
Mungos mungo MMC7 1	Leopardus pardalis T262 1	0,1481404
Paguma larvata PDDS11 2	Felis margarita NC028308 1	0,14814091
Salanoia concolor D378 1	Leopardus tigrinus NC028317 1	0,14814712
Ichneumia albicauda T603 1	Genetta abyssinica MG489822 1	0,14815438
Paradoxurus jerdoni MH464793 1	Leopardus colocolo NC028314 1	0,14815608
Otaria byronia OTAB 1	Monachus monachus NC044972 5	0,14817517
Leopardus guigna NC028321 1	Chrotogale owstoni T607 1	0,14820151
Civettictis civetta GLC19 1	Atilax paludinosus T606 1	0,14820327
Leopardus guigna NC028321 1	Fossa fossana D350 1	0,14820734
Viverra zibetha T609 1	Galidictis fasciata DM333 1	0,14820762
Parahyaena brunnea NC038159 15	Mungos mungo/gambianus SRR7704821 1	0,14821035
Prionailurus bengalensis CKM45 20	Eupleres goudotii D128 1	0,14821155
Viverra zibetha T609 1	Panthera tigris NC010642 35	0,14821494
Prionodon pardicolor NC024569 2	Felis chaus NC028307 1	0,14822179
Prionodon pardicolor NC024569 2	Prionailurus bengalensis CKM45 20	0,14822212
Helogale parvula SRR7637809 1	Civettictis civetta NC033378 1	0,14823948
Phoca fasciata NC008428 1	Arctocephalus forsteri NC004023 28	0,14826266
Pusa caspica NC008431 1	Arctocephalus australis MG023139 1	0,14826275
Phoca groenlandica NC008429 54	Arctocephalus forsteri KT693377 17	0,14826794
Prionodon linsang ERR2391707 1	Leptailurus serval NC028316 1	0,14826943
Viverricula indica KX891745 1	Cynictis penicillata T375 1	0,14826982
Paradoxurus jerdoni MH464793 1	Leopardus wiedii NC028318 1	0,14827219
Viverricula indica NC025296 2	Salanoia concolor D378 1	0,14827968
Leopardus pardalis NC028315 1	Galidictis fasciata DM333 1	0,14828248
Salanoia concolor D378 1	Prionailurus viverrinus NC028305 1	0,1482829
Zalophus californianus NC008416 1	Ommatophoca rossii AY377287etc 1	0,14829901
Phocarctos hookeri NC008418 1	Halichoerus grypus NC001602 2	0,14830185
Prionailurus planiceps NC028312 6	Urva semitorquata MH464789 1	0,1483409
Paguma larvata PDDS11 2	Lynx canadensis NC028313 1	0,14834197
Paradoxurus hermaphroditus NC039591 1	Otocolobus manul NC028323 1	0,14834213
Mungos mungo/gambianus SRR7704821 1	Leopardus pardalis T262 1	0,14834236
Mungos mungo/gambianus SRR7704821 1	Leopardus guigna NC028321 1	0,14834324
Prionailurus bengalensis CKM45 20	Urva semitorquata MH464789 1	0,14834476
Salanoia concolor D378 1	Parahyaena brunnea NC038159 15	0,14834532
Viverricula indica NC025296 2	Panthera uncia KP202269 1	0,14834684
Viverricula indica KX891745 1	Ichneumia albicauda T603 1	0,14834704
Prionodon pardicolor NC024569 2	Lynx pardinus NC028319 161	0,14835225
Panthera onca KP202264 2	Genetta abyssinica MG489822 1	0,14835617
Vulpes zerda KJ603240 1	Urocyon cinereoargenteus NC026723 21	0,14838754
Prionodon pardicolor NC024569 2	Homotherium latidens MF871702 3	0,14839013
Zalophus wollebaeki SRR4431565 1	Ommatophoca rossii AY377287etc 1	0,14839156
Pusa sibirica NC008432 2	Arctocephalus australis MG023139 1	0,14839742
Nandinia binotata NC024567 1	Urva javanica T413 1	0,14839752
Phoca fasciata NC008428 1	Arctocephalus townsendi NC008420 1	0,14840165
Viverricula indica KX891745 1	Urva semitorquata MH464789 1	0,14840457
Genetta servalina NC024568 2	Bdeogale nigripes GLC15 1	0,14840497
Phoca vitulina NC001325 1	Neophoca cinerea NC008419 1	0,14840771
Urva semitorquata MH464789 1	Caracal caracal NC028306 1	0,14840914
Puma yagouaroundi NC028311 1	Paradoxurus hermaphroditus NLNC 1	0,14840959
Parahyaena brunnea NC038159 15	Galidia elegans D146 1	0,14841135
Parahyaena brunnea NC038159 15	Urva semitorquata MH464789 1	0,14841184
Prionailurus bengalensis CKM45 20	Helogale parvula SRR7637809 1	0,14841224
Leopardus tigrinus NC028317 1	Genetta genetta T297 1	0,14841239
Prionailurus bengalensis CKM45 20	Atilax paludinosus T606 1	0,14841288

Ursus americanus JX196366 3	Catopuma badia NC028300 1	0,18522042
Meles anakuma NC009677 1	Arctocephalus gazella BK010918 1	0,18522124
Mustela frenata NC020640 1	Cynogale bennetti KY117544 1	0,18522148
Smilodon populator MF871700 1	Arctocephalus pusillus NC008417 1	0,1852244
Ursus americanus JX196366 3	Caracal caracal NC028306 1	0,18522464
Mustela putorius NC020638 4	Arctocephalus australis MG023139 1	0,18522477
Phoca fasciata NC008428 1	Tapirus terrestris T358	0,18522488
Fossa fossana D350 1	Ailurus fulgens styani NC009691 1	0,1852278
Vulpes zerda KJ603240 1	Fossa fossana D350 1	0,18523011
Mungos mungo/gambianus SRR77048	Martes pennanti NC020664 16	0,18523158
Vulpes corsac NC023958 1	Viverra tangalunga MH464792 1	0,18523252
Profelis aurata NC028299 1	Enhydra lutris NC009692 1	0,18523352
Lutra lutra NC011358 9	Caracal caracal NC028306 1	0,18523364
Lutra lutra NC011358 9	Felis chaus NC028307 1	0,185234
Prionailurus planiceps KY682741 4	Ailurus fulgens NC011124 1	0,1852369
Mustela kathiah NC023210 1	Hyaena hyaena NC020669 1	0,18523695
Prionodon pardicolor NC024569 2	Martes americana NC020642 1	0,18523782
Prionodon pardicolor NC024569 2	Martes zibellina NC011579 39	0,18523787
Ursus maritimus NC003428 31	Fossa fossana D350 1	0,18523929
Ursus arctos pruinosus MG066703 3	Enhydra lutris NC009692 1	0,18523993
Mustela nigripes NC024942 1	Ichneumia albicauda T603 1	0,18524049
Vulpes vulpes NC008434 5	Pardofelis marmorata NLN3 2	0,18524063
Vulpes ferrilata NC027935 1	Lynx pardinus NC028319 161	0,18524092
Vulpes corsac NC023958 1	Prionailurus planiceps KY682741 4	0,18524104
Prionailurus rubiginosus NC028304 2	Arctocephalus gazella BK010918 1	0,18524134
Canis aureus KT448274 1	Bdeogale nigripes GLC15 1	0,18524137
Leopardus wiedii NC028318 1	Arctocephalus townsendi NC008420 1	0,18524191
Profelis aurata NC028299 1	Martes americana NC020642 1	0,18524204
Ursus thibetanus mupinensis NC00875	Mungotictis decemlineata NC027828 1	0,18524218
Lynx rufus NC014456 3	Arctocephalus gazella BK010918 1	0,1852423
Tremarctos ornatus NC009969 2	Paguma larvata PDDS11 2	0,18524361
Vulpes ferrilata NC027935 1	Mirounga angustirostris SRR10331586	0,18524383
Viverricula indica KX891751 1	Martes martes T302 3	0,18524392
Prionailurus bengalensis CKM45 20	Arctocephalus forsteri KT693377 17	0,18524437
Pardofelis marmorata NLN3 2	Arctocephalus pusillus NC008417 1	0,18524563
Speothos venaticus C48 2	Mustela erminea T305 2	0,18524568
Neophoca cinerea NC008419 1	Leopardus geoffroyi NC028320 1	0,18524583
Puma concolor NC016470 22	Arctocephalus pusillus NC008417 1	0,18524677
Lynx canadensis NC028313 1	Arctotherium sp NC030174 1	0,18524726
Arctocephalus gazella BK010918 1	Ailuropoda melanoleuca NC009492 5	0,18524763
Prionailurus viverrinus NC028305 1	Arctocephalus pusillus NC008417 1	0,18524899
Mephitis mephitis NC020648 1	Martes melampus NC009678 1	0,18524963
Otocyon megalotis SAF1 2	Lynx pardinus NC028319 161	0,18525015
Ursus arctos pruinosus MG066703 3	Neophoca cinerea NC008419 1	0,18525049
Ursus arctos pruinosus MG066703 3	Mustela nivalis T306 5	0,18525094
Speothos venaticus C48 2	Leopardus pardalis NC028315 1	0,18525212
Martes foina NC020643 1	Cryptoprocta ferox CFC13 1	0,1852522
Taxidea taxus NC020646 1	Panthera tigris amoyensis NC014770 2	0,18525349
Ursus maritimus GU573488 Svalbard	Martes foina NC020643 1	0,1852539
Martes flavigula NC012141 3	Canis mesomelas KT448280 1	0,18525393
Ursus arctos AP012576 6	Arctocephalus townsendi NC008420 1	0,18525405
Ursus thibetanus mupinensis NC00875	Paradoxurus jerdoni MH464793 1	0,18525416
Speothos venaticus C48 2	Prionailurus rubiginosus NC028304 2	0,1852548
Prionailurus bengalensis CKM45 20	Otaria byronia OTAB 1	0,18525498
Lutra lutra LC050126 1	Urva javanica/auropunctata NC006835	0,18525542
Zalophus wollebaeki SRR4431565 1	Ursus arctos pruinosus MG066703 3	0,18525644
Zalophus wollebaeki SRR4431565 1	Felis nigripes NC028309 1	0,18525654
Chrysocyon brachyurus NC024172 1	Arctotherium sp NC030174 1	0,1852566
Melursus ursinus NC009970 2	Ailurus fulgens NC011124 1	0,18525767
Melursus ursinus NC009970 2	Ailurus fulgens styani NC009691 1	0,18525782
Mustela itatsi NC034330 19	Ailuropoda melanoleuca NC009492 5	0,18525881
Ursus thibetanus formosanus NC00933	Lynx lynx NC027083 4	0,18526196
Ursus thibetanus laniger MH281753 2	Lynx canadensis NC028313 1	0,18526278
Galerella sanguinea T378 1	Canis lupus familiaris NC002008 1231	0,1852631
Ursus spelaeus NC011112 8	Lynx rufus NC014456 3	0,18526451
Ursus thibetanus laniger MH281753 2	Leptailurus serval NC028316 1	0,18526734
Ursus thibetanus formosanus NC00933	Prionailurus bengalensis CKM45 20	0,18526742
Zalophus californianus NC008416 1	Pardofelis marmorata NLN3 2	0,18527138
Zalophus californianus NC008416 1	Ailuropoda melanoleuca NC009492 5	0,18527557
Ursus spelaeus EU327344 13	Leopardus colocolo NC028314 1	0,18527904
Ichneumia albicauda T603 1	Gulo gulo NC009685 3	0,18528007
Leopardus pardalis T262 1	Arctocephalus forsteri NC004023 28	0,18528025
Leopardus colocolo NC028314 1	Galictis vittata T412 1	0,18528137
Ursus americanus JX196366 3	Leopardus pardalis T262 1	0,18528265
Viverra tangalunga MH464792 1	Cuon alpinus NC013445 3	0,18528365
Martes flavigula NC012141 3	Felis chaus NC028307 1	0,1852839
Urva javanica/auropunctata NC006835	Canis aureus KT448274 1	0,18528485
Otocolobus manul NC028323 1	Cuon alpinus NC013445 3	0,18528651
Ursus thibetanus mupinensis NC00875	Spilogale putorius NC010497 1	0,18528754
Martes americana NC020642 1	Urva semitorquata MH464789 1	0,18529236
Mungos mungo MMC7 1	Martes zibellina NC011579 39	0,18529323
Otocyon megalotis SAF1 2	Martes flavigula NC012141 3	0,18529338
Lontra canadensis SRR10409165 1	Arctocephalus townsendi NC008420 1	0,18529354
Lutra lutra NC011358 9	Galidia elegans D146 1	0,18529378
Ursus thibetanus thibetanus NC01111	Spilogale putorius NC010497 1	0,18529411
Mustela putorius NC020638 4	Cynictis penicillata T375 1	0,18529478
Prionailurus bengalensis CKM45 20	Cuon alpinus NC013445 3	0,18529485
Ursus arctos isabellinus 18B5 2	Smilodon populator MF871700 1	0,18529692
Leopardus wiedii NC028318 1	Conepatus chinga NC042596 1	0,18529753
Procyon lotor AB462046 3	Phocarctos hookeri NC008418 1	0,18529861
Procyon lotor AB462046 3	Mungos mungo MMC7 1	0,18529882
Vulpes lagopus NC026529 3	Galidictis fasciata DM333 1	0,18529904
Paguma larvata PDDS11 2	Lutra sumatrana NC035810 1	0,18529941
Mungos mungo MMC7 1	Aonyx cinerea NC035814 2	0,1853005
Mustela nivalis T306 5	Arctocephalus forsteri KT693377 17	0,18530256
Hyaena hyaena NC020669 1	Enhydra lutris NC009692 1	0,18530287
Mustela nivalis T306 5	Urva semitorquata MH464789 1	0,18530376
Taxidea taxus NC020646 1	Canis anthus NC027956 2	0,18530481
Parahyaena brunnea NC038159 15	Lutra lutra NC011358 9	0,18530566
Neovison vison NC020641 3	Bdeogale nigripes GLC15 1	0,18530579
Ursus arctos EU497665 29	Arctocephalus forsteri KT693377 17	0,18530607
Mungos mungo MMC7 1	Arctodus simus NC011116 1	0,18530631
Vulpes corsac NC023958 1	Lynx pardinus NC028319 161	0,18530799

Puma yagouaroundi NC028311 1	Mungotictis decemlineata NC027828 1	0,14841463
Neofelis nebulosa NC008450 3	Nandinia binotata NC024567 1	0,1484372
Leopardus wiedii NC028318 1	Diplogale hosei MH464790 1	0,14844979
Monachus schauinslandi NC008421 1	Arctocephalus townsendi NC008420 1	0,1484731
Phocarctos hookeri NC008418 1	Phoca vitulina NC001325 1	0,14847364
Paradoxurus hermaphroditus NLNC 1	Leopardus tigrinus NC028317 1	0,14847466
Viverricula indica NC025296 2	Cynictis penicillata T375 1	0,14847565
Prionailurus viverrinus NC028305 1	Atilax paludinosus T606 1	0,14847901
Prionailurus viverrinus NC028305 1	Crossarchus platycephalus C7R66 1	0,14847903
Salanoia concolor D378 1	Panthera pardus japonensis KJ866876 8	0,14848058
Leopardus wiedii NC028318 1	Galidia elegans D146 1	0,14848323
Prionodon pardicolor NC024569 2	Felis silvestris lybica KP202275 4	0,14848678
Prionodon pardicolor NC024569 2	Felis nigripes NC028309 1	0,14848724
Prionodon pardicolor NC024569 2	Felis margarita NC028308 1	0,14848728
Prionodon pardicolor NC024569 2	Otocolobus manul NC028323 1	0,14848892
Monachus monachus NC044972 5	Arctocephalus forsteri NC004023 28	0,14849417
Eupleres goudotii D128 1	Civettictis civetta NC033378 1	0,14850047
Prionailurus rubiginosus NC028304 2	Diplogale hosei MH464790 1	0,14851657
Felis margarita NC028308 1	Diplogale hosei MH464790 1	0,14852847
Cystophora cristata NC008427 1	Arctocephalus pusillus NC008417 1	0,14853539
Viverricula indica KX891751 1	Mungos mungo/gambianus SRR7704821 1	0,14853945
Prionailurus planiceps KY682741 4	Crossarchus platycephalus C7R66 1	0,14854282
Panthera onca KP202264 2	Fossa fossana D350 1	0,14854328
Leopardus pardalis T262 1	Urva semitorquata MH464789 1	0,14854488
Crossarchus platycephalus C7R66 1	Crocuta crocuta NC020670 3	0,14854878
Homotherium latidens MF871702 3	Cynogale bennetti KY117544 1	0,1485493
Salanoia concolor D378 1	Leopardus geoffroyi NC028320 1	0,14854977
Prionailurus bengalensis NC028301 12	Galidictis fasciata DM333 1	0,14854978
Leopardus guigna NC028321 1	Genetta genetta T297 1	0,14855087
Lynx rufus NC014456 3	Cryptoprocta ferox CFC13 1	0,14855251
Leopardus colocolo NC028314 1	Urva semitorquata MH464789 1	0,14855739
Prionodon pardicolor NC024569 2	Catopuma temminckii NC027115 41	0,14855897
Pusa sibirica NC008432 2	Arctocephalus townsendi NC008420 1	0,14859886
Cryptoprocta ferox CFC13 1	Civettictis civetta GLC19 1	0,14860952
Viverricula indica KX891745 1	Mungotictis decemlineata NC027828 1	0,14861071
Lynx lynx NC027083 4	Eupleres goudotii D128 1	0,14861149
Prionailurus planiceps NC028312 6	Eupleres goudotii D128 1	0,14861235
Mungotictis decemlineata NC027828 1	Catopuma temminckii NC027115 41	0,14861347
Leopardus jacobita NC028322 1	Crossarchus platycephalus C7R66 1	0,14861354
Pardofelis marmorata NLN3 2	Genetta genetta T297 1	0,14861456
Leopardus tigrinus NC028317 1	Galidictis fasciata DM333 1	0,1486187
Leopardus pardalis T262 1	Hyaena hyaena NC020669 1	0,14862579
Mungotictis decemlineata NC027828 1	Acinonyx jubatus NC005212 3	0,14863147
Xenogale naso C07XAR110 1	Civettictis civetta NC033378 1	0,14863819
Urva semitorquata MH464789 1	Civettictis civetta NC033378 1	0,14863884
Felis nigripes NC028309 1	Chrotogale owstoni T607 1	0,14866954
Phocarctos hookeri NC008418 1	Phoca groenlandica NC008429 54	0,14867151
Mungos mungo MMC7 1	Genetta genetta T297 1	0,14867552
Panthera onca KP202264 2	Genetta servalina NC024568 2	0,14868067
Panthera pardus japonensis KJ866876 8	Genetta servalina NC024568 2	0,14868076
Parahyaena brunnea NC038159 15	Helogale parvula SRR7637809 1	0,14868136
Smilodon populator MF871700 1	Helogale parvula SRR7637809 1	0,14868237
Leopardus geoffroyi NC028320 1	Galidictis fasciata DM333 1	0,14868379
Prionodon pardicolor NC024569 2	Caracal caracal NC028306 1	0,14868988
Leopardus guigna NC028321 1	Arctictis binturong T605 2	0,14869982
Vulpes ferrilata NC027935 1	Speothos venaticus C48 2	0,14873104
Paradoxurus jerdoni MH464793 1	Nandinia binotata NC024567 1	0,14874034
Phoca largha NC008430 1	Arctocephalus townsendi NC008420 1	0,1487446
Prionailurus planiceps KY682741 4	Atilax paludinosus T606 1	0,14874579
Lynx pardinus NC028319 161	Eupleres goudotii D128 1	0,14874631
Caracal caracal NC028306 1	Atilax paludinosus T606 1	0,14874651
Leopardus wiedii NC028318 1	Helogale parvula SRR7637809 1	0,14874689
Arctodus simus NC011116 1	Ailuropoda melanoleuca NC009492 5	0,14875087
Salanoia concolor D378 1	Prionailurus planiceps KY682741 4	0,14875087
Viverricula indica NC025296 2	Galidictis fasciata DM333 1	0,14875166
Panthera onca NC022842 1	Fossa fossana D350 1	0,14875378
Leopardus jacobita NC028322 1	Cryptoprocta ferox CFC13 1	0,14875835
Leopardus colocolo NC028314 1	Galidictis fasciata DM333 1	0,14876422
Mungos mungo/gambianus SRR7704821 1	Civettictis civetta NC033378 1	0,14877383
Mungos mungo MMC7 1	Civettictis civetta NC033378 1	0,14877446
Panthera pardus NC010641 5	Genetta servalina NC024568 2	0,1487793
Vulpes zerda KJ603240 1	Urocyon littoralis catalinae KP129018 15	0,14879149
Hemigalus derbyanus MH464791 1	Atilax paludinosus T606 1	0,14880347
Prionodon linsang ERR2391707 1	Prionailurus planiceps KY682741 4	0,14880773
Prionodon linsang ERR2391707 1	Genetta servalina NC024568 2	0,14880858
Xenogale naso C07XAR110 1	Civettictis civetta GLC19 1	0,14880905
Urva semitorquata MH464789 1	Catopuma badia NC028300 1	0,14881118
Paradoxurus hermaphroditus NLNC 1	Leopardus jacobita NC028322 1	0,14881144
Prionailurus planiceps KY682741 4	Urva semitorquata MH464789 1	0,1488122
Mungos mungo/gambianus SRR7704821 1	Leptailurus serval NC028316 1	0,14881279
Viverricula indica NC025296 2	Urva semitorquata MH464789 1	0,1488134
Leopardus pardalis NC028315 1	Urva semitorquata MH464789 1	0,14881402
Parahyaena brunnea NC038159 15	Eupleres goudotii D128 1	0,14881583
Cryptoprocta ferox CFC13 1	Catopuma badia NC028300 1	0,14881764
Hyaena hyaena NC020669 1	Galidictis fasciata DM333 1	0,14881811
Salanoia concolor D378 1	Prionailurus planiceps NC028312 6	0,14881834
Mungotictis decemlineata NC027828 1	Leopardus geoffroyi NC028320 1	0,14881867
Prionailurus bengalensis CKM45 20	Galidictis fasciata DM333 1	0,1488221
Smilodon populator MF871700 1	Urva semitorquata MH464789 1	0,14883052
Viverricula indica KX891745 1	Panthera leo NERO 19	0,14884587
Smilodon populator MF871700 1	Arctictis binturong T605 2	0,14887257
Paradoxurus hermaphroditus NLNC 1	Cynictis penicillata T375 1	0,14887524
Paradoxurus hermaphroditus NC039591 1	Leopardus pardalis NC028315 1	0,14887725
Monachus schauinslandi NC008421 1	Arctocephalus forsteri NC004023 28	0,14887802
Puma yagouaroundi NC028311 1	Crossarchus platycephalus C7R66 1	0,14888021
Puma yagouaroundi NC028311 1	Paradoxurus hermaphroditus NC039591 1	0,14888062
Leptailurus serval NC028316 1	Atilax paludinosus T606 1	0,14888133
Viverricula indica NC025296 2	Panthera leo spelaea KX258452 2	0,14888206
Paradoxurus jerdoni MH464793 1	Acinonyx jubatus NC005212 3	0,1488919
Urva semitorquata MH464789 1	Hemigalus derbyanus MH464791 1	0,14892706
Cystophora cristata NC008427 1	Arctocephalus gazella BK010918 1	0,14893594
Phoca fasciata NC008428 1	Neophoca cinerea NC008419 1	0,14894199
Genetta servalina NC024568 2	Atilax paludinosus T606 1	0,1489436

Catopuma badia NC028300 1	Arctocephalus gazella BK010918 1	0,18530846
Vulpes zerda KJ603240 1	Lynx lynx NC027083 4	0,18530889
Vulpes lagopus NC026529 3	Puma yagouaroundi NC028311 1	0,18530908
Nyctereutes procyonoides NC013700 3	Arctodus simus NC011116 1	0,18530931
Lynx pardinus NC028319 161	Arctocephalus gazella BK010918 1	0,18530947
Neophoca cinerea NC008419 1	Lynx canadensis NC028313 1	0,18530972
Leopardus guigna NC028321 1	Arctocephalus forsteri KT693377 17	0,18531032
Pardofelis marmorata NLN3 2	Canis latrans NC008093 7	0,1853106
Martes foina NC020643 1	Galidia elegans D146 1	0,18531083
Ursus arctos GU573491 207	Lutra sumatrana NC035810 1	0,18531086
Prionailurus viverrinus NC028305 1	Arctocephalus gazella BK010918 1	0,18531111
Lycaon pictus NC028427 2	Ichneumia albicauda T603 1	0,18531115
Proteles cristata T393 6	Lutra lutra NC011358 9	0,18531159
Phocarctos hookeri NC008418 1	Leopardus pardalis T262 1	0,18531197
Vulpes zerda KJ603240 1	Monachus schauinslandi NC008421 1	0,18531321
Viverricula indica NC025296 2	Aonyx cinerea NC035814 2	0,18531379
Viverricula indica KX891751 1	Ursus arctos GU573491 207	0,18531428
Martes pennanti NC020664 16	Leptailurus serval NC028316 1	0,18531573
Ursus maritimus NC003428 31	Mustela altaica NC021751 1	0,18531598
Martes flavigula NC012141 3	Chrotogale owstoni T607 1	0,18531707
Ursus arctos AP012576 6	Melogale moschata V0735A 1	0,18531765
Mustela eversmannii NC028013 1	Arctictis binturong T605 2	0,18532009
Lycaon pictus NC028427 2	Arctotherium sp NC030174 1	0,18532063
Crossarchus platycephalus C7R66 1	Chrysocyon brachyurus NC024172 1	0,18532185
Ictonyx striatus T299 1	Catopuma temminckii NC027115 41	0,18532425
Viverra tangalunga MH464792 1	Helarctos malayanus NC009968 2	0,18532631
Paradoxurus hermaphroditus NLNC 1	Martes flavigula NC012141 3	0,18532646
Vulpes lagopus NC026529 3	Leopardus colocolo NC028314 1	0,1853267
Martes zibellina NC011579 39	Urva javanica/auropunctata NC006835	0,18532844
Spilogale putorius NC010497 1	Cynogale bennetti KY117544 1	0,18533127
Lynx pardinus NC028319 161	Helarctos malayanus NC009968 2	0,1853313
Martes pennanti NC020664 16	Canis lupus familiaris NC002008 1231	0,18534121
Paradoxurus hermaphroditus NLNC 1	Ommatophoca rossii AY377287etc 1	0,18534505
Martes flavigula NC012141 3	Genetta genetta T297 1	0,1853468
Panthera onca KP202264 2	Martes flavigula NC012141 3	0,18535382
Pusa caspica NC008431 1	Tapirus terrestris T358	0,18535432
Ursus maritimus NC003428 31	Civettictis civetta NC033378 1	0,1853545
Nyctereutes procyonoides NC013700 3	Genetta abyssinica MG489822 1	0,18535496
Mustela itatsi NC034330 19	Canis mesomelas KT448280 1	0,18535551
Paguma larvata PDDS11 2	Lutra lutra NC011358 9	0,18535751
Lutra sumatrana NC035810 1	Xenogale naso C07XAR110 1	0,18535842
Enhydra lutris NC009692 1	Canis latrans NC008093 7	0,18535872
Ursus arctos EU497665 29	Gulo gulo NC009685 3	0,18535997
Mustela itatsi NC034330 19	Mungos mungo/gambianus SRR77048	0,18536077
Suricata suricatta SSM10 1	Mustela kathiah NC023210 1	0,18536101
Suricata suricatta SSM10 1	Mustela frenata NC020640 1	0,18536173
Mustela nigripes NC024942 1	Urva brachyura KY117547 1	0,18536176
Vulpes corsac NC023958 1	Mustela nigripes NC024942 1	0,18536636
Viverricula indica KX891745 1	Arctocephalus forsteri KT693377 17	0,18536833
Martes martes T302 3	Galidia elegans D146 1	0,18537052
Profelis aurata NC028299 1	Callorhinus ursinus NC008415 1	0,18537137
Melogale moschata KP726273 1	Hyaena hyaena NC020669 1	0,18537217
Ursus arctos EU497665 29	Arctocephalus townsendi NC008420 1	0,18537274
Lycalopex sechurae KT448284 1	Civettictis civetta NC033378 1	0,18537325
Ursus arctos EU497665 29	Enhydra lutris NC009692 1	0,18537438
Martes flavigula NC012141 3	Urva javanica/auropunctata NC006835	0,18537439
Mustela eversmannii NC028013 1	Felis chaus NC028307 1	0,1853752
Vulpes corsac NC023958 1	Leopardus pardalis NC028315 1	0,18537527
Vulpes corsac NC023958 1	Prionailurus viverrinus NC028305 1	0,18537536
Lynx rufus NC014456 3	Arctocephalus forsteri KT693377 17	0,1853756
Vulpes corsac NC023958 1	Puma concolor NC016470 22	0,18537576
Ursus arctos GU573491 207	Phocarctos hookeri NC008418 1	0,18537706
Tremarctos ornatus NC009969 2	Profelis aurata NC028299 1	0,18537717
Paradoxurus jerdoni MH464793 1	Nyctereutes procyonoides NC013700 3	0,18537785
Lontra canadensis SRR10409165 1	Leopardus wiedii NC028318 1	0,18537793
Taxidea taxus NC020646 1	Panthera uncia KP202269 1	0,18537834
Ursus maritimus NC003428 31	Meles anakuma NC009677 1	0,18537849
Ursus arctos isabellinus 18B5 2	Meles anakuma NC009677 1	0,18537851
Prionailurus bengalensis CKM45 20	Melogale moschata V0735A 1	0,18537937
Lynx pardinus NC028319 161	Lutrogale perspicillata NC035811 1	0,18538064
Felis catus NC001700 2	Arctocephalus pusillus NC008417 1	0,18538092
Ursus arctos pruinosus MG066703 3	Mustela itatsi NC034330 19	0,18538103
Puma concolor NC016470 22	Procyon lotor AB462049 4	0,18538134
Prionailurus planiceps NC028312 6	Lutrogale perspicillata NC035811 1	0,18538208
Leopardus jacobita NC028322 1	Arctocephalus pusillus NC008417 1	0,18538305
Ursus thibetanus laniger MH281753 2	Crossarchus platycephalus C7R66 1	0,1853849
Melursus ursinus NC009970 2	Martes pennanti NC020664 16	0,18538496
Vulpes vulpes NC008434 5	Arctictis binturong T605 2	0,18538509
Panthera onca NC022842 1	Mustela itatsi NC034330 19	0,18538576
Ursus maritimus NC003428 31	Martes foina NC020643 1	0,18538858
Ursus spelaeus NC011112 8	Chrotogale owstoni T607 1	0,1853888
Viverricula indica NC025296 2	Otaria byronia OTAB 1	0,18538882
Lutra lutra NC011358 9	Urva javanica/auropunctata NC006835	0,18538888
Zalophus wollebaeki SRR4431565 1	Catopuma temminckii NC027115 41	0,18539208
Ursus thibetanus thibetanus NC01111	Procyon lotor AB462049 4	0,18539225
Melursus ursinus NC009970 2	Genetta servalina NC024568 2	0,18539709
Paguma larvata PDDS11 2	Ailuropoda melanoleuca NC009492 5	0,18539762
Lynx canadensis NC028313 1	Helarctos malayanus NC009968 2	0,1853977
Ursus thibetanus mupinensis NC00875	Ictonyx striatus T299 1	0,18539797
Ursus spelaeus EU327344 13	Catopuma badia NC028300 1	0,18539941
Martes martes T302 3	Urva javanica/auropunctata NC006835	0,1854004
Civettictis civetta NC033378 1	Tapirus terrestris T358	0,18540122
Leopardus geoffroyi NC028320 1	Galictis vittata T412 1	0,18540227
Puma yagouaroundi NC028311 1	Melursus ursinus NC009970 2	0,18540315
Ursus spelaeus NC011112 8	Prionailurus bengalensis CKM45 20	0,18540464
Salanoia concolor D378 1	Gulo gulo NC009685 3	0,18540676
Meles anakuma NC009677 1	Canis lupus chanco NC010340 4	0,18540794
Enhydra lutris NC009692 1	Chrotogale owstoni T607 1	0,18541354
Ursus americanus JX196366 3	Paguma larvata PDDS11 2	0,18541729
Eumetopias jubatus NC004030 10	Civettictis civetta GLC19 1	0,18541893
Hemigalus derbyanus MH464791 1	Arctocephalus forsteri KT693377 17	0,18541944
Mustela putorius NC020638 4	Canis mesomelas KT448280 1	0,18542051
Prionailurus planiceps NC028312 6	Canis mesomelas KT448280 1	0,18542257

Lynx rufus NC014456 3	Urva semitorquata MH464789 1	0,14894602
Viverricula indica NC025296 2	Urva javanica T413 1	0,14894709
Genetta genetta T297 1	Galidictis fasciata DM333 1	0,14894864
Prionailurus planiceps KY682741 4	Eupleres goudotii D128 1	0,14894892
Prionailurus viverrinus NC028305 1	Helogale parvula SRR7637809 1	0,14894971
Paradoxurus jerdoni MH464793 1	Catopuma temminckii NC027115 41	0,14895648
Felis nigripes NC028309 1	Arctictis binturong T605 2	0,14896227
Neophoca cinerea NC008419 1	Cystophora cristata NC008427 1	0,14900236
Viverricula indica KX891745 1	Urva javanica T413 1	0,14901073
Lynx canadensis NC028313 1	Crossarchus platycephalus C7R66 1	0,14901286
Fossa fossana D350 1	Felis margarita NC028308 1	0,14901438
Phoca largha NC008430 1	Arctocephalus forsteri KT693377 17	0,1490145
Prionailurus planiceps KY682741 4	Paradoxurus hermaphroditus NC039591 1	0,14901486
Mungotictis decemlineata NC027828 1	Felis silvestris lybica KP202275 4	0,1490193
Viverricula indica NC025296 2	Galerella sanguinea T378 1	0,14902237
Leopardus guigna NC028321 1	Cryptoprocta ferox CFC13 1	0,14902397
Viverricula indica NC025296 2	Prionodon pardicolor NC024569 2	0,14902404
Leopardus tigrinus NC028317 1	Cryptoprocta ferox CFC13 1	0,14902794
Mungos mungo/gambianus SRR7704821 1	Leopardus colocolo NC028314 1	0,14903234
Panthera tigris amoyensis NC014770 2	Civettictis civetta NC033378 1	0,14906187
Leopardus jacobita NC028322 1	Diplogale hosei MH464790 1	0,14907092
Mirounga angustirostris SRR10331586 1	Arctocephalus gazella BK010918 1	0,14907874
Felis silvestris lybica KP202275 4	Crossarchus platycephalus C7R66 1	0,14908116
Lynx rufus NC014456 3	Atilax paludinosus T606 1	0,14908139
Fossa fossana D350 1	Felis catus NC001700 2	0,14908153
Phoca largha NC008430 1	Arctocephalus australis MG023139 1	0,14908178
Prionailurus rubiginosus NC028304 2	Mungos mungo/gambianus SRR7704821 1	0,14908192
Nandinia binotata NC024567 1	Ichneumia albicauda T603 1	0,14908203
Viverricula indica NC025296 2	Atilax paludinosus T606 1	0,14908262
Paradoxurus jerdoni MH464793 1	Otocolobus manul NC028323 1	0,14908318
Otaria byronia OTAB 1	Mirounga angustirostris SRR10331586 1	0,14908673
Paradoxurus hermaphroditus NLNC 1	Catopuma temminckii NC027115 41	0,14909084
Puma yagouaroundi NC028311 1	Diplogale hosei MH464790 1	0,1491383
Urva semitorquata MH464789 1	Felis margarita NC028308 1	0,14914887
Phoca groenlandica NC008429 54	Otaria byronia OTAB 1	0,14914888
Prionailurus planiceps KY682741 4	Helogale parvula SRR7637809 1	0,14914912
Prionailurus rubiginosus NC028304 2	Bdeogale nigripes GLC15 1	0,1491496
Phoca vitulina NC001325 1	Arctocephalus gazella BK010918 1	0,14914994
Viverricula indica KX891745 1	Panthera onca KP202264 2	0,14915152
Galidictis fasciata DM333 1	Catopuma badia NC028300 1	0,149152
Paradoxurus hermaphroditus NLNC 1	Leopardus geoffroyi NC028320 1	0,14915705
Mungotictis decemlineata NC027828 1	Leopardus colocolo NC028314 1	0,14917187
Panthera pardus NC010641 5	Genetta abyssinica MG489822 1	0,14921035
Phoca groenlandica NC008429 54	Arctocephalus australis MG023139 1	0,14921039
Mirounga angustirostris SRR10331586 1	Arctocephalus forsteri NC004023 28	0,14921114
Viverra zibetha T609 1	Urva javanica T413 1	0,14921287
Viverricula indica NC025296 2	Eupleres goudotii D128 1	0,14921464
Nandinia binotata NC024567 1	Cryptoprocta ferox CFC13 1	0,14921466
Phoca fasciata NC008428 1	Otaria byronia OTAB 1	0,14921553
Pusa hispida NC 008433 1	Arctocephalus forsteri NC004023 28	0,14921556
Prionailurus bengalensis NC028301 12	Mungos mungo/gambianus SRR7704821 1	0,14921664
Prionailurus planiceps NC028312 6	Paradoxurus hermaphroditus NC039591 1	0,1492168
Viverra zibetha T609 1	Salanoia concolor D378 1	0,14921726
Paguma larvata PDDS11 2	Felis nigripes NC028309 1	0,14921828
Mungotictis decemlineata NC027828 1	Leopardus guigna NC028321 1	0,14922215
Cryptoprocta ferox CFC13 1	Civettictis civetta NC033378 1	0,14924508
Halichoerus grypus NC001602 2	Arctocephalus townsendi NC008420 1	0,14924565
Smilodon populator MF871700 1	Paradoxurus jerdoni MH464793 1	0,14926688
Eupleres goudotii D128 1	Civettictis civetta GLC19 1	0,14927804
Hyaena hyaena NC020669 1	Urva semitorquata MH464789 1	0,14928523
Viverra zibetha T609 1	Panthera uncia NC010638 1	0,14928639
Leopardus guigna NC028321 1	Galidictis fasciata DM333 1	0,14928941
Prionodon pardicolor NC024569 2	Lynx lynx NC027083 4	0,14929399
Panthera onca NC022842 1	Genetta servalina NC024568 2	0,14929442
Viverra zibetha T609 1	Urva javanica/auropunctata NC006835 1	0,14931455
Phoca vitulina NC001325 1	Arctocephalus forsteri KT693377 17	0,1493514
Salanoia concolor D378 1	Genetta genetta T297 1	0,14935216
Leopardus pardalis T262 1	Ichneumia albicauda T603 1	0,14935358
Prionailurus bengalensis CKM45 20	Mungos mungo MMC7 1	0,14935392
Leopardus wiedii NC028318 1	Hyaena hyaena NC020669 1	0,14936571
Viverricula indica KX891751 1	Urva javanica/auropunctata NC006835 1	0,14938071
Halichoerus grypus NC001602 2	Arctocephalus forsteri KT693377 17	0,14938101
Salanoia concolor D378 1	Panthera pardus NC010641 5	0,14939038
Felis catus NC001700 2	Diplogale hosei MH464790 1	0,14940363
Viverricula indica KX891751 1	Bdeogale nigripes GLC15 1	0,14941439
Prionodon linsang ERR2391707 1	Prionailurus bengalensis CKM45 20	0,14941725
Otocolobus manul NC028323 1	Fossa fossana D350 1	0,14941852
Prionailurus planiceps KY682741 4	Mungotictis decemlineata NC027828 1	0,14942385
Prionodon pardicolor NC024569 2	Prionailurus viverrinus NC028305 1	0,14943074
Leopardus tigrinus NC028317 1	Arctictis binturong T605 2	0,14943239
Urva javanica T413 1	Genetta abyssinica MG489822 1	0,14943711
Nandinia binotata NC024567 1	Urva javanica/auropunctata NC006835 1	0,14943849
Panthera pardus japonensis KJ866876 8	Genetta abyssinica MG489822 1	0,14945482
Meles anakuma NC009677 1	Lontra canadensis SRR10409165 1	0,1494717
Cystophora cristata NC008427 1	Arctocephalus forsteri NC004023 28	0,14947371
Viverricula indica KX891751 1	Crossarchus platycephalus C7R66 1	0,14948206
Urva semitorquata MH464789 1	Genetta genetta T297 1	0,14948346
Paradoxurus jerdoni MH464793 1	Leopardus pardalis T262 1	0,14948402
Puma concolor NC016470 22	Fossa fossana D350 1	0,14948532
Catopuma badia NC028300 1	Atilax paludinosus T606 1	0,14948544
Puma concolor NC016470 22	Mungos mungo/gambianus SRR7704821 1	0,14948551
Prionailurus planiceps NC028312 6	Crossarchus platycephalus C7R66 1	0,1494856
Prionailurus planiceps NC028312 6	Paradoxurus jerdoni MH464793 1	0,14948647
Paradoxurus hermaphroditus NLNC 1	Felis nigripes NC028309 1	0,1494866
Paradoxurus jerdoni MH464793 1	Felis margarita NC028308 1	0,14948663
Panthera leo NERO 19	Hemigalus derbyanus MH464791 1	0,14948732
Salanoia concolor D378 1	Felis catus NC001700 2	0,149491
Xenogale naso C07XAR110 1	Hemigalus derbyanus MH464791 1	0,14951533
Taxidea taxus NC020646 1	Galictis vittata T412 1	0,1495166
Phoca groenlandica NC008429 54	Neophoca cinerea NC008419 1	0,14954811
Viverricula indica KX891745 1	Atilax paludinosus T606 1	0,1495491
Viverra zibetha T609 1	Cynictis penicillata T375 1	0,14954929
Paradoxurus hermaphroditus NC039591 1	Leopardus pardalis T262 1	0,14955077

Prionailurus bengalensis NC028301 12	Tapirus terrestris T358	0,18542467
Meles leucurus NC039173 4	Xenogale naso C07XAR110 1	0,18542627
Civettictis civetta GLC19 1	Tapirus terrestris T358	0,1854272
Melogale moschata KP726273 1	Xenogale naso C07XAR110 1	0,18542726
Martes zibellina NC011579 39	Bdeogale nigripes GLC15 1	0,18542808
Procyon lotor AB462046 3	Arctocephalus townsendi NC008420 1	0,18542996
Meles meles T303 3	Cynogale bennetti KY117544 1	0,18543106
Mungos mungo/gambianus SRR77048	Martes martes T302 3	0,18543112
Neofelis nebulosa NC008450 3	Canis adustus KT448271 1	0,18543252
Galerella sanguinea T378 1	Bassariscus sumichrasti SRX1099089 1	0,18543354
Salanoia concolor D378 1	Arctocephalus pusillus NC008417 1	0,18543574
Martes zibellina NC011579 39	Canis mesomelas KT448280 1	0,1854362
Vulpes zerda KJ603240 1	Mustela erminea T305 2	0,18543632
Viverricula indica KX891751 1	Melogale moschata V0735A 1	0,18543735
Pardofelis marmorata NLN3 2	Meles anakuma NC009677 1	0,1854376
Pusa caspica NC008431 1	Paradoxurus hermaphroditus NC03959	0,18543826
Mustela nivalis T306 5	Urva javanica T413 1	0,18543963
Ursus maritimus GU573488 Svalbard	Melogale moschata V0735A 1	0,18544082
Ursus maritimus NC003428 31	Melogale moschata V0735A 1	0,18544082
Ursus maritimus GU573488 Svalbard	Smilodon populator MF871700 1	0,18544137
Panthera onca KP202264 2	Mustela itatsi NC034330 19	0,18544145
Mungos mungo/gambianus SRR77048	Lycaon pictus NC028427 2	0,18544215
Mustela putorius NC020638 4	Ichneumia albicauda T603 1	0,18544215
Prionailurus bengalensis NC028301 12	Arctocephalus townsendi NC008420 1	0,18544225
Mustela eversmannii NC028013 1	Genetta genetta T297 1	0,18544263
Canis adustus KT448271 1	Callorhinus ursinus NC008415 1	0,18544403
Lontra canadensis SRR10409165 1	Catopuma badia NC028300 1	0,1854447
Leopardus tigrinus NC028317 1	Arctocephalus gazella BK010918 1	0,1854454
Vulpes zerda KJ603240 1	Mirounga angustirostris SRR10331586	0,18544549
Tremarctos ornatus NC009969 2	Felis margarita NC028308 1	0,18544571
Nasua nasua NC020647 1	Monachus schauinslandi NC008421 1	0,18544608
Nyctereutes procyonoides NC013700 3	Felis margarita NC028308 1	0,18544622
Profelis aurata NC028299 1	Nyctereutes procyonoides NC013700 3	0,18544693
Lynx lynx NC027083 4	Lutrogale perspicillata NC035811 1	0,18544734
Urocyon littoralis catalinae KP129018	Monachus schauinslandi NC008421 1	0,18544744
Urocyon cinereoargenteus NC026723 2	Monachus schauinslandi NC008421 1	0,1854475
Prionailurus viverrinus NC028305 1	Phocarctos hookeri NC008418 1	0,18544826
Panthera tigris NC010642 35	Mustela frenata NC020640 1	0,18544929
Poecilogale albinucha T602 1	Otocolobus manul NC028323 1	0,18545012
Profelis aurata NC028299 1	Arctocephalus pusillus NC008417 1	0,18545015
Panthera onca NC022842 1	Mustela eversmannii NC028013 1	0,18545288
Ursus spelaeus EU327344 13	Lutra lutra LC050126 1	0,18545315
Lutra lutra NC011358 9	Canis aureus KT448274 1	0,18545382
Ursus arctos pruinosus MG066703 3	Civettictis civetta GLC19 1	0,18545592
Ursus arctos EU497665 29	Martes foina NC020643 1	0,18545607
Ursus thibetanus mupinensis NC00875	Felis nigripes NC028309 1	0,18545614
Ursus arctos isabellinus 18B5 2	Leopardus geoffroyi NC028320 1	0,18545865
Eumetopias jubatus NC004030 10	Civettictis civetta NC033378 1	0,18545885
Zalophus wollebaeki SRR4431565 1	Leopardus jacobita NC028322 1	0,18546171
Zalophus californianus NC008416 1	Salanoia concolor D378 1	0,18546204
Leopardus colocolo NC028314 1	Arctocephalus gazella BK010918 1	0,18546386
Urva brachyura KY117547 1	Canis lupus chanco NC010340 4	0,18546401
Ursus arctos AP012576 6	Bassariscus sumichrasti SRX1099089 1	0,18546405
Urva javanica/auropunctata NC006835	Canis latrans NC008093 7	0,18546407
Ursus arctos AP012576 6	Martes martes T302 3	0,18546416
Ursus thibetanus formosanus NC00933	Leopardus jacobita NC028322 1	0,18546617
Otocyon megalotis SAF1 2	Leopardus colocolo NC028314 1	0,18546626
Mustela sibirica AP017394 11	Urva javanica/auropunctata NC006835	0,1854665
Galictis vittata T412 1	Felis silvestris lybica KP202275 4	0,18546662
Ursus thibetanus laniger MH281753 2	Felis nigripes NC028309 1	0,18546687
Civettictis civetta GLC19 1	Arctocephalus forsteri NC004023 28	0,18546757
Ichneumia albicauda T603 1	Canis lupus familiaris NC002008 1231	0,18546772
Prionailurus planiceps NC028312 6	Helarctos malayanus NC009968 2	0,18546793
Urva javanica/auropunctata NC006835	Arctonyx collaris NC020645 1	0,18546796
Melursus ursinus NC009970 2	Felis silvestris lybica KP202275 4	0,18546833
Martes flavigula NC012141 3	Xenogale naso C07XAR110 1	0,18547395
Lynx canadensis NC028313 1	Arctocephalus forsteri NC004023 28	0,18547606
Lycaon pictus NC028427 2	Urva javanica/auropunctata NC006835	0,18547766
Neofelis nebulosa NC008450 3	Mustela erminea T305 2	0,1854794
Pardofelis marmorata NLN3 2	Canis lupus chanco NC010340 4	0,1854821
Cuon alpinus NC013445 3	Civettictis civetta GLC19 1	0,18548737
Paradoxurus hermaphroditus NC03959	Cuon alpinus NC013445 3	0,18548788
Eumetopias jubatus NC004030 10	Catopuma temminckii NC027115 41	0,18548894
Leopardus jacobita NC028322 1	Eumetopias jubatus NC004030 10	0,18549022
Lutra lutra LC050126 1	Atilax paludinosus T606 1	0,18549091
Melogale moschata V0735A 1	Crossarchus platycephalus C7R66 1	0,18549295
Meles leucurus NC039173 4	Cynictis penicillata T375 1	0,18549402
Mustela sibirica AP017394 11	Mungos mungo/gambianus SRR77048	0,18549454
Martes americana NC020642 1	Urva javanica T413 1	0,18549487
Martes americana NC020642 1	Cynictis penicillata T375 1	0,18549514
Eupleres goudotii D128 1	Callorhinus ursinus NC008415 1	0,18549597
Mustela frenata NC020640 1	Helogale parvula SRR7637809 1	0,18549669
Viverra tangalunga MH464792 1	Urva brachyura KY117547 1	0,18549681
Smilodon populator MF871700 1	Aonyx cinerea NC035814 2	0,18549703
Potos flavus T414 1	Canis latrans NC008093 7	0,18549709
Phocarctos hookeri NC008418 1	Mungos mungo MMC7 1	0,18549785
Canis latrans NC008093 7	Bdeogale nigripes GLC15 1	0,1854984
Prionodon linsang ERR2391707 1	Arctonyx collaris NC020645 1	0,18549857
Nyctereutes procyonoides NC013700 3	Bdeogale nigripes GLC15 1	0,18549922
Procyon lotor AB462046 3	Fossa fossana D350 1	0,18549965
Atilax paludinosus T606 1	Aonyx cinerea NC035814 2	0,18549982
Urva semitorquata MH464789 1	Aonyx cinerea NC035814 2	0,18550029
Bassaricyon neblina SRX1097850 1	Arctocephalus gazella BK010918 1	0,18550065
Ichneumia albicauda T603 1	Canis anthus NC027956 2	0,18550135
Meles anakuma NC009677 1	Canis latrans NC008093 7	0,18550164
Conepatus chinga NC042596 1	Caracal caracal NC028306 1	0,18550211
Viverricula indica KX891745 1	Arctocephalus australis MG023139 1	0,18550278
Lynx rufus NC014456 3	Ailurus fulgens NC011124 1	0,18550424
Paradoxurus jerdoni MH464793 1	Melogale moschata V0735A 1	0,18550432
Melogale moschata NC020644 1	Canis latrans NC008093 7	0,18550603
Taxidea taxus NC020646 1	Canis latrans NC008093 7	0,18550643
Vulpes corsac NC023958 1	Genetta servalina NC024568 2	0,18550685
Vulpes zerda KJ603240 1	Genetta servalina NC024568 2	0,18550749

Prionodon pardicolor NC024569 2	Nandinia binotata NC024567 1	0,14955126
Panthera leo spelaea KX258452 2	Genetta servalina NC024568 2	0,14955308
Paradoxurus hermaphroditus NLNC 1	Lynx rufus NC014456 3	0,14955331
Leopardus pardalis NC028315 1	Atilax paludinosus T606 1	0,14955541
Leopardus tigrinus NC028317 1	Crossarchus platycephalus C7R66 1	0,14955615
Viverricula indica KX891745 1	Galerella sanguinea T378 1	0,14955685
Puma yagouaroundi NC028311 1	Galidictis fasciata DM333 1	0,14956003
Panthera tigris amoyensis NC014770 2	Civettictis civetta GLC19 1	0,14956166
Lynx canadensis NC028313 1	Cryptoprocta ferox CFC13 1	0,14956225
Viverricula indica NC025296 2	Panthera tigris NC010642 35	0,14956468
Genetta abyssinica MG489822 1	Cryptoprocta ferox CFC13 1	0,14958076
Panthera onca NC022842 1	Genetta abyssinica MG489822 1	0,14959538
Prionodon linsang ERR2391707 1	Prionailurus planiceps NC028312 6	0,14961573
Prionailurus rubiginosus NC028304 2	Mungos mungo MMC7 1	0,14962
Viverricula indica KX891745 1	Galidictis fasciata DM333 1	0,14962137
Pardofelis marmorata NLN3 2	Paguma larvata PDDS11 2	0,14962219
Leopardus pardalis T262 1	Helogale parvula SRR7637809 1	0,14962222
Mungos mungo MMC7 1	Leopardus guigna NC028321 1	0,14962268
Prionailurus planiceps NC028312 6	Mungotictis decemlineata NC027828 1	0,14962605
Viverricula indica KX891751 1	Panthera onca NC022842 1	0,14963049
Panthera leo NERO 19	Genetta abyssinica MG489822 1	0,14963241
Mirounga angustirostris SRR10331586 1	Arctocephalus townsendi NC008420 1	0,1496834
Urva javanica T413 1	Cryptoprocta ferox CFC13 1	0,14968559
Lynx pardinus NC028319 161	Crossarchus platycephalus C7R66 1	0,1496868
Urva semitorquata MH464789 1	Felis silvestris lybica KP202275 4	0,14968732
Phoca vitulina NC001325 1	Arctocephalus australis MG023139 1	0,14968793
Viverricula indica NC025296 2	Xenogale naso C07XAR110 1	0,14968807
Puma yagouaroundi NC028311 1	Paradoxurus jerdoni MH464793 1	0,14968893
Mungos mungo MMC7 1	Leopardus jacobita NC028322 1	0,14968948
Prionailurus viverrinus NC028305 1	Mungos mungo/gambianus SRR7704821 1	0,14968994
Viverricula indica KX891745 1	Panthera pardus japonensis KJ866876 8	0,14969015
Prionailurus bengalensis NC028301 12	Cryptoprocta ferox CFC13 1	0,14969751
Prionailurus bengalensis NC028301 12	Parahyaena brunnea NC038159 15	0,14970502
Halichoerus grypus NC001602 2	Arctocephalus australis MG023139 1	0,1497177
Prionailurus viverrinus NC028305 1	Diplogale hosei MH464790 1	0,14973408
Panthera tigris NC010642 35	Civettictis civetta NC033378 1	0,1497357
Nandinia binotata NC024567 1	Helogale parvula SRR7637809 1	0,14974414
Pusa caspica NC008431 1	Arctocephalus forsteri NC004023 28	0,14974473
Odobenus rosmarus NC004029 29	Callorhinus ursinus NC008415 1	0,14974864
Leopardus jacobita NC028322 1	Urva semitorquata MH464789 1	0,1497573
Ichneumia albicauda T603 1	Genetta servalina NC024568 2	0,14976446
Urva javanica/auropunctata NC006835 1	Genetta genetta T297 1	0,14978471
Prionailurus planiceps NC028312 6	Diplogale hosei MH464790 1	0,14979732
Hemigalus derbyanus MH464791 1	Crossarchus platycephalus C7R66 1	0,14979734
Phocarctos hookeri NC008418 1	Cystophora cristata NC008427 1	0,1498093
Prionodon linsang ERR2391707 1	Leopardus pardalis T262 1	0,14981589
Prionodon linsang ERR2391707 1	Leopardus pardalis NC028315 1	0,1498159
Viverricula indica KX891745 1	Panthera leo spelaea KX258452 2	0,14982192
Prionailurus viverrinus NC028305 1	Paradoxurus hermaphroditus NLNC 1	0,14982212
Viverricula indica NC025296 2	Urva brachyura KY117547 1	0,14982264
Crossarchus platycephalus C7R66 1	Caracal caracal NC028306 1	0,14982307
Paradoxurus jerdoni MH464793 1	Felis silvestris lybica KP202275 4	0,14982311
Prionailurus planiceps KY682741 4	Paradoxurus jerdoni MH464793 1	0,14982326
Prionailurus rubiginosus NC028304 2	Galerella sanguinea T378 1	0,14982361
Paradoxurus hermaphroditus NLNC 1	Felis chaus NC028307 1	0,14982392
Genetta abyssinica MG489822 1	Galidia elegans D146 1	0,14982793
Leptailurus serval NC028316 1	Galidictis fasciata DM333 1	0,14982961
Leopardus colocolo NC028314 1	Hyaena hyaena NC020669 1	0,14985214
Prionodon pardicolor NC024569 2	Hemigalus derbyanus MH464791 1	0,14985235
Neofelis nebulosa NC008450 3	Civettictis civetta GLC19 1	0,14985365
Panthera pardus NC010641 5	Genetta genetta T297 1	0,14985764
Prionailurus planiceps NC028312 6	Chrotogale owstoni T607 1	0,14987998
Cynictis penicillata T375 1	Civettictis civetta GLC19 1	0,14988641
Puma concolor NC016470 22	Crossarchus platycephalus C7R66 1	0,1498898
Viverra zibetha T609 1	Mungotictis decemlineata NC027828 1	0,14989035
Paradoxurus jerdoni MH464793 1	Felis nigripes NC028309 1	0,14989054
Leopardus guigna NC028321 1	Crossarchus platycephalus C7R66 1	0,14989148
Hyaena hyaena NC020669 1	Helogale parvula SRR7637809 1	0,14989176
Proteles cristata T393 6	Crossarchus platycephalus C7R66 1	0,14989468
Paradoxurus hermaphroditus NC039591 1	Catopuma temminckii NC027115 41	0,14989956
Smilodon populator MF871700 1	Galerella sanguinea T378 1	0,14990054
Cystophora cristata NC008427 1	Arctocephalus townsendi NC008420 1	0,14994451
Pusa sibirica NC008432 2	Arctocephalus forsteri NC004023 28	0,14994614
Prionailurus planiceps KY682741 4	Chrotogale owstoni T607 1	0,14994739
Phoca groenlandica NC008429 54	Arctocephalus forsteri NC004023 28	0,14994796
Suricata suricatta SSM10 1	Galidia elegans D146 1	0,14994892
Phoca vitulina NC001325 1	Arctocephalus townsendi NC008420 1	0,14995667
Puma yagouaroundi NC028311 1	Mungos mungo MMC7 1	0,14995669
Prionailurus planiceps NC028312 6	Helogale parvula SRR7637809 1	0,14995718
Paradoxurus hermaphroditus NLNC 1	Lynx pardinus NC028319 161	0,14995734
Smilodon populator MF871700 1	Atilax paludinosus T606 1	0,14995867
Panthera pardus japonensis KJ866876 8	Genetta genetta T297 1	0,14996153
Salanoia concolor D378 1	Felis margarita NC028308 1	0,14996275
Salanoia concolor D378 1	Puma concolor NC016470 22	0,1499631
Panthera tigris NC010642 35	Genetta servalina NC024568 2	0,14996566
Genetta abyssinica MG489822 1	Eupleres goudotii D128 1	0,14996792
Cynictis penicillata T375 1	Civettictis civetta NC033378 1	0,14998591
Genetta abyssinica MG489822 1	Bdeogale nigripes GLC15 1	0,14999554
Paradoxurus hermaphroditus NLNC 1	Mungos mungo MMC7 1	0,15001835
Paguma larvata PDDS11 2	Mungos mungo MMC7 1	0,15001953
Viverra zibetha T609 1	Urva semitorquata MH464789 1	0,15002081
Viverra zibetha T609 1	Helogale parvula SRR7637809 1	0,15002099
Felis silvestris lybica KP202275 4	Atilax paludinosus T606 1	0,1500247
Viverricula indica KX891745 1	Salanoia concolor D378 1	0,15002503
Puma concolor NC016470 22	Paradoxurus jerdoni MH464793 1	0,15002528
Viverricula indica NC025296 2	Helogale parvula SRR7637809 1	0,15002552
Mungos mungo/gambianus SRR7704821 1	Leopardus pardalis NC028315 1	0,15002556
Lynx rufus NC014456 3	Eupleres goudotii D128 1	0,15002578
Leopardus wiedii NC028318 1	Ichneumia albicauda T603 1	0,15002707
Galidictis fasciata DM333 1	Felis silvestris lybica KP202275 4	0,15002997
Viverricula indica NC025296 2	Panthera uncia NC010638 1	0,15003017
Prionailurus planiceps NC028312 6	Galidictis fasciata DM333 1	0,15003031
Salanoia concolor D378 1	Leopardus guigna NC028321 1	0,1500306

Viverra tangalunga MH464792 1	Panthera onca KP202264 2	0,1855083
Ursus maritimus GU573488 Svalbard	Fossa fossana D350 1	0,18550861
Cryptoprocta ferox CFC13 1	Aonyx cinerea NC035814 2	0,18550935
Panthera pardus japonensis KJ866876	Mustela nigripes NC024942 1	0,18550937
Felis silvestris lybica KP202275 4	Arctocephalus australis MG023139 1	0,18550953
Otocolobus manul NC028323 1	Arctocephalus australis MG023139 1	0,18550986
Lynx pardinus NC028319 161	Arctocephalus townsendi NC008420 1	0,18551016
Meles anakuma NC009677 1	Canis adustus KT448271 1	0,18551038
Vulpes corsac NC023958 1	Profelis aurata NC028299 1	0,18551049
Mustela nivalis T306 5	Canis anthus NC027956 2	0,18551135
Viverricula indica NC025296 2	Urocyon cinereoargenteus NC026723 2	0,18551151
Paguma larvata PDDS11 2	Nyctereutes procyonoides NC013700 3	0,18551162
Prionailurus rubiginosus NC028304 2	Lontra canadensis SRR10409165 1	0,18551247
Viverricula indica KX891745 1	Otocyon megalotis SAF1 2	0,18551247
Parahyaena brunnea NC038159 15	Mustela nigripes NC024942 1	0,1855125
Tremarctos ornatus NC009969 2	Puma yagouaroundi NC028311 1	0,18551286
Ursus arctos EU497665 29	Smilodon populator MF871700 1	0,18551296
Ursus arctos pruinosus MG066703 3	Melogale moschata V0735A 1	0,18551438
Lynx rufus NC014456 3	Aonyx cinerea NC035814 2	0,18551499
Ursus thibetanus mupinensis NC00875	Aonyx cinerea NC035814 2	0,185516
Viverricula indica KX891751 1	Ursus maritimus GU573488 Svalbard	0,18551646
Ursus arctos isabellinus 18B5 2	Aonyx cinerea NC035814 2	0,18551651
Phoca vitulina NC001325 1	Paradoxurus hermaphroditus NC03959	0,18551716
Prionailurus rubiginosus NC028304 2	Lutrogale perspicillata NC035811 1	0,18551737
Puma yagouaroundi NC028311 1	Aonyx cinerea NC035814 2	0,1855185
Panthera onca NC022842 1	Callorhinus ursinus NC008415 1	0,18551853
Viverricula indica KX891745 1	Otaria byronia OTAB 1	0,18551907
Ursus arctos pruinosus MG066703 3	Poecilogale albinucha T602 1	0,18552054
Puma yagouaroundi NC028311 1	Otaria byronia OTAB 1	0,1855215
Ursus arctos GU573486 5	Aonyx cinerea NC035814 2	0,18552235
Speothos venaticus C48 2	Paradoxurus jerdoni MH464793 1	0,18552303
Panthera onca KP202264 2	Ictonyx striatus T299 1	0,18552479
Proteles cristata T393 6	Phoca groenlandica NC008429 54	0,18552575
Zalophus wollebaeki SRR4431565 1	Pardofelis marmorata NLN3 2	0,18552633
Procyon lotor AB462046 3	Helarctos malayanus NC009968 2	0,18552773
Xenogale naso C07XAR110 1	Canis lupus chanco NC010340 4	0,18552962
Lycaon pictus NC028427 2	Catopuma temminckii NC027115 41	0,18552993
Genetta genetta T297 1	Canis aureus KT448274 1	0,1855302
Gulo gulo NC009685 3	Eupleres goudotii D128 1	0,18553238
Ursus thibetanus formosanus NC00933	Felis catus NC001700 2	0,18553273
Ursus spelaeus EU327344 13	Paguma larvata PDDS11 2	0,18553354
Prionailurus rubiginosus NC028304 2	Galictis vittata T412 1	0,18553446
Zalophus wollebaeki SRR4431565 1	Galictis vittata T412 1	0,185535
Ursus thibetanus formosanus NC00933	Lycaon pictus NC028427 2	0,18553594
Procyon lotor AB462046 3	Arctocephalus forsteri NC004023 28	0,185536
Mungos mungo/gambianus SRR77048	Arctocephalus forsteri NC004023 28	0,18553644
Ursus thibetanus laniger MH281753 2	Otocolobus manul NC028323 1	0,18553661
Puma yagouaroundi NC028311 1	Helarctos malayanus NC009968 2	0,18553731
Panthera onca NC022842 1	Canis aureus KT448274 1	0,18554135
Zalophus californianus NC008416 1	Felis chaus NC028307 1	0,18554144
Zalophus wollebaeki SRR4431565 1	Leopardus colocolo NC028314 1	0,18554789
Ursus arctos EU497665 29	Civettictis civetta NC033378 1	0,18555641
Urva javanica T413 1	Enhydra lutris NC009692 1	0,18555781
Melogale moschata V0735A 1	Eupleres goudotii D128 1	0,18556079
Meles meles T303 3	Bdeogale nigripes GLC15 1	0,18556149
Mustela itatsi NC034330 19	Urva brachyura KY117547 1	0,18556403
Ursus thibetanus formosanus NC00933	Spilogale putorius NC010497 1	0,18556423
Neophoca cinerea NC008419 1	Eupleres goudotii D128 1	0,18556511
Melogale moschata KP726273 1	Galidia elegans D146 1	0,18556516
Galidictis fasciata DM333 1	Callorhinus ursinus NC008415 1	0,18556729
Xenogale naso C07XAR110 1	Aonyx cinerea NC035814 2	0,18556747
Mustela nigripes NC024942 1	Galidictis fasciata DM333 1	0,18556778
Cynogale bennetti KY117544 1	Ailurus fulgens NC011124 1	0,18556787
Prionodon linsang ERR2391707 1	Canis anthus NC027956 2	0,18556871
Vulpes zerda KJ603240 1	Mustela frenata NC020640 1	0,18556958
Civettictis civetta GLC19 1	Arctocephalus townsendi NC008420 1	0,18557176
Viverricula indica KX891751 1	Martes americana NC020642 1	0,18557194
Prionodon pardicolor NC024569 2	Lutra sumatrana NC035810 1	0,18557294
Martes zibellina NC011579 39	Cryptoprocta ferox CFC13 1	0,18557322
Profelis aurata NC028299 1	Meles anakuma NC009677 1	0,18557325
Leopardus guigna NC028321 1	Conepatus chinga NC042596 1	0,18557341
Prionailurus planiceps NC028312 6	Ailurus fulgens NC011124 1	0,18557352
Melogale moschata NC020644 1	Galidia elegans D146 1	0,18557358
Smilodon populator MF871700 1	Arctocephalus gazella BK010918 1	0,18557388
Martes americana NC020642 1	Ichneumia albicauda T603 1	0,18557437
Mustela sibirica AP017394 11	Hyaena hyaena NC020669 1	0,18557473
Mustela putorius NC020638 4	Cryptoprocta ferox CFC13 1	0,18557544
Parahyaena brunnea NC038159 15	Lutra sumatrana NC035810 1	0,18557568
Ursus arctos GU573491 207	Fossa fossana D350 1	0,18557583
Prionailurus rubiginosus NC028304 2	Arctocephalus townsendi NC008420 1	0,18557666
Vulpes corsac NC023958 1	Leopardus wiedii NC028318 1	0,18557738
Ursus arctos isabellinus 18B5 2	Chrotogale owstoni T607 1	0,18557759
Vulpes ferrilata NC027935 1	Felis silvestris lybica KP202275 4	0,18557778
Galerella sanguinea T378 1	Arctocephalus pusillus NC008417 1	0,18557829
Tremarctos ornatus NC009969 2	Leopardus jacobita NC028322 1	0,18557836
Ursus arctos isabellinus 18B5 2	Phocarctos hookeri NC008418 1	0,18557923
Vulpes zerda KJ603240 1	Prionailurus viverrinus NC028305 1	0,18557929
Leopardus pardalis T262 1	Arctocephalus gazella BK010918 1	0,18557984
Lycalopex sechurae KT448284 1	Arctotherium sp NC030174 1	0,18557984
Ursus arctos isabellinus 18B5 2	Lutra sumatrana NC035810 1	0,1855803
Poecilogale albinucha T602 1	Crossarchus platycephalus C7R66 1	0,18558055
Proteles cristata T393 6	Lutra lutra LC050126 1	0,18558128
Vulpes ferrilata NC027935 1	Catopuma badia NC028300 1	0,18558149
Martes martes T302 3	Cryptoprocta ferox CFC13 1	0,18558159
Genetta genetta T297 1	Arctotherium sp NC030174 1	0,18558227
Viverra tangalunga MH464792 1	Ursus arctos isabellinus 18B5 2	0,18558261
Viverra tangalunga MH464792 1	Ursus arctos EU497665 29	0,18558267
Galictis vittata T412 1	Arctocephalus forsteri KT693377 17	0,1855848
Ursus arctos pruinosus MG066703 3	Mustela altaica NC021751 1	0,18558532
Lutra lutra NC011358 9	Helarctos malayanus NC009968 2	0,1855856
Mustela nivalis T306 5	Genetta genetta T297 1	0,18558642
Otaria byronia OTAB 1	Catopuma temminckii NC027115 41	0,18558677
Profelis aurata NC028299 1	Martes martes T302 3	0,18558775

Leopardus pardalis NC028315 1	Galidia elegans D146 1	0,15003183
Lynx canadensis NC028313 1	Hyaena hyaena NC020669 1	0,15003644
Prionailurus planiceps KY682741 4	Diplogale hosei MH464790 1	0,15006764
Viverra zibetha T609 1	Atilax paludinosus T606 1	0,15008818
Fossa fossana D350 1	Catopuma temminckii NC027115 41	0,15008937
Profelis aurata NC028299 1	Crossarchus platycephalus C7R66 1	0,15009242
Mungotictis decemlineata NC027828 1	Genetta genetta T297 1	0,15009262
Mungotictis decemlineata NC027828 1	Hyaena hyaena NC020669 1	0,15009606
Salanoia concolor D378 1	Caracal caracal NC028306 1	0,15009841
Salanoia concolor D378 1	Leptailurus serval NC028316 1	0,15009886
Viverricula indica KX891751 1	Prionodon pardicolor NC024569 2	0,15009941
Felis silvestris lybica KP202275 4	Cryptoprocta ferox CFC13 1	0,15010105
Prionodon pardicolor NC024569 2	Genetta servalina NC024568 2	0,1501013
Prionodon pardicolor NC024569 2	Lynx canadensis NC028313 1	0,15010241
Prionodon pardicolor NC024569 2	Felis catus NC001700 2	0,15010284
Genetta abyssinica MG489822 1	Atilax paludinosus T606 1	0,15011761
Urva semitorquata MH464789 1	Genetta abyssinica MG489822 1	0,15012553
Prionodon linsang ERR2391707 1	Lynx pardinus NC028319 161	0,15015407
Viverra zibetha T609 1	Mungos mungo MMC7 1	0,15015526
Mungotictis decemlineata NC027828 1	Homotherium latidens MF871702 3	0,15015839
Pardofelis marmorata NLN3 2	Fossa fossana D350 1	0,15015897
Parahyaena brunnea NC038159 15	Galerella sanguinea T378 1	0,15016314
Prionodon linsang ERR2391707 1	Genetta abyssinica MG489822 1	0,15016373
Fossa fossana D350 1	Crocuta crocuta NC020670 3	0,15016565
Helogale parvula SRR7637809 1	Crocuta crocuta NC020670 3	0,15016623
Lynx lynx NC027083 4	Hyaena hyaena NC020669 1	0,1501715
Urva javanica/auropunctata NC006835 1	Cryptoprocta ferox CFC13 1	0,1501946
Lynx pardinus NC028319 161	Chrotogale owstoni T607 1	0,15021801
Mungos mungo MMC7 1	Leptailurus serval NC028316 1	0,15022567
Prionailurus rubiginosus NC028304 2	Cynictis penicillata T375 1	0,15022591
Felis nigripes NC028309 1	Crossarchus platycephalus C7R66 1	0,15022594
Paradoxurus hermaphroditus NC039591 1	Felis margarita NC028308 1	0,15022697
Leptailurus serval NC028316 1	Crossarchus platycephalus C7R66 1	0,15022707
Leptailurus serval NC028316 1	Urva semitorquata MH464789 1	0,15022722
Paradoxurus hermaphroditus NLNC 1	Felis margarita NC028308 1	0,15022734
Mungos mungo/gambianus SRR7704821 1	Leopardus tigrinus NC028317 1	0,15022829
Arctotherium sp NC030174 1	Ailuropoda melanoleuca NC009492 5	0,15022835
Viverricula indica KX891745 1	Panthera uncia KP202269 1	0,15022881
Leopardus guigna NC028321 1	Atilax paludinosus T606 1	0,15022916
Viverra zibetha T609 1	Prionodon pardicolor NC024569 2	0,15023547
Prionailurus viverrinus NC028305 1	Galidictis fasciata DM333 1	0,15023614
Prionodon linsang ERR2391707 1	Leopardus colocolo NC028314 1	0,15023682
Viverricula indica NC025296 2	Panthera tigris amoyensis NC014770 2	0,15023839
Leopardus pardalis NC028315 1	Hyaena hyaena NC020669 1	0,15024161
Pardofelis marmorata NLN3 2	Arctictis binturong T605 2	0,15024196
Homotherium latidens MF871702 3	Crossarchus platycephalus C7R66 1	0,15024716
Puma concolor NC016470 22	Diplogale hosei MH464790 1	0,1502735
Prionodon linsang ERR2391707 1	Leopardus wiedii NC028318 1	0,15028745
Mungos mungo MMC7 1	Homotherium latidens MF871702 3	0,15028824
Viverricula indica KX891745 1	Suricata suricatta SSM10 1	0,15028998
Genetta servalina NC024568 2	Cynictis penicillata T375 1	0,15029024
Paradoxurus jerdoni MH464793 1	Leopardus pardalis NC028315 1	0,15029196
Neofelis nebulosa NC008450 3	Civettictis civetta NC033378 1	0,15029351
Paguma larvata PDDS11 2	Felis catus NC001700 2	0,15029542
Galidictis fasciata DM333 1	Catopuma temminckii NC027115 41	0,15029638
Hyaena hyaena NC020669 1	Eupleres goudotii D128 1	0,15029664
Parahyaena brunnea NC038159 15	Xenogale naso C07XAR110 1	0,15029696
Paradoxurus hermaphroditus NLNC 1	Leopardus guigna NC028321 1	0,15030189
Parahyaena brunnea NC038159 15	Lynx lynx NC027083 4	0,15030819
Galidictis fasciata DM333 1	Acinonyx jubatus NC005212 3	0,15031619
Viverricula indica KX891751 1	Panthera leo NERO 19	0,1503274
Paradoxurus hermaphroditus NLNC 1	Fossa fossana D350 1	0,15035592
Puma concolor NC016470 22	Prionodon linsang ERR2391707 1	0,15035631
Viverra zibetha T609 1	Prionodon linsang ERR2391707 1	0,15035711
Genetta genetta T297 1	Crossarchus platycephalus C7R66 1	0,15035947
Mungos mungo MMC7 1	Catopuma badia NC028300 1	0,15036002
Eupleres goudotii D128 1	Catopuma badia NC028300 1	0,15036041
Prionodon linsang ERR2391707 1	Catopuma badia NC028300 1	0,15036213
Felis nigripes NC028309 1	Eupleres goudotii D128 1	0,15036231
Paradoxurus jerdoni MH464793 1	Caracal caracal NC028306 1	0,1503624
Salanoia concolor D378 1	Otocolobus manul NC028323 1	0,15036853
Urocyon cinereoargenteus NC026723 21	Cuon alpinus NC013445 3	0,15036917
Leopardus geoffroyi NC028320 1	Cryptoprocta ferox CFC13 1	0,15037058
Parahyaena brunnea NC038159 15	Lynx canadensis NC028313 1	0,1503756
Genetta abyssinica MG489822 1	Cynictis penicillata T375 1	0,15038618
Neofelis nebulosa NC008450 3	Genetta servalina NC024568 2	0,15039267
Viverricula indica KX891745 1	Panthera pardus NC010641 5	0,15039456
Hemigalus derbyanus MH464791 1	Eupleres goudotii D128 1	0,15041208
Viverricula indica KX891751 1	Urva javanica T413 1	0,15042467
Proteles cristata T393 6	Fossa fossana D350 1	0,15043159
Prionailurus bengalensis CKM45 20	Mungos mungo/gambianus SRR7704821 1	0,15043217
Prionodon linsang ERR2391707 1	Acinonyx jubatus NC005212 3	0,15043534
Viverricula indica KX891745 1	Prionodon pardicolor NC024569 2	0,15043578
Ichneumia albicauda T603 1	Civettictis civetta GLC19 1	0,15043625
Parahyaena brunnea NC038159 15	Cryptoprocta ferox CFC13 1	0,15043934
Viverra zibetha T609 1	Neofelis nebulosa NC008450 3	0,15046045
Urva javanica/auropunctata NC006835 1	Catopuma badia NC028300 1	0,15046078
Urva javanica T413 1	Genetta servalina NC024568 2	0,15049244
Puma concolor NC016470 22	Mungos mungo MMC7 1	0,15049495
Leopardus pardalis T262 1	Urva javanica T413 1	0,1504955
Felis margarita NC028308 1	Crossarchus platycephalus C7R66 1	0,15049557
Fossa fossana D350 1	Felis nigripes NC028309 1	0,15049568
Paradoxurus jerdoni MH464793 1	Lynx pardinus NC028319 161	0,15049601
Fossa fossana D350 1	Felis chaus NC028307 1	0,15049605
Panthera leo spelaea KX258452 2	Genetta genetta T297 1	0,15049675
Leopardus geoffroyi NC028320 1	Urva semitorquata MH464789 1	0,15049765
Panthera pardus japonensis KJ866876 8	Mungotictis decemlineata NC027828 1	0,15050039
Galidictis fasciata DM333 1	Caracal caracal NC028306 1	0,15050255
Prionodon pardicolor NC024569 2	Civettictis civetta GLC19 1	0,15050304
Prionailurus bengalensis CKM45 20	Parahyaena brunnea NC038159 15	0,15052186
Pardofelis marmorata NLN3 2	Hemigalus derbyanus MH464791 1	0,15053008
Salanoia concolor D378 1	Panthera leo NERO 19	0,15053426
Urva brachyura KY117547 1	Galidia elegans D146 1	0,15055574

Ursus arctos EU497665 29	Poecilogale albinucha T602 1	0,18558812
Prionailurus bengalensis NC028301 12	Otaria byronia OTAB 1	0,18558837
Panthera onca NC022842 1	Canis latrans NC008093 7	0,18558942
Neovison vison NC020641 3	Caracal caracal NC028306 1	0,18558961
Phocarctos hookeri NC008418 1	Melursus ursinus NC009970 2	0,1855901
Pardofelis marmorata NLN3 2	Lycaon pictus NC028427 2	0,18559152
Ictonyx striatus T299 1	Felis nigripes NC028309 1	0,18559203
Ursus thibetanus mupinensis NC00875	Leptailurus serval NC028316 1	0,18559307
Lycaon pictus NC028427 2	Leopardus geoffroyi NC028320 1	0,18559609
Ursus spelaeus EU327344 13	Phocarctos hookeri NC008418 1	0,18559693
Ursus spelaeus EU327344 13	Lynx rufus NC014456 3	0,18559718
Lynx canadensis NC028313 1	Galictis vittata T412 1	0,18559775
Ursus spelaeus EU327344 13	Felis margarita NC028308 1	0,18559808
Meles anakuma NC009677 1	Canis lupus familiaris NC002008 1231	0,18560055
Lynx rufus NC014456 3	Helarctos malayanus NC009968 2	0,18560056
Prionodon linsang ERR2391707 1	Canis lupus familiaris NC002008 1231	0,18560109
Leopardus pardalis NC028315 1	Galictis vittata T412 1	0,18560195
Ursus thibetanus laniger MH281753 2	Puma concolor NC016470 22	0,18560244
Puma concolor NC016470 22	Chrysocyon brachyurus NC024172 1	0,18560331
Melursus ursinus NC009970 2	Leopardus wiedii NC028318 1	0,18560362
Genetta abyssinica MG489822 1	Arctocephalus australis MG023139 1	0,18560392
Leopardus geoffroyi NC028320 1	Helarctos malayanus NC009968 2	0,18560626
Gulo gulo NC009685 3	Galidictis fasciata DM333 1	0,18560889
Lynx lynx NC027083 4	Arctocephalus forsteri NC004023 28	0,18561137
Pardofelis marmorata NLN3 2	Canis lupus familiaris NC002008 1231	0,1856126
Leopardus wiedii NC028318 1	Cuon alpinus NC013445 3	0,18561959
Viverra tangalunga MH464792 1	Canis mesomelas KT448280 1	0,18562092
Ursus arctos isabellinus 18B5 2	Civettictis civetta NC033378 1	0,18562368
Lutra lutra NC011358 9	Xenogale naso C07XAR110 1	0,18562466
Fossa fossana D350 1	Conepatus chinga NC042596 1	0,18562539
Martes melampus NC009678 1	Atilax paludinosus T606 1	0,18562593
Martes americana NC020642 1	Xenogale naso C07XAR110 1	0,18562917
Mustela putorius NC020638 4	Mungos mungo/gambianus SRR77048	0,18562949
Melogale moschata KP726273 1	Urva javanica T413 1	0,18562962
Crossarchus platycephalus C7R66 1	Bassaricyon neblina SRX1097850 1	0,18562999
Mustela sibirica NC020637 6	Atilax paludinosus T606 1	0,1856304
Galerella sanguinea T378 1	Canis anthus NC027956 2	0,18563277
Mungos mungo MMC7 1	Martes martes T302 3	0,18563382
Lutrogale perspicillata NC035811 1	Fossa fossana D350 1	0,18563519
Smilodon populator MF871700 1	Conepatus chinga NC042596 1	0,18563521
Vulpes ferrilata NC027935 1	Mustela nigripes NC024942 1	0,18563636
Genetta genetta T297 1	Enhydra lutris NC009692 1	0,18563691
Ursus americanus JX196366 3	Acinonyx jubatus NC005212 3	0,18563727
Martes melampus NC009678 1	Ichneumia albicauda T603 1	0,18563788
Mustela altaica NC021751 1	Arctocephalus australis MG023139 1	0,18563958
Viverra tangalunga MH464792 1	Galerella sanguinea T378 1	0,18564039
Panthera pardus japonensis KJ866876	Enhydra lutris NC009692 1	0,18564071
Tremarctos ornatus NC009969 2	Mustela altaica NC021751 1	0,18564276
Mungos mungo MMC7 1	Ictonyx striatus T299 1	0,18564357
Ursus thibetanus mupinensis NC00875	Crossarchus platycephalus C7R66 1	0,18564397
Vulpes corsac NC023958 1	Felis catus NC001700 2	0,18564451
Panthera uncia NC010638 1	Mustela erminea T305 2	0,18564467
Vulpes ferrilata NC027935 1	Felis nigripes NC028309 1	0,18564485
Viverricula indica KX891745 1	Aonyx cinerea NC035814 2	0,18564606
Martes martes T302 3	Canis mesomelas KT448280 1	0,18564694
Lontra canadensis SRR10409165 1	Felis nigripes NC028309 1	0,18564703
Hemigalus derbyanus MH464791 1	Arctocephalus australis MG023139 1	0,18564726
Ursus maritimus NC003428 31	Lutra sumatrana NC035810 1	0,18564774
Neophoca cinerea NC008419 1	Catopuma badia NC028300 1	0,18564795
Prionailurus rubiginosus NC028304 2	Neophoca cinerea NC008419 1	0,18564862
Ursus arctos GU573486 5	Melogale moschata V0735A 1	0,18564893
Neophoca cinerea NC008419 1	Leptailurus serval NC028316 1	0,18564928
Otocolobus manul NC028323 1	Neophoca cinerea NC008419 1	0,18564932
Catopuma temminckii NC027115 41	Arctocephalus pusillus NC008417 1	0,18565048
Lutrogale perspicillata NC035811 1	Felis silvestris lybica KP202275 4	0,18565057
Prionailurus bengalensis CKM45 20	Lontra canadensis SRR10409165 1	0,18565062
Procyon lotor AB462049 4	Leopardus jacobita NC028322 1	0,18565169
Viverricula indica NC025296 2	Ursus arctos isabellinus 18B5 2	0,18565272
Neovison vison NC020641 3	Hyaena hyaena NC020669 1	0,18565315
Ursus arctos GU573486 5	Meles anakuma NC009677 1	0,18565436
Panthera leo spelaea KX258452 2	Mustela erminea T305 2	0,18565485
Viverricula indica KX891751 1	Speothos venaticus C48 2	0,18565539
Zalophus wollebaeki SRR4431565 1	Viverricula indica KX891751 1	0,18565886
Speothos venaticus C48 2	Prionailurus bengalensis NC028301 12	0,18565923
Vulpes ferrilata NC027935 1	Leopardus colocolo NC028314 1	0,18566331
Ursus arctos AP012576 6	Lutra sumatrana NC035810 1	0,18566382
Urva brachyura KY117547 1	Canis lupus familiaris NC002008 1231	0,18566661
Ursus thibetanus thibetanus NC01111	Paradoxurus jerdoni MH464793 1	0,18566664
Ursus arctos GU573491 207	Canis adustus KT448271 1	0,18566762
Zalophus californianus NC008416 1	Galidictis fasciata DM333 1	0,18566798
Prionailurus viverrinus NC028305 1	Chrysocyon brachyurus NC024172 1	0,18566956
Ursus spelaeus NC011112 8	Felis chaus NC028307 1	0,18566996
Helogale parvula SRR7637809 1	Canis adustus KT448271 1	0,18567076
Ursus arctos GU573486 5	Arctocephalus forsteri NC004023 28	0,18567316
Melursus ursinus NC009970 2	Leptailurus serval NC028316 1	0,18567319
Ursus spelaeus NC011112 8	Leopardus guigna NC028321 1	0,18567386
Gulo gulo NC009685 3	Canis latrans NC008093 7	0,18567483
Eumetopias jubatus NC004030 10	Bdeogale nigripes GLC15 1	0,18567815
Zalophus californianus NC008416 1	Leopardus guigna NC028321 1	0,18567896
Mustela nivalis T306 5	Cuon alpinus NC013445 3	0,18568984
Ursus maritimus GU573488 Svalbard	Civettictis civetta NC033378 1	0,18569121
Martes melampus NC009678 1	Xenogale naso C07XAR110 1	0,18569346
Mustela nigripes NC024942 1	Eupleres goudotii D128 1	0,18569705
Ursus arctos GU573486 5	Civettictis civetta NC033378 1	0,18569756
Mustela kathiah NC023210 1	Xenogale naso C07XAR110 1	0,18569845
Tremarctos ornatus NC009969 2	Lutrogale perspicillata NC035811 1	0,18569855
Bdeogale nigripes GLC15 1	Arctocephalus forsteri KT693377 17	0,18569884
Procyon lotor AB462049 4	Arctocephalus townsendi NC008420 1	0,18569928
Vulpes zerda KJ603240 1	Melogale moschata KP726273 1	0,1856999
Xenogale naso C07XAR110 1	Canis latrans NC008093 7	0,18570009
Procyon lotor AB462049 4	Arctocephalus forsteri KT693377 17	0,18570016
Lynx lynx NC027083 4	Canis mesomelas KT448280 1	0,18570086
Ursus spelaeus EU327344 13	Spilogale putorius NC010497 1	0,18570104

Prionailurus bengalensis NC028301 12	Mungos mungo MMC7 1	0,15056261
Lynx canadensis NC028313 1	Eupleres goudotii D128 1	0,15056427
Prionailurus viverrinus NC028305 1	Urva semitorquata MH464789 1	0,15056579
Panthera onca KP202264 2	Genetta genetta T297 1	0,15056734
Salanoia concolor D378 1	Pardofelis marmorata NLN3 2	0,15056862
Viverricula indica KX891751 1	Ichneumia albicauda T603 1	0,15056896
Prionailurus planiceps KY682741 4	Galidictis fasciata DM333 1	0,15056908
Felis margarita NC028308 1	Cryptoprocta ferox CFC13 1	0,15057254
Puma concolor NC016470 22	Cryptoprocta ferox CFC13 1	0,15057282
Prionodon pardicolor NC024569 2	Civettictis civetta NC033378 1	0,15060516
Paradoxurus hermaphroditus NC039591 1	Nandinia binotata NC024567 1	0,15062572
Phocarctos hookeri NC008418 1	Mirounga angustirostris SRR10331586 1	0,15062763
Neophoca cinerea NC008419 1	Mirounga angustirostris SRR10331586 1	0,15062882
Mungos mungo/gambianus SRR7704821 1	Catopuma temminckii NC027115 41	0,15062925
Paradoxurus hermaphroditus NC039591 1	Felis silvestris lybica KP202275 4	0,15063083
Prionailurus bengalensis NC028301 12	Bdeogale nigripes GLC15 1	0,15063117
Viverricula indica KX891751 1	Panthera onca KP202264 2	0,1506325
Mungos mungo MMC7 1	Leopardus geoffroyi NC028320 1	0,15063263
Parahyaena brunnea NC038159 15	Atilax paludinosus T606 1	0,15063322
Parahyaena brunnea NC038159 15	Cynictis penicillata T375 1	0,15063509
Salanoia concolor D378 1	Profelis aurata NC028299 1	0,15063734
Parahyaena brunnea NC038159 15	Mungos mungo MMC7 1	0,15063737
Profelis aurata NC028299 1	Galidictis fasciata DM333 1	0,15063755
Urocyon littoralis catalinae KP129018 15	Cuon alpinus NC013445 3	0,15063863
Viverricula indica KX891745 1	Panthera tigris NC010642 35	0,1506393
Profelis aurata NC028299 1	Diplogale hosei MH464790 1	0,15067005
Leopardus guigna NC028321 1	Diplogale hosei MH464790 1	0,1506831
Viverricula indica KX891745 1	Xenogale naso C07XAR110 1	0,1506936
Felis chaus NC028307 1	Diplogale hosei MH464790 1	0,15069535
Lynx rufus NC014456 3	Helogale parvula SRR7637809 1	0,15069664
Paradoxurus jerdoni MH464793 1	Leopardus jacobita NC028322 1	0,15069667
Viverricula indica KX891751 1	Mungotictis decemlineata NC027828 1	0,15069844
Felis silvestris lybica KP202275 4	Eupleres goudotii D128 1	0,15069906
Prionodon pardicolor NC024569 2	Paguma larvata PDDS11 2	0,15070577
Otocolobus manul NC028323 1	Cryptoprocta ferox CFC13 1	0,15070786
Panthera leo NERO 19	Genetta genetta T297 1	0,15073277
Nandinia binotata NC024567 1	Urva brachyura KY117547 1	0,15075411
Viverricula indica KX891745 1	Eupleres goudotii D128 1	0,15075813
Puma yagouaroundi NC028311 1	Prionodon linsang ERR2391707 1	0,1507605
Viverricula indica KX891751 1	Cynictis penicillata T375 1	0,15076111
Paradoxurus jerdoni MH464793 1	Felis chaus NC028307 1	0,15076648
Mungos mungo/gambianus SRR7704821 1	Hyaena hyaena NC020669 1	0,15076655
Parahyaena brunnea NC038159 15	Bdeogale nigripes GLC15 1	0,15076922
Salanoia concolor D378 1	Felis nigripes NC028309 1	0,15077087
Smilodon populator MF871700 1	Diplogale hosei MH464790 1	0,15080331
Smilodon populator MF871700 1	Eupleres goudotii D128 1	0,15082301
Urva semitorquata MH464789 1	Catopuma temminckii NC027115 41	0,15083061
Catopuma temminckii NC027115 41	Atilax paludinosus T606 1	0,15083146
Viverra zibetha T609 1	Galidia elegans D146 1	0,15083283
Prionailurus rubiginosus NC028304 2	Eupleres goudotii D128 1	0,15083462
Prionailurus bengalensis CKM45 20	Hyaena hyaena NC020669 1	0,15084702
Otocolobus manul NC028323 1	Diplogale hosei MH464790 1	0,15084861
Lynx rufus NC014456 3	Chrotogale owstoni T607 1	0,15089177
Suricata suricatta SSM10 1	Prionailurus rubiginosus NC028304 2	0,15089905
Puma concolor NC016470 22	Urva semitorquata MH464789 1	0,1508998
Puma yagouaroundi NC028311 1	Mungos mungo/gambianus SRR7704821 1	0,15090007
Paradoxurus jerdoni MH464793 1	Felis catus NC001700 2	0,15090041
Leopardus pardalis NC028315 1	Helogale parvula SRR7637809 1	0,15090139
Profelis aurata NC028299 1	Atilax paludinosus T606 1	0,15090147
Leopardus guigna NC028321 1	Urva semitorquata MH464789 1	0,15090182
Leopardus pardalis T262 1	Galerella sanguinea T378 1	0,15090184
Leopardus wiedii NC028318 1	Galerella sanguinea T378 1	0,15090196
Leopardus jacobita NC028322 1	Atilax paludinosus T606 1	0,15090264
Phoca largha NC008430 1	Arctocephalus forsteri NC004023 28	0,15090384
Mungotictis decemlineata NC027828 1	Felis catus NC001700 2	0,15090483
Parahyaena brunnea NC038159 15	Ichneumia albicauda T603 1	0,15090628
Prionailurus bengalensis NC028301 12	Hyaena hyaena NC020669 1	0,15091212
Eupleres goudotii D128 1	Acinonyx jubatus NC005212 3	0,15091679
Smilodon populator MF871700 1	Mungos mungo MMC7 1	0,15091747
Cynictis penicillata T375 1	Chrotogale owstoni T607 1	0,15094915
Urva brachyura KY117547 1	Genetta servalina NC024568 2	0,15096386
Mungos mungo/gambianus SRR7704821 1	Genetta servalina NC024568 2	0,15096398
Felis margarita NC028308 1	Atilax paludinosus T606 1	0,15096772
Paradoxurus hermaphroditus NLNC 1	Felis silvestris lybica KP202275 4	0,15096787
Viverricula indica KX891751 1	Panthera pardus japonensis KJ866876 8	0,15096915
Leopardus pardalis NC028315 1	Ichneumia albicauda T603 1	0,15096942
Prionailurus viverrinus NC028305 1	Eupleres goudotii D128 1	0,15097164
Salanoia concolor D378 1	Hyaena hyaena NC020669 1	0,15097207
Leopardus pardalis T262 1	Galidia elegans D146 1	0,15097463
Viverricula indica KX891745 1	Panthera tigris amoyensis NC014770 2	0,15097632
Prionailurus planiceps NC028312 6	Cryptoprocta ferox CFC13 1	0,15097691
Prionailurus bengalensis NC028301 12	Urva javanica/auropunctata NC006835 1	0,15099879
Halichoerus grypus NC001602 2	Arctocephalus forsteri NC004023 28	0,1510019
Tremarctos ornatus NC009969 2	Ailuropoda melanoleuca NC009492 5	0,15102033
Urva javanica T413 1	Civettictis civetta GLC19 1	0,15103145
Mungos mungo MMC7 1	Lynx lynx NC027083 4	0,15103269
Nandinia binotata NC024567 1	Galerella sanguinea T378 1	0,15103282
Salanoia concolor D378 1	Panthera leo spelaea KX258452 2	0,15103328
Prionailurus bengalensis NC028301 12	Cynictis penicillata T375 1	0,15103416
Panthera tigris amoyensis NC014770 2	Genetta servalina NC024568 2	0,15104298
Panthera tigris NC010642 35	Civettictis civetta GLC19 1	0,15104339
Felis catus NC001700 2	Cryptoprocta ferox CFC13 1	0,15104355
Fossa fossana D350 1	Arctictis binturong T605 2	0,15104519
Prionailurus bengalensis CKM45 20	Cryptoprocta ferox CFC13 1	0,15104747
Galidia elegans D146 1	Civettictis civetta NC033378 1	0,15106235
Phocarctos hookeri NC008418 1	Monachus monachus NC044972 5	0,15106243
Prionodon linsang ERR2391707 1	Civettictis civetta GLC19 1	0,15109809
Helogale parvula SRR7637809 1	Catopuma temminckii NC027115 41	0,15110064
Paradoxurus jerdoni MH464793 1	Leopardus tigrinus NC028317 1	0,15110064
Paradoxurus jerdoni MH464793 1	Lynx lynx NC027083 4	0,15110157
Prionodon linsang ERR2391707 1	Leopardus geoffroyi NC028320 1	0,15110213
Puma yagouaroundi NC028311 1	Atilax paludinosus T606 1	0,15110321
Leopardus geoffroyi NC028320 1	Helogale parvula SRR7637809 1	0,15110423

Galidia elegans D146 1	Callorhinus ursinus NC008415 1	0,18570124
Vulpes lagopus NC026529 3	Mustela frenata NC020640 1	0,18570128
Panthera onca NC022842 1	Martes flavigula NC012141 3	0,18570133
Mustela putorius NC020638 4	Galidictis fasciata DM333 1	0,1857021
Salanoia concolor D378 1	Bassariscus sumichrasti SRX1099089 1	0,18570215
Salanoia concolor D378 1	Mustela putorius NC020638 4	0,18570228
Procyon lotor AB462046 3	Atilax paludinosus T606 1	0,1857024
Martes pennanti NC020664 16	Chrotogale owstoni T607 1	0,18570268
Salanoia concolor D378 1	Lutrogale perspicillata NC035811 1	0,18570313
Melogale moschata NC020644 1	Xenogale naso C07XAR110 1	0,18570462
Panthera uncia KP202269 1	Lutra lutra LC050126 1	0,18570745
Mustela altaica NC021751 1	Urva javanica T413 1	0,18570775
Viverricula indica NC025296 2	Arctocephalus forsteri KT693377 17	0,18570879
Martes zibellina NC011579 39	Genetta genetta T297 1	0,18570963
Potos flavus T414 1	Panthera pardus japonensis KJ866876	0,18571028
Ursus arctos EU497665 29	Martes pennanti NC020664 16	0,18571036
Ursus arctos pruinosus MG066703 3	Martes pennanti NC020664 16	0,18571042
Mungos mungo MMC7 1	Lycaon pictus NC028427 2	0,18571118
Prionailurus planiceps KY682741 4	Arctocephalus townsendi NC008420 1	0,18571131
Poecilogale albinucha T602 1	Catopuma temminckii NC027115 41	0,18571182
Melogale moschata V0735A 1	Felis catus NC001700 2	0,18571213
Parahyaena brunnea NC038159 15	Melogale moschata KP726273 1	0,18571247
Neophoca cinerea NC008419 1	Lynx lynx NC027083 4	0,18571354
Paguma larvata PDDS11 2	Martes pennanti NC020664 16	0,18571359
Neovison vison NC020641 3	Mungotictis decemlineata NC027828 1	0,18571369
Vulpes zerda KJ603240 1	Prionailurus bengalensis NC028301 12	0,18571398
Martes flavigula NC012141 3	Lycalopex sechurae KT448284 1	0,18571436
Leopardus guigna NC028321 1	Arctocephalus gazella BK010918 1	0,18571545
Panthera tigris NC010642 35	Meles leucurus NC039173 4	0,18571557
Panthera tigris amoyensis NC014770 2	Meles leucurus NC039173 4	0,18571566
Panthera uncia KP202269 1	Bassariscus sumichrasti SRX1099089 1	0,18571727
Viverricula indica KX891751 1	Ursus maritimus NC003428 31	0,18571846
Panthera onca NC022842 1	Arctonyx collaris NC020645 1	0,18571964
Martes pennanti NC020664 16	Felis nigripes NC028309 1	0,1857197
Lycalopex sechurae KT448284 1	Chrotogale owstoni T607 1	0,18572029
Ursus arctos GU573491 207	Otaria byronia OTAB 1	0,18572082
Speothos venaticus C48 2	Leopardus pardalis T262 1	0,18572375
Prionailurus viverrinus NC028305 1	Otaria byronia OTAB 1	0,18572438
Viverricula indica KX891751 1	Ursus arctos GU573486 5	0,18572496
Viverricula indica KX891751 1	Ursus arctos pruinosus MG066703 3	0,18572518
Arctocephalus forsteri KT693377 17	Acinonyx jubatus NC005212 3	0,18572806
Ursus arctos EU497665 29	Leopardus geoffroyi NC028320 1	0,18572831
Ursus thibetanus thibetanus NC01111	Canis anthus NC027956 2	0,1857288
Lycaon pictus NC028427 2	Leopardus guigna NC028321 1	0,18573041
Martes americana NC020642 1	Canis lupus familiaris NC002008 1231	0,18573359
Viverra zibetha T609 1	Arctocephalus forsteri NC004023 28	0,18573412
Ursus thibetanus formosanus NC0093	Prionailurus planiceps NC028312 6	0,18573497
Panthera onca KP202264 2	Canis aureus KT448274 1	0,18573514
Melursus ursinus NC009970 2	Lynx lynx NC027083 4	0,18573558
Ursus thibetanus thibetanus NC01111	Felis nigripes NC028309 1	0,18573676
Urva semitorquata MH464789 1	Gulo gulo NC009685 3	0,18573718
Lynx canadensis NC028313 1	Chrysocyon brachyurus NC024172 1	0,18573742
Ursus thibetanus laniger MH281753 2	Prionailurus rubiginosus NC028304 2	0,18573762
Ursus thibetanus thibetanus NC01111	Prionailurus planiceps NC028312 6	0,18573773
Prionailurus bengalensis NC028301 12	Helarctos malayanus NC009968 2	0,18573879
Ursus spelaeus NC011112 8	Leptailurus serval NC028316 1	0,18573898
Zalophus californianus NC008416 1	Viverricula indica KX891751 1	0,18574014
Panthera uncia KP202269 1	Canis lupus chanco NC010340 4	0,18574123
Zalophus californianus NC008416 1	Puma concolor NC016470 22	0,18574397
Panthera leo NERO 19	Mustela nigripes NC024942 1	0,18574554
Lycalopex sechurae KT448284 1	Leopardus tigrinus NC028317 1	0,18574603
Mustela sibirica AP017394 11	Cuon alpinus NC013445 3	0,18574636
Zalophus californianus NC008416 1	Parahyaena brunnea NC038159 15	0,18574767
Prionodon linsang ERR2391707 1	Lycalopex sechurae KT448284 1	0,18575047
Mustela sibirica AP017394 11	Lycalopex sechurae KT448284 1	0,18575294
Melogale moschata KP726273 1	Atilax paludinosus T606 1	0,18576354
Poecilogale albinucha T602 1	Nasua nasua NC020647 1	0,18576431
Viverra tangalunga MH464792 1	Mungos mungo/gambianus SRR77048	0,18576494
Mustela sibirica AP017394 11	Atilax paludinosus T606 1	0,18576497
Meles anakuma NC009677 1	Cynogale bennetti KY117544 1	0,18576504
Mustela nigripes NC024942 1	Atilax paludinosus T606 1	0,1857655
Potos flavus T414 1	Xenogale naso C07XAR110 1	0,18576726
Crossarchus platycephalus C7R66 1	Conepatus chinga NC042596 1	0,18576822
Lutra lutra NC011358 9	Genetta genetta T297 1	0,18576941
Viverricula indica KX891745 1	Callorhinus ursinus NC008415 1	0,18576972
Paradoxurus jerdoni MH464793 1	Arctocephalus australis MG023139 1	0,18576974
Vulpes vulpes NC008434 5	Mustela nigripes NC024942 1	0,18577009
Neophoca cinerea NC008419 1	Lutrogale perspicillata NC035811 1	0,18577096
Lynx canadensis NC028313 1	Ailurus fulgens styani NC009691 1	0,18577272
Martes melampus NC009678 1	Genetta genetta T297 1	0,18577352
Cynictis penicillata T375 1	Arctotherium sp NC030174 1	0,18577413
Genetta servalina NC024568 2	Arctocephalus gazella BK010918 1	0,18577459
Gulo gulo NC009685 3	Canis lupus familiaris NC002008 1231	0,18577529
Panthera onca KP202264 2	Enhydra lutris NC009692 1	0,18577543
Mustela nivalis T306 5	Cynictis penicillata T375 1	0,18577668
Ursus arctos EU497665 29	Arctocephalus australis MG023139 1	0,18577738
Poecilogale albinucha T602 1	Fossa fossana D350 1	0,18577787
Vulpes lagopus NC026529 3	Lynx lynx NC027083 4	0,18577945
Vulpes lagopus NC026529 3	Prionailurus planiceps NC028312 6	0,18577988
Ommatophoca rossii AY377287etc 1	Galidia elegans D146 1	0,18578051
Panthera onca KP202264 2	Canis latrans NC008093 7	0,18578057
Viverricula indica NC025296 2	Urocyon littoralis catalinae KP129018 1	0,18578085
Leopardus guigna NC028321 1	Ailurus fulgens NC011124 1	0,18578124
Tremarctos ornatus NC009969 2	Felis catus NC001700 2	0,18578175
Proteles cristata T393 6	Ommatophoca rossii AY377287etc 1	0,18578225
Nyctereutes procyonoides NC013700 3	Neovison vison NC020641 3	0,18578255
Prionailurus viverrinus NC028305 1	Lontra canadensis SRR10409165 1	0,18578286
Phocarctos hookeri NC008418 1	Leopardus wiedii NC028318 1	0,18578349
Vulpes corsac NC023958 1	Leopardus geoffroyi NC028320 1	0,18578357
Vulpes vulpes NC008434 5	Leopardus guigna NC028321 1	0,18578368
Procyon lotor AB462046 3	Diplogale hosei MH464790 1	0,18578383
Procyon lotor AB462049 4	Paradoxurus jerdoni MH464793 1	0,18578456
Prionodon pardicolor NC024569 2	Martes pennanti NC020664 16	0,185785

Proteles cristata T393 6	Helogale parvula SRR7637809 1	0,15110666
Prionailurus rubiginosus NC028304 2	Galidia elegans D146 1	0,15110745
Viverricula indica KX891751 1	Panthera tigris NC010642 35	0,15110974
Suricata suricatta SSM10 1	Civettictis civetta NC033378 1	0,15113102
Phoca groenlandica NC008429 54	Arctocephalus townsendi NC008420 1	0,15116215
Viverricula indica KX891751 1	Urva semitorquata MH464789 1	0,15116516
Viverricula indica KX891745 1	Urva brachyura KY117547 1	0,15116534
Viverricula indica NC025296 2	Suricata suricatta SSM10 1	0,15116973
Eupleres goudotii D128 1	Crocuta crocuta NC020670 3	0,15117538
Prionodon pardicolor NC024569 2	Paradoxurus hermaphroditus NLNC 1	0,15117742
Parahyaena brunnea NC038159 15	Leopardus pardalis T262 1	0,15118858
Urva javanica/auropunctata NC006835 1	Civettictis civetta GLC19 1	0,15120243
Paradoxurus hermaphroditus NC039591 1	Cynictis penicillata T375 1	0,15123203
Mungos mungo MMC7 1	Lynx rufus NC014456 3	0,15123516
Leptailurus serval NC028316 1	Urva brachyura KY117547 1	0,1512356
Leopardus pardalis T262 1	Cynictis penicillata T375 1	0,15123627
Cynictis penicillata T375 1	Catopuma badia NC028300 1	0,15123665
Paradoxurus hermaphroditus NC039591 1	Felis catus NC001700 2	0,15123679
Panthera pardus japonensis KJ866876 8	Fossa fossana D350 1	0,15123709
Leopardus geoffroyi NC028320 1	Atilax paludinosus T606 1	0,15123908
Proteles cristata T393 6	Mungotictis decemlineata NC027828 1	0,15123956
Leopardus guigna NC028321 1	Eupleres goudotii D128 1	0,15123993
Salanoia concolor D378 1	Proteles cristata T393 6	0,15124001
Galidictis fasciata DM333 1	Felis margarita NC028308 1	0,15124249
Paguma larvata PDDS11 2	Ichneumia albicauda T603 1	0,15124613
Profelis aurata NC028299 1	Cryptoprocta ferox CFC13 1	0,15124615
Prionodon pardicolor NC024569 2	Pardofelis marmorata NLN3 2	0,15124868
Mungos mungo MMC7 1	Acinonyx jubatus NC005212 3	0,15125005
Prionailurus viverrinus NC028305 1	Parahyaena brunnea NC038159 15	0,15125585
Panthera leo NERO 19	Fossa fossana D350 1	0,15127249
Ichneumia albicauda T603 1	Civettictis civetta NC033378 1	0,15127647
Viverricula indica KX891751 1	Atilax paludinosus T606 1	0,15129977
Viverra zibetha T609 1	Mungos mungo/gambianus SRR7704821 1	0,15130037
Mungos mungo MMC7 1	Felis margarita NC028308 1	0,1513027
Phocarctos hookeri NC008418 1	Monachus schauinslandi NC008421 1	0,15130304
Felis catus NC001700 2	Crossarchus platycephalus C7R66 1	0,15130311
Felis catus NC001700 2	Atilax paludinosus T606 1	0,15130401
Paradoxurus jerdoni MH464793 1	Lynx rufus NC014456 3	0,15130411
Paradoxurus hermaphroditus NLNC 1	Felis catus NC001700 2	0,15130448
Profelis aurata NC028299 1	Paradoxurus jerdoni MH464793 1	0,15130518
Leopardus geoffroyi NC028320 1	Crossarchus platycephalus C7R66 1	0,15130542
Prionailurus rubiginosus NC028304 2	Ichneumia albicauda T603 1	0,15130574
Ichneumia albicauda T603 1	Catopuma badia NC028300 1	0,151306
Puma concolor NC016470 22	Mungotictis decemlineata NC027828 1	0,15130955
Parahyaena brunnea NC038159 15	Lynx pardinus NC028319 161	0,15131889
Leopardus colocolo NC028314 1	Urva javanica T413 1	0,15132035
Prionodon linsang ERR2391707 1	Civettictis civetta NC033378 1	0,15133108
Parahyaena brunnea NC038159 15	Urva javanica/auropunctata NC006835 1	0,15134141
Diplogale hosei MH464790 1	Caracal caracal NC028306 1	0,15136829
Urva javanica/auropunctata NC006835 1	Civettictis civetta NC033378 1	0,15136963
Urva semitorquata MH464789 1	Felis catus NC001700 2	0,15137063
Lynx rufus NC014456 3	Ichneumia albicauda T603 1	0,15137154
Panthera tigris NC010642 35	Genetta abyssinica MG489822 1	0,15137173
Viverricula indica KX891751 1	Salanoia concolor D378 1	0,15137209
Paradoxurus hermaphroditus NC039591 1	Felis chaus NC028307 1	0,15137228
Paradoxurus hermaphroditus NC039591 1	Leopardus geoffroyi NC028320 1	0,1513793
Profelis aurata NC028299 1	Prionodon pardicolor NC024569 2	0,15138394
Ursus thibetanus thibetanus NC011118 4	Ailuropoda melanoleuca NC009492 5	0,15138851
Viverricula indica KX891751 1	Panthera pardus NC010641 5	0,15140407
Panthera pardus NC010641 5	Mungotictis decemlineata NC027828 1	0,15141013
Lynx lynx NC027083 4	Helogale parvula SRR7637809 1	0,15143685
Leopardus pardalis NC028315 1	Cynictis penicillata T375 1	0,15143809
Felis margarita NC028308 1	Eupleres goudotii D128 1	0,15144001
Leopardus tigrinus NC028317 1	Urva semitorquata MH464789 1	0,1514408
Leopardus jacobita NC028322 1	Eupleres goudotii D128 1	0,15144368
Mungotictis decemlineata NC027828 1	Felis margarita NC028308 1	0,15144391
Cynictis penicillata T375 1	Arctictis binturong T605 2	0,15144825
Mungos mungo/gambianus SRR7704821 1	Homotherium latidens MF871702 3	0,15146597
Suricata suricatta SSM10 1	Genetta abyssinica MG489822 1	0,15147297
Paradoxurus jerdoni MH464793 1	Mungos mungo MMC7 1	0,15149957
Prionailurus planiceps NC028312 6	Mungos mungo/gambianus SRR7704821 1	0,15150563
Paradoxurus hermaphroditus NC039591 1	Lynx pardinus NC028319 161	0,15150569
Panthera uncia KP202269 1	Genetta abyssinica MG489822 1	0,15150616
Puma concolor NC016470 22	Paradoxurus hermaphroditus NC039591 1	0,15150642
Leopardus pardalis NC028315 1	Galerella sanguinea T378 1	0,15150774
Viverricula indica KX891751 1	Galerella sanguinea T378 1	0,15150943
Lynx rufus NC014456 3	Galidia elegans D146 1	0,15150987
Mungotictis decemlineata NC027828 1	Caracal caracal NC028306 1	0,15151222
Phoca vitulina NC001325 1	Arctocephalus forsteri NC004023 28	0,15151265
Leopardus jacobita NC028322 1	Galidia elegans D146 1	0,15151304
Otocolobus manul NC028323 1	Galidictis fasciata DM333 1	0,15151323
Viverra zibetha T609 1	Ichneumia albicauda T603 1	0,1515155
Prionodon linsang ERR2391707 1	Homotherium latidens MF871702 3	0,15151794
Leopardus colocolo NC028314 1	Galerella sanguinea T378 1	0,15152404
Leopardus colocolo NC028314 1	Helogale parvula SRR7637809 1	0,15152453
Leopardus colocolo NC028314 1	Atilax paludinosus T606 1	0,1515248
Urva javanica T413 1	Civettictis civetta NC033378 1	0,151535
Panthera leo NERO 19	Mungotictis decemlineata NC027828 1	0,15154402
Pardofelis marmorata NLN3 2	Chrotogale owstoni T607 1	0,15156542
Paradoxurus hermaphroditus NC039591 1	Leopardus tigrinus NC028317 1	0,15157142
Lynx lynx NC027083 4	Urva semitorquata MH464789 1	0,15157163
Genetta servalina NC024568 2	Cryptoprocta ferox CFC13 1	0,15157229
Prionailurus bengalensis NC028301 12	Urva javanica T413 1	0,15157261
Leptailurus serval NC028316 1	Urva javanica T413 1	0,15157281
Leopardus wiedii NC028318 1	Urva javanica T413 1	0,15157303
Galidia elegans D146 1	Civettictis civetta GLC19 1	0,15157305
Leptailurus serval NC028316 1	Cynictis penicillata T375 1	0,15157305
Paradoxurus hermaphroditus NC039591 1	Felis nigripes NC028309 1	0,1515737
Mungotictis decemlineata NC027828 1	Crocuta crocuta NC020670 3	0,15157887
Prionodon linsang ERR2391707 1	Arctictis binturong T605 2	0,15158062
Viverricula indica KX891751 1	Panthera tigris amoyensis NC014770 2	0,15158141
Parahyaena brunnea NC038159 15	Leopardus wiedii NC028318 1	0,15159249
Ommatophoca rossii AY377287etc 1	Arctocephalus gazella BK010918 1	0,1516294

Prionailurus planiceps NC028312 6	Arctocephalus pusillus NC008417 1	0,18578508
Galictis vittata T412 1	Conepatus chinga NC042596 1	0,18578571
Martes martes T302 3	Lycaon pictus NC028427 2	0,18578631
Leopardus pardalis T262 1	Arctotherium sp NC030174 1	0,18578698
Felis silvestris lybica KP202275 4	Arctotherium sp NC030174 1	0,18578714
Paradoxurus hermaphroditus NC03959	Mustela nivalis T306 5	0,18578722
Prionailurus bengalensis NC028301 12	Arctotherium sp NC030174 1	0,18578762
Caracal caracal NC028306 1	Arctotherium sp NC030174 1	0,18578772
Puma concolor NC016470 22	Otocyon megalotis SAF1 2	0,18578847
Ursus arctos AP012576 6	Fossa fossana D350 1	0,18578885
Ursus thibetanus mupinensis NC00875	Ichneumia albicauda T603 1	0,18578905
Monachus monachus NC044972 5	Tapirus terrestris T358	0,18578917
Melursus ursinus NC009970 2	Fossa fossana D350 1	0,18578982
Ursus spelaeus NC011112 8	Lutrogale perspicillata NC035811 1	0,18579096
Cuon alpinus NC013445 3	Civettictis civetta NC033378 1	0,18579304
Ursus thibetanus laniger MH281753 2	Chrotogale owstoni T607 1	0,18579373
Galictis vittata T412 1	Arctocephalus pusillus NC008417 1	0,1857941
Panthera uncia KP202269 1	Ictonyx striatus T299 1	0,1857942
Speothos venaticus C48 2	Neovison vison NC020641 3	0,18579435
Ursus maritimus NC003428 31	Catopuma temminckii NC027115 41	0,18579492
Ursus thibetanus laniger MH281753 2	Procyon lotor AB462049 4	0,18579559
Mungos mungo MMC7 1	Canis mesomelas KT448280 1	0,1857958
Ictonyx striatus T299 1	Arctotherium sp NC030174 1	0,18579592
Canis lupus chanco NC010340 4	Bdeogale nigripes GLC15 1	0,18579608
Helarctos malayanus NC009968 2	Canis latrans NC008093 7	0,18579629
Mustela eversmannii NC028013 1	Ailuropoda melanoleuca NC009492 5	0,18579692
Ursus thibetanus thibetanus NC01111	Procyon lotor AB462046 3	0,18579701
Panthera onca NC022842 1	Neovison vison NC020641 3	0,18579721
Diplogale hosei MH464790 1	Canis lupus familiaris NC002008 1231	0,18579844
Panthera onca NC022842 1	Mustela nivalis T306 5	0,1857986
Ursus thibetanus laniger MH281753 2	Ichneumia albicauda T603 1	0,18579951
Diplogale hosei MH464790 1	Canis aureus KT448274 1	0,18580264
Mustela nigripes NC024942 1	Urva javanica/auropunctata NC006835	0,18580411
Profelis aurata NC028299 1	Helarctos malayanus NC009968 2	0,18580686
Prionailurus bengalensis CKM45 20	Melursus ursinus NC009970 2	0,18580994
Viverricula indica KX891745 1	Gulo gulo NC009685 3	0,1858117
Homotherium latidens MF871702 3	Arctocephalus gazella BK010918 1	0,18581207
Leopardus jacobita NC028322 1	Canis lupus familiaris NC002008 1231	0,18581349
Ursus arctos pruinosus MG066703 3	Genetta abyssinica MG489822 1	0,18581358
Homotherium latidens MF871702 3	Bassaricyon neblina SRX1097850 1	0,18581468
Cynictis penicillata T375 1	Canis mesomelas KT448280 1	0,18582124
Prionailurus viverrinus NC028305 1	Canis lupus chanco NC010340 4	0,18582378
Lutra lutra NC011358 9	Urva javanica T413 1	0,18582478
Eumetopias jubatus NC004030 10	Cryptoprocta ferox CFC13 1	0,18582582
Urva javanica T413 1	Callorhinus ursinus NC008415 1	0,18582961
Mustela sibirica NC020637 6	Canis mesomelas KT448280 1	0,18582991
Pusa hispida NC 008433 1	Tapirus terrestris T358	0,18583241
Phocarctos hookeri NC008418 1	Mustela eversmannii NC028013 1	0,18583269
Mustela sibirica NC020637 6	Xenogale naso C07XAR110 1	0,1858328
Bassaricyon neblina SRX1097850 1	Arctocephalus australis MG023139 1	0,18583542
Mustela sibirica AP017394 11	Galidictis fasciata DM333 1	0,18583669
Vulpes corsac NC023958 1	Mustela sibirica AP017394 11	0,18583713
Salanoia concolor D378 1	Mustela nigripes NC024942 1	0,18583716
Vulpes ferrilata NC027935 1	Mungotictis decemlineata NC027828 1	0,1858377
Otaria byronia OTAB 1	Bassaricyon neblina SRX1097850 1	0,18583778
Leopardus colocolo NC028314 1	Canis mesomelas KT448280 1	0,18583797
Mungotictis decemlineata NC027828 1	Canis latrans NC008093 7	0,18583862
Melogale moschata NC020644 1	Atilax paludinosus T606 1	0,18583877
Chrotogale owstoni T607 1	Arctotherium sp NC030174 1	0,1858391
Martes americana NC020642 1	Galerella sanguinea T378 1	0,18584086
Melursus ursinus NC009970 2	Gulo gulo NC009685 3	0,18584116
Prionodon linsang ERR2391707 1	Poecilogale albinucha T602 1	0,18584128
Viverra zibetha T609 1	Lontra canadensis SRR10409165 1	0,18584175
Prionailurus planiceps NC028312 6	Ailurus fulgens styani NC009691 1	0,18584269
Viverricula indica NC025296 2	Melogale moschata V0735A 1	0,18584429
Vulpes zerda KJ603240 1	Viverricula indica KX891751 1	0,18584454
Vulpes lagopus NC026529 3	Prionodon pardicolor NC024569 2	0,18584573
Panthera onca KP202264 2	Melogale moschata KP726273 1	0,18584579
Cryptoprocta ferox CFC13 1	Arctocephalus pusillus NC008417 1	0,18584746
Nyctereutes procyonoides NC013700 3	Genetta servalina NC024568 2	0,18584746
Caracal caracal NC028306 1	Arctocephalus gazella BK010918 1	0,18584754
Mephitis mephitis NC020648 1	Callorhinus ursinus NC008415 1	0,18584783
Panthera uncia KP202269 1	Canis latrans NC008093 7	0,18584785
Ursus thibetanus formosanus NC00933	Conepatus chinga NC042596 1	0,18584795
Ursus thibetanus mupinensis NC00875	Phocarctos hookeri NC008418 1	0,18584816
Lontra canadensis SRR10409165 1	Felis silvestris lybica KP202275 4	0,18584883
Vulpes zerda KJ603240 1	Catopuma badia NC028300 1	0,18584901
Prionailurus planiceps NC028312 6	Phocarctos hookeri NC008418 1	0,18584976
Vulpes zerda KJ603240 1	Leopardus geoffroyi NC028320 1	0,1858504
Melogale moschata KP726273 1	Chrysocyon brachyurus NC024172 1	0,18585059
Ichneumia albicauda T603 1	Arctocephalus pusillus NC008417 1	0,18585065
Poecilogale albinucha T602 1	Felis catus NC001700 2	0,18585283
Martes pennanti NC020664 16	Felis catus NC001700 2	0,18585405
Martes pennanti NC020664 16	Felis margarita NC028308 1	0,18585436
Viverra zibetha T609 1	Otaria byronia OTAB 1	0,18585621
Ursus spelaeus NC011112 8	Lutra lutra NC011358 9	0,18585666
Procyon lotor AB462049 4	Leopardus guigna NC028321 1	0,1858568
Panthera onca NC022842 1	Melogale moschata V0735A 1	0,18585695
Martes pennanti NC020664 16	Lycaon pictus NC028427 2	0,18585753
Ursus thibetanus formosanus NC00933	Phocarctos hookeri NC008418 1	0,18585876
Mustela nivalis T306 5	Lycaon pictus NC028427 2	0,18585928
Salanoia concolor D378 1	Canis aureus KT448274 1	0,18585943
Ursus arctos AP012576 6	Martes melampus NC009678 1	0,18585952
Ursus arctos isabellinus 18B5 2	Prionailurus bengalensis NC028301 12	0,18585968
Canis aureus KT448274 1	Ailurus fulgens styani NC009691 1	0,18586142
Ursus thibetanus mupinensis NC00875	Otocolobus manul NC028323 1	0,18586256
Ursus arctos isabellinus 18B5 2	Prionailurus bengalensis CKM45 20	0,18586257
Viverra tangalunga MH464792 1	Melursus ursinus NC009970 2	0,18586352
Arctotherium sp NC030174 1	Arctictis binturong T605 2	0,18586512
Ursus spelaeus EU327344 13	Felis catus NC001700 2	0,18586682
Ursus thibetanus thibetanus NC01111	Ichneumia albicauda T603 1	0,1858685
Ursus arctos GU573491 207	Arctocephalus forsteri NC004023 28	0,1858694
Ursus arctos AP012576 6	Aonyx cinerea NC035814 2	0,18586949

Paradoxurus hermaphroditus NC039591 1	Leopardus jacobita NC028322 1	0,15163875
Viverricula indica KX891751 1	Panthera leo spelaea KX258452 2	0,15164042
Otocolobus manul NC028323 1	Eupleres goudotii D128 1	0,15164278
Mungotictis decemlineata NC027828 1	Leptailurus serval NC028316 1	0,15164724
Felis nigripes NC028309 1	Cryptoprocta ferox CFC13 1	0,15164973
Crossarchus platycephalus C7R66 1	Acinonyx jubatus NC005212 3	0,15165603
Cryptoprocta ferox CFC13 1	Acinonyx jubatus NC005212 3	0,15166578
Prionodon linsang ERR2391707 1	Nandinia binotata NC024567 1	0,15169674
Prionodon linsang ERR2391707 1	Felis nigripes NC028309 1	0,15170331
Paguma larvata PDDS11 2	Mungos mungo/gambianus SRR7704821 1	0,15170377
Pardofelis marmorata NLN3 2	Atilax paludinosus T606 1	0,15170807
Puma yagouaroundi NC028311 1	Urva semitorquata MH464789 1	0,15170844
Mungos mungo/gambianus SRR7704821 1	Leopardus jacobita NC028322 1	0,15170968
Mungos mungo MMC7 1	Hyaena hyaena NC020669 1	0,15171145
Galidictis fasciata DM333 1	Felis catus NC001700 2	0,15171333
Prionailurus rubiginosus NC028304 2	Hyaena hyaena NC020669 1	0,15171953
Ommatophoca rossii AY377287etc 1	Arctocephalus pusillus NC008417 1	0,15171996
Lynx pardinus NC028319 161	Hyaena hyaena NC020669 1	0,15172009
Parahyaena brunnea NC038159 15	Lynx rufus NC014456 3	0,15172298
Prionodon pardicolor NC024569 2	Genetta abyssinica MG489822 1	0,15174761
Panthera leo NERO 19	Arctictis binturong T605 2	0,15175645
Lynx canadensis NC028313 1	Chrotogale owstoni T607 1	0,151767
Prionodon linsang ERR2391707 1	Prionailurus viverrinus NC028305 1	0,15176988
Paguma larvata PDDS11 2	Cynictis penicillata T375 1	0,15177086
Mungos mungo/gambianus SRR7704821 1	Lynx pardinus NC028319 161	0,15177388
Mungos mungo MMC7 1	Lynx pardinus NC028319 161	0,1517739
Prionailurus bengalensis NC028301 12	Urva brachyura KY117547 1	0,15177399
Helogale parvula SRR7637809 1	Catopuma badia NC028300 1	0,15177453
Urva semitorquata MH464789 1	Felis nigripes NC028309 1	0,15177482
Leopardus wiedii NC028318 1	Xenogale naso C07XAR110 1	0,15177558
Otocolobus manul NC028323 1	Atilax paludinosus T606 1	0,15177695
Leopardus geoffroyi NC028320 1	Urva javanica T413 1	0,15177727
Pardofelis marmorata NLN3 2	Mungotictis decemlineata NC027828 1	0,15178029
Leopardus tigrinus NC028317 1	Eupleres goudotii D128 1	0,15178053
Salanoia concolor D378 1	Crocuta crocuta NC020670 3	0,1517809
Puma concolor NC016470 22	Galidictis fasciata DM333 1	0,15178142
Panthera tigris NC010642 35	Genetta genetta T297 1	0,15178537
Diplogale hosei MH464790 1	Catopuma temminckii NC027115 41	0,15179057
Leopardus colocolo NC028314 1	Galidia elegans D146 1	0,15179519
Prionailurus rubiginosus NC028304 2	Urva javanica/auropunctata NC006835 1	0,15180619
Parahyaena brunnea NC038159 15	Leopardus colocolo NC028314 1	0,15181008
Urva brachyura KY117547 1	Civettictis civetta GLC19 1	0,15183951
Prionailurus rubiginosus NC028304 2	Urva brachyura KY117547 1	0,15184118
Mungos mungo/gambianus SRR7704821 1	Catopuma badia NC028300 1	0,15184185
Paradoxurus hermaphroditus NLNC 1	Lynx lynx NC027083 4	0,15184227
Prionodon linsang ERR2391707 1	Leopardus guigna NC028321 1	0,15184289
Viverricula indica KX891751 1	Galidictis fasciata DM333 1	0,15184379
Leopardus geoffroyi NC028320 1	Cynictis penicillata T375 1	0,15184462
Leopardus geoffroyi NC028320 1	Ichneumia albicauda T603 1	0,15184684
Panthera pardus japonensis KJ866876 8	Galidictis fasciata DM333 1	0,15184711
Prionailurus rubiginosus NC028304 2	Cryptoprocta ferox CFC13 1	0,15185253
Panthera onca NC022842 1	Genetta genetta T297 1	0,15185457
Salanoia concolor D378 1	Homotherium latidens MF871702 3	0,15185921
Puma yagouaroundi NC028311 1	Parahyaena brunnea NC038159 15	0,15185964
Prionailurus bengalensis NC028301 12	Cynogale bennettii KY117544 1	0,15188162
Viverricula indica KX891751 1	Xenogale naso C07XAR110 1	0,1519056
Viverricula indica KX891745 1	Helogale parvula SRR7637809 1	0,15190615
Panthera leo spelaea KX258452 2	Mungotictis decemlineata NC027828 1	0,15190777
Lynx lynx NC027083 4	Bdeogale nigripes GLC15 1	0,15190823
Leopardus pardalis T262 1	Urva brachyura KY117547 1	0,15190902
Pardofelis marmorata NLN3 2	Urva semitorquata MH464789 1	0,15190935
Prionailurus planiceps KY682741 4	Mungos mungo/gambianus SRR7704821 1	0,1519096
Leopardus wiedii NC028318 1	Cynictis penicillata T375 1	0,15190968
Prionailurus bengalensis NC028301 12	Galerella sanguinea T378 1	0,15191105
Prionailurus planiceps KY682741 4	Cryptoprocta ferox CFC13 1	0,15191957
Puma yagouaroundi NC028311 1	Cryptoprocta ferox CFC13 1	0,15191993
Leopardus colocolo NC028314 1	Cynictis penicillata T375 1	0,15192629
Smilodon populator MF871700 1	Cynictis penicillata T375 1	0,15193202
Paguma larvata PDDS11 2	Crossarchus platycephalus C7R66 1	0,15197323
Viverra zibetha T609 1	Bdeogale nigripes GLC15 1	0,15197343
Crossarchus platycephalus C7R66 1	Catopuma temminckii NC027115 41	0,15197422
Prionailurus viverrinus NC028305 1	Paradoxurus hermaphroditus NC039591 1	0,15197612
Lynx lynx NC027083 4	Atilax paludinosus T606 1	0,1519763
Leopardus pardalis T262 1	Xenogale naso C07XAR110 1	0,15197721
Prionailurus viverrinus NC028305 1	Urva javanica T413 1	0,15197773
Pardofelis marmorata NLN3 2	Paradoxurus hermaphroditus NLNC 1	0,15197799
Panthera tigris amoyensis NC014770 2	Genetta abyssinica MG489822 1	0,15197828
Hyaena hyaena NC020669 1	Galerella sanguinea T378 1	0,15197866
Hyaena hyaena NC020669 1	Galidia elegans D146 1	0,15198047
Lynx lynx NC027083 4	Galidia elegans D146 1	0,15198053
Prionailurus bengalensis CKM45 20	Bdeogale nigripes GLC15 1	0,15198117
Prionailurus rubiginosus NC028304 2	Parahyaena brunnea NC038159 15	0,15199402
Lynx pardinus NC028319 161	Diplogale hosei MH464790 1	0,15203145
Ommatophoca rossii AY377287etc 1	Arctocephalus forsteri NC004023 28	0,15203856
Mungos mungo/gambianus SRR7704821 1	Genetta genetta T297 1	0,15204257
Mungos mungo MMC7 1	Catopuma temminckii NC027115 41	0,15204273
Cynictis penicillata T375 1	Caracal caracal NC028306 1	0,1520444
Galerella sanguinea T378 1	Catopuma badia NC028300 1	0,15204492
Prionailurus viverrinus NC028305 1	Mungos mungo MMC7 1	0,15204617
Galidia elegans D146 1	Felis silvestris lybica KP202275 4	0,15204917
Paguma larvata PDDS11 2	Galerella sanguinea T378 1	0,15205242
Ursus thibetanus mupinensis NC008753 2	Ailuropoda melanoleuca NC009492 5	0,15205458
Helarctos malayanus NC009968 2	Ailuropoda melanoleuca NC009492 5	0,15206185
Urva javanica/auropunctata NC006835 1	Arctictis binturong T605 2	0,15208726
Urocyon cinereoargenteus NC026723 21	Lycaon pictus NC028427 2	0,15209682
Paradoxurus jerdoni MH464793 1	Fossa fossana D350 1	0,15210666
Genetta genetta T297 1	Atilax paludinosus T606 1	0,1521094
Lynx canadensis NC028313 1	Urva semitorquata MH464789 1	0,15211049
Prionailurus planiceps NC028312 6	Mungos mungo MMC7 1	0,1521112
Leopardus pardalis NC028315 1	Urva javanica T413 1	0,15211143
Urva javanica T413 1	Catopuma badia NC028300 1	0,15211211
Suricata suricatta SSM10 1	Leopardus pardalis T262 1	0,15211223
Leptailurus serval NC028316 1	Eupleres goudotii D128 1	0,152114

Ursus thibetanus formosanus NC00933	Felis nigripes NC028309 1	0,1858696
Ursus thibetanus thibetanus NC011118	Panthera pardus japonensis KJ866876	0,18587025
Neophoca cinerea NC008419 1	Leopardus colocolo NC028314 1	0,1858703
Puma concolor NC016470 22	Galictis vittata T412 1	0,18587089
Leptailurus serval NC028316 1	Galictis vittata T412 1	0,18587144
Prionailurus planiceps KY682741 4	Melursus ursinus NC009970 2	0,18587323
Zalophus californianus NC008416 1	Cryptoprocta ferox CFC13 1	0,18587331
Ursus thibetanus thibetanus NC011118	Otocolobus manul NC028323 1	0,18587372
Prionailurus viverrinus NC028305 1	Melursus ursinus NC009970 2	0,18587397
Urva brachyura KY117547 1	Eumetopias jubatus NC004030 10	0,18587881
Ursus americanus JX196366 3	Phocarctos hookeri NC008418 1	0,18588173
Lycalopex sechurae KT448284 1	Leopardus geoffroyi NC028320 1	0,18588406
Zalophus californianus NC008416 1	Panthera tigris NC010642 35	0,18588465
Viverra tangalunga MH464792 1	Lycalopex sechurae KT448284 1	0,18588947
Mustela eversmannii NC028013 1	Lycalopex sechurae KT448284 1	0,18588983
Melursus ursinus NC009970 2	Leopardus colocolo NC028314 1	0,18589164
Ursus arctos GU573491 207	Civettictis civetta NC033378 1	0,18589313
Paradoxurus hermaphroditus NLNC 1	Eumetopias jubatus NC004030 10	0,18589321
Prionailurus rubiginosus NC028304 2	Cuon alpinus NC013445 3	0,18589745
Meles leucurus NC039173 4	Urva brachyura KY117547 1	0,18589783
Vulpes corsac NC023958 1	Genetta abyssinica MG489822 1	0,18589872
Mustela putorius NC020638 4	Urva javanica T413 1	0,18590066
Neophoca cinerea NC008419 1	Mungos mungo/gambianus SRR77048	0,18590188
Leopardus geoffroyi NC028320 1	Cuon alpinus NC013445 3	0,18590248
Meles anakuma NC009677 1	Galidictis fasciata DM333 1	0,18590304
Salanoia concolor D378 1	Nyctereutes procyonoides NC013700 3	0,18590473
Mungos mungo MMC7 1	Melogale moschata NC020644 1	0,18590477
Atilax paludinosus T606 1	Arctocephalus pusillus NC008417 1	0,18590498
Nyctereutes procyonoides NC013700 3	Mustela nigripes NC024942 1	0,18590556
Vulpes ferrilata NC027935 1	Smilodon populator MF871700 1	0,18590613
Mustela erminea T305 2	Cynogale bennetti KY117544 1	0,18590625
Lutrogale perspicillata NC035811 1	Arctocephalus australis MG023139 1	0,18590626
Vulpes ferrilata NC027935 1	Mustela erminea T305 2	0,18590716
Chrotogale owstoni T607 1	Canis anthus NC027956 2	0,18590774
Leopardus pardalis T262 1	Ailurus fulgens NC011124 1	0,1859083
Mustela sibirica NC020637 6	Galerella sanguinea T378 1	0,1859087
Felis margarita NC028308 1	Conepatus chinga NC042596 1	0,18590961
Gulo gulo NC009685 3	Ailuropoda melanoleuca NC009492 5	0,18591012
Mustela nivalis T306 5	Xenogale naso C07XAR110 1	0,18591034
Suricata suricatta SSM10 1	Neovison vison NC020641 3	0,18591061
Viverricula indica NC025296 2	Arctocephalus australis MG023139 1	0,1859107
Vulpes zerda KJ603240 1	Martes martes T302 3	0,18591075
Vulpes vulpes NC008434 5	Martes martes T302 3	0,18591115
Mustela sibirica NC020637 6	Hyaena hyaena NC020669 1	0,18591163
Helogale parvula SRR7637809 1	Arctodus simus NC011116 1	0,18591215
Leptailurus serval NC028316 1	Conepatus chinga NC042596 1	0,18591224
Panthera onca KP202264 2	Mustela sibirica AP017394 11	0,18591265
Paradoxurus hermaphroditus NC03959	Mustela altaica NC021751 1	0,18591291
Suricata suricatta SSM10 1	Martes foina NC020643 1	0,1859131
Pardofelis marmorata NLN3 2	Melogale moschata KP726273 1	0,18591318
Panthera uncia KP202269 1	Lutra sumatrana NC035810 1	0,1859135
Tremarctos ornatus NC009969 2	Leopardus pardalis T262 1	0,1859152
Martes martes T302 3	Hemigalus derbyanus MH464791 1	0,18591546
Neophoca cinerea NC008419 1	Ictonyx striatus T299 1	0,18591674
Smilodon populator MF871700 1	Mephitis mephitis NC020648 1	0,18591782
Conepatus chinga NC042596 1	Ailuropoda melanoleuca NC009492 5	0,18591823
Neophoca cinerea NC008419 1	Caracal caracal NC028306 1	0,18591841
Mungotictis decemlineata NC027828 1	Ictonyx striatus T299 1	0,18591879
Neophoca cinerea NC008419 1	Leopardus guigna NC028321 1	0,18591915
Procyon lotor AB462046 3	Paradoxurus hermaphroditus NLNC 1	0,1859199
Martes martes T302 3	Genetta genetta T297 1	0,18592031
Viverricula indica KX891751 1	Ursus arctos EU497665 29	0,18592052
Profelis aurata NC028299 1	Melogale moschata NC020644 1	0,18592145
Leopardus wiedii NC028318 1	Arctotherium sp NC030174 1	0,18592179
Ursus arctos GU573491 207	Mustela altaica NC021751 1	0,18592198
Otocolobus manul NC028323 1	Aonyx cinerea NC035814 2	0,18592267
Chrysocyon brachyurus NC024172 1	Arctonyx collaris NC020645 1	0,18592399
Ursus arctos isabellinus 18B5 2	Caracal caracal NC028306 1	0,18592684
Leopardus colocolo NC028314 1	Tapirus terrestris T358	0,18592791
Ursus spelaeus NC011112 8	Salanoia concolor D378 1	0,18592916
Helarctos malayanus NC009968 2	Aonyx cinerea NC035814 2	0,18592997
Ursus maritimus GU573488 Svalbard	Leopardus geoffroyi NC028320 1	0,18593024
Arctocephalus pusillus NC008417 1	Ailuropoda melanoleuca NC009492 5	0,1859304
Fossa fossana D350 1	Canis lupus chanco NC010340 4	0,18593085
Procyon lotor AB462049 4	Helarctos malayanus NC009968 2	0,18593121
Ursus arctos AP012576 6	Meles anakuma NC009677 1	0,18593256
Panthera pardus japonensis KJ866876	Canis aureus KT448274 1	0,18593355
Leopardus colocolo NC028314 1	Arctocephalus australis MG023139 1	0,18593432
Leopardus tigrinus NC028317 1	Galictis vittata T412 1	0,18593682
Ursus arctos AP012576 6	Mustela altaica NC021751 1	0,18593735
Zalophus wollebaeki SRR4431565 1	Parahyaena brunnea NC038159 15	0,18593737
Ursus arctos AP012576 6	Mustela nivalis T306 5	0,18593957
Lynx pardinus NC028319 161	Chrysocyon brachyurus NC024172 1	0,18593958
Vulpes zerda KJ603240 1	Genetta abyssinica MG489822 1	0,18593995
Leopardus colocolo NC028314 1	Arctotherium sp NC030174 1	0,18594085
Procyon lotor AB462046 3	Canis lupus familiaris NC002008 1231	0,1859412
Otocolobus manul NC028323 1	Helarctos malayanus NC009968 2	0,18594158
Leopardus guigna NC028321 1	Helarctos malayanus NC009968 2	0,18594302
Ursus spelaeus NC011112 8	Catopuma badia NC028300 1	0,18594338
Otaria byronia OTAB 1	Leopardus colocolo NC028314 1	0,18594342
Ursus arctos isabellinus 18B5 2	Leopardus colocolo NC028314 1	0,18594393
Martes foina NC020643 1	Canis lupus chanco NC010340 4	0,18595053
Ursus arctos AP012576 6	Arctictis binturong T605 2	0,18595141
Ursus americanus JX196366 3	Civettictis civetta GLC19 1	0,18595409
Genetta abyssinica MG489822 1	Arctocephalus townsendi NC008420 1	0,18595427
Urva javanica/auropunctata NC006835	Chrysocyon brachyurus NC024172 1	0,18595588
Leopardus pardalis T262 1	Cuon alpinus NC013445 3	0,18595615
Martes flavigula NC012141 3	Catopuma temminckii NC027115 41	0,18595676
Ursus spelaeus NC011112 8	Ictonyx striatus T299 1	0,18595774
Neophoca cinerea NC008419 1	Civettictis civetta NC033378 1	0,18595994
Panthera onca KP202264 2	Eumetopias jubatus NC004030 10	0,18596242
Panthera pardus NC010641 5	Melogale moschata NC020644 1	0,18596368
Lutra sumatrana NC035810 1	Urva javanica T413 1	0,18596415

Leopardus colocolo NC028314 1	Bdeogale nigripes GLC15 1	0,15212917
Leopardus colocolo NC028314 1	Ichneumia albicauda T603 1	0,1521311
Urocyon littoralis catalinae KP129018 15	Lycaon pictus NC028427 2	0,15216416
Genetta servalina NC024568 2	Eupleres goudotii D128 1	0,15217288
Suricata suricatta SSM10 1	Civettictis civetta GLC19 1	0,15217592
Mungos mungo/gambianus SRR7704821 1	Lynx rufus NC014456 3	0,15217793
Lynx rufus NC014456 3	Xenogale naso C07XAR110 1	0,15217805
Helogale parvula SRR7637809 1	Felis silvestris lybica KP202275 4	0,15217865
Catopuma badia NC028300 1	Bdeogale nigripes GLC15 1	0,15217972
Parahyaena brunnea NC038159 15	Urva javanica T413 1	0,15218151
Leptailurus serval NC028316 1	Ichneumia albicauda T603 1	0,15218152
Panthera uncia KP202269 1	Genetta servalina NC024568 2	0,15218233
Lynx pardinus NC028319 161	Galidia elegans D146 1	0,15218337
Paradoxurus hermaphroditus NC039591 1	Leopardus guigna NC028321 1	0,15218742
Mungos mungo MMC7 1	Arctictis binturong T605 2	0,1521882
Urva semitorquata MH464789 1	Acinonyx jubatus NC005212 3	0,1521941
Panthera onca KP202264 2	Hemigalus derbyanus MH464791 1	0,15219487
Panthera leo spelaea KX258452 2	Hemigalus derbyanus MH464791 1	0,15220106
Panthera onca NC022842 1	Hemigalus derbyanus MH464791 1	0,15222564
Ichneumia albicauda T603 1	Chrotogale owstoni T607 1	0,15224212
Mungos mungo MMC7 1	Caracal caracal NC028306 1	0,15224595
Smilodon populator MF871700 1	Urva javanica/auropunctata NC006835 1	0,15224683
Profelis aurata NC028299 1	Mungos mungo/gambianus SRR7704821 1	0,15224701
Galidictis fasciata DM333 1	Felis nigripes NC028309 1	0,15225232
Prionodon pardicolor NC024569 2	Panthera onca KP202264 2	0,15225761
Atilax paludinosus T606 1	Acinonyx jubatus NC005212 3	0,15226211
Ursus thibetanus laniger MH281753 2	Ailuropoda melanoleuca NC009492 5	0,15226255
Paradoxurus hermaphroditus NLNC 1	Urva javanica/auropunctata NC006835 1	0,15227346
Viverricula indica NC025296 2	Neofelis nebulosa NC008450 3	0,15228209
Paradoxurus hermaphroditus NC039591 1	Mungos mungo MMC7 1	0,15230736
Prionodon linsang ERR2391707 1	Leopardus tigrinus NC028317 1	0,15230738
Prionodon linsang ERR2391707 1	Leopardus jacobita NC028322 1	0,15230772
Pardofelis marmorata NLN3 2	Helogale parvula SRR7637809 1	0,15231337
Hyaena hyaena NC020669 1	Xenogale naso C07XAR110 1	0,15231465
Puma yagouaroundi NC028311 1	Ichneumia albicauda T603 1	0,15231605
Viverricula indica KX891745 1	Panthera uncia NC010638 1	0,15231615
Leopardus guigna NC028321 1	Galerella sanguinea T378 1	0,15231693
Hyaena hyaena NC020669 1	Cryptoprocta ferox CFC13 1	0,15232769
Leopardus tigrinus NC028317 1	Hyaena hyaena NC020669 1	0,15232964
Leopardus pardalis T262 1	Urva javanica/auropunctata NC006835 1	0,15234642
Panthera pardus japonensis KJ866876 8	Hemigalus derbyanus MH464791 1	0,15235974
Crossarchus platycephalus C7R66 1	Chrotogale owstoni T607 1	0,15236008
Cynogale bennetti KY117544 1	Acinonyx jubatus NC005212 3	0,15236397
Lynx lynx NC027083 4	Chrotogale owstoni T607 1	0,1523733
Paguma larvata PDDS11 2	Urva semitorquata MH464789 1	0,15237699
Xenogale naso C07XAR110 1	Genetta servalina NC024568 2	0,1523775
Mungos mungo/gambianus SRR7704821 1	Lynx lynx NC027083 4	0,15237924
Mungos mungo MMC7 1	Felis silvestris lybica KP202275 4	0,1523798
Leopardus wiedii NC028318 1	Urva brachyura KY117547 1	0,15238041
Profelis aurata NC028299 1	Urva semitorquata MH464789 1	0,15238194
Otocolobus manul NC028323 1	Crossarchus platycephalus C7R66 1	0,15238199
Leopardus jacobita NC028322 1	Ichneumia albicauda T603 1	0,15238413
Smilodon populator MF871700 1	Ichneumia albicauda T603 1	0,15238523
Pardofelis marmorata NLN3 2	Galidictis fasciata DM333 1	0,15238722
Paradoxurus jerdoni MH464793 1	Leopardus geoffroyi NC028320 1	0,15238941
Pardofelis marmorata NLN3 2	Cryptoprocta ferox CFC13 1	0,15239109
Smilodon populator MF871700 1	Bdeogale nigripes GLC15 1	0,15239951
Ichneumia albicauda T603 1	Diplogale hosei MH464790 1	0,15241219
Smilodon populator MF871700 1	Cynogale bennetti KY117544 1	0,15241227
Mungos mungo/gambianus SRR7704821 1	Chrotogale owstoni T607 1	0,15242846
Prionodon linsang ERR2391707 1	Lynx rufus NC014456 3	0,15244351
Paradoxurus hermaphroditus NC039591 1	Lynx rufus NC014456 3	0,15244846
Otocolobus manul NC028323 1	Urva semitorquata MH464789 1	0,15244946
Puma concolor NC016470 22	Ichneumia albicauda T603 1	0,15245017
Hemigalus derbyanus MH464791 1	Helogale parvula SRR7637809 1	0,1524564
Diplogale hosei MH464790 1	Atilax paludinosus T606 1	0,15246572
Zalophus californianus NC008416 1	Odobenus rosmarus NC004029 29	0,1524671
Mungos mungo MMC7 1	Diplogale hosei MH464790 1	0,15247315
Viverricula indica KX891745 1	Neofelis nebulosa NC008450 3	0,1524808
Suricata suricatta SSM10 1	Nandinia binotata NC024567 1	0,15250471
Paradoxurus hermaphroditus NLNC 1	Mungos mungo/gambianus SRR7704821 1	0,15251154
Lynx pardinus NC028319 161	Urva semitorquata MH464789 1	0,1525151
Ichneumia albicauda T603 1	Catopuma temminckii NC027115 41	0,15251742
Galidia elegans D146 1	Catopuma badia NC028300 1	0,15251854
Lynx canadensis NC028313 1	Galidia elegans D146 1	0,15251969
Parahyaena brunnea NC038159 15	Leopardus pardalis NC028315 1	0,15253505
Panthera pardus NC010641 5	Galidictis fasciata DM333 1	0,15255629
Viverra zibetha T609 1	Suricata suricatta SSM10 1	0,15257962
Viverra zibetha T609 1	Urva brachyura KY117547 1	0,15257971
Lynx rufus NC014456 3	Urva javanica T413 1	0,15258101
Viverra zibetha T609 1	Cryptoprocta ferox CFC13 1	0,15258232
Prionailurus planiceps KY682741 4	Mungos mungo MMC7 1	0,15258245
Prionailurus viverrinus NC028305 1	Paradoxurus jerdoni MH464793 1	0,15258282
Paradoxurus hermaphroditus NLNC 1	Lynx canadensis NC028313 1	0,15258303
Felis nigripes NC028309 1	Atilax paludinosus T606 1	0,15258361
Hyaena hyaena NC020669 1	Cynictis penicillata T375 1	0,15258408
Mungos mungo MMC7 1	Leopardus tigrinus NC028317 1	0,15258513
Ommatophoca rossii AY377287etc 1	Arctocephalus forsteri KT693377 17	0,15258551
Leopardus tigrinus NC028317 1	Galerella sanguinea T378 1	0,15258558
Prionailurus bengalensis CKM45 20	Galerella sanguinea T378 1	0,15258764
Ursus arctos isabellinus 18B5 2	Ailuropoda melanoleuca NC009492 5	0,15258832
Otocolobus manul NC028323 1	Mungotictis decemlineata NC027828 1	0,15259022
Hyaena hyaena NC020669 1	Catopuma badia NC028300 1	0,15259333
Parahyaena brunnea NC038159 15	Leopardus guigna NC028321 1	0,15260126
Homotherium latidens MF871702 3	Galerella sanguinea T378 1	0,15260206
Helogale parvula SRR7637809 1	Genetta abyssinica MG489822 1	0,15263847
Urva brachyura KY117547 1	Genetta abyssinica MG489822 1	0,15263937
Paguma larvata PDDS11 2	Eupleres goudotii D128 1	0,15264252
Paradoxurus jerdoni MH464793 1	Cynictis penicillata T375 1	0,15264589
Prionodon linsang ERR2391707 1	Otocolobus manul NC028323 1	0,15264639
Suricata suricatta SSM10 1	Prionailurus bengalensis NC028301 12	0,15264985
Puma yagouaroundi NC028311 1	Cynictis penicillata T375 1	0,15265013
Viverricula indica KX891745 1	Galidia elegans D146 1	0,15265058

Mustela itatsi NC034330 19	Eupleres goudotii D128 1	0,18596649
Martes americana NC020642 1	Bdeogale nigripes GLC15 1	0,18596651
Fossa fossana D350 1	Canis anthus NC027956 2	0,18596879
Mungos mungo/gambianus SRR77048	Ailurus fulgens styani NC009691 1	0,18596923
Neophoca cinerea NC008419 1	Urva semitorquata MH464789 1	0,18596943
Neophoca cinerea NC008419 1	Bassaricyon neblina SRX1097850 1	0,18596974
Nyctereutes procyonoides NC013700 3	Mungos mungo MMC7 1	0,1859708
Vulpes vulpes NC008434 5	Mungotictis decemlineata NC027828 1	0,18597175
Vulpes corsac NC023958 1	Mustela putorius NC020638 4	0,18597206
Urva semitorquata MH464789 1	Arctocephalus pusillus NC008417 1	0,18597214
Martes pennanti NC020664 16	Eupleres goudotii D128 1	0,18597326
Panthera onca NC022842 1	Eumetopias jubatus NC004030 10	0,1859739
Felis nigripes NC028309 1	Canis mesomelas KT448280 1	0,18597457
Martes melampus NC009678 1	Cryptoprocta ferox CFC13 1	0,18597527
Lynx pardinus NC028319 161	Ailurus fulgens styani NC009691 1	0,1859756
Lutra lutra LC050126 1	Hyaena hyaena NC020669 1	0,18597676
Panthera uncia NC010638 1	Enhydra lutris NC009692 1	0,18597707
Mustela altaica NC021751 1	Mungos mungo/gambianus SRR77048	0,18597709
Felis nigripes NC028309 1	Callorhinus ursinus NC008415 1	0,18597716
Lutrogale perspicillata NC035811 1	Leopardus pardalis T262 1	0,1859774
Mephitis mephitis NC020648 1	Civettictis civetta NC033378 1	0,18597807
Catopuma badia NC028300 1	Arctocephalus australis MG023139 1	0,18598083
Parahyaena brunnea NC038159 15	Martes zibellina NC011579 39	0,1859818
Vulpes ferrilata NC027935 1	Felis margarita NC028308 1	0,18598205
Vulpes ferrilata NC027935 1	Puma yagouaroundi NC028311 1	0,1859825
Lynx rufus NC014456 3	Canis anthus NC027956 2	0,18598275
Mustela frenata NC020640 1	Cryptoprocta ferox CFC13 1	0,18598381
Felis nigripes NC028309 1	Aonyx cinerea NC035814 2	0,1859877
Zalophus wollebaeki SRR4431565 1	Galidictis fasciata DM333 1	0,1859881
Poecilogale albinucha T602 1	Civettictis civetta GLC19 1	0,1859885
Ursus arctos isabellinus 18B5 2	Paradoxurus jerdoni MH464793 1	0,18598931
Ursus arctos EU497665 29	Otaria byronia OTAB 1	0,1859903
Otocyon megalotis SAF1 2	Felis catus NC001700 2	0,18599062
Parahyaena brunnea NC038159 15	Arctodus simus NC011116 1	0,18599351
Parahyaena brunnea NC038159 15	Canis adustus KT448271 1	0,1859937
Lycaon pictus NC028427 2	Leopardus tigrinus NC028317 1	0,18599522
Lutra sumatrana NC035810 1	Ailuropoda melanoleuca NC009492 5	0,18599552
Zalophus wollebaeki SRR4431565 1	Genetta genetta T297 1	0,18599623
Ursus arctos isabellinus 18B5 2	Catopuma temminckii NC027115 41	0,1859968
Ursus thibetanus mupinensis NC00875	Lycaon pictus NC028427 2	0,18599881
Panthera onca NC022842 1	Bassariscus sumichrasti SRX1099089 1	0,18599917
Paradoxurus hermaphroditus NC03959	Lycaon pictus NC028427 2	0,18599947
Lontra canadensis SRR10409165 1	Leopardus colocolo NC028314 1	0,18600123
Taxidea taxus NC020646 1	Panthera leo spelaea KX258452 2	0,18600138
Viverricula indica KX891751 1	Ursus arctos AP012576 6	0,18600228
Urva javanica/auropunctata NC006835	Canis anthus NC027956 2	0,18600248
Ursus arctos isabellinus 18B5 2	Arctocephalus forsteri NC004023 28	0,18600538
Ursus arctos AP012576 6	Otaria byronia OTAB 1	0,18600602
Helarctos malayanus NC009968 2	Felis margarita NC028308 1	0,18600671
Mungos mungo MMC7 1	Arctocephalus forsteri NC004023 28	0,1860116
Poecilogale albinucha T602 1	Canis lupus familiaris NC002008 1231	0,18601188
Zalophus californianus NC008416 1	Genetta genetta T297 1	0,18601191
Panthera pardus japonensis KJ866876	Canis lupus chanco NC010340 4	0,18601404
Civettictis civetta NC033378 1	Arctocephalus australis MG023139 1	0,18601507
Civettictis civetta NC033378 1	Arctocephalus forsteri KT693377 17	0,18601513
Neofelis nebulosa NC008450 3	Mustela frenata NC020640 1	0,18601556
Mustela nivalis T306 5	Urva javanica/auropunctata NC006835	0,18601595
Ursus arctos GU573486 5	Acinonyx jubatus NC005212 3	0,1860161
Mustela sibirica NC020637 6	Cuon alpinus NC013445 3	0,18601781
Zalophus californianus NC008416 1	Panthera tigris amoyensis NC014770 2	0,18601938
Potos flavus T414 1	Panthera pardus NC010641 5	0,18602155
Galictis vittata T412 1	Acinonyx jubatus NC005212 3	0,18602192
Canis lupus chanco NC010340 4	Bassariscus sumichrasti SRX1099089 1	0,18602289
Canis lupus chanco NC010340 4	Arctotherium sp NC030174 1	0,18602298
Leopardus guigna NC028321 1	Arctocephalus forsteri NC004023 28	0,18602306
Spilogale putorius NC010497 1	Genetta servalina NC024568 2	0,18602541
Ursus americanus JX196366 3	Leopardus geoffroyi NC028320 1	0,18602806
Martes melampus NC009678 1	Eupleres goudotii D128 1	0,18603075
Leopardus tigrinus NC028317 1	Cuon alpinus NC013445 3	0,18603079
Prionailurus viverrinus NC028305 1	Tapirus terrestris T358	0,18603139
Ursus thibetanus laniger MH281753 2	Canis lupus familiaris NC002008 1231	0,18603314
Martes americana NC020642 1	Atilax paludinosus T606 1	0,18603322
Phocarctos hookeri NC008418 1	Chrotogale owstoni T607 1	0,1860334
Atilax paludinosus T606 1	Arctonyx collaris NC020645 1	0,1860335
Martes zibellina NC011579 39	Cynictis penicillata T375 1	0,18603399
Bdeogale nigripes GLC15 1	Arctonyx collaris NC020645 1	0,1860342
Mustela putorius NC020638 4	Urva brachyura KY117547 1	0,18603471
Vulpes zerda KJ603240 1	Melogale moschata V0735A 1	0,18603615
Urva semitorquata MH464789 1	Canis anthus NC027956 2	0,18603656
Melogale moschata KP726273 1	Canis latrans NC008093 7	0,18603697
Procyon lotor AB462049 4	Arctocephalus australis MG023139 1	0,18603705
Neophoca cinerea NC008419 1	Atilax paludinosus T606 1	0,18603732
Lynx pardinus NC028319 161	Canis mesomelas KT448280 1	0,18603799
Mustela eversmannii NC028013 1	Diplogale hosei MH464790 1	0,18603812
Meles leucurus NC039173 4	Canis anthus NC027956 2	0,18603839
Viverricula indica KX891751 1	Callorhinus ursinus NC008415 1	0,18603864
Mustela sibirica NC020637 6	Galidictis fasciata DM333 1	0,1860387
Salanoia concolor D378 1	Mustela sibirica NC020637 6	0,18603885
Martes martes T302 3	Eupleres goudotii D128 1	0,18604089
Melogale moschata NC020644 1	Cynictis penicillata T375 1	0,18604158
Mustela putorius NC020638 4	Galerella sanguinea T378 1	0,18604384
Mustela altaica NC021751 1	Eupleres goudotii D128 1	0,18604483
Genetta genetta T297 1	Arctocephalus townsendi NC008420 1	0,18604498
Canis latrans NC008093 7	Bassariscus sumichrasti SRX1099089 1	0,18604587
Prionodon linsang ERR2391707 1	Arctodus simus NC011116 1	0,18604599
Ursus arctos EU497665 29	Melogale moschata V0735A 1	0,18604685
Viverra tangalunga MH464792 1	Panthera pardus japonensis KJ866876	0,18604755
Pardofelis marmorata NLN3 2	Melogale moschata V0735A 1	0,18604805
Mustela nivalis T306 5	Galidictis fasciata DM333 1	0,18604812
Vulpes corsac NC023958 1	Mustela nivalis T306 5	0,18604819
Potos flavus T414 1	Cryptoprocta ferox CFC13 1	0,18604934
Vulpes lagopus NC026529 3	Leopardus tigrinus NC028317 1	0,18604939
Prionodon pardicolor NC024569 2	Mustela eversmannii NC028013 1	0,18604946

Felis chaus NC028307 1	Eupleres goudotii D128 1	0,15265171
Prionailurus bengalensis NC028301 12	Ichneumia albicauda T603 1	0,15265253
Otocolobus manul NC028323 1	Ichneumia albicauda T603 1	0,1526531
Viverricula indica NC025296 2	Cryptoprocta ferox CFC13 1	0,1526555
Profelis aurata NC028299 1	Mungotictis decemlineata NC027828 1	0,15265727
Prionodon pardicolor NC024569 2	Paradoxurus hermaphroditus NC039591 1	0,15265913
Urva javanica T413 1	Arctictis binturong T605 2	0,15266033
Urva semitorquata MH464789 1	Arctictis binturong T605 2	0,15266103
Mungos mungo/gambianus SRR7704821 1	Arctictis binturong T605 2	0,15266109
Lynx rufus NC014456 3	Hyaena hyaena NC020669 1	0,15266274
Ursus thibetanus formosanus NC009331 1	Ailuropoda melanoleuca NC009492 5	0,15266322
Prionailurus viverrinus NC028305 1	Cryptoprocta ferox CFC13 1	0,15266402
Lynx canadensis NC028313 1	Diplogale hosei MH464790 1	0,15270182
Viverra zibetha T609 1	Eupleres goudotii D128 1	0,15271114
Genetta genetta T297 1	Bdeogale nigripes GLC15 1	0,15271579
Mungos mungo MMC7 1	Lynx canadensis NC028313 1	0,15271629
Profelis aurata NC028299 1	Urva brachyura KY117547 1	0,15271699
Lynx pardinus NC028319 161	Bdeogale nigripes GLC15 1	0,15271704
Galerella sanguinea T378 1	Catopuma temminckii NC027115 41	0,15271775
Genetta servalina NC024568 2	Galidia elegans D146 1	0,15271789
Leopardus jacobita NC028322 1	Cynictis penicillata T375 1	0,15271804
Homotherium latidens MF871702 3	Galidictis fasciata DM333 1	0,1527189
Panthera pardus NC010641 5	Hemigalus derbyanus MH464791 1	0,1527205
Cryptoprocta ferox CFC13 1	Catopuma temminckii NC027115 41	0,15272281
Zalophus wollebaeki SRR4431565 1	Odobenus rosmarus NC004029 29	0,15272374
Paradoxurus jerdoni MH464793 1	Leopardus guigna NC028321 1	0,15272613
Salanoia concolor D378 1	Hemigalus derbyanus MH464791 1	0,15275313
Panthera pardus NC010641 5	Fossa fossana D350 1	0,15275484
Viverricula indica KX891751 1	Eupleres goudotii D128 1	0,15277776
Prionodon linsang ERR2391707 1	Genetta genetta T297 1	0,15278216
Smilodon populator MF871700 1	Mungos mungo/gambianus SRR7704821 1	0,15278429
Otocolobus manul NC028323 1	Mungos mungo/gambianus SRR7704821 1	0,15278576
Prionodon linsang ERR2391707 1	Catopuma temminckii NC027115 41	0,15278646
Otocolobus manul NC028323 1	Galerella sanguinea T378 1	0,15278703
Prionailurus bengalensis CKM45 20	Urva brachyura KY117547 1	0,15278724
Leopardus guigna NC028321 1	Urva javanica T413 1	0,15278748
Leopardus tigrinus NC028317 1	Atilax paludinosus T606 1	0,15278807
Proteles cristata T393 6	Galidictis fasciata DM333 1	0,15278919
Genetta genetta T297 1	Galerella sanguinea T378 1	0,15279399
Leopardus jacobita NC028322 1	Hyaena hyaena NC020669 1	0,15280066
Smilodon populator MF871700 1	Cryptoprocta ferox CFC13 1	0,15280679
Suricata suricatta SSM10 1	Hemigalus derbyanus MH464791 1	0,15281686
Otaria byronia OTAB 1	Ommatophoca rossii AY377287etc 1	0,15283197
Viverricula indica KX891751 1	Suricata suricatta SSM10 1	0,15284853
Potos flavus T414 1	Bassariscus sumichrasti SRX1099089 1	0,15284859
Urva javanica T413 1	Genetta genetta T297 1	0,1528503
Panthera leo spelaea KX258452 2	Fossa fossana D350 1	0,15285181
Prionailurus rubiginosus NC028304 2	Urva javanica T413 1	0,15285191
Lynx lynx NC027083 4	Ichneumia albicauda T603 1	0,15285224
Panthera uncia KP202269 1	Fossa fossana D350 1	0,15285267
Felis catus NC001700 2	Eupleres goudotii D128 1	0,15285368
Galidia elegans D146 1	Catopuma temminckii NC027115 41	0,15285505
Leopardus geoffroyi NC028320 1	Eupleres goudotii D128 1	0,15285589
Ursus maritimus GU573488 Svalbard	Ailuropoda melanoleuca NC009492 5	0,15285763
Prionailurus viverrinus NC028305 1	Hyaena hyaena NC020669 1	0,15286708
Panthera leo spelaea KX258452 2	Genetta abyssinica MG489822 1	0,1528721
Proteles cristata T393 6	Prionailurus bengalensis NC028301 12	0,15287469
Leopardus colocolo NC028314 1	Urva javanica/auropunctata NC006835 1	0,15290016
Paguma larvata PDDS11 2	Xenogale naso C07XAR110 1	0,15291548
Viverricula indica KX891751 1	Urva brachyura KY117547 1	0,152916
Viverricula indica KX891751 1	Helogale parvula SRR7637809 1	0,15291603
Phocarctos hookeri NC008418 1	Odobenus rosmarus NC004029 29	0,15291867
Leopardus pardalis NC028315 1	Urva brachyura KY117547 1	0,1529188
Pardofelis marmorata NLN3 2	Crossarchus platycephalus C7R66 1	0,15291917
Urva brachyura KY117547 1	Catopuma badia NC028300 1	0,15291952
Lynx pardinus NC028319 161	Atilax paludinosus T606 1	0,15291975
Lynx pardinus NC028319 161	Ichneumia albicauda T603 1	0,15292037
Helogale parvula SRR7637809 1	Caracal caracal NC028306 1	0,15292077
Leopardus guigna NC028321 1	Cynictis penicillata T375 1	0,15292218
Ichneumia albicauda T603 1	Hyaena hyaena NC020669 1	0,15292294
Panthera uncia KP202269 1	Galidictis fasciata DM333 1	0,15292433
Puma yagouaroundi NC028311 1	Galidia elegans D146 1	0,15292604
Cynictis penicillata T375 1	Acinonyx jubatus NC005212 3	0,15293311
Leopardus guigna NC028321 1	Hyaena hyaena NC020669 1	0,15293464
Prionodon linsang ERR2391707 1	Felis chaus NC028307 1	0,15298319
Suricata suricatta SSM10 1	Genetta servalina NC024568 2	0,1529838
Genetta genetta T297 1	Cynictis penicillata T375 1	0,15298484
Lynx pardinus NC028319 161	Helogale parvula SRR7637809 1	0,15298627
Felis chaus NC028307 1	Crossarchus platycephalus C7R66 1	0,15298714
Felis chaus NC028307 1	Atilax paludinosus T606 1	0,1529882
Puma yagouaroundi NC028311 1	Eupleres goudotii D128 1	0,15298933
Leopardus jacobita NC028322 1	Galerella sanguinea T378 1	0,1529897
Leopardus geoffroyi NC028320 1	Galerella sanguinea T378 1	0,15299021
Hemigalus derbyanus MH464791 1	Galidictis fasciata DM333 1	0,15299523
Panthera tigris amoyensis NC014770 2	Genetta genetta T297 1	0,15299737
Homotherium latidens MF871702 3	Atilax paludinosus T606 1	0,15301009
Panthera leo NERO 19	Galidictis fasciata DM333 1	0,15302557
Neofelis nebulosa NC008450 3	Genetta abyssinica MG489822 1	0,15303725
Helogale parvula SRR7637809 1	Genetta servalina NC024568 2	0,15305136
Lynx rufus NC014456 3	Bdeogale nigripes GLC15 1	0,15305361
Lynx canadensis NC028313 1	Atilax paludinosus T606 1	0,15305384
Xenogale naso C07XAR110 1	Caracal caracal NC028306 1	0,15305389
Mungos mungo/gambianus SRR7704821 1	Caracal caracal NC028306 1	0,15305515
Viverricula indica KX891751 1	Panthera uncia KP202269 1	0,1530565
Suricata suricatta SSM10 1	Prionailurus bengalensis CKM45 20	0,15305765
Panthera uncia KP202269 1	Genetta genetta T297 1	0,15305875
Viverra zibetha T609 1	Galerella sanguinea T378 1	0,15306269
Mungos mungo/gambianus SRR7704821 1	Acinonyx jubatus NC005212 3	0,15306893
Galidia elegans D146 1	Acinonyx jubatus NC005212 3	0,15307538
Prionodon pardicolor NC024569 2	Arctictis binturong T605 2	0,1530755
Homotherium latidens MF871702 3	Urva semitorquata MH464789 1	0,15308549
Urocyon cinereoargenteus NC026723 21	Speothos venaticus C48 2	0,15310735
Leptailurus serval NC028316 1	Xenogale naso C07XAR110 1	0,15312123

Melogale moschata V0735A 1	Caracal caracal NC028306 1	0,18604955
Neophoca cinerea NC008419 1	Ichneumia albicauda T603 1	0,18604964
Vulpes zerda KJ603240 1	Smilodon populator MF871700 1	0,18605037
Phocarctos hookeri NC008418 1	Lynx rufus NC014456 3	0,18605095
Martes pennanti NC020664 16	Galerella sanguinea T378 1	0,18605132
Phocarctos hookeri NC008418 1	Leopardus pardalis NC028315 1	0,18605216
Vulpes lagopus NC026529 3	Leopardus geoffroyi NC028320 1	0,18605256
Genetta genetta T297 1	Arctocephalus pusillus NC008417 1	0,18605407
Mustela nivalis T306 5	Hyaena hyaena NC020669 1	0,18605632
Martes pennanti NC020664 16	Felis chaus NC028307 1	0,18605685
Panthera onca KP202264 2	Arctodus simus NC011116 1	0,1860572
Ursus arctos GU573486 5	Lutra sumatrana NC035810 1	0,18605836
Otaria byronia OTAB 1	Leopardus guigna NC028321 1	0,18605934
Ursus arctos isabellinus 18B5 2	Prionailurus rubiginosus NC028304 2	0,18606129
Panthera onca NC022842 1	Nyctereutes procyonoides NC013700 3	0,18606143
Ursus arctos EU497665 29	Catopuma temminckii NC027115 41	0,18606456
Enhydra lutris NC009692 1	Canis lupus familiaris NC002008 1231	0,18606627
Ommatophoca rossii AY377287etc 1	Tapirus terrestris T358	0,18606766
Salanoia concolor D378 1	Canis lupus chanco NC010340 4	0,1860688
Galictis vittata T412 1	Civettictis civetta GLC19 1	0,18606897
Ursus spelaeus EU327344 13	Puma concolor NC016470 22	0,18606928
Ursus arctos GU573486 5	Leopardus geoffroyi NC028320 1	0,18607105
Paradoxurus jerdoni MH464793 1	Martes flavigula NC012141 3	0,18607184
Urva javanica/auropunctata NC006835	Enhydra lutris NC009692 1	0,18607195
Galictis vittata T412 1	Felis catus NC001700 2	0,18607253
Melursus ursinus NC009970 2	Lynx canadensis NC028313 1	0,18607275
Ursus spelaeus EU327344 13	Prionailurus bengalensis CKM45 20	0,18607295
Mustela putorius NC020638 4	Urva javanica/auropunctata NC006835	0,18607329
Ursus thibetanus laniger MH281753 2	Prionailurus planiceps KY682741 4	0,18607344
Ursus thibetanus laniger MH281753 2	Prionailurus bengalensis NC028301 12	0,18607484
Ursus thibetanus thibetanus NC01111	Profelis aurata NC028299 1	0,18607549
Vulpes zerda KJ603240 1	Homotherium latidens MF871702 3	0,1860767
Ursus maritimus GU573488 Svalbard	Acinonyx jubatus NC005212 3	0,18607716
Ursus maritimus NC003428 31	Acinonyx jubatus NC005212 3	0,18607719
Spilogale putorius NC010497 1	Melogale moschata V0735A 1	0,18607721
Spilogale putorius NC010497 1	Melogale moschata KP726273 1	0,18607795
Chrysocyon brachyurus NC024172 1	Ailuropoda melanoleuca NC009492 5	0,18608193
Prionailurus viverrinus NC028305 1	Canis lupus familiaris NC002008 1231	0,18608357
Mustela putorius NC020638 4	Cuon alpinus NC013445 3	0,186085
Otocolobus manul NC028323 1	Arctocephalus forsteri NC004023 28	0,18608524
Nyctereutes procyonoides NC013700 3	Martes flavigula NC012141 3	0,1860856
Felis silvestris lybica KP202275 4	Cuon alpinus NC013445 3	0,18609223
Smilodon populator MF871700 1	Cuon alpinus NC013445 3	0,18609279
Ursus americanus JX196366 3	Prionailurus rubiginosus NC028304 2	0,18609346
Mustela altaica NC021751 1	Cuon alpinus NC013445 3	0,18609409
Felis margarita NC028308 1	Eumetopias jubatus NC004030 10	0,186096
Profelis aurata NC028299 1	Tapirus terrestris T358	0,18609752
Prionodon pardicolor NC024569 2	Canis lupus chanco NC010340 4	0,18609914
Ursus arctos GU573486 5	Smilodon populator MF871700 1	0,1860999
Urva brachyura KY117547 1	Arctonyx collaris NC020645 1	0,18610057
Mustela sibirica NC020637 6	Eupleres goudotii D128 1	0,18610074
Martes melampus NC009678 1	Galidia elegans D146 1	0,18610153
Lutra sumatrana NC035810 1	Galidia elegans D146 1	0,18610314
Bassaricyon neblina SRX1097850 1	Arctocephalus townsendi NC008420 1	0,18610382
Suricata suricatta SSM10 1	Potos flavus T414 1	0,18610385
Salanoia concolor D378 1	Mustela kathiah NC023210 1	0,18610764
Galidia elegans D146 1	Arctonyx collaris NC020645 1	0,18610767
Meles leucurus NC039173 4	Galerella sanguinea T378 1	0,18610825
Melogale moschata NC020644 1	Urva javanica T413 1	0,18610837
Leopardus pardalis NC028315 1	Tapirus terrestris T358	0,18610841
Vulpes vulpes NC008434 5	Melogale moschata NC020644 1	0,18611139
Panthera onca KP202264 2	Arctonyx collaris NC020645 1	0,18611217
Prionodon linsang ERR2391707 1	Ommatophoca rossii AY377287etc 1	0,18611224
Genetta genetta T297 1	Arctocephalus forsteri KT693377 17	0,18611312
Caracal caracal NC028306 1	Ailurus fulgens NC011124 1	0,18611379
Martes zibellina NC011579 39	Lycaon pictus NC028427 2	0,18611404
Tremarctos ornatus NC009969 2	Genetta genetta T297 1	0,18611406
Mustela nigripes NC024942 1	Hyaena hyaena NC020669 1	0,18611486
Otocolobus manul NC028323 1	Arctocephalus forsteri KT693377 17	0,18611594
Parahyaena brunnea NC038159 15	Martes americana NC020642 1	0,18611643
Vulpes ferrilata NC027935 1	Profelis aurata NC028299 1	0,18611677
Vulpes zerda KJ603240 1	Felis silvestris lybica KP202275 4	0,18611684
Bassaricyon neblina SRX1097850 1	Arctodus simus NC011116 1	0,18611711
Leopardus jacobita NC028322 1	Canis latrans NC008093 7	0,18611859
Ursus maritimus GU573488 Svalbard	Lutra sumatrana NC035810 1	0,18611917
Prionailurus bengalensis CKM45 20	Arctocephalus townsendi NC008420 1	0,18611947
Ursus arctos pruinosus MG066703 3	Arctocephalus gazella BK010918 1	0,18612231
Viverricula indica KX891745 1	Ursus maritimus GU573488 Svalbard	0,1861224
Viverricula indica KX891745 1	Ursus maritimus NC003428 31	0,18612242
Galictis vittata T412 1	Arctocephalus australis MG023139 1	0,18612331
Mustela sibirica AP017394 11	Ailuropoda melanoleuca NC009492 5	0,18612759
Leopardus geoffroyi NC028320 1	Arctotherium sp NC030174 1	0,18612768
Helogale parvula SRR7637809 1	Canis aureus KT448274 1	0,18612868
Urva javanica T413 1	Canis aureus KT448274 1	0,18612885
Vulpes ferrilata NC027935 1	Ommatophoca rossii AY377287etc 1	0,1861293
Melursus ursinus NC009970 2	Meles anakuma NC009677 1	0,1861316
Parahyaena brunnea NC038159 15	Martes foina NC020643 1	0,18613169
Ictonyx striatus T299 1	Genetta genetta T297 1	0,18613246
Ursus thibetanus thibetanus NC01111	Canis latrans NC008093 7	0,18613295
Ursus thibetanus laniger MH281753 2	Phocarctos hookeri NC008418 1	0,18613301
Zalophus wollebaeki SRR4431565 1	Ailuropoda melanoleuca NC009492 5	0,18613428
Zalophus wollebaeki SRR4431565 1	Leopardus guigna NC028321 1	0,18613522
Ursus spelaeus EU327344 13	Leopardus tigrinus NC028317 1	0,18613599
Prionodon pardicolor NC024569 2	Canis aureus KT448274 1	0,18613678
Ursus spelaeus EU327344 13	Felis chaus NC028307 1	0,18613699
Mustela eversmannii NC028013 1	Arctocephalus forsteri NC004023 28	0,18613772
Bassariscus sumichrasti SRX1099089 1	Ailuropoda melanoleuca NC009492 5	0,18613966
Canis aureus KT448274 1	Arctotherium sp NC030174 1	0,18614005
Prionailurus planiceps KY682741 4	Galictis vittata T412 1	0,18614054
Vulpes corsac NC023958 1	Ommatophoca rossii AY377287etc 1	0,18614163
Ursus arctos pruinosus MG066703 3	Arctictis binturong T605 2	0,18614449
Panthera uncia NC010638 1	Ommatophoca rossii AY377287etc 1	0,18614875
Lutra lutra NC011358 9	Chrotogale owstoni T607 1	0,18615364

Puma concolor NC016470 22	Helogale parvula SRR7637809 1	0,15312195
Leptailurus serval NC028316 1	Galerella sanguinea T378 1	0,15312347
Pardofelis marmorata NLN3 2	Eupleres goudotii D128 1	0,15312357
Leopardus geoffroyi NC028320 1	Galidia elegans D146 1	0,15312783
Ursus arctos GU573486 5	Ailuropoda melanoleuca NC009492 5	0,15313278
Leopardus colocolo NC028314 1	Xenogale naso C07XAR110 1	0,153138
Hyaena hyaena NC020669 1	Urva javanica/auropunctata NC006835 1	0,1531567
Lynx rufus NC014456 3	Galerella sanguinea T378 1	0,15318869
Genetta genetta T297 1	Galidia elegans D146 1	0,15318985
Leopardus guigna NC028321 1	Helogale parvula SRR7637809 1	0,15319191
Leopardus guigna NC028321 1	Ichneumia albicauda T603 1	0,15319365
Salanoia concolor D378 1	Felis chaus NC028307 1	0,15319557
Cryptoprocta ferox CFC13 1	Caracal caracal NC028306 1	0,15319927
Leopardus geoffroyi NC028320 1	Hyaena hyaena NC020669 1	0,15320374
Leopardus colocolo NC028314 1	Urva brachyura KY117547 1	0,15320586
Parahyaena brunnea NC038159 15	Leopardus geoffroyi NC028320 1	0,15320751
Prionodon pardicolor NC024569 2	Panthera onca NC022842 1	0,15320876
Urva semitorquata MH464789 1	Chrotogale owstoni T607 1	0,15323789
Prionodon linsang ERR2391707 1	Lynx lynx NC027083 4	0,15325116
Mungos mungo/gambianus SRR7704821 1	Felis silvestris lybica KP202275 4	0,15325566
Mungos mungo/gambianus SRR7704821 1	Felis margarita NC028308 1	0,15325607
Helogale parvula SRR7637809 1	Felis margarita NC028308 1	0,15325637
Leopardus pardalis T262 1	Bdeogale nigripes GLC15 1	0,15325768
Mungotictis decemlineata NC027828 1	Felis nigripes NC028309 1	0,15326205
Prionodon pardicolor NC024569 2	Paradoxurus jerdoni MH464793 1	0,15326495
Ursus spelaeus NC011112 8	Ailuropoda melanoleuca NC009492 5	0,15327267
Nandinia binotata NC024567 1	Diplogale hosei MH464790 1	0,15328267
Urva brachyura KY117547 1	Civettictis civetta NC033378 1	0,1532862
Smilodon populator MF871700 1	Galidictis fasciata DM333 1	0,15330154
Xenogale naso C07XAR110 1	Genetta abyssinica MG489822 1	0,15331778
Paguma larvata PDDS11 2	Bdeogale nigripes GLC15 1	0,15331963
Profelis aurata NC028299 1	Prionodon linsang ERR2391707 1	0,15331976
Lynx canadensis NC028313 1	Helogale parvula SRR7637809 1	0,1533224
Felis silvestris lybica KP202275 4	Cynictis penicillata T375 1	0,15332247
Prionailurus planiceps KY682741 4	Bdeogale nigripes GLC15 1	0,15332398
Prionailurus planiceps NC028312 6	Galerella sanguinea T378 1	0,15332454
Leopardus guigna NC028321 1	Urva brachyura KY117547 1	0,15332566
Prionailurus bengalensis CKM45 20	Cynictis penicillata T375 1	0,15332679
Otaria byronia OTAB 1	Odobenus rosmarus NC004029 29	0,15332703
Panthera onca NC022842 1	Paguma larvata PDDS11 2	0,15333456
Urva javanica/auropunctata NC006835 1	Caracal caracal NC028306 1	0,15335611
Paradoxurus hermaphroditus NLNC 1	Panthera leo NERO 19	0,15335618
Prionodon linsang ERR2391707 1	Diplogale hosei MH464790 1	0,15337904
Genetta genetta T297 1	Eupleres goudotii D128 1	0,15338655
Profelis aurata NC028299 1	Helogale parvula SRR7637809 1	0,15339208
Leopardus wiedii NC028318 1	Bdeogale nigripes GLC15 1	0,1533924
Galidictis fasciata DM333 1	Crocuta crocuta NC020670 3	0,15339595
Galidia elegans D146 1	Felis margarita NC028308 1	0,15339653
Panthera tigris amoyensis NC014770 2	Fossa fossana D350 1	0,15339799
Leptailurus serval NC028316 1	Cryptoprocta ferox CFC13 1	0,15340132
Urva javanica T413 1	Diplogale hosei MH464790 1	0,15341173
Smilodon populator MF871700 1	Mungotictis decemlineata NC027828 1	0,15342522
Lynx rufus NC014456 3	Urva javanica/auropunctata NC006835 1	0,15342684
Odobenus rosmarus NC004029 29	Eumetopias jubatus NC004030 10	0,15343158
Cynogale bennetti KY117544 1	Catopuma badia NC028300 1	0,15343894
Urocyon littoralis catalinae KP129018 15	Speothos venaticus C48 2	0,15344399
Urva javanica/auropunctata NC006835 1	Diplogale hosei MH464790 1	0,1534458
Prionodon linsang ERR2391707 1	Lynx canadensis NC028313 1	0,15345326
Xenogale naso C07XAR110 1	Catopuma badia NC028300 1	0,15345666
Eupleres goudotii D128 1	Catopuma temminckii NC027115 41	0,15345713
Profelis aurata NC028299 1	Mungos mungo MMC7 1	0,15345785
Prionailurus planiceps NC028312 6	Bdeogale nigripes GLC15 1	0,1534587
Prionailurus viverrinus NC028305 1	Bdeogale nigripes GLC15 1	0,15346046
Leopardus guigna NC028321 1	Galidia elegans D146 1	0,15346442
Prionodon pardicolor NC024569 2	Genetta genetta T297 1	0,15346946
Xenogale naso C07XAR110 1	Diplogale hosei MH464790 1	0,15347438
Leopardus geoffroyi NC028320 1	Urva javanica/auropunctata NC006835 1	0,15349281
Leopardus colocolo NC028314 1	Cynogale bennetti KY117544 1	0,15351996
Prionodon linsang ERR2391707 1	Felis silvestris lybica KP202275 4	0,15352127
Lynx rufus NC014456 3	Urva brachyura KY117547 1	0,15352312
Paradoxurus jerdoni MH464793 1	Lynx canadensis NC028313 1	0,15352577
Otocolobus manul NC028323 1	Urva javanica T413 1	0,15352596
Puma concolor NC016470 22	Bdeogale nigripes GLC15 1	0,15352607
Puma yagouaroundi NC028311 1	Helogale parvula SRR7637809 1	0,15352648
Eupleres goudotii D128 1	Caracal caracal NC028306 1	0,15352805
Prionailurus bengalensis CKM45 20	Urva javanica T413 1	0,15352857
Ursus maritimus NC003428 31	Ailuropoda melanoleuca NC009492 5	0,15353099
Homotherium latidens MF871702 3	Xenogale naso C07XAR110 1	0,15353853
Ichneumia albicauda T603 1	Acinonyx jubatus NC005212 3	0,15354189
Lynx lynx NC027083 4	Diplogale hosei MH464790 1	0,15357073
Homotherium latidens MF871702 3	Bdeogale nigripes GLC15 1	0,15358081
Paradoxurus hermaphroditus NC039591 1	Fossa fossana D350 1	0,15358789
Mungos mungo/gambianus SRR7704821 1	Lynx canadensis NC028313 1	0,15359148
Pardofelis marmorata NLN3 2	Mungos mungo/gambianus SRR7704821 1	0,15359236
Lynx canadensis NC028313 1	Ichneumia albicauda T603 1	0,15359311
Prionailurus viverrinus NC028305 1	Cynictis penicillata T375 1	0,15359397
Puma yagouaroundi NC028311 1	Galerella sanguinea T378 1	0,15359457
Leopardus geoffroyi NC028320 1	Urva brachyura KY117547 1	0,15359493
Salanoia concolor D378 1	Panthera uncia KP202269 1	0,15359795
Mungos mungo/gambianus SRR7704821 1	Felis chaus NC028307 1	0,15366034
Galerella sanguinea T378 1	Felis silvestris lybica KP202275 4	0,1536607
Viverricula indica NC025296 2	Galidia elegans D146 1	0,15366575
Paguma larvata PDDS11 2	Urva brachyura KY117547 1	0,15372404
Paradoxurus hermaphroditus NC039591 1	Lynx canadensis NC028313 1	0,15372734
Otocolobus manul NC028323 1	Mungos mungo MMC7 1	0,15372747
Helogale parvula SRR7637809 1	Felis nigripes NC028309 1	0,15372767
Caracal caracal NC028306 1	Bdeogale nigripes GLC15 1	0,15372868
Leopardus pardalis NC028315 1	Bdeogale nigripes GLC15 1	0,1537288
Leptailurus serval NC028316 1	Bdeogale nigripes GLC15 1	0,15372884
Hyaena hyaena NC020669 1	Bdeogale nigripes GLC15 1	0,15372922
Prionailurus bengalensis CKM45 20	Xenogale naso C07XAR110 1	0,15373041
Prionailurus viverrinus NC028305 1	Galerella sanguinea T378 1	0,15373085
Smilodon populator MF871700 1	Salanoia concolor D378 1	0,15373227

Canis lupus familiaris NC002008 1231	Arctodus simus NC011116 1	0,18615371
Ursus thibetanus mupinensis NC00875	Canis lupus familiaris NC002008 1231	0,18615603
Ursus arctos GU573491 207	Spilogale putorius NC010497 1	0,18615827
Conepatus chinga NC042596 1	Canis adustus KT448271 1	0,1861618
Mustela itatsi NC034330 19	Lycalopex sechurae KT448284 1	0,18616195
Poecilogale albinucha T602 1	Canis mesomelas KT448280 1	0,18616214
Pardofelis marmorata NLN3 2	Eumetopias jubatus NC004030 10	0,18616302
Prionodon linsang ERR2391707 1	Canis mesomelas KT448280 1	0,18616515
Martes americana NC020642 1	Eupleres goudotii D128 1	0,18616778
Cynogale bennetti KY117544 1	Conepatus chinga NC042596 1	0,18616791
Melogale moschata V0735A 1	Xenogale naso C07XAR110 1	0,18616793
Ursus arctos pruinosus MG066703 3	Gulo gulo NC009685 3	0,18616842
Mustela putorius NC020638 4	Atilax paludinosus T606 1	0,18616915
Mustela sibirica AP017394 11	Xenogale naso C07XAR110 1	0,1861694
Mustela eversmannii NC028013 1	Cynictis penicillata T375 1	0,1861698
Mustela sibirica AP017394 11	Cynictis penicillata T375 1	0,18616986
Profelis aurata NC028299 1	Canis mesomelas KT448280 1	0,1861725
Martes americana NC020642 1	Hemigalus derbyanus MH464791 1	0,18617332
Suricata suricatta SSM10 1	Procyon lotor AB462046 3	0,18617371
Panthera tigris NC010642 35	Eumetopias jubatus NC004030 10	0,18617393
Salanoia concolor D378 1	Mustela itatsi NC034330 19	0,18617438
Mustela kathiah NC023210 1	Galidictis fasciata DM333 1	0,18617485
Lynx canadensis NC028313 1	Conepatus chinga NC042596 1	0,18617652
Viverra zibetha T609 1	Arctocephalus townsendi NC008420 1	0,18617655
Cynictis penicillata T375 1	Bassariscus sumichrasti SRX1099089 1	0,18617722
Vulpes vulpes NC008434 5	Paradoxurus jerdoni MH464793 1	0,18617892
Panthera pardus NC010641 5	Canis aureus KT448274 1	0,18617903
Vulpes corsac NC023958 1	Arctodus simus NC011116 1	0,18617968
Mustela altaica NC021751 1	Canis mesomelas KT448280 1	0,18617979
Ursus thibetanus laniger MH281753 2	Canis lupus chanco NC010340 4	0,1861802
Pardofelis marmorata NLN3 2	Mustela itatsi NC034330 19	0,18618174
Martes flavigula NC012141 3	Cuon alpinus NC013445 3	0,18618296
Hyaena hyaena NC020669 1	Aonyx cinerea NC035814 2	0,18618354
Parahyaena brunnea NC038159 15	Aonyx cinerea NC035814 2	0,18618396
Vulpes zerda KJ603240 1	Pardofelis marmorata NLN3 2	0,18618412
Otocolobus manul NC028323 1	Melogale moschata V0735A 1	0,18618447
Lynx canadensis NC028313 1	Arctocephalus gazella BK010918 1	0,18618505
Nyctereutes procyonoides NC013700 3	Felis catus NC001700 2	0,1861864
Procyon lotor AB462046 3	Genetta genetta T297 1	0,18618822
Mungos mungo MMC7 1	Chrysocyon brachyurus NC024172 1	0,18618836
Potos flavus T414 1	Parahyaena brunnea NC038159 15	0,1861885
Panthera uncia KP202269 1	Aonyx cinerea NC035814 2	0,18618858
Zalophus wollebaeki SRR4431565 1	Atilax paludinosus T606 1	0,18618873
Speothos venaticus C48 2	Mustela kathiah NC023210 1	0,18618874
Mustela altaica NC021751 1	Galerella sanguinea T378 1	0,18619068
Puma yagouaroundi NC028311 1	Otocyon megalotis SAF1 2	0,18619213
Otocyon megalotis SAF1 2	Otocolobus manul NC028323 1	0,18619217
Prionailurus planiceps NC028312 6	Otaria byronia OTAB 1	0,18619218
Ursus arctos isabellinus 18B5 2	Paguma larvata PDDS11 2	0,18619245
Odobenus rosmarus NC004029 29	Mustela erminea T305 2	0,18619248
Prionailurus viverrinus NC028305 1	Aonyx cinerea NC035814 2	0,18619248
Mustela itatsi NC034330 19	Chrysocyon brachyurus NC024172 1	0,1861928
Mustela nivalis T306 5	Ichneumia albicauda T603 1	0,18619317
Ursus spelaeus EU327344 13	Lutra lutra NC011358 9	0,18619338
Panthera onca NC022842 1	Mustela sibirica NC020637 6	0,1861937
Genetta abyssinica MG489822 1	Galictis vittata T412 1	0,18619429
Salanoia concolor D378 1	Helarctos malayanus NC009968 2	0,18619622
Ursus arctos GU573491 207	Martes foina NC020643 1	0,18619668
Ursus arctos pruinosus MG066703 3	Genetta servalina NC024568 2	0,18619716
Meles anakuma NC009677 1	Ailuropoda melanoleuca NC009492 5	0,18619874
Genetta abyssinica MG489822 1	Cuon alpinus NC013445 3	0,1861988
Ursus maritimus NC003428 31	Catopuma badia NC028300 1	0,18619977
Zalophus californianus NC008416 1	Urva brachyura KY117547 1	0,18620202
Ursus maritimus GU573488 Svalbard	Arctocephalus forsteri NC004023 28	0,18620664
Diplogale hosei MH464790 1	Canis lupus chanco NC010340 4	0,1862067
Lynx pardinus NC028319 161	Galictis vittata T412 1	0,18620713
Panthera leo NERO 19	Ailurus fulgens styani NC009691 1	0,18620723
Ursus thibetanus laniger MH281753 2	Cryptoprocta ferox CFC13 1	0,18620847
Prionailurus viverrinus NC028305 1	Galictis vittata T412 1	0,18620889
Ursus spelaeus NC011112 8	Prionailurus viverrinus NC028305 1	0,18620905
Melursus ursinus NC009970 2	Felis margarita NC028308 1	0,18620974
Ursus arctos EU497665 29	Acinonyx jubatus NC005212 3	0,18621185
Panthera pardus NC010641 5	Arctonyx collaris NC020645 1	0,18622047
Lutra lutra LC050126 1	Chrotogale owstoni T607 1	0,18622082
Leopardus pardalis NC028315 1	Cuon alpinus NC013445 3	0,18622564
Ursus arctos isabellinus 18B5 2	Spilogale putorius NC010497 1	0,18622564
Paradoxurus jerdoni MH464793 1	Lutra lutra LC050126 1	0,18623078
Lutra lutra LC050126 1	Xenogale naso C07XAR110 1	0,18623132
Enhydra lutris NC009692 1	Cynictis penicillata T375 1	0,18623148
Ursus thibetanus formosanus NC0093:	Canis lupus familiaris NC002008 1231	0,18623196
Helogale parvula SRR7637809 1	Arctonyx collaris NC020645 1	0,18623444
Vulpes vulpes NC008434 5	Enhydra lutris NC009692 1	0,18623595
Fossa fossana D350 1	Bassaricyon neblina SRX1097850 1	0,18623698
Mungos mungo MMC7 1	Ailurus fulgens styani NC009691 1	0,18623698
Mustela eversmannii NC028013 1	Canis mesomelas KT448280 1	0,1862372
Crossarchus platycephalus C7R66 1	Ailurus fulgens styani NC009691 1	0,18623765
Melogale moschata V0735A 1	Galidia elegans D146 1	0,1862382
Meles leucurus NC039173 4	Galidia elegans D146 1	0,18623871
Nyctereutes procyonoides NC013700 3	Urva semitorquata MH464789 1	0,1862392
Nyctereutes procyonoides NC013700 3	Galerella sanguinea T378 1	0,18623944
Mustela altaica NC021751 1	Lycalopex sechurae KT448284 1	0,18624057
Procyon lotor AB462049 4	Phocarctos hookeri NC008418 1	0,18624072
Procyon lotor AB462049 4	Mungos mungo MMC7 1	0,18624103
Panthera tigris amoyensis NC014770 2	Eumetopias jubatus NC004030 10	0,18624134
Diplogale hosei MH464790 1	Chrysocyon brachyurus NC024172 1	0,18624143
Vulpes zerda KJ603240 1	Salanoia concolor D378 1	0,18624175
Suricata suricatta SSM10 1	Bassariscus sumichrasti SRX1099089 1	0,18624207
Tremarctos ornatus NC009969 2	Chrotogale owstoni T607 1	0,18624231
Lontra canadensis SRR10409165 1	Canis adustus KT448271 1	0,18624233
Galictis vittata T412 1	Civettictis civetta NC033378 1	0,18624237
Martes pennanti NC020664 16	Atilax paludinosus T606 1	0,18624301
Lutrogale perspicillata NC035811 1	Arctocephalus forsteri KT693377 17	0,18624307
Lynx lynx NC027083 4	Ailurus fulgens styani NC009691 1	0,18624392

Galidia elegans D146 1	Felis catus NC001700 2	0,15373252
Proteles cristata T393 6	Mungos mungo MMC7 1	0,15373598
Panthera onca NC022842 1	Atilax paludinosus T606 1	0,15373677
Salanoia concolor D378 1	Panthera tigris amoyensis NC014770 2	0,15373819
Ursus arctos AP012576 6	Ailuropoda melanoleuca NC009492 5	0,15374576
Pardofelis marmorata NLN3 2	Diplogale hosei MH464790 1	0,15374732
Panthera uncia KP202269 1	Hemigalus derbyanus MH464791 1	0,15375456
Panthera leo NERO 19	Crossarchus platycephalus C7R66 1	0,15375991
Prionailurus viverrinus NC028305 1	Urva javanica/auropunctata NC006835 1	0,15376057
Leopardus wiedii NC028318 1	Urva javanica/auropunctata NC006835 1	0,1537609
Fossa fossana D350 1	Diplogale hosei MH464790 1	0,15376467
Puma yagouaroundi NC028311 1	Cynogale bennetti KY117544 1	0,15376736
Paradoxurus jerdoni MH464793 1	Crossarchus platycephalus C7R66 1	0,15379092
Prionodon pardicolor NC024569 2	Chrotogale owstoni T607 1	0,15379199
Smilodon populator MF871700 1	Urva brachyura KY117547 1	0,15379338
Profelis aurata NC028299 1	Xenogale naso C07XAR110 1	0,1537948
Leopardus pardalis NC028315 1	Xenogale naso C07XAR110 1	0,15379536
Suricata suricatta SSM10 1	Leopardus wiedii NC028318 1	0,15379572
Prionailurus planiceps KY682741 4	Galerella sanguinea T378 1	0,1537959
Mungotictis decemlineata NC027828 1	Felis chaus NC028307 1	0,15380135
Leopardus tigrinus NC028317 1	Galidia elegans D146 1	0,15380275
Parahyaena brunnea NC038159 15	Leopardus tigrinus NC028317 1	0,15381554
Paguma larvata PDDS11 2	Urva javanica T413 1	0,15385838
Odobenus rosmarus NC004029 29	Arctocephalus forsteri KT693377 17	0,15385886
Prionailurus rubiginosus NC028304 2	Xenogale naso C07XAR110 1	0,15386152
Paradoxurus hermaphroditus NC039591 1	Lynx lynx NC027083 4	0,15386195
Hyaena hyaena NC020669 1	Atilax paludinosus T606 1	0,15386382
Galerella sanguinea T378 1	Caracal caracal NC028306 1	0,15386395
Puma concolor NC016470 22	Eupleres goudotii D128 1	0,15386462
Panthera uncia NC010638 1	Genetta servalina NC024568 2	0,15386553
Panthera onca NC022842 1	Crossarchus platycephalus C7R66 1	0,15387091
Ichneumia albicauda T603 1	Genetta genetta T297 1	0,15387289
Puma concolor NC016470 22	Parahyaena brunnea NC038159 15	0,15387933
Ommatophoca rossii AY377287etc 1	Arctocephalus australis MG023139 1	0,15390785
Panthera tigris NC010642 35	Hemigalus derbyanus MH464791 1	0,15390955
Panthera pardus japonensis KJ866876 8	Chrotogale owstoni T607 1	0,15392548
Mungos mungo MMC7 1	Felis catus NC001700 2	0,1539284
Prionailurus bengalensis NC028301 12	Xenogale naso C07XAR110 1	0,15392891
Pardofelis marmorata NLN3 2	Paradoxurus hermaphroditus NC039591 1	0,15393037
Leopardus geoffroyi NC028320 1	Bdeogale nigripes GLC15 1	0,15393257
Proteles cristata T393 6	Eupleres goudotii D128 1	0,153933
Ursus arctos GU573491 207	Ailuropoda melanoleuca NC009492 5	0,15393505
Prionailurus bengalensis CKM45 20	Ichneumia albicauda T603 1	0,15393513
Panthera tigris NC010642 35	Fossa fossana D350 1	0,15393685
Paradoxurus hermaphroditus NLNC 1	Ichneumia albicauda T603 1	0,15394017
Crossarchus platycephalus C7R66 1	Arctictis binturong T605 2	0,15394136
Galerella sanguinea T378 1	Arctictis binturong T605 2	0,1539529
Paguma larvata PDDS11 2	Urva javanica/auropunctata NC006835 1	0,15395847
Paradoxurus hermaphroditus NLNC 1	Urva semitorquata MH464789 1	0,15399259
Paguma larvata PDDS11 2	Atilax paludinosus T606 1	0,15399283
Lynx pardinus NC028319 161	Cynictis penicillata T375 1	0,15399526
Pardofelis marmorata NLN3 2	Mungos mungo MMC7 1	0,15399578
Pardofelis marmorata NLN3 2	Paradoxurus jerdoni MH464793 1	0,15399793
Paradoxurus hermaphroditus NLNC 1	Panthera onca KP202264 2	0,15399852
Leopardus tigrinus NC028317 1	Helogale parvula SRR7637809 1	0,15399969
Profelis aurata NC028299 1	Ichneumia albicauda T603 1	0,1539997
Proteles cristata T393 6	Urva semitorquata MH464789 1	0,15400279
Panthera tigris amoyensis NC014770 2	Galidictis fasciata DM333 1	0,15400803
Cryptoprocta ferox CFC13 1	Crocuta crocuta NC020670 3	0,15400897
Ursus spelaeus EU327344 13	Ailuropoda melanoleuca NC009492 5	0,15401366
Melursus ursinus NC009970 2	Ailuropoda melanoleuca NC009492 5	0,15401579
Diplogale hosei MH464790 1	Cynictis penicillata T375 1	0,15402769
Leopardus guigna NC028321 1	Urva javanica/auropunctata NC006835 1	0,15403177
Chrotogale owstoni T607 1	Atilax paludinosus T606 1	0,15404694
Prionodon linsang ERR2391707 1	Felis catus NC001700 2	0,15405998
Prionodon linsang ERR2391707 1	Pardofelis marmorata NLN3 2	0,15406001
Prionodon linsang ERR2391707 1	Felis margarita NC028308 1	0,15406023
Profelis aurata NC028299 1	Galerella sanguinea T378 1	0,1540661
Leopardus jacobita NC028322 1	Helogale parvula SRR7637809 1	0,15406705
Panthera uncia KP202269 1	Mungotictis decemlineata NC027828 1	0,15406924
Prionailurus planiceps KY682741 4	Galidia elegans D146 1	0,15407006
Panthera pardus japonensis KJ866876 8	Arctictis binturong T605 2	0,1540803
Smilodon populator MF871700 1	Urva javanica T413 1	0,15409194
Leptailurus serval NC028316 1	Urva javanica/auropunctata NC006835 1	0,15409686
Panthera leo NERO 19	Paguma larvata PDDS11 2	0,15409839
Panthera tigris amoyensis NC014770 2	Hemigalus derbyanus MH464791 1	0,15412369
Paradoxurus hermaphroditus NLNC 1	Urva javanica T413 1	0,15412737
Paradoxurus hermaphroditus NLNC 1	Crossarchus platycephalus C7R66 1	0,15412758
Panthera uncia KP202269 1	Crossarchus platycephalus C7R66 1	0,15413076
Helogale parvula SRR7637809 1	Felis catus NC001700 2	0,15413129
Leopardus tigrinus NC028317 1	Cynictis penicillata T375 1	0,15413211
Galerella sanguinea T378 1	Felis margarita NC028308 1	0,15413243
Diplogale hosei MH464790 1	Bdeogale nigripes GLC15 1	0,15414
Paradoxurus hermaphroditus NLNC 1	Galerella sanguinea T378 1	0,15414034
Mungos mungo MMC7 1	Crocuta crocuta NC020670 3	0,1541431
Parahyaena brunnea NC038159 15	Leptailurus serval NC028316 1	0,15414928
Parahyaena brunnea NC038159 15	Leopardus jacobita NC028322 1	0,15415232
Mungotictis decemlineata NC027828 1	Hemigalus derbyanus MH464791 1	0,15415335
Neofelis nebulosa NC008450 3	Fossa fossana D350 1	0,15416011
Prionodon linsang ERR2391707 1	Caracal caracal NC028306 1	0,15419486
Paguma larvata PDDS11 2	Helogale parvula SRR7637809 1	0,15419513
Panthera leo spelaea KX258452 2	Galidictis fasciata DM333 1	0,15419684
Puma concolor NC016470 22	Cynictis penicillata T375 1	0,15419827
Urva javanica T413 1	Caracal caracal NC028306 1	0,15419894
Suricata suricatta SSM10 1	Prionailurus viverrinus NC028305 1	0,15420084
Panthera onca KP202264 2	Paguma larvata PDDS11 2	0,15420136
Parahyaena brunnea NC038159 15	Urva brachyura KY117547 1	0,15420184
Leopardus guigna NC028321 1	Bdeogale nigripes GLC15 1	0,1542021
Helogale parvula SRR7637809 1	Acinonyx jubatus NC005212 3	0,15421426
Panthera leo NERO 19	Chrotogale owstoni T607 1	0,15422513
Panthera uncia NC010638 1	Genetta abyssinica MG489822 1	0,15423809
Suricata suricatta SSM10 1	Catopuma badia NC028300 1	0,15426512
Lynx canadensis NC028313 1	Bdeogale nigripes GLC15 1	0,15426522

Mustela frenata NC020640 1	Galidia elegans D146 1	0,18624477
Paradoxurus hermaphroditus NLNC 1	Melogale moschata V0735A 1	0,18624492
Viverricula indica NC025296 2	Arctocephalus townsendi NC008420 1	0,18624675
Mustela altaica NC021751 1	Xenogale naso C07XAR110 1	0,18624739
Panthera onca KP202264 2	Mustela eversmannii NC028013 1	0,18624941
Felis margarita NC028308 1	Arctocephalus australis MG023139 1	0,18625036
Mustela nivalis T306 5	Mungotictis decemlineata NC027828 1	0,18625066
Lycaon pictus NC028427 2	Bdeogale nigripes GLC15 1	0,18625109
Panthera onca KP202264 2	Nyctereutes procyonoides NC013700 3	0,186252
Leopardus jacobita NC028322 1	Arctocephalus australis MG023139 1	0,18625247
Prionailurus viverrinus NC028305 1	Arctocephalus australis MG023139 1	0,18625256
Puma concolor NC016470 22	Lontra canadensis SRR10409165 1	0,18625304
Prionodon pardicolor NC024569 2	Potos flavus T414 1	0,18625344
Phocarctos hookeri NC008418 1	Catopuma badia NC028300 1	0,18625373
Panthera tigris NC010642 35	Meles meles T303 3	0,1862544
Nyctereutes procyonoides NC013700 3	Caracal caracal NC028306 1	0,18625495
Viverricula indica KX891745 1	Ursus arctos GU573491 207	0,1862569
Mephitis mephitis NC020648 1	Chrotogale owstoni T607 1	0,1862572
Hyaena hyaena NC020669 1	Arctodus simus NC011116 1	0,18625724
Mustela nivalis T306 5	Galerella sanguinea T378 1	0,18625756
Phoca largha NC008430 1	Paradoxurus hermaphroditus NC03959	0,18625772
Leopardus jacobita NC028322 1	Arctotherium sp NC030174 1	0,18625858
Diplogale hosei MH464790 1	Canis latrans NC008093 7	0,18625948
Otocyon megalotis SAF1 2	Leptailurus serval NC028316 1	0,18625955
Panthera onca NC022842 1	Mustela sibirica AP017394 11	0,18626098
Ursus thibetanus laniger MH281753 2	Prionodon linsang ERR2391707 1	0,1862637
Ursus maritimus GU573488 Svalbard	Catopuma temminckii NC027115 41	0,18626638
Ursus thibetanus mupinensis NC00875	Profelis aurata NC028299 1	0,18626645
Arctonyx collaris NC020645 1	Ailuropoda melanoleuca NC009492 5	0,1862673
Zalophus wollebaeki SRR4431565 1	Puma concolor NC016470 22	0,18626797
Ursus arctos AP012576 6	Phocarctos hookeri NC008418 1	0,18626836
Martes melampus NC009678 1	Hemigalus derbyanus MH464791 1	0,18626894
Prionailurus bengalensis NC028301 12	Mephitis mephitis NC020648 1	0,18627045
Ursus spelaeus EU327344 13	Lynx canadensis NC028313 1	0,18627048
Xenogale naso C07XAR110 1	Canis lupus familiaris NC002008 1231	0,18627287
Mustela sibirica NC020637 6	Urva javanica/auropunctata NC006835	0,18627483
Otocolobus manul NC028323 1	Galictis vittata T412 1	0,18627539
Lutra sumatrana NC035810 1	Urva javanica/auropunctata NC006835	0,18627566
Leopardus jacobita NC028322 1	Galictis vittata T412 1	0,18627582
Melursus ursinus NC009970 2	Felis catus NC001700 2	0,18627618
Odobenus rosmarus NC004029 29	Martes foina NC020643 1	0,1862765
Genetta abyssinica MG489822 1	Tapirus terrestris T358	0,18627761
Ursus arctos isabellinus 18B5 2	Acinonyx jubatus NC005212 3	0,18627872
Panthera leo NERO 19	Canis latrans NC008093 7	0,18628305
Panthera leo NERO 19	Arctonyx collaris NC020645 1	0,18628332
Eupleres goudotii D128 1	Eumetopias jubatus NC004030 10	0,18628469
Prionailurus rubiginosus NC028304 2	Arctocephalus forsteri NC004023 28	0,18628849
Gulo gulo NC009685 3	Caracal caracal NC028306 1	0,18629198
Ursus americanus JX196366 3	Leopardus wiedii NC028318 1	0,18629335
Poecilogale albinucha T602 1	Civettictis civetta NC033378 1	0,18629539
Ichneumia albicauda T603 1	Eumetopias jubatus NC004030 10	0,18629608
Panthera leo spelaea KX258452 2	Canis lupus chanco NC010340 4	0,18629654
Mungos mungo MMC7 1	Martes melampus NC009678 1	0,18629763
Leopardus tigrinus NC028317 1	Canis mesomelas KT448280 1	0,18629836
Paradoxurus hermaphroditus NLNC 1	Lutra lutra LC050126 1	0,18629849
Martes melampus NC009678 1	Bdeogale nigripes GLC15 1	0,18629965
Meles anakuma NC009677 1	Eupleres goudotii D128 1	0,18630053
Lontra canadensis SRR10409165 1	Crossarchus platycephalus C7R66 1	0,18630126
Mungos mungo MMC7 1	Tapirus terrestris T358	0,18630134
Otaria byronia OTAB 1	Civettictis civetta NC033378 1	0,18630193
Melogale moschata KP726273 1	Helogale parvula SRR7637809 1	0,18630205
Mustela itatsi NC034330 19	Cynictis penicillata T375 1	0,18630531
Viverra tangalunga MH464792 1	Bdeogale nigripes GLC15 1	0,18630536
Vulpes ferrilata NC027935 1	Crossarchus platycephalus C7R66 1	0,18630636
Nyctereutes procyonoides NC013700 3	Atilax paludinosus T606 1	0,18630658
Galidictis fasciata DM333 1	Aonyx cinerea NC035814 2	0,18630888
Mustela itatsi NC034330 19	Galidictis fasciata DM333 1	0,18630894
Galidictis fasciata DM333 1	Canis latrans NC008093 7	0,18630968
Spilogale putorius NC010497 1	Helarctos malayanus NC009968 2	0,18631005
Panthera uncia KP202269 1	Lutra lutra NC011358 9	0,18631355
Mustela itatsi NC034330 19	Galerella sanguinea T378 1	0,18631393
Paradoxurus hermaphroditus NC03959	Mustela sibirica NC020637 6	0,1863163
Civettictis civetta NC033378 1	Arctocephalus forsteri NC004023 28	0,18631631
Mustela itatsi NC034330 19	Cryptoprocta ferox CFC13 1	0,18631697
Martes foina NC020643 1	Xenogale naso C07XAR110 1	0,18631718
Vulpes vulpes NC008434 5	Panthera pardus japonensis KJ866876	0,18631734
Otocolobus manul NC028323 1	Arctocephalus townsendi NC008420 1	0,18631764
Leptailurus serval NC028316 1	Arctocephalus forsteri KT693377 17	0,18631792
Lynx rufus NC014456 3	Arctocephalus townsendi NC008420 1	0,18631797
Lynx lynx NC027083 4	Arctocephalus townsendi NC008420 1	0,18631802
Mustela itatsi NC034330 19	Ichneumia albicauda T603 1	0,18631807
Vulpes ferrilata NC027935 1	Lynx lynx NC027083 4	0,18631819
Vulpes ferrilata NC027935 1	Prionailurus viverrinus NC028305 1	0,18631847
Vulpes ferrilata NC027935 1	Leopardus tigrinus NC028317 1	0,18631859
Paradoxurus jerdoni MH464793 1	Martes pennanti NC020664 16	0,18631877
Leopardus tigrinus NC028317 1	Arctocephalus townsendi NC008420 1	0,18631947
Canis latrans NC008093 7	Arctotherium sp NC030174 1	0,18631949
Potos flavus T414 1	Hyaena hyaena NC020669 1	0,18631971
Tremarctos ornatus NC009969 2	Puma concolor NC016470 22	0,18631977
Paradoxurus hermaphroditus NLNC 1	Nyctereutes procyonoides NC013700 3	0,18632038
Felis chaus NC028307 1	Canis anthus NC027956 2	0,18632187
Tremarctos ornatus NC009969 2	Felis chaus NC028307 1	0,18632284
Panthera tigris NC010642 35	Meles anakuma NC009677 1	0,18632405
Ursus arctos EU497665 29	Mustela altaica NC021751 1	0,18632586
Otocyon megalotis SAF1 2	Martes foina NC020643 1	0,1863299
Viverricula indica KX891745 1	Ursus arctos GU573486 5	0,18633085
Lutrogale perspicillata NC035811 1	Leopardus colocolo NC028314 1	0,18633235
Ursus thibetanus mupinensis NC00875	Prionailurus viverrinus NC028305 1	0,18633242
Ursus maritimus GU573488 Svalbard	Catopuma badia NC028300 1	0,18633449
Martes melampus NC009678 1	Urva javanica/auropunctata NC006835	0,18633465
Mustela itatsi NC034330 19	Melursus ursinus NC009970 2	0,18633503
Paradoxurus hermaphroditus NC03959	Ommatophoca rossii AY377287etc 1	0,18633692
Proteles cristata T393 6	Martes foina NC020643 1	0,18633702

Prionailurus planiceps NC028312 6	Xenogale naso C07XAR110 1	0,15426548
Prionailurus viverrinus NC028305 1	Urva brachyura KY117547 1	0,1542666
Nandinia binotata NC024567 1	Hyaena hyaena NC020669 1	0,15426764
Lynx pardinus NC028319 161	Urva javanica/auropunctata NC006835 1	0,15429671
Odobenus rosmarus NC004029 29	Arctocephalus australis MG023139 1	0,15433013
Lynx pardinus NC028319 161	Urva javanica T413 1	0,15433197
Panthera onca KP202264 2	Atilax paludinosus T606 1	0,15433335
Cynictis penicillata T375 1	Catopuma temminckii NC027115 41	0,15433353
Prionailurus viverrinus NC028305 1	Xenogale naso C07XAR110 1	0,15433517
Panthera uncia NC010638 1	Galidictis fasciata DM333 1	0,15433845
Prionailurus planiceps NC028312 6	Galidia elegans D146 1	0,15433951
Galidictis fasciata DM333 1	Felis chaus NC028307 1	0,15434077
Salanoia concolor D378 1	Arctictis binturong T605 2	0,15435179
Leopardus pardalis NC028315 1	Urva javanica/auropunctata NC006835 1	0,15436698
Profelis aurata NC028299 1	Urva javanica/auropunctata NC006835 1	0,15436706
Leopardus jacobita NC028322 1	Cynogale bennetti KY117544 1	0,15437151
Xenogale naso C07XAR110 1	Chrotogale owstoni T607 1	0,15438378
Homotherium latidens MF871702 3	Cynictis penicillata T375 1	0,15439595
Urva javanica T413 1	Felis silvestris lybica KP202275 4	0,15439977
Panthera onca KP202264 2	Crossarchus platycephalus C7R66 1	0,15439989
Puma concolor NC016470 22	Urva javanica T413 1	0,15440018
Leopardus jacobita NC028322 1	Urva javanica T413 1	0,15440144
Salanoia concolor D378 1	Paguma larvata PDDS11 2	0,15440264
Urva javanica/auropunctata NC006835 1	Acinonyx jubatus NC005212 3	0,15445027
Odobenus rosmarus NC004029 29	Arctocephalus gazella BK010918 1	0,1544656
Helogale parvula SRR7637809 1	Genetta genetta T297 1	0,15446633
Suricata suricatta SSM10 1	Lynx lynx NC027083 4	0,1544672
Mungos mungo/gambianus SRR7704821 1	Felis catus NC001700 2	0,15446763
Urva semitorquata MH464789 1	Felis chaus NC028307 1	0,15446863
Suricata suricatta SSM10 1	Leopardus pardalis NC028315 1	0,15446897
Ursus arctos EU497665 29	Ailuropoda melanoleuca NC009492 5	0,15447368
Puma yagouaroundi NC028311 1	Hyaena hyaena NC020669 1	0,15448073
Galerella sanguinea T378 1	Acinonyx jubatus NC005212 3	0,15448397
Prionailurus planiceps NC028312 6	Urva javanica/auropunctata NC006835 1	0,15449981
Neofelis nebulosa NC008450 3	Genetta genetta T297 1	0,15450227
Puma concolor NC016470 22	Urva javanica/auropunctata NC006835 1	0,15450346
Homotherium latidens MF871702 3	Urva brachyura KY117547 1	0,15451176
Panthera pardus NC010641 5	Arctictis binturong T605 2	0,15451397
Galerella sanguinea T378 1	Chrotogale owstoni T607 1	0,15453032
Prionailurus planiceps KY682741 4	Urva javanica T413 1	0,15453477
Prionailurus planiceps NC028312 6	Urva javanica T413 1	0,1545348
Suricata suricatta SSM10 1	Prionailurus planiceps NC028312 6	0,15453506
Panthera uncia NC010638 1	Fossa fossana D350 1	0,1545359
Ichneumia albicauda T603 1	Caracal caracal NC028306 1	0,15453836
Odobenus rosmarus NC004029 29	Mirounga leonina NC008422 1	0,15453944
Panthera onca KP202264 2	Galidictis fasciata DM333 1	0,15454067
Felis chaus NC028307 1	Cryptoprocta ferox CFC13 1	0,15454552
Panthera leo spelaea KX258452 2	Arctictis binturong T605 2	0,1545545
Prionailurus planiceps KY682741 4	Urva javanica/auropunctata NC006835 1	0,15456697
Leptailurus serval NC028316 1	Cynogale bennetti KY117544 1	0,15457518
Suricata suricatta SSM10 1	Diplogale hosei MH464790 1	0,15457973
Urva brachyura KY117547 1	Caracal caracal NC028306 1	0,15460242
Suricata suricatta SSM10 1	Pardofelis marmorata NLN3 2	0,15460251
Profelis aurata NC028299 1	Urva javanica T413 1	0,15460287
Parahyaena brunnea NC038159 15	Nandinia binotata NC024567 1	0,15460695
Salanoia concolor D378 1	Panthera uncia NC010638 1	0,15460798
Parahyaena brunnea NC038159 15	Acinonyx jubatus NC005212 3	0,15463877
Paradoxurus hermaphroditus NC039591 1	Urva semitorquata MH464789 1	0,15466608
Lynx canadensis NC028313 1	Urva javanica T413 1	0,15466803
Lynx rufus NC014456 3	Cynictis penicillata T375 1	0,15466854
Suricata suricatta SSM10 1	Prionailurus planiceps KY682741 4	0,15466973
Paguma larvata PDDS11 2	Galidictis fasciata DM333 1	0,15467229
Puma concolor NC016470 22	Galidia elegans D146 1	0,1546763
Homotherium latidens MF871702 3	Urva javanica/auropunctata NC006835 1	0,15467742
Mungos mungo/gambianus SRR7704821 1	Crocuta crocuta NC020670 3	0,15467809
Panthera uncia NC010638 1	Hemigalus derbyanus MH464791 1	0,15468143
Ichneumia albicauda T603 1	Arctictis binturong T605 2	0,15469515
Prionailurus bengalensis CKM45 20	Urva javanica/auropunctata NC006835 1	0,15470649
Lynx rufus NC014456 3	Diplogale hosei MH464790 1	0,15470666
Galerella sanguinea T378 1	Diplogale hosei MH464790 1	0,15471897
Paradoxurus hermaphroditus NLNC 1	Bdeogale nigripes GLC15 1	0,1547333
Paradoxurus hermaphroditus NLNC 1	Urva brachyura KY117547 1	0,15473361
Felis margarita NC028308 1	Cynictis penicillata T375 1	0,15473677
Viverricula indica KX891751 1	Galidia elegans D146 1	0,15473834
Leopardus jacobita NC028322 1	Xenogale naso C07XAR110 1	0,15473846
Profelis aurata NC028299 1	Eupleres goudotii D128 1	0,15473971
Galidia elegans D146 1	Crocuta crocuta NC020670 3	0,15474284
Panthera tigris amoyensis NC014770 2	Mungotictis decemlineata NC027828 1	0,15474786
Urva brachyura KY117547 1	Arctictis binturong T605 2	0,15474814
Parahyaena brunnea NC038159 15	Catopuma badia NC028300 1	0,15475199
Suricata suricatta SSM10 1	Leopardus colocolo NC028314 1	0,15475528
Puma yagouaroundi NC028311 1	Proteles cristata T393 6	0,15476
Urva javanica/auropunctata NC006835 1	Catopuma temminckii NC027115 41	0,1547702
Paradoxurus hermaphroditus NLNC 1	Xenogale naso C07XAR110 1	0,15480049
Paradoxurus hermaphroditus NLNC 1	Atilax paludinosus T606 1	0,15480056
Paradoxurus jerdoni MH464793 1	Urva semitorquata MH464789 1	0,15480083
Viverricula indica KX891745 1	Cryptoprocta ferox CFC13 1	0,15480439
Otocolobus manul NC028323 1	Helogale parvula SRR7637809 1	0,1548062
Ichneumia albicauda T603 1	Felis silvestris lybica KP202275 4	0,15480633
Viverricula indica KX891751 1	Panthera uncia NC010638 1	0,15480718
Parahyaena brunnea NC038159 15	Felis silvestris lybica KP202275 4	0,15482192
Prionailurus planiceps NC028312 6	Parahyaena brunnea NC038159 15	0,15482232
Odobenus rosmarus NC004029 29	Arctocephalus forsteri NC004023 28	0,15486868
Prionailurus planiceps NC028312 6	Urva brachyura KY117547 1	0,15487085
Felis catus NC001700 2	Cynictis penicillata T375 1	0,15487111
Puma yagouaroundi NC028311 1	Urva javanica T413 1	0,15487215
Genetta genetta T297 1	Cryptoprocta ferox CFC13 1	0,15487249
Paradoxurus hermaphroditus NLNC 1	Panthera pardus japonensis KJ866876 8	0,15487379
Paradoxurus jerdoni MH464793 1	Galerella sanguinea T378 1	0,1548811
Panthera onca NC022842 1	Eupleres goudotii D128 1	0,15488157
Prionodon pardicolor NC024569 2	Panthera pardus japonensis KJ866876 8	0,15488391
Panthera pardus NC010641 5	Chrotogale owstoni T607 1	0,15490209
Leopardus pardalis T262 1	Cynogale bennetti KY117544 1	0,15491015

Martes martes T302 3	Arctictis binturong T605 2	0,18633729
Lycalopex sechurae KT448284 1	Helarctos malayanus NC009968 2	0,18634009
Ursus thibetanus formosanus NC00933	Leptailurus serval NC028316 1	0,18634236
Leopardus jacobita NC028322 1	Helarctos malayanus NC009968 2	0,18634355
Poecilogale albinucha T602 1	Helarctos malayanus NC009968 2	0,18634406
Panthera leo NERO 19	Callorhinus ursinus NC008415 1	0,18634504
Procyon lotor AB462049 4	Canis lupus familiaris NC002008 1231	0,18634641
Ursus arctos EU497665 29	Leopardus colocolo NC028314 1	0,18634859
Meles meles T303 3	Lycalopex sechurae KT448284 1	0,18634869
Ursus maritimus NC003428 31	Leopardus colocolo NC028314 1	0,18634869
Spilogale putorius NC010497 1	Arctotherium sp NC030174 1	0,18634932
Potos flavus T414 1	Urva javanica/auropunctata NC006835	0,18635133
Mephitis mephitis NC020648 1	Leopardus colocolo NC028314 1	0,1863558
Mustela putorius NC020638 4	Lycalopex sechurae KT448284 1	0,18635947
Viverra zibetha T609 1	Spilogale putorius NC010497 1	0,18636237
Felis silvestris lybica KP202275 4	Eumetopias jubatus NC004030 10	0,18636516
Prionodon pardicolor NC024569 2	Cuon alpinus NC013445 3	0,18636656
Mungos mungo MMC7 1	Conepatus chinga NC042596 1	0,18636765
Spilogale putorius NC010497 1	Galictis vittata T412 1	0,18636845
Meles anakuma NC009677 1	Urva brachyura KY117547 1	0,18636891
Leopardus guigna NC028321 1	Canis mesomelas KT448280 1	0,18636982
Viverra tangalunga MH464792 1	Eupleres goudotii D128 1	0,18637049
Tremarctos ornatus NC009969 2	Cynictis penicillata T375 1	0,18637101
Tremarctos ornatus NC009969 2	Mungos mungo MMC7 1	0,18637137
Eupleres goudotii D128 1	Arctocephalus forsteri KT693377 17	0,18637301
Paradoxurus jerdoni MH464793 1	Martes melampus NC009678 1	0,18637444
Spilogale putorius NC010497 1	Chrysocyon brachyurus NC024172 1	0,18637453
Salanoia concolor D378 1	Mustela sibirica AP017394 11	0,1863755
Otaria byronia OTAB 1	Lutrogale perspicillata NC035811 1	0,18637682
Cynictis penicillata T375 1	Arctocephalus pusillus NC008417 1	0,1863777
Chrotogale owstoni T607 1	Canis latrans NC008093 7	0,18637773
Ursus arctos AP012576 6	Civettictis civetta NC033378 1	0,18637812
Lynx lynx NC027083 4	Ailurus fulgens NC011124 1	0,18637879
Procyon lotor AB462046 3	Nyctereutes procyonoides NC013700 3	0,18637961
Vulpes lagopus NC026529 3	Genetta abyssinica MG489822 1	0,1863801
Felis chaus NC028307 1	Conepatus chinga NC042596 1	0,18638127
Mustela itatsi NC034330 19	Hyaena hyaena NC020669 1	0,1863839
Catopuma temminckii NC027115 41	Arctocephalus australis MG023139 1	0,18638475
Enhydra lutris NC009692 1	Canis aureus KT448274 1	0,18638493
Vulpes corsac NC023958 1	Prionailurus planiceps NC028312 6	0,18638572
Lynx lynx NC027083 4	Arctocephalus gazella BK010918 1	0,18638678
Urocyon cinereoargenteus NC026723 2	Lynx pardinus NC028319 161	0,18638687
Ursus thibetanus laniger MH281753 2	Conepatus chinga NC042596 1	0,18638815
Proteles cristata T393 6	Martes americana NC020642 1	0,18638881
Ursus arctos pruinosus MG066703 3	Lutra sumatrana NC035810 1	0,18638881
Zalophus wollebaeki SRR4431565 1	Urva brachyura KY117547 1	0,18638955
Odobenus rosmarus NC004029 29	Mustela frenata NC020640 1	0,18639208
Mephitis mephitis NC020648 1	Melogale moschata V0735A 1	0,18639237
Felis nigripes NC028309 1	Arctotherium sp NC030174 1	0,18639313
Pardofelis marmorata NLN3 2	Arctotherium sp NC030174 1	0,18639316
Prionailurus viverrinus NC028305 1	Arctotherium sp NC030174 1	0,18639384
Otocyon megalotis SAF1 2	Neovison vison NC020641 3	0,18639416
Lycaon pictus NC028427 2	Bassariscus sumichrasti SRX1099089 1	0,18639486
Urva semitorquata MH464789 1	Galictis vittata T412 1	0,18639579
Ursus maritimus NC003428 31	Lynx lynx NC027083 4	0,18639614
Poecilogale albinucha T602 1	Catopuma badia NC028300 1	0,18639621
Ursus maritimus GU573488 Svalbard	Leopardus pardalis NC028315 1	0,18639696
Ursus maritimus NC003428 31	Leopardus pardalis NC028315 1	0,18639699
Prionailurus bengalensis CKM45 20	Otocyon megalotis SAF1 2	0,18639798
Ursus thibetanus laniger MH281753 2	Lontra canadensis SRR10409165 1	0,18639843
Speothos venaticus C48 2	Puma concolor NC016470 22	0,1863995
Zalophus californianus NC008416 1	Lontra canadensis SRR10409165 1	0,18640004
Speothos venaticus C48 2	Caracal caracal NC028306 1	0,1864001
Martes pennanti NC020664 16	Canis mesomelas KT448280 1	0,18640041
Ursus spelaeus EU327344 13	Salanoia concolor D378 1	0,18640077
Ursus arctos pruinosus MG066703 3	Canis adustus KT448271 1	0,18640141
Chrysocyon brachyurus NC024172 1	Arctodus simus NC011116 1	0,18640511
Melursus ursinus NC009970 2	Civettictis civetta GLC19 1	0,18640687
Ursus arctos GU573486 5	Catopuma temminckii NC027115 41	0,18640704
Melogale moschata KP726273 1	Canis lupus familiaris NC002008 1231	0,18640763
Urva javanica T413 1	Canis lupus familiaris NC002008 1231	0,186408
Ursus arctos GU573491 207	Galictis vittata T412 1	0,18640849
Ursus thibetanus laniger MH281753 2	Paradoxurus hermaphroditus NLNC 1	0,186409
Helarctos malayanus NC009968 2	Felis silvestris lybica KP202275 4	0,18641041
Urva semitorquata MH464789 1	Eumetopias jubatus NC004030 10	0,18641683
Panthera onca KP202264 2	Canis lupus familiaris NC002008 1231	0,18642054
Panthera uncia KP202269 1	Canis lupus familiaris NC002008 1231	0,18642193
Ursus americanus JX196366 3	Paradoxurus jerdoni MH464793 1	0,18642637
Ursus americanus JX196366 3	Lynx pardinus NC028319 161	0,18642731
Panthera leo NERO 19	Bassariscus sumichrasti SRX1099089 1	0,186428
Martes flavigula NC012141 3	Ichneumia albicauda T603 1	0,18642886
Prionailurus planiceps KY682741 4	Cuon alpinus NC013445 3	0,1864295
Melursus ursinus NC009970 2	Canis adustus KT448271 1	0,18643099
Mustela kathiah NC023210 1	Lycalopex sechurae KT448284 1	0,18643115
Bdeogale nigripes GLC15 1	Tapirus terrestris T358	0,18643375
Felis nigripes NC028309 1	Eumetopias jubatus NC004030 10	0,18643391
Nasua nasua NC020647 1	Lutrogale perspicillata NC035811 1	0,18643495
Mustela kathiah NC023210 1	Helogale parvula SRR7637809 1	0,18643852
Vulpes vulpes NC008434 5	Melogale moschata KP726273 1	0,18643907
Martes zibellina NC011579 39	Galidia elegans D146 1	0,18644014
Leopardus pardalis NC028315 1	Conepatus chinga NC042596 1	0,18644167
Vulpes vulpes NC008434 5	Mustela sibirica AP017394 11	0,18644284
Procyon lotor AB462046 3	Urva semitorquata MH464789 1	0,18644296
Martes pennanti NC020664 16	Xenogale naso C07XAR110 1	0,18644537
Lutra lutra LC050126 1	Ichneumia albicauda T603 1	0,18644564
Martes pennanti NC020664 16	Cynictis penicillata T375 1	0,18644589
Ursus thibetanus formosanus NC00933	Canis lupus chanco NC010340 4	0,18644667
Viverricula indica KX891751 1	Martes zibellina NC011579 39	0,18644802
Ichneumia albicauda T603 1	Tapirus terrestris T358	0,18644828
Meles leucurus NC039173 4	Hyaena hyaena NC020669 1	0,18644832
Prionodon pardicolor NC024569 2	Conepatus chinga NC042596 1	0,18644987
Prionodon pardicolor NC024569 2	Melogale moschata V0735A 1	0,18645023
Urva brachyura KY117547 1	Arctodus simus NC011116 1	0,18645073

Mungos mungo MMC7 1	Chrotogale owstoni T607 1	0,15491693
Homotherium latidens MF871702 3	Urva javanica T413 1	0,15492646
Panthera onca KP202264 2	Chrotogale owstoni T607 1	0,15493459
Panthera uncia NC010638 1	Crossarchus platycephalus C7R66 1	0,15493898
Panthera onca KP202264 2	Eupleres goudotii D128 1	0,15494047
Taxidea taxus NC020646 1	Bassariscus sumichrasti SRX1099089 1	0,15494402
Prionailurus bengalensis NC028301 12	Galidia elegans D146 1	0,154946
Salanoia concolor D378 1	Panthera tigris NC010642 35	0,15495031
Helogale parvula SRR7637809 1	Arctictis binturong T605 2	0,15495056
Panthera onca NC022842 1	Galidictis fasciata DM333 1	0,15495281
Paradoxurus hermaphroditus NC039591 1	Urva javanica/auropunctata NC006835 1	0,15496697
Ommatophoca rossii AY377287etc 1	Neophoca cinerea NC008419 1	0,15496898
Homotherium latidens MF871702 3	Helogale parvula SRR7637809 1	0,15497735
Diplogale hosei MH464790 1	Crossarchus platycephalus C7R66 1	0,15498111
Parahyaena brunnea NC038159 15	Genetta abyssinica MG489822 1	0,15498441
Mungos mungo MMC7 1	Felis nigripes NC028309 1	0,15500592
Leopardus tigrinus NC028317 1	Urva brachyura KY117547 1	0,15500675
Urva javanica T413 1	Catopuma temminckii NC027115 41	0,15500696
Profelis aurata NC028299 1	Cynictis penicillata T375 1	0,15500706
Felis silvestris lybica KP202275 4	Bdeogale nigripes GLC15 1	0,15500708
Leopardus tigrinus NC028317 1	Ichneumia albicauda T603 1	0,15501022
Galidia elegans D146 1	Caracal caracal NC028306 1	0,15501291
Paradoxurus hermaphroditus NLNC 1	Panthera onca NC022842 1	0,15501752
Proteles cristata T393 6	Cryptoprocta ferox CFC13 1	0,15502165
Parahyaena brunnea NC038159 15	Caracal caracal NC028306 1	0,1550238
Lynx canadensis NC028313 1	Crocuta crocuta NC020670 3	0,1550255
Proteles cristata T393 6	Prionailurus bengalensis CKM45 20	0,15503958
Prionodon pardicolor NC024569 2	Neofelis nebulosa NC008450 3	0,15505284
Smilodon populator MF871700 1	Galidia elegans D146 1	0,15507008
Prionailurus planiceps KY682741 4	Urva brachyura KY117547 1	0,15507276
Felis margarita NC028308 1	Bdeogale nigripes GLC15 1	0,15507463
Pardofelis marmorata NLN3 2	Galerella sanguinea T378 1	0,15507485
Urva semitorquata MH464789 1	Diplogale hosei MH464790 1	0,15507518
Puma concolor NC016470 22	Galerella sanguinea T378 1	0,15507534
Paguma larvata PDDS11 2	Mungotictis decemlineata NC027828 1	0,15507564
Suricata suricatta SSM10 1	Parahyaena brunnea NC038159 15	0,15507852
Prionailurus bengalensis CKM45 20	Galidia elegans D146 1	0,15508382
Panthera tigris NC010642 35	Mungotictis decemlineata NC027828 1	0,15508455
Proteles cristata T393 6	Lynx lynx NC027083 4	0,15509307
Proteles cristata T393 6	Leopardus pardalis NC028315 1	0,15509757
Otocolobus manul NC028323 1	Cynogale bennetti KY117544 1	0,15511443
Prionailurus bengalensis CKM45 20	Cynogale bennetti KY117544 1	0,15511775
Xenogale naso C07XAR110 1	Catopuma temminckii NC027115 41	0,15514034
Prionailurus planiceps KY682741 4	Xenogale naso C07XAR110 1	0,15514086
Prionailurus planiceps NC028312 6	Cynictis penicillata T375 1	0,15514088
Felis chaus NC028307 1	Cynictis penicillata T375 1	0,15514113
Helogale parvula SRR7637809 1	Felis chaus NC028307 1	0,15514193
Panthera uncia NC010638 1	Genetta genetta T297 1	0,15514606
Crocuta crocuta NC020670 3	Atilax paludinosus T606 1	0,15514778
Prionailurus viverrinus NC028305 1	Galidia elegans D146 1	0,15515035
Hyaena hyaena NC020669 1	Caracal caracal NC028306 1	0,1551531
Suricata suricatta SSM10 1	Homotherium latidens MF871702 3	0,15520225
Paradoxurus jerdoni MH464793 1	Mungos mungo/gambianus SRR7704821 1	0,15520479
Galerella sanguinea T378 1	Felis catus NC001700 2	0,15520933
Suricata suricatta SSM10 1	Hyaena hyaena NC020669 1	0,15521106
Paradoxurus hermaphroditus NLNC 1	Galidictis fasciata DM333 1	0,15521157
Panthera tigris NC010642 35	Galidictis fasciata DM333 1	0,15522012
Urva brachyura KY117547 1	Acinonyx jubatus NC005212 3	0,15522207
Urva brachyura KY117547 1	Diplogale hosei MH464790 1	0,1552238
Urva javanica/auropunctata NC006835 1	Chrotogale owstoni T607 1	0,1552275
Viverricula indica KX891751 1	Neofelis nebulosa NC008450 3	0,15524186
Leopardus jacobita NC028322 1	Urva javanica/auropunctata NC006835 1	0,15524291
Urva brachyura KY117547 1	Felis margarita NC028308 1	0,15527487
Suricata suricatta SSM10 1	Catopuma temminckii NC027115 41	0,15527539
Odobenus rosmarus NC004029 29	Arctocephalus pusillus NC008417 1	0,15527686
Proteles cristata T393 6	Galerella sanguinea T378 1	0,15528248
Panthera tigris NC010642 35	Paguma larvata PDDS11 2	0,15528442
Parahyaena brunnea NC038159 15	Homotherium latidens MF871702 3	0,15529053
Paradoxurus jerdoni MH464793 1	Urva javanica/auropunctata NC006835 1	0,15530364
Leopardus wiedii NC028318 1	Cynogale bennetti KY117544 1	0,1553141
Otocolobus manul NC028323 1	Cynictis penicillata T375 1	0,15534395
Panthera onca NC022842 1	Chrotogale owstoni T607 1	0,15534699
Panthera tigris NC010642 35	Crossarchus platycephalus C7R66 1	0,15534978
Lynx lynx NC027083 4	Crocuta crocuta NC020670 3	0,15536183
Proteles cristata T393 6	Prionailurus rubiginosus NC028304 2	0,15536578
Lynx pardinus NC028319 161	Urva brachyura KY117547 1	0,15540865
Lynx lynx NC027083 4	Xenogale naso C07XAR110 1	0,15540977
Panthera onca KP202264 2	Urva semitorquata MH464789 1	0,15540993
Felis nigripes NC028309 1	Cynictis penicillata T375 1	0,15540999
Hyaena hyaena NC020669 1	Urva javanica T413 1	0,15541029
Salanoia concolor D378 1	Panthera onca KP202264 2	0,15541607
Paradoxurus jerdoni MH464793 1	Ichneumia albicauda T603 1	0,1554216
Profelis aurata NC028299 1	Hyaena hyaena NC020669 1	0,15542184
Urva javanica T413 1	Acinonyx jubatus NC005212 3	0,15542468
Suricata suricatta SSM10 1	Acinonyx jubatus NC005212 3	0,15542495
Ichneumia albicauda T603 1	Homotherium latidens MF871702 3	0,15544762
Mungos mungo/gambianus SRR7704821 1	Diplogale hosei MH464790 1	0,15546586
Bassariscus sumichrasti SRX1099089 1	Bassaricyon neblina SRX1097850 1	0,15547214
Puma concolor NC016470 22	Xenogale naso C07XAR110 1	0,1554776
Leopardus guigna NC028321 1	Xenogale naso C07XAR110 1	0,15547965
Ichneumia albicauda T603 1	Felis chaus NC028307 1	0,15548038
Leptailurus serval NC028316 1	Galidia elegans D146 1	0,15548538
Nandinia binotata NC024567 1	Crocuta crocuta NC020670 3	0,15548539
Panthera tigris amoyensis NC014770 2	Atilax paludinosus T606 1	0,15548612
Panthera tigris NC010642 35	Atilax paludinosus T606 1	0,15548632
Panthera onca NC022842 1	Urva semitorquata MH464789 1	0,15548688
Leptailurus serval NC028316 1	Hyaena hyaena NC020669 1	0,15549061
Otocolobus manul NC028323 1	Urva javanica/auropunctata NC006835 1	0,15551142
Prionodon pardicolor NC024569 2	Panthera pardus NC010641 5	0,15552095
Urva brachyura KY117547 1	Genetta genetta T297 1	0,15554358
Leptailurus serval NC028316 1	Helogale parvula SRR7637809 1	0,15554677
Leopardus tigrinus NC028317 1	Bdeogale nigripes GLC15 1	0,15554745
Proteles cristata T393 6	Mungos mungo/gambianus SRR7704821 1	0,15555184

Mustela sibirica NC020637 6	Cryptoprocta ferox CFC13 1	0,18645087
Galerella sanguinea T378 1	Arctocephalus gazella BK010918 1	0,18645102
Panthera onca KP202264 2	Melogale moschata V0735A 1	0,18645217
Panthera onca KP202264 2	Lutra sumatrana NC035810 1	0,18645222
Tremarctos ornatus NC009969 2	Ichneumia albicauda T603 1	0,18645276
Vulpes lagopus NC026529 3	Lynx canadensis NC028313 1	0,18645294
Galidia elegans D146 1	Arctodus simus NC011116 1	0,18645371
Parahyaena brunnea NC038159 15	Mustela sibirica NC020637 6	0,18645433
Ursus arctos EU497665 29	Phocarctos hookeri NC008418 1	0,18645461
Phocarctos hookeri NC008418 1	Ictonyx striatus T299 1	0,18645526
Pardofelis marmorata NLN3 2	Nyctereutes procyonoides NC013700 3	0,18645577
Neophoca cinerea NC008419 1	Felis silvestris lybica KP202275 4	0,18645608
Ursus arctos pruinosus MG066703 3	Arctocephalus forsteri KT693377 17	0,18645765
Ursus arctos pruinosus MG066703 3	Bassariscus sumichrasti SRX1099089 1	0,18645859
Martes pennanti NC020664 16	Caracal caracal NC028306 1	0,18646009
Otaria byronia OTAB 1	Civettictis civetta GLC19 1	0,18646035
Felis margarita NC028308 1	Arctotherium sp NC030174 1	0,18646049
Otaria byronia OTAB 1	Genetta servalina NC024568 2	0,18646079
Viverricula indica NC025296 2	Ursus maritimus GU573488 Svalbard	0,18646111
Prionailurus rubiginosus NC028304 2	Otocyon megalotis SAF1 2	0,18646166
Panthera leo spelaea KX258452 2	Arctonyx collaris NC020645 1	0,18646175
Procyon lotor AB462049 4	Catopuma temminckii NC027115 41	0,1864621
Ursus thibetanus laniger MH281753 2	Mungos mungo MMC7 1	0,18646304
Ursus arctos isabellinus 18B5 2	Leopardus pardalis T262 1	0,18646398
Ursus arctos EU497665 29	Leopardus pardalis NC028315 1	0,18646425
Profelis aurata NC028299 1	Neovison vison NC020641 3	0,18646577
Ursus maritimus NC003428 31	Profelis aurata NC028299 1	0,18646605
Ursus maritimus NC003428 31	Puma yagouaroundi NC028311 1	0,18646614
Speothos venaticus C48 2	Leptailurus serval NC028316 1	0,18646763
Zalophus californianus NC008416 1	Atilax paludinosus T606 1	0,1864713
Tremarctos ornatus NC009969 2	Leopardus colocolo NC028314 1	0,18647243
Ursus spelaeus EU327344 13	Leopardus jacobita NC028322 1	0,18647264
Viverra zibetha T609 1	Galictis vittata T412 1	0,18647324
Ursus maritimus NC003428 31	Arctocephalus forsteri NC004023 28	0,18647657
Leptailurus serval NC028316 1	Helarctos malayanus NC009968 2	0,1864803
Ursus arctos GU573486 5	Ictonyx striatus T299 1	0,18648143
Ursus maritimus GU573488 Svalbard	Leopardus colocolo NC028314 1	0,18648331
Eumetopias jubatus NC004030 10	Atilax paludinosus T606 1	0,18648593
Panthera leo NERO 19	Mustela putorius NC020638 4	0,18648666
Panthera pardus NC010641 5	Ailurus fulgens styani NC009691 1	0,18648712
Poecilogale albinucha T602 1	Canis lupus chanco NC010340 4	0,1864879
Melursus ursinus NC009970 2	Galictis vittata T412 1	0,18648818
Ursus spelaeus NC011112 8	Galictis vittata T412 1	0,18649004
Gulo gulo NC009685 3	Canis aureus KT448274 1	0,18649542
Phocarctos hookeri NC008418 1	Civettictis civetta NC033378 1	0,18649562
Zalophus wollebaeki SRR4431565 1	Spilogale putorius NC010497 1	0,18649563
Ursus americanus JX196366 3	Puma yagouaroundi NC028311 1	0,18649711
Pusa sibirica NC008432 2	Cynogale bennetti KY117544 1	0,18649839
Lutra lutra NC011358 9	Bdeogale nigripes GLC15 1	0,18649943
Urva brachyura KY117547 1	Enhydra lutris NC009692 1	0,18649949
Mungos mungo MMC7 1	Melogale moschata V0735A 1	0,18650398
Melogale moschata V0735A 1	Atilax paludinosus T606 1	0,18650429
Nyctereutes procyonoides NC013700 3	Martes melampus NC009678 1	0,18650548
Prionodon linsang ERR2391707 1	Meles anakuma NC009677 1	0,18650676
Martes americana NC020642 1	Canis anthus NC027956 2	0,18650772
Lutra sumatrana NC035810 1	Canis latrans NC008093 7	0,18650797
Nyctereutes procyonoides NC013700 3	Mungos mungo/gambianus SRR77048	0,18650877
Suricata suricatta SSM10 1	Procyon lotor AB462049 4	0,18651032
Vulpes vulpes NC008434 5	Mustela putorius NC020638 4	0,18651043
Xenogale naso C07XAR110 1	Arctocephalus pusillus NC008417 1	0,18651129
Hemigalus derbyanus MH464791 1	Arctocephalus forsteri NC004023 28	0,18651174
Mustela erminea T305 2	Galidia elegans D146 1	0,18651216
Lynx canadensis NC028313 1	Ailurus fulgens NC011124 1	0,18651362
Phoca vitulina NC001325 1	Tapirus terrestris T358	0,18651566
Vulpes vulpes NC008434 5	Arctodus simus NC011116 1	0,18651582
Prionodon linsang ERR2391707 1	Monachus schauinslandi NC008421 1	0,18651775
Mustela altaica NC021751 1	Canis anthus NC027956 2	0,18651819
Ursus arctos isabellinus 18B5 2	Fossa fossana D350 1	0,18651842
Mustela putorius NC020638 4	Hyaena hyaena NC020669 1	0,18651851
Panthera leo NERO 19	Canis lupus familiaris NC002008 1231	0,18651868
Martes foina NC020643 1	Urva brachyura KY117547 1	0,186519
Panthera uncia NC010638 1	Lutra sumatrana NC035810 1	0,18651946
Leptailurus serval NC028316 1	Arctocephalus australis MG023139 1	0,18651989
Neophoca cinerea NC008419 1	Genetta servalina NC024568 2	0,18651991
Vulpes corsac NC023958 1	Lynx lynx NC027083 4	0,18651992
Parahyaena brunnea NC038159 15	Callorhinus ursinus NC008415 1	0,1865201
Ictonyx striatus T299 1	Atilax paludinosus T606 1	0,18652019
Catopuma temminckii NC027115 41	Arctocephalus gazella BK010918 1	0,18652053
Vulpes ferrilata NC027935 1	Puma concolor NC016470 22	0,18652076
Martes foina NC020643 1	Canis anthus NC027956 2	0,18652098
Paradoxurus hermaphroditus NLNC 1	Martes pennanti NC020664 16	0,18652114
Viverricula indica NC025296 2	Neophoca cinerea NC008419 1	0,18652399
Odobenus rosmarus NC004029 29	Ailurus fulgens styani NC009691 1	0,18652438
Martes melampus NC009678 1	Arctictis binturong T605 2	0,18652481
Viverra tangalunga MH464792 1	Ursus arctos pruinosus MG066703 3	0,18652543
Panthera leo spelaea KX258452 2	Ommatophoca rossii AY377287etc 1	0,18652549
Meles meles T303 3	Chrysocyon brachyurus NC024172 1	0,18652715
Panthera pardus NC010641 5	Canis lupus chanco NC010340 4	0,18652792
Prionodon linsang ERR2391707 1	Chrysocyon brachyurus NC024172 1	0,18652824
Viverricula indica NC025296 2	Ursus maritimus NC003428 31	0,18652849
Ursus maritimus NC003428 31	Paguma larvata PDDS11 2	0,1865288
Prionailurus bengalensis NC028301 12	Otocyon megalotis SAF1 2	0,1865291
Panthera onca NC022842 1	Lutra sumatrana NC035810 1	0,18653078
Leopardus guigna NC028321 1	Arctotherium sp NC030174 1	0,18653181
Ursus arctos EU497665 29	Leopardus pardalis T262 1	0,18653183
Speothos venaticus C48 2	Felis silvestris lybica KP202275 4	0,18653345
Odobenus rosmarus NC004029 29	Martes americana NC020642 1	0,18653356
Ursus thibetanus formosanus NC0093	Procyon lotor AB462049 4	0,18653427
Martes americana NC020642 1	Ailuropoda melanoleuca NC009492 5	0,18653524
Zalophus wollebaeki SRR4431565 1	Felis chaus NC028307 1	0,18653658
Leopardus colocolo NC028314 1	Arctocephalus forsteri KT693377 17	0,18654045
Helogale parvula SRR7637809 1	Canis lupus familiaris NC002008 1231	0,18654165
Nyctereutes procyonoides NC013700 3	Urva javanica/auropunctata NC006835	0,18654236

Urva semitorquata MH464789 1	Crocuta crocuta NC020670 3	0,15555264
Otocolobus manul NC028323 1	Hyaena hyaena NC020669 1	0,15555786
Paradoxurus hermaphroditus NLNC 1	Panthera pardus NC010641 5	0,15557683
Profelis aurata NC028299 1	Cynogale bennetti KY117544 1	0,15558484
Lynx pardinus NC028319 161	Xenogale naso C07XAR110 1	0,15561196
Urva javanica T413 1	Felis margarita NC028308 1	0,15561216
Otocolobus manul NC028323 1	Urva brachyura KY117547 1	0,15561262
Leopardus jacobita NC028322 1	Urva brachyura KY117547 1	0,1556128
Leopardus jacobita NC028322 1	Bdeogale nigripes GLC15 1	0,15561482
Panthera tigris amoyensis NC014770 2	Crossarchus platycephalus C7R66 1	0,15561911
Panthera pardus japonensis KJ866876 8	Cryptoprocta ferox CFC13 1	0,15562236
Ursus arctos pruinosus MG066703 3	Ailuropoda melanoleuca NC009492 5	0,15562434
Pardofelis marmorata NLN3 2	Parahyaena brunnea NC038159 15	0,15562923
Panthera onca NC022842 1	Cryptoprocta ferox CFC13 1	0,15563014
Mungotictis decemlineata NC027828 1	Arctictis binturong T605 2	0,15563072
Proteles cristata T393 6	Lynx pardinus NC028319 161	0,156329
Panthera onca NC022842 1	Arctictis binturong T605 2	0,15563801
Panthera leo NERO 19	Eupleres goudotii D128 1	0,1556444
Odobenus rosmarus NC004029 29	Arctocephalus townsendi NC008420 1	0,15567672
Lynx canadensis NC028313 1	Urva brachyura KY117547 1	0,15567741
Urva javanica T413 1	Felis catus NC001700 2	0,15567906
Puma yagouaroundi NC028311 1	Bdeogale nigripes GLC15 1	0,15568131
Urva javanica T413 1	Crocuta crocuta NC020670 3	0,15568648
Otocolobus manul NC028323 1	Galidia elegans D146 1	0,15568733
Hyaena hyaena NC020669 1	Catopuma temminckii NC027115 41	0,15569074
Atilax paludinosus T606 1	Arctictis binturong T605 2	0,15569115
Panthera leo NERO 19	Mungos mungo MMC7 1	0,15569785
Prionailurus bengalensis NC028301 12	Crocuta crocuta NC020670 3	0,15570231
Lynx canadensis NC028313 1	Urva javanica/auropunctata NC006835 1	0,15571065
Panthera uncia KP202269 1	Chrotogale owstoni T607 1	0,15574287
Odobenus rosmarus NC004029 29	Neophoca cinerea NC008419 1	0,15574618
Suricata suricatta SSM10 1	Lynx pardinus NC028319 161	0,15574682
Mungos mungo/gambianus SRR7704821 1	Felis nigripes NC028309 1	0,15574737
Galerella sanguinea T378 1	Felis nigripes NC028309 1	0,1557484
Panthera tigris amoyensis NC014770 2	Paguma larvata PDDS11 2	0,15575559
Salanoia concolor D378 1	Panthera onca NC022842 1	0,15576093
Parahyaena brunnea NC038159 15	Panthera onca KP202264 2	0,15576252
Parahyaena brunnea NC038159 15	Felis margarita NC028308 1	0,15576485
Proteles cristata T393 6	Lynx canadensis NC028313 1	0,15576655
Leopardus pardalis T262 1	Crocuta crocuta NC020670 3	0,1557717
Homotherium latidens MF871702 3	Eupleres goudotii D128 1	0,15577519
Smilodon populator MF871700 1	Hyaena hyaena NC020669 1	0,15577751
Leopardus tigrinus NC028317 1	Urva javanica/auropunctata NC006835 1	0,15578078
Puma yagouaroundi NC028311 1	Urva javanica/auropunctata NC006835 1	0,15578096
Urva javanica/auropunctata NC006835 1	Felis margarita NC028308 1	0,15578394
Panthera pardus NC010641 5	Eupleres goudotii D128 1	0,1557847
Leopardus geoffroyi NC028320 1	Cynogale bennetti KY117544 1	0,15579583
Otocolobus manul NC028323 1	Bdeogale nigripes GLC15 1	0,15581639
Ichneumia albicauda T603 1	Felis margarita NC028308 1	0,15581667
Prionailurus viverrinus NC028305 1	Ichneumia albicauda T603 1	0,15581875
Pardofelis marmorata NLN3 2	Galidia elegans D146 1	0,15581989
Panthera uncia NC010638 1	Mungotictis decemlineata NC027828 1	0,15581995
Bdeogale nigripes GLC15 1	Arctictis binturong T605 2	0,15582529
Homotherium latidens MF871702 3	Cryptoprocta ferox CFC13 1	0,15582728
Paguma larvata PDDS11 2	Neofelis nebulosa NC008450 3	0,1558494
Urva javanica/auropunctata NC006835 1	Felis silvestris lybica KP202275 4	0,15585111
Nandinia binotata NC024567 1	Cynogale bennetti KY117544 1	0,15586887
Panthera pardus japonensis KJ866876 8	Helogale parvula SRR7637809 1	0,15588158
Hyaena hyaena NC020669 1	Urva brachyura KY117547 1	0,15588202
Leopardus geoffroyi NC028320 1	Xenogale naso C07XAR110 1	0,15588365
Salanoia concolor D378 1	Paradoxurus hermaphroditus NLNC 1	0,15588467
Prionodon linsang ERR2391707 1	Urva javanica/auropunctata NC006835 1	0,15590477
Ommatophoca rossii AY377287etc 1	Arctocephalus townsendi NC008420 1	0,15591522
Ursus americanus JX196366 3	Ailuropoda melanoleuca NC009492 5	0,15591681
Puma concolor NC016470 22	Cynogale bennetti KY117544 1	0,155922
Panthera leo NERO 19	Cryptoprocta ferox CFC13 1	0,15592676
Eupleres goudotii D128 1	Chrotogale owstoni T607 1	0,15593144
Panthera leo spelaea KX258452 2	Chrotogale owstoni T607 1	0,1559469
Lynx lynx NC027083 4	Urva javanica T413 1	0,1559473
Prionailurus planiceps KY682741 4	Cynictis penicillata T375 1	0,15594885
Panthera leo spelaea KX258452 2	Crossarchus platycephalus C7R66 1	0,15595056
Pardofelis marmorata NLN3 2	Ichneumia albicauda T603 1	0,15595111
Panthera tigris amoyensis NC014770 2	Urva semitorquata MH464789 1	0,1559561
Xenogale naso C07XAR110 1	Acinonyx jubatus NC005212 3	0,15596353
Panthera pardus NC010641 5	Paguma larvata PDDS11 2	0,15598284
Felis chaus NC028307 1	Cynogale bennetti KY117544 1	0,15598811
Prionailurus rubiginosus NC028304 2	Cynogale bennetti KY117544 1	0,15598963
Prionodon pardicolor NC024569 2	Panthera leo NERO 19	0,15599027
Puma yagouaroundi NC028311 1	Xenogale naso C07XAR110 1	0,15601623
Pardofelis marmorata NLN3 2	Bdeogale nigripes GLC15 1	0,1560168
Panthera onca KP202264 2	Galidia elegans D146 1	0,15602152
Smilodon populator MF871700 1	Xenogale naso C07XAR110 1	0,15602323
Panthera onca NC022842 1	Mungotictis decemlineata NC027828 1	0,15602972
Prionailurus planiceps KY682741 4	Parahyaena brunnea NC038159 15	0,1560345
Leopardus pardalis NC028315 1	Crocuta crocuta NC020670 3	0,15604076
Proteles cristata T393 6	Leopardus pardalis T262 1	0,15604104
Panthera pardus NC010641 5	Crossarchus platycephalus C7R66 1	0,15604949
Urva brachyura KY117547 1	Felis silvestris lybica KP202275 4	0,15608257
Smilodon populator MF871700 1	Parahyaena brunnea NC038159 15	0,1560829
Panthera uncia KP202269 1	Eupleres goudotii D128 1	0,15608537
Panthera onca NC022842 1	Urva javanica T413 1	0,15609188
Panthera onca KP202264 2	Cryptoprocta ferox CFC13 1	0,15609354
Proteles cristata T393 6	Prionailurus planiceps NC028312 6	0,15610589
Panthera leo NERO 19	Mungos mungo/gambianus SRR7704821 1	0,1561133
Panthera pardus NC010641 5	Helogale parvula SRR7637809 1	0,15611655
Urva javanica T413 1	Chrotogale owstoni T607 1	0,15613352
Paradoxurus jerdoni MH464793 1	Bdeogale nigripes GLC15 1	0,15614738
Viverricula indica NC025296 2	Hyaena hyaena NC020669 1	0,15614998
Felis nigripes NC028309 1	Bdeogale nigripes GLC15 1	0,15615176
Panthera tigris amoyensis NC014770 2	Chrotogale owstoni T607 1	0,1561541
Odobenus rosmarus NC004029 29	Leptonychotes weddellii NC008424 1	0,1561577
Prionailurus planiceps NC028312 6	Hyaena hyaena NC020669 1	0,15616474
Panthera onca KP202264 2	Arctictis binturong T605 2	0,15616795

Ursus arctos GU573491 207	Arctictis binturong T605 2	0,18654236
Canis lupus familiaris NC002008 1231	Ailurus fulgens styani NC009691 1	0,18654358
Zalophus wollebaeki SRR4431565 1	Panthera tigris amoyensis NC014770 2	0,18654406
Ursus spelaeus EU327344 13	Leopardus guigna NC028321 1	0,18654418
Ursus thibetanus formosanus NC0093	Prionailurus bengalensis NC028301 12	0,18654418
Ursus spelaeus NC011112 8	Felis nigripes NC028309 1	0,1865447
Melursus ursinus NC009970 2	Caracal caracal NC028306 1	0,18654825
Melursus ursinus NC009970 2	Leopardus guigna NC028321 1	0,18654945
Panthera leo NERO 19	Meles meles T303 3	0,18655042
Mustela eversmannii NC028013 1	Cuon alpinus NC013445 3	0,18655574
Puma concolor NC016470 22	Gulo gulo NC009685 3	0,18656084
Panthera onca KP202264 2	Gulo gulo NC009685 3	0,18656307
Lutra lutra NC011358 9	Urva brachyura KY117547 1	0,18656461
Panthera leo spelaea KX258452 2	Canis lupus familiaris NC002008 1231	0,18656792
Martes flavigula NC012141 3	Hyaena hyaena NC020669 1	0,18656848
Mungos mungo/gambianus SRR77048	Meles anakuma NC009677 1	0,18656908
Meles leucurus NC039173 4	Helogale parvula SRR7637809 1	0,18657003
Mustela sibirica NC020637 6	Mungos mungo/gambianus SRR77048	0,18657194
Mustela putorius NC020638 4	Eupleres goudotii D128 1	0,18657205
Martes zibellina NC011579 39	Chrotogale owstoni T607 1	0,18657325
Vulpes lagopus NC026529 3	Martes melampus NC009678 1	0,18657352
Vulpes zerda KJ603240 1	Martes melampus NC009678 1	0,18657523
Vulpes vulpes NC008434 5	Mustela sibirica NC020637 6	0,18657768
Nyctereutes procyonoides NC013700 3	Mustela sibirica NC020637 6	0,18657848
Neophoca cinerea NC008419 1	Galidictis fasciata DM333 1	0,1865789
Bdeogale nigripes GLC15 1	Arctocephalus pusillus NC008417 1	0,18657926
Martes flavigula NC012141 3	Ailuropoda melanoleuca NC009492 5	0,18658027
Vulpes vulpes NC008434 5	Taxidea taxus NC020646 1	0,18658088
Ursus americanus JX196366 3	Leopardus colocolo NC028314 1	0,18658167
Felis catus NC001700 2	Conepatus chinga NC042596 1	0,18658218
Mustela altaica NC021751 1	Urva brachyura KY117547 1	0,18658261
Conepatus chinga NC042596 1	Catopuma temminckii NC027115 41	0,18658356
Mustela altaica NC021751 1	Urva semitorquata MH464789 1	0,18658394
Galerella sanguinea T378 1	Arctocephalus forsteri KT693377 17	0,18658398
Panthera pardus japonensis KJ866876	Arctonyx collaris NC020645 1	0,18658444
Poecilogale albinucha T602 1	Mungos mungo MMC7 1	0,18658489
Vulpes zerda KJ603240 1	Melogale moschata NC020644 1	0,18658496
Meles leucurus NC039173 4	Canis aureus KT448274 1	0,18658514
Panthera onca KP202264 2	Mustela sibirica NC020637 6	0,18658602
Prionailurus planiceps NC028312 6	Arctocephalus townsendi NC008420 1	0,18658683
Ictonyx striatus T299 1	Xenogale naso C07XAR110 1	0,18658808
Leopardus pardalis T262 1	Arctocephalus townsendi NC008420 1	0,18658848
Vulpes lagopus NC026529 3	Otocolobus manul NC028323 1	0,18658857
Vulpes zerda KJ603240 1	Puma concolor NC016470 22	0,18658872
Parahyaena brunnea NC038159 15	Bassariscus sumichrasti SRX1099089 1	0,18658908
Pardofelis marmorata NLN3 2	Canis anthus NC027956 2	0,18658955
Puma yagouaroundi NC028311 1	Phocarctos hookeri NC008418 1	0,18659122
Vulpes zerda KJ603240 1	Leopardus guigna NC028321 1	0,18659122
Lynx rufus NC014456 3	Bassaricyon neblina SRX1097850 1	0,18659213
Vulpes lagopus NC026529 3	Panthera tigris NC010642 35	0,18659557
Parahyaena brunnea NC038159 15	Melogale moschata NC020644 1	0,1865966
Otocyon megalotis SAF1 2	Catopuma temminckii NC027115 41	0,18659711
Profelis aurata NC028299 1	Mustela nivalis T306 5	0,18659751
Chrysocyon brachyurus NC024172 1	Ailurus fulgens NC011124 1	0,18659928
Panthera pardus NC010641 5	Canis lupus familiaris NC002008 1231	0,18659931
Panthera tigris amoyensis NC014770 2	Arctocephalus pusillus NC008417 1	0,18660043
Ursus maritimus GU573488 Svalbard	Profelis aurata NC028299 1	0,18660076
Ursus maritimus GU573488 Svalbard	Puma yagouaroundi NC028311 1	0,18660079
Ursus arctos EU497665 29	Puma yagouaroundi NC028311 1	0,18660085
Leopardus jacobita NC028322 1	Canis aureus KT448274 1	0,18660206
Mephitis mephitis NC020648 1	Martes zibellina NC011579 39	0,18660212
Ursus arctos isabellinus 18B5 2	Catopuma badia NC028300 1	0,18660361
Ursus thibetanus laniger MH281753 2	Procyon lotor AB462046 3	0,18660428
Panthera tigris amoyensis NC014770 2	Melogale moschata NC020644 1	0,1866044
Mephitis mephitis NC020648 1	Arctotherium sp NC030174 1	0,18660527
Vulpes zerda KJ603240 1	Acinonyx jubatus NC005212 3	0,18660565
Martes foina NC020643 1	Ailuropoda melanoleuca NC009492 5	0,18660611
Ommatophoca rossii AY377287etc 1	Neofelis nebulosa NC008450 3	0,18660651
Chrysocyon brachyurus NC024172 1	Bassariscus sumichrasti SRX1099089 1	0,18660656
Ursus arctos GU573486 5	Profelis aurata NC028299 1	0,18660694
Viverricula indica KX891745 1	Melursus ursinus NC009970 2	0,18660921
Viverricula indica KX891751 1	Melursus ursinus NC009970 2	0,18660949
Mungotictis decemlineata NC027828 1	Canis aureus KT448274 1	0,18660967
Lutra sumatrana NC035810 1	Canis lupus familiaris NC002008 1231	0,18660991
Bassaricyon neblina SRX1097850 1	Arctocephalus forsteri NC004023 28	0,18661007
Ursus arctos pruinosus MG066703 3	Galictis vittata T412 1	0,18661045
Ursus maritimus NC003428 31	Galictis vittata T412 1	0,18661049
Lycalopex sechurae KT448284 1	Gulo gulo NC009685 3	0,18661079
Prionailurus planiceps NC028312 6	Galictis vittata T412 1	0,18661195
Salanoia concolor D378 1	Canis lupus familiaris NC002008 1231	0,18661235
Melursus ursinus NC009970 2	Lynx pardinus NC028319 161	0,18661237
Panthera onca KP202264 2	Canis lupus chanco NC010340 4	0,18661307
Prionodon pardicolor NC024569 2	Helarctos malayanus NC009968 2	0,18661596
Melogale moschata KP726273 1	Canis lupus chanco NC010340 4	0,18661615
Ursus arctos GU573486 5	Galictis vittata T412 1	0,18661651
Panthera leo NERO 19	Mustela sibirica AP017394 11	0,18662059
Ursus americanus JX196366 3	Felis margarita NC028308 1	0,18663077
Paradoxurus hermaphroditus NC03959	Halichoerus grypus NC001602 2	0,18663082
Genetta genetta T297 1	Eumetopias jubatus NC004030 10	0,18663266
Panthera onca NC022842 1	Canis lupus familiaris NC002008 1231	0,18663493
Cynictis penicillata T375 1	Tapirus terrestris T358	0,18663551
Ursus americanus JX196366 3	Smilodon populator MF871700 1	0,18663624
Xenogale naso C07XAR110 1	Enhydra lutris NC009692 1	0,1866367
Mustela sibirica AP017394 11	Eupleres goudotii D128 1	0,18663915
Mungos mungo MMC7 1	Martes americana NC020642 1	0,18663958
Viverra tangalunga MH464792 1	Helogale parvula SRR7637809 1	0,18664093
Phocarctos hookeri NC008418 1	Mungos mungo/gambianus SRR77048	0,18664111
Prionailurus planiceps KY682741 4	Tapirus terrestris T358	0,18664142
Urva semitorquata MH464789 1	Arctocephalus australis MG023139 1	0,18664142
Potos flavus T414 1	Bdeogale nigripes GLC15 1	0,18664227
Vulpes corsac NC023958 1	Fossa fossana D350 1	0,18664244
Galidia elegans D146 1	Enhydra lutris NC009692 1	0,18664327
Procyon lotor AB462049 4	Crossarchus platycephalus C7R66 1	0,18664356

Proteles cristata T393 6	Lynx rufus NC014456 3	0,15617092
Pardofelis marmorata NLN3 2	Urva javanica/auropunctata NC006835 1	0,15618205
Panthera leo NERO 19	Helogale parvula SRR7637809 1	0,15618323
Leopardus colocolo NC028314 1	Crocuta crocuta NC020670 3	0,15619226
Puma yagouaroundi NC028311 1	Urva brachyura KY117547 1	0,15621823
Pardofelis marmorata NLN3 2	Xenogale naso C07XAR110 1	0,15621853
Ichneumia albicauda T603 1	Felis catus NC001700 2	0,15622027
Prionailurus planiceps NC028312 6	Ichneumia albicauda T603 1	0,15622067
Suricata suricatta SSM10 1	Leopardus geoffroyi NC028320 1	0,15622069
Panthera tigris NC010642 35	Chrotogale owstoni T607 1	0,15622136
Parahyaena brunnea NC038159 15	Panthera onca NC022842 1	0,15624288
Lynx lynx NC027083 4	Urva javanica/auropunctata NC006835 1	0,15624925
Paradoxurus hermaphroditus NLNC 1	Eupleres goudotii D128 1	0,15627752
Urva brachyura KY117547 1	Felis catus NC001700 2	0,15628445
Mungos mungo MMC7 1	Felis chaus NC028307 1	0,1562853
Profelis aurata NC028299 1	Bdeogale nigripes GLC15 1	0,15628745
Xenogale naso C07XAR110 1	Arctictis binturong T605 2	0,15629677
Hyaena hyaena NC020669 1	Felis silvestris lybica KP202275 4	0,15629867
Parahyaena brunnea NC038159 15	Otocolobus manul NC028323 1	0,15630446
Puma concolor NC016470 22	Proteles cristata T393 6	0,15630768
Salanoia concolor D378 1	Neofelis nebulosa NC008450 3	0,1563177
Paradoxurus hermaphroditus NC039591 1	Panthera leo NERO 19	0,15631889
Urva javanica/auropunctata NC006835 1	Crocuta crocuta NC020670 3	0,15632339
Phocarctos hookeri NC008418 1	Ommatophoca rossii AY377287etc 1	0,15632834
Urva brachyura KY117547 1	Chrotogale owstoni T607 1	0,15633431
Paradoxurus hermaphroditus NLNC 1	Helogale parvula SRR7637809 1	0,15634951
Suricata suricatta SSM10 1	Paguma larvata PDDS11 2	0,15634969
Prionodon linsang ERR2391707 1	Panthera onca NC022842 1	0,15635842
Panthera onca KP202264 2	Mungotictis decemlineata NC027828 1	0,15635844
Proteles cristata T393 6	Xenogale naso C07XAR110 1	0,1563597
Galerella sanguinea T378 1	Crocuta crocuta NC020670 3	0,15636197
Odobenus rosmarus NC004029 29	Lobodon carcinophaga NC008423 1	0,15636253
Profelis aurata NC028299 1	Parahyaena brunnea NC038159 15	0,156371
Suricata suricatta SSM10 1	Chrotogale owstoni T607 1	0,15640204
Prionodon pardicolor NC024569 2	Diplogale hosei MH464790 1	0,15641525
Suricata suricatta SSM10 1	Puma yagouaroundi NC028311 1	0,15642039
Suricata suricatta SSM10 1	Leptailurus serval NC028316 1	0,15642087
Felis catus NC001700 2	Bdeogale nigripes GLC15 1	0,15642107
Suricata suricatta SSM10 1	Leopardus jacobita NC028322 1	0,15642205
Bdeogale nigripes GLC15 1	Acinonyx jubatus NC005212 3	0,15643589
Proteles cristata T393 6	Felis nigripes NC028309 1	0,15644184
Proteles cristata T393 6	Prionailurus planiceps KY682741 4	0,15644264
Proteles cristata T393 6	Leopardus geoffroyi NC028320 1	0,15644355
Nandinia binotata NC024567 1	Leptonychotes weddellii NC008424 1	0,15647724
Paradoxurus jerdoni MH464793 1	Xenogale naso C07XAR110 1	0,15648387
Neofelis nebulosa NC008450 3	Hemigalus derbyanus MH464791 1	0,15648633
Taxidea taxus NC020646 1	Hydrurga leptonyx NC008425 1	0,15648736
Panthera pardus japonensis KJ866876 8	Crossarchus platycephalus C7R66 1	0,15648761
Panthera uncia KP202269 1	Atilax paludinosus T606 1	0,15648843
Prionailurus planiceps KY682741 4	Ichneumia albicauda T603 1	0,15649001
Galidia elegans D146 1	Felis chaus NC028307 1	0,15649466
Galidictis fasciata DM333 1	Arctictis binturong T605 2	0,15650722
Panthera onca NC022842 1	Hyaena hyaena NC020669 1	0,15650879
Panthera pardus NC010641 5	Urva semitorquata MH464789 1	0,1565208
Cynogale bennetti KY117544 1	Caracal caracal NC028306 1	0,15652798
Leopardus guigna NC028321 1	Cynogale bennetti KY117544 1	0,15653679
Prionodon linsang ERR2391707 1	Panthera onca KP202264 2	0,15655184
Prionodon pardicolor NC024569 2	Fossa fossana D350 1	0,15655354
Panthera pardus japonensis KJ866876 8	Mungos mungo/gambianus SRR7704821 1	0,15655454
Panthera pardus japonensis KJ866876 8	Urva semitorquata MH464789 1	0,15655507
Viverricula indica KX891751 1	Cryptoprocta ferox CFC13 1	0,15655512
Lynx pardinus NC028319 161	Galerella sanguinea T378 1	0,15655591
Suricata suricatta SSM10 1	Leopardus guigna NC028321 1	0,15655735
Proteles cristata T393 6	Atilax paludinosus T606 1	0,15656163
Viverricula indica NC025296 2	Parahyaena brunnea NC038159 15	0,15656202
Panthera onca NC022842 1	Cynictis penicillata T375 1	0,15656327
Paradoxurus hermaphroditus NC039591 1	Ichneumia albicauda T603 1	0,15656677
Panthera pardus NC010641 5	Cryptoprocta ferox CFC13 1	0,15660174
Chrotogale owstoni T607 1	Bdeogale nigripes GLC15 1	0,15660489
Prionodon linsang ERR2391707 1	Chrotogale owstoni T607 1	0,15660696
Panthera onca KP202264 2	Helogale parvula SRR7637809 1	0,15662198
Leopardus tigrinus NC028317 1	Urva javanica T413 1	0,15662354
Panthera pardus japonensis KJ866876 8	Eupleres goudotii D128 1	0,15662396
Panthera pardus japonensis KJ866876 8	Paguma larvata PDDS11 2	0,15662548
Proteles cristata T393 6	Nandinia binotata NC024567 1	0,15662952
Prionodon pardicolor NC024569 2	Panthera leo spelaea KX258452 2	0,15663759
Prionodon pardicolor NC024569 2	Panthera uncia KP202269 1	0,15663925
Proteles cristata T393 6	Caracal caracal NC028306 1	0,15664259
Paradoxurus jerdoni MH464793 1	Atilax paludinosus T606 1	0,1566859
Urva javanica T413 1	Felis nigripes NC028309 1	0,1566893
Leopardus tigrinus NC028317 1	Xenogale naso C07XAR110 1	0,15669096
Panthera onca KP202264 2	Hyaena hyaena NC020669 1	0,15670195
Hyaena hyaena NC020669 1	Felis nigripes NC028309 1	0,15670221
Parahyaena brunnea NC038159 15	Felis chaus NC028307 1	0,15670717
Lynx pardinus NC028319 161	Crocuta crocuta NC020670 3	0,15670967
Hyaena hyaena NC020669 1	Genetta abyssinica MG489822 1	0,15673955
Xenogale naso C07XAR110 1	Genetta genetta T297 1	0,15675536
Xenogale naso C07XAR110 1	Felis silvestris lybica KP202275 4	0,15675704
Leopardus geoffroyi NC028320 1	Crocuta crocuta NC020670 3	0,15677947
Hyaena hyaena NC020669 1	Acinonyx jubatus NC005212 3	0,15678744
Panthera pardus NC010641 5	Atilax paludinosus T606 1	0,15679057
Panthera leo NERO 19	Atilax paludinosus T606 1	0,15679248
Urva javanica/auropunctata NC006835 1	Felis catus NC001700 2	0,15679392
Felis margarita NC028308 1	Cynogale bennetti KY117544 1	0,15679666
Hyaena hyaena NC020669 1	Homotherium latidens MF871702 3	0,15679996
Panthera onca KP202264 2	Mungos mungo MMC7 1	0,15682301
Suricata suricatta SSM10 1	Lynx canadensis NC028313 1	0,15682407
Otocolobus manul NC028323 1	Xenogale naso C07XAR110 1	0,15682477
Suricata suricatta SSM10 1	Otocolobus manul NC028323 1	0,15682495
Suricata suricatta SSM10 1	Leopardus tigrinus NC028317 1	0,15682602
Ichneumia albicauda T603 1	Felis nigripes NC028309 1	0,15682659
Salanoia concolor D378 1	Paradoxurus jerdoni MH464793 1	0,1568276
Panthera tigris NC010642 35	Urva semitorquata MH464789 1	0,15683164

Vulpes vulpes NC008434 5	Mustela kathiah NC023210 1	0,1866454
Vulpes ferrilata NC027935 1	Mustela putorius NC020638 4	0,18664598
Otaria byronia OTAB 1	Genetta abyssinica MG489822 1	0,18664621
Bassaricyon neblina SRX1097850 1	Ailurus fulgens styani NC009691 1	0,18664703
Nyctereutes procyonoides NC013700 3	Arctocephalus pusillus NC008417 1	0,1866474
Ursus americanus JX196366 3	Canis aureus KT448274 1	0,18664771
Mustela nivalis T306 5	Arctocephalus australis MG023139 1	0,18664911
Genetta genetta T297 1	Conepatus chinga NC042596 1	0,18664965
Ichneumia albicauda T603 1	Callorhinus ursinus NC008415 1	0,18665054
Mustela nivalis T306 5	Urva brachyura KY117547 1	0,18665133
Enhydra lutris NC009692 1	Cryptoprocta ferox CFC13 1	0,18665269
Mustela eversmannii NC028013 1	Ichneumia albicauda T603 1	0,18665385
Panthera pardus japonensis KJ866876	Mustela putorius NC020638 4	0,18665388
Phocarctos hookeri NC008418 1	Civettictis civetta GLC19 1	0,18665402
Leptailurus serval NC028316 1	Arctocephalus townsendi NC008420 1	0,18665418
Caracal caracal NC028306 1	Arctocephalus forsteri KT693377 17	0,18665461
Viverra zibetha T609 1	Neophoca cinerea NC008419 1	0,18665498
Leopardus pardalis NC028315 1	Arctocephalus townsendi NC008420 1	0,18665554
Ursus arctos EU497665 29	Chrotogale owstoni T607 1	0,18665615
Leopardus jacobita NC028322 1	Arctocephalus forsteri KT693377 17	0,18665637
Viverra zibetha T609 1	Otocyon megalotis SAF1 2	0,18665761
Prionodon pardicolor NC024569 2	Canis anthus NC027956 2	0,18665802
Ursus arctos EU497665 29	Meles anakuma NC009677 1	0,18665805
Ursus arctos pruinosus MG066703 3	Meles anakuma NC009677 1	0,18665823
Prionailurus rubiginosus NC028304 2	Phocarctos hookeri NC008418 1	0,18665831
Nyctereutes procyonoides NC013700 3	Felis chaus NC028307 1	0,18665858
Vulpes ferrilata NC027935 1	Leopardus geoffroyi NC028320 1	0,18665893
Procyon lotor AB462049 4	Genetta genetta T297 1	0,18665907
Procyon lotor AB462046 3	Panthera onca KP202264 2	0,1866596
Viverra tangalunga MH464792 1	Ursus arctos GU573491 207	0,18665977
Procyon lotor AB462049 4	Paradoxurus hermaphroditus NLNC 1	0,18665999
Cryptoprocta ferox CFC13 1	Arctodus simus NC011116 1	0,18666202
Puma yagouaroundi NC028311 1	Lutrogale perspicillata NC035811 1	0,18666238
Panthera uncia KP202269 1	Mustela nivalis T306 5	0,18666299
Prionodon pardicolor NC024569 2	Bassariscus sumichrasti SRX1099089 1	0,18666382
Ursus thibetanus formosanus NC00933	Prionodon linsang ERR2391707 1	0,1866639
Vulpes corsac NC023958 1	Arctictis binturong T605 2	0,18666448
Otaria byronia OTAB 1	Felis margarita NC028308 1	0,18666449
Parahyaena brunnea NC038159 15	Martes pennanti NC020664 16	0,18666498
Ursus arctos isabellinus 18B5 2	Lynx rufus NC014456 3	0,1866653
Ursus americanus JX196366 3	Spilogale putorius NC010497 1	0,18666662
Ursus spelaeus NC011112 8	Crossarchus platycephalus C7R66 1	0,18666737
Homotherium latidens MF871702 3	Arctocephalus forsteri NC004023 28	0,1866677
Taxidea taxus NC020646 1	Panthera tigris NC010642 35	0,18666804
Smilodon populator MF871700 1	Melogale moschata V0735A 1	0,1866685
Mustela altaica NC021751 1	Ailuropoda melanoleuca NC009492 5	0,18666916
Panthera uncia NC010638 1	Ictonyx striatus T299 1	0,18666951
Poecilogale albinucha T602 1	Chrysocyon brachyurus NC024172 1	0,18666987
Ursus thibetanus laniger MH281753 2	Canis latrans NC008093 7	0,18667196
Panthera tigris amoyensis NC014770 2	Otocyon megalotis SAF1 2	0,18667281
Urva javanica/auropunctata NC00683	Callorhinus ursinus NC008415 1	0,18667419
Ursus thibetanus formosanus NC00933	Ichneumia albicauda T603 1	0,1866747
Ursus arctos GU573486 5	Catopuma badia NC028300 1	0,18667725
Ursus thibetanus formosanus NC00933	Profelis aurata NC028299 1	0,18667905
Viverra tangalunga MH464792 1	Urva javanica/auropunctata NC006835	0,1866806
Martes flavigula NC012141 3	Urva brachyura KY117547 1	0,18668154
Prionailurus bengalensis NC028301 12	Melursus ursinus NC009970 2	0,18668272
Tremarctos ornatus NC009969 2	Spilogale putorius NC010497 1	0,18668315
Melursus ursinus NC009970 2	Leopardus geoffroyi NC028320 1	0,1866841
Zalophus californianus NC008416 1	Panthera onca KP202264 2	0,1866843
Ursus thibetanus thibetanus NC01111	Lycalopex sechurae KT448284 1	0,1866885
Panthera tigris amoyensis NC014770 2	Canis lupus chanco NC010340 4	0,18669297
Panthera leo NERO 19	Otocyon megalotis SAF1 2	0,18669331
Procyon lotor AB462046 3	Panthera leo NERO 19	0,18669578
Panthera pardus NC010641 5	Lutra sumatrana NC035810 1	0,18669744
Cuon alpinus NC013445 3	Arctonyx collaris NC020645 1	0,18669811
Chrotogale owstoni T607 1	Arctocephalus townsendi NC008420 1	0,18669952
Lutra lutra LC050126 1	Urva brachyura KY117547 1	0,18670002
Lutra lutra NC011358 9	Cynictis penicillata T375 1	0,18670079
Vulpes zerda KJ603240 1	Martes flavigula NC012141 3	0,18670079
Lutra lutra LC050126 1	Cynictis penicillata T375 1	0,18670148
Lutra lutra LC050126 1	Bdeogale nigripes GLC15 1	0,18670204
Genetta servalina NC024568 2	Tapirus terrestris T358	0,18670267
Lynx lynx NC027083 4	Cuon alpinus NC013445 3	0,1867038
Xenogale naso C07XAR110 1	Tapirus terrestris T358	0,18670499
Canis mesomelas KT448280 1	Bdeogale nigripes GLC15 1	0,18670543
Panthera onca NC022842 1	Gulo gulo NC009685 3	0,18670826
Urva semitorquata MH464789 1	Arctocephalus gazella BK010918 1	0,18671013
Tremarctos ornatus NC009969 2	Lontra canadensis SRR10409165 1	0,18671112
Procyon lotor AB462046 3	Xenogale naso C07XAR110 1	0,18671282
Mustela putorius NC020638 4	Mungotictis decemlineata NC027828 1	0,18671283
Pardofelis marmorata NLN3 2	Lycalopex sechurae KT448284 1	0,18671384
Caracal caracal NC028306 1	Canis mesomelas KT448280 1	0,18671423
Diplogale hosei MH464790 1	Canis mesomelas KT448280 1	0,18671437
Puma yagouaroundi NC028311 1	Conepatus chinga NC042596 1	0,18671499
Martes pennanti NC020664 16	Bdeogale nigripes GLC15 1	0,18671522
Nyctereutes procyonoides NC013700 3	Chrotogale owstoni T607 1	0,18671522
Nyctereutes procyonoides NC013700 3	Martes martes T302 3	0,18671609
Pardofelis marmorata NLN3 2	Ailurus fulgens styani NC009691 1	0,18671691
Mustela nigripes NC024942 1	Galerella sanguinea T378 1	0,18671761
Panthera leo NERO 19	Canis lupus chanco NC010340 4	0,18671806
Phoca largha NC008430 1	Tapirus terrestris T358	0,18671811
Vulpes ferrilata NC027935 1	Viverra zibetha T609 1	0,18671869
Lutra sumatrana NC035810 1	Hyaena hyaena NC020669 1	0,18671899
Genetta genetta T297 1	Arctocephalus australis MG023139 1	0,18671907
Helarctos malayanus NC009968 2	Canis lupus chanco NC010340 4	0,18671924
Martes americana NC020642 1	Genetta genetta T297 1	0,1867194
Urocyon littoralis catalinae KP129018	Homotherium latidens MF871702 3	0,1867194
Vulpes zerda KJ603240 1	Viverra zibetha T609 1	0,18671957
Viverricula indica NC025296 2	Arctocephalus gazella BK010918 1	0,18671997
Potos flavus T414 1	Panthera uncia NC010638 1	0,18672062
Panthera uncia KP202269 1	Mustela putorius NC020638 4	0,18672064
Panthera uncia NC010638 1	Mustela nigripes NC024942 1	0,18672082

Odobenus rosmarus NC004029 29	Hydrurga leptonyx NC008425 1	0,15683436
Pardofelis marmorata NLN3 2	Hyaena hyaena NC020669 1	0,1568377
Puma concolor NC016470 22	Hyaena hyaena NC020669 1	0,15683791
Viverricula indica KX891745 1	Parahyaena brunnea NC038159 15	0,15683939
Parahyaena brunnea NC038159 15	Felis nigripes NC028309 1	0,15684167
Prionailurus rubiginosus NC028304 2	Crocuta crocuta NC020670 3	0,15684624
Proteles cristata T393 6	Prionailurus viverrinus NC028305 1	0,15684728
Proteles cristata T393 6	Leopardus jacobita NC028322 1	0,15684947
Prionailurus bengalensis CKM45 20	Crocuta crocuta NC020670 3	0,15684997
Leopardus pardalis NC028315 1	Cynogale bennetti KY117544 1	0,15686309
Paradoxurus hermaphroditus NC039591 1	Atilax paludinosus T606 1	0,15688792
Mustela frenata NC020640 1	Bassariscus sumichrasti SRX1099089 1	0,15688808
Lynx lynx NC027083 4	Cynictis penicillata T375 1	0,15689008
Leptonychotes weddellii NC008424 1	Arctodus simus NC011116 1	0,15689068
Lynx lynx NC027083 4	Galerella sanguinea T378 1	0,15689181
Suricata suricatta SSM10 1	Felis chaus NC028307 1	0,15689249
Paradoxurus hermaphroditus NLNC 1	Panthera uncia KP202269 1	0,15689398
Panthera uncia KP202269 1	Cryptoprocta ferox CFC13 1	0,15690169
Parahyaena brunnea NC038159 15	Felis catus NC001700 2	0,15690927
Leopardus wiedii NC028318 1	Crocuta crocuta NC020670 3	0,15691686
Panthera pardus NC010641 5	Mungos mungo/gambianus SRR7704821 1	0,15692456
Taxidea taxus NC020646 1	Leptonychotes weddellii NC008424 1	0,1569514
Pardofelis marmorata NLN3 2	Cynictis penicillata T375 1	0,15695852
Hyaena hyaena NC020669 1	Civettictis civetta GLC19 1	0,15696761
Suricata suricatta SSM10 1	Panthera onca NC022842 1	0,15696914
Parahyaena brunnea NC038159 15	Civettictis civetta GLC19 1	0,15697348
Parahyaena brunnea NC038159 15	Panthera pardus japonensis KJ866876 8	0,1569749
Panthera tigris NC010642 35	Cryptoprocta ferox CFC13 1	0,15697505
Prionodon pardicolor NC024569 2	Panthera uncia NC010638 1	0,15697569
Neofelis nebulosa NC008450 3	Crossarchus platycephalus C7R66 1	0,15698789
Panthera leo NERO 19	Urva semitorquata MH464789 1	0,15699151
Suricata suricatta SSM10 1	Panthera pardus NC010641 5	0,15699327
Paradoxurus hermaphroditus NC039591 1	Bdeogale nigripes GLC15 1	0,15702271
Lynx canadensis NC028313 1	Cynictis penicillata T375 1	0,15702485
Panthera leo spelaea KX258452 2	Mungos mungo/gambianus SRR7704821 1	0,15702686
Galerella sanguinea T378 1	Felis chaus NC028307 1	0,15702826
Suricata suricatta SSM10 1	Arctictis binturong T605 2	0,1570379
Panthera leo NERO 19	Ichneumia albicauda T603 1	0,15705699
Panthera leo NERO 19	Galidia elegans D146 1	0,15706559
Prionailurus planiceps NC028312 6	Cynogale bennetti KY117544 1	0,15706679
Paradoxurus jerdoni MH464793 1	Eupleres goudotii D128 1	0,15708539
Viverricula indica KX891745 1	Hyaena hyaena NC020669 1	0,15710138
Mungotictis decemlineata NC027828 1	Diplogale hosei MH464790 1	0,15711533
Nandinia binotata NC024567 1	Hydrurga leptonyx NC008425 1	0,15715784
Panthera onca KP202264 2	Cynictis penicillata T375 1	0,15715964
Panthera uncia KP202269 1	Urva semitorquata MH464789 1	0,15716097
Urva javanica T413 1	Felis chaus NC028307 1	0,15716116
Xenogale naso C07XAR110 1	Felis nigripes NC028309 1	0,15716118
Catopuma temminckii NC027115 41	Bdeogale nigripes GLC15 1	0,15716194
Panthera onca NC022842 1	Mungos mungo MMC7 1	0,15716985
Panthera tigris amoyensis NC014770 2	Galidia elegans D146 1	0,15717195
Crocuta crocuta NC020670 3	Catopuma badia NC028300 1	0,15717994
Proteles cristata T393 6	Pardofelis marmorata NLN3 2	0,15718244
Proteles cristata T393 6	Felis silvestris lybica KP202275 4	0,15718274
Proteles cristata T393 6	Leopardus guigna NC028321 1	0,15718454
Prionailurus planiceps KY682741 4	Cynogale bennetti KY117544 1	0,15720148
Hyaena hyaena NC020669 1	Civettictis civetta NC033378 1	0,15720782
Panthera onca KP202264 2	Diplogale hosei MH464790 1	0,15721928
Paradoxurus hermaphroditus NC039591 1	Mungos mungo/gambianus SRR7704821 1	0,15722494
Suricata suricatta SSM10 1	Genetta genetta T297 1	0,15722692
Suricata suricatta SSM10 1	Caracal caracal NC028306 1	0,15722886
Panthera pardus japonensis KJ866876 8	Atilax paludinosus T606 1	0,15722911
Helogale parvula SRR7637809 1	Chrotogale owstoni T607 1	0,15727709
Panthera onca KP202264 2	Mungos mungo/gambianus SRR7704821 1	0,15729497
Suricata suricatta SSM10 1	Felis silvestris lybica KP202275 4	0,15729591
Paradoxurus jerdoni MH464793 1	Galidictis fasciata DM333 1	0,15729922
Crocuta crocuta NC020670 3	Bdeogale nigripes GLC15 1	0,15730457
Panthera tigris amoyensis NC014770 2	Cryptoprocta ferox CFC13 1	0,15731171
Odobenus rosmarus NC004029 29	Mirounga angustirostris SRR10331586 1	0,15731248
Parahyaena brunnea NC038159 15	Panthera pardus NC010641 5	0,15734805
Paradoxurus jerdoni MH464793 1	Urva javanica T413 1	0,15735945
Lynx lynx NC027083 4	Urva brachyura KY117547 1	0,15736066
Xenogale naso C07XAR110 1	Crocuta crocuta NC020670 3	0,15737025
Pusa hispida NC 008433 1	Odobenus rosmarus NC004029 29	0,15737383
Prionailurus planiceps KY682741 4	Hyaena hyaena NC020669 1	0,15737692
Urva javanica/auropunctata NC006835 1	Felis nigripes NC028309 1	0,15739523
Homotherium latidens MF871702 3	Galidia elegans D146 1	0,15740049
Prionodon pardicolor NC024569 2	Cynictis penicillata T375 1	0,15742987
Panthera pardus NC010641 5	Mungos mungo MMC7 1	0,15746242
Prionailurus viverrinus NC028305 1	Cynogale bennetti KY117544 1	0,15746912
Neofelis nebulosa NC008450 3	Arctictis binturong T605 2	0,15747988
Panthera onca KP202264 2	Urva javanica T413 1	0,15749627
Lynx canadensis NC028313 1	Xenogale naso C07XAR110 1	0,15749721
Paradoxurus hermaphroditus NC039591 1	Panthera pardus japonensis KJ866876 8	0,15749952
Proteles cristata T393 6	Bdeogale nigripes GLC15 1	0,15750514
Hyaena hyaena NC020669 1	Genetta servalina NC024568 2	0,15750568
Hyaena hyaena NC020669 1	Felis margarita NC028308 1	0,15751095
Parahyaena brunnea NC038159 15	Genetta servalina NC024568 2	0,15751204
Eupleres goudotii D128 1	Diplogale hosei MH464790 1	0,15752086
Phoca largha NC008430 1	Odobenus rosmarus NC004029 29	0,15752263
Homotherium latidens MF871702 3	Crocuta crocuta NC020670 3	0,1575391
Galidictis fasciata DM333 1	Chrotogale owstoni T607 1	0,15755634
Suricata suricatta SSM10 1	Paradoxurus hermaphroditus NLNC 1	0,15756132
Panthera pardus japonensis KJ866876 8	Mungos mungo MMC7 1	0,15756373
Suricata suricatta SSM10 1	Panthera onca KP202264 2	0,15756565
Panthera leo spelaea KX258452 2	Eupleres goudotii D128 1	0,15756789
Profelis aurata NC028299 1	Galidia elegans D146 1	0,15757231
Panthera onca NC022842 1	Mungos mungo/gambianus SRR7704821 1	0,15757416
Proteles cristata T393 6	Leopardus wiedii NC028318 1	0,15758997
Panthera pardus NC010641 5	Galerella sanguinea T378 1	0,15759857
Proteles cristata T393 6	Urva javanica/auropunctata NC006835 1	0,15760954
Parahyaena brunnea NC038159 15	Civettictis civetta NC033378 1	0,15761803
Panthera leo NERO 19	Urva javanica/auropunctata NC006835 1	0,15762636

Profelis aurata NC028299 1	Mustela nigripes NC024942 1	0,18672206
Vulpes zerda KJ603240 1	Felis nigripes NC028309 1	0,18672271
Vulpes lagopus NC026529 3	Profelis aurata NC028299 1	0,18672277
Taxidea taxus NC020646 1	Galidia elegans D146 1	0,1867231
Parahyaena brunnea NC038159 15	Mustela sibirica AP017394 11	0,18672377
Lontra canadensis SRR10409165 1	Leopardus pardalis T262 1	0,18672435
Otaria byronia OTAB 1	Galerella sanguinea T378 1	0,18672483
Urocyon cinereoargenteus NC026723 2	Homotherium latidens MF871702 3	0,18672486
Lontra canadensis SRR10409165 1	Leptailurus serval NC028316 1	0,18672514
Martes melampus NC009678 1	Ailuropoda melanoleuca NC009492 5	0,18672557
Speothos venaticus C48 2	Mustela nigripes NC024942 1	0,18672597
Viverra tangalunga MH464792 1	Speothos venaticus C48 2	0,18672651
Poecilogale albinucha T602 1	Felis nigripes NC028309 1	0,18672881
Panthera pardus japonensis KJ866876	Melogale moschata NC020644 1	0,18673041
Lycaon pictus NC028427 2	Ailurus fulgens styani NC009691 1	0,18673043
Ursus arctos EU497665 29	Paguma larvata PDDS11 2	0,18673085
Neophoca cinerea NC008419 1	Galictis vittata T412 1	0,18673208
Ursus arctos isabellinus 18B5 2	Lynx lynx NC027083 4	0,18673226
Ursus maritimus NC003428 31	Leopardus pardalis T262 1	0,1867339
Otaria byronia OTAB 1	Leptailurus serval NC028316 1	0,18673454
Lontra canadensis SRR10409165 1	Helarctos malayanus NC009968 2	0,18673456
Procyon lotor AB462049 4	Melursus ursinus NC009970 2	0,18673848
Ursus arctos pruinosus MG066703 3	Leopardus geoffroyi NC028320 1	0,18673858
Ursus arctos GU573486 5	Lynx lynx NC027083 4	0,18673901
Ursus arctos GU573486 5	Puma yagouaroundi NC028311 1	0,18674177
Ursus thibetanus thibetanus NC01111	Otocyon megalotis SAF1 2	0,18674234
Ursus spelaeus EU327344 13	Genetta genetta T297 1	0,18674468
Zalophus wollebaeki SRR4431565 1	Panthera tigris NC010642 35	0,18674615
Ursus thibetanus thibetanus NC01111	Pardofelis marmorata NLN3 2	0,18674649
Ursus thibetanus formosanus NC0093	Otocolobus manul NC028323 1	0,18674682
Mephitis mephitis NC020648 1	Genetta genetta T297 1	0,18674761
Helarctos malayanus NC009968 2	Felis chaus NC028307 1	0,18674847
Paradoxurus hermaphroditus NC03959	Mephitis mephitis NC020648 1	0,18674965
Zalophus californianus NC008416 1	Profelis aurata NC028299 1	0,18675369
Meles meles T303 3	Cuon alpinus NC013445 3	0,18675637
Viverra tangalunga MH464792 1	Neofelis nebulosa NC008450 3	0,18675804
Melogale moschata NC020644 1	Canis lupus chanco NC010340 4	0,18675867
Panthera pardus japonensis KJ866876	Canis lupus familiaris NC002008 1231	0,18675928
Lynx rufus NC014456 3	Arctocephalus forsteri NC004023 28	0,18676049
Taxidea taxus NC020646 1	Neofelis nebulosa NC008450 3	0,18676145
Speothos venaticus C48 2	Civettictis civetta NC033378 1	0,18676673
Eumetopias jubatus NC004030 10	Caracal caracal NC028306 1	0,18677005
Meles leucurus NC039173 4	Atilax paludinosus T606 1	0,18677245
Helogale parvula SRR7637809 1	Callorhinus ursinus NC008415 1	0,18677323
Mustela nigripes NC024942 1	Bdeogale nigripes GLC15 1	0,1867763
Fossa fossana D350 1	Ailurus fulgens NC011124 1	0,18677631
Ursus thibetanus laniger MH281753 2	Spilogale putorius NC010497 1	0,18677901
Smilodon populator MF871700 1	Arctocephalus townsendi NC008420 1	0,18677913
Prionodon pardicolor NC024569 2	Enhydra lutris NC009692 1	0,18677971
Vulpes vulpes NC008434 5	Chrotogale owstoni T607 1	0,18678027
Salanoia concolor D378 1	Arctocephalus australis MG023139 1	0,1867812
Lycaon pictus NC028427 2	Lutra lutra LC050126 1	0,18678147
Nyctereutes procyonoides NC013700 3	Arctonyx collaris NC020645 1	0,18678229
Vulpes corsac NC023958 1	Taxidea taxus NC020646 1	0,18678255
Lynx pardinus NC028319 161	Ailurus fulgens NC011124 1	0,18678385
Ichneumia albicauda T603 1	Arctocephalus forsteri KT693377 17	0,18678787
Felis silvestris lybica KP202275 4	Arctocephalus forsteri KT693377 17	0,18678883
Puma yagouaroundi NC028311 1	Arctocephalus townsendi NC008420 1	0,18678889
Profelis aurata NC028299 1	Melogale moschata KP726273 1	0,18679009
Vulpes zerda KJ603240 1	Felis chaus NC028307 1	0,18679014
Genetta genetta T297 1	Canis latrans NC008093 7	0,18679062
Vulpes zerda KJ603240 1	Profelis aurata NC028299 1	0,18679067
Lycaon pictus NC028427 2	Urva brachyura KY117547 1	0,18679073
Ursus arctos pruinosus MG066703 3	Smilodon populator MF871700 1	0,18679079
Viverra tangalunga MH464792 1	Parahyaena brunnea NC038159 15	0,18679179
Galictis vittata T412 1	Callorhinus ursinus NC008415 1	0,1867936
Phocarctos hookeri NC008418 1	Leopardus guigna NC028321 1	0,18679389
Panthera tigris amoyensis NC014770 2	Meles anakuma NC009677 1	0,18679555
Prionodon linsang ERR2391707 1	Ictonyx striatus T299 1	0,18679641
Pardofelis marmorata NLN3 2	Martes pennanti NC020664 16	0,18679656
Leopardus wiedii NC028318 1	Bassaricyon neblina SRX1097850 1	0,18679744
Panthera uncia KP202269 1	Arctodus simus NC011116 1	0,18679793
Panthera tigris amoyensis NC014770 2	Melogale moschata V0735A 1	0,18679829
Otocyon megalotis SAF1 2	Felis nigripes NC028309 1	0,18679889
Proteles cristata T393 6	Mustela frenata NC020640 1	0,18679941
Ursus maritimus GU573488 Svalbard	Lynx lynx NC027083 4	0,18680017
Ursus thibetanus mupinensis NC00875	Panthera pardus japonensis KJ866876	0,18680103
Martes pennanti NC020664 16	Chrysocyon brachyurus NC024172 1	0,1868027
Viverricula indica NC025296 2	Ursus arctos GU573486 5	0,18680444
Lutra lutra NC011358 9	Canis lupus familiaris NC002008 1231	0,18680491
Procyon lotor AB462046 3	Melursus ursinus NC009970 2	0,18680625
Ursus arctos GU573486 5	Leopardus pardalis NC028315 1	0,18680735
Panthera onca NC022842 1	Arctodus simus NC011116 1	0,18680853
Felis chaus NC028307 1	Ailuropoda melanoleuca NC009492 5	0,18680872
Otocolobus manul NC028323 1	Ailuropoda melanoleuca NC009492 5	0,18680991
Ursus spelaeus EU327344 13	Prionailurus viverrinus NC028305 1	0,18681005
Ursus spelaeus EU327344 13	Leptailurus serval NC028316 1	0,18681091
Ursus arctos isabellinus 18B5 2	Ictonyx striatus T299 1	0,18681149
Ursus spelaeus EU327344 13	Paradoxurus jerdoni MH464793 1	0,18681179
Melursus ursinus NC009970 2	Lynx rufus NC014456 3	0,18681411
Ursus spelaeus NC011112 8	Prionailurus planiceps KY682741 4	0,18681492
Prionailurus bengalensis CKM45 20	Galictis vittata T412 1	0,18681787
Ursus arctos GU573491 207	Acinonyx jubatus NC005212 3	0,18681787
Otocolobus manul NC028323 1	Melursus ursinus NC009970 2	0,18681794
Lutrogale perspicillata NC035811 1	Arctocephalus forsteri NC004023 28	0,18681841
Genetta servalina NC024568 2	Arctocephalus forsteri NC004023 28	0,18681896
Canis lupus familiaris NC002008 1231	Bassariscus sumichrasti SRX1099089 1	0,18681929
Panthera leo NERO 19	Mustela sibirica NC020637 6	0,18682288
Vulpes vulpes NC008434 5	Martes flavigula NC012141 3	0,18682611
Spilogale putorius NC010497 1	Canis latrans NC008093 7	0,18682648
Panthera pardus NC010641 5	Mustela nigripes NC024942 1	0,18683013
Viverra tangalunga MH464792 1	Ursus americanus JX196366 3	0,18683243
Spilogale putorius NC010497 1	Civettictis civetta GLC19 1	0,18683344

Urva brachyura KY117547 1	Catopuma temminckii NC027115 41	0,15763241
Panthera uncia NC010638 1	Urva semitorquata MH464789 1	0,15763253
Felis chaus NC028307 1	Bdeogale nigripes GLC15 1	0,15763365
Hyaena hyaena NC020669 1	Felis catus NC001700 2	0,15764491
Panthera onca NC022842 1	Galidia elegans D146 1	0,15764558
Panthera onca NC022842 1	Urva javanica/auropunctata NC006835 1	0,15767279
Proteles cristata T393 6	Homotherium latidens MF871702 3	0,15769476
Taxidea taxus NC020646 1	Lobodon carcinophaga NC008423 1	0,15769868
Lynx canadensis NC028313 1	Galerella sanguinea T378 1	0,15770002
Paradoxurus hermaphroditus NC039591 1	Panthera onca KP202264 2	0,1577016
Paradoxurus jerdoni MH464793 1	Panthera pardus japonensis KJ866876 8	0,15770189
Panthera tigris NC010642 35	Galidia elegans D146 1	0,15771066
Panthera onca KP202264 2	Urva javanica/auropunctata NC006835 1	0,15773001
Paradoxurus hermaphroditus NC039591 1	Urva brachyura KY117547 1	0,15776369
Puma concolor NC016470 22	Urva brachyura KY117547 1	0,15776634
Panthera uncia KP202269 1	Galidia elegans D146 1	0,15777238
Galidia elegans D146 1	Felis nigripes NC028309 1	0,15777339
Ichneumia albicauda T603 1	Crocuta crocuta NC020670 3	0,15777822
Smilodon populator MF871700 1	Crocuta crocuta NC020670 3	0,15777883
Hyaena hyaena NC020669 1	Felis chaus NC028307 1	0,15777896
Parahyaena brunnea NC038159 15	Catopuma temminckii NC027115 41	0,15778238
Suricata suricatta SSM10 1	Smilodon populator MF871700 1	0,15778588
Felis nigripes NC028309 1	Cynogale bennetti KY117544 1	0,15780652
Salanoia concolor D378 1	Chrotogale owstoni T607 1	0,15782522
Procyon lotor AB462046 3	Potos flavus T414 1	0,15782677
Paradoxurus hermaphroditus NC039591 1	Urva javanica T413 1	0,15783073
Suricata suricatta SSM10 1	Lynx rufus NC014456 3	0,15783428
Suricata suricatta SSM10 1	Puma concolor NC016470 22	0,15783446
Suricata suricatta SSM10 1	Felis margarita NC028308 1	0,15783484
Panthera pardus japonensis KJ866876 8	Galidia elegans D146 1	0,15784003
Panthera leo spelaea KX258452 2	Cryptoprocta ferox CFC13 1	0,15784353
Proteles cristata T393 6	Ichneumia albicauda T603 1	0,15784389
Pardofelis marmorata NLN3 2	Crocuta crocuta NC020670 3	0,157855
Paradoxurus jerdoni MH464793 1	Panthera leo NERO 19	0,15786888
Proteles cristata T393 6	Leopardus colocolo NC028314 1	0,15787376
Panthera leo spelaea KX258452 2	Mungos mungo MMC7 1	0,1578901
Prionodon linsang ERR2391707 1	Fossa fossana D350 1	0,1578912
Suricata suricatta SSM10 1	Panthera pardus japonensis KJ866876 8	0,15790272
Paradoxurus hermaphroditus NLNC 1	Panthera leo spelaea KX258452 2	0,15790404
Panthera tigris amoyensis NC014770 2	Mungos mungo/gambianus SRR7704821 1	0,1579085
Paradoxurus hermaphroditus NLNC 1	Panthera tigris NC010642 35	0,15791009
Suricata suricatta SSM10 1	Crocuta crocuta NC020670 3	0,1579105
Paradoxurus hermaphroditus NC039591 1	Galerella sanguinea T378 1	0,15791158
Paradoxurus jerdoni MH464793 1	Urva brachyura KY117547 1	0,1579657
Panthera leo spelaea KX258452 2	Urva semitorquata MH464789 1	0,15796705
Pardofelis marmorata NLN3 2	Urva brachyura KY117547 1	0,15796773
Panthera uncia KP202269 1	Mungos mungo MMC7 1	0,1579679
Urva brachyura KY117547 1	Felis chaus NC028307 1	0,1579686
Xenogale naso C07XAR110 1	Felis margarita NC028308 1	0,1579693
Paradoxurus hermaphroditus NLNC 1	Mungotictis decemlineata NC027828 1	0,1579717
Paradoxurus hermaphroditus NC039591 1	Galidictis fasciata DM333 1	0,1579728
Panthera tigris NC010642 35	Mungos mungo/gambianus SRR7704821 1	0,15797572
Odobenus rosmarus NC004029 29	Erignathus barbatus NC008426 1	0,15797828
Panthera onca NC022842 1	Helogale parvula SRR7637809 1	0,15797968
Panthera leo NERO 19	Diplogale hosei MH464790 1	0,15799117
Prionodon linsang ERR2391707 1	Panthera leo NERO 19	0,15799341
Phoca vitulina NC001325 1	Odobenus rosmarus NC004029 29	0,15799379
Urva javanica/auropunctata NC006835 1	Felis chaus NC028307 1	0,15800239
Lynx pardinus NC028319 161	Cynogale bennetti KY117544 1	0,1580088
Cynogale bennetti KY117544 1	Catopuma temminckii NC027115 41	0,15801765
Paradoxurus hermaphroditus NC039591 1	Crossarchus platycephalus C7R66 1	0,15803294
Pardofelis marmorata NLN3 2	Urva javanica T413 1	0,15803576
Panthera uncia KP202269 1	Paguma larvata PDDS11 2	0,15803939
Panthera leo spelaea KX258452 2	Paguma larvata PDDS11 2	0,15804033
Proteles cristata T393 6	Cynictis penicillata T375 1	0,15804408
Galidictis fasciata DM333 1	Diplogale hosei MH464790 1	0,15804611
Leopardus guigna NC028321 1	Crocuta crocuta NC020670 3	0,15805875
Panthera tigris amoyensis NC014770 2	Arctictis binturong T605 2	0,15805918
Mungotictis decemlineata NC027828 1	Chrotogale owstoni T607 1	0,15809517
Prionodon linsang ERR2391707 1	Panthera uncia KP202269 1	0,1581005
Panthera tigris amoyensis NC014770 2	Mungos mungo MMC7 1	0,15811032
Paradoxurus hermaphroditus NLNC 1	Panthera tigris amoyensis NC014770 2	0,15811169
Panthera onca NC022842 1	Bdeogale nigripes GLC15 1	0,15811315
Panthera leo spelaea KX258452 2	Urva javanica/auropunctata NC006835 1	0,15813482
Panthera pardus NC010641 5	Galidia elegans D146 1	0,158145
Suricata suricatta SSM10 1	Felis nigripes NC028309 1	0,15817147
Proteles cristata T393 6	Urva javanica T413 1	0,15817864
Proteles cristata T393 6	Catopuma temminckii NC027115 41	0,15819089
Panthera leo NERO 19	Cynictis penicillata T375 1	0,15820082
Suricata suricatta SSM10 1	Panthera leo NERO 19	0,15820552
Panthera leo spelaea KX258452 2	Helogale parvula SRR7637809 1	0,15823677
Xenogale naso C07XAR110 1	Felis catus NC001700 2	0,15823847
Paradoxurus hermaphroditus NLNC 1	Panthera uncia NC010638 1	0,15824062
Panthera pardus japonensis KJ866876 8	Hyaena hyaena NC020669 1	0,1582507
Phoca fasciata NC008428 1	Odobenus rosmarus NC004029 29	0,1582552
Paradoxurus hermaphroditus NC039591 1	Xenogale naso C07XAR110 1	0,15830182
Panthera leo spelaea KX258452 2	Galidia elegans D146 1	0,15830635
Proteles cristata T393 6	Galidia elegans D146 1	0,1583098
Proteles cristata T393 6	Felis margarita NC028308 1	0,15832807
Neofelis nebulosa NC008450 3	Eupleres goudotii D128 1	0,15833384
Panthera pardus japonensis KJ866876 8	Galerella sanguinea T378 1	0,15837382
Urva brachyura KY117547 1	Crocuta crocuta NC020670 3	0,15838119
Eupleres goudotii D128 1	Arctictis binturong T605 2	0,15838142
Cynictis penicillata T375 1	Crocuta crocuta NC020670 3	0,15838298
Panthera onca NC022842 1	Ichneumia albicauda T603 1	0,15838397
Proteles cristata T393 6	Leptailurus serval NC028316 1	0,15839732
Panthera leo NERO 19	Bdeogale nigripes GLC15 1	0,15840387
Paradoxurus jerdoni MH464793 1	Panthera pardus NC010641 5	0,15840547
Panthera pardus NC010641 5	Hyaena hyaena NC020669 1	0,15842129
Procyon lotor AB462049 4	Potos flavus T414 1	0,15843268
Mustela erminea T305 2	Hydrurga leptonyx NC008425 1	0,158437
Panthera onca NC022842 1	Xenogale naso C07XAR110 1	0,15845029
Panthera uncia KP202269 1	Arctictis binturong T605 2	0,15845761

Pusa caspica NC008431 1	Cynogale bennetti KY117544 1	0,18683532
Mustela sibirica NC020637 6	Lycalopex sechurae KT448284 1	0,18683612
Meles anakuma NC009677 1	Atilax paludinosus T606 1	0,18684039
Mustela eversmannii NC028013 1	Xenogale naso C07XAR110 1	0,18684268
Mustela eversmannii NC028013 1	Urva javanica T413 1	0,18684298
Phocarctos hookeri NC008418 1	Eupleres goudotii D128 1	0,18684304
Urva semitorquata MH464789 1	Arctocephalus forsteri KT693377 17	0,18684351
Genetta abyssinica MG489822 1	Arctocephalus gazella BK010918 1	0,18684357
Paradoxurus hermaphroditus NC03959	Callorhinus ursinus NC008415 1	0,18684408
Otocyon megalotis SAF1 2	Genetta abyssinica MG489822 1	0,18684511
Paguma larvata PDDS11 2	Martes melampus NC009678 1	0,18684614
Felis nigripes NC028309 1	Tapirus terrestris T358	0,18684688
Vulpes corsac NC023958 1	Mustela sibirica NC020637 6	0,1868473
Meles anakuma NC009677 1	Canis anthus NC027956 2	0,18684882
Viverra tangalunga MH464792 1	Nyctereutes procyonoides NC013700 3	0,18684913
Salanoia concolor D378 1	Arctocephalus gazella BK010918 1	0,18684959
Vulpes corsac NC023958 1	Paradoxurus jerdoni MH464793 1	0,18685283
Felis catus NC001700 2	Arctocephalus australis MG023139 1	0,18685611
Salanoia concolor D378 1	Otocyon megalotis SAF1 2	0,18685652
Vulpes vulpes NC008434 5	Martes foina NC020643 1	0,18685684
Vulpes ferrilata NC027935 1	Felis catus NC001700 2	0,18685688
Vulpes corsac NC023958 1	Leopardus tigrinus NC028317 1	0,18685694
Viverricula indica KX891751 1	Martes pennanti NC020664 16	0,186857
Panthera onca KP202264 2	Lycalopex sechurae KT448284 1	0,18685707
Profelis aurata NC028299 1	Melogale moschata V0735A 1	0,18685746
Leptailurus serval NC028316 1	Arctocephalus gazella BK010918 1	0,18685758
Smilodon populator MF871700 1	Lontra canadensis SRR10409165 1	0,18685836
Zalophus wollebaeki SRR4431565 1	Lontra canadensis SRR10409165 1	0,18685849
Melogale moschata V0735A 1	Chrysocyon brachyurus NC024172 1	0,18686011
Phocarctos hookeri NC008418 1	Otocolobus manul NC028323 1	0,18686111
Panthera tigris NC010642 35	Lutra lutra LC050126 1	0,18686187
Ursus arctos GU573486 5	Crossarchus platycephalus C7R66 1	0,18686211
Lycaon pictus NC028427 2	Chrotogale owstoni T607 1	0,1868622
Mustela altaica NC021751 1	Genetta genetta T297 1	0,18686293
Profelis aurata NC028299 1	Mustela altaica NC021751 1	0,18686308
Ursus thibetanus mupinensis NC00875	Cryptoprocta ferox CFC13 1	0,18686339
Zalophus wollebaeki SRR4431565 1	Paradoxurus hermaphroditus NLNC 1	0,18686518
Otaria byronia OTAB 1	Felis silvestris lybica KP202275 4	0,1868659
Lycalopex sechurae KT448284 1	Fossa fossana D350 1	0,18686596
Otocyon megalotis SAF1 2	Felis chaus NC028307 1	0,18686602
Potos flavus T414 1	Panthera onca NC022842 1	0,1868668
Vulpes lagopus NC026529 3	Panthera onca NC022842 1	0,18686697
Neophoca cinerea NC008419 1	Hemigalus derbyanus MH464791 1	0,18686788
Ursus spelaeus EU327344 13	Lutrogale perspicillata NC035811 1	0,18686816
Ursus arctos isabellinus 18B5 2	Prionailurus planiceps KY682741 4	0,18686849
Ursus arctos EU497665 29	Leopardus wiedii NC028318 1	0,18686867
Speothos venaticus C48 2	Lynx rufus NC014456 3	0,1868691
Ursus thibetanus mupinensis NC00875	Prionodon pardicolor NC024569 2	0,1868698
Ursus arctos pruinosus MG066703 3	Martes foina NC020643 1	0,18687019
Otaria byronia OTAB 1	Galictis vittata T412 1	0,18687081
Ursus spelaeus EU327344 13	Lontra canadensis SRR10409165 1	0,18687244
Ursus thibetanus laniger MH281753 2	Canis anthus NC027956 2	0,18687378
Genetta abyssinica MG489822 1	Arctocephalus forsteri NC004023 28	0,18687384
Zalophus californianus NC008416 1	Cynictis penicillata T375 1	0,18687604
Prionailurus bengalensis NC028301 12	Ailuropoda melanoleuca NC009492 5	0,18687631
Ursus arctos GU573491 207	Ictonyx striatus T299 1	0,18687882
Ursus maritimus GU573488 Svalbard	Arctictis binturong T605 2	0,18687904
Poecilogale albinucha T602 1	Ailuropoda melanoleuca NC009492 5	0,18687987
Ursus arctos EU497665 29	Galictis vittata T412 1	0,18687995
Ursus thibetanus laniger MH281753 2	Prionodon pardicolor NC024569 2	0,1868808
Mungos mungo MMC7 1	Gulo gulo NC009685 3	0,18688087
Pardofelis marmorata NLN3 2	Chrysocyon brachyurus NC024172 1	0,18688194
Urva brachyura KY117547 1	Gulo gulo NC009685 3	0,18688266
Ursus thibetanus laniger MH281753 2	Profelis aurata NC028299 1	0,18688343
Leopardus jacobita NC028322 1	Canis lupus chanco NC010340 4	0,18688485
Mephitis mephitis NC020648 1	Diplogale hosei MH464790 1	0,18688503
Ictonyx striatus T299 1	Chrysocyon brachyurus NC024172 1	0,18688662
Zalophus californianus NC008416 1	Ichneumia albicauda T603 1	0,18688846
Martes pennanti NC020664 16	Canis aureus KT448274 1	0,18688938
Mungotictis decemlineata NC027828 1	Eumetopias jubatus NC004030 10	0,18689412
Galidia elegans D146 1	Eumetopias jubatus NC004030 10	0,18689516
Mungotictis decemlineata NC027828 1	Martes flavigula NC012141 3	0,18689717
Ursus americanus JX196366 3	Lynx lynx NC027083 4	0,18689719
Ursus arctos AP012576 6	Arctocephalus forsteri NC004023 28	0,18689951
Ursus americanus JX196366 3	Prionailurus viverrinus NC028305 1	0,18690044
Felis margarita NC028308 1	Cuon alpinus NC013445 3	0,18690066
Felis chaus NC028307 1	Cuon alpinus NC013445 3	0,18690126
Profelis aurata NC028299 1	Cuon alpinus NC013445 3	0,1869022
Conepatus chinga NC042596 1	Arctotherium sp NC030174 1	0,18690263
Lutra lutra LC050126 1	Helogale parvula SRR7637809 1	0,18690385
Gulo gulo NC009685 3	Arctictis binturong T605 2	0,18690625
Ursus arctos isabellinus 18B5 2	Canis lupus chanco NC010340 4	0,1869096
Vulpes lagopus NC026529 3	Martes zibellina NC011579 39	0,18691032
Martes martes T302 3	Urva brachyura KY117547 1	0,1869133
Nyctereutes procyonoides NC013700 3	Urva javanica T413 1	0,18691349
Suricata suricatta SSM10 1	Arctocephalus pusillus NC008417 1	0,18691504
Salanoia concolor D378 1	Neophoca cinerea NC008419 1	0,18691522
Vulpes zerda KJ603240 1	Galidictis fasciata DM333 1	0,1869155
Vulpes ferrilata NC027935 1	Mustela kathiah NC023210 1	0,18691579
Salanoia concolor D378 1	Canis anthus NC027956 2	0,18691584
Bdeogale nigripes GLC15 1	Bassariscus sumichrasti SRX1099089 1	0,1869162
Zalophus californianus NC008416 1	Spilogale putorius NC010497 1	0,18691652
Prionodon linsang ERR2391707 1	Mustela altaica NC021751 1	0,18691732
Ursus thibetanus mupinensis NC00875	Conepatus chinga NC042596 1	0,18691774
Viverricula indica KX891745 1	Arctocephalus gazella BK010918 1	0,1869182
Martes americana NC020642 1	Cryptoprocta ferox CFC13 1	0,18691956
Lutrogale perspicillata NC035811 1	Leopardus pardalis NC028315 1	0,18691979
Meles anakuma NC009677 1	Ichneumia albicauda T603 1	0,18692069
Ommatophoca rossii AY377287etc 1	Chrysocyon brachyurus NC024172 1	0,18692115
Otaria byronia OTAB 1	Cryptoprocta ferox CFC13 1	0,18692192
Lycaon pictus NC028427 2	Atilax paludinosus T606 1	0,18692288
Vulpes vulpes NC008434 5	Mustela nivalis T306 5	0,18692333
Vulpes lagopus NC026529 3	Panthera onca KP202264 2	0,18692364

Panthera tigris NC010642 35	Arctictis binturong T605 2	0,15846328
Proteles cristata T393 6	Leopardus tigrinus NC028317 1	0,158466
Mustela erminea T305 2	Bassariscus sumichrasti SRX1099089 1	0,15850273
Hyaena hyaena NC020669 1	Hemigalus derbyanus MH464791 1	0,15850614
Urva brachyura KY117547 1	Felis nigripes NC028309 1	0,15850674
Panthera uncia KP202269 1	Helogale parvula SRR7637809 1	0,15850773
Hydrurga leptonyx NC008425 1	Arctodus simus NC011116 1	0,15851952
Neofelis nebulosa NC008450 3	Mungotictis decemlineata NC027828 1	0,15853965
Salanoia concolor D378 1	Diplogale hosei MH464790 1	0,15855727
Panthera onca NC022842 1	Diplogale hosei MH464790 1	0,15857254
Panthera uncia NC010638 1	Eupleres goudotii D128 1	0,15857702
Paradoxurus jerdoni MH464793 1	Panthera onca KP202264 2	0,15857714
Viverra zibetha T609 1	Hyaena hyaena NC020669 1	0,15858325
Paradoxurus hermaphroditus NC039591 1	Panthera onca NC022842 1	0,15858607
Odobenus rosmarus NC004029 29	Monachus schauinslandi NC008421 1	0,15859479
Puma yagouaroundi NC028311 1	Crocuta crocuta NC020670 3	0,15859793
Panthera pardus NC010641 5	Xenogale naso C07XAR110 1	0,15860915
Panthera uncia NC010638 1	Chrotogale owstoni T607 1	0,15863746
Mustela frenata NC020640 1	Lobodon carcinophaga NC008423 1	0,15863867
Prionodon pardicolor NC024569 2	Mungos mungo MMC7 1	0,15864062
Suricata suricatta SSM10 1	Profelis aurata NC028299 1	0,15864314
Hemigalus derbyanus MH464791 1	Galidia elegans D146 1	0,15864584
Panthera uncia NC010638 1	Galidia elegans D146 1	0,15864789
Otocolobus manul NC028323 1	Crocuta crocuta NC020670 3	0,15866527
Felis silvestris lybica KP202275 4	Cynogale bennetti KY117544 1	0,15868264
Prionodon linsang ERR2391707 1	Urva javanica T413 1	0,15869917
Paradoxurus jerdoni MH464793 1	Helogale parvula SRR7637809 1	0,15870614
Panthera onca KP202264 2	Bdeogale nigripes GLC15 1	0,15870945
Panthera uncia NC010638 1	Atilax paludinosus T606 1	0,1587106
Smilodon populator MF871700 1	Proteles cristata T393 6	0,15873089
Leopardus tigrinus NC028317 1	Crocuta crocuta NC020670 3	0,15873514
Procyon lotor AB462046 3	Bassaricyon neblina SRX1097850 1	0,15877327
Panthera uncia KP202269 1	Mungos mungo/gambianus SRR7704821 1	0,1587657
Galidia elegans D146 1	Arctictis binturong T605 2	0,1587954
Proteles cristata T393 6	Catopuma badia NC028300 1	0,15879779
Neofelis nebulosa NC008450 3	Galidictis fasciata DM333 1	0,15880951
Panthera leo NERO 19	Galerella sanguinea T378 1	0,15881003
Parahyaena brunnea NC038159 15	Panthera leo NERO 19	0,15882784
Prionodon linsang ERR2391707 1	Mungos mungo/gambianus SRR7704821 1	0,15883383
Galidia elegans D146 1	Chrotogale owstoni T607 1	0,15883704
Xenogale naso C07XAR110 1	Felis chaus NC028307 1	0,15884495
Paradoxurus hermaphroditus NLNC 1	Galidia elegans D146 1	0,15884693
Panthera tigris amoyensis NC014770 2	Eupleres goudotii D128 1	0,15885267
Prionodon pardicolor NC024569 2	Panthera tigris amoyensis NC014770 2	0,15886274
Panthera leo NERO 19	Urva javanica T413 1	0,15887415
Suricata suricatta SSM10 1	Felis catus NC001700 2	0,15891199
Paradoxurus jerdoni MH464793 1	Mungotictis decemlineata NC027828 1	0,1589146
Panthera onca NC022842 1	Galerella sanguinea T378 1	0,1589216
Lynx rufus NC014456 3	Crocuta crocuta NC020670 3	0,1589323
Paradoxurus hermaphroditus NLNC 1	Neofelis nebulosa NC008450 3	0,15894762
Prionodon pardicolor NC024569 2	Mungos mungo/gambianus SRR7704821 1	0,15897812
Prionailurus planiceps KY682741 4	Crocuta crocuta NC020670 3	0,15900075
Paradoxurus hermaphroditus NC039591 1	Panthera pardus NC010641 5	0,1590109
Panthera pardus japonensis KJ866876 8	Diplogale hosei MH464790 1	0,15902187
Prionodon linsang ERR2391707 1	Ichneumia albicauda T603 1	0,15903582
Suricata suricatta SSM10 1	Paradoxurus jerdoni MH464793 1	0,15904266
Panthera onca KP202264 2	Galerella sanguinea T378 1	0,15904681
Panthera tigris amoyensis NC014770 2	Diplogale hosei MH464790 1	0,15904732
Panthera leo spelaea KX258452 2	Urva javanica T413 1	0,15904784
Panthera leo spelaea KX258452 2	Atilax paludinosus T606 1	0,15905136
Panthera onca NC022842 1	Urva brachyura KY117547 1	0,1590544
Helogale parvula SRR7637809 1	Diplogale hosei MH464790 1	0,15905863
Panthera uncia KP202269 1	Hyaena hyaena NC020669 1	0,15905944
Prionodon pardicolor NC024569 2	Panthera tigris NC010642 35	0,15906456
Panthera pardus NC010641 5	Diplogale hosei MH464790 1	0,15906712
Genetta abyssinica MG489822 1	Crocuta crocuta NC020670 3	0,15909082
Mustela erminea T305 2	Leptonychotes weddellii NC008424 1	0,15910043
Potos flavus T414 1	Mustela erminea T305 2	0,15910317
Paradoxurus hermaphroditus NC039591 1	Helogale parvula SRR7637809 1	0,1591102
Proteles cristata T393 6	Genetta abyssinica MG489822 1	0,15913007
Leopardus jacobita NC028322 1	Crocuta crocuta NC020670 3	0,15913901
Prionailurus viverrinus NC028305 1	Crocuta crocuta NC020670 3	0,15913908
Panthera leo NERO 19	Hyaena hyaena NC020669 1	0,15916018
Mellivora capensis T370 1	Martes zibellina NC011579 39	0,15916279
Potos flavus T414 1	Mustela kathiah NC023210 1	0,15916751
Panthera uncia KP202269 1	Urva javanica T413 1	0,15918003
Panthera pardus japonensis KJ866876 8	Bdeogale nigripes GLC15 1	0,15918104
Panthera uncia NC010638 1	Paguma larvata PDDS11 2	0,15918401
Panthera uncia KP202269 1	Diplogale hosei MH464790 1	0,15919043
Prionodon pardicolor NC024569 2	Cynogale bennetti KY117544 1	0,15923066
Mellivora capensis T370 1	Martes martes T302 3	0,15924053
Panthera pardus japonensis KJ866876 8	Xenogale naso C07XAR110 1	0,15924929
Puma concolor NC016470 22	Crocuta crocuta NC020670 3	0,15927055
Leopardus tigrinus NC028317 1	Cynogale bennetti KY117544 1	0,15928851
Odobenus rosmarus NC004029 29	Monachus monachus NC044972 5	0,15929468
Panthera onca KP202264 2	Xenogale naso C07XAR110 1	0,15931626
Parahyaena brunnea NC038159 15	Panthera uncia KP202269 1	0,15933181
Panthera leo NERO 19	Urva brachyura KY117547 1	0,15934549
Panthera leo NERO 19	Xenogale naso C07XAR110 1	0,15934846
Panthera pardus NC010641 5	Urva javanica T413 1	0,15934849
Taxidea taxus NC020646 1	Erignathus barbatus NC008426 1	0,15937598
Panthera tigris NC010642 35	Mungos mungo MMC7 1	0,15938964
Suricata suricatta SSM10 1	Proteles cristata T393 6	0,15939046
Panthera tigris NC010642 35	Helogale parvula SRR7637809 1	0,1593909
Panthera uncia NC010638 1	Cryptoprocta ferox CFC13 1	0,15939337
Panthera uncia NC010638 1	Arctictis binturong T605 2	0,15940021
Prionodon linsang ERR2391707 1	Neofelis nebulosa NC008450 3	0,15941155
Panthera pardus NC010641 5	Ichneumia albicauda T603 1	0,15941641
Neofelis nebulosa NC008450 3	Cryptoprocta ferox CFC13 1	0,15941849
Mellivora capensis T370 1	Martes melampus NC009678 1	0,15943199
Prionodon linsang ERR2391707 1	Panthera tigris NC010642 35	0,15945338
Salanoia concolor D378 1	Paradoxurus hermaphroditus NC039591 1	0,1594539
Prionodon pardicolor NC024569 2	Eupleres goudotii D128 1	0,15945403

Ursus arctos GU573491 207	Chrotogale owstoni T607 1	0,18692458
Gulo gulo NC009685 3	Canis lupus chanco NC010340 4	0,18692543
Vulpes zerda KJ603240 1	Leptailurus serval NC028316 1	0,18692568
Vulpes zerda KJ603240 1	Otocolobus manul NC028323 1	0,18692575
Leopardus geoffroyi NC028320 1	Arctocephalus townsendi NC008420 1	0,18692584
Prionailurus viverrinus NC028305 1	Canis anthus NC027956 2	0,18692657
Viverricula indica KX891751 1	Otocyon megalotis SAF1 2	0,18692666
Ursus arctos EU497665 29	Lutra sumatrana NC035810 1	0,18692724
Ursus arctos pruinosus MG066703 3	Arctocephalus australis MG023139 1	0,18692888
Odobenus rosmarus NC004029 29	Ailurus fulgens NC011124 1	0,18692903
Ursus arctos pruinosus MG066703 3	Fossa fossana D350 1	0,18692914
Xenogale naso C07XAR110 1	Canis aureus KT448274 1	0,18693005
Otocyon megalotis SAF1 2	Martes martes T302 3	0,18693072
Paradoxurus jerdoni MH464793 1	Martes foina NC020643 1	0,18693089
Panthera tigris amoyensis NC014770 2	Melogale moschata KP726273 1	0,18693213
Ursus arctos pruinosus MG066703 3	Phocarctos hookeri NC008418 1	0,18693289
Melogale moschata KP726273 1	Arctictis binturong T605 2	0,18693404
Ursus americanus JX196366 3	Civettictis civetta NC033378 1	0,18693656
Viverricula indica KX891745 1	Ursus arctos pruinosus MG066703 3	0,18693715
Ursus thibetanus formosanus NC00933	Procyon lotor AB462046 3	0,18693888
Martes foina NC020643 1	Chrysocyon brachyurus NC024172 1	0,1869406
Panthera tigris NC010642 35	Melogale moschata NC020644 1	0,18694118
Ursus maritimus GU573488 Svalbard	Galictis vittata T412 1	0,18694721
Ursus arctos isabellinus 18B5 2	Galictis vittata T412 1	0,18694726
Ursus spelaeus NC011112 8	Parahyaena brunnea NC038159 15	0,18694754
Panthera tigris amoyensis NC014770 2	Ictonyx striatus T299 1	0,18694839
Panthera leo NERO 19	Lutra lutra LC050126 1	0,18695016
Melursus ursinus NC009970 2	Leopardus jacobita NC028322 1	0,18695091
Lycalopex sechurae KT448284 1	Arctonyx collaris NC020645 1	0,18695404
Melogale moschata NC020644 1	Canis lupus familiaris NC002008 1231	0,18695422
Civettictis civetta NC033378 1	Arctocephalus townsendi NC008420 1	0,18695705
Neofelis nebulosa NC008450 3	Mustela kathiah NC023210 1	0,18695959
Profelis aurata NC028299 1	Gulo gulo NC009685 3	0,1869663
Felis catus NC001700 2	Cuon alpinus NC013445 3	0,18696722
Paradoxurus jerdoni MH464793 1	Lutra lutra NC011358 9	0,18697052
Lutra lutra LC050126 1	Canis mesomelas KT448280 1	0,18697163
Tremarctos ornatus NC009969 2	Urva semitorquata MH464789 1	0,18697426
Prionailurus planiceps NC028312 6	Tapirus terrestris T358	0,18697427
Lutra sumatrana NC035810 1	Cynictis penicillata T375 1	0,18697461
Meles leucurus NC039173 4	Bdeogale nigripes GLC15 1	0,186975
Xenogale naso C07XAR110 1	Callorhinus ursinus NC008415 1	0,18697536
Meles anakuma NC009677 1	Xenogale naso C07XAR110 1	0,1869754
Mustela eversmannii NC028013 1	Urva semitorquata MH464789 1	0,18697613
Prionodon linsang ERR2391707 1	Potos flavus T414 1	0,18697622
Lontra canadensis SRR10409165 1	Arctocephalus pusillus NC008417 1	0,18697856
Bdeogale nigripes GLC15 1	Arctocephalus gazella BK010918 1	0,18697912
Urva brachyura KY117547 1	Canis anthus NC027956 2	0,18698058
Viverra tangalunga MH464792 1	Cynogale bennetti KY117544 1	0,18698104
Prionodon linsang ERR2391707 1	Mustela kathiah NC023210 1	0,18698125
Odobenus rosmarus NC004029 29	Martes flavigula NC012141 3	0,18698235
Melogale moschata KP726273 1	Diplogale hosei MH464790 1	0,1869826
Viverricula indica KX891745 1	Arctocephalus townsendi NC008420 1	0,18698351
Lutra lutra NC011358 9	Ichneumia albicauda T603 1	0,18698354
Catopuma temminckii NC027115 41	Tapirus terrestris T358	0,18698363
Urva javanica T413 1	Arctocephalus pusillus NC008417 1	0,18698373
Urva brachyura KY117547 1	Arctocephalus pusillus NC008417 1	0,1869842
Lynx pardinus NC028319 161	Conepatus chinga NC042596 1	0,18698581
Melursus ursinus NC009970 2	Civettictis civetta NC033378 1	0,18698661
Panthera uncia NC010638 1	Lutra lutra LC050126 1	0,18698665
Panthera onca KP202264 2	Meles anakuma NC009677 1	0,18698736
Lutrogale perspicillata NC035811 1	Arctotherium sp NC030174 1	0,18698943
Poecilogale albinucha T602 1	Canis latrans NC008093 7	0,18698956
Parahyaena brunnea NC038159 15	Martes melampus NC009678 1	0,18698977
Potos flavus T414 1	Panthera onca KP202264 2	0,18698977
Vulpes lagopus NC026529 3	Genetta genetta T297 1	0,18698984
Catopuma temminckii NC027115 41	Arctocephalus forsteri KT693377 17	0,18699077
Catopuma badia NC028300 1	Arctocephalus forsteri KT693377 17	0,18699087
Vulpes vulpes NC008434 5	Panthera uncia NC010638 1	0,18699089
Puma concolor NC016470 22	Arctocephalus forsteri KT693377 17	0,18699118
Otocyon megalotis SAF1 2	Galidictis fasciata DM333 1	0,18699154
Vulpes vulpes NC008434 5	Leopardus jacobita NC028322 1	0,18699209
Felis chaus NC028307 1	Canis mesomelas KT448280 1	0,18699211
Ursus maritimus NC003428 31	Chrotogale owstoni T607 1	0,18699227
Neovison vison NC020641 3	Galidictis fasciata DM333 1	0,18699288
Urocyon littoralis catalinae KP129018	Lynx pardinus NC028319 161	0,18699294
Prionailurus viverrinus NC028305 1	Arctocephalus townsendi NC008420 1	0,18699302
Proteles cristata T393 6	Martes zibellina NC011579 39	0,18699491
Ursus arctos pruinosus MG066703 3	Arctocephalus townsendi NC008420 1	0,1869956
Puma concolor NC016470 22	Lutrogale perspicillata NC035811 1	0,18699816
Felis chaus NC028307 1	Arctocephalus pusillus NC008417 1	0,18699856
Melogale moschata KP726273 1	Ailuropoda melanoleuca NC009492 5	0,18699858
Otocyon megalotis SAF1 2	Caracal caracal NC028306 1	0,18700059
Mungos mungo/gambianus SRR77048	Galictis vittata T412 1	0,18700089
Melursus ursinus NC009970 2	Lutrogale perspicillata NC035811 1	0,18700129
Martes americana NC020642 1	Arctictis binturong T605 2	0,18700151
Xenogale naso C07XAR110 1	Helarctos malayanus NC009968 2	0,18700309
Paguma larvata PDDS11 2	Otaria byronia OTAB 1	0,18700367
Mungos mungo/gambianus SRR77048	Helarctos malayanus NC009968 2	0,187004
Helarctos malayanus NC009968 2	Cynictis penicillata T375 1	0,18700415
Helarctos malayanus NC009968 2	Galidictis fasciata DM333 1	0,18700448
Ursus maritimus GU573488 Svalbard	Prionailurus rubiginosus NC028304 2	0,18700458
Zalophus wollebaeki SRR4431565 1	Cryptoprocta ferox CFC13 1	0,18700616
Ursus thibetanus thibetanus NC011118	Lontra canadensis SRR10409165 1	0,18700656
Ursus spelaeus NC011112 8	Bassaricyon neblina SRX1097850 1	0,18700667
Zalophus wollebaeki SRR4431565 1	Profelis aurata NC028299 1	0,18700777
Leopardus pardalis T262 1	Ailuropoda melanoleuca NC009492 5	0,18700871
Speothos venaticus C48 2	Catopuma badia NC028300 1	0,18701021
Galidictis fasciata DM333 1	Canis lupus chanco NC010340 4	0,18701123
Lynx rufus NC014456 3	Galictis vittata T412 1	0,18701518
Galictis vittata T412 1	Felis margarita NC028308 1	0,18701573
Helarctos malayanus NC009968 2	Felis catus NC001700 2	0,18701625
Ursus arctos pruinosus MG066703 3	Leopardus colocolo NC028314 1	0,18702221
Galictis vittata T412 1	Arctictis binturong T605 2	0,1870233

Proteles cristata T393 6	Profelis aurata NC028299 1	0,15947074
Prionodon linsang ERR2391707 1	Panthera pardus NC010641 5	0,15947817
Prionodon linsang ERR2391707 1	Mungos mungo MMC7 1	0,15950558
Paradoxurus hermaphroditus NC039591 1	Eupleres goudotii D128 1	0,15950915
Panthera uncia KP202269 1	Urva brachyura KY117547 1	0,15951619
Prionodon pardicolor NC024569 2	Urva semitorquata MH464789 1	0,15951623
Prionodon pardicolor NC024569 2	Galerella sanguinea T378 1	0,15951677
Panthera leo spelaea KX258452 2	Ichneumia albicauda T603 1	0,15951702
Hyaena hyaena NC020669 1	Genetta genetta T297 1	0,15952726
Mellivora capensis T370 1	Martes foina NC020643 1	0,15957312
Panthera uncia NC010638 1	Mungos mungo MMC7 1	0,15958416
Panthera pardus japonensis KJ866876 8	Ichneumia albicauda T603 1	0,15958467
Neovison vison NC020641 3	Bassariscus sumichrasti SRX1099089 1	0,15958944
Proteles cristata T393 6	Genetta servalina NC024568 2	0,15959549
Genetta servalina NC024568 2	Crocuta crocuta NC020670 3	0,15960564
Proteles cristata T393 6	Felis catus NC001700 2	0,15960689
Panthera pardus NC010641 5	Urva javanica/auropunctata NC006835 1	0,15965064
Panthera uncia NC010638 1	Helogale parvula SRR7637809 1	0,15965259
Paguma larvata PDDS11 2	Galidia elegans D146 1	0,15965435
Cryptoprocta ferox CFC13 1	Arctictis binturong T605 2	0,15966894
Prionailurus planiceps NC028312 6	Crocuta crocuta NC020670 3	0,15967448
Paradoxurus hermaphroditus NC039591 1	Neofelis nebulosa NC008450 3	0,15968841
Crocuta crocuta NC020670 3	Acinonyx jubatus NC005212 3	0,15969436
Prionodon linsang ERR2391707 1	Panthera pardus japonensis KJ866876 8	0,1597169
Suricata suricatta SSM10 1	Panthera leo spelaea KX258452 2	0,15972015
Panthera leo spelaea KX258452 2	Cynictis penicillata T375 1	0,15972017
Panthera tigris amoyensis NC014770 2	Helogale parvula SRR7637809 1	0,15972754
Suricata suricatta SSM10 1	Panthera tigris amoyensis NC014770 2	0,15972796
Parahyaena brunnea NC038159 15	Panthera leo spelaea KX258452 2	0,15973032
Neofelis nebulosa NC008450 3	Urva semitorquata MH464789 1	0,15974948
Panthera pardus NC010641 5	Bdeogale nigripes GLC15 1	0,15975317
Mellivora capensis T370 1	Martes pennanti NC020664 16	0,15978019
Odobenus rosmarus NC004029 29	Halichoerus grypus NC001602 2	0,15978335
Panthera onca KP202264 2	Urva brachyura KY117547 1	0,15978514
Panthera pardus japonensis KJ866876 8	Urva javanica T413 1	0,159786
Viverricula indica NC025296 2	Proteles cristata T393 6	0,1598025
Phoca groenlandica NC008429 54	Odobenus rosmarus NC004029 29	0,15980428
Proteles cristata T393 6	Panthera pardus japonensis KJ866876 8	0,15980826
Mellivora capensis T370 1	Martes flavigula NC012141 3	0,15982376
Prionodon linsang ERR2391707 1	Urva brachyura KY117547 1	0,15984379
Procyon lotor AB462049 4	Bassaricyon neblina SRX1097850 1	0,15985011
Prionodon pardicolor NC024569 2	Crossarchus platycephalus C7R66 1	0,15985255
Panthera leo spelaea KX258452 2	Bdeogale nigripes GLC15 1	0,15985332
Prionodon linsang ERR2391707 1	Panthera tigris amoyensis NC014770 2	0,15985751
Proteles cristata T393 6	Civettictis civetta NC033378 1	0,15990123
Prionodon linsang ERR2391707 1	Panthera uncia NC010638 1	0,15991837
Panthera uncia KP202269 1	Cynictis penicillata T375 1	0,15992087
Paguma larvata PDDS11 2	Cryptoprocta ferox CFC13 1	0,15992188
Proteles cristata T393 6	Civettictis civetta GLC19 1	0,15993254
Paradoxurus jerdoni MH464793 1	Panthera onca NC022842 1	0,159933
Proteles cristata T393 6	Felis chaus NC028307 1	0,159945
Felis catus NC001700 2	Cynogale bennetti KY117544 1	0,15996182
Parahyaena brunnea NC038159 15	Neofelis nebulosa NC008450 3	0,15996949
Prionodon linsang ERR2391707 1	Urva semitorquata MH464789 1	0,15997845
Pusa caspica NC008431 1	Odobenus rosmarus NC004029 29	0,16000327
Panthera tigris amoyensis NC014770 2	Hyaena hyaena NC020669 1	0,16000775
Panthera tigris NC010642 35	Urva javanica/auropunctata NC006835 1	0,16002655
Prionodon pardicolor NC024569 2	Xenogale naso C07XAR110 1	0,16005515
Panthera onca KP202264 2	Ichneumia albicauda T603 1	0,1600576
Panthera tigris NC010642 35	Diplogale hosei MH464790 1	0,16006009
Neofelis nebulosa NC008450 3	Xenogale naso C07XAR110 1	0,16008975
Paradoxurus hermaphroditus NLNC 1	Cryptoprocta ferox CFC13 1	0,1601239
Panthera tigris amoyensis NC014770 2	Urva javanica T413 1	0,16012935
Viverricula indica KX891751 1	Hyaena hyaena NC020669 1	0,16013163
Leptailurus serval NC028316 1	Crocuta crocuta NC020670 3	0,16014731
Neofelis nebulosa NC008450 3	Atilax paludinosus T606 1	0,16015753
Leptonychotes weddellii NC008424 1	Arctotherium sp NC030174 1	0,16018587
Panthera pardus japonensis KJ866876 8	Cynictis penicillata T375 1	0,16019002
Panthera uncia KP202269 1	Xenogale naso C07XAR110 1	0,16019168
Suricata suricatta SSM10 1	Panthera uncia KP202269 1	0,16019173
Panthera pardus japonensis KJ866876 8	Urva brachyura KY117547 1	0,16025667
Panthera tigris NC010642 35	Eupleres goudotii D128 1	0,16026683
Panthera uncia NC010638 1	Hyaena hyaena NC020669 1	0,16027136
Pusa sibirica NC008432 2	Odobenus rosmarus NC004029 29	0,16027266
Proteles cristata T393 6	Otocolobus manul NC028323 1	0,16028285
Panthera pardus NC010641 5	Cynictis penicillata T375 1	0,16029148
Panthera tigris amoyensis NC014770 2	Urva javanica/auropunctata NC006835 1	0,16029586
Nandinia binotata NC024567 1	Lobodon carcinophaga NC008423 1	0,16032238
Prionodon linsang ERR2391707 1	Panthera leo spelaea KX258452 2	0,1603272
Panthera leo spelaea KX258452 2	Xenogale naso C07XAR110 1	0,16033068
Panthera leo spelaea KX258452 2	Hyaena hyaena NC020669 1	0,16033429
Viverricula indica KX891751 1	Parahyaena brunnea NC038159 15	0,16034168
Proteles cristata T393 6	Panthera leo NERO 19	0,16038163
Viverra zibetha T609 1	Parahyaena brunnea NC038159 15	0,16040879
Felis silvestris lybica KP202275 4	Crocuta crocuta NC020670 3	0,16041496
Panthera pardus japonensis KJ866876 8	Urva javanica/auropunctata NC006835 1	0,16042484
Lynx lynx NC027083 4	Cynogale bennetti KY117544 1	0,16043298
Mellivora capensis T370 1	Martes americana NC020642 1	0,16044149
Paradoxurus jerdoni MH464793 1	Galidia elegans D146 1	0,16046323
Hydrurga leptonyx NC008425 1	Bassariscus sumichrasti SRX1099089 1	0,16046441
Panthera tigris amoyensis NC014770 2	Galerella sanguinea T378 1	0,16046751
Parahyaena brunnea NC038159 15	Paguma larvata PDDS11 2	0,16046947
Hemigalus derbyanus MH464791 1	Cryptoprocta ferox CFC13 1	0,16047546
Parahyaena brunnea NC038159 15	Genetta genetta T297 1	0,16047595
Parahyaena brunnea NC038159 15	Panthera tigris amoyensis NC014770 2	0,16048291
Prionodon linsang ERR2391707 1	Atilax paludinosus T606 1	0,1605171
Panthera uncia KP202269 1	Galerella sanguinea T378 1	0,16052853
Odobenus rosmarus NC004029 29	Cystophora cristata NC008427 1	0,16053991
Neofelis nebulosa NC008450 3	Helogale parvula SRR7637809 1	0,16055772
Panthera uncia KP202269 1	Urva javanica/auropunctata NC006835 1	0,16055949
Lynx canadensis NC028313 1	Cynogale bennetti KY117544 1	0,16056772
Prionodon linsang ERR2391707 1	Cynictis penicillata T375 1	0,16058443
Taxidea taxus NC020646 1	Cystophora cristata NC008427 1	0,16059066

Panthera pardus NC010641 5	Ailurus fulgens NC011124 1	0,18702599
Ursus arctos GU573486 5	Leopardus colocolo NC028314 1	0,18702844
Profelis aurata NC028299 1	Martes flavigula NC012141 3	0,18703201
Otocyon megalotis SAF1 2	Civettictis civetta NC033378 1	0,18703666
Profelis aurata NC028299 1	Eumetopias jubatus NC004030 10	0,18703969
Urva javanica T413 1	Canis mesomelas KT448280 1	0,18704151
Cynictis penicillata T375 1	Callorhinus ursinus NC008415 1	0,18704168
Mungos mungo/gambianus SRR77048	Melogale moschata KP726273 1	0,18704231
Nyctereutes procyonoides NC013700 3	Enhydra lutris NC009692 1	0,18704459
Eupleres goudotii D128 1	Arctocephalus gazella BK010918 1	0,18704793
Prionailurus viverrinus NC028305 1	Conepatus chinga NC042596 1	0,18704918
Lutrogale perspicillata NC035811 1	Galidictis fasciata DM333 1	0,18705024
Melogale moschata NC020644 1	Helogale parvula SRR7637809 1	0,18705039
Mungotictis decemlineata NC027828 1	Arctocephalus pusillus NC008417 1	0,1870518
Lynx lynx NC027083 4	Conepatus chinga NC042596 1	0,18705206
Panthera pardus japonensis KJ866876	Ailurus fulgens styani NC009691 1	0,18705308
Vulpes lagopus NC026529 3	Arctodus simus NC011116 1	0,1870552
Galerella sanguinea T378 1	Arctocephalus australis MG023139 1	0,18705539
Nyctereutes procyonoides NC013700 3	Martes pennanti NC020664 16	0,18705602
Hyaena hyaena NC020669 1	Bassariscus sumichrasti SRX1099089 1	0,18705742
Lutra sumatrana NC035810 1	Canis aureus KT448274 1	0,18705769
Felis margarita NC028308 1	Arctocephalus forsteri KT693377 17	0,18705834
Puma yagouaroundi NC028311 1	Arctocephalus australis MG023139 1	0,18705851
Lutra sumatrana NC035810 1	Crocuta crocuta NC020670 3	0,18705867
Lynx canadensis NC028313 1	Arctocephalus townsendi NC008420 1	0,1870587
Ursus maritimus GU573488 Svalbard	Chrotogale owstoni T607 1	0,18705967
Ursus thibetanus mupinensis NC00875	Lontra canadensis SRR10409165 1	0,18706056
Meles meles T303 3	Lycaon pictus NC028427 2	0,18706077
Puma yagouaroundi NC028311 1	Neophoca cinerea NC008419 1	0,18706295
Urocyon littoralis catalinae KP129018	Catopuma badia NC028300 1	0,18706341
Catopuma temminckii NC027115 41	Aonyx cinerea NC035814 2	0,18706349
Pardofelis marmorata NLN3 2	Aonyx cinerea NC035814 2	0,18706393
Ursus arctos EU497665 29	Paradoxurus jerdoni MH464793 1	0,18706642
Viverra tangalunga MH464792 1	Panthera tigris amoyensis NC014770 2	0,18706673
Puma concolor NC016470 22	Arctotherium sp NC030174 1	0,18706696
Vulpes lagopus NC026529 3	Panthera tigris amoyensis NC014770 2	0,18706721
Ursus thibetanus thibetanus NC01111	Mungos mungo MMC7 1	0,18707117
Ursus arctos EU497665 29	Profelis aurata NC028299 1	0,18707219
Ursus spelaeus NC011112 8	Lontra canadensis SRR10409165 1	0,18707412
Zalophus wollebaeki SRR4431565 1	Ichneumia albicauda T603 1	0,18707627
Speothos venaticus C48 2	Catopuma temminckii NC027115 41	0,1870769
Speothos venaticus C48 2	Prionailurus bengalensis CKM45 20	0,187077
Canis latrans NC008093 7	Ailuropoda melanoleuca NC009492 5	0,18707736
Felis silvestris lybica KP202275 4	Ailuropoda melanoleuca NC009492 5	0,18707739
Viverricula indica KX891745 1	Galictis vittata T412 1	0,18707769
Mungotictis decemlineata NC027828 1	Canis lupus chanco NC010340 4	0,18707867
Ursus spelaeus EU327344 13	Prionailurus planiceps KY682741 4	0,18707947
Ursus thibetanus formosanus NC0093	Panthera pardus japonensis KJ866876	0,18707968
Ursus spelaeus NC011112 8	Paradoxurus hermaphroditus NLNC 1	0,18708093
Ursus arctos EU497665 29	Arctictis binturong T605 2	0,18708095
Ursus maritimus GU573488 Svalbard	Ictonyx striatus T299 1	0,18708108
Ursus spelaeus NC011112 8	Genetta genetta T297 1	0,18708144
Leopardus tigrinus NC028317 1	Helarctos malayanus NC009968 2	0,18708409
Ursus thibetanus thibetanus NC01111	Prionailurus viverrinus NC028305 1	0,18708494
Zalophus californianus NC008416 1	Paradoxurus hermaphroditus NLNC 1	0,18708517
Melursus ursinus NC009970 2	Felis chaus NC028307 1	0,187086
Ursus spelaeus EU327344 13	Galictis vittata T412 1	0,18709594
Ursus spelaeus EU327344 13	Mephitis mephitis NC020648 1	0,18709612
Mustela itatsi NC034330 19	Cuon alpinus NC013445 3	0,1870967
Meles leucurus NC039173 4	Lycalopex sechurae KT448284 1	0,18709797
Ursus americanus JX196366 3	Procyon lotor AB462046 3	0,18710022
Prionailurus bengalensis NC028301 12	Arctocephalus forsteri NC004023 28	0,18710087
Ictonyx striatus T299 1	Ailuropoda melanoleuca NC009492 5	0,1871015
Mustela eversmannii NC028013 1	Arctocephalus forsteri KT693377 17	0,18710968
Eupleres goudotii D128 1	Enhydra lutris NC009692 1	0,18710975
Mustela eversmannii NC028013 1	Mungos mungo/gambianus SRR77048	0,18711051
Cynictis penicillata T375 1	Arctocephalus australis MG023139 1	0,18711203
Mustela putorius NC020638 4	Bdeogale nigripes GLC15 1	0,1871126
Viverra tangalunga MH464792 1	Urva javanica T413 1	0,1871132
Vulpes ferrilata NC027935 1	Melogale moschata KP726273 1	0,18711357
Arctocephalus forsteri NC004023 28	Acinonyx jubatus NC005212 3	0,18711361
Xenogale naso C07XAR110 1	Canis anthus NC027956 2	0,18711409
Urva brachyura KY117547 1	Ailurus fulgens styani NC009691 1	0,18711485
Mustela sibirica AP017394 11	Mungotictis decemlineata NC027828 1	0,18711667
Paradoxurus jerdoni MH464793 1	Arctocephalus forsteri KT693377 17	0,18711671
Vulpes ferrilata NC027935 1	Mustela sibirica AP017394 11	0,18711708
Salanoia concolor D378 1	Ailurus fulgens styani NC009691 1	0,1871185
Mustela sibirica AP017394 11	Galerella sanguinea T378 1	0,18712068
Canis anthus NC027956 2	Bassariscus sumichrasti SRX1099089 1	0,18712206
Lutra sumatrana NC035810 1	Ichneumia albicauda T603 1	0,1871223
Martes foina NC020643 1	Lycalopex sechurae KT448284 1	0,18712242
Panthera uncia KP202269 1	Callorhinus ursinus NC008415 1	0,18712264
Mustela altaica NC021751 1	Atilax paludinosus T606 1	0,18712319
Mustela sibirica AP017394 11	Cryptoprocta ferox CFC13 1	0,18712392
Otocyon megalotis SAF1 2	Mungotictis decemlineata NC027828 1	0,18712589
Profelis aurata NC028299 1	Mustela sibirica AP017394 11	0,18712589
Paradoxurus hermaphroditus NC03959	Melogale moschata NC020644 1	0,18712603
Panthera pardus japonensis KJ866876	Lutra sumatrana NC035810 1	0,18712622
Leopardus guigna NC028321 1	Arctocephalus townsendi NC008420 1	0,18712769
Parahyaena brunnea NC038159 15	Mustela eversmannii NC028013 1	0,18712784
Viverricula indica NC025296 2	Phocarctos hookeri NC008418 1	0,18712844
Taxidea taxus NC020646 1	Panthera uncia NC010638 1	0,1871286
Conepatus chinga NC042596 1	Acinonyx jubatus NC005212 3	0,18712883
Ictonyx striatus T299 1	Urva javanica T413 1	0,18712966
Ursus spelaeus NC011112 8	Conepatus chinga NC042596 1	0,18712983
Zalophus wollebaeki SRR4431565 1	Cynictis penicillata T375 1	0,18713076
Panthera leo spelaea KX258452 2	Enhydra lutris NC009692 1	0,18713355
Viverricula indica NC025296 2	Ursus arctos GU573491 207	0,18713433
Viverricula indica NC025296 2	Ursus arctos EU497665 29	0,18713443
Phocarctos hookeri NC008418 1	Galictis vittata T412 1	0,18713465
Ursus maritimus GU573488 Svalbard	Genetta genetta T297 1	0,18713482
Ursus maritimus GU573488 Svalbard	Paguma larvata PDDS11 2	0,18713491
Puma concolor NC016470 22	Otaria byronia OTAB 1	0,18713556

Panthera uncia NC010638 1	Galerella sanguinea T378 1	0,16059595
Paradoxurus hermaphroditus NC039591 1	Mungotictis decemlineata NC027828 1	0,16059825
Crocuta crocuta NC020670 3	Civettictis civetta GLC19 1	0,16061515
Neofelis nebulosa NC008450 3	Chrotogale owstoni T607 1	0,16062416
Panthera uncia NC010638 1	Urva brachyura KY117547 1	0,16066106
Prionodon pardicolor NC024569 2	Urva brachyura KY117547 1	0,16066213
Panthera uncia NC010638 1	Mungos mungo/gambianus SRR7704821 1	0,16066217
Panthera tigris amoyensis NC014770 2	Xenogale naso C07XAR110 1	0,16067016
Parahyaena brunnea NC038159 15	Panthera uncia NC010638 1	0,16067877
Proteles cristata T393 6	Acinonyx jubatus NC005212 3	0,16070235
Proteles cristata T393 6	Panthera pardus NC010641 5	0,16072132
Crocuta crocuta NC020670 3	Civettictis civetta NC033378 1	0,16072179
Salanoia concolor D378 1	Prionodon pardicolor NC024569 2	0,16073391
Panthera tigris NC010642 35	Galerella sanguinea T378 1	0,16073695
Viverricula indica KX891745 1	Proteles cristata T393 6	0,16074009
Panthera tigris NC010642 35	Hyaena hyaena NC020669 1	0,16074857
Parahyaena brunnea NC038159 15	Panthera tigris NC010642 35	0,16075231
Monachus monachus NC044972 5	Arctodus simus NC011116 1	0,16076142
Leptonychotes weddellii NC008424 1	Bassariscus sumichrasti SRX1099089 1	0,16079096
Galerella sanguinea T378 1	Cynogale bennetti KY117544 1	0,16079557
Viverricula indica NC025296 2	Crocuta crocuta NC020670 3	0,16080226
Paradoxurus jerdoni MH464793 1	Panthera leo spelaea KX258452 2	0,16080995
Neofelis nebulosa NC008450 3	Galerella sanguinea T378 1	0,16082768
Neofelis nebulosa NC008450 3	Hyaena hyaena NC020669 1	0,16084103
Mungos mungo MMC7 1	Cynogale bennetti KY117544 1	0,1608492
Fossa fossana D350 1	Cynogale bennetti KY117544 1	0,16085185
Suricata suricatta SSM10 1	Paradoxurus hermaphroditus NC039591 1	0,16086076
Paradoxurus hermaphroditus NC039591 1	Panthera tigris amoyensis NC014770 2	0,16087186
Paradoxurus jerdoni MH464793 1	Panthera tigris amoyensis NC014770 2	0,16087243
Pusa hispida NC 008433 1	Arctodus simus NC011116 1	0,16087401
Panthera uncia NC010638 1	Urva javanica T413 1	0,1609309
Neovison vison NC020641 3	Lobodon carcinophaga NC008423 1	0,16093571
Viverricula indica KX891745 1	Crocuta crocuta NC020670 3	0,16095352
Proteles cristata T393 6	Panthera leo spelaea KX258452 2	0,16096033
Mustela erminea T305 2	Mellivora capensis T370 1	0,16099718
Panthera leo spelaea KX258452 2	Diplogale hosei MH464790 1	0,16099738
Panthera uncia KP202269 1	Bdeogale nigripes GLC15 1	0,16099921
Paguma larvata PDDS11 2	Hyaena hyaena NC020669 1	0,16100021
Phoca fasciata NC008428 1	Arctodus simus NC011116 1	0,16100423
Hydrurga leptonyx NC008425 1	Homotherium latidens MF871702 3	0,16101438
Proteles cristata T393 6	Panthera onca KP202264 2	0,16101991
Panthera pardus NC010641 5	Urva brachyura KY117547 1	0,16103198
Pusa sibirica NC008432 2	Arctodus simus NC011116 1	0,1610643
Martes foina NC020643 1	Bassariscus sumichrasti SRX1099089 1	0,16107215
Paradoxurus hermaphroditus NC039591 1	Panthera tigris NC010642 35	0,16107418
Neofelis nebulosa NC008450 3	Diplogale hosei MH464790 1	0,16111496
Procyon lotor AB462049 4	Mustela erminea T305 2	0,16112736
Panthera uncia KP202269 1	Ichneumia albicauda T603 1	0,16113539
Paradoxurus hermaphroditus NC039591 1	Panthera uncia KP202269 1	0,16113569
Lobodon carcinophaga NC008423 1	Bassariscus sumichrasti SRX1099089 1	0,16113845
Panthera tigris NC010642 35	Urva javanica T413 1	0,16113949
Paradoxurus hermaphroditus NC039591 1	Panthera leo spelaea KX258452 2	0,16114165
Prionodon linsang ERR2391707 1	Xenogale naso C07XAR110 1	0,16119041
Procyon lotor AB462046 3	Mustela erminea T305 2	0,16119477
Mustela erminea T305 2	Lobodon carcinophaga NC008423 1	0,16119775
Prionodon pardicolor NC024569 2	Bdeogale nigripes GLC15 1	0,16120046
Panthera leo spelaea KX258452 2	Galerella sanguinea T378 1	0,16120085
Urva javanica/auropunctata NC006835 1	Cynogale bennetti KY117544 1	0,16122255
Pardofelis marmorata NLN3 2	Cynogale bennetti KY117544 1	0,16124132
Prionodon linsang ERR2391707 1	Bdeogale nigripes GLC15 1	0,16125774
Panthera tigris NC010642 35	Xenogale naso C07XAR110 1	0,16127627
Mellivora capensis T370 1	Lutra sumatrana NC035810 1	0,16131638
Nasua nasua NC020647 1	Bassariscus sumichrasti SRX1099089 1	0,16132934
Pusa caspica NC008431 1	Arctodus simus NC011116 1	0,16140094
Neofelis nebulosa NC008450 3	Galidia elegans D146 1	0,16143544
Mustela altaica NC021751 1	Bassariscus sumichrasti SRX1099089 1	0,16147397
Panthera tigris amoyensis NC014770 2	Ichneumia albicauda T603 1	0,1614791
Taxidea taxus NC020646 1	Potos flavus T414 1	0,16151594
Panthera uncia NC010638 1	Xenogale naso C07XAR110 1	0,16153826
Suricata suricatta SSM10 1	Panthera tigris NC010642 35	0,16154598
Proteles cristata T393 6	Panthera onca NC022842 1	0,16156752
Neofelis nebulosa NC008450 3	Ichneumia albicauda T603 1	0,16156977
Cynogale bennetti KY117544 1	Bdeogale nigripes GLC15 1	0,16158916
Cynogale bennetti KY117544 1	Cynictis penicillata T375 1	0,1615894
Panthera uncia NC010638 1	Cynictis penicillata T375 1	0,16160443
Paradoxurus hermaphroditus NC039591 1	Galidia elegans D146 1	0,1616082
Prionodon pardicolor NC024569 2	Mungotictis decemlineata NC027828 1	0,16160917
Proteles cristata T393 6	Urva brachyura KY117547 1	0,16161306
Crocuta crocuta NC020670 3	Caracal caracal NC028306 1	0,16162826
Hemigalus derbyanus MH464791 1	Crocuta crocuta NC020670 3	0,16163258
Prionodon linsang ERR2391707 1	Cynogale bennetti KY117544 1	0,16164024
Prionodon linsang ERR2391707 1	Helogale parvula SRR7637809 1	0,16166173
Potos flavus T414 1	Leptonychotes weddellii NC008424 1	0,16166407
Erignathus barbatus NC008426 1	Arctodus simus NC011116 1	0,16167088
Potos flavus T414 1	Hydrurga leptonyx NC008425 1	0,16167472
Panthera tigris amoyensis NC014770 2	Bdeogale nigripes GLC15 1	0,16167967
Panthera onca KP202264 2	Crocuta crocuta NC020670 3	0,16169177
Panthera uncia NC010638 1	Diplogale hosei MH464790 1	0,16169225
Felis margarita NC028308 1	Crocuta crocuta NC020670 3	0,16169482
Neofelis nebulosa NC008450 3	Bdeogale nigripes GLC15 1	0,16170299
Martes melampus NC009678 1	Bassariscus sumichrasti SRX1099089 1	0,16172686
Prionodon pardicolor NC024569 2	Galidictis fasciata DM333 1	0,1617436
Urva brachyura KY117547 1	Cynogale bennetti KY117544 1	0,16185845
Hydrurga leptonyx NC008425 1	Enhydra lutris NC009692 1	0,16186605
Meles meles T303 3	Hydrurga leptonyx NC008425 1	0,16186769
Potos flavus T414 1	Lobodon carcinophaga NC008423 1	0,16187628
Panthera pardus NC010641 5	Cynogale bennetti KY117544 1	0,1618765
Lutra sumatrana NC035810 1	Bassariscus sumichrasti SRX1099089 1	0,16192968
Prionodon pardicolor NC024569 2	Atilax paludinosus T606 1	0,16194041
Panthera tigris amoyensis NC014770 2	Cynictis penicillata T375 1	0,16194765
Felis nigripes NC028309 1	Crocuta crocuta NC020670 3	0,16196311
Panthera tigris NC010642 35	Crocuta crocuta NC020670 3	0,16196764
Neofelis nebulosa NC008450 3	Mungos mungo MMC7 1	0,16197155

Speothos venaticus C48 2	Civettictis civetta GLC19 1	0,18713586
Ursus maritimus NC003428 31	Prionailurus rubiginosus NC028304 2	0,18713926
Ursus maritimus NC003428 31	Prionailurus bengalensis NC028301 12	0,1871395
Martes zibellina NC011579 39	Hemigalus derbyanus MH464791 1	0,18714049
Lycalopex sechurae KT448284 1	Genetta abyssinica MG489822 1	0,18714283
Mustela nivalis T306 5	Chrysocyon brachyurus NC024172 1	0,18714409
Genetta genetta T297 1	Ailuropoda melanoleuca NC009492 5	0,18714447
Lutra lutra NC011358 9	Canis lupus chanco NC010340 4	0,18714523
Galictis vittata T412 1	Arctotherium sp NC030174 1	0,18714556
Mustela nivalis T306 5	Ailuropoda melanoleuca NC009492 5	0,1871459
Caracal caracal NC028306 1	Ailuropoda melanoleuca NC009492 5	0,18714601
Spilogale putorius NC010497 1	Eumetopias jubatus NC004030 10	0,1871461
Ursus thibetanus thibetanus NC01111	Cryptoprocta ferox CFC13 1	0,18714671
Ursus thibetanus formosanus NC0093	Prionodon pardicolor NC024569 2	0,18714726
Viverricula indica KX891745 1	Ursus arctos AP012576 6	0,18714735
Ursus thibetanus laniger MH281753 2	Panthera pardus japonensis KJ866876	0,18714914
Phocarctos hookeri NC008418 1	Leopardus colocolo NC028314 1	0,18714944
Paradoxurus hermaphroditus NLNC 1	Helarctos malayanus NC009968 2	0,18715189
Puma concolor NC016470 22	Helarctos malayanus NC009968 2	0,18715225
Diplogale hosei MH464790 1	Tapirus terrestris T358	0,18715376
Procyon lotor AB462049 4	Arctocephalus forsteri NC004023 28	0,18715644
Martes pennanti NC020664 16	Urva javanica/auropunctata NC006835	0,18715714
Arctictis binturong T605 2	Ailuropoda melanoleuca NC009492 5	0,18716155
Panthera onca NC022842 1	Canis lupus chanco NC010340 4	0,18716288
Mustela altaica NC021751 1	Urva javanica/auropunctata NC006835	0,18716462
Viverra tangalunga MH464792 1	Panthera pardus NC010641 5	0,18716612
Lycalopex sechurae KT448284 1	Genetta genetta T297 1	0,18716635
Panthera leo NERO 19	Arctodus simus NC011116 1	0,18716785
Felis nigripes NC028309 1	Cuon alpinus NC013445 3	0,18716912
Prionailurus planiceps NC028312 6	Cuon alpinus NC013445 3	0,18717024
Prionailurus bengalensis CKM45 20	Arctocephalus forsteri NC004023 28	0,18717065
Ursus americanus JX196366 3	Otocolobus manul NC028323 1	0,18717158
Lutra lutra NC011358 9	Helogale parvula SRR7637809 1	0,18717247
Martes melampus NC009678 1	Urva brachyura KY117547 1	0,18717365
Ursus americanus JX196366 3	Prionailurus bengalensis CKM45 20	0,18717387
Paradoxurus hermaphroditus NC03959	Enhydra lutris NC009692 1	0,18717454
Mustela eversmannii NC028013 1	Arctocephalus australis MG023139 1	0,18717703
Melogale moschata KP726273 1	Cynictis penicillata T375 1	0,18717815
Mustela putorius NC020638 4	Helogale parvula SRR7637809 1	0,1871787
Ursus thibetanus mupinensis NC00875	Canis lupus chanco NC010340 4	0,18717924
Cynictis penicillata T375 1	Arctocephalus forsteri KT693377 17	0,18717948
Potos flavus T414 1	Urva brachyura KY117547 1	0,18718071
Prionodon pardicolor NC024569 2	Lutra lutra LC050126 1	0,18718219
Prionodon linsang ERR2391707 1	Mustela sibirica NC020637 6	0,18718255
Meles anakuma NC009677 1	Galidia elegans D146 1	0,1871832
Procyon lotor AB462049 4	Urva semitorquata MH464789 1	0,18718337
Vulpes zerda KJ603240 1	Mustela sibirica AP017394 11	0,18718487
Otaria byronia OTAB 1	Urva semitorquata MH464789 1	0,18718499
Martes pennanti NC020664 16	Urva javanica T413 1	0,18718611
Lynx rufus NC014456 3	Conepatus chinga NC042596 1	0,1871877
Vulpes lagopus NC026529 3	Paradoxurus jerdoni MH464793 1	0,18718943
Ursus maritimus NC003428 31	Lutra lutra LC050126 1	0,1871912
Mustela kathiah NC023210 1	Cryptoprocta ferox CFC13 1	0,18719152
Otocyon megalotis SAF1 2	Martes melampus NC009678 1	0,18719171
Viverricula indica KX891745 1	Urocyon littoralis catalinae KP129018	0,18719286
Lontra canadensis SRR10409165 1	Leopardus guigna NC028321 1	0,18719654
Crocuta crocuta NC020670 3	Callorhinus ursinus NC008415 1	0,18719681
Martes martes T302 3	Lycalopex sechurae KT448284 1	0,18719742
Melogale moschata NC020644 1	Lycalopex sechurae KT448284 1	0,18719779
Otocyon megalotis SAF1 2	Civettictis civetta GLC19 1	0,18719792
Meles anakuma NC009677 1	Canis aureus KT448274 1	0,187198
Zalophus wollebaeki SRR4431565 1	Helogale parvula SRR7637809 1	0,1871987
Viverricula indica KX891745 1	Ursus arctos EU497665 29	0,18719986
Paguma larvata PDDS11 2	Martes foina NC020643 1	0,18720053
Ursus arctos GU573486 5	Chrotogale owstoni T607 1	0,18720083
Ursus arctos GU573491 207	Paradoxurus jerdoni MH464793 1	0,18720107
Otaria byronia OTAB 1	Ichneumia albicauda T603 1	0,18720162
Otocyon megalotis SAF1 2	Felis margarita NC028308 1	0,187203
Taxidea taxus NC020646 1	Speothos venaticus C48 2	0,18720335
Melursus ursinus NC009970 2	Melogale moschata V0735A 1	0,18720388
Ictonyx striatus T299 1	Galerella sanguinea T378 1	0,18720393
Otaria byronia OTAB 1	Leopardus jacobita NC028322 1	0,18720402
Mustela sibirica NC020637 6	Ailuropoda melanoleuca NC009492 5	0,18720515
Ursus maritimus GU573488 Svalbard	Leopardus wiedii NC028318 1	0,18720539
Mungotictis decemlineata NC027828 1	Helarctos malayanus NC009968 2	0,18720606
Mungos mungo MMC7 1	Helarctos malayanus NC009968 2	0,18720642
Speothos venaticus C48 2	Felis margarita NC028308 1	0,18720683
Helarctos malayanus NC009968 2	Bassaricyon neblina SRX1097850 1	0,18720731
Speothos venaticus C48 2	Otocolobus manul NC028323 1	0,18720819
Zalophus wollebaeki SRR4431565 1	Panthera onca KP202264 2	0,18720882
Ursus maritimus NC003428 31	Prionailurus bengalensis CKM45 20	0,1872099
Ursus arctos pruinosus MG066703 3	Catopuma badia NC028300 1	0,18721019
Canis adustus KT448271 1	Aonyx cinerea NC035814 2	0,18721198
Zalophus californianus NC008416 1	Nyctereutes procyonoides NC013700 3	0,18721317
Zalophus wollebaeki SRR4431565 1	Mephitis mephitis NC020648 1	0,18721357
Viverra zibetha T609 1	Melursus ursinus NC009970 2	0,18721515
Ursus arctos EU497665 29	Ictonyx striatus T299 1	0,18721582
Mustela altaica NC021751 1	Melursus ursinus NC009970 2	0,18721633
Prionailurus bengalensis CKM45 20	Mephitis mephitis NC020648 1	0,18721707
Viverricula indica NC025296 2	Melursus ursinus NC009970 2	0,18721783
Panthera onca NC022842 1	Martes foina NC020643 1	0,18721817
Ursus thibetanus laniger MH281753 2	Prionailurus planiceps NC028312 6	0,18721825
Melursus ursinus NC009970 2	Leopardus tigrinus NC028317 1	0,18722035
Gulo gulo NC009685 3	Cynictis penicillata T375 1	0,1872205
Urva javanica T413 1	Gulo gulo NC009685 3	0,18722068
Leopardus guigna NC028321 1	Galictis vittata T412 1	0,18722077
Lycaon pictus NC028427 2	Helarctos malayanus NC009968 2	0,18722261
Ursus americanus JX196366 3	Lutra lutra LC050126 1	0,18722619
Urva semitorquata MH464789 1	Arctocephalus forsteri NC004023 28	0,18722621
Paradoxurus jerdoni MH464793 1	Gulo gulo NC009685 3	0,1872265
Mephitis mephitis NC020648 1	Melursus ursinus NC009970 2	0,18722762
Panthera pardus NC010641 5	Melogale moschata KP726273 1	0,18723455
Zalophus californianus NC008416 1	Panthera onca NC022842 1	0,187235

Prionodon pardicolor NC024569 2	Urva javanica/auropunctata NC006835 1	0,16197923
Mellivora capensis T370 1	Lutra lutra LC050126 1	0,16198936
Mellivora capensis T370 1	Lutra lutra NC011358 9	0,16198936
Potos flavus T414 1	Mustela frenata NC020640 1	0,16199714
Salanoia concolor D378 1	Prionodon linsang ERR2391707 1	0,16200498
Mirounga leonina NC008422 1	Arctodus simus NC011116 1	0,162008
Ichneumia albicauda T603 1	Cynogale bennetti KY117544 1	0,16200909
Lynx rufus NC014456 3	Cynogale bennetti KY117544 1	0,16204949
Parahyaena brunnea NC038159 15	Hemigalus derbyanus MH464791 1	0,16204961
Lutra lutra LC050126 1	Hydrurga leptonyx NC008425 1	0,16206732
Mustela frenata NC020640 1	Hydrurga leptonyx NC008425 1	0,16207294
Taxidea taxus NC020646 1	Pusa hispida NC 008433 1	0,16207913
Panthera tigris NC010642 35	Ichneumia albicauda T603 1	0,16208491
Neofelis nebulosa NC008450 3	Mungos mungo/gambianus SRR7704821 1	0,16210647
Cynogale bennetti KY117544 1	Atilax paludinosus T606 1	0,16212928
Lutra lutra LC050126 1	Lobodon carcinophaga NC008423 1	0,16213478
Lobodon carcinophaga NC008423 1	Arctodus simus NC011116 1	0,16215515
Panthera tigris amoyensis NC014770 2	Crocuta crocuta NC020670 3	0,16216969
Panthera onca NC022842 1	Crocuta crocuta NC020670 3	0,16217269
Panthera uncia NC010638 1	Urva javanica/auropunctata NC006835 1	0,1621761
Enhydra lutris NC009692 1	Bassariscus sumichrasti SRX1099089 1	0,16219983
Nandinia binotata NC024567 1	Cystophora cristata NC008427 1	0,16220038
Lutra sumatrana NC035810 1	Hydrurga leptonyx NC008425 1	0,16220348
Meles meles T303 3	Lobodon carcinophaga NC008423 1	0,16220471
Taxidea taxus NC020646 1	Monachus schauinslandi NC008421 1	0,16220872
Paradoxurus jerdoni MH464793 1	Panthera tigris NC010642 35	0,16221906
Martes flavigula NC012141 3	Bassariscus sumichrasti SRX1099089 1	0,16224001
Panthera pardus japonensis KJ866876 8	Cynogale bennetti KY117544 1	0,16224998
Halichoerus grypus NC001602 2	Arctodus simus NC011116 1	0,16226242
Lutra sumatrana NC035810 1	Lobodon carcinophaga NC008423 1	0,16227144
Mustela putorius NC020638 4	Bassariscus sumichrasti SRX1099089 1	0,16227192
Mustela itatsi NC034330 19	Mellivora capensis T370 1	0,16227557
Paradoxurus hermaphroditus NC039591 1	Panthera uncia NC010638 1	0,16228035
Proteles cristata T393 6	Hemigalus derbyanus MH464791 1	0,1623078
Potos flavus T414 1	Enhydra lutris NC009692 1	0,16232824
Galidia elegans D146 1	Diplogale hosei MH464790 1	0,16233514
Prionodon pardicolor NC024569 2	Ichneumia albicauda T603 1	0,16234732
Panthera tigris NC010642 35	Bdeogale nigripes GLC15 1	0,16235325
Urva semitorquata MH464789 1	Cynogale bennetti KY117544 1	0,16239931
Pusa hispida NC 008433 1	Mustela erminea T305 2	0,16241068
Taxidea taxus NC020646 1	Phoca largha NC008430 1	0,16242206
Proteles cristata T393 6	Genetta genetta T297 1	0,16242509
Potos flavus T414 1	Martes flavigula NC012141 3	0,16243912
Lutra lutra LC050126 1	Leptonychotes weddellii NC008424 1	0,16245918
Meles meles T303 3	Leptonychotes weddellii NC008424 1	0,16246407
Mungos mungo/gambianus SRR7704821 1	Cynogale bennetti KY117544 1	0,1624666
Meles leucurus NC039173 4	Hydrurga leptonyx NC008425 1	0,16247374
Panthera uncia NC010638 1	Bdeogale nigripes GLC15 1	0,16248074
Paradoxurus jerdoni MH464793 1	Panthera uncia KP202269 1	0,16248265
Phoca groenlandica NC008429 54	Arctodus simus NC011116 1	0,16248572
Hydrurga leptonyx NC008425 1	Arctotherium sp NC030174 1	0,16248879
Genetta genetta T297 1	Crocuta crocuta NC020670 3	0,16250154
Felis catus NC001700 2	Crocuta crocuta NC020670 3	0,16250236
Potos flavus T414 1	Lutra sumatrana NC035810 1	0,16253142
Urva javanica T413 1	Cynogale bennetti KY117544 1	0,16253179
Nandinia binotata NC024567 1	Erignathus barbatus NC008426 1	0,16253704
Martes americana NC020642 1	Bassariscus sumichrasti SRX1099089 1	0,16254199
Potos flavus T414 1	Neovison vison NC020641 3	0,16254295
Cystophora cristata NC008427 1	Arctodus simus NC011116 1	0,16254598
Suricata suricatta SSM10 1	Panthera uncia NC010638 1	0,16254832
Potos flavus T414 1	Martes melampus NC009678 1	0,16259699
Cryptoprocta ferox CFC13 1	Chrotogale owstoni T607 1	0,16260935
Hyaena hyaena NC020669 1	Chrotogale owstoni T607 1	0,16261248
Martes martes T302 3	Bassariscus sumichrasti SRX1099089 1	0,16261296
Prionodon pardicolor NC024569 2	Urva javanica T413 1	0,16261469
Parahyaena brunnea NC038159 15	Chrotogale owstoni T607 1	0,16262078
Phoca vitulina NC001325 1	Arctodus simus NC011116 1	0,16262472
Viverra zibetha T609 1	Proteles cristata T393 6	0,16262537
Prionodon linsang ERR2391707 1	Galerella sanguinea T378 1	0,1626717
Bassariscus sumichrasti SRX1099089 1	Arctonyx collaris NC020645 1	0,16267364
Meles meles T303 3	Bassariscus sumichrasti SRX1099089 1	0,16267549
Melogale moschata V0735A 1	Mellivora capensis T370 1	0,16272967
Phoca fasciata NC008428 1	Nandinia binotata NC024567 1	0,1627479
Diplogale hosei MH464790 1	Cryptoprocta ferox CFC13 1	0,16279256
Potos flavus T414 1	Mustela nigripes NC024942 1	0,16280419
Mustela frenata NC020640 1	Cystophora cristata NC008427 1	0,16280663
Panthera uncia NC010638 1	Ichneumia albicauda T603 1	0,16281892
Pusa hispida NC 008433 1	Arctotherium sp NC030174 1	0,16282414
Phoca largha NC008430 1	Arctodus simus NC011116 1	0,16282646
Paguma larvata PDDS11 2	Crocuta crocuta NC020670 3	0,16283335
Viverricula indica KX891751 1	Crocuta crocuta NC020670 3	0,16283996
Panthera onca NC022842 1	Cynogale bennetti KY117544 1	0,16286586
Taxidea taxus NC020646 1	Mirounga leonina NC008422 1	0,162873
Lutra lutra NC011358 9	Hydrurga leptonyx NC008425 1	0,16287532
Neovison vison NC020641 3	Hydrurga leptonyx NC008425 1	0,16288814
Leptonychotes weddellii NC008424 1	Enhydra lutris NC009692 1	0,16293165
Potos flavus T414 1	Mustela putorius NC020638 4	0,16293865
Pusa caspica NC008431 1	Mustela erminea T305 2	0,16294074
Mustela sibirica AP017394 11	Bassariscus sumichrasti SRX1099089 1	0,16294511
Potos flavus T414 1	Mustela nivalis T306 5	0,16294675
Panthera leo spelaea KX258452 2	Urva brachyura KY117547 1	0,16295241
Panthera tigris amoyensis NC014770 2	Urva brachyura KY117547 1	0,16295724
Mustela sibirica NC020637 6	Bassariscus sumichrasti SRX1099089 1	0,16301236
Nandinia binotata NC024567 1	Monachus schauinslandi NC008421 1	0,16301587
Paradoxurus jerdoni MH464793 1	Panthera uncia NC010638 1	0,16302128
Felis chaus NC028307 1	Crocuta crocuta NC020670 3	0,16304204
Paradoxurus jerdoni MH464793 1	Neofelis nebulosa NC008450 3	0,16305529
Potos flavus T414 1	Gulo gulo NC009685 3	0,16311198
Melogale moschata NC020644 1	Mellivora capensis T370 1	0,16314046
Mirounga angustirostris SRR10331586 1	Arctodus simus NC011116 1	0,16316584
Prionodon pardicolor NC024569 2	Helogale parvula SRR7637809 1	0,16322006
Crocuta crocuta NC020670 3	Catopuma temminckii NC027115 41	0,1632404
Proteles cristata T393 6	Panthera uncia KP202269 1	0,1632425

Helarctos malayanus NC009968 2	Canis lupus familiaris NC002008 1231	0,18723607
Ursus arctos AP012576 6	Acinonyx jubatus NC005212 3	0,18723769
Galictis vittata T412 1	Arctocephalus forsteri NC004023 28	0,18723911
Mellivora capensis T370 1	Bassariscus sumichrasti SRX1099089 1	0,18724577
Vulpes lagopus NC026529 3	Martes americana NC020642 1	0,18724708
Mustela itatsi NC034330 19	Bdeogale nigripes GLC15 1	0,18724789
Otaria byronia OTAB 1	Lontra canadensis SRR10409165 1	0,18724865
Canis anthus NC027956 2	Bdeogale nigripes GLC15 1	0,18724902
Tremarctos ornatus NC009969 2	Eupleres goudotii D128 1	0,18725146
Nyctereutes procyonoides NC013700 3	Mustela putorius NC020638 4	0,18725196
Vulpes zerda KJ603240 1	Mustela nigripes NC024942 1	0,18725273
Nyctereutes procyonoides NC013700 3	Bassariscus sumichrasti SRX1099089 1	0,18725443
Lutrogale perspicillata NC035811 1	Leopardus geoffroyi NC028320 1	0,18725693
Suricata suricatta SSM10 1	Mustela altaica NC021751 1	0,18725695
Otocyon megalotis SAF1 2	Melogale moschata KP726273 1	0,18725814
Martes americana NC020642 1	Lycaon pictus NC028427 2	0,18725879
Ursus maritimus GU573488 Svalbard	Crossarchus platycephalus C7R66 1	0,18725984
Panthera pardus japonensis KJ866876	Mustela sibirica NC020637 6	0,1872599
Vulpes zerda KJ603240 1	Taxidea taxus NC020646 1	0,18726029
Vulpes zerda KJ603240 1	Felis catus NC001700 2	0,1872613
Vulpes zerda KJ603240 1	Felis margarita NC028308 1	0,18726186
Leopardus jacobita NC028322 1	Arctocephalus gazella BK010918 1	0,18726343
Tremarctos ornatus NC009969 2	Felis nigripes NC028309 1	0,18726431
Callorhinus ursinus NC008415 1	Arctictis binturong T605 2	0,18726434
Phocarctos hookeri NC008418 1	Leptailurus serval NC028316 1	0,18726517
Urocyon cinereoargenteus NC026723 2	Catopuma badia NC028300 1	0,1872655
Lycaon pictus NC028427 2	Conepatus chinga NC042596 1	0,1872658
Melogale moschata NC020644 1	Hyaena hyaena NC020669 1	0,18726625
Panthera onca KP202264 2	Mustela altaica NC021751 1	0,18726768
Mephitis mephitis NC020648 1	Lycalopex sechurae KT448284 1	0,18726796
Panthera tigris NC010642 35	Martes melampus NC009678 1	0,18726872
Panthera uncia KP202269 1	Canis mesomelas KT448280 1	0,18726899
Prionailurus viverrinus NC028305 1	Otocyon megalotis SAF1 2	0,18727052
Melursus ursinus NC009970 2	Lutra lutra LC050126 1	0,18727072
Vulpes vulpes NC008434 5	Panthera onca NC022842 1	0,18727076
Melogale moschata KP726273 1	Canis aureus KT448274 1	0,18727113
Panthera leo spelaea KX258452 2	Lutra sumatrana NC035810 1	0,18727125
Viverra zibetha T609 1	Speothos venaticus C48 2	0,18727149
Ursus maritimus GU573488 Svalbard	Leopardus pardalis T262 1	0,18727259
Otaria byronia OTAB 1	Caracal caracal NC028306 1	0,18727273
Ursus maritimus NC003428 31	Leopardus wiedii NC028318 1	0,18727274
Canis aureus KT448274 1	Bassariscus sumichrasti SRX1099089 1	0,18727364
Panthera tigris NC010642 35	Arctocephalus pusillus NC008417 1	0,18727379
Ursus arctos GU573486 5	Paguma larvata PDDS11 2	0,18727429
Speothos venaticus C48 2	Prionailurus planiceps NC028312 6	0,18727471
Panthera onca NC022842 1	Arctocephalus pusillus NC008417 1	0,18727674
Urva javanica/auropunctata NC006835	Canis mesomelas KT448280 1	0,18727748
Ursus spelaeus NC011112 8	Procyon lotor AB462049 4	0,18727876
Zalophus californianus NC008416 1	Helogale parvula SRR7637809 1	0,18727957
Panthera tigris NC010642 35	Martes martes T302 3	0,18727963
Panthera pardus NC010641 5	Martes flavigula NC012141 3	0,18728037
Potos flavus T414 1	Canis lupus familiaris NC002008 1231	0,18728113
Ursus arctos AP012576 6	Smilodon populator MF871700 1	0,18728229
Ursus maritimus NC003428 31	Ictonyx striatus T299 1	0,18728298
Vulpes lagopus NC026529 3	Ommatophoca rossii AY377287etc 1	0,18728325
Canis lupus familiaris NC002008 1231	Bdeogale nigripes GLC15 1	0,18728341
Ursus thibetanus mupinensis NC00875	Galictis vittata T412 1	0,18728463
Panthera leo NERO 19	Ailurus fulgens NC011124 1	0,18728526
Nasua nasua NC020647 1	Monachus monachus NC044972 5	0,18728647
Ursus arctos isabellinus 18B5 2	Canis aureus KT448274 1	0,18728647
Panthera leo spelaea KX258452 2	Ictonyx striatus T299 1	0,18728934
Ursus arctos GU573486 5	Arctictis binturong T605 2	0,18728946
Ursus arctos AP012576 6	Martes foina NC020643 1	0,18729062
Panthera leo NERO 19	Lutra sumatrana NC035810 1	0,18729068
Viverricula indica KX891745 1	Arctocephalus forsteri NC004023 28	0,18729183
Ursus thibetanus thibetanus NC01111	Galictis vittata T412 1	0,1872972
Lycalopex sechurae KT448284 1	Lutra lutra LC050126 1	0,18729736
Melursus ursinus NC009970 2	Arctictis binturong T605 2	0,18729867
Canis lupus chanco NC010340 4	Ailurus fulgens styani NC009691 1	0,18730004
Lycalopex sechurae KT448284 1	Cynictis penicillata T375 1	0,18730301
Ursus americanus JX196366 3	Puma concolor NC016470 22	0,18730488
Ursus americanus JX196366 3	Prionailurus bengalensis NC028301 12	0,18730582
Paradoxurus hermaphroditus NC03959	Tapirus terrestris T358	0,18730687
Melogale moschata V0735A 1	Urva javanica T413 1	0,18731289
Mustela eversmannii NC028013 1	Atilax paludinosus T606 1	0,18731353
Mustela sibirica NC020637 6	Cynictis penicillata T375 1	0,18731454
Eupleres goudotii D128 1	Arctocephalus townsendi NC008420 1	0,18731497
Vulpes corsac NC023958 1	Melogale moschata KP726273 1	0,18731524
Martes americana NC020642 1	Galidia elegans D146 1	0,18731526
Nyctereutes procyonoides NC013700 3	Martes zibellina NC011579 39	0,18731569
Neophoca cinerea NC008419 1	Bdeogale nigripes GLC15 1	0,18731707
Panthera leo NERO 19	Canis aureus KT448274 1	0,18731773
Mungotictis decemlineata NC027828 1	Bassariscus sumichrasti SRX1099089 1	0,1873184
Mustela sibirica NC020637 6	Mungotictis decemlineata NC027828 1	0,18731868
Vulpes lagopus NC026529 3	Mustela kathiah NC023210 1	0,18731907
Viverra tangalunga MH464792 1	Mungotictis decemlineata NC027828 1	0,18731967
Lynx rufus NC014456 3	Tapirus terrestris T358	0,18732096
Galidia elegans D146 1	Arctocephalus pusillus NC008417 1	0,18732122
Paguma larvata PDDS11 2	Arctocephalus gazella BK010918 1	0,18732132
Otaria byronia OTAB 1	Bdeogale nigripes GLC15 1	0,18732214
Panthera pardus japonensis KJ866876	Meles leucurus NC039173 4	0,18732235
Mustela nivalis T306 5	Helogale parvula SRR7637809 1	0,18732393
Spilogale putorius NC010497 1	Diplogale hosei MH464790 1	0,18732536
Enhydra lutris NC009692 1	Chrysocyon brachyurus NC024172 1	0,18732575
Phocarctos hookeri NC008418 1	Genetta servalina NC024568 2	0,18732695
Melogale moschata KP726273 1	Lycaon pictus NC028427 2	0,18732743
Lutrogale perspicillata NC035811 1	Cryptoprocta ferox CFC13 1	0,18732791
Puma concolor NC016470 22	Arctocephalus australis MG023139 1	0,18732795
Profelis aurata NC028299 1	Mustela itatsi NC034330 19	0,18732814
Otocyon megalotis SAF1 2	Mustela erminea T305 2	0,18732867
Panthera pardus japonensis KJ866876	Canis latrans NC008093 7	0,18732971
Salanoia concolor D378 1	Lycaon pictus NC028427 2	0,18733019
Neophoca cinerea NC008419 1	Genetta genetta T297 1	0,18733057

Profelis aurata NC028299 1	Crocuta crocuta NC020670 3	0,16324496
Gulo gulo NC009685 3	Bassariscus sumichrasti SRX1099089 1	0,16325185
Potos flavus T414 1	Lutra lutra LC050126 1	0,16326971
Mustela frenata NC020640 1	Leptonychotes weddellii NC008424 1	0,16327555
Prionodon linsang ERR2391707 1	Galidictis fasciata DM333 1	0,16328451
Phoca largha NC008430 1	Mustela erminea T305 2	0,16328943
Lutra lutra LC050126 1	Bassariscus sumichrasti SRX1099089 1	0,16334312
Nandinia binotata NC024567 1	Mirounga leonina NC008422 1	0,16334501
Pusa sibirica NC008432 2	Arctotherium sp NC030174 1	0,16335051
Mustela kathiah NC023210 1	Mellivora capensis T370 1	0,16335227
Prionodon pardicolor NC024569 2	Galidia elegans D146 1	0,16335899
Prionodon linsang ERR2391707 1	Eupleres goudotii D128 1	0,16340952
Mustela erminea T305 2	Erignathus barbatus NC008426 1	0,16341078
Cynogale bennetti KY117544 1	Crossarchus platycephalus C7R66 1	0,16341195
Lobodon carcinophaga NC008423 1	Enhydra lutris NC009692 1	0,16341496
Mustela nigripes NC024942 1	Bassariscus sumichrasti SRX1099089 1	0,16341607
Meles leucurus NC039173 4	Bassariscus sumichrasti SRX1099089 1	0,16341631
Tremarctos ornatus NC009969 2	Hydrurga leptonyx NC008425 1	0,1634194
Taxidea taxus NC020646 1	Phoca groenlandica NC008429 54	0,16342448
Viverricula indica KX891751 1	Proteles cristata T393 6	0,16343342
Leptonychotes weddellii NC008424 1	Arctonyx collaris NC020645 1	0,16347274
Prionodon linsang ERR2391707 1	Crossarchus platycephalus C7R66 1	0,16347967
Hydrurga leptonyx NC008425 1	Arctonyx collaris NC020645 1	0,16348115
Taxidea taxus NC020646 1	Pusa caspica NC008431 1	0,1634866
Taxidea taxus NC020646 1	Procyon lotor AB462046 3	0,16349141
Phoca fasciata NC008428 1	Arctotherium sp NC030174 1	0,16349167
Martes melampus NC009678 1	Leptonychotes weddellii NC008424 1	0,16353612
Lutra lutra NC011358 9	Lobodon carcinophaga NC008423 1	0,16354857
Phoca fasciata NC008428 1	Lutra sumatrana NC035810 1	0,16354911
Potos flavus T414 1	Martes martes T302 3	0,16355054
Taxidea taxus NC020646 1	Procyon lotor AB462049 4	0,16355848
Mellivora capensis T370 1	Gulo gulo NC009685 3	0,16358627
Leptonychotes weddellii NC008424 1	Homotherium latidens MF871702 3	0,16358867
Meles leucurus NC039173 4	Leptonychotes weddellii NC008424 1	0,1636083
Mustela erminea T305 2	Mirounga leonina NC008422 1	0,16361268
Panthera leo NERO 19	Crocuta crocuta NC020670 3	0,1636152
Martes zibellina NC011579 39	Bassariscus sumichrasti SRX1099089 1	0,16361898
Prionodon linsang ERR2391707 1	Mungotictis decemlineata NC027828 1	0,16362087
Mustela erminea T305 2	Mirounga angustirostris SRR10331586 1	0,16362475
Panthera tigris NC010642 35	Cynictis penicillata T375 1	0,16363084
Hydrurga leptonyx NC008425 1	Hemigalus derbyanus MH464791 1	0,16365996
Lutra lutra NC011358 9	Leptonychotes weddellii NC008424 1	0,16367052
Potos flavus T414 1	Arctonyx collaris NC020645 1	0,16367693
Martes melampus NC009678 1	Hydrurga leptonyx NC008425 1	0,16368429
Hyaena hyaena NC020669 1	Arctictis binturong T605 2	0,16371438
Potos flavus T414 1	Meles meles T303 3	0,16374472
Pusa sibirica NC008432 2	Mustela erminea T305 2	0,16374901
Lobodon carcinophaga NC008423 1	Arctonyx collaris NC020645 1	0,16375102
Mustela nigripes NC024942 1	Mellivora capensis T370 1	0,1637561
Hydrurga leptonyx NC008425 1	Crossarchus platycephalus C7R66 1	0,16375811
Panthera tigris NC010642 35	Urva brachyura KY117547 1	0,16376531
Viverra zibetha T609 1	Crocuta crocuta NC020670 3	0,16378178
Panthera leo spelaea KX258452 2	Crocuta crocuta NC020670 3	0,16378339
Paradoxurus hermaphroditus NC039591 1	Cryptoprocta ferox CFC13 1	0,16382734
Martes foina NC020643 1	Bassaricyon neblina SRX1097850 1	0,16382873
Mellivora capensis T370 1	Lontra canadensis SRR10409165 1	0,16387375
Procyon lotor AB462046 3	Meles leucurus NC039173 4	0,16388382
Pusa hispida NC 008433 1	Lutra sumatrana NC035810 1	0,16388978
Potos flavus T414 1	Martes foina NC020643 1	0,16389446
Panthera pardus japonensis KJ866876 8	Crocuta crocuta NC020670 3	0,16391444
Potos flavus T414 1	Mustela sibirica NC020637 6	0,16394833
Potos flavus T414 1	Martes americana NC020642 1	0,16394895
Potos flavus T414 1	Erignathus barbatus NC008426 1	0,1639528
Cystophora cristata NC008427 1	Arctotherium sp NC030174 1	0,1639564
Erignathus barbatus NC008426 1	Arctotherium sp NC030174 1	0,16395663
Paradoxurus hermaphroditus NLNC 1	Hyaena hyaena NC020669 1	0,16396053
Panthera onca KP202264 2	Cynogale bennetti KY117544 1	0,16400127
Neofelis nebulosa NC008450 3	Crocuta crocuta NC020670 3	0,16400954
Mustela sibirica AP017394 11	Lobodon carcinophaga NC008423 1	0,16402507
Paradoxurus jerdoni MH464793 1	Cryptoprocta ferox CFC13 1	0,16402936
Taxidea taxus NC020646 1	Phoca vitulina NC001325 1	0,16403782
Lutra sumatrana NC035810 1	Leptonychotes weddellii NC008424 1	0,164076
Potos flavus T414 1	Meles leucurus NC039173 4	0,16408078
Mustela itatsi NC034330 19	Bassariscus sumichrasti SRX1099089 1	0,16408946
Pusa hispida NC 008433 1	Lutra lutra LC050126 1	0,16409068
Neovison vison NC020641 3	Leptonychotes weddellii NC008424 1	0,1640912
Phoca vitulina NC001325 1	Mustela erminea T305 2	0,16409741
Phoca largha NC008430 1	Arctotherium sp NC030174 1	0,16410254
Panthera leo NERO 19	Cynogale bennetti KY117544 1	0,16410547
Taxidea taxus NC020646 1	Monachus monachus NC044972 5	0,16412252
Tremarctos ornatus NC009969 2	Leptonychotes weddellii NC008424 1	0,16414636
Viverra tangalunga MH464792 1	Leptonychotes weddellii NC008424 1	0,164149
Pusa caspica NC008431 1	Arctotherium sp NC030174 1	0,16415845
Taxidea taxus NC020646 1	Mirounga angustirostris SRR10331586 1	0,16416419
Xenogale naso C07XAR110 1	Cynogale bennetti KY117544 1	0,1642168
Lobodon carcinophaga NC008423 1	Arctotherium sp NC030174 1	0,16423906
Martes flavigula NC012141 3	Leptonychotes weddellii NC008424 1	0,1642516
Mustela erminea T305 2	Halichoerus grypus NC001602 2	0,16426598
Taxidea taxus NC020646 1	Halichoerus grypus NC001602 2	0,16427383
Panthera pardus NC010641 5	Crocuta crocuta NC020670 3	0,16428968
Ursus thibetanus thibetanus NC011118 4	Hydrurga leptonyx NC008425 1	0,16432558
Mirounga leonina NC008422 1	Meles meles T303 3	0,16435049
Suricata suricatta SSM10 1	Cynogale bennetti KY117544 1	0,16435182
Lutra lutra NC011358 9	Bassariscus sumichrasti SRX1099089 1	0,16435239
Mustela eversmannii NC028013 1	Bassariscus sumichrasti SRX1099089 1	0,16435824
Phoca largha NC008430 1	Lutra sumatrana NC035810 1	0,1643624
Martes martes T302 3	Hydrurga leptonyx NC008425 1	0,16436751
Ursus thibetanus thibetanus NC011118 4	Phoca groenlandica NC008429 54	0,16438456
Proteles cristata T393 6	Neofelis nebulosa NC008450 3	0,1644146
Pusa caspica NC008431 1	Nandinia binotata NC024567 1	0,1644223
Potos flavus T414 1	Martes pennanti NC020664 16	0,16442305
Prionodon linsang ERR2391707 1	Galidia elegans D146 1	0,16442849
Panthera uncia KP202269 1	Cynogale bennetti KY117544 1	0,16447318

Puma yagouaroundi NC028311 1	Lontra canadensis SRR10409165 1	0,18733059
Lontra canadensis SRR10409165 1	Felis chaus NC028307 1	0,18733123
Hemigalus derbyanus MH464791 1	Aonyx cinerea NC035814 2	0,18733163
Neophoca cinerea NC008419 1	Felis margarita NC028308 1	0,18733183
Ursus spelaeus EU327344 13	Conepatus chinga NC042596 1	0,1873319
Ursus maritimus NC003428 31	Hyaena hyaena NC020669 1	0,18733206
Arctocephalus townsendi NC008420 1	Ailuropoda melanoleuca NC009492 5	0,18733211
Arctocephalus australis MG023139 1	Ailuropoda melanoleuca NC009492 5	0,18733297
Panthera onca KP202264 2	Bassariscus sumichrasti SRX1099089 1	0,18733361
Lutrogale perspicillata NC035811 1	Felis margarita NC028308 1	0,18733422
Spilogale putorius NC010497 1	Smilodon populator MF871700 1	0,18733433
Procyon lotor AB462046 3	Pardofelis marmorata NLN3 2	0,18733451
Felis chaus NC028307 1	Aonyx cinerea NC035814 2	0,18733551
Felis catus NC001700 2	Arctotherium sp NC030174 1	0,18733566
Ursus maritimus GU573488 Svalbard	Paradoxurus jerdoni MH464793 1	0,1873358
Mustela nivalis T306 5	Cryptoprocta ferox CFC13 1	0,18733586
Panthera onca NC022842 1	Martes melampus NC009678 1	0,18733747
Mungos mungo MMC7 1	Galictis vittata T412 1	0,1873382
Potos flavus T414 1	Odobenus rosmarus NC004029 29	0,18733881
Panthera pardus japonensis KJ866876	Neovison vison NC020641 3	0,18733901
Parahyaena brunnea NC038159 15	Mustela nivalis T306 5	0,18733941
Ursus arctos GU573491 207	Leopardus pardalis NC028315 1	0,18733963
Ursus thibetanus thibetanus NC01111	Prionodon linsang ERR2391707 1	0,18734014
Ursus spelaeus NC011112 8	Mungos mungo MMC7 1	0,18734019
Ursus thibetanus mupinensis NC00875	Pardofelis marmorata NLN3 2	0,18734086
Mustela altaica NC021751 1	Chrysocyon brachyurus NC024172 1	0,18734093
Ursus arctos GU573491 207	Profelis aurata NC028299 1	0,18734127
Ursus arctos EU497665 29	Prionailurus rubiginosus NC028304 2	0,18734132
Panthera onca KP202264 2	Martes foina NC020643 1	0,18734144
Ursus maritimus GU573488 Svalbard	Prionailurus bengalensis NC028301 12	0,18734154
Ursus arctos pruinosus MG066703 3	Profelis aurata NC028299 1	0,18734165
Ursus maritimus GU573488 Svalbard	Caracal caracal NC028306 1	0,18734165
Ursus thibetanus formosanus NC0093	Canis anthus NC027956 2	0,18734229
Ursus arctos EU497665 29	Catopuma badia NC028300 1	0,18734474
Canis aureus KT448274 1	Ailurus fulgens NC011124 1	0,18734601
Ursus spelaeus NC011112 8	Hyaena hyaena NC020669 1	0,18734603
Tremarctos ornatus NC009969 2	Arctictis binturong T605 2	0,18734717
Genetta servalina NC024568 2	Galictis vittata T412 1	0,18734851
Ursus arctos AP012576 6	Paradoxurus jerdoni MH464793 1	0,18734895
Viverricula indica NC025296 2	Ursus arctos AP012576 6	0,18735136
Ursus thibetanus formosanus NC0093	Prionailurus viverrinus NC028305 1	0,18735152
Panthera tigris NC010642 35	Ictonyx striatus T299 1	0,18735258
Puma yagouaroundi NC028311 1	Galictis vittata T412 1	0,1873527
Procyon lotor AB462046 3	Urva javanica/auropunctata NC006835	0,18735552
Vulpes vulpes NC008434 5	Panthera leo NERO 19	0,18735823
Helogale parvula SRR7637809 1	Eumetopias jubatus NC004030 10	0,18736005
Paguma larvata PDDS11 2	Gulo gulo NC009685 3	0,1873612
Meles leucurus NC039173 4	Cuon alpinus NC013445 3	0,18736176
Gulo gulo NC009685 3	Galerella sanguinea T378 1	0,18736662
Catopuma badia NC028300 1	Arctocephalus forsteri NC004023 28	0,18736914
Panthera pardus NC010641 5	Melogale moschata V0735A 1	0,18736933
Otocyon megalotis SAF1 2	Ommatophoca rossii AY377287etc 1	0,18737255
Pusa sibirica NC008432 2	Tapirus terrestris T358	0,18737577
Lynx canadensis NC028313 1	Cuon alpinus NC013445 3	0,18737714
Panthera leo NERO 19	Ictonyx striatus T299 1	0,18737775
Suricata suricatta SSM10 1	Callorhinus ursinus NC008415 1	0,187379
Vulpes vulpes NC008434 5	Martes melampus NC009678 1	0,18738094
Mustela nigripes NC024942 1	Helogale parvula SRR7637809 1	0,18738104
Mungos mungo MMC7 1	Ailurus fulgens NC011124 1	0,18738152
Mustela sibirica NC020637 6	Bdeogale nigripes GLC15 1	0,18738174
Atilax paludinosus T606 1	Arctocephalus australis MG023139 1	0,18738216
Eupleres goudotii D128 1	Arctocephalus australis MG023139 1	0,1873828
Tremarctos ornatus NC009969 2	Mungotictis decemlineata NC027828 1	0,18738306
Mungotictis decemlineata NC027828 1	Callorhinus ursinus NC008415 1	0,18738431
Procyon lotor AB462046 3	Mungos mungo/gambianus SRR77048	0,18738577
Panthera pardus NC010641 5	Ictonyx striatus T299 1	0,18738581
Puma concolor NC016470 22	Conepatus chinga NC042596 1	0,18738648
Vulpes ferrilata NC027935 1	Mustela sibirica NC020637 6	0,18738659
Ichneumia albicauda T603 1	Enhydra lutris NC009692 1	0,18738672
Vulpes ferrilata NC027935 1	Genetta abyssinica MG489822 1	0,18738745
Otocolobus manul NC028323 1	Conepatus chinga NC042596 1	0,18738969
Procyon lotor AB462046 3	Lycalopex sechurae KT448284 1	0,18738975
Viverricula indica KX891745 1	Urocyon cinereoargenteus NC026723 2	0,18739486
Profelis aurata NC028299 1	Mustela putorius NC020638 4	0,18739523
Vulpes ferrilata NC027935 1	Pardofelis marmorata NLN3 2	0,18739557
Proteles cristata T393 6	Meles leucurus NC039173 4	0,18739623
Felis margarita NC028308 1	Arctocephalus gazella BK010918 1	0,1873965
Parahyaena brunnea NC038159 15	Meles leucurus NC039173 4	0,18739665
Vulpes zerda KJ603240 1	Puma yagouaroundi NC028311 1	0,18739715
Prionailurus viverrinus NC028305 1	Arctocephalus forsteri KT693377 17	0,1873972
Proteles cristata T393 6	Martes melampus NC009678 1	0,18739741
Salanoia concolor D378 1	Mustela altaica NC021751 1	0,18739749
Ursus thibetanus mupinensis NC00875	Prionodon linsang ERR2391707 1	0,18739757
Salanoia concolor D378 1	Poecilogale albinucha T602 1	0,18739907
Odobenus rosmarus NC004029 29	Arctonyx collaris NC020645 1	0,18739919
Speothos venaticus C48 2	Mustela itatsi NC034330 19	0,18740013
Chrysocyon brachyurus NC024172 1	Atilax paludinosus T606 1	0,18740016
Galerella sanguinea T378 1	Arctotherium sp NC030174 1	0,18740188
Odobenus rosmarus NC004029 29	Lutra lutra LC050126 1	0,18740189
Martes zibellina NC011579 39	Ailuropoda melanoleuca NC009492 5	0,18740191
Martes foina NC020643 1	Canis mesomelas KT448280 1	0,18740192
Profelis aurata NC028299 1	Poecilogale albinucha T602 1	0,18740265
Ursus maritimus NC003428 31	Paradoxurus jerdoni MH464793 1	0,1874031
Parahyaena brunnea NC038159 15	Martes martes T302 3	0,18740346
Ursus maritimus NC003428 31	Genetta genetta T297 1	0,18740419
Ursus arctos GU573491 207	Paguma larvata PDDS11 2	0,1874042
Martes pennanti NC020664 16	Lycalopex sechurae KT448284 1	0,18740511
Panthera leo NERO 19	Gulo gulo NC009685 3	0,18740617
Urva brachyura KY117547 1	Canis aureus KT448274 1	0,18740728
Ursus arctos GU573491 207	Puma yagouaroundi NC028311 1	0,18740872
Ursus maritimus NC003428 31	Leptailurus serval NC028316 1	0,18740915
Ursus arctos GU573491 207	Catopuma badia NC028300 1	0,18741167
Ursus maritimus GU573488 Svalbard	Prionailurus bengalensis CKM45 20	0,18741196

Lutra lutra LC050126 1	Cystophora cristata NC008427 1	0,16448307
Mustela sibirica AP017394 11	Leptonychotes weddellii NC008424 1	0,16448505
Pusa sibirica NC008432 2	Nandinia binotata NC024567 1	0,16448963
Phoca groenlandica NC008429 54	Arctotherium sp NC030174 1	0,16450202
Phoca vitulina NC001325 1	Arctotherium sp NC030174 1	0,1645067
Mirounga leonina NC008422 1	Meles leucurus NC039173 4	0,16455218
Taxidea taxus NC020646 1	Nandinia binotata NC024567 1	0,1645609
Phoca groenlandica NC008429 54	Mustela erminea T305 2	0,16456583
Nandinia binotata NC024567 1	Mirounga angustirostris SRR10331586 1	0,16456906
Puma yagouaroundi NC028311 1	Hydrurga leptonyx NC008425 1	0,16457599
Leopardus pardalis T262 1	Hydrurga leptonyx NC008425 1	0,16457821
Suricata suricatta SSM10 1	Neofelis nebulosa NC008450 3	0,16459966
Mustela erminea T305 2	Cystophora cristata NC008427 1	0,16462363
Melogale moschata NC020644 1	Bassariscus sumichrasti SRX1099089 1	0,16463256
Taxidea taxus NC020646 1	Mellivora capensis T370 1	0,16464491
Potos flavus T414 1	Mustela itatsi NC034330 19	0,1646896
Potos flavus T414 1	Martes zibellina NC011579 39	0,16468979
Phoca fasciata NC008428 1	Lutra lutra LC050126 1	0,16469155
Neofelis nebulosa NC008450 3	Urva javanica/auropunctata NC006835 1	0,16469885
Mustela frenata NC020640 1	Mirounga angustirostris SRR10331586 1	0,16470154
Prionailurus bengalensis NC028301 12	Leptonychotes weddellii NC008424 1	0,16470167
Prionodon pardicolor NC024569 2	Cryptoprocta ferox CFC13 1	0,16471747
Hyaena hyaena NC020669 1	Diplogale hosei MH464790 1	0,16472508
Ursus thibetanus laniger MH281753 2	Hydrurga leptonyx NC008425 1	0,16472709
Melogale moschata KP726273 1	Mellivora capensis T370 1	0,16474864
Potos flavus T414 1	Lutra lutra NC011358 9	0,16475028
Erignathus barbatus NC008426 1	Arctonyx collaris NC020645 1	0,16475375
Homotherium latidens MF871702 3	Canis adustus KT448271 1	0,16475984
Meles leucurus NC039173 4	Lobodon carcinophaga NC008423 1	0,16476332
Phoca vitulina NC001325 1	Lutra sumatrana NC035810 1	0,16476628
Tremarctos ornatus NC009969 2	Lobodon carcinophaga NC008423 1	0,16476656
Phoca groenlandica NC008429 54	Nandinia binotata NC024567 1	0,16476795
Prionailurus bengalensis NC028301 12	Hydrurga leptonyx NC008425 1	0,16477814
Lobodon carcinophaga NC008423 1	Aonyx cinerea NC035814 2	0,16477862
Leopardus pardalis NC028315 1	Hydrurga leptonyx NC008425 1	0,16478004
Ursus spelaeus NC011112 8	Hydrurga leptonyx NC008425 1	0,16479692
Neofelis nebulosa NC008450 3	Urva javanica T413 1	0,16480035
Lutra sumatrana NC035810 1	Cystophora cristata NC008427 1	0,16482061
Phoca largha NC008430 1	Lutra lutra LC050126 1	0,16483316
Mustela itatsi NC034330 19	Lobodon carcinophaga NC008423 1	0,16483325
Taxidea taxus NC020646 1	Pusa sibirica NC008432 2	0,16483332
Paradoxurus jerdoni MH464793 1	Hyaena hyaena NC020669 1	0,16484281
Neofelis nebulosa NC008450 3	Cynictis penicillata T375 1	0,16486755
Martes flavigula NC012141 3	Lobodon carcinophaga NC008423 1	0,16486821
Mustela itatsi NC034330 19	Leptonychotes weddellii NC008424 1	0,16488978
Pusa hispida NC 008433 1	Nandinia binotata NC024567 1	0,16490038
Pusa hispida NC 008433 1	Mustela frenata NC020640 1	0,16490251
Nandinia binotata NC024567 1	Monachus monachus NC044972 5	0,16492492
Tremarctos ornatus NC009969 2	Erignathus barbatus NC008426 1	0,16495466
Martes martes T302 3	Leptonychotes weddellii NC008424 1	0,16495962
Leptonychotes weddellii NC008424 1	Crossarchus platycephalus C7R66 1	0,16496095
Martes martes T302 3	Erignathus barbatus NC008426 1	0,1649611
Potos flavus T414 1	Bassaricyon neblina SRX1097850 1	0,16496253
Mustela nivalis T306 5	Bassariscus sumichrasti SRX1099089 1	0,16497366
Lobodon carcinophaga NC008423 1	Homotherium latidens MF871702 3	0,1649828
Proteles cristata T393 6	Panthera uncia NC010638 1	0,16499342
Ursus spelaeus NC011112 8	Lobodon carcinophaga NC008423 1	0,16499856
Hydrurga leptonyx NC008425 1	Genetta abyssinica MG489822 1	0,16500244
Mustela frenata NC020640 1	Mirounga leonina NC008422 1	0,16502495
Monachus schauinslandi NC008421 1	Arctonyx collaris NC020645 1	0,16503069
Mustela sibirica AP017394 11	Hydrurga leptonyx NC008425 1	0,16503476
Viverra tangalunga MH464792 1	Lobodon carcinophaga NC008423 1	0,16503479
Ursus thibetanus mupinensis NC008753 2	Phoca groenlandica NC008429 54	0,16504684
Ursus thibetanus laniger MH281753 2	Phoca groenlandica NC008429 54	0,16505592
Panthera uncia KP202269 1	Crocuta crocuta NC020670 3	0,16505899
Martes flavigula NC012141 3	Hydrurga leptonyx NC008425 1	0,16507086
Mellivora capensis T370 1	Enhydra lutris NC009692 1	0,16508514
Meles meles T303 3	Erignathus barbatus NC008426 1	0,16509068
Mustela sibirica NC020637 6	Lobodon carcinophaga NC008423 1	0,16510229
Panthera leo spelaea KX258452 2	Cynogale bennetti KY117544 1	0,16515379
Phoca fasciata NC008428 1	Lutra lutra NC011358 9	0,16516221
Hydrurga leptonyx NC008425 1	Fossa fossana D350 1	0,16517283
Leptonychotes weddellii NC008424 1	Leopardus pardalis T262 1	0,16517442
Prionailurus rubiginosus NC028304 2	Hydrurga leptonyx NC008425 1	0,16518258
Parahyaena brunnea NC038159 15	Paradoxurus hermaphroditus NLNC 1	0,1651829
Taxidea taxus NC020646 1	Ommatophoca rossii AY377287etc 1	0,16522273
Mustela putorius NC020638 4	Leptonychotes weddellii NC008424 1	0,16522597
Leptonychotes weddellii NC008424 1	Fossa fossana D350 1	0,16523027
Suricata suricatta SSM10 1	Prionodon linsang ERR2391707 1	0,16523027
Meles anakuma NC009677 1	Hydrurga leptonyx NC008425 1	0,16523181
Phoca fasciata NC008428 1	Mustela erminea T305 2	0,16523854
Paradoxurus hermaphroditus NC039591 1	Hyaena hyaena NC020669 1	0,16523877
Phoca vitulina NC001325 1	Mustela frenata NC020640 1	0,16524273
Phoca vitulina NC001325 1	Nandinia binotata NC024567 1	0,165244
Meles anakuma NC009677 1	Leptonychotes weddellii NC008424 1	0,16528928
Potos flavus T414 1	Mustela sibirica AP017394 11	0,1652948
Cystophora cristata NC008427 1	Bassariscus sumichrasti SRX1099089 1	0,16529997
Potos flavus T414 1	Mustela altaica NC021751 1	0,16530356
Mustela sibirica AP017394 11	Mellivora capensis T370 1	0,16530371
Mustela putorius NC020638 4	Lobodon carcinophaga NC008423 1	0,16530442
Melursus ursinus NC009970 2	Cystophora cristata NC008427 1	0,16531868
Martes foina NC020643 1	Lobodon carcinophaga NC008423 1	0,1653195
Prionailurus bengalensis CKM45 20	Hydrurga leptonyx NC008425 1	0,16532083
Mustela frenata NC020640 1	Erignathus barbatus NC008426 1	0,16536377
Mustela erminea T305 2	Ailurus fulgens styani NC009691 1	0,16536734
Mustela kathiah NC023210 1	Bassariscus sumichrasti SRX1099089 1	0,16536929
Phoca largha NC008430 1	Nandinia binotata NC024567 1	0,1653786
Viverricula indica KX891745 1	Hydrurga leptonyx NC008425 1	0,16538182
Viverra zibetha T609 1	Hydrurga leptonyx NC008425 1	0,16538329
Leopardus jacobita NC028322 1	Hydrurga leptonyx NC008425 1	0,16538629
Ursus spelaeus NC011112 8	Leptonychotes weddellii NC008424 1	0,16538885
Ursus spelaeus EU327344 13	Hydrurga leptonyx NC008425 1	0,16540293
Mirounga leonina NC008422 1	Homotherium latidens MF871702 3	0,16540316

Viverra tangalunga MH464792 1	Ailuropoda melanoleuca NC009492 5	0,1874139
Procyon lotor AB462046 3	Arctictis binturong T605 2	0,18741588
Ursus arctos isabellinus 18B5 2	Lycalopex sechurae KT448284 1	0,18741976
Poecilogale albinucha T602 1	Melursus ursinus NC009970 2	0,1874201
Ursus arctos EU497665 29	Arctocephalus forsteri NC004023 28	0,18742359
Prionodon linsang ERR2391707 1	Gulo gulo NC009685 3	0,18742442
Martes flavigula NC012141 3	Helogale parvula SRR7637809 1	0,18742676
Prionodon pardicolor NC024569 2	Canis lupus familiaris NC002008 1231	0,18743132
Ursus spelaeus NC011112 8	Mephitis mephitis NC020648 1	0,18743259
Panthera leo NERO 19	Aonyx cinerea NC035814 2	0,187433
Hemigalus derbyanus MH464791 1	Arctocephalus gazella BK010918 1	0,18743356
Cuon alpinus NC013445 3	Arctodus simus NC011116 1	0,18743896
Melursus ursinus NC009970 2	Ictonyx striatus T299 1	0,18743931
Tremarctos ornatus NC009969 2	Conepatus chinga NC042596 1	0,18744054
Leopardus jacobita NC028322 1	Arctocephalus forsteri NC004023 28	0,18744104
Genetta servalina NC024568 2	Cuon alpinus NC013445 3	0,1874412
Panthera pardus NC010641 5	Bassariscus sumichrasti SRX1099089 1	0,18744425
Leopardus jacobita NC028322 1	Canis mesomelas KT448280 1	0,18744515
Ursus americanus JX196366 3	Poecilogale albinucha T602 1	0,18744551
Urva semitorquata MH464789 1	Tapirus terrestris T358	0,18744753
Parahyaena brunnea NC038159 15	Eumetopias jubatus NC004030 10	0,18744861
Atilax paludinosus T606 1	Arctocephalus forsteri KT693377 17	0,1874494
Arctictis binturong T605 2	Tapirus terrestris T358	0,18745041
Nyctereutes procyonoides NC013700 3	Martes americana NC020642 1	0,18745047
Prionodon pardicolor NC024569 2	Lutra lutra NC011358 9	0,18745057
Mustela eversmannii NC028013 1	Galidictis fasciata DM333 1	0,18745242
Mungotictis decemlineata NC027828 1	Lutrogale perspicillata NC035811 1	0,18745334
Nyctereutes procyonoides NC013700 3	Mungotictis decemlineata NC027828 1	0,18745351
Nyctereutes procyonoides NC013700 3	Mustela sibirica AP017394 11	0,18745366
Otaria byronia OTAB 1	Atilax paludinosus T606 1	0,18745483
Procyon lotor AB462049 4	Nyctereutes procyonoides NC013700 3	0,18745637
Melogale moschata KP726273 1	Cryptoprocta ferox CFC13 1	0,18745756
Lutra lutra NC011358 9	Hyaena hyaena NC020669 1	0,18745825
Prionailurus viverrinus NC028305 1	Lutrogale perspicillata NC035811 1	0,18745869
Vulpes ferrilata NC027935 1	Mustela altaica NC021751 1	0,18746053
Urocyon cinereoargenteus NC026723 2	Martes pennanti NC020664 16	0,18746118
Panthera onca KP202264 2	Martes melampus NC009678 1	0,18746141
Vulpes zerda KJ603240 1	Martes pennanti NC020664 16	0,18746153
Vulpes lagopus NC026529 3	Panthera uncia KP202269 1	0,18746251
Vulpes corsac NC023958 1	Lynx canadensis NC028313 1	0,18746275
Parahyaena brunnea NC038159 15	Arctonyx collaris NC020645 1	0,18746315
Enhydra lutris NC009692 1	Crocuta crocuta NC020670 3	0,18746346
Genetta genetta T297 1	Canis anthus NC027956 2	0,18746361
Vulpes ferrilata NC027935 1	Leopardus jacobita NC028322 1	0,18746366
Prionodon linsang ERR2391707 1	Bassariscus sumichrasti SRX1099089 1	0,18746388
Pardofelis marmorata NLN3 2	Lontra canadensis SRR10409165 1	0,18746409
Paradoxurus hermaphroditus NLNC 1	Arctotherium sp NC030174 1	0,18746598
Canis anthus NC027956 2	Arctodus simus NC011116 1	0,18746704
Urocyon cinereoargenteus NC026723 2	Catopuma temminckii NC027115 41	0,18746741
Parahyaena brunnea NC038159 15	Lutrogale perspicillata NC035811 1	0,18746763
Neophoca cinerea NC008419 1	Leopardus jacobita NC028322 1	0,18746789
Hyaena hyaena NC020669 1	Canis aureus KT448274 1	0,18746857
Ursus arctos isabellinus 18B5 2	Genetta genetta T297 1	0,18747152
Panthera tigris amoyensis NC014770 2	Lutra sumatrana NC035810 1	0,18747218
Melogale moschata V0735A 1	Arctictis binturong T605 2	0,18747242
Panthera uncia KP202269 1	Otocyon megalotis SAF1 2	0,1874727
Ursus arctos EU497665 29	Lynx lynx NC027083 4	0,18747364
Ursus arctos isabellinus 18B5 2	Lynx pardinus NC028319 161	0,1874737
Odobenus rosmarus NC004029 29	Bassariscus sumichrasti SRX1099089 1	0,18747598
Ursus thibetanus formosanus NC00933	Canis latrans NC008093 7	0,18747716
Poecilogale albinucha T602 1	Canis aureus KT448274 1	0,18747765
Ursus arctos GU573491 207	Catopuma temminckii NC027115 41	0,1874782
Paradoxurus hermaphroditus NLNC 1	Ictonyx striatus T299 1	0,18747823
Melursus ursinus NC009970 2	Chrotogale owstoni T607 1	0,18747954
Ursus thibetanus laniger MH281753 2	Otocyon megalotis SAF1 2	0,18748251
Viverra tangalunga MH464792 1	Ursus arctos AP012576 6	0,18748325
Crossarchus platycephalus C7R66 1	Canis mesomelas KT448280 1	0,18748346
Suricata suricatta SSM10 1	Canis lupus familiaris NC002008 1231	0,18748449
Enhydra lutris NC009692 1	Canis lupus chanco NC010340 4	0,18748567
Paradoxurus jerdoni MH464793 1	Melursus ursinus NC009970 2	0,18748568
Ursus thibetanus mupinensis NC00875	Lycalopex sechurae KT448284 1	0,18748698
Halichoerus grypus NC001602 2	Tapirus terrestris T358	0,18748805
Ursus thibetanus laniger MH281753 2	Prionailurus viverrinus NC028305 1	0,18748806
Prionailurus planiceps NC028312 6	Melursus ursinus NC009970 2	0,18748946
Ursus americanus JX196366 3	Galidictis fasciata DM333 1	0,18749546
Panthera pardus NC010641 5	Meles leucurus NC039173 4	0,18749693
Canis adustus KT448271 1	Tapirus terrestris T358	0,1874979
Martes foina NC020643 1	Urva javanica/auropunctata NC006835	0,18750072
Ursus americanus JX196366 3	Procyon lotor AB462049 4	0,18750383
Puma concolor NC016470 22	Arctocephalus forsteri NC004023 28	0,18750432
Ursus americanus JX196366 3	Bassariscus sumichrasti SRX1099089 1	0,18750535
Felis catus NC001700 2	Eumetopias jubatus NC004030 10	0,187511
Neovison vison NC020641 3	Neofelis nebulosa NC008450 3	0,18751152
Lutra sumatrana NC035810 1	Bdeogale nigripes GLC15 1	0,18751304
Ursus arctos pruinosus MG066703 3	Civettictis civetta NC033378 1	0,18751584
Phocarctos hookeri NC008418 1	Urva semitorquata MH464789 1	0,18751678
Mungos mungo/gambianus SRR77048	Ailurus fulgens NC011124 1	0,18751772
Felis margarita NC028308 1	Tapirus terrestris T358	0,18752004
Felis silvestris lybica KP202275 4	Tapirus terrestris T358	0,187521
Pardofelis marmorata NLN3 2	Tapirus terrestris T358	0,18752104
Mustela nigripes NC024942 1	Mungotictis decemlineata NC027828 1	0,18752107
Vulpes corsac NC023958 1	Mustela kathiah NC023210 1	0,18752119
Diplogale hosei MH464790 1	Arctotherium sp NC030174 1	0,18752351
Lycaon pictus NC028427 2	Enhydra lutris NC009692 1	0,18752386
Panthera uncia NC010638 1	Lutra lutra NC011358 9	0,18752533
Procyon lotor AB462046 3	Canis latrans NC008093 7	0,18752539
Vulpes vulpes NC008434 5	Paradoxurus hermaphroditus NLNC 1	0,1875255
Lutrogale perspicillata NC035811 1	Leopardus tigrinus NC028317 1	0,18752608
Vulpes lagopus NC026529 3	Paguma larvata PDDS11 2	0,1875262
Lutrogale perspicillata NC035811 1	Leopardus jacobita NC028322 1	0,18752632
Lynx canadensis NC028313 1	Canis mesomelas KT448280 1	0,1875266
Hyaena hyaena NC020669 1	Callorhinus ursinus NC008415 1	0,18752697
Urocyon littoralis catalinae KP129018 1	Martes pennanti NC020664 16	0,18752858

Panthera uncia NC010638 1	Cynogale bennetti KY117544 1	0,16541623
Viverra tangalunga MH464792 1	Potos flavus T414 1	0,16543011
Mustela sibirica NC020637 6	Mellivora capensis T370 1	0,16543847
Mustela sibirica NC020637 6	Hydrurga leptonyx NC008425 1	0,16543873
Taxidea taxus NC020646 1	Phoca fasciata NC008428 1	0,16544479
Martes pennanti NC020664 16	Lobodon carcinophaga NC008423 1	0,16544613
Martes foina NC020643 1	Hydrurga leptonyx NC008425 1	0,16545378
Ursus thibetanus formosanus NC009331 1	Phoca groenlandica NC008429 54	0,16545978
Leptonychotes weddellii NC008424 1	Leopardus colocolo NC028314 1	0,16546214
Mirounga leonina NC008422 1	Arctonyx collaris NC020645 1	0,16549349
Martes americana NC020642 1	Leptonychotes weddellii NC008424 1	0,16549508
Martes zibellina NC011579 39	Leptonychotes weddellii NC008424 1	0,16549526
Potos flavus T414 1	Mustela eversmannii NC028013 1	0,16549672
Procyon lotor AB462049 4	Meles leucurus NC039173 4	0,16549902
Mustela kathiah NC023210 1	Lobodon carcinophaga NC008423 1	0,16550586
Mustela nigripes NC024942 1	Lobodon carcinophaga NC008423 1	0,16550651
Martes zibellina NC011579 39	Hydrurga leptonyx NC008425 1	0,16550934
Prionodon pardicolor NC024569 2	Parahyaena brunnea NC038159 15	0,16552722
Mustela eversmannii NC028013 1	Mellivora capensis T370 1	0,16557309
Viverra tangalunga MH464792 1	Hydrurga leptonyx NC008425 1	0,16557316
Martes martes T302 3	Lobodon carcinophaga NC008423 1	0,16557918
Mustela nivalis T306 5	Mellivora capensis T370 1	0,1655798
Ursus thibetanus thibetanus NC011118 4	Leptonychotes weddellii NC008424 1	0,16559075
Mirounga angustirostris SRR10331586 1	Homotherium latidens MF871702 3	0,16559613
Neofelis nebulosa NC008450 3	Urva brachyura KY117547 1	0,16560835
Halichoerus grypus NC001602 2	Arctotherium sp NC030174 1	0,1656251
Pusa hispida NC 008433 1	Lutra lutra NC011358 9	0,16563912
Mirounga leonina NC008422 1	Bassariscus sumichrasti SRX1099089 1	0,16564301
Martes foina NC020643 1	Leptonychotes weddellii NC008424 1	0,16564308
Pusa sibirica NC008432 2	Martes foina NC020643 1	0,16564454
Procyon lotor AB462046 3	Mustela altaica NC021751 1	0,1656449
Leptonychotes weddellii NC008424 1	Leopardus pardalis NC028315 1	0,16564536
Prionailurus bengalensis CKM45 20	Leptonychotes weddellii NC008424 1	0,16564801
Viverricula indica KX891745 1	Lobodon carcinophaga NC008423 1	0,1656511
Leptailurus serval NC028316 1	Hydrurga leptonyx NC008425 1	0,1656539
Parahyaena brunnea NC038159 15	Paradoxurus jerdoni MH464793 1	0,16566244
Ursus spelaeus NC011112 8	Phoca fasciata NC008428 1	0,16566417
Gulo gulo NC009685 3	Erignathus barbatus NC008426 1	0,16566658
Ursus thibetanus formosanus NC009331 1	Hydrurga leptonyx NC008425 1	0,16567083
Meles leucurus NC039173 4	Cystophora cristata NC008427 1	0,16569671
Lobodon carcinophaga NC008423 1	Canis adustus KT448271 1	0,16570839
Mustela itatsi NC034330 19	Hydrurga leptonyx NC008425 1	0,16570847
Phoca fasciata NC008428 1	Mustela frenata NC020640 1	0,16570914
Ursus thibetanus mupinensis NC008753 2	Hydrurga leptonyx NC008425 1	0,16572809
Ommatophoca rossii AY377287etc 1	Odobenus rosmarus NC004029 29	0,16574116
Parahyaena brunnea NC038159 15	Arctictis binturong T605 2	0,16574527
Martes melampus NC009678 1	Erignathus barbatus NC008426 1	0,16576017
Martes zibellina NC011579 39	Erignathus barbatus NC008426 1	0,16576564
Procyon lotor AB462046 3	Meles anakuma NC009677 1	0,16576695
Meles anakuma NC009677 1	Bassariscus sumichrasti SRX1099089 1	0,16576859
Melogale moschata KP726273 1	Bassariscus sumichrasti SRX1099089 1	0,1657735
Mustela putorius NC020638 4	Hydrurga leptonyx NC008425 1	0,16577557
Leptonychotes weddellii NC008424 1	Leopardus jacobita NC028322 1	0,16578072
Prionodon pardicolor NC024569 2	Hyaena hyaena NC020669 1	0,16578934
Leopardus tigrinus NC028317 1	Hydrurga leptonyx NC008425 1	0,1657901
Pusa hispida NC 008433 1	Martes foina NC020643 1	0,16579039
Phoca largha NC008430 1	Martes foina NC020643 1	0,16579178
Monachus monachus NC044972 5	Arctotherium sp NC030174 1	0,16580845
Mustela sibirica NC020637 6	Leptonychotes weddellii NC008424 1	0,16583171
Mustela nigripes NC024942 1	Leptonychotes weddellii NC008424 1	0,16583222
Procyon lotor AB462046 3	Arctonyx collaris NC020645 1	0,16583483
Martes melampus NC009678 1	Lobodon carcinophaga NC008423 1	0,16583939
Melogale moschata V0735A 1	Bassariscus sumichrasti SRX1099089 1	0,16584107
Phoca largha NC008430 1	Mustela frenata NC020640 1	0,16584894
Lynx rufus NC014456 3	Hydrurga leptonyx NC008425 1	0,16585618
Viverricula indica NC025296 2	Hydrurga leptonyx NC008425 1	0,16585784
Leopardus geoffroyi NC028320 1	Hydrurga leptonyx NC008425 1	0,16585849
Ursus spelaeus EU327344 13	Leptonychotes weddellii NC008424 1	0,16586024
Martes flavigula NC012141 3	Erignathus barbatus NC008426 1	0,16587044
Ommatophoca rossii AY377287etc 1	Nandinia binotata NC024567 1	0,16587208
Lutra sumatrana NC035810 1	Halichoerus grypus NC001602 2	0,16588031
Crocuta crocuta NC020670 3	Arctictis binturong T605 2	0,16588834
Viverra tangalunga MH464792 1	Procyon lotor AB462046 3	0,16590515
Leptonychotes weddellii NC008424 1	Leopardus wiedii NC028318 1	0,16591525
Potos flavus T414 1	Monachus schauinslandi NC008421 1	0,16591632
Leopardus wiedii NC028318 1	Hydrurga leptonyx NC008425 1	0,16592525
Leptonychotes weddellii NC008424 1	Genetta abyssinica MG489822 1	0,16595319
Pusa hispida NC 008433 1	Meles meles T303 3	0,16597524
Mustela putorius NC020638 4	Mellivora capensis T370 1	0,16597694
Neovison vison NC020641 3	Mellivora capensis T370 1	0,16598308
Mustela altaica NC021751 1	Mellivora capensis T370 1	0,16598324
Mustela nivalis T306 5	Lobodon carcinophaga NC008423 1	0,16598791
Ursus spelaeus NC011112 8	Cystophora cristata NC008427 1	0,16599394
Paradoxurus hermaphroditus NLNC 1	Crocuta crocuta NC020670 3	0,16599997
Ursus spelaeus EU327344 13	Phoca fasciata NC008428 1	0,16600084
Mellivora capensis T370 1	Aonyx cinerea NC035814 2	0,16602732
Panthera tigris amoyensis NC014770 2	Cynogale bennetti KY117544 1	0,16602783
Lutra lutra NC011358 9	Cystophora cristata NC008427 1	0,16603096
Potos flavus T414 1	Meles anakuma NC009677 1	0,16603187
Phoca largha NC008430 1	Lutra lutra NC011358 9	0,16604461
Potos flavus T414 1	Mirounga angustirostris SRR10331586 1	0,16605195
Hydrurga leptonyx NC008425 1	Felis nigripes NC028309 1	0,16605899
Viverricula indica NC025296 2	Lobodon carcinophaga NC008423 1	0,16605946
Ursus thibetanus thibetanus NC011118 4	Lobodon carcinophaga NC008423 1	0,1660762
Proteles cristata T393 6	Panthera tigris NC010642 35	0,16607714
Hydrurga leptonyx NC008425 1	Gulo gulo NC009685 3	0,1660801
Mustela nigripes NC024942 1	Cystophora cristata NC008427 1	0,16610366
Mustela erminea T305 2	Monachus schauinslandi NC008421 1	0,16611373
Mirounga angustirostris SRR10331586 1	Meles meles T303 3	0,16611473
Martes americana NC020642 1	Lobodon carcinophaga NC008423 1	0,16611528
Puma concolor NC016470 22	Hydrurga leptonyx NC008425 1	0,16612542
Ursus spelaeus NC011112 8	Erignathus barbatus NC008426 1	0,16612908
Meles meles T303 3	Cystophora cristata NC008427 1	0,16616832

Panthera uncia KP202269 1	Mustela sibirica NC020637 6	0,18752865
Panthera uncia KP202269 1	Mustela eversmannii NC028013 1	0,18752876
Panthera pardus japonensis KJ866876	Mustela eversmannii NC028013 1	0,18752933
Ictonyx striatus T299 1	Urva semitorquata MH464789 1	0,18752937
Vulpes corsac NC023958 1	Caracal caracal NC028306 1	0,18753088
Vulpes ferrilata NC027935 1	Neovison vison NC020641 3	0,18753095
Vulpes corsac NC023958 1	Otocolobus manul NC028323 1	0,18753116
Puma concolor NC016470 22	Arctocephalus gazella BK010918 1	0,18753123
Vulpes zerda KJ603240 1	Prionailurus planiceps KY682741 4	0,18753152
Panthera onca KP202264 2	Arctocephalus pusillus NC008417 1	0,18753447
Proteles cristata T393 6	Arctonyx collaris NC020645 1	0,1875347
Panthera tigris amoyensis NC014770 2	Lutra lutra LC050126 1	0,18753512
Zalophus wollebaeki SRR4431565 1	Nyctereutes procyonoides NC013700 3	0,18753548
Galictis vittata T412 1	Fossa fossana D350 1	0,1875366
Procyon lotor AB462046 3	Paradoxurus hermaphroditus NC03959	0,18753663
Urva brachyura KY117547 1	Chrysocyon brachyurus NC024172 1	0,18753672
Poecilogale albinucha T602 1	Caracal caracal NC028306 1	0,18753766
Profelis aurata NC028299 1	Martes pennanti NC020664 16	0,18753809
Panthera pardus japonensis KJ866876	Mustela nivalis T306 5	0,18753877
Ichneumia albicauda T603 1	Chrysocyon brachyurus NC024172 1	0,18753886
Galictis vittata T412 1	Arctocephalus townsendi NC008420 1	0,18753913
Panthera tigris NC010642 35	Lutra sumatrana NC035810 1	0,18753958
Melursus ursinus NC009970 2	Crossarchus platycephalus C7R66 1	0,18754052
Ursus maritimus NC003428 31	Lynx rufus NC014456 3	0,18754128
Ursus arctos EU497665 29	Lynx rufus NC014456 3	0,18754141
Helogale parvula SRR7637809 1	Canis lupus chanco NC010340 4	0,1875418
Tremarctos ornatus NC009969 2	Lycalopex sechurae KT448284 1	0,18754268
Ursus arctos EU497665 29	Caracal caracal NC028306 1	0,18754372
Ursus arctos AP012576 6	Lutra lutra LC050126 1	0,18754408
Panthera leo spelaea KX258452 2	Canis latrans NC008093 7	0,18754499
Ursus arctos GU573486 5	Genetta genetta T297 1	0,18754511
Ursus arctos AP012576 6	Chrotogale owstoni T607 1	0,18754542
Ursus arctos GU573491 207	Leopardus geoffroyi NC028320 1	0,18754633
Ursus arctos EU497665 29	Prionailurus bengalensis CKM45 20	0,1875466
Paradoxurus jerdoni MH464793 1	Arctocephalus forsteri NC004023 28	0,18754701
Ursus arctos GU573486 5	Leopardus pardalis T262 1	0,18754841
Ursus arctos GU573486 5	Leopardus wiedii NC028318 1	0,18754853
Ursus spelaeus EU327344 13	Procyon lotor AB462049 4	0,18754856
Paguma larvata PDDS11 2	Melursus ursinus NC009970 2	0,18755366
Panthera onca NC022842 1	Lycaon pictus NC028427 2	0,18755399
Helarctos malayanus NC009968 2	Felis nigripes NC028309 1	0,18755522
Prionailurus viverrinus NC028305 1	Helarctos malayanus NC009968 2	0,18755609
Fossa fossana D350 1	Canis mesomelas KT448280 1	0,18755868
Eupleres goudotii D128 1	Arctocephalus forsteri NC004023 28	0,18755893
Potos flavus T414 1	Panthera leo NERO 19	0,18756336
Ursus thibetanus formosanus NC00933	Galictis vittata T412 1	0,18756366
Ursus thibetanus laniger MH281753 2	Galictis vittata T412 1	0,18756495
Vulpes vulpes NC008434 5	Eumetopias jubatus NC004030 10	0,18756767
Conepatus chinga NC042596 1	Arctocephalus australis MG023139 1	0,18758047
Lontra canadensis SRR10409165 1	Eupleres goudotii D128 1	0,18758124
Bdeogale nigripes GLC15 1	Arctocephalus australis MG023139 1	0,18758398
Crossarchus platycephalus C7R66 1	Ailurus fulgens NC011124 1	0,18758419
Lontra canadensis SRR10409165 1	Galidictis fasciata DM333 1	0,18758439
Neofelis nebulosa NC008450 3	Ictonyx striatus T299 1	0,18758439
Panthera pardus NC010641 5	Martes foina NC020643 1	0,18758659
Nyctereutes procyonoides NC013700 3	Urva brachyura KY117547 1	0,18758722
Vulpes corsac NC023958 1	Galidictis fasciata DM333 1	0,18758869
Meles anakuma NC009677 1	Galerella sanguinea T378 1	0,18759024
Meles leucurus NC039173 4	Cryptoprocta ferox CFC13 1	0,1875916
Xenogale naso C07XAR110 1	Arctotherium sp NC030174 1	0,18759209
Procyon lotor AB462049 4	Canis latrans NC008093 7	0,18759228
Phoca largha NC008430 1	Cynogale bennetti KY117544 1	0,18759489
Mustela eversmannii NC028013 1	Cryptoprocta ferox CFC13 1	0,18759525
Ichneumia albicauda T603 1	Arctocephalus australis MG023139 1	0,18759587
Vulpes vulpes NC008434 5	Genetta genetta T297 1	0,18759617
Vulpes corsac NC023958 1	Pardofelis marmorata NLN3 2	0,18759726
Profelis aurata NC028299 1	Mustela sibirica NC020637 6	0,1875973
Vulpes ferrilata NC027935 1	Prionailurus planiceps NC028312 6	0,18759811
Felis silvestris lybica KP202275 4	Arctocephalus gazella BK010918 1	0,18759817
Smilodon populator MF871700 1	Galictis vittata T412 1	0,18759817
Urocyon cinereoargenteus NC026723 2	Lynx lynx NC027083 4	0,1875985
Parahyaena brunnea NC038159 15	Mustela kathiah NC023210 1	0,18759918
Ursus thibetanus mupinensis NC00875	Bassaricyon neblina SRX1097850 1	0,18759952
Lontra canadensis SRR10409165 1	Leopardus pardalis NC028315 1	0,1875996
Otocyon megalotis SAF1 2	Genetta servalina NC024568 2	0,18760188
Panthera pardus japonensis KJ866876	Aonyx cinerea NC035814 2	0,18760224
Ictonyx striatus T299 1	Eupleres goudotii D128 1	0,18760271
Chrysocyon brachyurus NC024172 1	Bdeogale nigripes GLC15 1	0,18760273
Lutrogale perspicillata NC035811 1	Felis catus NC001700 2	0,18760301
Leopardus guigna NC028321 1	Canis anthus NC027956 2	0,18760332
Panthera uncia KP202269 1	Neovison vison NC020641 3	0,18760341
Melogale moschata NC020644 1	Lycaon pictus NC028427 2	0,1876041
Martes martes T302 3	Hyaena hyaena NC020669 1	0,18760526
Panthera tigris NC010642 35	Melogale moschata KP726273 1	0,18760571
Otocolobus manul NC028323 1	Arctotherium sp NC030174 1	0,18760598
Cynictis penicillata T375 1	Ailuropoda melanoleuca NC009492 5	0,18760608
Melursus ursinus NC009970 2	Lutra lutra NC011358 9	0,18760674
Galictis vittata T412 1	Crossarchus platycephalus C7R66 1	0,18760805
Ursus arctos pruinosus MG066703 3	Lynx lynx NC027083 4	0,18760841
Ursus maritimus NC003428 31	Lynx canadensis NC028313 1	0,1876085
Ursus thibetanus formosanus NC00933	Mungos mungo MMC7 1	0,1876086
Speothos venaticus C48 2	Lynx lynx NC027083 4	0,18760896
Ursus thibetanus formosanus NC00933	Lontra canadensis SRR10409165 1	0,18760955
Ursus arctos isabellinus 18B5 2	Leptailurus serval NC028316 1	0,18761058
Speothos venaticus C48 2	Felis catus NC001700 2	0,1876107
Ursus maritimus NC003428 31	Caracal caracal NC028306 1	0,18761098
Odobenus rosmarus NC004029 29	Neovison vison NC020641 3	0,18761253
Ictonyx striatus T299 1	Canis latrans NC008093 7	0,18761285
Ursus arctos pruinosus MG066703 3	Catopuma temminckii NC027115 41	0,1876135
Melursus ursinus NC009970 2	Hyaena hyaena NC020669 1	0,18761374
Ursus arctos GU573486 5	Lynx rufus NC014456 3	0,18761463
Martes martes T302 3	Ailuropoda melanoleuca NC009492 5	0,18761495
Ursus arctos GU573486 5	Prionailurus rubiginosus NC028304 2	0,1876169

Leptonychotes weddellii NC008424 1	Aonyx cinerea NC035814 2	0,166177
Monachus schauinslandi NC008421 1	Meles leucurus NC039173 4	0,16617784
Phoca vitulina NC001325 1	Lutra lutra LC050126 1	0,1661796
Monachus schauinslandi NC008421 1	Arctodus simus NC011116 1	0,16619566
Viverra tangalunga MH464792 1	Bassariscus sumichrasti SRX1099089 1	0,16624364
Potos flavus T414 1	Phoca fasciata NC008428 1	0,16625002
Procyon lotor AB462049 4	Martes martes T302 3	0,16625084
Otocolobus manul NC028323 1	Hydrurga leptonyx NC008425 1	0,16625972
Mirounga angustirostris SRR10331586 1	Bassariscus sumichrasti SRX1099089 1	0,16626208
Crocuta crocuta NC020670 3	Chrotogale owstoni T607 1	0,16626252
Mellivora capensis T370 1	Lutrogale perspicillata NC035811 1	0,16629584
Pusa sibirica NC008432 2	Lutra sumatrana NC035810 1	0,16630268
Martes americana NC020642 1	Erignathus barbatus NC008426 1	0,16630414
Potos flavus T414 1	Mirounga leonina NC008422 1	0,16630886
Procyon lotor AB462046 3	Mustela putorius NC020638 4	0,16630949
Suricata suricatta SSM10 1	Prionodon pardicolor NC024569 2	0,16631791
Panthera uncia NC010638 1	Crocuta crocuta NC020670 3	0,16633856
Hydrurga leptonyx NC008425 1	Acinonyx jubatus NC005212 3	0,16634227
Mellivora capensis T370 1	Meles leucurus NC039173 4	0,16636382
Martes melampus NC009678 1	Cystophora cristata NC008427 1	0,16636774
Pusa caspica NC008431 1	Mustela frenata NC020640 1	0,16637562
Pusa hispida NC 008433 1	Arctonyx collaris NC020645 1	0,16637896
Mustela frenata NC020640 1	Mellivora capensis T370 1	0,16638112
Mustela nigripes NC024942 1	Hydrurga leptonyx NC008425 1	0,16638164
Ursus thibetanus mupinensis NC008753 2	Leptonychotes weddellii NC008424 1	0,16638671
Pusa hispida NC 008433 1	Potos flavus T414 1	0,16638713
Proteles cristata T393 6	Paguma larvata PDDS11 2	0,1664037
Ursus thibetanus formosanus NC009331 1	Lobodon carcinophaga NC008423 1	0,16641058
Martes flavigula NC012141 3	Cystophora cristata NC008427 1	0,16641554
Pusa hispida NC 008433 1	Gulo gulo NC009685 3	0,16642192
Monachus schauinslandi NC008421 1	Lutra lutra LC050126 1	0,16644318
Potos flavus T414 1	Cystophora cristata NC008427 1	0,16644402
Leptonychotes weddellii NC008424 1	Felis silvestris lybica KP202275 4	0,16645131
Martes americana NC020642 1	Hydrurga leptonyx NC008425 1	0,16645171
Phoca largha NC008430 1	Martes martes T302 3	0,16645744
Hydrurga leptonyx NC008425 1	Felis silvestris lybica KP202275 4	0,16646205
Ursus thibetanus mupinensis NC008753 2	Lobodon carcinophaga NC008423 1	0,1664688
Phoca fasciata NC008428 1	Melursus ursinus NC009970 2	0,16647068
Ursus thibetanus thibetanus NC011118 4	Phoca fasciata NC008428 1	0,16647229
Potos flavus T414 1	Monachus monachus NC044972 5	0,16648152
Procyon lotor AB462046 3	Neovison vison NC020641 3	0,16651873
Hydrurga leptonyx NC008425 1	Ailurus fulgens styani NC009691 1	0,16651908
Leptonychotes weddellii NC008424 1	Ictonyx striatus T299 1	0,16652067
Ursus thibetanus mupinensis NC008753 2	Phoca fasciata NC008428 1	0,16652788
Ursus spelaeus EU327344 13	Erignathus barbatus NC008426 1	0,16653314
Proteles cristata T393 6	Panthera tigris amoyensis NC014770 2	0,16654864
Leopardus colocolo NC028314 1	Hydrurga leptonyx NC008425 1	0,16654901
Potos flavus T414 1	Halichoerus grypus NC001602 2	0,16656007
Lutra sumatrana NC035810 1	Erignathus barbatus NC008426 1	0,16656943
Eupleres goudotii D128 1	Cynogale bennetti KY117544 1	0,16657175
Meles anakuma NC009677 1	Lobodon carcinophaga NC008423 1	0,16657901
Phoca groenlandica NC008429 54	Meles meles T303 3	0,16658179
Ursus arctos isabellinus 18B5 2	Leptonychotes weddellii NC008424 1	0,16658789
Ursus maritimus NC003428 31	Leptonychotes weddellii NC008424 1	0,16658798
Potos flavus T414 1	Poecilogale albinucha T602 1	0,16659142
Hydrurga leptonyx NC008425 1	Genetta servalina NC024568 2	0,16659604
Hydrurga leptonyx NC008425 1	Canis adustus KT448271 1	0,16660258
Phoca fasciata NC008428 1	Helarctos malayanus NC009968 2	0,16660571
Phoca groenlandica NC008429 54	Mustela frenata NC020640 1	0,1666519
Procyon lotor AB462046 3	Martes martes T302 3	0,16665466
Pusa caspica NC008431 1	Meles meles T303 3	0,16670716
Procyon lotor AB462049 4	Arctonyx collaris NC020645 1	0,16670987
Procyon lotor AB462046 3	Mustela nigripes NC024942 1	0,16671327
Pusa hispida NC 008433 1	Martes melampus NC009678 1	0,16671678
Neovison vison NC020641 3	Cystophora cristata NC008427 1	0,16671902
Leptonychotes weddellii NC008424 1	Leopardus tigrinus NC028317 1	0,16672327
Martes pennanti NC020664 16	Hydrurga leptonyx NC008425 1	0,16672485
Melogale moschata NC020644 1	Lobodon carcinophaga NC008423 1	0,16672514
Melursus ursinus NC009970 2	Leptonychotes weddellii NC008424 1	0,16673359
Prionailurus viverrinus NC028305 1	Hydrurga leptonyx NC008425 1	0,16673402
Nandinia binotata NC024567 1	Halichoerus grypus NC001602 2	0,16675968
Pusa caspica NC008431 1	Lutra sumatrana NC035810 1	0,16677369
Pusa hispida NC 008433 1	Meles leucurus NC039173 4	0,16678321
Erignathus barbatus NC008426 1	Bassariscus sumichrasti SRX1099089 1	0,16678336
Monachus monachus NC044972 5	Homotherium latidens MF871702 3	0,16678368
Mustela nivalis T306 5	Leptonychotes weddellii NC008424 1	0,16678402
Viverra tangalunga MH464792 1	Mellivora capensis T370 1	0,1667847
Mustela frenata NC020640 1	Monachus schauinslandi NC008421 1	0,16678563
Martes foina NC020643 1	Cystophora cristata NC008427 1	0,16678876
Prionailurus viverrinus NC028305 1	Leptonychotes weddellii NC008424 1	0,16679111
Viverricula indica NC025296 2	Leptonychotes weddellii NC008424 1	0,16679318
Ursus arctos GU573486 5	Leptonychotes weddellii NC008424 1	0,16679643
Ursus thibetanus formosanus NC009331 1	Leptonychotes weddellii NC008424 1	0,16680083
Ursus thibetanus laniger MH281753 2	Phoca fasciata NC008428 1	0,16680653
Procyon lotor AB462049 4	Martes flavigula NC012141 3	0,16682206
Meles leucurus NC039173 4	Erignathus barbatus NC008426 1	0,16684097
Tremarctos ornatus NC009969 2	Phoca fasciata NC008428 1	0,16684748
Procyon lotor AB462046 3	Mustela frenata NC020640 1	0,16684772
Pusa hispida NC 008433 1	Mustela itatsi NC034330 19	0,1668538
Lobodon carcinophaga NC008423 1	Genetta abyssinica MG489822 1	0,16685423
Prionailurus rubiginosus NC028304 2	Leptonychotes weddellii NC008424 1	0,16685608
Leptonychotes weddellii NC008424 1	Leptailurus serval NC028316 1	0,16685651
Lobodon carcinophaga NC008423 1	Leopardus jacobita NC028322 1	0,1668677
Phoca vitulina NC001325 1	Martes foina NC020643 1	0,1668693
Ursus arctos isabellinus 18B5 2	Hydrurga leptonyx NC008425 1	0,16687199
Mustela erminea T305 2	Monachus monachus NC044972 5	0,16687779
Mustela frenata NC020640 1	Halichoerus grypus NC001602 2	0,16689168
Mustela kathiah NC023210 1	Leptonychotes weddellii NC008424 1	0,16690901
Pusa sibirica NC008432 2	Meles meles T303 3	0,16690929
Mirounga leonina NC008422 1	Arctotherium sp NC030174 1	0,16691865
Procyon lotor AB462049 4	Neovison vison NC020641 3	0,16692239
Viverricula indica KX891745 1	Leptonychotes weddellii NC008424 1	0,16692284
Pusa caspica NC008431 1	Martes foina NC020643 1	0,16692381

Ursus thibetanus formosanus NC0093	Paradoxurus hermaphroditus NLNC 1	0,18761691
Melogale moschata KP726273 1	Urva javanica/auropunctata NC006835	0,18761893
Ursus maritimus NC003428 31	Arctictis binturong T605 2	0,18761984
Ursus spelaeus NC011112 8	Panthera onca KP202264 2	0,18762082
Galictis vittata T412 1	Catopuma temminckii NC027115 41	0,18762268
Ursus arctos pruinosus MG066703 3	Acinonyx jubatus NC005212 3	0,18762627
Enhydra lutris NC009692 1	Cuon alpinus NC013445 3	0,18762995
Mungotictis decemlineata NC027828 1	Gulo gulo NC009685 3	0,18763006
Panthera leo NERO 19	Mustela eversmannii NC028013 1	0,1876311
Prionodon linsang ERR2391707 1	Cuon alpinus NC013445 3	0,18763272
Panthera pardus NC010641 5	Lutra lutra LC050126 1	0,18763553
Ursus arctos AP012576 6	Ictonyx striatus T299 1	0,18763565
Mephitis mephitis NC020648 1	Helarctos malayanus NC009968 2	0,18763709
Panthera tigris amoyensis NC014770 2	Canis aureus KT448274 1	0,18763748
Ursus americanus JX196366 3	Lynx rufus NC014456 3	0,18763895
Ursus americanus JX196366 3	Leptailurus serval NC028316 1	0,1876432
Enhydra lutris NC009692 1	Bdeogale nigripes GLC15 1	0,18764615
Martes foina NC020643 1	Cuon alpinus NC013445 3	0,18764733
Mungos mungo/gambianus SRR77048	Melogale moschata V0735A 1	0,18764818
Suricata suricatta SSM10 1	Meles meles T303 3	0,18764818
Enhydra lutris NC009692 1	Canis anthus NC027956 2	0,18764862
Martes zibellina NC011579 39	Eupleres goudotii D128 1	0,18764928
Urva semitorquata MH464789 1	Arctocephalus townsendi NC008420 1	0,18765059
Nyctereutes procyonoides NC013700 3	Meles leucurus NC039173 4	0,18765357
Martes flavigula NC012141 3	Cryptoprocta ferox CFC13 1	0,18765412
Procyon lotor AB462049 4	Atilax paludinosus T606 1	0,18765475
Cynictis penicillata T375 1	Aonyx cinerea NC035814 2	0,18765499
Mustela itatsi NC034330 19	Mungotictis decemlineata NC027828 1	0,1876561
Salanoia concolor D378 1	Arctocephalus forsteri KT693377 17	0,18765651
Otocolobus manul NC028323 1	Tapirus terrestris T358	0,18765669
Spilogale putorius NC010497 1	Leopardus colocolo NC028314 1	0,1876588
Zalophus californianus NC008416 1	Urva javanica/auropunctata NC006835	0,18765882
Paradoxurus hermaphroditus NC03959	Melogale moschata KP726273 1	0,18765973
Otocyon megalotis SAF1 2	Fossa fossana D350 1	0,18766041
Spilogale putorius NC010497 1	Canis aureus KT448274 1	0,1876619
Ursus arctos GU573491 207	Lutra lutra LC050126 1	0,18766247
Viverra tangalunga MH464792 1	Panthera uncia NC010638 1	0,18766294
Panthera uncia KP202269 1	Mustela sibirica AP017394 11	0,1876633
Nyctereutes procyonoides NC013700 3	Arctotherium sp NC030174 1	0,18766344
Catopuma badia NC028300 1	Arctocephalus townsendi NC008420 1	0,18766348
Vulpes ferrilata NC027935 1	Lynx canadensis NC028313 1	0,18766506
Vulpes lagopus NC026529 3	Caracal caracal NC028306 1	0,18766589
Lontra canadensis SRR10409165 1	Catopuma temminckii NC027115 41	0,18766661
Lontra canadensis SRR10409165 1	Felis catus NC001700 2	0,18766682
Urocyon cinereoargenteus NC026723 2	Leopardus wiedii NC028318 1	0,18766697
Neophoca cinerea NC008419 1	Felis catus NC001700 2	0,1876681
Vulpes lagopus NC026529 3	Leopardus guigna NC028321 1	0,18766874
Viverra tangalunga MH464792 1	Panthera tigris NC010642 35	0,18767259
Panthera tigris amoyensis NC014770 2	Martes melampus NC009678 1	0,18767264
Panthera pardus japonensis KJ866876	Otocyon megalotis SAF1 2	0,18767448
Martes zibellina NC011579 39	Arctictis binturong T605 2	0,18767537
Speothos venaticus C48 2	Genetta abyssinica MG489822 1	0,18767592
Ursus maritimus GU573488 Svalbard	Lynx rufus NC014456 3	0,18767595
Ursus arctos isabellinus 18B5 2	Leopardus wiedii NC028318 1	0,18767628
Galictis vittata T412 1	Arctocephalus gazella BK010918 1	0,18767653
Ursus arctos isabellinus 18B5 2	Prionailurus viverrinus NC028305 1	0,18767688
Ursus maritimus NC003428 31	Prionailurus planiceps KY682741 4	0,18767707
Helarctos malayanus NC009968 2	Bdeogale nigripes GLC15 1	0,18767715
Neophoca cinerea NC008419 1	Ailuropoda melanoleuca NC009492 5	0,18767773
Salanoia concolor D378 1	Melursus ursinus NC009970 2	0,18767777
Prionodon linsang ERR2391707 1	Helarctos malayanus NC009968 2	0,18767844
Ursus arctos pruinosus MG066703 3	Puma yagouaroundi NC028311 1	0,18767852
Ursus maritimus GU573488 Svalbard	Leptailurus serval NC028316 1	0,18767852
Martes foina NC020643 1	Hyaena hyaena NC020669 1	0,18767911
Speothos venaticus C48 2	Felis chaus NC028307 1	0,1876794
Panthera pardus japonensis KJ866876	Ictonyx striatus T299 1	0,18768054
Ursus arctos pruinosus MG066703 3	Prionailurus bengalensis CKM45 20	0,18768141
Suricata suricatta SSM10 1	Canis lupus chanco NC010340 4	0,1876817
Ursus thibetanus formosanus NC0093	Otocyon megalotis SAF1 2	0,18768302
Ursus arctos GU573486 5	Prionailurus planiceps KY682741 4	0,18768327
Panthera tigris amoyensis NC014770 2	Martes martes T302 3	0,18768351
Ursus arctos GU573486 5	Caracal caracal NC028306 1	0,18768473
Puma yagouaroundi NC028311 1	Mephitis mephitis NC020648 1	0,18768486
Mungotictis decemlineata NC027828 1	Canis lupus familiaris NC002008 1231	0,18769008
Mephitis mephitis NC020648 1	Catopuma badia NC028300 1	0,18769079
Melogale moschata NC020644 1	Canis aureus KT448274 1	0,18769184
Spilogale putorius NC010497 1	Genetta abyssinica MG489822 1	0,18769205
Zalophus wollebaeki SRR4431565 1	Panthera onca NC022842 1	0,18769208
Xenogale naso C07XAR110 1	Gulo gulo NC009685 3	0,18769261
Zalophus californianus NC008416 1	Panthera uncia KP202269 1	0,18769434
Lutra sumatrana NC035810 1	Canis lupus chanco NC010340 4	0,18769683
Ursus arctos AP012576 6	Catopuma badia NC028300 1	0,18769698
Panthera leo NERO 19	Mustela itatsi NC034330 19	0,18769836
Cuon alpinus NC013445 3	Ailurus fulgens styani NC009691 1	0,18770193
Cynogale bennetti KY117544 1	Tapirus terrestris T358	0,18770285
Panthera tigris NC010642 35	Canis lupus chanco NC010340 4	0,1877033
Mungos mungo/gambianus SRR77048	Cuon alpinus NC013445 3	0,18770494
Canis mesomelas KT448280 1	Bassariscus sumichrasti SRX1099089 1	0,18770567
Caracal caracal NC028306 1	Arctocephalus forsteri NC004023 28	0,18770693
Vulpes vulpes NC008434 5	Panthera pardus NC010641 5	0,18770702
Ursus americanus JX196366 3	Prionailurus planiceps KY682741 4	0,18770841
Urocyon cinereoargenteus NC026723 2	Martes flavigula NC012141 3	0,18770845
Ursus americanus JX196366 3	Aonyx cinerea NC035814 2	0,18770881
Panthera pardus NC010641 5	Canis latrans NC008093 7	0,18770885
Gulo gulo NC009685 3	Chrysocyon brachyurus NC024172 1	0,18770987
Lutra sumatrana NC035810 1	Helogale parvula SRR7637809 1	0,18771388
Nasua nasua NC020647 1	Homotherium latidens MF871702 3	0,18771434
Atilax paludinosus T606 1	Tapirus terrestris T358	0,1877162
Tremarctos ornatus NC009969 2	Mungos mungo/gambianus SRR77048	0,1877186
Salanoia concolor D378 1	Lontra canadensis SRR10409165 1	0,1877189
Urocyon cinereoargenteus NC026723 2	Nandinia binotata NC024567 1	0,18772025
Suricata suricatta SSM10 1	Canis latrans NC008093 7	0,18772033
Prionodon linsang ERR2391707 1	Mustela sibirica AP017394 11	0,18772107

Ursus maritimus GU573488 Svalbard	Leptonychotes weddellii NC008424 1	0,16692467
Pusa hispida NC 008433 1	Martes martes T302 3	0,16692841
Phoca fasciata NC008428 1	Martes foina NC020643 1	0,16693164
Ursus spelaeus EU327344 13	Cystophora cristata NC008427 1	0,16693674
Leptonychotes weddellii NC008424 1	Gulo gulo NC009685 3	0,16694324
Melursus ursinus NC009970 2	Lobodon carcinophaga NC008423 1	0,16695121
Lobodon carcinophaga NC008423 1	Leopardus colocolo NC028314 1	0,16695252
Lobodon carcinophaga NC008423 1	Gulo gulo NC009685 3	0,16695632
Pusa caspica NC008431 1	Lutra lutra LC050126 1	0,16697496
Mustela eversmannii NC028013 1	Leptonychotes weddellii NC008424 1	0,16697614
Mustela kathiah NC023210 1	Cystophora cristata NC008427 1	0,16697849
Fossa fossana D350 1	Cystophora cristata NC008427 1	0,16698088
Martes zibellina NC011579 39	Bassaricyon neblina SRX1097850 1	0,16698122
Viverra tangalunga MH464792 1	Procyon lotor AB462049 4	0,16698197
Phoca fasciata NC008428 1	Mustela kathiah NC023210 1	0,16698727
Phoca largha NC008430 1	Arctonyx collaris NC020645 1	0,16698922
Lynx pardinus NC028319 161	Leptonychotes weddellii NC008424 1	0,16698942
Melogale moschata KP726273 1	Lobodon carcinophaga NC008423 1	0,16699031
Ursus thibetanus mupinensis NC008753 2	Erignathus barbatus NC008426 1	0,1669922
Pusa sibirica NC008432 2	Melursus ursinus NC009970 2	0,16700134
Leopardus guigna NC028321 1	Hydrurga leptonyx NC008425 1	0,16700324
Lutra lutra LC050126 1	Halichoerus grypus NC001602 2	0,16702454
Lutra lutra LC050126 1	Erignathus barbatus NC008426 1	0,1670391
Tremarctos ornatus NC009969 2	Pusa sibirica NC008432 2	0,16704278
Pusa caspica NC008431 1	Meles leucurus NC039173 4	0,16704349
Pusa sibirica NC008432 2	Mustela frenata NC020640 1	0,1670491
Martes pennanti NC020664 16	Bassariscus sumichrasti SRX1099089 1	0,16705482
Phoca groenlandica NC008429 54	Mustela kathiah NC023210 1	0,16705528
Mirounga angustirostris SRR10331586 1	Arctonyx collaris NC020645 1	0,16705611
Lynx canadensis NC028313 1	Leptonychotes weddellii NC008424 1	0,16705631
Lynx rufus NC014456 3	Leptonychotes weddellii NC008424 1	0,16705658
Procyon lotor AB462049 4	Mustela altaica NC021751 1	0,16705814
Otocolobus manul NC028323 1	Leptonychotes weddellii NC008424 1	0,1670586
Viverricula indica NC025296 2	Erignathus barbatus NC008426 1	0,16706184
Ursus thibetanus laniger MH281753 2	Leptonychotes weddellii NC008424 1	0,16706926
Pusa hispida NC 008433 1	Melursus ursinus NC009970 2	0,16708311
Procyon lotor AB462046 3	Martes flavigula NC012141 3	0,16709059
Pusa sibirica NC008432 2	Martes melampus NC009678 1	0,16710927
Helogale parvula SRR7637809 1	Cynogale bennetti KY117544 1	0,16711384
Martes martes T302 3	Cystophora cristata NC008427 1	0,16711763
Mustela erminea T305 2	Ailurus fulgens NC011124 1	0,16711819
Phoca vitulina NC001325 1	Arctonyx collaris NC020645 1	0,16712391
Mirounga angustirostris SRR10331586 1	Meles leucurus NC039173 4	0,16712455
Helarctos malayanus NC009968 2	Erignathus barbatus NC008426 1	0,16713782
Leptonychotes weddellii NC008424 1	Helarctos malayanus NC009968 2	0,16713873
Leptonychotes weddellii NC008424 1	Acinonyx jubatus NC005212 3	0,16714139
Ursus thibetanus laniger MH281753 2	Lobodon carcinophaga NC008423 1	0,16715141
Ursus spelaeus EU327344 13	Lobodon carcinophaga NC008423 1	0,16715349
Panthera tigris NC010642 35	Cynogale bennetti KY117544 1	0,16717272
Lutra lutra NC011358 9	Erignathus barbatus NC008426 1	0,16717324
Procyon lotor AB462049 4	Meles anakuma NC009677 1	0,16718039
Meles leucurus NC039173 4	Ailurus fulgens styani NC009691 1	0,16718398
Procyon lotor AB462049 4	Mustela putorius NC020638 4	0,16718437
Procyon lotor AB462049 4	Martes zibellina NC011579 39	0,16718736
Tremarctos ornatus NC009969 2	Pusa hispida NC 008433 1	0,16719019
Lynx lynx NC027083 4	Leptonychotes weddellii NC008424 1	0,16719085
Viverricula indica KX891751 1	Leptonychotes weddellii NC008424 1	0,16719213
Viverricula indica KX891751 1	Hydrurga leptonyx NC008425 1	0,16719958
Ursus thibetanus thibetanus NC011118 4	Erignathus barbatus NC008426 1	0,16720651
Mellivora capensis T370 1	Arctonyx collaris NC020645 1	0,16723871
Mustela frenata NC020640 1	Ailurus fulgens styani NC009691 1	0,16724752
Martes melampus NC009678 1	Bassaricyon neblina SRX1097850 1	0,1672478
Procyon lotor AB462046 3	Martes melampus NC009678 1	0,16724868
Pusa sibirica NC008432 2	Martes martes T302 3	0,16725308
Phoca vitulina NC001325 1	Lutra lutra NC011358 9	0,16725639
Mustela nivalis T306 5	Hydrurga leptonyx NC008425 1	0,16726714
Prionailurus planiceps NC028312 6	Hydrurga leptonyx NC008425 1	0,16726974
Phoca groenlandica NC008429 54	Melursus ursinus NC009970 2	0,16727899
Leptonychotes weddellii NC008424 1	Hemigalus derbyanus MH464791 1	0,16729271
Pusa caspica NC008431 1	Martes melampus NC009678 1	0,16731091
Melogale moschata NC020644 1	Leptonychotes weddellii NC008424 1	0,167317
Procyon lotor AB462049 4	Mustela kathiah NC023210 1	0,1673182
Procyon lotor AB462046 3	Mustela kathiah NC023210 1	0,16731825
Tremarctos ornatus NC009969 2	Phoca groenlandica NC008429 54	0,16731925
Mustela kathiah NC023210 1	Hydrurga leptonyx NC008425 1	0,16732365
Phoca largha NC008430 1	Martes melampus NC009678 1	0,16732381
Mustela eversmannii NC028013 1	Lobodon carcinophaga NC008423 1	0,1673245
Leptonychotes weddellii NC008424 1	Felis catus NC001700 2	0,16732654
Viverricula indica KX891751 1	Erignathus barbatus NC008426 1	0,16732675
Homotherium latidens MF871702 3	Erignathus barbatus NC008426 1	0,1673294
Pusa hispida NC 008433 1	Melogale moschata NC020644 1	0,16733241
Ursus arctos GU573486 5	Cystophora cristata NC008427 1	0,16733387
Mustela altaica NC021751 1	Hydrurga leptonyx NC008425 1	0,16733557
Hydrurga leptonyx NC008425 1	Catopuma badia NC028300 1	0,16733646
Lynx canadensis NC028313 1	Hydrurga leptonyx NC008425 1	0,16733758
Hydrurga leptonyx NC008425 1	Caracal caracal NC028306 1	0,167338
Ursus thibetanus formosanus NC009331 1	Phoca fasciata NC008428 1	0,16734514
Phoca largha NC008430 1	Melursus ursinus NC009970 2	0,16734979
Phoca vitulina NC001325 1	Melursus ursinus NC009970 2	0,16734998
Ursus thibetanus thibetanus NC011118 4	Pusa hispida NC 008433 1	0,16735354
Leptonychotes weddellii NC008424 1	Civettictis civetta NC033378 1	0,16736454
Ommatophoca rossii AY377287etc 1	Mustela erminea T305 2	0,1673733
Pusa sibirica NC008432 2	Lutra lutra LC050126 1	0,16737914
Melogale moschata KP726273 1	Leptonychotes weddellii NC008424 1	0,16738011
Procyon lotor AB462049 4	Lutra sumatrana NC035810 1	0,16738382
Phoca fasciata NC008428 1	Martes melampus NC009678 1	0,16738497
Arctonyx collaris NC020645 1	Ailurus fulgens styani NC009691 1	0,16738603
Monachus schauinslandi NC008421 1	Lutra sumatrana NC035810 1	0,16738866
Phoca largha NC008430 1	Martes americana NC020642 1	0,16739455
Lobodon carcinophaga NC008423 1	Ailurus fulgens styani NC009691 1	0,16739476
Leptonychotes weddellii NC008424 1	Civettictis civetta GLC19 1	0,16739503
Martes zibellina NC011579 39	Lobodon carcinophaga NC008423 1	0,16739509
Monachus monachus NC044972 5	Ailurus fulgens styani NC009691 1	0,16742398

Prionodon linsang ERR2391707 1	Mustela nigripes NC024942 1	0,18772162
Mungos mungo/gambianus SRR77048	Melogale moschata NC020644 1	0,18772237
Canis latrans NC008093 7	Ailurus fulgens styani NC009691 1	0,18772589
Lynx lynx NC027083 4	Tapirus terrestris T358	0,18772604
Mustela eversmannii NC028013 1	Galerella sanguinea T378 1	0,18772693
Lutrogale perspicillata NC035811 1	Leopardus wiedii NC028318 1	0,18772785
Ommatophoca rossii AY377287etc 1	Crocuta crocuta NC020670 3	0,18772811
Prionailurus planiceps NC028312 6	Conepatus chinga NC042596 1	0,18772899
Mustela nivalis T306 5	Bdeogale nigripes GLC15 1	0,187729
Ursus maritimus NC003428 31	Lutra lutra NC011358 9	0,18772942
Phoca vitulina NC001325 1	Cynogale bennetti KY117544 1	0,18772969
Otocyon megalotis SAF1 2	Martes americana NC020642 1	0,18772988
Viverra tangalunga MH464792 1	Hyaena hyaena NC020669 1	0,18773036
Vulpes vulpes NC008434 5	Panthera onca KP202264 2	0,1877315
Caracal caracal NC028306 1	Arctocephalus townsendi NC008420 1	0,18773153
Paradoxurus jerdoni MH464793 1	Neophoca cinerea NC008419 1	0,18773282
Vulpes ferrilata NC027935 1	Caracal caracal NC028306 1	0,18773311
Urocyon cinereoargenteus NC026723 2	Prionailurus rubiginosus NC028304 2	0,18773498
Ursus maritimus GU573488 Svalbard	Hyaena hyaena NC020669 1	0,18773599
Ursus maritimus GU573488 Svalbard	Procyon lotor AB462049 4	0,187736
Ursus arctos EU497665 29	Procyon lotor AB462049 4	0,18773605
Ursus arctos GU573486 5	Lutra lutra LC050126 1	0,18773665
Parahyaena brunnea NC038159 15	Otaria byronia OTAB 1	0,18773694
Odobenus rosmarus NC004029 29	Homotherium latidens MF871702 3	0,18773727
Crocuta crocuta NC020670 3	Bassariscus sumichrasti SRX1099089 1	0,18773735
Panthera tigris amoyensis NC014770 2	Arctonyx collaris NC020645 1	0,18773781
Panthera onca NC022842 1	Meles anakuma NC009677 1	0,18773919
Leopardus pardalis NC028315 1	Bassaricyon neblina SRX1097850 1	0,18773977
Felis chaus NC028307 1	Arctotherium sp NC030174 1	0,18774033
Panthera onca KP202264 2	Martes pennanti NC020664 16	0,18774037
Zalophus wollebaeki SRR4431565 1	Mungotictis decemlineata NC027828 1	0,18774109
Vulpes corsac NC023958 1	Helarctos malayanus NC009968 2	0,18774191
Spilogale putorius NC010497 1	Canis lupus chanco NC010340 4	0,18774216
Speothos venaticus C48 2	Genetta servalina NC024568 2	0,18774249
Ursus thibetanus thibetanus NC01111	Bdeogale nigripes GLC15 1	0,18774417
Martes martes T302 3	Chrysocyon brachyurus NC024172 1	0,18774445
Ursus arctos pruinosus MG066703 3	Prionailurus bengalensis NC028301 12	0,18774576
Speothos venaticus C48 2	Prionailurus planiceps KY682741 4	0,18774605
Ursus arctos pruinosus MG066703 3	Paguma larvata PDDS11 2	0,18774718
Lycalopex sechurae KT448284 1	Crossarchus platycephalus C7R66 1	0,18774793
Procyon lotor AB462046 3	Panthera onca NC022842 1	0,18774895
Taxidea taxus NC020646 1	Proteles cristata T393 6	0,18775025
Ursus arctos GU573486 5	Prionailurus bengalensis NC028301 12	0,18775189
Viverricula indica NC025296 2	Galictis vittata T412 1	0,1877559
Mephitis mephitis NC020648 1	Arctocephalus pusillus NC008417 1	0,18775661
Zalophus californianus NC008416 1	Mungotictis decemlineata NC027828 1	0,18775716
Ursus arctos AP012576 6	Profelis aurata NC028299 1	0,18775873
Zalophus californianus NC008416 1	Galerella sanguinea T378 1	0,18776149
Ursus arctos GU573491 207	Leopardus colocolo NC028314 1	0,18776298
Mungos mungo MMC7 1	Cuon alpinus NC013445 3	0,1877635
Viverra tangalunga MH464792 1	Panthera leo NERO 19	0,18776512
Ailuropoda melanoleuca NC009492 5	Acinonyx jubatus NC005212 3	0,18776619
Ursus spelaeus EU327344 13	Canis aureus KT448274 1	0,18776637
Panthera leo spelaea KX258452 2	Canis aureus KT448274 1	0,18776932
Felis margarita NC028308 1	Arctocephalus forsteri NC004023 28	0,18777257
Ursus thibetanus laniger MH281753 2	Lycalopex sechurae KT448284 1	0,18777269
Ursus americanus JX196366 3	Paradoxurus hermaphroditus NLNC 1	0,18777525
Lycalopex sechurae KT448284 1	Leopardus jacobita NC028322 1	0,18777584
Lycalopex sechurae KT448284 1	Diplogale hosei MH464790 1	0,18777955
Felis chaus NC028307 1	Eumetopias jubatus NC004030 10	0,18778105
Mungos mungo/gambianus SRR77048	Martes americana NC020642 1	0,1877829
Martes zibellina NC011579 39	Urva brachyura KY117547 1	0,1877843
Vulpes vulpes NC008434 5	Melogale moschata V0735A 1	0,18778525
Xenogale naso C07XAR110 1	Arctocephalus forsteri KT693377 17	0,18778585
Vulpes lagopus NC026529 3	Cynictis penicillata T375 1	0,18778652
Vulpes lagopus NC026529 3	Galerella sanguinea T378 1	0,18778652
Urva semitorquata MH464789 1	Ailurus fulgens styani NC009691 1	0,18778661
Parahyaena brunnea NC038159 15	Martes flavigula NC012141 3	0,18778685
Helogale parvula SRR7637809 1	Canis latrans NC008093 7	0,18778709
Xenogale naso C07XAR110 1	Bassaricyon neblina SRX1097850 1	0,18778747
Eupleres goudotii D128 1	Aonyx cinerea NC035814 2	0,18778801
Urva javanica T413 1	Canis anthus NC027956 2	0,18778807
Helogale parvula SRR7637809 1	Aonyx cinerea NC035814 2	0,1877894
Mungos mungo/gambianus SRR77048	Aonyx cinerea NC035814 2	0,18778983
Nyctereutes procyonoides NC013700 3	Meles anakuma NC009677 1	0,18779024
Paradoxurus hermaphroditus NLNC 1	Lutra sumatrana NC035810 1	0,18779038
Helogale parvula SRR7637809 1	Arctocephalus pusillus NC008417 1	0,18779039
Vulpes zerda KJ603240 1	Mustela sibirica NC020637 6	0,18779099
Lynx pardinus NC028319 161	Tapirus terrestris T358	0,18779208
Melogale moschata NC020644 1	Bdeogale nigripes GLC15 1	0,18779214
Procyon lotor AB462046 3	Ichneumia albicauda T603 1	0,18779396
Suricata suricatta SSM10 1	Arctotherium sp NC030174 1	0,18779409
Galerella sanguinea T378 1	Arctocephalus townsendi NC008420 1	0,18779466
Lutra lutra LC050126 1	Chrysocyon brachyurus NC024172 1	0,18779548
Eumetopias jubatus NC004030 10	Ailuropoda melanoleuca NC009492 5	0,18779565
Vulpes vulpes NC008434 5	Mustela altaica NC021751 1	0,18779586
Lutra lutra LC050126 1	Arctictis binturong T605 2	0,18779626
Smilodon populator MF871700 1	Tapirus terrestris T358	0,18779628
Vulpes corsac NC023958 1	Mustela altaica NC021751 1	0,18779643
Ursus arctos isabellinus 18B5 2	Lutra lutra LC050126 1	0,18779682
Ursus maritimus GU573488 Svalbard	Lutra lutra LC050126 1	0,18779717
Catopuma temminckii NC027115 41	Arctocephalus townsendi NC008420 1	0,18779817
Ursus arctos isabellinus 18B5 2	Crossarchus platycephalus C7R66 1	0,18779829
Salanoia concolor D378 1	Arctotherium sp NC030174 1	0,18779851
Panthera pardus japonensis KJ866876	Mustela sibirica AP017394 11	0,18779853
Panthera pardus japonensis KJ866876	Mustela itatsi NC034330 19	0,18779871
Puma yagouaroundi NC028311 1	Arctocephalus forsteri KT693377 17	0,18779922
Vulpes ferrilata NC027935 1	Mustela nivalis T306 5	0,18779936
Lutra lutra LC050126 1	Crocuta crocuta NC020670 3	0,18779951
Ursus thibetanus mupinensis NC00875	Mungos mungo MMC7 1	0,18779972
Vulpes lagopus NC026529 3	Leopardus jacobita NC028322 1	0,18780039
Vulpes zerda KJ603240 1	Neovison vison NC020641 3	0,18780071
Martes foina NC020643 1	Diplogale hosei MH464790 1	0,18780117

Pusa hispida NC 008433 1	Martes flavigula NC012141 3	0,16743805
Mirounga leonina NC008422 1	Enhydra lutris NC009692 1	0,16744143
Martes zibellina NC011579 39	Cystophora cristata NC008427 1	0,16744907
Procyon lotor AB462049 4	Martes melampus NC009678 1	0,1674507
Taxidea taxus NC020646 1	Ailurus fulgens styani NC009691 1	0,16745079
Puma yagouaroundi NC028311 1	Leptonychotes weddellii NC008424 1	0,16746233
Ursus maritimus NC003428 31	Cystophora cristata NC008427 1	0,16746247
Leptonychotes weddellii NC008424 1	Caracal caracal NC028306 1	0,16746254
Phoca vitulina NC001325 1	Martes martes T302 3	0,1674674
Mirounga angustirostris SRR10331586 1	Arctotherium sp NC030174 1	0,16747066
Hydrurga leptonyx NC008425 1	Felis margarita NC028308 1	0,16747212
Lynx lynx NC027083 4	Hydrurga leptonyx NC008425 1	0,16747215
Cystophora cristata NC008427 1	Arctonyx collaris NC020645 1	0,1675158
Potos flavus T414 1	Melogale moschata KP726273 1	0,16751672
Martes americana NC020642 1	Bassaricyon neblina SRX1097850 1	0,16751942
Meles meles T303 3	Ailurus fulgens styani NC009691 1	0,16752061
Procyon lotor AB462049 4	Mustela nigripes NC024942 1	0,16752087
Procyon lotor AB462046 3	Martes zibellina NC011579 39	0,16752408
Phoca vitulina NC001325 1	Meles meles T303 3	0,16752703
Potos flavus T414 1	Ictonyx striatus T299 1	0,16752756
Leptonychotes weddellii NC008424 1	Felis nigripes NC028309 1	0,16752879
Leptonychotes weddellii NC008424 1	Leopardus geoffroyi NC028320 1	0,16753179
Monachus monachus NC044972 5	Arctonyx collaris NC020645 1	0,16754477
Phoca groenlandica NC008429 54	Gulo gulo NC009685 3	0,16756287
Viverra tangalunga MH464792 1	Erignathus barbatus NC008426 1	0,16758394
Mustela eversmannii NC028013 1	Hydrurga leptonyx NC008425 1	0,1675936
Lobodon carcinophaga NC008423 1	Ailurus fulgens NC011124 1	0,16759692
Potos flavus T414 1	Phoca largha NC008430 1	0,16760148
Pusa caspica NC008431 1	Melursus ursinus NC009970 2	0,16760743
Lobodon carcinophaga NC008423 1	Leopardus pardalis NC028315 1	0,16760787
Tremarctos ornatus NC009969 2	Monachus monachus NC044972 5	0,16761616
Pusa hispida NC 008433 1	Helarctos malayanus NC009968 2	0,16762063
Phoca fasciata NC008428 1	Gulo gulo NC009685 3	0,1676293
Pusa sibirica NC008432 2	Arctonyx collaris NC020645 1	0,16765146
Martes martes T302 3	Bassaricyon neblina SRX1097850 1	0,16766061
Phoca largha NC008430 1	Enhydra lutris NC009692 1	0,16766208
Mirounga leonina NC008422 1	Martes foina NC020643 1	0,16766341
Puma concolor NC016470 22	Leptonychotes weddellii NC008424 1	0,16766387
Leopardus pardalis T262 1	Cystophora cristata NC008427 1	0,16766788
Prionailurus rubiginosus NC028304 2	Lobodon carcinophaga NC008423 1	0,16767392
Ursus thibetanus formosanus NC009331 1	Cystophora cristata NC008427 1	0,16767451
Helarctos malayanus NC009968 2	Cystophora cristata NC008427 1	0,16767554
Ursus arctos GU573486 5	Phoca fasciata NC008428 1	0,16767746
Ictonyx striatus T299 1	Hydrurga leptonyx NC008425 1	0,16767806
Leptonychotes weddellii NC008424 1	Canis adustus KT448271 1	0,1676791
Ursus thibetanus laniger MH281753 2	Mirounga angustirostris SRR10331586 1	0,16768952
Hydrurga leptonyx NC008425 1	Helarctos malayanus NC009968 2	0,16769214
Meles meles T303 3	Halichoerus grypus NC001602 2	0,16769668
Phoca largha NC008430 1	Martes flavigula NC012141 3	0,167712
Meles anakuma NC009677 1	Cystophora cristata NC008427 1	0,16771646
Prionodon linsang ERR2391707 1	Hyaena hyaena NC020669 1	0,16772407
Pusa caspica NC008431 1	Martes martes T302 3	0,16772412
Martes pennanti NC020664 16	Cystophora cristata NC008427 1	0,16772421
Procyon lotor AB462049 4	Martes americana NC020642 1	0,16772546
Phoca vitulina NC001325 1	Meles leucurus NC039173 4	0,16772851
Phoca largha NC008430 1	Melogale moschata KP726273 1	0,16773047
Hydrurga leptonyx NC008425 1	Ailurus fulgens NC011124 1	0,16773134
Monachus schauinslandi NC008421 1	Ailurus fulgens styani NC009691 1	0,1677321
Monachus schauinslandi NC008421 1	Homotherium latidens MF871702 3	0,16773877
Ursus thibetanus thibetanus NC011118 4	Pusa sibirica NC008432 2	0,16774411
Ursus thibetanus thibetanus NC011118 4	Cystophora cristata NC008427 1	0,16774482
Gulo gulo NC009685 3	Cystophora cristata NC008427 1	0,16775656
Mellivora capensis T370 1	Meles anakuma NC009677 1	0,16777711
Pusa caspica NC008431 1	Arctonyx collaris NC020645 1	0,1677857
Mustela erminea T305 2	Bassaricyon neblina SRX1097850 1	0,1677884
Procyon lotor AB462046 3	Mustela sibirica NC020637 6	0,16779007
Melogale moschata NC020644 1	Cystophora cristata NC008427 1	0,16779117
Procyon lotor AB462046 3	Leptonychotes weddellii NC008424 1	0,16779165
Pusa hispida NC 008433 1	Enhydra lutris NC009692 1	0,16779432
Melursus ursinus NC009970 2	Halichoerus grypus NC001602 2	0,16779502
Mustela altaica NC021751 1	Leptonychotes weddellii NC008424 1	0,16779549
Phoca largha NC008430 1	Meles meles T303 3	0,16779618
Melogale moschata KP726273 1	Hydrurga leptonyx NC008425 1	0,16779773
Phoca largha NC008430 1	Martes zibellina NC011579 39	0,16779871
Phoca fasciata NC008428 1	Melogale moschata NC020644 1	0,16779914
Ursus maritimus GU573488 Svalbard	Cystophora cristata NC008427 1	0,16779917
Phoca fasciata NC008428 1	Aonyx cinerea NC035814 2	0,16780041
Ursus thibetanus thibetanus NC011118 4	Phoca largha NC008430 1	0,16782285
Melursus ursinus NC009970 2	Hydrurga leptonyx NC008425 1	0,16782659
Pusa sibirica NC008432 2	Martes americana NC020642 1	0,16785313
Mungos mungo/gambianus SRR7704821 1	Leptonychotes weddellii NC008424 1	0,16785619
Leptonychotes weddellii NC008424 1	Ailurus fulgens styani NC009691 1	0,16785702
Mirounga angustirostris SRR10331586 1	Enhydra lutris NC009692 1	0,16785744
Phoca groenlandica NC008429 54	Lutra sumatrana NC035810 1	0,16785897
Neovison vison NC020641 3	Mirounga leonina NC008422 1	0,16786303
Lobodon carcinophaga NC008423 1	Hemigalus derbyanus MH464791 1	0,16786328
Leptonychotes weddellii NC008424 1	Felis margarita NC028308 1	0,16786563
Lobodon carcinophaga NC008423 1	Fossa fossana D350 1	0,16786596
Ursus thibetanus mupinensis NC008753 2	Cystophora cristata NC008427 1	0,16786689
Pusa hispida NC 008433 1	Bassariscus sumichrasti SRX1099089 1	0,16786702
Ursus arctos GU573491 207	Leptonychotes weddellii NC008424 1	0,16786733
Neofelis nebulosa NC008450 3	Cynogale bennetti KY117544 1	0,16787507
Prionailurus bengalensis NC028301 12	Lobodon carcinophaga NC008423 1	0,16787566
Otocolobus manul NC028323 1	Lobodon carcinophaga NC008423 1	0,16787572
Lynx pardinus NC028319 161	Hydrurga leptonyx NC008425 1	0,16787614
Melursus ursinus NC009970 2	Erignathus barbatus NC008426 1	0,16787821
Lobodon carcinophaga NC008423 1	Leopardus geoffroyi NC028320 1	0,16787888
Ursus thibetanus laniger MH281753 2	Pusa hispida NC 008433 1	0,16788945
Pusa caspica NC008431 1	Gulo gulo NC009685 3	0,16789354
Spilogale putorius NC010497 1	Leptonychotes weddellii NC008424 1	0,16789806
Ursus spelaeus NC011112 8	Monachus schauinslandi NC008421 1	0,16789861
Phoca largha NC008430 1	Gulo gulo NC009685 3	0,16790618
Pusa sibirica NC008432 2	Meles leucurus NC039173 4	0,16791894

Mustela altaica NC021751 1	Mungotictis decemlineata NC027828 1	0,18780144
Tremarctos ornatus NC009969 2	Otocolobus manul NC028323 1	0,18780179
Spilogale putorius NC010497 1	Canis lupus familiaris NC002008 1231	0,18780288
Martes pennanti NC020664 16	Cryptoprocta ferox CFC13 1	0,18780342
Viverricula indica KX891751 1	Arctocephalus pusillus NC008417 1	0,18780358
Urva semitorquata MH464789 1	Chrysocyon brachyurus NC024172 1	0,1878044
Arctocephalus forsteri KT693377 17	Ailuropoda melanoleuca NC009492 5	0,18780443
Otocyon megalotis SAF1 2	Arctodus simus NC011116 1	0,1878057
Panthera pardus japonensis KJ866876	Bassariscus sumichrasti SRX1099089 1	0,18780619
Panthera tigris NC010642 35	Melogale moschata V0735A 1	0,18780848
Otaria byronia OTAB 1	Felis catus NC001700 2	0,18780856
Panthera leo spelaea KX258452 2	Ailurus fulgens styani NC009691 1	0,18780884
Otocyon megalotis SAF1 2	Leopardus guigna NC028321 1	0,18781066
Ursus thibetanus laniger MH281753 2	Bassaricyon neblina SRX1097850 1	0,18781188
Ursus arctos EU497665 29	Puma concolor NC016470 22	0,18781237
Ursus arctos isabellinus 18B5 2	Puma yagouaroundi NC028311 1	0,1878124
Ursus arctos GU573491 207	Caracal caracal NC028306 1	0,18781286
Ursus arctos GU573486 5	Paradoxurus jerdoni MH464793 1	0,18781342
Viverricula indica NC025296 2	Ursus arctos pruinosus MG066703 3	0,18781452
Zalophus wollebaeki SRR4431565 1	Galerella sanguinea T378 1	0,18781483
Ursus spelaeus EU327344 13	Bassaricyon neblina SRX1097850 1	0,18781516
Ursus arctos GU573486 5	Leptailurus serval NC028316 1	0,18781954
Ursus spelaeus NC011112 8	Paradoxurus jerdoni MH464793 1	0,18782176
Ursus spelaeus EU327344 13	Paradoxurus hermaphroditus NLNC 1	0,18782201
Ursus thibetanus thibetanus NC01111	Prionodon pardicolor NC024569 2	0,18782343
Ursus arctos AP012576 6	Paguma larvata PDDS11 2	0,18782356
Mustela eversmannii NC028013 1	Urva javanica/auropunctata NC006835	0,18782365
Urva javanica/auropunctata NC006835	Arctocephalus pusillus NC008417 1	0,18783486
Canis lupus familiaris NC002008 1231	Arctotherium sp NC030174 1	0,18783534
Zalophus californianus NC008416 1	Mephitis mephitis NC020648 1	0,18783678
Galerella sanguinea T378 1	Arctocephalus forsteri NC004023 28	0,18783948
Crocuta crocuta NC020670 3	Canis adustus KT448271 1	0,18784259
Mellivora capensis T370 1	Leptonychotes weddellii NC008424 1	0,18784736
Diplogale hosei MH464790 1	Callorhinus ursinus NC008415 1	0,18784952
Mustela sibirica AP017394 11	Bdeogale nigripes GLC15 1	0,18785298
Phocarctos hookeri NC008418 1	Bassaricyon neblina SRX1097850 1	0,18785448
Ursus arctos AP012576 6	Spilogale putorius NC010497 1	0,18785648
Vulpes lagopus NC026529 3	Mustela nigripes NC024942 1	0,18785764
Puma concolor NC016470 22	Tapirus terrestris T358	0,18785772
Viverra tangalunga MH464792 1	Galidictis fasciata DM333 1	0,18785778
Vulpes corsac NC023958 1	Mustela itatsi NC034330 19	0,18785778
Suricata suricatta SSM10 1	Martes pennanti NC020664 16	0,1878583
Vulpes ferrilata NC027935 1	Mustela itatsi NC034330 19	0,18785834
Viverricula indica KX891751 1	Arctocephalus australis MG023139 1	0,18785912
Nyctereutes procyonoides NC013700 3	Mustela kathiah NC023210 1	0,18785913
Urva semitorquata MH464789 1	Arctotherium sp NC030174 1	0,18785974
Prionodon linsang ERR2391707 1	Melogale moschata NC020644 1	0,187861
Pardofelis marmorata NLN3 2	Ailurus fulgens NC011124 1	0,18786189
Prionailurus planiceps KY682741 4	Conepatus chinga NC042596 1	0,18786374
Nyctereutes procyonoides NC013700 3	Melogale moschata NC020644 1	0,18786381
Ursus arctos pruinosus MG066703 3	Lutra lutra LC050126 1	0,1878649
Conepatus chinga NC042596 1	Catopuma badia NC028300 1	0,18786494
Martes americana NC020642 1	Hyaena hyaena NC020669 1	0,18786637
Ursus thibetanus mupinensis NC00875	Bdeogale nigripes GLC15 1	0,18786681
Urocyon littoralis catalinae KP129018	Lynx lynx NC027083 4	0,18786785
Nyctereutes procyonoides NC013700 3	Mustela nivalis T306 5	0,18786792
Melogale moschata NC020644 1	Galerella sanguinea T378 1	0,18786873
Meles leucurus NC039173 4	Lycaon pictus NC028427 2	0,18786891
Lynx lynx NC027083 4	Bassaricyon neblina SRX1097850 1	0,18786967
Phocarctos hookeri NC008418 1	Leopardus jacobita NC028322 1	0,18787137
Urocyon littoralis catalinae KP129018	Catopuma temminckii NC027115 41	0,18787147
Paradoxurus hermaphroditus NLNC 1	Arctocephalus pusillus NC008417 1	0,18787202
Panthera tigris amoyensis NC014770 2	Enhydra lutris NC009692 1	0,18787208
Lutrogale perspicillata NC035811 1	Catopuma badia NC028300 1	0,18787226
Mungos mungo/gambianus SRR77048	Chrysocyon brachyurus NC024172 1	0,18787272
Panthera uncia KP202269 1	Melogale moschata NC020644 1	0,18787448
Chrysocyon brachyurus NC024172 1	Chrotogale owstoni T607 1	0,18787491
Ursus thibetanus mupinensis NC00875	Galerella sanguinea T378 1	0,18787543
Prionodon pardicolor NC024569 2	Arctodus simus NC011116 1	0,18787605
Ursus arctos GU573486 5	Procyon lotor AB462049 4	0,18787703
Ursus maritimus GU573488 Svalbard	Lynx canadensis NC028313 1	0,18787786
Ursus arctos isabellinus 18B5 2	Leopardus pardalis NC028315 1	0,18787791
Ursus arctos pruinosus MG066703 3	Leopardus pardalis NC028315 1	0,18787852
Ursus arctos pruinosus MG066703 3	Leopardus pardalis T262 1	0,18787871
Ursus arctos AP012576 6	Lutra lutra NC011358 9	0,18788044
Ursus thibetanus mupinensis NC00875	Paradoxurus hermaphroditus NLNC 1	0,18788095
Ursus arctos pruinosus MG066703 3	Otaria byronia OTAB 1	0,18788263
Speothos venaticus C48 2	Leopardus guigna NC028321 1	0,18788405
Ursus thibetanus formosanus NC0093	Cryptoprocta ferox CFC13 1	0,18788528
Ursus spelaeus EU327344 13	Panthera onca KP202264 2	0,18788721
Ursus spelaeus NC011112 8	Panthera pardus japonensis KJ866876	0,18789048
Ichneumia albicauda T603 1	Ailuropoda melanoleuca NC009492 5	0,18789075
Ursus spelaeus NC011112 8	Prionailurus planiceps NC028312 6	0,18789251
Ursus spelaeus NC011112 8	Otocolobus manul NC028323 1	0,18789379
Eumetopias jubatus NC004030 10	Cynictis penicillata T375 1	0,18790044
Ursus americanus JX196366 3	Conepatus chinga NC042596 1	0,18790285
Urva javanica/auropunctata NC006835	Arctodus simus NC011116 1	0,18790461
Gulo gulo NC009685 3	Genetta genetta T297 1	0,187905
Ursus arctos AP012576 6	Galictis vittata T412 1	0,18790588
Panthera pardus NC010641 5	Mustela putorius NC020638 4	0,18790719
Spilogale putorius NC010497 1	Prionailurus bengalensis NC028301 12	0,18790729
Ursus americanus JX196366 3	Genetta genetta T297 1	0,18790904
Panthera leo NERO 19	Mustela nivalis T306 5	0,18790984
Suricata suricatta SSM10 1	Lutra lutra LC050126 1	0,18791342
Ursus arctos AP012576 6	Leopardus colocolo NC028314 1	0,18791386
Zalophus wollebaeki SRR4431565 1	Urva javanica/auropunctata NC006835	0,18791395
Paradoxurus jerdoni MH464793 1	Tapirus terrestris T358	0,18791594
Lontra canadensis SRR10409165 1	Urva semitorquata MH464789 1	0,18791817
Ursus americanus JX196366 3	Profelis aurata NC028299 1	0,18791845
Panthera tigris amoyensis NC014770 2	Gulo gulo NC009685 3	0,18791944
Mustela eversmannii NC028013 1	Urva brachyura KY117547 1	0,18791969
Lontra canadensis SRR10409165 1	Arctocephalus forsteri KT693377 17	0,18791993
Caracal caracal NC028306 1	Tapirus terrestris T358	0,18791997

Mellivora capensis T370 1	Ictonyx striatus T299 1	0,16792683
Phoca fasciata NC008428 1	Arctonyx collaris NC020645 1	0,16792853
Pusa hispida NC 008433 1	Melogale moschata KP726273 1	0,16793238
Pusa hispida NC 008433 1	Martes zibellina NC011579 39	0,16793349
Leopardus wiedii NC028318 1	Erignathus barbatus NC008426 1	0,16793604
Melogale moschata NC020644 1	Hydrurga leptonyx NC008425 1	0,16793698
Ursus thibetanus laniger MH281753 2	Cystophora cristata NC008427 1	0,16794387
Pusa hispida NC 008433 1	Homotherium latidens MF871702 3	0,16794889
Proteles cristata T393 6	Arctictis binturong T605 2	0,16796114
Halichoerus grypus NC001602 2	Arctonyx collaris NC020645 1	0,16796632
Melogale moschata KP726273 1	Cystophora cristata NC008427 1	0,16798727
Mustela itatsi NC034330 19	Cystophora cristata NC008427 1	0,16798903
Procyon lotor AB462049 4	Mustela frenata NC020640 1	0,1679918
Procyon lotor AB462049 4	Nasua nasua NC020647 1	0,16799271
Phoca fasciata NC008428 1	Meles meles T303 3	0,16799541
Galidictis fasciata DM333 1	Cynogale bennetti KY117544 1	0,16799982
Leopardus pardalis NC028315 1	Cystophora cristata NC008427 1	0,16800427
Proteles cristata T393 6	Chrotogale owstoni T607 1	0,16800976
Prionailurus planiceps NC028312 6	Lobodon carcinophaga NC008423 1	0,16801049
Prionailurus planiceps KY682741 4	Hydrurga leptonyx NC008425 1	0,16801062
Ursus thibetanus thibetanus NC011118 4	Pusa caspica NC008431 1	0,16801345
Ursus thibetanus mupinensis NC008753 2	Pusa hispida NC 008433 1	0,16801414
Ursus maritimus NC003428 31	Hydrurga leptonyx NC008425 1	0,16801704
Leopardus colocolo NC028314 1	Erignathus barbatus NC008426 1	0,16802083
Ursus americanus JX196366 3	Cystophora cristata NC008427 1	0,16802561
Lobodon carcinophaga NC008423 1	Canis aureus KT448274 1	0,16802797
Lutra lutra NC011358 9	Halichoerus grypus NC001602 2	0,16803437
Ursus spelaeus EU327344 13	Monachus monachus NC044972 5	0,1680494
Ursus spelaeus NC011112 8	Monachus monachus NC044972 5	0,16804954
Martes americana NC020642 1	Cystophora cristata NC008427 1	0,16805486
Mustela kathiah NC023210 1	Mirounga leonina NC008422 1	0,16805491
Procyon lotor AB462049 4	Enhydra lutris NC009692 1	0,16805649
Potos flavus T414 1	Melogale moschata NC020644 1	0,16805917
Phoca vitulina NC001325 1	Martes melampus NC009678 1	0,1680644
Phoca vitulina NC001325 1	Martes americana NC020642 1	0,16806807
Ursus arctos isabellinus 18B5 2	Cystophora cristata NC008427 1	0,16806847
Ursus arctos EU497665 29	Leptonychotes weddellii NC008424 1	0,16806938
Phoca largha NC008430 1	Bassariscus sumichrasti SRX1099089 1	0,16807109
Viverricula indica NC025296 2	Phoca fasciata NC008428 1	0,16807359
Parahyaena brunnea NC038159 15	Paradoxurus hermaphroditus NC039591 1	0,16807827
Hydrurga leptonyx NC008425 1	Felis catus NC001700 2	0,16807859
Ursus thibetanus laniger MH281753 2	Phoca largha NC008430 1	0,16808914
Ommatophoca rossii AY377287etc 1	Arctodus simus NC011116 1	0,16810727
Phoca vitulina NC001325 1	Gulo gulo NC009685 3	0,16810797
Mirounga leonina NC008422 1	Meles anakuma NC009677 1	0,16811793
Melogale moschata V0735A 1	Leptonychotes weddellii NC008424 1	0,16812068
Procyon lotor AB462046 3	Lutra sumatrana NC035810 1	0,16812404
Mungotictis decemlineata NC027828 1	Cynogale bennetti KY117544 1	0,16813281
Melogale moschata V0735A 1	Lobodon carcinophaga NC008423 1	0,16813561
Procyon lotor AB462046 3	Mustela nivalis T306 5	0,16813575
Viverricula indica NC025296 2	Mirounga leonina NC008422 1	0,16813888
Phoca vitulina NC001325 1	Bassariscus sumichrasti SRX1099089 1	0,16813929
Viverricula indica KX891751 1	Lobodon carcinophaga NC008423 1	0,16814218
Hydrurga leptonyx NC008425 1	Aonyx cinerea NC035814 2	0,16814493
Ursus thibetanus laniger MH281753 2	Pusa sibirica NC008432 2	0,16814511
Ursus thibetanus formosanus NC009331 1	Pusa caspica NC008431 1	0,16814543
Lobodon carcinophaga NC008423 1	Leopardus wiedii NC028318 1	0,16814715
Ursus arctos EU497665 29	Lobodon carcinophaga NC008423 1	0,16815138
Lobodon carcinophaga NC008423 1	Acinonyx jubatus NC005212 3	0,16816051
Pusa sibirica NC008432 2	Gulo gulo NC009685 3	0,16816321
Tremarctos ornatus NC009969 2	Pusa caspica NC008431 1	0,16818711
Pusa caspica NC008431 1	Enhydra lutris NC009692 1	0,16818798
Pusa caspica NC008431 1	Martes americana NC020642 1	0,16818979
Procyon lotor AB462049 4	Lutra lutra LC050126 1	0,16819122
Procyon lotor AB462049 4	Melogale moschata NC020644 1	0,16820021
Pusa hispida NC 008433 1	Mustela nigripes NC024942 1	0,16820055
Leptonychotes weddellii NC008424 1	Genetta servalina NC024568 2	0,16820289
Prionailurus bengalensis NC028301 12	Cystophora cristata NC008427 1	0,16820544
Ursus maritimus NC003428 31	Phoca fasciata NC008428 1	0,16820991
Pusa hispida NC 008433 1	Ictonyx striatus T299 1	0,16821387
Ursus thibetanus formosanus NC009331 1	Pusa hispida NC 008433 1	0,16822584
Monachus monachus NC044972 5	Ailurus fulgens NC011124 1	0,1682265
Ursus thibetanus thibetanus NC011118 4	Phoca vitulina NC001325 1	0,16822708
Lobodon carcinophaga NC008423 1	Helarctos malayanus NC009968 2	0,16823104
Pusa sibirica NC008432 2	Lutra lutra NC011358 9	0,16825385
Tremarctos ornatus NC009969 2	Cystophora cristata NC008427 1	0,168255
Pusa caspica NC008431 1	Meles anakuma NC009677 1	0,16825555
Pusa caspica NC008431 1	Mustela sibirica AP017394 11	0,16825789
Martes pennanti NC020664 16	Leptonychotes weddellii NC008424 1	0,16825964
Leptonychotes weddellii NC008424 1	Ailurus fulgens NC011124 1	0,16826087
Monachus schauinslandi NC008421 1	Lutra lutra NC011358 9	0,16826101
Potos flavus T414 1	Nasua nasua NC020647 1	0,16826257
Phoca fasciata NC008428 1	Martes martes T302 3	0,16826932
Ursus thibetanus mupinensis NC008753 2	Pusa caspica NC008431 1	0,16827019
Ursus thibetanus mupinensis NC008753 2	Pusa sibirica NC008432 2	0,16827025
Ursus thibetanus formosanus NC009331 1	Pusa sibirica NC008432 2	0,16827996
Ursus arctos isabellinus 18B5 2	Monachus schauinslandi NC008421 1	0,16828582
Viverricula indica KX891745 1	Monachus monachus NC044972 5	0,16829915
Ursus americanus JX196366 3	Erignathus barbatus NC008426 1	0,16830289
Spilogale putorius NC010497 1	Hydrurga leptonyx NC008425 1	0,16831166
Mellivora capensis T370 1	Meles meles T303 3	0,1683155
Ursus americanus JX196366 3	Hydrurga leptonyx NC008425 1	0,16831842
Halichoerus grypus NC001602 2	Gulo gulo NC009685 3	0,16832128
Procyon lotor AB462046 3	Enhydra lutris NC009692 1	0,1683256
Procyon lotor AB462046 3	Meles meles T303 3	0,1683259
Phoca groenlandica NC008429 54	Meles leucurus NC039173 4	0,16833189
Potos flavus T414 1	Aonyx cinerea NC035814 2	0,16833313
Neovison vison NC020641 3	Erignathus barbatus NC008426 1	0,16833434
Pusa hispida NC 008433 1	Mustela kathiah NC023210 1	0,1683345
Tremarctos ornatus NC009969 2	Phoca vitulina NC001325 1	0,1683346
Tremarctos ornatus NC009969 2	Mirounga angustirostris SRR10331586 1	0,1683348
Martes foina NC020643 1	Erignathus barbatus NC008426 1	0,16833642
Lobodon carcinophaga NC008423 1	Crossarchus platycephalus C7R66 1	0,16833663

Mustela kathiah NC023210 1	Bdeogale nigripes GLC15 1	0,1879207
Spilogale putorius NC010497 1	Ailuropoda melanoleuca NC009492 5	0,18792121
Vulpes zerda KJ603240 1	Martes americana NC020642 1	0,18792148
Tremarctos ornatus NC009969 2	Salanoia concolor D378 1	0,18792179
Urva brachyura KY117547 1	Canis latrans NC008093 7	0,18792322
Salanoia concolor D378 1	Mustela eversmannii NC028013 1	0,1879238
Prionodon linsang ERR2391707 1	Mustela itatsi NC034330 19	0,18792394
Procyon lotor AB462049 4	Xenogale naso C07XAR110 1	0,18792449
Vulpes zerda KJ603240 1	Mungotictis decemlineata NC027828 1	0,1879247
Mustela kathiah NC023210 1	Mungotictis decemlineata NC027828 1	0,18792619
Vulpes vulpes NC008434 5	Martes pennanti NC020664 16	0,18793024
Lycaon pictus NC028427 2	Helogale parvula SRR7637809 1	0,1879333
Vulpes corsac NC023958 1	Prionodon pardicolor NC024569 2	0,18793331
Felis nigripes NC028309 1	Arctocephalus australis MG023139 1	0,1879337
Panthera pardus japonensis KJ866876	Melogale moschata KP726273 1	0,18793374
Puma yagouaroundi NC028311 1	Arctocephalus gazella BK010918 1	0,18793474
Panthera uncia KP202269 1	Canis anthus NC027956 2	0,18793517
Procyon lotor AB462049 4	Diplogale hosei MH464790 1	0,18793534
Parahyaena brunnea NC038159 15	Meles meles T303 3	0,18793547
Panthera tigris amoyensis NC014770 2	Ailurus fulgens styani NC009691 1	0,18793648
Paradoxurus hermaphroditus NC03959	Nyctereutes procyonoides NC013700 3	0,18793681
Urocyon littoralis catalinae KP129018	Prionailurus rubiginosus NC028304 2	0,18793707
Ursus arctos GU573491 207	Procyon lotor AB462049 4	0,18793787
Ursus maritimus GU573488 Svalbard	Procyon lotor AB462046 3	0,18793818
Tremarctos ornatus NC009969 2	Paradoxurus jerdoni MH464793 1	0,1879383
Ommatophoca rossii AY377287etc 1	Nasua nasua NC020647 1	0,18793987
Prionodon linsang ERR2391707 1	Lycaon pictus NC028427 2	0,18794023
Otocyon megalotis SAF1 2	Melogale moschata NC020644 1	0,18794182
Panthera pardus japonensis KJ866876	Arctodus simus NC011116 1	0,18794259
Speothos venaticus C48 2	Mustela altaica NC021751 1	0,18794294
Otaria byronia OTAB 1	Felis nigripes NC028309 1	0,18794395
Potos flavus T414 1	Panthera leo spelaea KX258452 2	0,18794504
Ursus maritimus GU573488 Svalbard	Lynx pardinus NC028319 161	0,18794569
Ursus maritimus NC003428 31	Lynx pardinus NC028319 161	0,18794569
Ursus maritimus GU573488 Svalbard	Prionailurus planiceps KY682741 4	0,18794644
Ursus maritimus NC003428 31	Leopardus jacobita NC028322 1	0,18794644
Ursus arctos EU497665 29	Prionailurus planiceps KY682741 4	0,18794657
Prionailurus viverrinus NC028305 1	Mephitis mephitis NC020648 1	0,18795167
Panthera onca NC022842 1	Martes martes T302 3	0,18795477
Martes americana NC020642 1	Urva javanica/auropunctata NC006835	0,18795502
Ursus thibetanus thibetanus NC01111	Paradoxurus hermaphroditus NLNC 1	0,1879566
Ursus thibetanus formosanus NC0093	Pardofelis marmorata NLN3 2	0,1879569
Suricata suricatta SSM10 1	Canis aureus KT448274 1	0,18796162
Ursus arctos AP012576 6	Puma yagouaroundi NC028311 1	0,18796338
Arctocephalus forsteri NC004023 28	Ailuropoda melanoleuca NC009492 5	0,18796788
Helarctos malayanus NC009968 2	Galictis vittata T412 1	0,18796951
Martes flavigula NC012141 3	Eupleres goudotii D128 1	0,18797354
Ursus maritimus GU573488 Svalbard	Spilogale putorius NC010497 1	0,18797693
Leptailurus serval NC028316 1	Arctocephalus forsteri NC004023 28	0,18797776
Prionodon linsang ERR2391707 1	Lutra lutra LC050126 1	0,18797794
Paradoxurus hermaphroditus NLNC 1	Lutra lutra NC011358 9	0,18798082
Canis mesomelas KT448280 1	Atilax paludinosus T606 1	0,18798207
Nasua nasua NC020647 1	Conepatus chinga NC042596 1	0,18798403
Mustela eversmannii NC028013 1	Eupleres goudotii D128 1	0,1879857
Lontra canadensis SRR10409165 1	Xenogale naso C07XAR110 1	0,18798605
Mustela sibirica NC020637 6	Helogale parvula SRR7637809 1	0,18798643
Bdeogale nigripes GLC15 1	Arctocephalus townsendi NC008420 1	0,18798691
Eupleres goudotii D128 1	Bassariscus sumichrasti SRX1099089 1	0,18798712
Vulpes corsac NC023958 1	Martes melampus NC009678 1	0,18798722
Vulpes corsac NC023958 1	Cynictis penicillata T375 1	0,18798806
Prionodon linsang ERR2391707 1	Melogale moschata KP726273 1	0,18798843
Melogale moschata NC020644 1	Diplogale hosei MH464790 1	0,18798907
Melogale moschata V0735A 1	Canis latrans NC008093 7	0,18798949
Urocyon littoralis catalinae KP129018	Nandinia binotata NC024567 1	0,18798959
Nyctereutes procyonoides NC013700 3	Xenogale naso C07XAR110 1	0,18799001
Suricata suricatta SSM10 1	Nyctereutes procyonoides NC013700 3	0,1879901
Nyctereutes procyonoides NC013700 3	Ichneumia albicauda T603 1	0,1879905
Ictonyx striatus T299 1	Canis lupus chanco NC010340 4	0,1879911
Salanoia concolor D378 1	Phocarctos hookeri NC008418 1	0,1879912
Galidictis fasciata DM333 1	Bassariscus sumichrasti SRX1099089 1	0,1879915
Vulpes vulpes NC008434 5	Galidictis fasciata DM333 1	0,18799253
Mustela itatsi NC034330 19	Galidia elegans D146 1	0,18799289
Paradoxurus jerdoni MH464793 1	Martes americana NC020642 1	0,18799582
Panthera pardus japonensis KJ866876	Ailurus fulgens NC011124 1	0,18799601
Lutrogale perspicillata NC035811 1	Leopardus guigna NC028321 1	0,18799776
Ursus arctos isabellinus 18B5 2	Lutra lutra NC011358 9	0,18799834
Ursus arctos GU573491 207	Lutra lutra NC011358 9	0,18799867
Otocyon megalotis SAF1 2	Melogale moschata V0735A 1	0,18799891
Martes melampus NC009678 1	Hyaena hyaena NC020669 1	0,18799914
Ursus arctos GU573491 207	Lutrogale perspicillata NC035811 1	0,18799983
Otocyon megalotis SAF1 2	Mustela putorius NC020638 4	0,18800072
Profelis aurata NC028299 1	Arctocephalus australis MG023139 1	0,18800128
Ichneumia albicauda T603 1	Arctocephalus gazella BK010918 1	0,18800165
Vulpes zerda KJ603240 1	Mustela nivalis T306 5	0,18800181
Martes zibellina NC011579 39	Lycalopex sechurae KT448284 1	0,18800192
Melogale moschata KP726273 1	Lycalopex sechurae KT448284 1	0,18800212
Ursus maritimus NC003428 31	Procyon lotor AB462049 4	0,18800528
Ursus arctos GU573486 5	Lutra lutra NC011358 9	0,18800545
Procyon lotor AB462049 4	Panthera onca KP202264 2	0,18800624
Martes americana NC020642 1	Canis mesomelas KT448280 1	0,1880075
Martes pennanti NC020664 16	Hyaena hyaena NC020669 1	0,1880092
Panthera leo spelaea KX258452 2	Mustela nigripes NC024942 1	0,18801065
Ursus arctos isabellinus 18B5 2	Leopardus jacobita NC028322 1	0,18801321
Ursus thibetanus thibetanus NC01111	Cynictis penicillata T375 1	0,18801396
Tremarctos ornatus NC009969 2	Mephitis mephitis NC020648 1	0,18801644
Eupleres goudotii D128 1	Canis lupus familiaris NC002008 1231	0,18801913
Ursus arctos GU573486 5	Lynx pardinus NC028319 161	0,18801923
Panthera tigris NC010642 35	Otocyon megalotis SAF1 2	0,18801962
Ursus spelaeus EU327344 13	Felis nigripes NC028309 1	0,18802209
Hemigalus derbyanus MH464791 1	Arctocephalus townsendi NC008420 1	0,1880229
Galictis vittata T412 1	Felis nigripes NC028309 1	0,18802448
Leopardus geoffroyi NC028320 1	Ailuropoda melanoleuca NC009492 5	0,1880267
Melogale moschata NC020644 1	Urva javanica/auropunctata NC006835	0,18803059

Leptonychotes weddellii NC008424 1	Catopuma badia NC028300 1	0,16833681
Prionailurus planiceps NC028312 6	Leptonychotes weddellii NC008424 1	0,168337
Pusa hispida NC 008433 1	Martes americana NC020642 1	0,16833711
Melogale moschata V0735A 1	Hydrurga leptonyx NC008425 1	0,16833736
Leptonychotes weddellii NC008424 1	Leopardus guigna NC028321 1	0,16833988
Pusa hispida NC 008433 1	Aonyx cinerea NC035814 2	0,16834392
Procyon lotor AB462049 4	Martes foina NC020643 1	0,1683443
Procyon lotor AB462046 3	Martes foina NC020643 1	0,16834454
Pusa caspica NC008431 1	Helarctos malayanus NC009968 2	0,16834795
Ursus thibetanus laniger MH281753 2	Erignathus barbatus NC008426 1	0,16834861
Ursus thibetanus formosanus NC009331 1	Erignathus barbatus NC008426 1	0,16834868
Ursus arctos AP012576 6	Leptonychotes weddellii NC008424 1	0,168354
Ursus arctos GU573486 5	Phoca largha NC008430 1	0,16835496
Monachus monachus NC044972 5	Meles meles T303 3	0,16835515
Proteles cristata T393 6	Prionodon pardicolor NC024569 2	0,16835682
Ursus arctos GU573486 5	Hydrurga leptonyx NC008425 1	0,168359
Mustela nigripes NC024942 1	Halichoerus grypus NC001602 2	0,16837311
Hydrurga leptonyx NC008425 1	Civettictis civetta NC033378 1	0,16838512
Procyon lotor AB462046 3	Mustela eversmannii NC028013 1	0,16839583
Procyon lotor AB462046 3	Martes americana NC020642 1	0,16839871
Mustela altaica NC021751 1	Ailurus fulgens styani NC009691 1	0,16839932
Salanoia concolor D378 1	Cynogale bennetti KY117544 1	0,16840276
Potos flavus T414 1	Phoca groenlandica NC008429 54	0,16840449
Viverra zibetha T609 1	Leptonychotes weddellii NC008424 1	0,1684045
Viverricula indica KX891745 1	Phoca fasciata NC008428 1	0,16840617
Leopardus pardalis T262 1	Erignathus barbatus NC008426 1	0,16840735
Phoca largha NC008430 1	Melogale moschata NC020644 1	0,16840985
Ursus maritimus GU573488 Svalbard	Phoca fasciata NC008428 1	0,16841197
Ursus thibetanus laniger MH281753 2	Pusa caspica NC008431 1	0,16841449
Proteles cristata T393 6	Paradoxurus hermaphroditus NLNC 1	0,1684161
Ursus thibetanus mupinensis NC008753 2	Phoca largha NC008430 1	0,16841626
Ursus thibetanus thibetanus NC011118 4	Mirounga angustirostris SRR10331586 1	0,16841914
Ursus arctos isabellinus 18B5 2	Lobodon carcinophaga NC008423 1	0,16842014
Mustela frenata NC020640 1	Monachus monachus NC044972 5	0,16842306
Ursus thibetanus formosanus NC009331 1	Phoca largha NC008430 1	0,16842631
Ursus spelaeus EU327344 13	Phoca largha NC008430 1	0,16842894
Viverricula indica KX891751 1	Monachus monachus NC044972 5	0,16843335
Parahyaena brunnea NC038159 15	Diplogale hosei MH464790 1	0,16843526
Tremarctos ornatus NC009969 2	Mirounga leonina NC008422 1	0,16845655
Hydrurga leptonyx NC008425 1	Chrotogale owstoni T607 1	0,16845845
Meles anakuma NC009677 1	Ailurus fulgens styani NC009691 1	0,16846111
Mirounga leonina NC008422 1	Ailurus fulgens styani NC009691 1	0,16846411
Procyon lotor AB462046 3	Melogale moschata NC020644 1	0,16846949
Viverricula indica KX891745 1	Erignathus barbatus NC008426 1	0,1684714
Prionodon linsang ERR2391707 1	Parahyaena brunnea NC038159 15	0,16847357
Phoca fasciata NC008428 1	Bassariscus sumichrasti SRX1099089 1	0,16847581
Potos flavus T414 1	Phoca vitulina NC001325 1	0,16847694
Monachus schauinslandi NC008421 1	Bassariscus sumichrasti SRX1099089 1	0,16847996
Lobodon carcinophaga NC008423 1	Catopuma badia NC028300 1	0,16848205
Poecilogale albinucha T602 1	Hydrurga leptonyx NC008425 1	0,16848353
Ursus thibetanus laniger MH281753 2	Mirounga leonina NC008422 1	0,16848452
Ursus spelaeus NC011112 8	Mirounga leonina NC008422 1	0,16848627
Paradoxurus hermaphroditus NC039591 1	Crocuta crocuta NC020670 3	0,1684908
Ursus spelaeus NC011112 8	Phoca groenlandica NC008429 54	0,1684925
Ursus americanus JX196366 3	Leptonychotes weddellii NC008424 1	0,16850454
Ursus americanus JX196366 3	Lobodon carcinophaga NC008423 1	0,16852072
Pusa sibirica NC008432 2	Enhydra lutris NC009692 1	0,16852489
Mustela kathiah NC023210 1	Erignathus barbatus NC008426 1	0,16852614
Mustela putorius NC020638 4	Cystophora cristata NC008427 1	0,16852726
Pusa caspica NC008431 1	Mustela sibirica NC020637 6	0,16852731
Phoca groenlandica NC008429 54	Lutra lutra LC050126 1	0,16852956
Melogale moschata NC020644 1	Erignathus barbatus NC008426 1	0,16853014
Phoca largha NC008430 1	Meles leucurus NC039173 4	0,16853637
Tremarctos ornatus NC009969 2	Phoca largha NC008430 1	0,16853701
Procyon lotor AB462046 3	Poecilogale albinucha T602 1	0,1685371
Pusa hispida NC 008433 1	Mustela putorius NC020638 4	0,1685371
Procyon lotor AB462046 3	Lobodon carcinophaga NC008423 1	0,16854385
Pusa hispida NC 008433 1	Neovison vison NC020641 3	0,16854478
Lobodon carcinophaga NC008423 1	Leopardus tigrinus NC028317 1	0,16855067
Ursus spelaeus EU327344 13	Mirounga leonina NC008422 1	0,16855364
Ursus thibetanus thibetanus NC011118 4	Mirounga leonina NC008422 1	0,16855371
Ursus thibetanus mupinensis NC008753 2	Mirounga angustirostris SRR10331586 1	0,16855574
Tremarctos ornatus NC009969 2	Halichoerus grypus NC001602 2	0,16857743
Melogale moschata KP726273 1	Erignathus barbatus NC008426 1	0,16859256
Pusa sibirica NC008432 2	Martes zibellina NC011579 39	0,16859402
Mustela eversmannii NC028013 1	Cystophora cristata NC008427 1	0,16859426
Pusa caspica NC008431 1	Mustela nigripes NC024942 1	0,16859502
Procyon lotor AB462046 3	Mustela sibirica AP017394 11	0,16859771
Procyon lotor AB462046 3	Nasua nasua NC020647 1	0,16859832
Pusa hispida NC 008433 1	Meles anakuma NC009677 1	0,1686009
Pusa hispida NC 008433 1	Mustela sibirica AP017394 11	0,16860449
Phoca fasciata NC008428 1	Fossa fossana D350 1	0,16860579
Mungos mungo/gambianus SRR7704821 1	Hydrurga leptonyx NC008425 1	0,16860686
Neovison vison NC020641 3	Ailurus fulgens styani NC009691 1	0,16860873
Procyon lotor AB462046 3	Hydrurga leptonyx NC008425 1	0,16861105
Puma yagouaroundi NC028311 1	Lobodon carcinophaga NC008423 1	0,16861658
Profelis aurata NC028299 1	Hydrurga leptonyx NC008425 1	0,16861689
Ursus thibetanus mupinensis NC008753 2	Phoca vitulina NC001325 1	0,16861865
Ursus thibetanus formosanus NC009331 1	Mirounga leonina NC008422 1	0,16861871
Ursus maritimus GU573488 Svalbard	Hydrurga leptonyx NC008425 1	0,16862312
Pusa sibirica NC008432 2	Homotherium latidens MF871702 3	0,16862553
Ursus spelaeus EU327344 13	Phoca groenlandica NC008429 54	0,16862714
Hemigalus derbyanus MH464791 1	Erignathus barbatus NC008426 1	0,1686302
Ursus thibetanus formosanus NC009331 1	Mirounga angustirostris SRR10331586 1	0,1686321
Neovison vison NC020641 3	Monachus monachus NC044972 5	0,16863564
Meles anakuma NC009677 1	Erignathus barbatus NC008426 1	0,16865763
Procyon lotor AB462049 4	Mustela eversmannii NC028013 1	0,16866498
Monachus schauinslandi NC008421 1	Enhydra lutris NC009692 1	0,1686659
Phoca fasciata NC008428 1	Meles leucurus NC039173 4	0,16866811
Phoca vitulina NC001325 1	Melogale moschata KP726273 1	0,1686732
Ursus arctos EU497665 29	Cystophora cristata NC008427 1	0,16867462
Ursus arctos GU573491 207	Erignathus barbatus NC008426 1	0,16867493
Prionailurus bengalensis NC028301 12	Erignathus barbatus NC008426 1	0,16867545

Eumetopias jubatus NC004030 10	Diplogale hosei MH464790 1	0,18803133
Panthera leo NERO 19	Canis anthus NC027956 2	0,188033
Ursus arctos AP012576 6	Leopardus geoffroyi NC028320 1	0,18803413
Ursus spelaeus EU327344 13	Lycalopex sechurae KT448284 1	0,18803523
Ursus arctos isabellinus 18B5 2	Canis lupus familiaris NC002008 1231	0,18804061
Catopuma temminckii NC027115 41	Arctocephalus forsteri NC004023 28	0,18804176
Felis silvestris lybica KP202275 4	Arctocephalus forsteri NC004023 28	0,18804274
Mungos mungo/gambianus SRR77048	Canis mesomelas KT448280 1	0,18804342
Ichneumia albicauda T603 1	Arctocephalus forsteri NC004023 28	0,18804379
Parahyaena brunnea NC038159 15	Gulo gulo NC009685 3	0,18804657
Lycalopex sechurae KT448284 1	Urva javanica T413 1	0,18804818
Paradoxurus hermaphroditus NLNC 1	Tapirus terrestris T358	0,18804881
Lutrogale perspicillata NC035811 1	Civettictis civetta NC033378 1	0,1880495
Panthera pardus NC010641 5	Arctodus simus NC011116 1	0,18804967
Lutra lutra LC050126 1	Canis anthus NC027956 2	0,18805134
Panthera pardus NC010641 5	Mustela nivalis T306 5	0,18805152
Meles anakuma NC009677 1	Cynictis penicillata T375 1	0,18805292
Meles anakuma NC009677 1	Bdeogale nigripes GLC15 1	0,18805304
Viverra zibetha T609 1	Tapirus terrestris T358	0,18805304
Vulpes zerda KJ603240 1	Hemigalus derbyanus MH464791 1	0,18805519
Vulpes corsac NC023958 1	Melogale moschata V0735A 1	0,18805523
Vulpes lagopus NC026529 3	Urva semitorquata MH464789 1	0,18805654
Nyctereutes procyonoides NC013700 3	Cynictis penicillata T375 1	0,18805833
Galidia elegans D146 1	Bassariscus sumichrasti SRX1099089 1	0,18805841
Vulpes lagopus NC026529 3	Mustela erminea T305 2	0,18806104
Galidictis fasciata DM333 1	Arctocephalus australis MG023139 1	0,18806113
Vulpes vulpes NC008434 5	Bassariscus sumichrasti SRX1099089 1	0,18806134
Mungotictis decemlineata NC027828 1	Arctocephalus gazella BK010918 1	0,18806142
Profelis aurata NC028299 1	Conepatus chinga NC042596 1	0,18806238
Mephitis mephitis NC020648 1	Eumetopias jubatus NC004030 10	0,18806341
Otaria byronia OTAB 1	Cynictis penicillata T375 1	0,18806383
Vulpes corsac NC023958 1	Melogale moschata NC020644 1	0,18806468
Vulpes lagopus NC026529 3	Paradoxurus hermaphroditus NLNC 1	0,18806477
Poecilogale albinucha T602 1	Urva semitorquata MH464789 1	0,18806639
Cryptoprocta ferox CFC13 1	Arctonyx collaris NC020645 1	0,18806665
Otocyon megalotis SAF1 2	Martes zibellina NC011579 39	0,1880669
Ursus arctos GU573491 207	Crossarchus platycephalus C7R66 1	0,18806775
Lycaon pictus NC028427 2	Xenogale naso C07XAR110 1	0,18806823
Suricata suricatta SSM10 1	Lycaon pictus NC028427 2	0,18806864
Galerella sanguinea T378 1	Ailurus fulgens styani NC009691 1	0,18806891
Felis chaus NC028307 1	Arctocephalus gazella BK010918 1	0,18806973
Urocyon cinereoargenteus NC026723 2	Lynx canadensis NC028313 1	0,18807058
Urocyon littoralis catalinae KP129018	Leopardus wiedii NC028318 1	0,18807104
Ursus maritimus NC003428 31	Salanoia concolor D378 1	0,18807107
Prionodon pardicolor NC024569 2	Nyctereutes procyonoides NC013700 3	0,1880715
Paguma larvata PDDS11 2	Martes martes T302 3	0,18807192
Tremarctos ornatus NC009969 2	Lycaon pictus NC028427 2	0,18807207
Meles leucurus NC039173 4	Chrysocyon brachyurus NC024172 1	0,18807563
Panthera tigris NC010642 35	Martes zibellina NC011579 39	0,18807879
Ursus thibetanus laniger MH281753 2	Helogale parvula SRR7637809 1	0,18807915
Ursus arctos EU497665 29	Panthera pardus japonensis KJ866876	0,18807986
Ursus thibetanus mupinensis NC00875	Panthera onca KP202264 2	0,18808015
Proteles cristata T393 6	Martes martes T302 3	0,18808079
Ursus arctos GU573491 207	Leopardus jacobita NC028322 1	0,18808107
Ursus arctos EU497665 29	Leopardus jacobita NC028322 1	0,18808109
Ursus maritimus GU573488 Svalbard	Leopardus jacobita NC028322 1	0,1880811
Ursus thibetanus thibetanus NC01111	Helogale parvula SRR7637809 1	0,18808152
Melursus ursinus NC009970 2	Lontra canadensis SRR10409165 1	0,18808156
Ursus arctos EU497665 29	Prionailurus bengalensis NC028301 12	0,18808234
Ursus arctos EU497665 29	Leptailurus serval NC028316 1	0,18808253
Ursus arctos isabellinus 18B5 2	Leopardus guigna NC028321 1	0,18808499
Parahyaena brunnea NC038159 15	Ictonyx striatus T299 1	0,18808641
Helarctos malayanus NC009968 2	Cryptoprocta ferox CFC13 1	0,18808672
Ursus spelaeus NC011112 8	Procyon lotor AB462046 3	0,18808699
Halichoerus grypus NC001602 2	Cynogale bennetti KY117544 1	0,18808727
Ursus arctos GU573486 5	Puma concolor NC016470 22	0,18808803
Zalophus californianus NC008416 1	Xenogale naso C07XAR110 1	0,18808819
Galidictis fasciata DM333 1	Canis aureus KT448274 1	0,18808995
Ursus arctos GU573486 5	Prionailurus bengalensis CKM45 20	0,18809164
Melursus ursinus NC009970 2	Genetta genetta T297 1	0,18809222
Galidictis fasciata DM333 1	Canis lupus familiaris NC002008 1231	0,18809378
Aonyx cinerea NC035814 2	Acinonyx jubatus NC005212 3	0,18809427
Panthera leo NERO 19	Lutra lutra NC011358 9	0,18809427
Paradoxurus hermaphroditus NC03959	Helarctos malayanus NC009968 2	0,18809481
Vulpes zerda KJ603240 1	Gulo gulo NC009685 3	0,18810368
Prionodon linsang ERR2391707 1	Enhydra lutris NC009692 1	0,18811385
Conepatus chinga NC042596 1	Aonyx cinerea NC035814 2	0,18811579
Panthera leo NERO 19	Neovison vison NC020641 3	0,18811581
Ursus arctos pruinosus MG066703 3	Spilogale putorius NC010497 1	0,18811817
Vulpes vulpes NC008434 5	Lutra lutra LC050126 1	0,18811974
Melogale moschata V0735A 1	Bdeogale nigripes GLC15 1	0,18812083
Panthera pardus NC010641 5	Neovison vison NC020641 3	0,18812105
Vulpes vulpes NC008434 5	Bdeogale nigripes GLC15 1	0,1881227
Lontra canadensis SRR10409165 1	Arctocephalus gazella BK010918 1	0,1881232
Tremarctos ornatus NC009969 2	Galidictis fasciata DM333 1	0,18812382
Urocyon cinereoargenteus NC026723 2	Martes melampus NC009678 1	0,18812406
Atilax paludinosus T606 1	Arctocephalus gazella BK010918 1	0,18812463
Martes martes T302 3	Helogale parvula SRR7637809 1	0,18812609
Bassaricyon neblina SRX1097850 1	Ailurus fulgens NC011124 1	0,18812855
Enhydra lutris NC009692 1	Canis mesomelas KT448280 1	0,18812943
Martes melampus NC009678 1	Lycalopex sechurae KT448284 1	0,18812943
Paguma larvata PDDS11 2	Martes americana NC020642 1	0,18813001
Lontra canadensis SRR10409165 1	Galerella sanguinea T378 1	0,18813039
Galerella sanguinea T378 1	Tapirus terrestris T358	0,1881308
Ursus thibetanus thibetanus NC01111	Panthera pardus NC010641 5	0,18813109
Suricata suricatta SSM10 1	Mustela nivalis T306 5	0,18813194
Vulpes zerda KJ603240 1	Arctodus simus NC011116 1	0,18813349
Otocyon megalotis SAF1 2	Enhydra lutris NC009692 1	0,18813357
Ursus arctos pruinosus MG066703 3	Lutra lutra NC011358 9	0,18813376
Neophoca cinerea NC008419 1	Conepatus chinga NC042596 1	0,18813387
Martes foina NC020643 1	Helogale parvula SRR7637809 1	0,18813497
Panthera uncia KP202269 1	Melogale moschata KP726273 1	0,1881355
Ictonyx striatus T299 1	Helogale parvula SRR7637809 1	0,18813567

Hydrurga leptonyx NC008425 1	Felis chaus NC028307 1	0,16868474
Phoca groenlandica NC008429 54	Helarctos malayanus NC009968 2	0,16869371
Ursus arctos GU573491 207	Monachus monachus NC044972 5	0,16871022
Erignathus barbatus NC008426 1	Enhydra lutris NC009692 1	0,1687236
Mirounga leonina NC008422 1	Ailurus fulgens NC011124 1	0,16873333
Mungos mungo MMC7 1	Leptonychotes weddellii NC008424 1	0,16873366
Monachus schauinslandi NC008421 1	Meles meles T303 3	0,1687362
Phoca fasciata NC008428 1	Martes zibellina NC011579 39	0,16873644
Phoca groenlandica NC008429 54	Arctonyx collaris NC020645 1	0,16873685
Phoca largha NC008430 1	Mustela nigripes NC024942 1	0,16874149
Phoca vitulina NC001325 1	Mustela nigripes NC024942 1	0,16874157
Homotherium latidens MF871702 3	Cystophora cristata NC008427 1	0,16874234
Leopardus pardalis NC028315 1	Erignathus barbatus NC008426 1	0,16874364
Pusa hispida NC 008433 1	Martes pennanti NC020664 16	0,16874628
Prionailurus planiceps KY682741 4	Lobodon carcinophaga NC008423 1	0,16875133
Ursus arctos GU573486 5	Lobodon carcinophaga NC008423 1	0,16876353
Meles leucurus NC039173 4	Halichoerus grypus NC001602 2	0,16877417
Monachus schauinslandi NC008421 1	Martes melampus NC009678 1	0,16880196
Mustela sibirica AP017394 11	Monachus schauinslandi NC008421 1	0,16880624
Helarctos malayanus NC009968 2	Halichoerus grypus NC001602 2	0,16880789
Phoca vitulina NC001325 1	Martes zibellina NC011579 39	0,16880891
Ursus arctos EU497665 29	Phoca fasciata NC008428 1	0,16881615
Lobodon carcinophaga NC008423 1	Genetta servalina NC024568 2	0,16881764
Hydrurga leptonyx NC008425 1	Civettictis civetta GLC19 1	0,16881923
Ursus maritimus NC003428 31	Phoca largha NC008430 1	0,16882032
Phoca largha NC008430 1	Helarctos malayanus NC009968 2	0,1688336
Paradoxurus jerdoni MH464793 1	Crocuta crocuta NC020670 3	0,16883609
Pusa sibirica NC008432 2	Martes flavigula NC012141 3	0,16884397
Melogale moschata KP726273 1	Halichoerus grypus NC001602 2	0,16884589
Martes foina NC020643 1	Halichoerus grypus NC001602 2	0,16885939
Pusa caspica NC008431 1	Lutra lutra NC011358 9	0,1688596
Viverra tangalunga MH464792 1	Mirounga leonina NC008422 1	0,16886312
Pusa caspica NC008431 1	Martes zibellina NC011579 39	0,16886324
Taxidea taxus NC020646 1	Ailurus fulgens NC011124 1	0,16886485
Pusa caspica NC008431 1	Bassariscus sumichrasti SRX1099089 1	0,16886736
Phoca fasciata NC008428 1	Martes americana NC020642 1	0,16887088
Mirounga leonina NC008422 1	Hemigalus derbyanus MH464791 1	0,1688765
Ursus arctos GU573491 207	Cystophora cristata NC008427 1	0,16887656
Phoca groenlandica NC008429 54	Martes foina NC020643 1	0,1688842
Monachus schauinslandi NC008421 1	Arctotherium sp NC030174 1	0,16888577
Pusa sibirica NC008432 2	Helarctos malayanus NC009968 2	0,16888662
Ursus arctos AP012576 6	Cystophora cristata NC008427 1	0,16889179
Ursus arctos EU497665 29	Hydrurga leptonyx NC008425 1	0,16889249
Ursus thibetanus laniger MH281753 2	Phoca vitulina NC001325 1	0,16889755
Ursus spelaeus NC011112 8	Phoca largha NC008430 1	0,16890042
Phoca vitulina NC001325 1	Martes flavigula NC012141 3	0,16892362
Hydrurga leptonyx NC008425 1	Conepatus chinga NC042596 1	0,16893084
Procyon lotor AB462049 4	Mustela sibirica NC020637 6	0,16893412
Bassariscus sumichrasti SRX1099089 1	Aonyx cinerea NC035814 2	0,16893587
Leptonychotes weddellii NC008424 1	Diplogale hosei MH464790 1	0,16893734
Monachus schauinslandi NC008421 1	Ailurus fulgens NC011124 1	0,16894434
Ursus arctos GU573486 5	Erignathus barbatus NC008426 1	0,16895062
Phoca vitulina NC001325 1	Neovison vison NC020641 3	0,16895252
Ursus arctos GU573491 207	Lobodon carcinophaga NC008423 1	0,16895884
Ursus maritimus GU573488 Svalbard	Lobodon carcinophaga NC008423 1	0,1689591
Pusa sibirica NC008432 2	Leopardus colocolo NC028314 1	0,1689658
Phoca vitulina NC001325 1	Homotherium latidens MF871702 3	0,16897495
Viverricula indica NC025296 2	Monachus monachus NC044972 5	0,16897592
Leptonychotes weddellii NC008424 1	Conepatus chinga NC042596 1	0,1689907
Pusa caspica NC008431 1	Melogale moschata KP726273 1	0,16899723
Procyon lotor AB462049 4	Meles meles T303 3	0,16899878
Nandinia binotata NC024567 1	Mustela erminea T305 2	0,16899952
Pusa sibirica NC008432 2	Bassariscus sumichrasti SRX1099089 1	0,16900182
Procyon lotor AB462049 4	Melogale moschata KP726273 1	0,16900332
Mustela nivalis T306 5	Erignathus barbatus NC008426 1	0,16900697
Pusa hispida NC 008433 1	Mustela sibirica NC020637 6	0,16900845
Leptonychotes weddellii NC008424 1	Felis chaus NC028307 1	0,16901056
Ursus arctos isabellinus 18B5 2	Erignathus barbatus NC008426 1	0,1690114
Viverricula indica KX891751 1	Phoca fasciata NC008428 1	0,16901219
Monachus schauinslandi NC008421 1	Martes martes T302 3	0,16901505
Ursus arctos GU573486 5	Pusa sibirica NC008432 2	0,16901696
Ursus arctos GU573486 5	Pusa hispida NC 008433 1	0,16903096
Ursus thibetanus formosanus NC009331 1	Phoca vitulina NC001325 1	0,16903271
Prionodon pardicolor NC024569 2	Crocuta crocuta NC020670 3	0,16903403
Ursus americanus JX196366 3	Phoca fasciata NC008428 1	0,16904345
Monachus monachus NC044972 5	Bassariscus sumichrasti SRX1099089 1	0,16904439
Pusa sibirica NC008432 2	Melogale moschata KP726273 1	0,16906469
Arctonyx collaris NC020645 1	Ailurus fulgens NC011124 1	0,16906951
Pusa sibirica NC008432 2	Potos flavus T414 1	0,16907017
Pusa caspica NC008431 1	Potos flavus T414 1	0,16907021
Pusa sibirica NC008432 2	Aonyx cinerea NC035814 2	0,16907062
Pusa caspica NC008431 1	Melogale moschata NC020644 1	0,16907087
Pusa sibirica NC008432 2	Melogale moschata NC020644 1	0,16907111
Phoca fasciata NC008428 1	Melogale moschata KP726273 1	0,16907284
Prionailurus bengalensis NC028301 12	Mirounga leonina NC008422 1	0,16907713
Mustela nivalis T306 5	Ailurus fulgens styani NC009691 1	0,16907763
Viverricula indica KX891745 1	Mirounga leonina NC008422 1	0,16907766
Phoca largha NC008430 1	Mustela putorius NC020638 4	0,16907781
Phoca vitulina NC001325 1	Mustela putorius NC020638 4	0,16907793
Phoca largha NC008430 1	Martes pennanti NC020664 16	0,16908342
Phoca largha NC008430 1	Aonyx cinerea NC035814 2	0,16908413
Ursus arctos pruinosus MG066703 3	Cystophora cristata NC008427 1	0,16908492
Hydrurga leptonyx NC008425 1	Catopuma temminckii NC027115 41	0,16908763
Pusa hispida NC 008433 1	Catopuma badia NC028300 1	0,16908884
Ursus arctos GU573491 207	Hydrurga leptonyx NC008425 1	0,16909422
Viverricula indica NC025296 2	Mirounga angustirostris SRR10331586 1	0,16909714
Monachus monachus NC044972 5	Hemigalus derbyanus MH464791 1	0,1690973
Mustela putorius NC020638 4	Halichoerus grypus NC001602 2	0,16911164
Martes americana NC020642 1	Halichoerus grypus NC001602 2	0,16911611
Ursus thibetanus mupinensis NC008753 2	Halichoerus grypus NC001602 2	0,16913164
Ursus thibetanus formosanus NC009331 1	Halichoerus grypus NC001602 2	0,16914167
Mustela sibirica NC020637 6	Monachus schauinslandi NC008421 1	0,16914287
Pusa hispida NC 008433 1	Melogale moschata V0735A 1	0,1691449

Felis nigripes NC028309 1	Arctocephalus forsteri KT693377 17	0,18813579
Viverricula indica KX891745 1	Neophoca cinerea NC008419 1	0,18813583
Vulpes ferrilata NC027935 1	Prionailurus planiceps KY682741 4	0,18813686
Vulpes lagopus NC026529 3	Martes foina NC020643 1	0,18813792
Mungotictis decemlineata NC027828 1	Lycaon pictus NC028427 2	0,18813857
Parahyaena brunnea NC038159 15	Mustela itatsi NC034330 19	0,18813883
Speothos venaticus C48 2	Mustela putorius NC020638 4	0,18813962
Ursus arctos GU573491 207	Procyon lotor AB462046 3	0,18814018
Ursus arctos isabellinus 18B5 2	Procyon lotor AB462049 4	0,18814018
Zalophus wollebaeki SRR4431565 1	Xenogale naso C07XAR110 1	0,18814172
Ursus thibetanus mupinensis NC00875	Canis anthus NC027956 2	0,18814187
Urva javanica T413 1	Chrysocyon brachyurus NC024172 1	0,18814231
Martes pennanti NC020664 16	Ailuropoda melanoleuca NC009492 5	0,18814234
Neovison vison NC020641 3	Galidia elegans D146 1	0,1881427
Galictis vittata T412 1	Chrotogale owstoni T607 1	0,18814304
Mustela frenata NC020640 1	Crocuta crocuta NC020670 3	0,18814358
Lutrogale perspicillata NC035811 1	Caracal caracal NC028306 1	0,18814363
Lutrogale perspicillata NC035811 1	Helarctos malayanus NC009968 2	0,1881447
Otaria byronia OTAB 1	Genetta genetta T297 1	0,18814574
Panthera tigris amoyensis NC014770 2	Canis latrans NC008093 7	0,18814668
Ursus arctos GU573491 207	Lynx lynx NC027083 4	0,1881468
Ursus arctos EU497665 29	Lynx canadensis NC028313 1	0,18814728
Mungos mungo MMC7 1	Melursus ursinus NC009970 2	0,18814749
Ursus arctos EU497665 29	Lynx pardinus NC028319 161	0,18814781
Melursus ursinus NC009970 2	Galidictis fasciata DM333 1	0,18814828
Nyctereutes procyonoides NC013700 3	Arctictis binturong T605 2	0,18815288
Ursus arctos AP012576 6	Procyon lotor AB462049 4	0,18815495
Ursus thibetanus laniger MH281753 2	Paradoxurus hermaphroditus NC03959	0,18815982
Ursus thibetanus thibetanus NC01111	Panthera onca KP202264 2	0,18815985
Martes flavigula NC012141 3	Diplogale hosei MH464790 1	0,18816034
Urva javanica/auropunctata NC006835	Arctocephalus forsteri KT693377 17	0,18816131
Ursus arctos AP012576 6	Catopuma temminckii NC027115 41	0,18816738
Smilodon populator MF871700 1	Lutrogale perspicillata NC035811 1	0,18816741
Ursus americanus JX196366 3	Salanoia concolor D378 1	0,18816999
Ursus thibetanus formosanus NC0093	Mephitis mephitis NC020648 1	0,18817128
Lontra canadensis SRR10409165 1	Arctocephalus forsteri NC004023 28	0,1881723
Ursus spelaeus NC011112 8	Lycalopex sechurae KT448284 1	0,18817264
Ursus thibetanus laniger MH281753 2	Mephitis mephitis NC020648 1	0,18817377
Panthera leo NERO 19	Arctocephalus pusillus NC008417 1	0,18817562
Panthera pardus NC010641 5	Mustela sibirica NC020637 6	0,1881762
Potos flavus T414 1	Cynogale bennetti KY117544 1	0,18817881
Panthera pardus japonensis KJ866876	Martes flavigula NC012141 3	0,18818137
Panthera uncia KP202269 1	Eumetopias jubatus NC004030 10	0,18818486
Meles leucurus NC039173 4	Canis mesomelas KT448280 1	0,1881866
Eupleres goudotii D128 1	Canis latrans NC008093 7	0,18818741
Ursus thibetanus thibetanus NC01111	Canis mesomelas KT448280 1	0,18818778
Panthera tigris NC010642 35	Gulo gulo NC009685 3	0,18818887
Vulpes vulpes NC008434 5	Martes americana NC020642 1	0,18818958
Lycaon pictus NC028427 2	Diplogale hosei MH464790 1	0,18819012
Vulpes zerda KJ603240 1	Martes zibellina NC011579 39	0,18819103
Suricata suricatta SSM10 1	Canis anthus NC027956 2	0,1881915
Melogale moschata KP726273 1	Canis anthus NC027956 2	0,18819186
Tremarctos ornatus NC009969 2	Helogale parvula SRR7637809 1	0,18819231
Urocyon littoralis catalinae KP129018	Mustela nigripes NC024942 1	0,18819679
Melogale moschata KP726273 1	Galerella sanguinea T378 1	0,18819739
Panthera uncia KP202269 1	Meles meles T303 3	0,18819746
Vulpes corsac NC023958 1	Martes martes T302 3	0,18819746
Vulpes corsac NC023958 1	Arctotherium sp NC030174 1	0,18819892
Panthera pardus japonensis KJ866876	Lutra lutra LC050126 1	0,1881991
Cryptoprocta ferox CFC13 1	Bassaricyon neblina SRX1097850 1	0,18819982
Melogale moschata V0735A 1	Hyaena hyaena NC020669 1	0,18819991
Genetta genetta T297 1	Arctocephalus gazella BK010918 1	0,18820126
Prionailurus viverrinus NC028305 1	Canis mesomelas KT448280 1	0,18820153
Panthera pardus japonensis KJ866876	Melogale moschata V0735A 1	0,18820344
Lynx rufus NC014456 3	Lontra canadensis SRR10409165 1	0,18820445
Panthera leo spelaea KX258452 2	Lutra lutra LC050126 1	0,18820626
Paradoxurus jerdoni MH464793 1	Martes martes T302 3	0,18820688
Tremarctos ornatus NC009969 2	Cryptoprocta ferox CFC13 1	0,18820733
Lutra lutra LC050126 1	Ailuropoda melanoleuca NC009492 5	0,18820886
Ursus thibetanus mupinensis NC00875	Canis latrans NC008093 7	0,1882094
Parahyaena brunnea NC038159 15	Arctocephalus pusillus NC008417 1	0,18821022
Proteles cristata T393 6	Aonyx cinerea NC035814 2	0,18821082
Vulpes vulpes NC008434 5	Panthera tigris amoyensis NC014770 2	0,1882109
Lycaon pictus NC028427 2	Ailurus fulgens NC011124 1	0,18821178
Ursus arctos GU573486 5	Procyon lotor AB462046 3	0,18821399
Ursus maritimus NC003428 31	Panthera onca KP202264 2	0,18821429
Phocarctos hookeri NC008418 1	Genetta abyssinica MG489822 1	0,18821454
Helarctos malayanus NC009968 2	Conepatus chinga NC042596 1	0,1882153
Ursus arctos isabellinus 18B5 2	Puma concolor NC016470 22	0,18821584
Ursus spelaeus EU327344 13	Crossarchus platycephalus C7R66 1	0,18821606
Panthera onca NC022842 1	Lycalopex sechurae KT448284 1	0,18821849
Melogale moschata NC020644 1	Ailuropoda melanoleuca NC009492 5	0,18821965
Mephitis mephitis NC020648 1	Leopardus pardalis T262 1	0,18822049
Salanoia concolor D378 1	Chrysocyon brachyurus NC024172 1	0,18822117
Ursus spelaeus EU327344 13	Procyon lotor AB462046 3	0,18822217
Canis lupus familiaris NC002008 1231	Ailurus fulgens NC011124 1	0,1882271
Puma concolor NC016470 22	Melursus ursinus NC009970 2	0,18823071
Panthera pardus NC010641 5	Meles meles T303 3	0,18823816
Ursus americanus JX196366 3	Canis anthus NC027956 2	0,18824126
Poecilogale albinucha T602 1	Cuon alpinus NC013445 3	0,18824439
Ursus americanus JX196366 3	Felis chaus NC028307 1	0,18824747
Helarctos malayanus NC009968 2	Canis mesomelas KT448280 1	0,18824859
Meles anakuma NC009677 1	Helogale parvula SRR7637809 1	0,18825325
Vulpes corsac NC023958 1	Lutra lutra LC050126 1	0,18825413
Lontra canadensis SRR10409165 1	Atilax paludinosus T606 1	0,18825516
Melogale moschata KP726273 1	Bdeogale nigripes GLC15 1	0,18825541
Tremarctos ornatus NC009969 2	Xenogale naso C07XAR110 1	0,18825649
Suricata suricatta SSM10 1	Arctocephalus forsteri KT693377 17	0,18825693
Vulpes vulpes NC008434 5	Meles meles T303 3	0,1882578
Vulpes lagopus NC026529 3	Mungos mungo MMC7 1	0,18825859
Conepatus chinga NC042596 1	Bassaricyon neblina SRX1097850 1	0,18825983
Procyon lotor AB462049 4	Mungos mungo/gambianus SRR77048	0,18826073
Vulpes corsac NC023958 1	Mustela eversmannii NC028013 1	0,18826084

Viverricula indica KX891751 1	Mirounga leonina NC008422 1	0,16914499
Ursus arctos EU497665 29	Erignathus barbatus NC008426 1	0,16914633
Neovison vison NC020641 3	Ailurus fulgens NC011124 1	0,16914754
Panthera onca KP202264 2	Hydrurga leptonyx NC008425 1	0,16915691
Ursus maritimus GU573488 Svalbard	Phoca largha NC008430 1	0,16915701
Pusa sibirica NC008432 2	Meles anakuma NC009677 1	0,16919851
Mustela sibirica NC020637 6	Cystophora cristata NC008427 1	0,16920029
Pusa caspica NC008431 1	Mustela putorius NC020638 4	0,16920073
Phoca vitulina NC001325 1	Meles anakuma NC009677 1	0,16920993
Ursus arctos GU573486 5	Pusa caspica NC008431 1	0,16921897
Lobodon carcinophaga NC008423 1	Leopardus pardalis T262 1	0,16922428
Prionailurus bengalensis CKM45 20	Lobodon carcinophaga NC008423 1	0,16922674
Ursus maritimus NC003428 31	Lobodon carcinophaga NC008423 1	0,16922837
Ursus maritimus NC003428 31	Monachus schauinslandi NC008421 1	0,16922879
Monachus monachus NC044972 5	Meles leucurus NC039173 4	0,16923049
Ursus arctos GU573486 5	Monachus schauinslandi NC008421 1	0,16923554
Phoca fasciata NC008428 1	Leopardus colocolo NC028314 1	0,16924208
Pusa caspica NC008431 1	Martes flavigula NC012141 3	0,1692448
Ursus spelaeus EU327344 13	Monachus schauinslandi NC008421 1	0,16924576
Ursus americanus JX196366 3	Phoca groenlandica NC008429 54	0,16924581
Halichoerus grypus NC001602 2	Bassariscus sumichrasti SRX1099089 1	0,16924956
Martes martes T302 3	Halichoerus grypus NC001602 2	0,16925628
Pusa caspica NC008431 1	Mustela kathiah NC023210 1	0,1692678
Erignathus barbatus NC008426 1	Crossarchus platycephalus C7R66 1	0,16927013
Leptonychotes weddellii NC008424 1	Eupleres goudotii D128 1	0,16927044
Martes pennanti NC020664 16	Erignathus barbatus NC008426 1	0,16927108
Procyon lotor AB462049 4	Leptonychotes weddellii NC008424 1	0,16927236
Viverra tangalunga MH464792 1	Ailurus fulgens styani NC009691 1	0,16927263
Mirounga angustirostris SRR10331586 1	Lutra sumatrana NC035810 1	0,16927283
Phoca largha NC008430 1	Mustela itatsi NC034330 19	0,16928026
Puma yagouaroundi NC028311 1	Erignathus barbatus NC008426 1	0,16928147
Phoca vitulina NC001325 1	Melogale moschata NC020644 1	0,16928525
Ursus arctos isabellinus 18B5 2	Phoca fasciata NC008428 1	0,16928735
Phoca largha NC008430 1	Neovison vison NC020641 3	0,16928895
Mirounga leonina NC008422 1	Leopardus colocolo NC028314 1	0,16929808
Pusa hispida NC 008433 1	Leopardus colocolo NC028314 1	0,16931103
Mustela itatsi NC034330 19	Halichoerus grypus NC001602 2	0,16931574
Pusa caspica NC008431 1	Melogale moschata V0735A 1	0,16933382
Mustela sibirica AP017394 11	Cystophora cristata NC008427 1	0,1693348
Mustela frenata NC020640 1	Ailurus fulgens NC011124 1	0,16933489
Pusa caspica NC008431 1	Mustela itatsi NC034330 19	0,1693358
Nandinia binotata NC024567 1	Mustela frenata NC020640 1	0,16933586
Fossa fossana D350 1	Erignathus barbatus NC008426 1	0,16933747
Procyon lotor AB462046 3	Mustela itatsi NC034330 19	0,16933808
Potos flavus T414 1	Nandinia binotata NC024567 1	0,16933914
Phoca fasciata NC008428 1	Meles anakuma NC009677 1	0,16934064
Pardofelis marmorata NLN3 2	Leptonychotes weddellii NC008424 1	0,16934641
Phoca largha NC008430 1	Mustela sibirica AP017394 11	0,16934662
Phoca vitulina NC001325 1	Mustela itatsi NC034330 19	0,16934775
Mustela altaica NC021751 1	Lobodon carcinophaga NC008423 1	0,16935505
Ursus maritimus GU573488 Svalbard	Monachus schauinslandi NC008421 1	0,16936357
Ursus thibetanus thibetanus NC011118 4	Monachus monachus NC044972 5	0,16939682
Potos flavus T414 1	Lontra canadensis SRR10409165 1	0,1694023
Phoca fasciata NC008428 1	Enhydra lutris NC009692 1	0,16940567
Phoca groenlandica NC008429 54	Mustela nigripes NC024942 1	0,16941202
Pusa sibirica NC008432 2	Neovison vison NC020641 3	0,16941239
Phoca largha NC008430 1	Mustela kathiah NC023210 1	0,16941373
Ursus thibetanus thibetanus NC011118 4	Halichoerus grypus NC001602 2	0,16941404
Procyon lotor AB462049 4	Mustela nivalis T306 5	0,16941439
Hydrurga leptonyx NC008425 1	Atilax paludinosus T606 1	0,16941604
Ursus thibetanus mupinensis NC008753 2	Mirounga leonina NC008422 1	0,1694179
Procyon lotor AB462049 4	Hydrurga leptonyx NC008425 1	0,16941901
Puma concolor NC016470 22	Lobodon carcinophaga NC008423 1	0,169425
Prionailurus bengalensis NC028301 12	Mirounga angustirostris SRR10331586 1	0,16942723
Martes flavigula NC012141 3	Halichoerus grypus NC001602 2	0,16943221
Monachus schauinslandi NC008421 1	Leopardus colocolo NC028314 1	0,16944367
Pusa sibirica NC008432 2	Mustela itatsi NC034330 19	0,1694707
Meles meles T303 3	Ailurus fulgens NC011124 1	0,16947319
Meles leucurus NC039173 4	Ailurus fulgens NC011124 1	0,1694732
Phoca fasciata NC008428 1	Melogale moschata V0735A 1	0,1694767
Ursus thibetanus laniger MH281753 2	Halichoerus grypus NC001602 2	0,16947825
Phoca largha NC008430 1	Melogale moschata V0735A 1	0,16948113
Phoca vitulina NC001325 1	Mustela sibirica AP017394 11	0,1694814
Prionailurus planiceps KY682741 4	Leptonychotes weddellii NC008424 1	0,16948172
Mungos mungo MMC7 1	Hydrurga leptonyx NC008425 1	0,16948316
Ursus arctos pruinosus MG066703 3	Leptonychotes weddellii NC008424 1	0,16948864
Phoca groenlandica NC008429 54	Neovison vison NC020641 3	0,16948912
Viverricula indica KX891745 1	Monachus schauinslandi NC008421 1	0,16948947
Monachus monachus NC044972 5	Lutra lutra LC050126 1	0,16949007
Ursus arctos isabellinus 18B5 2	Phoca largha NC008430 1	0,16949359
Phoca largha NC008430 1	Ictonyx striatus T299 1	0,16949596
Panthera onca NC022842 1	Hydrurga leptonyx NC008425 1	0,16950506
Martes melampus NC009678 1	Halichoerus grypus NC001602 2	0,16951647
Mustela sibirica AP017394 11	Erignathus barbatus NC008426 1	0,16953593
Procyon lotor AB462049 4	Mustela sibirica AP017394 11	0,16953988
Cystophora cristata NC008427 1	Aonyx cinerea NC035814 2	0,1695424
Phoca fasciata NC008428 1	Mustela nigripes NC024942 1	0,16954635
Pusa hispida NC 008433 1	Mustela eversmannii NC028013 1	0,16954722
Phoca vitulina NC001325 1	Enhydra lutris NC009692 1	0,16954723
Ursus maritimus NC003428 31	Pusa sibirica NC008432 2	0,16954964
Mustela kathiah NC023210 1	Mirounga angustirostris SRR10331586 1	0,16954985
Pusa sibirica NC008432 2	Catopuma badia NC028300 1	0,16955177
Leopardus wiedii NC028318 1	Cystophora cristata NC008427 1	0,16955347
Phoca vitulina NC001325 1	Martes pennanti NC020664 16	0,16955487
Lobodon carcinophaga NC008423 1	Galerella sanguinea T378 1	0,16956018
Ursus arctos GU573486 5	Phoca groenlandica NC008429 54	0,16956313
Ursus arctos EU497665 29	Monachus monachus NC044972 5	0,16958573
Leptonychotes weddellii NC008424 1	Chrotogale owstoni T607 1	0,16959606
Pusa caspica NC008431 1	Mustela eversmannii NC028013 1	0,16960439
Phoca groenlandica NC008429 54	Lutra lutra NC011358 9	0,16960622
Mirounga leonina NC008422 1	Fossa fossana D350 1	0,16960679
Procyon lotor AB462049 4	Poecilogale albinucha T602 1	0,16961385
Phoca groenlandica NC008429 54	Ailurus fulgens styani NC009691 1	0,16961585

Procyon lotor AB462046 3	Galerella sanguinea T378 1	0,18826142
Nyctereutes procyonoides NC013700 3	Mustela eversmannii NC028013 1	0,1882616
Vulpes ferrilata NC027935 1	Viverra tangalunga MH464792 1	0,18826284
Nasua nasua NC020647 1	Arctocephalus pusillus NC008417 1	0,18826294
Viverricula indica KX891751 1	Arctocephalus forsteri KT693377 17	0,18826331
Mungos mungo/gambianus SRR77048	Arctotherium sp NC030174 1	0,18826489
Mungos mungo MMC7 1	Arctotherium sp NC030174 1	0,18826513
Ursus arctos EU497665 29	Lutra lutra LC050126 1	0,1882686
Ursus maritimus NC003428 31	Crossarchus platycephalus C7R66 1	0,18826985
Ursus arctos EU497665 29	Crossarchus platycephalus C7R66 1	0,18826988
Felis catus NC001700 2	Arctocephalus forsteri KT693377 17	0,18827009
Felis margarita NC028308 1	Arctocephalus townsendi NC008420 1	0,18827014
Parahyaena brunnea NC038159 15	Lontra canadensis SRR10409165 1	0,18827014
Parahyaena brunnea NC038159 15	Melogale moschata V0735A 1	0,18827075
Vulpes zerda KJ603240 1	Caracal caracal NC028306 1	0,1882724
Ictonyx striatus T299 1	Cynictis penicillata T375 1	0,18827245
Viverra zibetha T609 1	Aonyx cinerea NC035814 2	0,18827249
Leopardus jacobita NC028322 1	Canis anthus NC027956 2	0,1882307
Ursus maritimus GU573488 Svalbard	Salanoia concolor D378 1	0,18827315
Zalophus wollebaeki SRR4431565 1	Vulpes vulpes NC008434 5	0,18827394
Puma concolor NC016470 22	Neophoca cinerea NC008419 1	0,18827434
Proteles cristata T393 6	Lutra sumatrana NC035810 1	0,18827501
Odobenus rosmarus NC004029 29	Meles meles T303 3	0,18827538
Hyaena hyaena NC020669 1	Arctocephalus pusillus NC008417 1	0,18827635
Procyon lotor AB462046 3	Panthera uncia KP202269 1	0,18827659
Ursus americanus JX196366 3	Canis lupus familiaris NC002008 1231	0,1882772
Leopardus pardalis T262 1	Bassaricyon neblina SRX1097850 1	0,18827863
Urva javanica/auropunctata NC006835	Eumetopias jubatus NC004030 10	0,1882796
Ursus arctos EU497665 29	Genetta genetta T297 1	0,18827964
Ursus thibetanus laniger MH281753 2	Lutrogale perspicillata NC035811 1	0,18828009
Urva javanica T413 1	Galictis vittata T412 1	0,18828051
Viverricula indica KX891751 1	Otaria byronia OTAB 1	0,1882811
Poecilogale albinucha T602 1	Lycaon pictus NC028427 2	0,18828298
Ursus arctos pruinosus MG066703 3	Leopardus jacobita NC028322 1	0,18828326
Ursus maritimus NC003428 31	Prionailurus viverrinus NC028305 1	0,18828344
Ursus arctos pruinosus MG066703 3	Caracal caracal NC028306 1	0,18828455
Ursus arctos pruinosus MG066703 3	Paradoxurus jerdoni MH464793 1	0,18828498
Zalophus wollebaeki SRR4431565 1	Panthera uncia KP202269 1	0,18828616
Ursus maritimus NC003428 31	Leopardus guigna NC028321 1	0,18828721
Helarctos malayanus NC009968 2	Canis anthus NC027956 2	0,1882875
Panthera leo spelaea KX258452 2	Arctodus simus NC011116 1	0,18828888
Urocyon cinereoargenteus NC026723 2	Leopardus colocolo NC028314 1	0,18828954
Ursus thibetanus mupinensis NC00875	Diplogale hosei MH464790 1	0,18829089
Panthera pardus NC010641 5	Eumetopias jubatus NC004030 10	0,18829247
Lontra canadensis SRR10409165 1	Hemigalus derbyanus MH464791 1	0,18829274
Ictonyx striatus T299 1	Canis mesomelas KT448280 1	0,18829346
Ursus thibetanus laniger MH281753 2	Panthera onca KP202264 2	0,18829367
Ursus thibetanus laniger MH281753 2	Pardofelis marmorata NLN3 2	0,18829466
Ursus spelaeus NC011112 8	Ichneumia albicauda T603 1	0,18829545
Otocyon megalotis SAF1 2	Acinonyx jubatus NC005212 3	0,18829601
Ursus americanus JX196366 3	Crossarchus platycephalus C7R66 1	0,18829834
Nyctereutes procyonoides NC013700 3	Gulo gulo NC009685 3	0,18830206
Spilogale putorius NC010497 1	Prionodon linsang ERR2391707 1	0,18830356
Neofelis nebulosa NC008450 3	Lutra sumatrana NC035810 1	0,18830486
Canis mesomelas KT448280 1	Ailurus fulgens styani NC009691 1	0,1883074
Ursus americanus JX196366 3	Felis silvestris lybica KP202275 4	0,18831353
Ursus maritimus NC003428 31	Spilogale putorius NC010497 1	0,18831377
Urocyon littoralis catalinae KP129018	Martes flavigula NC012141 3	0,18831456
Melursus ursinus NC009970 2	Acinonyx jubatus NC005212 3	0,18831497
Ursus americanus JX196366 3	Leopardus guigna NC028321 1	0,18831803
Suricata suricatta SSM10 1	Enhydra lutris NC009692 1	0,18831873
Helogale parvula SRR7637809 1	Enhydra lutris NC009692 1	0,188319
Lontra canadensis SRR10409165 1	Genetta abyssinica MG489822 1	0,18832092
Mustela sibirica AP017394 11	Helogale parvula SRR7637809 1	0,18832298
Urva brachyura KY117547 1	Arctocephalus forsteri KT693377 17	0,18832341
Phocarctos hookeri NC008418 1	Lontra canadensis SRR10409165 1	0,18832371
Mustela eversmannii NC028013 1	Bdeogale nigripes GLC15 1	0,18832427
Helogale parvula SRR7637809 1	Arctocephalus australis MG023139 1	0,18832438
Vulpes vulpes NC008434 5	Atilax paludinosus T606 1	0,18832502
Nasua nasua NC020647 1	Nandinia binotata NC024567 1	0,18832532
Helogale parvula SRR7637809 1	Canis anthus NC027956 2	0,18832575
Vulpes ferrilata NC027935 1	Fossa fossana D350 1	0,18832646
Vulpes vulpes NC008434 5	Mustela eversmannii NC028013 1	0,18832792
Galidictis fasciata DM333 1	Arctocephalus forsteri KT693377 17	0,18833044
Meles meles T303 3	Hyaena hyaena NC020669 1	0,18833436
Mustela altaica NC021751 1	Bdeogale nigripes GLC15 1	0,1883346
Ursus maritimus GU573488 Svalbard	Lutra lutra NC011358 9	0,18833539
Poecilogale albinucha T602 1	Mungos mungo/gambianus SRR77048	0,1883357
Felis chaus NC028307 1	Arctocephalus forsteri KT693377 17	0,18833773
Lontra canadensis SRR10409165 1	Canis aureus KT448274 1	0,1883385
Panthera onca KP202264 2	Canis anthus NC027956 2	0,18833953
Panthera pardus japonensis KJ866876	Canis anthus NC027956 2	0,18833989
Cryptoprocta ferox CFC13 1	Arctocephalus gazella BK010918 1	0,18834086
Puma concolor NC016470 22	Phocarctos hookeri NC008418 1	0,18834122
Speothos venaticus C48 2	Mustela eversmannii NC028013 1	0,18834123
Ursus maritimus NC003428 31	Procyon lotor AB462046 3	0,18834215
Ursus arctos EU497665 29	Procyon lotor AB462046 3	0,18834218
Panthera tigris NC010642 35	Arctonyx collaris NC020645 1	0,18834355
Panthera tigris NC010642 35	Enhydra lutris NC009692 1	0,18834367
Procyon lotor AB462049 4	Paradoxurus hermaphroditus NC03959	0,188344
Tremarctos ornatus NC009969 2	Parahyaena brunnea NC038159 15	0,18834433
Otocyon megalotis SAF1 2	Genetta genetta T297 1	0,18834469
Prionodon linsang ERR2391707 1	Ailuropoda melanoleuca NC009492 5	0,18834585
Profelis aurata NC028299 1	Aonyx cinerea NC035814 2	0,18834619
Ursus arctos GU573486 5	Salanoia concolor D378 1	0,18834649
Ursus arctos GU573491 207	Genetta genetta T297 1	0,1883468
Panthera tigris amoyensis NC014770 2	Martes zibellina NC011579 39	0,18834797
Ursus arctos isabellinus 18B5 2	Lynx canadensis NC028313 1	0,18834865
Ursus arctos EU497665 29	Panthera onca KP202264 2	0,18834905
Ursus maritimus GU573488 Svalbard	Puma concolor NC016470 22	0,18835105
Ursus maritimus NC003428 31	Puma concolor NC016470 22	0,18835106
Speothos venaticus C48 2	Prionailurus viverrinus NC028305 1	0,18835139
Speothos venaticus C48 2	Felis nigripes NC028309 1	0,18835177

Profelis aurata NC028299 1	Leptonychotes weddellii NC008424 1	0,16961712
Ursus maritimus NC003428 31	Erignathus barbatus NC008426 1	0,16961755
Ursus maritimus GU573488 Svalbard	Erignathus barbatus NC008426 1	0,16961756
Pusa sibirica NC008432 2	Ictonyx striatus T299 1	0,1696185
Mirounga angustirostris SRR10331586 1	Hemigalus derbyanus MH464791 1	0,16962063
Ictonyx striatus T299 1	Bassariscus sumichrasti SRX1099089 1	0,16962098
Ursus maritimus NC003428 31	Pusa hispida NC 008433 1	0,16963072
Monachus schauinslandi NC008421 1	Martes flavigula NC012141 3	0,16964933
Halichoerus grypus NC001602 2	Enhydra lutris NC009692 1	0,16965184
Ursus maritimus NC003428 31	Monachus monachus NC044972 5	0,16965294
Phoca fasciata NC008428 1	Martes flavigula NC012141 3	0,16965331
Diplogale hosei MH464790 1	Crocuta crocuta NC020670 3	0,16966327
Monachus monachus NC044972 5	Helarctos malayanus NC009968 2	0,16966738
Procyon lotor AB462046 3	Lutra lutra LC050126 1	0,16967177
Pusa sibirica NC008432 2	Mustela nigripes NC024942 1	0,16967252
Leptonychotes weddellii NC008424 1	Atilax paludinosus T606 1	0,16967411
Mirounga leonina NC008422 1	Crossarchus platycephalus C7R66 1	0,16967411
Leptonychotes weddellii NC008424 1	Canis latrans NC008093 7	0,16967522
Procyon lotor AB462046 3	Melogale moschata KP726273 1	0,16967647
Pusa caspica NC008431 1	Aonyx cinerea NC035814 2	0,16967684
Mustela putorius NC020638 4	Monachus schauinslandi NC008421 1	0,16968167
Ursus maritimus GU573488 Svalbard	Pusa sibirica NC008432 2	0,16968432
Phoca groenlandica NC008429 54	Melogale moschata NC020644 1	0,16968469
Ursus americanus JX196366 3	Monachus monachus NC044972 5	0,16968751
Lobodon carcinophaga NC008423 1	Leopardus guigna NC028321 1	0,16969709
Phoca vitulina NC001325 1	Ictonyx striatus T299 1	0,16969807
Ursus spelaeus EU327344 13	Phoca vitulina NC001325 1	0,16970849
Mustela sibirica AP017394 11	Halichoerus grypus NC001602 2	0,16971937
Smilodon populator MF871700 1	Hydrurga leptonyx NC008425 1	0,16972138
Martes zibellina NC011579 39	Halichoerus grypus NC001602 2	0,16972241
Potos flavus T414 1	Melogale moschata V0735A 1	0,16973843
Pusa sibirica NC008432 2	Mustela kathiah NC023210 1	0,16973935
Pusa sibirica NC008432 2	Fossa fossana D350 1	0,16974145
Procyon lotor AB462046 3	Mirounga leonina NC008422 1	0,1697455
Taxidea taxus NC020646 1	Bassaricyon neblina SRX1097850 1	0,1697496
Viverra tangalunga MH464792 1	Mirounga angustirostris SRR10331586 1	0,16975181
Phoca groenlandica NC008429 54	Mustela nivalis T306 5	0,16975789
Pardofelis marmorata NLN3 2	Hydrurga leptonyx NC008425 1	0,16976222
Neovison vison NC020641 3	Mirounga angustirostris SRR10331586 1	0,16976234
Ursus arctos GU573486 5	Phoca vitulina NC001325 1	0,16976944
Lobodon carcinophaga NC008423 1	Ailuropoda melanoleuca NC009492 5	0,1697744
Phoca largha NC008430 1	Homotherium latidens MF871702 3	0,16977443
Mirounga leonina NC008422 1	Martes melampus NC009678 1	0,16979839
Mustela itatsi NC034330 19	Ailurus fulgens styani NC009691 1	0,16981098
Pusa sibirica NC008432 2	Martes pennanti NC020664 16	0,16981176
Procyon lotor AB462046 3	Martes pennanti NC020664 16	0,16981561
Mustela altaica NC021751 1	Cystophora cristata NC008427 1	0,16981736
Phoca fasciata NC008428 1	Ailurus fulgens styani NC009691 1	0,16981761
Lynx rufus NC014456 3	Cystophora cristata NC008427 1	0,16981959
Ursus maritimus GU573488 Svalbard	Phoca groenlandica NC008429 54	0,16982619
Leptonychotes weddellii NC008424 1	Ailuropoda melanoleuca NC009492 5	0,16982901
Ursus maritimus GU573488 Svalbard	Pusa hispida NC 008433 1	0,1698328
Ursus arctos EU497665 29	Monachus schauinslandi NC008421 1	0,16983528
Ursus spelaeus NC011112 8	Mirounga angustirostris SRR10331586 1	0,16984662
Mirounga angustirostris SRR10331586 1	Gulo gulo NC009685 3	0,16984665
Ommatophoca rossii AY377287etc 1	Arctotherium sp NC030174 1	0,16986935
Enhydra lutris NC009692 1	Cystophora cristata NC008427 1	0,16987041
Pusa sibirica NC008432 2	Melogale moschata V0735A 1	0,16987269
Viverra tangalunga MH464792 1	Pusa caspica NC008431 1	0,16987428
Viverra tangalunga MH464792 1	Pusa sibirica NC008432 2	0,16987459
Pusa caspica NC008431 1	Fossa fossana D350 1	0,16987611
Monachus schauinslandi NC008421 1	Meles anakuma NC009677 1	0,16987836
Phoca groenlandica NC008429 54	Melogale moschata KP726273 1	0,16988112
Phoca fasciata NC008428 1	Mustela sibirica AP017394 11	0,16988233
Procyon lotor AB462049 4	Martes pennanti NC020664 16	0,16988295
Mirounga leonina NC008422 1	Leopardus pardalis T262 1	0,16988569
Ursus maritimus GU573488 Svalbard	Pusa caspica NC008431 1	0,16988626
Pusa sibirica NC008432 2	Leopardus pardalis NC028315 1	0,16989106
Phoca vitulina NC001325 1	Aonyx cinerea NC035814 2	0,16989227
Ursus arctos isabellinus 18B5 2	Phoca groenlandica NC008429 54	0,16989352
Ursus arctos GU573491 207	Phoca largha NC008430 1	0,16989781
Ursus arctos pruinosus MG066703 3	Phoca largha NC008430 1	0,16990399
Mirounga leonina NC008422 1	Gulo gulo NC009685 3	0,16990747
Mustela sibirica NC020637 6	Halichoerus grypus NC001602 2	0,1699214
Melogale moschata NC020644 1	Halichoerus grypus NC001602 2	0,16992956
Viverra tangalunga MH464792 1	Phoca fasciata NC008428 1	0,16995022
Phoca largha NC008430 1	Mustela sibirica NC020637 6	0,16995268
Ursus maritimus NC003428 31	Pusa caspica NC008431 1	0,16995357
Phoca largha NC008430 1	Fossa fossana D350 1	0,16995756
Phoca fasciata NC008428 1	Neovison vison NC020641 3	0,16996007
Ursus arctos GU573491 207	Phoca groenlandica NC008429 54	0,16996101
Ursus arctos EU497665 29	Phoca groenlandica NC008429 54	0,16996102
Ursus arctos EU497665 29	Phoca largha NC008430 1	0,16996513
Procyon lotor AB462046 3	Gulo gulo NC009685 3	0,16998634
Meles anakuma NC009677 1	Halichoerus grypus NC001602 2	0,16998649
Procyon lotor AB462049 4	Gulo gulo NC009685 3	0,16998659
Melogale moschata V0735A 1	Halichoerus grypus NC001602 2	0,16999096
Enhydra lutris NC009692 1	Ailurus fulgens styani NC009691 1	0,16999969
Mustela itatsi NC034330 19	Erignathus barbatus NC008426 1	0,17000797
Mirounga leonina NC008422 1	Martes pennanti NC020664 16	0,1700114
Viverra tangalunga MH464792 1	Ailurus fulgens NC011124 1	0,17001356
Mirounga angustirostris SRR10331586 1	Meles anakuma NC009677 1	0,17001694
Phoca vitulina NC001325 1	Mustela sibirica NC020637 6	0,1700201
Phoca vitulina NC001325 1	Mustela eversmannii NC028013 1	0,17002028
Ursus arctos isabellinus 18B5 2	Pusa sibirica NC008432 2	0,17002082
Prionailurus viverrinus NC028305 1	Cystophora cristata NC008427 1	0,17002515
Viverricula indica KX891751 1	Mirounga angustirostris SRR10331586 1	0,17003608
Ursus arctos GU573491 207	Monachus schauinslandi NC008421 1	0,17003702
Phoca largha NC008430 1	Leopardus pardalis NC028315 1	0,17003874
Hydrurga leptonyx NC008425 1	Arctictis binturong T605 2	0,17004237
Phoca vitulina NC001325 1	Helarctos malayanus NC009968 2	0,170046
Ursus maritimus GU573488 Svalbard	Monachus monachus NC044972 5	0,17005703
Monachus monachus NC044972 5	Martes flavigula NC012141 3	0,1700732

Tremarctos ornatus NC009969 2	Galictis vittata T412 1	0,18835206
Ursus thibetanus laniger MH281753 2	Hyaena hyaena NC020669 1	0,18835396
Ursus arctos GU573486 5	Lynx canadensis NC028313 1	0,18835542
Leopardus wiedii NC028318 1	Ailuropoda melanoleuca NC009492 5	0,18835564
Panthera onca NC022842 1	Mustela altaica NC021751 1	0,18835643
Zalophus californianus NC008416 1	Vulpes vulpes NC008434 5	0,18835656
Ursus spelaeus EU327344 13	Otocolobus manul NC028323 1	0,18835987
Ursus thibetanus thibetanus NC01111	Paradoxurus hermaphroditus NC03959	0,18836008
Prionailurus bengalensis CKM45 20	Ailuropoda melanoleuca NC009492 5	0,18836109
Canis adustus KT448271 1	Bassaricyon neblina SRX1097850 1	0,18836369
Potos flavus T414 1	Canis lupus chanco NC010340 4	0,18836375
Gulo gulo NC009685 3	Bdeogale nigripes GLC15 1	0,1883657
Ursus americanus JX196366 3	Canis latrans NC008093 7	0,18837602
Conepatus chinga NC042596 1	Arctocephalus townsendi NC008420 1	0,18838771
Lynx pardinus NC028319 161	Cuon alpinus NC013445 3	0,18838802
Mungos mungo/gambianus SRR77048	Lontra canadensis SRR10409165 1	0,18838918
Lycalopex sechurae KT448284 1	Leopardus guigna NC028321 1	0,18838999
Mustela eversmannii NC028013 1	Helogale parvula SRR7637809 1	0,1883904
Vulpes ferrilata NC027935 1	Enhydra lutris NC009692 1	0,18839157
Vulpes vulpes NC008434 5	Cynictis penicillata T375 1	0,18839176
Vulpes corsac NC023958 1	Meles meles T303 3	0,18839291
Urocyon littoralis catalinae KP129018	Martes melampus NC009678 1	0,18839347
Urva javanica T413 1	Aonyx cinerea NC035814 2	0,18839598
Galidictis fasciata DM333 1	Canis anthus NC027956 2	0,18839687
Urocyon cinereoargenteus NC026723 2	Mustela nigripes NC024942 1	0,18839873
Vulpes ferrilata NC027935 1	Melogale moschata NC020644 1	0,18840182
Lycaon pictus NC028427 2	Lutra sumatrana NC035810 1	0,18840304
Zalophus californianus NC008416 1	Panthera leo NERO 19	0,18840382
Panthera uncia KP202269 1	Martes melampus NC009678 1	0,18840386
Vulpes vulpes NC008434 5	Prionodon pardicolor NC024569 2	0,18840444
Felis silvestris lybica KP202275 4	Arctocephalus townsendi NC008420 1	0,18840475
Panthera onca KP202264 2	Arctocephalus townsendi NC008420 1	0,18840489
Martes melampus NC009678 1	Chrysocyon brachyurus NC024172 1	0,18840493
Otocyon megalotis SAF1 2	Mustela nigripes NC024942 1	0,18840495
Vulpes lagopus NC026529 3	Neovison vison NC020641 3	0,18840596
Vulpes zerda KJ603240 1	Martes foina NC020643 1	0,18840688
Lontra canadensis SRR10409165 1	Felis margarita NC028308 1	0,18840778
Neophoca cinerea NC008419 1	Felis nigripes NC028309 1	0,18840924
Vulpes ferrilata NC027935 1	Leopardus guigna NC028321 1	0,18840994
Panthera tigris NC010642 35	Lutra lutra NC011358 9	0,18841124
Urocyon cinereoargenteus NC026723 2	Prionailurus bengalensis CKM45 20	0,188412
Catopuma temminckii NC027115 41	Bassaricyon neblina SRX1097850 1	0,18841268
Eupleres goudotii D128 1	Canis aureus KT448274 1	0,18841292
Prionailurus planiceps KY682741 4	Otocyon megalotis SAF1 2	0,18841499
Panthera leo NERO 19	Martes flavigula NC012141 3	0,18841526
Ursus maritimus GU573488 Svalbard	Panthera onca KP202264 2	0,18841633
Ursus arctos GU573491 207	Leopardus pardalis T262 1	0,1884173
Ursus thibetanus thibetanus NC01111	Lutrogale perspicillata NC035811 1	0,18841742
Ursus arctos pruinosus MG066703 3	Prionailurus rubiginosus NC028304 2	0,18841879
Ursus thibetanus formosanus NC0093	Bassaricyon neblina SRX1097850 1	0,18841903
Panthera uncia KP202269 1	Martes foina NC020643 1	0,1884193
Panthera pardus japonensis KJ866876	Martes foina NC020643 1	0,18841936
Lycalopex sechurae KT448284 1	Urva javanica/auropunctata NC00683	0,18842016
Mephitis mephitis NC020648 1	Cynogale bennetti KY117544 1	0,18842091
Paradoxurus jerdoni MH464793 1	Galictis vittata T412 1	0,18842153
Procyon lotor AB462046 3	Ailuropoda melanoleuca NC009492 5	0,18842228
Ictonyx striatus T299 1	Cryptoprocta ferox CFC13 1	0,18842345
Otocyon megalotis SAF1 2	Hemigalus derbyanus MH464791 1	0,18842368
Urocyon cinereoargenteus NC026723 2	Acinonyx jubatus NC005212 3	0,18842442
Prionailurus rubiginosus NC028304 2	Mephitis mephitis NC020648 1	0,18842475
Ursus thibetanus formosanus NC0093	Galerella sanguinea T378 1	0,18842489
Panthera leo spelaea KX258452 2	Melogale moschata NC020644 1	0,18842585
Melogale moschata V0735A 1	Canis lupus familiaris NC002008 1231	0,18842784
Lontra canadensis SRR10409165 1	Canis lupus familiaris NC002008 1231	0,18842867
Leopardus guigna NC028321 1	Ailuropoda melanoleuca NC009492 5	0,18843085
Ursus arctos AP012576 6	Lynx lynx NC027083 4	0,18843284
Ursus arctos AP012576 6	Leopardus pardalis NC028315 1	0,18843368
Ursus americanus JX196366 3	Lutra lutra NC011358 9	0,18843758
Ursus thibetanus thibetanus NC01111	Mephitis mephitis NC020648 1	0,18844044
Viverricula indica NC025296 2	Arctocephalus forsteri NC004023 28	0,18844368
Gulo gulo NC009685 3	Cryptoprocta ferox CFC13 1	0,1884463
Gulo gulo NC009685 3	Canis mesomelas KT448280 1	0,1884471
Ursus arctos EU497665 29	Spilogale putorius NC010497 1	0,18844843
Ictonyx striatus T299 1	Helarctos malayanus NC009968 2	0,18844911
Melogale moschata NC020644 1	Cuon alpinus NC013445 3	0,18844922
Panthera pardus japonensis KJ866876	Gulo gulo NC009685 3	0,18844944
Martes flavigula NC012141 3	Galidia elegans D146 1	0,18845119
Lynx rufus NC014456 3	Cuon alpinus NC013445 3	0,18845505
Conepatus chinga NC042596 1	Arctocephalus forsteri KT693377 17	0,18845598
Prionodon linsang ERR2391707 1	Conepatus chinga NC042596 1	0,18845698
Neophoca cinerea NC008419 1	Lontra canadensis SRR10409165 1	0,18845868
Vulpes ferrilata NC027935 1	Melogale moschata V0735A 1	0,18845958
Phocarctos hookeri NC008418 1	Cynictis penicillata T375 1	0,18846096
Urva javanica T413 1	Ailurus fulgens styani NC009691 1	0,18846107
Vulpes vulpes NC008434 5	Lutra sumatrana NC035810 1	0,18846118
Lycaon pictus NC028427 2	Lutra lutra NC011358 9	0,1884634
Speothos venaticus C48 2	Smilodon populator MF871700 1	0,18846367
Lutra lutra NC011358 9	Canis mesomelas KT448280 1	0,18846379
Procyon lotor AB462046 3	Urva javanica T413 1	0,18846484
Vulpes ferrilata NC027935 1	Paradoxurus jerdoni MH464793 1	0,18846945
Ursus arctos EU497665 29	Lutra lutra NC011358 9	0,18847014
Meles anakuma NC009677 1	Hyaena hyaena NC020669 1	0,18847063
Panthera uncia KP202269 1	Martes americana NC020642 1	0,18847305
Ursus thibetanus mupinensis NC0087	Cynictis penicillata T375 1	0,18847328
Vulpes ferrilata NC027935 1	Otocolobus manul NC028323 1	0,18847418
Ictonyx striatus T299 1	Bdeogale nigripes GLC15 1	0,18847433
Lycaon pictus NC028427 2	Galidictis fasciata DM333 1	0,18847446
Crocuta crocuta NC020670 3	Aonyx cinerea NC035814 2	0,18847457
Lontra canadensis SRR10409165 1	Leopardus tigrinus NC028317 1	0,18847537
Panthera uncia NC010638 1	Aonyx cinerea NC035814 2	0,18847822
Panthera leo spelaea KX258452 2	Meles meles T303 3	0,18848044
Ursus arctos isabellinus 18B5 2	Paradoxurus hermaphroditus NLNC 1	0,18848121
Panthera onca KP202264 2	Martes martes T302 3	0,18848258

Melogale moschata V0735A 1	Cystophora cristata NC008427 1	0,17007445
Phoca groenlandica NC008429 54	Homotherium latidens MF871702 3	0,17007646
Procyon lotor AB462049 4	Mustela itatsi NC034330 19	0,17007834
Erignathus barbatus NC008426 1	Ailurus fulgens styani NC009691 1	0,17007874
Mustela putorius NC020638 4	Ailurus fulgens styani NC009691 1	0,17007965
Viverra tangalunga MH464792 1	Pusa hispida NC 008433 1	0,17008539
Mustela nigripes NC024942 1	Monachus schauinslandi NC008421 1	0,17008579
Leptonychotes weddellii NC008424 1	Catopuma temminckii NC027115 41	0,17008686
Phoca largha NC008430 1	Mustela eversmannii NC028013 1	0,17008752
Leptailurus serval NC028316 1	Erignathus barbatus NC008426 1	0,1700898
Hydrurga leptonyx NC008425 1	Eupleres goudotii D128 1	0,17009103
Neovison vison NC020641 3	Monachus schauinslandi NC008421 1	0,17009388
Mirounga angustirostris SRR10331586 1	Ailurus fulgens styani NC009691 1	0,17009619
Pusa hispida NC 008433 1	Mustela altaica NC021751 1	0,17009695
Hydrurga leptonyx NC008425 1	Genetta genetta T297 1	0,17009979
Ursus spelaeus EU327344 13	Pusa caspica NC008431 1	0,17010105
Prionailurus bengalensis NC028301 12	Monachus monachus NC044972 5	0,17011937
Phoca vitulina NC001325 1	Leopardus colocolo NC028314 1	0,17012346
Ursus arctos GU573486 5	Monachus monachus NC044972 5	0,17013109
Procyon lotor AB462049 4	Lutra lutra NC011358 9	0,17014311
Poecilogale albinucha T602 1	Mellivora capensis T370 1	0,17014472
Phoca groenlandica NC008429 54	Martes melampus NC009678 1	0,1701459
Mustela nigripes NC024942 1	Ailurus fulgens styani NC009691 1	0,1701472
Erignathus barbatus NC008426 1	Aonyx cinerea NC035814 2	0,1701493
Phoca groenlandica NC008429 54	Mustela sibirica NC020637 6	0,17015228
Pusa hispida NC 008433 1	Ailurus fulgens styani NC009691 1	0,17015332
Potos flavus T414 1	Ommatophoca rossii AY377287etc 1	0,17015468
Lynx canadensis NC028313 1	Erignathus barbatus NC008426 1	0,17015496
Ursus arctos isabellinus 18B5 2	Mirounga leonina NC008422 1	0,17015652
Leopardus geoffroyi NC028320 1	Cystophora cristata NC008427 1	0,17015961
Prionailurus bengalensis CKM45 20	Cystophora cristata NC008427 1	0,17016146
Ursus maritimus NC003428 31	Phoca groenlandica NC008429 54	0,1701628
Cystophora cristata NC008427 1	Ailuropoda melanoleuca NC009492 5	0,17016427
Lobodon carcinophaga NC008423 1	Caracal caracal NC028306 1	0,17016597
Pusa caspica NC008431 1	Homotherium latidens MF871702 3	0,17016675
Ommatophoca rossii AY377287etc 1	Bassariscus sumichrasti SRX1099089 1	0,17016748
Phoca largha NC008430 1	Catopuma badia NC028300 1	0,17017029
Monachus monachus NC044972 5	Meles anakuma NC009677 1	0,17017089
Ursus spelaeus EU327344 13	Pusa hispida NC 008433 1	0,17018181
Ursus spelaeus EU327344 13	Mirounga angustirostris SRR10331586 1	0,17018352
Ursus arctos isabellinus 18B5 2	Monachus monachus NC044972 5	0,17019153
Eupleres goudotii D128 1	Cystophora cristata NC008427 1	0,17021282
Mustela itatsi NC034330 19	Monachus schauinslandi NC008421 1	0,17022051
Panthera onca KP202264 2	Leptonychotes weddellii NC008424 1	0,17022142
Pusa hispida NC 008433 1	Fossa fossana D350 1	0,17022169
Hydrurga leptonyx NC008425 1	Helogale parvula SRR7637809 1	0,17022344
Viverricula indica NC025296 2	Phoca groenlandica NC008429 54	0,17022808
Pusa hispida NC 008433 1	Mustela nivalis T306 5	0,17023018
Viverricula indica KX891745 1	Mirounga angustirostris SRR10331586 1	0,17023784
Ursus arctos AP012576 6	Erignathus barbatus NC008426 1	0,17023863
Erignathus barbatus NC008426 1	Acinonyx jubatus NC005212 3	0,17023967
Ommatophoca rossii AY377287etc 1	Lutra sumatrana NC035810 1	0,17024317
Ursus arctos AP012576 6	Lobodon carcinophaga NC008423 1	0,17025374
Mirounga angustirostris SRR10331586 1	Leopardus colocolo NC028314 1	0,17025387
Ursus arctos AP012576 6	Hydrurga leptonyx NC008425 1	0,17025451
Phoca fasciata NC008428 1	Genetta abyssinica MG489822 1	0,17026508
Ursus thibetanus formosanus NC009331 1	Monachus monachus NC044972 5	0,17027007
Ursus arctos AP012576 6	Monachus monachus NC044972 5	0,17027093
Mustela sibirica AP017394 11	Mirounga leonina NC008422 1	0,17027648
Ursus arctos GU573486 5	Halichoerus grypus NC001602 2	0,17028052
Mirounga leonina NC008422 1	Martes martes T302 3	0,1702809
Phoca groenlandica NC008429 54	Martes americana NC020642 1	0,17028521
Phoca fasciata NC008428 1	Mustela sibirica NC020637 6	0,17028645
Phoca fasciata NC008428 1	Mustela putorius NC020638 4	0,1702866
Phoca largha NC008430 1	Meles anakuma NC009677 1	0,17028722
Phoca groenlandica NC008429 54	Fossa fossana D350 1	0,17028928
Viverra tangalunga MH464792 1	Phoca largha NC008430 1	0,1702902
Ursus arctos GU573491 207	Pusa sibirica NC008432 2	0,17029025
Ursus arctos EU497665 29	Pusa sibirica NC008432 2	0,17029035
Poecilogale albinucha T602 1	Bassariscus sumichrasti SRX1099089 1	0,17029078
Prionailurus rubiginosus NC028304 2	Cystophora cristata NC008427 1	0,17029259
Viverricula indica NC025296 2	Monachus schauinslandi NC008421 1	0,1703015
Ursus spelaeus EU327344 13	Pusa sibirica NC008432 2	0,17030296
Leopardus colocolo NC028314 1	Cystophora cristata NC008427 1	0,17031175
Ursus arctos pruinosus MG066703 3	Lobodon carcinophaga NC008423 1	0,17031284
Ursus spelaeus NC011112 8	Phoca vitulina NC001325 1	0,17031467
Mustela kathiah NC023210 1	Halichoerus grypus NC001602 2	0,17032181
Mustela eversmannii NC028013 1	Halichoerus grypus NC001602 2	0,17032596
Ursus thibetanus mupinensis NC008753 2	Monachus monachus NC044972 5	0,17032933
Monachus monachus NC044972 5	Ailuropoda melanoleuca NC009492 5	0,17033492
Lobodon carcinophaga NC008423 1	Civettictis civetta NC033378 1	0,17033783
Ursus thibetanus laniger MH281753 2	Monachus monachus NC044972 5	0,17033842
Catopuma badia NC028300 1	Canis adustus KT448271 1	0,1703416
Mustela nigripes NC024942 1	Erignathus barbatus NC008426 1	0,17034435
Potos flavus T414 1	Ailurus fulgens styani NC009691 1	0,17034742
Phoca vitulina NC001325 1	Mustela kathiah NC023210 1	0,17035659
Erignathus barbatus NC008426 1	Catopuma badia NC028300 1	0,1703583
Prionailurus bengalensis CKM45 20	Erignathus barbatus NC008426 1	0,17036235
Hydrurga leptonyx NC008425 1	Galidictis fasciata DM333 1	0,17036399
Lynx canadensis NC028313 1	Lobodon carcinophaga NC008423 1	0,17036764
Ursus arctos isabellinus 18B5 2	Pusa hispida NC 008433 1	0,17037103
Mirounga leonina NC008422 1	Helarctos malayanus NC009968 2	0,17037201
Phoca vitulina NC001325 1	Leopardus pardalis NC028315 1	0,1703753
Mirounga leonina NC008422 1	Genetta abyssinica MG489822 1	0,17038591
Mirounga leonina NC008422 1	Martes americana NC020642 1	0,17041107
Pusa sibirica NC008432 2	Mustela sibirica AP017394 11	0,17041261
Pusa sibirica NC008432 2	Mustela sibirica NC020637 6	0,17041272
Mustela kathiah NC023210 1	Ailurus fulgens styani NC009691 1	0,17041765
Mustela nivalis T306 5	Ailurus fulgens NC011124 1	0,17042449
Mungos mungo MMC7 1	Lobodon carcinophaga NC008423 1	0,17042536
Phoca groenlandica NC008429 54	Aonyx cinerea NC035814 2	0,17042693
Procyon lotor AB462049 4	Lobodon carcinophaga NC008423 1	0,17042919
Ursus arctos GU573486 5	Mirounga leonina NC008422 1	0,17043255

Ursus arctos GU573486 5	Hyaena hyaena NC020669 1	0,18848273
Panthera onca KP202264 2	Otocyon megalotis SAF1 2	0,18848288
Prionodon pardicolor NC024569 2	Canis mesomelas KT448280 1	0,18848288
Vulpes lagopus NC026529 3	Arctictis binturong T605 2	0,18848309
Ursus thibetanus laniger MH281753 2	Bdeogale nigripes GLC15 1	0,18848334
Ursus arctos isabellinus 18B5 2	Felis margarita NC028308 1	0,18848465
Martes foina NC020643 1	Lycaon pictus NC028427 2	0,18848486
Helogale parvula SRR7637809 1	Helarctos malayanus NC009968 2	0,18848501
Ursus arctos GU573491 207	Prionailurus bengalensis NC028301 12	0,18848613
Parahyaena brunnea NC038159 15	Mustela altaica NC021751 1	0,18848634
Ictonyx striatus T299 1	Diplogale hosei MH464790 1	0,18848638
Leopardus pardalis NC028315 1	Ailuropoda melanoleuca NC009492 5	0,18848996
Galictis vittata T412 1	Galerella sanguinea T378 1	0,18849017
Neovison vison NC020641 3	Crocuta crocuta NC020670 3	0,18849021
Prionailurus viverrinus NC028305 1	Ailuropoda melanoleuca NC009492 5	0,18849063
Panthera leo spelaea KX258452 2	Otocyon megalotis SAF1 2	0,18849289
Mephitis mephitis NC020648 1	Caracal caracal NC028306 1	0,18849328
Ursus arctos AP012576 6	Genetta genetta T297 1	0,18849523
Ursus thibetanus laniger MH281753 2	Panthera uncia KP202269 1	0,18849583
Ursus thibetanus mupinensis NC00875	Mephitis mephitis NC020648 1	0,1885004
Gulo gulo NC009685 3	Canis anthus NC027956 2	0,18850465
Speothos venaticus C48 2	Leopardus colocolo NC028314 1	0,1885051
Gulo gulo NC009685 3	Galidia elegans D146 1	0,18850587
Ursus thibetanus formosanus NC0093	Lycalopex sechurae KT448284 1	0,18851233
Procyon lotor AB462049 4	Panthera leo NERO 19	0,18851446
Ursus americanus JX196366 3	Leopardus jacobita NC028322 1	0,18851636
Puma yagouaroundi NC028311 1	Arctocephalus forsteri NC004023 28	0,18851888
Martes melampus NC009678 1	Helogale parvula SRR7637809 1	0,18852027
Suricata suricatta SSM10 1	Martes melampus NC009678 1	0,18852028
Lutra sumatrana NC035810 1	Urva brachyura KY117547 1	0,18852188
Panthera pardus japonensis KJ866876	Eumetopias jubatus NC004030 10	0,18852212
Ursus arctos GU573486 5	Spilogale putorius NC010497 1	0,18852219
Ursus americanus JX196366 3	Lycaon pictus NC028427 2	0,18852313
Ursus thibetanus laniger MH281753 2	Canis mesomelas KT448280 1	0,18852453
Martes americana NC020642 1	Urva brachyura KY117547 1	0,18852463
Melogale moschata V0735A 1	Cynictis penicillata T375 1	0,18852477
Xenogale naso C07XAR110 1	Arctocephalus australis MG023139 1	0,18852654
Vulpes ferrilata NC027935 1	Martes americana NC020642 1	0,18852717
Bdeogale nigripes GLC15 1	Ailurus fulgens styani NC009691 1	0,18852805
Salanoia concolor D378 1	Otaria byronia OTAB 1	0,18853207
Nasua nasua NC020647 1	Ailurus fulgens styani NC009691 1	0,18853264
Vulpes corsac NC023958 1	Paguma larvata PDDS11 2	0,18853605
Prionodon pardicolor NC024569 2	Lontra canadensis SRR10409165 1	0,18853673
Melogale moschata NC020644 1	Canis anthus NC027956 2	0,18853675
Panthera uncia NC010638 1	Mustela putorius NC020638 4	0,18853867
Lutrogale perspicillata NC035811 1	Civettictis civetta GLC19 1	0,18853918
Viverricula indica KX891751 1	Urocyon cinereoargenteus NC026723 2	0,18853986
Tremarctos ornatus NC009969 2	Pardofelis marmorata NLN3 2	0,18854111
Urocyon littoralis catalinae KP129018	Lynx canadensis NC028313 1	0,1885419
Ursus arctos GU573491 207	Salanoia concolor D378 1	0,18854244
Panthera tigris amoyensis NC014770 2	Ailurus fulgens NC011124 1	0,18854256
Proteles cristata T393 6	Melogale moschata KP726273 1	0,18854287
Pardofelis marmorata NLN3 2	Neophoca cinerea NC008419 1	0,18854305
Paradoxurus jerdoni MH464793 1	Arctotherium sp NC030174 1	0,18854322
Parahyaena brunnea NC038159 15	Meles anakuma NC009677 1	0,18854327
Ursus arctos pruinosus MG066703 3	Lutrogale perspicillata NC035811 1	0,18854426
Vulpes corsac NC023958 1	Leopardus guigna NC028321 1	0,18854463
Procyon lotor AB462049 4	Lycalopex sechurae KT448284 1	0,18854539
Procyon lotor AB462046 3	Canis mesomelas KT448280 1	0,18854542
Meles leucurus NC039173 4	Crocuta crocuta NC020670 3	0,18854621
Catopuma badia NC028300 1	Bassaricyon neblina SRX1097850 1	0,18854733
Pardofelis marmorata NLN3 2	Canis mesomelas KT448280 1	0,18854766
Lutrogale perspicillata NC035811 1	Leptailurus serval NC028316 1	0,1885477
Panthera uncia KP202269 1	Martes pennanti NC020664 16	0,188548
Proteles cristata T393 6	Arctocephalus pusillus NC008417 1	0,18854974
Zalophus wollebaeki SRR4431565 1	Diplogale hosei MH464790 1	0,18855017
Mephitis mephitis NC020648 1	Aonyx cinerea NC035814 2	0,1885519
Ursus arctos GU573491 207	Leopardus wiedii NC028318 1	0,1885521
Ursus maritimus NC003428 31	Leopardus tigrinus NC028317 1	0,18855251
Ursus arctos GU573491 207	Leptailurus serval NC028316 1	0,18855377
Ursus arctos GU573491 207	Prionailurus bengalensis CKM45 20	0,18855645
Ursus arctos EU497665 29	Leopardus guigna NC028321 1	0,18855669
Spilogale putorius NC010497 1	Civettictis civetta NC033378 1	0,18855704
Ursus arctos GU573486 5	Leopardus jacobita NC028322 1	0,18855894
Panthera uncia NC010638 1	Canis aureus KT448274 1	0,18855989
Procyon lotor AB462049 4	Urva javanica/auropunctata NC006835	0,18856779
Zalophus californianus NC008416 1	Diplogale hosei MH464790 1	0,18856782
Martes melampus NC009678 1	Cuon alpinus NC013445 3	0,18856801
Ursus arctos EU497665 29	Canis aureus KT448274 1	0,18857141
Eupleres goudotii D128 1	Tapirus terrestris T358	0,18857764
Procyon lotor AB462046 3	Canis lupus chanco NC010340 4	0,18857786
Mephitis mephitis NC020648 1	Ailuropoda melanoleuca NC009492 5	0,18857811
Martes pennanti NC020664 16	Cuon alpinus NC013445 3	0,18858045
Ursus americanus JX196366 3	Lynx canadensis NC028313 1	0,18858148
Hyaena hyaena NC020669 1	Gulo gulo NC009685 3	0,18858297
Canis mesomelas KT448280 1	Ailurus fulgens NC011124 1	0,18858433
Ursus arctos AP012576 6	Canis aureus KT448274 1	0,18858466
Panthera pardus NC010641 5	Canis anthus NC027956 2	0,18858477
Panthera tigris amoyensis NC014770 2	Canis lupus familiaris NC002008 1231	0,18858525
Paguma larvata PDDS11 2	Tapirus terrestris T358	0,18858984
Ursus thibetanus mupinensis NC00875	Panthera pardus NC010641 5	0,18859108
Martes americana NC020642 1	Helogale parvula SRR7637809 1	0,18859124
Canis aureus KT448274 1	Ailuropoda melanoleuca NC009492 5	0,18859144
Suricata suricatta SSM10 1	Mustela nigripes NC024942 1	0,1885931
Vulpes vulpes NC008434 5	Xenogale naso C07XAR110 1	0,18859392
Otaria byronia OTAB 1	Galidictis fasciata DM333 1	0,18859927
Panthera onca KP202264 2	Ailurus fulgens styani NC009691 1	0,18860137
Panthera pardus japonensis KJ866876	Meles meles T303 3	0,18860188
Bdeogale nigripes GLC15 1	Arctotherium sp NC030174 1	0,18860191
Felis nigripes NC028309 1	Conepatus chinga NC042596 1	0,18860358
Mustela altaica NC021751 1	Helogale parvula SRR7637809 1	0,18860362
Martes americana NC020642 1	Lycalopex sechurae KT448284 1	0,18860584
Neophoca cinerea NC008419 1	Cryptoprocta ferox CFC13 1	0,18860666

Lobodon carcinophaga NC008423 1	Felis silvestris lybica KP202275 4	0,17043503
Ursus maritimus GU573488 Svalbard	Phoca vitulina NC001325 1	0,17043668
Ursus spelaeus NC011112 8	Pusa caspica NC008431 1	0,17043772
Ursus arctos pruinosus MG066703 3	Phoca fasciata NC008428 1	0,1704386
Pusa hispida NC 008433 1	Prionailurus bengalensis CKM45 20	0,17043974
Ursus thibetanus formosanus NC009331 1	Monachus schauinslandi NC008421 1	0,1704516
Mustela nivalis T306 5	Cystophora cristata NC008427 1	0,17048889
Mirounga leonina NC008422 1	Leopardus wiedii NC028318 1	0,17049184
Viverra zibetha T609 1	Mirounga leonina NC008422 1	0,17049186
Lobodon carcinophaga NC008423 1	Leptailurus serval NC028316 1	0,17050229
Lycaon pictus NC028427 2	Lobodon carcinophaga NC008423 1	0,17050461
Ursus arctos pruinosus MG066703 3	Phoca groenlandica NC008429 54	0,17050616
Ursus arctos GU573486 5	Mirounga angustirostris SRR10331586 1	0,17051348
Phoca groenlandica NC008429 54	Martes flavigula NC012141 3	0,17052531
Neovison vison NC020641 3	Halichoerus grypus NC001602 2	0,17053622
Halichoerus grypus NC001602 2	Aonyx cinerea NC035814 2	0,17053869
Mustela sibirica NC020637 6	Erignathus barbatus NC008426 1	0,17054595
Ursus spelaeus EU327344 13	Halichoerus grypus NC001602 2	0,17055926
Felis silvestris lybica KP202275 4	Erignathus barbatus NC008426 1	0,17055976
Leopardus jacobita NC028322 1	Erignathus barbatus NC008426 1	0,1705624
Salanoia concolor D378 1	Hydrurga leptonyx NC008425 1	0,17056561
Lynx pardinus NC028319 161	Lobodon carcinophaga NC008423 1	0,17056973
Phoca fasciata NC008428 1	Catopuma badia NC028300 1	0,17056975
Phoca fasciata NC008428 1	Leopardus pardalis NC028315 1	0,17057054
Lutrogale perspicillata NC035811 1	Hydrurga leptonyx NC008425 1	0,17057069
Ursus arctos AP012576 6	Monachus schauinslandi NC008421 1	0,17059079
Prionailurus bengalensis CKM45 20	Monachus monachus NC044972 5	0,17059472
Phoca largha NC008430 1	Leopardus colocolo NC028314 1	0,17059501
Ommatophoca rossii AY377287etc 1	Mustela frenata NC020640 1	0,17059766
Erignathus barbatus NC008426 1	Chrotogale owstoni T607 1	0,17060965
Mustela putorius NC020638 4	Erignathus barbatus NC008426 1	0,17061342
Leptonychotes weddellii NC008424 1	Galerella sanguinea T378 1	0,17062555
Poecilogale albinucha T602 1	Ailurus fulgens styani NC009691 1	0,17062671
Phoca groenlandica NC008429 54	Martes pennanti NC020664 16	0,17062716
Felis margarita NC028308 1	Erignathus barbatus NC008426 1	0,17062742
Nandinia binotata NC024567 1	Martes foina NC020643 1	0,17062795
Mirounga angustirostris SRR10331586 1	Fossa fossana D350 1	0,17062834
Hydrurga leptonyx NC008425 1	Canis latrans NC008093 7	0,17062902
Lobodon carcinophaga NC008423 1	Canis latrans NC008093 7	0,17062947
Mirounga angustirostris SRR10331586 1	Martes foina NC020643 1	0,17063813
Ursus maritimus NC003428 31	Phoca vitulina NC001325 1	0,1706387
Mirounga angustirostris SRR10331586 1	Leopardus pardalis T262 1	0,17063928
Ursus spelaeus NC011112 8	Pusa sibirica NC008432 2	0,17063963
Hydrurga leptonyx NC008425 1	Cryptoprocta ferox CFC13 1	0,17064302
Pusa hispida NC 008433 1	Acinonyx jubatus NC005212 3	0,17065454
Genetta abyssinica MG489822 1	Cystophora cristata NC008427 1	0,17067011
Monachus monachus NC044972 5	Melursus ursinus NC009970 2	0,17067714
Viverra tangalunga MH464792 1	Cystophora cristata NC008427 1	0,17068216
Pusa sibirica NC008432 2	Mustela putorius NC020638 4	0,17068224
Mustela altaica NC021751 1	Ailurus fulgens NC011124 1	0,17068876
Phoca groenlandica NC008429 54	Martes martes T302 3	0,17069356
Mustela sibirica AP017394 11	Mirounga angustirostris SRR10331586 1	0,17069377
Viverricula indica KX891745 1	Phoca groenlandica NC008429 54	0,17069557
Mirounga angustirostris SRR10331586 1	Martes pennanti NC020664 16	0,17069669
Viverricula indica NC025296 2	Cystophora cristata NC008427 1	0,17069755
Smilodon populator MF871700 1	Leptonychotes weddellii NC008424 1	0,1706987
Paguma larvata PDDS11 2	Hydrurga leptonyx NC008425 1	0,17070058
Mirounga angustirostris SRR10331586 1	Ailurus fulgens NC011124 1	0,17070211
Lynx rufus NC014456 3	Lobodon carcinophaga NC008423 1	0,17070457
Monachus monachus NC044972 5	Enhydra lutris NC009692 1	0,1707076
Ursus arctos GU573491 207	Pusa hispida NC 008433 1	0,17070794
Mirounga leonina NC008422 1	Martes flavigula NC012141 3	0,17071344
Proteles cristata T393 6	Paradoxurus jerdoni MH464793 1	0,17071378
Pusa caspica NC008431 1	Leopardus colocolo NC028314 1	0,17071679
Ursus thibetanus thibetanus NC011118 4	Monachus schauinslandi NC008421 1	0,17072316
Melogale moschata V0735A 1	Erignathus barbatus NC008426 1	0,17074725
Phoca groenlandica NC008429 54	Meles anakuma NC009677 1	0,17075516
Phoca groenlandica NC008429 54	Mustela putorius NC020638 4	0,17075835
Pusa caspica NC008431 1	Mustela nivalis T306 5	0,17075838
Lynx rufus NC014456 3	Erignathus barbatus NC008426 1	0,17076105
Ursus arctos EU497665 29	Pusa caspica NC008431 1	0,17076166
Ursus maritimus GU573488 Svalbard	Mirounga leonina NC008422 1	0,17076261
Viverricula indica KX891751 1	Phoca groenlandica NC008429 54	0,17076291
Leptonychotes weddellii NC008424 1	Genetta genetta T297 1	0,17076293
Ursus arctos GU573491 207	Phoca fasciata NC008428 1	0,1707688
Lobodon carcinophaga NC008423 1	Felis nigripes NC028309 1	0,17077222
Proteles cristata T393 6	Paradoxurus hermaphroditus NC039591 1	0,17077264
Pusa hispida NC 008433 1	Leptailurus serval NC028316 1	0,1707736
Prionailurus viverrinus NC028305 1	Lobodon carcinophaga NC008423 1	0,17077406
Ursus maritimus GU573488 Svalbard	Mirounga angustirostris SRR10331586 1	0,17077628
Ursus spelaeus NC011112 8	Pusa hispida NC 008433 1	0,17078796
Monachus monachus NC044972 5	Catopuma badia NC028300 1	0,17079307
Mirounga angustirostris SRR10331586 1	Canis adustus KT448271 1	0,17081194
Ursus maritimus GU573488 Svalbard	Halichoerus grypus NC001602 2	0,17081323
Mustela itatsi NC034330 19	Mirounga leonina NC008422 1	0,17081596
Leptonychotes weddellii NC008424 1	Bassaricyon neblina SRX1097850 1	0,17081681
Meles anakuma NC009677 1	Ailurus fulgens NC011124 1	0,17081791
Salanoia concolor D378 1	Leptonychotes weddellii NC008424 1	0,1708221
Phoca vitulina NC001325 1	Melogale moschata V0735A 1	0,17082785
Viverra zibetha T609 1	Erignathus barbatus NC008426 1	0,17082828
Ursus arctos isabellinus 18B5 2	Pusa caspica NC008431 1	0,1708288
Mirounga leonina NC008422 1	Catopuma badia NC028300 1	0,17082882
Prionailurus rubiginosus NC028304 2	Erignathus barbatus NC008426 1	0,17082995
Ursus arctos pruinosus MG066703 3	Erignathus barbatus NC008426 1	0,17083634
Phoca vitulina NC001325 1	Mustela nivalis T306 5	0,17083767
Lobodon carcinophaga NC008423 1	Catopuma temminckii NC027115 41	0,17083926
Pusa hispida NC 008433 1	Prionailurus bengalensis NC028301 12	0,17084041
Mirounga angustirostris SRR10331586 1	Melursus ursinus NC009970 2	0,17084181
Pusa caspica NC008431 1	Canis adustus KT448271 1	0,17084348
Monachus monachus NC044972 5	Lutra sumatrana NC035810 1	0,17084572
Ursus arctos pruinosus MG066703 3	Hydrurga leptonyx NC008425 1	0,17085215
Ursus arctos AP012576 6	Phoca largha NC008430 1	0,17085594
Monachus monachus NC044972 5	Caracal caracal NC028306 1	0,17085855

Vulpes corsac NC023958 1	Leopardus jacobita NC028322 1	0,18860814
Leopardus colocolo NC028314 1	Arctocephalus forsteri NC004023 28	0,18860941
Urocyon cinereoargenteus NC026723 2	Martes foina NC020643 1	0,18861018
Neophoca cinerea NC008419 1	Catopuma temminckii NC027115 41	0,18861061
Urocyon cinereoargenteus NC026723 2	Prionailurus bengalensis NC028301 12	0,18861125
Lynx rufus NC014456 3	Lutrogale perspicillata NC035811 1	0,18861248
Viverra tangalunga MH464792 1	Proteles cristata T393 6	0,18861346
Procyon lotor AB462049 4	Pardofelis marmorata NLN3 2	0,18861359
Urocyon littoralis catalinae KP129018	Prionailurus bengalensis CKM45 20	0,18861412
Poecilogale albinucha T602 1	Pardofelis marmorata NLN3 2	0,18861434
Helarctos malayanus NC009968 2	Eupleres goudotii D128 1	0,18861453
Proteles cristata T393 6	Mustela nigripes NC024942 1	0,18861496
Vulpes ferrilata NC027935 1	Panthera tigris amoyensis NC014770 2	0,18861543
Panthera tigris NC010642 35	Martes americana NC020642 1	0,18861669
Speothos venaticus C48 2	Arctotherium sp NC030174 1	0,18861905
Ursus arctos isabellinus 18B5 2	Felis chaus NC028307 1	0,18861985
Ursus thibetanus thibetanus NC011118	Mungos mungo/gambianus SRR77048	0,18862024
Vulpes zerda KJ603240 1	Ommatophoca rossii AY377287etc 1	0,18862042
Poecilogale albinucha T602 1	Diplogale hosei MH464790 1	0,18862069
Paradoxurus hermaphroditus NLNC 1	Otaria byronia OTAB 1	0,18862237
Mungos mungo MMC7 1	Mephitis mephitis NC020648 1	0,18862306
Mephitis mephitis NC020648 1	Fossa fossana D350 1	0,18862435
Panthera uncia KP202269 1	Chrysocyon brachyurus NC024172 1	0,18862566
Panthera pardus NC010641 5	Gulo gulo NC009685 3	0,18862574
Felis catus NC001700 2	Ailuropoda melanoleuca NC009492 5	0,18862584
Odobenus rosmarus NC004029 29	Martes martes T302 3	0,18862726
Ursus thibetanus thibetanus NC011118	Galerella sanguinea T378 1	0,18862888
Hyaena hyaena NC020669 1	Helarctos malayanus NC009968 2	0,18862905
Ursus americanus JX196366 3	Canis lupus chanco NC010340 4	0,18862927
Galictis vittata T412 1	Caracal caracal NC028306 1	0,18863231
Neofelis nebulosa NC008450 3	Meles meles T303 3	0,18863916
Bdeogale nigripes GLC15 1	Arctocephalus forsteri NC004023 28	0,18864068
Ursus arctos pruinosus MG066703 3	Canis aureus KT448274 1	0,18864173
Ursus arctos pruinosus MG066703 3	Arctocephalus forsteri NC004023 28	0,18864469
Canis lupus chanco NC010340 4	Ailurus fulgens NC011124 1	0,18864714
Panthera leo NERO 19	Melogale moschata NC020644 1	0,18864816
Zalophus californianus NC008416 1	Canis aureus KT448274 1	0,18864854
Panthera leo NERO 19	Mustela altaica NC021751 1	0,18864873
Ursus spelaeus EU327344 13	Odobenus rosmarus NC004029 29	0,18865092
Ursus americanus JX196366 3	Prionailurus planiceps NC028312 6	0,18865135
Urocyon littoralis catalinae KP129018	Ommatophoca rossii AY377287etc 1	0,18865314
Urva brachyura KY117547 1	Tapirus terrestris T358	0,18865365
Urva semitorquata MH464789 1	Conepatus chinga NC042596 1	0,18865557
Vulpes corsac NC023958 1	Enhydra lutris NC009692 1	0,18865962
Prionodon linsang ERR2391707 1	Lutra sumatrana NC035810 1	0,18865974
Urva brachyura KY117547 1	Arctocephalus australis MG023139 1	0,1886602
Vulpes vulpes NC008434 5	Martes zibellina NC011579 39	0,18866083
Tremarctos ornatus NC009969 2	Bdeogale nigripes GLC15 1	0,18866181
Paradoxurus hermaphroditus NLNC 1	Martes melampus NC009678 1	0,18866372
Ursus spelaeus NC011112 8	Panthera leo NERO 19	0,18866581
Nyctereutes procyonoides NC013700 3	Mustela itatsi NC034330 19	0,18866651
Potos flavus T414 1	Galidia elegans D146 1	0,18866661
Prionodon linsang ERR2391707 1	Nyctereutes procyonoides NC013700 3	0,18866673
Procyon lotor AB462046 3	Cynictis penicillata T375 1	0,18866681
Helarctos malayanus NC009968 2	Diplogale hosei MH464790 1	0,188667
Lutra lutra NC011358 9	Chrysocyon brachyurus NC024172 1	0,18866961
Canis lupus chanco NC010340 4	Ailuropoda melanoleuca NC009492 5	0,18867087
Vulpes ferrilata NC027935 1	Arctodus simus NC011116 1	0,1886715
Mustela eversmannii NC028013 1	Hyaena hyaena NC020669 1	0,18867272
Viverricula indica KX891745 1	Phocarctos hookeri NC008418 1	0,18867308
Panthera uncia KP202269 1	Lycalopex sechurae KT448284 1	0,18867366
Lutrogale perspicillata NC035811 1	Genetta servalina NC024568 2	0,18867382
Ursus arctos AP012576 6	Canis lupus chanco NC010340 4	0,18867423
Felis chaus NC028307 1	Arctocephalus australis MG023139 1	0,18867442
Felis catus NC001700 2	Arctocephalus gazella BK010918 1	0,18867563
Ursus arctos EU497665 29	Salanoia concolor D378 1	0,18867727
Ursus arctos EU497665 29	Hyaena hyaena NC020669 1	0,18867861
Lutra sumatrana NC035810 1	Chrysocyon brachyurus NC024172 1	0,18867888
Phocarctos hookeri NC008418 1	Caracal caracal NC028306 1	0,18867891
Ursus thibetanus mupinensis NC00875	Hyaena hyaena NC020669 1	0,18868062
Poecilogale albinucha T602 1	Galerella sanguinea T378 1	0,18868078
Lutra sumatrana NC035810 1	Arctictis binturong T605 2	0,18868153
Panthera uncia NC010638 1	Mustela nivalis T306 5	0,18868306
Ursus thibetanus formosanus NC00933	Lutrogale perspicillata NC035811 1	0,1886839
Panthera tigris NC010642 35	Canis latrans NC008093 7	0,18868533
Ursus thibetanus thibetanus NC011118	Xenogale naso C07XAR110 1	0,18868683
Panthera leo spelaea KX258452 2	Callorhinus ursinus NC008415 1	0,18868686
Ursus arctos EU497665 29	Leopardus tigrinus NC028317 1	0,18868715
Speothos venaticus C48 2	Lynx pardinus NC028319 161	0,18868746
Ursus arctos pruinosus MG066703 3	Prionailurus planiceps KY682741 4	0,18868746
Profelis aurata NC028299 1	Otaria byronia OTAB 1	0,18868758
Paradoxurus hermaphroditus NLNC 1	Martes foina NC020643 1	0,18868768
Prionailurus planiceps KY682741 4	Mephitis mephitis NC020648 1	0,18869317
Ursus arctos GU573486 5	Felis margarita NC028308 1	0,18869337
Urocyon littoralis catalinae KP129018	Leopardus colocolo NC028314 1	0,18869355
Ursus thibetanus laniger MH281753 2	Galerella sanguinea T378 1	0,18869418
Ursus spelaeus EU327344 13	Prionailurus planiceps NC028312 6	0,18869567
Ursus spelaeus NC011112 8	Galerella sanguinea T378 1	0,18869571
Panthera tigris NC010642 35	Martes foina NC020643 1	0,18869768
Mungos mungo/gambianus SRR77048	Gulo gulo NC009685 3	0,1886998
Ursus arctos GU573491 207	Canis aureus KT448274 1	0,18870592
Procyon lotor AB462049 4	Canis lupus chanco NC010340 4	0,18871193
Galictis vittata T412 1	Canis adustus KT448271 1	0,18871236
Panthera leo NERO 19	Martes martes T302 3	0,18871281
Taxidea taxus NC020646 1	Cuon alpinus NC013445 3	0,18871348
Urocyon cinereoargenteus NC026723 2	Ommatophoca rossii AY377287etc 1	0,18871787
Panthera leo NERO 19	Lycaon pictus NC028427 2	0,1887196
Helogale parvula SRR7637809 1	Tapirus terrestris T358	0,18872042
Ursus americanus JX196366 3	Panthera pardus japonensis KJ866876	0,1887207
Ursus spelaeus EU327344 13	Diplogale hosei MH464790 1	0,18872184
Potos flavus T414 1	Mellivora capensis T370 1	0,18872397
Viverra tangalunga MH464792 1	Suricata suricatta SSM10 1	0,18872792
Ursus maritimus GU573488 Svalbard	Canis lupus chanco NC010340 4	0,18872799

Monachus schauinslandi NC008421 1	Gulo gulo NC009685 3	0,17086326
Ursus maritimus NC003428 31	Halichoerus grypus NC001602 2	0,17088059
Homotherium latidens MF871702 3	Halichoerus grypus NC001602 2	0,17088069
Nandinia binotata NC024567 1	Meles meles T303 3	0,17088157
Phoca fasciata NC008428 1	Homotherium latidens MF871702 3	0,1708831
Erignathus barbatus NC008426 1	Ailurus fulgens NC011124 1	0,17088662
Phoca groenlandica NC008429 54	Martes zibellina NC011579 39	0,17089137
Mustela altaica NC021751 1	Mirounga leonina NC008422 1	0,17089172
Viverricula indica KX891751 1	Cystophora cristata NC008427 1	0,17089507
Ursus arctos GU573491 207	Pusa caspica NC008431 1	0,17089622
Ursus maritimus NC003428 31	Mirounga leonina NC008422 1	0,17089725
Felis silvestris lybica KP202275 4	Cystophora cristata NC008427 1	0,17089772
Profelis aurata NC028299 1	Lobodon carcinophaga NC008423 1	0,17090631
Lutrogale perspicillata NC035811 1	Lobodon carcinophaga NC008423 1	0,17090714
Pusa hispida NC 008433 1	Lynx rufus NC014456 3	0,17090805
Monachus schauinslandi NC008421 1	Martes foina NC020643 1	0,17090847
Ursus arctos pruinosus MG066703 3	Monachus schauinslandi NC008421 1	0,17091957
Phoca groenlandica NC008429 54	Leopardus colocolo NC028314 1	0,17092551
Monachus monachus NC044972 5	Canis adustus KT448271 1	0,17092782
Halichoerus grypus NC001602 2	Catopuma badia NC028300 1	0,1709417
Ursus arctos isabellinus 18B5 2	Halichoerus grypus NC001602 2	0,17094763
Nandinia binotata NC024567 1	Mustela nigripes NC024942 1	0,1709505
Lutrogale perspicillata NC035811 1	Leptonychotes weddellii NC008424 1	0,17095841
Pusa caspica NC008431 1	Neovison vison NC020641 3	0,17096062
Felis nigripes NC028309 1	Cystophora cristata NC008427 1	0,17096512
Tremarctos ornatus NC009969 2	Monachus schauinslandi NC008421 1	0,17096646
Phoca vitulina NC001325 1	Fossa fossana D350 1	0,1709677
Ursus arctos pruinosus MG066703 3	Pusa sibirica NC008432 2	0,1709699
Phoca fasciata NC008428 1	Lynx canadensis NC028313 1	0,17097129
Phoca fasciata NC008428 1	Lynx rufus NC014456 3	0,17097145
Viverricula indica NC025296 2	Pusa hispida NC 008433 1	0,17097348
Phoca fasciata NC008428 1	Leptailurus serval NC028316 1	0,170974
Phoca fasciata NC008428 1	Leopardus jacobita NC028322 1	0,17097524
Monachus schauinslandi NC008421 1	Leopardus geoffroyi NC028320 1	0,17097639
Ursus arctos isabellinus 18B5 2	Mirounga angustirostris SRR10331586 1	0,17097829
Phoca largha NC008430 1	Leopardus pardalis T262 1	0,17098194
Pusa sibirica NC008432 2	Acinonyx jubatus NC005212 3	0,17098331
Urva javanica/auropunctata NC006835 1	Cystophora cristata NC008427 1	0,1709853
Hydrurga leptonyx NC008425 1	Ailuropoda melanoleuca NC009492 5	0,17098691
Ommatophoca rossii AY377287etc 1	Meles meles T303 3	0,17099161
Phoca vitulina NC001325 1	Canis adustus KT448271 1	0,17100458
Phoca fasciata NC008428 1	Hemigalus derbyanus MH464791 1	0,17100567
Monachus monachus NC044972 5	Acinonyx jubatus NC005212 3	0,17100929
Mirounga leonina NC008422 1	Lutra sumatrana NC035810 1	0,17101167
Ictonyx striatus T299 1	Halichoerus grypus NC001602 2	0,17101382
Pusa sibirica NC008432 2	Mustela eversmannii NC028013 1	0,17101857
Procyon lotor AB462049 4	Mirounga leonina NC008422 1	0,17102417
Phoca groenlandica NC008429 54	Mustela sibirica AP017394 11	0,17102744
Mirounga leonina NC008422 1	Leopardus jacobita NC028322 1	0,17103069
Mirounga angustirostris SRR10331586 1	Crossarchus platycephalus C7R66 1	0,17103164
Lynx pardinus NC028319 161	Cystophora cristata NC008427 1	0,17103194
Lontra canadensis SRR10409165 1	Hydrurga leptonyx NC008425 1	0,17103234
Leopardus guigna NC028321 1	Erignathus barbatus NC008426 1	0,17103417
Pusa hispida NC 008433 1	Puma yagouaroundi NC028311 1	0,17104293
Ursus maritimus NC003428 31	Mirounga angustirostris SRR10331586 1	0,17104562
Phoca vitulina NC001325 1	Catopuma badia NC028300 1	0,17104582
Phoca vitulina NC001325 1	Leopardus pardalis T262 1	0,17104906
Ursus arctos AP012576 6	Phoca groenlandica NC008429 54	0,17105334
Lobodon carcinophaga NC008423 1	Galictis vittata T412 1	0,1710546
Mirounga leonina NC008422 1	Martes zibellina NC011579 39	0,17108446
Mustela nigripes NC024942 1	Mirounga leonina NC008422 1	0,1710851
Pusa caspica NC008431 1	Martes pennanti NC020664 16	0,17109065
Procyon lotor AB462046 3	Erignathus barbatus NC008426 1	0,1710909
Mellivora capensis T370 1	Galictis vittata T412 1	0,17109302
Phoca groenlandica NC008429 54	Mustela itatsi NC034330 19	0,1710957
Martes foina NC020643 1	Ailurus fulgens styani NC009691 1	0,17109627
Lynx lynx NC027083 4	Erignathus barbatus NC008426 1	0,17109758
Viverra tangalunga MH464792 1	Phoca vitulina NC001325 1	0,17109829
Pusa caspica NC008431 1	Ictonyx striatus T299 1	0,17109963
Leptonychotes weddellii NC008424 1	Cryptoprocta ferox CFC13 1	0,17110001
Pusa sibirica NC008432 2	Leptailurus serval NC028316 1	0,17110233
Ursus arctos pruinosus MG066703 3	Pusa caspica NC008431 1	0,1711046
Procyon lotor AB462046 3	Mirounga angustirostris SRR10331586 1	0,17110611
Viverra zibetha T609 1	Lobodon carcinophaga NC008423 1	0,17110677
Phoca largha NC008430 1	Mustela nivalis T306 5	0,17110687
Monachus schauinslandi NC008421 1	Leopardus jacobita NC028322 1	0,17110896
Phoca groenlandica NC008429 54	Leopardus pardalis T262 1	0,17110944
Mirounga angustirostris SRR10331586 1	Leopardus jacobita NC028322 1	0,17111094
Ursus arctos EU497665 29	Pusa hispida NC 008433 1	0,17111227
Ursus arctos AP012576 6	Phoca fasciata NC008428 1	0,17112087
Gulo gulo NC009685 3	Ailurus fulgens styani NC009691 1	0,17112486
Mirounga angustirostris SRR10331586 1	Helarctos malayanus NC009968 2	0,1711261
Hyaena hyaena NC020669 1	Cynogale bennetti KY117544 1	0,17114377
Erignathus barbatus NC008426 1	Canis adustus KT448271 1	0,17114615
Nandinia binotata NC024567 1	Arctonyx collaris NC020645 1	0,1711504
Lobodon carcinophaga NC008423 1	Chrotogale owstoni T607 1	0,17115152
Lobodon carcinophaga NC008423 1	Conepatus chinga NC042596 1	0,17115466
Mungos mungo/gambianus SRR7704821 1	Cystophora cristata NC008427 1	0,17115539
Procyon lotor AB462049 4	Melogale moschata V0735A 1	0,17115748
Leptonychotes weddellii NC008424 1	Galidictis fasciata DM333 1	0,17115921
Mustela altaica NC021751 1	Erignathus barbatus NC008426 1	0,17116295
Viverricula indica KX891745 1	Cystophora cristata NC008427 1	0,17116439
Pusa caspica NC008431 1	Mustela altaica NC021751 1	0,17116442
Lynx pardinus NC028319 161	Erignathus barbatus NC008426 1	0,17116545
Cystophora cristata NC008427 1	Catopuma badia NC028300 1	0,17116711
Felis chaus NC028307 1	Cystophora cristata NC008427 1	0,17116757
Pusa sibirica NC008432 2	Prionailurus bengalensis NC028301 12	0,17116946
Leopardus guigna NC028321 1	Cystophora cristata NC008427 1	0,1711697
Pusa sibirica NC008432 2	Prionailurus bengalensis CKM45 20	0,17117232
Potos flavus T414 1	Galictis vittata T412 1	0,17117555
Lynx lynx NC027083 4	Lobodon carcinophaga NC008423 1	0,17117581
Panthera onca NC022842 1	Leptonychotes weddellii NC008424 1	0,17117588
Phoca fasciata NC008428 1	Leopardus pardalis T262 1	0,17117688

Ursus arctos pruinosus MG066703 3	Canis lupus chanco NC010340 4	0,18872802
Urva semitorquata MH464789 1	Ailurus fulgens NC011124 1	0,18872909
Atilax paludinosus T606 1	Ailurus fulgens styani NC009691 1	0,18872914
Mungotictis decemlineata NC027828 1	Lontra canadensis SRR10409165 1	0,18872921
Vulpes ferrilata NC027935 1	Prionodon linsang ERR2391707 1	0,18872939
Vulpes lagopus NC026529 3	Tremarctos ornatus NC009969 2	0,18872954
Nyctereutes procyonoides NC013700 3	Arctocephalus forsteri KT693377 17	0,18873098
Panthera leo spelaea KX258452 2	Martes flavigula NC012141 3	0,188731
Neophoca cinerea NC008419 1	Cynictis penicillata T375 1	0,18873133
Vulpes vulpes NC008434 5	Mustela itatsi NC034330 19	0,18873279
Nyctereutes procyonoides NC013700 3	Neophoca cinerea NC008419 1	0,18873362
Vulpes zerda KJ603240 1	Mustela kathiah NC023210 1	0,18873362
Vulpes zerda KJ603240 1	Viverra tangalunga MH464792 1	0,18873437
Mungotictis decemlineata NC027828 1	Ailurus fulgens styani NC009691 1	0,18873458
Prionodon pardicolor NC024569 2	Tapirus terrestris T358	0,18873656
Hyaena hyaena NC020669 1	Arctonyx collaris NC020645 1	0,1887372
Vulpes lagopus NC026529 3	Martes pennanti NC020664 16	0,18873885
Ichneumia albicauda T603 1	Arctocephalus townsendi NC008420 1	0,18873949
Vulpes ferrilata NC027935 1	Martes martes T302 3	0,18874024
Urocyon cinereoargenteus NC026723 2	Martes martes T302 3	0,1887413
Urocyon littoralis catalinae KP129018 1	Genetta servalina NC024568 2	0,18874149
Phocarctos hookeri NC008418 1	Paradoxurus jerdoni MH464793 1	0,1887417
Ursus spelaeus EU327344 13	Canis lupus chanco NC010340 4	0,18874213
Ursus thibetanus mupinensis NC008753	Urva javanica T413 1	0,1887427
Ursus arctos pruinosus MG066703 3	Chrotogale owstoni T607 1	0,18874347
Martes americana NC020642 1	Crocuta crocuta NC020670 3	0,18874402
Ursus arctos isabellinus 18B5 2	Procyon lotor AB462046 3	0,18874651
Galerella sanguinea T378 1	Chrysocyon brachyurus NC024172 1	0,18874703
Procyon lotor AB462046 3	Panthera pardus japonensis KJ866876	0,18874849
Panthera uncia KP202269 1	Mustela altaica NC021751 1	0,188749
Vulpes ferrilata NC027935 1	Panthera tigris NC010642 35	0,18874994
Panthera tigris amoyensis NC014770 2	Martes americana NC020642 1	0,18875124
Panthera leo spelaea KX258452 2	Ailurus fulgens NC011124 1	0,18875241
Ursus thibetanus formosanus NC00933	Helogale parvula SRR7637809 1	0,18875304
Ursus arctos pruinosus MG066703 3	Lynx rufus NC014456 3	0,18875346
Ursus arctos GU573491 207	Prionailurus planiceps KY682741 4	0,18875438
Ursus maritimus GU573488 Svalbard	Prionailurus viverrinus NC028305 1	0,18875478
Ursus arctos EU497665 29	Prionailurus viverrinus NC028305 1	0,1887548
Ursus arctos pruinosus MG066703 3	Prionailurus viverrinus NC028305 1	0,18875496
Panthera onca KP202264 2	Lycaon pictus NC028427 2	0,18875499
Ursus americanus JX196366 3	Panthera pardus NC010641 5	0,18875921
Diplogale hosei MH464790 1	Arctocephalus pusillus NC008417 1	0,1887596
Ursus thibetanus formosanus NC00933	Paradoxurus hermaphroditus NC03959	0,18876156
Leopardus colocolo NC028314 1	Arctocephalus townsendi NC008420 1	0,18876229
Genetta genetta T297 1	Chrysocyon brachyurus NC024172 1	0,1887645
Mephitis mephitis NC020648 1	Lynx canadensis NC028313 1	0,18876524
Pardofelis marmorata NLN3 2	Galictis vittata T412 1	0,1887663
Melursus ursinus NC009970 2	Felis nigripes NC028309 1	0,18876843
Panthera tigris NC010642 35	Chrysocyon brachyurus NC024172 1	0,18876906
Neophoca cinerea NC008419 1	Urva javanica/auropunctata NC006835	0,1887707
Melogale moschata V0735A 1	Canis lupus chanco NC010340 4	0,18877119
Panthera leo NERO 19	Meles leucurus NC039173 4	0,18877283
Lycalopex sechurae KT448284 1	Urva semitorquata MH464789 1	0,18877648
Melogale moschata KP726273 1	Cuon alpinus NC013445 3	0,18877797
Vulpes corsac NC023958 1	Martes flavigula NC012141 3	0,18877886
Panthera pardus NC010641 5	Mustela eversmannii NC028013 1	0,18878274
Panthera pardus NC010641 5	Mustela itatsi NC034330 19	0,18878327
Panthera tigris NC010642 35	Canis aureus KT448274 1	0,18878482
Prionailurus viverrinus NC028305 1	Arctocephalus forsteri NC004023 28	0,18878832
Nyctereutes procyonoides NC013700 3	Lutra lutra LC050126 1	0,18879364
Ursus spelaeus EU327344 13	Canis mesomelas KT448280 1	0,18879424
Ursus arctos EU497665 29	Canis lupus chanco NC010340 4	0,18879567
Vulpes corsac NC023958 1	Urva brachyura KY117547 1	0,1887968
Cynictis penicillata T375 1	Arctocephalus gazella BK010918 1	0,18879689
Panthera pardus NC010641 5	Otocyon megalotis SAF1 2	0,18879689
Panthera leo NERO 19	Chrysocyon brachyurus NC024172 1	0,18879752
Felis chaus NC028307 1	Tapirus terrestris T358	0,18879803
Suricata suricatta SSM10 1	Martes martes T302 3	0,18879901
Paradoxurus hermaphroditus NLNC 1	Arctocephalus australis MG023139 1	0,1887999
Ursus thibetanus formosanus NC00933	Panthera pardus NC010641 5	0,18880238
Panthera uncia NC010638 1	Meles meles T303 3	0,1888037
Otocyon megalotis SAF1 2	Meles meles T303 3	0,18880434
Poecilogale albinucha T602 1	Cynictis penicillata T375 1	0,18880576
Urva javanica/auropunctata NC006835	Gulo gulo NC009685 3	0,18880753
Poecilogale albinucha T602 1	Canis anthus NC027956 2	0,18880786
Panthera leo NERO 19	Eumetopias jubatus NC004030 10	0,18880834
Viverricula indica KX891751 1	Urocyon littoralis catalinae KP129018 1	0,18880919
Ursus thibetanus mupinensis NC008753	Helogale parvula SRR7637809 1	0,18880925
Otaria byronia OTAB 1	Conepatus chinga NC042596 1	0,18881218
Phocarctos hookeri NC008418 1	Felis margarita NC028308 1	0,18881302
Panthera uncia KP202269 1	Arctocephalus pusillus NC008417 1	0,18881355
Speothos venaticus C48 2	Ailurus fulgens styani NC009691 1	0,18881401
Tremarctos ornatus NC009969 2	Paradoxurus hermaphroditus NLNC 1	0,18881404
Lutra lutra NC011358 9	Ailuropoda melanoleuca NC009492 5	0,18881443
Odobenus rosmarus NC004029 29	Lutra lutra NC011358 9	0,18881619
Vulpes vulpes NC008434 5	Panthera tigris NC010642 35	0,18881644
Diplogale hosei MH464790 1	Ailuropoda melanoleuca NC009492 5	0,18881796
Ursus arctos GU573491 207	Ichneumia albicauda T603 1	0,18881945
Vulpes corsac NC023958 1	Ursus thibetanus laniger MH281753 2	0,18881962
Ursus thibetanus formosanus NC00933	Bdeogale nigripes GLC15 1	0,18881984
Ursus maritimus NC003428 31	Panthera pardus japonensis KJ866876	0,1888205
Ursus thibetanus mupinensis NC008753	Panthera uncia KP202269 1	0,18882117
Panthera onca NC022842 1	Martes zibellina NC011579 39	0,18882123
Ursus arctos isabellinus 18B5 2	Prionailurus planiceps NC028312 6	0,18882141
Panthera onca NC022842 1	Canis anthus NC027956 2	0,18882158
Ursus arctos pruinosus MG066703 3	Leopardus wiedii NC028318 1	0,18882164
Ursus spelaeus NC011112 8	Mungos mungo/gambianus SRR77048	0,1888223
Ursus arctos GU573491 207	Prionailurus rubiginosus NC028304 2	0,18882255
Urva javanica/auropunctata NC006835	Tapirus terrestris T358	0,18882263
Speothos venaticus C48 2	Paradoxurus hermaphroditus NC03959	0,18882273
Ursus arctos pruinosus MG066703 3	Leptailurus serval NC028316 1	0,18882346
Ursus thibetanus thibetanus NC011118	Bassaricyon neblina SRX1097850 1	0,18882458
Salanoia concolor D378 1	Galictis vittata T412 1	0,18882486

Puma yagouaroundi NC028311 1	Phoca vitulina NC001325 1	0,17118193
Hydrurga leptonyx NC008425 1	Diplogale hosei MH464790 1	0,17121974
Leptonychotes weddellii NC008424 1	Bdeogale nigripes GLC15 1	0,17122273
Mustela sibirica AP017394 11	Ailurus fulgens styani NC009691 1	0,17122418
Mustela sibirica NC020637 6	Ailurus fulgens styani NC009691 1	0,17122422
Phoca groenlandica NC008429 54	Melogale moschata V0735A 1	0,1712278
Phoca fasciata NC008428 1	Ailurus fulgens NC011124 1	0,17123148
Phoca groenlandica NC008429 54	Ailurus fulgens NC011124 1	0,17123172
Ursus spelaeus NC011112 8	Halichoerus grypus NC001602 2	0,1712328
Felis margarita NC028308 1	Cystophora cristata NC008427 1	0,17123471
Leptailurus serval NC028316 1	Cystophora cristata NC008427 1	0,17123571
Hydrurga leptonyx NC008425 1	Bassaricyon neblina SRX1097850 1	0,17123716
Monachus schauinslandi NC008421 1	Catopuma badia NC028300 1	0,1712431
Leptonychotes weddellii NC008424 1	Galictis vittata T412 1	0,17124334
Lobodon carcinophaga NC008423 1	Felis margarita NC028308 1	0,1712434
Monachus schauinslandi NC008421 1	Leopardus guigna NC028321 1	0,17124574
Ursus americanus JX196366 3	Mirounga leonina NC008422 1	0,17126773
Ursus arctos GU573491 207	Halichoerus grypus NC001602 2	0,17128462
Mustela eversmannii NC028013 1	Erignathus barbatus NC008426 1	0,17128645
Mirounga leonina NC008422 1	Canis adustus KT448271 1	0,17128816
Mirounga angustirostris SRR10331586 1	Martes melampus NC009678 1	0,17129096
Felis catus NC001700 2	Erignathus barbatus NC008426 1	0,17130032
Prionailurus viverrinus NC028305 1	Erignathus barbatus NC008426 1	0,1713032
Pusa sibirica NC008432 2	Puma yagouaroundi NC028311 1	0,17130415
Prionailurus bengalensis NC028301 12	Phoca fasciata NC008428 1	0,17131048
Ursus arctos EU497665 29	Phoca vitulina NC001325 1	0,17131212
Pusa hispida NC 008433 1	Lynx lynx NC027083 4	0,17131251
Ursus arctos pruinosus MG066703 3	Pusa hispida NC 008433 1	0,17132072
Monachus schauinslandi NC008421 1	Helarctos malayanus NC009968 2	0,17132917
Ursus thibetanus laniger MH281753 2	Monachus schauinslandi NC008421 1	0,17133126
Cystophora cristata NC008427 1	Civettictis civetta NC033378 1	0,17133634
Halichoerus grypus NC001602 2	Fossa fossana D350 1	0,17133974
Leptonychotes weddellii NC008424 1	Urva semitorquata MH464789 1	0,17135739
Leptonychotes weddellii NC008424 1	Xenogale naso C07XAR110 1	0,17135739
Mungotictis decemlineata NC027828 1	Leptonychotes weddellii NC008424 1	0,17136064
Prionailurus planiceps NC028312 6	Mirounga leonina NC008422 1	0,17136556
Prionailurus planiceps KY682741 4	Mirounga leonina NC008422 1	0,17136566
Profelis aurata NC028299 1	Erignathus barbatus NC008426 1	0,17136885
Procyon lotor AB462049 4	Phoca groenlandica NC008429 54	0,17136943
Puma concolor NC016470 22	Cystophora cristata NC008427 1	0,17136967
Monachus monachus NC044972 5	Civettictis civetta NC033378 1	0,17136998
Viverricula indica NC025296 2	Pusa sibirica NC008432 2	0,17137067
Leopardus geoffroyi NC028320 1	Erignathus barbatus NC008426 1	0,1713708
Phoca groenlandica NC008429 54	Bassariscus sumichrasti SRX1099089 1	0,17137221
Mustela nivalis T306 5	Mirounga angustirostris SRR10331586 1	0,17137699
Prionailurus bengalensis NC028301 12	Monachus schauinslandi NC008421 1	0,17137699
Phoca vitulina NC001325 1	Mustela altaica NC021751 1	0,17137828
Phoca groenlandica NC008429 54	Leopardus jacobita NC028322 1	0,17137912
Mirounga angustirostris SRR10331586 1	Leopardus tigrinus NC028317 1	0,17137997
Phoca largha NC008430 1	Lynx lynx NC027083 4	0,17138292
Ursus arctos AP012576 6	Pusa sibirica NC008432 2	0,17138316
Phoca vitulina NC001325 1	Leopardus wiedii NC028318 1	0,17138571
Mirounga angustirostris SRR10331586 1	Ailuropoda melanoleuca NC009492 5	0,17139313
Leptonychotes weddellii NC008424 1	Canis aureus KT448274 1	0,17139329
Ommatophoca rossii AY377287etc 1	Arctonyx collaris NC020645 1	0,17139348
Monachus schauinslandi NC008421 1	Melursus ursinus NC009970 2	0,17139629
Ursus americanus JX196366 3	Phoca largha NC008430 1	0,17140362
Lutra lutra LC050126 1	Ailurus fulgens styani NC009691 1	0,1714116
Nandinia binotata NC024567 1	Mustela kathiah NC023210 1	0,17142166
Mirounga angustirostris SRR10331586 1	Lutra lutra LC050126 1	0,17142424
Pusa caspica NC008431 1	Ailurus fulgens styani NC009691 1	0,17142448
Pusa sibirica NC008432 2	Mustela nivalis T306 5	0,17143209
Prionodon linsang ERR2391707 1	Cryptoprocta ferox CFC13 1	0,17143415
Monachus schauinslandi NC008421 1	Martes zibellina NC011579 39	0,17143543
Mirounga angustirostris SRR10331586 1	Martes martes T302 3	0,17143697
Pusa sibirica NC008432 2	Leopardus pardalis T262 1	0,17144014
Parahyaena brunnea NC038159 15	Leptonychotes weddellii NC008424 1	0,17144109
Ursus arctos isabellinus 18B5 2	Phoca vitulina NC001325 1	0,17144664
Smilodon populator MF871700 1	Cystophora cristata NC008427 1	0,17144829
Ursus americanus JX196366 3	Pusa caspica NC008431 1	0,17145944
Monachus monachus NC044972 5	Felis silvestris lybica KP202275 4	0,17146617
Lobodon carcinophaga NC008423 1	Canis lupus familiaris NC002008 1231	0,17146689
Puma yagouaroundi NC028311 1	Monachus monachus NC044972 5	0,17146842
Lobodon carcinophaga NC008423 1	Canis lupus chanco NC010340 4	0,17147838
Ursus arctos EU497665 29	Halichoerus grypus NC001602 2	0,17148681
Procyon lotor AB462046 3	Melogale moschata V0735A 1	0,17149407
Phoca fasciata NC008428 1	Mustela eversmannii NC028013 1	0,17149822
Genetta abyssinica MG489822 1	Erignathus barbatus NC008426 1	0,17150073
Galerella sanguinea T378 1	Erignathus barbatus NC008426 1	0,17150106
Erignathus barbatus NC008426 1	Civettictis civetta GLC19 1	0,17150205
Monachus schauinslandi NC008421 1	Melogale moschata NC020644 1	0,17150635
Lobodon carcinophaga NC008423 1	Diplogale hosei MH464790 1	0,17150863
Viverricula indica KX891751 1	Monachus schauinslandi NC008421 1	0,17150927
Mustela nivalis T306 5	Monachus schauinslandi NC008421 1	0,17150974
Lobodon carcinophaga NC008423 1	Civettictis civetta GLC19 1	0,17151263
Proteles cristata T393 6	Prionodon linsang ERR2391707 1	0,17151362
Pusa hispida NC 008433 1	Leopardus pardalis NC028315 1	0,17151596
Phoca vitulina NC001325 1	Lynx lynx NC027083 4	0,17151743
Cystophora cristata NC008427 1	Acinonyx jubatus NC005212 3	0,17152078
Mephitis mephitis NC020648 1	Lobodon carcinophaga NC008423 1	0,17152224
Mustela kathiah NC023210 1	Monachus monachus NC044972 5	0,17152224
Otocolobus manul NC028323 1	Monachus monachus NC044972 5	0,17153329
Viverra tangalunga MH464792 1	Halichoerus grypus NC001602 2	0,17153829
Nandinia binotata NC024567 1	Meles leucurus NC039173 4	0,17155457
Mustela sibirica NC020637 6	Mirounga leonina NC008422 1	0,17155582
Mirounga leonina NC008422 1	Leopardus tigrinus NC028317 1	0,17156915
Mungos mungo/gambianus SRR7704821 1	Lobodon carcinophaga NC008423 1	0,17156936
Monachus schauinslandi NC008421 1	Melogale moschata KP726273 1	0,17156954
Mustela nigripes NC024942 1	Mirounga angustirostris SRR10331586 1	0,17156965
Prionailurus planiceps NC028312 6	Erignathus barbatus NC008426 1	0,17157014
Ursus arctos EU497665 29	Mirounga leonina NC008422 1	0,17157064
Leptonychotes weddellii NC008424 1	Hyaena hyaena NC020669 1	0,17157423
Mungotictis decemlineata NC027828 1	Hydrurga leptonyx NC008425 1	0,17157584

Panthera tigris amoyensis NC014770 2	Arctodus simus NC011116 1	0,18882601
Ursus thibetanus formosanus NC00933	Hyaena hyaena NC020669 1	0,18882611
Phocarctos hookeri NC008418 1	Hemigalus derbyanus MH464791 1	0,18882741
Ursus thibetanus thibetanus NC01111	Hyaena hyaena NC020669 1	0,18882777
Panthera onca NC022842 1	Otocyon megalotis SAF1 2	0,1888299
Panthera onca NC022842 1	Martes pennanti NC020664 16	0,1888302
Melogale moschata V0735A 1	Urva javanica/auropunctata NC006835	0,18883085
Ursus thibetanus thibetanus NC01111	Panthera uncia KP202269 1	0,18883342
Prionodon linsang ERR2391707 1	Martes flavigula NC012141 3	0,18883559
Melursus ursinus NC009970 2	Canis aureus KT448274 1	0,18883561
Ursus arctos AP012576 6	Caracal caracal NC028306 1	0,18883613
Ursus arctos AP012576 6	Leptailurus serval NC028316 1	0,18883649
Ursus arctos AP012576 6	Leopardus pardalis T262 1	0,18883796
Suricata suricatta SSM10 1	Eumetopias jubatus NC004030 10	0,18884148
Vulpes vulpes NC008434 5	Gulo gulo NC009685 3	0,1888416
Ursus maritimus GU573488 Svalbard	Canis aureus KT448274 1	0,18884405
Ursus thibetanus thibetanus NC01111	Chrysocyon brachyurus NC024172 1	0,18884639
Leopardus colocolo NC028314 1	Ailuropoda melanoleuca NC009492 5	0,18884706
Panthera pardus NC010641 5	Mustela sibirica AP017394 11	0,18884973
Arctonyx collaris NC020645 1	Tapirus terrestris T358	0,18885063
Ursus americanus JX196366 3	Felis catus NC001700 2	0,18885195
Otocyon megalotis SAF1 2	Gulo gulo NC009685 3	0,18885359
Panthera pardus NC010641 5	Aonyx cinerea NC035814 2	0,18885796
Suricata suricatta SSM10 1	Lutra sumatrana NC035810 1	0,18885852
Mungos mungo MMC7 1	Lontra canadensis SRR10409165 1	0,18886088
Cynictis penicillata T375 1	Arctocephalus townsendi NC008420 1	0,18886182
Nyctereutes procyonoides NC013700 3	Eupleres goudotii D128 1	0,18886249
Ursus maritimus NC003428 31	Canis lupus chanco NC010340 4	0,18886262
Ursus arctos GU573491 207	Canis lupus chanco NC010340 4	0,18886265
Vulpes corsac NC023958 1	Prionodon linsang ERR2391707 1	0,18886352
Vulpes lagopus NC026529 3	Meles meles T303 3	0,18886395
Urocyon cinereoargenteus NC026723 2	Meles meles T303 3	0,18886603
Viverra tangalunga MH464792 1	Prionodon linsang ERR2391707 1	0,18886672
Nyctereutes procyonoides NC013700 3	Arctocephalus gazella BK010918 1	0,18886729
Paradoxurus hermaphroditus NLNC 1	Arctocephalus forsteri KT693377 17	0,18886741
Vulpes ferrilata NC027935 1	Mustela eversmannii NC028013 1	0,18886743
Prionodon linsang ERR2391707 1	Ailurus fulgens styani NC009691 1	0,18886775
Paguma larvata PDDS11 2	Martes zibellina NC011579 39	0,18887141
Melursus ursinus NC009970 2	Canis lupus chanco NC010340 4	0,1888749
Mungotictis decemlineata NC027828 1	Arctotherium sp NC030174 1	0,18887588
Otocyon megalotis SAF1 2	Mustela itatsi NC034330 19	0,18887699
Panthera uncia KP202269 1	Nyctereutes procyonoides NC013700 3	0,18887794
Panthera tigris NC010642 35	Ailurus fulgens styani NC009691 1	0,18887913
Phocarctos hookeri NC008418 1	Felis silvestris lybica KP202275 4	0,18888007
Panthera onca KP202264 2	Lutrogale perspicillata NC035811 1	0,1888811
Taxidea taxus NC020646 1	Otocyon megalotis SAF1 2	0,18888198
Ursus arctos pruinosus MG066703 3	Crossarchus platycephalus C7R66 1	0,18888254
Cynictis penicillata T375 1	Chrysocyon brachyurus NC024172 1	0,18888289
Panthera uncia KP202269 1	Arctotherium sp NC030174 1	0,18888377
Ursus thibetanus mupinensis NC00875	Otocyon megalotis SAF1 2	0,18888536
Ursus thibetanus laniger MH281753 2	Mungos mungo/gambianus SRR77048	0,18888737
Ursus arctos isabellinus 18B5 2	Leopardus tigrinus NC028317 1	0,18888861
Speothos venaticus C48 2	Lynx canadensis NC028313 1	0,18888892
Ursus maritimus GU573488 Svalbard	Felis margarita NC028308 1	0,18888929
Galidia elegans D146 1	Canis lupus chanco NC010340 4	0,18888941
Speothos venaticus C48 2	Paradoxurus hermaphroditus NLNC 1	0,18888968
Panthera pardus japonensis KJ866876	Lycaon pictus NC028427 2	0,18889083
Paradoxurus hermaphroditus NC03959	Ictonyx striatus T299 1	0,18889213
Mephitis mephitis NC020648 1	Crossarchus platycephalus C7R66 1	0,18889344
Mephitis mephitis NC020648 1	Leopardus wiedii NC028318 1	0,18889403
Speothos venaticus C48 2	Leopardus geoffroyi NC028320 1	0,18889461
Urocyon littoralis catalinae KP129018	Acinonyx jubatus NC005212 3	0,18889584
Ursus arctos AP012576 6	Procyon lotor AB462046 3	0,18889584
Zalophus californianus NC008416 1	Suricata suricatta SSM10 1	0,18889633
Tremarctos ornatus NC009969 2	Canis aureus KT448274 1	0,18889682
Procyon lotor AB462049 4	Arctictis binturong T605 2	0,18889691
Melursus ursinus NC009970 2	Galerella sanguinea T378 1	0,18889701
Ursus thibetanus formosanus NC00933	Panthera onca KP202264 2	0,1888981
Ursus spelaeus EU327344 13	Parahyaena brunnea NC038159 15	0,1889004
Mephitis mephitis NC020648 1	Catopuma temminckii NC027115 41	0,18890235
Ursus arctos AP012576 6	Ichneumia albicauda T603 1	0,18890275
Canis lupus familiaris NC002008 1231	Arctocephalus pusillus NC008417 1	0,18890503
Profelis aurata NC028299 1	Melursus ursinus NC009970 2	0,18890549
Spilogale putorius NC010497 1	Aonyx cinerea NC035814 2	0,18891241
Ursus spelaeus NC011112 8	Canis aureus KT448274 1	0,18891262
Lontra canadensis SRR10409165 1	Civettictis civetta NC033378 1	0,18891419
Salanoia concolor D378 1	Arctocephalus forsteri NC004023 28	0,18891577
Ursus americanus JX196366 3	Ichneumia albicauda T603 1	0,18891607
Martes martes T302 3	Cuon alpinus NC013445 3	0,18891764
Melogale moschata V0735A 1	Helogale parvula SRR7637809 1	0,18892786
Nasua nasua NC020647 1	Callorhinus ursinus NC008415 1	0,18892857
Meles anakuma NC009677 1	Lycalopex sechurae KT448284 1	0,1889291
Suricata suricatta SSM10 1	Mustela sibirica NC020637 6	0,18892923
Vulpes vulpes NC008434 5	Prionodon linsang ERR2391707 1	0,18893056
Nyctereutes procyonoides NC013700 3	Melogale moschata KP726273 1	0,18893223
Neophoca cinerea NC008419 1	Xenogale naso C07XAR110 1	0,18893257
Urva brachyura KY117547 1	Ailurus fulgens NC011124 1	0,18893267
Vulpes zerda KJ603240 1	Enhydra lutris NC009692 1	0,18893327
Prionodon linsang ERR2391707 1	Ailurus fulgens NC011124 1	0,188935
Eumetopias jubatus NC004030 10	Arctictis binturong T605 2	0,18893518
Neophoca cinerea NC008419 1	Mungotictis decemlineata NC027828 1	0,18893524
Mungotictis decemlineata NC027828 1	Canis anthus NC027956 2	0,18893582
Mungos mungo MMC7 1	Lutrogale perspicillata NC035811 1	0,18893607
Panthera tigris amoyensis NC014770 2	Martes flavigula NC012141 3	0,18893759
Canis anthus NC027956 2	Ailurus fulgens styani NC009691 1	0,18893774
Procyon lotor AB462049 4	Ichneumia albicauda T603 1	0,18893822
Galidia elegans D146 1	Arctocephalus gazella BK010918 1	0,18893826
Lontra canadensis SRR10409165 1	Ichneumia albicauda T603 1	0,18893903
Vulpes vulpes NC008434 5	Paradoxurus hermaphroditus NC03959	0,18893929
Phocarctos hookeri NC008418 1	Cryptoprocta ferox CFC13 1	0,18894219
Prionodon linsang ERR2391707 1	Mustela nivalis T306 5	0,18894312
Otocyon megalotis SAF1 2	Mustela sibirica NC020637 6	0,18894337
Profelis aurata NC028299 1	Mustela eversmannii NC028013 1	0,18894401

Pusa hispida NC 008433 1	Leopardus pardalis T262 1	0,17158325
Puma concolor NC016470 22	Phoca largha NC008430 1	0,1715858
Hydrurga leptonyx NC008425 1	Galictis vittata T412 1	0,17159374
Spilogale putorius NC010497 1	Meles meles T303 3	0,17160037
Prionailurus rubiginosus NC028304 2	Monachus monachus NC044972 5	0,17160112
Panthera leo NERO 19	Hydrurga leptonyx NC008425 1	0,17161256
Nandinia binotata NC024567 1	Martes melampus NC009678 1	0,17161779
Nandinia binotata NC024567 1	Enhydra lutris NC009692 1	0,17161851
Enhydra lutris NC009692 1	Bassaricyon neblina SRX1097850 1	0,17162319
Cystophora cristata NC008427 1	Atilax paludinosus T606 1	0,17162672
Martes pennanti NC020664 16	Bassaricyon neblina SRX1097850 1	0,17163239
Mustela nivalis T306 5	Mirounga leonina NC008422 1	0,17163315
Phoca groenlandica NC008429 54	Mustela eversmannii NC028013 1	0,17163327
Smilodon populator MF871700 1	Mirounga leonina NC008422 1	0,17163479
Mustela itatsi NC034330 19	Mirounga angustirostris SRR10331586 1	0,1716371
Ursus arctos GU573491 207	Mirounga leonina NC008422 1	0,17163795
Lontra canadensis SRR10409165 1	Lobodon carcinophaga NC008423 1	0,1716385
Viverricula indica KX891751 1	Pusa hispida NC 008433 1	0,1716422
Mirounga leonina NC008422 1	Ailuropoda melanoleuca NC009492 5	0,17164306
Mustela altaica NC021751 1	Mirounga angustirostris SRR10331586 1	0,17164592
Pusa hispida NC 008433 1	Lynx canadensis NC028313 1	0,17164919
Phoca fasciata NC008428 1	Leopardus geoffroyi NC028320 1	0,17164947
Mirounga angustirostris SRR10331586 1	Leopardus wiedii NC028318 1	0,17164956
Viverricula indica NC025296 2	Phoca vitulina NC001325 1	0,17165196
Phoca groenlandica NC008429 54	Civettictis civetta NC033378 1	0,17167403
Ommatophoca rossii AY377287etc 1	Enhydra lutris NC009692 1	0,17168115
Mirounga leonina NC008422 1	Lutra lutra LC050126 1	0,17168154
Mirounga leonina NC008422 1	Chrotogale owstoni T607 1	0,17168449
Pusa hispida NC 008433 1	Genetta abyssinica MG489822 1	0,17169493
Mustela eversmannii NC028013 1	Ailurus fulgens styani NC009691 1	0,17169522
Mustela kathiah NC023210 1	Monachus schauinslandi NC008421 1	0,17170119
Pusa hispida NC 008433 1	Ailurus fulgens NC011124 1	0,17170221
Galerella sanguinea T378 1	Cystophora cristata NC008427 1	0,17170347
Smilodon populator MF871700 1	Monachus monachus NC044972 5	0,17170554
Pusa hispida NC 008433 1	Procyon lotor AB462049 4	0,17170691
Pusa sibirica NC008432 2	Leopardus wiedii NC028318 1	0,17170957
Ommatophoca rossii AY377287etc 1	Lutra lutra NC011358 9	0,17171127
Mirounga angustirostris SRR10331586 1	Genetta abyssinica MG489822 1	0,17171395
Hydrurga leptonyx NC008425 1	Galerella sanguinea T378 1	0,17171484
Lobodon carcinophaga NC008423 1	Felis catus NC001700 2	0,17171487
Phoca largha NC008430 1	Mustela altaica NC021751 1	0,17171499
Pardofelis marmorata NLN3 2	Lobodon carcinophaga NC008423 1	0,17171526
Viverricula indica NC025296 2	Canis adustus KT448271 1	0,17171957
Ursus arctos AP012576 6	Pusa hispida NC 008433 1	0,17172828
Spilogale putorius NC010497 1	Mirounga leonina NC008422 1	0,17173085
Prionailurus planiceps KY682741 4	Monachus monachus NC044972 5	0,17173605
Monachus monachus NC044972 5	Civettictis civetta GLC19 1	0,17173715
Enhydra lutris NC009692 1	Ailurus fulgens NC011124 1	0,17175046
Lontra canadensis SRR10409165 1	Leptonychotes weddellii NC008424 1	0,17175645
Mirounga leonina NC008422 1	Melogale moschata KP726273 1	0,17175733
Lontra canadensis SRR10409165 1	Cystophora cristata NC008427 1	0,17175797
Procyon lotor AB462046 3	Lutra lutra NC011358 9	0,17175826
Mungos mungo/gambianus SRR7704821 1	Erignathus barbatus NC008426 1	0,17176138
Cystophora cristata NC008427 1	Ailurus fulgens styani NC009691 1	0,17176153
Phoca fasciata NC008428 1	Mustela itatsi NC034330 19	0,17176847
Genetta servalina NC024568 2	Erignathus barbatus NC008426 1	0,17177141
Prionailurus planiceps KY682741 4	Erignathus barbatus NC008426 1	0,17177221
Pusa sibirica NC008432 2	Lynx rufus NC014456 3	0,17177363
Monachus monachus NC044972 5	Lutra lutra NC011358 9	0,17177904
Puma concolor NC016470 22	Phoca fasciata NC008428 1	0,17178142
Phoca fasciata NC008428 1	Ictonyx striatus T299 1	0,17178148
Ursus thibetanus mupinensis NC008753 2	Monachus schauinslandi NC008421 1	0,17178926
Monachus monachus NC044972 5	Leopardus geoffroyi NC028320 1	0,17180777
Martes pennanti NC020664 16	Halichoerus grypus NC001602 2	0,17181578
Urva javanica T413 1	Cystophora cristata NC008427 1	0,17182872
Ursus arctos pruinosus MG066703 3	Halichoerus grypus NC001602 2	0,17182995
Hydrurga leptonyx NC008425 1	Urva semitorquata MH464789 1	0,17183915
Pusa hispida NC 008433 1	Procyon lotor AB462046 3	0,17184165
Leopardus colocolo NC028314 1	Halichoerus grypus NC001602 2	0,1718428
Leopardus tigrinus NC028317 1	Cystophora cristata NC008427 1	0,17184301
Pusa caspica NC008431 1	Leopardus pardalis NC028315 1	0,17184377
Phoca groenlandica NC008429 54	Leopardus wiedii NC028318 1	0,17185022
Phoca fasciata NC008428 1	Leopardus wiedii NC028318 1	0,17185034
Lobodon carcinophaga NC008423 1	Ictonyx striatus T299 1	0,1718514
Ursus arctos EU497665 29	Mirounga angustirostris SRR10331586 1	0,17185378
Puma concolor NC016470 22	Phoca vitulina NC001325 1	0,17185497
Puma yagouaroundi NC028311 1	Phoca largha NC008430 1	0,17185551
Monachus monachus NC044972 5	Melogale moschata KP726273 1	0,17186134
Martes flavigula NC012141 3	Bassaricyon neblina SRX1097850 1	0,17187954
Ursus arctos pruinosus MG066703 3	Monachus monachus NC044972 5	0,17188241
Ursus americanus JX196366 3	Monachus schauinslandi NC008421 1	0,17188862
Nandinia binotata NC024567 1	Mustela sibirica NC020637 6	0,17189272
Mirounga angustirostris SRR10331586 1	Lutra lutra NC011358 9	0,1718947
Mirounga leonina NC008422 1	Melogale moschata NC020644 1	0,17189607
Phoca groenlandica NC008429 54	Genetta abyssinica MG489822 1	0,17189716
Phoca groenlandica NC008429 54	Enhydra lutris NC009692 1	0,17189723
Potos flavus T414 1	Lutrogale perspicillata NC035811 1	0,17190164
Pusa sibirica NC008432 2	Lynx lynx NC027083 4	0,17190811
Phoca groenlandica NC008429 54	Lynx canadensis NC028313 1	0,1719147
Mirounga leonina NC008422 1	Melursus ursinus NC009970 2	0,17191975
Phoca vitulina NC001325 1	Lynx rufus NC014456 3	0,17192142
Ursus arctos pruinosus MG066703 3	Phoca vitulina NC001325 1	0,17192455
Ursus americanus JX196366 3	Pusa sibirica NC008432 2	0,17193054
Phoca fasciata NC008428 1	Acinonyx jubatus NC005212 3	0,1719325
Gulo gulo NC009685 3	Ailurus fulgens NC011124 1	0,17193376
Ursus americanus JX196366 3	Pusa hispida NC 008433 1	0,1719455
Pusa sibirica NC008432 2	Canis adustus KT448271 1	0,17194992
Mustela nivalis T306 5	Halichoerus grypus NC001602 2	0,1719518
Smilodon populator MF871700 1	Erignathus barbatus NC008426 1	0,1719549
Lontra canadensis SRR10409165 1	Bassariscus sumichrasti SRX1099089 1	0,17195672
Leptonychotes weddellii NC008424 1	Urva javanica T413 1	0,17196338
Mirounga leonina NC008422 1	Lynx pardinus NC028319 161	0,17197107
Mirounga leonina NC008422 1	Leopardus pardalis NC028315 1	0,17197275

Cryptoprocta ferox CFC13 1	Arctocephalus australis MG023139 1	0,18894477
Cryptoprocta ferox CFC13 1	Arctocephalus forsteri KT693377 17	0,18894508
Panthera uncia NC010638 1	Canis latrans NC008093 7	0,18894546
Urocyon cinereoargenteus NC026723 2	Lynx rufus NC014456 3	0,18894577
Urocyon cinereoargenteus NC026723 2	Leopardus pardalis NC028315 1	0,18894601
Ursus arctos isabellinus 18B5 2	Salanoia concolor D378 1	0,18894659
Urocyon littoralis catalinae KP129018	Martes foina NC020643 1	0,18894686
Ursus arctos pruinosus MG066703 3	Procyon lotor AB462049 4	0,18894823
Speothos venaticus C48 2	Ailurus fulgens NC011124 1	0,18894847
Lycaon pictus NC028427 2	Arctocephalus pusillus NC008417 1	0,18894865
Ictonyx striatus T299 1	Galidia elegans D146 1	0,18895046
Odobenus rosmarus NC004029 29	Enhydra lutris NC009692 1	0,18895057
Urva semitorquata MH464789 1	Helarctos malayanus NC009968 2	0,18895307
Otocyon megalotis SAF1 2	Martes pennanti NC020664 16	0,18895325
Panthera leo spelaea KX258452 2	Mustela putorius NC020638 4	0,18895401
Ursus thibetanus laniger MH281753 2	Cynictis penicillata T375 1	0,18895516
Mustela altaica NC021751 1	Cryptoprocta ferox CFC13 1	0,18895531
Ursus arctos isabellinus 18B5 2	Felis silvestris lybica KP202275 4	0,18895586
Ursus spelaeus NC011112 8	Galidia elegans D146 1	0,18895893
Ursus spelaeus NC011112 8	Galidictis fasciata DM333 1	0,18895999
Ursus spelaeus EU327344 13	Hyaena hyaena NC020669 1	0,18896191
Leopardus jacobita NC028322 1	Ailuropoda melanoleuca NC009492 5	0,18896198
Panthera leo spelaea KX258452 2	Bassariscus sumichrasti SRX1099089 1	0,1889635
Chrotogale owstoni T607 1	Canis mesomelas KT448280 1	0,18896522
Suricata suricatta SSM10 1	Gulo gulo NC009685 3	0,18896972
Parahyaena brunnea NC038159 15	Galictis vittata T412 1	0,18897055
Panthera leo NERO 19	Martes melampus NC009678 1	0,18897325
Ursus spelaeus EU327344 13	Lycaon pictus NC028427 2	0,18897355
Mephitis mephitis NC020648 1	Galictis vittata T412 1	0,18897374
Vulpes lagopus NC026529 3	Neofelis nebulosa NC008450 3	0,1889779
Potos flavus T414 1	Neofelis nebulosa NC008450 3	0,1889782
Genetta genetta T297 1	Arctocephalus forsteri NC004023 28	0,18898416
Felis catus NC001700 2	Arctocephalus forsteri NC004023 28	0,18898675
Panthera tigris NC010642 35	Canis lupus familiaris NC002008 1231	0,18898916
Procyon lotor AB462046 3	Panthera pardus NC010641 5	0,18899135
Mungos mungo/gambianus SRR77048	Conepatus chinga NC042596 1	0,18899441
Ursus spelaeus NC011112 8	Canis mesomelas KT448280 1	0,1889977
Helogale parvula SRR7637809 1	Arctocephalus forsteri KT693377 17	0,18899779
Vulpes corsac NC023958 1	Martes americana NC020642 1	0,188998
Phocarctos hookeri NC008418 1	Bdeogale nigripes GLC15 1	0,18899927
Prionodon linsang ERR2391707 1	Mustela putorius NC020638 4	0,18900053
Ursus arctos isabellinus 18B5 2	Conepatus chinga NC042596 1	0,1890012
Vulpes lagopus NC026529 3	Galidia elegans D146 1	0,18900131
Mungotictis decemlineata NC027828 1	Arctocephalus australis MG023139 1	0,1890028
Salanoia concolor D378 1	Ailurus fulgens NC011124 1	0,18900368
Paradoxurus hermaphroditus NLNC 1	Martes americana NC020642 1	0,18900567
Urva brachyura KY117547 1	Arctotherium sp NC030174 1	0,18900567
Vulpes vulpes NC008434 5	Diplogale hosei MH464790 1	0,18900734
Otocyon megalotis SAF1 2	Lutra lutra LC050126 1	0,18900776
Diplogale hosei MH464790 1	Canis anthus NC027956 2	0,18900843
Felis catus NC001700 2	Arctocephalus townsendi NC008420 1	0,18901062
Urocyon littoralis catalinae KP129018	Martes martes T302 3	0,18901066
Lutra lutra NC011358 9	Crocuta crocuta NC020670 3	0,18901105
Profelis aurata NC028299 1	Arctocephalus forsteri KT693377 17	0,18901131
Ichneumia albicauda T603 1	Ailurus fulgens styani NC009691 1	0,18901508
Speothos venaticus C48 2	Mustela sibirica NC020637 6	0,18901512
Otaria byronia OTAB 1	Hyaena hyaena NC020669 1	0,18901607
Panthera onca NC022842 1	Ailurus fulgens styani NC009691 1	0,18901664
Zalophus wollebaeki SRR4431565 1	Suricata suricatta SSM10 1	0,18901673
Procyon lotor AB462049 4	Lycaon pictus NC028427 2	0,18901876
Mustela altaica NC021751 1	Hyaena hyaena NC020669 1	0,18901999
Vulpes ferrilata NC027935 1	Panthera onca NC022842 1	0,18902166
Panthera onca NC022842 1	Arctocephalus townsendi NC008420 1	0,18902245
Ursus arctos GU573491 207	Felis margarita NC028308 1	0,18902381
Ursus thibetanus thibetanus NC01111	Urva javanica T413 1	0,18902381
Ursus maritimus NC003428 31	Felis margarita NC028308 1	0,18902396
Panthera onca NC022842 1	Neophoca cinerea NC008419 1	0,18902591
Ursus arctos AP012576 6	Crossarchus platycephalus C7R66 1	0,1890278
Ursus arctos GU573486 5	Panthera onca KP202264 2	0,18902843
Leopardus tigrinus NC028317 1	Ailuropoda melanoleuca NC009492 5	0,18902922
Lynx pardinus NC028319 161	Ailuropoda melanoleuca NC009492 5	0,18902925
Ursus arctos AP012576 6	Salanoia concolor D378 1	0,18903084
Helarctos malayanus NC009968 2	Galerella sanguinea T378 1	0,18903251
Ursus arctos pruinosus MG066703 3	Ictonyx striatus T299 1	0,18903424
Pardofelis marmorata NLN3 2	Helarctos malayanus NC009968 2	0,18903606
Urva javanica/auropunctata NC00683	Arctocephalus australis MG023139 1	0,18903716
Canis mesomelas KT448280 1	Arctodus simus NC011116 1	0,1890378
Panthera tigris amoyensis NC014770 2	Chrysocyon brachyurus NC024172 1	0,18903863
Ursus arctos AP012576 6	Leopardus jacobita NC028322 1	0,18904032
Neofelis nebulosa NC008450 3	Lutra lutra LC050126 1	0,18904102
Panthera leo NERO 19	Melogale moschata KP726273 1	0,18904397
Panthera leo NERO 19	Melogale moschata V0735A 1	0,18904415
Xenogale naso C07XAR110 1	Eumetopias jubatus NC004030 10	0,18904598
Canis aureus KT448274 1	Arctocephalus pusillus NC008417 1	0,189046
Neofelis nebulosa NC008450 3	Mustela nigripes NC024942 1	0,18904778
Cuon alpinus NC013445 3	Ailurus fulgens NC011124 1	0,1890485
Panthera leo NERO 19	Lycalopex sechurae KT448284 1	0,18904942
Ursus maritimus GU573488 Svalbard	Canis lupus familiaris NC002008 1231	0,18905056
Ursus arctos GU573486 5	Canis aureus KT448274 1	0,18905251
Ictonyx striatus T299 1	Urva javanica/auropunctata NC006835	0,18905333
Lycalopex sechurae KT448284 1	Lutra lutra NC011358 9	0,18905856
Melogale moschata V0735A 1	Diplogale hosei MH464790 1	0,18906006
Otocyon megalotis SAF1 2	Eumetopias jubatus NC004030 10	0,18906021
Suricata suricatta SSM10 1	Martes zibellina NC011579 39	0,18906328
Ursus spelaeus EU327344 13	Canis lupus familiaris NC002008 1231	0,18906431
Helogale parvula SRR7637809 1	Ailurus fulgens styani NC009691 1	0,18906608
Urocyon littoralis catalinae KP129018	Martes zibellina NC011579 39	0,18906666
Tremarctos ornatus NC009969 2	Urva javanica T413 1	0,18906884
Suricata suricatta SSM10 1	Aonyx cinerea NC035814 2	0,18906927
Galidictis fasciata DM333 1	Arctocephalus gazella BK010918 1	0,18907236
Vulpes ferrilata NC027935 1	Taxidea taxus NC020646 1	0,18907367
Ursus spelaeus NC011112 8	Panthera pardus NC010641 5	0,18907368
Lontra canadensis SRR10409165 1	Civettictis civetta GLC19 1	0,18907379

Monachus schauinslandi NC008421 1	Martes americana NC020642 1	0,17197381
Hydrurga leptonyx NC008425 1	Urva javanica T413 1	0,17197397
Lycaon pictus NC028427 2	Leptonychotes weddellii NC008424 1	0,17197496
Prionailurus planiceps KY682741 4	Cystophora cristata NC008427 1	0,17197548
Leopardus tigrinus NC028317 1	Erignathus barbatus NC008426 1	0,17197631
Ursus arctos GU573491 207	Phoca vitulina NC001325 1	0,17198554
Pusa hispida NC 008433 1	Puma concolor NC016470 22	0,17198583
Ursus arctos AP012576 6	Pusa caspica NC008431 1	0,1719892
Prionailurus bengalensis NC028301 12	Phoca largha NC008430 1	0,17199041
Hemigalus derbyanus MH464791 1	Cystophora cristata NC008427 1	0,17199694
Potos flavus T414 1	Ailurus fulgens NC011124 1	0,17203067
Monachus schauinslandi NC008421 1	Conepatus chinga NC042596 1	0,17203121
Mustela itatsi NC034330 19	Ailurus fulgens NC011124 1	0,17203316
Mirounga leonina NC008422 1	Caracal caracal NC028306 1	0,17203993
Otocolobus manul NC028323 1	Mirounga leonina NC008422 1	0,17203993
Procyon lotor AB462046 3	Phoca groenlandica NC008429 54	0,17204329
Halichoerus grypus NC001602 2	Acinonyx jubatus NC005212 3	0,17204334
Puma yagouaroundi NC028311 1	Cystophora cristata NC008427 1	0,17204352
Ursus americanus JX196366 3	Halichoerus grypus NC001602 2	0,17205308
Pusa hispida NC 008433 1	Leopardus wiedii NC028318 1	0,17205518
Phoca largha NC008430 1	Lynx rufus NC014456 3	0,17205624
Prionailurus bengalensis NC028301 12	Phoca vitulina NC001325 1	0,17205758
Monachus schauinslandi NC008421 1	Acinonyx jubatus NC005212 3	0,17206375
Monachus monachus NC044972 5	Martes pennanti NC020664 16	0,17206737
Erignathus barbatus NC008426 1	Civettictis civetta NC033378 1	0,17207736
Phoca groenlandica NC008429 54	Hemigalus derbyanus MH464791 1	0,17207789
Viverra tangalunga MH464792 1	Phoca groenlandica NC008429 54	0,17210525
Monachus schauinslandi NC008421 1	Fossa fossana D350 1	0,17210841
Leptonychotes weddellii NC008424 1	Ichneumia albicauda T603 1	0,17210972
Prionailurus planiceps NC028312 6	Cystophora cristata NC008427 1	0,17211011
Homotherium latidens MF871702 3	Ailurus fulgens styani NC009691 1	0,1721154
Procyon lotor AB462049 4	Mirounga angustirostris SRR10331586 1	0,17211552
Phoca fasciata NC008428 1	Lynx lynx NC027083 4	0,17211592
Monachus schauinslandi NC008421 1	Felis chaus NC028307 1	0,17211872
Poecilogale albinucha T602 1	Lobodon carcinophaga NC008423 1	0,17211938
Phoca groenlandica NC008429 54	Catopuma badia NC028300 1	0,17211948
Martes flavigula NC012141 3	Ailurus fulgens styani NC009691 1	0,17212659
Mirounga angustirostris SRR10331586 1	Martes flavigula NC012141 3	0,1721402
Ursus americanus JX196366 3	Mirounga angustirostris SRR10331586 1	0,17214337
Spilogale putorius NC010497 1	Lobodon carcinophaga NC008423 1	0,17215125
Ommatophoca rossii AY377287etc 1	Lutra lutra LC050126 1	0,17215893
Puma yagouaroundi NC028311 1	Mirounga leonina NC008422 1	0,17217447
Phoca fasciata NC008428 1	Martes pennanti NC020664 16	0,17217539
Prionailurus viverrinus NC028305 1	Mirounga leonina NC008422 1	0,17217572
Pusa caspica NC008431 1	Catopuma badia NC028300 1	0,17217775
Viverricula indica NC025296 2	Pusa caspica NC008431 1	0,17217873
Viverricula indica KX891745 1	Pusa hispida NC 008433 1	0,17218097
Salanoia concolor D378 1	Lobodon carcinophaga NC008423 1	0,17218201
Mephitis mephitis NC020648 1	Leptonychotes weddellii NC008424 1	0,17218542
Prionailurus bengalensis NC028301 12	Phoca groenlandica NC008429 54	0,1721863
Monachus schauinslandi NC008421 1	Ictonyx striatus T299 1	0,17218861
Mirounga angustirostris SRR10331586 1	Catopuma badia NC028300 1	0,17218886
Prionailurus bengalensis CKM45 20	Phoca fasciata NC008428 1	0,17218921
Ursus arctos GU573491 207	Mirounga angustirostris SRR10331586 1	0,1721904
Phoca vitulina NC001325 1	Leptailurus serval NC028316 1	0,17219208
Phoca largha NC008430 1	Leopardus jacobita NC028322 1	0,17219427
Mustela erminea T305 2	Eumetopias jubatus NC004030 10	0,17220147
Monachus monachus NC044972 5	Felis nigripes NC028309 1	0,17220475
Smilodon populator MF871700 1	Lobodon carcinophaga NC008423 1	0,17221698
Mustela putorius NC020638 4	Mirounga leonina NC008422 1	0,17222936
Nandinia binotata NC024567 1	Mustela putorius NC020638 4	0,17222936
Leptonychotes weddellii NC008424 1	Helogale parvula SRR7637809 1	0,1722327
Leptonychotes weddellii NC008424 1	Urva brachyura KY117547 1	0,1722327
Taxidea taxus NC020646 1	Callorhinus ursinus NC008415 1	0,172233
Mustela nigripes NC024942 1	Ailurus fulgens NC011124 1	0,17223468
Mustela sibirica NC020637 6	Mirounga angustirostris SRR10331586 1	0,17224261
Mirounga leonina NC008422 1	Genetta servalina NC024568 2	0,17224288
Phoca largha NC008430 1	Ailurus fulgens styani NC009691 1	0,17224391
Phoca fasciata NC008428 1	Civettictis civetta GLC19 1	0,1722443
Otocolobus manul NC028323 1	Erignathus barbatus NC008426 1	0,17224437
Mirounga angustirostris SRR10331586 1	Lynx pardinus NC028319 161	0,17225289
Prionailurus bengalensis CKM45 20	Phoca vitulina NC001325 1	0,17226268
Leptonychotes weddellii NC008424 1	Canis lupus familiaris NC002008 1231	0,17226371
Prionailurus planiceps NC028312 6	Monachus monachus NC044972 5	0,17227469
Monachus monachus NC044972 5	Lynx pardinus NC028319 161	0,17227896
Lynx rufus NC014456 3	Halichoerus grypus NC001602 2	0,17229514
Pusa sibirica NC008432 2	Bassaricyon neblina SRX1097850 1	0,17229858
Procyon lotor AB462049 4	Erignathus barbatus NC008426 1	0,17230234
Procyon lotor AB462049 4	Cystophora cristata NC008427 1	0,1723025
Nandinia binotata NC024567 1	Bassariscus sumichrasti SRX1099089 1	0,17230503
Ursus arctos AP012576 6	Halichoerus grypus NC001602 2	0,17231046
Lobodon carcinophaga NC008423 1	Canis mesomelas KT448280 1	0,17231105
Cystophora cristata NC008427 1	Catopuma temminckii NC027115 41	0,17231113
Phoca vitulina NC001325 1	Ailurus fulgens styani NC009691 1	0,17231143
Pardofelis marmorata NLN3 2	Cystophora cristata NC008427 1	0,17231154
Phoca groenlandica NC008429 54	Civettictis civetta GLC19 1	0,17231163
Pusa caspica NC008431 1	Lynx lynx NC027083 4	0,17231205
Lobodon carcinophaga NC008423 1	Felis chaus NC028307 1	0,1723211
Phoca groenlandica NC008429 54	Leopardus pardalis NC028315 1	0,17232123
Poecilogale albinucha T602 1	Phoca fasciata NC008428 1	0,17232355
Phoca vitulina NC001325 1	Leopardus jacobita NC028322 1	0,17232879
Mustela sibirica AP017394 11	Monachus monachus NC044972 5	0,17233054
Leptonychotes weddellii NC008424 1	Urva javanica/auropunctata NC006835 1	0,17233807
Phoca vitulina NC001325 1	Acinonyx jubatus NC005212 3	0,17234264
Phoca largha NC008430 1	Acinonyx jubatus NC005212 3	0,17234294
Ommatophoca rossii AY377287etc 1	Martes flavigula NC012141 3	0,17234489
Monachus monachus NC044972 5	Lynx canadensis NC028313 1	0,17234689
Viverricula indica NC025296 2	Halichoerus grypus NC001602 2	0,17235996
Nandinia binotata NC024567 1	Mustela sibirica AP017394 11	0,17236388
Mustela putorius NC020638 4	Ailurus fulgens NC011124 1	0,17236917
Salanoia concolor D378 1	Pusa sibirica NC008432 2	0,17236994
Ictonyx striatus T299 1	Cystophora cristata NC008427 1	0,17237845
Pusa sibirica NC008432 2	Puma concolor NC016470 22	0,17238091

Paradoxurus jerdoni MH464793 1	Martes zibellina NC011579 39	0,18907383
Galerella sanguinea T378 1	Canis mesomelas KT448280 1	0,18907605
Cynogale bennetti KY117544 1	Ailuropoda melanoleuca NC009492 5	0,18907702
Urocyon cinereoargenteus NC026723 2	Genetta servalina NC024568 2	0,18907817
Paguma larvata PDDS11 2	Neophoca cinerea NC008419 1	0,18907897
Profelis aurata NC028299 1	Arctocephalus gazella BK010918 1	0,18907963
Pardofelis marmorata NLN3 2	Arctocephalus gazella BK010918 1	0,18907991
Phocarctos hookeri NC008418 1	Genetta genetta T297 1	0,18908001
Viverricula indica KX891751 1	Aonyx cinerea NC035814 2	0,18908028
Ictonyx striatus T299 1	Urva brachyura KY117547 1	0,18908055
Urocyon littoralis catalinae KP129018	Leopardus pardalis NC028315 1	0,1890807
Lynx canadensis NC028313 1	Bassaricyon neblina SRX1097850 1	0,1890824
Urocyon littoralis catalinae KP129018	Prionailurus bengalensis NC028301 12	0,18908262
Neophoca cinerea NC008419 1	Felis chaus NC028307 1	0,18908341
Lutrogale perspicillata NC035811 1	Felis nigripes NC028309 1	0,1890849
Urva javanica/auropunctata NC006835	Arctocephalus forsteri NC004023 28	0,18908571
Meles anakuma NC009677 1	Chrysocyon brachyurus NC024172 1	0,18908654
Pardofelis marmorata NLN3 2	Otaria byronia OTAB 1	0,18908678
Martes martes T302 3	Crocuta crocuta NC020670 3	0,18908796
Ursus arctos GU573491 207	Lynx rufus NC014456 3	0,18908989
Melursus ursinus NC009970 2	Helogale parvula SRR7637809 1	0,18908997
Ursus arctos pruinosus MG066703 3	Lynx pardinus NC028319 161	0,18909067
Speothos venaticus C48 2	Leopardus jacobita NC028322 1	0,1890915
Ursus spelaeus EU327344 13	Mungos mungo/gambianus SRR77048	0,18909161
Ursus maritimus GU573488 Svalbard	Felis chaus NC028307 1	0,18909176
Ursus thibetanus laniger MH281753 2	Urva javanica T413 1	0,18909316
Ursus arctos pruinosus MG066703 3	Genetta genetta T297 1	0,18909419
Ichneumia albicauda T603 1	Galictis vittata T412 1	0,18909611
Urocyon littoralis catalinae KP129018	Smilodon populator MF871700 1	0,18909799
Puma concolor NC016470 22	Ailuropoda melanoleuca NC009492 5	0,18909819
Lycaon pictus NC028427 2	Ictonyx striatus T299 1	0,18909869
Ursus spelaeus NC011112 8	Paradoxurus hermaphroditus NC03959	0,18910088
Prionodon pardicolor NC024569 2	Ailuropoda melanoleuca NC009492 5	0,18910089
Ursus arctos GU573486 5	Leopardus guigna NC028321 1	0,18910162
Ursus arctos EU497665 29	Diplogale hosei MH464790 1	0,18910892
Conepatus chinga NC042596 1	Arctocephalus forsteri NC004023 28	0,18910989
Ursus arctos AP012576 6	Prionailurus bengalensis CKM45 20	0,18911131
Urva javanica T413 1	Eumetopias jubatus NC004030 10	0,18911299
Ursus thibetanus formosanus NC00993	Diplogale hosei MH464790 1	0,18911406
Ursus americanus JX196366 3	Galerella sanguinea T378 1	0,18911654
Ursus arctos pruinosus MG066703 3	Canis lupus familiaris NC002008 1231	0,18911804
Urocyon cinereoargenteus NC026723 2	Smilodon populator MF871700 1	0,18912047
Ursus arctos EU497665 29	Panthera pardus NC010641 5	0,18912829
Procyon lotor AB462046 3	Cynogale bennetti KY117544 1	0,18913192
Vulpes corsac NC023958 1	Urva semitorquata MH464789 1	0,18913318
Vulpes lagopus NC026529 3	Urva brachyura KY117547 1	0,18913355
Salanoia concolor D378 1	Bassaricyon neblina SRX1097850 1	0,18913375
Ursus thibetanus thibetanus NC01111	Cuon alpinus NC013445 3	0,18913385
Phocarctos hookeri NC008418 1	Conepatus chinga NC042596 1	0,18913458
Panthera pardus NC010641 5	Lycaon pictus NC028427 2	0,18913627
Vulpes lagopus NC026529 3	Mustela sibirica AP017394 11	0,18913634
Vulpes lagopus NC026529 3	Mustela sibirica NC020637 6	0,18913655
Nyctereutes procyonoides NC013700 3	Galidictis fasciata DM333 1	0,18913704
Vulpes zerda KJ603240 1	Mustela putorius NC020638 4	0,18913759
Vulpes corsac NC023958 1	Chrotogale owstoni T607 1	0,18913761
Procyon lotor AB462049 4	Urva javanica T413 1	0,18913791
Otaria byronia OTAB 1	Mungotictis decemlineata NC027828 1	0,18913852
Ursus thibetanus laniger MH281753 2	Panthera pardus NC010641 5	0,18914087
Atilax paludinosus T606 1	Arctotherium sp NC030174 1	0,18914104
Vulpes corsac NC023958 1	Paradoxurus hermaphroditus NLNC 1	0,18914193
Vulpes lagopus NC026529 3	Paradoxurus hermaphroditus NC03959	0,18914211
Viverra zibetha T609 1	Urocyon cinereoargenteus NC026723 2	0,18914573
Paradoxurus hermaphroditus NC03959	Martes pennanti NC020664 16	0,18914731
Proteles cristata T393 6	Meles meles T303 3	0,18914789
Panthera onca KP202264 2	Neophoca cinerea NC008419 1	0,18914899
Melogale moschata NC020644 1	Cryptoprocta ferox CFC13 1	0,18914992
Ursus maritimus GU573488 Svalbard	Canis latrans NC008093 7	0,18914996
Panthera tigris amoyensis NC014770 2	Lutra lutra NC011358 9	0,18915172
Leopardus guigna NC028321 1	Bassaricyon neblina SRX1097850 1	0,18915334
Potos flavus T414 1	Panthera tigris amoyensis NC014770 2	0,18915361
Leopardus geoffroyi NC028320 1	Bassaricyon neblina SRX1097850 1	0,18915392
Ursus arctos pruinosus MG066703 3	Salanoia concolor D378 1	0,18915488
Panthera uncia NC010638 1	Arctodus simus NC011116 1	0,18915489
Pardofelis marmorata NLN3 2	Otocyon megalotis SAF1 2	0,18915603
Ursus maritimus GU573488 Svalbard	Panthera pardus japonensis KJ866876	0,18915725
Panthera onca NC022842 1	Martes americana NC020642 1	0,18915741
Ursus arctos GU573491 207	Lynx pardinus NC028319 161	0,18915759
Nyctereutes procyonoides NC013700 3	Helarctos malayanus NC009968 2	0,18915915
Ursus arctos GU573486 5	Cryptoprocta ferox CFC13 1	0,18915996
Speothos venaticus C48 2	Arctodus simus NC011116 1	0,18916029
Ursus thibetanus mupinensis NC00875	Paradoxurus hermaphroditus NC03959	0,18916053
Odobenus rosmarus NC004029 29	Martes zibellina NC011579 39	0,1891607
Panthera leo spelaea KX258452 2	Canis anthus NC027956 2	0,18916129
Ursus spelaeus EU327344 13	Galidictis fasciata DM333 1	0,18916206
Procyon lotor AB462049 4	Panthera onca NC022842 1	0,18916284
Ursus arctos pruinosus MG066703 3	Leopardus guigna NC028321 1	0,1891629
Prionodon pardicolor NC024569 2	Ictonyx striatus T299 1	0,18916407
Prionailurus planiceps NC028312 6	Ailuropoda melanoleuca NC009492 5	0,18916506
Prionailurus rubiginosus NC028304 2	Ailuropoda melanoleuca NC009492 5	0,18916576
Ursus americanus JX196366 3	Xenogale naso C07XAR110 1	0,18916805
Neofelis nebulosa NC008450 3	Enhydra lutris NC009692 1	0,18917681
Nyctereutes procyonoides NC013700 3	Eumetopias jubatus NC004030 10	0,18918195
Ursus thibetanus thibetanus NC01111	Diplogale hosei MH464790 1	0,18918318
Neofelis nebulosa NC008450 3	Melogale moschata NC020644 1	0,18918845
Ursus spelaeus NC011112 8	Odobenus rosmarus NC004029 29	0,18918975
Urva semitorquata MH464789 1	Canis mesomelas KT448280 1	0,1891921
Martes zibellina NC011579 39	Helogale parvula SRR7637809 1	0,18919763
Ursus spelaeus NC011112 8	Canis lupus familiaris NC002008 1231	0,18919875
Ursus arctos AP012576 6	Canis lupus familiaris NC002008 1231	0,18919902
Genetta genetta T297 1	Tapirus terrestris T358	0,18919915
Suricata suricatta SSM10 1	Arctocephalus australis MG023139 1	0,18919933
Nasua nasua NC020647 1	Arctocephalus townsendi NC008420 1	0,1892
Nyctereutes procyonoides NC013700 3	Lontra canadensis SRR10409165 1	0,18920062

Monachus schauinslandi NC008421 1	Martes pennanti NC020664 16	0,17238161
Leopardus jacobita NC028322 1	Cystophora cristata NC008427 1	0,17238176
Puma concolor NC016470 22	Phoca groenlandica NC008429 54	0,17238784
Monachus schauinslandi NC008421 1	Leopardus pardalis T262 1	0,1723882
Prionailurus planiceps KY682741 4	Mirounga angustirostris SRR10331586 1	0,17238893
Pusa hispida NC 008433 1	Prionailurus planiceps KY682741 4	0,17238951
Leptonychotes weddellii NC008424 1	Arctictis binturong T605 2	0,172392
Paradoxurus jerdoni MH464793 1	Canis adustus KT448271 1	0,17240092
Monachus monachus NC044972 5	Felis chaus NC028307 1	0,17240936
Monachus schauinslandi NC008421 1	Chrotogale owstoni T607 1	0,17243084
Mustela sibirica NC020637 6	Bassaricyon neblina SRX1097850 1	0,17243236
Mungos mungo MMC7 1	Erignathus barbatus NC008426 1	0,17243627
Pusa hispida NC 008433 1	Hemigalus derbyanus MH464791 1	0,1724382
Monachus schauinslandi NC008421 1	Hemigalus derbyanus MH464791 1	0,17244233
Mustela eversmannii NC028013 1	Monachus schauinslandi NC008421 1	0,17244242
Lobodon carcinophaga NC008423 1	Urva semitorquata MH464789 1	0,1724453
Erignathus barbatus NC008426 1	Caracal caracal NC028306 1	0,17244631
Leptailurus serval NC028316 1	Canis adustus KT448271 1	0,17244951
Phoca fasciata NC008428 1	Mustela altaica NC021751 1	0,17245074
Pusa hispida NC 008433 1	Lutrogale perspicillata NC035811 1	0,17245312
Puma yagouaroundi NC028311 1	Phoca fasciata NC008428 1	0,17245533
Ichneumia albicauda T603 1	Hydrurga leptonyx NC008425 1	0,17245753
Pusa hispida NC 008433 1	Leopardus geoffroyi NC028320 1	0,17245892
Prionailurus bengalensis CKM45 20	Phoca largha NC008430 1	0,1724648
Mustela itatsi NC034330 19	Monachus monachus NC044972 5	0,17246583
Monachus monachus NC044972 5	Leopardus jacobita NC028322 1	0,17247763
Mirounga leonina NC008422 1	Conepatus chinga NC042596 1	0,17249602
Pusa caspica NC008431 1	Bassaricyon neblina SRX1097850 1	0,17250076
Pusa sibirica NC008432 2	Ailurus fulgens styani NC009691 1	0,17250191
Mirounga angustirostris SRR10331586 1	Martes zibellina NC011579 39	0,17251015
Viverra zibetha T609 1	Cystophora cristata NC008427 1	0,17251132
Felis catus NC001700 2	Cystophora cristata NC008427 1	0,17251363
Poecilogale albinucha T602 1	Leptonychotes weddellii NC008424 1	0,17251426
Lobodon carcinophaga NC008423 1	Bassaricyon neblina SRX1097850 1	0,17251705
Ommatophoca rossii AY377287etc 1	Neovison vison NC020641 3	0,17251877
Monachus schauinslandi NC008421 1	Leopardus tigrinus NC028317 1	0,1725226
Lobodon carcinophaga NC008423 1	Genetta genetta T297 1	0,1725238
Phoca fasciata NC008428 1	Leopardus guigna NC028321 1	0,17252494
Phoca groenlandica NC008429 54	Ailuropoda melanoleuca NC009492 5	0,17252894
Proteles cristata T393 6	Diplogale hosei MH464790 1	0,17253453
Panthera leo NERO 19	Leptonychotes weddellii NC008424 1	0,17254061
Monachus monachus NC044972 5	Felis margarita NC028308 1	0,17254382
Halichoerus grypus NC001602 2	Ailurus fulgens styani NC009691 1	0,17254394
Monachus monachus NC044972 5	Leopardus guigna NC028321 1	0,1725487
Lontra canadensis SRR10409165 1	Erignathus barbatus NC008426 1	0,17256539
Leopardus wiedii NC028318 1	Halichoerus grypus NC001602 2	0,17256646
Parahyaena brunnea NC038159 15	Cynogale bennetti KY117544 1	0,1725696
Salanoia concolor D378 1	Pusa caspica NC008431 1	0,17257194
Procyon lotor AB462046 3	Cystophora cristata NC008427 1	0,17257236
Neovison vison NC020641 3	Nandinia binotata NC024567 1	0,17257613
Martes foina NC020643 1	Ailurus fulgens NC011124 1	0,17257746
Puma concolor NC016470 22	Mirounga leonina NC008422 1	0,17257795
Poecilogale albinucha T602 1	Ailurus fulgens NC011124 1	0,17257956
Lobodon carcinophaga NC008423 1	Helogale parvula SRR7637809 1	0,17257993
Procyon lotor AB462049 4	Phoca vitulina NC001325 1	0,17258269
Procyon lotor AB462046 3	Phoca vitulina NC001325 1	0,17258327
Pusa caspica NC008431 1	Leptailurus serval NC028316 1	0,17258365
Poecilogale albinucha T602 1	Cystophora cristata NC008427 1	0,17258476
Phoca fasciata NC008428 1	Mustela nivalis T306 5	0,17258513
Prionailurus planiceps KY682741 4	Phoca fasciata NC008428 1	0,17258927
Ursus arctos AP012576 6	Mirounga leonina NC008422 1	0,17259556
Prionailurus bengalensis CKM45 20	Mirounga angustirostris SRR10331586 1	0,17259589
Phoca largha NC008430 1	Leopardus wiedii NC028318 1	0,17259812
Parahyaena brunnea NC038159 15	Hydrurga leptonyx NC008425 1	0,17259929
Mustela nigripes NC024942 1	Monachus monachus NC044972 5	0,17260042
Panthera onca NC022842 1	Lobodon carcinophaga NC008423 1	0,1726024
Phoca fasciata NC008428 1	Canis adustus KT448271 1	0,17260502
Ursus arctos AP012576 6	Phoca vitulina NC001325 1	0,17260702
Hydrurga leptonyx NC008425 1	Urva javanica/auropunctata NC006835 1	0,17261414
Spilogale putorius NC010497 1	Mirounga angustirostris SRR10331586 1	0,17261437
Martes melampus NC009678 1	Ailurus fulgens styani NC009691 1	0,17262916
Lynx lynx NC027083 4	Halichoerus grypus NC001602 2	0,1726324
Urva semitorquata MH464789 1	Cystophora cristata NC008427 1	0,17263669
Pusa sibirica NC008432 2	Eupleres goudotii D128 1	0,17263681
Pusa caspica NC008431 1	Procyon lotor AB462046 3	0,17263927
Mirounga leonina NC008422 1	Lynx lynx NC027083 4	0,17264387
Hydrurga leptonyx NC008425 1	Cynictis penicillata T375 1	0,17264734
Procyon lotor AB462046 3	Phoca fasciata NC008428 1	0,1726485
Prionailurus bengalensis CKM45 20	Mirounga leonina NC008422 1	0,17264949
Pusa caspica NC008431 1	Puma concolor NC016470 22	0,17265022
Pusa caspica NC008431 1	Prionailurus bengalensis NC028301 12	0,17265083
Pusa caspica NC008431 1	Leopardus pardalis T262 1	0,17265213
Pusa hispida NC 008433 1	Canis adustus KT448271 1	0,17265585
Prionailurus planiceps NC028312 6	Mirounga angustirostris SRR10331586 1	0,17265821
Prionailurus bengalensis CKM45 20	Monachus schauinslandi NC008421 1	0,17266087
Zalophus californianus NC008416 1	Taxidea taxus NC020646 1	0,17267169
Monachus monachus NC044972 5	Leopardus pardalis NC028315 1	0,17267565
Monachus monachus NC044972 5	Leopardus pardalis T262 1	0,17267607
Monachus monachus NC044972 5	Felis catus NC001700 2	0,17267871
Monachus monachus NC044972 5	Genetta abyssinica MG489822 1	0,17268485
Erignathus barbatus NC008426 1	Conepatus chinga NC042596 1	0,17269444
Cystophora cristata NC008427 1	Crossarchus platycephalus C7R66 1	0,17270401
Mirounga leonina NC008422 1	Aonyx cinerea NC035814 2	0,17270894
Paguma larvata PDDS11 2	Leptonychotes weddellii NC008424 1	0,17271313
Cystophora cristata NC008427 1	Civettictis civetta GLC19 1	0,17271382
Mirounga angustirostris SRR10331586 1	Aonyx cinerea NC035814 2	0,1727197
Prionodon linsang ERR2391707 1	Crocuta crocuta NC020670 3	0,17272273
Phoca fasciata NC008428 1	Lynx pardinus NC028319 161	0,17272277
Felis silvestris lybica KP202275 4	Canis adustus KT448271 1	0,17272297
Viverricula indica KX891745 1	Canis adustus KT448271 1	0,17272645
Nandinia binotata NC024567 1	Lutra lutra LC050126 1	0,17276223
Lutra sumatrana NC035810 1	Ailurus fulgens styani NC009691 1	0,17276634
Leopardus pardalis T262 1	Halichoerus grypus NC001602 2	0,17276888

Urocyon cinereoargenteus NC026723 2	Martes zibellina NC011579 39	0,1892013
Nyctereutes procyonoides NC013700 3	Arctocephalus australis MG023139 1	0,18920229
Urocyon littoralis catalinae KP129018	Meles meles T303 3	0,18920273
Phocarctos hookeri NC008418 1	Mungotictis decemlineata NC027828 1	0,18920332
Phocarctos hookeri NC008418 1	Galidictis fasciata DM333 1	0,18920343
Mustela eversmannii NC028013 1	Mungotictis decemlineata NC027828 1	0,18920371
Felis catus NC001700 2	Tapirus terrestris T358	0,18920515
Urva javanica T413 1	Arctotherium sp NC030174 1	0,18920782
Ichneumia albicauda T603 1	Canis mesomelas KT448280 1	0,18920943
Viverricula indica NC025296 2	Lontra canadensis SRR10409165 1	0,18921162
Ursus arctos isabellinus 18B5 2	Lutrogale perspicillata NC035811 1	0,18921188
Vulpes corsac NC023958 1	Panthera uncia KP202269 1	0,18921298
Martes zibellina NC011579 39	Hyaena hyaena NC020669 1	0,18921313
Puma concolor NC016470 22	Arctocephalus townsendi NC008420 1	0,18921317
Ursus thibetanus mupinensis NC00875	Mungos mungo/gambianus SRR77048	0,1892135
Ursus thibetanus mupinensis NC00875	Galidia elegans D146 1	0,18921439
Poecilogale albinucha T602 1	Helogale parvula SRR7637809 1	0,18921466
Panthera pardus japonensis KJ866876	Nyctereutes procyonoides NC013700 3	0,18921569
Urocyon cinereoargenteus NC026723 2	Felis silvestris lybica KP202275 4	0,18921576
Ursus arctos GU573491 207	Bassaricyon neblina SRX1097850 1	0,18921638
Martes americana NC020642 1	Chrysocyon brachyurus NC024172 1	0,18921673
Poecilogale albinucha T602 1	Mungotictis decemlineata NC027828 1	0,18921675
Urocyon cinereoargenteus NC026723 2	Neovison vison NC020641 3	0,18921693
Xenogale naso C07XAR110 1	Chrysocyon brachyurus NC024172 1	0,18921839
Ursus arctos GU573486 5	Helogale parvula SRR7637809 1	0,1892186
Odobenus rosmarus NC004029 29	Martes melampus NC009678 1	0,18922351
Ursus arctos isabellinus 18B5 2	Ichneumia albicauda T603 1	0,18922356
Panthera tigris amoyensis NC014770 2	Canis anthus NC027956 2	0,18922369
Ursus thibetanus formosanus NC00993	Cynictis penicillata T375 1	0,18922371
Panthera leo spelaea KX258452 2	Melogale moschata V0735A 1	0,18922384
Ursus arctos isabellinus 18B5 2	Panthera onca KP202264 2	0,18922384
Helarctos malayanus NC009968 2	Atilax paludinosus T606 1	0,1892252
Ursus maritimus GU573488 Svalbard	Leopardus tigrinus NC028317 1	0,18922588
Ursus spelaeus NC011112 8	Helogale parvula SRR7637809 1	0,18922609
Paradoxurus jerdoni MH464793 1	Otaria byronia OTAB 1	0,1892285
Ursus maritimus GU573488 Svalbard	Leopardus guigna NC028321 1	0,18923
Ursus spelaeus EU327344 13	Canis latrans NC008093 7	0,18923119
Zalophus californianus NC008416 1	Vulpes lagopus NC026529 3	0,18923189
Felis margarita NC028308 1	Ailuropoda melanoleuca NC009492 5	0,18923244
Viverricula indica KX891751 1	Galictis vittata T412 1	0,18923253
Panthera onca KP202264 2	Helarctos malayanus NC009968 2	0,1892363
Mephitis mephitis NC020648 1	Lynx pardinus NC028319 161	0,18923748
Ursus spelaeus EU327344 13	Ichneumia albicauda T603 1	0,18923825
Catopuma temminckii NC027115 41	Ailuropoda melanoleuca NC009492 5	0,18923916
Melogale moschata NC020644 1	Canis mesomelas KT448280 1	0,1892392
Speothos venaticus C48 2	Helarctos malayanus NC009968 2	0,18924043
Ursus arctos AP012576 6	Lynx rufus NC014456 3	0,18924111
Ursus americanus JX196366 3	Helogale parvula SRR7637809 1	0,18924245
Ursus arctos AP012576 6	Prionailurus bengalensis NC028301 12	0,189243
Panthera uncia NC010638 1	Canis lupus chanco NC010340 4	0,18924344
Nyctereutes procyonoides NC013700 3	Arctocephalus forsteri NC004023 28	0,1892457
Tremarctos ornatus NC009969 2	Panthera leo NERO 19	0,18924622
Hyaena hyaena NC020669 1	Canis lupus familiaris NC002008 1231	0,18924633
Ursus americanus JX196366 3	Cryptoprocta ferox CFC13 1	0,18924829
Mephitis mephitis NC020648 1	Acinonyx jubatus NC005212 3	0,18924853
Poecilogale albinucha T602 1	Urva javanica/auropunctata NC006835	0,18925019
Panthera onca NC022842 1	Canis mesomelas KT448280 1	0,18925035
Ursus americanus JX196366 3	Pardofelis marmorata NLN3 2	0,18925601
Ursus maritimus NC003428 31	Diplogale hosei MH464790 1	0,18925774
Neofelis nebulosa NC008450 3	Mustela nivalis T306 5	0,1892584
Lycaon pictus NC028427 2	Eumetopias jubatus NC004030 10	0,18925884
Ursus americanus JX196366 3	Panthera onca KP202264 2	0,18925945
Paradoxurus hermaphroditus NC03959	Eumetopias jubatus NC004030 10	0,18926206
Ursus arctos isabellinus 18B5 2	Canis mesomelas KT448280 1	0,18926336
Zalophus wollebaeki SRR4431565 1	Panthera leo NERO 19	0,1892651
Vulpes ferrilata NC027935 1	Lutra lutra LC050126 1	0,18926515
Lontra canadensis SRR10409165 1	Arctocephalus australis MG023139 1	0,18926637
Phocarctos hookeri NC008418 1	Atilax paludinosus T606 1	0,18926793
Vulpes zerda KJ603240 1	Meles meles T303 3	0,18926865
Nyctereutes procyonoides NC013700 3	Arctocephalus townsendi NC008420 1	0,18926899
Ursus maritimus NC003428 31	Conepatus chinga NC042596 1	0,18927038
Vulpes ferrilata NC027935 1	Galidictis fasciata DM333 1	0,1892723
Mustela kathiah NC023210 1	Galidia elegans D146 1	0,1892727
Galidictis fasciata DM333 1	Ailurus fulgens styani NC009691 1	0,18927339
Ursus americanus JX196366 3	Diplogale hosei MH464790 1	0,1892786
Lontra canadensis SRR10409165 1	Hyaena hyaena NC020669 1	0,18927889
Ursus arctos EU497665 29	Lutrogale perspicillata NC035811 1	0,18927906
Ursus thibetanus mupinensis NC00875	Xenogale naso C07XAR110 1	0,18928027
Phocarctos hookeri NC008418 1	Paradoxurus hermaphroditus NLNC 1	0,18928041
Vulpes lagopus NC026529 3	Panthera pardus japonensis KJ866876	0,18928042
Ursus spelaeus NC011112 8	Canis lupus chanco NC010340 4	0,18928057
Panthera uncia KP202269 1	Melogale moschata V0735A 1	0,1892806
Phocarctos hookeri NC008418 1	Ichneumia albicauda T603 1	0,18928072
Cryptoprocta ferox CFC13 1	Arctocephalus townsendi NC008420 1	0,18928097
Tremarctos ornatus NC009969 2	Panthera onca KP202264 2	0,18928179
Ursus americanus JX196366 3	Ictonyx striatus T299 1	0,18928237
Urocyon littoralis catalinae KP129018	Lynx rufus NC014456 3	0,1892824
Urocyon cinereoargenteus NC026723 2	Puma yagouaroundi NC028311 1	0,18928412
Speothos venaticus C48 2	Mustela sibirica AP017394 11	0,18928427
Ursus arctos pruinosus MG066703 3	Procyon lotor AB462046 3	0,1892851
Ursus arctos isabellinus 18B5 2	Canis latrans NC008093 7	0,18928532
Lynx pardinus NC028319 161	Bassaricyon neblina SRX1097850 1	0,18928567
Profelis aurata NC028299 1	Neophoca cinerea NC008419 1	0,18928589
Panthera uncia NC010638 1	Bassariscus sumichrasti SRX1099089 1	0,18928693
Eumetopias jubatus NC004030 10	Canis aureus KT448274 1	0,18928711
Potos flavus T414 1	Panthera tigris NC010642 35	0,18928839
Odobenus rosmarus NC004029 29	Mustela sibirica AP017394 11	0,18928951
Ursus maritimus NC003428 31	Ichneumia albicauda T603 1	0,18929084
Ursus maritimus GU573488 Svalbard	Ichneumia albicauda T603 1	0,18929087
Panthera leo spelaea KX258452 2	Melogale moschata KP726273 1	0,18929142
Mungos mungo/gambianus SRR77048	Melursus ursinus NC009970 2	0,18929207
Ursus arctos pruinosus MG066703 3	Lynx canadensis NC028313 1	0,18929209
Otaria byronia OTAB 1	Felis chaus NC028307 1	0,18929276

Mustela sibirica AP017394 11	Bassaricyon neblina SRX1097850 1	0,17276888
Lutrogale perspicillata NC035811 1	Bassariscus sumichrasti SRX1099089 1	0,17277224
Pusa caspica NC008431 1	Procyon lotor AB462049 4	0,17277351
Mirounga angustirostris SRR10331586 1	Martes americana NC020642 1	0,17277944
Mirounga angustirostris SRR10331586 1	Melogale moschata KP726273 1	0,17277977
Hydrurga leptonyx NC008425 1	Xenogale naso C07XAR110 1	0,17278302
Profelis aurata NC028299 1	Cystophora cristata NC008427 1	0,17278412
Cystophora cristata NC008427 1	Caracal caracal NC028306 1	0,17278416
Nyctereutes procyonoides NC013700 3	Lobodon carcinophaga NC008423 1	0,17278573
Salanoia concolor D378 1	Pusa hispida NC 008433 1	0,17278583
Pusa hispida NC 008433 1	Prionailurus planiceps NC028312 6	0,1727936
Pusa hispida NC 008433 1	Prionailurus viverrinus NC028305 1	0,17279615
Phoca vitulina NC001325 1	Lynx canadensis NC028313 1	0,17279701
Monachus monachus NC044972 5	Martes melampus NC009678 1	0,17279768
Viverra tangalunga MH464792 1	Monachus monachus NC044972 5	0,17280209
Smilodon populator MF871700 1	Mirounga angustirostris SRR10331586 1	0,17280255
Phoca groenlandica NC008429 54	Acinonyx jubatus NC005212 3	0,17280796
Procyon lotor AB462046 3	Monachus monachus NC044972 5	0,17281032
Lobodon carcinophaga NC008423 1	Urva javanica/auropunctata NC006835 1	0,17281596
Pusa hispida NC 008433 1	Civettictis civetta NC033378 1	0,17282717
Leptailurus serval NC028316 1	Halichoerus grypus NC001602 2	0,17283206
Mustela kathiah NC023210 1	Ailurus fulgens NC011124 1	0,17284182
Viverricula indica KX891751 1	Pusa sibirica NC008432 2	0,17284746
Mirounga leonina NC008422 1	Leptailurus serval NC028316 1	0,17284808
Mirounga leonina NC008422 1	Ictonyx striatus T299 1	0,17284904
Lynx canadensis NC028313 1	Cystophora cristata NC008427 1	0,17284961
Procyon lotor AB462049 4	Phoca largha NC008430 1	0,17285199
Monachus schauinslandi NC008421 1	Caracal caracal NC028306 1	0,17285632
Monachus schauinslandi NC008421 1	Lynx pardinus NC028319 161	0,17285846
Pusa hispida NC 008433 1	Lynx pardinus NC028319 161	0,17286132
Pusa hispida NC 008433 1	Leopardus jacobita NC028322 1	0,17286295
Viverricula indica NC025296 2	Phoca largha NC008430 1	0,17286398
Phoca largha NC008430 1	Leptailurus serval NC028316 1	0,17286567
Martes flavigula NC012141 3	Ailurus fulgens NC011124 1	0,17286812
Pusa caspica NC008431 1	Acinonyx jubatus NC005212 3	0,17286889
Profelis aurata NC028299 1	Monachus monachus NC044972 5	0,17288052
Ursus americanus JX196366 3	Phoca vitulina NC001325 1	0,1728843
Mustela altaica NC021751 1	Halichoerus grypus NC001602 2	0,17289667
Neovison vison NC020641 3	Hemigalus derbyanus MH464791 1	0,17290124
Erignathus barbatus NC008426 1	Bassaricyon neblina SRX1097850 1	0,17290466
Pusa caspica NC008431 1	Ailurus fulgens NC011124 1	0,17290567
Suricata suricatta SSM10 1	Leptonychotes weddellii NC008424 1	0,17290601
Galidia elegans D146 1	Cynogale bennetti KY117544 1	0,1729137
Viverricula indica KX891751 1	Pusa caspica NC008431 1	0,1729148
Hemigalus derbyanus MH464791 1	Arctonyx collaris NC020645 1	0,17291613
Hydrurga leptonyx NC008425 1	Bdeogale nigripes GLC15 1	0,17291722
Procyon lotor AB462049 4	Phoca fasciata NC008428 1	0,17291731
Pusa sibirica NC008432 2	Leopardus geoffroyi NC028320 1	0,17292129
Monachus schauinslandi NC008421 1	Canis adustus KT448271 1	0,17292276
Pusa hispida NC 008433 1	Genetta servalina NC024568 2	0,17292373
Smilodon populator MF871700 1	Phoca fasciata NC008428 1	0,17292398
Phoca groenlandica NC008429 54	Lynx lynx NC027083 4	0,17292468
Phoca groenlandica NC008429 54	Lynx rufus NC014456 3	0,17292494
Monachus schauinslandi NC008421 1	Lynx canadensis NC028313 1	0,17292605
Lobodon carcinophaga NC008423 1	Ichneumia albicauda T603 1	0,17292918
Otocolobus manul NC028323 1	Mirounga angustirostris SRR10331586 1	0,17292932
Phoca largha NC008430 1	Lynx canadensis NC028313 1	0,17293184
Lobodon carcinophaga NC008423 1	Arctictis binturong T605 2	0,17293766
Lutra sumatrana NC035810 1	Bassaricyon neblina SRX1097850 1	0,17296904
Cystophora cristata NC008427 1	Cynictis penicillata T375 1	0,17297333
Monachus monachus NC044972 5	Diplogale hosei MH464790 1	0,17297645
Viverra tangalunga MH464792 1	Monachus schauinslandi NC008421 1	0,17298038
Mustela putorius NC020638 4	Mirounga angustirostris SRR10331586 1	0,1729835
Mirounga leonina NC008422 1	Civettictis civetta GLC19 1	0,17298371
Pusa sibirica NC008432 2	Lynx canadensis NC028313 1	0,17298567
Vulpes vulpes NC008434 5	Lobodon carcinophaga NC008423 1	0,17298791
Salanoia concolor D378 1	Phoca largha NC008430 1	0,17299014
Ursus arctos pruinosus MG066703 3	Mirounga leonina NC008422 1	0,17299162
Phoca fasciata NC008428 1	Galerella sanguinea T378 1	0,17299214
Prionailurus planiceps NC028312 6	Phoca fasciata NC008428 1	0,17299326
Prionailurus viverrinus NC028305 1	Phoca fasciata NC008428 1	0,17299525
Pusa hispida NC 008433 1	Poecilogale albinucha T602 1	0,17299837
Phoca fasciata NC008428 1	Ailuropoda melanoleuca NC009492 5	0,17300047
Neovison vison NC020641 3	Mephitis mephitis NC020648 1	0,17300771
Cystophora cristata NC008427 1	Canis adustus KT448271 1	0,17300808
Ommatophoca rossii AY377287etc 1	Meles leucurus NC039173 4	0,17301138
Hydrurga leptonyx NC008425 1	Canis lupus familiaris NC002008 1231	0,17301549
Prionailurus viverrinus NC028305 1	Monachus monachus NC044972 5	0,17301704
Spilogale putorius NC010497 1	Mustela erminea T305 2	0,17301747
Mustela itatsi NC034330 19	Bassaricyon neblina SRX1097850 1	0,1730381
Mirounga angustirostris SRR10331586 1	Chrotogale owstoni T607 1	0,17304635
Viverricula indica KX891745 1	Pusa sibirica NC008432 2	0,17304949
Prionailurus rubiginosus NC028304 2	Mirounga leonina NC008422 1	0,17304952
Hydrurga leptonyx NC008425 1	Urva brachyura KY117547 1	0,1730507
Phoca fasciata NC008428 1	Genetta servalina NC024568 2	0,17305233
Phoca groenlandica NC008429 54	Genetta servalina NC024568 2	0,17305233
Pusa caspica NC008431 1	Lynx rufus NC014456 3	0,17305297
Mustela erminea T305 2	Leopardus wiedii NC028318 1	0,17305338
Mustela erminea T305 2	Arctodus simus NC011116 1	0,17305788
Puma yagouaroundi NC028311 1	Monachus schauinslandi NC008421 1	0,17306013
Prionailurus rubiginosus NC028304 2	Phoca fasciata NC008428 1	0,17306118
Phoca fasciata NC008428 1	Leopardus tigrinus NC028317 1	0,17306261
Halichoerus grypus NC001602 2	Canis adustus KT448271 1	0,17306739
Monachus schauinslandi NC008421 1	Ailuropoda melanoleuca NC009492 5	0,1730745
Ursus arctos AP012576 6	Mirounga angustirostris SRR10331586 1	0,17308086
Paradoxurus hermaphroditus NLNC 1	Canis adustus KT448271 1	0,17308283
Puma concolor NC016470 22	Monachus monachus NC044972 5	0,17308283
Nandinia binotata NC024567 1	Meles anakuma NC009677 1	0,17310156
Lutra lutra LC050126 1	Bassaricyon neblina SRX1097850 1	0,17310363
Leopardus pardalis NC028315 1	Halichoerus grypus NC001602 2	0,17310533
Viverricula indica KX891751 1	Canis adustus KT448271 1	0,17310745
Leptonychotes weddellii NC008424 1	Cynictis penicillata T375 1	0,173108
Smilodon populator MF871700 1	Canis adustus KT448271 1	0,17310858

Ursus arctos EU497665 29	Felis margarita NC028308 1	0,18929332
Ursus spelaeus EU327344 13	Helogale parvula SRR7637809 1	0,18929338
Panthera tigris amoyensis NC014770 2	Aonyx cinerea NC035814 2	0,1892952
Crocuta crocuta NC020670 3	Arctodus simus NC011116 1	0,18929559
Proteles cristata T393 6	Arctodus simus NC011116 1	0,18929578
Ursus spelaeus NC011112 8	Mungotictis decemlineata NC027828 1	0,18929604
Procyon lotor AB462049 4	Ailuropoda melanoleuca NC009492 5	0,18929705
Ursus arctos GU573486 5	Leopardus tigrinus NC028317 1	0,18929969
Ursus arctos GU573486 5	Prionailurus viverrinus NC028305 1	0,18929988
Panthera leo spelaea KX258452 2	Aonyx cinerea NC035814 2	0,18930039
Ursus thibetanus formosanus NC00933	Parahyaena brunnea NC038159 15	0,18930309
Galidia elegans D146 1	Canis aureus KT448274 1	0,18930456
Ursus arctos isabellinus 18B5 2	Mephitis mephitis NC020648 1	0,18930487
Lutrogale perspicillata NC035811 1	Genetta abyssinica MG489822 1	0,18930501
Panthera onca KP202264 2	Melursus ursinus NC009970 2	0,18930569
Ursus maritimus NC003428 31	Canis aureus KT448274 1	0,18931329
Panthera uncia NC010638 1	Canis lupus familiaris NC002008 1231	0,18931811
Helarctos malayanus NC009968 2	Chrysocyon brachyurus NC024172 1	0,18931909
Atilax paludinosus T606 1	Arctocephalus forsteri NC004023 28	0,18931918
Ursus maritimus GU573488 Svalbard	Diplogale hosei MH464790 1	0,18931937
Ursus maritimus NC003428 31	Canis lupus familiaris NC002008 1231	0,18931989
Xenogale naso C07XAR110 1	Arctocephalus townsendi NC008420 1	0,18933369
Prionodon linsang ERR2391707 1	Martes zibellina NC011579 39	0,18933493
Lontra canadensis SRR10409165 1	Canis latrans NC008093 7	0,18933529
Helogale parvula SRR7637809 1	Arctocephalus gazella BK010918 1	0,18933573
Nyctereutes procyonoides NC013700 3	Lutra sumatrana NC035810 1	0,18933627
Potos flavus T414 1	Nyctereutes procyonoides NC013700 3	0,18933687
Lycalopex sechurae KT448284 1	Enhydra lutris NC009692 1	0,1893374
Ursus maritimus GU573488 Svalbard	Conepatus chinga NC042596 1	0,18933775
Paradoxurus hermaphroditus NC03959	Arctocephalus australis MG023139 1	0,18933827
Paradoxurus jerdoni MH464793 1	Arctocephalus gazella BK010918 1	0,18933982
Paguma larvata PDDS11 2	Arctocephalus australis MG023139 1	0,18934005
Ursus arctos GU573486 5	Canis lupus chanco NC010340 4	0,18934035
Canis latrans NC008093 7	Ailurus fulgens NC011124 1	0,18934178
Panthera uncia KP202269 1	Ailurus fulgens styani NC009691 1	0,18934241
Ursus americanus JX196366 3	Mephitis mephitis NC020648 1	0,18934244
Vulpes lagopus NC026529 3	Taxidea taxus NC020646 1	0,18934247
Paradoxurus hermaphroditus NC03959	Melogale moschata V0735A 1	0,18934262
Lutra lutra NC011358 9	Arctictis binturong T605 2	0,18934421
Panthera uncia NC010638 1	Mustela eversmannii NC028013 1	0,18934679
Otocyon megalotis SAF1 2	Mustela sibirica AP017394 11	0,18934715
Martes melampus NC009678 1	Crocuta crocuta NC020670 3	0,18934725
Lycalopex sechurae KT448284 1	Lutra sumatrana NC035810 1	0,18934746
Prionodon linsang ERR2391707 1	Martes foina NC020643 1	0,18934759
Otocyon megalotis SAF1 2	Lutra sumatrana NC035810 1	0,18934813
Paradoxurus hermaphroditus NLNC 1	Neophoca cinerea NC008419 1	0,18934898
Vulpes zerda KJ603240 1	Prionailurus planiceps NC028312 6	0,18934956
Nyctereutes procyonoides NC013700 3	Martes foina NC020643 1	0,18935005
Eumetopias jubatus NC004030 10	Canis lupus familiaris NC002008 1231	0,18935126
Lutrogale perspicillata NC035811 1	Hyaena hyaena NC020669 1	0,18935238
Proteles cristata T393 6	Enhydra lutris NC009692 1	0,18935315
Mustela nigripes NC024942 1	Crocuta crocuta NC020670 3	0,18935381
Ursus maritimus GU573488 Svalbard	Cryptoprocta ferox CFC13 1	0,18935604
Ursus thibetanus formosanus NC00933	Urva javanica T413 1	0,18935886
Neofelis nebulosa NC008450 3	Gulo gulo NC009685 3	0,18936054
Ursus thibetanus formosanus NC00933	Galidia elegans D146 1	0,18936056
Ursus maritimus NC003428 31	Felis chaus NC028307 1	0,18936107
Panthera onca NC022842 1	Lutrogale perspicillata NC035811 1	0,18936312
Ursus arctos AP012576 6	Lutrogale perspicillata NC035811 1	0,18936317
Speothos venaticus C48 2	Profelis aurata NC028299 1	0,18936335
Paguma larvata PDDS11 2	Galictis vittata T412 1	0,18936567
Pardofelis marmorata NLN3 2	Ailuropoda melanoleuca NC009492 5	0,18936621
Ursus spelaeus EU327344 13	Galerella sanguinea T378 1	0,18936896
Zalophus wollebaeki SRR4431565 1	Hyaena hyaena NC020669 1	0,1893698
Panthera leo spelaea KX258452 2	Neovison vison NC020641 3	0,18937086
Ursus maritimus NC003428 31	Mephitis mephitis NC020648 1	0,18937191
Melogale moschata V0735A 1	Canis aureus KT448274 1	0,18937458
Suricata suricatta SSM10 1	Martes flavigula NC012141 3	0,18937658
Melursus ursinus NC009970 2	Lycaon pictus NC028427 2	0,18937685
Ursus arctos AP012576 6	Prionailurus rubiginosus NC028304 2	0,18937735
Vulpes lagopus NC026529 3	Panthera leo NERO 19	0,18937807
Cynictis penicillata T375 1	Arctocephalus forsteri NC004023 28	0,18938185
Ursus americanus JX196366 3	Mungotictis decemlineata NC027828 1	0,18938233
Zalophus californianus NC008416 1	Hyaena hyaena NC020669 1	0,18938297
Panthera pardus NC010641 5	Lutra lutra NC011358 9	0,18938665
Cuon alpinus NC013445 3	Crossarchus platycephalus C7R66 1	0,18938957
Galidictis fasciata DM333 1	Arctocephalus forsteri NC004023 28	0,18939036
Panthera pardus NC010641 5	Arctocephalus pusillus NC008417 1	0,18939216
Prionailurus viverrinus NC028305 1	Cuon alpinus NC013445 3	0,18939741
Suricata suricatta SSM10 1	Melogale moschata KP726273 1	0,18939927
Ursus thibetanus formosanus NC00933	Canis mesomelas KT448280 1	0,18939935
Hyaena hyaena NC020669 1	Eumetopias jubatus NC004030 10	0,1894004
Lontra canadensis SRR10409165 1	Cynictis penicillata T375 1	0,18940048
Spilogale putorius NC010497 1	Lycaon pictus NC028427 2	0,18940084
Suricata suricatta SSM10 1	Mustela itatsi NC034330 19	0,18940131
Vulpes ferrilata NC027935 1	Urva semitorquata MH464789 1	0,18940295
Suricata suricatta SSM10 1	Ailurus fulgens styani NC009691 1	0,18940313
Lutrogale perspicillata NC035811 1	Urva semitorquata MH464789 1	0,18940525
Galidia elegans D146 1	Canis latrans NC008093 7	0,1894062
Procyon lotor AB462046 3	Bdeogale nigripes GLC15 1	0,18940647
Panthera tigris NC010642 35	Martes flavigula NC012141 3	0,18940906
Melogale moschata V0735A 1	Cryptoprocta ferox CFC13 1	0,18941015
Poecilogale albinucha T602 1	Urva javanica T413 1	0,18941174
Ichneumia albicauda T603 1	Bassaricyon neblina SRX1097850 1	0,1894128
Panthera uncia KP202269 1	Mustela itatsi NC034330 19	0,18941416
Felis chaus NC028307 1	Arctocephalus townsendi NC008420 1	0,18941468
Mustela nivalis T306 5	Galidia elegans D146 1	0,18941504
Viverra zibetha T609 1	Urocyon littoralis catalinae KP129018 1	0,18941504
Viverra tangalunga MH464792 1	Otocyon megalotis SAF1 2	0,18941565
Nyctereutes procyonoides NC013700 3	Mustela altaica NC021751 1	0,18941572
Panthera onca KP202264 2	Martes zibellina NC011579 39	0,18941621
Ursus maritimus NC003428 31	Lontra canadensis SRR10409165 1	0,18941793
Ursus arctos EU497665 29	Bassaricyon neblina SRX1097850 1	0,18941855

Procyon lotor AB462049 4	Aonyx cinerea NC035814 2	0,1731106
Salanoia concolor D378 1	Cystophora cristata NC008427 1	0,17311099
Profelis aurata NC028299 1	Mirounga leonina NC008422 1	0,17311716
Nandinia binotata NC024567 1	Arctodus simus NC011116 1	0,17311829
Lynx lynx NC027083 4	Cystophora cristata NC008427 1	0,17311877
Phoca fasciata NC008428 1	Lutrogale perspicillata NC035811 1	0,17311968
Mirounga angustirostris SRR10331586 1	Melogale moschata NC020644 1	0,17312053
Mungotictis decemlineata NC027828 1	Lobodon carcinophaga NC008423 1	0,17312486
Monachus schauinslandi NC008421 1	Leopardus pardalis NC028315 1	0,17312893
Panthera pardus japonensis KJ866876 8	Lobodon carcinophaga NC008423 1	0,17312998
Prionailurus bengalensis CKM45 20	Phoca groenlandica NC008429 54	0,17313233
Zalophus californianus NC008416 1	Mustela erminea T305 2	0,17313696
Phoca fasciata NC008428 1	Civettictis civetta NC033378 1	0,17315601
Mustela eversmannii NC028013 1	Mirounga leonina NC008422 1	0,17317162
Mustela nigripes NC024942 1	Bassaricyon neblina SRX1097850 1	0,1731727
Mustela sibirica AP017394 11	Ailurus fulgens NC011124 1	0,17317708
Pusa sibirica NC008432 2	Lynx pardinus NC028319 161	0,17318803
Pusa hispida NC 008433 1	Felis nigripes NC028309 1	0,17319787
Smilodon populator MF871700 1	Monachus schauinslandi NC008421 1	0,17320918
Monachus monachus NC044972 5	Martes martes T302 3	0,17321379
Mirounga leonina NC008422 1	Lutra lutra NC011358 9	0,17322952
Lutra lutra LC050126 1	Ailurus fulgens NC011124 1	0,17322969
Martes americana NC020642 1	Ailurus fulgens styani NC009691 1	0,17324117
Monachus monachus NC044972 5	Gulo gulo NC009685 3	0,17324398
Mirounga leonina NC008422 1	Lynx rufus NC014456 3	0,17325011
Phoca fasciata NC008428 1	Eupleres goudotii D128 1	0,17325103
Pusa sibirica NC008432 2	Mustela altaica NC021751 1	0,17325194
Pardofelis marmorata NLN3 2	Erignathus barbatus NC008426 1	0,17325316
Felis nigripes NC028309 1	Erignathus barbatus NC008426 1	0,17325333
Lobodon carcinophaga NC008423 1	Atilax paludinosus T606 1	0,173254
Lobodon carcinophaga NC008423 1	Canis anthus NC027956 2	0,17325617
Pusa sibirica NC008432 2	Prionailurus rubiginosus NC028304 2	0,17325644
Prionailurus planiceps KY682741 4	Phoca groenlandica NC008429 54	0,17326294
Prionailurus rubiginosus NC028304 2	Phoca groenlandica NC008429 54	0,17326358
Pusa hispida NC 008433 1	Leopardus guigna NC028321 1	0,17326683
Lobodon carcinophaga NC008423 1	Chrysocyon brachyurus NC024172 1	0,17327124
Mephitis mephitis NC020648 1	Hydrurga leptonyx NC008425 1	0,17327248
Monachus monachus NC044972 5	Melogale moschata NC020644 1	0,17327936
Leptonychotes weddellii NC008424 1	Canis lupus chanco NC010340 4	0,17328453
Callorhinus ursinus NC008415 1	Arctonyx collaris NC020645 1	0,17330128
Cystophora cristata NC008427 1	Ailurus fulgens NC011124 1	0,17331018
Mirounga leonina NC008422 1	Lynx canadensis NC028313 1	0,17331733
Ictonyx striatus T299 1	Erignathus barbatus NC008426 1	0,17332034
Procyon lotor AB462046 3	Phoca largha NC008430 1	0,17332375
Viverricula indica KX891745 1	Phoca vitulina NC001325 1	0,1733311
Monachus schauinslandi NC008421 1	Leopardus wiedii NC028318 1	0,1733312
Puma yagouaroundi NC028311 1	Phoca groenlandica NC008429 54	0,17333122
Panthera onca KP202264 2	Lobodon carcinophaga NC008423 1	0,17333196
Pusa hispida NC 008433 1	Felis silvestris lybica KP202275 4	0,17333259
Puma yagouaroundi NC028311 1	Mirounga angustirostris SRR10331586 1	0,1733329
Viverra zibetha T609 1	Mirounga angustirostris SRR10331586 1	0,1733354
Prionailurus viverrinus NC028305 1	Phoca largha NC008430 1	0,17333917
Procyon lotor AB462046 3	Halichoerus grypus NC001602 2	0,17336136
Prionailurus bengalensis CKM45 20	Halichoerus grypus NC001602 2	0,17337641
Melogale moschata NC020644 1	Ailurus fulgens styani NC009691 1	0,17338049
Panthera uncia KP202269 1	Leptonychotes weddellii NC008424 1	0,17338623
Pusa caspica NC008431 1	Hemigalus derbyanus MH464791 1	0,17338628
Spilogale putorius NC010497 1	Monachus monachus NC044972 5	0,17338711
Monachus schauinslandi NC008421 1	Crossarchus platycephalus C7R66 1	0,17338718
Pusa sibirica NC008432 2	Genetta abyssinica MG489822 1	0,1733886
Phoca vitulina NC001325 1	Ailurus fulgens NC011124 1	0,17338867
Mirounga leonina NC008422 1	Leopardus geoffroyi NC028320 1	0,17338964
Pusa sibirica NC008432 2	Felis silvestris lybica KP202275 4	0,17339043
Pusa sibirica NC008432 2	Prionailurus planiceps KY682741 4	0,1733907
Pusa hispida NC 008433 1	Bassaricyon neblina SRX1097850 1	0,17339078
Hyaena hyaena NC020669 1	Cystophora cristata NC008427 1	0,17339164
Mustela altaica NC021751 1	Monachus schauinslandi NC008421 1	0,17339469
Monachus monachus NC044972 5	Chrotogale owstoni T607 1	0,17339638
Prionailurus rubiginosus NC028304 2	Monachus schauinslandi NC008421 1	0,1733973
Viverricula indica KX891751 1	Phoca vitulina NC001325 1	0,17339809
Mirounga angustirostris SRR10331586 1	Leopardus pardalis NC028315 1	0,17339988
Mirounga angustirostris SRR10331586 1	Genetta servalina NC024568 2	0,17340261
Procyon lotor AB462049 4	Monachus monachus NC044972 5	0,17341609
Taxidea taxus NC020646 1	Eumetopias jubatus NC004030 10	0,1734179
Monachus monachus NC044972 5	Catopuma temminckii NC027115 41	0,17341961
Hydrurga leptonyx NC008425 1	Canis lupus chanco NC010340 4	0,17342629
Nandinia binotata NC024567 1	Martes americana NC020642 1	0,17344168
Mungos mungo MMC7 1	Mirounga leonina NC008422 1	0,17344581
Mustela sibirica NC020637 6	Ailurus fulgens NC011124 1	0,17344639
Felis chaus NC028307 1	Erignathus barbatus NC008426 1	0,17345566
Puma concolor NC016470 22	Erignathus barbatus NC008426 1	0,17345566
Zalophus wollebaeki SRR4431565 1	Taxidea taxus NC020646 1	0,17346695
Ommatophoca rossii AY377287etc 1	Martes melampus NC009678 1	0,17346765
Poecilogale albinucha T602 1	Mirounga angustirostris SRR10331586 1	0,17346993
Taxidea taxus NC020646 1	Hemigalus derbyanus MH464791 1	0,1734793
Panthera pardus NC010641 5	Lobodon carcinophaga NC008423 1	0,17349979
Lynx canadensis NC028313 1	Halichoerus grypus NC001602 2	0,17350806
Pusa sibirica NC008432 2	Procyon lotor AB462049 4	0,17351437
Procyon lotor AB462046 3	Aonyx cinerea NC035814 2	0,17351449
Viverricula indica KX891745 1	Pusa caspica NC008431 1	0,17352079
Phoca fasciata NC008428 1	Mungos mungo/gambianus SRR7704821 1	0,17352186
Suricata suricatta SSM10 1	Lobodon carcinophaga NC008423 1	0,17352241
Mirounga leonina NC008422 1	Leopardus guigna NC028321 1	0,17352436
Phoca largha NC008430 1	Hemigalus derbyanus MH464791 1	0,1735266
Paguma larvata PDDS11 2	Lobodon carcinophaga NC008423 1	0,17352855
Mirounga angustirostris SRR10331586 1	Lynx lynx NC027083 4	0,17353112
Monachus schauinslandi NC008421 1	Leptailurus serval NC028316 1	0,17353198
Viverricula indica KX891751 1	Phoca largha NC008430 1	0,17353255
Lycaon pictus NC028427 2	Hydrurga leptonyx NC008425 1	0,17353436
Mirounga angustirostris SRR10331586 1	Leptailurus serval NC028316 1	0,17353556
Mirounga leonina NC008422 1	Acinonyx jubatus NC005212 3	0,17353652
Poecilogale albinucha T602 1	Phoca vitulina NC001325 1	0,17354028
Ursus arctos pruinosus MG066703 3	Mirounga angustirostris SRR10331586 1	0,17354418

Melogale moschata KP726273 1	Crocuta crocuta NC020670 3	0,18941878
Zalophus wollebaeki SRR4431565 1	Vulpes lagopus NC026529 3	0,18941881
Ursus maritimus NC003428 31	Canis latrans NC008093 7	0,18941927
Procyon lotor AB462049 4	Parahyaena brunnea NC038159 15	0,18941984
Profelis aurata NC028299 1	Phocarctos hookeri NC008418 1	0,18942028
Nyctereutes procyonoides NC013700 3	Ictonyx striatus T299 1	0,18942249
Crocuta crocuta NC020670 3	Arctocephalus pusillus NC008417 1	0,18942291
Prionodon pardicolor NC024569 2	Poecilogale albinucha T602 1	0,18942353
Panthera tigris NC010642 35	Canis anthus NC027956 2	0,18942563
Ursus arctos isabellinus 18B5 2	Prionodon pardicolor NC024569 2	0,18942586
Ursus arctos isabellinus 18B5 2	Panthera pardus japonensis KJ866876	0,18942596
Ursus maritimus NC003428 31	Prionailurus planiceps NC028312 6	0,18942799
Gulo gulo NC009685 3	Cuon alpinus NC013445 3	0,18942817
Panthera onca NC022842 1	Lontra canadensis SRR10409165 1	0,1894284
Ursus arctos EU497665 29	Felis chaus NC028307 1	0,18942843
Ursus arctos GU573486 5	Ichneumia albicauda T603 1	0,18943164
Ursus spelaeus NC011112 8	Canis latrans NC008093 7	0,18943293
Zalophus californianus NC008416 1	Vulpes zerda KJ603240 1	0,18943319
Ursus arctos GU573486 5	Felis silvestris lybica KP202275 4	0,18943396
Ursus arctos GU573486 5	Felis chaus NC028307 1	0,18943465
Parahyaena brunnea NC038159 15	Melursus ursinus NC009970 2	0,1894362
Hyaena hyaena NC020669 1	Galictis vittata T412 1	0,18943821
Procyon lotor AB462046 3	Canis aureus KT448274 1	0,18943843
Ursus thibetanus thibetanus NC01111	Parahyaena brunnea NC038159 15	0,18943924
Panthera onca KP202264 2	Galictis vittata T412 1	0,18943949
Prionodon pardicolor NC024569 2	Melursus ursinus NC009970 2	0,18943989
Pardofelis marmorata NLN3 2	Melursus ursinus NC009970 2	0,18944132
Ursus thibetanus mupinensis NC00875	Chrysocyon brachyurus NC024172 1	0,18944244
Ursus arctos AP012576 6	Lynx pardinus NC028319 161	0,18944339
Ursus americanus JX196366 3	Mungos mungo/gambianus SRR77048	0,18944471
Ursus arctos isabellinus 18B5 2	Diplogale hosei MH464790 1	0,189447
Ursus arctos EU497665 29	Canis lupus familiaris NC002008 1231	0,18945476
Cuon alpinus NC013445 3	Arctotherium sp NC030174 1	0,1894563
Ursus thibetanus mupinensis NC00875	Canis mesomelas KT448280 1	0,18945834
Prionodon linsang ERR2391707 1	Lutra lutra NC011358 9	0,18945843
Tremarctos ornatus NC009969 2	Urva javanica/auropunctata NC006835	0,18945956
Neofelis nebulosa NC008450 3	Bassariscus sumichrasti SRX1099089 1	0,18946108
Suricata suricatta SSM10 1	Lutra lutra NC011358 9	0,18946123
Paradoxurus hermaphroditus NC03959	Lutra lutra LC050126 1	0,1894628
Panthera uncia NC010638 1	Eumetopias jubatus NC004030 10	0,18946475
Mustela itatsi NC034330 19	Helogale parvula SRR7637809 1	0,18946843
Urva javanica T413 1	Arctocephalus forsteri KT693377 17	0,18946846
Vulpes ferrilata NC027935 1	Martes melampus NC009678 1	0,1894691
Vulpes corsac NC023958 1	Martes zibellina NC011579 39	0,18946923
Urva brachyura KY117547 1	Arctocephalus gazella BK010918 1	0,18946955
Vulpes lagopus NC026529 3	Atilax paludinosus T606 1	0,18947028
Urva javanica T413 1	Ailurus fulgens NC011124 1	0,18947084
Vulpes corsac NC023958 1	Tremarctos ornatus NC009969 2	0,18947102
Xenogale naso C07XAR110 1	Arctocephalus gazella BK010918 1	0,18947114
Cynictis penicillata T375 1	Ailurus fulgens styani NC009691 1	0,18947119
Mustela sibirica NC020637 6	Galidia elegans D146 1	0,18947317
Vulpes lagopus NC026529 3	Mustela putorius NC020638 4	0,18947323
Vulpes lagopus NC026529 3	Chrotogale owstoni T607 1	0,18947402
Mungotictis decemlineata NC027828 1	Arctocephalus forsteri KT693377 17	0,18947405
Galidia elegans D146 1	Arctocephalus australis MG023139 1	0,18947423
Panthera uncia KP202269 1	Ailurus fulgens NC011124 1	0,18947726
Vulpes lagopus NC026529 3	Arctotherium sp NC030174 1	0,18947813
Ursus maritimus GU573488 Svalbard	Helogale parvula SRR7637809 1	0,18948178
Vulpes ferrilata NC027935 1	Genetta genetta T297 1	0,18948199
Vulpes lagopus NC026529 3	Panthera uncia NC010638 1	0,18948254
Panthera onca KP202264 2	Arctocephalus forsteri KT693377 17	0,18948257
Vulpes zerda KJ603240 1	Genetta genetta T297 1	0,18948281
Panthera onca KP202264 2	Martes americana NC020642 1	0,18948303
Melursus ursinus NC009970 2	Conepatus chinga NC042596 1	0,18948404
Panthera uncia KP202269 1	Lontra canadensis SRR10409165 1	0,18948467
Vulpes zerda KJ603240 1	Parahyaena brunnea NC038159 15	0,18948476
Urocyon cinereoargenteus NC026723 2	Leopardus pardalis T262 1	0,18948486
Panthera tigris NC010642 35	Ailurus fulgens NC011124 1	0,18948521
Parahyaena brunnea NC038159 15	Nyctereutes procyonoides NC013700 3	0,18948549
Urocyon littoralis catalinae KP129018	Neovison vison NC020641 3	0,18948632
Poecilogale albinucha T602 1	Galidictis fasciata DM333 1	0,18948701
Proteles cristata T393 6	Mustela sibirica NC020637 6	0,18948999
Zalophus wollebaeki SRR4431565 1	Paradoxurus hermaphroditus NC03959	0,18949081
Ursus thibetanus laniger MH281753 2	Xenogale naso C07XAR110 1	0,18949288
Ursus arctos GU573486 5	Canis latrans NC008093 7	0,18949297
Ursus thibetanus mupinensis NC00875	Parahyaena brunnea NC038159 15	0,18949361
Ursus arctos isabellinus 18B5 2	Felis catus NC001700 2	0,18949431
Ursus spelaeus NC011112 8	Bdeogale nigripes GLC15 1	0,18949459
Ursus thibetanus laniger MH281753 2	Galidia elegans D146 1	0,18949509
Speothos venaticus C48 2	Martes pennanti NC020664 16	0,18949549
Ursus arctos pruinosus MG066703 3	Felis margarita NC028308 1	0,18949552
Ursus americanus JX196366 3	Panthera leo NERO 19	0,18949561
Ictonyx striatus T299 1	Hyaena hyaena NC020669 1	0,18949758
Panthera tigris NC010642 35	Otaria byronia OTAB 1	0,18949847
Ursus arctos GU573486 5	Prionailurus planiceps NC028312 6	0,18950163
Puma yagouaroundi NC028311 1	Ailuropoda melanoleuca NC009492 5	0,18950309
Panthera tigris amoyensis NC014770 2	Martes foina NC020643 1	0,18950585
Ursus maritimus GU573488 Svalbard	Mephitis mephitis NC020648 1	0,18950668
Zalophus wollebaeki SRR4431565 1	Canis aureus KT448274 1	0,18950991
Zalophus californianus NC008416 1	Otocyon megalotis SAF1 2	0,18951041
Ictonyx striatus T299 1	Canis aureus KT448274 1	0,18951249
Cynictis penicillata T375 1	Cuon alpinus NC013445 3	0,18951547
Lontra canadensis SRR10409165 1	Canis lupus chanco NC010340 4	0,18951567
Vulpes lagopus NC026529 3	Gulo gulo NC009685 3	0,18951568
Nasua nasua NC020647 1	Eumetopias jubatus NC004030 10	0,18952234
Mephitis mephitis NC020648 1	Chrysocyon brachyurus NC024172 1	0,18952242
Spilogale putorius NC010497 1	Lynx pardinus NC028319 161	0,18952911
Spilogale putorius NC010497 1	Prionailurus bengalensis CKM45 20	0,18953118
Mephitis mephitis NC020648 1	Urva javanica/auropunctata NC006835	0,18953383
Canis lupus familiaris NC002008 1231	Ailuropoda melanoleuca NC009492 5	0,18953539
Vulpes lagopus NC026529 3	Enhydra lutris NC009692 1	0,18953553
Vulpes vulpes NC008434 5	Urva javanica T413 1	0,1895368
Galidictis fasciata DM333 1	Bassaricyon neblina SRX1097850 1	0,18953773

Monachus monachus NC044972 5	Fossa fossana D350 1	0,17354715
Mirounga leonina NC008422 1	Civettictis civetta NC033378 1	0,17355949
Nandinia binotata NC024567 1	Canis adustus KT448271 1	0,17356104
Viverricula indica KX891751 1	Halichoerus grypus NC001602 2	0,17356764
Prionailurus bengalensis NC028301 12	Halichoerus grypus NC001602 2	0,17357506
Nandinia binotata NC024567 1	Mustela eversmannii NC028013 1	0,17357577
Nandinia binotata NC024567 1	Mustela itatsi NC034330 19	0,17357661
Lobodon carcinophaga NC008423 1	Urva brachyura KY117547 1	0,17358921
Phoca largha NC008430 1	Ailurus fulgens NC011124 1	0,17359047
Cystophora cristata NC008427 1	Cryptoprocta ferox CFC13 1	0,17359065
Lobodon carcinophaga NC008423 1	Eupleres goudotii D128 1	0,17359208
Pusa sibirica NC008432 2	Leopardus jacobita NC028322 1	0,1735952
Pusa hispida NC 008433 1	Civettictis civetta GLC19 1	0,17359801
Ommatophoca rossii AY377287etc 1	Leopardus pardalis T262 1	0,1735982
Viverricula indica KX891745 1	Phoca largha NC008430 1	0,17360023
Phoca groenlandica NC008429 54	Leptailurus serval NC028316 1	0,17360064
Phoca largha NC008430 1	Leopardus geoffroyi NC028320 1	0,17360793
Pusa hispida NC 008433 1	Ailuropoda melanoleuca NC009492 5	0,17361057
Panthera tigris amoyensis NC014770 2	Hydrurga leptonyx NC008425 1	0,17361175
Ommatophoca rossii AY377287etc 1	Gulo gulo NC009685 3	0,17361777
Procyon lotor AB462049 4	Halichoerus grypus NC001602 2	0,17363058
Martes melampus NC009678 1	Ailurus fulgens NC011124 1	0,17363934
Nandinia binotata NC024567 1	Lutra sumatrana NC035810 1	0,17363951
Mustela eversmannii NC028013 1	Ailurus fulgens NC011124 1	0,17364807
Phoca fasciata NC008428 1	Chrotogale owstoni T607 1	0,17365013
Prionailurus bengalensis NC028301 12	Ommatophoca rossii AY377287etc 1	0,17365293
Mirounga leonina NC008422 1	Felis chaus NC028307 1	0,17365529
Panthera pardus japonensis KJ866876 8	Leptonychotes weddellii NC008424 1	0,17365555
Paguma larvata PDDS11 2	Mirounga leonina NC008422 1	0,17365612
Mirounga leonina NC008422 1	Genetta genetta T297 1	0,17365839
Otocolobus manul NC028323 1	Cystophora cristata NC008427 1	0,17365983
Viverra zibetha T609 1	Canis adustus KT448271 1	0,1736626
Phoca largha NC008430 1	Lutrogale perspicillata NC035811 1	0,17366344
Pusa hispida NC 008433 1	Prionailurus rubiginosus NC028304 2	0,17366874
Prionailurus rubiginosus NC028304 2	Mirounga angustirostris SRR10331586 1	0,17366904
Profelis aurata NC028299 1	Mirounga angustirostris SRR10331586 1	0,17366987
Prionailurus viverrinus NC028305 1	Mirounga angustirostris SRR10331586 1	0,17367071
Ommatophoca rossii AY377287etc 1	Mustela kathiah NC023210 1	0,17367394
Smilodon populator MF871700 1	Pusa sibirica NC008432 2	0,17367463
Viverra zibetha T609 1	Monachus monachus NC044972 5	0,17368786
Mirounga angustirostris SRR10331586 1	Diplogale hosei MH464790 1	0,1736903
Phoca largha NC008430 1	Canis adustus KT448271 1	0,17369874
Nandinia binotata NC024567 1	Lutra lutra NC011358 9	0,17370407
Monachus monachus NC044972 5	Leopardus colocolo NC028314 1	0,17370883
Suricata suricatta SSM10 1	Hydrurga leptonyx NC008425 1	0,17372452
Phoca fasciata NC008428 1	Mungos mungo MMC7 1	0,17372544
Erignathus barbatus NC008426 1	Cryptoprocta ferox CFC13 1	0,17372561
Salanoia concolor D378 1	Phoca fasciata NC008428 1	0,17372614
Monachus schauinslandi NC008421 1	Lynx lynx NC027083 4	0,17373389
Monachus schauinslandi NC008421 1	Aonyx cinerea NC035814 2	0,17373398
Prionailurus planiceps NC028312 6	Phoca groenlandica NC008429 54	0,17373426
Pusa hispida NC 008433 1	Leopardus tigrinus NC028317 1	0,17373833
Viverricula indica NC025296 2	Neovison vison NC020641 3	0,1737427
Nandinia binotata NC024567 1	Gulo gulo NC009685 3	0,17374432
Mirounga angustirostris SRR10331586 1	Acinonyx jubatus NC005212 3	0,17375255
Smilodon populator MF871700 1	Phoca groenlandica NC008429 54	0,17376299
Spilogale putorius NC010497 1	Neovison vison NC020641 3	0,17376843
Cystophora cristata NC008427 1	Chrotogale owstoni T607 1	0,17377576
Mungos mungo/gambianus SRR7704821 1	Mirounga leonina NC008422 1	0,17378131
Urva brachyura KY117547 1	Cystophora cristata NC008427 1	0,17378131
Xenogale naso C07XAR110 1	Erignathus barbatus NC008426 1	0,17378131
Nandinia binotata NC024567 1	Martes martes T302 3	0,17378257
Pusa sibirica NC008432 2	Procyon lotor AB462046 3	0,173784
Lutrogale perspicillata NC035811 1	Cystophora cristata NC008427 1	0,1737849
Otaria byronia OTAB 1	Mustela erminea T305 2	0,17378774
Paguma larvata PDDS11 2	Erignathus barbatus NC008426 1	0,17379047
Phoca groenlandica NC008429 54	Mustela altaica NC021751 1	0,17379773
Poecilogale albinucha T602 1	Monachus schauinslandi NC008421 1	0,1738027
Pusa hispida NC 008433 1	Otocolobus manul NC028323 1	0,17380346
Monachus monachus NC044972 5	Conepatus chinga NC042596 1	0,17380719
Mustela nivalis T306 5	Monachus monachus NC044972 5	0,17382288
Panthera leo NERO 19	Lobodon carcinophaga NC008423 1	0,17383493
Xenogale naso C07XAR110 1	Cystophora cristata NC008427 1	0,17384864
Taxidea taxus NC020646 1	Arctocephalus pusillus NC008417 1	0,17385735
Phoca largha NC008430 1	Bassaricyon neblina SRX1097850 1	0,17385989
Pusa sibirica NC008432 2	Hemigalus derbyanus MH464791 1	0,17386249
Pusa caspica NC008431 1	Poecilogale albinucha T602 1	0,17386484
Phoca fasciata NC008428 1	Felis nigripes NC028309 1	0,17386822
Pusa hispida NC 008433 1	Caracal caracal NC028306 1	0,1738717
Mirounga angustirostris SRR10331586 1	Civettictis civetta GLC19 1	0,17387392
Prionailurus viverrinus NC028305 1	Phoca vitulina NC001325 1	0,17387772
Monachus monachus NC044972 5	Leptailurus serval NC028316 1	0,17389062
Spilogale putorius NC010497 1	Meles leucurus NC039173 4	0,17389581
Hydrurga leptonyx NC008425 1	Canis aureus KT448274 1	0,17389688
Puma concolor NC016470 22	Halichoerus grypus NC001602 2	0,17391184
Pusa sibirica NC008432 2	Lutrogale perspicillata NC035811 1	0,17391925
Cynogale bennetti KY117544 1	Cryptoprocta ferox CFC13 1	0,17392257
Lobodon carcinophaga NC008423 1	Urva javanica T413 1	0,17392667
Mungos mungo/gambianus SRR7704821 1	Mirounga angustirostris SRR10331586 1	0,17392826
Pusa caspica NC008431 1	Felis silvestris lybica KP202275 4	0,17392911
Pusa caspica NC008431 1	Prionailurus bengalensis CKM45 20	0,17393333
Profelis aurata NC028299 1	Monachus schauinslandi NC008421 1	0,17393343
Puma concolor NC016470 22	Monachus schauinslandi NC008421 1	0,17393586
Phoca groenlandica NC008429 54	Leopardus tigrinus NC028317 1	0,17393793
Phoca vitulina NC001325 1	Genetta servalina NC024568 2	0,17393798
Puma concolor NC016470 22	Mirounga angustirostris SRR10331586 1	0,17393807
Mirounga angustirostris SRR10331586 1	Caracal caracal NC028306 1	0,17393926
Phoca vitulina NC001325 1	Leopardus geoffroyi NC028320 1	0,17394471
Mustela sibirica NC020637 6	Monachus monachus NC044972 5	0,17394678
Homotherium latidens MF871702 3	Ailurus fulgens NC011124 1	0,17395106
Monachus schauinslandi NC008421 1	Genetta abyssinica MG489822 1	0,17395465
Monachus monachus NC044972 5	Leopardus wiedii NC028318 1	0,17395602
Panthera pardus NC010641 5	Hydrurga leptonyx NC008425 1	0,17397168

Spilogale putorius NC010497 1	Acinonyx jubatus NC005212 3	0,18953818
Neophoca cinerea NC008419 1	Nasua nasua NC020647 1	0,18953902
Odobenus rosmarus NC004029 29	Gulo gulo NC009685 3	0,18954003
Melogale moschata NC020644 1	Urva brachyura KY117547 1	0,1895414
Panthera onca KP202264 2	Ailurus fulgens NC011124 1	0,18954429
Procyon lotor AB462049 4	Canis anthus NC027956 2	0,18954469
Speothos venaticus C48 2	Ommatophoca rossii AY377287etc 1	0,18954492
Otaria byronia OTAB 1	Urva javanica T413 1	0,18954554
Panthera pardus japonensis KJ866876	Callorhinus ursinus NC008415 1	0,18954661
Panthera uncia KP202269 1	Arctocephalus forsteri KT693377 17	0,18954973
Vulpes ferrilata NC027935 1	Panthera onca KP202264 2	0,18954979
Pardofelis marmorata NLN3 2	Arctocephalus australis MG023139 1	0,18955013
Galidictis fasciata DM333 1	Arctotherium sp NC030174 1	0,18955074
Martes zibellina NC011579 39	Chrysocyon brachyurus NC024172 1	0,18955332
Odobenus rosmarus NC004029 29	Meles anakuma NC009677 1	0,18955481
Viverra tangalunga MH464792 1	Crocuta crocuta NC020670 3	0,18955588
Proteles cristata T393 6	Mustela sibirica AP017394 11	0,18955711
Panthera uncia NC010638 1	Melogale moschata NC020644 1	0,18955784
Ursus maritimus NC003428 31	Cryptoprocta ferox CFC13 1	0,18955803
Panthera tigris amoyensis NC014770 2	Mustela nigripes NC024942 1	0,18955848
Conepatus chinga NC042596 1	Canis aureus KT448274 1	0,1895591
Prionailurus planiceps NC028312 6	Otocyon megalotis SAF1 2	0,18955976
Panthera uncia KP202269 1	Martes martes T302 3	0,18955988
Vulpes ferrilata NC027935 1	Helarctos malayanus NC009968 2	0,18956013
Ursus thibetanus formosanus NC0093	Mungos mungo/gambianus SRR77048	0,18956104
Ursus arctos EU497665 29	Panthera uncia KP202269 1	0,18956125
Lutra sumatrana NC035810 1	Canis mesomelas KT448280 1	0,18956131
Ursus spelaeus EU327344 13	Mungos mungo MMC7 1	0,1895624
Speothos venaticus C48 2	Leopardus tigrinus NC028317 1	0,18956243
Speothos venaticus C48 2	Mustela nivalis T306 5	0,18956296
Ursus arctos isabellinus 18B5 2	Otocolobus manul NC028323 1	0,18956332
Ursus spelaeus EU327344 13	Galidia elegans D146 1	0,18956523
Panthera tigris amoyensis NC014770 2	Otaria byronia OTAB 1	0,18956609
Melogale moschata V0735A 1	Lycalopex sechurae KT448284 1	0,18956678
Procyon lotor AB462046 3	Panthera leo spelaea KX258452 2	0,18956849
Mephitis mephitis NC020648 1	Canis latrans NC008093 7	0,18956859
Ictonyx striatus T299 1	Crocuta crocuta NC020670 3	0,18956934
Ursus spelaeus EU327344 13	Panthera pardus japonensis KJ866876	0,18957102
Ursus spelaeus EU327344 13	Paradoxurus hermaphroditus NC03959	0,18957257
Gulo gulo NC009685 3	Atilax paludinosus T606 1	0,18957706
Lycalopex sechurae KT448284 1	Eumetopias jubatus NC004030 10	0,18957868
Neofelis nebulosa NC008450 3	Meles leucurus NC039173 4	0,18958127
Panthera onca KP202264 2	Canis mesomelas KT448280 1	0,18958215
Panthera leo NERO 19	Meles anakuma NC009677 1	0,18958247
Xenogale naso C07XAR110 1	Arctocephalus forsteri NC004023 28	0,18958497
Ursus arctos GU573491 207	Canis lupus familiaris NC002008 1231	0,18958931
Spilogale putorius NC010497 1	Prionailurus planiceps KY682741 4	0,18958984
Pardofelis marmorata NLN3 2	Cuon alpinus NC013445 3	0,18959381
Ursus arctos GU573486 5	Canis lupus familiaris NC002008 1231	0,1895957
Panthera leo NERO 19	Martes foina NC020643 1	0,18959606
Lycalopex sechurae KT448284 1	Ailuropoda melanoleuca NC009492 5	0,18959735
Ursus spelaeus NC011112 8	Diplogale hosei MH464790 1	0,18960035
Vulpes lagopus NC026529 3	Lutra lutra LC050126 1	0,18960114
Vulpes corsac NC023958 1	Eupleres goudotii D128 1	0,18960179
Vulpes vulpes NC008434 5	Eupleres goudotii D128 1	0,18960203
Prionodon linsang ERR2391707 1	Martes americana NC020642 1	0,18960425
Tremarctos ornatus NC009969 2	Atilax paludinosus T606 1	0,18960433
Tremarctos ornatus NC009969 2	Urva brachyura KY117547 1	0,18960503
Neophoca cinerea NC008419 1	Urva javanica T413 1	0,18960646
Ursus arctos GU573491 207	Conepatus chinga NC042596 1	0,18960716
Vulpes zerda KJ603240 1	Mustela eversmannii NC028013 1	0,18960851
Proteles cristata T393 6	Martes flavigula NC012141 3	0,1896086
Suricata suricatta SSM10 1	Melogale moschata NC020644 1	0,18960878
Martes pennanti NC020664 16	Urva brachyura KY117547 1	0,1896092
Otaria byronia OTAB 1	Helogale parvula SRR7637809 1	0,18960924
Nasua nasua NC020647 1	Ailurus fulgens NC011124 1	0,18961018
Otaria byronia OTAB 1	Nasua nasua NC020647 1	0,18961181
Otocyon megalotis SAF1 2	Lutra lutra NC011358 9	0,18961352
Martes pennanti NC020664 16	Galidia elegans D146 1	0,18961388
Vulpes ferrilata NC027935 1	Panthera uncia KP202269 1	0,18961731
Pardofelis marmorata NLN3 2	Arctocephalus forsteri KT693377 17	0,18961737
Lynx canadensis NC028313 1	Tapirus terrestris T358	0,18961774
Ichneumia albicauda T603 1	Aonyx cinerea NC035814 2	0,18961886
Canis anthus NC027956 2	Arctotherium sp NC030174 1	0,18961915
Urocyon cinereoargenteus NC026723 2	Prionailurus viverrinus NC028305 1	0,18962108
Ursus arctos EU497665 29	Canis latrans NC008093 7	0,18962146
Lutrogale perspicillata NC035811 1	Catopuma temminckii NC027115 41	0,18962197
Procyon lotor AB462046 3	Parahyaena brunnea NC038159 15	0,18962233
Panthera tigris amoyensis NC014770 2	Arctocephalus australis MG023139 1	0,18962619
Panthera tigris amoyensis NC014770 2	Arctocephalus forsteri KT693377 17	0,18962635
Panthera leo spelaea KX258452 2	Meles leucurus NC039173 4	0,1896271
Panthera uncia NC010638 1	Otocyon megalotis SAF1 2	0,18962757
Xenogale naso C07XAR110 1	Galictis vittata T412 1	0,18962786
Ursus arctos GU573491 207	Lynx canadensis NC028313 1	0,18962839
Ursus maritimus GU573488 Svalbard	Felis silvestris lybica KP202275 4	0,18962982
Ursus arctos EU497665 29	Prionailurus planiceps NC028312 6	0,18963015
Ursus arctos GU573491 207	Felis chaus NC028307 1	0,18963028
Zalophus wollebaeki SRR4431565 1	Otocyon megalotis SAF1 2	0,18963115
Cynogale bennetti KY117544 1	Canis lupus familiaris NC002008 1231	0,18963206
Ictonyx striatus T299 1	Ichneumia albicauda T603 1	0,18963239
Procyon lotor AB462049 4	Canis mesomelas KT448280 1	0,18963294
Spilogale putorius NC010497 1	Cuon alpinus NC013445 3	0,18963322
Ursus arctos GU573486 5	Panthera pardus japonensis KJ866876	0,1896347
Prionailurus planiceps KY682741 4	Ailuropoda melanoleuca NC009492 5	0,18963628
Otocyon megalotis SAF1 2	Helarctos malayanus NC009968 2	0,1896366
Cynogale bennetti KY117544 1	Canis aureus KT448274 1	0,18963692
Profelis aurata NC028299 1	Mephitis mephitis NC020648 1	0,1896386
Panthera uncia KP202269 1	Helarctos malayanus NC009968 2	0,18964061
Zalophus californianus NC008416 1	Paradoxurus hermaphroditus NC03959	0,18964383
Martes americana NC020642 1	Cuon alpinus NC013445 3	0,18964539
Neofelis nebulosa NC008450 3	Melogale moschata KP726273 1	0,18965162
Potos flavus T414 1	Cuon alpinus NC013445 3	0,18965413
Panthera leo NERO 19	Neophoca cinerea NC008419 1	0,18965529

Pusa caspica NC008431 1	Eupleres goudotii D128 1	0,17398354
Mustela altaica NC021751 1	Bassaricyon neblina SRX1097850 1	0,17398973
Poecilogale albinucha T602 1	Mirounga leonina NC008422 1	0,17399423
Genetta genetta T297 1	Erignathus barbatus NC008426 1	0,17399499
Pusa sibirica NC008432 2	Felis margarita NC028308 1	0,17399667
Procyon lotor AB462049 4	Ictonyx striatus T299 1	0,17399675
Pusa hispida NC 008433 1	Felis margarita NC028308 1	0,17400597
Phoca vitulina NC001325 1	Felis silvestris lybica KP202275 4	0,17400947
Phoca vitulina NC001325 1	Leopardus guigna NC028321 1	0,1740122
Lynx pardinus NC028319 161	Canis adustus KT448271 1	0,17401379
Phoca groenlandica NC008429 54	Canis adustus KT448271 1	0,17402118
Smilodon populator MF871700 1	Pusa hispida NC 008433 1	0,17403981
Lutrogale perspicillata NC035811 1	Halichoerus grypus NC001602 2	0,17404205
Lutra sumatrana NC035810 1	Ailurus fulgens NC011124 1	0,17404589
Mirounga leonina NC008422 1	Urva brachyura KY117547 1	0,17405063
Pusa sibirica NC008432 2	Crossarchus platycephalus C7R66 1	0,17405063
Mirounga leonina NC008422 1	Felis nigripes NC028309 1	0,17405893
Erignathus barbatus NC008426 1	Catopuma temminckii NC027115 41	0,17406113
Monachus schauinslandi NC008421 1	Melogale moschata V0735A 1	0,17406133
Pusa caspica NC008431 1	Lynx canadensis NC028313 1	0,17406301
Pusa sibirica NC008432 2	Prionailurus viverrinus NC028305 1	0,17406674
Salanoia concolor D378 1	Phoca vitulina NC001325 1	0,17406772
Proteles cristata T393 6	Leptonychotes weddellii NC008424 1	0,17406872
Phoca groenlandica NC008429 54	Lynx pardinus NC028319 161	0,17407008
Mirounga angustirostris SRR10331586 1	Lynx canadensis NC028313 1	0,17407027
Panthera uncia KP202269 1	Hydrurga leptonyx NC008425 1	0,1740725
Phoca fasciata NC008428 1	Urva javanica/auropunctata NC006835 1	0,17409316
Halichoerus grypus NC001602 2	Genetta abyssinica MG489822 1	0,17412646
Ommatophoca rossii AY377287etc 1	Leopardus wiedii NC028318 1	0,1741267
Viverricula indica NC025296 2	Taxidea taxus NC020646 1	0,17412792
Mustela erminea T305 2	Leopardus pardalis T262 1	0,17413032
Mungos mungo MMC7 1	Mirounga angustirostris SRR10331586 1	0,17413186
Paradoxurus jerdoni MH464793 1	Lobodon carcinophaga NC008423 1	0,17413364
Pusa sibirica NC008432 2	Leopardus tigrinus NC028317 1	0,17413373
Phoca vitulina NC001325 1	Lutrogale perspicillata NC035811 1	0,17413497
Lobodon carcinophaga NC008423 1	Galidictis fasciata DM333 1	0,17413525
Lycalopex sechurae KT448284 1	Lobodon carcinophaga NC008423 1	0,17413639
Profelis aurata NC028299 1	Phoca fasciata NC008428 1	0,17413883
Prionailurus viverrinus NC028305 1	Phoca groenlandica NC008429 54	0,17414002
Monachus monachus NC044972 5	Martes zibellina NC011579 39	0,17415184
Monachus monachus NC044972 5	Leopardus tigrinus NC028317 1	0,17416123
Monachus monachus NC044972 5	Martes foina NC020643 1	0,17416374
Procyon lotor AB462046 3	Lontra canadensis SRR10409165 1	0,17418011
Ommatophoca rossii AY377287etc 1	Mustela sibirica AP017394 11	0,17418046
Mirounga leonina NC008422 1	Lontra canadensis SRR10409165 1	0,1741816
Leopardus jacobita NC028322 1	Halichoerus grypus NC001602 2	0,17418288
Mustela eversmannii NC028013 1	Mirounga angustirostris SRR10331586 1	0,17419502
Ommatophoca rossii AY377287etc 1	Hemigalus derbyanus MH464791 1	0,1741958
Pusa sibirica NC008432 2	Prionailurus planiceps NC028312 6	0,17419875
Pusa caspica NC008431 1	Leopardus wiedii NC028318 1	0,174201
Zalophus wollebaeki SRR4431565 1	Mustela erminea T305 2	0,17420128
Monachus schauinslandi NC008421 1	Civettictis civetta GLC19 1	0,17420642
Phoca largha NC008430 1	Genetta servalina NC024568 2	0,17420708
Pusa caspica NC008431 1	Genetta abyssinica MG489822 1	0,1742077
Phoca vitulina NC001325 1	Lynx pardinus NC028319 161	0,17421141
Prionailurus planiceps KY682741 4	Phoca largha NC008430 1	0,17421199
Prionailurus rubiginosus NC028304 2	Phoca vitulina NC001325 1	0,17421214
Spilogale putorius NC010497 1	Erignathus barbatus NC008426 1	0,1742194
Ommatophoca rossii AY377287etc 1	Leptailurus serval NC028316 1	0,17422202
Pusa sibirica NC008432 2	Civettictis civetta NC033378 1	0,17423095
Lynx pardinus NC028319 161	Halichoerus grypus NC001602 2	0,17424887
Poecilogale albinucha T602 1	Halichoerus grypus NC001602 2	0,17425032
Martes americana NC020642 1	Ailurus fulgens NC011124 1	0,17425115
Pusa sibirica NC008432 2	Ailurus fulgens NC011124 1	0,17425244
Paguma larvata PDDS11 2	Mustela erminea T305 2	0,17426471
Viverra zibetha T609 1	Mustela erminea T305 2	0,17426509
Leptonychotes weddellii NC008424 1	Canis mesomelas KT448280 1	0,17426725
Pusa sibirica NC008432 2	Poecilogale albinucha T602 1	0,17426863
Erignathus barbatus NC008426 1	Ailuropoda melanoleuca NC009492 5	0,1742724
Prionailurus rubiginosus NC028304 2	Ommatophoca rossii AY377287etc 1	0,17427242
Leptonychotes weddellii NC008424 1	Crocuta crocuta NC020670 3	0,17427243
Hydrurga leptonyx NC008425 1	Canis mesomelas KT448280 1	0,17427664
Mirounga angustirostris SRR10331586 1	Leopardus guigna NC028321 1	0,17427793
Mirounga angustirostris SRR10331586 1	Leopardus geoffroyi NC028320 1	0,17427805
Poecilogale albinucha T602 1	Phoca largha NC008430 1	0,17428133
Ommatophoca rossii AY377287etc 1	Leopardus colocolo NC028314 1	0,17428671
Phoca largha NC008430 1	Genetta abyssinica MG489822 1	0,17429198
Monachus schauinslandi NC008421 1	Civettictis civetta NC033378 1	0,17431137
Mirounga leonina NC008422 1	Melogale moschata V0735A 1	0,17431587
Leptonychotes weddellii NC008424 1	Canis anthus NC027956 2	0,17432098
Galidictis fasciata DM333 1	Cystophora cristata NC008427 1	0,17432357
Lobodon carcinophaga NC008423 1	Bdeogale nigripes GLC15 1	0,17433117
Phoca fasciata NC008428 1	Paguma larvata PDDS11 2	0,17433169
Poecilogale albinucha T602 1	Erignathus barbatus NC008426 1	0,17433546
Leptonychotes weddellii NC008424 1	Chrysocyon brachyurus NC024172 1	0,17433828
Phoca vitulina NC001325 1	Felis nigripes NC028309 1	0,17434603
Phoca largha NC008430 1	Lynx pardinus NC028319 161	0,17434625
Prionailurus planiceps NC028312 6	Phoca largha NC008430 1	0,1743467
Spilogale putorius NC010497 1	Arctonyx collaris NC020645 1	0,17436141
Ommatophoca rossii AY377287etc 1	Mustela itatsi NC034330 19	0,17436227
Gulo gulo NC009685 3	Bassaricyon neblina SRX1097850 1	0,17436332
Viverricula indica KX891745 1	Halichoerus grypus NC001602 2	0,17437643
Leopardus wiedii NC028318 1	Canis adustus KT448271 1	0,17437692
Hemigalus derbyanus MH464791 1	Ailurus fulgens NC011124 1	0,17437799
Mustela putorius NC020638 4	Bassaricyon neblina SRX1097850 1	0,17438418
Nandinia binotata NC024567 1	Melogale moschata NC020644 1	0,17438872
Leptonychotes weddellii NC008424 1	Galidia elegans D146 1	0,17439045
Viverra zibetha T609 1	Mustela frenata NC020640 1	0,17439462
Genetta servalina NC024568 2	Cystophora cristata NC008427 1	0,17439692
Lobodon carcinophaga NC008423 1	Cynictis penicillata T375 1	0,17439798
Panthera uncia KP202269 1	Cystophora cristata NC008427 1	0,17439869
Mustela frenata NC020640 1	Arctodus simus NC011116 1	0,17440112
Phoca fasciata NC008428 1	Caracal caracal NC028306 1	0,17440826

Vulpes lagopus NC026529 3	Panthera pardus NC010641 5	0,18965843
Felis chaus NC028307 1	Arctocephalus forsteri NC004023 28	0,18966149
Ursus thibetanus laniger MH281753 2	Diplogale hosei MH464790 1	0,18966173
Ursus arctos EU497665 29	Panthera leo NERO 19	0,18966334
Panthera uncia KP202269 1	Martes flavigula NC012141 3	0,18966369
Suricata suricatta SSM10 1	Martes americana NC020642 1	0,18966894
Mephitis mephitis NC020648 1	Canis lupus familiaris NC002008 1231	0,18966913
Atilax paludinosus T606 1	Arctocephalus townsendi NC008420 1	0,18967062
Vulpes corsac NC023958 1	Bdeogale nigripes GLC15 1	0,1896714
Vulpes corsac NC023958 1	Atilax paludinosus T606 1	0,18967162
Vulpes zerda KJ603240 1	Lutra lutra LC050126 1	0,18967232
Spilogale putorius NC010497 1	Melursus ursinus NC009970 2	0,18967293
Vulpes vulpes NC008434 5	Arctocephalus gazella BK010918 1	0,18967342
Neophoca cinerea NC008419 1	Urva brachyura KY117547 1	0,18967398
Panthera leo spelaea KX258452 2	Gulo gulo NC009685 3	0,18967421
Ursus maritimus GU573488 Svalbard	Canis mesomelas KT448280 1	0,18967451
Martes pennanti NC020664 16	Helogale parvula SRR7637809 1	0,18967654
Panthera uncia KP202269 1	Meles leucurus NC039173 4	0,18967853
Otaria byronia OTAB 1	Nyctereutes procyonoides NC013700 3	0,18968
Panthera pardus japonensis KJ866876	Lutra lutra NC011358 9	0,18968037
Panthera uncia KP202269 1	Arctonyx collaris NC020645 1	0,18968078
Panthera uncia NC010638 1	Mustela sibirica AP017394 11	0,18968333
Ursus thibetanus mupinensis NC00875	Lutrogale perspicillata NC035811 1	0,18968354
Potos flavus T414 1	Lycaon pictus NC028427 2	0,18968396
Panthera uncia KP202269 1	Arctocephalus australis MG023139 1	0,18968442
Vulpes ferrilata NC027935 1	Panthera pardus japonensis KJ866876	0,1896845
Otocyon megalotis SAF1 2	Arctocephalus gazella BK010918 1	0,18968499
Speothos venaticus C48 2	Cynictis penicillata T375 1	0,18968518
Speothos venaticus C48 2	Meles meles T303 3	0,18968584
Paradoxurus jerdoni MH464793 1	Otocyon megalotis SAF1 2	0,18968621
Ursus maritimus GU573488 Svalbard	Lontra canadensis SRR10409165 1	0,18968737
Potos flavus T414 1	Chrysocyon brachyurus NC024172 1	0,18968886
Zalophus wollebaeki SRR4431565 1	Vulpes zerda KJ603240 1	0,18968907
Panthera pardus japonensis KJ866876	Arctocephalus pusillus NC008417 1	0,18968949
Diplogale hosei MH464790 1	Arctocephalus australis MG023139 1	0,1896895
Poecilogale albinucha T602 1	Panthera onca KP202264 2	0,18969047
Panthera uncia NC010638 1	Neovison vison NC020641 3	0,18969084
Panthera tigris amoyensis NC014770 2	Mustela putorius NC020638 4	0,18969263
Neovison vison NC020641 3	Cryptoprocta ferox CFC13 1	0,18969283
Ursus arctos GU573486 5	Lontra canadensis SRR10409165 1	0,18969309
Mephitis mephitis NC020648 1	Galerella sanguinea T378 1	0,18969315
Ursus thibetanus formosanus NC00993	Xenogale naso C07XAR110 1	0,18969485
Vulpes corsac NC023958 1	Panthera onca NC022842 1	0,189695
Vulpes ferrilata NC027935 1	Arctictis binturong T605 2	0,18969527
Ursus maritimus GU573488 Svalbard	Prionailurus planiceps NC028312 6	0,18969736
Ursus spelaeus EU327344 13	Canis anthus NC027956 2	0,18970257
Lontra canadensis SRR10409165 1	Acinonyx jubatus NC005212 3	0,18970397
Ursus arctos GU573486 5	Otocolobus manul NC028323 1	0,18970487
Galidia elegans D146 1	Canis lupus familiaris NC002008 1231	0,1897105
Paradoxurus hermaphroditus NLNC 1	Arctocephalus forsteri NC004023 28	0,18971053
Helogale parvula SRR7637809 1	Gulo gulo NC009685 3	0,18971112
Canis lupus familiaris NC002008 1231	Aonyx cinerea NC035814 2	0,18971145
Mephitis mephitis NC020648 1	Leptailurus serval NC028316 1	0,18971185
Ursus arctos AP012576 6	Felis margarita NC028308 1	0,18971319
Canis lupus chanco NC010340 4	Aonyx cinerea NC035814 2	0,18971536
Panthera leo spelaea KX258452 2	Lycalopex sechurae KT448284 1	0,18971545
Panthera leo NERO 19	Martes americana NC020642 1	0,18971665
Vulpes vulpes NC008434 5	Urva javanica/auropunctata NC006835	0,18971665
Panthera leo NERO 19	Nyctereutes procyonoides NC013700 3	0,1897186
Panthera pardus NC010641 5	Callorhinus ursinus NC008415 1	0,18972063
Neofelis nebulosa NC008450 3	Mustela itatsi NC034330 19	0,18972149
Ursus thibetanus laniger MH281753 2	Chrysocyon brachyurus NC024172 1	0,18972254
Neofelis nebulosa NC008450 3	Aonyx cinerea NC035814 2	0,18972275
Canis mesomelas KT448280 1	Arctotherium sp NC030174 1	0,18972647
Panthera pardus NC010641 5	Martes melampus NC009678 1	0,18972664
Panthera pardus NC010641 5	Nyctereutes procyonoides NC013700 3	0,18972859
Salanoia concolor D378 1	Lycalopex sechurae KT448284 1	0,18973139
Lutra lutra NC011358 9	Canis anthus NC027956 2	0,18973354
Helogale parvula SRR7637809 1	Conepatus chinga NC042596 1	0,18973437
Vulpes vulpes NC008434 5	Lutra lutra NC011358 9	0,18973497
Prionodon linsang ERR2391707 1	Martes melampus NC009678 1	0,18973555
Panthera pardus NC010641 5	Martes martes T302 3	0,18973565
Eumetopias jubatus NC004030 10	Chrysocyon brachyurus NC024172 1	0,18973713
Potos flavus T414 1	Canis anthus NC027956 2	0,18973908
Tremarctos ornatus NC009969 2	Suricata suricatta SSM10 1	0,18973926
Urocyon cinereoargenteus NC026723 2	Crossarchus platycephalus C7R66 1	0,1897397
Lutra sumatrana NC035810 1	Canis anthus NC027956 2	0,18974055
Cynogale bennetti KY117544 1	Arctodus simus NC011116 1	0,1897409
Nasua nasua NC020647 1	Arctocephalus gazella BK010918 1	0,18974102
Paradoxurus hermaphroditus NC03959	Arctocephalus forsteri KT693377 17	0,18974249
Cuon alpinus NC013445 3	Ailuropoda melanoleuca NC009492 5	0,18974252
Vulpes zerda KJ603240 1	Mustela itatsi NC034330 19	0,18974416
Urocyon cinereoargenteus NC026723 2	Mustela putorius NC020638 4	0,18974513
Otaria byronia OTAB 1	Urva brachyura KY117547 1	0,18974757
Vulpes lagopus NC026529 3	Melogale moschata NC020644 1	0,18974826
Lycaon pictus NC028427 2	Arctocephalus forsteri KT693377 17	0,18974982
Ursus maritimus NC003428 31	Lutrogale perspicillata NC035811 1	0,18975051
Panthera uncia NC010638 1	Mustela sibirica NC020637 6	0,1897507
Otocyon megalotis SAF1 2	Mustela eversmannii NC028013 1	0,18975134
Mellivora capensis T370 1	Hydrurga leptonyx NC008425 1	0,18975145
Panthera uncia NC010638 1	Melogale moschata KP726273 1	0,18975158
Urocyon cinereoargenteus NC026723 2	Felis margarita NC028308 1	0,18975489
Proteles cristata T393 6	Mustela putorius NC020638 4	0,18975949
Tremarctos ornatus NC009969 2	Chrysocyon brachyurus NC024172 1	0,18975979
Ursus arctos EU497665 29	Cryptoprocta ferox CFC13 1	0,18975987
Vulpes corsac NC023958 1	Ursus thibetanus thibetanus NC01111{	0,18976201
Speothos venaticus C48 2	Genetta genetta T297 1	0,18976416
Mephitis mephitis NC020648 1	Arctocephalus gazella BK010918 1	0,18976596
Panthera tigris NC010642 35	Aonyx cinerea NC035814 2	0,18976659
Panthera onca KP202264 2	Chrysocyon brachyurus NC024172 1	0,18977021
Ursus arctos GU573486 5	Felis catus NC001700 2	0,18977036
Canis anthus NC027956 2	Ailuropoda melanoleuca NC009492 5	0,18977082
Ursus arctos AP012576 6	Hyaena hyaena NC020669 1	0,18977311

Panthera pardus japonensis KJ866876 8	Hydrurga leptonyx NC008425 1	0,17440951
Phoca vitulina NC001325 1	Felis margarita NC028308 1	0,17441364
Nandinia binotata NC024567 1	Martes flavigula NC012141 3	0,17442255
Martes martes T302 3	Homotherium latidens MF871702 3	0,17443363
Smilodon populator MF871700 1	Phoca vitulina NC001325 1	0,17443638
Mustela frenata NC020640 1	Conepatus chinga NC042596 1	0,17443801
Spilogale putorius NC010497 1	Monachus schauinslandi NC008421 1	0,17444122
Lutra lutra NC011358 9	Bassaricyon neblina SRX1097850 1	0,17444962
Pusa caspica NC008431 1	Lontra canadensis SRR10409165 1	0,17445123
Prionailurus viverrinus NC028305 1	Halichoerus grypus NC001602 2	0,17445345
Mustela erminea T305 2	Crossarchus platycephalus C7R66 1	0,17445371
Melogale moschata KP726273 1	Ailurus fulgens styani NC009691 1	0,17445415
Pusa caspica NC008431 1	Lutrogale perspicillata NC035811 1	0,17445806
Phoca fasciata NC008428 1	Conepatus chinga NC042596 1	0,17446268
Pusa hispida NC 008433 1	Eupleres goudotii D128 1	0,1744663
Lobodon carcinophaga NC008423 1	Xenogale naso C07XAR110 1	0,17446636
Mustela erminea T305 2	Leopardus pardalis NC028315 1	0,17446692
Phoca vitulina NC001325 1	Crossarchus platycephalus C7R66 1	0,17446935
Smilodon populator MF871700 1	Pusa caspica NC008431 1	0,17447111
Panthera tigris amoyensis NC014770 2	Leptonychotes weddellii NC008424 1	0,17447296
Hydrurga leptonyx NC008425 1	Hyaena hyaena NC020669 1	0,17448264
Panthera pardus NC010641 5	Leptonychotes weddellii NC008424 1	0,17449744
Monachus monachus NC044972 5	Ictonyx striatus T299 1	0,17450094
Hemigalus derbyanus MH464791 1	Halichoerus grypus NC001602 2	0,17450413
Mirounga angustirostris SRR10331586 1	Civettictis civetta NC033378 1	0,17451723
Pusa sibirica NC008432 2	Lontra canadensis SRR10409165 1	0,17451867
Nandinia binotata NC024567 1	Martes zibellina NC011579 39	0,17451905
Mirounga leonina NC008422 1	Urva semitorquata MH464789 1	0,17452195
Phoca fasciata NC008428 1	Lontra canadensis SRR10409165 1	0,17452648
Taxidea taxus NC020646 1	Arctocephalus gazella BK010918 1	0,17452724
Mustela erminea T305 2	Lynx pardinus NC028319 161	0,17453346
Pusa caspica NC008431 1	Puma yagouaroundi NC028311 1	0,17453627
Prionailurus viverrinus NC028305 1	Monachus schauinslandi NC008421 1	0,17454441
Lobodon carcinophaga NC008423 1	Cryptoprocta ferox CFC13 1	0,17454839
Prionailurus planiceps KY682741 4	Phoca vitulina NC001325 1	0,17454858
Monachus monachus NC044972 5	Crossarchus platycephalus C7R66 1	0,17455134
Phoca largha NC008430 1	Leopardus tigrinus NC028317 1	0,17455138
Mustela putorius NC020638 4	Monachus monachus NC044972 5	0,17455292
Felis margarita NC028308 1	Canis adustus KT448271 1	0,17455618
Monachus monachus NC044972 5	Genetta servalina NC024568 2	0,17456399
Halichoerus grypus NC001602 2	Ailurus fulgens NC011124 1	0,17456445
Monachus monachus NC044972 5	Lynx lynx NC027083 4	0,17456958
Lutra lutra NC011358 9	Ailurus fulgens styani NC009691 1	0,17457521
Salanoia concolor D378 1	Halichoerus grypus NC001602 2	0,17457928
Meles meles T303 3	Bassaricyon neblina SRX1097850 1	0,17458285
Pusa caspica NC008431 1	Crossarchus platycephalus C7R66 1	0,17458928
Neovison vison NC020641 3	Conepatus chinga NC042596 1	0,17458954
Taxidea taxus NC020646 1	Nasua nasua NC020647 1	0,17459631
Pusa hispida NC 008433 1	Crossarchus platycephalus C7R66 1	0,17459764
Paradoxurus jerdoni MH464793 1	Leptonychotes weddellii NC008424 1	0,17459825
Procyon lotor AB462046 3	Ictonyx striatus T299 1	0,17460252
Mirounga leonina NC008422 1	Lycaon pictus NC028427 2	0,17460352
Pusa caspica NC008431 1	Leopardus geoffroyi NC028320 1	0,17460462
Monachus monachus NC044972 5	Melogale moschata V0735A 1	0,17462261
Potos flavus T414 1	Conepatus chinga NC042596 1	0,17465393
Martes zibellina NC011579 39	Ailurus fulgens styani NC009691 1	0,17465504
Cystophora cristata NC008427 1	Bdeogale nigripes GLC15 1	0,17465661
Erignathus barbatus NC008426 1	Atilax paludinosus T606 1	0,17465661
Hemigalus derbyanus MH464791 1	Ailurus fulgens styani NC009691 1	0,1746622
Bassariscus sumichrasti SRX1099089 1	Arctocephalus pusillus NC008417 1	0,17466539
Potos flavus T414 1	Arctodus simus NC011116 1	0,17466584
Pusa caspica NC008431 1	Lynx pardinus NC028319 161	0,17466937
Pusa caspica NC008431 1	Prionailurus planiceps KY682741 4	0,17467016
Ictonyx striatus T299 1	Ailurus fulgens styani NC009691 1	0,17467447
Phoca fasciata NC008428 1	Catopuma temminckii NC027115 41	0,17467649
Phoca groenlandica NC008429 54	Pardofelis marmorata NLN3 2	0,17467664
Prionailurus planiceps NC028312 6	Phoca vitulina NC001325 1	0,17468329
Phoca largha NC008430 1	Leopardus guigna NC028321 1	0,1746856
Monachus schauinslandi NC008421 1	Mephitis mephitis NC020648 1	0,17468816
Monachus monachus NC044972 5	Genetta genetta T297 1	0,17470057
Halichoerus grypus NC001602 2	Ailuropoda melanoleuca NC009492 5	0,17472509
Phoca groenlandica NC008429 54	Conepatus chinga NC042596 1	0,17472512
Nyctereutes procyonoides NC013700 3	Leptonychotes weddellii NC008424 1	0,17472619
Melogale moschata NC020644 1	Bassaricyon neblina SRX1097850 1	0,17472871
Phoca largha NC008430 1	Lontra canadensis SRR10409165 1	0,17473336
Genetta servalina NC024568 2	Arctonyx collaris NC020645 1	0,17473349
Viverricula indica KX891745 1	Mustela kathiah NC023210 1	0,17473413
Pusa sibirica NC008432 2	Cryptoprocta ferox CFC13 1	0,1747351
Pusa caspica NC008431 1	Felis margarita NC028308 1	0,17473736
Phoca groenlandica NC008429 54	Felis margarita NC028308 1	0,1747442
Taxidea taxus NC020646 1	Homotherium latidens MF871702 3	0,17474599
Proteles cristata T393 6	Hydrurga leptonyx NC008425 1	0,17475632
Canis adustus KT448271 1	Acinonyx jubatus NC005212 3	0,17476685
Halichoerus grypus NC001602 2	Genetta servalina NC024568 2	0,17478225
Pusa sibirica NC008432 2	Cynictis penicillata T375 1	0,17479128
Urva semitorquata MH464789 1	Erignathus barbatus NC008426 1	0,17479128
Mungos mungo MMC7 1	Cystophora cristata NC008427 1	0,17479293
Pusa sibirica NC008432 2	Mungotictis decemlineata NC027828 1	0,17479384
Melogale moschata NC020644 1	Ailurus fulgens NC011124 1	0,17479469
Pusa hispida NC 008433 1	Urva semitorquata MH464789 1	0,17480035
Nasua nasua NC020647 1	Martes foina NC020643 1	0,17480165
Prionailurus rubiginosus NC028304 2	Canis adustus KT448271 1	0,17480174
Panthera onca KP202264 2	Cystophora cristata NC008427 1	0,17480263
Ichneumia albicauda T603 1	Cystophora cristata NC008427 1	0,17480446
Hydrurga leptonyx NC008425 1	Canis anthus NC027956 2	0,17480461
Pusa sibirica NC008432 2	Felis nigripes NC028309 1	0,17480461
Phoca largha NC008430 1	Crossarchus platycephalus C7R66 1	0,17480583
Pusa sibirica NC008432 2	Leopardus guigna NC028321 1	0,1748068
Pusa caspica NC008431 1	Leopardus jacobita NC028322 1	0,17480719
Prionodon pardicolor NC024569 2	Leptonychotes weddellii NC008424 1	0,17480832
Viverra zibetha T609 1	Pusa hispida NC 008433 1	0,17480859
Viverra zibetha T609 1	Monachus schauinslandi NC008421 1	0,17481041
Monachus schauinslandi NC008421 1	Felis margarita NC028308 1	0,17481171

Otocolobus manul NC028323 1	Mephitis mephitis NC020648 1	0,1897734
Hyaena hyaena NC020669 1	Canis lupus chanco NC010340 4	0,18977779
Ursus arctos AP012576 6	Lynx canadensis NC028313 1	0,18977977
Ursus arctos AP012576 6	Leopardus wiedii NC028318 1	0,18978082
Paguma larvata PDDS11 2	Arctocephalus forsteri NC004023 28	0,1897857
Eumetopias jubatus NC004030 10	Canis latrans NC008093 7	0,18978907
Spilogale putorius NC010497 1	Prionailurus viverrinus NC028305 1	0,18979082
Poecilogale albinucha T602 1	Neofelis nebulosa NC008450 3	0,18979502
Ursus americanus JX196366 3	Leopardus tigrinus NC028317 1	0,18979605
Mungos mungo/gambianus SRR77048	Lycalopex sechurae KT448284 1	0,18979643
Poecilogale albinucha T602 1	Panthera pardus NC010641 5	0,18979864
Lycalopex sechurae KT448284 1	Urva brachyura KY117547 1	0,18979882
Mephitis mephitis NC020648 1	Canis lupus chanco NC010340 4	0,18980426
Ursus arctos AP012576 6	Lycalopex sechurae KT448284 1	0,18980514
Panthera pardus NC010641 5	Lycalopex sechurae KT448284 1	0,18980557
Panthera leo NERO 19	Canis mesomelas KT448280 1	0,18980813
Urocyon cinereoargenteus NC026723 2	Meles leucurus NC039173 4	0,18980868
Mungos mungo MMC7 1	Bassaricyon neblina SRX1097850 1	0,18980956
Vulpes vulpes NC008434 5	Arctocephalus pusillus NC008417 1	0,18981147
Procyon lotor AB462046 3	Urva brachyura KY117547 1	0,18981168
Urocyon littoralis catalinae KP129018	Mustela putorius NC020638 4	0,1898125
Eumetopias jubatus NC004030 10	Canis lupus chanco NC010340 4	0,18981363
Prionodon linsang ERR2391707 1	Martes martes T302 3	0,18981652
Ursus maritimus GU573488 Svalbard	Lutrogale perspicillata NC035811 1	0,18981779
Leopardus jacobita NC028322 1	Arctocephalus townsendi NC008420 1	0,18982072
Urocyon cinereoargenteus NC026723 2	Felis catus NC001700 2	0,18982169
Ursus arctos isabellinus 18B5 2	Bassaricyon neblina SRX1097850 1	0,18982245
Odobenus rosmarus NC004029 29	Meles leucurus NC039173 4	0,18982358
Prionodon pardicolor NC024569 2	Ailurus fulgens styani NC009691 1	0,18982504
Vulpes corsac NC023958 1	Ailuropoda melanoleuca NC009492 5	0,18982548
Panthera uncia KP202269 1	Otaria byronia OTAB 1	0,18982673
Parahyaena brunnea NC038159 15	Arctotherium sp NC030174 1	0,18982875
Nasua nasua NC020647 1	Hemigalus derbyanus MH464791 1	0,18982911
Poecilogale albinucha T602 1	Ichneumia albicauda T603 1	0,18982911
Panthera leo spelaea KX258452 2	Mustela sibirica AP017394 11	0,18982936
Ursus maritimus NC003428 31	Parahyaena brunnea NC038159 15	0,18982963
Panthera leo spelaea KX258452 2	Mustela sibirica NC020637 6	0,18982983
Phocarctos hookeri NC008418 1	Ailuropoda melanoleuca NC009492 5	0,18983136
Panthera tigris amoyensis NC014770 2	Neophoca cinerea NC008419 1	0,1898315
Helarctos malayanus NC009968 2	Galidia elegans D146 1	0,18983163
Ursus arctos GU573491 207	Leopardus guigna NC028321 1	0,18983597
Ursus spelaeus NC011112 8	Canis anthus NC027956 2	0,18983686
Poecilogale albinucha T602 1	Panthera onca NC022842 1	0,18983707
Martes foina NC020643 1	Crocuta crocuta NC020670 3	0,1898372
Ursus thibetanus formosanus NC0093	Panthera uncia KP202269 1	0,18984108
Panthera leo spelaea KX258452 2	Lycaon pictus NC028427 2	0,18984863
Vulpes vulpes NC008434 5	Ursus americanus JX196366 3	0,1898528
Spilogale putorius NC010497 1	Felis nigripes NC028309 1	0,18985841
Odobenus rosmarus NC004029 29	Helarctos malayanus NC009968 2	0,18986268
Neofelis nebulosa NC008450 3	Martes foina NC020643 1	0,18986621
Spilogale putorius NC010497 1	Leptailurus serval NC028316 1	0,18986762
Vulpes corsac NC023958 1	Lutra lutra NC011358 9	0,18986931
Procyon lotor AB462049 4	Cynogale bennetti KY117544 1	0,18987197
Procyon lotor AB462049 4	Eupleres goudotii D128 1	0,18987349
Vulpes lagopus NC026529 3	Bdeogale nigripes GLC15 1	0,18987402
Nasua nasua NC020647 1	Arctocephalus australis MG023139 1	0,18987413
Mustela sibirica AP017394 11	Galidia elegans D146 1	0,18987705
Ursus maritimus NC003428 31	Canis mesomelas KT448280 1	0,18987705
Vulpes vulpes NC008434 5	Procyon lotor AB462046 3	0,1898789
Suricata suricatta SSM10 1	Otaria byronia OTAB 1	0,18987931
Otaria byronia OTAB 1	Xenogale naso C07XAR110 1	0,18987973
Canis latrans NC008093 7	Arctocephalus pusillus NC008417 1	0,18988068
Lycalopex sechurae KT448284 1	Galerella sanguinea T378 1	0,18988076
Meles meles T303 3	Cryptoprocta ferox CFC13 1	0,18988134
Otocyon megalotis SAF1 2	Mungos mungo MMC7 1	0,18988222
Urocyon cinereoargenteus NC026723 2	Taxidea taxus NC020646 1	0,1898835
Otocyon megalotis SAF1 2	Atilax paludinosus T606 1	0,18988352
Ursus arctos isabellinus 18B5 2	Helogale parvula SRR7637809 1	0,18988559
Panthera onca KP202264 2	Arctocephalus australis MG023139 1	0,18988657
Galerella sanguinea T378 1	Ailurus fulgens NC011124 1	0,18988693
Panthera leo spelaea KX258452 2	Lutra lutra NC011358 9	0,18988903
Hyaena hyaena NC020669 1	Arctocephalus australis MG023139 1	0,18988918
Ursus maritimus NC003428 31	Bassaricyon neblina SRX1097850 1	0,18988973
Ursus maritimus GU573488 Svalbard	Bassaricyon neblina SRX1097850 1	0,18988983
Urocyon littoralis catalinae KP129018	Puma yagouaroundi NC028311 1	0,1898901
Parahyaena brunnea NC038159 15	Neophoca cinerea NC008419 1	0,1898912
Paradoxurus hermaphroditus NC03959	Arctocephalus pusillus NC008417 1	0,18989253
Lycalopex sechurae KT448284 1	Cynogale bennetti KY117544 1	0,18989293
Procyon lotor AB462046 3	Lycaon pictus NC028427 2	0,18989505
Ursus arctos GU573486 5	Bassaricyon neblina SRX1097850 1	0,18989588
Ursus maritimus NC003428 31	Panthera uncia KP202269 1	0,18989794
Ursus thibetanus thibetanus NC01111	Atilax paludinosus T606 1	0,1898988
Ursus spelaeus NC011112 8	Cynictis penicillata T375 1	0,1898991
Ursus maritimus GU573488 Svalbard	Felis nigripes NC028309 1	0,18989912
Ursus maritimus NC003428 31	Felis silvestris lybica KP202275 4	0,18989916
Eupleres goudotii D128 1	Canis lupus chanco NC010340 4	0,18989936
Odobenus rosmarus NC004029 29	Melogale moschata KP726273 1	0,18989968
Mungotictis decemlineata NC027828 1	Melursus ursinus NC009970 2	0,18989991
Ursus arctos pruinosus MG066703 3	Felis chaus NC028307 1	0,18989995
Ursus arctos pruinosus MG066703 3	Ichneumia albicauda T603 1	0,18990336
Canis lupus chanco NC010340 4	Arctocephalus pusillus NC008417 1	0,18990451
Prionailurus planiceps NC028312 6	Mephitis mephitis NC020648 1	0,18990532
Proteles cristata T393 6	Neovison vison NC020641 3	0,18990628
Otaria byronia OTAB 1	Diplogale hosei MH464790 1	0,18990876
Otaria byronia OTAB 1	Ailuropoda melanoleuca NC009492 5	0,18991078
Urva javanica/auropunctata NC006835	Aonyx cinerea NC035814 2	0,1899126
Eumetopias jubatus NC004030 10	Tapirus terrestris T358	0,18991493
Martes zibellina NC011579 39	Cuon alpinus NC013445 3	0,18991704
Ursus americanus JX196366 3	Paradoxurus hermaphroditus NC03959	0,18992878
Ursus americanus JX196366 3	Prionodon pardicolor NC024569 2	0,18993071
Ursus maritimus NC003428 31	Panthera leo NERO 19	0,18993282
Suricata suricatta SSM10 1	Tapirus terrestris T358	0,18993285
Panthera pardus NC010641 5	Mustela altaica NC021751 1	0,18993513

Phoca largha NC008430 1	Pardofelis marmorata NLN3 2	0,17481743
Phoca largha NC008430 1	Felis silvestris lybica KP202275 4	0,17481771
Procyon lotor AB462049 4	Lontra canadensis SRR10409165 1	0,17485325
Puma yagouaroundi NC028311 1	Halichoerus grypus NC001602 2	0,17485468
Salanoia concolor D378 1	Erignathus barbatus NC008426 1	0,17486183
Mustela nivalis T306 5	Bassaricyon neblina SRX1097850 1	0,1748648
Ommatophoca rossii AY377287etc 1	Leopardus pardalis NC028315 1	0,1748658
Mustela frenata NC020640 1	Hemigalus derbyanus MH464791 1	0,17486712
Pusa caspica NC008431 1	Prionailurus viverrinus NC028305 1	0,1748748
Bassariscus sumichrasti SRX1099089 1	Ailurus fulgens styani NC009691 1	0,174877
Monachus schauinslandi NC008421 1	Genetta servalina NC024568 2	0,17487833
Phoca groenlandica NC008429 54	Felis silvestris lybica KP202275 4	0,17487864
Mirounga angustirostris SRR10331586 1	Lynx rufus NC014456 3	0,1748789
Panthera onca NC022842 1	Cystophora cristata NC008427 1	0,17488112
Mirounga angustirostris SRR10331586 1	Ictonyx striatus T299 1	0,17488234
Mirounga leonina NC008422 1	Diplogale hosei MH464790 1	0,17488317
Paguma larvata PDDS11 2	Mirounga angustirostris SRR10331586 1	0,17488447
Felis catus NC001700 2	Canis adustus KT448271 1	0,17488487
Otocolobus manul NC028323 1	Canis adustus KT448271 1	0,17488956
Mustela erminea T305 2	Leopardus colocolo NC028314 1	0,17488982
Meles meles T303 3	Hemigalus derbyanus MH464791 1	0,17490201
Phoca vitulina NC001325 1	Genetta abyssinica MG489822 1	0,17490733
Taxidea taxus NC020646 1	Civettictis civetta NC033378 1	0,17490771
Civettictis civetta NC033378 1	Canis adustus KT448271 1	0,17491935
Eumetopias jubatus NC004030 10	Arctodus simus NC011116 1	0,17492032
Mustela eversmannii NC028013 1	Bassaricyon neblina SRX1097850 1	0,1749226
Cystophora cristata NC008427 1	Bassaricyon neblina SRX1097850 1	0,17492504
Pusa caspica NC008431 1	Mungotictis decemlineata NC027828 1	0,17492852
Paradoxurus hermaphroditus NC039591 1	Canis adustus KT448271 1	0,17493472
Viverra zibetha T609 1	Phoca fasciata NC008428 1	0,17493771
Pusa caspica NC008431 1	Prionailurus planiceps NC028312 6	0,17493948
Mustela erminea T305 2	Leopardus geoffroyi NC028320 1	0,17494093
Phoca fasciata NC008428 1	Pardofelis marmorata NLN3 2	0,17494538
Phoca fasciata NC008428 1	Felis silvestris lybica KP202275 4	0,17494559
Prionailurus planiceps KY682741 4	Monachus schauinslandi NC008421 1	0,17494616
Mirounga leonina NC008422 1	Galictis vittata T412 1	0,17494631
Phoca fasciata NC008428 1	Otocolobus manul NC028323 1	0,17494719
Ursus thibetanus thibetanus NC011118 4	Meles meles T303 3	0,17495245
Prionailurus rubiginosus NC028304 2	Phoca largha NC008430 1	0,17495306
Phoca vitulina NC001325 1	Leopardus tigrinus NC028317 1	0,17495525
Martes zibellina NC011579 39	Homotherium latidens MF871702 3	0,17495552
Urva javanica/auropunctata NC006835 1	Erignathus barbatus NC008426 1	0,17495769
Monachus monachus NC044972 5	Urva javanica T413 1	0,1749611
Halichoerus grypus NC001602 2	Bassaricyon neblina SRX1097850 1	0,17497623
Mustela kathiah NC023210 1	Conepatus chinga NC042596 1	0,1749905
Potos flavus T414 1	Callorhinus ursinus NC008415 1	0,17499104
Leopardus geoffroyi NC028320 1	Halichoerus grypus NC001602 2	0,17499134
Mustela erminea T305 2	Lynx lynx NC027083 4	0,17500414
Panthera pardus japonensis KJ866876 8	Cystophora cristata NC008427 1	0,1750046
Pusa sibirica NC008432 2	Catopuma temminckii NC027115 41	0,17500536
Pusa sibirica NC008432 2	Otocolobus manul NC028323 1	0,17500778
Pusa hispida NC 008433 1	Felis catus NC001700 2	0,17501627
Mustela kathiah NC023210 1	Mephitis mephitis NC020648 1	0,17501712
Canis adustus KT448271 1	Arctictis binturong T605 2	0,17501736
Phoca vitulina NC001325 1	Pardofelis marmorata NLN3 2	0,17501928
Panthera leo spelaea KX258452 2	Hydrurga leptonyx NC008425 1	0,17502421
Panthera tigris NC010642 35	Hydrurga leptonyx NC008425 1	0,17502561
Zalophus californianus NC008416 1	Arctodus simus NC011116 1	0,17502823
Felis chaus NC028307 1	Canis adustus KT448271 1	0,17503096
Martes melampus NC009678 1	Homotherium latidens MF871702 3	0,17505503
Mustela frenata NC020640 1	Bassaricyon neblina SRX1097850 1	0,1750572
Pusa hispida NC 008433 1	Chrotogale owstoni T607 1	0,17506127
Monachus monachus NC044972 5	Urva javanica/auropunctata NC006835 1	0,17506193
Phoca groenlandica NC008429 54	Chrotogale owstoni T607 1	0,1750641
Taxidea taxus NC020646 1	Arctocephalus forsteri KT693377 17	0,17506477
Nandinia binotata NC024567 1	Arctotherium sp NC030174 1	0,17506702
Phoca fasciata NC008428 1	Urva brachyura KY117547 1	0,17507049
Prionodon linsang ERR2391707 1	Canis adustus KT448271 1	0,17507307
Pusa sibirica NC008432 2	Felis catus NC001700 2	0,17507361
Pusa caspica NC008431 1	Prionailurus rubiginosus NC028304 2	0,17507453
Nyctereutes procyonoides NC013700 3	Hydrurga leptonyx NC008425 1	0,17507499
Pusa hispida NC 008433 1	Galerella sanguinea T378 1	0,17508075
Monachus schauinslandi NC008421 1	Felis silvestris lybica KP202275 4	0,17508119
Mirounga angustirostris SRR10331586 1	Felis nigripes NC028309 1	0,17508222
Pusa hispida NC 008433 1	Catopuma temminckii NC027115 41	0,1750825
Pusa hispida NC 008433 1	Felis chaus NC028307 1	0,17508352
Galictis vittata T412 1	Bassariscus sumichrasti SRX1099089 1	0,17508624
Monachus schauinslandi NC008421 1	Diplogale hosei MH464790 1	0,17508677
Parahyaena brunnea NC038159 15	Lobodon carcinophaga NC008423 1	0,17509069
Poecilogale albinucha T602 1	Monachus monachus NC044972 5	0,17510459
Prionailurus bengalensis CKM45 20	Ommatophoca rossii AY377287etc 1	0,17510492
Ommatophoca rossii AY377287etc 1	Mustela putorius NC020638 4	0,17510594
Viverricula indica KX891745 1	Canis lupus chanco NC010340 4	0,1751104
Halichoerus grypus NC001602 2	Eupleres goudotii D128 1	0,17511053
Mustela erminea T305 2	Homotherium latidens MF871702 3	0,17511128
Ommatophoca rossii AY377287etc 1	Fossa fossana D350 1	0,17511196
Taxidea taxus NC020646 1	Conepatus chinga NC042596 1	0,17511211
Halichoerus grypus NC001602 2	Felis silvestris lybica KP202275 4	0,17512333
Urva brachyura KY117547 1	Erignathus barbatus NC008426 1	0,17512792
Martes martes T302 3	Ailurus fulgens styani NC009691 1	0,17512853
Taxidea taxus NC020646 1	Arctocephalus australis MG023139 1	0,1751319
Ommatophoca rossii AY377287etc 1	Mustela sibirica NC020637 6	0,17513496
Viverra tangalunga MH464792 1	Hemigalus derbyanus MH464791 1	0,17513682
Pusa hispida NC 008433 1	Lontra canadensis SRR10409165 1	0,17513932
Phoca vitulina NC001325 1	Bassaricyon neblina SRX1097850 1	0,17513947
Galictis vittata T412 1	Erignathus barbatus NC008426 1	0,17514664
Monachus schauinslandi NC008421 1	Felis nigripes NC028309 1	0,17514856
Genetta servalina NC024568 2	Canis adustus KT448271 1	0,1751496
Mirounga angustirostris SRR10331586 1	Felis chaus NC028307 1	0,17515044
Phoca largha NC008430 1	Catopuma temminckii NC027115 41	0,17515288
Monachus schauinslandi NC008421 1	Arctictis binturong T605 2	0,17516017
Phoca vitulina NC001325 1	Hemigalus derbyanus MH464791 1	0,17516154
Smilodon populator MF871700 1	Halichoerus grypus NC001602 2	0,17516546

Lycalopex sechurae KT448284 1	Atilax paludinosus T606 1	0,18993719
Melogale moschata KP726273 1	Urva brachyura KY117547 1	0,18993834
Taxidea taxus NC020646 1	Tapirus terrestris T358	0,18993838
Suricata suricatta SSM10 1	Mustela putorius NC020638 4	0,18993931
Ursus arctos EU497665 29	Canis mesomelas KT448280 1	0,18994164
Urocyon littoralis catalinae KP129018	Crossarchus platycephalus C7R66 1	0,18994166
Vulpes lagopus NC026529 3	Suricata suricatta SSM10 1	0,18994172
Cynictis penicillata T375 1	Ailurus fulgens NC011124 1	0,18994235
Vulpes lagopus NC026529 3	Lutra sumatrana NC035810 1	0,1899426
Proteles cristata T393 6	Eumetopias jubatus NC004030 10	0,18994336
Vulpes vulpes NC008434 5	Galidia elegans D146 1	0,18994412
Paguma larvata PDDS11 2	Arctocephalus townsendi NC008420 1	0,18994519
Potos flavus T414 1	Canis mesomelas KT448280 1	0,18994747
Procyon lotor AB462046 3	Canis anthus NC027956 2	0,1899492
Lontra canadensis SRR10409165 1	Genetta servalina NC024568 2	0,18994944
Lycaon pictus NC028427 2	Callorhinus ursinus NC008415 1	0,18994993
Vulpes ferrilata NC027935 1	Paguma larvata PDDS11 2	0,18995045
Ursus arctos GU573486 5	Conepatus chinga NC042596 1	0,18995067
Melogale moschata KP726273 1	Ichneumia albicauda T603 1	0,18995158
Ursus arctos isabellinus 18B5 2	Prionodon linsang ERR2391707 1	0,18995211
Urva javanica/auropunctata NC006835	Cuon alpinus NC013445 3	0,18995585
Lontra canadensis SRR10409165 1	Caracal caracal NC028306 1	0,1899569
Ursus arctos isabellinus 18B5 2	Canis anthus NC027956 2	0,18995862
Procyon lotor AB462049 4	Panthera uncia KP202269 1	0,18995976
Ursus thibetanus laniger MH281753 2	Urva semitorquata MH464789 1	0,18996134
Ursus arctos GU573491 207	Paradoxurus hermaphroditus NLNC 1	0,18996233
Panthera tigris NC010642 35	Mustela nigripes NC024942 1	0,18996254
Ursus maritimus GU573488 Svalbard	Panthera uncia KP202269 1	0,18996531
Panthera onca NC022842 1	Arctocephalus forsteri KT693377 17	0,18996546
Prionodon pardicolor NC024569 2	Otocyon megalotis SAF1 2	0,18996669
Ursus arctos pruinosus MG066703 3	Leopardus tigrinus NC028317 1	0,18996672
Urva brachyura KY117547 1	Helarctos malayanus NC009968 2	0,1899678
Proteles cristata T393 6	Mustela nivalis T306 5	0,18997045
Mephitis mephitis NC020648 1	Leopardus tigrinus NC028317 1	0,18997142
Galidictis fasciata DM333 1	Chrysocyon brachyurus NC024172 1	0,18997276
Panthera tigris NC010642 35	Neovison vison NC020641 3	0,18997313
Panthera onca NC022842 1	Otaria byronia OTAB 1	0,18997319
Taxidea taxus NC020646 1	Crocuta crocuta NC020670 3	0,18997322
Aonyx cinerea NC035814 2	Ailuropoda melanoleuca NC009492 5	0,18997353
Melursus ursinus NC009970 2	Ichneumia albicauda T603 1	0,18997584
Procyon lotor AB462049 4	Canis aureus KT448274 1	0,18997768
Ursus spelaeus NC011112 8	Panthera uncia KP202269 1	0,18997811
Speothos venaticus C48 2	Arctictis binturong T605 2	0,18998012
Ursus arctos AP012576 6	Prionailurus planiceps KY682741 4	0,18998295
Zalophus californianus NC008416 1	Panthera uncia NC010638 1	0,18998404
Ursus arctos GU573486 5	Mephitis mephitis NC020648 1	0,1899848
Ursus spelaeus NC011112 8	Proteles cristata T393 6	0,18998528
Ursus americanus JX196366 3	Lutrogale perspicillata NC035811 1	0,18998586
Ursus thibetanus thibetanus NC01111	Panthera onca NC022842 1	0,18998875
Meles anakuma NC009677 1	Cuon alpinus NC013445 3	0,18999034
Panthera pardus NC010641 5	Meles anakuma NC009677 1	0,18999121
Vulpes ferrilata NC027935 1	Martes flavigula NC012141 3	0,18999363
Cuon alpinus NC013445 3	Chrotogale owstoni T607 1	0,18999502
Leopardus jacobita NC028322 1	Cuon alpinus NC013445 3	0,18999639
Felis nigripes NC028309 1	Arctocephalus forsteri NC004023 28	0,19000125
Proteles cristata T393 6	Canis adustus KT448271 1	0,19000132
Ursus maritimus NC003428 31	Panthera pardus NC010641 5	0,19000401
Nyctereutes procyonoides NC013700 3	Lutra lutra NC011358 9	0,19000469
Suricata suricatta SSM10 1	Mustela eversmannii NC028013 1	0,19000646
Urva javanica T413 1	Arctocephalus australis MG023139 1	0,19000721
Urva semitorquata MH464789 1	Bassaricyon neblina SRX1097850 1	0,19000846
Vulpes vulpes NC008434 5	Aonyx cinerea NC035814 2	0,19001098
Paradoxurus jerdoni MH464793 1	Arctocephalus townsendi NC008420 1	0,1900111
Salanoia concolor D378 1	Arctocephalus townsendi NC008420 1	0,19001201
Galidictis fasciata DM333 1	Arctocephalus townsendi NC008420 1	0,19001277
Procyon lotor AB462049 4	Cynictis penicillata T375 1	0,19001313
Helogale parvula SRR7637809 1	Canis mesomelas KT448280 1	0,19001544
Panthera uncia NC010638 1	Arctonyx collaris NC020645 1	0,19001742
Panthera uncia NC010638 1	Callorhinus ursinus NC008415 1	0,19001816
Hyaena hyaena NC020669 1	Canis anthus NC027956 2	0,19001916
Ursus arctos GU573486 5	Canis mesomelas KT448280 1	0,19001926
Nyctereutes procyonoides NC013700 3	Hyaena hyaena NC020669 1	0,19002046
Phocarctos hookeri NC008418 1	Paguma larvata PDDS11 2	0,1900208
Vulpes zerda KJ603240 1	Mustela altaica NC021751 1	0,19002165
Felis nigripes NC028309 1	Arctocephalus gazella BK010918 1	0,1900225
Spilogale putorius NC010497 1	Urva javanica/auropunctata NC006835	0,19002283
Urocyon cinereoargenteus NC026723 2	Puma concolor NC016470 22	0,19002414
Urocyon littoralis catalinae KP129018	Prionailurus planiceps KY682741 4	0,1900242
Ursus thibetanus thibetanus NC01111	Cynogale bennetti KY117544 1	0,19002433
Urocyon littoralis catalinae KP129018	Prionailurus viverrinus NC028305 1	0,19002521
Ursus arctos isabellinus 18B5 2	Hyaena hyaena NC020669 1	0,19002552
Diplogale hosei MH464790 1	Arctocephalus forsteri KT693377 17	0,19002584
Proteles cristata T393 6	Callorhinus ursinus NC008415 1	0,19002679
Panthera pardus japonensis KJ866876	Mustela altaica NC021751 1	0,19002843
Felis margarita NC028308 1	Bassaricyon neblina SRX1097850 1	0,190029
Viverra tangalunga MH464792 1	Odobenus rosmarus NC004029 29	0,19002904
Odobenus rosmarus NC004029 29	Mustela nigripes NC024942 1	0,19003014
Diplogale hosei MH464790 1	Cuon alpinus NC013445 3	0,19003026
Vulpes vulpes NC008434 5	Ursus thibetanus thibetanus NC01111	0,19003141
Ursus arctos EU497665 29	Ichneumia albicauda T603 1	0,19003163
Panthera pardus japonensis KJ866876	Lycalopex sechurae KT448284 1	0,19003183
Ursus arctos pruinosus MG066703 3	Panthera onca KP202264 2	0,19003263
Ursus arctos isabellinus 18B5 2	Felis nigripes NC028309 1	0,19003335
Ursus maritimus NC003428 31	Felis nigripes NC028309 1	0,19003379
Helogale parvula SRR7637809 1	Chrysocyon brachyurus NC024172 1	0,19003387
Suricata suricatta SSM10 1	Helarctos malayanus NC009968 2	0,19003394
Ursus arctos GU573491 207	Prionailurus viverrinus NC028305 1	0,19003427
Ursus arctos GU573491 207	Puma concolor NC016470 22	0,1900344
Odobenus rosmarus NC004029 29	Melogale moschata V0735A 1	0,19003469
Ursus thibetanus thibetanus NC01111	Galidia elegans D146 1	0,19003636
Mephitis mephitis NC020648 1	Leopardus jacobita NC028322 1	0,19003877
Zalophus californianus NC008416 1	Nasua nasua NC020647 1	0,19003962
Zalophus californianus NC008416 1	Urva javanica T413 1	0,19004023

Ommatophoca rossii AY377287etc 1	Martes foina NC020643 1	0,17517203
Taxidea taxus NC020646 1	Genetta abyssinica MG489822 1	0,17518157
Phoca largha NC008430 1	Civettictis civetta NC033378 1	0,17518829
Halichoerus grypus NC001602 2	Felis margarita NC028308 1	0,17519036
Viverra tangalunga MH464792 1	Nandinia binotata NC024567 1	0,17519261
Mirounga leonina NC008422 1	Eupleres goudotii D128 1	0,17519465
Eupleres goudotii D128 1	Erignathus barbatus NC008426 1	0,17519529
Vulpes vulpes NC008434 5	Leptonychotes weddellii NC008424 1	0,17519666
Mungotictis decemlineata NC027828 1	Cystophora cristata NC008427 1	0,17519821
Pusa sibirica NC008432 2	Civettictis civetta GLC19 1	0,17520489
Taxidea taxus NC020646 1	Civettictis civetta GLC19 1	0,17520609
Phoca groenlandica NC008429 54	Lutrogale perspicillata NC035811 1	0,17520717
Phoca fasciata NC008428 1	Felis margarita NC028308 1	0,17521511
Viverricula indica KX891745 1	Ommatophoca rossii AY377287etc 1	0,17522003
Phoca vitulina NC001325 1	Ailuropoda melanoleuca NC009492 5	0,17522496
Ommatophoca rossii AY377287etc 1	Leopardus guigna NC028321 1	0,17523755
Lynx canadensis NC028313 1	Canis adustus KT448271 1	0,17523862
Panthera pardus NC010641 5	Cystophora cristata NC008427 1	0,17524402
Pusa hispida NC 008433 1	Conepatus chinga NC042596 1	0,17525814
Ommatophoca rossii AY377287etc 1	Homotherium latidens MF871702 3	0,17526047
Mustela erminea T305 2	Arctocephalus gazella BK010918 1	0,17526226
Lutrogale perspicillata NC035811 1	Erignathus barbatus NC008426 1	0,17526676
Viverricula indica KX891751 1	Taxidea taxus NC020646 1	0,17526692
Viverricula indica KX891745 1	Taxidea taxus NC020646 1	0,17526811
Viverra zibetha T609 1	Taxidea taxus NC020646 1	0,17526902
Viverricula indica NC025296 2	Ommatophoca rossii AY377287etc 1	0,17527072
Phoca fasciata NC008428 1	Urva semitorquata MH464789 1	0,17527251
Viverra zibetha T609 1	Phoca groenlandica NC008429 54	0,17527439
Phoca vitulina NC001325 1	Catopuma temminckii NC027115 41	0,17528756
Phoca largha NC008430 1	Felis margarita NC028308 1	0,17528921
Neovison vison NC020641 3	Arctodus simus NC011116 1	0,17529169
Mustela eversmannii NC028013 1	Monachus monachus NC044972 5	0,17529371
Mirounga leonina NC008422 1	Urva javanica/auropunctata NC006835 1	0,17529652
Ommatophoca rossii AY377287etc 1	Mustela nigripes NC024942 1	0,17531148
Mustela erminea T305 2	Fossa fossana D350 1	0,17532948
Panthera uncia KP202269 1	Erignathus barbatus NC008426 1	0,17534019
Ommatophoca rossii AY377287etc 1	Leopardus jacobita NC028322 1	0,17534027
Pusa sibirica NC008432 2	Pardofelis marmorata NLN3 2	0,17534304
Pusa sibirica NC008432 2	Caracal caracal NC028306 1	0,17534435
Mirounga angustirostris SRR10331586 1	Felis silvestris lybica KP202275 4	0,17535146
Pusa hispida NC 008433 1	Panthera onca KP202264 2	0,17535388
Paradoxurus jerdoni MH464793 1	Neovison vison NC020641 3	0,17535785
Smilodon populator MF871700 1	Phoca largha NC008430 1	0,1753621
Ommatophoca rossii AY377287etc 1	Meles anakuma NC009677 1	0,17536376
Mungos mungo/gambianus SRR7704821 1	Monachus monachus NC044972 5	0,17536519
Pusa hispida NC 008433 1	Panthera leo NERO 19	0,17538564
Melogale moschata V0735A 1	Ailurus fulgens styani NC009691 1	0,17539664
Martes pennanti NC020664 16	Ailurus fulgens styani NC009691 1	0,17540246
Nandinia binotata NC024567 1	Mustela nivalis T306 5	0,1754037
Mustela erminea T305 2	Lynx canadensis NC028313 1	0,1754083
Pusa caspica NC008431 1	Felis catus NC001700 2	0,17541032
Mirounga angustirostris SRR10331586 1	Urva brachyura KY117547 1	0,17541036
Prionailurus planiceps NC028312 6	Monachus schauinslandi NC008421 1	0,17541739
Monachus schauinslandi NC008421 1	Lynx rufus NC014456 3	0,17541757
Mirounga leonina NC008422 1	Mephitis mephitis NC020648 1	0,17542033
Pusa hispida NC 008433 1	Pardofelis marmorata NLN3 2	0,17542073
Pusa hispida NC 008433 1	Mephitis mephitis NC020648 1	0,1754248
Ursus thibetanus thibetanus NC011118 4	Mustela erminea T305 2	0,17542896
Pusa hispida NC 008433 1	Panthera onca NC022842 1	0,17543212
Prionailurus planiceps KY682741 4	Halichoerus grypus NC001602 2	0,17546012
Halichoerus grypus NC001602 2	Cryptoprocta ferox CFC13 1	0,17546551
Taxidea taxus NC020646 1	Arctodus simus NC011116 1	0,17546895
Lynx lynx NC027083 4	Enhydra lutris NC009692 1	0,17547236
Mirounga leonina NC008422 1	Felis silvestris lybica KP202275 4	0,1754728
Phoca groenlandica NC008429 54	Paguma larvata PDDS11 2	0,1754764
Parahyaena brunnea NC038159 15	Cystophora cristata NC008427 1	0,17548054
Taxidea taxus NC020646 1	Lynx lynx NC027083 4	0,17548105
Monachus schauinslandi NC008421 1	Felis catus NC001700 2	0,17548555
Panthera uncia KP202269 1	Lobodon carcinophaga NC008423 1	0,17548665
Phoca largha NC008430 1	Genetta genetta T297 1	0,17548849
Eumetopias jubatus NC004030 10	Bassariscus sumichrasti SRX1099089 1	0,17551111
Neovison vison NC020641 3	Genetta abyssinica MG489822 1	0,1755261
Viverra tangalunga MH464792 1	Bassaricyon neblina SRX1097850 1	0,17552833
Melogale moschata KP726273 1	Bassaricyon neblina SRX1097850 1	0,17552845
Ommatophoca rossii AY377287etc 1	Lynx lynx NC027083 4	0,17552884
Phoca vitulina NC001325 1	Conepatus chinga NC042596 1	0,17553155
Pusa caspica NC008431 1	Urva semitorquata MH464789 1	0,17553191
Civettictis civetta GLC19 1	Canis adustus KT448271 1	0,17553493
Neophoca cinerea NC008419 1	Bassariscus sumichrasti SRX1099089 1	0,1755355
Pusa hispida NC 008433 1	Mungos mungo/gambianus SRR7704821 1	0,175541
Pusa sibirica NC008432 2	Genetta servalina NC024568 2	0,17554137
Phoca fasciata NC008428 1	Atilax paludinosus T606 1	0,1755417
Phoca fasciata NC008428 1	Crossarchus platycephalus C7R66 1	0,17554197
Proteles cristata T393 6	Cynogale bennetti KY117544 1	0,17554246
Meles leucurus NC039173 4	Hemigalus derbyanus MH464791 1	0,17554373
Mirounga angustirostris SRR10331586 1	Urva semitorquata MH464789 1	0,17554377
Mustela erminea T305 2	Leopardus guigna NC028321 1	0,17554682
Taxidea taxus NC020646 1	Lynx rufus NC014456 3	0,17554717
Phoca largha NC008430 1	Mungotictis decemlineata NC027828 1	0,17554884
Phoca vitulina NC001325 1	Felis chaus NC028307 1	0,17555811
Viverricula indica KX891751 1	Neovison vison NC020641 3	0,17555837
Ursus thibetanus formosanus NC009331 1	Mustela erminea T305 2	0,17556128
Mustela erminea T305 2	Genetta abyssinica MG489822 1	0,17558423
Ursus thibetanus laniger MH281753 2	Ommatophoca rossii AY377287etc 1	0,17558548
Tremarctos ornatus NC009969 2	Ommatophoca rossii AY377287etc 1	0,17558762
Mirounga leonina NC008422 1	Canis aureus KT448274 1	0,17558847
Nasua nasua NC020647 1	Mustela erminea T305 2	0,17559583
Pusa sibirica NC008432 2	Mungos mungo MMC7 1	0,17560118
Potos flavus T414 1	Arctotherium sp NC030174 1	0,17560457
Pusa hispida NC 008433 1	Mungos mungo MMC7 1	0,17560892
Pusa sibirica NC008432 2	Galerella sanguinea T378 1	0,17560904
Nandinia binotata NC024567 1	Ictonyx striatus T299 1	0,17561045
Phoca vitulina NC001325 1	Mungos mungo/gambianus SRR7704821 1	0,17561305

Panthera leo spelaea KX258452 2	Mustela nivalis T306 5	0,19004124
Ursus thibetanus laniger MH281753 2	Panthera uncia NC010638 1	0,19004484
Urva javanica/auropunctata NC006835	Arctocephalus townsendi NC008420 1	0,19004558
Ursus spelaeus NC011112 8	Prionodon pardicolor NC024569 2	0,19004583
Ursus thibetanus laniger MH281753 2	Proteles cristata T393 6	0,19005005
Ursus spelaeus NC011112 8	Panthera onca NC022842 1	0,19005598
Neofelis nebulosa NC008450 3	Mustela putorius NC020638 4	0,1900578
Spilogale putorius NC010497 1	Leopardus pardalis T262 1	0,19006081
Ursus arctos GU573491 207	Lycalopex sechurae KT448284 1	0,19006117
Ursus arctos EU497665 29	Lycalopex sechurae KT448284 1	0,19006199
Ursus arctos isabellinus 18B5 2	Panthera leo NERO 19	0,19006751
Ursus thibetanus mupinensis NC00875	Panthera leo NERO 19	0,19006883
Spilogale putorius NC010497 1	Catopuma badia NC028300 1	0,19006947
Eupleres goudotii D128 1	Ailurus fulgens styani NC009691 1	0,19007167
Urva brachyura KY117547 1	Conepatus chinga NC042596 1	0,19007316
Cuon alpinus NC013445 3	Bassariscus sumichrasti SRX1099089 1	0,1900733
Cynogale bennetti KY117544 1	Canis latrans NC008093 7	0,19007391
Urva javanica T413 1	Arctocephalus gazella BK010918 1	0,19007576
Eumetopias jubatus NC004030 10	Crocuta crocuta NC020670 3	0,19007611
Ursus thibetanus thibetanus NC01111	Panthera leo NERO 19	0,19007799
Bdeogale nigripes GLC15 1	Aonyx cinerea NC035814 2	0,19007896
Urva brachyura KY117547 1	Aonyx cinerea NC035814 2	0,19007936
Galidia elegans D146 1	Arctocephalus forsteri KT693377 17	0,19008045
Viverricula indica KX891751 1	Arctocephalus townsendi NC008420 1	0,19008055
Paguma larvata PDDS11 2	Arctocephalus forsteri KT693377 17	0,19008099
Vulpes ferrilata NC027935 1	Chrotogale owstoni T607 1	0,19008107
Otocyon megalotis SAF1 2	Bassariscus sumichrasti SRX1099089 1	0,19008559
Viverra tangalunga MH464792 1	Cryptoprocta ferox CFC13 1	0,19008723
Vulpes corsac NC023958 1	Genetta genetta T297 1	0,19008723
Prionodon pardicolor NC024569 2	Callorhinus ursinus NC008415 1	0,19008756
Vulpes corsac NC023958 1	Panthera pardus japonensis KJ866876	0,19008823
Vulpes zerda KJ603240 1	Panthera pardus japonensis KJ866876	0,19008909
Ursus arctos isabellinus 18B5 2	Lontra canadensis SRR10409165 1	0,1900914
Zalophus californianus NC008416 1	Panthera pardus NC010641 5	0,19009189
Meles anakuma NC009677 1	Lycaon pictus NC028427 2	0,19009218
Parahyaena brunnea NC038159 15	Arctocephalus forsteri KT693377 17	0,19009301
Parahyaena brunnea NC038159 15	Arctocephalus australis MG023139 1	0,1900932
Ursus thibetanus laniger MH281753 2	Cynogale bennetti KY117544 1	0,19009364
Panthera onca NC022842 1	Ailurus fulgens NC011124 1	0,19009417
Poecilogale albinucha T602 1	Panthera pardus japonensis KJ866876	0,19009446
Parahyaena brunnea NC038159 15	Arctocephalus gazella BK010918 1	0,19009474
Proteles cristata T393 6	Bassariscus sumichrasti SRX1099089 1	0,19009505
Ursus arctos GU573491 207	Cryptoprocta ferox CFC13 1	0,19009648
Panthera tigris NC010642 35	Mustela putorius NC020638 4	0,19009669
Proteles cristata T393 6	Otaria byronia OTAB 1	0,19009766
Otocyon megalotis SAF1 2	Mustela nivalis T306 5	0,19009784
Panthera leo spelaea KX258452 2	Mustela eversmannii NC028013 1	0,19009833
Vulpes vulpes NC008434 5	Panthera leo spelaea KX258452 2	0,1901011
Melursus ursinus NC009970 2	Bassaricyon neblina SRX1097850 1	0,19010136
Ursus arctos pruinosus MG066703 3	Prionailurus planiceps NC028312 6	0,19010164
Ursus maritimus NC003428 31	Otocolobus manul NC028323 1	0,19010253
Panthera tigris NC010642 35	Bassariscus sumichrasti SRX1099089 1	0,19010387
Paradoxurus hermaphroditus NC03959	Martes flavigula NC012141 3	0,19010727
Odobenus rosmarus NC004029 29	Melogale moschata NC020644 1	0,19010891
Arctocephalus pusillus NC008417 1	Arctictis binturong T605 2	0,19011058
Canis aureus KT448274 1	Arctocephalus forsteri KT693377 17	0,19011247
Urva javanica/auropunctata NC006835	Ailurus fulgens styani NC009691 1	0,19011328
Speothos venaticus C48 2	Ailuropoda melanoleuca NC009492 5	0,19011371
Ursus arctos AP012576 6	Puma concolor NC016470 22	0,19011785
Ursus spelaeus NC011112 8	Lycaon pictus NC028427 2	0,19011842
Vulpes corsac NC023958 1	Gulo gulo NC009685 3	0,19012205
Panthera leo NERO 19	Arctotherium sp NC030174 1	0,1901316
Viverricula indica KX891751 1	Arctocephalus forsteri NC004023 28	0,19013396
Ursus maritimus GU573488 Svalbard	Panthera leo NERO 19	0,19013484
Urva javanica/auropunctata NC006835	Galictis vittata T412 1	0,19013599
Ursus arctos GU573486 5	Diplogale hosei MH464790 1	0,19013685
Suricata suricatta SSM10 1	Mustela sibirica AP017394 11	0,19014101
Bassaricyon neblina SRX1097850 1	Atilax paludinosus T606 1	0,19014309
Vulpes vulpes NC008434 5	Urva semitorquata MH464789 1	0,19014317
Urocyon littoralis catalinae KP129018	Fossa fossana D350 1	0,19014348
Phocarctos hookeri NC008418 1	Galidia elegans D146 1	0,19014685
Urocyon cinereoargenteus NC026723 2	Mustela sibirica AP017394 11	0,19014896
Viverricula indica KX891751 1	Arctocephalus gazella BK010918 1	0,19014983
Tremarctos ornatus NC009969 2	Bassaricyon neblina SRX1097850 1	0,19014993
Prionodon linsang ERR2391707 1	Martes pennanti NC020664 16	0,19015065
Ursus thibetanus mupinensis NC00875	Cynogale bennetti KY117544 1	0,19015121
Speothos venaticus C48 2	Atilax paludinosus T606 1	0,19015525
Urocyon littoralis catalinae KP129018	Leopardus pardalis T262 1	0,19015808
Urocyon cinereoargenteus NC026723 2	Felis nigripes NC028309 1	0,19015822
Ursus arctos GU573491 207	Lontra canadensis SRR10409165 1	0,19015859
Urocyon cinereoargenteus NC026723 2	Prionailurus planiceps KY682741 4	0,19015875
Ursus arctos GU573486 5	Lutrogale perspicillata NC035811 1	0,19016069
Panthera pardus japonensis KJ866876	Martes martes T302 3	0,19016434
Ursus maritimus GU573488 Svalbard	Otocyon megalotis SAF1 2	0,19016503
Vulpes vulpes NC008434 5	Ursus spelaeus EU327344 13	0,19016589
Vulpes corsac NC023958 1	Ursus spelaeus EU327344 13	0,19016603
Phocarctos hookeri NC008418 1	Panthera tigris amoyensis NC014770 2	0,19016764
Ursus arctos GU573491 207	Felis silvestris lybica KP202275 4	0,19016829
Ursus maritimus GU573488 Svalbard	Felis catus NC001700 2	0,19016831
Ursus arctos GU573491 207	Prionailurus planiceps NC028312 6	0,19016859
Panthera onca NC022842 1	Arctocephalus gazella BK010918 1	0,19016927
Ursus maritimus GU573488 Svalbard	Otocolobus manul NC028323 1	0,19016989
Procyon lotor AB462046 3	Panthera tigris NC010642 35	0,19017033
Procyon lotor AB462046 3	Panthera tigris amoyensis NC014770 2	0,19017035
Ursus spelaeus EU327344 13	Mungotictis decemlineata NC027828 1	0,19017164
Melursus ursinus NC009970 2	Canis latrans NC008093 7	0,19017285
Mungotictis decemlineata NC027828 1	Chrysocyon brachyurus NC024172 1	0,19017348
Ursus arctos pruinosus MG066703 3	Cryptoprocta ferox CFC13 1	0,19017372
Canis aureus KT448274 1	Arctocephalus gazella BK010918 1	0,19017552
Ursus thibetanus laniger MH281753 2	Parahyaena brunnea NC038159 15	0,19017734
Zalophus californianus NC008416 1	Lycalopex sechurae KT448284 1	0,19018075
Ursus arctos AP012576 6	Cryptoprocta ferox CFC13 1	0,19018161
Ursus arctos AP012576 6	Prionailurus viverrinus NC028305 1	0,19018173

Pusa caspica NC008431 1	Leopardus guigna NC028321 1	0,17561474
Mustela nigripes NC024942 1	Arctodus simus NC011116 1	0,17561497
Panthera leo spelaea KX258452 2	Leptonychotes weddellii NC008424 1	0,17561693
Ursus thibetanus mupinensis NC008753 2	Mustela erminea T305 2	0,17561901
Panthera tigris NC010642 35	Cystophora cristata NC008427 1	0,17561939
Viverra zibetha T609 1	Phoca largha NC008430 1	0,17562072
Phoca fasciata NC008428 1	Ichneumia albicauda T603 1	0,17562209
Viverra zibetha T609 1	Neovison vison NC020641 3	0,17562562
Lycalopex sechurae KT448284 1	Hydrurga leptonyx NC008425 1	0,17562576
Mirounga angustirostris SRR10331586 1	Genetta genetta T297 1	0,17562582
Pusa hispida NC 008433 1	Hyaena hyaena NC020669 1	0,1756261
Phoca largha NC008430 1	Otocolobus manul NC028323 1	0,17562678
Phoca largha NC008430 1	Ailuropoda melanoleuca NC009492 5	0,17562866
Mirounga leonina NC008422 1	Canis lupus familiaris NC002008 1231	0,17563151
Neofelis nebulosa NC008450 3	Leptonychotes weddellii NC008424 1	0,17564295
Neovison vison NC020641 3	Eumetopias jubatus NC004030 10	0,17564628
Helogale parvula SRR7637809 1	Cystophora cristata NC008427 1	0,17566657
Procyon lotor AB462046 3	Nandinia binotata NC024567 1	0,17566961
Pusa sibirica NC008432 2	Galidictis fasciata DM333 1	0,17566988
Mirounga angustirostris SRR10331586 1	Conepatus chinga NC042596 1	0,17567086
Nandinia binotata NC024567 1	Mustela altaica NC021751 1	0,17567381
Phoca groenlandica NC008429 54	Eupleres goudotii D128 1	0,17567487
Mustela frenata NC020640 1	Leopardus pardalis NC028315 1	0,17567594
Martes melampus NC009678 1	Lynx pardinus NC028319 161	0,17567609
Prionodon linsang ERR2391707 1	Hydrurga leptonyx NC008425 1	0,17567678
Genetta genetta T297 1	Cystophora cristata NC008427 1	0,1756779
Salanoia concolor D378 1	Phoca groenlandica NC008429 54	0,17567925
Mirounga angustirostris SRR10331586 1	Urva javanica T413 1	0,1756795
Pusa hispida NC 008433 1	Mungotictis decemlineata NC027828 1	0,17568144
Taxidea taxus NC020646 1	Lynx pardinus NC028319 161	0,17568204
Bassariscus sumichrasti SRX1099089 1	Ailurus fulgens NC011124 1	0,17568456
Paguma larvata PDDS11 2	Neovison vison NC020641 3	0,17569353
Hydrurga leptonyx NC008425 1	Chrysocyon brachyurus NC024172 1	0,17569548
Puma yagouaroundi NC028311 1	Canis adustus KT448271 1	0,17569605
Ommatophoca rossii AY377287etc 1	Leopardus geoffroyi NC028320 1	0,17569624
Viverra tangalunga MH464792 1	Ommatophoca rossii AY377287etc 1	0,17572056
Prionailurus rubiginosus NC028304 2	Halichoerus grypus NC001602 2	0,17572904
Hydrurga leptonyx NC008425 1	Cynogale bennetti KY117544 1	0,17573087
Viverra zibetha T609 1	Mustela kathiah NC023210 1	0,1757452
Ommatophoca rossii AY377287etc 1	Helarctos malayanus NC009968 2	0,17574562
Prionailurus viverrinus NC028305 1	Mustela erminea T305 2	0,17574799
Ursus thibetanus mupinensis NC008753 2	Meles meles T303 3	0,17574924
Procyon lotor AB462046 3	Monachus schauinslandi NC008421 1	0,17574973
Pusa sibirica NC008432 2	Ailuropoda melanoleuca NC009492 5	0,17575261
Mephitis mephitis NC020648 1	Cystophora cristata NC008427 1	0,17575318
Mephitis mephitis NC020648 1	Erignathus barbatus NC008426 1	0,17575464
Galictis vittata T412 1	Cystophora cristata NC008427 1	0,17575563
Phoca largha NC008430 1	Civettictis civetta GLC19 1	0,17575623
Taxidea taxus NC020646 1	Arctocephalus forsteri NC004023 28	0,1757572
Phoca vitulina NC001325 1	Felis catus NC001700 2	0,17576008
Ursus spelaeus NC011112 8	Mustela erminea T305 2	0,17576547
Lutra lutra NC011358 9	Ailurus fulgens NC011124 1	0,17578734
Felis nigripes NC028309 1	Canis adustus KT448271 1	0,1757988
Martes zibellina NC011579 39	Ailurus fulgens NC011124 1	0,1757997
Ursus spelaeus EU327344 13	Ommatophoca rossii AY377287etc 1	0,17580108
Pusa sibirica NC008432 2	Urva semitorquata MH464789 1	0,17580123
Vulpes lagopus NC026529 3	Leptonychotes weddellii NC008424 1	0,17580265
Mustela erminea T305 2	Arctocephalus pusillus NC008417 1	0,17580515
Neovison vison NC020641 3	Bassaricyon neblina SRX1097850 1	0,17580566
Martes pennanti NC020664 16	Ailurus fulgens NC011124 1	0,17580667
Mirounga leonina NC008422 1	Felis margarita NC028308 1	0,17580982
Panthera uncia NC010638 1	Leptonychotes weddellii NC008424 1	0,17581032
Paradoxurus hermaphroditus NLNC 1	Lobodon carcinophaga NC008423 1	0,1758173
Zalophus wollebaeki SRR4431565 1	Arctodus simus NC011116 1	0,17582181
Pusa hispida NC 008433 1	Genetta genetta T297 1	0,17582195
Phoca largha NC008430 1	Felis nigripes NC028309 1	0,17582774
Ommatophoca rossii AY377287etc 1	Aonyx cinerea NC035814 2	0,17584077
Mustela erminea T305 2	Callorhinus ursinus NC008415 1	0,17586435
Erignathus barbatus NC008426 1	Bdeogale nigripes GLC15 1	0,17586857
Conepatus chinga NC042596 1	Bassariscus sumichrasti SRX1099089 1	0,17586978
Nandinia binotata NC024567 1	Martes pennanti NC020664 16	0,17587031
Salanoia concolor D378 1	Mirounga leonina NC008422 1	0,17587297
Taxidea taxus NC020646 1	Neophoca cinerea NC008419 1	0,17587428
Paradoxurus hermaphroditus NLNC 1	Mirounga leonina NC008422 1	0,17587788
Phoca fasciata NC008428 1	Genetta genetta T297 1	0,17588165
Paradoxurus jerdoni MH464793 1	Hydrurga leptonyx NC008425 1	0,17588435
Pusa hispida NC 008433 1	Ichneumia albicauda T603 1	0,17589158
Fossa fossana D350 1	Canis adustus KT448271 1	0,17589942
Martes melampus NC009678 1	Eumetopias jubatus NC004030 10	0,17590685
Viverricula indica KX891745 1	Canis lupus familiaris NC002008 1231	0,17590888
Pusa hispida NC 008433 1	Urva javanica/auropunctata NC006835 1	0,17590915
Panthera leo NERO 19	Erignathus barbatus NC008426 1	0,17591085
Monachus monachus NC044972 5	Aonyx cinerea NC035814 2	0,1759122
Mirounga angustirostris SRR10331586 1	Urva javanica/auropunctata NC006835 1	0,17591585
Monachus monachus NC044972 5	Lynx rufus NC014456 3	0,17591593
Monachus monachus NC044972 5	Arctictis binturong T605 2	0,17592254
Mustela erminea T305 2	Hemigalus derbyanus MH464791 1	0,17592457
Lontra canadensis SRR10409165 1	Bassaricyon neblina SRX1097850 1	0,1759294
Hemigalus derbyanus MH464791 1	Canis adustus KT448271 1	0,1759315
Mustela frenata NC020640 1	Canis adustus KT448271 1	0,17593516
Melogale moschata KP726273 1	Ailurus fulgens NC011124 1	0,17593545
Procyon lotor AB462049 4	Lutrogale perspicillata NC035811 1	0,17593654
Prionailurus bengalensis NC028301 12	Meles meles T303 3	0,175944
Mirounga angustirostris SRR10331586 1	Melogale moschata V0735A 1	0,17594433
Mustela frenata NC020640 1	Leopardus pardalis T262 1	0,17594524
Phoca vitulina NC001325 1	Lontra canadensis SRR10409165 1	0,17594543
Mirounga leonina NC008422 1	Catopuma temminckii NC027115 41	0,17594576
Martes flavigula NC012141 3	Eumetopias jubatus NC004030 10	0,17594938
Pusa sibirica NC008432 2	Felis chaus NC028307 1	0,1759496
Vulpes vulpes NC008434 5	Hydrurga leptonyx NC008425 1	0,17595045
Mirounga angustirostris SRR10331586 1	Lutrogale perspicillata NC035811 1	0,17595178
Mustela frenata NC020640 1	Mephitis mephitis NC020648 1	0,175954
Ictonyx striatus T299 1	Ailurus fulgens NC011124 1	0,17595413

Zalophus californianus NC008416 1	Panthera pardus japonensis KJ866876	0,1901869
Enhydra lutris NC009692 1	Diplogale hosei MH464790 1	0,19019035
Panthera onca NC022842 1	Galictis vittata T412 1	0,19019184
Helogale parvula SRR7637809 1	Cuon alpinus NC013445 3	0,19019587
Ursus thibetanus formosanus NC00933	Odobenus rosmarus NC004029 29	0,1901973
Panthera pardus NC010641 5	Neophoca cinerea NC008419 1	0,19019857
Ursus arctos GU573491 207	Diplogale hosei MH464790 1	0,19019872
Spilogale putorius NC010497 1	Lynx lynx NC027083 4	0,19020222
Ursus maritimus GU573488 Svalbard	Panthera pardus NC010641 5	0,19020602
Vulpes zerda KJ603240 1	Lutra lutra NC011358 9	0,19021021
Prionodon linsang ERR2391707 1	Melogale moschata V0735A 1	0,19021036
Vulpes corsac NC023958 1	Mungos mungo MMC7 1	0,19021046
Urocyon cinereoargenteus NC026723 2	Fossa fossana D350 1	0,19021086
Phocarctos hookeri NC008418 1	Urva brachyura KY117547 1	0,19021124
Vulpes zerda KJ603240 1	Galerella sanguinea T378 1	0,19021174
Phocarctos hookeri NC008418 1	Nasua nasua NC020647 1	0,19021185
Otaria byronia OTAB 1	Eupleres goudotii D128 1	0,1902121
Urocyon cinereoargenteus NC026723 2	Salanoia concolor D378 1	0,19021389
Vulpes zerda KJ603240 1	Lutra sumatrana NC035810 1	0,19021392
Mustela nigripes NC024942 1	Galidia elegans D146 1	0,19021423
Galidia elegans D146 1	Arctocephalus townsendi NC008420 1	0,19021444
Neophoca cinerea NC008419 1	Galidia elegans D146 1	0,19021459
Vulpes lagopus NC026529 3	Arctocephalus pusillus NC008417 1	0,19021575
Urocyon littoralis catalinae KP129018	Mustela sibirica AP017394 11	0,19021638
Salanoia concolor D378 1	Procyon lotor AB462049 4	0,19021699
Panthera uncia NC010638 1	Meles leucurus NC039173 4	0,19021741
Viverricula indica KX891745 1	Lontra canadensis SRR10409165 1	0,19021778
Vulpes vulpes NC008434 5	Arctotherium sp NC030174 1	0,19021839
Meles anakuma NC009677 1	Canis mesomelas KT448280 1	0,19022021
Vulpes vulpes NC008434 5	Ursus thibetanus mupinensis NC00875	0,19022158
Lycaon pictus NC028427 2	Arctocephalus gazella BK010918 1	0,19022188
Ursus maritimus NC003428 31	Helogale parvula SRR7637809 1	0,19022244
Panthera onca KP202264 2	Arctocephalus gazella BK010918 1	0,19022512
Urocyon littoralis catalinae KP129018	Felis nigripes NC028309 1	0,19022562
Nyctereutes procyonoides NC013700 3	Genetta genetta T297 1	0,19022565
Urocyon littoralis catalinae KP129018	Prionailurus planiceps NC028312 6	0,19022639
Panthera pardus japonensis KJ866876	Neophoca cinerea NC008419 1	0,19022661
Mephitis mephitis NC020648 1	Arctocephalus forsteri KT693377 17	0,19022755
Otocyon megalotis SAF1 2	Arctotherium sp NC030174 1	0,19023007
Proteles cristata T393 6	Mustela kathiah NC023210 1	0,19023131
Otocolobus manul NC028323 1	Lutrogale perspicillata NC035811 1	0,19023134
Vulpes corsac NC023958 1	Panthera tigris amoyensis NC014770 2	0,19023152
Panthera tigris NC010642 35	Arctocephalus australis MG023139 1	0,19023237
Vulpes zerda KJ603240 1	Panthera onca NC022842 1	0,19023426
Phocarctos hookeri NC008418 1	Panthera tigris NC010642 35	0,19023491
Ursus spelaeus NC011112 8	Atilax paludinosus T606 1	0,19023544
Panthera tigris NC010642 35	Neophoca cinerea NC008419 1	0,19023545
Ursus arctos GU573491 207	Leopardus tigrinus NC028317 1	0,19023596
Ursus arctos pruinosus MG066703 3	Puma concolor NC016470 22	0,19023672
Procyon lotor AB462046 3	Chrysocyon brachyurus NC024172 1	0,1902374
Ursus spelaeus NC011112 8	Urva javanica T413 1	0,19023937
Neophoca cinerea NC008419 1	Arctictis binturong T605 2	0,19024026
Ursus arctos AP012576 6	Canis latrans NC008093 7	0,19024122
Ursus arctos GU573486 5	Felis nigripes NC028309 1	0,19024198
Canis aureus KT448274 1	Aonyx cinerea NC035814 2	0,19024576
Panthera leo NERO 19	Conepatus chinga NC042596 1	0,19024585
Ursus thibetanus mupinensis NC00875	Panthera onca NC022842 1	0,19024585
Ursus arctos AP012576 6	Felis chaus NC028307 1	0,19025187
Mungos mungo MMC7 1	Lycalopex sechurae KT448284 1	0,19025733
Ursus arctos AP012576 6	Mephitis mephitis NC020648 1	0,19026206
Vulpes lagopus NC026529 3	Martes flavigula NC012141 3	0,19026224
Vulpes lagopus NC026529 3	Eumetopias jubatus NC004030 10	0,19026318
Panthera tigris NC010642 35	Canis mesomelas KT448280 1	0,19026387
Spilogale putorius NC010497 1	Lycalopex sechurae KT448284 1	0,19026548
Panthera pardus NC010641 5	Martes americana NC020642 1	0,19026682
Panthera uncia KP202269 1	Gulo gulo NC009685 3	0,19026749
Ursus maritimus GU573488 Svalbard	Lycalopex sechurae KT448284 1	0,19026925
Canis latrans NC008093 7	Callorhinus ursinus NC008415 1	0,19027482
Urva brachyura KY117547 1	Arctocephalus townsendi NC008420 1	0,19027524
Urva javanica T413 1	Arctocephalus townsendi NC008420 1	0,19027539
Atilax paludinosus T606 1	Ailurus fulgens NC011124 1	0,19027762
Vulpes ferrilata NC027935 1	Cynictis penicillata T375 1	0,19027802
Lontra canadensis SRR10409165 1	Canis anthus NC027956 2	0,19027816
Lycalopex sechurae KT448284 1	Bassariscus sumichrasti SRX1099089 1	0,1902784
Bdeogale nigripes GLC15 1	Ailurus fulgens NC011124 1	0,19027853
Suricata suricatta SSM10 1	Neophoca cinerea NC008419 1	0,19027868
Prionodon linsang ERR2391707 1	Mustela eversmannii NC028013 1	0,19027959
Otocyon megalotis SAF1 2	Urva javanica T413 1	0,19028662
Otocyon megalotis SAF1 2	Galerella sanguinea T378 1	0,19028702
Ursus arctos pruinosus MG066703 3	Conepatus chinga NC042596 1	0,19028758
Melogale moschata V0735A 1	Lycaon pictus NC028427 2	0,19028999
Vulpes corsac NC023958 1	Panthera onca KP202264 2	0,19029024
Suricata suricatta SSM10 1	Ictonyx striatus T299 1	0,19029069
Viverricula indica KX891751 1	Neophoca cinerea NC008419 1	0,19029085
Otocyon megalotis SAF1 2	Aonyx cinerea NC035814 2	0,19029114
Ursus arctos GU573491 207	Galidia elegans D146 1	0,19029269
Panthera onca KP202264 2	Lontra canadensis SRR10409165 1	0,19029276
Urocyon littoralis catalinae KP129018	Felis silvestris lybica KP202275 4	0,19029322
Viverricula indica NC025296 2	Lutrogale perspicillata NC035811 1	0,19029382
Mephitis mephitis NC020648 1	Arctocephalus townsendi NC008420 1	0,190294
Ichneumia albicauda T603 1	Ailurus fulgens NC011124 1	0,19029433
Crocuta crocuta NC020670 3	Arctonyx collaris NC020645 1	0,19029538
Speothos venaticus C48 2	Melogale moschata NC020644 1	0,19029778
Panthera onca KP202264 2	Otaria byronia OTAB 1	0,19029814
Vulpes corsac NC023958 1	Ursus thibetanus formosanus NC00933	0,19029836
Vulpes corsac NC023958 1	Panthera tigris NC010642 35	0,19029848
Vulpes ferrilata NC027935 1	Ursus thibetanus laniger MH281753 2	0,19030114
Ursus arctos GU573491 207	Felis catus NC001700 2	0,19030273
Ursus maritimus NC003428 31	Felis catus NC001700 2	0,19030297
Proteles cristata T393 6	Martes pennanti NC020664 16	0,19030401
Panthera uncia KP202269 1	Lycaon pictus NC028427 2	0,19030404
Mustela nivalis T306 5	Crocuta crocuta NC020670 3	0,19030562
Prionodon linsang ERR2391707 1	Mephitis mephitis NC020648 1	0,19030645

Martes martes T302 3	Lynx pardinus NC028319 161	0,17595422
Phoca groenlandica NC008429 54	Felis nigripes NC028309 1	0,17595626
Neovison vison NC020641 3	Genetta servalina NC024568 2	0,17595671
Prionodon pardicolor NC024569 2	Hydrurga leptonyx NC008425 1	0,17595686
Pusa hispida NC 008433 1	Panthera uncia KP202269 1	0,17595994
Phoca vitulina NC001325 1	Genetta genetta T297 1	0,17596017
Phoca largha NC008430 1	Cryptoprocta ferox CFC13 1	0,17596181
Phoca vitulina NC001325 1	Otocolobus manul NC028323 1	0,17596333
Zalophus californianus NC008416 1	Mustela frenata NC020640 1	0,17596546
Martes foina NC020643 1	Homotherium latidens MF871702 3	0,1759695
Ommatophoca rossii AY377287etc 1	Ailurus fulgens styani NC009691 1	0,17597282
Mustela altaica NC021751 1	Monachus monachus NC044972 5	0,17597553
Ursus spelaeus NC011112 8	Ommatophoca rossii AY377287etc 1	0,17597914
Monachus schauinslandi NC008421 1	Urva javanica/auropunctata NC006835 1	0,17597987
Halichoerus grypus NC001602 2	Crossarchus platycephalus C7R66 1	0,17598452
Leopardus pardalis NC028315 1	Canis adustus KT448271 1	0,17598896
Monachus monachus NC044972 5	Mephitis mephitis NC020648 1	0,17599134
Halichoerus grypus NC001602 2	Felis nigripes NC028309 1	0,17599908
Bassaricyon neblina SRX1097850 1	Aonyx cinerea NC035814 2	0,17600083
Cystophora cristata NC008427 1	Canis latrans NC008093 7	0,17600419
Panthera onca KP202264 2	Erignathus barbatus NC008426 1	0,17601342
Prionailurus bengalensis NC028301 12	Mustela erminea T305 2	0,1760162
Ursus thibetanus thibetanus NC011118 4	Ommatophoca rossii AY377287etc 1	0,17601842
Hydrurga leptonyx NC008425 1	Galidia elegans D146 1	0,17602065
Phoca groenlandica NC008429 54	Felis catus NC001700 2	0,17602314
Ursus spelaeus NC011112 8	Potos flavus T414 1	0,17602605
Ursus thibetanus formosanus NC009331 1	Meles meles T303 3	0,17602703
Monachus monachus NC044972 5	Canis aureus KT448274 1	0,17602758
Viverricula indica KX891745 1	Neovison vison NC020641 3	0,17602991
Pusa caspica NC008431 1	Urva javanica/auropunctata NC006835 1	0,17603351
Monachus monachus NC044972 5	Urva semitorquata MH464789 1	0,17603393
Puma yagouaroundi NC028311 1	Ommatophoca rossii AY377287etc 1	0,17604424
Pusa caspica NC008431 1	Civettictis civetta NC033378 1	0,17604929
Martes melampus NC009678 1	Callorhinus ursinus NC008415 1	0,17606254
Ommatophoca rossii AY377287etc 1	Genetta servalina NC024568 2	0,17606477
Mustela kathiah NC023210 1	Hemigalus derbyanus MH464791 1	0,17606528
Prionailurus planiceps NC028312 6	Halichoerus grypus NC001602 2	0,17606644
Pusa caspica NC008431 1	Cynictis penicillata T375 1	0,17607056
Pusa caspica NC008431 1	Mungos mungo/gambianus SRR7704821 1	0,17607056
Cynogale bennetti KY117544 1	Crocuta crocuta NC020670 3	0,17608142
Viverra tangalunga MH464792 1	Prionailurus bengalensis NC028301 12	0,1760817
Prionailurus rubiginosus NC028304 2	Mustela erminea T305 2	0,17608342
Pusa sibirica NC008432 2	Ichneumia albicauda T603 1	0,17608385
Erignathus barbatus NC008426 1	Diplogale hosei MH464790 1	0,17608616
Phoca largha NC008430 1	Mungos mungo MMC7 1	0,17608647
Taxidea taxus NC020646 1	Leopardus pardalis NC028315 1	0,17608665
Vulpes zerda KJ603240 1	Lobodon carcinophaga NC008423 1	0,17608836
Ommatophoca rossii AY377287etc 1	Felis nigripes NC028309 1	0,17609078
Viverra zibetha T609 1	Phoca vitulina NC001325 1	0,1760923
Neovison vison NC020641 3	Canis adustus KT448271 1	0,1760938
Poecilogale albinucha T602 1	Phoca groenlandica NC008429 54	0,17609382
Pusa hispida NC 008433 1	Parahyaena brunnea NC038159 15	0,17609932
Prionailurus bengalensis NC028301 12	Canis adustus KT448271 1	0,17610103
Diplogale hosei MH464790 1	Canis adustus KT448271 1	0,17610438
Mirounga leonina NC008422 1	Canis lupus chanco NC010340 4	0,17611022
Meles leucurus NC039173 4	Bassaricyon neblina SRX1097850 1	0,17613091
Nandinia binotata NC024567 1	Melogale moschata V0735A 1	0,17613436
Nandinia binotata NC024567 1	Melogale moschata KP726273 1	0,17613442
Erignathus barbatus NC008426 1	Canis latrans NC008093 7	0,1761391
Viverricula indica KX891745 1	Canis aureus KT448274 1	0,17614295
Panthera onca KP202264 2	Mirounga leonina NC008422 1	0,17614557
Arctodus simus NC011116 1	Arctocephalus forsteri KT693377 17	0,17614645
Mustela frenata NC020640 1	Felis silvestris lybica KP202275 4	0,17614742
Viverricula indica KX891751 1	Mustela kathiah NC023210 1	0,17614864
Mirounga angustirostris SRR10331586 1	Eupleres goudotii D128 1	0,17614883
Taxidea taxus NC020646 1	Prionailurus bengalensis NC028301 12	0,17615017
Taxidea taxus NC020646 1	Prionailurus rubiginosus NC028304 2	0,17615034
Arctodus simus NC011116 1	Arctocephalus pusillus NC008417 1	0,17615354
Pusa sibirica NC008432 2	Parahyaena brunnea NC038159 15	0,17615423
Prionailurus bengalensis CKM45 20	Mustela erminea T305 2	0,176155
Panthera tigris NC010642 35	Leptonychotes weddellii NC008424 1	0,17615643
Phoca groenlandica NC008429 54	Felis chaus NC028307 1	0,17615862
Mirounga angustirostris SRR10331586 1	Catopuma temminckii NC027115 41	0,1761613
Pusa hispida NC 008433 1	Panthera pardus japonensis KJ866876 8	0,17616199
Ommatophoca rossii AY377287etc 1	Martes martes T302 3	0,17616253
Ursus thibetanus laniger MH281753 2	Meles meles T303 3	0,17616367
Monachus monachus NC044972 5	Martes americana NC020642 1	0,17616685
Halichoerus grypus NC001602 2	Civettictis civetta NC033378 1	0,17616734
Taxidea taxus NC020646 1	Spilogale putorius NC010497 1	0,17617249
Ommatophoca rossii AY377287etc 1	Lynx pardinus NC028319 161	0,17618252
Genetta abyssinica MG489822 1	Arctonyx collaris NC020645 1	0,17618787
Leptonychotes weddellii NC008424 1	Tapirus terrestris T358	0,17619088
Halichoerus grypus NC001602 2	Caracal caracal NC028306 1	0,17619973
Bassaricyon neblina SRX1097850 1	Arctonyx collaris NC020645 1	0,17619997
Ommatophoca rossii AY377287etc 1	Canis adustus KT448271 1	0,17620085
Mustela frenata NC020640 1	Genetta abyssinica MG489822 1	0,17620188
Prionodon pardicolor NC024569 2	Canis adustus KT448271 1	0,17620756
Lynx lynx NC027083 4	Canis adustus KT448271 1	0,17620799
Galidictis fasciata DM333 1	Erignathus barbatus NC008426 1	0,17620896
Mirounga leonina NC008422 1	Galidictis fasciata DM333 1	0,17621006
Paradoxurus hermaphroditus NC039591 1	Leptonychotes weddellii NC008424 1	0,17621435
Pusa caspica NC008431 1	Genetta servalina NC024568 2	0,17621474
Pusa caspica NC008431 1	Galerella sanguinea T378 1	0,17621502
Phoca groenlandica NC008429 54	Crossarchus platycephalus C7R66 1	0,17621544
Taxidea taxus NC020646 1	Otaria byronia OTAB 1	0,17621651
Mustela erminea T305 2	Leopardus jacobita NC028322 1	0,17621817
Pusa caspica NC008431 1	Pardofelis marmorata NLN3 2	0,17621833
Phoca largha NC008430 1	Eupleres goudotii D128 1	0,17621993
Taxidea taxus NC020646 1	Leopardus pardalis T262 1	0,17622069
Zalophus wollebaeki SRR4431565 1	Mustela frenata NC020640 1	0,17622077
Paradoxurus hermaphroditus NC039591 1	Lobodon carcinophaga NC008423 1	0,1762214
Taxidea taxus NC020646 1	Leopardus geoffroyi NC028320 1	0,17622207
Otocolobus manul NC028323 1	Monachus schauinslandi NC008421 1	0,17622496

Ursus arctos AP012576 6	Bassaricyon neblina SRX1097850 1	0,19030987
Paradoxurus hermaphroditus NLNC 1	Galictis vittata T412 1	0,19031169
Gulo gulo NC009685 3	Diplogale hosei MH464790 1	0,19031498
Panthera leo NERO 19	Tapirus terrestris T358	0,19031624
Ursus thibetanus laniger MH281753 2	Speothos venaticus C48 2	0,19031829
Tremarctos ornatus NC009969 2	Canis lupus familiaris NC002008 1231	0,19031903
Ursus americanus JX196366 3	Mungos mungo MMC7 1	0,19031955
Panthera pardus japonensis KJ866876	Canis mesomelas KT448280 1	0,19032101
Vulpes corsac NC023958 1	Panthera leo NERO 19	0,19032125
Ursus arctos AP012576 6	Leopardus guigna NC028321 1	0,19032413
Neofelis nebulosa NC008450 3	Mustela sibirica AP017394 11	0,19032732
Spilogale putorius NC010497 1	Leopardus pardalis NC028315 1	0,1903294
Urva brachyura KY117547 1	Arctocephalus forsteri NC004023 28	0,19032983
Spilogale putorius NC010497 1	Prionailurus planiceps NC028312 6	0,19033073
Panthera tigris amoyensis NC014770 2	Canis mesomelas KT448280 1	0,19033249
Ursus arctos GU573486 5	Panthera leo NERO 19	0,19034283
Vulpes lagopus NC026529 3	Eupleres goudotii D128 1	0,19034307
Smilodon populator MF871700 1	Bassaricyon neblina SRX1097850 1	0,19034319
Vulpes lagopus NC026529 3	Ichneumia albicauda T603 1	0,19034529
Nasua nasua NC020647 1	Arctocephalus forsteri KT693377 17	0,19034557
Mungos mungo/gambianus SRR77048	Bassaricyon neblina SRX1097850 1	0,19034559
Vulpes zerda KJ603240 1	Atilax paludinosus T606 1	0,19034657
Vulpes zerda KJ603240 1	Urva semitorquata MH464789 1	0,19034682
Parahyaena brunnea NC038159 15	Arctocephalus forsteri NC004023 28	0,1903477
Mustela itatsi NC034330 19	Cynogale bennetti KY117544 1	0,19034848
Procyon lotor AB462049 4	Helogale parvula SRR7637809 1	0,19034879
Procyon lotor AB462046 3	Helogale parvula SRR7637809 1	0,190349
Ursus thibetanus mupinensis NC00875	Eupleres goudotii D128 1	0,19035397
Vulpes zerda KJ603240 1	Paguma larvata PDDS11 2	0,19035544
Lutrogale perspicillata NC035811 1	Galerella sanguinea T378 1	0,19035622
Viverricula indica KX891751 1	Phocarctos hookeri NC008418 1	0,19035691
Ursus maritimus GU573488 Svalbard	Mungos mungo/gambianus SRR77048	0,19035712
Urocyon cinereoargenteus NC026723 2	Civettictis civetta GLC19 1	0,19035725
Pardofelis marmorata NLN3 2	Arctocephalus townsendi NC008420 1	0,19035795
Lycaon pictus NC028427 2	Aonyx cinerea NC035814 2	0,19035868
Martes zibellina NC011579 39	Crocuta crocuta NC020670 3	0,19036008
Phocarctos hookeri NC008418 1	Panthera onca KP202264 2	0,19036065
Urocyon cinereoargenteus NC026723 2	Prionailurus planiceps NC028312 6	0,19036093
Phocarctos hookeri NC008418 1	Catopuma temminckii NC027115 41	0,1903612
Genetta genetta T297 1	Aonyx cinerea NC035814 2	0,19036154
Phocarctos hookeri NC008418 1	Felis nigripes NC028309 1	0,19036184
Ursus arctos GU573491 207	Canis latrans NC008093 7	0,19036216
Parahyaena brunnea NC038159 15	Canis latrans NC008093 7	0,19036283
Procyon lotor AB462049 4	Prionodon pardicolor NC024569 2	0,19036344
Odobenus rosmarus NC004029 29	Mustela putorius NC020638 4	0,19036676
Panthera tigris amoyensis NC014770 2	Arctocephalus townsendi NC008420 1	0,19036677
Vulpes lagopus NC026529 3	Helarctos malayanus NC009968 2	0,19036742
Puma yagouaroundi NC028311 1	Bassaricyon neblina SRX1097850 1	0,19036749
Ursus maritimus GU573488 Svalbard	Prionodon pardicolor NC024569 2	0,1903687
Panthera onca NC022842 1	Arctocephalus australis MG023139 1	0,1903694
Proteles cristata T393 6	Melogale moschata NC020644 1	0,19037019
Ursus spelaeus NC011112 8	Prionodon linsang ERR2391707 1	0,19037028
Ursus arctos pruinosus MG066703 3	Felis catus NC001700 2	0,19037038
Ursus arctos EU497665 29	Felis silvestris lybica KP202275 4	0,19037053
Viverra tangalunga MH464792 1	Panthera leo spelaea KX258452 2	0,19037341
Ursus arctos GU573486 5	Panthera uncia KP202269 1	0,19037549
Parahyaena brunnea NC038159 15	Canis aureus KT448274 1	0,19037643
Zalophus wollebaeki SRR4431565 1	Arctictis binturong T605 2	0,19038124
Ursus thibetanus formosanus NC00933	Speothos venaticus C48 2	0,19038254
Ursus spelaeus NC011112 8	Pardofelis marmorata NLN3 2	0,19038308
Ursus arctos AP012576 6	Galerella sanguinea T378 1	0,19038469
Urva javanica/auropunctata NC006835	Arctocephalus gazella BK010918 1	0,19038522
Ursus thibetanus formosanus NC00933	Proteles cristata T393 6	0,19038767
Panthera onca NC022842 1	Chrysocyon brachyurus NC024172 1	0,19038792
Spilogale putorius NC010497 1	Arctocephalus gazella BK010918 1	0,1903946
Ursus americanus JX196366 3	Lontra canadensis SRR10409165 1	0,19039474
Eumetopias jubatus NC004030 10	Canis anthus NC027956 2	0,19039598
Spilogale putorius NC010497 1	Canis anthus NC027956 2	0,1903961
Conepatus chinga NC042596 1	Atilax paludinosus T606 1	0,19039854
Suricata suricatta SSM10 1	Arctocephalus forsteri NC004023 28	0,19039869
Mephitis mephitis NC020648 1	Arctocephalus forsteri NC004023 28	0,19040054
Salanoia concolor D378 1	Cuon alpinus NC013445 3	0,19040054
Cynictis penicillata T375 1	Conepatus chinga NC042596 1	0,19040361
Ictonyx striatus T299 1	Canis lupus familiaris NC002008 1231	0,19040753
Procyon lotor AB462046 3	Eupleres goudotii D128 1	0,19041255
Helogale parvula SRR7637809 1	Ailurus fulgens NC011124 1	0,19041258
Vulpes vulpes NC008434 5	Urva brachyura KY117547 1	0,1904126
Urocyon cinereoargenteus NC026723 2	Enhydra lutris NC009692 1	0,19041333
Vulpes zerda KJ603240 1	Helogale parvula SRR7637809 1	0,19041401
Ursus arctos EU497665 29	Conepatus chinga NC042596 1	0,19041543
Mustela putorius NC020638 4	Galidia elegans D146 1	0,19041586
Tremarctos ornatus NC009969 2	Galidia elegans D146 1	0,19041719
Salanoia concolor D378 1	Procyon lotor AB462046 3	0,1904195
Vulpes ferrilata NC027935 1	Arctotherium sp NC030174 1	0,19042125
Panthera pardus japonensis KJ866876	Meles anakuma NC009677 1	0,19042201
Ursus thibetanus mupinensis NC00875	Urva semitorquata MH464789 1	0,19042258
Vulpes corsac NC023958 1	Ursus thibetanus mupinensis NC00875	0,19042422
Viverricula indica KX891745 1	Lutrogale perspicillata NC035811 1	0,1904244
Panthera pardus japonensis KJ866876	Martes melampus NC009678 1	0,19042441
Vulpes ferrilata NC027935 1	Prionodon pardicolor NC024569 2	0,19042496
Vulpes zerda KJ603240 1	Panthera onca KP202264 2	0,19042583
Ursus maritimus NC003428 31	Galidictis fasciata DM333 1	0,19042805
Phocarctos hookeri NC008418 1	Felis catus NC001700 2	0,1904287
Profelis aurata NC028299 1	Lontra canadensis SRR10409165 1	0,1904289
Proteles cristata T393 6	Meles anakuma NC009677 1	0,19042931
Zalophus wollebaeki SRR4431565 1	Nasua nasua NC020647 1	0,19043024
Mustela putorius NC020638 4	Crocuta crocuta NC020670 3	0,19043052
Pardofelis marmorata NLN3 2	Lutrogale perspicillata NC035811 1	0,19043095
Panthera leo spelaea KX258452 2	Martes melampus NC009678 1	0,19043201
Otocyon megalotis SAF1 2	Mustela altaica NC021751 1	0,19043316
Proteles cristata T393 6	Mustela itatsi NC034330 19	0,1904332
Ursus thibetanus thibetanus NC01111	Urva semitorquata MH464789 1	0,19043523
Ursus arctos pruinosus MG066703 3	Bassaricyon neblina SRX1097850 1	0,19043528

Pardofelis marmorata NLN3 2	Monachus schauinslandi NC008421 1	0,17622638
Phoca groenlandica NC008429 54	Leopardus geoffroyi NC028320 1	0,17622909
Pusa hispida NC 008433 1	Cryptoprocta ferox CFC13 1	0,17622973
Mirounga leonina NC008422 1	Arctictis binturong T605 2	0,17622977
Pusa hispida NC 008433 1	Panthera tigris NC010642 35	0,17623773
Phoca fasciata NC008428 1	Mephitis mephitis NC020648 1	0,17624313
Spilogale putorius NC010497 1	Meles anakuma NC009677 1	0,17625098
Monachus monachus NC044972 5	Chrysocyon brachyurus NC024172 1	0,17625868
Phoca vitulina NC001325 1	Civettictis civetta NC033378 1	0,17626597
Otocolobus manul NC028323 1	Halichoerus grypus NC001602 2	0,17626712
Callorhinus ursinus NC008415 1	Bassariscus sumichrasti SRX1099089 1	0,17627235
Nandinia binotata NC024567 1	Arctocephalus pusillus NC008417 1	0,17627633
Paguma larvata PDDS11 2	Canis adustus KT448271 1	0,17628056
Lutra lutra LC050126 1	Homotherium latidens MF871702 3	0,17628081
Mustela frenata NC020640 1	Leptailurus serval NC028316 1	0,17628148
Phoca largha NC008430 1	Cynictis penicillata T375 1	0,17628588
Phoca fasciata NC008428 1	Felis catus NC001700 2	0,17629212
Lynx rufus NC014456 3	Canis adustus KT448271 1	0,17629285
Phoca groenlandica NC008429 54	Ictonyx striatus T299 1	0,17629382
Pusa hispida NC 008433 1	Profelis aurata NC028299 1	0,17629556
Mustela erminea T305 2	Mephitis mephitis NC020648 1	0,1762958
Ommatophoca rossii AY377287etc 1	Crossarchus platycephalus C7R66 1	0,17629839
Phoca largha NC008430 1	Felis chaus NC028307 1	0,17629901
Panthera tigris amoyensis NC014770 2	Lobodon carcinophaga NC008423 1	0,17630488
Mungos mungo MMC7 1	Monachus monachus NC044972 5	0,17630811
Pusa hispida NC 008433 1	Panthera leo spelaea KX258452 2	0,17630838
Martes martes T302 3	Eumetopias jubatus NC004030 10	0,17631522
Ommatophoca rossii AY377287etc 1	Lynx rufus NC014456 3	0,1763353
Ommatophoca rossii AY377287etc 1	Leopardus tigrinus NC028317 1	0,17633854
Vulpes vulpes NC008434 5	Erignathus barbatus NC008426 1	0,17634083
Nandinia binotata NC024567 1	Aonyx cinerea NC035814 2	0,17634426
Enhydra lutris NC009692 1	Arctodus simus NC011116 1	0,17634866
Prionailurus bengalensis NC028301 12	Mustela frenata NC020640 1	0,17634903
Puma concolor NC016470 22	Mustela frenata NC020640 1	0,17634906
Mustela frenata NC020640 1	Leopardus wiedii NC028318 1	0,17634943
Poecilogale albinucha T602 1	Nandinia binotata NC024567 1	0,1763515
Puma concolor NC016470 22	Mustela erminea T305 2	0,17635238
Meles meles T303 3	Arctodus simus NC011116 1	0,17635244
Phoca vitulina NC001325 1	Mungos mungo MMC7 1	0,17635588
Pusa sibirica NC008432 2	Mephitis mephitis NC020648 1	0,17635839
Lycalopex sechurae KT448284 1	Leptonychotes weddellii NC008424 1	0,17636079
Phoca largha NC008430 1	Felis catus NC001700 2	0,17636628
Mustela nivalis T306 5	Homotherium latidens MF871702 3	0,1763802
Mustela frenata NC020640 1	Homotherium latidens MF871702 3	0,17639174
Ursus thibetanus formosanus NC009331 1	Ommatophoca rossii AY377287etc 1	0,17639686
Erignathus barbatus NC008426 1	Cynictis penicillata T375 1	0,17640722
Arctonyx collaris NC020645 1	Arctocephalus pusillus NC008417 1	0,17640862
Nyctereutes procyonoides NC013700 3	Erignathus barbatus NC008426 1	0,17640918
Pardofelis marmorata NLN3 2	Mirounga leonina NC008422 1	0,17641547
Viverra zibetha T609 1	Pusa caspica NC008431 1	0,17641641
Viverricula indica KX891745 1	Mustela erminea T305 2	0,17641887
Phoca fasciata NC008428 1	Mungotictis decemlineata NC027828 1	0,17641927
Pusa caspica NC008431 1	Panthera pardus japonensis KJ866876 8	0,17641982
Phoca groenlandica NC008429 54	Genetta genetta T297 1	0,17642052
Vulpes corsac NC023958 1	Lobodon carcinophaga NC008423 1	0,176422
Pusa hispida NC 008433 1	Galidictis fasciata DM333 1	0,17642239
Pusa sibirica NC008432 2	Hyaena hyaena NC020669 1	0,17642243
Salanoia concolor D378 1	Mirounga angustirostris SRR10331586 1	0,17642306
Parahyaena brunnea NC038159 15	Erignathus barbatus NC008426 1	0,1764233
Ursus spelaeus EU327344 13	Mustela erminea T305 2	0,17643945
Martes zibellina NC011579 39	Eumetopias jubatus NC004030 10	0,17644598
Paguma larvata PDDS11 2	Monachus monachus NC044972 5	0,1764468
Monachus monachus NC044972 5	Urva brachyura KY117547 1	0,17644781
Ommatophoca rossii AY377287etc 1	Mustela nivalis T306 5	0,17644851
Ommatophoca rossii AY377287etc 1	Martes zibellina NC011579 39	0,17645009
Meles leucurus NC039173 4	Callorhinus ursinus NC008415 1	0,17646532
Meles meles T303 3	Callorhinus ursinus NC008415 1	0,17646587
Lutra sumatrana NC035810 1	Callorhinus ursinus NC008415 1	0,17646657
Nandinia binotata NC024567 1	Callorhinus ursinus NC008415 1	0,17647086
Leopardus tigrinus NC028317 1	Halichoerus grypus NC001602 2	0,17647344
Mirounga leonina NC008422 1	Atilax paludinosus T606 1	0,17647456
Procyon lotor AB462046 3	Lutrogale perspicillata NC035811 1	0,17647497
Vulpes corsac NC023958 1	Leptonychotes weddellii NC008424 1	0,1764759
Mirounga leonina NC008422 1	Canis latrans NC008093 7	0,17647699
Taxidea taxus NC020646 1	Genetta servalina NC024568 2	0,17648366
Monachus monachus NC044972 5	Canis lupus chanco NC010340 4	0,17648523
Pusa sibirica NC008432 2	Genetta genetta T297 1	0,1764863
Pusa sibirica NC008432 2	Panthera onca KP202264 2	0,17648721
Pusa caspica NC008431 1	Felis nigripes NC028309 1	0,17648792
Mustela putorius NC020638 4	Arctodus simus NC011116 1	0,17648998
Pusa caspica NC008431 1	Leopardus tigrinus NC028317 1	0,17649046
Phoca largha NC008430 1	Galerella sanguinea T378 1	0,176498
Phoca vitulina NC001325 1	Cryptoprocta ferox CFC13 1	0,17650074
Zalophus californianus NC008416 1	Arctonyx collaris NC020645 1	0,17650197
Ursus thibetanus laniger MH281753 2	Mustela erminea T305 2	0,1765044
Pusa hispida NC 008433 1	Galictis vittata T412 1	0,17650929
Spilogale putorius NC010497 1	Lutra sumatrana NC035810 1	0,1765105
Halichoerus grypus NC001602 2	Galerella sanguinea T378 1	0,17653231
Arctonyx collaris NC020645 1	Arctocephalus forsteri KT693377 17	0,17653641
Leopardus colocolo NC028314 1	Canis adustus KT448271 1	0,1765399
Leopardus guigna NC028321 1	Halichoerus grypus NC001602 2	0,17654042
Mustela frenata NC020640 1	Arctocephalus gazella BK010918 1	0,1765411
Mirounga leonina NC008422 1	Urva javanica T413 1	0,17654188
Suricata suricatta SSM10 1	Cystophora cristata NC008427 1	0,17654188
Urva javanica T413 1	Erignathus barbatus NC008426 1	0,17654188
Phoca groenlandica NC008429 54	Lontra canadensis SRR10409165 1	0,17654681
Pardofelis marmorata NLN3 2	Ommatophoca rossii AY377287etc 1	0,17654805
Mustela frenata NC020640 1	Genetta servalina NC024568 2	0,17654955
Pusa hispida NC 008433 1	Cynictis penicillata T375 1	0,17655127
Mustela erminea T305 2	Lynx rufus NC014456 3	0,17655344
Pusa caspica NC008431 1	Genetta genetta T297 1	0,17655347
Ichneumia albicauda T603 1	Erignathus barbatus NC008426 1	0,17655422
Pusa hispida NC 008433 1	Paguma larvata PDDS11 2	0,17655763

Vulpes lagopus NC026529 3	Ursus spelaeus NC011112 8	0,19043531
Vulpes zerda KJ603240 1	Ursus thibetanus thibetanus NC01111	0,1904356
Martes pennanti NC020664 16	Crocuta crocuta NC020670 3	0,19043642
Ursus thibetanus mupinensis NC00875	Panthera uncia NC010638 1	0,1904375
Procyon lotor AB462049 4	Panthera tigris NC010642 35	0,19043933
Zalophus wollebaeki SRR4431565 1	Panthera uncia NC010638 1	0,190441
Melursus ursinus NC009970 2	Canis anthus NC027956 2	0,19044217
Lynx rufus NC014456 3	Ailuropoda melanoleuca NC009492 5	0,19044311
Zalophus californianus NC008416 1	Canis latrans NC008093 7	0,19044463
Ursus spelaeus EU327344 13	Pardofelis marmorata NLN3 2	0,19044626
Paradoxurus hermaphroditus NC03959	Galictis vittata T412 1	0,19044676
Ursus thibetanus thibetanus NC01111	Panthera uncia NC010638 1	0,19044973
Ursus spelaeus NC011112 8	Cryptoprocta ferox CFC13 1	0,19045448
Vulpes ferrilata NC027935 1	Panthera leo NERO 19	0,19045599
Zalophus californianus NC008416 1	Lycaon pictus NC028427 2	0,19045727
Panthera leo NERO 19	Lontra canadensis SRR10409165 1	0,19045988
Speothos venaticus C48 2	Martes flavigula NC012141 3	0,19046469
Parahyaena brunnea NC038159 15	Canis lupus familiaris NC002008 1231	0,19046514
Zalophus californianus NC008416 1	Arctictis binturong T605 2	0,19046661
Spilogale putorius NC010497 1	Caracal caracal NC028306 1	0,1904669
Mephitis mephitis NC020648 1	Canis aureus KT448274 1	0,19046779
Procyon lotor AB462046 3	Cuon alpinus NC013445 3	0,19047077
Pardofelis marmorata NLN3 2	Arctocephalus forsteri NC004023 28	0,19047272
Ursus arctos isabellinus 18B5 2	Panthera pardus NC010641 5	0,19047493
Ommatophoca rossii AY377287etc 1	Cuon alpinus NC013445 3	0,19047676
Nyctereutes procyonoides NC013700 3	Callorhinus ursinus NC008415 1	0,19047903
Vulpes lagopus NC026529 3	Meles leucurus NC039173 4	0,19047925
Lontra canadensis SRR10409165 1	Galidia elegans D146 1	0,19047964
Melogale moschata V0735A 1	Canis anthus NC027956 2	0,19048102
Urocyon littoralis catalinae KP129018	Salanoia concolor D378 1	0,19048315
Mustela nigripes NC024942 1	Cynogale bennetti KY117544 1	0,19048327
Conepatus chinga NC042596 1	Canis latrans NC008093 7	0,19048485
Tremarctos ornatus NC009969 2	Canis latrans NC008093 7	0,19048531
Ursus thibetanus thibetanus NC01111	Urva javanica/auropunctata NC006835	0,19048536
Canis anthus NC027956 2	Arctocephalus pusillus NC008417 1	0,19048636
Otocyon megalotis SAF1 2	Meles leucurus NC039173 4	0,19048697
Otocyon megalotis SAF1 2	Bdeogale nigripes GLC15 1	0,19048901
Lycaon pictus NC028427 2	Arctocephalus australis MG023139 1	0,19049041
Vulpes ferrilata NC027935 1	Martes pennanti NC020664 16	0,19049047
Urocyon littoralis catalinae KP129018	Civettictis civetta GLC19 1	0,19049191
Ursus thibetanus mupinensis NC00875	Atilax paludinosus T606 1	0,19049208
Panthera uncia KP202269 1	Arctocephalus townsendi NC008420 1	0,19049214
Felis nigripes NC028309 1	Arctocephalus townsendi NC008420 1	0,1904922
Ursus thibetanus formosanus NC0093	Cynogale bennetti KY117544 1	0,19049323
Ursus maritimus GU573488 Svalbard	Galidia elegans D146 1	0,19049479
Hyaena hyaena NC020669 1	Arctocephalus forsteri KT693377 17	0,19049501
Phocarctos hookeri NC008418 1	Pardofelis marmorata NLN3 2	0,19049573
Xenogale naso C07XAR110 1	Canis mesomelas KT448280 1	0,19049582
Zalophus wollebaeki SRR4431565 1	Urva javanica T413 1	0,19049712
Ursus arctos GU573486 5	Mungos mungo/gambianus SRR77048	0,19049805
Mephitis mephitis NC020648 1	Lontra canadensis SRR10409165 1	0,19049837
Ursus arctos pruinosus MG066703 3	Hyaena hyaena NC020669 1	0,19050257
Vulpes lagopus NC026529 3	Ursus spelaeus EU327344 13	0,19050257
Proteles cristata T393 6	Arctocephalus gazella BK010918 1	0,19050279
Ursus maritimus GU573488 Svalbard	Parahyaena brunnea NC038159 15	0,190503
Ursus arctos GU573491 207	Panthera onca KP202264 2	0,19050366
Ursus arctos pruinosus MG066703 3	Felis silvestris lybica KP202275 4	0,19050531
Procyon lotor AB462049 4	Chrysocyon brachyurus NC024172 1	0,19050604
Poecilogale albinucha T602 1	Panthera tigris NC010642 35	0,1905075
Ursus arctos AP012576 6	Mungos mungo MMC7 1	0,19050817
Panthera tigris amoyensis NC014770 2	Bassariscus sumichrasti SRX1099089 1	0,19050829
Paradoxurus hermaphroditus NC03959	Otaria byronia OTAB 1	0,1905091
Otocyon megalotis SAF1 2	Arctictis binturong T605 2	0,19050947
Panthera uncia NC010638 1	Chrysocyon brachyurus NC024172 1	0,19051126
Lycaon pictus NC028427 2	Galictis vittata T412 1	0,19051726
Vulpes lagopus NC026529 3	Urva javanica/auropunctata NC006835	0,1905175
Melogale moschata KP726273 1	Canis mesomelas KT448280 1	0,19051968
Ursus thibetanus thibetanus NC01111	Speothos venaticus C48 2	0,19052003
Ursus spelaeus EU327344 13	Cryptoprocta ferox CFC13 1	0,1905221
Canis aureus KT448274 1	Arctocephalus australis MG023139 1	0,19052297
Nasua nasua NC020647 1	Arctocephalus forsteri NC004023 28	0,1905248
Panthera onca NC022842 1	Helarctos malayanus NC009968 2	0,19052644
Paradoxurus hermaphroditus NLNC 1	Gulo gulo NC009685 3	0,19052656
Vulpes corsac NC023958 1	Neofelis nebulosa NC008450 3	0,19052698
Ursus thibetanus formosanus NC0093	Chrysocyon brachyurus NC024172 1	0,19052748
Neofelis nebulosa NC008450 3	Mustela sibirica NC020637 6	0,1905296
Vulpes corsac NC023958 1	Eumetopias jubatus NC004030 10	0,19053197
Lycalopex sechurae KT448284 1	Eupleres goudotii D128 1	0,19053514
Vulpes corsac NC023958 1	Xenogale naso C07XAR110 1	0,19054668
Vulpes vulpes NC008434 5	Helogale parvula SRR7637809 1	0,190547
Vulpes vulpes NC008434 5	Neophoca cinerea NC008419 1	0,19055097
Galidictis fasciata DM333 1	Ailurus fulgens NC011124 1	0,19055261
Melogale moschata NC020644 1	Cynogale bennetti KY117544 1	0,19055383
Helogale parvula SRR7637809 1	Arctotherium sp NC030174 1	0,19055495
Lontra canadensis SRR10409165 1	Cryptoprocta ferox CFC13 1	0,19055496
Cryptoprocta ferox CFC13 1	Canis latrans NC008093 7	0,19055665
Speothos venaticus C48 2	Potos flavus T414 1	0,19055781
Tremarctos ornatus NC009969 2	Hyaena hyaena NC020669 1	0,19055793
Ursus arctos EU497665 29	Helogale parvula SRR7637809 1	0,19055912
Speothos venaticus C48 2	Bdeogale nigripes GLC15 1	0,19056009
Panthera pardus japonensis KJ866876	Martes americana NC020642 1	0,19056071
Panthera uncia KP202269 1	Martes zibellina NC011579 39	0,19056107
Otocyon megalotis SAF1 2	Arctocephalus pusillus NC008417 1	0,19056113
Ursus maritimus NC003428 31	Galidia elegans D146 1	0,19056205
Urocyon littoralis catalinae KP129018	Felis margarita NC028308 1	0,19056301
Hyaena hyaena NC020669 1	Arctocephalus gazella BK010918 1	0,19056372
Neophoca cinerea NC008419 1	Hyaena hyaena NC020669 1	0,19056396
Mustela sibirica AP017394 11	Crocuta crocuta NC020670 3	0,19056499
Ursus arctos pruinosus MG066703 3	Helogale parvula SRR7637809 1	0,19056551
Tremarctos ornatus NC009969 2	Panthera leo spelaea KX258452 2	0,19056749
Ursus arctos isabellinus 18B5 2	Cryptoprocta ferox CFC13 1	0,19056767
Panthera tigris amoyensis NC014770 2	Mustela sibirica AP017394 11	0,19056828
Ursus arctos pruinosus MG066703 3	Lontra canadensis SRR10409165 1	0,19056888

Salanoia concolor D378 1	Monachus schauinslandi NC008421 1	0,17655905
Viverricula indica KX891745 1	Arctodus simus NC011116 1	0,17656024
Phoca largha NC008430 1	Galidictis fasciata DM333 1	0,17656028
Phoca fasciata NC008428 1	Felis chaus NC028307 1	0,17656229
Profelis aurata NC028299 1	Phoca groenlandica NC008429 54	0,1765635
Phoca vitulina NC001325 1	Galerella sanguinea T378 1	0,17656529
Eumetopias jubatus NC004030 10	Ailurus fulgens styani NC009691 1	0,17658247
Spilogale putorius NC010497 1	Cystophora cristata NC008427 1	0,17658414
Lutra lutra NC011358 9	Callorhinus ursinus NC008415 1	0,17659891
Halichoerus grypus NC001602 2	Catopuma temminckii NC027115 41	0,17659947
Viverra tangalunga MH464792 1	Canis adustus KT448271 1	0,17660083
Halichoerus grypus NC001602 2	Civettictis civetta GLC19 1	0,17660198
Cynictis penicillata T375 1	Canis adustus KT448271 1	0,17660229
Spilogale putorius NC010497 1	Bassariscus sumichrasti SRX1099089 1	0,17660433
Lobodon carcinophaga NC008423 1	Cuon alpinus NC013445 3	0,17660511
Nandinia binotata NC024567 1	Lontra canadensis SRR10409165 1	0,17660576
Viverra tangalunga MH464792 1	Chrotogale owstoni T607 1	0,17660579
Ommatophoca rossii AY377287etc 1	Martes americana NC020642 1	0,17660853
Pusa sibirica NC008432 2	Bdeogale nigripes GLC15 1	0,17660922
Pusa sibirica NC008432 2	Mungos mungo/gambianus SRR7704821 1	0,17660922
Suricata suricatta SSM10 1	Erignathus barbatus NC008426 1	0,17660922
Pusa caspica NC008431 1	Canis latrans NC008093 7	0,17660965
Mustela erminea T305 2	Canis aureus KT448274 1	0,1766109
Pusa caspica NC008431 1	Galidictis fasciata DM333 1	0,17661242
Puma concolor NC016470 22	Meles leucurus NC039173 4	0,17661616
Paradoxurus jerdoni MH464793 1	Erignathus barbatus NC008426 1	0,17661822
Taxidea taxus NC020646 1	Caracal caracal NC028306 1	0,17662144
Phoca largha NC008430 1	Urva semitorquata MH464789 1	0,17662354
Panthera onca KP202264 2	Mirounga angustirostris SRR10331586 1	0,17662981
Phoca fasciata NC008428 1	Hyaena hyaena NC020669 1	0,17663196
Otocyon megalotis SAF1 2	Hydrurga leptonyx NC008425 1	0,17663299
Phoca groenlandica NC008429 54	Mephitis mephitis NC020648 1	0,17663974
Mirounga angustirostris SRR10331586 1	Mephitis mephitis NC020648 1	0,17664073
Mustela frenata NC020640 1	Eumetopias jubatus NC004030 10	0,17664793
Mustela sibirica NC020637 6	Homotherium latidens MF871702 3	0,17665085
Ommatophoca rossii AY377287etc 1	Ailurus fulgens NC011124 1	0,17665341
Phoca vitulina NC001325 1	Urva javanica/auropunctata NC006835 1	0,1766555
Neovison vison NC020641 3	Homotherium latidens MF871702 3	0,17665821
Martes melampus NC009678 1	Arctodus simus NC011116 1	0,17668264
Prionailurus rubiginosus NC028304 2	Mustela frenata NC020640 1	0,17668568
Viverra zibetha T609 1	Pusa sibirica NC008432 2	0,17668574
Phoca vitulina NC001325 1	Eupleres goudotii D128 1	0,17669122
Ursus thibetanus formosanus NC009331 1	Potos flavus T414 1	0,1766969
Panthera onca NC022842 1	Erignathus barbatus NC008426 1	0,17669858
Potos flavus T414 1	Homotherium latidens MF871702 3	0,17669909
Ursus thibetanus thibetanus NC011118 4	Potos flavus T414 1	0,17669977
Phoca vitulina NC001325 1	Panthera uncia KP202269 1	0,17670242
Paradoxurus hermaphroditus NLNC 1	Mirounga angustirostris SRR10331586 1	0,17670296
Paradoxurus hermaphroditus NLNC 1	Neovison vison NC020641 3	0,17670438
Zalophus californianus NC008416 1	Nandinia binotata NC024567 1	0,1767046
Pusa sibirica NC008432 2	Urva javanica/auropunctata NC006835 1	0,17670694
Mustela frenata NC020640 1	Canis lupus familiaris NC002008 1231	0,17671032
Mirounga angustirostris SRR10331586 1	Galictis vittata T412 1	0,17671071
Zalophus californianus NC008416 1	Bassariscus sumichrasti SRX1099089 1	0,17671154
Nandinia binotata NC024567 1	Eumetopias jubatus NC004030 10	0,1767152
Spilogale putorius NC010497 1	Mustela kathiah NC023210 1	0,17672007
Phoca largha NC008430 1	Urva javanica/auropunctata NC006835 1	0,17672276
Prionailurus viverrinus NC028305 1	Ommatophoca rossii AY377287etc 1	0,17672525
Lutra lutra LC050126 1	Callorhinus ursinus NC008415 1	0,17673391
Neofelis nebulosa NC008450 3	Hydrurga leptonyx NC008425 1	0,17673405
Mustela frenata NC020640 1	Arctocephalus forsteri KT693377 17	0,17674215
Monachus monachus NC044972 5	Canis lupus familiaris NC002008 1231	0,17674299
Melogale moschata V0735A 1	Ailurus fulgens NC011124 1	0,17674322
Helogale parvula SRR7637809 1	Erignathus barbatus NC008426 1	0,17674388
Pusa caspica NC008431 1	Cryptoprocta ferox CFC13 1	0,17675519
Taxidea taxus NC020646 1	Puma yagouaroundi NC028311 1	0,17675574
Pusa caspica NC008431 1	Ichneumia albicauda T603 1	0,17675725
Vulpes lagopus NC026529 3	Hydrurga leptonyx NC008425 1	0,17675871
Phoca vitulina NC001325 1	Mungotictis decemlineata NC027828 1	0,17676105
Zalophus wollebaeki SRR4431565 1	Bassariscus sumichrasti SRX1099089 1	0,17676473
Leopardus pardalis T262 1	Canis adustus KT448271 1	0,17676502
Phoca groenlandica NC008429 54	Otocolobus manul NC028323 1	0,1767658
Mustela altaica NC021751 1	Homotherium latidens MF871702 3	0,17677012
Mustela kathiah NC023210 1	Leopardus colocolo NC028314 1	0,17677177
Mephitis mephitis NC020648 1	Meles meles T303 3	0,17677179
Gulo gulo NC009685 3	Callorhinus ursinus NC008415 1	0,17677408
Lutra lutra LC050126 1	Eumetopias jubatus NC004030 10	0,17677638
Hemigalus derbyanus MH464791 1	Canis aureus KT448274 1	0,17679241
Gulo gulo NC009685 3	Arctotherium sp NC030174 1	0,17680082
Lutra lutra LC050126 1	Arctocephalus pusillus NC008417 1	0,176807
Halichoerus grypus NC001602 2	Felis catus NC001700 2	0,17680722
Phoca largha NC008430 1	Conepatus chinga NC042596 1	0,17681117
Tremarctos ornatus NC009969 2	Nandinia binotata NC024567 1	0,17681135
Mirounga leonina NC008422 1	Bassaricyon neblina SRX1097850 1	0,17681141
Martes martes T302 3	Ailurus fulgens NC011124 1	0,17681196
Phocarctos hookeri NC008418 1	Bassariscus sumichrasti SRX1099089 1	0,17681336
Viverricula indica NC025296 2	Canis aureus KT448274 1	0,1768194
Meles leucurus NC039173 4	Genetta servalina NC024568 2	0,17681941
Urva javanica/auropunctata NC006835 1	Halichoerus grypus NC001602 2	0,17682077
Pusa hispida NC 008433 1	Canis latrans NC008093 7	0,17682207
Mustela erminea T305 2	Leopardus tigrinus NC028317 1	0,17682403
Pusa sibirica NC008432 2	Panthera tigris NC010642 35	0,17683202
Monachus schauinslandi NC008421 1	Genetta genetta T297 1	0,17683344
Mephitis mephitis NC020648 1	Arctonyx collaris NC020645 1	0,17683385
Martes americana NC020642 1	Homotherium latidens MF871702 3	0,17683798
Ommatophoca rossii AY377287etc 1	Mustela eversmannii NC028013 1	0,17684111
Phoca largha NC008430 1	Parahyaena brunnea NC038159 15	0,17684208
Mirounga angustirostris SRR10331586 1	Arctictis binturong T605 2	0,17685279
Lontra canadensis SRR10409165 1	Halichoerus grypus NC001602 2	0,17686157
Viverra zibetha T609 1	Halichoerus grypus NC001602 2	0,17686966
Hydrurga leptonyx NC008425 1	Cuon alpinus NC013445 3	0,17686993
Catopuma temminckii NC027115 41	Canis adustus KT448271 1	0,17687173
Mustela frenata NC020640 1	Callorhinus ursinus NC008415 1	0,17687319

Vulpes corsac NC023958 1	Ursus spelaeus NC011112 8	0,19057012
Galictis vittata T412 1	Cynictis penicillata T375 1	0,19057018
Ursus arctos EU497665 29	Prionodon pardicolor NC024569 2	0,1905708
Ursus thibetanus formosanus NC0093:	Atilax paludinosus T606 1	0,19057101
Ursus arctos pruinosus MG066703 3	Panthera pardus japonensis KJ866876	0,19057147
Panthera leo spelaea KX258452 2	Martes martes T302 3	0,19057164
Ursus spelaeus EU327344 13	Prionodon linsang ERR2391707 1	0,19057226
Speothos venaticus C48 2	Pardofelis marmorata NLN3 2	0,19057371
Procyon lotor AB462049 4	Panthera tigris amoyensis NC014770 2	0,19057409
Panthera tigris amoyensis NC014770 2	Arctotherium sp NC030174 1	0,19057535
Arctictis binturong T605 2	Aonyx cinerea NC035814 2	0,19057786
Profelis aurata NC028299 1	Ailuropoda melanoleuca NC009492 5	0,19058037
Cynogale bennetti KY117544 1	Canis lupus chanco NC010340 4	0,19058302
Ursus arctos GU573491 207	Mephitis mephitis NC020648 1	0,19058412
Ursus arctos EU497665 29	Mephitis mephitis NC020648 1	0,19058429
Panthera uncia NC010638 1	Canis mesomelas KT448280 1	0,19058938
Ursus arctos pruinosus MG066703 3	Mephitis mephitis NC020648 1	0,19059119
Ursus spelaeus EU327344 13	Panthera onca NC022842 1	0,19059126
Panthera leo NERO 19	Martes zibellina NC011579 39	0,19059235
Zalophus californianus NC008416 1	Crocuta crocuta NC020670 3	0,19059959
Vulpes zerda KJ603240 1	Eumetopias jubatus NC004030 10	0,19059993
Urocyon littoralis catalinae KP129018	Civettictis civetta NC033378 1	0,19060017
Poecilogale albinucha T602 1	Panthera leo NERO 19	0,19060268
Otocyon megalotis SAF1 2	Neofelis nebulosa NC008450 3	0,19060336
Neofelis nebulosa NC008450 3	Arctodus simus NC011116 1	0,1906039
Ursus arctos pruinosus MG066703 3	Lycalopex sechurae KT448284 1	0,19060434
Spilogale putorius NC010497 1	Neophoca cinerea NC008419 1	0,19060481
Suricata suricatta SSM10 1	Arctonyx collaris NC020645 1	0,1906112
Lontra canadensis SRR10409165 1	Bdeogale nigripes GLC15 1	0,19061221
Melursus ursinus NC009970 2	Canis lupus familiaris NC002008 1231	0,19061247
Mungotictis decemlineata NC027828 1	Conepatus chinga NC042596 1	0,19061396
Vulpes lagopus NC026529 3	Urva javanica T413 1	0,19061437
Vulpes vulpes NC008434 5	Mungos mungo MMC7 1	0,19061461
Vulpes vulpes NC008434 5	Mungos mungo/gambianus SRR77048	0,19061469
Xenogale naso C07XAR110 1	Ailurus fulgens styani NC009691 1	0,19061475
Vulpes vulpes NC008434 5	Tremarctos ornatus NC009969 2	0,19061504
Nyctereutes procyonoides NC013700 3	Melogale moschata V0735A 1	0,19061536
Urocyon littoralis catalinae KP129018	Meles leucurus NC039173 4	0,1906167
Urva javanica T413 1	Bassaricyon neblina SRX1097850 1	0,19061695
Lycalopex sechurae KT448284 1	Helogale parvula SRR7637809 1	0,19061705
Ursus arctos GU573491 207	Canis mesomelas KT448280 1	0,19061783
Urva brachyura KY117547 1	Canis mesomelas KT448280 1	0,19061913
Mungotictis decemlineata NC027828 1	Ailurus fulgens NC011124 1	0,19061972
Ursus spelaeus EU327344 13	Panthera pardus NC010641 5	0,19061984
Suricata suricatta SSM10 1	Canis mesomelas KT448280 1	0,19062258
Speothos venaticus C48 2	Martes melampus NC009678 1	0,19062321
Speothos venaticus C48 2	Melogale moschata KP726273 1	0,19062644
Panthera uncia NC010638 1	Melogale moschata V0735A 1	0,19062726
Ursus arctos isabellinus 18B5 2	Galidia elegans D146 1	0,19062953
Urocyon littoralis catalinae KP129018	Puma concolor NC016470 22	0,19063018
Mephitis mephitis NC020648 1	Arctocephalus australis MG023139 1	0,19063185
Ursus arctos GU573491 207	Hyaena hyaena NC020669 1	0,19063187
Ursus arctos AP012576 6	Conepatus chinga NC042596 1	0,19063191
Panthera tigris NC010642 35	Arctocephalus townsendi NC008420 1	0,19063634
Vulpes vulpes NC008434 5	Melursus ursinus NC009970 2	0,19063781
Ursus maritimus NC003428 31	Prionodon pardicolor NC024569 2	0,19063802
Galictis vittata T412 1	Atilax paludinosus T606 1	0,19063807
Ursus arctos EU497665 29	Felis catus NC001700 2	0,19063966
Procyon lotor AB462049 4	Odobenus rosmarus NC004029 29	0,19064154
Diplogale hosei MH464790 1	Arctocephalus gazella BK010918 1	0,19064248
Ursus maritimus NC003428 31	Lycaon pictus NC028427 2	0,19064309
Ursus arctos GU573486 5	Prionodon pardicolor NC024569 2	0,19064558
Panthera tigris amoyensis NC014770 2	Neovison vison NC020641 3	0,19064662
Zalophus californianus NC008416 1	Vulpes corsac NC023958 1	0,19064711
Panthera leo spelaea KX258452 2	Arctocephalus pusillus NC008417 1	0,19064851
Galerella sanguinea T378 1	Ailuropoda melanoleuca NC009492 5	0,19064915
Ursus americanus JX196366 3	Cynictis penicillata T375 1	0,19065738
Ursus thibetanus thibetanus NC01111	Proteles cristata T393 6	0,19065893
Mephitis mephitis NC020648 1	Lycaon pictus NC028427 2	0,19066007
Urva javanica T413 1	Cuon alpinus NC013445 3	0,19066016
Ursus thibetanus laniger MH281753 2	Panthera onca NC022842 1	0,19066136
Cuon alpinus NC013445 3	Bdeogale nigripes GLC15 1	0,19066621
Xenogale naso C07XAR110 1	Cuon alpinus NC013445 3	0,19066623
Spilogale putorius NC010497 1	Genetta genetta T297 1	0,19067369
Ichneumia albicauda T603 1	Conepatus chinga NC042596 1	0,19067568
Vulpes ferrilata NC027935 1	Lutra lutra NC011358 9	0,1906784
Vulpes vulpes NC008434 5	Galerella sanguinea T378 1	0,1906812
Vulpes corsac NC023958 1	Urva javanica T413 1	0,19068161
Suricata suricatta SSM10 1	Ailurus fulgens NC011124 1	0,19068229
Vulpes corsac NC023958 1	Aonyx cinerea NC035814 2	0,19068372
Ursus thibetanus formosanus NC0093:	Panthera leo NERO 19	0,19068402
Lutrogale perspicillata NC035811 1	Urva javanica T413 1	0,19068435
Lutrogale perspicillata NC035811 1	Galidia elegans D146 1	0,19068512
Vulpes corsac NC023958 1	Procyon lotor AB462046 3	0,1906874
Urocyon cinereoargenteus NC026723 2	Mustela sibirica NC020637 6	0,19068761
Canis anthus NC027956 2	Ailurus fulgens NC011124 1	0,19068836
Urva javanica/auropunctata NC00683	Helarctos malayanus NC009968 2	0,19068851
Urocyon littoralis catalinae KP129018	Taxidea taxus NC020646 1	0,19069165
Vulpes corsac NC023958 1	Ursus arctos isabellinus 18B5 2	0,19069172
Speothos venaticus C48 2	Martes americana NC020642 1	0,19069283
Ursus maritimus NC003428 31	Mungos mungo/gambianus SRR77048	0,19069378
Otocyon megalotis SAF1 2	Mustela kathiah NC023210 1	0,19069414
Phocarctos hookeri NC008418 1	Diplogale hosei MH464790 1	0,19069837
Ursus maritimus GU573488 Svalbard	Canis anthus NC027956 2	0,19069871
Odobenus rosmarus NC004029 29	Nandinia binotata NC024567 1	0,19069979
Melogale moschata NC020644 1	Ichneumia albicauda T603 1	0,19070007
Ursus arctos isabellinus 18B5 2	Panthera uncia KP202269 1	0,19070546
Suricata suricatta SSM10 1	Melursus ursinus NC009970 2	0,19070639
Ursus spelaeus NC011112 8	Xenogale naso C07XAR110 1	0,19070663
Panthera leo spelaea KX258452 2	Meles anakuma NC009677 1	0,19070742
Ursus thibetanus thibetanus NC01111	Nyctereutes procyonoides NC013700 3	0,19070882
Mungotictis decemlineata NC027828 1	Galictis vittata T412 1	0,19071031
Ursus arctos AP012576 6	Helogale parvula SRR7637809 1	0,19071034

Ommatophoca rossii AY377287etc 1	Acinonyx jubatus NC005212 3	0,17687652
Otaria byronia OTAB 1	Lutra sumatrana NC035810 1	0,17687744
Homotherium latidens MF871702 3	Enhydra lutris NC009692 1	0,17688012
Neovison vison NC020641 3	Callorhinus ursinus NC008415 1	0,17688145
Lynx pardinus NC028319 161	Enhydra lutris NC009692 1	0,17688605
Mirounga angustirostris SRR10331586 1	Lontra canadensis SRR10409165 1	0,17688623
Otocyon megalotis SAF1 2	Leptonychotes weddellii NC008424 1	0,17688666
Arctodus simus NC011116 1	Arctocephalus australis MG023139 1	0,1768868
Phoca fasciata NC008428 1	Bassaricyon neblina SRX1097850 1	0,17688729
Paradoxurus jerdoni MH464793 1	Mirounga leonina NC008422 1	0,17688774
Paradoxurus hermaphroditus NLNC 1	Erignathus barbatus NC008426 1	0,17688775
Paradoxurus hermaphroditus NC039591 1	Mirounga leonina NC008422 1	0,17688784
Mustela frenata NC020640 1	Felis margarita NC028308 1	0,17688815
Phoca fasciata NC008428 1	Bdeogale nigripes GLC15 1	0,1768882
Viverra tangalunga MH464792 1	Lynx pardinus NC028319 161	0,17688823
Pusa caspica NC008431 1	Civettictis civetta GLC19 1	0,17688824
Pusa hispida NC 008433 1	Bdeogale nigripes GLC15 1	0,1768886
Otaria byronia OTAB 1	Bassariscus sumichrasti SRX1099089 1	0,17688921
Phoca groenlandica NC008429 54	Paradoxurus hermaphroditus NLNC 1	0,17689045
Pusa caspica NC008431 1	Catopuma temminckii NC027115 41	0,17689073
Prionailurus bengalensis NC028301 12	Potos flavus T414 1	0,17689109
Monachus schauinslandi NC008421 1	Canis latrans NC008093 7	0,17689218
Phoca largha NC008430 1	Mungos mungo/gambianus SRR7704821 1	0,17689227
Pusa caspica NC008431 1	Otocolobus manul NC028323 1	0,17689314
Procyon lotor AB462049 4	Monachus schauinslandi NC008421 1	0,17689432
Taxidea taxus NC020646 1	Leopardus guigna NC028321 1	0,17689551
Erignathus barbatus NC008426 1	Chrysocyon brachyurus NC024172 1	0,17689686
Ictonyx striatus T299 1	Bassaricyon neblina SRX1097850 1	0,1768997
Phoca groenlandica NC008429 54	Caracal caracal NC028306 1	0,1769001
Arctonyx collaris NC020645 1	Arctictis binturong T605 2	0,17690168
Paradoxurus jerdoni MH464793 1	Mirounga angustirostris SRR10331586 1	0,17690502
Mirounga leonina NC008422 1	Lycalopex sechurae KT448284 1	0,17690711
Prionailurus planiceps NC028312 6	Ommatophoca rossii AY377287etc 1	0,17691138
Eumetopias jubatus NC004030 10	Arctonyx collaris NC020645 1	0,17691199
Lutra sumatrana NC035810 1	Eumetopias jubatus NC004030 10	0,17691214
Mustela sibirica AP017394 11	Homotherium latidens MF871702 3	0,17691776
Otocolobus manul NC028323 1	Ommatophoca rossii AY377287etc 1	0,17692399
Ommatophoca rossii AY377287etc 1	Genetta abyssinica MG489822 1	0,1769305
Genetta abyssinica MG489822 1	Canis adustus KT448271 1	0,1769326
Martes flavigula NC012141 3	Arctodus simus NC011116 1	0,17693871
Arctonyx collaris NC020645 1	Arctocephalus australis MG023139 1	0,17694008
Bassariscus sumichrasti SRX1099089 1	Arctocephalus forsteri KT693377 17	0,17694704
Procyon lotor AB462049 4	Nandinia binotata NC024567 1	0,17694855
Mustela kathiah NC023210 1	Leopardus wiedii NC028318 1	0,17695524
Viverricula indica NC025296 2	Arctonyx collaris NC020645 1	0,17695689
Monachus schauinslandi NC008421 1	Urva javanica T413 1	0,17695701
Monachus schauinslandi NC008421 1	Atilax paludinosus T606 1	0,17695753
Viverricula indica KX891745 1	Canis latrans NC008093 7	0,17695807
Zalophus wollebaeki SRR4431565 1	Arctonyx collaris NC020645 1	0,1769582
Phoca fasciata NC008428 1	Galidictis fasciata DM333 1	0,17695867
Viverricula indica NC025296 2	Canis latrans NC008093 7	0,17695971
Phoca vitulina NC001325 1	Urva semitorquata MH464789 1	0,1769603
Phoca groenlandica NC008429 54	Galerella sanguinea T378 1	0,17696525
Panthera tigris amoyensis NC014770 2	Cystophora cristata NC008427 1	0,17696618
Viverricula indica KX891751 1	Ommatophoca rossii AY377287etc 1	0,17697993
Mustela putorius NC020638 4	Eumetopias jubatus NC004030 10	0,17698297
Panthera leo NERO 19	Cystophora cristata NC008427 1	0,17699312
Profelis aurata NC028299 1	Ommatophoca rossii AY377287etc 1	0,17699319
Pusa caspica NC008431 1	Chrotogale owstoni T607 1	0,17700872
Mustela kathiah NC023210 1	Bassaricyon neblina SRX1097850 1	0,17700902
Taxidea taxus NC020646 1	Smilodon populator MF871700 1	0,17701292
Aonyx cinerea NC035814 2	Ailurus fulgens styani NC009691 1	0,177018
Mustela frenata NC020640 1	Arctocephalus pusillus NC008417 1	0,17701821
Phoca fasciata NC008428 1	Urva javanica T413 1	0,17702302
Neovison vison NC020641 3	Chrotogale owstoni T607 1	0,17702332
Panthera uncia NC010638 1	Cystophora cristata NC008427 1	0,1770248
Taxidea taxus NC020646 1	Catopuma temminckii NC027115 41	0,17702565
Mustela erminea T305 2	Catopuma temminckii NC027115 41	0,17702739
Paradoxurus hermaphroditus NC039591 1	Hydrurga leptonyx NC008425 1	0,17702918
Pusa caspica NC008431 1	Ailuropoda melanoleuca NC009492 5	0,17703204
Galictis vittata T412 1	Ailurus fulgens styani NC009691 1	0,17703207
Phoca vitulina NC001325 1	Civettictis civetta GLC19 1	0,1770359
Ommatophoca rossii AY377287etc 1	Lynx canadensis NC028313 1	0,17704026
Pusa hispida NC 008433 1	Panthera tigris amoyensis NC014770 2	0,17704561
Diplogale hosei MH464790 1	Cystophora cristata NC008427 1	0,17704853
Taxidea taxus NC020646 1	Chrotogale owstoni T607 1	0,17706503
Mungotictis decemlineata NC027828 1	Halichoerus grypus NC001602 2	0,17707126
Chrotogale owstoni T607 1	Arctonyx collaris NC020645 1	0,17707229
Pusa hispida NC 008433 1	Panthera pardus NC010641 5	0,17707498
Mustela frenata NC020640 1	Arctocephalus townsendi NC008420 1	0,17707814
Pusa sibirica NC008432 2	Canis latrans NC008093 7	0,17708117
Bassariscus sumichrasti SRX1099089 1	Arctocephalus australis MG023139 1	0,17708161
Neophoca cinerea NC008419 1	Mustela frenata NC020640 1	0,17708246
Mungotictis decemlineata NC027828 1	Erignathus barbatus NC008426 1	0,17708369
Arctonyx collaris NC020645 1	Arctocephalus forsteri NC004023 28	0,17708571
Mustela kathiah NC023210 1	Leopardus pardalis T262 1	0,17708938
Prionailurus bengalensis NC028301 12	Mustela kathiah NC023210 1	0,17709124
Taxidea taxus NC020646 1	Catopuma badia NC028300 1	0,17709266
Pusa caspica NC008431 1	Panthera uncia KP202269 1	0,1770933
Viverricula indica NC025296 2	Mustela erminea T305 2	0,1770958
Taxidea taxus NC020646 1	Leopardus wiedii NC028318 1	0,17709664
Proteles cristata T393 6	Cystophora cristata NC008427 1	0,1770983
Genetta servalina NC024568 2	Arctodus simus NC011116 1	0,17709965
Ursus thibetanus laniger MH281753 2	Potos flavus T414 1	0,17710163
Mirounga angustirostris SRR10331586 1	Felis margarita NC028308 1	0,17710301
Ursus spelaeus EU327344 13	Potos flavus T414 1	0,1771034
Zalophus californianus NC008416 1	Arctotherium sp NC030174 1	0,17710452
Phoca largha NC008430 1	Ichneumia albicauda T603 1	0,17710724
Phoca vitulina NC001325 1	Caracal caracal NC028306 1	0,17710774
Panthera onca NC022842 1	Monachus schauinslandi NC008421 1	0,17711377
Ursus thibetanus mupinensis NC008753 2	Ommatophoca rossii AY377287etc 1	0,17711411
Hemigalus derbyanus MH464791 1	Conepatus chinga NC042596 1	0,17711842
Homotherium latidens MF871702 3	Arctodus simus NC011116 1	0,17713937

Ursus arctos GU573486 5	Parahyaena brunnea NC038159 15	0,1907109
Ursus thibetanus mupinensis NC00875	Speothos venaticus C48 2	0,19071105
Ursus arctos AP012576 6	Lontra canadensis SRR10409165 1	0,19071366
Puma concolor NC016470 22	Mephitis mephitis NC020648 1	0,19071409
Puma yagouaroundi NC028311 1	Odobenus rosmarus NC004029 29	0,19071645
Ursus americanus JX196366 3	Lycalopex sechurae KT448284 1	0,19071724
Mephitis mephitis NC020648 1	Lynx lynx NC027083 4	0,19071823
Ursus arctos AP012576 6	Felis silvestris lybica KP202275 4	0,19072327
Lycaon pictus NC028427 2	Ailuropoda melanoleuca NC009492 5	0,19072402
Ursus spelaeus NC011112 8	Panthera tigris NC010642 35	0,19072625
Ursus americanus JX196366 3	Bassaricyon neblina SRX1097850 1	0,19073011
Martes pennanti NC020664 16	Diplogale hosei MH464790 1	0,19073038
Spilogale putorius NC010497 1	Prionailurus rubiginosus NC028304 2	0,19073594
Spilogale putorius NC010497 1	Profelis aurata NC028299 1	0,19073617
Spilogale putorius NC010497 1	Canis mesomelas KT448280 1	0,19073817
Spilogale putorius NC010497 1	Catopuma temminckii NC027115 41	0,19074209
Ursus maritimus NC003428 31	Lycalopex sechurae KT448284 1	0,19074288
Suricata suricatta SSM10 1	Meles leucurus NC039173 4	0,19074488
Procyon lotor AB462046 3	Mellivora capensis T370 1	0,19074556
Eupleres goudotii D128 1	Canis anthus NC027956 2	0,19074598
Vulpes lagopus NC026529 3	Melogale moschata KP726273 1	0,19074854
Vulpes lagopus NC026529 3	Prionodon linsang ERR2391707 1	0,19074872
Vulpes lagopus NC026529 3	Xenogale naso C07XAR110 1	0,19074887
Ursus arctos GU573486 5	Lycalopex sechurae KT448284 1	0,19074908
Vulpes lagopus NC026529 3	Helogale parvula SRR7637809 1	0,1907493
Vulpes ferrilata NC027935 1	Atilax paludinosus T606 1	0,19074945
Neophoca cinerea NC008419 1	Helogale parvula SRR7637809 1	0,19075005
Vulpes zerda KJ603240 1	Mungos mungo MMC7 1	0,19075172
Vulpes lagopus NC026529 3	Mustela eversmannii NC028013 1	0,19075209
Procyon lotor AB462049 4	Galerella sanguinea T378 1	0,19075245
Paradoxurus hermaphroditus NLNC 1	Arctocephalus gazella BK010918 1	0,19075364
Urocyon littoralis catalinae KP129018	Mustela sibirica NC020637 6	0,19075502
Tremarctos ornatus NC009969 2	Canis anthus NC027956 2	0,19075561
Hyaena hyaena NC020669 1	Canis latrans NC008093 7	0,19076057
Ursus maritimus GU573488 Svalbard	Bdeogale nigripes GLC15 1	0,19076113
Procyon lotor AB462046 3	Cryptoprocta ferox CFC13 1	0,19076262
Parahyaena brunnea NC038159 15	Bassaricyon neblina SRX1097850 1	0,19076386
Ursus arctos EU497665 29	Galidia elegans D146 1	0,19076422
Ursus maritimus NC003428 31	Mungotictis decemlineata NC027828 1	0,19076423
Urocyon littoralis catalinae KP129018	Pardofelis marmorata NLN3 2	0,19076459
Urocyon cinereoargenteus NC026723 2	Pardofelis marmorata NLN3 2	0,19076468
Ursus maritimus GU573488 Svalbard	Galidictis fasciata DM333 1	0,19076473
Ursus arctos pruinosus MG066703 3	Canis latrans NC008093 7	0,19076616
Nyctereutes procyonoides NC013700 3	Diplogale hosei MH464790 1	0,19076815
Helarctos malayanus NC009968 2	Cynogale bennetti KY117544 1	0,19076994
Panthera pardus japonensis KJ866876	Otaria byronia OTAB 1	0,19077002
Panthera tigris amoyensis NC014770 2	Mustela eversmannii NC028013 1	0,19077027
Ursus spelaeus NC011112 8	Urva semitorquata MH464789 1	0,19077174
Ursus thibetanus formosanus NC0093	Nyctereutes procyonoides NC013700 3	0,19077374
Ursus spelaeus EU327344 13	Bdeogale nigripes GLC15 1	0,19077399
Urva javanica T413 1	Helarctos malayanus NC009968 2	0,19077525
Gulo gulo NC009685 3	Cynogale bennetti KY117544 1	0,1907782
Zalophus californianus NC008416 1	Canis anthus NC027956 2	0,19078161
Canis lupus familiaris NC002008 1231	Arctocephalus gazella BK010918 1	0,19078517
Panthera pardus japonensis KJ866876	Helarctos malayanus NC009968 2	0,19078523
Galictis vittata T412 1	Cryptoprocta ferox CFC13 1	0,19078588
Lutra lutra LC050126 1	Cuon alpinus NC013445 3	0,19078636
Ursus arctos AP012576 6	Prionodon pardicolor NC024569 2	0,19078709
Ursus americanus JX196366 3	Canis mesomelas KT448280 1	0,19079259
Galictis vittata T412 1	Ailuropoda melanoleuca NC009492 5	0,19079277
Vulpes zerda KJ603240 1	Panthera leo NERO 19	0,19079304
Vulpes vulpes NC008434 5	Neofelis nebulosa NC008450 3	0,19079612
Helogale parvula SRR7637809 1	Arctocephalus forsteri NC004023 28	0,19080301
Martes melampus NC009678 1	Diplogale hosei MH464790 1	0,19080482
Ursus thibetanus laniger MH281753 2	Odobenus rosmarus NC004029 29	0,19080538
Zalophus californianus NC008416 1	Panthera leo spelaea KX258452 2	0,19081034
Spilogale putorius NC010497 1	Arctocephalus pusillus NC008417 1	0,19081302
Salanoia concolor D378 1	Conepatus chinga NC042596 1	0,19081596
Vulpes ferrilata NC027935 1	Martes zibellina NC011579 39	0,19081606
Ursus arctos AP012576 6	Diplogale hosei MH464790 1	0,19081636
Nasua nasua NC020647 1	Crossarchus platycephalus C7R66 1	0,19081666
Ursus arctos GU573486 5	Panthera pardus NC010641 5	0,1908185
Ursus thibetanus laniger MH281753 2	Cuon alpinus NC013445 3	0,19081891
Poecilogale albinucha T602 1	Cynogale bennetti KY117544 1	0,19082209
Otocyon megalotis SAF1 2	Helogale parvula SRR7637809 1	0,19082509
Poecilogale albinucha T602 1	Eupleres goudotii D128 1	0,19082586
Poecilogale albinucha T602 1	Xenogale naso C07XAR110 1	0,19082655
Otocyon megalotis SAF1 2	Lontra canadensis SRR10409165 1	0,1908278
Panthera uncia NC010638 1	Mustela itatsi NC034330 19	0,19082817
Ursus arctos GU573491 207	Mungos mungo MMC7 1	0,1908282
Ursus maritimus GU573488 Svalbard	Xenogale naso C07XAR110 1	0,19082826
Speothos venaticus C48 2	Crossarchus platycephalus C7R66 1	0,19082868
Otocolobus manul NC028323 1	Lontra canadensis SRR10409165 1	0,19083242
Urocyon cinereoargenteus NC026723 2	Caracal caracal NC028306 1	0,19083321
Ursus arctos EU497665 29	Canis anthus NC027956 2	0,19083354
Arctocephalus australis MG023139 1	Arctictis binturong T605 2	0,19083652
Ursus thibetanus formosanus NC0093	Urva semitorquata MH464789 1	0,19083755
Panthera tigris amoyensis NC014770 2	Mustela sibirica NC020637 6	0,19083779
Ursus maritimus GU573488 Svalbard	Paradoxurus hermaphroditus NLNC 1	0,1908378
Potos flavus T414 1	Crocuta crocuta NC020670 3	0,19083816
Otaria byronia OTAB 1	Lycaon pictus NC028427 2	0,19083883
Vulpes vulpes NC008434 5	Ursus spelaeus NC011112 8	0,19083937
Zalophus wollebaeki SRR4431565 1	Lycalopex sechurae KT448284 1	0,19084047
Tremarctos ornatus NC009969 2	Panthera onca NC022842 1	0,19084144
Ursus arctos EU497665 29	Felis nigripes NC028309 1	0,19084196
Vulpes corsac NC023958 1	Mephitis mephitis NC020648 1	0,19084206
Panthera uncia NC010638 1	Lycaon pictus NC028427 2	0,19084264
Panthera tigris NC010642 35	Arctodus simus NC011116 1	0,19084628
Lutrogale perspicillata NC035811 1	Acinonyx jubatus NC005212 3	0,19085433
Zalophus californianus NC008416 1	Galidia elegans D146 1	0,19085588
Ursus arctos AP012576 6	Felis catus NC001700 2	0,19085763
Urocyon cinereoargenteus NC026723 2	Civettictis civetta NC033378 1	0,19086961
Otocyon megalotis SAF1 2	Urva javanica/auropunctata NC006835	0,19087128

Nasua nasua NC020647 1	Martes americana NC020642 1	0,17714566
Bassariscus sumichrasti SRX1099089 1	Arctocephalus gazella BK010918 1	0,17714976
Pusa caspica NC008431 1	Mungos mungo MMC7 1	0,17714979
Lynx lynx NC027083 4	Lutra lutra LC050126 1	0,17715535
Viverricula indica NC025296 2	Mustela frenata NC020640 1	0,17715679
Mirounga angustirostris SRR10331586 1	Atilax paludinosus T606 1	0,17715976
Zalophus wollebaeki SRR4431565 1	Nandinia binotata NC024567 1	0,1771604
Taxidea taxus NC020646 1	Puma concolor NC016470 22	0,17716135
Pusa sibirica NC008432 2	Profelis aurata NC028299 1	0,17716224
Pusa caspica NC008431 1	Caracal caracal NC028306 1	0,17716232
Pusa caspica NC008431 1	Hyaena hyaena NC020669 1	0,1771632
Mirounga angustirostris SRR10331586 1	Felis catus NC001700 2	0,1771695
Phoca largha NC008430 1	Caracal caracal NC028306 1	0,17717522
Panthera leo spelaea KX258452 2	Lobodon carcinophaga NC008423 1	0,17718355
Martes flavigula NC012141 3	Callorhinus ursinus NC008415 1	0,17718591
Galerella sanguinea T378 1	Canis adustus KT448271 1	0,17718939
Monachus schauinslandi NC008421 1	Canis lupus familiaris NC002008 1231	0,1771915
Spilogale putorius NC010497 1	Phoca groenlandica NC008429 54	0,17720063
Cystophora cristata NC008427 1	Conepatus chinga NC042596 1	0,17720565
Melogale moschata V0735A 1	Bassaricyon neblina SRX1097850 1	0,17721134
Pardofelis marmorata NLN3 2	Halichoerus grypus NC001602 2	0,17721179
Mephitis mephitis NC020648 1	Halichoerus grypus NC001602 2	0,17721334
Martes martes T302 3	Callorhinus ursinus NC008415 1	0,17721617
Meles meles T303 3	Lynx lynx NC027083 4	0,17722057
Viverricula indica KX891751 1	Arctonyx collaris NC020645 1	0,17722385
Leptailurus serval NC028316 1	Arctonyx collaris NC020645 1	0,17722388
Prionailurus rubiginosus NC028304 2	Mustela kathiah NC023210 1	0,17722577
Viverricula indica KX891751 1	Canis latrans NC008093 7	0,17722781
Neophoca cinerea NC008419 1	Arctodus simus NC011116 1	0,17722784
Mustela erminea T305 2	Leptailurus serval NC028316 1	0,17722884
Phoca largha NC008430 1	Atilax paludinosus T606 1	0,17722926
Phoca vitulina NC001325 1	Atilax paludinosus T606 1	0,17722933
Vulpes lagopus NC026529 3	Lobodon carcinophaga NC008423 1	0,17723018
Pusa sibirica NC008432 2	Galictis vittata T412 1	0,17723756
Viverricula indica KX891745 1	Bassariscus sumichrasti SRX1099089 1	0,17723772
Martes foina NC020643 1	Arctodus simus NC011116 1	0,17723949
Pusa hispida NC 008433 1	Panthera uncia NC010638 1	0,17723958
Mephitis mephitis NC020648 1	Meles anakuma NC009677 1	0,17724103
Ursus thibetanus thibetanus NC011118 4	Meles leucurus NC039173 4	0,17724161
Ictonyx striatus T299 1	Homotherium latidens MF871702 3	0,17725773
Pardofelis marmorata NLN3 2	Monachus monachus NC044972 5	0,177259
Pusa sibirica NC008432 2	Panthera leo NERO 19	0,17726016
Pusa caspica NC008431 1	Panthera leo NERO 19	0,17726067
Salanoia concolor D378 1	Monachus monachus NC044972 5	0,17726296
Viverricula indica NC025296 2	Canis lupus chanco NC010340 4	0,17726666
Zalophus californianus NC008416 1	Helarctos malayanus NC009968 2	0,17726848
Spilogale putorius NC010497 1	Canis adustus KT448271 1	0,17727056
Mirounga leonina NC008422 1	Canis anthus NC027956 2	0,17728481
Lutra lutra LC050126 1	Arctotherium sp NC030174 1	0,17728807
Viverricula indica KX891751 1	Canis aureus KT448274 1	0,17729186
Mustela nigripes NC024942 1	Leopardus pardalis NC028315 1	0,17729314
Ursus thibetanus mupinensis NC008753 2	Potos flavus T414 1	0,17729382
Phoca groenlandica NC008429 54	Cryptoprocta ferox CFC13 1	0,17729446
Prionailurus planiceps KY682741 4	Mustela erminea T305 2	0,17729513
Prionailurus bengalensis CKM45 20	Mustela frenata NC020640 1	0,17729576
Hyaena hyaena NC020669 1	Erignathus barbatus NC008426 1	0,17729643
Pusa sibirica NC008432 2	Crocuta crocuta NC020670 3	0,17730081
Pusa sibirica NC008432 2	Panthera onca NC022842 1	0,17730637
Phoca fasciata NC008428 1	Diplogale hosei MH464790 1	0,17730789
Profelis aurata NC028299 1	Phoca largha NC008430 1	0,17730969
Ursus spelaeus NC011112 8	Meles meles T303 3	0,17730969
Phoca vitulina NC001325 1	Parahyaena brunnea NC038159 15	0,17731363
Neovison vison NC020641 3	Arctictis binturong T605 2	0,17731817
Paradoxurus jerdoni MH464793 1	Monachus monachus NC044972 5	0,17732072
Viverricula indica NC025296 2	Canis lupus familiaris NC002008 1231	0,17732462
Procyon lotor AB462046 3	Ommatophoca rossii AY377287etc 1	0,1773337
Mustela putorius NC020638 4	Hemigalus derbyanus MH464791 1	0,17733532
Pusa sibirica NC008432 2	Conepatus chinga NC042596 1	0,17733859
Canis adustus KT448271 1	Arctodus simus NC011116 1	0,17733879
Mustela frenata NC020640 1	Chrotogale owstoni T607 1	0,17734009
Profelis aurata NC028299 1	Halichoerus grypus NC001602 2	0,17734419
Ommatophoca rossii AY377287etc 1	Melogale moschata NC020644 1	0,17734675
Meles leucurus NC039173 4	Genetta abyssinica MG489822 1	0,17735232
Viverricula indica KX891745 1	Arctonyx collaris NC020645 1	0,17735814
Phoca groenlandica NC008429 54	Bassaricyon neblina SRX1097850 1	0,17735918
Mustela frenata NC020640 1	Leopardus jacobita NC028322 1	0,17735952
Ursus arctos isabellinus 18B5 2	Potos flavus T414 1	0,17735954
Phoca fasciata NC008428 1	Cynictis penicillata T375 1	0,17735963
Mustela frenata NC020640 1	Arctotherium sp NC030174 1	0,17735991
Martes americana NC020642 1	Lynx pardinus NC028319 161	0,17736204
Suricata suricatta SSM10 1	Mirounga angustirostris SRR10331586 1	0,17736258
Prionailurus planiceps KY682741 4	Ommatophoca rossii AY377287etc 1	0,17736261
Taxidea taxus NC020646 1	Prionailurus viverrinus NC028305 1	0,17736481
Viverra tangalunga MH464792 1	Arctodus simus NC011116 1	0,17736585
Taxidea taxus NC020646 1	Prionailurus bengalensis CKM45 20	0,17736596
Viverricula indica KX891751 1	Arctodus simus NC011116 1	0,17736806
Panthera onca NC022842 1	Mirounga leonina NC008422 1	0,17736948
Ursus thibetanus laniger MH281753 2	Nandinia binotata NC024567 1	0,17737241
Panthera uncia NC010638 1	Hydrurga leptonyx NC008425 1	0,17737243
Mustela frenata NC020640 1	Acinonyx jubatus NC005212 3	0,17737408
Phoca groenlandica NC008429 54	Galictis vittata T412 1	0,17738006
Phoca fasciata NC008428 1	Galictis vittata T412 1	0,17738022
Lutra lutra NC011358 9	Eumetopias jubatus NC004030 10	0,17738205
Mustela sibirica AP017394 11	Eumetopias jubatus NC004030 10	0,17738748
Mustela itatsi NC034330 19	Eumetopias jubatus NC004030 10	0,17738764
Halichoerus grypus NC001602 2	Cynictis penicillata T375 1	0,17739649
Panthera pardus NC010641 5	Erignathus barbatus NC008426 1	0,17739831
Ommatophoca rossii AY377287etc 1	Catopuma badia NC028300 1	0,17739856
Meles anakuma NC009677 1	Hemigalus derbyanus MH464791 1	0,17740107
Ichneumia albicauda T603 1	Halichoerus grypus NC001602 2	0,1774102
Nasua nasua NC020647 1	Mustela frenata NC020640 1	0,17741215
Pusa sibirica NC008432 2	Chrotogale owstoni T607 1	0,17741284
Martes melampus NC009678 1	Arctocephalus pusillus NC008417 1	0,17741299

Eumetopias jubatus NC004030 10	Canis mesomelas KT448280 1	0,19087279
Neofelis nebulosa NC008450 3	Mustela altaica NC021751 1	0,19087307
Panthera pardus NC010641 5	Otaria byronia OTAB 1	0,19087677
Proteles cristata T393 6	Gulo gulo NC009685 3	0,19088081
Galidictis fasciata DM333 1	Conepatus chinga NC042596 1	0,19088209
Ursus thibetanus formosanus NC0093	Cuon alpinus NC013445 3	0,1908828
Zalophus californianus NC008416 1	Canis lupus familiaris NC002008 1231	0,19088362
Canis latrans NC008093 7	Arctocephalus gazella BK010918 1	0,19088475
Bdeogale nigripes GLC15 1	Bassaricyon neblina SRX1097850 1	0,19088535
Zalophus wollebaeki SRR4431565 1	Panthera pardus NC010641 5	0,19088545
Lutrogale perspicillata NC035811 1	Xenogale naso C07XAR110 1	0,19088624
Canis latrans NC008093 7	Aonyx cinerea NC035814 2	0,19088694
Procyon lotor AB462049 4	Bdeogale nigripes GLC15 1	0,19088748
Vulpes vulpes NC008434 5	Arctonyx collaris NC020645 1	0,19088785
Vulpes lagopus NC026529 3	Viverra tangalunga MH464792 1	0,19088797
Viverra tangalunga MH464792 1	Galidia elegans D146 1	0,19088806
Procyon lotor AB462049 4	Urva brachyura KY117547 1	0,19088855
Meles anakuma NC009677 1	Cryptoprocta ferox CFC13 1	0,19089343
Panthera uncia NC010638 1	Martes americana NC020642 1	0,19089711
Tremarctos ornatus NC009969 2	Panthera uncia KP202269 1	0,19089799
Panthera pardus japonensis KJ866876	Lontra canadensis SRR10409165 1	0,19089894
Urocyon littoralis catalinae KP129018	Felis catus NC001700 2	0,19089913
Parahyaena brunnea NC038159 15	Canis anthus NC027956 2	0,19090134
Mustela sibirica NC020637 6	Crocuta crocuta NC020670 3	0,19090137
Mustela itatsi NC034330 19	Crocuta crocuta NC020670 3	0,19090275
Ursus maritimus NC003428 31	Paradoxurus hermaphroditus NLNC 1	0,19090509
Panthera tigris NC010642 35	Mustela eversmannii NC028013 1	0,19090511
Ursus arctos isabellinus 18B5 2	Paradoxurus hermaphroditus NC03959	0,1909052
Ursus arctos AP012576 6	Canis mesomelas KT448280 1	0,19090603
Vulpes vulpes NC008434 5	Ursus thibetanus laniger MH281753 2	0,1909067
Urva brachyura KY117547 1	Galictis vittata T412 1	0,19090694
Leptailurus serval NC028316 1	Bassaricyon neblina SRX1097850 1	0,19090737
Ursus thibetanus laniger MH281753 2	Suricata suricatta SSM10 1	0,19090776
Speothos venaticus C48 2	Paguma larvata PDDS11 2	0,19090864
Ursus maritimus NC003428 31	Pardofelis marmorata NLN3 2	0,1909087
Ursus arctos EU497665 29	Otocolobus manul NC028323 1	0,19091068
Zalophus californianus NC008416 1	Tapirus terrestris T358	0,19091091
Zalophus wollebaeki SRR4431565 1	Lycaon pictus NC028427 2	0,19091256
Zalophus wollebaeki SRR4431565 1	Panthera pardus japonensis KJ866876	0,19091307
Panthera pardus japonensis KJ866876	Ailuropoda melanoleuca NC009492 5	0,19091344
Genetta genetta T297 1	Galictis vittata T412 1	0,19091986
Ursus arctos AP012576 6	Panthera onca KP202264 2	0,19091998
Mephitis mephitis NC020648 1	Leopardus geoffroyi NC028320 1	0,19092196
Vulpes corsac NC023958 1	Ursus americanus JX196366 3	0,19093093
Vulpes ferrilata NC027935 1	Gulo gulo NC009685 3	0,1909312
Melogale moschata V0735A 1	Cuon alpinus NC013445 3	0,19093211
Spilogale putorius NC010497 1	Felis silvestris lybica KP202275 4	0,19093661
Ursus arctos pruinosus MG066703 3	Panthera leo NERO 19	0,19094324
Urocyon cinereoargenteus NC026723 2	Genetta abyssinica MG489822 1	0,19094864
Panthera pardus NC010641 5	Ailuropoda melanoleuca NC009492 5	0,1909506
Vulpes ferrilata NC027935 1	Meles meles T303 3	0,19095188
Vulpes corsac NC023958 1	Lutra sumatrana NC035810 1	0,19095273
Vulpes zerda KJ603240 1	Mungos mungo/gambianus SRR77048	0,19095312
Mungotictis decemlineata NC027828 1	Lycalopex sechurae KT448284 1	0,19095315
Panthera leo NERO 19	Galictis vittata T412 1	0,19095446
Vulpes lagopus NC026529 3	Arctonyx collaris NC020645 1	0,19095597
Urocyon cinereoargenteus NC026723 2	Mustela frenata NC020640 1	0,19095656
Panthera uncia KP202269 1	Tapirus terrestris T358	0,1909577
Melogale moschata V0735A 1	Galerella sanguinea T378 1	0,19095795
Viverricula indica KX891751 1	Lontra canadensis SRR10409165 1	0,19095883
Poecilogale albinucha T602 1	Bdeogale nigripes GLC15 1	0,19096078
Vulpes zerda KJ603240 1	Paradoxurus jerdoni MH464793 1	0,19096097
Neophoca cinerea NC008419 1	Diplogale hosei MH464790 1	0,19096133
Vulpes zerda KJ603240 1	Ursus thibetanus mupinensis NC00875	0,19096234
Vulpes zerda KJ603240 1	Hyaena hyaena NC020669 1	0,19096254
Ursus maritimus GU573488 Svalbard	Mungotictis decemlineata NC027828 1	0,19096631
Hyaena hyaena NC020669 1	Bassaricyon neblina SRX1097850 1	0,19096634
Ursus arctos isabellinus 18B5 2	Mungotictis decemlineata NC027828 1	0,1909664
Ursus arctos EU497665 29	Galidictis fasciata DM333 1	0,19096671
Proteles cristata T393 6	Melogale moschata V0735A 1	0,19096708
Ursus arctos isabellinus 18B5 2	Galidictis fasciata DM333 1	0,19096714
Poecilogale albinucha T602 1	Lycalopex sechurae KT448284 1	0,19096813
Felis silvestris lybica KP202275 4	Bassaricyon neblina SRX1097850 1	0,19097054
Genetta abyssinica MG489822 1	Bassaricyon neblina SRX1097850 1	0,19097155
Mephitis mephitis NC020648 1	Xenogale naso C07XAR110 1	0,19097205
Poecilogale albinucha T602 1	Panthera uncia KP202269 1	0,19097251
Panthera tigris NC010642 35	Mustela sibirica NC020637 6	0,19097252
Odobenus rosmarus NC004029 29	Fossa fossana D350 1	0,19097281
Ursus maritimus NC003428 31	Otocyon megalotis SAF1 2	0,19097306
Eupleres goudotii D128 1	Chrysocyon brachyurus NC024172 1	0,19097355
Panthera pardus NC010641 5	Canis mesomelas KT448280 1	0,19097376
Mephitis mephitis NC020648 1	Cynictis penicillata T375 1	0,19097398
Panthera leo spelaea KX258452 2	Tapirus terrestris T358	0,19097431
Ursus arctos GU573491 207	Galerella sanguinea T378 1	0,19097458
Ursus maritimus GU573488 Svalbard	Galerella sanguinea T378 1	0,19097462
Ursus arctos EU497665 29	Panthera uncia NC010638 1	0,19097561
Ursus spelaeus EU327344 13	Xenogale naso C07XAR110 1	0,1909759
Ursus thibetanus laniger MH281753 2	Nyctereutes procyonoides NC013700 3	0,19097711
Ursus arctos pruinosus MG066703 3	Paradoxurus hermaphroditus NLNC 1	0,19097905
Ursus maritimus GU573488 Svalbard	Lycaon pictus NC028427 2	0,19097994
Canis mesomelas KT448280 1	Arctocephalus gazella BK010918 1	0,1909824
Mephitis mephitis NC020648 1	Felis margarita NC028308 1	0,19098304
Urva javanica/auropunctata NC006835	Bassaricyon neblina SRX1097850 1	0,19098543
Ursus arctos AP012576 6	Panthera pardus japonensis KJ866876	0,19098777
Parahyaena brunnea NC038159 15	Helarctos malayanus NC009968 2	0,19099148
Ursus spelaeus EU327344 13	Panthera tigris NC010642 35	0,19099237
Ursus arctos AP012576 6	Leopardus tigrinus NC028317 1	0,19099337
Panthera tigris amoyensis NC014770 2	Lycalopex sechurae KT448284 1	0,1909935
Cynogale bennetti KY117544 1	Cuon alpinus NC013445 3	0,19099382
Ursus thibetanus formosanus NC0093	Panthera onca NC022842 1	0,19099638
Procyon lotor AB462049 4	Cuon alpinus NC013445 3	0,1910089
Ursus americanus JX196366 3	Panthera uncia KP202269 1	0,19101056
Ursus thibetanus mupinensis NC00875	Cuon alpinus NC013445 3	0,19101056

Tremarctos ornatus NC009969 2	Potos flavus T414 1	0,17741309
Callorhinus ursinus NC008415 1	Arctodus simus NC011116 1	0,17742248
Lynx canadensis NC028313 1	Enhydra lutris NC009692 1	0,17742497
Paradoxurus hermaphroditus NLNC 1	Leptonychotes weddellii NC008424 1	0,17742634
Mustela frenata NC020640 1	Caracal caracal NC028306 1	0,1774265
Phoca fasciata NC008428 1	Canis latrans NC008093 7	0,17742708
Pusa hispida NC 008433 1	Atilax paludinosus T606 1	0,1774275
Mustela frenata NC020640 1	Arctocephalus forsteri NC004023 28	0,17742763
Viverricula indica KX891745 1	Canis anthus NC027956 2	0,17742915
Catopuma badia NC028300 1	Canis aureus KT448274 1	0,17742947
Paradoxurus hermaphroditus NLNC 1	Hydrurga leptonyx NC008425 1	0,17743319
Phoca vitulina NC001325 1	Galidictis fasciata DM333 1	0,17743589
Galictis vittata T412 1	Ailurus fulgens NC011124 1	0,17743601
Phoca groenlandica NC008429 54	Leopardus guigna NC028321 1	0,17744127
Phoca largha NC008430 1	Panthera onca KP202264 2	0,17744324
Pusa caspica NC008431 1	Canis lupus familiaris NC002008 1231	0,17744961
Monachus monachus NC044972 5	Galerella sanguinea T378 1	0,17746446
Mustela erminea T305 2	Canis adustus KT448271 1	0,17746758
Spilogale putorius NC010497 1	Ailurus fulgens styani NC009691 1	0,17746814
Gulo gulo NC009685 3	Eumetopias jubatus NC004030 10	0,17747135
Genetta genetta T297 1	Canis adustus KT448271 1	0,17747244
Fossa fossana D350 1	Arctonyx collaris NC020645 1	0,17748076
Lutra sumatrana NC035810 1	Arctocephalus gazella BK010918 1	0,17748104
Mirounga leonina NC008422 1	Helogale parvula SRR7637809 1	0,17748451
Pusa caspica NC008431 1	Atilax paludinosus T606 1	0,17748451
Pusa sibirica NC008432 2	Atilax paludinosus T606 1	0,17748451
Taxidea taxus NC020646 1	Crossarchus platycephalus C7R66 1	0,17749177
Mirounga leonina NC008422 1	Felis catus NC001700 2	0,17749274
Viverra zibetha T609 1	Potos flavus T414 1	0,17749299
Mustela erminea T305 2	Catopuma badia NC028300 1	0,17749861
Pusa caspica NC008431 1	Profelis aurata NC028299 1	0,17749882
Martes americana NC020642 1	Arctodus simus NC011116 1	0,17749907
Mustela eversmannii NC028013 1	Arctodus simus NC011116 1	0,17749964
Mustela itatsi NC034330 19	Arctodus simus NC011116 1	0,17750058
Martes martes T302 3	Arctodus simus NC011116 1	0,17750315
Procyon lotor AB462049 4	Galictis vittata T412 1	0,17750686
Otocyon megalotis SAF1 2	Lobodon carcinophaga NC008423 1	0,17750844
Martes foina NC020643 1	Lynx lynx NC027083 4	0,17750938
Phoca vitulina NC001325 1	Panthera onca KP202264 2	0,17751038
Caracal caracal NC028306 1	Canis adustus KT448271 1	0,17751156
Potos flavus T414 1	Mephitis mephitis NC020648 1	0,17751206
Phoca fasciata NC008428 1	Panthera onca NC022842 1	0,17751473
Ommatophoca rossii AY377287etc 1	Ictonyx striatus T299 1	0,17752358
Nasua nasua NC020647 1	Martes flavigula NC012141 3	0,17752893
Eumetopias jubatus NC004030 10	Arctotherium sp NC030174 1	0,17753237
Ursus americanus JX196366 3	Meles meles T303 3	0,17753379
Smilodon populator MF871700 1	Arctodus simus NC011116 1	0,17753659
Mustela putorius NC020638 4	Callorhinus ursinus NC008415 1	0,17754592
Erignathus barbatus NC008426 1	Canis aureus KT448274 1	0,17754902
Taxidea taxus NC020646 1	Phocarctos hookeri NC008418 1	0,17755711
Otaria byronia OTAB 1	Arctonyx collaris NC020645 1	0,17755838
Prionailurus bengalensis NC028301 12	Meles leucurus NC039173 4	0,17755975
Paradoxurus jerdoni MH464793 1	Arctonyx collaris NC020645 1	0,17756039
Paguma larvata PDDS11 2	Arctonyx collaris NC020645 1	0,17756043
Pusa hispida NC 008433 1	Urva brachyura KY117547 1	0,17756052
Mirounga leonina NC008422 1	Galerella sanguinea T378 1	0,17756219
Panthera uncia NC010638 1	Erignathus barbatus NC008426 1	0,17756228
Phoca fasciata NC008428 1	Paradoxurus hermaphroditus NLNC 1	0,1775638
Pusa caspica NC008431 1	Panthera onca KP202264 2	0,17756459
Pusa sibirica NC008432 2	Panthera pardus japonensis KJ866876 8	0,17756467
Pusa sibirica NC008432 2	Panthera uncia KP202269 1	0,17756468
Mirounga angustirostris SRR10331586 1	Cynictis penicillata T375 1	0,1775651
Phoca vitulina NC001325 1	Cynictis penicillata T375 1	0,17756544
Homotherium latidens MF871702 3	Arctonyx collaris NC020645 1	0,17757209
Viverricula indica NC025296 2	Arctodus simus NC011116 1	0,17757276
Puma concolor NC016470 22	Neovison vison NC020641 3	0,17757435
Profelis aurata NC028299 1	Phoca vitulina NC001325 1	0,17757891
Meles meles T303 3	Eumetopias jubatus NC004030 10	0,17758608
Mustela nigripes NC024942 1	Eumetopias jubatus NC004030 10	0,17758903
Mirounga angustirostris SRR10331586 1	Canis lupus familiaris NC002008 1231	0,17759906
Mungos mungo MMC7 1	Halichoerus grypus NC001602 2	0,17760089
Lutra lutra LC050126 1	Fossa fossana D350 1	0,17761245
Halichoerus grypus NC001602 2	Genetta genetta T297 1	0,17761341
Meles meles T303 3	Fossa fossana D350 1	0,17761464
Panthera onca KP202264 2	Halichoerus grypus NC001602 2	0,17761664
Prionodon linsang ERR2391707 1	Erignathus barbatus NC008426 1	0,17761917
Pusa caspica NC008431 1	Bdeogale nigripes GLC15 1	0,17761917
Mungos mungo MMC7 1	Canis adustus KT448271 1	0,17761926
Meles leucurus NC039173 4	Arctocephalus pusillus NC008417 1	0,17761987
Vulpes zerda KJ603240 1	Leptonychotes weddellii NC008424 1	0,17762204
Taxidea taxus NC020646 1	Arctocephalus townsendi NC008420 1	0,17762284
Meles leucurus NC039173 4	Lynx lynx NC027083 4	0,17762417
Mustela frenata NC020640 1	Lynx pardinus NC028319 161	0,17762886
Panthera pardus japonensis KJ866876 8	Erignathus barbatus NC008426 1	0,17762956
Lycaon pictus NC028427 2	Erignathus barbatus NC008426 1	0,17763147
Melogale moschata KP726273 1	Leopardus wiedii NC028318 1	0,17763186
Meles meles T303 3	Homotherium latidens MF871702 3	0,17763215
Potos flavus T414 1	Leopardus pardalis NC028315 1	0,17763266
Mustela kathiah NC023210 1	Arctodus simus NC011116 1	0,17763434
Nyctereutes procyonoides NC013700 3	Monachus schauinslandi NC008421 1	0,17763464
Neovison vison NC020641 3	Leopardus pardalis T262 1	0,17764041
Nandinia binotata NC024567 1	Helarctos malayanus NC009968 2	0,17764285
Martes foina NC020643 1	Leopardus pardalis NC028315 1	0,17764588
Phoca vitulina NC001325 1	Ichneumia albicauda T603 1	0,17764597
Martes foina NC020643 1	Leopardus pardalis T262 1	0,17764607
Mustela nigripes NC024942 1	Homotherium latidens MF871702 3	0,17764615
Phoca vitulina NC001325 1	Hyaena hyaena NC020669 1	0,1776485
Hydrurga leptonyx NC008425 1	Crocuta crocuta NC020670 3	0,17765327
Monachus monachus NC044972 5	Canis latrans NC008093 7	0,17765622
Viverricula indica KX891751 1	Canis lupus familiaris NC002008 1231	0,1776602
Nasua nasua NC020647 1	Gulo gulo NC009685 3	0,17766142
Profelis aurata NC028299 1	Canis adustus KT448271 1	0,17767747
Catopuma badia NC028300 1	Canis lupus chanco NC010340 4	0,17767859

Ursus thibetanus mupinensis NC00875	Urva javanica/auropunctata NC006835	0,19101286
Cryptoprocta ferox CFC13 1	Arctocephalus forsteri NC004023 28	0,19101314
Vulpes lagopus NC026529 3	Lutra lutra NC011358 9	0,19101416
Melogale moschata V0735A 1	Urva brachyura KY117547 1	0,19101562
Canis latrans NC008093 7	Arctocephalus forsteri KT693377 17	0,19101815
Vulpes lagopus NC026529 3	Diplogale hosei MH464790 1	0,19101924
Urocyon littoralis catalinae KP129018	Enhydra lutris NC009692 1	0,19101937
Martes flavigula NC012141 3	Crocuta crocuta NC020670 3	0,19102019
Ursus spelaeus EU327344 13	Panthera leo NERO 19	0,19102077
Vulpes zerda KJ603240 1	Galidia elegans D146 1	0,19102164
Ursus thibetanus formosanus NC0093	Urva javanica/auropunctata NC006835	0,19102259
Ursus thibetanus laniger MH281753 2	Panthera leo NERO 19	0,19102263
Urocyon cinereoargenteus NC026723 2	Mustela itatsi NC034330 19	0,19102493
Urocyon cinereoargenteus NC026723 2	Mustela erminea T305 2	0,19102542
Otocyon megalotis SAF1 2	Urva semitorquata MH464789 1	0,19102772
Galerella sanguinea T378 1	Bassaricyon neblina SRX1097850 1	0,19102795
Ursus arctos EU497665 29	Mungos mungo/gambianus SRR77048	0,19103049
Melursus ursinus NC009970 2	Lycalopex sechurae KT448284 1	0,19103281
Mustela altaica NC021751 1	Galidia elegans D146 1	0,19103354
Ursus arctos GU573491 207	Mungotictis decemlineata NC027828 1	0,19103359
Genetta genetta T297 1	Canis mesomelas KT448280 1	0,19103561
Ursus arctos pruinosus MG066703 3	Mungos mungo MMC7 1	0,19103687
Fossa fossana D350 1	Ailuropoda melanoleuca NC009492 5	0,19103956
Ursus arctos GU573486 5	Mungotictis decemlineata NC027828 1	0,19103958
Vulpes zerda KJ603240 1	Ursus thibetanus formosanus NC0093	0,19103985
Speothos venaticus C48 2	Martes martes T302 3	0,1910401
Panthera tigris NC010642 35	Arctocephalus forsteri KT693377 17	0,19104064
Ursus arctos EU497665 29	Otocyon megalotis SAF1 2	0,19104066
Mephitis mephitis NC020648 1	Urva javanica T413 1	0,19104151
Poecilogale albinucha T602 1	Cryptoprocta ferox CFC13 1	0,19104198
Parahyaena brunnea NC038159 15	Otocyon megalotis SAF1 2	0,19104314
Ursus maritimus GU573488 Svalbard	Pardofelis marmorata NLN3 2	0,19104338
Vulpes ferrilata NC027935 1	Mephitis mephitis NC020648 1	0,19104379
Ursus arctos GU573486 5	Paradoxurus hermaphroditus NLNC 1	0,19104625
Parahyaena brunnea NC038159 15	Lycaon pictus NC028427 2	0,19104663
Panthera tigris NC010642 35	Martes pennanti NC020664 16	0,19105038
Zalophus wollebaeki SRR4431565 1	Chrysocyon brachyurus NC024172 1	0,19105405
Ursus arctos AP012576 6	Paradoxurus hermaphroditus NLNC 1	0,1910557
Ursus spelaeus EU327344 13	Prionodon pardicolor NC024569 2	0,19105621
Bassaricyon neblina SRX1097850 1	Acinonyx jubatus NC005212 3	0,1910565
Ursus americanus JX196366 3	Bdeogale nigripes GLC15 1	0,19106102
Panthera tigris NC010642 35	Lycalopex sechurae KT448284 1	0,19106264
Urva javanica/auropunctata NC006835	Arctotherium sp NC030174 1	0,19106625
Tremarctos ornatus NC009969 2	Canis lupus chanco NC010340 4	0,19106701
Ursus americanus JX196366 3	Hyaena hyaena NC020669 1	0,1910689
Zalophus californianus NC008416 1	Chrysocyon brachyurus NC024172 1	0,19107087
Cynogale bennetti KY117544 1	Canis anthus NC027956 2	0,19108345
Panthera leo spelaea KX258452 2	Canis mesomelas KT448280 1	0,19108351
Canis anthus NC027956 2	Arctocephalus forsteri KT693377 17	0,19108583
Vulpes ferrilata NC027935 1	Lutra sumatrana NC035810 1	0,19108807
Vulpes lagopus NC026529 3	Mustela itatsi NC034330 19	0,19108959
Urocyon cinereoargenteus NC026723 2	Bassariscus sumichrasti SRX1099089 1	0,19109058
Ursus americanus JX196366 3	Galictis vittata T412 1	0,19109109
Tremarctos ornatus NC009969 2	Nyctereutes procyonoides NC013700 3	0,19109181
Cryptoprocta ferox CFC13 1	Bassariscus sumichrasti SRX1099089 1	0,19109441
Vulpes ferrilata NC027935 1	Paradoxurus hermaphroditus NLNC 1	0,19109501
Poecilogale albinucha T602 1	Atilax paludinosus T606 1	0,19109623
Speothos venaticus C48 2	Fossa fossana D350 1	0,19109729
Ursus maritimus NC003428 31	Bdeogale nigripes GLC15 1	0,19109778
Otocyon megalotis SAF1 2	Arctonyx collaris NC020645 1	0,1910982
Panthera pardus japonensis KJ866876	Arctocephalus australis MG023139 1	0,19109881
Genetta servalina NC024568 2	Bassaricyon neblina SRX1097850 1	0,19110005
Paradoxurus hermaphroditus NLNC 1	Aonyx cinerea NC035814 2	0,19110067
Phocarctos hookeri NC008418 1	Panthera pardus japonensis KJ866876	0,19110185
Chrysocyon brachyurus NC024172 1	Arctocephalus gazella BK010918 1	0,1911021
Panthera leo spelaea KX258452 2	Martes americana NC020642 1	0,19110385
Speothos venaticus C48 2	Chrotogale owstoni T607 1	0,19110397
Ursus arctos GU573486 5	Bdeogale nigripes GLC15 1	0,19110428
Vulpes vulpes NC008434 5	Ailuropoda melanoleuca NC009492 5	0,1911045
Otaria byronia OTAB 1	Crocuta crocuta NC020670 3	0,1911048
Procyon lotor AB462046 3	Panthera uncia NC010638 1	0,19110492
Panthera uncia NC010638 1	Arctotherium sp NC030174 1	0,19110601
Meles anakuma NC009677 1	Crocuta crocuta NC020670 3	0,19110624
Melursus ursinus NC009970 2	Canis mesomelas KT448280 1	0,19110663
Prionailurus rubiginosus NC028304 2	Bassaricyon neblina SRX1097850 1	0,19110672
Ursus arctos GU573486 5	Galidia elegans D146 1	0,19110686
Neophoca cinerea NC008419 1	Chrysocyon brachyurus NC024172 1	0,19110775
Urva brachyura KY117547 1	Ailuropoda melanoleuca NC009492 5	0,19110778
Lycaon pictus NC028427 2	Hyaena hyaena NC020669 1	0,19110847
Ursus thibetanus thibetanus NC01111	Eupleres goudotii D128 1	0,1911087
Ursus arctos GU573491 207	Parahyaena brunnea NC038159 15	0,19110915
Ursus spelaeus EU327344 13	Cynictis penicillata T375 1	0,19111118
Galidictis fasciata DM333 1	Galictis vittata T412 1	0,19111516
Ursus arctos GU573486 5	Galerella sanguinea T378 1	0,19111551
Vulpes corsac NC023958 1	Diplogale hosei MH464790 1	0,19111598
Mustela altaica NC021751 1	Crocuta crocuta NC020670 3	0,19111656
Lycalopex sechurae KT448284 1	Aonyx cinerea NC035814 2	0,19111666
Ursus spelaeus EU327344 13	Panthera uncia KP202269 1	0,19111954
Ursus arctos EU497665 29	Panthera onca NC022842 1	0,19112042
Spilogale putorius NC010497 1	Arctocephalus townsendi NC008420 1	0,19112477
Ursus spelaeus EU327344 13	Panthera tigris amoyensis NC014770 2	0,19112688
Ursus thibetanus formosanus NC0093	Panthera tigris amoyensis NC014770 2	0,19112836
Neofelis nebulosa NC008450 3	Mustela eversmannii NC028013 1	0,19113532
Vulpes ferrilata NC027935 1	Eumetopias jubatus NC004030 10	0,19113921
Mungotictis decemlineata NC027828 1	Arctocephalus forsteri NC004023 28	0,19114219
Ursus americanus JX196366 3	Felis nigripes NC028309 1	0,19114225
Ursus thibetanus thibetanus NC01111	Odobenus rosmarus NC004029 29	0,19114338
Paradoxurus hermaphroditus NC03959	Lutra lutra NC011358 9	0,19114518
Spilogale putorius NC010497 1	Leopardus geoffroyi NC028320 1	0,19114667
Urocyon littoralis catalinae KP129018	Genetta abyssinica MG489822 1	0,19114757
Lontra canadensis SRR10409165 1	Urva javanica T413 1	0,19115068
Vulpes corsac NC023958 1	Galerella sanguinea T378 1	0,19115265
Phocarctos hookeri NC008418 1	Helogale parvula SRR7637809 1	0,1911529

Suricata suricatta SSM10 1	Pusa sibirica NC008432 2	0,17768651
Lynx rufus NC014456 3	Enhydra lutris NC009692 1	0,17769401
Prionailurus planiceps KY682741 4	Mustela frenata NC020640 1	0,17769593
Phoca fasciata NC008428 1	Helogale parvula SRR7637809 1	0,17769639
Arctodus simus NC011116 1	Arctocephalus gazella BK010918 1	0,17769665
Phoca groenlandica NC008429 54	Mungotictis decemlineata NC027828 1	0,17769907
Mustela frenata NC020640 1	Leopardus geoffroyi NC028320 1	0,17769967
Monachus schauinslandi NC008421 1	Bassaricyon neblina SRX1097850 1	0,17770264
Zalophus californianus NC008416 1	Martes melampus NC009678 1	0,17770973
Martes foina NC020643 1	Lynx canadensis NC028313 1	0,17771022
Neovison vison NC020641 3	Leopardus pardalis NC028315 1	0,17771243
Mephitis mephitis NC020648 1	Meles leucurus NC039173 4	0,17771439
Zalophus californianus NC008416 1	Neovison vison NC020641 3	0,17772593
Eumetopias jubatus NC004030 10	Ailurus fulgens NC011124 1	0,17772774
Phoca fasciata NC008428 1	Panthera leo NERO 19	0,17773849
Nasua nasua NC020647 1	Martes melampus NC009678 1	0,1777468
Lutra sumatrana NC035810 1	Arctocephalus pusillus NC008417 1	0,17775127
Panthera uncia KP202269 1	Halichoerus grypus NC001602 2	0,17775139
Otaria byronia OTAB 1	Lutra lutra LC050126 1	0,17775165
Mustela erminea T305 2	Arctocephalus forsteri KT693377 17	0,17775245
Meles leucurus NC039173 4	Homotherium latidens MF871702 3	0,17775704
Poecilogale albinucha T602 1	Homotherium latidens MF871702 3	0,17775868
Paguma larvata PDDS11 2	Mustela frenata NC020640 1	0,17775874
Taxidea taxus NC020646 1	Mungos mungo MMC7 1	0,17775901
Meles leucurus NC039173 4	Lynx pardinus NC028319 161	0,1777596
Neovison vison NC020641 3	Crossarchus platycephalus C7R66 1	0,1777619
Martes foina NC020643 1	Callorhinus ursinus NC008415 1	0,17776218
Viverricula indica NC025296 2	Mustela kathiah NC023210 1	0,17776647
Mustela sibirica AP017394 11	Arctodus simus NC011116 1	0,1777667
Mustela erminea T305 2	Arctotherium sp NC030174 1	0,17776679
Taxidea taxus NC020646 1	Felis silvestris lybica KP202275 4	0,17776775
Vulpes ferrilata NC027935 1	Lobodon carcinophaga NC008423 1	0,17776953
Pusa sibirica NC008432 2	Panthera tigris amoyensis NC014770 2	0,17777467
Pusa caspica NC008431 1	Panthera tigris NC010642 35	0,17777483
Pusa caspica NC008431 1	Galictis vittata T412 1	0,1777762
Meles leucurus NC039173 4	Leopardus colocolo NC028314 1	0,17777941
Ursus arctos GU573486 5	Mustela erminea T305 2	0,17777967
Erignathus barbatus NC008426 1	Canis lupus familiaris NC002008 1231	0,17778648
Monachus schauinslandi NC008421 1	Galictis vittata T412 1	0,17778924
Phoca largha NC008430 1	Panthera onca NC022842 1	0,17779115
Mirounga leonina NC008422 1	Canis mesomelas KT448280 1	0,17779547
Mustela erminea T305 2	Canis lupus chanco NC010340 4	0,17779726
Mustela kathiah NC023210 1	Canis adustus KT448271 1	0,17779842
Mungos mungo/gambianus SRR7704821 1	Halichoerus grypus NC001602 2	0,17780143
Spilogale putorius NC010497 1	Ailurus fulgens NC011124 1	0,17780462
Mirounga leonina NC008422 1	Cuon alpinus NC013445 3	0,177806
Smilodon populator MF871700 1	Mustela erminea T305 2	0,17780609
Pusa caspica NC008431 1	Conepatus chinga NC042596 1	0,17781016
Mustela kathiah NC023210 1	Callorhinus ursinus NC008415 1	0,17781439
Martes zibellina NC011579 39	Callorhinus ursinus NC008415 1	0,17781755
Mustela erminea T305 2	Arctocephalus townsendi NC008420 1	0,17781895
Phocarctos hookeri NC008418 1	Mustela erminea T305 2	0,17782079
Nandinia binotata NC024567 1	Canis latrans NC008093 7	0,17782222
Paradoxurus hermaphroditus NLNC 1	Mustela frenata NC020640 1	0,17782421
Poecilogale albinucha T602 1	Ommatophoca rossii AY377287etc 1	0,17782477
Aonyx cinerea NC035814 2	Ailurus fulgens NC011124 1	0,17782573
Lutra lutra LC050126 1	Arctodus simus NC011116 1	0,17782891
Monachus schauinslandi NC008421 1	Urva brachyura KY117547 1	0,17783159
Viverra tangalunga MH464792 1	Puma concolor NC016470 22	0,17783189
Zalophus wollebaeki SRR4431565 1	Martes melampus NC009678 1	0,17783208
Pusa caspica NC008431 1	Felis chaus NC028307 1	0,177835
Prionailurus bengalensis CKM45 20	Mustela kathiah NC023210 1	0,17783587
Urocyon littoralis catalinae KP129018 15	Hydrurga leptonyx NC008425 1	0,17783777
Urocyon cinereoargenteus NC026723 21	Hydrurga leptonyx NC008425 1	0,17783782
Ursus thibetanus mupinensis NC008753 2	Arctonyx collaris NC020645 1	0,17783956
Monachus schauinslandi NC008421 1	Canis aureus KT448274 1	0,17784092
Phoca groenlandica NC008429 54	Catopuma temminckii NC027115 41	0,1778425
Paradoxurus hermaphroditus NC039591 1	Mirounga angustirostris SRR10331586 1	0,17784795
Lobodon carcinophaga NC008423 1	Hyaena hyaena NC020669 1	0,17784885
Ommatophoca rossii AY377287etc 1	Martes pennanti NC020664 16	0,17785624
Erignathus barbatus NC008426 1	Canis lupus chanco NC010340 4	0,17785634
Ursus americanus JX196366 3	Potos flavus T414 1	0,17786483
Ommatophoca rossii AY377287etc 1	Caracal caracal NC028306 1	0,1778672
Nandinia binotata NC024567 1	Tapirus terrestris T358	0,17787299
Hemigalus derbyanus MH464791 1	Arctodus simus NC011116 1	0,17787586
Meles anakuma NC009677 1	Callorhinus ursinus NC008415 1	0,17787904
Leptonychotes weddellii NC008424 1	Cynogale bennetti KY117544 1	0,17788021
Pusa caspica NC008431 1	Urva brachyura KY117547 1	0,1778885
Pusa caspica NC008431 1	Urva javanica T413 1	0,1778885
Hyaena hyaena NC020669 1	Halichoerus grypus NC001602 2	0,17788929
Ursus arctos isabellinus 18B5 2	Ommatophoca rossii AY377287etc 1	0,17789268
Mungotictis decemlineata NC027828 1	Mirounga leonina NC008422 1	0,17789274
Zalophus californianus NC008416 1	Martes flavigula NC012141 3	0,17789291
Martes martes T302 3	Arctocephalus pusillus NC008417 1	0,17789404
Neovison vison NC020641 3	Arctocephalus australis MG023139 1	0,1778942
Taxidea taxus NC020646 1	Atilax paludinosus T606 1	0,17789549
Puma yagouaroundi NC028311 1	Meles leucurus NC039173 4	0,1778959
Otaria byronia OTAB 1	Mustela frenata NC020640 1	0,17789702
Zalophus wollebaeki SRR4431565 1	Arctotherium sp NC030174 1	0,177898
Pusa caspica NC008431 1	Lycaon pictus NC028427 2	0,17789906
Prionailurus planiceps NC028312 6	Mustela erminea T305 2	0,17790101
Martes zibellina NC011579 39	Lynx pardinus NC028319 161	0,17790124
Arctonyx collaris NC020645 1	Arctodus simus NC011116 1	0,17790239
Homotherium latidens MF871702 3	Tapirus terrestris T358	0,17790779
Neovison vison NC020641 3	Leopardus wiedii NC028318 1	0,17791028
Procyon lotor AB462046 3	Galictis vittata T412 1	0,17791124
Ursus thibetanus thibetanus NC011118 4	Nandinia binotata NC024567 1	0,17791361
Phoca largha NC008430 1	Panthera uncia KP202269 1	0,17791478
Viverra tangalunga MH464792 1	Acinonyx jubatus NC005212 3	0,1779152
Mirounga angustirostris SRR10331586 1	Lycaon pictus NC028427 2	0,17791803
Phoca fasciata NC008428 1	Chrysocyon brachyurus NC024172 1	0,17792268
Phoca largha NC008430 1	Galictis vittata T412 1	0,1779238
Martes americana NC020642 1	Eumetopias jubatus NC004030 10	0,17792693

Vulpes corsac NC023958 1	Meles leucurus NC039173 4	0,19115303
Mungos mungo/gambianus SRR77048	Lutrogale perspicillata NC035811 1	0,19115637
Urocyon littoralis catalinae KP129018	Mustela itatsi NC034330 19	0,19115965
Speothos venaticus C48 2	Enhydra lutris NC009692 1	0,19116053
Lycaon pictus NC028427 2	Eupleres goudotii D128 1	0,19116116
Vulpes vulpes NC008434 5	Otaria byronia OTAB 1	0,1911613
Otocyon megalotis SAF1 2	Cynictis penicillata T375 1	0,19116187
Phocarctos hookeri NC008418 1	Galerella sanguinea T378 1	0,19116333
Urocyon cinereoargenteus NC026723 2	Melogale moschata NC020644 1	0,19116446
Urocyon littoralis catalinae KP129018	Melogale moschata NC020644 1	0,19116446
Ursus maritimus NC003428 31	Xenogale naso C07XAR110 1	0,19116494
Vulpes ferrilata NC027935 1	Ursus thibetanus mupinensis NC00875	0,19116504
Speothos venaticus C48 2	Mungos mungo MMC7 1	0,19116546
Procyon lotor AB462046 3	Otocyon megalotis SAF1 2	0,191166
Prionodon pardicolor NC024569 2	Aonyx cinerea NC035814 2	0,19116688
Panthera uncia NC010638 1	Lontra canadensis SRR10409165 1	0,19116804
Ursus arctos EU497665 29	Mungotictis decemlineata NC027828 1	0,1911684
Panthera uncia KP202269 1	Neophoca cinerea NC008419 1	0,19116883
Melogale moschata V0735A 1	Crocuta crocuta NC020670 3	0,19116887
Lycaon pictus NC028427 2	Cynogale bennetti KY117544 1	0,19116895
Paradoxurus hermaphroditus NC03959	Arctotherium sp NC030174 1	0,19116943
Urocyon cinereoargenteus NC026723 2	Leptailurus serval NC028316 1	0,19117017
Panthera uncia NC010638 1	Arctocephalus pusillus NC008417 1	0,19117047
Phocarctos hookeri NC008418 1	Felis chaus NC028307 1	0,19117071
Cryptoprocta ferox CFC13 1	Arctotherium sp NC030174 1	0,19117127
Procyon lotor AB462046 3	Prionodon pardicolor NC024569 2	0,19117209
Otocyon megalotis SAF1 2	Hyaena hyaena NC020669 1	0,19117393
Ursus thibetanus laniger MH281753 2	Atilax paludinosus T606 1	0,19117619
Zalophus wollebaeki SRR4431565 1	Galidia elegans D146 1	0,19117619
Vulpes ferrilata NC027935 1	Ursus thibetanus thibetanus NC01111	0,19117638
Vulpes lagopus NC026529 3	Panthera leo spelaea KX258452 2	0,19117802
Ursus arctos EU497665 29	Pardofelis marmorata NLN3 2	0,1911781
Ursus arctos GU573491 207	Felis nigripes NC028309 1	0,1911784
Ursus arctos AP012576 6	Prionodon linsang ERR2391707 1	0,19117929
Lontra canadensis SRR10409165 1	Ailuropoda melanoleuca NC009492 5	0,19118083
Ursus arctos GU573486 5	Otocyon megalotis SAF1 2	0,1911809
Ictonyx striatus T299 1	Canis anthus NC027956 2	0,19118302
Ursus arctos AP012576 6	Canis anthus NC027956 2	0,19118392
Ursus maritimus NC003428 31	Panthera onca NC022842 1	0,19118765
Panthera leo spelaea KX258452 2	Mustela altaica NC021751 1	0,19118783
Poecilogale albinucha T602 1	Panthera leo spelaea KX258452 2	0,1911885
Canis lupus chanco NC010340 4	Callorhinus ursinus NC008415 1	0,1911917
Paradoxurus hermaphroditus NC03959	Arctocephalus forsteri NC004023 28	0,19119222
Ursus americanus JX196366 3	Urva javanica T413 1	0,19119619
Galerella sanguinea T378 1	Cuon alpinus NC013445 3	0,19119743
Panthera leo NERO 19	Arctocephalus australis MG023139 1	0,1912019
Zalophus californianus NC008416 1	Canis mesomelas KT448280 1	0,19120301
Cuon alpinus NC013445 3	Atilax paludinosus T606 1	0,19120526
Urva brachyura KY117547 1	Cuon alpinus NC013445 3	0,19120583
Vulpes ferrilata NC027935 1	Panthera pardus NC010641 5	0,1912092
Profelis aurata NC028299 1	Arctocephalus forsteri NC004023 28	0,19121715
Vulpes vulpes NC008434 5	Lontra canadensis SRR10409165 1	0,1912189
Martes martes T302 3	Diplogale hosei MH464790 1	0,19121967
Vulpes ferrilata NC027935 1	Urva brachyura KY117547 1	0,19122116
Ursus americanus JX196366 3	Panthera onca NC022842 1	0,19122268
Urva brachyura KY117547 1	Bassaricyon neblina SRX1097850 1	0,19122285
Tremarctos ornatus NC009969 2	Prionodon linsang ERR2391707 1	0,1912239
Urocyon littoralis catalinae KP129018	Mustela frenata NC020640 1	0,19122593
Canis mesomelas KT448280 1	Ailuropoda melanoleuca NC009492 5	0,19122919
Vulpes corsac NC023958 1	Ursus maritimus NC003428 31	0,19123021
Vulpes lagopus NC026529 3	Cryptoprocta ferox CFC13 1	0,19123064
Ursus arctos isabellinus 18B5 2	Mungos mungo/gambianus SRR77048	0,19123226
Ursus arctos GU573491 207	Helogale parvula SRR7637809 1	0,19123232
Poecilogale albinucha T602 1	Nyctereutes procyonoides NC013700 3	0,19123286
Profelis aurata NC028299 1	Arctocephalus townsendi NC008420 1	0,19123301
Zalophus wollebaeki SRR4431565 1	Tapirus terrestris T358	0,19123323
Speothos venaticus C48 2	Meles leucurus NC039173 4	0,19123444
Paguma larvata PDDS11 2	Aonyx cinerea NC035814 2	0,19123463
Eupleres goudotii D128 1	Canis mesomelas KT448280 1	0,19123465
Paradoxurus hermaphroditus NC03959	Neophoca cinerea NC008419 1	0,19123474
Panthera uncia NC010638 1	Canis anthus NC027956 2	0,19123483
Galidictis fasciata DM333 1	Canis mesomelas KT448280 1	0,19123507
Nasua nasua NC020647 1	Lynx lynx NC027083 4	0,19123566
Zalophus wollebaeki SRR4431565 1	Canis anthus NC027956 2	0,19123696
Otocyon megalotis SAF1 2	Otaria byronia OTAB 1	0,191237
Urocyon cinereoargenteus NC026723 2	Otocolobus manul NC028323 1	0,19123739
Ursus maritimus NC003428 31	Canis anthus NC027956 2	0,19123739
Zalophus wollebaeki SRR4431565 1	Vulpes corsac NC023958 1	0,19123767
Speothos venaticus C48 2	Arctonyx collaris NC020645 1	0,19123855
Urocyon cinereoargenteus NC026723 2	Leopardus geoffroyi NC028320 1	0,19123855
Tremarctos ornatus NC009969 2	Paradoxurus hermaphroditus NC03959	0,1912394
Procyon lotor AB462049 4	Panthera pardus japonensis KJ866876	0,19123973
Melogale moschata V0735A 1	Ailuropoda melanoleuca NC009492 5	0,1912405
Speothos venaticus C48 2	Procyon lotor AB462046 3	0,19124052
Vulpes vulpes NC008434 5	Ursus thibetanus formosanus NC0093	0,1912407
Leopardus tigrinus NC028317 1	Bassaricyon neblina SRX1097850 1	0,19124098
Vulpes zerda KJ603240 1	Panthera tigris NC010642 35	0,19124161
Panthera tigris NC010642 35	Mustela sibirica AP017394 11	0,19124167
Ursus arctos EU497665 29	Paradoxurus hermaphroditus NLNC 1	0,19124182
Proteles cristata T393 6	Potos flavus T414 1	0,19124208
Odobenus rosmarus NC004029 29	Mustela itatsi NC034330 19	0,19124219
Vulpes vulpes NC008434 5	Helarctos malayanus NC009968 2	0,19124292
Vulpes lagopus NC026529 3	Ursus thibetanus laniger MH281753 2	0,19124337
Vulpes corsac NC023958 1	Melursus ursinus NC009970 2	0,19124408
Ursus maritimus NC003428 31	Panthera uncia NC010638 1	0,19124494
Mungos mungo/gambianus SRR77048	Mephitis mephitis NC020648 1	0,19125037
Odobenus rosmarus NC004029 29	Aonyx cinerea NC035814 2	0,19125347
Canis aureus KT448274 1	Callorhinus ursinus NC008415 1	0,1912557
Paradoxurus hermaphroditus NLNC 1	Melursus ursinus NC009970 2	0,19125731
Ursus arctos AP012576 6	Prionailurus planiceps NC028312 6	0,19126254
Lycalopex sechurae KT448284 1	Ictonyx striatus T299 1	0,191264
Vulpes corsac NC023958 1	Spilogale putorius NC010497 1	0,19126569
Meles leucurus NC039173 4	Tapirus terrestris T358	0,19127165

Zalophus wollebaeki SRR4431565 1	Helarctos malayanus NC009968 2	0,17792851
Spilogale putorius NC010497 1	Mustela sibirica NC020637 6	0,17793144
Pusa caspica NC008431 1	Panthera pardus NC010641 5	0,17793871
Spilogale putorius NC010497 1	Ommatophoca rossii AY377287etc 1	0,17794299
Tremarctos ornatus NC009969 2	Taxidea taxus NC020646 1	0,17794859
Meles anakuma NC009677 1	Bassaricyon neblina SRX1097850 1	0,17794953
Mustela sibirica NC020637 6	Callorhinus ursinus NC008415 1	0,17794968
Meles meles T303 3	Conepatus chinga NC042596 1	0,1779542
Mustela erminea T305 2	Urva semitorquata MH464789 1	0,1779563
Erignathus barbatus NC008426 1	Canis anthus NC027956 2	0,17795695
Tremarctos ornatus NC009969 2	Mustela erminea T305 2	0,17795838
Parahyaena brunnea NC038159 15	Halichoerus grypus NC001602 2	0,17795846
Taxidea taxus NC020646 1	Paguma larvata PDDS11 2	0,17796242
Prionailurus rubiginosus NC028304 2	Meles meles T303 3	0,17796347
Martes zibellina NC011579 39	Arctodus simus NC011116 1	0,17796549
Pusa hispida NC 008433 1	Xenogale naso C07XAR110 1	0,17796625
Arctotherium sp NC030174 1	Ailurus fulgens styani NC009691 1	0,1779667
Viverra tangalunga MH464792 1	Puma yagouaroundi NC028311 1	0,17796706
Mustela nigripes NC024942 1	Leopardus wiedii NC028318 1	0,17796709
Phoca fasciata NC008428 1	Paradoxurus jerdoni MH464793 1	0,17796782
Viverra tangalunga MH464792 1	Leptailurus serval NC028316 1	0,17796783
Ursus thibetanus mupinensis NC008753 2	Nandinia binotata NC024567 1	0,17796788
Pusa caspica NC008431 1	Mephitis mephitis NC020648 1	0,17797479
Mustela nigripes NC024942 1	Hemigalus derbyanus MH464791 1	0,17797998
Prionailurus rubiginosus NC028304 2	Martes foina NC020643 1	0,17798211
Monachus schauinslandi NC008421 1	Cryptoprocta ferox CFC13 1	0,1779826
Zalophus californianus NC008416 1	Martes zibellina NC011579 39	0,17798354
Mustela frenata NC020640 1	Leopardus colocolo NC028314 1	0,1779843
Taxidea taxus NC020646 1	Leopardus colocolo NC028314 1	0,17798947
Ommatophoca rossii AY377287etc 1	Melogale moschata KP726273 1	0,17799804
Catopuma badia NC028300 1	Canis lupus familiaris NC002008 1231	0,17800635
Chrotogale owstoni T607 1	Canis adustus KT448271 1	0,1780189
Nasua nasua NC020647 1	Martes zibellina NC011579 39	0,17802092
Mustela erminea T305 2	Conepatus chinga NC042596 1	0,17802283
Nyctereutes procyonoides NC013700 3	Nandinia binotata NC024567 1	0,17802495
Mirounga leonina NC008422 1	Lutrogale perspicillata NC035811 1	0,17802797
Meles leucurus NC039173 4	Leopardus tigrinus NC028317 1	0,1780297
Lynx lynx NC027083 4	Lutra lutra NC011358 9	0,17803076
Viverra tangalunga MH464792 1	Lynx lynx NC027083 4	0,17803219
Phoca groenlandica NC008429 54	Mungos mungo/gambianus SRR7704821 1	0,17803317
Monachus schauinslandi NC008421 1	Lontra canadensis SRR10409165 1	0,17803542
Puma yagouaroundi NC028311 1	Mustela erminea T305 2	0,17803618
Lynx lynx NC027083 4	Bassariscus sumichrasti SRX1099089 1	0,17803741
Taxidea taxus NC020646 1	Leopardus jacobita NC028322 1	0,17803851
Ursus thibetanus mupinensis NC008753 2	Meles leucurus NC039173 4	0,17803879
Mungotictis decemlineata NC027828 1	Monachus schauinslandi NC008421 1	0,17804071
Ursus maritimus GU573488 Svalbard	Mustela erminea T305 2	0,17804249
Panthera onca KP202264 2	Monachus schauinslandi NC008421 1	0,17804493
Ursus thibetanus formosanus NC009331 1	Nandinia binotata NC024567 1	0,17804557
Panthera uncia NC010638 1	Lobodon carcinophaga NC008423 1	0,17804569
Meles leucurus NC039173 4	Acinonyx jubatus NC005212 3	0,17804632
Mustela altaica NC021751 1	Hemigalus derbyanus MH464791 1	0,17804742
Zalophus californianus NC008416 1	Lutra sumatrana NC035810 1	0,17804796
Neovison vison NC020641 3	Civettictis civetta GLC19 1	0,17804945
Viverricula indica NC025296 2	Bassariscus sumichrasti SRX1099089 1	0,17804953
Nandinia binotata NC024567 1	Canis lupus familiaris NC002008 1231	0,17805275
Gulo gulo NC009685 3	Conepatus chinga NC042596 1	0,17805631
Phoca groenlandica NC008429 54	Diplogale hosei MH464790 1	0,17806047
Hemigalus derbyanus MH464791 1	Helarctos malayanus NC009968 2	0,17806655
Lycalopex sechurae KT448284 1	Erignathus barbatus NC008426 1	0,17807215
Spilogale putorius NC010497 1	Potos flavus T414 1	0,17807403
Viverricula indica KX891751 1	Canis lupus chanco NC010340 4	0,17807451
Phoca vitulina NC001325 1	Panthera leo NERO 19	0,17808118
Mustela sibirica AP017394 11	Callorhinus ursinus NC008415 1	0,17808411
Ommatophoca rossii AY377287etc 1	Civettictis civetta NC033378 1	0,17808656
Crossarchus platycephalus C7R66 1	Bassariscus sumichrasti SRX1099089 1	0,17809276
Nasua nasua NC020647 1	Martes pennanti NC020664 16	0,17809333
Neovison vison NC020641 3	Arctocephalus forsteri KT693377 17	0,17809629
Prionailurus bengalensis NC028301 12	Enhydra lutris NC009692 1	0,17809652
Panthera onca NC022842 1	Halichoerus grypus NC001602 2	0,17809927
Mustela frenata NC020640 1	Felis catus NC001700 2	0,17810008
Prionailurus viverrinus NC028305 1	Mustela frenata NC020640 1	0,17810056
Ommatophoca rossii AY377287etc 1	Diplogale hosei MH464790 1	0,17810169
Lycaon pictus NC028427 2	Cystophora cristata NC008427 1	0,17810179
Phoca groenlandica NC008429 54	Mungos mungo MMC7 1	0,17810197
Meles leucurus NC039173 4	Arctodus simus NC011116 1	0,17810306
Mustela nigripes NC024942 1	Catopuma temminckii NC027115 41	0,17810418
Taxidea taxus NC020646 1	Leopardus tigrinus NC028317 1	0,17810678
Panthera leo spelaea KX258452 2	Erignathus barbatus NC008426 1	0,1781072
Meles meles T303 3	Genetta abyssinica MG489822 1	0,17810836
Phoca fasciata NC008428 1	Panthera onca KP202264 2	0,17810935
Paguma larvata PDDS11 2	Arctodus simus NC011116 1	0,17811187
Monachus schauinslandi NC008421 1	Galerella sanguinea T378 1	0,17811249
Ursus thibetanus formosanus NC009331 1	Meles leucurus NC039173 4	0,1781143
Helarctos malayanus NC009968 2	Arctocephalus pusillus NC008417 1	0,17811911
Ursus spelaeus EU327344 13	Hemigalus derbyanus MH464791 1	0,17812012
Halichoerus grypus NC001602 2	Conepatus chinga NC042596 1	0,17812042
Panthera onca NC022842 1	Mirounga angustirostris SRR10331586 1	0,17812333
Phoca vitulina NC001325 1	Panthera onca NC022842 1	0,1781278
Mustela erminea T305 2	Canis lupus familiaris NC002008 1231	0,17812854
Paradoxurus hermaphroditus NLNC 1	Monachus monachus NC044972 5	0,17812911
Martes flavigula NC012141 3	Arctocephalus gazella BK010918 1	0,1781365
Halichoerus grypus NC001602 2	Bdeogale nigripes GLC15 1	0,17813797
Mirounga angustirostris SRR10331586 1	Canis lupus chanco NC010340 4	0,17814593
Zalophus wollebaeki SRR4431565 1	Martes flavigula NC012141 3	0,17814887
Mirounga leonina NC008422 1	Cynogale bennetti KY117544 1	0,17814897
Halichoerus grypus NC001602 2	Galidictis fasciata DM333 1	0,17815107
Pusa sibirica NC008432 2	Urva brachyura KY117547 1	0,17815782
Vulpes vulpes NC008434 5	Cystophora cristata NC008427 1	0,1781581
Pusa sibirica NC008432 2	Paguma larvata PDDS11 2	0,17816649
Smilodon populator MF871700 1	Potos flavus T414 1	0,1781669
Viverra tangalunga MH464792 1	Leopardus pardalis T262 1	0,17816781
Paguma larvata PDDS11 2	Mustela kathiah NC023210 1	0,17816885

Vulpes zerda KJ603240 1	Panthera pardus NC010641 5	0,19127215
Panthera pardus NC010641 5	Arctocephalus australis MG023139 1	0,19127242
Panthera leo spelaea KX258452 2	Chrysocyon brachyurus NC024172 1	0,19127244
Spilogale putorius NC010497 1	Lynx canadensis NC028313 1	0,19128002
Martes americana NC020642 1	Diplogale hosei MH464790 1	0,19128065
Spilogale putorius NC010497 1	Otaria byronia OTAB 1	0,19128184
Eupleres goudotii D128 1	Ailurus fulgens NC011124 1	0,19128367
Ursus spelaeus NC011112 8	Urva javanica/auropunctata NC006835	0,19128543
Vulpes vulpes NC008434 5	Potos flavus T414 1	0,19128709
Vulpes lagopus NC026529 3	Potos flavus T414 1	0,19128719
Panthera tigris NC010642 35	Arctocephalus forsteri NC004023 28	0,19128834
Suricata suricatta SSM10 1	Bassaricyon neblina SRX1097850 1	0,19128835
Lontra canadensis SRR10409165 1	Chrotogale owstoni T607 1	0,19128938
Panthera onca KP202264 2	Conepatus chinga NC042596 1	0,19128947
Nyctereutes procyonoides NC013700 3	Conepatus chinga NC042596 1	0,1912896
Ursus thibetanus laniger MH281753 2	Urva javanica/auropunctata NC006835	0,19129264
Ursus spelaeus NC011112 8	Neofelis nebulosa NC008450 3	0,19129273
Otaria byronia OTAB 1	Galidia elegans D146 1	0,19129321
Paradoxurus hermaphroditus NC03959	Martes americana NC020642 1	0,19129525
Vulpes corsac NC023958 1	Paradoxurus hermaphroditus NC03959	0,19129641
Panthera pardus NC010641 5	Helarctos malayanus NC009968 2	0,19129667
Vulpes corsac NC023958 1	Ursus maritimus GU573488 Svalbard	0,19129759
Vulpes lagopus NC026529 3	Mustela altaica NC021751 1	0,19129804
Zalophus wollebaeki SRR4431565 1	Canis latrans NC008093 7	0,19130421
Paradoxurus hermaphroditus NLNC 1	Martes martes T302 3	0,19130445
Urocyon littoralis catalinae KP129018	Caracal caracal NC028306 1	0,19130459
Phocarctos hookeri NC008418 1	Crocuta crocuta NC020670 3	0,19130474
Vulpes ferrilata NC027935 1	Ailuropoda melanoleuca NC009492 5	0,19130689
Speothos venaticus C48 2	Procyon lotor AB462049 4	0,19130729
Mustela kathiah NC023210 1	Crocuta crocuta NC020670 3	0,19130734
Crocuta crocuta NC020670 3	Arctocephalus forsteri KT693377 17	0,1913079
Panthera onca KP202264 2	Arctotherium sp NC030174 1	0,19130811
Panthera pardus japonensis KJ866876	Arctotherium sp NC030174 1	0,19130829
Prionailurus bengalensis NC028301 12	Bassaricyon neblina SRX1097850 1	0,19130901
Odobenus rosmarus NC004029 29	Mustela sibirica NC020637 6	0,19130965
Ursus arctos EU497665 29	Parahyaena brunnea NC038159 15	0,19131097
Tremarctos ornatus NC009969 2	Panthera tigris amoyensis NC014770 2	0,19131126
Melursus ursinus NC009970 2	Xenogale naso C07XAR110 1	0,19131178
Melogale moschata NC020644 1	Crocuta crocuta NC020670 3	0,19131251
Mephitis mephitis NC020648 1	Ichneumia albicauda T603 1	0,19131365
Paradoxurus hermaphroditus NC03959	Martes foina NC020643 1	0,19131437
Procyon lotor AB462046 3	Odobenus rosmarus NC004029 29	0,19131465
Panthera tigris NC010642 35	Arctotherium sp NC030174 1	0,19131616
Panthera leo spelaea KX258452 2	Nyctereutes procyonoides NC013700 3	0,19131671
Panthera tigris amoyensis NC014770 2	Mustela nivalis T306 5	0,19131815
Ursus arctos GU573486 5	Pardofelis marmorata NLN3 2	0,19131893
Panthera tigris amoyensis NC014770 2	Martes pennanti NC020664 16	0,19131957
Ursus arctos AP012576 6	Galidia elegans D146 1	0,19131967
Mephitis mephitis NC020648 1	Bassaricyon neblina SRX1097850 1	0,19132252
Cryptoprocta ferox CFC13 1	Canis aureus KT448274 1	0,19132267
Ursus thibetanus formosanus NC0093	Panthera uncia NC010638 1	0,19132277
Galictis vittata T412 1	Felis chaus NC028307 1	0,1913258
Zalophus wollebaeki SRR4431565 1	Crocuta crocuta NC020670 3	0,19132808
Speothos venaticus C48 2	Ictonyx striatus T299 1	0,19132961
Spilogale putorius NC010497 1	Urva javanica T413 1	0,19133204
Ichneumia albicauda T603 1	Cuon alpinus NC013445 3	0,19133623
Spilogale putorius NC010497 1	Leopardus wiedii NC028318 1	0,1913404
Ursus americanus JX196366 3	Parahyaena brunnea NC038159 15	0,19134338
Zalophus californianus NC008416 1	Canis lupus chanco NC010340 4	0,19134483
Prionodon linsang ERR2391707 1	Callorhinus ursinus NC008415 1	0,19134842
Urva javanica T413 1	Tapirus terrestris T358	0,19135103
Panthera uncia NC010638 1	Martes flavigula NC012141 3	0,19135199
Vulpes zerda KJ603240 1	Lontra canadensis SRR10409165 1	0,19135486
Urocyon littoralis catalinae KP129018	Melogale moschata KP726273 1	0,19135676
Vulpes vulpes NC008434 5	Ichneumia albicauda T603 1	0,19135766
Mustela putorius NC020638 4	Cynogale bennetti KY117544 1	0,19135803
Vulpes vulpes NC008434 5	Procyon lotor AB462049 4	0,19135974
Urocyon littoralis catalinae KP129018	Bassariscus sumichrasti SRX1099089 1	0,19135991
Galidia elegans D146 1	Ailurus fulgens styani NC009691 1	0,19136019
Panthera pardus japonensis KJ866876	Tapirus terrestris T358	0,1913611
Panthera uncia NC010638 1	Ailurus fulgens styani NC009691 1	0,19136274
Poecilogale albinucha T602 1	Urva brachyura KY117547 1	0,1913649
Ursus arctos GU573491 207	Mungos mungo/gambianus SRR77048	0,19136699
Otaria byronia OTAB 1	Canis latrans NC008093 7	0,19136713
Procyon lotor AB462049 4	Cryptoprocta ferox CFC13 1	0,19136822
Ursus americanus JX196366 3	Odobenus rosmarus NC004029 29	0,1913687
Panthera uncia NC010638 1	Mustela altaica NC021751 1	0,19137506
Panthera leo spelaea KX258452 2	Mustela itatsi NC034330 19	0,19137585
Panthera uncia NC010638 1	Martes pennanti NC020664 16	0,19137609
Mungos mungo MMC7 1	Ailuropoda melanoleuca NC009492 5	0,19137621
Vulpes zerda KJ603240 1	Ursus thibetanus laniger MH281753 2	0,19137768
Ursus arctos GU573491 207	Panthera pardus japonensis KJ866876	0,19137913
Ursus arctos pruinosus MG066703 3	Felis nigripes NC028309 1	0,19138077
Conepatus chinga NC042596 1	Chrysocyon brachyurus NC024172 1	0,19138198
Panthera uncia NC010638 1	Martes foina NC020643 1	0,19138218
Ursus thibetanus thibetanus NC01111	Nasua nasua NC020647 1	0,191383
Lynx lynx NC027083 4	Ailuropoda melanoleuca NC009492 5	0,19138511
Canis lupus familiaris NC002008 1231	Callorhinus ursinus NC008415 1	0,19138703
Ursus arctos AP012576 6	Mungotictis decemlineata NC027828 1	0,19138749
Ursus maritimus GU573488 Svalbard	Panthera onca NC022842 1	0,19138972
Odobenus rosmarus NC004029 29	Martes pennanti NC020664 16	0,19139095
Vulpes corsac NC023958 1	Urva javanica/auropunctata NC006835	0,19139299
Panthera tigris amoyensis NC014770 2	Helarctos malayanus NC009968 2	0,19139867
Panthera tigris NC010642 35	Helarctos malayanus NC009968 2	0,19139872
Neofelis nebulosa NC008450 3	Martes melampus NC009678 1	0,19140096
Melogale moschata V0735A 1	Canis mesomelas KT448280 1	0,19140494
Phocarctos hookeri NC008418 1	Panthera leo NERO 19	0,19140615
Ursus arctos pruinosus MG066703 3	Diplogale hosei MH464790 1	0,19140704
Speothos venaticus C48 2	Gulo gulo NC009685 3	0,19141103
Panthera pardus NC010641 5	Lontra canadensis SRR10409165 1	0,19141239
Callorhinus ursinus NC008415 1	Tapirus terrestris T358	0,19141687
Ursus arctos pruinosus MG066703 3	Panthera pardus NC010641 5	0,19141838
Urva javanica/auropunctata NC006835	Ailuropoda melanoleuca NC009492 5	0,19141915

Mustela erminea T305 2	Genetta servalina NC024568 2	0,17817026
Viverricula indica NC025296 2	Canis anthus NC027956 2	0,17817143
Prionailurus bengalensis CKM45 20	Potos flavus T414 1	0,17817423
Lobodon carcinophaga NC008423 1	Galidia elegans D146 1	0,17817573
Phoca vitulina NC001325 1	Panthera pardus japonensis KJ866876 8	0,17818375
Taxidea taxus NC020646 1	Acinonyx jubatus NC005212 3	0,17818636
Panthera tigris NC010642 35	Lobodon carcinophaga NC008423 1	0,17819059
Mustela sibirica NC020637 6	Eumetopias jubatus NC004030 10	0,17819534
Bassariscus sumichrasti SRX1099089 1	Acinonyx jubatus NC005212 3	0,17819667
Mustela frenata NC020640 1	Canis lupus chanco NC010340 4	0,17819959
Urva semitorquata MH464789 1	Halichoerus grypus NC001602 2	0,17820662
Spilogale putorius NC010497 1	Procyon lotor AB462046 3	0,17821555
Puma concolor NC016470 22	Ommatophoca rossii AY377287etc 1	0,17822222
Mustela frenata NC020640 1	Arctocephalus australis MG023139 1	0,17822343
Prionodon linsang ERR2391707 1	Leptonychotes weddellii NC008424 1	0,17822516
Neophoca cinerea NC008419 1	Mustela erminea T305 2	0,1782257
Vulpes lagopus NC026529 3	Erignathus barbatus NC008426 1	0,17822607
Viverricula indica KX891745 1	Mustela frenata NC020640 1	0,17823114
Phoca vitulina NC001325 1	Chrotogale owstoni T607 1	0,17823267
Phoca largha NC008430 1	Chrotogale owstoni T607 1	0,17823303
Mustela frenata NC020640 1	Civettictis civetta GLC19 1	0,17823404
Martes melampus NC009678 1	Lynx canadensis NC028313 1	0,17823438
Lutra sumatrana NC035810 1	Arctotherium sp NC030174 1	0,17823525
Mungos mungo/gambianus SRR7704821 1	Monachus schauinslandi NC008421 1	0,17823572
Puma yagouaroundi NC028311 1	Lutra sumatrana NC035810 1	0,17823599
Taxidea taxus NC020646 1	Genetta genetta T297 1	0,17823745
Potos flavus T414 1	Leopardus pardalis T262 1	0,17823887
Mungotictis decemlineata NC027828 1	Mirounga angustirostris SRR10331586 1	0,178241
Taxidea taxus NC020646 1	Lynx canadensis NC028313 1	0,17824109
Mirounga angustirostris SRR10331586 1	Galidictis fasciata DM333 1	0,17824115
Monachus schauinslandi NC008421 1	Galidictis fasciata DM333 1	0,17824295
Martes foina NC020643 1	Arctotherium sp NC030174 1	0,17824703
Potos flavus T414 1	Helarctos malayanus NC009968 2	0,1782476
Leopardus pardalis T262 1	Arctodus simus NC011116 1	0,1782479
Leptailurus serval NC028316 1	Arctodus simus NC011116 1	0,17824884
Mustela kathiah NC023210 1	Acinonyx jubatus NC005212 3	0,1782507
Zalophus californianus NC008416 1	Meles meles T303 3	0,17825221
Zalophus californianus NC008416 1	Martes martes T302 3	0,17825767
Hemigalus derbyanus MH464791 1	Canis lupus chanco NC010340 4	0,17827436
Leptonychotes weddellii NC008424 1	Cuon alpinus NC013445 3	0,17827773
Leptailurus serval NC028316 1	Canis lupus chanco NC010340 4	0,17827998
Mustela erminea T305 2	Civettictis civetta NC033378 1	0,17828105
Arctodus simus NC011116 1	Arctocephalus townsendi NC008420 1	0,17830014
Prionailurus viverrinus NC028305 1	Meles meles T303 3	0,17830066
Mustela kathiah NC023210 1	Leopardus pardalis NC028315 1	0,17830136
Mustela frenata NC020640 1	Felis chaus NC028307 1	0,17830183
Otocolobus manul NC028323 1	Mustela frenata NC020640 1	0,17830189
Martes melampus NC009678 1	Lynx lynx NC027083 4	0,17830192
Mustela nigripes NC024942 1	Lynx lynx NC027083 4	0,1783022
Helarctos malayanus NC009968 2	Eumetopias jubatus NC004030 10	0,17830226
Mustela nigripes NC024942 1	Lynx pardinus NC028319 161	0,1783029
Mustela sibirica AP017394 11	Leopardus wiedii NC028318 1	0,17830345
Meles leucurus NC039173 4	Catopuma badia NC028300 1	0,17830351
Phoca fasciata NC008428 1	Nyctereutes procyonoides NC013700 3	0,17830375
Viverra tangalunga MH464792 1	Prionailurus viverrinus NC028305 1	0,17830417
Puma yagouaroundi NC028311 1	Mustela nigripes NC024942 1	0,17830423
Paradoxurus jerdoni MH464793 1	Mustela erminea T305 2	0,17830451
Mustela erminea T305 2	Felis silvestris lybica KP202275 4	0,17830525
Mustela sibirica NC020637 6	Arctodus simus NC011116 1	0,17830551
Mustela frenata NC020640 1	Leopardus guigna NC028321 1	0,17830573
Phoca largha NC008430 1	Urva brachyura KY117547 1	0,17830607
Monachus monachus NC044972 5	Canis mesomelas KT448280 1	0,17830725
Prionodon pardicolor NC024569 2	Erignathus barbatus NC008426 1	0,17830935
Mustela nivalis T306 5	Leopardus pardalis T262 1	0,17831308
Galictis vittata T412 1	Bassaricyon neblina SRX1097850 1	0,17831339
Phoca fasciata NC008428 1	Panthera tigris NC010642 35	0,17831975
Martes foina NC020643 1	Leopardus geoffroyi NC028320 1	0,17832023
Ursus thibetanus thibetanus NC011118 4	Mustela sibirica NC020637 6	0,17832023
Cystophora cristata NC008427 1	Canis lupus familiaris NC002008 1231	0,17832567
Phoca vitulina NC001325 1	Panthera tigris NC010642 35	0,17832713
Halichoerus grypus NC001602 2	Chrotogale owstoni T607 1	0,17833298
Neovison vison NC020641 3	Leopardus colocolo NC028314 1	0,17833446
Ursus arctos isabellinus 18B5 2	Hemigalus derbyanus MH464791 1	0,17833516
Mustela putorius NC020638 4	Homotherium latidens MF871702 3	0,17833532
Panthera onca KP202264 2	Monachus monachus NC044972 5	0,17833656
Halichoerus grypus NC001602 2	Canis latrans NC008093 7	0,17834374
Spilogale putorius NC010497 1	Procyon lotor AB462049 4	0,17835018
Lobodon carcinophaga NC008423 1	Cynogale bennetti KY117544 1	0,17835753
Bassariscus sumichrasti SRX1099089 1	Arctocephalus townsendi NC008420 1	0,17835921
Mirounga leonina NC008422 1	Xenogale naso C07XAR110 1	0,17835982
Pusa sibirica NC008432 2	Urva javanica T413 1	0,17835982
Mustela frenata NC020640 1	Canis latrans NC008093 7	0,17836246
Lynx lynx NC027083 4	Arctonyx collaris NC020645 1	0,17836647
Lynx pardinus NC028319 161	Lutra lutra LC050126 1	0,17836708
Phoca fasciata NC008428 1	Canis aureus KT448274 1	0,17836782
Paradoxurus hermaphroditus NLNC 1	Cystophora cristata NC008427 1	0,17836863
Paguma larvata PDDS11 2	Cystophora cristata NC008427 1	0,17836866
Suricata suricatta SSM10 1	Pusa hispida NC 008433 1	0,17836919
Phoca groenlandica NC008429 54	Canis latrans NC008093 7	0,17837015
Martes americana NC020642 1	Lynx lynx NC027083 4	0,17837179
Taxidea taxus NC020646 1	Otocolobus manul NC028323 1	0,17837217
Lutra sumatrana NC035810 1	Arctodus simus NC011116 1	0,17837237
Ursus thibetanus laniger MH281753 2	Eumetopias jubatus NC004030 10	0,17837393
Viverra tangalunga MH464792 1	Prionailurus bengalensis CKM45 20	0,17837512
Melogale moschata NC020644 1	Arctodus simus NC011116 1	0,1783755
Mustela erminea T305 2	Arctocephalus forsteri NC004023 28	0,17837788
Homotherium latidens MF871702 3	Conepatus chinga NC042596 1	0,17838512
Phoca vitulina NC001325 1	Panthera uncia NC010638 1	0,17838596
Ursus spelaeus NC011112 8	Meles leucurus NC039173 4	0,17838678
Prionailurus bengalensis NC028301 12	Ictonyx striatus T299 1	0,17838691
Ursus spelaeus EU327344 13	Meles meles T303 3	0,17838743
Potos flavus T414 1	Genetta abyssinica MG489822 1	0,17838795
Phoca vitulina NC001325 1	Mephitis mephitis NC020648 1	0,1783902

Eupleres goudotii D128 1	Bassaricyon neblina SRX1097850 1	0,19142163
Vulpes vulpes NC008434 5	Arctocephalus forsteri KT693377 17	0,19142213
Urocyon cinereoargenteus NC026723 2	Martes americana NC020642 1	0,19142327
Mungotictis decemlineata NC027828 1	Bassaricyon neblina SRX1097850 1	0,19142379
Mustela altaica NC021751 1	Cynogale bennetti KY117544 1	0,1914261
Urocyon cinereoargenteus NC026723 2	Mungotictis decemlineata NC027828 1	0,19142617
Urocyon littoralis catalinae KP129018 1	Mustela erminea T305 2	0,19142942
Vulpes corsac NC023958 1	Panthera uncia NC010638 1	0,1914351
Tremarctos ornatus NC009969 2	Otocyon megalotis SAF1 2	0,19143642
Neophoca cinerea NC008419 1	Lycaon pictus NC028427 2	0,19143802
Nasua nasua NC020647 1	Canis adustus KT448271 1	0,19144106
Poecilogale albinucha T602 1	Panthera uncia NC010638 1	0,1914438
Bdeogale nigripes GLC15 1	Ailuropoda melanoleuca NC009492 5	0,19144401
Ursus arctos GU573486 5	Galidictis fasciata DM333 1	0,19144438
Crossarchus platycephalus C7R66 1	Ailuropoda melanoleuca NC009492 5	0,19144453
Ursus arctos GU573486 5	Canis anthus NC027956 2	0,1914456
Vulpes ferrilata NC027935 1	Ursus spelaeus EU327344 13	0,19144571
Ursus arctos isabellinus 18B5 2	Pardofelis marmorata NLN3 2	0,19144692
Ursus maritimus GU573488 Svalbard	Panthera uncia NC010638 1	0,19144699
Ursus thibetanus thibetanus NC01111	Suricata suricatta SSM10 1	0,19144861
Ursus arctos GU573491 207	Otocolobus manul NC028323 1	0,19144915
Otocyon megalotis SAF1 2	Ictonyx striatus T299 1	0,19145206
Ursus thibetanus mupinensis NC00875	Proteles cristata T393 6	0,19145358
Panthera leo spelaea KX258452 2	Martes foina NC020643 1	0,191459
Conepatus chinga NC042596 1	Canis lupus chanco NC010340 4	0,19145906
Eumetopias jubatus NC004030 10	Cuon alpinus NC013445 3	0,1914597
Urva javanica/auropunctata NC006835	Conepatus chinga NC042596 1	0,1914645
Proteles cristata T393 6	Helarctos malayanus NC009968 2	0,1914648
Vulpes vulpes NC008434 5	Arctocephalus forsteri NC004023 28	0,19146736
Ursus americanus JX196366 3	Prionodon linsang ERR2391707 1	0,19146822
Neofelis nebulosa NC008450 3	Martes americana NC020642 1	0,1914698
Panthera leo NERO 19	Arctocephalus forsteri KT693377 17	0,19147094
Lutrogale perspicillata NC035811 1	Canis adustus KT448271 1	0,19147711
Panthera uncia NC010638 1	Gulo gulo NC009685 3	0,19148004
Neofelis nebulosa NC008450 3	Martes martes T302 3	0,1914801
Procyon lotor AB462049 4	Panthera pardus NC010641 5	0,19148295
Cynictis penicillata T375 1	Bassaricyon neblina SRX1097850 1	0,19149145
Nyctereutes procyonoides NC013700 3	Aonyx cinerea NC035814 2	0,1914929
Galidia elegans D146 1	Canis anthus NC027956 2	0,19149318
Ursus thibetanus laniger MH281753 2	Neofelis nebulosa NC008450 3	0,19149399
Urocyon littoralis catalinae KP129018 1	Mustela eversmannii NC028013 1	0,19149549
Otocyon megalotis SAF1 2	Mungos mungo/gambianus SRR77048	0,19149818
Lutra sumatrana NC035810 1	Diplogale hosei MH464790 1	0,19149932
Vulpes corsac NC023958 1	Martes pennanti NC020664 16	0,19149964
Ursus arctos pruinosus MG066703 3	Canis mesomelas KT448280 1	0,19150065
Ursus arctos GU573491 207	Xenogale naso C07XAR110 1	0,19150148
Ursus maritimus GU573488 Svalbard	Cynictis penicillata T375 1	0,19150165
Procyon lotor AB462049 4	Hyaena hyaena NC020669 1	0,19150189
Nasua nasua NC020647 1	Arctodus simus NC011116 1	0,19150305
Chrysocyon brachyurus NC024172 1	Callorhinus ursinus NC008415 1	0,19150315
Panthera uncia KP202269 1	Arctocephalus gazella BK010918 1	0,19150427
Ursus arctos EU497665 29	Lontra canadensis SRR10409165 1	0,19150551
Parahyaena brunnea NC038159 15	Arctocephalus townsendi NC008420 1	0,19150655
Cynogale bennetti KY117544 1	Chrysocyon brachyurus NC024172 1	0,19150709
Canis aureus KT448274 1	Arctocephalus forsteri NC004023 28	0,19150738
Nasua nasua NC020647 1	Felis silvestris lybica KP202275 4	0,19150785
Meles meles T303 3	Crocuta crocuta NC020670 3	0,19150913
Ursus thibetanus formosanus NC00993	Eupleres goudotii D128 1	0,19150963
Ursus americanus JX196366 3	Cuon alpinus NC013445 3	0,19151235
Ursus arctos GU573491 207	Prionodon pardicolor NC024569 2	0,1915134
Melursus ursinus NC009970 2	Atilax paludinosus T606 1	0,19151363
Canis lupus chanco NC010340 4	Arctocephalus gazella BK010918 1	0,19151495
Ursus thibetanus thibetanus NC01111	Urva brachyura KY117547 1	0,19151564
Cynogale bennetti KY117544 1	Canis mesomelas KT448280 1	0,19151844
Panthera tigris NC010642 35	Mustela nivalis T306 5	0,19152027
Procyon lotor AB462049 4	Panthera leo spelaea KX258452 2	0,1915227
Canis lupus familiaris NC002008 1231	Arctocephalus forsteri KT693377 17	0,19152465
Odobenus rosmarus NC004029 29	Lynx lynx NC027083 4	0,19152768
Neofelis nebulosa NC008450 3	Meles anakuma NC009677 1	0,19153632
Panthera leo NERO 19	Lutrogale perspicillata NC035811 1	0,19153699
Ursus spelaeus EU327344 13	Chrysocyon brachyurus NC024172 1	0,1915406
Spilogale putorius NC010497 1	Leopardus tigrinus NC028317 1	0,19154269
Xenogale naso C07XAR110 1	Conepatus chinga NC042596 1	0,19154528
Spilogale putorius NC010497 1	Bassaricyon neblina SRX1097850 1	0,19154905
Ursus arctos AP012576 6	Odobenus rosmarus NC004029 29	0,19154914
Genetta genetta T297 1	Cuon alpinus NC013445 3	0,19155115
Panthera onca KP202264 2	Arctocephalus forsteri NC004023 28	0,19155131
Panthera leo NERO 19	Ailuropoda melanoleuca NC009492 5	0,19155364
Urocyon cinereoargenteus NC026723 2	Melogale moschata KP726273 1	0,19155879
Vulpes lagopus NC026529 3	Meles anakuma NC009677 1	0,19156027
Urocyon littoralis catalinae KP129018 1	Mungotictis decemlineata NC027828 1	0,19156076
Vulpes corsac NC023958 1	Bassariscus sumichrasti SRX1099089 1	0,19156373
Panthera onca KP202264 2	Tapirus terrestris T358	0,19156376
Panthera uncia NC010638 1	Ailurus fulgens NC011124 1	0,19156495
Ursus maritimus GU573488 Svalbard	Prionodon linsang ERR2391707 1	0,19156829
Ursus maritimus GU573488 Svalbard	Mungos mungo MMC7 1	0,191569
Ursus arctos isabellinus 18B5 2	Urva javanica T413 1	0,19156902
Vulpes corsac NC023958 1	Martes foina NC020643 1	0,19157052
Hyaena hyaena NC020669 1	Arctotherium sp NC030174 1	0,19157371
Urocyon littoralis catalinae KP129018 1	Otocolobus manul NC028323 1	0,19157408
Ursus arctos GU573486 5	Cynictis penicillata T375 1	0,19157518
Profelis aurata NC028299 1	Lutrogale perspicillata NC035811 1	0,19157735
Arctocephalus forsteri KT693377 17	Arctictis binturong T605 2	0,19157746
Vulpes zerda KJ603240 1	Panthera tigris amoyensis NC014770 2	0,19157852
Ursus arctos pruinosus MG066703 3	Galidia elegans D146 1	0,19157854
Vulpes lagopus NC026529 3	Ursus thibetanus thibetanus NC01111	0,19158015
Ursus maritimus NC003428 31	Galerella sanguinea T378 1	0,19158065
Ursus arctos EU497665 29	Galerella sanguinea T378 1	0,19158074
Ursus spelaeus NC011112 8	Nyctereutes procyonoides NC013700 3	0,19158426
Poecilogale albinucha T602 1	Panthera tigris amoyensis NC014770 2	0,19158514
Ursus arctos EU497665 29	Lycaon pictus NC028427 2	0,19158599
Neophoca cinerea NC008419 1	Mephitis mephitis NC020648 1	0,19159061
Panthera uncia NC010638 1	Helarctos malayanus NC009968 2	0,19159377

Melogale moschata KP726273 1	Leopardus colocolo NC028314 1	0,17839059
Mustela erminea T305 2	Helarctos malayanus NC009968 2	0,17839143
Mustela nigripes NC024942 1	Canis adustus KT448271 1	0,17839241
Monachus monachus NC044972 5	Eupleres goudotii D128 1	0,17839678
Martes foina NC020643 1	Eumetopias jubatus NC004030 10	0,17840049
Spilogale putorius NC010497 1	Pusa hispida NC 008433 1	0,17840184
Ursus thibetanus laniger MH281753 2	Neovison vison NC020641 3	0,17840485
Martes flavigula NC012141 3	Lynx pardinus NC028319 161	0,17841551
Viverricula indica KX891751 1	Canis mesomelas KT448280 1	0,17841826
Mustela nigripes NC024942 1	Callorhinus ursinus NC008415 1	0,17842154
Lutra lutra NC011358 9	Arctocephalus pusillus NC008417 1	0,17842197
Taxidea taxus NC020646 1	Arctotherium sp NC030174 1	0,17842831
Nasua nasua NC020647 1	Martes martes T302 3	0,17843062
Puma yagouaroundi NC028311 1	Meles meles T303 3	0,17843507
Viverricula indica KX891751 1	Meles leucurus NC039173 4	0,17843534
Ursus maritimus GU573488 Svalbard	Potos flavus T414 1	0,17843713
Melogale moschata KP726273 1	Arctodus simus NC011116 1	0,17843722
Phoca groenlandica NC008429 54	Urva semitorquata MH464789 1	0,17843723
Martes melampus NC009678 1	Leopardus pardalis T262 1	0,17843729
Paradoxurus hermaphroditus NLNC 1	Mustela kathiah NC023210 1	0,17843829
Prionailurus bengalensis NC028301 12	Mustela nigripes NC024942 1	0,17843895
Phoca fasciata NC008428 1	Cryptoprocta ferox CFC13 1	0,17843916
Phoca groenlandica NC008429 54	Paradoxurus jerdoni MH464793 1	0,17843916
Zalophus wollebaeki SRR4431565 1	Meles meles T303 3	0,17843929
Mirounga angustirostris SRR10331586 1	Helogale parvula SRR7637809 1	0,17843978
Ommatophoca rossii AY377287etc 1	Melogale moschata V0735A 1	0,17843989
Viverra zibetha T609 1	Mustela altaica NC021751 1	0,17844293
Pusa caspica NC008431 1	Parahyaena brunnea NC038159 15	0,1784437
Ursus arctos GU573486 5	Potos flavus T414 1	0,17844385
Ursus thibetanus formosanus NC009331 1	Taxidea taxus NC020646 1	0,17844553
Martes martes T302 3	Lynx lynx NC027083 4	0,17844559
Zalophus wollebaeki SRR4431565 1	Martes martes T302 3	0,17844737
Potos flavus T414 1	Hemigalus derbyanus MH464791 1	0,17844847
Monachus schauinslandi NC008421 1	Catopuma temminckii NC027115 41	0,17844883
Ursus thibetanus laniger MH281753 2	Meles leucurus NC039173 4	0,17845316
Monachus monachus NC044972 5	Cynogale bennetti KY117544 1	0,17845403
Phoca largha NC008430 1	Mephitis mephitis NC020648 1	0,17845729
Ursus thibetanus thibetanus NC011118 4	Mustela nigripes NC024942 1	0,17845795
Phoca fasciata NC008428 1	Canis lupus familiaris NC002008 1231	0,1784687
Martes flavigula NC012141 3	Arctocephalus forsteri KT693377 17	0,17847234
Monachus schauinslandi NC008421 1	Canis lupus chanco NC010340 4	0,17847548
Procyon lotor AB462046 3	Eumetopias jubatus NC004030 10	0,17847939
Conepatus chinga NC042596 1	Arctonyx collaris NC020645 1	0,17848226
Arctonyx collaris NC020645 1	Arctocephalus townsendi NC008420 1	0,17848818
Ommatophoca rossii AY377287etc 1	Mungos mungo/gambianus SRR7704821 1	0,17849135
Procyon lotor AB462046 3	Conepatus chinga NC042596 1	0,17849153
Martes zibellina NC011579 39	Arctocephalus townsendi NC008420 1	0,17849246
Mustela erminea T305 2	Arctocephalus australis MG023139 1	0,17849305
Urocyon cinereoargenteus NC026723 21	Leptonychotes weddellii NC008424 1	0,17849599
Procyon lotor AB462049 4	Homotherium latidens MF871702 3	0,17850053
Pusa hispida NC 008433 1	Lycalopex sechurae KT448284 1	0,17850114
Ursus thibetanus mupinensis NC008753 2	Taxidea taxus NC020646 1	0,17850251
Mustela nigripes NC024942 1	Lynx canadensis NC028313 1	0,17850438
Pusa hispida NC 008433 1	Urva javanica T413 1	0,17850439
Mustela erminea T305 2	Felis nigripes NC028309 1	0,17850748
Neovison vison NC020641 3	Arctocephalus forsteri NC004023 28	0,17851304
Bassariscus sumichrasti SRX1099089 1	Arctocephalus forsteri NC004023 28	0,1785132
Phoca fasciata NC008428 1	Panthera uncia KP202269 1	0,17851353
Ursus maritimus NC003428 31	Mustela erminea T305 2	0,17851377
Pusa hispida NC 008433 1	Lycaon pictus NC028427 2	0,17851496
Pusa caspica NC008431 1	Panthera tigris amoyensis NC014770 2	0,17851533
Prionailurus bengalensis NC028301 12	Martes martes T302 3	0,17851582
Panthera leo spelaea KX258452 2	Cystophora cristata NC008427 1	0,17851853
Zalophus californianus NC008416 1	Ailurus fulgens styani NC009691 1	0,17852119
Ursus thibetanus formosanus NC009331 1	Mustela nigripes NC024942 1	0,17852262
Prionailurus bengalensis CKM45 20	Martes foina NC020643 1	0,17852451
Pusa hispida NC 008433 1	Canis lupus familiaris NC002008 1231	0,17852908
Monachus monachus NC044972 5	Atilax paludinosus T606 1	0,17853148
Vulpes vulpes NC008434 5	Monachus monachus NC044972 5	0,17853183
Mephitis mephitis NC020648 1	Bassariscus sumichrasti SRX1099089 1	0,17853387
Pusa caspica NC008431 1	Canis lupus chanco NC010340 4	0,17853579
Vulpes vulpes NC008434 5	Civettictis civetta NC033378 1	0,17853783
Viverra tangalunga MH464792 1	Spilogale putorius NC010497 1	0,1785394
Ursus spelaeus NC011112 8	Neovison vison NC020641 3	0,17853989
Mustela frenata NC020640 1	Civettictis civetta NC033378 1	0,17854033
Martes flavigula NC012141 3	Arctocephalus pusillus NC008417 1	0,1785406
Panthera onca NC022842 1	Monachus monachus NC044972 5	0,17855054
Crossarchus platycephalus C7R66 1	Canis adustus KT448271 1	0,17855853
Meles leucurus NC039173 4	Conepatus chinga NC042596 1	0,17855993
Nandinia binotata NC024567 1	Ailurus fulgens styani NC009691 1	0,17856258
Urocyon littoralis catalinae KP129018 15	Leptonychotes weddellii NC008424 1	0,17856327
Vulpes ferrilata NC027935 1	Leptonychotes weddellii NC008424 1	0,17856367
Martes pennanti NC020664 16	Arctodus simus NC011116 1	0,17856859
Ommatophoca rossii AY377287etc 1	Chrotogale owstoni T607 1	0,1785699
Otocyon megalotis SAF1 2	Cystophora cristata NC008427 1	0,17857113
Prionailurus bengalensis NC028301 12	Martes melampus NC009678 1	0,17857149
Mustela frenata NC020640 1	Felis nigripes NC028309 1	0,17857152
Ursus americanus JX196366 3	Ommatophoca rossii AY377287etc 1	0,17857191
Prionailurus planiceps NC028312 6	Mustela kathiah NC023210 1	0,17857201
Prionailurus planiceps KY682741 4	Mustela kathiah NC023210 1	0,17857213
Mustela putorius NC020638 4	Leopardus pardalis NC028315 1	0,17857231
Meles leucurus NC039173 4	Leopardus geoffroyi NC028320 1	0,17857262
Ursus maritimus NC003428 31	Ommatophoca rossii AY377287etc 1	0,1785745
Martes foina NC020643 1	Arctocephalus pusillus NC008417 1	0,17857605
Zalophus wollebaeki SRR4431565 1	Martes zibellina NC011579 39	0,17857707
Parahyaena brunnea NC038159 15	Mirounga leonina NC008422 1	0,17857768
Arctodus simus NC011116 1	Ailurus fulgens styani NC009691 1	0,17857789
Ursus spelaeus NC011112 8	Homotherium latidens MF871702 3	0,17857812
Phoca vitulina NC001325 1	Paguma larvata PDDS11 2	0,17858244
Leopardus pardalis T262 1	Bassariscus sumichrasti SRX1099089 1	0,17858294
Ursus arctos AP012576 6	Potos flavus T414 1	0,17858294
Pardofelis marmorata NLN3 2	Mirounga angustirostris SRR10331586 1	0,17858346
Leopardus pardalis NC028315 1	Arctodus simus NC011116 1	0,17858432

Odobenus rosmarus NC004029 29	Lynx canadensis NC028313 1	0,19159469
Urva javanica/auropunctata NC006835	Ailurus fulgens NC011124 1	0,19159479
Ursus arctos AP012576 6	Parahyaena brunnea NC038159 15	0,19159774
Ursus spelaeus NC011112 8	Panthera tigris amoyensis NC014770 2	0,19160162
Ursus thibetanus mupinensis NC00875	Odobenus rosmarus NC004029 29	0,19160222
Panthera onca NC022842 1	Melursus ursinus NC009970 2	0,19160584
Zalophus wollebaeki SRR4431565 1	Canis lupus familiaris NC002008 1231	0,19160832
Ursus americanus JX196366 3	Otocyon megalotis SAF1 2	0,19161014
Panthera pardus NC010641 5	Martes zibellina NC011579 39	0,19161418
Spilogale putorius NC010497 1	Prionodon pardicolor NC024569 2	0,19161777
Procyon lotor AB462049 4	Mellivora capensis T370 1	0,19162039
Phocarctos hookeri NC008418 1	Xenogale naso C07XAR110 1	0,19162463
Vulpes lagopus NC026529 3	Mungos mungo/gambianus SRR77048	0,19162512
Urocyon littoralis catalinae KP129018 1	Martes americana NC020642 1	0,19162525
Panthera tigris amoyensis NC014770 2	Arctocephalus forsteri NC004023 28	0,19162612
Paradoxurus hermaphroditus NC03959	Martes melampus NC009678 1	0,19162677
Lutrogale perspicillata NC035811 1	Atilax paludinosus T606 1	0,19162707
Mungotictis decemlineata NC027828 1	Arctocephalus townsendi NC008420 1	0,19162764
Suricata suricatta SSM10 1	Lycalopex sechurae KT448284 1	0,19162835
Pardofelis marmorata NLN3 2	Conepatus chinga NC042596 1	0,19162853
Vulpes corsac NC023958 1	Arctocephalus pusillus NC008417 1	0,19163011
Tremarctos ornatus NC009969 2	Diplogale hosei MH464790 1	0,19163318
Vulpes corsac NC023958 1	Ursus arctos EU497665 29	0,19163448
Melogale moschata V0735A 1	Ichneumia albicauda T603 1	0,19163485
Ursus maritimus NC003428 31	Prionodon linsang ERR2391707 1	0,19163567
Vulpes lagopus NC026529 3	Ursus thibetanus mupinensis NC00875	0,19163595
Ursus arctos isabellinus 18B5 2	Bdeogale nigripes GLC15 1	0,19163623
Galidia elegans D146 1	Arctotherium sp NC030174 1	0,19163646
Speothos venaticus C48 2	Mungos mungo/gambianus SRR77048	0,19163752
Panthera pardus japonensis KJ866876	Arctocephalus gazella BK010918 1	0,19163883
Panthera uncia KP202269 1	Lutrogale perspicillata NC035811 1	0,1916423
Ursus arctos GU573486 5	Xenogale naso C07XAR110 1	0,19164255
Prionailurus rubiginosus NC028304 2	Nasua nasua NC020647 1	0,19164361
Ursus arctos pruinosus MG066703 3	Galidictis fasciata DM333 1	0,19164641
Profelis aurata NC028299 1	Otocyon megalotis SAF1 2	0,19164695
Spilogale putorius NC010497 1	Arctocephalus forsteri NC004023 28	0,19164801
Vulpes lagopus NC026529 3	Mephitis mephitis NC020648 1	0,19164936
Prionailurus bengalensis CKM45 20	Bassaricyon neblina SRX1097850 1	0,19164979
Ursus arctos pruinosus MG066703 3	Otocolobus manul NC028323 1	0,19165152
Ursus arctos AP012576 6	Xenogale naso C07XAR110 1	0,19165273
Panthera leo spelaea KX258452 2	Lontra canadensis SRR10409165 1	0,19165319
Panthera leo spelaea KX258452 2	Neophoca cinerea NC008419 1	0,1916571
Zalophus wollebaeki SRR4431565 1	Canis mesomelas KT448280 1	0,1916604
Parahyaena brunnea NC038159 15	Canis lupus chanco NC010340 4	0,19167028
Spilogale putorius NC010497 1	Felis margarita NC028308 1	0,19167799
Mustela frenata NC020640 1	Tapirus terrestris T358	0,19167891
Spilogale putorius NC010497 1	Puma yagouaroundi NC028311 1	0,19167921
Galerella sanguinea T378 1	Conepatus chinga NC042596 1	0,19168073
Otocyon megalotis SAF1 2	Arctocephalus forsteri NC004023 28	0,19168341
Suricata suricatta SSM10 1	Melogale moschata V0735A 1	0,19168839
Suricata suricatta SSM10 1	Lontra canadensis SRR10409165 1	0,19168903
Vulpes vulpes NC008434 5	Arctocephalus australis MG023139 1	0,19169147
Galictis vittata T412 1	Canis lupus chanco NC010340 4	0,1916918
Canis anthus NC027956 2	Aonyx cinerea NC035814 2	0,19169459
Ursus spelaeus EU327344 13	Cuon alpinus NC013445 3	0,19169475
Paradoxurus hermaphroditus NC03959	Lutra sumatrana NC035810 1	0,19169602
Urocyon cinereoargenteus NC026723 2	Mustela eversmannii NC028013 1	0,19169742
Urocyon cinereoargenteus NC026723 2	Meles anakuma NC009677 1	0,19169743
Prionodon linsang ERR2391707 1	Arctotherium sp NC030174 1	0,19169895
Paradoxurus hermaphroditus NLNC 1	Martes zibellina NC011579 39	0,19169962
Otocyon megalotis SAF1 2	Eupleres goudotii D128 1	0,19170015
Hyaena hyaena NC020669 1	Ailurus fulgens styani NC009691 1	0,19170333
Otocyon megalotis SAF1 2	Meles anakuma NC009677 1	0,19170387
Phocarctos hookeri NC008418 1	Paradoxurus hermaphroditus NC03959	0,19170481
Panthera uncia NC010638 1	Nyctereutes procyonoides NC013700 3	0,19170603
Ursus thibetanus mupinensis NC00875	Nyctereutes procyonoides NC013700 3	0,19170693
Ursus arctos pruinosus MG066703 3	Xenogale naso C07XAR110 1	0,19171017
Proteles cristata T393 6	Mustela eversmannii NC028013 1	0,19171186
Crocuta crocuta NC020670 3	Arctocephalus australis MG023139 1	0,19171208
Arctocephalus gazella BK010918 1	Arctictis binturong T605 2	0,19171332
Galictis vittata T412 1	Eupleres goudotii D128 1	0,19171518
Ursus thibetanus formosanus NC00933	Suricata suricatta SSM10 1	0,19171552
Canis lupus chanco NC010340 4	Arctocephalus forsteri KT693377 17	0,19171575
Ursus thibetanus formosanus NC00933	Nasua nasua NC020647 1	0,19171771
Ursus spelaeus EU327344 13	Nyctereutes procyonoides NC013700 3	0,19171903
Proteles cristata T393 6	Arctotherium sp NC030174 1	0,1917204
Ursus arctos GU573491 207	Lycaon pictus NC028427 2	0,19172078
Melursus ursinus NC009970 2	Cryptoprocta ferox CFC13 1	0,19172366
Ursus arctos GU573486 5	Lycaon pictus NC028427 2	0,19172724
Ursus arctos isabellinus 18B5 2	Chrysocyon brachyurus NC024172 1	0,19172846
Panthera pardus japonensis KJ866876	Galictis vittata T412 1	0,19172965
Neofelis nebulosa NC008450 3	Lutra lutra NC011358 9	0,19173455
Lontra canadensis SRR10409165 1	Urva javanica/auropunctata NC006835	0,19173495
Speothos venaticus C48 2	Diplogale hosei MH464790 1	0,19173524
Zalophus californianus NC008416 1	Prionodon pardicolor NC024569 2	0,19173783
Ursus spelaeus NC011112 8	Panthera leo spelaea KX258452 2	0,19173832
Panthera pardus NC010641 5	Tapirus terrestris T358	0,19174051
Cuon alpinus NC013445 3	Arctocephalus gazella BK010918 1	0,19174276
Neophoca cinerea NC008419 1	Canis aureus KT448274 1	0,19174331
Zalophus californianus NC008416 1	Proteles cristata T393 6	0,1917444
Panthera pardus NC010641 5	Arctocephalus gazella BK010918 1	0,19174558
Meles meles T303 3	Tapirus terrestris T358	0,19174716
Spilogale putorius NC010497 1	Nyctereutes procyonoides NC013700 3	0,19174948
Ursus arctos GU573491 207	Panthera leo NERO 19	0,19175148
Panthera leo spelaea KX258452 2	Eumetopias jubatus NC004030 10	0,19175385
Vulpes corsac NC023958 1	Mungos mungo/gambianus SRR77048	0,19175935
Ursus thibetanus formosanus NC00933	Neofelis nebulosa NC008450 3	0,19176155
Ictonyx striatus T299 1	Cuon alpinus NC013445 3	0,19176586
Otocyon megalotis SAF1 2	Callorhinus ursinus NC008415 1	0,19176784
Vulpes lagopus NC026529 3	Ursus arctos isabellinus 18B5 2	0,19176865
Otocyon megalotis SAF1 2	Arctocephalus forsteri KT693377 17	0,19177037
Ursus arctos EU497665 29	Xenogale naso C07XAR110 1	0,19177094
Paradoxurus jerdoni MH464793 1	Aonyx cinerea NC035814 2	0,191774

Mirounga angustirostris SRR10331586 1	Galerella sanguinea T378 1	0,1785848
Viverricula indica KX891751 1	Bassariscus sumichrasti SRX1099089 1	0,17858543
Pusa caspica NC008431 1	Panthera onca NC022842 1	0,17858572
Erignathus barbatus NC008426 1	Arctictis binturong T605 2	0,17858654
Zalophus californianus NC008416 1	Ailurus fulgens NC011124 1	0,17858836
Phoca groenlandica NC008429 54	Arctictis binturong T605 2	0,17858905
Ursus thibetanus laniger MH281753 2	Mustela frenata NC020640 1	0,1785893
Ursus thibetanus laniger MH281753 2	Arctonyx collaris NC020645 1	0,17859104
Ursus thibetanus thibetanus NC011118 4	Mustela putorius NC020638 4	0,17859247
Ursus thibetanus thibetanus NC011118 4	Mustela itatsi NC034330 19	0,17859264
Eumetopias jubatus NC004030 10	Enhydra lutris NC009692 1	0,17859568
Cystophora cristata NC008427 1	Canis lupus chanco NC010340 4	0,17859824
Urva javanica/auropunctata NC006835 1	Canis adustus KT448271 1	0,17860018
Ursus thibetanus mupinensis NC008753 2	Hemigalus derbyanus MH464791 1	0,17860144
Taxidea taxus NC020646 1	Canis adustus KT448271 1	0,17860407
Spilogale putorius NC010497 1	Mustela sibirica AP017394 11	0,17860502
Pusa sibirica NC008432 2	Panthera pardus NC010641 5	0,17861234
Mustela itatsi NC034330 19	Callorhinus ursinus NC008415 1	0,17862344
Otaria byronia OTAB 1	Martes melampus NC009678 1	0,17862645
Pusa sibirica NC008432 2	Xenogale naso C07XAR110 1	0,17862915
Martes zibellina NC011579 39	Arctocephalus pusillus NC008417 1	0,17862947
Meles meles T303 3	Arctocephalus pusillus NC008417 1	0,17862992
Fossa fossana D350 1	Bassariscus sumichrasti SRX1099089 1	0,17863086
Potos flavus T414 1	Fossa fossana D350 1	0,17863092
Mustela sibirica NC020637 6	Canis adustus KT448271 1	0,17863244
Lutra lutra LC050126 1	Leopardus pardalis T262 1	0,17863686
Mustela sibirica AP017394 11	Hemigalus derbyanus MH464791 1	0,17863709
Otocyon megalotis SAF1 2	Erignathus barbatus NC008426 1	0,17863786
Prionailurus planiceps NC028312 6	Mustela frenata NC020640 1	0,17863855
Prionailurus rubiginosus NC028304 2	Martes melampus NC009678 1	0,1786388
Mustela sibirica NC020637 6	Leopardus pardalis NC028315 1	0,17863968
Taxidea taxus NC020646 1	Leptailurus serval NC028316 1	0,17864054
Mustela kathiah NC023210 1	Civettictis civetta GLC19 1	0,17864075
Prionailurus rubiginosus NC028304 2	Mustela nigripes NC024942 1	0,17864081
Ursus thibetanus thibetanus NC011118 4	Eumetopias jubatus NC004030 10	0,17864086
Paradoxurus hermaphroditus NLNC 1	Mustela erminea T305 2	0,17864136
Cystophora cristata NC008427 1	Canis aureus KT448274 1	0,17864233
Zalophus wollebaeki SRR4431565 1	Fossa fossana D350 1	0,17864762
Phoca fasciata NC008428 1	Panthera pardus japonensis KJ866876 8	0,17864794
Ursus thibetanus mupinensis NC008753 2	Mustela itatsi NC034330 19	0,17864837
Civettictis civetta GLC19 1	Bassariscus sumichrasti SRX1099089 1	0,17864865
Neovison vison NC020641 3	Lynx rufus NC014456 3	0,17864992
Zalophus wollebaeki SRR4431565 1	Neovison vison NC020641 3	0,17865404
Phoca largha NC008430 1	Panthera pardus japonensis KJ866876 8	0,17865536
Ursus thibetanus formosanus NC009331 1	Mustela itatsi NC034330 19	0,17865741
Panthera tigris amoyensis NC014770 2	Monachus schauinslandi NC008421 1	0,17866071
Ursus thibetanus mupinensis NC008753 2	Neovison vison NC020641 3	0,17866375
Ursus thibetanus thibetanus NC011118 4	Mustela nivalis T306 5	0,17866439
Mustela nigripes NC024942 1	Canis lupus familiaris NC002008 1231	0,17866671
Homotherium latidens MF871702 3	Gulo gulo NC009685 3	0,17868486
Meles meles T303 3	Arctocephalus australis MG023139 1	0,17869005
Procyon lotor AB462049 4	Conepatus chinga NC042596 1	0,1786928
Mustela frenata NC020640 1	Crossarchus platycephalus C7R66 1	0,17869616
Pusa caspica NC008431 1	Xenogale naso C07XAR110 1	0,17869647
Mustela frenata NC020640 1	Urva semitorquata MH464789 1	0,17869653
Mustela sibirica AP017394 11	Canis adustus KT448271 1	0,17869702
Pusa caspica NC008431 1	Canis aureus KT448274 1	0,17870106
Meles anakuma NC009677 1	Genetta servalina NC024568 2	0,17870443
Mustela frenata NC020640 1	Lynx rufus NC014456 3	0,17870595
Mustela kathiah NC023210 1	Leopardus jacobita NC028322 1	0,17870604
Vulpes corsac NC023958 1	Civettictis civetta GLC19 1	0,17870651
Paguma larvata PDDS11 2	Mustela putorius NC020638 4	0,17870684
Mustela sibirica NC020637 6	Leopardus wiedii NC028318 1	0,17870757
Melogale moschata KP726273 1	Lynx canadensis NC028313 1	0,1787076
Prionailurus rubiginosus NC028304 2	Mustela sibirica NC020637 6	0,178708
Phoca fasciata NC008428 1	Galidia elegans D146 1	0,17870839
Pusa sibirica NC008432 2	Panthera uncia NC010638 1	0,17870943
Phoca largha NC008430 1	Xenogale naso C07XAR110 1	0,17871055
Mustela kathiah NC023210 1	Leopardus geoffroyi NC028320 1	0,1787108
Ommatophoca rossii AY377287etc 1	Felis silvestris lybica KP202275 4	0,1787118
Mirounga angustirostris SRR10331586 1	Canis latrans NC008093 7	0,17871423
Zalophus californianus NC008416 1	Lutra lutra NC011358 9	0,17871875
Zalophus californianus NC008416 1	Lutra lutra LC050126 1	0,17871918
Mustela itatsi NC034330 19	Mephitis mephitis NC020648 1	0,17872004
Cystophora cristata NC008427 1	Chrysocyon brachyurus NC024172 1	0,17872134
Catopuma badia NC028300 1	Arctodus simus NC011116 1	0,17872231
Neovison vison NC020641 3	Leopardus guigna NC028321 1	0,17872286
Ursus thibetanus thibetanus NC011118 4	Mustela sibirica AP017394 11	0,17872423
Mustela erminea T305 2	Acinonyx jubatus NC005212 3	0,17872554
Mustela nigripes NC024942 1	Leopardus colocolo NC028314 1	0,17872579
Ursus thibetanus thibetanus NC011118 4	Arctonyx collaris NC020645 1	0,17872675
Viverra tangalunga MH464792 1	Genetta abyssinica MG489822 1	0,17873085
Phoca largha NC008430 1	Panthera tigris NC010642 35	0,17873113
Phoca vitulina NC001325 1	Panthera leo spelaea KX258452 2	0,17873399
Puma concolor NC016470 22	Canis adustus KT448271 1	0,17875488
Phoca largha NC008430 1	Panthera leo NERO 19	0,1787553
Viverricula indica KX891745 1	Canis mesomelas KT448280 1	0,17875567
Tremarctos ornatus NC009969 2	Mustela frenata NC020640 1	0,17876568
Martes martes T302 3	Arctocephalus forsteri KT693377 17	0,17876801
Neovison vison NC020641 3	Arctocephalus townsendi NC008420 1	0,17876893
Mustela erminea T305 2	Canis latrans NC008093 7	0,17877035
Neovison vison NC020641 3	Arctocephalus gazella BK010918 1	0,17877068
Puma concolor NC016470 22	Arctonyx collaris NC020645 1	0,17877144
Monachus schauinslandi NC008421 1	Urva semitorquata MH464789 1	0,17877448
Mirounga angustirostris SRR10331586 1	Canis aureus KT448274 1	0,17877546
Mustela erminea T305 2	Felis margarita NC028308 1	0,17877696
Phoca largha NC008430 1	Bdeogale nigripes GLC15 1	0,17877735
Potos flavus T414 1	Leopardus wiedii NC028318 1	0,17877781
Leptailurus serval NC028316 1	Canis aureus KT448274 1	0,1787779
Vulpes corsac NC023958 1	Hydrurga leptonyx NC008425 1	0,17877874
Ommatophoca rossii AY377287etc 1	Genetta genetta T297 1	0,17877948
Martes martes T302 3	Lynx canadensis NC028313 1	0,17878217
Otaria byronia OTAB 1	Arctodus simus NC011116 1	0,17878366

Phocarctos hookeri NC008418 1	Panthera uncia KP202269 1	0,19177462
Ursus arctos GU573491 207	Galidictis fasciata DM333 1	0,1917748
Urocyon littoralis catalinae KP129018 1	Leopardus jacobita NC028322 1	0,19177519
Urocyon cinereoargenteus NC026723 2	Leopardus jacobita NC028322 1	0,19177528
Urocyon littoralis catalinae KP129018 1	Leptailurus serval NC028316 1	0,19177625
Ursus arctos pruinosus MG066703 3	Canis anthus NC027956 2	0,19177635
Ursus arctos pruinosus MG066703 3	Prionodon linsang ERR2391707 1	0,19177665
Nasua nasua NC020647 1	Lynx rufus NC014456 3	0,19177693
Urocyon littoralis catalinae KP129018 1	Leopardus geoffroyi NC028320 1	0,19177717
Prionodon pardicolor NC024569 2	Ailurus fulgens NC011124 1	0,19177772
Puma concolor NC016470 22	Bassaricyon neblina SRX1097850 1	0,19177957
Speothos venaticus C48 2	Bassariscus sumichrasti SRX1099089 1	0,19178227
Ursus arctos AP012576 6	Mungos mungo/gambianus SRR77048	0,19178784
Lycalopex sechurae KT448284 1	Arctocephalus gazella BK010918 1	0,19178991
Ursus maritimus GU573488 Svalbard	Panthera tigris amoyensis NC014770 2	0,19179137
Proteles cristata T393 6	Ictonyx striatus T299 1	0,19179453
Panthera tigris amoyensis NC014770 2	Lycaon pictus NC028427 2	0,19179532
Mephitis mephitis NC020648 1	Leopardus guigna NC028321 1	0,19179742
Spilogale putorius NC010497 1	Lontra canadensis SRR10409165 1	0,19179976
Spilogale putorius NC010497 1	Arctocephalus australis MG023139 1	0,19179979
Ursus arctos GU573486 5	Panthera onca NC022842 1	0,1918
Ursus thibetanus formosanus NC00933	Panthera tigris NC010642 35	0,1918017
Zalophus wollebaeki SRR4431565 1	Panthera leo spelaea KX258452 2	0,19180636
Ursus spelaeus NC011112 8	Chrysocyon brachyurus NC024172 1	0,19180988
Arctocephalus forsteri KT693377 17	Tapirus terrestris T358	0,1918153
Gulo gulo NC009685 3	Crocuta crocuta NC020670 3	0,19181991
Canis anthus NC027956 2	Callorhinus ursinus NC008415 1	0,19182372
Vulpes ferrilata NC027935 1	Xenogale naso C07XAR110 1	0,19182654
Helogale parvula SRR7637809 1	Bassaricyon neblina SRX1097850 1	0,19182655
Vulpes zerda KJ603240 1	Xenogale naso C07XAR110 1	0,1918273
Phocarctos hookeri NC008418 1	Urva javanica T413 1	0,19182734
Vulpes corsac NC023958 1	Arctocephalus gazella BK010918 1	0,19182768
Galidia elegans D146 1	Bassaricyon neblina SRX1097850 1	0,19182774
Lutrogale perspicillata NC035811 1	Eupleres goudotii D128 1	0,19182833
Procyon lotor AB462046 3	Galidictis fasciata DM333 1	0,19183333
Procyon lotor AB462046 3	Prionodon linsang ERR2391707 1	0,19183429
Ursus arctos AP012576 6	Panthera leo NERO 19	0,19183522
Vulpes zerda KJ603240 1	Arctotherium sp NC030174 1	0,19183603
Speothos venaticus C48 2	Martes zibellina NC011579 39	0,19183762
Ursus arctos EU497665 29	Prionodon linsang ERR2391707 1	0,19183768
Panthera uncia NC010638 1	Martes melampus NC009678 1	0,1918378
Ursus arctos isabellinus 18B5 2	Cynictis penicillata T375 1	0,19183807
Otocyon megalotis SAF1 2	Ichneumia albicauda T603 1	0,19183815
Mustela nivalis T306 5	Cynogale bennetti KY117544 1	0,19183984
Phocarctos hookeri NC008418 1	Parahyaena brunnea NC038159 15	0,1918419
Lycalopex sechurae KT448284 1	Conepatus chinga NC042596 1	0,19184199
Speothos venaticus C48 2	Ichneumia albicauda T603 1	0,19184256
Viverricula indica NC025296 2	Bassaricyon neblina SRX1097850 1	0,19184528
Lutrogale perspicillata NC035811 1	Felis chaus NC028307 1	0,19184586
Felis nigripes NC028309 1	Bassaricyon neblina SRX1097850 1	0,19184712
Vulpes lagopus NC026529 3	Ursus thibetanus formosanus NC00933	0,19184732
Vulpes ferrilata NC027935 1	Ursus thibetanus formosanus NC00933	0,19184756
Ursus arctos pruinosus MG066703 3	Mungotictis decemlineata NC027828 1	0,19184797
Ursus arctos isabellinus 18B5 2	Panthera uncia NC010638 1	0,19185038
Prionodon pardicolor NC024569 2	Arctocephalus pusillus NC008417 1	0,19185074
Profelis aurata NC028299 1	Bassaricyon neblina SRX1097850 1	0,19185075
Panthera tigris amoyensis NC014770 2	Nyctereutes procyonoides NC013700 3	0,19185106
Ursus thibetanus laniger MH281753 2	Eupleres goudotii D128 1	0,19185287
Ursus arctos GU573486 5	Panthera uncia NC010638 1	0,19185732
Ursus arctos isabellinus 18B5 2	Panthera leo spelaea KX258452 2	0,19185928
Panthera tigris NC010642 35	Lycaon pictus NC028427 2	0,19186249
Ursus spelaeus NC011112 8	Panthera uncia NC010638 1	0,19186375
Ursus americanus JX196366 3	Eupleres goudotii D128 1	0,19186418
Canis mesomelas KT448280 1	Arctocephalus pusillus NC008417 1	0,19186454
Conepatus chinga NC042596 1	Canis lupus familiaris NC002008 1231	0,19186482
Lutra lutra LC050126 1	Diplogale hosei MH464790 1	0,19186959
Ursus thibetanus thibetanus NC01111	Panthera tigris amoyensis NC014770 2	0,19187145
Neofelis nebulosa NC008450 3	Ailurus fulgens styani NC009691 1	0,19187267
Neofelis nebulosa NC008450 3	Melogale moschata V0735A 1	0,19187392
Spilogale putorius NC010497 1	Phocarctos hookeri NC008418 1	0,19187394
Spilogale putorius NC010497 1	Leopardus jacobita NC028322 1	0,19187933
Panthera leo NERO 19	Martes pennanti NC020664 16	0,19188282
Spilogale putorius NC010497 1	Speothos venaticus C48 2	0,19188948
Vulpes corsac NC023958 1	Callorhinus ursinus NC008415 1	0,19189048
Panthera pardus NC010641 5	Martes pennanti NC020664 16	0,19189067
Vulpes vulpes NC008434 5	Callorhinus ursinus NC008415 1	0,19189101
Vulpes lagopus NC026529 3	Melogale moschata V0735A 1	0,19189294
Panthera leo NERO 19	Helarctos malayanus NC009968 2	0,19189835
Ursus thibetanus thibetanus NC01111	Neofelis nebulosa NC008450 3	0,19189872
Potos flavus T414 1	Otocyon megalotis SAF1 2	0,19190285
Suricata suricatta SSM10 1	Poecilogale albinucha T602 1	0,19190398
Ursus arctos isabellinus 18B5 2	Mungos mungo MMC7 1	0,19190523
Ursus arctos EU497665 29	Mungos mungo MMC7 1	0,19190556
Ursus maritimus NC003428 31	Mungos mungo MMC7 1	0,1919056
Ursus thibetanus mupinensis NC00875	Suricata suricatta SSM10 1	0,19190669
Vulpes corsac NC023958 1	Ursus arctos GU573486 5	0,19190958
Ursus arctos GU573491 207	Canis anthus NC027956 2	0,19191094
Nasua nasua NC020647 1	Leopardus pardalis T262 1	0,19191244
Panthera tigris amoyensis NC014770 2	Mustela itatsi NC034330 19	0,19191505
Crocuta crocuta NC020670 3	Arctocephalus gazella BK010918 1	0,1919152
Nyctereutes procyonoides NC013700 3	Melursus ursinus NC009970 2	0,19191907
Ursus arctos isabellinus 18B5 2	Lycaon pictus NC028427 2	0,19192307
Mephitis mephitis NC020648 1	Leopardus pardalis NC028315 1	0,19192398
Ursus arctos pruinosus MG066703 3	Galerella sanguinea T378 1	0,19192564
Ursus arctos AP012576 6	Galidictis fasciata DM333 1	0,19192727
Mephitis mephitis NC020648 1	Felis chaus NC028307 1	0,19192737
Ursus spelaeus NC011112 8	Otocyon megalotis SAF1 2	0,19192807
Odobenus rosmarus NC004029 29	Lynx pardinus NC028319 161	0,19193098
Eumetopias jubatus NC004030 10	Cynogale bennetti KY117544 1	0,19193134
Neofelis nebulosa NC008450 3	Tapirus terrestris T358	0,19193135
Nasua nasua NC020647 1	Leopardus colocolo NC028314 1	0,19193213
Spilogale putorius NC010497 1	Arctocephalus forsteri KT693377 17	0,19193463
Zalophus wollebaeki SRR4431565 1	Canis lupus chanco NC010340 4	0,19193521

Prionailurus rubiginosus NC028304 2	Martes martes T302 3	0,17878506
Nandinia binotata NC024567 1	Canis aureus KT448274 1	0,17878771
Viverricula indica KX891745 1	Lycaon pictus NC028427 2	0,17879038
Zalophus californianus NC008416 1	Mustela putorius NC020638 4	0,17879187
Viverricula indica NC025296 2	Lycaon pictus NC028427 2	0,17879203
Ursus thibetanus formosanus NC009331 1	Hemigalus derbyanus MH464791 1	0,17879441
Meles leucurus NC039173 4	Eumetopias jubatus NC004030 10	0,17879856
Ursus thibetanus thibetanus NC011118 4	Hemigalus derbyanus MH464791 1	0,17880423
Meles meles T303 3	Civettictis civetta NC033378 1	0,17880887
Leopardus geoffroyi NC028320 1	Canis adustus KT448271 1	0,17881456
Mustela kathiah NC023210 1	Civettictis civetta NC033378 1	0,17881577
Nasua nasua NC020647 1	Lutra sumatrana NC035810 1	0,17882393
Arctotherium sp NC030174 1	Arctocephalus australis MG023139 1	0,17882508
Spilogale putorius NC010497 1	Phoca fasciata NC008428 1	0,17882575
Halichoerus grypus NC001602 2	Felis chaus NC028307 1	0,17882659
Mirounga leonina NC008422 1	Cynictis penicillata T375 1	0,17883113
Pusa sibirica NC008432 2	Helogale parvula SRR7637809 1	0,17883113
Phocarctos hookeri NC008418 1	Mustela frenata NC020640 1	0,1788322
Galidia elegans D146 1	Cystophora cristata NC008427 1	0,17883395
Meles meles T303 3	Felis silvestris lybica KP202275 4	0,1788384
Paradoxurus hermaphroditus NLNC 1	Arctonyx collaris NC020645 1	0,17883974
Paradoxurus hermaphroditus NLNC 1	Meles leucurus NC039173 4	0,17883983
Vulpes vulpes NC008434 5	Civettictis civetta GLC19 1	0,17884068
Neovison vison NC020641 3	Atilax paludinosus T606 1	0,17884107
Ursus arctos EU497665 29	Potos flavus T414 1	0,17884113
Ursus maritimus NC003428 31	Potos flavus T414 1	0,17884117
Paguma larvata PDDS11 2	Mustela sibirica AP017394 11	0,17884159
Mirounga angustirostris SRR10331586 1	Bassaricyon neblina SRX1097850 1	0,1788426
Prionailurus bengalensis NC028301 12	Mustela sibirica NC020637 6	0,17884277
Prionailurus bengalensis CKM45 20	Meles meles T303 3	0,17884311
Phoca largha NC008430 1	Urva javanica T413 1	0,17884454
Mustela frenata NC020640 1	Arctictis binturong T605 2	0,17884968
Civettictis civetta GLC19 1	Arctodus simus NC011116 1	0,17885
Arctodus simus NC011116 1	Arctocephalus forsteri NC004023 28	0,17885219
Monachus schauinslandi NC008421 1	Lutrogale perspicillata NC035811 1	0,17885265
Prionailurus rubiginosus NC028304 2	Mustela nivalis T306 5	0,17885265
Phoca groenlandica NC008429 54	Ichneumia albicauda T603 1	0,17885384
Prionailurus bengalensis NC028301 12	Arctodus simus NC011116 1	0,17885457
Ursus thibetanus formosanus NC009331 1	Arctonyx collaris NC020645 1	0,17885822
Phoca largha NC008430 1	Hyaena hyaena NC020669 1	0,1788609
Ursus thibetanus thibetanus NC011118 4	Mustela eversmannii NC028013 1	0,17886123
Zalophus californianus NC008416 1	Fossa fossana D350 1	0,17886208
Spilogale putorius NC010497 1	Pusa sibirica NC008432 2	0,17886367
Mustela kathiah NC023210 1	Canis lupus familiaris NC002008 1231	0,17886987
Neovison vison NC020641 3	Acinonyx jubatus NC005212 3	0,17887019
Spilogale putorius NC010497 1	Mustela frenata NC020640 1	0,17887029
Paradoxurus jerdoni MH464793 1	Canis lupus familiaris NC002008 1231	0,17887448
Martes flavigula NC012141 3	Arctocephalus townsendi NC008420 1	0,17887507
Zalophus californianus NC008416 1	Ursus thibetanus thibetanus NC011118 4	0,17888033
Zalophus californianus NC008416 1	Ursus thibetanus laniger MH281753 2	0,17888223
Ommatophoca rossii AY377287etc 1	Eupleres goudotii D128 1	0,17888433
Mustela itatsi NC034330 19	Hemigalus derbyanus MH464791 1	0,17889406
Mirounga leonina NC008422 1	Bdeogale nigripes GLC15 1	0,17889847
Mustela frenata NC020640 1	Canis aureus KT448274 1	0,17889856
Lynx pardinus NC028319 161	Arctonyx collaris NC020645 1	0,17890565
Mustela nivalis T306 5	Crossarchus platycephalus C7R66 1	0,17890608
Spilogale putorius NC010497 1	Martes flavigula NC012141 3	0,17890796
Martes foina NC020643 1	Arctocephalus forsteri KT693377 17	0,17890958
Phoca vitulina NC001325 1	Urva javanica T413 1	0,17891198
Taxidea taxus NC020646 1	Mungotictis decemlineata NC027828 1	0,17891212
Prionailurus viverrinus NC028305 1	Potos flavus T414 1	0,17891225
Phoca vitulina NC001325 1	Xenogale naso C07XAR110 1	0,17891273
Leopardus tigrinus NC028317 1	Canis adustus KT448271 1	0,1789159
Salanoia concolor D378 1	Ommatophoca rossii AY377287etc 1	0,17891636
Ursus thibetanus laniger MH281753 2	Taxidea taxus NC020646 1	0,17891638
Pusa caspica NC008431 1	Crocuta crocuta NC020670 3	0,17891739
Phoca largha NC008430 1	Paguma larvata PDDS11 2	0,17891891
Ursus thibetanus formosanus NC009331 1	Mustela sibirica NC020637 6	0,17892389
Phoca fasciata NC008428 1	Parahyaena brunnea NC038159 15	0,17892396
Phoca fasciata NC008428 1	Panthera tigris amoyensis NC014770 2	0,17892554
Ursus thibetanus formosanus NC009331 1	Mustela putorius NC020638 4	0,17892621
Ursus spelaeus NC011112 8	Mustela sibirica AP017394 11	0,17892659
Ursus thibetanus thibetanus NC011118 4	Mustela frenata NC020640 1	0,17892714
Homotherium latidens MF871702 3	Chrysocyon brachyurus NC024172 1	0,1789286
Mephitis mephitis NC020648 1	Ailurus fulgens styani NC009691 1	0,17893118
Nandinia binotata NC024567 1	Canis lupus chanco NC010340 4	0,17893143
Melogale moschata NC020644 1	Leopardus colocolo NC028314 1	0,17893587
Mustela eversmannii NC028013 1	Eumetopias jubatus NC004030 10	0,17893627
Prionailurus bengalensis CKM45 20	Canis adustus KT448271 1	0,17893699
Phoca groenlandica NC008429 54	Urva javanica/auropunctata NC006835 1	0,17894241
Panthera uncia KP202269 1	Monachus monachus NC044972 5	0,17894264
Neofelis nebulosa NC008450 3	Cystophora cristata NC008427 1	0,1789438
Ursus arctos GU573486 5	Ommatophoca rossii AY377287etc 1	0,17894787
Nandinia binotata NC024567 1	Conepatus chinga NC042596 1	0,17895249
Spilogale putorius NC010497 1	Poecilogale albinucha T602 1	0,17895658
Civettictis civetta NC033378 1	Bassariscus sumichrasti SRX1099089 1	0,17895977
Mustela erminea T305 2	Chrotogale owstoni T607 1	0,17896083
Viverra tangalunga MH464792 1	Homotherium latidens MF871702 3	0,17896371
Ommatophoca rossii AY377287etc 1	Catopuma temminckii NC027115 41	0,178964
Chrotogale owstoni T607 1	Ailurus fulgens styani NC009691 1	0,17896472
Vulpes corsac NC023958 1	Cystophora cristata NC008427 1	0,17896616
Homotherium latidens MF871702 3	Callorhinus ursinus NC008415 1	0,17896768
Neovison vison NC020641 3	Diplogale hosei MH464790 1	0,17896822
Prionailurus planiceps KY682741 4	Canis adustus KT448271 1	0,17896953
Meles meles T303 3	Lynx pardinus NC028319 161	0,17897171
Leopardus tigrinus NC028317 1	Arctonyx collaris NC020645 1	0,1789728
Lutra lutra NC011358 9	Leopardus pardalis T262 1	0,17897362
Pusa caspica NC008431 1	Paguma larvata PDDS11 2	0,17897451
Phoca groenlandica NC008429 54	Bdeogale nigripes GLC15 1	0,17897563
Viverricula indica NC025296 2	Meles leucurus NC039173 4	0,17897612
Mustela nigripes NC024942 1	Leopardus pardalis T262 1	0,17897664
Meles meles T303 3	Catopuma badia NC028300 1	0,17897671
Mustela putorius NC020638 4	Leopardus wiedii NC028318 1	0,17897692

Otaria byronia OTAB 1	Urva javanica/auropunctata NC006835	0,19194053
Phocarctos hookeri NC008418 1	Urva javanica/auropunctata NC006835	0,19194174
Nyctereutes procyonoides NC013700 3	Neofelis nebulosa NC008450 3	0,19194566
Spilogale putorius NC010497 1	Mungos mungo MMC7 1	0,19194685
Phocarctos hookeri NC008418 1	Panthera pardus NC010641 5	0,19194895
Odobenus rosmarus NC004029 29	Melursus ursinus NC009970 2	0,19195189
Panthera pardus NC010641 5	Arctotherium sp NC030174 1	0,1919547
Martes zibellina NC011579 39	Diplogale hosei MH464790 1	0,19195834
Urocyon littoralis catalinae KP129018	Mungos mungo MMC7 1	0,19196248
Canis anthus NC027956 2	Arctocephalus gazella BK010918 1	0,1919625
Mustela sibirica NC020637 6	Cynogale bennetti KY117544 1	0,19196351
Phocarctos hookeri NC008418 1	Nyctereutes procyonoides NC013700 3	0,19196419
Panthera uncia KP202269 1	Arctocephalus forsteri NC004023 28	0,19196551
Urocyon littoralis catalinae KP129018	Mustela kathiah NC023210 1	0,19196782
Potos flavus T414 1	Lycalopex sechurae KT448284 1	0,19196968
Vulpes ferrilata NC027935 1	Ursus arctos isabellinus 18B5 2	0,19197118
Ursus maritimus NC003428 31	Cynictis penicillata T375 1	0,19197293
Ursus arctos isabellinus 18B5 2	Atilax paludinosus T606 1	0,19197297
Ursus arctos EU497665 29	Bdeogale nigripes GLC15 1	0,19197315
Panthera pardus NC010641 5	Galictis vittata T412 1	0,19197331
Speothos venaticus C48 2	Galerella sanguinea T378 1	0,19197364
Diplogale hosei MH464790 1	Arctocephalus townsendi NC008420 1	0,19197389
Panthera pardus japonensis KJ866876	Arctocephalus forsteri KT693377 17	0,191974
Vulpes ferrilata NC027935 1	Panthera uncia NC010638 1	0,19197412
Lycaon pictus NC028427 2	Galidia elegans D146 1	0,19197538
Phocarctos hookeri NC008418 1	Hyaena hyaena NC020669 1	0,19197589
Urocyon cinereoargenteus NC026723 2	Leopardus tigrinus NC028317 1	0,19197626
Martes foina NC020643 1	Cynogale bennetti KY117544 1	0,19197702
Ursus arctos GU573486 5	Mungos mungo MMC7 1	0,1919795
Ursus arctos isabellinus 18B5 2	Otocyon megalotis SAF1 2	0,19198309
Ursus spelaeus EU327344 13	Urva semitorquata MH464789 1	0,19198382
Vulpes corsac NC023958 1	Panthera leo spelaea KX258452 2	0,19198579
Zalophus californianus NC008416 1	Prionodon linsang ERR2391707 1	0,19198824
Ursus spelaeus EU327344 13	Urva javanica T413 1	0,19199045
Panthera uncia KP202269 1	Ailuropoda melanoleuca NC009492 5	0,19199097
Ursus arctos pruinosus MG066703 3	Prionodon pardicolor NC024569 2	0,19199117
Panthera onca NC022842 1	Arctotherium sp NC030174 1	0,19199176
Odobenus rosmarus NC004029 29	Mustela altaica NC021751 1	0,19199272
Ursus maritimus NC003428 31	Panthera tigris amoyensis NC014770 2	0,19199321
Ursus thibetanus mupinensis NC00875	Panthera tigris NC010642 35	0,19199395
Ursus arctos isabellinus 18B5 2	Panthera onca NC022842 1	0,19199519
Paradoxurus hermaphroditus NC03959	Melursus ursinus NC009970 2	0,19199792
Otaria byronia OTAB 1	Mephitis mephitis NC020648 1	0,19199854
Ursus arctos AP012576 6	Panthera uncia KP202269 1	0,19200179
Ursus americanus JX196366 3	Suricata suricatta SSM10 1	0,19200449
Ursus arctos isabellinus 18B5 2	Odobenus rosmarus NC004029 29	0,19200504
Ursus thibetanus thibetanus NC011118	Panthera tigris NC010642 35	0,19200607
Vulpes vulpes NC008434 5	Spilogale putorius NC010497 1	0,1920069
Panthera pardus NC010641 5	Arctocephalus forsteri KT693377 17	0,19201332
Otaria byronia OTAB 1	Canis aureus KT448274 1	0,19201522
Urva javanica T413 1	Arctocephalus forsteri NC004023 28	0,19201697
Spilogale putorius NC010497 1	Lutrogale perspicillata NC035811 1	0,19201712
Ursus thibetanus mupinensis NC00875	Neofelis nebulosa NC008450 3	0,19202156
Panthera pardus NC010641 5	Lutrogale perspicillata NC035811 1	0,19202188
Galictis vittata T412 1	Canis lupus familiaris NC002008 1231	0,19202219
Suricata suricatta SSM10 1	Meles anakuma NC009677 1	0,19202399
Ursus arctos GU573491 207	Panthera pardus NC010641 5	0,19202451
Vulpes ferrilata NC027935 1	Eupleres goudotii D128 1	0,19202635
Suricata suricatta SSM10 1	Phocarctos hookeri NC008418 1	0,19202811
Canis anthus NC027956 2	Arctocephalus australis MG023139 1	0,19202846
Urocyon cinereoargenteus NC026723 2	Mungos mungo MMC7 1	0,19202991
Urocyon littoralis catalinae KP129018	Arctocephalus pusillus NC008417 1	0,19203423
Urocyon cinereoargenteus NC026723 2	Arctocephalus pusillus NC008417 1	0,19203442
Vulpes zerda KJ603240 1	Otaria byronia OTAB 1	0,19203518
Tremarctos ornatus NC009969 2	Nasua nasua NC020647 1	0,19203523
Procyon lotor AB462049 4	Mungotictis decemlineata NC027828 1	0,19203526
Speothos venaticus C48 2	Lontra canadensis SRR10409165 1	0,19203934
Ursus arctos GU573491 207	Atilax paludinosus T606 1	0,19204038
Ursus maritimus GU573488 Svalbard	Atilax paludinosus T606 1	0,19204049
Panthera uncia NC010638 1	Arctocephalus australis MG023139 1	0,19204107
Tremarctos ornatus NC009969 2	Panthera uncia NC010638 1	0,19204342
Vulpes vulpes NC008434 5	Parahyaena brunnea NC038159 15	0,19204421
Tremarctos ornatus NC009969 2	Speothos venaticus C48 2	0,19204451
Urocyon cinereoargenteus NC026723 2	Leopardus guigna NC028321 1	0,19204625
Ursus arctos pruinosus MG066703 3	Mungos mungo/gambianus SRR77048	0,19204709
Nasua nasua NC020647 1	Felis margarita NC028308 1	0,19204739
Arctocephalus townsendi NC008420 1	Arctictis binturong T605 2	0,19204804
Proteles cristata T393 6	Lutrogale perspicillata NC035811 1	0,19204947
Otocyon megalotis SAF1 2	Diplogale hosei MH464790 1	0,19205084
Ursus thibetanus formosanus NC0093	Urva brachyura KY117547 1	0,19205247
Vulpes ferrilata NC027935 1	Panthera leo spelaea KX258452 2	0,19205515
Speothos venaticus C48 2	Prionodon pardicolor NC024569 2	0,19205642
Ursus arctos AP012576 6	Cynictis penicillata T375 1	0,19205643
Ursus arctos pruinosus MG066703 3	Parahyaena brunnea NC038159 15	0,19205764
Mephitis mephitis NC020648 1	Felis silvestris lybica KP202275 4	0,19205987
Ursus arctos EU497665 29	Panthera leo spelaea KX258452 2	0,19206139
Ursus arctos GU573491 207	Odobenus rosmarus NC004029 29	0,19207253
Neofelis nebulosa NC008450 3	Callorhinus ursinus NC008415 1	0,19207491
Enhydra lutris NC009692 1	Cynogale bennetti KY117544 1	0,19208431
Vulpes lagopus NC026529 3	Callorhinus ursinus NC008415 1	0,19209334
Vulpes corsac NC023958 1	Arctocephalus forsteri KT693377 17	0,19209534
Vulpes ferrilata NC027935 1	Urva javanica T413 1	0,19209595
Procyon lotor AB462049 4	Galidictis fasciata DM333 1	0,19210227
Chrotogale owstoni T607 1	Aonyx cinerea NC035814 2	0,1921026
Vulpes zerda KJ603240 1	Chrotogale owstoni T607 1	0,19210288
Galidictis fasciata DM333 1	Tapirus terrestris T358	0,19210385
Otocyon megalotis SAF1 2	Xenogale naso C07XAR110 1	0,19210477
Speothos venaticus C48 2	Cynogale bennetti KY117544 1	0,19210529
Otocyon megalotis SAF1 2	Galidia elegans D146 1	0,1921088
Vulpes zerda KJ603240 1	Panthera uncia KP202269 1	0,19210935
Ursus thibetanus mupinensis NC00875	Nasua nasua NC020647 1	0,19211103
Urocyon cinereoargenteus NC026723 2	Prionodon pardicolor NC024569 2	0,19211122
Lycalopex sechurae KT448284 1	Xenogale naso C07XAR110 1	0,19211133

Vulpes vulpes NC008434 5	Viverricula indica NC025296 2	0,17897736
Vulpes vulpes NC008434 5	Pusa hispida NC 008433 1	0,17897758
Nandinia binotata NC024567 1	Lycaon pictus NC028427 2	0,17897825
Phoca vitulina NC001325 1	Canis latrans NC008093 7	0,17897908
Prionailurus bengalensis CKM45 20	Ailurus fulgens styani NC009691 1	0,17897922
Taxidea taxus NC020646 1	Felis margarita NC028308 1	0,17897958
Taxidea taxus NC020646 1	Salanoia concolor D378 1	0,17897981
Prionailurus rubiginosus NC028304 2	Martes zibellina NC011579 39	0,17897999
Ursus arctos EU497665 29	Mustela erminea T305 2	0,17898531
Mirounga leonina NC008422 1	Chrysocyon brachyurus NC024172 1	0,17898588
Phoca fasciata NC008428 1	Lycaon pictus NC028427 2	0,1789868
Prionailurus planiceps KY682741 4	Mustela nivalis T306 5	0,17898709
Martes martes T302 3	Felis silvestris lybica KP202275 4	0,17898731
Meles meles T303 3	Acinonyx jubatus NC005212 3	0,17898941
Monachus schauinslandi NC008421 1	Lycaon pictus NC028427 2	0,17899053
Ursus spelaeus EU327344 13	Nandinia binotata NC024567 1	0,17899092
Martes foina NC020643 1	Felis silvestris lybica KP202275 4	0,17899188
Mustela nigripes NC024942 1	Acinonyx jubatus NC005212 3	0,17899306
Ursus thibetanus laniger MH281753 2	Mustela nigripes NC024942 1	0,17899504
Panthera leo NERO 19	Halichoerus grypus NC001602 2	0,17899682
Smilodon populator MF871700 1	Enhydra lutris NC009692 1	0,17899759
Ursus americanus JX196366 3	Eumetopias jubatus NC004030 10	0,17899847
Lobodon carcinophaga NC008423 1	Crocuta crocuta NC020670 3	0,17900006
Monachus monachus NC044972 5	Galidictis fasciata DM333 1	0,17900962
Halichoerus grypus NC001602 2	Atilax paludinosus T606 1	0,17901514
Lutra lutra NC011358 9	Fossa fossana D350 1	0,17902577
Suricata suricatta SSM10 1	Mirounga leonina NC008422 1	0,17903313
Mustela frenata NC020640 1	Canis anthus NC027956 2	0,17903602
Mustela itatsi NC034330 19	Arctocephalus pusillus NC008417 1	0,17903662
Martes martes T302 3	Arctocephalus townsendi NC008420 1	0,17903729
Prionailurus rubiginosus NC028304 2	Enhydra lutris NC009692 1	0,17903933
Meles leucurus NC039173 4	Leopardus wiedii NC028318 1	0,17903955
Paradoxurus jerdoni MH464793 1	Cystophora cristata NC008427 1	0,17904177
Puma yagouaroundi NC028311 1	Mustela frenata NC020640 1	0,17904209
Paradoxurus hermaphroditus NC039591 1	Arctonyx collaris NC020645 1	0,17904238
Mustela frenata NC020640 1	Lynx lynx NC027083 4	0,17904279
Phoca fasciata NC008428 1	Xenogale naso C07XAR110 1	0,17904282
Pusa hispida NC 008433 1	Helogale parvula SRR7637809 1	0,17904297
Phoca groenlandica NC008429 54	Atilax paludinosus T606 1	0,17904307
Phoca fasciata NC008428 1	Canis mesomelas KT448280 1	0,17904323
Paguma larvata PDDS11 2	Mustela eversmannii NC028013 1	0,17904325
Phocarctos hookeri NC008418 1	Arctodus simus NC011116 1	0,1790448
Martes americana NC020642 1	Lynx canadensis NC028313 1	0,17904501
Prionailurus viverrinus NC028305 1	Martes melampus NC009678 1	0,1790451
Otocolobus manul NC028323 1	Mustela nigripes NC024942 1	0,17904562
Phoca groenlandica NC008429 54	Galidictis fasciata DM333 1	0,17904641
Mirounga leonina NC008422 1	Ichneumia albicauda T603 1	0,17904645
Homotherium latidens MF871702 3	Arctocephalus pusillus NC008417 1	0,17904669
Phoca vitulina NC001325 1	Bdeogale nigripes GLC15 1	0,17904688
Neovison vison NC020641 3	Arctocephalus pusillus NC008417 1	0,17904742
Prionailurus bengalensis CKM45 20	Mustela nigripes NC024942 1	0,1790489
Urocyon cinereoargenteus NC026723 21	Lobodon carcinophaga NC008423 1	0,17905016
Ursus thibetanus mupinensis NC008753 2	Mustela nigripes NC024942 1	0,17905188
Martes pennanti NC020664 16	Lynx pardinus NC028319 161	0,17905231
Arctodus simus NC011116 1	Aonyx cinerea NC035814 2	0,17905329
Ursus thibetanus thibetanus NC011118 4	Taxidea taxus NC020646 1	0,17905338
Helarctos malayanus NC009968 2	Arctocephalus forsteri KT693377 17	0,17905623
Neovison vison NC020641 3	Arctotherium sp NC030174 1	0,17905915
Ursus spelaeus NC011112 8	Hemigalus derbyanus MH464791 1	0,17905951
Ursus thibetanus laniger MH281753 2	Mustela sibirica NC020637 6	0,17905964
Neophoca cinerea NC008419 1	Helarctos malayanus NC009968 2	0,17905982
Phoca groenlandica NC008429 54	Chrysocyon brachyurus NC024172 1	0,17906715
Phoca vitulina NC001325 1	Panthera tigris amoyensis NC014770 2	0,17906775
Arctodus simus NC011116 1	Acinonyx jubatus NC005212 3	0,17907155
Martes flavigula NC012141 3	Homotherium latidens MF871702 3	0,17907207
Monachus monachus NC044972 5	Cryptoprocta ferox CFC13 1	0,17908448
Ursus maritimus GU573488 Svalbard	Ommatophoca rossii AY377287etc 1	0,17908667
Zalophus californianus NC008416 1	Gulo gulo NC009685 3	0,17908669
Arctotherium sp NC030174 1	Arctocephalus forsteri KT693377 17	0,17909457
Lutra lutra LC050126 1	Arctocephalus gazella BK010918 1	0,17909463
Otaria byronia OTAB 1	Lutra lutra NC011358 9	0,17909697
Phoca vitulina NC001325 1	Panthera pardus NC010641 5	0,17909725
Panthera uncia NC010638 1	Halichoerus grypus NC001602 2	0,17909871
Ursus arctos EU497665 29	Ommatophoca rossii AY377287etc 1	0,17910123
Nyctereutes procyonoides NC013700 3	Cystophora cristata NC008427 1	0,17910223
Mustela kathiah NC023210 1	Homotherium latidens MF871702 3	0,17910728
Puma concolor NC016470 22	Meles meles T303 3	0,17910796
Smilodon populator MF871700 1	Arctonyx collaris NC020645 1	0,17910935
Prionailurus bengalensis CKM45 20	Enhydra lutris NC009692 1	0,17911077
Martes melampus NC009678 1	Felis silvestris lybica KP202275 4	0,17911084
Arctotherium sp NC030174 1	Arctonyx collaris NC020645 1	0,17911102
Viverra zibetha T609 1	Mustela sibirica AP017394 11	0,17911114
Paguma larvata PDDS11 2	Mustela nigripes NC024942 1	0,17911132
Viverricula indica NC025296 2	Meles meles T303 3	0,17911163
Zalophus wollebaeki SRR4431565 1	Lutra sumatrana NC035810 1	0,17911288
Pusa caspica NC008431 1	Panthera uncia NC010638 1	0,17911342
Prionailurus planiceps KY682741 4	Neovison vison NC020641 3	0,17912322
Prionailurus bengalensis NC028301 12	Neovison vison NC020641 3	0,17912427
Mustela nivalis T306 5	Leopardus geoffroyi NC028320 1	0,17912447
Zalophus californianus NC008416 1	Meles leucurus NC039173 4	0,17912585
Smilodon populator MF871700 1	Ommatophoca rossii AY377287etc 1	0,17912634
Prionailurus planiceps KY682741 4	Martes foina NC020643 1	0,17912677
Parahyaena brunnea NC038159 15	Mirounga angustirostris SRR10331586 1	0,17912786
Phoca fasciata NC008428 1	Arctictis binturong T605 2	0,17912817
Ursus spelaeus NC011112 8	Mustela sibirica NC020637 6	0,17912853
Ursus thibetanus laniger MH281753 2	Mustela putorius NC020638 4	0,1791295
Procyon lotor AB462046 3	Homotherium latidens MF871702 3	0,17913744
Spilogale putorius NC010497 1	Mustela nigripes NC024942 1	0,17914477
Monachus monachus NC044972 5	Lycaon pictus NC028427 2	0,17914999
Lobodon carcinophaga NC008423 1	Tapirus terrestris T358	0,179157
Meles leucurus NC039173 4	Chrotogale owstoni T607 1	0,17916188
Arctocephalus australis MG023139 1	Ailurus fulgens styani NC009691 1	0,17916384
Suricata suricatta SSM10 1	Pusa caspica NC008431 1	0,17916779

Zalophus californianus NC008416 1	Cuon alpinus NC013445 3	0,19211231
Ursus arctos GU573486 5	Prionodon linsang ERR2391707 1	0,19211313
Melursus ursinus NC009970 2	Urva semitorquata MH464789 1	0,19211647
Proteles cristata T393 6	Arctocephalus forsteri KT693377 17	0,1921176
Otocolobus manul NC028323 1	Bassaricyon neblina SRX1097850 1	0,19211836
Ursus arctos pruinosus MG066703 3	Panthera uncia KP202269 1	0,19212024
Ursus spelaeus EU327344 13	Atilax paludinosus T606 1	0,19212091
Ursus spelaeus NC011112 8	Urva brachyura KY117547 1	0,19212139
Panthera leo spelaea KX258452 2	Martes pennanti NC020664 16	0,19212456
Panthera uncia NC010638 1	Lycalopex sechurae KT448284 1	0,19213042
Spilogale putorius NC010497 1	Atilax paludinosus T606 1	0,19213934
Prionodon linsang ERR2391707 1	Tapirus terrestris T358	0,19214706
Vulpes corsac NC023958 1	Panthera pardus NC010641 5	0,19215162
Neofelis nebulosa NC008450 3	Martes pennanti NC020664 16	0,19215237
Mellivora capensis T370 1	Cystophora cristata NC008427 1	0,19215806
Lycaon pictus NC028427 2	Arctocephalus forsteri NC004023 28	0,19215854
Hyaena hyaena NC020669 1	Arctocephalus forsteri NC004023 28	0,19216107
Urocyon littoralis catalinae KP129018	Callorhinus ursinus NC008415 1	0,19216256
Suricata suricatta SSM10 1	Arctocephalus gazella BK010918 1	0,19216365
Vulpes ferrilata NC027935 1	Aonyx cinerea NC035814 2	0,19216555
Paradoxurus hermaphroditus NLNC 1	Arctocephalus townsendi NC008420 1	0,19216581
Lutrogale perspicillata NC035811 1	Helogale parvula SRR7637809 1	0,19216599
Paradoxurus hermaphroditus NC03959	Arctocephalus gazella BK010918 1	0,19216742
Vulpes lagopus NC026529 3	Ursus arctos EU497665 29	0,19217271
Vulpes ferrilata NC027935 1	Martes foina NC020643 1	0,192177
Hyaena hyaena NC020669 1	Arctocephalus townsendi NC008420 1	0,1921773
Panthera pardus japonensis KJ866876	Lutrogale perspicillata NC035811 1	0,19218047
Zalophus wollebaeki SRR4431565 1	Vulpes ferrilata NC027935 1	0,19218074
Urocyon littoralis catalinae KP129018	Leopardus guigna NC028321 1	0,19218084
Leopardus jacobita NC028322 1	Bassaricyon neblina SRX1097850 1	0,19218479
Urva javanica T413 1	Ailuropoda melanoleuca NC009492 5	0,19218479
Ursus arctos GU573491 207	Otocyon megalotis SAF1 2	0,19218497
Ursus arctos isabellinus 18B5 2	Galerella sanguinea T378 1	0,19218677
Ursus spelaeus EU327344 13	Urva brachyura KY117547 1	0,19218871
Prionodon linsang ERR2391707 1	Melursus ursinus NC009970 2	0,19219024
Phocarctos hookeri NC008418 1	Panthera onca NC022842 1	0,19219038
Neofelis nebulosa NC008450 3	Martes flavigula NC012141 3	0,1921948
Zalophus wollebaeki SRR4431565 1	Prionodon pardicolor NC024569 2	0,19219522
Ursus thibetanus mupinensis NC00875	Panthera tigris amoyensis NC014770 2	0,19219621
Ursus arctos AP012576 6	Otocyon megalotis SAF1 2	0,19220123
Odobenus rosmarus NC004029 29	Leopardus guigna NC028321 1	0,19220185
Ursus maritimus GU573488 Svalbard	Cuon alpinus NC013445 3	0,19221909
Spilogale putorius NC010497 1	Nasua nasua NC020647 1	0,19222105
Galidia elegans D146 1	Arctocephalus forsteri NC004023 28	0,19222601
Helogale parvula SRR7637809 1	Arctocephalus townsendi NC008420 1	0,19222905
Urocyon cinereoargenteus NC026723 2	Callorhinus ursinus NC008415 1	0,19222985
Ursus americanus JX196366 3	Panthera leo spelaea KX258452 2	0,19223168
Vulpes zerda KJ603240 1	Bdeogale nigripes GLC15 1	0,19223182
Vulpes corsac NC023958 1	Galidia elegans D146 1	0,19223361
Neofelis nebulosa NC008450 3	Canis aureus KT448274 1	0,19223605
Vulpes zerda KJ603240 1	Arctonyx collaris NC020645 1	0,19223621
Ursus arctos GU573491 207	Prionodon linsang ERR2391707 1	0,19224156
Panthera uncia NC010638 1	Arctocephalus forsteri KT693377 17	0,19224304
Chrysocyon brachyurus NC024172 1	Arctocephalus australis MG023139 1	0,1922455
Urva semitorquata MH464789 1	Ailuropoda melanoleuca NC009492 5	0,19225048
Parahyaena brunnea NC038159 15	Ailurus fulgens styani NC009691 1	0,19225051
Neophoca cinerea NC008419 1	Crocuta crocuta NC020670 3	0,19225065
Odobenus rosmarus NC004029 29	Mustela eversmannii NC028013 1	0,19225268
Panthera uncia NC010638 1	Martes martes T302 3	0,19225337
Vulpes ferrilata NC027935 1	Ursus spelaeus NC011112 8	0,19225383
Ursus arctos GU573491 207	Panthera uncia KP202269 1	0,19225463
Vulpes zerda KJ603240 1	Arctictis binturong T605 2	0,19225523
Ursus spelaeus EU327344 13	Suricata suricatta SSM10 1	0,19225663
Mephitis mephitis NC020648 1	Helogale parvula SRR7637809 1	0,19226031
Mephitis mephitis NC020648 1	Canis anthus NC027956 2	0,1922618
Felis nigripes NC028309 1	Ailuropoda melanoleuca NC009492 5	0,19226234
Ursus maritimus GU573488 Svalbard	Panthera tigris NC010642 35	0,19226277
Zalophus californianus NC008416 1	Vulpes ferrilata NC027935 1	0,19226336
Mephitis mephitis NC020648 1	Lynx rufus NC014456 3	0,19226791
Spilogale putorius NC010497 1	Mungos mungo/gambianus SRR77048	0,19228485
Urocyon littoralis catalinae KP129018	Eumetopias jubatus NC004030 10	0,19228493
Panthera uncia KP202269 1	Cuon alpinus NC013445 3	0,19228587
Arctocephalus gazella BK010918 1	Tapirus terrestris T358	0,19228789
Procyon lotor AB462046 3	Neofelis nebulosa NC008450 3	0,19228804
Vulpes lagopus NC026529 3	Conepatus chinga NC042596 1	0,19229127
Spilogale putorius NC010497 1	Leopardus guigna NC028321 1	0,19229157
Lontra canadensis SRR10409165 1	Conepatus chinga NC042596 1	0,19229396
Mustela erminea T305 2	Tapirus terrestris T358	0,19229487
Lontra canadensis SRR10409165 1	Urva brachyura KY117547 1	0,19229516
Vulpes ferrilata NC027935 1	Callorhinus ursinus NC008415 1	0,19229542
Diplogale hosei MH464790 1	Arctocephalus forsteri NC004023 28	0,1922989
Melogale moschata KP726273 1	Cynogale bennetti KY117544 1	0,19229956
Mustela eversmannii NC028013 1	Cynogale bennetti KY117544 1	0,19230041
Vulpes lagopus NC026529 3	Bassariscus sumichrasti SRX1099089 1	0,19230308
Urocyon cinereoargenteus NC026723 2	Mustela kathiah NC023210 1	0,19230445
Procyon lotor AB462046 3	Mungotictis decemlineata NC027828 1	0,19230501
Ursus arctos AP012576 6	Panthera pardus NC010641 5	0,19230646
Otocyon megalotis SAF1 2	Urva brachyura KY117547 1	0,1923065
Panthera onca NC022842 1	Arctocephalus forsteri NC004023 28	0,19230793
Vulpes lagopus NC026529 3	Hyaena hyaena NC020669 1	0,19230841
Urocyon littoralis catalinae KP129018	Prionodon pardicolor NC024569 2	0,19231325
Speothos venaticus C48 2	Meles anakuma NC009677 1	0,19231388
Ursus spelaeus NC011112 8	Cynogale bennetti KY117544 1	0,19231476
Ursus arctos GU573486 5	Atilax paludinosus T606 1	0,19231626
Proteles cristata T393 6	Neophoca cinerea NC008419 1	0,19231911
Smilodon populator MF871700 1	Odobenus rosmarus NC004029 29	0,19231981
Canis mesomelas KT448280 1	Callorhinus ursinus NC008415 1	0,19232194
Tremarctos ornatus NC009969 2	Proteles cristata T393 6	0,19232553
Ursus arctos isabellinus 18B5 2	Panthera tigris amoyensis NC014770 2	0,19232887
Panthera pardus japonensis KJ866876	Chrysocyon brachyurus NC024172 1	0,19232953
Vulpes ferrilata NC027935 1	Urva javanica/auropunctata NC006835	0,19233602
Prionodon pardicolor NC024569 2	Mephitis mephitis NC020648 1	0,19233929
Vulpes lagopus NC026529 3	Ursus americanus JX196366 3	0,19234067

Martes zibellina NC011579 39	Arctocephalus gazella BK010918 1	0,17916784
Galidia elegans D146 1	Erignathus barbatus NC008426 1	0,17917092
Pusa caspica NC008431 1	Lycalopex sechurae KT448284 1	0,1791731
Otaria byronia OTAB 1	Mustela putorius NC020638 4	0,17917401
Otaria byronia OTAB 1	Mustela nigripes NC024942 1	0,17917432
Panthera tigris NC010642 35	Halichoerus grypus NC001602 2	0,17917432
Potos flavus T414 1	Paguma larvata PDDS11 2	0,17917491
Taxidea taxus NC020646 1	Urva semitorquata MH464789 1	0,17917556
Viverricula indica KX891745 1	Potos flavus T414 1	0,17917593
Suricata suricatta SSM10 1	Phoca groenlandica NC008429 54	0,17917788
Mustela sibirica AP017394 11	Leopardus pardalis NC028315 1	0,17917825
Paguma larvata PDDS11 2	Mustela sibirica NC020637 6	0,17917828
Viverra zibetha T609 1	Mustela sibirica NC020637 6	0,1791786
Monachus schauinslandi NC008421 1	Cynictis penicillata T375 1	0,17917864
Prionailurus planiceps KY682741 4	Mustela nigripes NC024942 1	0,17917924
Martes foina NC020643 1	Crossarchus platycephalus C7R66 1	0,1791793
Prionailurus rubiginosus NC028304 2	Mustela sibirica AP017394 11	0,17917931
Melogale moschata KP726273 1	Leopardus pardalis NC028315 1	0,17918009
Mustela erminea T305 2	Civettictis civetta GLC19 1	0,17918088
Zalophus wollebaeki SRR4431565 1	Mustela putorius NC020638 4	0,17918165
Leopardus guigna NC028321 1	Canis adustus KT448271 1	0,17918251
Ursus thibetanus mupinensis NC008753 2	Mustela sibirica NC020637 6	0,17918369
Urocyon littoralis catalinae KP129018 15	Lobodon carcinophaga NC008423 1	0,17918478
Mustela frenata NC020640 1	Chrysocyon brachyurus NC024172 1	0,17918479
Panthera tigris NC010642 35	Erignathus barbatus NC008426 1	0,17918703
Martes americana NC020642 1	Canis adustus KT448271 1	0,17918782
Smilodon populator MF871700 1	Mustela frenata NC020640 1	0,17918903
Galidia elegans D146 1	Canis adustus KT448271 1	0,17919053
Mirounga angustirostris SRR10331586 1	Cryptoprocta ferox CFC13 1	0,17919064
Zalophus californianus NC008416 1	Viverra tangalunga MH464792 1	0,17919277
Phoca largha NC008430 1	Panthera uncia NC010638 1	0,17919427
Leopardus colocolo NC028314 1	Arctonyx collaris NC020645 1	0,17919435
Zalophus californianus NC008416 1	Mustela sibirica AP017394 11	0,17919502
Ursus thibetanus thibetanus NC011118 4	Meles anakuma NC009677 1	0,17919607
Procyon lotor AB462049 4	Ommatophoca rossii AY377287etc 1	0,17919888
Panthera tigris NC010642 35	Monachus schauinslandi NC008421 1	0,17919905
Pusa hispida NC 008433 1	Crocuta crocuta NC020670 3	0,17919958
Phoca largha NC008430 1	Panthera tigris amoyensis NC014770 2	0,17920241
Ursus americanus JX196366 3	Hemigalus derbyanus MH464791 1	0,17920375
Ommatophoca rossii AY377287etc 1	Mustela altaica NC021751 1	0,17920706
Mustela sibirica NC020637 6	Hemigalus derbyanus MH464791 1	0,17920902
Ursus thibetanus formosanus NC009331 1	Neovison vison NC020641 3	0,17921108
Mustela nigripes NC024942 1	Canis lupus chanco NC010340 4	0,17921178
Eumetopias jubatus NC004030 10	Aonyx cinerea NC035814 2	0,17921346
Zalophus californianus NC008416 1	Ursus thibetanus formosanus NC009331 1	0,17921507
Mungotictis decemlineata NC027828 1	Monachus monachus NC044972 5	0,17921624
Vulpes lagopus NC026529 3	Smilodon populator MF871700 1	0,17921735
Viverricula indica NC025296 2	Lycalopex sechurae KT448284 1	0,17922503
Lutra lutra LC050126 1	Arctocephalus townsendi NC008420 1	0,17922834
Tremarctos ornatus NC009969 2	Martes pennanti NC020664 16	0,17922944
Arctotherium sp NC030174 1	Arctocephalus gazella BK010918 1	0,1792309
Lycalopex sechurae KT448284 1	Cystophora cristata NC008427 1	0,179231
Panthera pardus japonensis KJ866876 8	Halichoerus grypus NC001602 2	0,17923366
Callorhinus ursinus NC008415 1	Aonyx cinerea NC035814 2	0,17923811
Otaria byronia OTAB 1	Arctotherium sp NC030174 1	0,17923926
Meles meles T303 3	Lynx canadensis NC028313 1	0,17924051
Otaria byronia OTAB 1	Mustela sibirica AP017394 11	0,17924091
Meles leucurus NC039173 4	Leopardus pardalis NC028315 1	0,17924097
Lynx pardinus NC028319 161	Lutra lutra NC011358 9	0,17924245
Lutra lutra LC050126 1	Leopardus wiedii NC028318 1	0,17924295
Meles meles T303 3	Civettictis civetta GLC19 1	0,17924431
Civettictis civetta GLC19 1	Arctonyx collaris NC020645 1	0,17924543
Lutra sumatrana NC035810 1	Leopardus pardalis T262 1	0,17924646
Canis adustus KT448271 1	Atilax paludinosus T606 1	0,17924716
Mirounga leonina NC008422 1	Cryptoprocta ferox CFC13 1	0,17924726
Mirounga angustirostris SRR10331586 1	Xenogale naso C07XAR110 1	0,17924748
Melogale moschata KP726273 1	Leopardus pardalis T262 1	0,17924756
Prionailurus rubiginosus NC028304 2	Melogale moschata KP726273 1	0,17924844
Taxidea taxus NC020646 1	Felis catus NC001700 2	0,1792489
Taxidea taxus NC020646 1	Felis nigripes NC028309 1	0,17924896
Phoca vitulina NC001325 1	Helogale parvula SRR7637809 1	0,17924918
Prionailurus bengalensis NC028301 12	Martes zibellina NC011579 39	0,17924966
Ursus thibetanus mupinensis NC008753 2	Mustela putorius NC020638 4	0,17925353
Mustela altaica NC021751 1	Arctodus simus NC011116 1	0,17925437
Panthera tigris amoyensis NC014770 2	Erignathus barbatus NC008426 1	0,17925441
Ursus arctos isabellinus 18B5 2	Mustela erminea T305 2	0,17925455
Catopuma badia NC028300 1	Canis latrans NC008093 7	0,17925483
Mustela nivalis T306 5	Arctodus simus NC011116 1	0,17925623
Martes melampus NC009678 1	Canis adustus KT448271 1	0,17925902
Ursus thibetanus mupinensis NC008753 2	Genetta abyssinica MG489822 1	0,17925923
Pusa sibirica NC008432 2	Panthera leo spelaea KX258452 2	0,17925987
Pusa caspica NC008431 1	Panthera leo spelaea KX258452 2	0,17926043
Prionailurus planiceps NC028312 6	Martes foina NC020643 1	0,17926137
Nyctereutes procyonoides NC013700 3	Homotherium latidens MF871702 3	0,17926298
Lutra sumatrana NC035810 1	Hemigalus derbyanus MH464791 1	0,17926323
Ursus spelaeus NC011112 8	Mustela frenata NC020640 1	0,17926389
Neophoca cinerea NC008419 1	Gulo gulo NC009685 3	0,17926519
Paradoxurus hermaphroditus NC039591 1	Monachus monachus NC044972 5	0,17927355
Monachus monachus NC044972 5	Lutrogale perspicillata NC035811 1	0,17927973
Ursus thibetanus thibetanus NC011118 4	Neovison vison NC020641 3	0,1792811
Mustela nivalis T306 5	Eumetopias jubatus NC004030 10	0,17928365
Panthera leo NERO 19	Monachus schauinslandi NC008421 1	0,17928986
Spilogale putorius NC010497 1	Paradoxurus jerdoni MH464793 1	0,17928986
Meles meles T303 3	Arctocephalus forsteri KT693377 17	0,17929617
Viverricula indica NC025296 2	Canis mesomelas KT448280 1	0,17929773
Neovison vison NC020641 3	Civettictis civetta NC033378 1	0,1792984
Lutrogale perspicillata NC035811 1	Bassaricyon neblina SRX1097850 1	0,17929964
Mustela kathiah NC023210 1	Fossa fossana D350 1	0,17930007
Vulpes vulpes NC008434 5	Pusa sibirica NC008432 2	0,17930183
Neophoca cinerea NC008419 1	Nandinia binotata NC024567 1	0,17930232
Nandinia binotata NC024567 1	Ailurus fulgens NC011124 1	0,17930315
Hemigalus derbyanus MH464791 1	Canis latrans NC008093 7	0,17930511
Prionailurus rubiginosus NC028304 2	Lutra lutra LC050126 1	0,17930844

Vulpes ferrilata NC027935 1	Neofelis nebulosa NC008450 3	0,1923449
Panthera leo NERO 19	Arctocephalus gazella BK010918 1	0,19234863
Urocyon cinereoargenteus NC026723 2	Eumetopias jubatus NC004030 10	0,19235261
Panthera leo NERO 19	Otaria byronia OTAB 1	0,19235575
Cuon alpinus NC013445 3	Arctocephalus pusillus NC008417 1	0,19235703
Vulpes vulpes NC008434 5	Conepatus chinga NC042596 1	0,19235995
Civettictis civetta NC033378 1	Bassaricyon neblina SRX1097850 1	0,19236004
Urocyon cinereoargenteus NC026723 2	Lutra lutra NC011358 9	0,19236316
Urocyon cinereoargenteus NC026723 2	Lutra lutra LC050126 1	0,19236405
Vulpes ferrilata NC027935 1	Arctocephalus gazella BK010918 1	0,1923673
Vulpes zerda KJ603240 1	Ichneumia albicauda T603 1	0,19236885
Neophoca cinerea NC008419 1	Canis latrans NC008093 7	0,19237073
Panthera pardus NC010641 5	Chrysocyon brachyurus NC024172 1	0,19237146
Lycaon pictus NC028427 2	Lontra canadensis SRR10409165 1	0,19237563
Nyctereutes procyonoides NC013700 3	Cryptoprocta ferox CFC13 1	0,19237602
Hyaena hyaena NC020669 1	Ailurus fulgens NC011124 1	0,19237661
Procyon lotor AB462046 3	Hyaena hyaena NC020669 1	0,19237767
Ursus arctos GU573491 207	Paradoxurus hermaphroditus NC03959	0,19238628
Panthera pardus japonensis KJ866876	Martes pennanti NC020664 16	0,19238679
Prionailurus viverrinus NC028305 1	Bassaricyon neblina SRX1097850 1	0,19238858
Panthera tigris NC010642 35	Arctocephalus gazella BK010918 1	0,192389
Speothos venaticus C48 2	Martes foina NC020643 1	0,19239005
Nyctereutes procyonoides NC013700 3	Ailuropoda melanoleuca NC009492 5	0,19239157
Canis lupus chanco NC010340 4	Arctocephalus australis MG023139 1	0,1923922
Lynx canadensis NC028313 1	Ailuropoda melanoleuca NC009492 5	0,1923954
Panthera leo spelaea KX258452 2	Arctocephalus australis MG023139 1	0,19239557
Mephitis mephitis NC020648 1	Lutrogale perspicillata NC035811 1	0,19239716
Proteles cristata T393 6	Mustela altaica NC021751 1	0,19239728
Poecilogale albinucha T602 1	Parahyaena brunnea NC038159 15	0,19239768
Lutrogale perspicillata NC035811 1	Urva javanica/auropunctata NC006835	0,19240275
Ursus arctos GU573486 5	Panthera tigris amoyensis NC014770 2	0,19240364
Ursus arctos GU573486 5	Chrysocyon brachyurus NC024172 1	0,19240367
Zalophus wollebaeki SRR4431565 1	Proteles cristata T393 6	0,19240528
Ursus arctos AP012576 6	Lycaon pictus NC028427 2	0,19240798
Ursus americanus JX196366 3	Urva brachyura KY117547 1	0,19240846
Ursus spelaeus EU327344 13	Proteles cristata T393 6	0,19240977
Melursus ursinus NC009970 2	Diplogale hosei MH464790 1	0,19241345
Otaria byronia OTAB 1	Canis mesomelas KT448280 1	0,19241668
Fossa fossana D350 1	Cuon alpinus NC013445 3	0,19241776
Odobenus rosmarus NC004029 29	Leopardus colocolo NC028314 1	0,1924179
Ursus arctos isabellinus 18B5 2	Cuon alpinus NC013445 3	0,19242138
Ursus spelaeus EU327344 13	Urva javanica/auropunctata NC006835	0,19243057
Canis latrans NC008093 7	Arctocephalus townsendi NC008420 1	0,19243135
Vulpes zerda KJ603240 1	Potos flavus T414 1	0,19243379
Vulpes zerda KJ603240 1	Suricata suricatta SSM10 1	0,19243422
Lutrogale perspicillata NC035811 1	Cynictis penicillata T375 1	0,19243462
Nyctereutes procyonoides NC013700 3	Galidia elegans D146 1	0,19243559
Mustela eversmannii NC028013 1	Galidia elegans D146 1	0,19243562
Neophoca cinerea NC008419 1	Canis anthus NC027956 2	0,19243801
Urocyon littoralis catalinae KP129018	Meles anakuma NC009677 1	0,19243804
Panthera uncia NC010638 1	Tapirus terrestris T358	0,19244005
Vulpes corsac NC023958 1	Ursus arctos pruinosus MG066703 3	0,19244242
Ursus arctos GU573491 207	Cynictis penicillata T375 1	0,19244413
Panthera pardus japonensis KJ866876	Arctocephalus townsendi NC008420 1	0,19244522
Otocyon megalotis SAF1 2	Neophoca cinerea NC008419 1	0,19244634
Chrysocyon brachyurus NC024172 1	Arctocephalus forsteri KT693377 17	0,19244756
Suricata suricatta SSM10 1	Chrysocyon brachyurus NC024172 1	0,1924516
Felis catus NC001700 2	Bassaricyon neblina SRX1097850 1	0,19245206
Odobenus rosmarus NC004029 29	Mustela kathiah NC023210 1	0,19245288
Chrysocyon brachyurus NC024172 1	Aonyx cinerea NC035814 2	0,19245324
Lycalopex sechurae KT448284 1	Bdeogale nigripes GLC15 1	0,19245516
Vulpes zerda KJ603240 1	Melursus ursinus NC009970 2	0,19245546
Mephitis mephitis NC020648 1	Urva brachyura KY117547 1	0,19245583
Panthera tigris amoyensis NC014770 2	Lontra canadensis SRR10409165 1	0,1924559
Mungotictis decemlineata NC027828 1	Canis mesomelas KT448280 1	0,19245602
Vulpes ferrilata NC027935 1	Melursus ursinus NC009970 2	0,19245644
Nyctereutes procyonoides NC013700 3	Galictis vittata T412 1	0,19245896
Nasua nasua NC020647 1	Helarctos malayanus NC009968 2	0,19246012
Mephitis mephitis NC020648 1	Felis nigripes NC028309 1	0,19246416
Ursus maritimus NC003428 31	Panthera tigris NC010642 35	0,1924646
Nyctereutes procyonoides NC013700 3	Mephitis mephitis NC020648 1	0,19246855
Galictis vittata T412 1	Chrysocyon brachyurus NC024172 1	0,1924738
Arctocephalus australis MG023139 1	Tapirus terrestris T358	0,19248904
Galictis vittata T412 1	Canis aureus KT448274 1	0,19249125
Vulpes zerda KJ603240 1	Callorhinus ursinus NC008415 1	0,19250022
Urocyon littoralis catalinae KP129018	Mungos mungo/gambianus SRR77048	0,19250126
Vulpes ferrilata NC027935 1	Tremarctos ornatus NC009969 2	0,19250199
Ursus spelaeus NC011112 8	Cuon alpinus NC013445 3	0,19250263
Procyon lotor AB462049 4	Prionodon linsang ERR2391707 1	0,19250739
Vulpes corsac NC023958 1	Otaria byronia OTAB 1	0,19250935
Otocyon megalotis SAF1 2	Arctocephalus australis MG023139 1	0,19251117
Ursus maritimus NC003428 31	Atilax paludinosus T606 1	0,1925118
Tremarctos ornatus NC009969 2	Panthera pardus japonensis KJ866876	0,1925142
Paguma larvata PDDS11 2	Otocyon megalotis SAF1 2	0,19251485
Neofelis nebulosa NC008450 3	Lycalopex sechurae KT448284 1	0,19251519
Panthera tigris NC010642 35	Lontra canadensis SRR10409165 1	0,19252349
Nasua nasua NC020647 1	Melursus ursinus NC009970 2	0,19252601
Ursus spelaeus NC011112 8	Suricata suricatta SSM10 1	0,19252612
Otocyon megalotis SAF1 2	Ailuropoda melanoleuca NC009492 5	0,19252825
Ursus arctos pruinosus MG066703 3	Lycaon pictus NC028427 2	0,19252867
Poecilogale albinucha T602 1	Odobenus rosmarus NC004029 29	0,19252992
Ursus maritimus NC003428 31	Panthera leo spelaea KX258452 2	0,19253305
Panthera uncia KP202269 1	Melursus ursinus NC009970 2	0,19253835
Parahyaena brunnea NC038159 15	Mephitis mephitis NC020648 1	0,19254017
Zalophus californianus NC008416 1	Speothos venaticus C48 2	0,19254334
Otaria byronia OTAB 1	Canis lupus familiaris NC002008 1231	0,19254707
Conepatus chinga NC042596 1	Bdeogale nigripes GLC15 1	0,19255726
Canis latrans NC008093 7	Arctocephalus australis MG023139 1	0,19256681
Vulpes ferrilata NC027935 1	Bdeogale nigripes GLC15 1	0,1925673
Xenogale naso C07XAR110 1	Ailurus fulgens NC011124 1	0,1925673
Vulpes zerda KJ603240 1	Prionodon linsang ERR2391707 1	0,19256802
Vulpes zerda KJ603240 1	Aonyx cinerea NC035814 2	0,19256921
Vulpes zerda KJ603240 1	Arctocephalus gazella BK010918 1	0,19257191

Ommatophoca rossii AY377287etc 1	Civettictis civetta GLC19 1	0,17930934
Lutra lutra NC011358 9	Homotherium latidens MF871702 3	0,17931059
Vulpes vulpes NC008434 5	Phoca fasciata NC008428 1	0,17931116
Paradoxurus hermaphroditus NC039591 1	Erignathus barbatus NC008426 1	0,17931174
Mustela frenata NC020640 1	Leopardus tigrinus NC028317 1	0,17931238
Tremarctos ornatus NC009969 2	Martes foina NC020643 1	0,17931242
Lynx lynx NC027083 4	Lutra sumatrana NC035810 1	0,17931256
Zalophus wollebaeki SRR4431565 1	Meles leucurus NC039173 4	0,17931271
Viverra tangalunga MH464792 1	Leopardus wiedii NC028318 1	0,1793129
Paguma larvata PDDS11 2	Mustela itatsi NC034330 19	0,17931375
Mustela frenata NC020640 1	Catopuma temminckii NC027115 41	0,17931402
Viverricula indica NC025296 2	Potos flavus T414 1	0,17931445
Ommatophoca rossii AY377287etc 1	Felis margarita NC028308 1	0,17931591
Ursus arctos pruinosus MG066703 3	Potos flavus T414 1	0,17931929
Mustela nivalis T306 5	Leopardus pardalis NC028315 1	0,17932293
Neovison vison NC020641 3	Lynx pardinus NC028319 161	0,17932346
Leopardus colocolo NC028314 1	Enhydra lutris NC009692 1	0,17932427
Arctonyx collaris NC020645 1	Acinonyx jubatus NC005212 3	0,17932576
Ursus thibetanus thibetanus NC011118 4	Melogale moschata KP726273 1	0,17932633
Helarctos malayanus NC009968 2	Arctocephalus gazella BK010918 1	0,17932709
Ursus thibetanus formosanus NC009331 1	Mustela sibirica AP017394 11	0,17932783
Leopardus colocolo NC028314 1	Bassariscus sumichrasti SRX1099089 1	0,17932849
Meles anakuma NC009677 1	Genetta abyssinica MG489822 1	0,17932911
Paradoxurus hermaphroditus NC039591 1	Neovison vison NC020641 3	0,17933141
Lutra sumatrana NC035810 1	Leopardus colocolo NC028314 1	0,17933145
Ursus thibetanus thibetanus NC011118 4	Mustela kathiah NC023210 1	0,1793323
Canis adustus KT448271 1	Ailurus fulgens styani NC009691 1	0,1793351
Phoca vitulina NC001325 1	Galictis vittata T412 1	0,17933802
Vulpes corsac NC023958 1	Monachus monachus NC044972 5	0,17933969
Zalophus wollebaeki SRR4431565 1	Gulo gulo NC009685 3	0,17934234
Martes flavigula NC012141 3	Arctocephalus australis MG023139 1	0,17934787
Urva brachyura KY117547 1	Halichoerus grypus NC001602 2	0,17934917
Ommatophoca rossii AY377287etc 1	Melursus ursinus NC009970 2	0,17935303
Tremarctos ornatus NC009969 2	Eumetopias jubatus NC004030 10	0,17935511
Martes flavigula NC012141 3	Lynx canadensis NC028313 1	0,17935945
Tremarctos ornatus NC009969 2	Martes melampus NC009678 1	0,17936295
Arctocephalus gazella BK010918 1	Ailurus fulgens styani NC009691 1	0,17936721
Procyon lotor AB462046 3	Callorhinus ursinus NC008415 1	0,17936787
Ursus thibetanus mupinensis NC008753 2	Martes flavigula NC012141 3	0,17936796
Otaria byronia OTAB 1	Meles meles T303 3	0,17937414
Mustela altaica NC021751 1	Callorhinus ursinus NC008415 1	0,17937462
Viverricula indica KX891751 1	Mustela frenata NC020640 1	0,17937534
Phoca fasciata NC008428 1	Lycalopex sechurae KT448284 1	0,17937544
Spilogale putorius NC010497 1	Hemigalus derbyanus MH464791 1	0,17937773
Leopardus geoffroyi NC028320 1	Enhydra lutris NC009692 1	0,17937881
Puma yagouaroundi NC028311 1	Ailurus fulgens styani NC009691 1	0,17937958
Procyon lotor AB462046 3	Ailurus fulgens styani NC009691 1	0,17937973
Zalophus wollebaeki SRR4431565 1	Ailurus fulgens styani NC009691 1	0,17937979
Mustela frenata NC020640 1	Genetta genetta T297 1	0,17937994
Arctotherium sp NC030174 1	Ailurus fulgens NC011124 1	0,17938073
Viverra tangalunga MH464792 1	Prionailurus rubiginosus NC028304 2	0,17938097
Ursus thibetanus thibetanus NC011118 4	Martes flavigula NC012141 3	0,17938131
Genetta genetta T297 1	Arctonyx collaris NC020645 1	0,17938153
Viverricula indica KX891751 1	Canis anthus NC027956 2	0,17938226
Ursus arctos GU573491 207	Meles meles T303 3	0,1793834
Ursus thibetanus mupinensis NC008753 2	Martes americana NC020642 1	0,17938505
Martes martes T302 3	Leopardus pardalis T262 1	0,17938928
Felis silvestris lybica KP202275 4	Arctodus simus NC011116 1	0,17939201
Mustela nigripes NC024942 1	Mephitis mephitis NC020648 1	0,17939349
Puma yagouaroundi NC028311 1	Neovison vison NC020641 3	0,17939354
Ursus thibetanus mupinensis NC008753 2	Mustela nivalis T306 5	0,17939372
Catopuma temminckii NC027115 41	Arctodus simus NC011116 1	0,17939484
Martes foina NC020643 1	Lynx pardinus NC028319 161	0,17939505
Leopardus pardalis NC028315 1	Ictonyx striatus T299 1	0,17939548
Ursus spelaeus EU327344 13	Meles leucurus NC039173 4	0,17939721
Zalophus californianus NC008416 1	Mustela itatsi NC034330 19	0,17939846
Ursus spelaeus NC011112 8	Mustela nigripes NC024942 1	0,17940061
Pusa hispida NC 008433 1	Arctictis binturong T605 2	0,17940086
Mustela eversmannii NC028013 1	Hemigalus derbyanus MH464791 1	0,17940196
Monachus monachus NC044972 5	Bassaricyon neblina SRX1097850 1	0,1794063
Monachus schauinslandi NC008421 1	Lycalopex sechurae KT448284 1	0,17941919
Spilogale putorius NC010497 1	Mustela nivalis T306 5	0,17942318
Nasua nasua NC020647 1	Enhydra lutris NC009692 1	0,17942946
Callorhinus ursinus NC008415 1	Ailurus fulgens styani NC009691 1	0,17943107
Urva brachyura KY117547 1	Canis adustus KT448271 1	0,17943408
Arctotherium sp NC030174 1	Arctocephalus pusillus NC008417 1	0,17943734
Lutra lutra LC050126 1	Genetta servalina NC024568 2	0,17943753
Mustela erminea T305 2	Xenogale naso C07XAR110 1	0,17943855
Taxidea taxus NC020646 1	Fossa fossana D350 1	0,1794436
Prionailurus rubiginosus NC028304 2	Meles leucurus NC039173 4	0,1794447
Viverricula indica KX891751 1	Potos flavus T414 1	0,17944489
Viverricula indica KX891745 1	Meles leucurus NC039173 4	0,17944495
Meles leucurus NC039173 4	Arctotherium sp NC030174 1	0,179446
Lynx pardinus NC028319 161	Callorhinus ursinus NC008415 1	0,17944641
Prionailurus bengalensis NC028301 12	Callorhinus ursinus NC008415 1	0,17944668
Ursus arctos GU573491 207	Potos flavus T414 1	0,1794471
Meles leucurus NC039173 4	Leopardus guigna NC028321 1	0,17944799
Prionailurus planiceps KY682741 4	Mustela putorius NC020638 4	0,17944837
Catopuma badia NC028300 1	Arctonyx collaris NC020645 1	0,17944866
Martes foina NC020643 1	Arctocephalus gazella BK010918 1	0,17944992
Phoca largha NC008430 1	Canis latrans NC008093 7	0,17944993
Zalophus wollebaeki SRR4431565 1	Mustela sibirica AP017394 11	0,17945011
Taxidea taxus NC020646 1	Prionailurus planiceps KY682741 4	0,17945066
Phoca largha NC008430 1	Helogale parvula SRR7637809 1	0,17945103
Mustela kathiah NC023210 1	Leopardus guigna NC028321 1	0,17945126
Nasua nasua NC020647 1	Hydrurga leptonyx NC008425 1	0,17945245
Pusa hispida NC 008433 1	Galidia elegans D146 1	0,17945288
Ursus thibetanus mupinensis NC008753 2	Mustela sibirica AP017394 11	0,17945305
Viverricula indica NC025296 2	Nyctereutes procyonoides NC013700 3	0,17945315
Pusa hispida NC 008433 1	Canis mesomelas KT448280 1	0,17945488
Vulpes zerda KJ603240 1	Hydrurga leptonyx NC008425 1	0,17945498
Cystophora cristata NC008427 1	Crocuta crocuta NC020670 3	0,1794562
Prionailurus rubiginosus NC028304 2	Mustela altaica NC021751 1	0,17945623

Vulpes corsac NC023958 1	Procyon lotor AB462049 4	0,19257236
Panthera leo NERO 19	Melursus ursinus NC009970 2	0,19257346
Paguma larvata PDDS11 2	Lontra canadensis SRR10409165 1	0,19257441
Vulpes zerda KJ603240 1	Cryptoprocta ferox CFC13 1	0,19257805
Parahyaena brunnea NC038159 15	Tapirus terrestris T358	0,1925811
Ursus thibetanus mupinensis NC00875	Urva brachyura KY117547 1	0,19258125
Panthera onca NC022842 1	Conepatus chinga NC042596 1	0,19258265
Nasua nasua NC020647 1	Leopardus pardalis NC028315 1	0,19258505
Salanoia concolor D378 1	Canis mesomelas KT448280 1	0,19258718
Otocyon megalotis SAF1 2	Cryptoprocta ferox CFC13 1	0,19258861
Suricata suricatta SSM10 1	Ailuropoda melanoleuca NC009492 5	0,19258897
Tremarctos ornatus NC009969 2	Panthera tigris NC010642 35	0,1925915
Panthera tigris NC010642 35	Nyctereutes procyonoides NC013700 3	0,19259179
Galidia elegans D146 1	Galictis vittata T412 1	0,19259524
Galidia elegans D146 1	Chrysocyon brachyurus NC024172 1	0,19259711
Ursus arctos EU497665 29	Chrysocyon brachyurus NC024172 1	0,19259919
Ursus arctos EU497665 29	Panthera tigris amoyensis NC014770 2	0,19259922
Ursus maritimus GU573488 Svalbard	Panthera leo spelaea KX258452 2	0,19260037
Panthera pardus japonensis KJ866876	Melursus ursinus NC009970 2	0,19260588
Neophoca cinerea NC008419 1	Canis lupus familiaris NC002008 1231	0,19260749
Canis mesomelas KT448280 1	Arctocephalus forsteri KT693377 17	0,19260752
Ursus arctos AP012576 6	Felis nigripes NC028309 1	0,19260874
Vulpes ferrilata NC027935 1	Ursus americanus JX196366 3	0,19261102
Ursus arctos EU497665 29	Cuon alpinus NC013445 3	0,19262309
Urva javanica T413 1	Conepatus chinga NC042596 1	0,19263209
Vulpes vulpes NC008434 5	Arctocephalus townsendi NC008420 1	0,19263317
Lontra canadensis SRR10409165 1	Canis mesomelas KT448280 1	0,19263507
Lycalopex sechurae KT448284 1	Galidictis fasciata DM333 1	0,19263723
Salanoia concolor D378 1	Tapirus terrestris T358	0,19264132
Vulpes lagopus NC026529 3	Otaria byronia OTAB 1	0,19264245
Phoca vitulina NC001325 1	Mellivora capensis T370 1	0,19264297
Panthera uncia KP202269 1	Meles anakuma NC009677 1	0,19264321
Vulpes lagopus NC026529 3	Ursus maritimus NC003428 31	0,19264388
Otocyon megalotis SAF1 2	Arctocephalus townsendi NC008420 1	0,19264527
Lontra canadensis SRR10409165 1	Genetta genetta T297 1	0,1926457
Ursus arctos isabellinus 18B5 2	Xenogale naso C07XAR110 1	0,19264607
Speothos venaticus C48 2	Melogale moschata V0735A 1	0,19264622
Ursus arctos EU497665 29	Atilax paludinosus T606 1	0,1926465
Neofelis nebulosa NC008450 3	Canis lupus familiaris NC002008 1231	0,19265352
Prionodon pardicolor NC024569 2	Arctotherium sp NC030174 1	0,19265409
Ursus maritimus GU573488 Svalbard	Paradoxurus hermaphroditus NC03959	0,19265573
Panthera tigris NC010642 35	Mustela itatsi NC034330 19	0,19265583
Ursus arctos isabellinus 18B5 2	Parahyaena brunnea NC038159 15	0,19265814
Ursus thibetanus laniger MH281753 2	Urva brachyura KY117547 1	0,19265904
Poecilogale albinucha T602 1	Hyaena hyaena NC020669 1	0,19266042
Ursus arctos AP012576 6	Bdeogale nigripes GLC15 1	0,19266251
Ursus arctos isabellinus 18B5 2	Panthera tigris NC010642 35	0,1926658
Ursus arctos pruinosus MG066703 3	Panthera onca NC022842 1	0,19266923
Cryptoprocta ferox CFC13 1	Canis lupus familiaris NC002008 1231	0,19267949
Vulpes zerda KJ603240 1	Ursus americanus JX196366 3	0,1926813
Neofelis nebulosa NC008450 3	Arctocephalus pusillus NC008417 1	0,19268847
Mellivora capensis T370 1	Bassaricyon neblina SRX1097850 1	0,1926941
Urocyon cinereoargenteus NC026723 2	Eupleres goudotii D128 1	0,19269949
Vulpes lagopus NC026529 3	Lontra canadensis SRR10409165 1	0,19270031
Nasua nasua NC020647 1	Mungos mungo/gambianus SRR77048	0,19270319
Vulpes lagopus NC026529 3	Aonyx cinerea NC035814 2	0,19270332
Mustela sibirica AP017394 11	Cynogale bennetti KY117544 1	0,19270431
Neofelis nebulosa NC008450 3	Helarctos malayanus NC009968 2	0,19270577
Vulpes zerda KJ603240 1	Neophoca cinerea NC008419 1	0,19270654
Vulpes zerda KJ603240 1	Procyon lotor AB462046 3	0,19270697
Vulpes zerda KJ603240 1	Arctocephalus pusillus NC008417 1	0,19270721
Arctocephalus pusillus NC008417 1	Tapirus terrestris T358	0,19270907
Vulpes lagopus NC026529 3	Ursus maritimus GU573488 Svalbard	0,19271122
Panthera pardus NC010641 5	Melursus ursinus NC009970 2	0,19271395
Lutrogale perspicillata NC035811 1	Genetta genetta T297 1	0,19271599
Viverricula indica KX891745 1	Bassaricyon neblina SRX1097850 1	0,19271645
Ursus spelaeus EU327344 13	Cynogale bennetti KY117544 1	0,19271882
Procyon lotor AB462049 4	Panthera uncia NC010638 1	0,19272064
Puma concolor NC016470 22	Nasua nasua NC020647 1	0,1927207
Prionailurus bengalensis NC028301 12	Nasua nasua NC020647 1	0,19272131
Prionailurus planiceps NC028312 6	Bassaricyon neblina SRX1097850 1	0,19272175
Prionailurus viverrinus NC028305 1	Nasua nasua NC020647 1	0,19272207
Phocarctos hookeri NC008418 1	Mephitis mephitis NC020648 1	0,19272584
Galictis vittata T412 1	Canis latrans NC008093 7	0,19272797
Martes flavigula NC012141 3	Cynogale bennetti KY117544 1	0,1927329
Canis lupus familiaris NC002008 1231	Arctocephalus australis MG023139 1	0,19273669
Poecilogale albinucha T602 1	Crocuta crocuta NC020670 3	0,19274054
Urocyon cinereoargenteus NC026723 2	Gulo gulo NC009685 3	0,19275172
Panthera pardus NC010641 5	Arctocephalus townsendi NC008420 1	0,19275388
Spilogale putorius NC010497 1	Puma concolor NC016470 22	0,192756
Ursus maritimus NC003428 31	Cuon alpinus NC013445 3	0,19275779
Spilogale putorius NC010497 1	Fossa fossana D350 1	0,1927581
Ursus arctos GU573486 5	Cuon alpinus NC013445 3	0,19276419
Tremarctos ornatus NC009969 2	Canis mesomelas KT448280 1	0,19276856
Vulpes ferrilata NC027935 1	Mungos mungo MMC7 1	0,19276978
Vulpes zerda KJ603240 1	Cynictis penicillata T375 1	0,19277035
Urocyon littoralis catalinae KP129018	Melogale moschata V0735A 1	0,1927706
Urocyon cinereoargenteus NC026723 2	Lutra sumatrana NC035810 1	0,19277212
Panthera pardus japonensis KJ866876	Martes zibellina NC011579 39	0,19278319
Civettictis civetta GLC19 1	Bassaricyon neblina SRX1097850 1	0,19278343
Urocyon littoralis catalinae KP129018	Leopardus tigrinus NC028317 1	0,19278432
Viverra zibetha T609 1	Bassaricyon neblina SRX1097850 1	0,19278443
Nasua nasua NC020647 1	Lynx canadensis NC028313 1	0,19278477
Zalophus wollebaeki SRR4431565 1	Prionodon linsang ERR2391707 1	0,19278634
Panthera tigris amoyensis NC014770 2	Arctocephalus gazella BK010918 1	0,19279312
Speothos venaticus C48 2	Panthera onca KP202264 2	0,1927947
Ursus thibetanus laniger MH281753 2	Nasua nasua NC020647 1	0,19279513
Ursus spelaeus NC011112 8	Nasua nasua NC020647 1	0,19279841
Ursus spelaeus EU327344 13	Nasua nasua NC020647 1	0,19279847
Ursus arctos pruinosus MG066703 3	Chrysocyon brachyurus NC024172 1	0,1928011
Ursus maritimus GU573488 Svalbard	Chrysocyon brachyurus NC024172 1	0,19280121
Ursus spelaeus EU327344 13	Panthera uncia NC010638 1	0,19280286
Cryptoprocta ferox CFC13 1	Canis lupus chanco NC010340 4	0,19280736

Melogale moschata NC020644 1	Leopardus wiedii NC028318 1	0,17945683
Ursus spelaeus NC011112 8	Taxidea taxus NC020646 1	0,17945737
Panthera uncia KP202269 1	Monachus schauinslandi NC008421 1	0,17945911
Leopardus wiedii NC028318 1	Bassariscus sumichrasti SRX1099089 1	0,17945923
Procyon lotor AB462046 3	Hemigalus derbyanus MH464791 1	0,17946179
Monachus schauinslandi NC008421 1	Ichneumia albicauda T603 1	0,17946211
Prionailurus bengalensis CKM45 20	Mustela nivalis T306 5	0,1794626
Martes melampus NC009678 1	Acinonyx jubatus NC005212 3	0,17946339
Mirounga angustirostris SRR10331586 1	Ichneumia albicauda T603 1	0,17946383
Ursus thibetanus formosanus NC009331 1	Mustela eversmannii NC028013 1	0,17946462
Prionailurus bengalensis CKM45 20	Neovison vison NC020641 3	0,17946482
Ursus thibetanus laniger MH281753 2	Mustela eversmannii NC028013 1	0,1794657
Pusa sibirica NC008432 2	Canis lupus familiaris NC002008 1231	0,17946999
Mustela itatsi NC034330 19	Homotherium latidens MF871702 3	0,17947014
Monachus monachus NC044972 5	Bdeogale nigripes GLC15 1	0,17947319
Ommatophoca rossii AY377287etc 1	Canis latrans NC008093 7	0,17947757
Panthera onca NC022842 1	Ommatophoca rossii AY377287etc 1	0,17947862
Monachus monachus NC044972 5	Ichneumia albicauda T603 1	0,179487
Pusa hispida NC 008433 1	Canis lupus chanco NC010340 4	0,1794897
Ursus thibetanus laniger MH281753 2	Hemigalus derbyanus MH464791 1	0,17949122
Puma yagouaroundi NC028311 1	Canis lupus chanco NC010340 4	0,17949401
Martes melampus NC009678 1	Arctocephalus forsteri KT693377 17	0,1794991
Arctocephalus forsteri KT693377 17	Ailurus fulgens styani NC009691 1	0,17950052
Mustela sibirica NC020637 6	Crossarchus platycephalus C7R66 1	0,17950124
Ommatophoca rossii AY377287etc 1	Galerella sanguinea T378 1	0,17950207
Mustela nigripes NC024942 1	Canis aureus KT448274 1	0,17950709
Nyctereutes procyonoides NC013700 3	Mirounga leonina NC008422 1	0,17950791
Neophoca cinerea NC008419 1	Martes martes T302 3	0,17950901
Viverra zibetha T609 1	Meles leucurus NC039173 4	0,1795122
Mirounga angustirostris SRR10331586 1	Cynogale bennetti KY117544 1	0,17951372
Viverra tangalunga MH464792 1	Lynx canadensis NC028313 1	0,17951377
Mustela sibirica AP017394 11	Lynx canadensis NC028313 1	0,17951415
Martes melampus NC009678 1	Leopardus wiedii NC028318 1	0,17951526
Viverra zibetha T609 1	Viverra tangalunga MH464792 1	0,17951596
Prionailurus planiceps NC028312 6	Canis adustus KT448271 1	0,17951679
Arctodus simus NC011116 1	Ailurus fulgens NC011124 1	0,17952055
Mirounga angustirostris SRR10331586 1	Canis anthus NC027956 2	0,17952195
Taxidea taxus NC020646 1	Helarctos malayanus NC009968 2	0,17952502
Prionailurus planiceps NC028312 6	Neovison vison NC020641 3	0,17952716
Ursus spelaeus NC011112 8	Nandinia binotata NC024567 1	0,17952971
Ursus thibetanus formosanus NC009331 1	Martes americana NC020642 1	0,1795298
Ursus thibetanus laniger MH281753 2	Mustela sibirica AP017394 11	0,17953096
Ursus thibetanus thibetanus NC011118 4	Martes americana NC020642 1	0,17953295
Phoca fasciata NC008428 1	Crocuta crocuta NC020670 3	0,17953318
Mustela frenata NC020640 1	Helarctos malayanus NC009968 2	0,1795335
Panthera leo spelaea KX258452 2	Monachus schauinslandi NC008421 1	0,1795354
Ursus thibetanus formosanus NC009331 1	Mustela nivalis T306 5	0,17953758
Phoca largha NC008430 1	Panthera leo spelaea KX258452 2	0,17954758
Melogale moschata NC020644 1	Hemigalus derbyanus MH464791 1	0,17955203
Otaria byronia OTAB 1	Martes flavigula NC012141 3	0,17955254
Monachus monachus NC044972 5	Hyaena hyaena NC020669 1	0,17955445
Panthera onca KP202264 2	Ommatophoca rossii AY377287etc 1	0,17955483
Nasua nasua NC020647 1	Meles leucurus NC039173 4	0,17956328
Lutra sumatrana NC035810 1	Arctocephalus forsteri KT693377 17	0,1795674
Mustela nigripes NC024942 1	Fossa fossana D350 1	0,17956959
Lutra sumatrana NC035810 1	Homotherium latidens MF871702 3	0,17956972
Martes zibellina NC011579 39	Arctocephalus forsteri KT693377 17	0,17957054
Erignathus barbatus NC008426 1	Canis mesomelas KT448280 1	0,17957136
Mustela frenata NC020640 1	Fossa fossana D350 1	0,17957166
Nasua nasua NC020647 1	Leptonychotes weddellii NC008424 1	0,17957219
Neovison vison NC020641 3	Nasua nasua NC020647 1	0,17957452
Meles leucurus NC039173 4	Lynx canadensis NC028313 1	0,17957695
Puma concolor NC016470 22	Lutra lutra LC050126 1	0,17957772
Lynx canadensis NC028313 1	Arctonyx collaris NC020645 1	0,17957859
Lutra lutra NC011358 9	Leopardus wiedii NC028318 1	0,1795797
Halichoerus grypus NC001602 2	Galictis vittata T412 1	0,17958006
Meles meles T303 3	Genetta servalina NC024568 2	0,17958029
Mustela kathiah NC023210 1	Felis silvestris lybica KP202275 4	0,17958219
Mustela putorius NC020638 4	Leopardus pardalis T262 1	0,17958248
Ursus arctos isabellinus 18B5 2	Nandinia binotata NC024567 1	0,17958312
Mustela itatsi NC034330 19	Genetta servalina NC024568 2	0,1795835
Lutra sumatrana NC035810 1	Leopardus wiedii NC028318 1	0,17958352
Viverricula indica KX891745 1	Ailurus fulgens styani NC009691 1	0,17958425
Melogale moschata KP726273 1	Leopardus jacobita NC028322 1	0,17958482
Suricata suricatta SSM10 1	Phoca largha NC008430 1	0,1795858
Paradoxurus hermaphroditus NLNC 1	Canis latrans NC008093 7	0,17958611
Paradoxurus jerdoni MH464793 1	Canis latrans NC008093 7	0,17958623
Martes foina NC020643 1	Conepatus chinga NC042596 1	0,17958672
Martes foina NC020643 1	Galidictis fasciata DM333 1	0,17958787
Halichoerus grypus NC001602 2	Canis lupus familiaris NC002008 1231	0,17958869
Melogale moschata NC020644 1	Leopardus pardalis T262 1	0,17959076
Ommatophoca rossii AY377287etc 1	Atilax paludinosus T606 1	0,1795909
Bassariscus sumichrasti SRX1099089 1	Arctodus simus NC011116 1	0,17959093
Prionailurus bengalensis NC028301 12	Canis aureus KT448274 1	0,17959218
Cystophora cristata NC008427 1	Arctictis binturong T605 2	0,17959632
Speothos venaticus C48 2	Lobodon carcinophaga NC008423 1	0,17959663
Urva semitorquata MH464789 1	Canis adustus KT448271 1	0,17959753
Pusa hispida NC 008433 1	Chrysocyon brachyurus NC024172 1	0,1795976
Prionailurus viverrinus NC028305 1	Martes foina NC020643 1	0,17959991
Tremarctos ornatus NC009969 2	Homotherium latidens MF871702 3	0,17960027
Paradoxurus hermaphroditus NLNC 1	Bassariscus sumichrasti SRX1099089 1	0,17960101
Phoca largha NC008430 1	Crocuta crocuta NC020670 3	0,17960559
Monachus monachus NC044972 5	Canis anthus NC027956 2	0,17960954
Nyctereutes procyonoides NC013700 3	Monachus monachus NC044972 5	0,17961213
Canis adustus KT448271 1	Ailuropoda melanoleuca NC009492 5	0,17961374
Spilogale putorius NC010497 1	Phoca largha NC008430 1	0,17961562
Neofelis nebulosa NC008450 3	Erignathus barbatus NC008426 1	0,17961721
Viverra zibetha T609 1	Gulo gulo NC009685 3	0,17961812
Martes flavigula NC012141 3	Lynx lynx NC027083 4	0,1796293
Viverra zibetha T609 1	Ommatophoca rossii AY377287etc 1	0,17963983
Canis adustus KT448271 1	Bdeogale nigripes GLC15 1	0,17964055
Otaria byronia OTAB 1	Meles leucurus NC039173 4	0,17964406
Otaria byronia OTAB 1	Mustela sibirica NC020637 6	0,17964475

Spilogale putorius NC010497 1	Urva brachyura KY117547 1	0,19281399
Ursus arctos GU573486 5	Odobenus rosmarus NC004029 29	0,19282046
Neofelis nebulosa NC008450 3	Canis latrans NC008093 7	0,19282155
Odobenus rosmarus NC004029 29	Ailuropoda melanoleuca NC009492 5	0,19282161
Urva semitorquata MH464789 1	Cuon alpinus NC013445 3	0,19282243
Suricata suricatta SSM10 1	Conepatus chinga NC042596 1	0,19282638
Viverra tangalunga MH464792 1	Tapirus terrestris T358	0,19282752
Helarctos malayanus NC009968 2	Cuon alpinus NC013445 3	0,19283918
Canis lupus chanco NC010340 4	Arctocephalus forsteri NC004023 28	0,1928412
Otocyon megalotis SAF1 2	Ailurus fulgens styani NC009691 1	0,19284476
Vulpes ferrilata NC027935 1	Paradoxurus hermaphroditus NC03959	0,19284554
Lutrogale perspicillata NC035811 1	Conepatus chinga NC042596 1	0,1928461
Vulpes lagopus NC026529 3	Mustela nivalis T306 5	0,19284879
Zalophus wollebaeki SRR4431565 1	Cynogale bennetti KY117544 1	0,19284883
Paradoxurus hermaphroditus NLNC 1	Otocyon megalotis SAF1 2	0,19285096
Odobenus rosmarus NC004029 29	Canis adustus KT448271 1	0,19285156
Poecilogale albinucha T602 1	Galidia elegans D146 1	0,19285251
Urocyon cinereoargenteus NC026723 2	Felis chaus NC028307 1	0,1928535
Nasua nasua NC020647 1	Leopardus wiedii NC028318 1	0,19285622
Ursus maritimus NC003428 31	Paradoxurus hermaphroditus NC03959	0,19285771
Vulpes zerda KJ603240 1	Ursus spelaeus NC011112 8	0,19285969
Ursus arctos GU573486 5	Paradoxurus hermaphroditus NC03959	0,19286413
Urocyon cinereoargenteus NC026723 2	Arctictis binturong T605 2	0,19286489
Panthera onca KP202264 2	Ailuropoda melanoleuca NC009492 5	0,19286625
Nasua nasua NC020647 1	Mephitis mephitis NC020648 1	0,19287041
Ursus spelaeus EU327344 13	Otocyon megalotis SAF1 2	0,19287086
Ursus thibetanus mupinensis NC00875	Panthera leo spelaea KX258452 2	0,1928715
Otocyon megalotis SAF1 2	Melursus ursinus NC009970 2	0,19287159
Canis aureus KT448274 1	Arctocephalus townsendi NC008420 1	0,19287224
Panthera leo NERO 19	Arctocephalus forsteri NC004023 28	0,19287266
Parahyaena brunnea NC038159 15	Lycalopex sechurae KT448284 1	0,19287494
Panthera onca NC022842 1	Ailuropoda melanoleuca NC009492 5	0,19287698
Ursus maritimus GU573488 Svalbard	Odobenus rosmarus NC004029 29	0,19288091
Ursus thibetanus laniger MH281753 2	Panthera tigris amoyensis NC014770 2	0,19288112
Ursus arctos pruinosus MG066703 3	Odobenus rosmarus NC004029 29	0,19288117
Panthera leo NERO 19	Arctocephalus townsendi NC008420 1	0,1928847
Ursus americanus JX196366 3	Galidia elegans D146 1	0,19288488
Neofelis nebulosa NC008450 3	Martes zibellina NC011579 39	0,19288495
Spilogale putorius NC010497 1	Otocolobus manul NC028323 1	0,19289261
Urocyon littoralis catalinae KP129018	Lutra lutra LC050126 1	0,19290274
Prionodon pardicolor NC024569 2	Eumetopias jubatus NC004030 10	0,19290619
Vulpes ferrilata NC027935 1	Arctocephalus pusillus NC008417 1	0,19290975
Panthera uncia NC010638 1	Meles anakuma NC009677 1	0,1929125
Vulpes corsac NC023958 1	Ursus arctos GU573491 207	0,19291362
Vulpes vulpes NC008434 5	Hyaena hyaena NC020669 1	0,19291389
Odobenus rosmarus NC004029 29	Chrotogale owstoni T607 1	0,19291404
Vulpes ferrilata NC027935 1	Galerella sanguinea T378 1	0,19291465
Speothos venaticus C48 2	Urva semitorquata MH464789 1	0,19291635
Mephitis mephitis NC020648 1	Cuon alpinus NC013445 3	0,19291727
Lontra canadensis SRR10409165 1	Crocuta crocuta NC020670 3	0,19291793
Viverricula indica KX891751 1	Bassaricyon neblina SRX1097850 1	0,19291873
Prionodon pardicolor NC024569 2	Arctocephalus forsteri KT693377 17	0,19291911
Vulpes lagopus NC026529 3	Ursus arctos GU573486 5	0,19291916
Nasua nasua NC020647 1	Lynx pardinus NC028319 161	0,19292085
Ursus arctos pruinosus MG066703 3	Cynictis penicillata T375 1	0,19292213
Ursus arctos pruinosus MG066703 3	Atilax paludinosus T606 1	0,19292256
Pardofelis marmorata NLN3 2	Bassaricyon neblina SRX1097850 1	0,1929239
Prionodon pardicolor NC024569 2	Phocarctos hookeri NC008418 1	0,19292422
Prionodon linsang ERR2391707 1	Galictis vittata T412 1	0,19292581
Vulpes lagopus NC026529 3	Melursus ursinus NC009970 2	0,19292723
Prionailurus bengalensis CKM45 20	Nasua nasua NC020647 1	0,1929279
Vulpes lagopus NC026529 3	Ictonyx striatus T299 1	0,19293243
Ursus arctos AP012576 6	Atilax paludinosus T606 1	0,19293249
Vulpes ferrilata NC027935 1	Diplogale hosei MH464790 1	0,19293293
Zalophus californianus NC008416 1	Cynogale bennetti KY117544 1	0,19293535
Speothos venaticus C48 2	Panthera tigris NC010642 35	0,1929381
Ursus arctos GU573486 5	Panthera tigris NC010642 35	0,19294241
Ursus arctos AP012576 6	Otocolobus manul NC028323 1	0,19294684
Vulpes lagopus NC026529 3	Spilogale putorius NC010497 1	0,19294959
Neofelis nebulosa NC008450 3	Ailurus fulgens NC011124 1	0,19295032
Speothos venaticus C48 2	Urva javanica/auropunctata NC006835	0,19295336
Urocyon littoralis catalinae KP129018	Gulo gulo NC009685 3	0,19295357
Panthera leo spelaea KX258452 2	Galictis vittata T412 1	0,19295469
Cuon alpinus NC013445 3	Conepatus chinga NC042596 1	0,19296031
Lutrogale perspicillata NC035811 1	Hemigalus derbyanus MH464791 1	0,1929688
Urocyon littoralis catalinae KP129018	Eupleres goudotii D128 1	0,19296883
Vulpes vulpes NC008434 5	Meles leucurus NC039173 4	0,19297026
Vulpes vulpes NC008434 5	Suricata suricatta SSM10 1	0,19297118
Nasua nasua NC020647 1	Fossa fossana D350 1	0,19297157
Vulpes zerda KJ603240 1	Urva javanica T413 1	0,19297221
Urocyon cinereoargenteus NC026723 2	Mungos mungo/gambianus SRR77048	0,19297262
Melogale moschata V0735A 1	Cynogale bennetti KY117544 1	0,19297282
Vulpes zerda KJ603240 1	Bassariscus sumichrasti SRX1099089 1	0,19297364
Urocyon littoralis catalinae KP129018	Lutra sumatrana NC035810 1	0,19297417
Panthera pardus japonensis KJ866876	Conepatus chinga NC042596 1	0,19297424
Neofelis nebulosa NC008450 3	Canis lupus chanco NC010340 4	0,19298559
Urocyon cinereoargenteus NC026723 2	Profelis aurata NC028299 1	0,19298854
Proteles cristata T393 6	Arctocephalus australis MG023139 1	0,1929929
Ursus arctos GU573491 207	Chrysocyon brachyurus NC024172 1	0,19300334
Zalophus californianus NC008416 1	Urocyon littoralis catalinae KP129018	0,19300443
Ursus arctos GU573491 207	Panthera onca NC022842 1	0,19300561
Panthera leo spelaea KX258452 2	Otaria byronia OTAB 1	0,19300719
Ursus arctos AP012576 6	Paradoxurus hermaphroditus NC03959	0,19300865
Neophoca cinerea NC008419 1	Lycalopex sechurae KT448284 1	0,19301277
Ursus americanus JX196366 3	Cynogale bennetti KY117544 1	0,19301648
Ursus americanus JX196366 3	Panthera uncia NC010638 1	0,19303095
Urocyon littoralis catalinae KP129018	Lutra lutra NC011358 9	0,1930365
Vulpes corsac NC023958 1	Arctocephalus australis MG023139 1	0,19303797
Ursus americanus JX196366 3	Panthera tigris NC010642 35	0,19303805
Ursus americanus JX196366 3	Panthera tigris amoyensis NC014770 2	0,19303811
Chrysocyon brachyurus NC024172 1	Arctocephalus forsteri NC004023 28	0,19303882
Vulpes ferrilata NC027935 1	Bassariscus sumichrasti SRX1099089 1	0,19304472
Ursus arctos isabellinus 18B5 2	Eupleres goudotii D128 1	0,19304703

Otaria byronia OTAB 1	Mustela itatsi NC034330 19	0,17964609
Arctocephalus pusillus NC008417 1	Ailurus fulgens styani NC009691 1	0,17964685
Ursus thibetanus formosanus NC009331 1	Martes flavigula NC012141 3	0,17964755
Viverricula indica KX891751 1	Meles meles T303 3	0,17964788
Ursus thibetanus formosanus NC009331 1	Eumetopias jubatus NC004030 10	0,17964852
Mustela itatsi NC034330 19	Lynx pardinus NC028319 161	0,17964952
Martes foina NC020643 1	Arctocephalus australis MG023139 1	0,17965011
Prionailurus planiceps NC028312 6	Mustela nigripes NC024942 1	0,17965046
Puma yagouaroundi NC028311 1	Mustela putorius NC020638 4	0,17965071
Prionailurus rubiginosus NC028304 2	Mustela itatsi NC034330 19	0,17965083
Prionailurus bengalensis NC028301 12	Lycalopex sechurae KT448284 1	0,1796525
Viverricula indica NC025296 2	Viverra tangalunga MH464792 1	0,17965262
Mustela nigripes NC024942 1	Leopardus geoffroyi NC028320 1	0,17965318
Panthera onca NC022842 1	Canis adustus KT448271 1	0,17965423
Paguma larvata PDDS11 2	Monachus schauinslandi NC008421 1	0,17965645
Pusa hispida NC 008433 1	Canis aureus KT448274 1	0,17965668
Hemigalus derbyanus MH464791 1	Bassariscus sumichrasti SRX1099089 1	0,17965706
Ursus arctos GU573486 5	Nandinia binotata NC024567 1	0,17965712
Viverra zibetha T609 1	Bassariscus sumichrasti SRX1099089 1	0,17966133
Prionailurus rubiginosus NC028304 2	Neovison vison NC020641 3	0,17966257
Prionailurus bengalensis CKM45 20	Martes martes T302 3	0,1796643
Lycaon pictus NC028427 2	Felis silvestris lybica KP202275 4	0,17966607
Nandinia binotata NC024567 1	Lycalopex sechurae KT448284 1	0,17966826
Ommatophoca rossii AY377287etc 1	Conepatus chinga NC042596 1	0,17966838
Tremarctos ornatus NC009969 2	Smilodon populator MF871700 1	0,17967039
Mustela putorius NC020638 4	Canis adustus KT448271 1	0,17967808
Phoca groenlandica NC008429 54	Canis lupus familiaris NC002008 1231	0,17968151
Smilodon populator MF871700 1	Meles leucurus NC039173 4	0,17968592
Mirounga angustirostris SRR10331586 1	Lycalopex sechurae KT448284 1	0,1796892
Prionailurus rubiginosus NC028304 2	Martes flavigula NC012141 3	0,17969164
Ursus americanus JX196366 3	Mustela erminea T305 2	0,17969376
Monachus schauinslandi NC008421 1	Canis mesomelas KT448280 1	0,17969677
Erignathus barbatus NC008426 1	Cynogale bennetti KY117544 1	0,17969716
Smilodon populator MF871700 1	Mustela kathiah NC023210 1	0,17969757
Mustela eversmannii NC028013 1	Callorhinus ursinus NC008415 1	0,17970027
Paguma larvata PDDS11 2	Halichoerus grypus NC001602 2	0,17970099
Meles leucurus NC039173 4	Fossa fossana D350 1	0,17970173
Procyon lotor AB462049 4	Hemigalus derbyanus MH464791 1	0,17970243
Phoca largha NC008430 1	Panthera pardus NC010641 5	0,17970341
Nandinia binotata NC024567 1	Arctocephalus australis MG023139 1	0,17970455
Genetta abyssinica MG489822 1	Bassariscus sumichrasti SRX1099089 1	0,17970464
Nandinia binotata NC024567 1	Arctocephalus gazella BK010918 1	0,17970531
Taxidea taxus NC020646 1	Paradoxurus jerdoni MH464793 1	0,17970796
Martes flavigula NC012141 3	Arctocephalus forsteri NC004023 28	0,17970876
Meles meles T303 3	Leopardus wiedii NC028318 1	0,17971338
Pusa sibirica NC008432 2	Lycalopex sechurae KT448284 1	0,17971356
Martes martes T302 3	Conepatus chinga NC042596 1	0,17971428
Mustela frenata NC020640 1	Lynx canadensis NC028313 1	0,17971623
Viverricula indica KX891745 1	Mustela sibirica AP017394 11	0,17971624
Leptailurus serval NC028316 1	Ailurus fulgens styani NC009691 1	0,17971687
Mustela nigripes NC024942 1	Lynx rufus NC014456 3	0,17971691
Monachus schauinslandi NC008421 1	Helogale parvula SRR7637809 1	0,17971784
Ursus maritimus GU573488 Svalbard	Nandinia binotata NC024567 1	0,17971802
Viverricula indica KX891751 1	Mustela erminea T305 2	0,17971838
Otocolobus manul NC028323 1	Mustela putorius NC020638 4	0,17971876
Monachus schauinslandi NC008421 1	Eupleres goudotii D128 1	0,17971945
Viverricula indica NC025296 2	Mustela itatsi NC034330 19	0,17972003
Prionailurus bengalensis CKM45 20	Martes melampus NC009678 1	0,17972006
Taxidea taxus NC020646 1	Prionailurus planiceps NC028312 6	0,17972007
Mustela erminea T305 2	Felis chaus NC028307 1	0,17972043
Taxidea taxus NC020646 1	Pardofelis marmorata NLN3 2	0,17972165
Spilogale putorius NC010497 1	Halichoerus grypus NC001602 2	0,17972173
Lynx canadensis NC028313 1	Bassariscus sumichrasti SRX1099089 1	0,17972194
Mustela erminea T305 2	Lycaon pictus NC028427 2	0,17972304
Neovison vison NC020641 3	Canis aureus KT448274 1	0,17972323
Ursus thibetanus mupinensis NC008753 2	Mustela eversmannii NC028013 1	0,1797245
Prionailurus planiceps NC028312 6	Mustela nivalis T306 5	0,1797276
Otocolobus manul NC028323 1	Canis aureus KT448274 1	0,17972802
Neovison vison NC020641 3	Genetta genetta T297 1	0,17972933
Caracal caracal NC028306 1	Arctodus simus NC011116 1	0,17973007
Mustela nivalis T306 5	Leopardus guigna NC028321 1	0,17973042
Ursus spelaeus NC011112 8	Enhydra lutris NC009692 1	0,17973067
Neovison vison NC020641 3	Leptailurus serval NC028316 1	0,17973126
Prionodon pardicolor NC024569 2	Phoca groenlandica NC008429 54	0,17973222
Prionailurus bengalensis NC028301 12	Martes foina NC020643 1	0,17973316
Martes foina NC020643 1	Leptailurus serval NC028316 1	0,17973329
Mustela putorius NC020638 4	Acinonyx jubatus NC005212 3	0,1797335
Monachus monachus NC044972 5	Helogale parvula SRR7637809 1	0,17973845
Mustela sibirica AP017394 11	Canis lupus familiaris NC002008 1231	0,17974411
Leopardus colocolo NC028314 1	Arctodus simus NC011116 1	0,1797477
Spilogale putorius NC010497 1	Phoca vitulina NC001325 1	0,17975053
Meles leucurus NC039173 4	Civettictis civetta NC033378 1	0,17975089
Martes foina NC020643 1	Leopardus colocolo NC028314 1	0,1797516
Ursus spelaeus EU327344 13	Neovison vison NC020641 3	0,17975262
Gulo gulo NC009685 3	Arctocephalus forsteri NC004023 28	0,17975673
Meles anakuma NC009677 1	Conepatus chinga NC042596 1	0,17976904
Martes melampus NC009678 1	Arctocephalus gazella BK010918 1	0,17976929
Tremarctos ornatus NC009969 2	Martes zibellina NC011579 39	0,17976975
Martes americana NC020642 1	Callorhinus ursinus NC008415 1	0,17976986
Neophoca cinerea NC008419 1	Arctotherium sp NC030174 1	0,17977177
Pusa sibirica NC008432 2	Galidia elegans D146 1	0,17977634
Paradoxurus jerdoni MH464793 1	Mustela frenata NC020640 1	0,1797768
Mustela eversmannii NC028013 1	Homotherium latidens MF871702 3	0,17977713
Taxidea taxus NC020646 1	Mungos mungo/gambianus SRR7704821 1	0,17977853
Martes martes T302 3	Arctocephalus gazella BK010918 1	0,17977871
Meles leucurus NC039173 4	Lynx rufus NC014456 3	0,17977956
Puma concolor NC016470 22	Enhydra lutris NC009692 1	0,17978019
Meles anakuma NC009677 1	Lynx lynx NC027083 4	0,17978024
Vulpes corsac NC023958 1	Phoca fasciata NC008428 1	0,1797829
Lynx lynx NC027083 4	Callorhinus ursinus NC008415 1	0,17978305
Lynx canadensis NC028313 1	Callorhinus ursinus NC008415 1	0,17978309
Mustela sibirica AP017394 11	Lynx lynx NC027083 4	0,17978331
Neovison vison NC020641 3	Neophoca cinerea NC008419 1	0,17978375

Bassaricyon neblina SRX1097850 1	Arctotherium sp NC030174 1	0,19304994
Otaria byronia OTAB 1	Canis anthus NC027956 2	0,19304998
Ursus arctos EU497665 29	Urva javanica T413 1	0,1930505
Panthera tigris amoyensis NC014770 2	Tapirus terrestris T358	0,19305162
Panthera tigris NC010642 35	Tapirus terrestris T358	0,19305168
Mustela eversmannii NC028013 1	Crocuta crocuta NC020670 3	0,19305603
Nasua nasua NC020647 1	Leopardus tigrinus NC028317 1	0,19305727
Canis lupus familiaris NC002008 1231	Arctocephalus forsteri NC004023 28	0,19305846
Ursus arctos EU497665 29	Paradoxurus hermaphroditus NC03959	0,19305974
Ursus arctos pruinosus MG066703 3	Otocyon megalotis SAF1 2	0,19306056
Vulpes corsac NC023958 1	Ursus arctos AP012576 6	0,19306158
Galictis vittata T412 1	Bdeogale nigripes GLC15 1	0,19306177
Urocyon littoralis catalinae KP129018	Arctictis binturong T605 2	0,19306688
Mephitis mephitis NC020648 1	Hyaena hyaena NC020669 1	0,19307019
Otaria byronia OTAB 1	Canis lupus chanco NC010340 4	0,1930739
Speothos venaticus C48 2	Galictis vittata T412 1	0,19307478
Lutrogale perspicillata NC035811 1	Canis lupus familiaris NC002008 1231	0,193076
Lycalopex sechurae KT448284 1	Arctocephalus pusillus NC008417 1	0,19307636
Paradoxurus hermaphroditus NC03959	Gulo gulo NC009685 3	0,19308659
Canis anthus NC027956 2	Arctocephalus forsteri NC004023 28	0,19308735
Panthera leo spelaea KX258452 2	Melursus ursinus NC009970 2	0,19308868
Spilogale putorius NC010497 1	Felis catus NC001700 2	0,1930915
Tremarctos ornatus NC009969 2	Panthera pardus NC010641 5	0,19309406
Vulpes zerda KJ603240 1	Eupleres goudotii D128 1	0,19310559
Zalophus wollebaeki SRR4431565 1	Cuon alpinus NC013445 3	0,19310568
Vulpes vulpes NC008434 5	Cryptoprocta ferox CFC13 1	0,19311577
Ursus arctos GU573491 207	Bdeogale nigripes GLC15 1	0,19311763
Vulpes zerda KJ603240 1	Prionodon pardicolor NC024569 2	0,19311793
Procyon lotor AB462049 4	Otocyon megalotis SAF1 2	0,19311878
Speothos venaticus C48 2	Prionodon linsang ERR2391707 1	0,19312115
Lycaon pictus NC028427 2	Cryptoprocta ferox CFC13 1	0,19312565
Puma yagouaroundi NC028311 1	Nasua nasua NC020647 1	0,19312614
Suricata suricatta SSM10 1	Galictis vittata T412 1	0,1931293
Vulpes vulpes NC008434 5	Mephitis mephitis NC020648 1	0,19313152
Ursus arctos AP012576 6	Urva javanica T413 1	0,19313382
Ursus spelaeus NC011112 8	Eupleres goudotii D128 1	0,1931354
Panthera leo spelaea KX258452 2	Arctotherium sp NC030174 1	0,19313905
Ursus arctos pruinosus MG066703 3	Panthera leo spelaea KX258452 2	0,19313918
Odobenus rosmarus NC004029 29	Lutrogale perspicillata NC035811 1	0,19314076
Ursus americanus JX196366 3	Atilax paludinosus T606 1	0,19314142
Odobenus rosmarus NC004029 29	Leopardus geoffroyi NC028320 1	0,19314487
Ursus arctos GU573486 5	Panthera leo spelaea KX258452 2	0,19314547
Panthera leo spelaea KX258452 2	Ailuropoda melanoleuca NC009492 5	0,19315071
Ursus arctos AP012576 6	Panthera onca NC022842 1	0,19315292
Neofelis nebulosa NC008450 3	Arctonyx collaris NC020645 1	0,1931538
Lontra canadensis SRR10409165 1	Cuon alpinus NC013445 3	0,19315428
Arctocephalus forsteri NC004023 28	Tapirus terrestris T358	0,19315596
Hyaena hyaena NC020669 1	Conepatus chinga NC042596 1	0,19315684
Suricata suricatta SSM10 1	Arctocephalus townsendi NC008420 1	0,1931712
Arctocephalus forsteri NC004023 28	Arctictis binturong T605 2	0,19317144
Vulpes corsac NC023958 1	Lontra canadensis SRR10409165 1	0,1931721
Vulpes lagopus NC026529 3	Arctocephalus forsteri KT693377 17	0,19317324
Lutrogale perspicillata NC035811 1	Canis latrans NC008093 7	0,19317363
Urocyon cinereoargenteus NC026723 2	Melogale moschata V0735A 1	0,1931746
Vulpes corsac NC023958 1	Ichneumia albicauda T603 1	0,19317566
Ursus arctos EU497665 29	Cynictis penicillata T375 1	0,19318499
Ursus maritimus GU573488 Svalbard	Urva javanica T413 1	0,19318518
Urocyon littoralis catalinae KP129018	Felis chaus NC028307 1	0,19319026
Speothos venaticus C48 2	Conepatus chinga NC042596 1	0,19319193
Prionailurus planiceps KY682741 4	Bassaricyon neblina SRX1097850 1	0,1931932
Parahyaena brunnea NC038159 15	Ailurus fulgens NC011124 1	0,19319321
Panthera onca NC022842 1	Tapirus terrestris T358	0,19319364
Melursus ursinus NC009970 2	Eupleres goudotii D128 1	0,19319464
Vulpes lagopus NC026529 3	Ursus arctos AP012576 6	0,19319555
Mephitis mephitis NC020648 1	Bdeogale nigripes GLC15 1	0,1931956
Ursus americanus JX196366 3	Urva javanica/auropunctata NC006835	0,19319746
Ursus arctos pruinosus MG066703 3	Pardofelis marmorata NLN3 2	0,19319838
Viverra zibetha T609 1	Odobenus rosmarus NC004029 29	0,19320019
Lycaon pictus NC028427 2	Crocuta crocuta NC020670 3	0,19320594
Neofelis nebulosa NC008450 3	Eumetopias jubatus NC004030 10	0,19320595
Zalophus californianus NC008416 1	Urocyon cinereoargenteus NC026723 2	0,1932068
Leopardus colocolo NC028314 1	Bassaricyon neblina SRX1097850 1	0,19320893
Proteles cristata T393 6	Melursus ursinus NC009970 2	0,19321504
Ursus arctos EU497665 29	Odobenus rosmarus NC004029 29	0,19321778
Ursus thibetanus thibetanus NC01111	Panthera leo spelaea KX258452 2	0,1932205
Vulpes corsac NC023958 1	Arctocephalus forsteri NC004023 28	0,19322294
Spilogale putorius NC010497 1	Felis chaus NC028307 1	0,19322714
Otaria byronia OTAB 1	Cuon alpinus NC013445 3	0,19323118
Vulpes ferrilata NC027935 1	Potos flavus T414 1	0,19324003
Vulpes ferrilata NC027935 1	Mungos mungo/gambianus SRR77048	0,19324102
Vulpes corsac NC023958 1	Arctonyx collaris NC020645 1	0,19324498
Neofelis nebulosa NC008450 3	Melursus ursinus NC009970 2	0,19324571
Lycaon pictus NC028427 2	Arctocephalus townsendi NC008420 1	0,19324988
Ursus maritimus NC003428 31	Urva javanica T413 1	0,1932525
Otaria byronia OTAB 1	Tapirus terrestris T358	0,19325261
Zalophus californianus NC008416 1	Neofelis nebulosa NC008450 3	0,1932544
Phocarctos hookeri NC008418 1	Lycaon pictus NC028427 2	0,19325469
Ursus arctos GU573486 5	Eupleres goudotii D128 1	0,19325539
Panthera uncia NC010638 1	Lutrogale perspicillata NC035811 1	0,19325851
Nasua nasua NC020647 1	Felis catus NC001700 2	0,19325875
Ursus arctos GU573486 5	Urva javanica T413 1	0,19325902
Lycalopex sechurae KT448284 1	Callorhinus ursinus NC008415 1	0,19326043
Panthera leo spelaea KX258452 2	Martes zibellina NC011579 39	0,19326332
Crocuta crocuta NC020670 3	Arctotherium sp NC030174 1	0,19326533
Lycalopex sechurae KT448284 1	Galidia elegans D146 1	0,19326875
Vulpes zerda KJ603240 1	Panthera leo spelaea KX258452 2	0,19327046
Panthera leo spelaea KX258452 2	Arctocephalus forsteri KT693377 17	0,19327122
Mephitis mephitis NC020648 1	Felis catus NC001700 2	0,19327177
Phocarctos hookeri NC008418 1	Panthera leo spelaea KX258452 2	0,19327371
Parahyaena brunnea NC038159 15	Chrysocyon brachyurus NC024172 1	0,19327645
Odobenus rosmarus NC004029 29	Catopuma badia NC028300 1	0,19327722
Cryptoprocta ferox CFC13 1	Chrysocyon brachyurus NC024172 1	0,19327728
Lycalopex sechurae KT448284 1	Arctocephalus forsteri KT693377 17	0,19327846

Panthera leo spelaea KX258452 2	Halichoerus grypus NC001602 2	0,17978439
Meles meles T303 3	Catopuma temminckii NC027115 41	0,17978446
Martes melampus NC009678 1	Leopardus geoffroyi NC028320 1	0,17978467
Meles meles T303 3	Leopardus geoffroyi NC028320 1	0,1797847
Mungos mungo MMC7 1	Monachus schauinslandi NC008421 1	0,17978491
Melogale moschata V0735A 1	Lynx lynx NC027083 4	0,17978521
Prionailurus bengalensis NC028301 12	Mustela putorius NC020638 4	0,1797854
Prionailurus rubiginosus NC028304 2	Mustela eversmannii NC028013 1	0,1797854
Prionailurus bengalensis NC028301 12	Mustela sibirica AP017394 11	0,17978542
Lutra sumatrana NC035810 1	Leopardus geoffroyi NC028320 1	0,17978579
Melogale moschata V0735A 1	Leopardus wiedii NC028318 1	0,1797868
Viverricula indica KX891745 1	Nyctereutes procyonoides NC013700 3	0,17978719
Ursus maritimus NC003428 31	Meles meles T303 3	0,17978749
Ursus arctos isabellinus 18B5 2	Meles meles T303 3	0,17978768
Mustela frenata NC020640 1	Lycaon pictus NC028427 2	0,17978792
Viverra tangalunga MH464792 1	Catopuma temminckii NC027115 41	0,17978792
Zalophus wollebaeki SRR4431565 1	Mustela itatsi NC034330 19	0,17978819
Civettictis civetta NC033378 1	Canis lupus chanco NC010340 4	0,17978834
Urva javanica T413 1	Canis adustus KT448271 1	0,17978968
Prionodon pardicolor NC024569 2	Mirounga leonina NC008422 1	0,1797902
Melogale moschata NC020644 1	Lynx lynx NC027083 4	0,17979248
Lutra sumatrana NC035810 1	Arctocephalus forsteri NC004023 28	0,17979495
Mustela kathiah NC023210 1	Chrysocyon brachyurus NC024172 1	0,17979625
Ursus thibetanus formosanus NC009331 1	Mustela frenata NC020640 1	0,17979985
Zalophus californianus NC008416 1	Mustela sibirica NC020637 6	0,17980111
Ursus thibetanus laniger MH281753 2	Mustela itatsi NC034330 19	0,17980325
Neovison vison NC020641 3	Chrysocyon brachyurus NC024172 1	0,1798068
Spilogale putorius NC010497 1	Pusa caspica NC008431 1	0,17980698
Martes martes T302 3	Canis adustus KT448271 1	0,17980915
Zalophus californianus NC008416 1	Ursus thibetanus mupinensis NC008753 2	0,17981072
Enhydra lutris NC009692 1	Conepatus chinga NC042596 1	0,17981462
Panthera leo NERO 19	Canis adustus KT448271 1	0,17981769
Erignathus barbatus NC008426 1	Cuon alpinus NC013445 3	0,17982492
Monachus monachus NC044972 5	Galictis vittata T412 1	0,17982925
Viverricula indica KX891751 1	Lycalopex sechurae KT448284 1	0,17983446
Fossa fossana D350 1	Enhydra lutris NC009692 1	0,1798352
Mustela itatsi NC034330 19	Crossarchus platycephalus C7R66 1	0,17983861
Viverra tangalunga MH464792 1	Crossarchus platycephalus C7R66 1	0,17983885
Phocarctos hookeri NC008418 1	Martes zibellina NC011579 39	0,17983915
Mustela erminea T305 2	Eupleres goudotii D128 1	0,17984128
Mirounga angustirostris SRR10331586 1	Cuon alpinus NC013445 3	0,17984204
Otaria byronia OTAB 1	Martes martes T302 3	0,1798469
Prionailurus planiceps NC028312 6	Enhydra lutris NC009692 1	0,17984785
Lynx rufus NC014456 3	Arctonyx collaris NC020645 1	0,17984837
Prionailurus viverrinus NC028305 1	Meles leucurus NC039173 4	0,1798487
Otocolobus manul NC028323 1	Arctonyx collaris NC020645 1	0,17984976
Zalophus wollebaeki SRR4431565 1	Ailurus fulgens NC011124 1	0,17985103
Mustela sibirica AP017394 11	Lynx pardinus NC028319 161	0,17985135
Viverricula indica KX891745 1	Viverra tangalunga MH464792 1	0,17985168
Viverricula indica NC025296 2	Conepatus chinga NC042596 1	0,17985168
Mustela eversmannii NC028013 1	Leopardus pardalis NC028315 1	0,1798517
Mustela sibirica AP017394 11	Leopardus pardalis T262 1	0,17985174
Meles leucurus NC039173 4	Catopuma temminckii NC027115 41	0,17985183
Viverra tangalunga MH464792 1	Felis silvestris lybica KP202275 4	0,17985213
Mustela nigripes NC024942 1	Catopuma badia NC028300 1	0,17985468
Mustela erminea T305 2	Caracal caracal NC028306 1	0,17985469
Ursus maritimus GU573488 Svalbard	Meles meles T303 3	0,17985488
Phoca groenlandica NC008429 54	Lycalopex sechurae KT448284 1	0,17985749
Viverra zibetha T609 1	Arctodus simus NC011116 1	0,17985976
Taxidea taxus NC020646 1	Galidictis fasciata DM333 1	0,1798605
Neovison vison NC020641 3	Lynx lynx NC027083 4	0,1798609
Neovison vison NC020641 3	Lynx canadensis NC028313 1	0,17986138
Panthera pardus japonensis KJ866876 8	Mirounga angustirostris SRR10331586 1	0,17986254
Genetta servalina NC024568 2	Bassariscus sumichrasti SRX1099089 1	0,17986262
Mustela sibirica AP017394 11	Mephitis mephitis NC020648 1	0,17986444
Catopuma badia NC028300 1	Bassariscus sumichrasti SRX1099089 1	0,17986599
Melursus ursinus NC009970 2	Meles meles T303 3	0,17986669
Martes americana NC020642 1	Helarctos malayanus NC009968 2	0,17986682
Spilogale putorius NC010497 1	Lutra lutra LC050126 1	0,17986867
Martes foina NC020643 1	Leopardus jacobita NC028322 1	0,1798688
Leopardus geoffroyi NC028320 1	Ictonyx striatus T299 1	0,17986992
Viverricula indica KX891745 1	Ursus thibetanus thibetanus NC011118 4	0,17987415
Proteles cristata T393 6	Lobodon carcinophaga NC008423 1	0,17987466
Monachus monachus NC044972 5	Cynictis penicillata T375 1	0,17987688
Mustela putorius NC020638 4	Canis lupus familiaris NC002008 1231	0,17987849
Mustela sibirica NC020637 6	Canis lupus familiaris NC002008 1231	0,17987888
Panthera leo NERO 19	Mirounga leonina NC008422 1	0,17988542
Civettictis civetta NC033378 1	Arctonyx collaris NC020645 1	0,17988814
Civettictis civetta NC033378 1	Canis latrans NC008093 7	0,17988896
Potos flavus T414 1	Eumetopias jubatus NC004030 10	0,17988928
Xenogale naso C07XAR110 1	Halichoerus grypus NC001602 2	0,17989042
Martes melampus NC009678 1	Arctocephalus townsendi NC008420 1	0,1799027
Mustela putorius NC020638 4	Conepatus chinga NC042596 1	0,17990689
Mustela erminea T305 2	Mungos mungo MMC7 1	0,17990874
Mustela erminea T305 2	Atilax paludinosus T606 1	0,17990953
Vulpes vulpes NC008434 5	Nandinia binotata NC024567 1	0,17990954
Potos flavus T414 1	Crossarchus platycephalus C7R66 1	0,1799106
Tremarctos ornatus NC009969 2	Martes martes T302 3	0,17991087
Otocolobus manul NC028323 1	Meles meles T303 3	0,1799171
Mustela putorius NC020638 4	Lynx lynx NC027083 4	0,17991801
Mustela sibirica NC020637 6	Lynx lynx NC027083 4	0,17991801
Vulpes corsac NC023958 1	Viverricula indica KX891745 1	0,17991805
Martes melampus NC009678 1	Leopardus pardalis NC028315 1	0,17991862
Mustela sibirica NC020637 6	Lynx pardinus NC028319 161	0,17991874
Homotherium latidens MF871702 3	Canis lupus chanco NC010340 4	0,17991909
Ommatophoca rossii AY377287etc 1	Lontra canadensis SRR10409165 1	0,17991915
Prionailurus rubiginosus NC028304 2	Mustela putorius NC020638 4	0,17991996
Mustela nigripes NC024942 1	Felis silvestris lybica KP202275 4	0,17992002
Puma yagouaroundi NC028311 1	Mustela eversmannii NC028013 1	0,17992014
Martes zibellina NC011579 39	Lynx lynx NC027083 4	0,1799108
Viverricula indica NC025296 2	Mustela sibirica AP017394 11	0,17992112
Mirounga angustirostris SRR10331586 1	Bdeogale nigripes GLC15 1	0,17992151
Canis adustus KT448271 1	Arctonyx collaris NC020645 1	0,17992155

Ursus maritimus NC003428 31	Odobenus rosmarus NC004029 29	0,193285
Ursus thibetanus laniger MH281753 2	Panthera leo spelaea KX258452 2	0,19328654
Eupleres goudotii D128 1	Cuon alpinus NC013445 3	0,19328722
Ursus arctos GU573491 207	Cuon alpinus NC013445 3	0,19329655
Mustela kathiah NC023210 1	Tapirus terrestris T358	0,19329955
Vulpes corsac NC023958 1	Potos flavus T414 1	0,19330682
Vulpes corsac NC023958 1	Helogale parvula SRR7637809 1	0,1933077
Speothos venaticus C48 2	Panthera pardus NC010641 5	0,19330922
Hyaena hyaena NC020669 1	Tapirus terrestris T358	0,19331548
Ursus maritimus GU573488 Svalbard	Eupleres goudotii D128 1	0,1933167
Proteles cristata T393 6	Conepatus chinga NC042596 1	0,19331686
Vulpes ferrilata NC027935 1	Ursus maritimus GU573488 Svalbard	0,19331789
Mellivora capensis T370 1	Lobodon carcinophaga NC008423 1	0,19332023
Ursus arctos pruinosus MG066703 3	Panthera uncia NC010638 1	0,19333257
Melursus ursinus NC009970 2	Cynictis penicillata T375 1	0,193333
Speothos venaticus C48 2	Panthera pardus japonensis KJ866876	0,19333334
Panthera tigris amoyensis NC014770 2	Mustela altaica NC021751 1	0,19333653
Odobenus rosmarus NC004029 29	Lontra canadensis SRR10409165 1	0,1933375
Ursus arctos GU573491 207	Proteles cristata T393 6	0,19333954
Ursus arctos EU497665 29	Panthera tigris NC010642 35	0,19333991
Nasua nasua NC020647 1	Acinonyx jubatus NC005212 3	0,19334398
Ursus arctos AP012576 6	Panthera uncia NC010638 1	0,19334875
Canis mesomelas KT448280 1	Arctocephalus australis MG023139 1	0,19335166
Ursus spelaeus EU327344 13	Panthera leo spelaea KX258452 2	0,19335218
Vulpes ferrilata NC027935 1	Spilogale putorius NC010497 1	0,19335362
Mephitis mephitis NC020648 1	Canis mesomelas KT448280 1	0,19336329
Vulpes zerda KJ603240 1	Conepatus chinga NC042596 1	0,19336417
Parahyaena brunnea NC038159 15	Conepatus chinga NC042596 1	0,19336496
Canis anthus NC027956 2	Arctocephalus townsendi NC008420 1	0,19337437
Vulpes ferrilata NC027935 1	Lontra canadensis SRR10409165 1	0,19337445
Vulpes corsac NC023958 1	Meles anakuma NC009677 1	0,19337822
Ursus arctos AP012576 6	Cuon alpinus NC013445 3	0,1933783
Galidia elegans D146 1	Ailurus fulgens NC011124 1	0,19338006
Vulpes ferrilata NC027935 1	Arctonyx collaris NC020645 1	0,19338021
Ursus arctos GU573491 207	Eupleres goudotii D128 1	0,19338402
Vulpes ferrilata NC027935 1	Otaria byronia OTAB 1	0,19338451
Vulpes corsac NC023958 1	Hyaena hyaena NC020669 1	0,19338483
Vulpes lagopus NC026529 3	Parahyaena brunnea NC038159 15	0,19339101
Zalophus wollebaeki SRR4431565 1	Urocyon littoralis catalinae KP129018	0,19339348
Crocuta crocuta NC020670 3	Arctocephalus townsendi NC008420 1	0,19339417
Panthera uncia NC010638 1	Otaria byronia OTAB 1	0,1933957
Caracal caracal NC028306 1	Bassaricyon neblina SRX1097850 1	0,19339803
Ursus spelaeus EU327344 13	Eupleres goudotii D128 1	0,19340491
Pardofelis marmorata NLN3 2	Mephitis mephitis NC020648 1	0,19340661
Ursus maritimus NC003428 31	Chrysocyon brachyurus NC024172 1	0,19340728
Lycalopex sechurae KT448284 1	Hyaena hyaena NC020669 1	0,19341281
Ursus arctos GU573486 5	Proteles cristata T393 6	0,19341281
Profelis aurata NC028299 1	Galictis vittata T412 1	0,19341394
Ursus spelaeus EU327344 13	Speothos venaticus C48 2	0,19341664
Ursus spelaeus NC011112 8	Crocuta crocuta NC020670 3	0,19341683
Ursus thibetanus laniger MH281753 2	Panthera tigris NC010642 35	0,19341983
Helarctos malayanus NC009968 2	Crocuta crocuta NC020670 3	0,19341992
Canis mesomelas KT448280 1	Aonyx cinerea NC035814 2	0,19343656
Prionodon linsang ERR2391707 1	Lontra canadensis SRR10409165 1	0,19344221
Urocyon cinereoargenteus NC026723 2	Potos flavus T414 1	0,19344328
Paradoxurus hermaphroditus NC03959	Arctocephalus townsendi NC008420 1	0,19344476
Vulpes lagopus NC026529 3	Neophoca cinerea NC008419 1	0,19344635
Ursus arctos EU497665 29	Eupleres goudotii D128 1	0,19345132
Vulpes zerda KJ603240 1	Paradoxurus hermaphroditus NLNC 1	0,193452
Urocyon cinereoargenteus NC026723 2	Paguma larvata PDDS11 2	0,19345583
Panthera uncia NC010638 1	Martes zibellina NC011579 39	0,19345643
Prionodon pardicolor NC024569 2	Arctocephalus gazella BK010918 1	0,19345883
Melursus ursinus NC009970 2	Galidia elegans D146 1	0,19346835
Lycalopex sechurae KT448284 1	Ichneumia albicauda T603 1	0,19346894
Ursus arctos pruinosus MG066703 3	Paradoxurus hermaphroditus NC03959	0,19347033
Canis mesomelas KT448280 1	Arctocephalus forsteri NC004023 28	0,19347131
Ursus maritimus GU573488 Svalbard	Proteles cristata T393 6	0,19347428
Ursus arctos EU497665 29	Proteles cristata T393 6	0,19347437
Speothos venaticus C48 2	Panthera tigris amoyensis NC014770 2	0,19347697
Galictis vittata T412 1	Diplogale hosei MH464790 1	0,19347748
Odobenus rosmarus NC004029 29	Leopardus tigrinus NC028317 1	0,19347863
Lutra lutra NC011358 9	Diplogale hosei MH464790 1	0,19349125
Crocuta crocuta NC020670 3	Canis aureus KT448274 1	0,19349827
Urocyon littoralis catalinae KP129018	Galerella sanguinea T378 1	0,19351122
Urocyon cinereoargenteus NC026723 2	Galerella sanguinea T378 1	0,19351129
Vulpes lagopus NC026529 3	Arctocephalus gazella BK010918 1	0,19351153
Speothos venaticus C48 2	Lutra lutra LC050126 1	0,1935156
Ursus maritimus NC003428 31	Eupleres goudotii D128 1	0,19351873
Vulpes ferrilata NC027935 1	Ursus maritimus NC003428 31	0,19351986
Urocyon cinereoargenteus NC026723 2	Arctotherium sp NC030174 1	0,19352095
Viverra zibetha T609 1	Lutrogale perspicillata NC035811 1	0,19352143
Panthera uncia NC010638 1	Arctocephalus townsendi NC008420 1	0,19352221
Urocyon littoralis catalinae KP129018	Paguma larvata PDDS11 2	0,19352315
Urocyon littoralis catalinae KP129018	Mustela altaica NC021751 1	0,19352363
Prionodon pardicolor NC024569 2	Arctocephalus australis MG023139 1	0,19352514
Vulpes vulpes NC008434 5	Ursus arctos GU573486 5	0,19352518
Ursus arctos pruinosus MG066703 3	Bdeogale nigripes GLC15 1	0,19352858
Nasua nasua NC020647 1	Leopardus geoffroyi NC028320 1	0,19353043
Panthera leo spelaea KX258452 2	Lutrogale perspicillata NC035811 1	0,19353205
Zalophus wollebaeki SRR4431565 1	Speothos venaticus C48 2	0,19353706
Panthera uncia NC010638 1	Ailuropoda melanoleuca NC009492 5	0,19353977
Odobenus rosmarus NC004029 29	Lynx rufus NC014456 3	0,19354731
Prionailurus viverrinus NC028305 1	Odobenus rosmarus NC004029 29	0,19354732
Ursus arctos AP012576 6	Proteles cristata T393 6	0,19355914
Eupleres goudotii D128 1	Conepatus chinga NC042596 1	0,19355986
Canis latrans NC008093 7	Arctocephalus forsteri NC004023 28	0,19356267
Spilogale putorius NC010497 1	Crossarchus platycephalus C7R66 1	0,19356602
Neofelis nebulosa NC008450 3	Lycaon pictus NC028427 2	0,19357567
Ursus spelaeus EU327344 13	Neofelis nebulosa NC008450 3	0,19357978
Vulpes corsac NC023958 1	Poecilogale albinucha T602 1	0,19358602
Vulpes vulpes NC008434 5	Ursus arctos GU573491 207	0,19358638
Prionodon pardicolor NC024569 2	Lutrogale perspicillata NC035811 1	0,19358858
Pusa hispida NC 008433 1	Mellivora capensis T370 1	0,19358981

Phoca vitulina NC001325 1	Urva brachyura KY117547 1	0,17992224
Mustela sibirica AP017394 11	Leopardus geoffroyi NC028320 1	0,17992234
Suricata suricatta SSM10 1	Phoca vitulina NC001325 1	0,17992263
Mustela erminea T305 2	Genetta genetta T297 1	0,17992319
Prionailurus bengalensis CKM45 20	Mustela putorius NC020638 4	0,17992404
Ursus maritimus NC003428 31	Hemigalus derbyanus MH464791 1	0,17992812
Helarctos malayanus NC009968 2	Arctocephalus townsendi NC008420 1	0,17993051
Otocolobus manul NC028323 1	Mustela nivalis T306 5	0,17993091
Prionailurus bengalensis CKM45 20	Lycalopex sechurae KT448284 1	0,17993224
Prionailurus viverrinus NC028305 1	Neovison vison NC020641 3	0,17993227
Paguma larvata PDDS11 2	Bassariscus sumichrasti SRX1099089 1	0,17993444
Meles meles T303 3	Leopardus colocolo NC028314 1	0,17993493
Meles meles T303 3	Helarctos malayanus NC009968 2	0,17993528
Zalophus californianus NC008416 1	Aonyx cinerea NC035814 2	0,17994158
Monachus monachus NC044972 5	Lontra canadensis SRR10409165 1	0,17994393
Martes foina NC020643 1	Arctocephalus forsteri NC004023 28	0,17994568
Ursus thibetanus formosanus NC009331 1	Genetta abyssinica MG489822 1	0,17994764
Eumetopias jubatus NC004030 10	Crossarchus platycephalus C7R66 1	0,17994888
Ursus thibetanus thibetanus NC011118 4	Genetta abyssinica MG489822 1	0,17995112
Mustela kathiah NC023210 1	Genetta abyssinica MG489822 1	0,17995279
Phoca vitulina NC001325 1	Canis lupus familiaris NC002008 1231	0,17995462
Caracal caracal NC028306 1	Canis lupus familiaris NC002008 1231	0,17995618
Vulpes corsac NC023958 1	Civettictis civetta NC033378 1	0,17995629
Ursus americanus JX196366 3	Arctocephalus pusillus NC008417 1	0,17995803
Phoca fasciata NC008428 1	Neofelis nebulosa NC008450 3	0,17996186
Gulo gulo NC009685 3	Arctodus simus NC011116 1	0,1799688
Lutra sumatrana NC035810 1	Arctocephalus townsendi NC008420 1	0,17997065
Mustela putorius NC020638 4	Crossarchus platycephalus C7R66 1	0,17997272
Nandinia binotata NC024567 1	Arctocephalus forsteri KT693377 17	0,179974
Neophoca cinerea NC008419 1	Martes zibellina NC011579 39	0,17997447
Otaria byronia OTAB 1	Martes zibellina NC011579 39	0,17997646
Nandinia binotata NC024567 1	Canis anthus NC027956 2	0,17997685
Tremarctos ornatus NC009969 2	Lutra sumatrana NC035810 1	0,17997724
Puma yagouaroundi NC028311 1	Enhydra lutris NC009692 1	0,17998168
Chrotogale owstoni T607 1	Bassariscus sumichrasti SRX1099089 1	0,17998175
Puma concolor NC016470 22	Lutra lutra NC011358 9	0,17998177
Salanoia concolor D378 1	Mustela frenata NC020640 1	0,17998272
Meles leucurus NC039173 4	Felis silvestris lybica KP202275 4	0,17998309
Prionailurus bengalensis NC028301 12	Meles anakuma NC009677 1	0,17998353
Meles leucurus NC039173 4	Leptailurus serval NC028316 1	0,17998418
Puma concolor NC016470 22	Mustela kathiah NC023210 1	0,17998604
Leopardus pardalis T262 1	Callorhinus ursinus NC008415 1	0,17998676
Mustela nigripes NC024942 1	Leopardus jacobita NC028322 1	0,17998721
Otocolobus manul NC028323 1	Mustela kathiah NC023210 1	0,17998724
Ursus thibetanus mupinensis NC008753 2	Melogale moschata KP726273 1	0,17998743
Phoca fasciata NC008428 1	Paradoxurus hermaphroditus NC039591 1	0,17998788
Mirounga leonina NC008422 1	Hyaena hyaena NC020669 1	0,1799881
Pardofelis marmorata NLN3 2	Mustela erminea T305 2	0,17998828
Paradoxurus jerdoni MH464793 1	Conepatus chinga NC042596 1	0,17998892
Martes zibellina NC011579 39	Felis silvestris lybica KP202275 4	0,17998973
Martes pennanti NC020664 16	Genetta servalina NC024568 2	0,17999057
Viverricula indica KX891745 1	Arctotherium sp NC030174 1	0,17999059
Vulpes vulpes NC008434 5	Monachus schauinslandi NC008421 1	0,17999126
Melogale moschata NC020644 1	Lynx canadensis NC028313 1	0,17999458
Prionailurus bengalensis NC028301 12	Mustela altaica NC021751 1	0,17999487
Phoca fasciata NC008428 1	Panthera uncia NC010638 1	0,17999493
Prionailurus rubiginosus NC028304 2	Melogale moschata NC020644 1	0,17999554
Ursus arctos GU573486 5	Meles meles T303 3	0,17999589
Phoca groenlandica NC008429 54	Lycaon pictus NC028427 2	0,1799974
Martes martes T302 3	Felis margarita NC028308 1	0,17999774
Ursus thibetanus laniger MH281753 2	Melogale moschata KP726273 1	0,17999795
Neovison vison NC020641 3	Leopardus jacobita NC028322 1	0,17999822
Prionailurus rubiginosus NC028304 2	Bassariscus sumichrasti SRX1099089 1	0,17999893
Pusa hispida NC 008433 1	Diplogale hosei MH464790 1	0,18000084
Mustela kathiah NC023210 1	Arctictis binturong T605 2	0,18000093
Ursus thibetanus formosanus NC009331 1	Mustela kathiah NC023210 1	0,18000303
Leopardus colocolo NC028314 1	Callorhinus ursinus NC008415 1	0,18000422
Ursus thibetanus thibetanus NC011118 4	Melogale moschata NC020644 1	0,18000713
Mustela sibirica AP017394 11	Canis lupus chanco NC010340 4	0,18001974
Ursus maritimus GU573488 Svalbard	Hemigalus derbyanus MH464791 1	0,18002033
Genetta abyssinica MG489822 1	Arctodus simus NC011116 1	0,18002276
Leptailurus serval NC028316 1	Canis lupus familiaris NC002008 1231	0,18002309
Mustela nivalis T306 5	Hemigalus derbyanus MH464791 1	0,18002495
Procyon lotor AB462049 4	Eumetopias jubatus NC004030 10	0,18002793
Hemigalus derbyanus MH464791 1	Canis lupus familiaris NC002008 1231	0,18003133
Panthera pardus NC010641 5	Monachus schauinslandi NC008421 1	0,18003133
Enhydra lutris NC009692 1	Callorhinus ursinus NC008415 1	0,18003306
Meles leucurus NC039173 4	Arctocephalus townsendi NC008420 1	0,18003549
Lutra lutra NC011358 9	Arctocephalus townsendi NC008420 1	0,18003589
Lutra lutra NC011358 9	Arctocephalus gazella BK010918 1	0,18003701
Chrotogale owstoni T607 1	Ailurus fulgens NC011124 1	0,18004224
Neophoca cinerea NC008419 1	Ailurus fulgens styani NC009691 1	0,18004681
Martes pennanti NC020664 16	Arctocephalus australis MG023139 1	0,18004726
Lutra lutra NC011358 9	Arctotherium sp NC030174 1	0,18004841
Lutra lutra NC011358 9	Arctodus simus NC011116 1	0,18005039
Puma yagouaroundi NC028311 1	Arctonyx collaris NC020645 1	0,1800507
Leopardus pardalis NC028315 1	Enhydra lutris NC009692 1	0,18005097
Otocolobus manul NC028323 1	Meles leucurus NC039173 4	0,18005152
Potos flavus T414 1	Civettictis civetta GLC19 1	0,18005255
Prionailurus bengalensis NC028301 12	Ailurus fulgens styani NC009691 1	0,18005298
Zalophus wollebaeki SRR4431565 1	Lutra lutra NC011358 9	0,18005298
Vulpes ferrilata NC027935 1	Civettictis civetta GLC19 1	0,18005337
Canis adustus KT448271 1	Bassariscus sumichrasti SRX1099089 1	0,18005353
Neovison vison NC020641 3	Urva javanica T413 1	0,18005358
Lynx pardinus NC028319 161	Lutra sumatrana NC035810 1	0,1800537
Pusa sibirica NC008432 2	Lycaon pictus NC028427 2	0,18005376
Viverra tangalunga MH464792 1	Pardofelis marmorata NLN3 2	0,18005376
Martes melampus NC009678 1	Leopardus guigna NC028321 1	0,18005389
Melogale moschata KP726273 1	Lynx lynx NC027083 4	0,1800542
Prionailurus planiceps NC028312 6	Mustela putorius NC020638 4	0,18005423
Mustela eversmannii NC028013 1	Leopardus wiedii NC028318 1	0,18005431
Puma concolor NC016470 22	Mustela nigripes NC024942 1	0,18005449
Vulpes corsac NC023958 1	Viverricula indica NC025296 2	0,18005459

Hemigalus derbyanus MH464791 1	Bassaricyon neblina SRX1097850 1	0,1935921
Speothos venaticus C48 2	Salanoia concolor D378 1	0,19359255
Vulpes corsac NC023958 1	Parahyaena brunnea NC038159 15	0,19359313
Zalophus wollebaeki SRR4431565 1	Urocyon cinereoargenteus NC026723 2	0,19359577
Ursus arctos pruinosus MG066703 3	Urva javanica T413 1	0,19359601
Nyctereutes procyonoides NC013700 3	Crocuta crocuta NC020670 3	0,19359714
Lutrogale perspicillata NC035811 1	Crocuta crocuta NC020670 3	0,19359747
Mephitis mephitis NC020648 1	Urva semitorquata MH464789 1	0,19359837
Vulpes zerda KJ603240 1	Ursus spelaeus EU327344 13	0,19360034
Tremarctos ornatus NC009969 2	Crocuta crocuta NC020670 3	0,19360121
Urocyon cinereoargenteus NC026723 2	Helarctos malayanus NC009968 2	0,19360192
Ursus arctos GU573491 207	Pardofelis marmorata NLN3 2	0,1936021
Crocuta crocuta NC020670 3	Arctocephalus forsteri NC004023 28	0,19360216
Ursus maritimus NC003428 31	Proteles cristata T393 6	0,19360894
Odobenus rosmarus NC004029 29	Genetta genetta T297 1	0,19360928
Canis lupus familiaris NC002008 1231	Arctocephalus townsendi NC008420 1	0,19361158
Ursus arctos AP012576 6	Pardofelis marmorata NLN3 2	0,19361871
Speothos venaticus C48 2	Melursus ursinus NC009970 2	0,19361898
Spilogale putorius NC010497 1	Cynictis penicillata T375 1	0,19362232
Ursus arctos AP012576 6	Chrysocyon brachyurus NC024172 1	0,19362426
Mungotictis decemlineata NC027828 1	Cuon alpinus NC013445 3	0,19363223
Procyon lotor AB462049 4	Neofelis nebulosa NC008450 3	0,19363448
Vulpes corsac NC023958 1	Conepatus chinga NC042596 1	0,19363981
Urocyon cinereoargenteus NC026723 2	Urva semitorquata MH464789 1	0,19364554
Urocyon cinereoargenteus NC026723 2	Otaria byronia OTAB 1	0,19365309
Nyctereutes procyonoides NC013700 3	Cynogale bennetti KY117544 1	0,19365358
Vulpes vulpes NC008434 5	Ursus maritimus GU573488 Svalbard	0,19365385
Vulpes lagopus NC026529 3	Ursus arctos GU573491 207	0,19365399
Nasua nasua NC020647 1	Arctotherium sp NC030174 1	0,19365436
Ursus arctos isabellinus 18B5 2	Urva brachyura KY117547 1	0,19365636
Speothos venaticus C48 2	Urva javanica T413 1	0,19365801
Urocyon cinereoargenteus NC026723 2	Mustela altaica NC021751 1	0,19365822
Ursus arctos pruinosus MG066703 3	Eupleres goudotii D128 1	0,19365947
Urocyon littoralis catalinae KP129018 1	Profelis aurata NC028299 1	0,19366185
Ursus arctos AP012576 6	Eupleres goudotii D128 1	0,1936646
Vulpes vulpes NC008434 5	Ictonyx striatus T299 1	0,19366512
Atilax paludinosus T606 1	Ailuropoda melanoleuca NC009492 5	0,19366649
Canis lupus chanco NC010340 4	Arctocephalus townsendi NC008420 1	0,19366775
Vulpes zerda KJ603240 1	Diplogale hosei MH464790 1	0,19367407
Salanoia concolor D378 1	Mephitis mephitis NC020648 1	0,19368133
Ursus spelaeus NC011112 8	Speothos venaticus C48 2	0,19368568
Ursus thibetanus formosanus NC00933	Panthera leo spelaea KX258452 2	0,19368927
Cynogale bennetti KY117544 1	Callorhinus ursinus NC008415 1	0,19369516
Melursus ursinus NC009970 2	Chrysocyon brachyurus NC024172 1	0,19369593
Suricata suricatta SSM10 1	Cuon alpinus NC013445 3	0,19369763
Martes pennanti NC020664 16	Cynogale bennetti KY117544 1	0,19370754
Speothos venaticus C48 2	Eupleres goudotii D128 1	0,19371916
Procyon lotor AB462046 3	Galidia elegans D146 1	0,19371924
Vulpes vulpes NC008434 5	Ursus arctos isabellinus 18B5 2	0,19372097
Cryptoprocta ferox CFC13 1	Ailurus fulgens styani NC009691 1	0,19372107
Vulpes vulpes NC008434 5	Ursus arctos EU497665 29	0,19372115
Ictonyx striatus T299 1	Cynogale bennetti KY117544 1	0,19372313
Urocyon cinereoargenteus NC026723 2	Mustela nivalis T306 5	0,19372691
Speothos venaticus C48 2	Galidictis fasciata DM333 1	0,19372703
Prionailurus planiceps NC028312 6	Nasua nasua NC020647 1	0,1937301
Prionailurus planiceps KY682741 4	Nasua nasua NC020647 1	0,19373031
Nasua nasua NC020647 1	Leptailurus serval NC028316 1	0,19373289
Helogale parvula SRR7637809 1	Ailuropoda melanoleuca NC009492 5	0,19373374
Vulpes vulpes NC008434 5	Ursus arctos AP012576 6	0,19373438
Ursus arctos GU573491 207	Panthera uncia NC010638 1	0,19373631
Urocyon littoralis catalinae KP129018 1	Helarctos malayanus NC009968 2	0,19373667
Speothos venaticus C48 2	Panthera uncia KP202269 1	0,19373739
Panthera leo NERO 19	Cuon alpinus NC013445 3	0,19373868
Lutra lutra NC011358 9	Cuon alpinus NC013445 3	0,19374803
Speothos venaticus C48 2	Panthera onca NC022842 1	0,19374852
Ursus thibetanus formosanus NC00933	Crocuta crocuta NC020670 3	0,19375113
Lycalopex sechurae KT448284 1	Arctocephalus australis MG023139 1	0,19375196
Canis mesomelas KT448280 1	Arctocephalus townsendi NC008420 1	0,19376059
Speothos venaticus C48 2	Panthera leo NERO 19	0,19376907
Urocyon littoralis catalinae KP129018 1	Urva semitorquata MH464789 1	0,19378018
Urocyon littoralis catalinae KP129018 1	Arctocephalus forsteri KT693377 17	0,19378139
Vulpes zerda KJ603240 1	Arctocephalus forsteri KT693377 17	0,19378167
Vulpes lagopus NC026529 3	Phocarctos hookeri NC008418 1	0,19378176
Chrotogale owstoni T607 1	Bassaricyon neblina SRX1097850 1	0,19378218
Urocyon cinereoargenteus NC026723 2	Tremarctos ornatus NC009969 2	0,19378299
Urocyon littoralis catalinae KP129018 1	Tremarctos ornatus NC009969 2	0,19378306
Prionodon linsang ERR2391707 1	Neophoca cinerea NC008419 1	0,19378395
Vulpes ferrilata NC027935 1	Procyon lotor AB462046 3	0,193785
Vulpes vulpes NC008434 5	Ursus maritimus NC003428 31	0,1937885
Ursus maritimus GU573488 Svalbard	Suricata suricatta SSM10 1	0,19379094
Ursus maritimus GU573488 Svalbard	Urva brachyura KY117547 1	0,19379121
Phocarctos hookeri NC008418 1	Otocyon megalotis SAF1 2	0,19379234
Tremarctos ornatus NC009969 2	Prionodon pardicolor NC024569 2	0,19379458
Lutrogale perspicillata NC035811 1	Ailuropoda melanoleuca NC009492 5	0,19379942
Mephitis mephitis NC020648 1	Atilax paludinosus T606 1	0,19380003
Galictis vittata T412 1	Canis anthus NC027956 2	0,19380538
Parahyaena brunnea NC038159 15	Ailuropoda melanoleuca NC009492 5	0,19381575
Prionailurus bengalensis CKM45 20	Odobenus rosmarus NC004029 29	0,19381937
Panthera onca NC022842 1	Mephitis mephitis NC020648 1	0,19382495
Ursus arctos GU573491 207	Cynogale bennetti KY117544 1	0,19384919
Urocyon cinereoargenteus NC026723 2	Arctonyx collaris NC020645 1	0,1938539
Urocyon littoralis catalinae KP129018 1	Otaria byronia OTAB 1	0,19385494
Vulpes ferrilata NC027935 1	Ursus arctos EU497665 29	0,19385681
Lontra canadensis SRR10409165 1	Chrysocyon brachyurus NC024172 1	0,19386193
Panthera leo spelaea KX258452 2	Conepatus chinga NC042596 1	0,19386424
Nasua nasua NC020647 1	Felis nigripes NC028309 1	0,19386533
Odobenus rosmarus NC004029 29	Mungos mungo MMC7 1	0,1938671
Urocyon littoralis catalinae KP129018 1	Urva javanica/auropunctata NC006835	0,1938861
Spilogale putorius NC010497 1	Urva semitorquata MH464789 1	0,19389161
Tremarctos ornatus NC009969 2	Cuon alpinus NC013445 3	0,19389175
Spilogale putorius NC010497 1	Galerella sanguinea T378 1	0,19389202
Phocarctos hookeri NC008418 1	Canis aureus KT448274 1	0,19389509
Galidia elegans D146 1	Canis mesomelas KT448280 1	0,19389707

Otocolobus manul NC028323 1	Mustela sibirica NC020637 6	0,18005548
Prionailurus rubiginosus NC028304 2	Potos flavus T414 1	0,18005556
Martes zibellina NC011579 39	Lynx canadensis NC028313 1	0,18005566
Speothos venaticus C48 2	Leptonychotes weddellii NC008424 1	0,18005581
Puma yagouaroundi NC028311 1	Mustela altaica NC021751 1	0,18006219
Taxidea taxus NC020646 1	Arctictis binturong T605 2	0,18006282
Panthera onca KP202264 2	Canis adustus KT448271 1	0,18006478
Phoca vitulina NC001325 1	Lycalopex sechurae KT448284 1	0,1800659
Leopardus wiedii NC028318 1	Arctodus simus NC011116 1	0,18006614
Ursus thibetanus thibetanus NC011118 4	Arctocephalus gazella BK010918 1	0,18006822
Homotherium latidens MF871702 3	Bassariscus sumichrasti SRX1099089 1	0,18006825
Martes foina NC020643 1	Felis margarita NC028308 1	0,18006948
Spilogale putorius NC010497 1	Lutra lutra NC011358 9	0,18006954
Martes foina NC020643 1	Leopardus wiedii NC028318 1	0,18007046
Mustela putorius NC020638 4	Leopardus colocolo NC028314 1	0,1800722
Ailurus fulgens NC011124 1	Ailuropoda melanoleuca NC009492 5	0,1800729
Ursus spelaeus NC011112 8	Mustela kathiah NC023210 1	0,18007325
Mephitis mephitis NC020648 1	Ailurus fulgens NC011124 1	0,18007618
Panthera uncia KP202269 1	Canis adustus KT448271 1	0,18007974
Leopardus pardalis T262 1	Chrysocyon brachyurus NC024172 1	0,18008003
Pusa sibirica NC008432 2	Canis lupus chanco NC010340 4	0,18008508
Phoca largha NC008430 1	Canis lupus familiaris NC002008 1231	0,18008898
Otocolobus manul NC028323 1	Canis lupus familiaris NC002008 1231	0,18009041
Neophoca cinerea NC008419 1	Martes flavigula NC012141 3	0,18009096
Poecilogale albinucha T602 1	Eumetopias jubatus NC004030 10	0,18009537
Prionailurus rubiginosus NC028304 2	Canis lupus chanco NC010340 4	0,18009634
Civettictis civetta NC033378 1	Arctodus simus NC011116 1	0,18009925
Viverricula indica KX891745 1	Lycalopex sechurae KT448284 1	0,18010071
Ursus arctos GU573486 5	Homotherium latidens MF871702 3	0,18010178
Lycaon pictus NC028427 2	Halichoerus grypus NC001602 2	0,18010187
Meles leucurus NC039173 4	Arctocephalus australis MG023139 1	0,18010364
Lutra lutra LC050126 1	Arctocephalus forsteri KT693377 17	0,18010372
Melogale moschata KP726273 1	Callorhinus ursinus NC008415 1	0,18010467
Mustela kathiah NC023210 1	Chrotogale owstoni T607 1	0,18010539
Neophoca cinerea NC008419 1	Lutra sumatrana NC035810 1	0,18010595
Mustela nigripes NC024942 1	Crossarchus platycephalus C7R66 1	0,18010777
Tremarctos ornatus NC009969 2	Meles meles T303 3	0,18010975
Vulpes lagopus NC026529 3	Pusa caspica NC008431 1	0,18011054
Meles anakuma NC009677 1	Arctocephalus pusillus NC008417 1	0,1801106
Ursus spelaeus EU327344 13	Homotherium latidens MF871702 3	0,1801113
Enhydra lutris NC009692 1	Civettictis civetta GLC19 1	0,1801133
Arctocephalus pusillus NC008417 1	Aonyx cinerea NC035814 2	0,18011588
Prionailurus planiceps KY682741 4	Enhydra lutris NC009692 1	0,1801171
Otaria byronia OTAB 1	Mustela kathiah NC023210 1	0,18011846
Meles meles T303 3	Arctotherium sp NC030174 1	0,18011905
Paradoxurus jerdoni MH464793 1	Meles meles T303 3	0,18011934
Prionailurus rubiginosus NC028304 2	Canis aureus KT448274 1	0,18011951
Vulpes vulpes NC008434 5	Viverricula indica KX891751 1	0,1801199
Mustela itatsi NC034330 19	Lynx lynx NC027083 4	0,18012019
Mustela sibirica NC020637 6	Lynx canadensis NC028313 1	0,1801202
Viverra tangalunga MH464792 1	Leopardus pardalis NC028315 1	0,18012038
Meles anakuma NC009677 1	Homotherium latidens MF871702 3	0,18012047
Zalophus wollebaeki SRR4431565 1	Lutra lutra LC050126 1	0,18012053
Viverra zibetha T609 1	Mustela nigripes NC024942 1	0,18012145
Mustela frenata NC020640 1	Catopuma badia NC028300 1	0,18012194
Leopardus geoffroyi NC028320 1	Arctonyx collaris NC020645 1	0,18012221
Zalophus wollebaeki SRR4431565 1	Mustela sibirica NC020637 6	0,18012348
Viverricula indica NC025296 2	Ailurus fulgens styani NC009691 1	0,18012401
Paradoxurus jerdoni MH464793 1	Ailurus fulgens styani NC009691 1	0,18012469
Meles meles T303 3	Arctocephalus forsteri NC004023 28	0,18012485
Pusa caspica NC008431 1	Canis mesomelas KT448280 1	0,18012577
Spilogale putorius NC010497 1	Gulo gulo NC009685 3	0,180126
Prionailurus bengalensis CKM45 20	Mustela sibirica NC020637 6	0,18012609
Ursus thibetanus mupinensis NC008753 2	Mustela frenata NC020640 1	0,18012775
Puma concolor NC016470 22	Mustela altaica NC021751 1	0,18012953
Procyon lotor AB462046 3	Prionailurus bengalensis NC028301 12	0,18012971
Ursus arctos GU573491 207	Mustela erminea T305 2	0,18012975
Ursus thibetanus mupinensis NC008753 2	Martes martes T302 3	0,18013036
Prionailurus bengalensis NC028301 12	Mustela nivalis T306 5	0,18013208
Leopardus geoffroyi NC028320 1	Bassariscus sumichrasti SRX1099089 1	0,18013424
Viverra tangalunga MH464792 1	Mephitis mephitis NC020648 1	0,18013494
Prionodon pardicolor NC024569 2	Mirounga angustirostris SRR10331586 1	0,18013713
Ursus spelaeus EU327344 13	Mustela sibirica AP017394 11	0,18013896
Ursus thibetanus laniger MH281753 2	Mustela kathiah NC023210 1	0,18013948
Ursus spelaeus EU327344 13	Mustela frenata NC020640 1	0,18013953
Mustela itatsi NC034330 19	Leopardus colocolo NC028314 1	0,18014005
Homotherium latidens MF871702 3	Canis lupus familiaris NC002008 1231	0,18014099
Ursus thibetanus laniger MH281753 2	Genetta abyssinica MG489822 1	0,18014376
Martes flavigula NC012141 3	Crossarchus platycephalus C7R66 1	0,18014811
Ursus arctos GU573491 207	Hemigalus derbyanus MH464791 1	0,18015589
Chrysocyon brachyurus NC024172 1	Acinonyx jubatus NC005212 3	0,18016259
Mustela nivalis T306 5	Genetta abyssinica MG489822 1	0,18016885
Callorhinus ursinus NC008415 1	Arctotherium sp NC030174 1	0,18016915
Nasua nasua NC020647 1	Lutra lutra LC050126 1	0,18016987
Martes pennanti NC020664 16	Hemigalus derbyanus MH464791 1	0,18017049
Neophoca cinerea NC008419 1	Lutra lutra LC050126 1	0,18017143
Viverra tangalunga MH464792 1	Conepatus chinga NC042596 1	0,18017235
Lutra sumatrana NC035810 1	Arctocephalus australis MG023139 1	0,18017313
Procyon lotor AB462049 4	Callorhinus ursinus NC008415 1	0,18017568
Mustela nigripes NC024942 1	Conepatus chinga NC042596 1	0,18017673
Vulpes lagopus NC026529 3	Mirounga leonina NC008422 1	0,1801794
Prionailurus viverrinus NC028305 1	Canis adustus KT448271 1	0,18017955
Ommatophoca rossii AY377287etc 1	Felis chaus NC028307 1	0,18018056
Tremarctos ornatus NC009969 2	Mustela kathiah NC023210 1	0,18018076
Felis silvestris lybica KP202275 4	Enhydra lutris NC009692 1	0,18018489
Viverricula indica KX891745 1	Meles meles T303 3	0,18018626
Vulpes vulpes NC008434 5	Viverricula indica KX891745 1	0,1801872
Melogale moschata V0735A 1	Arctodus simus NC011116 1	0,18018773
Prionailurus bengalensis CKM45 20	Lutra lutra LC050126 1	0,18018773
Ursus thibetanus laniger MH281753 2	Martes flavigula NC012141 3	0,18018796
Mustela putorius NC020638 4	Genetta servalina NC024568 2	0,1801886
Martes foina NC020643 1	Arctocephalus townsendi NC008420 1	0,18018879
Ursus spelaeus EU327344 13	Martes flavigula NC012141 3	0,18018913

Vulpes lagopus NC026529 3	Arctocephalus forsteri NC004023 28	0,1938998
Hyaena hyaena NC020669 1	Canis mesomelas KT448280 1	0,19390465
Ursus maritimus NC003428 31	Neofelis nebulosa NC008450 3	0,1939057
Ursus arctos isabellinus 18B5 2	Urva javanica/auropunctata NC006835	0,19390774
Nasua nasua NC020647 1	Genetta abyssinica MG489822 1	0,19390808
Arctocephalus townsendi NC008420 1	Tapirus terrestris T358	0,19390854
Vulpes zerda KJ603240 1	Arctocephalus australis MG023139 1	0,19391634
Urocyon littoralis catalinae KP129018	Arctocephalus gazella BK010918 1	0,19391772
Urocyon cinereoargenteus NC026723 2	Arctocephalus gazella BK010918 1	0,19391781
Ursus americanus JX196366 3	Chrysocyon brachyurus NC024172 1	0,19391948
Ursus arctos isabellinus 18B5 2	Suricata suricatta SSM10 1	0,19392541
Ursus arctos EU497665 29	Suricata suricatta SSM10 1	0,19392563
Vulpes zerda KJ603240 1	Panthera uncia NC010638 1	0,19393273
Speothos venaticus C48 2	Mungotictis decemlineata NC027828 1	0,19392908
Genetta genetta T297 1	Bassaricyon neblina SRX1097850 1	0,19393048
Proteles cristata T393 6	Bassaricyon neblina SRX1097850 1	0,19393115
Mephitis mephitis NC020648 1	Eupleres goudotii D128 1	0,19393155
Procyon lotor AB462049 4	Crocuta crocuta NC020670 3	0,19393502
Speothos venaticus C48 2	Poecilogale albinucha T602 1	0,19393644
Proteles cristata T393 6	Procyon lotor AB462049 4	0,19393708
Odobenus rosmarus NC004029 29	Leopardus pardalis T262 1	0,19395003
Odobenus rosmarus NC004029 29	Felis silvestris lybica KP202275 4	0,193951
Proteles cristata T393 6	Canis aureus KT448274 1	0,19395456
Otaria byronia OTAB 1	Arctictis binturong T605 2	0,19395697
Galictis vittata T412 1	Crocuta crocuta NC020670 3	0,19395745
Proteles cristata T393 6	Galictis vittata T412 1	0,19395763
Panthera leo spelaea KX258452 2	Helarctos malayanus NC009968 2	0,1939632
Neophoca cinerea NC008419 1	Canis mesomelas KT448280 1	0,1939635
Cynogale bennetti KY117544 1	Arctocephalus australis MG023139 1	0,19396792
Ursus arctos pruinosus MG066703 3	Cuon alpinus NC013445 3	0,19396974
Melursus ursinus NC009970 2	Urva javanica/auropunctata NC006835	0,1939787
Galidia elegans D146 1	Conepatus chinga NC042596 1	0,19397998
Vulpes zerda KJ603240 1	Tremarctos ornatus NC009969 2	0,19398157
Urocyon littoralis catalinae KP129018	Potos flavus T414 1	0,19398192
Panthera pardus japonensis KJ866876	Arctocephalus forsteri NC004023 28	0,19398212
Vulpes ferrilata NC027935 1	Galidia elegans D146 1	0,19398451
Vulpes corsac NC023958 1	Neophoca cinerea NC008419 1	0,19398549
Urocyon littoralis catalinae KP129018	Arctonyx collaris NC020645 1	0,19398856
Phoca largha NC008430 1	Mellivora capensis T370 1	0,19398974
Viverricula indica KX891751 1	Lutrogale perspicillata NC035811 1	0,19399281
Speothos venaticus C48 2	Xenogale naso C07XAR110 1	0,1939936
Urocyon littoralis catalinae KP129018	Mustela nivalis T306 5	0,19399628
Phocarctos hookeri NC008418 1	Panthera uncia NC010638 1	0,19399677
Panthera uncia NC010638 1	Neophoca cinerea NC008419 1	0,19399694
Vulpes lagopus NC026529 3	Ailuropoda melanoleuca NC009492 5	0,19400002
Proteles cristata T393 6	Arctocephalus townsendi NC008420 1	0,19400175
Odobenus rosmarus NC004029 29	Genetta servalina NC024568 2	0,19400935
Panthera tigris NC010642 35	Mustela altaica NC021751 1	0,1940099
Prionodon pardicolor NC024569 2	Otaria byronia OTAB 1	0,19401075
Panthera onca KP202264 2	Mephitis mephitis NC020648 1	0,19401138
Urocyon littoralis catalinae KP129018	Hemigalus derbyanus MH464791 1	0,19401196
Ursus arctos isabellinus 18B5 2	Proteles cristata T393 6	0,19401297
Odobenus rosmarus NC004029 29	Leopardus jacobita NC028322 1	0,19401757
Panthera pardus NC010641 5	Conepatus chinga NC042596 1	0,19402528
Spilogale putorius NC010497 1	Ichneumia albicauda T603 1	0,19402954
Panthera onca KP202264 2	Cuon alpinus NC013445 3	0,19403674
Neophoca cinerea NC008419 1	Tapirus terrestris T358	0,1940444
Urocyon cinereoargenteus NC026723 2	Arctocephalus forsteri KT693377 17	0,19405077
Nyctereutes procyonoides NC013700 3	Ailurus fulgens styani NC009691 1	0,1940541
Urocyon littoralis catalinae KP129018	Neophoca cinerea NC008419 1	0,19405417
Vulpes vulpes NC008434 5	Poecilogale albinucha T602 1	0,19405775
Ursus arctos GU573491 207	Urva javanica T413 1	0,19406037
Speothos venaticus C48 2	Arctocephalus pusillus NC008417 1	0,19406676
Ursus arctos GU573486 5	Urva brachyura KY117547 1	0,19406693
Xenogale naso C07XAR110 1	Ailuropoda melanoleuca NC009492 5	0,19407018
Vulpes zerda KJ603240 1	Ictonyx striatus T299 1	0,19407184
Neophoca cinerea NC008419 1	Canis lupus chanco NC010340 4	0,19407873
Urocyon cinereoargenteus NC026723 2	Hemigalus derbyanus MH464791 1	0,1940829
Puma concolor NC016470 22	Odobenus rosmarus NC004029 29	0,19408499
Urocyon cinereoargenteus NC026723 2	Urva javanica/auropunctata NC006835	0,19408821
Ursus thibetanus thibetanus NC01111	Crocuta crocuta NC020670 3	0,19409001
Cynogale bennetti KY117544 1	Arctocephalus forsteri KT693377 17	0,1941026
Panthera uncia NC010638 1	Cuon alpinus NC013445 3	0,19410425
Otaria byronia OTAB 1	Neofelis nebulosa NC008450 3	0,19410621
Ursus maritimus GU573488 Svalbard	Neofelis nebulosa NC008450 3	0,19410774
Panthera pardus japonensis KJ866876	Cuon alpinus NC013445 3	0,19410996
Urocyon littoralis catalinae KP129018	Arctocephalus australis MG023139 1	0,19411789
Vulpes vulpes NC008434 5	Phocarctos hookeri NC008418 1	0,19411826
Vulpes lagopus NC026529 3	Procyon lotor AB462046 3	0,19412103
Mungotictis decemlineata NC027828 1	Tapirus terrestris T358	0,19412291
Cryptoprocta ferox CFC13 1	Canis anthus NC027956 2	0,19412525
Panthera tigris amoyensis NC014770 2	Conepatus chinga NC042596 1	0,19412781
Suricata suricatta SSM10 1	Speothos venaticus C48 2	0,19412871
Paradoxurus hermaphroditus NC03959	Aonyx cinerea NC035814 2	0,19413091
Poecilogale albinucha T602 1	Otocyon megalotis SAF1 2	0,19413334
Conepatus chinga NC042596 1	Canis mesomelas KT448280 1	0,19413516
Nasua nasua NC020647 1	Leopardus guigna NC028321 1	0,19413666
Proteles cristata T393 6	Arctocephalus forsteri NC004023 28	0,19413826
Melursus ursinus NC009970 2	Urva javanica T413 1	0,194141
Ursus thibetanus mupinensis NC00875	Crocuta crocuta NC020670 3	0,19414306
Panthera uncia KP202269 1	Galictis vittata T412 1	0,19415402
Ursus americanus JX196366 3	Urva semitorquata MH464789 1	0,19415483
Ursus arctos pruinosus MG066703 3	Proteles cristata T393 6	0,19415772
Ursus arctos AP012576 6	Panthera leo spelaea KX258452 2	0,19416605
Otaria byronia OTAB 1	Lycalopex sechurae KT448284 1	0,1941745
Spilogale putorius NC010497 1	Lynx rufus NC014456 3	0,19417652
Phocarctos hookeri NC008418 1	Tapirus terrestris T358	0,19417778
Lontra canadensis SRR10409165 1	Helogale parvula SRR7637809 1	0,19417986
Mustela nigripes NC024942 1	Tapirus terrestris T358	0,19418342
Vulpes zerda KJ603240 1	Urva brachyura KY117547 1	0,19418459
Galidia elegans D146 1	Tapirus terrestris T358	0,19418997
Procyon lotor AB462049 4	Galidia elegans D146 1	0,1941902
Paradoxurus hermaphroditus NLNC 1	Lontra canadensis SRR10409165 1	0,19419053

Melogale moschata KP726273 1	Lynx pardinus NC028319 161	0,18018959
Mustela nigripes NC024942 1	Felis margarita NC028308 1	0,18018973
Viverra tangalunga MH464792 1	Civettictis civetta GLC19 1	0,18018974
Prionailurus viverrinus NC028305 1	Mustela nigripes NC024942 1	0,18019035
Martes americana NC020642 1	Felis silvestris lybica KP202275 4	0,18019132
Mustela putorius NC020638 4	Catopuma temminckii NC027115 41	0,1801914
Potos flavus T414 1	Leopardus geoffroyi NC028320 1	0,18019302
Prionailurus bengalensis CKM45 20	Mustela sibirica AP017394 11	0,18019336
Monachus monachus NC044972 5	Lycalopex sechurae KT448284 1	0,18019447
Poecilogale albinucha T602 1	Genetta servalina NC024568 2	0,18019676
Zalophus californianus NC008416 1	Enhydra lutris NC009692 1	0,18019706
Poecilogale albinucha T602 1	Leopardus pardalis T262 1	0,18019712
Leopardus wiedii NC028318 1	Arctocephalus pusillus NC008417 1	0,18019742
Mustela nivalis T306 5	Leopardus wiedii NC028318 1	0,18019889
Lynx rufus NC014456 3	Arctodus simus NC011116 1	0,18019925
Viverricula indica KX891745 1	Ursus thibetanus mupinensis NC008753 2	0,18020037
Martes pennanti NC020664 16	Genetta abyssinica MG489822 1	0,18020086
Phoca largha NC008430 1	Lycalopex sechurae KT448284 1	0,18020111
Ursus thibetanus laniger MH281753 2	Arctocephalus gazella BK010918 1	0,180205
Ursus thibetanus laniger MH281753 2	Meles anakuma NC009677 1	0,18020573
Martes foina NC020643 1	Leopardus guigna NC028321 1	0,18020581
Ursus thibetanus laniger MH281753 2	Melogale moschata NC020644 1	0,18020733
Potos flavus T414 1	Leopardus colocolo NC028314 1	0,18020749
Mustela erminea T305 2	Melursus ursinus NC009970 2	0,18020765
Viverricula indica KX891751 1	Ursus thibetanus thibetanus NC011118 4	0,18021074
Ursus thibetanus thibetanus NC011118 4	Mustela altaica NC021751 1	0,18021147
Phoca vitulina NC001325 1	Crocuta crocuta NC020670 3	0,18021163
Paradoxurus jerdoni MH464793 1	Canis aureus KT448274 1	0,1802119
Zalophus wollebaeki SRR4431565 1	Ursus thibetanus laniger MH281753 2	0,18021472
Viverricula indica NC025296 2	Mephitis mephitis NC020648 1	0,18021613
Taxidea taxus NC020646 1	Urva javanica/auropunctata NC006835 1	0,18022023
Tremarctos ornatus NC009969 2	Gulo gulo NC009685 3	0,18022418
Urva javanica T413 1	Halichoerus grypus NC001602 2	0,18022481
Potos flavus T414 1	Civettictis civetta NC033378 1	0,18022999
Lutra sumatrana NC035810 1	Conepatus chinga NC042596 1	0,18023029
Zalophus californianus NC008416 1	Ursus spelaeus NC011112 8	0,18023206
Martes melampus NC009678 1	Crossarchus platycephalus C7R66 1	0,18023682
Phoca vitulina NC001325 1	Neofelis nebulosa NC008450 3	0,18023698
Meles leucurus NC039173 4	Arctocephalus forsteri KT693377 17	0,1802385
Tremarctos ornatus NC009969 2	Callorhinus ursinus NC008415 1	0,18023935
Viverra tangalunga MH464792 1	Arctocephalus gazella BK010918 1	0,18024322
Poecilogale albinucha T602 1	Bassaricyon neblina SRX1097850 1	0,18024379
Pusa caspica NC008431 1	Canis anthus NC027956 2	0,18024564
Vulpes corsac NC023958 1	Erignathus barbatus NC008426 1	0,1802461
Mustela erminea T305 2	Cynictis penicillata T375 1	0,18024692
Leopardus jacobita NC028322 1	Canis adustus KT448271 1	0,1802472
Mustela nigripes NC024942 1	Arctocephalus pusillus NC008417 1	0,18024839
Martes martes T302 3	Arctocephalus australis MG023139 1	0,18024924
Ursus thibetanus laniger MH281753 2	Gulo gulo NC009685 3	0,18025193
Prionailurus planiceps NC028312 6	Meles leucurus NC039173 4	0,18025215
Meles meles T303 3	Felis margarita NC028308 1	0,18025292
Lutra lutra LC050126 1	Leopardus geoffroyi NC028320 1	0,18025339
Meles leucurus NC039173 4	Civettictis civetta GLC19 1	0,18025367
Neovison vison NC020641 3	Mungos mungo MMC7 1	0,18025394
Mustela kathiah NC023210 1	Leopardus tigrinus NC028317 1	0,18025453
Puma concolor NC016470 22	Martes melampus NC009678 1	0,18025467
Viverricula indica KX891745 1	Conepatus chinga NC042596 1	0,18025577
Mustela sibirica NC020637 6	Leopardus pardalis T262 1	0,18025585
Ursus arctos GU573491 207	Melogale moschata KP726273 1	0,18025617
Meles meles T303 3	Leopardus guigna NC028321 1	0,1802562
Prionailurus planiceps KY682741 4	Mustela sibirica NC020637 6	0,1802564
Puma yagouaroundi NC028311 1	Mustela sibirica AP017394 11	0,18025671
Genetta servalina NC024568 2	Ailurus fulgens styani NC009691 1	0,1802572
Otocolobus manul NC028323 1	Mustela sibirica AP017394 11	0,18025745
Vulpes lagopus NC026529 3	Lynx rufus NC014456 3	0,18025765
Panthera onca KP202264 2	Mustela erminea T305 2	0,18025805
Viverra tangalunga MH464792 1	Catopuma badia NC028300 1	0,18025905
Pusa hispida NC 008433 1	Paradoxurus jerdoni MH464793 1	0,18026002
Zalophus wollebaeki SRR4431565 1	Mustela nigripes NC024942 1	0,18026021
Pusa hispida NC 008433 1	Paradoxurus hermaphroditus NLNC 1	0,1802603
Viverra zibetha T609 1	Canis aureus KT448274 1	0,18026096
Viverricula indica KX891745 1	Poecilogale albinucha T602 1	0,18026364
Prionodon linsang ERR2391707 1	Neovison vison NC020641 3	0,18026404
Ursus spelaeus EU327344 13	Taxidea taxus NC020646 1	0,18026501
Mustela nivalis T306 5	Lynx pardinus NC028319 161	0,18026528
Ursus thibetanus formosanus NC009331 1	Melogale moschata KP726273 1	0,18026619
Zalophus wollebaeki SRR4431565 1	Aonyx cinerea NC035814 2	0,18026666
Prionailurus planiceps NC028312 6	Bassariscus sumichrasti SRX1099089 1	0,18026698
Leopardus jacobita NC028322 1	Bassariscus sumichrasti SRX1099089 1	0,18026727
Ursus thibetanus mupinensis NC008753 2	Mustela altaica NC021751 1	0,18026778
Mustela putorius NC020638 4	Mephitis mephitis NC020648 1	0,18026859
Lutra lutra NC011358 9	Leopardus colocolo NC028314 1	0,18026979
Zalophus californianus NC008416 1	Tremarctos ornatus NC009969 2	0,18027006
Ursus thibetanus formosanus NC009331 1	Meles anakuma NC009677 1	0,18027098
Prionailurus rubiginosus NC028304 2	Ictonyx striatus T299 1	0,18027206
Ursus thibetanus thibetanus NC011118 4	Lutra sumatrana NC035810 1	0,18027323
Martes martes T302 3	Arctocephalus forsteri NC004023 28	0,18027416
Zalophus californianus NC008416 1	Mustela nigripes NC024942 1	0,18027452
Zalophus wollebaeki SRR4431565 1	Ursus thibetanus thibetanus NC011118 4	0,18028006
Ursus maritimus GU573488 Svalbard	Homotherium latidens MF871702 3	0,18028219
Mustela erminea T305 2	Diplogale hosei MH464790 1	0,18028334
Mephitis mephitis NC020648 1	Hemigalus derbyanus MH464791 1	0,18028359
Lutra lutra LC050126 1	Conepatus chinga NC042596 1	0,18028957
Spilogale putorius NC010497 1	Mustela altaica NC021751 1	0,18029265
Mephitis mephitis NC020648 1	Arctictis binturong T605 2	0,18029627
Phoca groenlandica NC008429 54	Panthera leo NERO 19	0,18029726
Phoca fasciata NC008428 1	Panthera pardus NC010641 5	0,18030241
Nasua nasua NC020647 1	Meles meles T303 3	0,18030395
Halichoerus grypus NC001602 2	Canis aureus KT448274 1	0,1803048
Lycalopex sechurae KT448284 1	Halichoerus grypus NC001602 2	0,18030532
Neophoca cinerea NC008419 1	Martes melampus NC009678 1	0,18030696
Melogale moschata V0735A 1	Arctocephalus gazella BK010918 1	0,18031181
Martes melampus NC009678 1	Arctotherium sp NC030174 1	0,18031694

Panthera uncia NC010638 1	Arctocephalus forsteri NC004023 28	0,19419384
Urocyon littoralis catalinae KP129018	Arctotherium sp NC030174 1	0,19419432
Otocyon megalotis SAF1 2	Chrotogale owstoni T607 1	0,19419464
Vulpes ferrilata NC027935 1	Ursus arctos GU573486 5	0,19419928
Nasua nasua NC020647 1	Catopuma badia NC028300 1	0,19420379
Procyon lotor AB462046 3	Crocuta crocuta NC020670 3	0,19420524
Canis lupus familiaris NC002008 1231	Tapirus terrestris T358	0,19420741
Ursus arctos GU573491 207	Panthera leo spelaea KX258452 2	0,19421716
Prionodon pardicolor NC024569 2	Galictis vittata T412 1	0,19421989
Odobenus rosmarus NC004029 29	Leopardus pardalis NC028315 1	0,19421995
Odobenus rosmarus NC004029 29	Leopardus wiedii NC028318 1	0,19422017
Lycalopex sechurae KT448284 1	Arctocephalus townsendi NC008420 1	0,19422692
Lutra sumatrana NC035810 1	Cuon alpinus NC013445 3	0,19422841
Cuon alpinus NC013445 3	Arctocephalus forsteri KT693377 17	0,19423327
Vulpes zerda KJ603240 1	Arctocephalus forsteri NC004023 28	0,19423947
Ursus americanus JX196366 3	Speothos venaticus C48 2	0,19424543
Martes melampus NC009678 1	Cynogale bennetti KY117544 1	0,19424801
Vulpes lagopus NC026529 3	Arctocephalus townsendi NC008420 1	0,1942496
Urocyon cinereoargenteus NC026723 2	Arctocephalus australis MG023139 1	0,1942526
Lutrogale perspicillata NC035811 1	Bdeogale nigripes GLC15 1	0,19425289
Ursus arctos EU497665 29	Cynogale bennetti KY117544 1	0,19425318
Vulpes zerda KJ603240 1	Phocarctos hookeri NC008418 1	0,19425388
Urocyon cinereoargenteus NC026723 2	Neophoca cinerea NC008419 1	0,1942562
Ailurus fulgens styani NC009691 1	Tapirus terrestris T358	0,19425844
Urocyon cinereoargenteus NC026723 2	Hyaena hyaena NC020669 1	0,19426197
Ursus maritimus NC003428 31	Suricata suricatta SSM10 1	0,19426227
Ursus maritimus NC003428 31	Urva brachyura KY117547 1	0,19426255
Paradoxurus hermaphroditus NC03959	Martes martes T302 3	0,19426797
Ursus arctos GU573486 5	Suricata suricatta SSM10 1	0,19426852
Odobenus rosmarus NC004029 29	Genetta abyssinica MG489822 1	0,19426911
Vulpes corsac NC023958 1	Ictonyx striatus T299 1	0,19427182
Mungos mungo/gambianus SRR77048	Ailuropoda melanoleuca NC009492 5	0,19427255
Neofelis nebulosa NC008450 3	Cuon alpinus NC013445 3	0,19427526
Chrysocyon brachyurus NC024172 1	Arctocephalus pusillus NC008417 1	0,19427577
Hyaena hyaena NC020669 1	Chrysocyon brachyurus NC024172 1	0,19427928
Panthera leo spelaea KX258452 2	Arctocephalus gazella BK010918 1	0,19428395
Prionailurus rubiginosus NC028304 2	Odobenus rosmarus NC004029 29	0,19428648
Panthera pardus NC010641 5	Arctocephalus forsteri NC004023 28	0,19429323
Spilogale putorius NC010497 1	Xenogale naso C07XAR110 1	0,19429511
Cuon alpinus NC013445 3	Arctocephalus australis MG023139 1	0,19430046
Spilogale putorius NC010497 1	Panthera onca KP202264 2	0,19430287
Mirounga leonina NC008422 1	Mellivora capensis T370 1	0,1943119
Mellivora capensis T370 1	Erignathus barbatus NC008426 1	0,19431196
Vulpes corsac NC023958 1	Suricata suricatta SSM10 1	0,19431785
Ursus arctos GU573486 5	Neofelis nebulosa NC008450 3	0,19431964
Vulpes corsac NC023958 1	Ailurus fulgens styani NC009691 1	0,19431992
Ursus arctos isabellinus 18B5 2	Cynogale bennetti KY117544 1	0,19432075
Cynogale bennetti KY117544 1	Arctocephalus pusillus NC008417 1	0,19432113
Vulpes zerda KJ603240 1	Procyon lotor AB462049 4	0,19432263
Nyctereutes procyonoides NC013700 3	Ailurus fulgens NC011124 1	0,19432335
Otocyon megalotis SAF1 2	Ailurus fulgens NC011124 1	0,19432615
Ursus arctos GU573491 207	Urva semitorquata MH464789 1	0,19432653
Urocyon cinereoargenteus NC026723 2	Arctodus simus NC011116 1	0,19432996
Nasua nasua NC020647 1	Genetta servalina NC024568 2	0,19433172
Panthera uncia NC010638 1	Arctocephalus gazella BK010918 1	0,19433232
Ursus arctos AP012576 6	Cynogale bennetti KY117544 1	0,19433717
Nasua nasua NC020647 1	Felis chaus NC028307 1	0,19433862
Proteles cristata T393 6	Canis latrans NC008093 7	0,19433914
Odobenus rosmarus NC004029 29	Crossarchus platycephalus C7R66 1	0,1943396
Vulpes zerda KJ603240 1	Helarctos malayanus NC009968 2	0,19434009
Smilodon populator MF871700 1	Nasua nasua NC020647 1	0,19434272
Otaria byronia OTAB 1	Chrysocyon brachyurus NC024172 1	0,19434901
Odobenus rosmarus NC004029 29	Mustela nivalis T306 5	0,19435126
Lutrogale perspicillata NC035811 1	Canis lupus chanco NC010340 4	0,19435933
Panthera tigris amoyensis NC014770 2	Galictis vittata T412 1	0,19436464
Pusa sibirica NC008432 2	Mellivora capensis T370 1	0,19438006
Urocyon cinereoargenteus NC026723 2	Atilax paludinosus T606 1	0,19438611
Panthera uncia KP202269 1	Conepatus chinga NC042596 1	0,19438842
Phoca groenlandica NC008429 54	Mellivora capensis T370 1	0,19438943
Lycalopex sechurae KT448284 1	Lontra canadensis SRR10409165 1	0,19438984
Ursus arctos GU573486 5	Cynogale bennetti KY117544 1	0,19439454
Panthera leo spelaea KX258452 2	Cuon alpinus NC013445 3	0,1943974
Spilogale putorius NC010497 1	Panthera leo NERO 19	0,1944048
Prionodon pardicolor NC024569 2	Neophoca cinerea NC008419 1	0,19440687
Lontra canadensis SRR10409165 1	Arctictis binturong T605 2	0,19440831
Salanoia concolor D378 1	Ailuropoda melanoleuca NC009492 5	0,19441187
Phocarctos hookeri NC008418 1	Arctictis binturong T605 2	0,1944142
Mungotictis decemlineata NC027828 1	Mephitis mephitis NC020648 1	0,19442181
Prionodon linsang ERR2391707 1	Eumetopias jubatus NC004030 10	0,1944344
Meles anakuma NC009677 1	Tapirus terrestris T358	0,19443524
Ursus arctos isabellinus 18B5 2	Neofelis nebulosa NC008450 3	0,194444
Ursus arctos GU573491 207	Urva javanica/auropunctata NC006835	0,19444661
Zalophus wollebaeki SRR4431565 1	Neofelis nebulosa NC008450 3	0,19445182
Vulpes ferrilata NC027935 1	Helogale parvula SRR7637809 1	0,19445269
Ursus maritimus NC003428 31	Cynogale bennetti KY117544 1	0,19445536
Ursus maritimus GU573488 Svalbard	Cynogale bennetti KY117544 1	0,19445537
Phocarctos hookeri NC008418 1	Canis latrans NC008093 7	0,19445652
Conepatus chinga NC042596 1	Canis anthus NC027956 2	0,19445841
Paradoxurus jerdoni MH464793 1	Lontra canadensis SRR10409165 1	0,19445984
Vulpes vulpes NC008434 5	Ursus arctos pruinosus MG066703 3	0,19446182
Vulpes lagopus NC026529 3	Ursus arctos pruinosus MG066703 3	0,19446191
Profelis aurata NC028299 1	Nasua nasua NC020647 1	0,1944745
Proteles cristata T393 6	Procyon lotor AB462046 3	0,19447631
Panthera tigris amoyensis NC014770 2	Lutrogale perspicillata NC035811 1	0,19447941
Odobenus rosmarus NC004029 29	Hemigalus derbyanus MH464791 1	0,19448167
Panthera pardus NC010641 5	Cuon alpinus NC013445 3	0,19448628
Lycalopex sechurae KT448284 1	Arctocephalus forsteri NC004023 28	0,19448835
Panthera tigris NC010642 35	Ailuropoda melanoleuca NC009492 5	0,19449019
Neofelis nebulosa NC008450 3	Canis mesomelas KT448280 1	0,19449027
Cuon alpinus NC013445 3	Callorhinus ursinus NC008415 1	0,19450061
Vulpes corsac NC023958 1	Arctocephalus townsendi NC008420 1	0,19451825
Vulpes ferrilata NC027935 1	Meles leucurus NC039173 4	0,1945197
Vulpes lagopus NC026529 3	Arctocephalus australis MG023139 1	0,19451988

Tremarctos ornatus NC009969 2	Arctocephalus pusillus NC008417 1	0,18031782
Eumetopias jubatus NC004030 10	Acinonyx jubatus NC005212 3	0,18031804
Conepatus chinga NC042596 1	Ailurus fulgens styani NC009691 1	0,18031891
Puma concolor NC016470 22	Meles anakuma NC009677 1	0,18031989
Prionailurus rubiginosus NC028304 2	Arctonyx collaris NC020645 1	0,18032031
Paguma larvata PDDS11 2	Meles meles T303 3	0,18032046
Meles anakuma NC009677 1	Leptailurus serval NC028316 1	0,18032086
Salanoia concolor D378 1	Melogale moschata NC020644 1	0,18032096
Martes foina NC020643 1	Fossa fossana D350 1	0,18032195
Phoca fasciata NC008428 1	Canis anthus NC027956 2	0,18032214
Puma yagouaroundi NC028311 1	Ailurus fulgens NC011124 1	0,18032251
Mustela kathiah NC023210 1	Arctotherium sp NC030174 1	0,1803235
Prionailurus planiceps KY682741 4	Mustela eversmannii NC028013 1	0,18032379
Ursus spelaeus NC011112 8	Martes flavigula NC012141 3	0,18032381
Viverra tangalunga MH464792 1	Genetta servalina NC024568 2	0,18032417
Speothos venaticus C48 2	Cystophora cristata NC008427 1	0,18032421
Phoca groenlandica NC008429 54	Paradoxurus hermaphroditus NC039591 1	0,18032455
Monachus schauinslandi NC008421 1	Xenogale naso C07XAR110 1	0,18032475
Prionailurus planiceps KY682741 4	Potos flavus T414 1	0,18032481
Prionailurus planiceps NC028312 6	Potos flavus T414 1	0,18032482
Viverra zibetha T609 1	Ailurus fulgens styani NC009691 1	0,18032485
Vulpes lagopus NC026529 3	Pusa hispida NC 008433 1	0,18032555
Potos flavus T414 1	Leopardus tigrinus NC028317 1	0,1803266
Ursus maritimus GU573488 Svalbard	Mustela sibirica NC020637 6	0,18032727
Martes pennanti NC020664 16	Lynx lynx NC027083 4	0,18033125
Ursus thibetanus mupinensis NC008753 2	Mustela kathiah NC023210 1	0,18033166
Melogale moschata NC020644 1	Lynx pardinus NC028319 161	0,18033168
Helarctos malayanus NC009968 2	Callorhinus ursinus NC008415 1	0,18033311
Ursus spelaeus EU327344 13	Martes melampus NC009678 1	0,18033433
Puma concolor NC016470 22	Bassariscus sumichrasti SRX1099089 1	0,18033494
Mustela eversmannii NC028013 1	Mephitis mephitis NC020648 1	0,18033542
Prionailurus rubiginosus NC028304 2	Arctodus simus NC011116 1	0,18033566
Phoca largha NC008430 1	Lycaon pictus NC028427 2	0,1803358
Panthera pardus japonensis KJ866876 8	Canis adustus KT448271 1	0,18033603
Neovison vison NC020641 3	Leopardus geoffroyi NC028320 1	0,1803392
Prionodon pardicolor NC024569 2	Lobodon carcinophaga NC008423 1	0,18034041
Ursus spelaeus NC011112 8	Mustela putorius NC020638 4	0,18034324
Smilodon populator MF871700 1	Ailurus fulgens styani NC009691 1	0,18034503
Viverricula indica KX891745 1	Helarctos malayanus NC009968 2	0,18034644
Panthera pardus NC010641 5	Halichoerus grypus NC001602 2	0,18034944
Viverra tangalunga MH464792 1	Eumetopias jubatus NC004030 10	0,18035004
Martes flavigula NC012141 3	Fossa fossana D350 1	0,1803526
Viverricula indica KX891745 1	Mephitis mephitis NC020648 1	0,18035319
Hemigalus derbyanus MH464791 1	Chrysocyon brachyurus NC024172 1	0,18035428
Lutra lutra NC011358 9	Conepatus chinga NC042596 1	0,18035592
Spilogale putorius NC010497 1	Mustela putorius NC020638 4	0,18035649
Mustela putorius NC020638 4	Canis lupus chanco NC010340 4	0,18035664
Ursus arctos EU497665 29	Hemigalus derbyanus MH464791 1	0,18035983
Civettictis civetta GLC19 1	Canis lupus chanco NC010340 4	0,18036072
Mustela frenata NC020640 1	Cuon alpinus NC013445 3	0,18036211
Ursus maritimus NC003428 31	Homotherium latidens MF871702 3	0,18036233
Lynx pardinus NC028319 161	Gulo gulo NC009685 3	0,18036415
Martes martes T302 3	Civettictis civetta NC033378 1	0,18036571
Spilogale putorius NC010497 1	Paradoxurus hermaphroditus NLNC 1	0,18036748
Pusa hispida NC 008433 1	Neofelis nebulosa NC008450 3	0,18037049
Viverricula indica NC025296 2	Eumetopias jubatus NC004030 10	0,18037193
Lutra lutra LC050126 1	Arctocephalus australis MG023139 1	0,18037295
Nasua nasua NC020647 1	Mustela kathiah NC023210 1	0,18037336
Phocarctos hookeri NC008418 1	Martes melampus NC009678 1	0,18037416
Mirounga angustirostris SRR10331586 1	Canis mesomelas KT448280 1	0,18037483
Mustela sibirica AP017394 11	Crossarchus platycephalus C7R66 1	0,18037637
Ursus thibetanus mupinensis NC008753 2	Eumetopias jubatus NC004030 10	0,18037823
Mustela frenata NC020640 1	Mungos mungo MMC7 1	0,18037848
Potos flavus T414 1	Neophoca cinerea NC008419 1	0,18038182
Martes zibellina NC011579 39	Conepatus chinga NC042596 1	0,18038221
Mustela kathiah NC023210 1	Canis latrans NC008093 7	0,18038575
Meles meles T303 3	Lynx rufus NC014456 3	0,18038587
Enhydra lutris NC009692 1	Catopuma badia NC028300 1	0,18038595
Panthera tigris amoyensis NC014770 2	Halichoerus grypus NC001602 2	0,18038662
Prionailurus planiceps NC028312 6	Meles meles T303 3	0,18038726
Lutra lutra LC050126 1	Leopardus pardalis NC028315 1	0,18038762
Ursus arctos pruinosus MG066703 3	Hemigalus derbyanus MH464791 1	0,18038802
Puma yagouaroundi NC028311 1	Martes melampus NC009678 1	0,18038942
Phoca groenlandica NC008429 54	Xenogale naso C07XAR110 1	0,18038952
Zalophus wollebaeki SRR4431565 1	Viverra tangalunga MH464792 1	0,18039031
Viverra zibetha T609 1	Mustela putorius NC020638 4	0,18039036
Puma yagouaroundi NC028311 1	Mustela kathiah NC023210 1	0,18039055
Lutra sumatrana NC035810 1	Leopardus pardalis NC028315 1	0,1803911
Viverra zibetha T609 1	Mustela itatsi NC034330 19	0,18039112
Melogale moschata V0735A 1	Lynx canadensis NC028313 1	0,18039122
Melogale moschata V0735A 1	Leopardus pardalis NC028315 1	0,18039237
Pusa hispida NC 008433 1	Nyctereutes procyonoides NC013700 3	0,1803925
Taxidea taxus NC020646 1	Mephitis mephitis NC020648 1	0,18039399
Melogale moschata KP726273 1	Leopardus geoffroyi NC028320 1	0,1803944
Nyctereutes procyonoides NC013700 3	Leopardus pardalis T262 1	0,18039502
Lynx pardinus NC028319 161	Bassariscus sumichrasti SRX1099089 1	0,18039757
Proteles cristata T393 6	Erignathus barbatus NC008426 1	0,18039867
Nandinia binotata NC024567 1	Chrysocyon brachyurus NC024172 1	0,18039888
Prionailurus viverrinus NC028305 1	Martes martes T302 3	0,18040068
Ursus arctos GU573486 5	Mustela sibirica NC020637 6	0,18040103
Prionailurus planiceps KY682741 4	Bassariscus sumichrasti SRX1099089 1	0,18040149
Mustela nivalis T306 5	Leptailurus serval NC028316 1	0,18040207
Mustela eversmannii NC028013 1	Canis adustus KT448271 1	0,18040217
Meles anakuma NC009677 1	Leopardus colocolo NC028314 1	0,18040535
Melursus ursinus NC009970 2	Martes americana NC020642 1	0,18040572
Lycaon pictus NC028427 2	Hemigalus derbyanus MH464791 1	0,18040629
Martes foina NC020643 1	Leopardus tigrinus NC028317 1	0,18040708
Paradoxurus hermaphroditus NLNC 1	Canis aureus KT448274 1	0,18040749
Zalophus californianus NC008416 1	Martes americana NC020642 1	0,18040797
Paradoxurus jerdoni MH464793 1	Bassariscus sumichrasti SRX1099089 1	0,18040925
Gulo gulo NC009685 3	Arctocephalus forsteri KT693377 17	0,18040961
Leopardus pardalis NC028315 1	Chrysocyon brachyurus NC024172 1	0,1804167
Martes martes T302 3	Acinonyx jubatus NC005212 3	0,180418

Parahyaena brunnea NC038159 15	Cuon alpinus NC013445 3	0,1945221
Phoca fasciata NC008428 1	Mellivora capensis T370 1	0,19452333
Prionodon linsang ERR2391707 1	Aonyx cinerea NC035814 2	0,19452447
Panthera onca NC022842 1	Cuon alpinus NC013445 3	0,19452602
Paradoxurus hermaphroditus NC03959	Martes zibellina NC011579 39	0,19452788
Urocyon cinereoargenteus NC026723 2	Paradoxurus jerdoni MH464793 1	0,19453397
Speothos venaticus C48 2	Galidia elegans D146 1	0,19453442
Lutrogale perspicillata NC035811 1	Canis aureus KT448274 1	0,19454484
Ursus arctos pruinosus MG066703 3	Panthera tigris amoyensis NC014770 2	0,19455193
Otocyon megalotis SAF1 2	Galictis vittata T412 1	0,19455576
Neofelis nebulosa NC008450 3	Arctocephalus forsteri KT693377 17	0,19456908
Neofelis nebulosa NC008450 3	Canis anthus NC027956 2	0,1945719
Spilogale putorius NC010497 1	Panthera uncia KP202269 1	0,19457228
Ursus arctos EU497665 29	Neofelis nebulosa NC008450 3	0,19457923
Ursus americanus JX196366 3	Proteles cristata T393 6	0,19458023
Vulpes vulpes NC008434 5	Meles anakuma NC009677 1	0,19458948
Urocyon littoralis catalinae KP129018 1	Galidictis fasciata DM333 1	0,19459043
Vulpes ferrilata NC027935 1	Neophoca cinerea NC008419 1	0,19459186
Ursus arctos isabellinus 18B5 2	Urva semitorquata MH464789 1	0,19459595
Vulpes ferrilata NC027935 1	Hyaena hyaena NC020669 1	0,19459735
Vulpes ferrilata NC027935 1	Ichneumia albicauda T603 1	0,19460087
Urocyon cinereoargenteus NC026723 2	Paradoxurus hermaphroditus NC03959	0,19460161
Panthera leo spelaea KX258452 2	Arctocephalus forsteri NC004023 28	0,19460881
Prionailurus bengalensis NC028301 12	Odobenus rosmarus NC004029 29	0,19462231
Proteles cristata T393 6	Canis lupus chanco NC010340 4	0,19463091
Ursus americanus JX196366 3	Nasua nasua NC020647 1	0,19463633
Tremarctos ornatus NC009969 2	Neofelis nebulosa NC008450 3	0,19463889
Speothos venaticus C48 2	Eumetopias jubatus NC004030 10	0,19465023
Mustela putorius NC020638 4	Tapirus terrestris T358	0,19465038
Vulpes ferrilata NC027935 1	Arctocephalus australis MG023139 1	0,19465473
Ursus arctos GU573486 5	Urva javanica/auropunctata NC006835	0,19465505
Urocyon littoralis catalinae KP129018 1	Atilax paludinosus T606 1	0,19465537
Vulpes corsac NC023958 1	Phocarctos hookeri NC008418 1	0,19465765
Urocyon littoralis catalinae KP129018 1	Phocarctos hookeri NC008418 1	0,19465913
Ursus maritimus GU573488 Svalbard	Urva semitorquata MH464789 1	0,19466312
Suricata suricatta SSM10 1	Otocyon megalotis SAF1 2	0,19466327
Vulpes lagopus NC026529 3	Poecilogale albinucha T602 1	0,19466357
Vulpes ferrilata NC027935 1	Ursus arctos pruinosus MG066703 3	0,19466469
Ursus arctos EU497665 29	Urva brachyura KY117547 1	0,19466656
Odobenus rosmarus NC004029 29	Conepatus chinga NC042596 1	0,19466667
Otocyon megalotis SAF1 2	Lutrogale perspicillata NC035811 1	0,19466707
Bassariscus sumichrasti SRX1099089 1	Tapirus terrestris T358	0,19466732
Lutrogale perspicillata NC035811 1	Ichneumia albicauda T603 1	0,19466843
Ursus arctos GU573486 5	Urva semitorquata MH464789 1	0,19466956
Panthera onca KP202264 2	Bassaricyon neblina SRX1097850 1	0,19467427
Galictis vittata T412 1	Canis mesomelas KT448280 1	0,19467646
Felis chaus NC028307 1	Bassaricyon neblina SRX1097850 1	0,19467905
Panthera tigris NC010642 35	Lutrogale perspicillata NC035811 1	0,1946816
Canis aureus KT448274 1	Bassaricyon neblina SRX1097850 1	0,19470643
Prionodon pardicolor NC024569 2	Arctocephalus forsteri NC004023 28	0,19472279
Nasua nasua NC020647 1	Mungos mungo MMC7 1	0,19472493
Melursus ursinus NC009970 2	Cuon alpinus NC013445 3	0,19472998
Urocyon littoralis catalinae KP129018 1	Paradoxurus jerdoni MH464793 1	0,19473597
Cryptoprocta ferox CFC13 1	Tapirus terrestris T358	0,19473974
Nasua nasua NC020647 1	Leopardus jacobita NC028322 1	0,19474204
Speothos venaticus C48 2	Hyaena hyaena NC020669 1	0,19474448
Panthera leo spelaea KX258452 2	Arctocephalus townsendi NC008420 1	0,19475286
Panthera tigris NC010642 35	Galictis vittata T412 1	0,19476889
Hyaena hyaena NC020669 1	Cuon alpinus NC013445 3	0,19478288
Urocyon cinereoargenteus NC026723 2	Lontra canadensis SRR10409165 1	0,19478965
Vulpes ferrilata NC027935 1	Suricata suricatta SSM10 1	0,19478965
Nyctereutes procyonoides NC013700 3	Lutrogale perspicillata NC035811 1	0,19479063
Speothos venaticus C48 2	Lutra lutra NC011358 9	0,19479387
Prionodon linsang ERR2391707 1	Otocyon megalotis SAF1 2	0,19479731
Urocyon littoralis catalinae KP129018 1	Hyaena hyaena NC020669 1	0,19480062
Urocyon littoralis catalinae KP129018 1	Arctodus simus NC011116 1	0,19480133
Otocyon megalotis SAF1 2	Bassaricyon neblina SRX1097850 1	0,19480309
Proteles cristata T393 6	Lontra canadensis SRR10409165 1	0,19480525
Ursus arctos AP012576 6	Urva semitorquata MH464789 1	0,19481528
Crocuta crocuta NC020670 3	Ailurus fulgens styani NC009691 1	0,19481562
Ursus arctos EU497665 29	Speothos venaticus C48 2	0,19481574
Lycalopex sechurae KT448284 1	Cryptoprocta ferox CFC13 1	0,19482453
Ursus americanus JX196366 3	Nyctereutes procyonoides NC013700 3	0,19483435
Ursus maritimus GU573488 Svalbard	Urva javanica/auropunctata NC006835	0,19485082
Urocyon cinereoargenteus NC026723 2	Galidictis fasciata DM333 1	0,1948598
Ursus arctos AP012576 6	Urva javanica/auropunctata NC006835	0,19486657
Vulpes ferrilata NC027935 1	Ursus arctos GU573491 207	0,1948666
Ursus arctos GU573491 207	Suricata suricatta SSM10 1	0,19486825
Martes martes T302 3	Cynogale bennetti KY117544 1	0,19486906
Speothos venaticus C48 2	Lutra sumatrana NC035810 1	0,19486909
Urocyon littoralis catalinae KP129018 1	Paradoxurus hermaphroditus NC03959	0,19487095
Paradoxurus hermaphroditus NC03959	Otocyon megalotis SAF1 2	0,19487143
Vulpes ferrilata NC027935 1	Ursus arctos AP012576 6	0,1948797
Melursus ursinus NC009970 2	Bdeogale nigripes GLC15 1	0,19488132
Ursus arctos AP012576 6	Urva brachyura KY117547 1	0,19488508
Ursus arctos GU573491 207	Panthera tigris amoyensis NC014770 2	0,19488819
Panthera uncia NC010638 1	Melursus ursinus NC009970 2	0,19489543
Ursus americanus JX196366 3	Urocyon littoralis catalinae KP129018 1	0,19490284
Urocyon littoralis catalinae KP129018 1	Arctocephalus forsteri NC004023 28	0,19490956
Spilogale putorius NC010497 1	Salanoia concolor D378 1	0,19491833
Vulpes ferrilata NC027935 1	Arctocephalus forsteri KT693377 17	0,19492404
Urocyon cinereoargenteus NC026723 2	Xenogale naso C07XAR110 1	0,19492507
Vulpes vulpes NC008434 5	Ailurus fulgens styani NC009691 1	0,19492562
Panthera tigris NC010642 35	Cuon alpinus NC013445 3	0,19492741
Urocyon cinereoargenteus NC026723 2	Prionodon linsang ERR2391707 1	0,19492827
Ursus arctos EU497665 29	Urva semitorquata MH464789 1	0,19493245
Ursus arctos GU573491 207	Urva brachyura KY117547 1	0,19493574
Prionodon pardicolor NC024569 2	Arctocephalus townsendi NC008420 1	0,19493814
Viverra zibetha T609 1	Nasua nasua NC020647 1	0,1949382
Vulpes ferrilata NC027935 1	Parahyaena brunnea NC038159 15	0,19493988
Panthera onca KP202264 2	Nasua nasua NC020647 1	0,19494152
Vulpes zerda KJ603240 1	Proteles cristata T393 6	0,19494317
Vulpes ferrilata NC027935 1	Ictonyx striatus T299 1	0,19494528

Ursus arctos AP012576 6	Hemigalus derbyanus MH464791 1	0,18042213
Spilogale putorius NC010497 1	Mustela itatsi NC034330 19	0,18042439
Prionailurus bengalensis NC028301 12	Canis lupus familiaris NC002008 1231	0,18042675
Ommatophoca rossii AY377287etc 1	Felis catus NC001700 2	0,18042684
Homotherium latidens MF871702 3	Canis latrans NC008093 7	0,18042772
Viverra zibetha T609 1	Canis lupus chanco NC010340 4	0,18043007
Phoca largha NC008430 1	Canis lupus chanco NC010340 4	0,18043421
Halichoerus grypus NC001602 2	Galidia elegans D146 1	0,18043931
Ursus arctos AP012576 6	Homotherium latidens MF871702 3	0,18043931
Meles anakuma NC009677 1	Chrotogale owstoni T607 1	0,18043955
Catopuma temminckii NC027115 41	Canis lupus chanco NC010340 4	0,18043964
Nasua nasua NC020647 1	Mustela itatsi NC034330 19	0,18044133
Mungos mungo MMC7 1	Meles meles T303 3	0,18044314
Tremarctos ornatus NC009969 2	Melogale moschata KP726273 1	0,18044502
Pusa caspica NC008431 1	Helogale parvula SRR7637809 1	0,18044707
Martes americana NC020642 1	Arctocephalus pusillus NC008417 1	0,18044734
Chrotogale owstoni T607 1	Arctocephalus pusillus NC008417 1	0,18044822
Mustela putorius NC020638 4	Arctocephalus pusillus NC008417 1	0,18044981
Ursus americanus JX196366 3	Neovison vison NC020641 3	0,18044981
Melogale moschata NC020644 1	Fossa fossana D350 1	0,18045035
Nandinia binotata NC024567 1	Lutrogale perspicillata NC035811 1	0,18045166
Otaria byronia OTAB 1	Nandinia binotata NC024567 1	0,18045209
Cynogale bennetti KY117544 1	Canis adustus KT448271 1	0,1804522
Mustela nigripes NC024942 1	Canis latrans NC008093 7	0,18045256
Potos flavus T414 1	Paradoxurus jerdoni MH464793 1	0,18045311
Meles meles T303 3	Leopardus pardalis NC028315 1	0,18045354
Paguma larvata PDDS11 2	Meles anakuma NC009677 1	0,18045391
Lutra lutra LC050126 1	Felis silvestris lybica KP202275 4	0,18045394
Meles meles T303 3	Leopardus tigrinus NC028317 1	0,18045424
Meles meles T303 3	Felis nigripes NC028309 1	0,18045473
Lutra lutra NC011358 9	Leopardus pardalis NC028315 1	0,18045501
Prionailurus viverrinus NC028305 1	Enhydra lutris NC009692 1	0,18045527
Mustela kathiah NC023210 1	Lynx lynx NC027083 4	0,18045564
Panthera leo NERO 19	Monachus monachus NC044972 5	0,18045601
Viverra tangalunga MH464792 1	Lynx rufus NC014456 3	0,18045698
Martes melampus NC009678 1	Felis margarita NC028308 1	0,18045764
Mustela kathiah NC023210 1	Genetta servalina NC024568 2	0,18045792
Martes melampus NC009678 1	Felis chaus NC028307 1	0,18045822
Paradoxurus hermaphroditus NLNC 1	Mustela nigripes NC024942 1	0,18045825
Mustela itatsi NC034330 19	Leopardus wiedii NC028318 1	0,18045837
Viverra tangalunga MH464792 1	Felis margarita NC028308 1	0,18045853
Puma yagouaroundi NC028311 1	Mustela itatsi NC034330 19	0,18045893
Martes americana NC020642 1	Arctotherium sp NC030174 1	0,18045928
Puma yagouaroundi NC028311 1	Canis aureus KT448274 1	0,1804599
Mustela erminea T305 2	Felis catus NC001700 2	0,18046002
Mustela eversmannii NC028013 1	Catopuma temminckii NC027115 41	0,18046077
Prionailurus rubiginosus NC028304 2	Martes americana NC020642 1	0,1804608
Ursus maritimus NC003428 31	Mustela sibirica NC020637 6	0,18046193
Zalophus wollebaeki SRR4431565 1	Martes americana NC020642 1	0,18046261
Prionailurus rubiginosus NC028304 2	Canis latrans NC008093 7	0,18046308
Prionailurus bengalensis NC028301 12	Canis latrans NC008093 7	0,18046369
Phoca groenlandica NC008429 54	Panthera onca KP202264 2	0,18046655
Mustela altaica NC021751 1	Felis silvestris lybica KP202275 4	0,18046677
Lycalopex sechurae KT448284 1	Hemigalus derbyanus MH464791 1	0,18046755
Neophoca cinerea NC008419 1	Homotherium latidens MF871702 3	0,18046798
Ursus thibetanus mupinensis NC008753 2	Civettictis civetta GLC19 1	0,1804692
Viverricula indica KX891751 1	Ursus thibetanus mupinensis NC008753 2	0,18047003
Prionailurus bengalensis NC028301 12	Bassariscus sumichrasti SRX1099089 1	0,18047182
Halichoerus grypus NC001602 2	Canis lupus chanco NC010340 4	0,18047351
Ursus arctos AP012576 6	Nandinia binotata NC024567 1	0,18047516
Ailurus fulgens styani NC009691 1	Ailuropoda melanoleuca NC009492 5	0,1804772
Melogale moschata V0735A 1	Leopardus colocolo NC028314 1	0,18047813
Ursus spelaeus EU327344 13	Mustela nigripes NC024942 1	0,18047884
Ursus thibetanus thibetanus NC011118 4	Martes martes T302 3	0,18048058
Viverra zibetha T609 1	Helarctos malayanus NC009968 2	0,18048118
Ursus americanus JX196366 3	Taxidea taxus NC020646 1	0,18048155
Helogale parvula SRR7637809 1	Halichoerus grypus NC001602 2	0,1804958
Prionailurus planiceps NC028312 6	Martes flavigula NC012141 3	0,18050091
Neofelis nebulosa NC008450 3	Lobodon carcinophaga NC008423 1	0,18050599
Hydrurga leptonyx NC008425 1	Tapirus terrestris T358	0,18051
Lontra canadensis SRR10409165 1	Ailurus fulgens styani NC009691 1	0,18051241
Martes americana NC020642 1	Arctocephalus forsteri KT693377 17	0,18051301
Martes zibellina NC011579 39	Arctocephalus australis MG023139 1	0,18051301
Phocarctos hookeri NC008418 1	Nandinia binotata NC024567 1	0,18051311
Vulpes lagopus NC026529 3	Cystophora cristata NC008427 1	0,18051483
Cystophora cristata NC008427 1	Canis anthus NC027956 2	0,18051535
Pusa caspica NC008431 1	Nyctereutes procyonoides NC013700 3	0,18051566
Pusa caspica NC008431 1	Galidia elegans D146 1	0,18051703
Procyon lotor AB462046 3	Neophoca cinerea NC008419 1	0,18051948
Prionailurus bengalensis NC028301 12	Lutra lutra LC050126 1	0,18052009
Leopardus pardalis T262 1	Enhydra lutris NC009692 1	0,18052204
Arctocephalus pusillus NC008417 1	Ailurus fulgens NC011124 1	0,18052205
Melogale moschata KP726273 1	Arctotherium sp NC030174 1	0,18052216
Melogale moschata V0735A 1	Arctotherium sp NC030174 1	0,18052235
Potos flavus T414 1	Genetta servalina NC024568 2	0,18052337
Paradoxurus hermaphroditus NC039591 1	Meles leucurus NC039173 4	0,18052344
Caracal caracal NC028306 1	Callorhinus ursinus NC008415 1	0,18052368
Procyon lotor AB462049 4	Ailurus fulgens styani NC009691 1	0,18052375
Mustela eversmannii NC028013 1	Lynx lynx NC027083 4	0,18052413
Viverricula indica KX891745 1	Mustela sibirica NC020637 6	0,18052441
Phoca groenlandica NC008429 54	Urva javanica T413 1	0,18052442
Martes flavigula NC012141 3	Helarctos malayanus NC009968 2	0,1805246
Panthera pardus NC010641 5	Monachus monachus NC044972 5	0,1805246
Leopardus pardalis NC028315 1	Callorhinus ursinus NC008415 1	0,18052517
Meles anakuma NC009677 1	Leopardus geoffroyi NC028320 1	0,18052538
Paradoxurus jerdoni MH464793 1	Mustela kathiah NC023210 1	0,18052539
Prionailurus rubiginosus NC028304 2	Lutra sumatrana NC035810 1	0,18052551
Mustela sibirica NC020637 6	Felis silvestris lybica KP202275 4	0,18052588
Catopuma temminckii NC027115 41	Arctonyx collaris NC020645 1	0,18052651
Viverricula indica KX891745 1	Ailurus fulgens NC011124 1	0,18052678
Viverricula indica KX891751 1	Ailurus fulgens styani NC009691 1	0,18052733
Paguma larvata PDDS11 2	Ailurus fulgens styani NC009691 1	0,18052775
Ursus arctos EU497665 29	Meles meles T303 3	0,18052834

Urocyon littoralis catalinae KP129018	Panthera onca NC022842 1	0,19494943
Galidictis fasciata DM333 1	Ailuropoda melanoleuca NC009492 5	0,19495222
Ursus arctos AP012576 6	Suricata suricatta SSM10 1	0,19495261
Cuon alpinus NC013445 3	Arctocephalus forsteri NC004023 28	0,1949576
Otocolobus manul NC028323 1	Odobenus rosmarus NC004029 29	0,19495893
Ursus thibetanus laniger MH281753 2	Crocuta crocuta NC020670 3	0,19496226
Neofelis nebulosa NC008450 3	Lutrogale perspicillata NC035811 1	0,19497426
Urocyon cinereoargenteus NC026723 2	Arctocephalus forsteri NC004023 28	0,19497716
Vulpes vulpes NC008434 5	Lutrogale perspicillata NC035811 1	0,19499108
Ailurus fulgens NC011124 1	Tapirus terrestris T358	0,19499999
Ursus arctos pruinosus MG066703 3	Suricata suricatta SSM10 1	0,19500314
Urocyon cinereoargenteus NC026723 2	Paradoxurus hermaphroditus NLNC 1	0,19500522
Ursus thibetanus laniger MH281753 2	Urocyon cinereoargenteus NC026723 2	0,19501697
Ursus thibetanus thibetanus NC01111	Urocyon cinereoargenteus NC026723 2	0,19501738
Vulpes zerda KJ603240 1	Urva javanica/auropunctata NC006835	0,19502975
Ursus arctos AP012576 6	Panthera tigris amoyensis NC014770 2	0,19503553
Vulpes vulpes NC008434 5	Cynogale bennetti KY117544 1	0,19505173
Vulpes ferrilata NC027935 1	Arctocephalus forsteri NC004023 28	0,19505195
Galictis vittata T412 1	Cuon alpinus NC013445 3	0,19505911
Vulpes lagopus NC026529 3	Procyon lotor AB462049 4	0,19506325
Chrysocyon brachyurus NC024172 1	Arctocephalus townsendi NC008420 1	0,19507244
Urocyon cinereoargenteus NC026723 2	Panthera onca NC022842 1	0,19508414
Ursus arctos isabellinus 18B5 2	Speothos venaticus C48 2	0,19508535
Odobenus rosmarus NC004029 29	Civettictis civetta GLC19 1	0,19508894
Panthera tigris amoyensis NC014770 2	Ailuropoda melanoleuca NC009492 5	0,19509622
Ursus spelaeus EU327344 13	Crocuta crocuta NC020670 3	0,19510041
Ursus americanus JX196366 3	Urocyon cinereoargenteus NC026723 2	0,19510497
Vulpes zerda KJ603240 1	Neofelis nebulosa NC008450 3	0,19510534
Odobenus rosmarus NC004029 29	Acinonyx jubatus NC005212 3	0,19511038
Urocyon cinereoargenteus NC026723 2	Panthera pardus NC010641 5	0,19511615
Tremarctos ornatus NC009969 2	Cynogale bennetti KY117544 1	0,19512406
Urocyon littoralis catalinae KP129018	Lontra canadensis SRR10409165 1	0,19512632
Urocyon littoralis catalinae KP129018	Arctocephalus townsendi NC008420 1	0,19512731
Vulpes zerda KJ603240 1	Arctocephalus townsendi NC008420 1	0,19512737
Suricata suricatta SSM10 1	Lutrogale perspicillata NC035811 1	0,19512856
Odobenus rosmarus NC004029 29	Civettictis civetta NC033378 1	0,1951299
Canis latrans NC008093 7	Bassaricyon neblina SRX1097850 1	0,19513041
Urocyon cinereoargenteus NC026723 2	Phocarctos hookeri NC008418 1	0,19513041
Ursus maritimus NC003428 31	Urva semitorquata MH464789 1	0,19513448
Cryptoprocta ferox CFC13 1	Ailurus fulgens NC011124 1	0,19513492
Vulpes zerda KJ603240 1	Ursus arctos isabellinus 18B5 2	0,19513623
Urocyon littoralis catalinae KP129018	Paradoxurus hermaphroditus NLNC 1	0,1951399
Ursus arctos isabellinus 18B5 2	Nasua nasua NC020647 1	0,1951412
Speothos venaticus C48 2	Neophoca cinerea NC008419 1	0,19514257
Hyaena hyaena NC020669 1	Ailuropoda melanoleuca NC009492 5	0,19515415
Proteles cristata T393 6	Otocyon megalotis SAF1 2	0,19515485
Speothos venaticus C48 2	Parahyaena brunnea NC038159 15	0,19515558
Crocuta crocuta NC020670 3	Canis lupus chanco NC010340 4	0,19517958
Galidia elegans D146 1	Cuon alpinus NC013445 3	0,19518097
Arctodus simus NC011116 1	Tapirus terrestris T358	0,19519188
Urocyon cinereoargenteus NC026723 2	Urva javanica T413 1	0,19519517
Vulpes ferrilata NC027935 1	Phocarctos hookeri NC008418 1	0,19519673
Otaria byronia OTAB 1	Cynogale bennetti KY117544 1	0,1952064
Speothos venaticus C48 2	Urva brachyura KY117547 1	0,19520719
Eupleres goudotii D128 1	Ailuropoda melanoleuca NC009492 5	0,19521004
Nasua nasua NC020647 1	Caracal caracal NC028306 1	0,19521366
Lutrogale perspicillata NC035811 1	Arctictis binturong T605 2	0,19522029
Cynogale bennetti KY117544 1	Arctocephalus forsteri NC004023 28	0,19522208
Panthera pardus japonensis KJ866876	Mephitis mephitis NC020648 1	0,19522405
Odobenus rosmarus NC004029 29	Leptailurus serval NC028316 1	0,19522861
Neofelis nebulosa NC008450 3	Arctocephalus australis MG023139 1	0,19524242
Phocarctos hookeri NC008418 1	Neofelis nebulosa NC008450 3	0,19524559
Mellivora capensis T370 1	Halichoerus grypus NC001602 2	0,19524738
Ursus maritimus NC003428 31	Urva javanica/auropunctata NC006835	0,19525494
Ursus arctos EU497665 29	Urva javanica/auropunctata NC006835	0,195255
Vulpes ferrilata NC027935 1	Conepatus chinga NC042596 1	0,19525526
Neovison vison NC020641 3	Tapirus terrestris T358	0,1952638
Panthera tigris amoyensis NC014770 2	Cuon alpinus NC013445 3	0,19526431
Speothos venaticus C48 2	Helogale parvula SRR7637809 1	0,19527316
Urocyon littoralis catalinae KP129018	Panthera onca KP202264 2	0,1952756
Ursus maritimus GU573488 Svalbard	Nasua nasua NC020647 1	0,19527566
Urocyon cinereoargenteus NC026723 2	Panthera onca KP202264 2	0,19527571
Ursus maritimus GU573488 Svalbard	Speothos venaticus C48 2	0,1952872
Ursus arctos pruinosus MG066703 3	Panthera tigris NC010642 35	0,1952928
Prionailurus planiceps NC028312 6	Odobenus rosmarus NC004029 29	0,19529676
Prionailurus planiceps KY682741 4	Odobenus rosmarus NC004029 29	0,195297
Cynogale bennetti KY117544 1	Arctocephalus gazella BK010918 1	0,19531548
Conepatus chinga NC042596 1	Tapirus terrestris T358	0,19531696
Vulpes zerda KJ603240 1	Meles leucurus NC039173 4	0,19532739
Urocyon cinereoargenteus NC026723 2	Arctocephalus townsendi NC008420 1	0,19532934
Neofelis nebulosa NC008450 3	Galictis vittata T412 1	0,19533259
Vulpes zerda KJ603240 1	Paradoxurus hermaphroditus NC03959	0,19533744
Speothos venaticus C48 2	Arctocephalus gazella BK010918 1	0,1953401
Ursus maritimus NC003428 31	Nasua nasua NC020647 1	0,19534285
Lycalopex sechurae KT448284 1	Galictis vittata T412 1	0,19534481
Crocuta crocuta NC020670 3	Canis latrans NC008093 7	0,19534974
Vulpes corsac NC023958 1	Galictis vittata T412 1	0,19535218
Ursus maritimus NC003428 31	Speothos venaticus C48 2	0,19535454
Profelis aurata NC028299 1	Odobenus rosmarus NC004029 29	0,19536358
Neofelis nebulosa NC008450 3	Arctocephalus townsendi NC008420 1	0,19537675
Ursus arctos pruinosus MG066703 3	Neofelis nebulosa NC008450 3	0,1953873
Lutrogale perspicillata NC035811 1	Canis anthus NC027956 2	0,19539528
Urocyon littoralis catalinae KP129018	Xenogale naso C07XAR110 1	0,19539633
Vulpes ferrilata NC027935 1	Meles anakuma NC009677 1	0,19539849
Panthera uncia KP202269 1	Bassaricyon neblina SRX1097850 1	0,19541471
Proteles cristata T393 6	Nyctereutes procyonoides NC013700 3	0,19541572
Helogale parvula SRR7637809 1	Galictis vittata T412 1	0,19541924
Bassaricyon neblina SRX1097850 1	Arctictis binturong T605 2	0,19542785
Mephitis mephitis NC020648 1	Galidictis fasciata DM333 1	0,19543239
Galidictis fasciata DM333 1	Cuon alpinus NC013445 3	0,195452
Proteles cristata T393 6	Canis lupus familiaris NC002008 1231	0,19545266
Mellivora capensis T370 1	Homotherium latidens MF871702 3	0,19545577
Mustela sibirica AP017394 11	Tapirus terrestris T358	0,19545828

Ursus maritimus GU573488 Svalbard	Mustela sibirica AP017394 11	0,18052925
Neovison vison NC020641 3	Canis latrans NC008093 7	0,18053061
Nyctereutes procyonoides NC013700 3	Mirounga angustirostris SRR10331586 1	0,18053159
Martes martes T302 3	Civettictis civetta GLC19 1	0,18053167
Ursus thibetanus formosanus NC009331 1	Martes melampus NC009678 1	0,18053298
Leopardus pardalis T262 1	Canis aureus KT448274 1	0,1805333
Catopuma badia NC028300 1	Canis anthus NC027956 2	0,18053375
Mustela nivalis T306 5	Lynx canadensis NC028313 1	0,18053401
Melogale moschata NC020644 1	Felis nigripes NC028309 1	0,18053462
Lynx lynx NC027083 4	Arctodus simus NC011116 1	0,18053558
Martes martes T302 3	Leopardus pardalis NC028315 1	0,18053572
Martes martes T302 3	Felis catus NC001700 2	0,18053626
Leopardus tigrinus NC028317 1	Arctodus simus NC011116 1	0,1805377
Callorhinus ursinus NC008415 1	Acinonyx jubatus NC005212 3	0,18053937
Lycaon pictus NC028427 2	Felis catus NC001700 2	0,18054137
Ursus spelaeus EU327344 13	Mustela sibirica NC020637 6	0,18054295
Ursus spelaeus EU327344 13	Martes americana NC020642 1	0,18054374
Ursus spelaeus NC011112 8	Mustela eversmannii NC028013 1	0,18054464
Genetta abyssinica MG489822 1	Ailurus fulgens styani NC009691 1	0,18054576
Paradoxurus jerdoni MH464793 1	Chrysocyon brachyurus NC024172 1	0,18054636
Ursus arctos GU573486 5	Hemigalus derbyanus MH464791 1	0,18054813
Leopardus wiedii NC028318 1	Canis lupus chanco NC010340 4	0,1805533
Mustela kathiah NC023210 1	Eumetopias jubatus NC004030 10	0,18055372
Suricata suricatta SSM10 1	Halichoerus grypus NC001602 2	0,1805629
Phoca fasciata NC008428 1	Canis lupus chanco NC010340 4	0,18056618
Neovison vison NC020641 3	Canis lupus chanco NC010340 4	0,1805737
Meles meles T303 3	Chrotogale owstoni T607 1	0,18057449
Martes melampus NC009678 1	Conepatus chinga NC042596 1	0,18057747
Mustela putorius NC020638 4	Fossa fossana D350 1	0,18057919
Tremarctos ornatus NC009969 2	Lutra lutra LC050126 1	0,18057984
Mustela frenata NC020640 1	Galerella sanguinea T378 1	0,18058215
Phocarctos hookeri NC008418 1	Martes martes T302 3	0,18058518
Conepatus chinga NC042596 1	Ailurus fulgens NC011124 1	0,1805881
Leopardus jacobita NC028322 1	Enhydra lutris NC009692 1	0,18058926
Paguma larvata PDDS11 2	Meles leucurus NC039173 4	0,18058951
Prionailurus bengalensis NC028301 12	Arctonyx collaris NC020645 1	0,18058962
Leopardus wiedii NC028318 1	Enhydra lutris NC009692 1	0,18058966
Arctotherium sp NC030174 1	Arctocephalus forsteri NC004023 28	0,18058969
Salanoia concolor D378 1	Canis adustus KT448271 1	0,18059063
Mustela altaica NC021751 1	Crossarchus platycephalus C7R66 1	0,18059129
Phoca groenlandica NC008429 54	Urva brachyura KY117547 1	0,18059187
Mustela erminea T305 2	Galerella sanguinea T378 1	0,18059206
Phocarctos hookeri NC008418 1	Martes foina NC020643 1	0,18059227
Puma concolor NC016470 22	Lutra sumatrana NC035810 1	0,18059233
Viverricula indica KX891745 1	Mustela itatsi NC034330 19	0,18059249
Viverra tangalunga MH464792 1	Felis nigripes NC028309 1	0,18059301
Mustela sibirica NC020637 6	Felis margarita NC028308 1	0,18059358
Smilodon populator MF871700 1	Lutra lutra LC050126 1	0,18059433
Viverra zibetha T609 1	Canis latrans NC008093 7	0,18059435
Ursus thibetanus mupinensis NC008753 2	Arctocephalus gazella BK010918 1	0,18059444
Ursus arctos GU573491 207	Meles leucurus NC039173 4	0,18059523
Prionailurus bengalensis CKM45 20	Ailurus fulgens NC011124 1	0,1805955
Mustela putorius NC020638 4	Leopardus geoffroyi NC028320 1	0,18059574
Melogale moschata NC020644 1	Arctotherium sp NC030174 1	0,18059576
Homotherium latidens MF871702 3	Aonyx cinerea NC035814 2	0,18059583
Puma yagouaroundi NC028311 1	Canis latrans NC008093 7	0,18059836
Phoca largha NC008430 1	Galidia elegans D146 1	0,18059891
Ictonyx striatus T299 1	Conepatus chinga NC042596 1	0,18059975
Tremarctos ornatus NC009969 2	Neovison vison NC020641 3	0,18060023
Arctocephalus forsteri NC004023 28	Ailurus fulgens styani NC009691 1	0,1806003
Melogale moschata NC020644 1	Leopardus pardalis NC028315 1	0,18060087
Ursus arctos pruinosus MG066703 3	Mustela erminea T305 2	0,18060151
Puma concolor NC016470 22	Mustela nivalis T306 5	0,180603
Ursus thibetanus thibetanus NC011118 4	Martes melampus NC009678 1	0,18060312
Melogale moschata KP726273 1	Hemigalus derbyanus MH464791 1	0,18060352
Panthera pardus japonensis KJ866876 8	Monachus schauinslandi NC008421 1	0,18060364
Lynx pardinus NC028319 161	Arctodus simus NC011116 1	0,18060368
Helarctos malayanus NC009968 2	Arctocephalus australis MG023139 1	0,18060479
Zalophus californianus NC008416 1	Melogale moschata V0735A 1	0,18060805
Phoca groenlandica NC008429 54	Panthera tigris NC010642 35	0,18060981
Lycaon pictus NC028427 2	Felis margarita NC028308 1	0,18060991
Ursus arctos AP012576 6	Meles meles T303 3	0,18061027
Mustela sibirica AP017394 11	Leopardus colocolo NC028314 1	0,18061085
Viverricula indica KX891745 1	Ursus thibetanus formosanus NC009331 1	0,18061174
Ursus arctos AP012576 6	Mustela erminea T305 2	0,18061657
Phoca vitulina NC001325 1	Arctictis binturong T605 2	0,18061784
Viverra zibetha T609 1	Canis lupus familiaris NC002008 1231	0,18062416
Hemigalus derbyanus MH464791 1	Arctotherium sp NC030174 1	0,18062514
Ursus americanus JX196366 3	Arctocephalus gazella BK010918 1	0,18062694
Ursus americanus JX196366 3	Nandinia binotata NC024567 1	0,18062884
Martes flavigula NC012141 3	Civettictis civetta GLC19 1	0,18063146
Spilogale putorius NC010497 1	Martes foina NC020643 1	0,18063477
Conepatus chinga NC042596 1	Chrotogale owstoni T607 1	0,18063775
Panthera leo NERO 19	Mirounga angustirostris SRR10331586 1	0,18063998
Mustela itatsi NC034330 19	Genetta abyssinica MG489822 1	0,18064241
Ommatophoca rossii AY377287etc 1	Mungos mungo MMC7 1	0,18064263
Mustela itatsi NC034330 19	Arctocephalus townsendi NC008420 1	0,18064559
Enhydra lutris NC009692 1	Arctocephalus pusillus NC008417 1	0,18064661
Martes melampus NC009678 1	Galidictis fasciata DM333 1	0,1806471
Phocarctos hookeri NC008418 1	Melogale moschata V0735A 1	0,18064745
Ommatophoca rossii AY377287etc 1	Galictis vittata T412 1	0,18064781
Crossarchus platycephalus C7R66 1	Arctocephalus australis MG023139 1	0,18064861
Lutrogale perspicillata NC035811 1	Ailurus fulgens styani NC009691 1	0,18065114
Prionailurus rubiginosus NC028304 2	Lutra lutra NC011358 9	0,18065509
Puma yagouaroundi NC028311 1	Meles anakuma NC009677 1	0,18065648
Viverra zibetha T609 1	Meles anakuma NC009677 1	0,1806567
Prionailurus planiceps KY682741 4	Meles meles T303 3	0,18065678
Pusa sibirica NC008432 2	Otocyon megalotis SAF1 2	0,1806588
Mustela eversmannii NC028013 1	Leopardus pardalis T262 1	0,18065985
Leopardus tigrinus NC028317 1	Callorhinus ursinus NC008415 1	0,18066007
Crossarchus platycephalus C7R66 1	Arctodus simus NC011116 1	0,1806602
Prionailurus planiceps NC028312 6	Mustela eversmannii NC028013 1	0,18066034
Puma yagouaroundi NC028311 1	Mustela sibirica NC020637 6	0,18066074

Cynogale bennetti KY117544 1	Arctotherium sp NC030174 1	0,19546062
Urocyon cinereoargenteus NC026723 2	Bdeogale nigripes GLC15 1	0,19546406
Martes foina NC020643 1	Tapirus terrestris T358	0,19546995
Urocyon cinereoargenteus NC026723 2	Cryptoprocta ferox CFC13 1	0,19547217
Nasua nasua NC020647 1	Hyaena hyaena NC020669 1	0,19547674
Ursus arctos GU573486 5	Nasua nasua NC020647 1	0,19548413
Ursus thibetanus laniger MH281753 2	Urocyon littoralis catalinae KP129018 1	0,19548839
Ursus thibetanus thibetanus NC011118	Urocyon littoralis catalinae KP129018 1	0,19548886
Pardofelis marmorata NLN3 2	Odobenus rosmarus NC004029 29	0,19550049
Ursus arctos AP012576 6	Speothos venaticus C48 2	0,1955038
Vulpes zerda KJ603240 1	Spilogale putorius NC010497 1	0,19550826
Mustela sibirica NC020637 6	Tapirus terrestris T358	0,19552593
Spilogale putorius NC010497 1	Panthera onca NC022842 1	0,19552834
Vulpes ferrilata NC027935 1	Arctocephalus townsendi NC008420 1	0,19552898
Lycalopex sechurae KT448284 1	Tapirus terrestris T358	0,19552939
Vulpes vulpes NC008434 5	Ailurus fulgens NC011124 1	0,19553143
Ursus thibetanus mupinensis NC00875	Urocyon cinereoargenteus NC026723 2	0,1955438
Crocuta crocuta NC020670 3	Bassaricyon neblina SRX1097850 1	0,19554627
Nasua nasua NC020647 1	Catopuma temminckii NC027115 41	0,19555008
Otocolobus manul NC028323 1	Nasua nasua NC020647 1	0,19555099
Odobenus rosmarus NC004029 29	Helogale parvula SRR7637809 1	0,19555242
Ursus arctos GU573491 207	Speothos venaticus C48 2	0,19555658
Ursus americanus JX196366 3	Neofelis nebulosa NC008450 3	0,19555812
Odobenus rosmarus NC004029 29	Bassaricyon neblina SRX1097850 1	0,19556439
Melursus ursinus NC009970 2	Crocuta crocuta NC020670 3	0,1955684
Canis lupus familiaris NC002008 1231	Bassaricyon neblina SRX1097850 1	0,19557306
Spilogale putorius NC010497 1	Bdeogale nigripes GLC15 1	0,19557534
Panthera onca NC022842 1	Odobenus rosmarus NC004029 29	0,19558065
Pusa caspica NC008431 1	Mellivora capensis T370 1	0,19559191
Urocyon cinereoargenteus NC026723 2	Urva brachyura KY117547 1	0,19559933
Martes americana NC020642 1	Cynogale bennetti KY117544 1	0,19560127
Lutrogale perspicillata NC035811 1	Chrotogale owstoni T607 1	0,19560295
Speothos venaticus C48 2	Callorhinus ursinus NC008415 1	0,19560575
Urocyon cinereoargenteus NC026723 2	Panthera uncia KP202269 1	0,19561219
Ursus arctos EU497665 29	Nasua nasua NC020647 1	0,19561237
Vulpes zerda KJ603240 1	Mephitis mephitis NC020648 1	0,19562118
Ursus arctos GU573491 207	Panthera tigris NC010642 35	0,19562905
Phocarctos hookeri NC008418 1	Canis lupus familiaris NC002008 1231	0,19563743
Speothos venaticus C48 2	Mephitis mephitis NC020648 1	0,19564231
Suricata suricatta SSM10 1	Spilogale putorius NC010497 1	0,1956438
Odobenus rosmarus NC004029 29	Galictis vittata T412 1	0,19564773
Lutra lutra LC050126 1	Cynogale bennetti KY117544 1	0,1956524
Lycalopex sechurae KT448284 1	Lutrogale perspicillata NC035811 1	0,19565846
Spilogale putorius NC010497 1	Galidictis fasciata DM333 1	0,19566031
Vulpes zerda KJ603240 1	Meles anakuma NC009677 1	0,19566856
Urocyon littoralis catalinae KP129018 1	Ichneumia albicauda T603 1	0,19566901
Ursus arctos isabellinus 18B5 2	Nyctereutes procyonoides NC013700 3	0,19567817
Diplogale hosei MH464790 1	Bassaricyon neblina SRX1097850 1	0,19568186
Phocarctos hookeri NC008418 1	Canis lupus chanco NC010340 4	0,19569287
Otocyon megalotis SAF1 2	Mephitis mephitis NC020648 1	0,19569513
Odobenus rosmarus NC004029 29	Felis margarita NC028308 1	0,19570173
Panthera pardus japonensis KJ866876	Odobenus rosmarus NC004029 29	0,19570427
Ursus arctos AP012576 6	Panthera tigris NC010642 35	0,19570898
Panthera tigris NC010642 35	Melursus ursinus NC009970 2	0,19571075
Spilogale putorius NC010497 1	Pardofelis marmorata NLN3 2	0,19571854
Ursus arctos pruinosus MG066703 3	Urva javanica/auropunctata NC006835	0,19573323
Lutrogale perspicillata NC035811 1	Urva brachyura KY117547 1	0,19573429
Viverra tangalunga MH464792 1	Urocyon cinereoargenteus NC026723 2	0,195738
Phocarctos hookeri NC008418 1	Canis mesomelas KT448280 1	0,19573919
Prionodon linsang ERR2391707 1	Arctocephalus pusillus NC008417 1	0,19573928
Vulpes zerda KJ603240 1	Ursus arctos EU497665 29	0,19574259
Urocyon cinereoargenteus NC026723 2	Poecilogale albinucha T602 1	0,19574596
Mungotictis decemlineata NC027828 1	Ailuropoda melanoleuca NC009492 5	0,19575837
Ursus arctos pruinosus MG066703 3	Speothos venaticus C48 2	0,19575839
Otocyon megalotis SAF1 2	Crocuta crocuta NC020670 3	0,19576047
Urocyon littoralis catalinae KP129018 1	Panthera pardus NC010641 5	0,1957895
Mellivora capensis T370 1	Ailurus fulgens styani NC009691 1	0,19579566
Urocyon littoralis catalinae KP129018 1	Urva javanica T413 1	0,19580112
Vulpes ferrilata NC027935 1	Ailurus fulgens styani NC009691 1	0,19580141
Vulpes lagopus NC026529 3	Bassaricyon neblina SRX1097850 1	0,19580202
Panthera pardus NC010641 5	Mephitis mephitis NC020648 1	0,19580291
Urocyon littoralis catalinae KP129018 1	Prionodon linsang ERR2391707 1	0,19580358
Speothos venaticus C48 2	Arctocephalus forsteri KT693377 17	0,19581014
Ursus arctos pruinosus MG066703 3	Urva semitorquata MH464789 1	0,19581428
Urocyon cinereoargenteus NC026723 2	Ailuropoda melanoleuca NC009492 5	0,19582337
Panthera uncia NC010638 1	Galictis vittata T412 1	0,19583765
Speothos venaticus C48 2	Panthera leo spelaea KX258452 2	0,19583942
Cuon alpinus NC013445 3	Aonyx cinerea NC035814 2	0,1958445
Neofelis nebulosa NC008450 3	Conepatus chinga NC042596 1	0,19584519
Panthera tigris amoyensis NC014770 2	Melursus ursinus NC009970 2	0,19584559
Enhydra lutris NC009692 1	Tapirus terrestris T358	0,1958492
Parahyaena brunnea NC038159 15	Canis mesomelas KT448280 1	0,19586053
Urocyon littoralis catalinae KP129018 1	Bdeogale nigripes GLC15 1	0,19586797
Vulpes corsac NC023958 1	Ailurus fulgens NC011124 1	0,19586836
Monachus schauinslandi NC008421 1	Mellivora capensis T370 1	0,19587912
Urocyon cinereoargenteus NC026723 2	Panthera pardus japonensis KJ866876	0,19588168
Vulpes vulpes NC008434 5	Proteles cristata T393 6	0,19588581
Crocuta crocuta NC020670 3	Canis anthus NC027956 2	0,19588807
Ursus arctos AP012576 6	Nasua nasua NC020647 1	0,19589342
Crocuta crocuta NC020670 3	Canis lupus familiaris NC002008 1231	0,19592503
Cryptoprocta ferox CFC13 1	Canis mesomelas KT448280 1	0,19592734
Mustela eversmannii NC028013 1	Tapirus terrestris T358	0,19592984
Urocyon littoralis catalinae KP129018 1	Helogale parvula SRR7637809 1	0,19593483
Nasua nasua NC020647 1	Helogale parvula SRR7637809 1	0,19593498
Urocyon littoralis catalinae KP129018 1	Urva brachyura KY117547 1	0,19593595
Panthera uncia NC010638 1	Conepatus chinga NC042596 1	0,19593786
Urocyon cinereoargenteus NC026723 2	Ichneumia albicauda T603 1	0,19593839
Speothos venaticus C48 2	Arctocephalus townsendi NC008420 1	0,19594376
Vulpes corsac NC023958 1	Cryptoprocta ferox CFC13 1	0,19594401
Vulpes ferrilata NC027935 1	Cryptoprocta ferox CFC13 1	0,19594418
Vulpes zerda KJ603240 1	Ursus maritimus GU573488 Svalbard	0,19594447
Crocuta crocuta NC020670 3	Tapirus terrestris T358	0,19594617
Pardofelis marmorata NLN3 2	Nasua nasua NC020647 1	0,1959523

Prionailurus bengalensis NC028301 12	Mustela eversmannii NC028013 1	0,18066086
Puma concolor NC016470 22	Potos flavus T414 1	0,18066165
Melogale moschata V0735A 1	Leopardus pardalis T262 1	0,18066181
Viverricula indica NC025296 2	Mustela sibirica NC020637 6	0,18066207
Viverra tangalunga MH464792 1	Leopardus geoffroyi NC028320 1	0,18066323
Martes zibellina NC011579 39	Leopardus pardalis T262 1	0,18066354
Ursus maritimus NC003428 31	Mustela sibirica AP017394 11	0,1806639
Prionailurus planiceps KY682741 4	Canis aureus KT448274 1	0,18066555
Ursus thibetanus mupinensis NC008753 2	Meles anakuma NC009677 1	0,18066612
Ursus arctos isabellinus 18B5 2	Mustela kathiah NC023210 1	0,18066615
Pusa sibirica NC008432 2	Proteles cristata T393 6	0,18066674
Nyctereutes procyonoides NC013700 3	Catopuma badia NC028300 1	0,18066686
Mustela altaica NC021751 1	Leptailurus serval NC028316 1	0,18066816
Melogale moschata NC020644 1	Lynx rufus NC014456 3	0,18066822
Martes martes T302 3	Leopardus jacobita NC028322 1	0,18066894
Phoca groenlandica NC008429 54	Canis aureus KT448274 1	0,18067038
Potos flavus T414 1	Arctictis binturong T605 2	0,18067098
Neovison vison NC020641 3	Leopardus tigrinus NC028317 1	0,18067135
Ursus arctos AP012576 6	Melogale moschata KP726273 1	0,18067135
Prionailurus bengalensis CKM45 20	Mustela altaica NC021751 1	0,18067214
Mustela sibirica NC020637 6	Mephitis mephitis NC020648 1	0,18067263
Prionailurus planiceps KY682741 4	Lycalopex sechurae KT448284 1	0,18067344
Puma concolor NC016470 22	Martes foina NC020643 1	0,18067527
Puma yagouaroundi NC028311 1	Martes foina NC020643 1	0,18067566
Mustela altaica NC021751 1	Mephitis mephitis NC020648 1	0,18067721
Prionailurus rubiginosus NC028304 2	Canis mesomelas KT448280 1	0,18067929
Gulo gulo NC009685 3	Arctocephalus pusillus NC008417 1	0,18067966
Panthera tigris amoyensis NC014770 2	Mirounga angustirostris SRR10331586 1	0,18067983
Hemigalus derbyanus MH464791 1	Canis anthus NC027956 2	0,1806801
Mustela kathiah NC023210 1	Canis lupus chanco NC010340 4	0,18068321
Ursus thibetanus formosanus NC009331 1	Martes foina NC020643 1	0,18068473
Martes foina NC020643 1	Helarctos malayanus NC009968 2	0,18068534
Ursus thibetanus thibetanus NC011118 4	Leopardus pardalis T262 1	0,18068622
Ursus thibetanus thibetanus NC011118 4	Martes foina NC020643 1	0,18068789
Melogale moschata V0735A 1	Eumetopias jubatus NC004030 10	0,18068966
Nandinia binotata NC024567 1	Canis mesomelas KT448280 1	0,18069017
Zalophus californianus NC008416 1	Martes foina NC020643 1	0,18069063
Mustela sibirica NC020637 6	Canis lupus chanco NC010340 4	0,18069336
Lutra lutra LC050126 1	Hemigalus derbyanus MH464791 1	0,18069535
Melogale moschata NC020644 1	Eumetopias jubatus NC004030 10	0,18069538
Puma yagouaroundi NC028311 1	Canis lupus familiaris NC002008 1231	0,18069662
Canis adustus KT448271 1	Ailurus fulgens NC011124 1	0,18069962
Ichneumia albicauda T603 1	Canis adustus KT448271 1	0,180702
Ommatophoca rossii AY377287etc 1	Mephitis mephitis NC020648 1	0,18070577
Melogale moschata NC020644 1	Conepatus chinga NC042596 1	0,18070915
Meles meles T303 3	Arctocephalus townsendi NC008420 1	0,18070923
Mustela itatsi NC034330 19	Canis adustus KT448271 1	0,1807097
Nasua nasua NC020647 1	Mustela nigripes NC024942 1	0,1807106
Neophoca cinerea NC008419 1	Arctonyx collaris NC020645 1	0,18071482
Tremarctos ornatus NC009969 2	Martes americana NC020642 1	0,18071617
Procyon lotor AB462046 3	Canis adustus KT448271 1	0,18071641
Mustela frenata NC020640 1	Atilax paludinosus T606 1	0,18071721
Otaria byronia OTAB 1	Melogale moschata KP726273 1	0,18071926
Ommatophoca rossii AY377287etc 1	Canis lupus familiaris NC002008 1231	0,18071982
Martes pennanti NC020664 16	Arctocephalus forsteri KT693377 17	0,18072072
Otaria byronia OTAB 1	Meles anakuma NC009677 1	0,18072164
Neophoca cinerea NC008419 1	Martes pennanti NC020664 16	0,18072174
Halichoerus grypus NC001602 2	Crocuta crocuta NC020670 3	0,18072231
Leopardus pardalis NC028315 1	Arctonyx collaris NC020645 1	0,18072292
Prionailurus viverrinus NC028305 1	Ailurus fulgens styani NC009691 1	0,18072404
Otaria byronia OTAB 1	Martes pennanti NC020664 16	0,1807256
Suricata suricatta SSM10 1	Phoca fasciata NC008428 1	0,18072639
Mustela putorius NC020638 4	Lynx pardinus NC028319 161	0,18072671
Mustela kathiah NC023210 1	Felis margarita NC028308 1	0,1807273
Mungos mungo/gambianus SRR7704821 1	Canis aureus KT448274 1	0,18072736
Lutra sumatrana NC035810 1	Felis silvestris lybica KP202275 4	0,18072775
Ursus maritimus NC003428 31	Nandinia binotata NC024567 1	0,18072802
Viverra tangalunga MH464792 1	Caracal caracal NC028306 1	0,18072832
Viverra tangalunga MH464792 1	Arctotherium sp NC030174 1	0,18072863
Mustela nigripes NC024942 1	Leptailurus serval NC028316 1	0,18072897
Viverricula indica KX891751 1	Nyctereutes procyonoides NC013700 3	0,18073025
Mustela erminea T305 2	Ichneumia albicauda T603 1	0,18073076
Mustela itatsi NC034330 19	Leopardus geoffroyi NC028320 1	0,18073085
Prionailurus bengalensis CKM45 20	Lutra sumatrana NC035810 1	0,18073155
Pusa sibirica NC008432 2	Chrysocyon brachyurus NC024172 1	0,18073243
Puma concolor NC016470 22	Nyctereutes procyonoides NC013700 3	0,18073259
Leptailurus serval NC028316 1	Canis anthus NC027956 2	0,18073276
Prionailurus bengalensis NC028301 12	Nyctereutes procyonoides NC013700 3	0,18073294
Ursus thibetanus mupinensis NC008753 2	Melogale moschata NC020644 1	0,18073517
Mustela altaica NC021751 1	Lynx lynx NC027083 4	0,18073595
Mustela altaica NC021751 1	Lynx pardinus NC028319 161	0,18073601
Mustela altaica NC021751 1	Felis margarita NC028308 1	0,18073621
Ursus thibetanus mupinensis NC008753 2	Ailurus fulgens styani NC009691 1	0,18073621
Ursus arctos GU573486 5	Mustela sibirica AP017394 11	0,1807376
Mustela nivalis T306 5	Leopardus jacobita NC028322 1	0,18073766
Procyon lotor AB462046 3	Prionailurus bengalensis CKM45 20	0,18073954
Leopardus guigna NC028321 1	Bassariscus sumichrasti SRX1099089 1	0,1807401
Nandinia binotata NC024567 1	Melursus ursinus NC009970 2	0,18074012
Smilodon populator MF871700 1	Lutra sumatrana NC035810 1	0,18074016
Ursus spelaeus EU327344 13	Martes zibellina NC011579 39	0,18074034
Prionailurus viverrinus NC028305 1	Bassariscus sumichrasti SRX1099089 1	0,18074046
Ursus spelaeus NC011112 8	Melogale moschata KP726273 1	0,18074051
Arctictis binturong T605 2	Ailurus fulgens styani NC009691 1	0,18074377
Ursus thibetanus laniger MH281753 2	Fossa fossana D350 1	0,18074414
Mustela sibirica NC020637 6	Leopardus colocolo NC028314 1	0,18074544
Helarctos malayanus NC009968 2	Ailurus fulgens NC011124 1	0,18074645
Ursus thibetanus laniger MH281753 2	Mustela nivalis T306 5	0,18075086
Prionailurus rubiginosus NC028304 2	Chrysocyon brachyurus NC024172 1	0,18075472
Enhydra lutris NC009692 1	Civettictis civetta NC033378 1	0,18075635
Ursus americanus JX196366 3	Callorhinus ursinus NC008415 1	0,18075766
Diplogale hosei MH464790 1	Arctonyx collaris NC020645 1	0,18075974
Paradoxurus hermaphroditus NLNC 1	Canis lupus chanco NC010340 4	0,18076174
Martes melampus NC009678 1	Civettictis civetta NC033378 1	0,18076184

Ursus arctos pruinosus MG066703 3	Urva brachyura KY117547 1	0,19595265
Crocuta crocuta NC020670 3	Ailurus fulgens NC011124 1	0,19596002
Ursus arctos GU573486 5	Speothos venaticus C48 2	0,19596714
Panthera onca KP202264 2	Odobenus rosmarus NC004029 29	0,19597435
Canis aureus KT448274 1	Tapirus terrestris T358	0,19597541
Canis anthus NC027956 2	Tapirus terrestris T358	0,19599886
Otocyon megalotis SAF1 2	Conepatus chinga NC042596 1	0,19600415
Phocarctos hookeri NC008418 1	Canis anthus NC027956 2	0,19600506
Neofelis nebulosa NC008450 3	Chrysocyon brachyurus NC024172 1	0,19600518
Ursus arctos AP012576 6	Neofelis nebulosa NC008450 3	0,19600634
Paradoxurus hermaphroditus NLNC 1	Lutrogale perspicillata NC035811 1	0,19601215
Panthera tigris NC010642 35	Conepatus chinga NC042596 1	0,19601376
Ursus thibetanus mupinensis NC00875	Urocyon littoralis catalinae KP129018	0,19601518
Urocyon littoralis catalinae KP129018	Panthera uncia KP202269 1	0,19601612
Vulpes lagopus NC026529 3	Proteles cristata T393 6	0,19602077
Melursus ursinus NC009970 2	Urva brachyura KY117547 1	0,19602674
Odobenus rosmarus NC004029 29	Caracal caracal NC028306 1	0,19603758
Neofelis nebulosa NC008450 3	Lontra canadensis SRR10409165 1	0,1960507
Procyon lotor AB462046 3	Tapirus terrestris T358	0,19606584
Vulpes zerda KJ603240 1	Lutrogale perspicillata NC035811 1	0,19606914
Mustela altaica NC021751 1	Tapirus terrestris T358	0,19607247
Martes zibellina NC011579 39	Cynogale bennetti KY117544 1	0,19607273
Vulpes ferrilata NC027935 1	Poecilogale albinucha T602 1	0,19607803
Vulpes corsac NC023958 1	Proteles cristata T393 6	0,19608815
Proteles cristata T393 6	Canis anthus NC027956 2	0,19608973
Vulpes lagopus NC026529 3	Galictis vittata T412 1	0,19609261
Ursus maritimus NC003428 31	Crocuta crocuta NC020670 3	0,19609641
Ursus maritimus GU573488 Svalbard	Crocuta crocuta NC020670 3	0,19609644
Neofelis nebulosa NC008450 3	Arctotherium sp NC030174 1	0,1961233
Neophoca cinerea NC008419 1	Cuon alpinus NC013445 3	0,196126
Ursus arctos GU573491 207	Neofelis nebulosa NC008450 3	0,19612801
Spilogale putorius NC010497 1	Panthera tigris amoyensis NC014770 2	0,19613038
Nasua nasua NC020647 1	Chrotogale owstoni T607 1	0,19613396
Urocyon cinereoargenteus NC026723 2	Suricata suricatta SSM10 1	0,19613715
Proteles cristata T393 6	Poecilogale albinucha T602 1	0,19617429
Neofelis nebulosa NC008450 3	Arctocephalus forsteri NC004023 28	0,19617887
Panthera leo NERO 19	Mephitis mephitis NC020648 1	0,19619755
Urocyon littoralis catalinae KP129018	Suricata suricatta SSM10 1	0,19620445
Vulpes ferrilata NC027935 1	Procyon lotor AB462049 4	0,19620857
Urocyon cinereoargenteus NC026723 2	Procyon lotor AB462049 4	0,19621091
Urocyon littoralis catalinae KP129018	Cryptoprocta ferox CFC13 1	0,19621284
Vulpes zerda KJ603240 1	Ursus maritimus NC003428 31	0,19621386
Chrysocyon brachyurus NC024172 1	Tapirus terrestris T358	0,1962211
Suricata suricatta SSM10 1	Mephitis mephitis NC020648 1	0,19622627
Spilogale putorius NC010497 1	Eupleres goudotii D128 1	0,19623081
Cryptoprocta ferox CFC13 1	Ailuropoda melanoleuca NC009492 5	0,19623414
Canis lupus chanco NC010340 4	Tapirus terrestris T358	0,19623442
Panthera onca NC022842 1	Bassaricyon neblina SRX1097850 1	0,19623488
Odobenus rosmarus NC004029 29	Felis catus NC001700 2	0,19624132
Spilogale putorius NC010497 1	Panthera tigris NC010642 35	0,19626506
Spilogale putorius NC010497 1	Panthera leo spelaea KX258452 2	0,19627117
Ursus arctos pruinosus MG066703 3	Cynogale bennetti KY117544 1	0,19627377
Canis anthus NC027956 2	Bassaricyon neblina SRX1097850 1	0,19627501
Ursus arctos GU573491 207	Nyctereutes procyonoides NC013700 3	0,19628398
Vulpes zerda KJ603240 1	Ursus arctos GU573486 5	0,1962872
Phocarctos hookeri NC008418 1	Chrysocyon brachyurus NC024172 1	0,19629142
Speothos venaticus C48 2	Otaria byronia OTAB 1	0,1962938
Ursus thibetanus formosanus NC00933	Urocyon cinereoargenteus NC026723 2	0,19629404
Odobenus rosmarus NC004029 29	Galidictis fasciata DM333 1	0,19630148
Cuon alpinus NC013445 3	Arctocephalus townsendi NC008420 1	0,19631958
Urocyon littoralis catalinae KP129018	Spilogale putorius NC010497 1	0,19632052
Urocyon cinereoargenteus NC026723 2	Spilogale putorius NC010497 1	0,1963207
Speothos venaticus C48 2	Arctocephalus australis MG023139 1	0,19634886
Ursus thibetanus thibetanus NC01111	Tapirus terrestris T358	0,19635724
Ursus arctos GU573486 5	Nyctereutes procyonoides NC013700 3	0,1963574
Vulpes lagopus NC026529 3	Crocuta crocuta NC020670 3	0,19635801
Odobenus rosmarus NC004029 29	Mungos mungo/gambianus SRR77048	0,19636051
Urocyon littoralis catalinae KP129018	Panthera tigris NC010642 35	0,19636075
Proteles cristata T393 6	Lycaon pictus NC028427 2	0,19636697
Mellivora capensis T370 1	Ailurus fulgens NC011124 1	0,19640163
Speothos venaticus C48 2	Neofelis nebulosa NC008450 3	0,19640177
Urocyon cinereoargenteus NC026723 2	Helogale parvula SRR7637809 1	0,19640619
Vulpes ferrilata NC027935 1	Cynogale bennetti KY117544 1	0,19640768
Mirounga angustirostris SRR10331586	Mellivora capensis T370 1	0,19641212
Nasua nasua NC020647 1	Civettictis civetta GLC19 1	0,19641922
Urocyon cinereoargenteus NC026723 2	Panthera tigris NC010642 35	0,19642805
Proteles cristata T393 6	Tapirus terrestris T358	0,19643165
Ursus arctos GU573486 5	Crocuta crocuta NC020670 3	0,19643918
Monachus monachus NC044972 5	Mellivora capensis T370 1	0,1964428
Neofelis nebulosa NC008450 3	Arctocephalus gazella BK010918 1	0,19645669
Neophoca cinerea NC008419 1	Neofelis nebulosa NC008450 3	0,19645865
Neophoca cinerea NC008419 1	Cynogale bennetti KY117544 1	0,19647065
Nyctereutes procyonoides NC013700 3	Bassaricyon neblina SRX1097850 1	0,1964757
Urocyon littoralis catalinae KP129018	Procyon lotor AB462049 4	0,1964801
Urocyon cinereoargenteus NC026723 2	Chrotogale owstoni T607 1	0,19648141
Panthera tigris NC010642 35	Bassaricyon neblina SRX1097850 1	0,19650078
Panthera tigris amoyensis NC014770 2	Mephitis mephitis NC020648 1	0,19651535
Mephitis mephitis NC020648 1	Crocuta crocuta NC020670 3	0,19652016
Panthera leo spelaea KX258452 2	Mephitis mephitis NC020648 1	0,19652078
Spilogale putorius NC010497 1	Panthera pardus japonensis KJ866876	0,19652544
Canis latrans NC008093 7	Tapirus terrestris T358	0,19653367
Vulpes corsac NC023958 1	Cynogale bennetti KY117544 1	0,19654125
Panthera pardus NC010641 5	Odobenus rosmarus NC004029 29	0,19655074
Urocyon littoralis catalinae KP129018	Poecilogale albinucha T602 1	0,19655389
Ursus arctos GU573491 207	Nasua nasua NC020647 1	0,19655502
Ursus arctos pruinosus MG066703 3	Nasua nasua NC020647 1	0,19655523
Urocyon cinereoargenteus NC026723 2	Parahyaena brunnea NC038159 15	0,19655629
Melursus ursinus NC009970 2	Cynogale bennetti KY117544 1	0,19656056
Urocyon cinereoargenteus NC026723 2	Ictonyx striatus T299 1	0,1965639
Urocyon littoralis catalinae KP129018	Ailuropoda melanoleuca NC009492 5	0,19656409
Viverricula indica KX891745 1	Odobenus rosmarus NC004029 29	0,19656438
Vulpes ferrilata NC027935 1	Galictis vittata T412 1	0,1965649
Bassaricyon neblina SRX1097850 1	Ailuropoda melanoleuca NC009492 5	0,19656914

Phocarctos hookeri NC008418 1	Martes flavigula NC012141 3	0,18076529
Martes flavigula NC012141 3	Leopardus wiedii NC028318 1	0,18077214
Phoca largha NC008430 1	Neofelis nebulosa NC008450 3	0,18077573
Lutra lutra NC011358 9	Arctocephalus forsteri KT693377 17	0,18077663
Martes melampus NC009678 1	Arctocephalus australis MG023139 1	0,18077812
Phocarctos hookeri NC008418 1	Lutra sumatrana NC035810 1	0,18077935
Ursus thibetanus laniger MH281753 2	Homotherium latidens MF871702 3	0,18078041
Mungos mungo MMC7 1	Callorhinus ursinus NC008415 1	0,18078139
Vulpes vulpes NC008434 5	Pusa caspica NC008431 1	0,18078329
Prionailurus planiceps KY682741 4	Lutra lutra LC050126 1	0,18079026
Mustela kathiah NC023210 1	Canis aureus KT448274 1	0,18079048
Viverra zibetha T609 1	Martes americana NC020642 1	0,18079142
Paradoxurus hermaphroditus NLNC 1	Meles meles T303 3	0,18079273
Meles meles T303 3	Canis adustus KT448271 1	0,18079303
Civettictis civetta NC033378 1	Canis lupus familiaris NC002008 1231	0,18079366
Meles leucurus NC039173 4	Genetta genetta T297 1	0,18079408
Martes melampus NC009678 1	Felis catus NC001700 2	0,18079434
Prionailurus viverrinus NC028305 1	Mustela kathiah NC023210 1	0,18079461
Mustela itatsi NC034330 19	Leopardus jacobita NC028322 1	0,18079501
Mustela putorius NC020638 4	Leopardus jacobita NC028322 1	0,18079503
Viverra tangalunga MH464792 1	Canis aureus KT448274 1	0,18079512
Mustela kathiah NC023210 1	Leptailurus serval NC028316 1	0,18079534
Prionailurus bengalensis NC028301 12	Mustela itatsi NC034330 19	0,18079564
Otocolobus manul NC028323 1	Mustela eversmannii NC028013 1	0,18079621
Puma concolor NC016470 22	Martes americana NC020642 1	0,18079707
Mustela sibirica NC020637 6	Catopuma temminckii NC027115 41	0,18079737
Monachus schauinslandi NC008421 1	Canis anthus NC027956 2	0,18079825
Otaria byronia OTAB 1	Ailurus fulgens styani NC009691 1	0,18079855
Vulpes ferrilata NC027935 1	Hydrurga leptonyx NC008425 1	0,18079981
Ursus spelaeus NC011112 8	Eumetopias jubatus NC004030 10	0,18080123
Ursus thibetanus mupinensis NC008753 2	Ailurus fulgens NC011124 1	0,18080334
Martes martes T302 3	Leopardus wiedii NC028318 1	0,18080388
Martes melampus NC009678 1	Arctocephalus forsteri NC004023 28	0,18080433
Mirounga leonina NC008422 1	Crocuta crocuta NC020670 3	0,18080468
Leopardus pardalis NC028315 1	Aonyx cinerea NC035814 2	0,18080471
Ursus spelaeus NC011112 8	Martes melampus NC009678 1	0,18080568
Felis margarita NC028308 1	Arctodus simus NC011116 1	0,18080634
Phoca vitulina NC001325 1	Lycaon pictus NC028427 2	0,18080765
Ursus spelaeus EU327344 13	Enhydra lutris NC009692 1	0,18080805
Lutra lutra LC050126 1	Canis adustus KT448271 1	0,18080869
Mustela frenata NC020640 1	Ailuropoda melanoleuca NC009492 5	0,18080878
Ursus thibetanus mupinensis NC008753 2	Leopardus pardalis T262 1	0,1808092
Viverricula indica KX891751 1	Lycaon pictus NC028427 2	0,18081126
Zalophus californianus NC008416 1	Mustela eversmannii NC028013 1	0,18081184
Mustela eversmannii NC028013 1	Leopardus colocolo NC028314 1	0,18081285
Gulo gulo NC009685 3	Arctocephalus australis MG023139 1	0,1808136
Helarctos malayanus NC009968 2	Ailurus fulgens styani NC009691 1	0,180814
Monachus schauinslandi NC008421 1	Chrysocyon brachyurus NC024172 1	0,1808144
Parahyaena brunnea NC038159 15	Monachus schauinslandi NC008421 1	0,1808154
Ursus thibetanus laniger MH281753 2	Arctocephalus pusillus NC008417 1	0,18081588
Zalophus wollebaeki SRR4431565 1	Ursus thibetanus formosanus NC009331 1	0,18081678
Viverricula indica NC025296 2	Chrysocyon brachyurus NC024172 1	0,18081801
Viverricula indica KX891751 1	Helarctos malayanus NC009968 2	0,18081814
Mustela altaica NC021751 1	Leopardus colocolo NC028314 1	0,18082114
Ommatophoca rossii AY377287etc 1	Urva javanica/auropunctata NC006835 1	0,18082437
Paradoxurus hermaphroditus NLNC 1	Canis lupus familiaris NC002008 1231	0,18082689
Mustela altaica NC021751 1	Eumetopias jubatus NC004030 10	0,18083443
Spilogale putorius NC010497 1	Paradoxurus hermaphroditus NC039591 1	0,18083842
Canis lupus chanco NC010340 4	Arctictis binturong T605 2	0,18084508
Canis lupus familiaris NC002008 1231	Acinonyx jubatus NC005212 3	0,18084592
Arctocephalus australis MG023139 1	Ailurus fulgens NC011124 1	0,1808472
Meles meles T303 3	Urva semitorquata MH464789 1	0,18084756
Tremarctos ornatus NC009969 2	Enhydra lutris NC009692 1	0,18084794
Ursus arctos pruinosus MG066703 3	Homotherium latidens MF871702 3	0,18084982
Potos flavus T414 1	Arctocephalus australis MG023139 1	0,18085162
Potos flavus T414 1	Arctocephalus gazella BK010918 1	0,180852
Eupleres goudotii D128 1	Canis adustus KT448271 1	0,18085337
Martes pennanti NC020664 16	Fossa fossana D350 1	0,18085423
Viverra tangalunga MH464792 1	Arctocephalus pusillus NC008417 1	0,1808544
Leptailurus serval NC028316 1	Enhydra lutris NC009692 1	0,18085715
Meles leucurus NC039173 4	Leopardus pardalis T262 1	0,18085717
Martes martes T302 3	Galidictis fasciata DM333 1	0,1808579
Ursus thibetanus thibetanus NC011118 4	Gulo gulo NC009685 3	0,18085805
Viverricula indica KX891751 1	Meles anakuma NC009677 1	0,18085817
Lynx rufus NC014456 3	Lutra lutra LC050126 1	0,18085822
Meles meles T303 3	Leopardus jacobita NC028322 1	0,18085833
Meles meles T303 3	Felis catus NC001700 2	0,18085842
Pusa sibirica NC008432 2	Paradoxurus hermaphroditus NLNC 1	0,18085971
Procyon lotor AB462046 3	Arctocephalus pusillus NC008417 1	0,18086042
Zalophus wollebaeki SRR4431565 1	Meles anakuma NC009677 1	0,18086091
Procyon lotor AB462046 3	Ailurus fulgens NC011124 1	0,18086107
Prionodon linsang ERR2391707 1	Lobodon carcinophaga NC008423 1	0,18086126
Mustela kathiah NC023210 1	Felis catus NC001700 2	0,18086162
Prionailurus planiceps KY682741 4	Mustela sibirica AP017394 11	0,1808624
Mustela sibirica AP017394 11	Felis margarita NC028308 1	0,18086287
Vulpes vulpes NC008434 5	Lynx rufus NC014456 3	0,18086368
Potos flavus T414 1	Catopuma badia NC028300 1	0,18086423
Pusa sibirica NC008432 2	Diplogale hosei MH464790 1	0,18086471
Pusa hispida NC 008433 1	Nasua nasua NC020647 1	0,18086512
Paradoxurus hermaphroditus NLNC 1	Ailurus fulgens styani NC009691 1	0,18086545
Ommatophoca rossii AY377287etc 1	Lutrogale perspicillata NC035811 1	0,18086645
Pusa caspica NC008431 1	Chrysocyon brachyurus NC024172 1	0,18086746
Mustela altaica NC021751 1	Civettictis civetta GLC19 1	0,18086831
Monachus schauinslandi NC008421 1	Galidia elegans D146 1	0,1808694
Martes pennanti NC020664 16	Lynx canadensis NC028313 1	0,1808697
Mustela nivalis T306 5	Caracal caracal NC028306 1	0,18087329
Nandinia binotata NC024567 1	Galictis vittata T412 1	0,18087403
Potos flavus T414 1	Melursus ursinus NC009970 2	0,18087424
Martes martes T302 3	Felis chaus NC028307 1	0,18087429
Caracal caracal NC028306 1	Canis aureus KT448274 1	0,18087447
Zalophus californianus NC008416 1	Meles anakuma NC009677 1	0,180876
Viverricula indica NC025296 2	Ursus thibetanus mupinensis NC008753 2	0,18087608
Ursus thibetanus mupinensis NC008753 2	Martes foina NC020643 1	0,18087672

Panthera uncia KP202269 1	Mephitis mephitis NC020648 1	0,19657069
Nasua nasua NC020647 1	Urva semitorquata MH464789 1	0,19660759
Phocarctos hookeri NC008418 1	Lycalopex sechurae KT448284 1	0,19660988
Urocyon cinereoargenteus NC026723 2	Procyon lotor AB462046 3	0,1966154
Ursus maritimus GU573488 Svalbard	Nyctereutes procyonoides NC013700 3	0,19662052
Ursus maritimus NC003428 31	Nyctereutes procyonoides NC013700 3	0,19662054
Urocyon littoralis catalinae KP129018	Panthera pardus japonensis KJ866876	0,1966223
Canis lupus chanco NC010340 4	Bassaricyon neblina SRX1097850 1	0,1966546
Panthera leo NERO 19	Nasua nasua NC020647 1	0,19665949
Vulpes lagopus NC026529 3	Ailurus fulgens styani NC009691 1	0,19667605
Vulpes zerda KJ603240 1	Ursus arctos GU573491 207	0,19668506
Urocyon littoralis catalinae KP129018	Genetta genetta T297 1	0,1966892
Proteles cristata T393 6	Phocarctos hookeri NC008418 1	0,19669443
Nasua nasua NC020647 1	Ailuropoda melanoleuca NC009492 5	0,19670241
Martes flavigula NC012141 3	Tapirus terrestris T358	0,19670674
Lutra sumatrana NC035810 1	Tapirus terrestris T358	0,19673057
Urocyon cinereoargenteus NC026723 2	Ailurus fulgens styani NC009691 1	0,19674727
Vulpes zerda KJ603240 1	Ursus arctos pruinosus MG066703 3	0,19675243
Paradoxurus jerdoni MH464793 1	Lutrogale perspicillata NC035811 1	0,19675274
Vulpes zerda KJ603240 1	Ursus arctos AP012576 6	0,19676422
Ursus thibetanus formosanus NC00933	Urocyon littoralis catalinae KP129018	0,19676547
Speothos venaticus C48 2	Panthera uncia NC010638 1	0,19676779
Lutra lutra LC050126 1	Tapirus terrestris T358	0,19679192
Viverra tangalunga MH464792 1	Urocyon littoralis catalinae KP129018	0,1968153
Urocyon littoralis catalinae KP129018	Chrotogale owstoni T607 1	0,19681802
Vulpes vulpes NC008434 5	Crocuta crocuta NC020670 3	0,19682959
Odobenus rosmarus NC004029 29	Urva semitorquata MH464789 1	0,19683129
Vulpes vulpes NC008434 5	Galictis vittata T412 1	0,19683363
Urocyon cinereoargenteus NC026723 2	Diplogale hosei MH464790 1	0,19683436
Panthera tigris amoyensis NC014770 2	Bassaricyon neblina SRX1097850 1	0,19683778
Nasua nasua NC020647 1	Canis lupus chanco NC010340 4	0,19685505
Spilogale putorius NC010497 1	Mungotictis decemlineata NC027828	0,19687098
Urocyon cinereoargenteus NC026723 2	Cynictis penicillata T375 1	0,19687849
Urocyon littoralis catalinae KP129018	Procyon lotor AB462046 3	0,1968846
Panthera uncia KP202269 1	Nasua nasua NC020647 1	0,19689435
Paradoxurus jerdoni MH464793 1	Odobenus rosmarus NC004029 29	0,19689758
Proteles cristata T393 6	Chrysocyon brachyurus NC024172 1	0,19692457
Spilogale putorius NC010497 1	Panthera uncia NC010638 1	0,19692986
Phocarctos hookeri NC008418 1	Cynogale bennetti KY117544 1	0,19694047
Prionodon linsang ERR2391707 1	Lutrogale perspicillata NC035811 1	0,19694546
Poecilogale albinucha T602 1	Tapirus terrestris T358	0,19694765
Lycaon pictus NC028427 2	Tapirus terrestris T358	0,19695424
Ursus thibetanus laniger MH281753 2	Tapirus terrestris T358	0,1969641
Lutrogale perspicillata NC035811 1	Canis mesomelas KT448280 1	0,1969644
Urocyon littoralis catalinae KP129018	Ictonyx striatus T299 1	0,196968
Spilogale putorius NC010497 1	Panthera pardus NC010641 5	0,19696951
Mephitis mephitis NC020648 1	Galidia elegans D146 1	0,19698055
Nasua nasua NC020647 1	Canis aureus KT448274 1	0,19699159
Spilogale putorius NC010497 1	Hyaena hyaena NC020669 1	0,19699523
Cuon alpinus NC013445 3	Cryptoprocta ferox CFC13 1	0,19700569
Prionodon linsang ERR2391707 1	Arctocephalus gazella BK010918 1	0,19700854
Neofelis nebulosa NC008450 3	Ailuropoda melanoleuca NC009492 5	0,19701181
Vulpes ferrilata NC027935 1	Ailurus fulgens NC011124 1	0,19701315
Nasua nasua NC020647 1	Galidictis fasciata DM333 1	0,19701558
Urocyon cinereoargenteus NC026723 2	Genetta genetta T297 1	0,19702588
Proteles cristata T393 6	Ailurus fulgens styani NC009691 1	0,19703369
Salanoia concolor D378 1	Odobenus rosmarus NC004029 29	0,19704197
Canis mesomelas KT448280 1	Tapirus terrestris T358	0,19708727
Urocyon littoralis catalinae KP129018	Parahyaena brunnea NC038159 15	0,19709481
Panthera pardus japonensis KJ866876	Nasua nasua NC020647 1	0,19709706
Ursus spelaeus NC011112 8	Urocyon cinereoargenteus NC026723 2	0,19710484
Ursus arctos AP012576 6	Nyctereutes procyonoides NC013700 3	0,19710489
Ursus spelaeus NC011112 8	Urocyon littoralis catalinae KP129018	0,19710495
Gulo gulo NC009685 3	Tapirus terrestris T358	0,19710852
Nasua nasua NC020647 1	Canis lupus familiaris NC002008 1231	0,19712012
Cynogale bennetti KY117544 1	Arctocephalus townsendi NC008420 1	0,1971321
Lutra sumatrana NC035810 1	Cynogale bennetti KY117544 1	0,19713295
Vulpes vulpes NC008434 5	Bassaricyon neblina SRX1097850 1	0,19714858
Urocyon littoralis catalinae KP129018	Diplogale hosei MH464790 1	0,19715098
Vulpes zerda KJ603240 1	Poecilogale albinucha T602 1	0,19715714
Ursus arctos EU497665 29	Crocuta crocuta NC020670 3	0,19717385
Odobenus rosmarus NC004029 29	Felis nigripes NC028309 1	0,19718367
Panthera tigris NC010642 35	Mephitis mephitis NC020648 1	0,19718887
Spilogale putorius NC010497 1	Odobenus rosmarus NC004029 29	0,19720221
Ursus americanus JX196366 3	Crocuta crocuta NC020670 3	0,19720772
Ursus arctos EU497665 29	Nyctereutes procyonoides NC013700 3	0,19722661
Viverricula indica KX891745 1	Nasua nasua NC020647 1	0,197227
Speothos venaticus C48 2	Aonyx cinerea NC035814 2	0,19722724
Nasua nasua NC020647 1	Genetta genetta T297 1	0,19722949
Paradoxurus hermaphroditus NLNC 1	Bassaricyon neblina SRX1097850 1	0,19723059
Urocyon littoralis catalinae KP129018	Panthera tigris amoyensis NC014770 2	0,19723618
Ursus spelaeus EU327344 13	Urocyon cinereoargenteus NC026723 2	0,19723988
Urocyon littoralis catalinae KP129018	Aonyx cinerea NC035814 2	0,197282
Urocyon cinereoargenteus NC026723 2	Aonyx cinerea NC035814 2	0,19728205
Nasua nasua NC020647 1	Bdeogale nigripes GLC15 1	0,19728254
Mustela nivalis T306 5	Tapirus terrestris T358	0,1972882
Paguma larvata PDDS11 2	Nasua nasua NC020647 1	0,1972954
Urocyon cinereoargenteus NC026723 2	Panthera tigris amoyensis NC014770 2	0,19730346
Odobenus rosmarus NC004029 29	Nasua nasua NC020647 1	0,19731186
Odobenus rosmarus NC004029 29	Catopuma temminckii NC027115 41	0,1973193
Odobenus rosmarus NC004029 29	Ictonyx striatus T299 1	0,19731967
Spilogale putorius NC010497 1	Parahyaena brunnea NC038159 15	0,19733937
Vulpes lagopus NC026529 3	Ailurus fulgens NC011124 1	0,19734918
Urocyon littoralis catalinae KP129018	Ailurus fulgens styani NC009691 1	0,19735326
Mellivora capensis T370 1	Leopardus pardalis NC028315 1	0,19736178
Ursus thibetanus formosanus NC00933	Tapirus terrestris T358	0,19736752
Vulpes zerda KJ603240 1	Galictis vittata T412 1	0,19737191
Ursus spelaeus EU327344 13	Urocyon littoralis catalinae KP129018	0,19737464
Panthera onca NC022842 1	Nasua nasua NC020647 1	0,19737774
Panthera leo spelaea KX258452 2	Nasua nasua NC020647 1	0,19738369
Prionodon linsang ERR2391707 1	Arctocephalus forsteri KT693377 17	0,19741161
Vulpes corsac NC023958 1	Lutrogale perspicillata NC035811 1	0,19741505
Speothos venaticus C48 2	Arctocephalus forsteri NC004023 28	0,19742042

Neophoca cinerea NC008419 1	Melursus ursinus NC009970 2	0,18087766
Lycalopex sechurae KT448284 1	Catopuma badia NC028300 1	0,18087971
Ursus spelaeus NC011112 8	Martes americana NC020642 1	0,1808801
Spilogale putorius NC010497 1	Enhydra lutris NC009692 1	0,18088068
Ursus thibetanus thibetanus NC011118 4	Arctocephalus pusillus NC008417 1	0,18088083
Ursus thibetanus formosanus NC009331 1	Martes martes T302 3	0,18088163
Lycaon pictus NC028427 2	Leptailurus serval NC028316 1	0,18088204
Phocarctos hookeri NC008418 1	Gulo gulo NC009685 3	0,18088204
Phoca groenlandica NC008429 54	Panthera onca NC022842 1	0,18088213
Phoca fasciata NC008428 1	Panthera leo spelaea KX258452 2	0,1808846
Vulpes lagopus NC026529 3	Monachus monachus NC044972 5	0,18088955
Martes flavigula NC012141 3	Canis latrans NC008093 7	0,1808905
Zalophus californianus NC008416 1	Mustela altaica NC021751 1	0,18089154
Genetta servalina NC024568 2	Canis lupus familiaris NC002008 1231	0,18089376
Mungos mungo/gambianus SRR7704821 1	Eumetopias jubatus NC004030 10	0,180894
Paguma larvata PDDS11 2	Canis lupus chanco NC010340 4	0,18089415
Panthera pardus japonensis KJ866876 8	Monachus monachus NC044972 5	0,18089569
Helarctos malayanus NC009968 2	Arctocephalus forsteri NC004023 28	0,18089776
Vulpes lagopus NC026529 3	Homotherium latidens MF871702 3	0,18090314
Phoca vitulina NC001325 1	Canis lupus chanco NC010340 4	0,18090597
Martes flavigula NC012141 3	Arctotherium sp NC030174 1	0,18090756
Otocolobus manul NC028323 1	Canis lupus chanco NC010340 4	0,1809113
Ursus arctos GU573491 207	Ommatophoca rossii AY377287etc 1	0,18091135
Enhydra lutris NC009692 1	Arctocephalus gazella BK010918 1	0,18091348
Ommatophoca rossii AY377287etc 1	Arctictis binturong T605 2	0,18091431
Mustela eversmannii NC028013 1	Crossarchus platycephalus C7R66 1	0,18091509
Melogale moschata KP726273 1	Fossa fossana D350 1	0,18091519
Tremarctos ornatus NC009969 2	Melogale moschata V0735A 1	0,1809164
Monachus schauinslandi NC008421 1	Cynogale bennetti KY117544 1	0,18091692
Halichoerus grypus NC001602 2	Chrysocyon brachyurus NC024172 1	0,1809171
Viverricula indica KX891745 1	Enhydra lutris NC009692 1	0,18091783
Melogale moschata NC020644 1	Callorhinus ursinus NC008415 1	0,18091957
Mustela sibirica NC020637 6	Arctocephalus pusillus NC008417 1	0,18092082
Martes americana NC020642 1	Conepatus chinga NC042596 1	0,18092114
Fossa fossana D350 1	Aonyx cinerea NC035814 2	0,18092136
Paradoxurus hermaphroditus NC039591 1	Mustela frenata NC020640 1	0,18092138
Martes pennanti NC020664 16	Arctocephalus pusillus NC008417 1	0,18092477
Meles meles T303 3	Leopardus pardalis T262 1	0,18092504
Paradoxurus hermaphroditus NLNC 1	Meles anakuma NC009677 1	0,1809254
Meles anakuma NC009677 1	Lynx pardinus NC028319 161	0,18092546
Panthera pardus japonensis KJ866876 8	Mirounga leonina NC008422 1	0,18092659
Leptailurus serval NC028316 1	Callorhinus ursinus NC008415 1	0,18092729
Leopardus guigna NC028321 1	Enhydra lutris NC009692 1	0,18092752
Mustela putorius NC020638 4	Lynx canadensis NC028313 1	0,1809282
Pusa caspica NC008431 1	Otocyon megalotis SAF1 2	0,1809282
Prionodon pardicolor NC024569 2	Cystophora cristata NC008427 1	0,18092829
Mustela itatsi NC034330 19	Lynx canadensis NC028313 1	0,18092838
Mustela putorius NC020638 4	Canis aureus KT448274 1	0,18092841
Otocolobus manul NC028323 1	Martes melampus NC009678 1	0,18092875
Martes melampus NC009678 1	Felis nigripes NC028309 1	0,18092877
Mustela eversmannii NC028013 1	Lynx pardinus NC028319 161	0,18092881
Viverra tangalunga MH464792 1	Prionailurus planiceps NC028312 6	0,18092927
Mustela sibirica AP017394 11	Leopardus jacobita NC028322 1	0,18092953
Puma concolor NC016470 22	Mustela sibirica NC020637 6	0,18092966
Mustela nigripes NC024942 1	Genetta servalina NC024568 2	0,18092974
Prionodon pardicolor NC024569 2	Meles leucurus NC039173 4	0,18092987
Ursus arctos GU573491 207	Nandinia binotata NC024567 1	0,18092996
Melogale moschata KP726273 1	Lynx rufus NC014456 3	0,18092997
Prionailurus bengalensis CKM45 20	Meles leucurus NC039173 4	0,18093015
Melogale moschata V0735A 1	Lynx rufus NC014456 3	0,1809302
Vulpes vulpes NC008434 5	Prionailurus rubiginosus NC028304 2	0,18093175
Mustela sibirica AP017394 11	Catopuma temminckii NC027115 41	0,18093212
Martes americana NC020642 1	Leopardus pardalis T262 1	0,18093246
Mustela kathiah NC023210 1	Catopuma badia NC028300 1	0,18093279
Taxidea taxus NC020646 1	Ichneumia albicauda T603 1	0,18093315
Otocolobus manul NC028323 1	Canis latrans NC008093 7	0,18093492
Phoca groenlandica NC008429 54	Otocyon megalotis SAF1 2	0,18093552
Homotherium latidens MF871702 3	Eumetopias jubatus NC004030 10	0,18093589
Mustela altaica NC021751 1	Lynx canadensis NC028313 1	0,18093795
Viverricula indica NC025296 2	Poecilogale albinucha T602 1	0,18093975
Ursus arctos GU573486 5	Mustela kathiah NC023210 1	0,18094179
Mustela nivalis T306 5	Catopuma temminckii NC027115 41	0,18094194
Lutra lutra LC050126 1	Leopardus colocolo NC028314 1	0,18094346
Martes foina NC020643 1	Catopuma badia NC028300 1	0,18094438
Mustela sibirica NC020637 6	Acinonyx jubatus NC005212 3	0,18094534
Mustela sibirica AP017394 11	Acinonyx jubatus NC005212 3	0,18094535
Monachus schauinslandi NC008421 1	Hyaena hyaena NC020669 1	0,18094592
Ursus thibetanus mupinensis NC008753 2	Leopardus geoffroyi NC028320 1	0,18094794
Ursus thibetanus thibetanus NC011118 4	Ailurus fulgens NC011124 1	0,18094817
Viverricula indica KX891751 1	Ursus thibetanus formosanus NC009331 1	0,18094826
Gulo gulo NC009685 3	Arctocephalus gazella BK010918 1	0,18094912
Viverricula indica NC025296 2	Ursus thibetanus thibetanus NC011118 4	0,18095371
Poecilogale albinucha T602 1	Hemigalus derbyanus MH464791 1	0,18095542
Vulpes corsac NC023958 1	Homotherium latidens MF871702 3	0,1809583
Hemigalus derbyanus MH464791 1	Gulo gulo NC009685 3	0,18095863
Paguma larvata PDDS11 2	Canis lupus familiaris NC002008 1231	0,18096107
Spilogale putorius NC010497 1	Nandinia binotata NC024567 1	0,18096355
Vulpes vulpes NC008434 5	Halichoerus grypus NC001602 2	0,18097354
Canis lupus familiaris NC002008 1231	Arctictis binturong T605 2	0,18097472
Mustela sibirica AP017394 11	Genetta abyssinica MG489822 1	0,18098181
Mustela sibirica NC020637 6	Fossa fossana D350 1	0,18098319
Meles meles T303 3	Galidictis fasciata DM333 1	0,18098696
Martes martes T302 3	Crossarchus platycephalus C7R66 1	0,18098703
Pusa sibirica NC008432 2	Nyctereutes procyonoides NC013700 3	0,18098705
Mustela erminea T305 2	Urva javanica T413 1	0,18098733
Vulpes zerda KJ603240 1	Cystophora cristata NC008427 1	0,18098775
Mustela sibirica AP017394 11	Arctocephalus pusillus NC008417 1	0,18098815
Nasua nasua NC020647 1	Mustela altaica NC021751 1	0,18098924
Viverra zibetha T609 1	Martes melampus NC009678 1	0,18098964
Ursus arctos EU497665 29	Homotherium latidens MF871702 3	0,18099
Phocarctos hookeri NC008418 1	Melogale moschata NC020644 1	0,18099107
Arctocephalus gazella BK010918 1	Aonyx cinerea NC035814 2	0,18099165
Zalophus wollebaeki SRR4431565 1	Enhydra lutris NC009692 1	0,18099168

Paguma larvata PDDS11 2	Lutrogale perspicillata NC035811 1	0,19742627
Prionodon linsang ERR2391707 1	Otaria byronia OTAB 1	0,19743058
Nyctereutes procyonoides NC013700 3	Tapirus terrestris T358	0,19747739
Prionodon linsang ERR2391707 1	Arctocephalus townsendi NC008420 1	0,19747782
Paradoxurus jerdoni MH464793 1	Bassaricyon neblina SRX1097850 1	0,19749989
Proteles cristata T393 6	Ailuropoda melanoleuca NC009492 5	0,19752578
Nasua nasua NC020647 1	Civettictis civetta NC033378 1	0,19753945
Nasua nasua NC020647 1	Mellivora capensis T370 1	0,1975408
Suricata suricatta SSM10 1	Nasua nasua NC020647 1	0,19755161
Urocyon littoralis catalinae KP129018	Cynictis penicillata T375 1	0,19755174
Viverricula indica NC025296 2	Nasua nasua NC020647 1	0,19756758
Ursus arctos isabellinus 18B5 2	Crocuta crocuta NC020670 3	0,1975781
Urocyon littoralis catalinae KP129018	Panthera leo spelaea KX258452 2	0,19758533
Urocyon cinereoargenteus NC026723 2	Panthera leo NERO 19	0,19759719
Martes martes T302 3	Tapirus terrestris T358	0,19761613
Nasua nasua NC020647 1	Atilax paludinosus T606 1	0,19761781
Nasua nasua NC020647 1	Xenogale naso C07XAR110 1	0,19761825
Vulpes zerda KJ603240 1	Ailurus fulgens styani NC009691 1	0,19761865
Prionodon linsang ERR2391707 1	Phocarctos hookeri NC008418 1	0,19762066
Nasua nasua NC020647 1	Galerella sanguinea T378 1	0,19762841
Lycaon pictus NC028427 2	Lutrogale perspicillata NC035811 1	0,19762881
Lycalopex sechurae KT448284 1	Bassaricyon neblina SRX1097850 1	0,19764441
Urocyon cinereoargenteus NC026723 2	Panthera leo spelaea KX258452 2	0,19765265
Odobenus rosmarus NC004029 29	Felis chaus NC028307 1	0,19765489
Ursus arctos AP012576 6	Crocuta crocuta NC020670 3	0,19766328
Urocyon littoralis catalinae KP129018	Panthera leo NERO 19	0,19766447
Urocyon cinereoargenteus NC026723 2	Neofelis nebulosa NC008450 3	0,19766781
Speothos venaticus C48 2	Phocarctos hookeri NC008418 1	0,19770011
Mephitis mephitis NC020648 1	Tapirus terrestris T358	0,19770236
Urocyon cinereoargenteus NC026723 2	Proteles cristata T393 6	0,19770473
Paguma larvata PDDS11 2	Odobenus rosmarus NC004029 29	0,19770876
Martes americana NC020642 1	Tapirus terrestris T358	0,19774491
Vulpes lagopus NC026529 3	Cynogale bennetti KY117544 1	0,1977544
Paguma larvata PDDS11 2	Bassaricyon neblina SRX1097850 1	0,19776793
Viverricula indica NC025296 2	Odobenus rosmarus NC004029 29	0,19777974
Odobenus rosmarus NC004029 29	Mephitis mephitis NC020648 1	0,19779351
Panthera leo spelaea KX258452 2	Odobenus rosmarus NC004029 29	0,19780469
Spilogale putorius NC010497 1	Helogale parvula SRR7637809 1	0,19780715
Urocyon cinereoargenteus NC026723 2	Ailurus fulgens NC011124 1	0,19782442
Ursus thibetanus mupinensis NC00875	Tapirus terrestris T358	0,19782937
Panthera uncia NC010638 1	Nasua nasua NC020647 1	0,19783707
Proteles cristata T393 6	Ailurus fulgens NC011124 1	0,19784166
Viverricula indica KX891751 1	Odobenus rosmarus NC004029 29	0,19784309
Crocuta crocuta NC020670 3	Chrysocyon brachyurus NC024172 1	0,197862
Lutra lutra NC011358 9	Tapirus terrestris T358	0,19786929
Phocarctos hookeri NC008418 1	Cuon alpinus NC013445 3	0,19787526
Martes zibellina NC011579 39	Tapirus terrestris T358	0,1978803
Nyctereutes procyonoides NC013700 3	Nasua nasua NC020647 1	0,19788978
Nasua nasua NC020647 1	Canis anthus NC027956 2	0,19789037
Urocyon cinereoargenteus NC026723 2	Bassaricyon neblina SRX1097850 1	0,19789158
Neofelis nebulosa NC008450 3	Mephitis mephitis NC020648 1	0,19789222
Odobenus rosmarus NC004029 29	Diplogale hosei MH464790 1	0,19790055
Nandinia binotata NC024567 1	Mellivora capensis T370 1	0,19794817
Mustela itatsi NC034330 19	Tapirus terrestris T358	0,19795604
Urocyon littoralis catalinae KP129018	Bassaricyon neblina SRX1097850 1	0,1979589
Lycalopex sechurae KT448284 1	Crocuta crocuta NC020670 3	0,19796056
Vulpes zerda KJ603240 1	Crocuta crocuta NC020670 3	0,19797234
Paradoxurus hermaphroditus NLNC 1	Odobenus rosmarus NC004029 29	0,19797505
Ursus arctos GU573491 207	Crocuta crocuta NC020670 3	0,19798194
Crocuta crocuta NC020670 3	Ailuropoda melanoleuca NC009492 5	0,19799617
Lutra lutra NC011358 9	Cynogale bennetti KY117544 1	0,19800888
Otocyon megalotis SAF1 2	Cynogale bennetti KY117544 1	0,19801983
Nasua nasua NC020647 1	Urva brachyura KY117547 1	0,19802386
Panthera leo NERO 19	Odobenus rosmarus NC004029 29	0,19802992
Ursus arctos pruinosus MG066703 3	Nyctereutes procyonoides NC013700 3	0,1980346
Galictis vittata T412 1	Cynogale bennetti KY117544 1	0,19804613
Panthera tigris NC010642 35	Nasua nasua NC020647 1	0,19804825
Vulpes zerda KJ603240 1	Odobenus rosmarus NC004029 29	0,19811897
Vulpes ferrilata NC027935 1	Lutrogale perspicillata NC035811 1	0,19815591
Nasua nasua NC020647 1	Canis latrans NC008093 7	0,1981594
Mellivora capensis T370 1	Leopardus pardalis T262 1	0,1981701
Odobenus rosmarus NC004029 29	Urva javanica T413 1	0,19817747
Panthera uncia NC010638 1	Mephitis mephitis NC020648 1	0,1981872
Lontra canadensis SRR10409165 1	Diplogale hosei MH464790 1	0,19822483
Ursus arctos isabellinus 18B5 2	Urocyon cinereoargenteus NC026723 2	0,1982366
Ursus arctos isabellinus 18B5 2	Urocyon littoralis catalinae KP129018	0,19823664
Lycaon pictus NC028427 2	Bassaricyon neblina SRX1097850 1	0,19823782
Prionodon pardicolor NC024569 2	Nasua nasua NC020647 1	0,19823827
Prionodon pardicolor NC024569 2	Bassaricyon neblina SRX1097850 1	0,19823828
Vulpes ferrilata NC027935 1	Proteles cristata T393 6	0,19824331
Vulpes ferrilata NC027935 1	Crocuta crocuta NC020670 3	0,19824402
Odobenus rosmarus NC004029 29	Atilax paludinosus T606 1	0,19824687
Panthera uncia KP202269 1	Odobenus rosmarus NC004029 29	0,1982635
Urocyon littoralis catalinae KP129018	Neofelis nebulosa NC008450 3	0,19827378
Martes melampus NC009678 1	Tapirus terrestris T358	0,19827679
Mellivora capensis T370 1	Lynx pardinus NC028319 161	0,19830418
Panthera uncia NC010638 1	Bassaricyon neblina SRX1097850 1	0,19831043
Lutrogale perspicillata NC035811 1	Chrysocyon brachyurus NC024172 1	0,19831662
Tremarctos ornatus NC009969 2	Tapirus terrestris T358	0,19835408
Paradoxurus hermaphroditus NC03959	Lontra canadensis SRR10409165 1	0,19836564
Proteles cristata T393 6	Lycalopex sechurae KT448284 1	0,19837402
Diplogale hosei MH464790 1	Aonyx cinerea NC035814 2	0,19837487
Speothos venaticus C48 2	Cryptoprocta ferox CFC13 1	0,1983784
Odobenus rosmarus NC004029 29	Eupleres goudotii D128 1	0,19838332
Canis mesomelas KT448280 1	Bassaricyon neblina SRX1097850 1	0,1983985
Panthera tigris amoyensis NC014770 2	Odobenus rosmarus NC004029 29	0,19840829
Prionodon linsang ERR2391707 1	Arctocephalus forsteri NC004023 28	0,19841263
Prionodon linsang ERR2391707 1	Arctocephalus australis MG023139 1	0,19842151
Vulpes zerda KJ603240 1	Ailurus fulgens NC011124 1	0,19842638
Vulpes corsac NC023958 1	Crocuta crocuta NC020670 3	0,19844607
Vulpes ferrilata NC027935 1	Odobenus rosmarus NC004029 29	0,19845158
Crocuta crocuta NC020670 3	Conepatus chinga NC042596 1	0,19849028
Procyon lotor AB462049 4	Tapirus terrestris T358	0,19849047

Lutra sumatrana NC035810 1	Genetta servalina NC024568 2	0,18099199
Lutra lutra LC050126 1	Leopardus jacobita NC028322 1	0,18099329
Otocyon megalotis SAF1 2	Nandinia binotata NC024567 1	0,18099335
Lynx canadensis NC028313 1	Lutra lutra NC011358 9	0,18099336
Meles meles T303 3	Leptailurus serval NC028316 1	0,1809942
Vulpes lagopus NC026529 3	Phoca fasciata NC008428 1	0,18099503
Martes melampus NC009678 1	Catopuma temminckii NC027115 41	0,18099526
Taxidea taxus NC020646 1	Prionodon pardicolor NC024569 2	0,1809956
Prionailurus planiceps KY682741 4	Martes melampus NC009678 1	0,18099572
Phoca groenlandica NC008429 54	Helogale parvula SRR7637809 1	0,1809959
Mustela sibirica AP017394 11	Lynx rufus NC014456 3	0,18099602
Zalophus wollebaeki SRR4431565 1	Tremarctos ornatus NC009969 2	0,18099681
Puma concolor NC016470 22	Mustela sibirica AP017394 11	0,18099697
Pardofelis marmorata NLN3 2	Mustela nigripes NC024942 1	0,18099701
Mustela sibirica AP017394 11	Felis silvestris lybica KP202275 4	0,18099718
Suricata suricatta SSM10 1	Canis adustus KT448271 1	0,18099731
Pusa hispida NC 008433 1	Canis anthus NC027956 2	0,18099745
Vulpes corsac NC023958 1	Pusa hispida NC 008433 1	0,18099777
Taxidea taxus NC020646 1	Felis chaus NC028307 1	0,18099783
Martes americana NC020642 1	Lynx rufus NC014456 3	0,18099803
Potos flavus T414 1	Otocolobus manul NC028323 1	0,18099853
Vulpes vulpes NC008434 5	Lynx pardinus NC028319 161	0,18099855
Zalophus wollebaeki SRR4431565 1	Melogale moschata V0735A 1	0,18099882
Viverricula indica KX891751 1	Poecilogale albinucha T602 1	0,18100388
Procyon lotor AB462049 4	Prionailurus bengalensis NC028301 12	0,18100467
Otaria byronia OTAB 1	Neovison vison NC020641 3	0,18100482
Procyon lotor AB462046 3	Leopardus wiedii NC028318 1	0,18100516
Melogale moschata NC020644 1	Leopardus jacobita NC028322 1	0,18100522
Otocyon megalotis SAF1 2	Leopardus pardalis T262 1	0,18100716
Ommatophoca rossii AY377287etc 1	Mungotictis decemlineata NC027828 1	0,18100987
Mungos mungo/gambianus SRR7704821 1	Canis lupus chanco NC010340 4	0,18101035
Speothos venaticus C48 2	Hydrurga leptonyx NC008425 1	0,18101069
Poecilogale albinucha T602 1	Arctodus simus NC011116 1	0,1810108
Neovison vison NC020641 3	Felis chaus NC028307 1	0,18101087
Zalophus wollebaeki SRR4431565 1	Mustela altaica NC021751 1	0,18101186
Ursus thibetanus laniger MH281753 2	Martes americana NC020642 1	0,18101206
Prionailurus planiceps KY682741 4	Ictonyx striatus T299 1	0,18101239
Leopardus wiedii NC028318 1	Ictonyx striatus T299 1	0,18101247
Meles leucurus NC039173 4	Helarctos malayanus NC009968 2	0,18101268
Pusa hispida NC 008433 1	Prionodon pardicolor NC024569 2	0,18101297
Ursus thibetanus formosanus NC009331 1	Melogale moschata NC020644 1	0,18101433
Ursus spelaeus NC011112 8	Meles anakuma NC009677 1	0,18101482
Ursus thibetanus thibetanus NC011118 4	Ailurus fulgens styani NC009691 1	0,1810156
Ursus spelaeus NC011112 8	Ailurus fulgens styani NC009691 1	0,18101592
Smilodon populator MF871700 1	Canis lupus familiaris NC002008 1231	0,18101616
Prionailurus bengalensis CKM45 20	Ictonyx striatus T299 1	0,18101721
Melursus ursinus NC009970 2	Martes foina NC020643 1	0,18102232
Chrysocyon brachyurus NC024172 1	Catopuma temminckii NC027115 41	0,18102324
Fossa fossana D350 1	Eumetopias jubatus NC004030 10	0,18102577
Neofelis nebulosa NC008450 3	Mirounga leonina NC008422 1	0,18103071
Paradoxurus jerdoni MH464793 1	Canis lupus chanco NC010340 4	0,18103501
Catopuma temminckii NC027115 41	Canis lupus familiaris NC002008 1231	0,18103732
Prionailurus planiceps KY682741 4	Martes flavigula NC012141 3	0,1810399
Mustela altaica NC021751 1	Civettictis civetta NC033378 1	0,18104227
Lutra lutra NC011358 9	Arctocephalus australis MG023139 1	0,18104595
Mustela sibirica NC020637 6	Genetta abyssinica MG489822 1	0,18104888
Martes foina NC020643 1	Civettictis civetta NC033378 1	0,18104975
Mustela itatsi NC034330 19	Conepatus chinga NC042596 1	0,18105273
Vulpes lagopus NC026529 3	Pusa sibirica NC008432 2	0,18105295
Meles meles T303 3	Canis latrans NC008093 7	0,18105581
Puma yagouaroundi NC028311 1	Lutra lutra LC050126 1	0,18105844
Meles anakuma NC009677 1	Leopardus wiedii NC028318 1	0,18105982
Meles leucurus NC039173 4	Leopardus jacobita NC028322 1	0,18105988
Leopardus jacobita NC028322 1	Arctonyx collaris NC020645 1	0,18106015
Leopardus wiedii NC028318 1	Arctonyx collaris NC020645 1	0,18106022
Prionailurus planiceps KY682741 4	Meles leucurus NC039173 4	0,18106034
Melursus ursinus NC009970 2	Eumetopias jubatus NC004030 10	0,18106276
Viverra tangalunga MH464792 1	Prionailurus planiceps KY682741 4	0,18106404
Ursus arctos isabellinus 18B5 2	Melogale moschata KP726273 1	0,1810641
Suricata suricatta SSM10 1	Monachus schauinslandi NC008421 1	0,18106425
Prionailurus planiceps NC028312 6	Mustela sibirica NC020637 6	0,18106428
Viverra tangalunga MH464792 1	Otocolobus manul NC028323 1	0,18106511
Vulpes ferrilata NC027935 1	Viverricula indica NC025296 2	0,18106511
Civettictis civetta GLC19 1	Canis latrans NC008093 7	0,18106531
Otocolobus manul NC028323 1	Mustela erminea T305 2	0,18106684
Ursus arctos isabellinus 18B5 2	Meles leucurus NC039173 4	0,18106687
Ursus arctos pruinosus MG066703 3	Meles meles T303 3	0,18106703
Ursus arctos isabellinus 18B5 2	Mustela sibirica AP017394 11	0,18106773
Ursus arctos isabellinus 18B5 2	Mustela sibirica NC020637 6	0,18106778
Procyon lotor AB462046 3	Arctodus simus NC011116 1	0,18106851
Paradoxurus jerdoni MH464793 1	Monachus schauinslandi NC008421 1	0,18106942
Ursus maritimus NC003428 31	Mustela kathiah NC023210 1	0,18107006
Genetta abyssinica MG489822 1	Canis lupus familiaris NC002008 1231	0,18107133
Canis adustus KT448271 1	Arctotherium sp NC030174 1	0,18107204
Prionailurus planiceps KY682741 4	Mustela altaica NC021751 1	0,18107243
Phoca groenlandica NC008429 54	Panthera pardus japonensis KJ866876 8	0,18107247
Melogale moschata NC020644 1	Felis silvestris lybica KP202275 4	0,18107316
Smilodon populator MF871700 1	Ailurus fulgens NC011124 1	0,18107356
Melogale moschata NC020644 1	Leopardus guigna NC028321 1	0,18107435
Melogale moschata NC020644 1	Leopardus geoffroyi NC028320 1	0,18107456
Puma yagouaroundi NC028311 1	Martes martes T302 3	0,18107463
Ommatophoca rossii AY377287etc 1	Urva semitorquata MH464789 1	0,18107608
Neovison vison NC020641 3	Felis margarita NC028308 1	0,18107677
Ursus thibetanus thibetanus NC011118 4	Fossa fossana D350 1	0,18107806
Ursus thibetanus formosanus NC009331 1	Ailurus fulgens NC011124 1	0,18107872
Leopardus pardalis T262 1	Ictonyx striatus T299 1	0,18107912
Prionailurus rubiginosus NC028304 2	Lycalopex sechurae KT448284 1	0,18107957
Zalophus californianus NC008416 1	Mustela kathiah NC023210 1	0,18108143
Zalophus wollebaeki SRR4431565 1	Martes foina NC020643 1	0,18108156
Ursus spelaeus EU327344 13	Mustela kathiah NC023210 1	0,18108395
Pusa hispida NC 008433 1	Proteles cristata T393 6	0,18108447
Ursus thibetanus formosanus NC009331 1	Mustela altaica NC021751 1	0,18108463
Ursus spelaeus NC011112 8	Aonyx cinerea NC035814 2	0,18108504

Paradoxurus hermaphroditus NLNC 1	Nasua nasua NC020647 1	0,19850844
Parahyaena brunnea NC038159 15	Nasua nasua NC020647 1	0,19850925
Panthera pardus japonensis KJ866876	Bassaricyon neblina SRX1097850 1	0,19851296
Vulpes vulpes NC008434 5	Odobenus rosmarus NC004029 29	0,19851752
Vulpes corsac NC023958 1	Odobenus rosmarus NC004029 29	0,19851777
Ursus arctos pruinosus MG066703 3	Crocuta crocuta NC020670 3	0,19852702
Urocyon cinereoargenteus NC026723 2	Panthera uncia NC010638 1	0,19857515
Paradoxurus hermaphroditus NC03959	Odobenus rosmarus NC004029 29	0,1985811
Galidia elegans D146 1	Ailuropoda melanoleuca NC009492 5	0,1985863
Martes pennanti NC020664 16	Tapirus terrestris T358	0,19861932
Vulpes zerda KJ603240 1	Bassaricyon neblina SRX1097850 1	0,1986295
Vulpes ferrilata NC027935 1	Bassaricyon neblina SRX1097850 1	0,19863079
Proteles cristata T393 6	Cuon alpinus NC013445 3	0,19863352
Prionodon linsang ERR2391707 1	Bassaricyon neblina SRX1097850 1	0,19863413
Urocyon littoralis catalinae KP129018	Proteles cristata T393 6	0,19864742
Proteles cristata T393 6	Mephitis mephitis NC020648 1	0,19866508
Panthera leo NERO 19	Bassaricyon neblina SRX1097850 1	0,19867964
Urocyon littoralis catalinae KP129018	Bassaricyon neblina SRX1097850 1	0,19869789
Urocyon littoralis catalinae KP129018	Ailurus fulgens NC011124 1	0,19869971
Pardofelis marmorata NLN3 2	Mellivora capensis T370 1	0,19870885
Proteles cristata T393 6	Nasua nasua NC020647 1	0,19871473
Panthera tigris amoyensis NC014770 2	Nasua nasua NC020647 1	0,19872186
Chrysocyon brachyurus NC024172 1	Bassaricyon neblina SRX1097850 1	0,19872521
Nasua nasua NC020647 1	Arctictis binturong T605 2	0,19872561
Smilodon populator MF871700 1	Mellivora capensis T370 1	0,19874826
Panthera pardus NC010641 5	Bassaricyon neblina SRX1097850 1	0,19875902
Urocyon littoralis catalinae KP129018	Panthera uncia NC010638 1	0,19877708
Speothos venaticus C48 2	Bassaricyon neblina SRX1097850 1	0,19877887
Viverricula indica KX891751 1	Nasua nasua NC020647 1	0,19884332
Urocyon littoralis catalinae KP129018	Melursus ursinus NC009970 2	0,19885546
Odobenus rosmarus NC004029 29	Cryptoprocta ferox CFC13 1	0,19887085
Ursus maritimus GU573488 Svalbard	Urocyon cinereoargenteus NC026723 2	0,19890985
Ursus maritimus GU573488 Svalbard	Urocyon littoralis catalinae KP129018	0,19890989
Mellivora capensis T370 1	Leopardus wiedii NC028318 1	0,19891113
Spilogale putorius NC010497 1	Tapirus terrestris T358	0,1989211
Urocyon cinereoargenteus NC026723 2	Melursus ursinus NC009970 2	0,19892272
Neofelis nebulosa NC008450 3	Bassaricyon neblina SRX1097850 1	0,19895336
Ursus arctos EU497665 29	Urocyon cinereoargenteus NC026723 2	0,19897729
Vulpes zerda KJ603240 1	Ailuropoda melanoleuca NC009492 5	0,19898374
Odobenus rosmarus NC004029 29	Xenogale naso C07XAR110 1	0,19898811
Helarctos malayanus NC009968 2	Tapirus terrestris T358	0,19898869
Salanoia concolor D378 1	Nasua nasua NC020647 1	0,19903491
Paradoxurus jerdoni MH464793 1	Nasua nasua NC020647 1	0,19904721
Viverricula indica NC025296 2	Mellivora capensis T370 1	0,19910671
Melogale moschata NC020644 1	Tapirus terrestris T358	0,19911003
Mellivora capensis T370 1	Lynx canadensis NC028313 1	0,19911182
Ursus arctos EU497665 29	Urocyon littoralis catalinae KP129018	0,199112
Prionailurus viverrinus NC028305 1	Mellivora capensis T370 1	0,19911408
Speothos venaticus C48 2	Proteles cristata T393 6	0,19913156
Urocyon littoralis catalinae KP129018	Conepatus chinga NC042596 1	0,19916312
Mellivora capensis T370 1	Leopardus tigrinus NC028317 1	0,19918044
Odobenus rosmarus NC004029 29	Urva brachyura KY117547 1	0,199186
Vulpes lagopus NC026529 3	Odobenus rosmarus NC004029 29	0,19919245
Ursus arctos AP012576 6	Urocyon cinereoargenteus NC026723 2	0,19919571
Ursus arctos AP012576 6	Urocyon littoralis catalinae KP129018	0,19919579
Odobenus rosmarus NC004029 29	Arctictis binturong T605 2	0,19920538
Urocyon littoralis catalinae KP129018	Cynogale bennetti KY117544 1	0,1992413
Nasua nasua NC020647 1	Ichneumia albicauda T603 1	0,19924858
Urocyon cinereoargenteus NC026723 2	Conepatus chinga NC042596 1	0,19929783
Odobenus rosmarus NC004029 29	Galerella sanguinea T378 1	0,19933659
Mellivora capensis T370 1	Genetta servalina NC024568 2	0,19937405
Urocyon cinereoargenteus NC026723 2	Cynogale bennetti KY117544 1	0,19937615
Odobenus rosmarus NC004029 29	Nyctereutes procyonoides NC013700 3	0,19939533
Cuon alpinus NC013445 3	Tapirus terrestris T358	0,19940962
Parahyaena brunnea NC038159 15	Odobenus rosmarus NC004029 29	0,19941206
Cryptoprocta ferox CFC13 1	Conepatus chinga NC042596 1	0,19944093
Mephitis mephitis NC020648 1	Cryptoprocta ferox CFC13 1	0,19946693
Panthera uncia NC010638 1	Odobenus rosmarus NC004029 29	0,19947594
Nasua nasua NC020647 1	Urva javanica/auropunctata NC006835	0,19947733
Odobenus rosmarus NC004029 29	Crocuta crocuta NC020670 3	0,1994828
Panthera pardus NC010641 5	Nasua nasua NC020647 1	0,19949719
Mellivora capensis T370 1	Lynx lynx NC027083 4	0,19951552
Ursus arctos GU573491 207	Urocyon cinereoargenteus NC026723 2	0,19951582
Ursus arctos GU573491 207	Urocyon littoralis catalinae KP129018	0,19951586
Panthera onca KP202264 2	Mellivora capensis T370 1	0,19951701
Potos flavus T414 1	Tapirus terrestris T358	0,19955854
Urocyon littoralis catalinae KP129018	Galidia elegans D146 1	0,19957256
Vulpes lagopus NC026529 3	Lutrogale perspicillata NC035811 1	0,19963661
Ictonyx striatus T299 1	Tapirus terrestris T358	0,19964534
Melogale moschata KP726273 1	Tapirus terrestris T358	0,19965041
Ursus arctos isabellinus 18B5 2	Tapirus terrestris T358	0,19965065
Mellivora capensis T370 1	Leopardus geoffroyi NC028320 1	0,19965321
Crocuta crocuta NC020670 3	Canis mesomelas KT448280 1	0,19965732
Odobenus rosmarus NC004029 29	Mungotictis decemlineata NC027828 1	0,19966868
Mellivora capensis T370 1	Fossa fossana D350 1	0,1996994
Urocyon cinereoargenteus NC026723 2	Galidia elegans D146 1	0,19970722
Odobenus rosmarus NC004029 29	Ichneumia albicauda T603 1	0,19974133
Odobenus rosmarus NC004029 29	Urva javanica/auropunctata NC006835	0,19976789
Vulpes zerda KJ603240 1	Cynogale bennetti KY117544 1	0,19977784
Ursus arctos pruinosus MG066703 3	Tapirus terrestris T358	0,19979341
Panthera tigris NC010642 35	Odobenus rosmarus NC004029 29	0,19982255
Ursus maritimus NC003428 31	Urocyon cinereoargenteus NC026723 2	0,1998525
Ursus maritimus NC003428 31	Urocyon littoralis catalinae KP129018	0,19985254
Ursus maritimus GU573488 Svalbard	Tapirus terrestris T358	0,19985326
Ursus arctos GU573486 5	Urocyon cinereoargenteus NC026723 2	0,19985852
Ursus arctos GU573486 5	Urocyon littoralis catalinae KP129018	0,19985852
Mellivora capensis T370 1	Callorhinus ursinus NC008415 1	0,19990081
Lontra canadensis SRR10409165 1	Cynogale bennetti KY117544 1	0,19990942
Paradoxurus hermaphroditus NC03959	Lutrogale perspicillata NC035811 1	0,19991729
Ursus arctos GU573491 207	Tapirus terrestris T358	0,19992043
Spilogale putorius NC010497 1	Proteles cristata T393 6	0,19997446
Odobenus rosmarus NC004029 29	Neofelis nebulosa NC008450 3	0,1999844
Speothos venaticus C48 2	Lutrogale perspicillata NC035811 1	0,19998492

Proteles cristata T393 6	Phoca vitulina NC001325 1	0,18108578
Viverricula indica KX891745 1	Chrysocyon brachyurus NC024172 1	0,18108596
Ursus thibetanus laniger MH281753 2	Martes foina NC020643 1	0,18108957
Zalophus wollebaeki SRR4431565 1	Ursus spelaeus NC011112 8	0,18109263
Zalophus californianus NC008416 1	Ursus arctos GU573486 5	0,18109594
Martes foina NC020643 1	Acinonyx jubatus NC005212 3	0,1810962
Ursus americanus JX196366 3	Arctocephalus forsteri KT693377 17	0,1810964
Prionailurus rubiginosus NC028304 2	Canis lupus familiaris NC002008 1231	0,18110041
Neovison vison NC020641 3	Canis lupus familiaris NC002008 1231	0,18110071
Smilodon populator MF871700 1	Bassariscus sumichrasti SRX1099089 1	0,1811008
Martes flavigula NC012141 3	Lynx rufus NC014456 3	0,18110992
Neophoca cinerea NC008419 1	Lutra lutra NC011358 9	0,18111348
Mustela nigripes NC024942 1	Genetta abyssinica MG489822 1	0,18111435
Arctonyx collaris NC020645 1	Arctocephalus gazella BK010918 1	0,1811149
Martes americana NC020642 1	Arctocephalus australis MG023139 1	0,18111882
Phocarctos hookeri NC008418 1	Melogale moschata KP726273 1	0,18111882
Martes pennanti NC020664 16	Callorhinus ursinus NC008415 1	0,18112177
Zalophus californianus NC008416 1	Ursus americanus JX196366 3	0,18112242
Taxidea taxus NC020646 1	Suricata suricatta SSM10 1	0,18112522
Martes pennanti NC020664 16	Arctotherium sp NC030174 1	0,18112548
Otaria byronia OTAB 1	Mustela eversmannii NC028013 1	0,18112601
Lynx canadensis NC028313 1	Lutra lutra LC050126 1	0,18112801
Leopardus tigrinus NC028317 1	Enhydra lutris NC009692 1	0,18112814
Neovison vison NC020641 3	Fossa fossana D350 1	0,18112825
Meles anakuma NC009677 1	Felis silvestris lybica KP202275 4	0,18112859
Felis margarita NC028308 1	Arctonyx collaris NC020645 1	0,18112901
Felis margarita NC028308 1	Ailurus fulgens styani NC009691 1	0,18112916
Poecilogale albinucha T602 1	Arctocephalus gazella BK010918 1	0,18112974
Viverricula indica KX891745 1	Mustela nigripes NC024942 1	0,18113065
Ursus thibetanus mupinensis NC008753 2	Martes zibellina NC011579 39	0,18113089
Halichoerus grypus NC001602 2	Arctictis binturong T605 2	0,18113099
Prionailurus bengalensis NC028301 12	Lutra sumatrana NC035810 1	0,18113129
Mustela sibirica NC020637 6	Genetta servalina NC024568 2	0,18113135
Viverra tangalunga MH464792 1	Felis catus NC001700 2	0,18113156
Mustela sibirica NC020637 6	Civettictis civetta GLC19 1	0,18113178
Paradoxurus hermaphroditus NC039591 1	Mustela erminea T305 2	0,18113321
Paradoxurus hermaphroditus NLNC 1	Conepatus chinga NC042596 1	0,18113343
Melursus ursinus NC009970 2	Martes flavigula NC012141 3	0,18113364
Ursus maritimus GU573488 Svalbard	Meles leucurus NC039173 4	0,18113409
Zalophus wollebaeki SRR4431565 1	Mustela eversmannii NC028013 1	0,18113422
Prionailurus bengalensis NC028301 12	Martes americana NC020642 1	0,18113445
Mustela sibirica AP017394 11	Canis aureus KT448274 1	0,18113616
Prionailurus bengalensis CKM45 20	Mustela itatsi NC034330 19	0,18113631
Paradoxurus hermaphroditus NLNC 1	Monachus schauinslandi NC008421 1	0,18113698
Meles leucurus NC039173 4	Arctocephalus forsteri NC004023 28	0,1811374
Phoca vitulina NC001325 1	Galidia elegans D146 1	0,18113792
Prionailurus bengalensis CKM45 20	Martes zibellina NC011579 39	0,18113859
Mustela nivalis T306 5	Lynx rufus NC014456 3	0,18114063
Leopardus pardalis NC028315 1	Bassariscus sumichrasti SRX1099089 1	0,18114127
Panthera onca NC022842 1	Mustela kathiah NC023210 1	0,18114246
Zalophus wollebaeki SRR4431565 1	Ursus thibetanus mupinensis NC008753 2	0,18114311
Lynx lynx NC027083 4	Canis aureus KT448274 1	0,18114352
Ursus thibetanus thibetanus NC011118 4	Arctocephalus forsteri KT693377 17	0,18114366
Ursus arctos GU573486 5	Arctocephalus pusillus NC008417 1	0,18114406
Ursus thibetanus mupinensis NC008753 2	Leopardus pardalis NC028315 1	0,18114555
Ursus thibetanus formosanus NC009331 1	Lutra sumatrana NC035810 1	0,18114574
Phoca groenlandica NC008429 54	Panthera tigris amoyensis NC014770 2	0,18114841
Profelis aurata NC028299 1	Ictonyx striatus T299 1	0,18114933
Ursus spelaeus NC011112 8	Arctocephalus gazella BK010918 1	0,18115
Ursus spelaeus NC011112 8	Ailurus fulgens NC011124 1	0,18115056
Martes americana NC020642 1	Acinonyx jubatus NC005212 3	0,18115073
Helarctos malayanus NC009968 2	Civettictis civetta GLC19 1	0,18115364
Zalophus californianus NC008416 1	Melogale moschata NC020644 1	0,18115367
Mustela altaica NC021751 1	Acinonyx jubatus NC005212 3	0,18115477
Meles meles T303 3	Diplogale hosei MH464790 1	0,18115573
Mirounga angustirostris SRR10331586 1	Chrysocyon brachyurus NC024172 1	0,18115692
Helarctos malayanus NC009968 2	Genetta abyssinica MG489822 1	0,18116367
Spilogale putorius NC010497 1	Mustela eversmannii NC028013 1	0,18116386
Phoca groenlandica NC008429 54	Canis lupus chanco NC010340 4	0,18116421
Mustela frenata NC020640 1	Diplogale hosei MH464790 1	0,1811679
Leopardus colocolo NC028314 1	Canis aureus KT448274 1	0,18116818
Martes flavigula NC012141 3	Leptailurus serval NC028316 1	0,18117213
Panthera pardus NC010641 5	Mirounga angustirostris SRR10331586 1	0,1811755
Vulpes lagopus NC026529 3	Halichoerus grypus NC001602 2	0,1811758
Melogale moschata V0735A 1	Callorhinus ursinus NC008415 1	0,18118231
Mustela sibirica AP017394 11	Arctocephalus townsendi NC008420 1	0,18118393
Martes pennanti NC020664 16	Canis adustus KT448271 1	0,18118523
Arctocephalus gazella BK010918 1	Ailurus fulgens NC011124 1	0,18118531
Fossa fossana D350 1	Callorhinus ursinus NC008415 1	0,18118571
Fossa fossana D350 1	Arctocephalus forsteri KT693377 17	0,1811873
Potos flavus T414 1	Arctocephalus forsteri KT693377 17	0,18118806
Martes pennanti NC020664 16	Homotherium latidens MF871702 3	0,18118836
Viverra tangalunga MH464792 1	Neophoca cinerea NC008419 1	0,18118848
Crossarchus platycephalus C7R66 1	Arctocephalus pusillus NC008417 1	0,18119058
Mustela erminea T305 2	Canis anthus NC027956 2	0,18119434
Potos flavus T414 1	Otaria byronia OTAB 1	0,18119529
Mustela kathiah NC023210 1	Lynx canadensis NC028313 1	0,18119653
Mustela eversmannii NC028013 1	Lynx canadensis NC028313 1	0,18119764
Phoca groenlandica NC008429 54	Cynictis penicillata T375 1	0,18119766
Viverra zibetha T609 1	Mustela eversmannii NC028013 1	0,18119812
Potos flavus T414 1	Leptailurus serval NC028316 1	0,18120056
Viverricula indica NC025296 2	Ailurus fulgens NC011124 1	0,1812012
Prionodon pardicolor NC024569 2	Mustela kathiah NC023210 1	0,18120255
Vulpes lagopus NC026529 3	Monachus schauinslandi NC008421 1	0,18120335
Mungos mungo/gambianus SRR7704821 1	Canis adustus KT448271 1	0,18120416
Procyon lotor AB462046 3	Lynx lynx NC027083 4	0,18120453
Leptailurus serval NC028316 1	Canis latrans NC008093 7	0,18120469
Ursus maritimus GU573488 Svalbard	Mustela kathiah NC023210 1	0,18120487
Otaria byronia OTAB 1	Mustela altaica NC021751 1	0,18120551
Leopardus pardalis NC028315 1	Arctocephalus pusillus NC008417 1	0,1812069
Martes martes T302 3	Leopardus geoffroyi NC028320 1	0,18120749
Martes martes T302 3	Lynx rufus NC014456 3	0,18120822
Mustela frenata NC020640 1	Canis mesomelas KT448280 1	0,18120866

Prionailurus rubiginosus NC028304 2	Mellivora capensis T370 1	0,19998908
Suricata suricatta SSM10 1	Odobenus rosmarus NC004029 29	0,19999596
Neofelis nebulosa NC008450 3	Nasua nasua NC020647 1	0,20003101
Cuon alpinus NC013445 3	Bassaricyon neblina SRX1097850 1	0,20003273
Nasua nasua NC020647 1	Eupleres goudotii D128 1	0,20004144
Nasua nasua NC020647 1	Urva javanica T413 1	0,2000436
Mellivora capensis T370 1	Lynx rufus NC014456 3	0,20005473
Urocyon cinereoargenteus NC026723 2	Lutrogale perspicillata NC035811 1	0,20010833
Mellivora capensis T370 1	Felis chaus NC028307 1	0,20012437
Spilogale putorius NC010497 1	Neofelis nebulosa NC008450 3	0,20013738
Spilogale putorius NC010497 1	Otocyon megalotis SAF1 2	0,20016198
Odobenus rosmarus NC004029 29	Canis aureus KT448274 1	0,20019491
Odobenus rosmarus NC004029 29	Cynictis penicillata T375 1	0,20019792
Odobenus rosmarus NC004029 29	Hyaena hyaena NC020669 1	0,20021586
Ursus americanus JX196366 3	Tapirus terrestris T358	0,20021819
Nasua nasua NC020647 1	Cynictis penicillata T375 1	0,20024487
Viverricula indica KX891745 1	Mellivora capensis T370 1	0,20024848
Mellivora capensis T370 1	Civettictis civetta GLC19 1	0,20024927
Mellivora capensis T370 1	Leptailurus serval NC028316 1	0,20025901
Otocolobus manul NC028323 1	Mellivora capensis T370 1	0,20025918
Urocyon cinereoargenteus NC026723 2	Mephitis mephitis NC020648 1	0,20027289
Prionodon pardicolor NC024569 2	Odobenus rosmarus NC004029 29	0,20028119
Lutrogale perspicillata NC035811 1	Cuon alpinus NC013445 3	0,20028444
Vulpes vulpes NC008434 5	Tapirus terrestris T358	0,20031521
Nasua nasua NC020647 1	Cryptoprocta ferox CFC13 1	0,20038959
Ursus arctos pruinosus MG066703 3	Urocyon cinereoargenteus NC026723 2	0,20039134
Ursus maritimus NC003428 31	Tapirus terrestris T358	0,2003922
Nasua nasua NC020647 1	Lycaon pictus NC028427 2	0,20039243
Odobenus rosmarus NC004029 29	Bdeogale nigripes GLC15 1	0,20040143
Urocyon littoralis catalinae KP129018	Lutrogale perspicillata NC035811 1	0,20044498
Melogale moschata V0735A 1	Tapirus terrestris T358	0,2004602
Ursus spelaeus EU327344 13	Tapirus terrestris T358	0,20047837
Odobenus rosmarus NC004029 29	Canis lupus familiaris NC002008 1231	0,2004941
Nasua nasua NC020647 1	Mungotictis decemlineata NC027828	0,20051624
Cuon alpinus NC013445 3	Crocuta crocuta NC020670 3	0,20052309
Urocyon cinereoargenteus NC026723 2	Crocuta crocuta NC020670 3	0,20053306
Ursus arctos GU573486 5	Tapirus terrestris T358	0,20053658
Ursus thibetanus thibetanus NC01111	Mellivora capensis T370 1	0,20053989
Proteles cristata T393 6	Canis mesomelas KT448280 1	0,20055962
Arctotherium sp NC030174 1	Tapirus terrestris T358	0,20057255
Ursus arctos pruinosus MG066703 3	Urocyon littoralis catalinae KP129018	0,20059338
Ursus arctos AP012576 6	Tapirus terrestris T358	0,20060861
Urocyon littoralis catalinae KP129018	Mephitis mephitis NC020648 1	0,20060953
Odobenus rosmarus NC004029 29	Lycalopex sechurae KT448284 1	0,20069282
Lutrogale perspicillata NC035811 1	Diplogale hosei MH464790 1	0,20072176
Ailuropoda melanoleuca NC009492 5	Tapirus terrestris T358	0,2007256
Ursus thibetanus mupinensis NC00875	Mellivora capensis T370 1	0,20072868
Mellivora capensis T370 1	Genetta abyssinica MG489822 1	0,20074116
Speothos venaticus C48 2	Crocuta crocuta NC020670 3	0,20074947
Nasua nasua NC020647 1	Cuon alpinus NC013445 3	0,20077194
Ursus spelaeus NC011112 8	Tapirus terrestris T358	0,20081571
Spilogale putorius NC010497 1	Galidia elegans D146 1	0,20084403
Nasua nasua NC020647 1	Lycalopex sechurae KT448284 1	0,20095637
Ommatophoca rossii AY377287etc 1	Mellivora capensis T370 1	0,20096067
Mellivora capensis T370 1	Civettictis civetta NC033378 1	0,20096782
Viverricula indica KX891751 1	Mellivora capensis T370 1	0,2009894
Otocyon megalotis SAF1 2	Tapirus terrestris T358	0,20098971
Galictis vittata T412 1	Tapirus terrestris T358	0,20099823
Puma yagouaroundi NC028311 1	Mellivora capensis T370 1	0,2009991
Paradoxurus hermaphroditus NC03959	Bassaricyon neblina SRX1097850 1	0,20100228
Odobenus rosmarus NC004029 29	Canis lupus chanco NC010340 4	0,20105249
Odobenus rosmarus NC004029 29	Canis latrans NC008093 7	0,20107473
Vulpes zerda KJ603240 1	Tapirus terrestris T358	0,20111981
Nasua nasua NC020647 1	Diplogale hosei MH464790 1	0,20113058
Puma concolor NC016470 22	Mellivora capensis T370 1	0,20120053
Prionailurus bengalensis CKM45 20	Mellivora capensis T370 1	0,2012046
Urocyon littoralis catalinae KP129018	Crocuta crocuta NC020670 3	0,20120622
Nasua nasua NC020647 1	Crocuta crocuta NC020670 3	0,20120759
Mellivora capensis T370 1	Crossarchus platycephalus C7R66 1	0,20124722
Vulpes zerda KJ603240 1	Nasua nasua NC020647 1	0,20125546
Odobenus rosmarus NC004029 29	Lycaon pictus NC028427 2	0,20128854
Mellivora capensis T370 1	Leopardus jacobita NC028322 1	0,20133524
Ursus arctos EU497665 29	Tapirus terrestris T358	0,20133568
Mellivora capensis T370 1	Leopardus guigna NC028321 1	0,20133664
Panthera leo spelaea KX258452 2	Bassaricyon neblina SRX1097850 1	0,20135613
Panthera onca NC022842 1	Mellivora capensis T370 1	0,201415
Urocyon cinereoargenteus NC026723 2	Tapirus terrestris T358	0,20144764
Prionailurus bengalensis NC028301 12	Mellivora capensis T370 1	0,20147039
Zalophus wollebaeki SRR4431565 1	Mellivora capensis T370 1	0,20153518
Ursus thibetanus formosanus NC0093	Mellivora capensis T370 1	0,20154697
Ursus spelaeus NC011112 8	Mellivora capensis T370 1	0,20155013
Vulpes corsac NC023958 1	Tapirus terrestris T358	0,20158853
Odobenus rosmarus NC004029 29	Cuon alpinus NC013445 3	0,20159587
Viverra zibetha T609 1	Mellivora capensis T370 1	0,2015959
Urocyon littoralis catalinae KP129018	Galictis vittata T412 1	0,20168337
Urocyon cinereoargenteus NC026723 2	Odobenus rosmarus NC004029 29	0,2016879
Mellivora capensis T370 1	Leopardus colocolo NC028314 1	0,20169024
Urocyon littoralis catalinae KP129018	Tapirus terrestris T358	0,20171387
Ursus maritimus NC003428 31	Mellivora capensis T370 1	0,20173912
Mellivora capensis T370 1	Acinonyx jubatus NC005212 3	0,20175695
Urocyon cinereoargenteus NC026723 2	Galictis vittata T412 1	0,20181808
Odobenus rosmarus NC004029 29	Chrysocyon brachyurus NC024172 1	0,20183006
Otocyon megalotis SAF1 2	Odobenus rosmarus NC004029 29	0,20183259
Spilogale putorius NC010497 1	Crocuta crocuta NC020670 3	0,20186022
Mellivora capensis T370 1	Genetta genetta T297 1	0,20186898
Profelis aurata NC028299 1	Mellivora capensis T370 1	0,2018753
Nasua nasua NC020647 1	Chrysocyon brachyurus NC024172 1	0,20188883
Urocyon littoralis catalinae KP129018	Odobenus rosmarus NC004029 29	0,20188977
Ursus maritimus GU573488 Svalbard	Mellivora capensis T370 1	0,20194121
Mellivora capensis T370 1	Felis silvestris lybica KP202275 4	0,20194131
Paradoxurus hermaphroditus NC03959	Nasua nasua NC020647 1	0,20194316
Prionodon linsang ERR2391707 1	Odobenus rosmarus NC004029 29	0,20194568
Mellivora capensis T370 1	Helarctos malayanus NC009968 2	0,20195159

Genetta genetta T297 1	Bassariscus sumichrasti SRX1099089 1	0,18120915
Phoca largha NC008430 1	Diplogale hosei MH464790 1	0,18120924
Martes foina NC020643 1	Civettictis civetta GLC19 1	0,18120998
Viverra zibetha T609 1	Martes foina NC020643 1	0,18121237
Conepatus chinga NC042596 1	Arctictis binturong T605 2	0,18121386
Martes zibellina NC011579 39	Arctocephalus forsteri NC004023 28	0,18121444
Martes americana NC020642 1	Arctocephalus forsteri NC004023 28	0,18121463
Leopardus wiedii NC028318 1	Canis aureus KT448274 1	0,18121468
Mustela eversmannii NC028013 1	Acinonyx jubatus NC005212 3	0,18121499
Proteles cristata T393 6	Phoca fasciata NC008428 1	0,18121544
Phoca largha NC008430 1	Chrysocyon brachyurus NC024172 1	0,1812159
Melogale moschata NC020644 1	Helarctos malayanus NC009968 2	0,18121754
Melursus ursinus NC009970 2	Martes martes T302 3	0,18121772
Zalophus californianus NC008416 1	Martes pennanti NC020664 16	0,1812207
Ursus thibetanus laniger MH281753 2	Leopardus pardalis T262 1	0,1812236
Mustela frenata NC020640 1	Urva javanica/auropunctata NC006835 1	0,18122457
Felis silvestris lybica KP202275 4	Chrysocyon brachyurus NC024172 1	0,18122521
Mustela nivalis T306 5	Leopardus colocolo NC028314 1	0,18122746
Neovison vison NC020641 3	Melursus ursinus NC009970 2	0,18123347
Neovison vison NC020641 3	Helarctos malayanus NC009968 2	0,181235
Zalophus californianus NC008416 1	Ursus spelaeus EU327344 13	0,18124279
Melogale moschata V0735A 1	Conepatus chinga NC042596 1	0,18124297
Martes flavigula NC012141 3	Leopardus geoffroyi NC028320 1	0,18124473
Nasua nasua NC020647 1	Lontra canadensis SRR10409165 1	0,18124573
Neofelis nebulosa NC008450 3	Mirounga angustirostris SRR10331586 1	0,18124619
Callorhinus ursinus NC008415 1	Ailurus fulgens NC011124 1	0,18124899
Arctocephalus forsteri KT693377 17	Ailurus fulgens NC011124 1	0,18125123
Martes zibellina NC011579 39	Crossarchus platycephalus C7R66 1	0,1812517
Neophoca cinerea NC008419 1	Meles meles T303 3	0,18125215
Viverra tangalunga MH464792 1	Arctocephalus forsteri KT693377 17	0,18125235
Meles meles T303 3	Urva javanica T413 1	0,18125257
Phocarctos hookeri NC008418 1	Arctonyx collaris NC020645 1	0,18125285
Paguma larvata PDDS11 2	Ommatophoca rossii AY377287etc 1	0,18125385
Melogale moschata KP726273 1	Arctocephalus gazella BK010918 1	0,18125418
Nyctereutes procyonoides NC013700 3	Crossarchus platycephalus C7R66 1	0,18125609
Vulpes ferrilata NC027935 1	Erignathus barbatus NC008426 1	0,18125652
Taxidea taxus NC020646 1	Paradoxurus hermaphroditus NLNC 1	0,18125726
Crossarchus platycephalus C7R66 1	Aonyx cinerea NC035814 2	0,18125837
Tremarctos ornatus NC009969 2	Melogale moschata NC020644 1	0,18126014
Martes melampus NC009678 1	Civettictis civetta GLC19 1	0,18126032
Mustela erminea T305 2	Mungotictis decemlineata NC027828 1	0,18126041
Viverra zibetha T609 1	Melogale moschata KP726273 1	0,18126304
Melogale moschata KP726273 1	Genetta servalina NC024568 2	0,18126324
Martes melampus NC009678 1	Catopuma badia NC028300 1	0,18126395
Mustela altaica NC021751 1	Fossa fossana D350 1	0,18126419
Neovison vison NC020641 3	Urva semitorquata MH464789 1	0,18126433
Pusa sibirica NC008432 2	Canis aureus KT448274 1	0,18126537
Fossa fossana D350 1	Arctodus simus NC011116 1	0,18126565
Mustela sibirica AP017394 11	Civettictis civetta GLC19 1	0,18126626
Mustela sibirica NC020637 6	Leopardus jacobita NC028322 1	0,18126631
Mustela nigripes NC024942 1	Leopardus tigrinus NC028317 1	0,1812664
Mustela putorius NC020638 4	Felis silvestris lybica KP202275 4	0,18126652
Ursus arctos EU497665 29	Nandinia binotata NC024567 1	0,18126673
Lutra sumatrana NC035810 1	Leopardus jacobita NC028322 1	0,18126678
Melogale moschata V0735A 1	Lynx pardinus NC028319 161	0,18126725
Tremarctos ornatus NC009969 2	Prionailurus bengalensis NC028301 12	0,18126772
Paradoxurus jerdoni MH464793 1	Ailurus fulgens NC011124 1	0,18126924
Prionodon pardicolor NC024569 2	Arctonyx collaris NC020645 1	0,18126936
Melogale moschata NC020644 1	Civettictis civetta GLC19 1	0,18126981
Nyctereutes procyonoides NC013700 3	Leopardus wiedii NC028318 1	0,18127076
Prionailurus viverrinus NC028305 1	Martes zibellina NC011579 39	0,18127103
Phoca vitulina NC001325 1	Canis aureus KT448274 1	0,18127148
Pusa sibirica NC008432 2	Prionodon pardicolor NC024569 2	0,18127237
Puma concolor NC016470 22	Martes martes T302 3	0,18127359
Mustela altaica NC021751 1	Leopardus pardalis T262 1	0,18127443
Mustela nivalis T306 5	Lynx lynx NC027083 4	0,18127458
Leopardus pardalis T262 1	Arctocephalus pusillus NC008417 1	0,1812748
Prionailurus planiceps KY682741 4	Aonyx cinerea NC035814 2	0,18127485
Zalophus wollebaeki SRR4431565 1	Melogale moschata NC020644 1	0,181275
Prionailurus bengalensis CKM45 20	Nyctereutes procyonoides NC013700 3	0,18127516
Taxidea taxus NC020646 1	Melursus ursinus NC009970 2	0,18127675
Ursus thibetanus formosanus NC009331 1	Arctocephalus gazella BK010918 1	0,18127817
Meles anakuma NC009677 1	Acinonyx jubatus NC005212 3	0,18127842
Ursus spelaeus EU327344 13	Melogale moschata KP726273 1	0,18127918
Ursus thibetanus thibetanus NC011118 4	Enhydra lutris NC009692 1	0,18127953
Ursus thibetanus laniger MH281753 2	Lutra sumatrana NC035810 1	0,18128078
Ursus thibetanus mupinensis NC008753 2	Leopardus wiedii NC028318 1	0,18128084
Ursus thibetanus formosanus NC009331 1	Ailurus fulgens styani NC009691 1	0,18128096
Leopardus jacobita NC028322 1	Ictonyx striatus T299 1	0,18128166
Puma concolor NC016470 22	Ictonyx striatus T299 1	0,18128173
Leptailurus serval NC028316 1	Bassariscus sumichrasti SRX1099089 1	0,18128209
Mustela erminea T305 2	Arctictis binturong T605 2	0,18128243
Neovison vison NC020641 3	Catopuma badia NC028300 1	0,18128254
Melursus ursinus NC009970 2	Arctocephalus pusillus NC008417 1	0,18128262
Prionailurus viverrinus NC028305 1	Ictonyx striatus T299 1	0,18128324
Panthera tigris NC010642 35	Mirounga angustirostris SRR10331586 1	0,18128598
Lycaon pictus NC028427 2	Catopuma badia NC028300 1	0,18128787
Ursus thibetanus laniger MH281753 2	Mustela altaica NC021751 1	0,18128814
Ursus thibetanus thibetanus NC011118 4	Civettictis civetta GLC19 1	0,1812883
Ursus spelaeus NC011112 8	Arctocephalus pusillus NC008417 1	0,18129018
Ursus arctos GU573486 5	Neovison vison NC020641 3	0,18129498
Ursus arctos AP012576 6	Ommatophoca rossii AY377287etc 1	0,18129881
Felis silvestris lybica KP202275 4	Canis lupus familiaris NC002008 1231	0,18130247
Pusa sibirica NC008432 2	Cuon alpinus NC013445 3	0,18130386
Ursus americanus JX196366 3	Meles leucurus NC039173 4	0,18130517
Tremarctos ornatus NC009969 2	Martes flavigula NC012141 3	0,18130656
Caracal caracal NC028306 1	Canis lupus chanco NC010340 4	0,18130904
Ictonyx striatus T299 1	Eumetopias jubatus NC004030 10	0,18131068
Civettictis civetta NC033378 1	Canis aureus KT448274 1	0,18131216
Felis silvestris lybica KP202275 4	Canis lupus chanco NC010340 4	0,18131317
Arctotherium sp NC030174 1	Arctocephalus townsendi NC008420 1	0,18131547
Nasua nasua NC020647 1	Mustela sibirica AP017394 11	0,18131621
Lutra sumatrana NC035810 1	Fossa fossana D350 1	0,18131669

Mellivora capensis T370 1	Eumetopias jubatus NC004030 10	0,20204078
Ursus arctos EU497665 29	Mellivora capensis T370 1	0,20207594
Mellivora capensis T370 1	Catopuma temminckii NC027115 41	0,20207621
Ursus arctos GU573486 5	Mellivora capensis T370 1	0,20208959
Nasua nasua NC020647 1	Galidia elegans D146 1	0,20213209
Vulpes lagopus NC026529 3	Tapirus terrestris T358	0,20219526
Speothos venaticus C48 2	Nasua nasua NC020647 1	0,20221199
Zalophus californianus NC008416 1	Mellivora capensis T370 1	0,20222272
Nasua nasua NC020647 1	Canis mesomelas KT448280 1	0,20224939
Mephitis mephitis NC020648 1	Mellivora capensis T370 1	0,20228952
Mellivora capensis T370 1	Felis margarita NC028308 1	0,20241305
Ursus thibetanus laniger MH281753 2	Mellivora capensis T370 1	0,20242409
Odobenus rosmarus NC004029 29	Galidia elegans D146 1	0,20243077
Spilogale putorius NC010497 1	Cryptoprocta ferox CFC13 1	0,20245615
Panthera pardus japonensis KJ866876	Mellivora capensis T370 1	0,20248017
Panthera pardus NC010641 5	Mellivora capensis T370 1	0,20252299
Mellivora capensis T370 1	Felis nigripes NC028309 1	0,20254762
Mellivora capensis T370 1	Caracal caracal NC028306 1	0,20254865
Ursus spelaeus EU327344 13	Mellivora capensis T370 1	0,20256062
Melursus ursinus NC009970 2	Tapirus terrestris T358	0,20256156
Otaria byronia OTAB 1	Mellivora capensis T370 1	0,20259994
Ursus arctos pruinosus MG066703 3	Mellivora capensis T370 1	0,20261468
Mellivora capensis T370 1	Cryptoprocta ferox CFC13 1	0,20267402
Prionailurus planiceps NC028312 6	Mellivora capensis T370 1	0,20268184
Bassaricyon neblina SRX1097850 1	Tapirus terrestris T358	0,2027915
Tremarctos ornatus NC009969 2	Mellivora capensis T370 1	0,20280869
Otocyon megalotis SAF1 2	Nasua nasua NC020647 1	0,20281485
Odobenus rosmarus NC004029 29	Canis anthus NC027956 2	0,20282707
Ursus arctos AP012576 6	Mellivora capensis T370 1	0,20283328
Mellivora capensis T370 1	Arctocephalus pusillus NC008417 1	0,20293534
Ursus arctos GU573491 207	Mellivora capensis T370 1	0,2029511
Mellivora capensis T370 1	Felis catus NC001700 2	0,20301878
Salanoia concolor D378 1	Mellivora capensis T370 1	0,20306881
Parahyaena brunnea NC038159 15	Mellivora capensis T370 1	0,20308198
Cynogale bennetti KY117544 1	Aonyx cinerea NC035814 2	0,20315261
Prionailurus planiceps KY682741 4	Mellivora capensis T370 1	0,20315333
Speothos venaticus C48 2	Tapirus terrestris T358	0,2032821
Mellivora capensis T370 1	Canis adustus KT448271 1	0,2032878
Spilogale putorius NC010497 1	Mellivora capensis T370 1	0,2033219
Vulpes vulpes NC008434 5	Nasua nasua NC020647 1	0,20334263
Aonyx cinerea NC035814 2	Tapirus terrestris T358	0,20335643
Vulpes ferrilata NC027935 1	Tapirus terrestris T358	0,20341125
Ursus arctos isabellinus 18B5 2	Mellivora capensis T370 1	0,20342259
Panthera tigris NC010642 35	Mellivora capensis T370 1	0,20350003
Mellivora capensis T370 1	Arctocephalus townsendi NC008420 1	0,20353942
Prionodon linsang ERR2391707 1	Nasua nasua NC020647 1	0,20354778
Neophoca cinerea NC008419 1	Mellivora capensis T370 1	0,20360872
Mellivora capensis T370 1	Arctotherium sp NC030174 1	0,20362231
Mellivora capensis T370 1	Arctocephalus gazella BK010918 1	0,20381133
Vulpes lagopus NC026529 3	Nasua nasua NC020647 1	0,2038139
Odobenus rosmarus NC004029 29	Canis mesomelas KT448280 1	0,20381857
Odobenus rosmarus NC004029 29	Tapirus terrestris T358	0,20389692
Panthera leo NERO 19	Mellivora capensis T370 1	0,20392874
Mungos mungo MMC7 1	Mellivora capensis T370 1	0,20400834
Mellivora capensis T370 1	Arctocephalus australis MG023139 1	0,2040786
Panthera uncia KP202269 1	Mellivora capensis T370 1	0,20409606
Phocarctos hookeri NC008418 1	Mellivora capensis T370 1	0,20414692
Mellivora capensis T370 1	Galerella sanguinea T378 1	0,20415433
Panthera leo spelaea KX258452 2	Mellivora capensis T370 1	0,20417823
Ursus americanus JX196366 3	Mellivora capensis T370 1	0,20420757
Mellivora capensis T370 1	Catopuma badia NC028300 1	0,20423128
Proteles cristata T393 6	Odobenus rosmarus NC004029 29	0,20426704
Mellivora capensis T370 1	Arctocephalus forsteri KT693377 17	0,20428088
Mellivora capensis T370 1	Galidictis fasciata DM333 1	0,20434824
Vulpes corsac NC023958 1	Nasua nasua NC020647 1	0,20435296
Melursus ursinus NC009970 2	Mellivora capensis T370 1	0,20437674
Urocyon cinereoargenteus NC026723 2	Nasua nasua NC020647 1	0,20442204
Mellivora capensis T370 1	Hemigalus derbyanus MH464791 1	0,20452136
Mellivora capensis T370 1	Atilax paludinosus T606 1	0,20454814
Nasua nasua NC020647 1	Cynogale bennetti KY117544 1	0,2045524
Panthera tigris amoyensis NC014770 2	Mellivora capensis T370 1	0,20457761
Vulpes ferrilata NC027935 1	Nasua nasua NC020647 1	0,20462243
Mellivora capensis T370 1	Conepatus chinga NC042596 1	0,20469165
Mungotictis decemlineata NC027828 1	Mellivora capensis T370 1	0,20475245
Mungos mungo/gambianus SRR77048	Mellivora capensis T370 1	0,20481634
Cynogale bennetti KY117544 1	Bassaricyon neblina SRX1097850 1	0,20495895
Speothos venaticus C48 2	Odobenus rosmarus NC004029 29	0,20512755
Suricata suricatta SSM10 1	Mellivora capensis T370 1	0,20515364
Mellivora capensis T370 1	Arctocephalus forsteri NC004023 28	0,20525558
Urocyon littoralis catalinae KP129018	Nasua nasua NC020647 1	0,20536457
Neofelis nebulosa NC008450 3	Mellivora capensis T370 1	0,20541008
Odobenus rosmarus NC004029 29	Cynogale bennetti KY117544 1	0,20550106
Mellivora capensis T370 1	Ichneumia albicauda T603 1	0,20550406
Mellivora capensis T370 1	Arctodus simus NC011116 1	0,20557566
Mellivora capensis T370 1	Hyaena hyaena NC020669 1	0,20563875
Mellivora capensis T370 1	Canis latrans NC008093 7	0,20589773
Mellivora capensis T370 1	Galidia elegans D146 1	0,20603189
Mellivora capensis T370 1	Bdeogale nigripes GLC15 1	0,20609707
Paguma larvata PDDS11 2	Mellivora capensis T370 1	0,2061743
Mellivora capensis T370 1	Xenogale naso C07XAR110 1	0,20623136
Nasua nasua NC020647 1	Tapirus terrestris T358	0,20630716
Mellivora capensis T370 1	Eupleres goudotii D128 1	0,2063646
Proteles cristata T393 6	Mellivora capensis T370 1	0,2065203
Mellivora capensis T370 1	Canis lupus familiaris NC002008 1231	0,20667845
Mellivora capensis T370 1	Urva semitorquata MH464789 1	0,20670201
Lontra canadensis SRR10409165 1	Tapirus terrestris T358	0,2070578
Panthera uncia NC010638 1	Mellivora capensis T370 1	0,20712605
Nyctereutes procyonoides NC013700 3	Mellivora capensis T370 1	0,20731172
Lutrogale perspicillata NC035811 1	Cynogale bennetti KY117544 1	0,20732377
Otocyon megalotis SAF1 2	Mellivora capensis T370 1	0,20739174
Mellivora capensis T370 1	Canis aureus KT448274 1	0,20752613
Mellivora capensis T370 1	Crocuta crocuta NC020670 3	0,20772958
Vulpes lagopus NC026529 3	Mellivora capensis T370 1	0,20784815

Ursus thibetanus mupinensis NC008753 2	Gulo gulo NC009685 3	0,18131822
Mustela sibirica AP017394 11	Fossa fossana D350 1	0,18131964
Mustela frenata NC020640 1	Urva javanica T413 1	0,18132141
Melogale moschata NC020644 1	Genetta abyssinica MG489822 1	0,18132198
Nasua nasua NC020647 1	Melogale moschata NC020644 1	0,18132387
Martes martes T302 3	Fossa fossana D350 1	0,18132453
Martes pennanti NC020664 16	Arctocephalus gazella BK010918 1	0,18132783
Melogale moschata NC020644 1	Arctocephalus gazella BK010918 1	0,18132831
Meles anakuma NC009677 1	Lynx rufus NC014456 3	0,18132941
Meles leucurus NC039173 4	Felis margarita NC028308 1	0,18133011
Ursus maritimus NC003428 31	Callorhinus ursinus NC008415 1	0,18133058
Viverra zibetha T609 1	Arctonyx collaris NC020645 1	0,18133157
Viverricula indica NC025296 2	Meles anakuma NC009677 1	0,18133168
Mustela sibirica NC020637 6	Lynx rufus NC014456 3	0,18133271
Mustela putorius NC020638 4	Lynx rufus NC014456 3	0,18133272
Paradoxurus jerdoni MH464793 1	Mustela sibirica AP017394 11	0,18133302
Leopardus guigna NC028321 1	Arctonyx collaris NC020645 1	0,18133412
Phoca groenlandica NC008429 54	Nyctereutes procyonoides NC013700 3	0,1813342
Vulpes ferrilata NC027935 1	Pusa hispida NC 008433 1	0,18133536
Vulpes vulpes NC008434 5	Phoca vitulina NC001325 1	0,18133542
Ursus maritimus NC003428 31	Meles leucurus NC039173 4	0,18133603
Martes zibellina NC011579 39	Felis margarita NC028308 1	0,18133667
Martes americana NC020642 1	Leptailurus serval NC028316 1	0,18133692
Ursus maritimus GU573488 Svalbard	Ailurus fulgens styani NC009691 1	0,18133815
Nyctereutes procyonoides NC013700 3	Catopuma temminckii NC027115 41	0,18133973
Panthera tigris amoyensis NC014770 2	Mirounga leonina NC008422 1	0,18133974
Prionailurus bengalensis CKM45 20	Martes americana NC020642 1	0,18133996
Procyon lotor AB462046 3	Felis silvestris lybica KP202275 4	0,18134053
Puma concolor NC016470 22	Melogale moschata NC020644 1	0,18134165
Mustela altaica NC021751 1	Lynx rufus NC014456 3	0,18134184
Zalophus wollebaeki SRR4431565 1	Martes pennanti NC020664 16	0,18134192
Erignathus barbatus NC008426 1	Crocuta crocuta NC020670 3	0,18134196
Prionailurus bengalensis NC028301 12	Martes pennanti NC020664 16	0,18134221
Ursus arctos GU573486 5	Meles leucurus NC039173 4	0,18134227
Smilodon populator MF871700 1	Meles anakuma NC009677 1	0,18134233
Mustela nivalis T306 5	Civettictis civetta GLC19 1	0,1813426
Ursus spelaeus NC011112 8	Callorhinus ursinus NC008415 1	0,18134345
Martes martes T302 3	Catopuma temminckii NC027115 41	0,18134351
Prionailurus planiceps KY682741 4	Arctodus simus NC011116 1	0,18134505
Ursus thibetanus thibetanus NC011118 4	Martes zibellina NC011579 39	0,18134598
Mustela erminea T305 2	Canis mesomelas KT448280 1	0,18134683
Taxidea taxus NC020646 1	Odobenus rosmarus NC004029 29	0,18135028
Genetta servalina NC024568 2	Ailuropoda melanoleuca NC009492 5	0,18135154
Ursus thibetanus laniger MH281753 2	Ailurus fulgens styani NC009691 1	0,18135282
Panthera pardus NC010641 5	Canis adustus KT448271 1	0,18135364
Neofelis nebulosa NC008450 3	Halichoerus grypus NC001602 2	0,18135406
Ursus spelaeus NC011112 8	Melogale moschata NC020644 1	0,18135427
Otaria byronia OTAB 1	Helarctos malayanus NC009968 2	0,18135506
Crossarchus platycephalus C7R66 1	Canis lupus familiaris NC002008 1231	0,18135516
Viverra zibetha T609 1	Ursus thibetanus thibetanus NC011118 4	0,18135561
Ursus thibetanus thibetanus NC011118 4	Genetta servalina NC024568 2	0,18135563
Ursus arctos isabellinus 18B5 2	Neovison vison NC020641 3	0,18135571
Zalophus californianus NC008416 1	Procyon lotor AB462046 3	0,18135834
Phoca largha NC008430 1	Arctictis binturong T605 2	0,18135847
Ursus thibetanus thibetanus NC011118 4	Leopardus pardalis NC028315 1	0,18135945
Ursus thibetanus laniger MH281753 2	Puma yagouaroundi NC028311 1	0,18136334
Civettictis civetta GLC19 1	Canis lupus familiaris NC002008 1231	0,18136515
Martes pennanti NC020664 16	Eumetopias jubatus NC004030 10	0,18136735
Martes flavigula NC012141 3	Genetta servalina NC024568 2	0,18136899
Vulpes ferrilata NC027935 1	Civettictis civetta NC033378 1	0,18137118
Phoca fasciata NC008428 1	Cuon alpinus NC013445 3	0,18137419
Ursus americanus JX196366 3	Arctonyx collaris NC020645 1	0,1813764
Nasua nasua NC020647 1	Meles anakuma NC009677 1	0,18137985
Prionailurus bengalensis NC028301 12	Canis lupus chanco NC010340 4	0,18138285
Neophoca cinerea NC008419 1	Enhydra lutris NC009692 1	0,18138544
Martes americana NC020642 1	Arctocephalus townsendi NC008420 1	0,18138738
Viverra tangalunga MH464792 1	Fossa fossana D350 1	0,18138807
Fossa fossana D350 1	Arctocephalus australis MG023139 1	0,18138918
Salanoia concolor D378 1	Meles meles T303 3	0,18139084
Mustela erminea T305 2	Urva brachyura KY117547 1	0,18139085
Enhydra lutris NC009692 1	Canis adustus KT448271 1	0,1813949
Ursus thibetanus formosanus NC009331 1	Gulo gulo NC009685 3	0,18139493
Meles leucurus NC039173 4	Felis nigripes NC028309 1	0,18139727
Martes martes T302 3	Canis latrans NC008093 7	0,18140005
Paradoxurus jerdoni MH464793 1	Mustela putorius NC020638 4	0,18140031
Mustela sibirica AP017394 11	Genetta servalina NC024568 2	0,18140051
Mustela itatsi NC034330 19	Leopardus pardalis T262 1	0,18140058
Ursus maritimus GU573488 Svalbard	Melogale moschata KP726273 1	0,18140088
Ursus thibetanus mupinensis NC008753 2	Fossa fossana D350 1	0,18140125
Tremarctos ornatus NC009969 2	Lynx pardinus NC028319 161	0,18140145
Monachus schauinslandi NC008421 1	Bdeogale nigripes GLC15 1	0,18140148
Mustela putorius NC020638 4	Felis margarita NC028308 1	0,18140155
Vulpes vulpes NC008434 5	Leptailurus serval NC028316 1	0,18140335
Mustela sibirica NC020637 6	Leopardus geoffroyi NC028320 1	0,18140364
Leopardus pardalis T262 1	Canis latrans NC008093 7	0,18140437
Ursus arctos GU573491 207	Mustela sibirica NC020637 6	0,18140461
Nasua nasua NC020647 1	Lobodon carcinophaga NC008423 1	0,18140557
Ursus thibetanus mupinensis NC008753 2	Arctocephalus pusillus NC008417 1	0,1814068
Ursus arctos pruinosus MG066703 3	Melogale moschata KP726273 1	0,18140709
Panthera tigris NC010642 35	Mirounga leonina NC008422 1	0,18140726
Pusa caspica NC008431 1	Proteles cristata T393 6	0,18140756
Prionailurus planiceps KY682741 4	Martes martes T302 3	0,18140869
Felis silvestris lybica KP202275 4	Aonyx cinerea NC035814 2	0,18140897
Monachus monachus NC044972 5	Cuon alpinus NC013445 3	0,18140909
Mustela altaica NC021751 1	Leopardus wiedii NC028318 1	0,18140933
Cystophora cristata NC008427 1	Canis mesomelas KT448280 1	0,1814103
Prionailurus planiceps NC028312 6	Arctodus simus NC011116 1	0,18141244
Meles leucurus NC039173 4	Arctictis binturong T605 2	0,18141247
Mustela altaica NC021751 1	Leopardus guigna NC028321 1	0,18141288
Neovison vison NC020641 3	Felis nigripes NC028309 1	0,18141317
Prionailurus bengalensis CKM45 20	Martes pennanti NC020664 16	0,18141337
Martes foina NC020643 1	Catopuma temminckii NC027115 41	0,18141524
Phocarctos hookeri NC008418 1	Helarctos malayanus NC009968 2	0,18141557

Mellivora capensis T370 1	Canis lupus chanco NC010340 4	0,20786706
Mellivora capensis T370 1	Urva javanica T413 1	0,20791475
Lutrogale perspicillata NC035811 1	Tapirus terrestris T358	0,20813854
Mellivora capensis T370 1	Ailuropoda melanoleuca NC009492 5	0,20814802
Mellivora capensis T370 1	Urva javanica/auropunctata NC006835	0,20816867
Mellivora capensis T370 1	Cynictis penicillata T375 1	0,20818415
Mellivora capensis T370 1	Chrotogale owstoni T607 1	0,20819071
Prionodon pardicolor NC024569 2	Mellivora capensis T370 1	0,2084063
Mellivora capensis T370 1	Canis mesomelas KT448280 1	0,20852031
Mellivora capensis T370 1	Helogale parvula SRR7637809 1	0,20858723
Mellivora capensis T370 1	Arctictis binturong T605 2	0,20861408
Mellivora capensis T370 1	Canis anthus NC027956 2	0,20892796
Mellivora capensis T370 1	Urva brachyura KY117547 1	0,20899194
Paradoxurus jerdoni MH464793 1	Mellivora capensis T370 1	0,20920435
Vulpes zerda KJ603240 1	Mellivora capensis T370 1	0,20926316
Vulpes vulpes NC008434 5	Mellivora capensis T370 1	0,20932892
Mellivora capensis T370 1	Lycalopex sechurae KT448284 1	0,21012127
Mellivora capensis T370 1	Lycaon pictus NC028427 2	0,21048811
Odobenus rosmarus NC004029 29	Mellivora capensis T370 1	0,21051067
Mellivora capensis T370 1	Chrysocyon brachyurus NC024172 1	0,21069489
Paradoxurus hermaphroditus NLNC 1	Mellivora capensis T370 1	0,21102241
Mellivora capensis T370 1	Cuon alpinus NC013445 3	0,21166278
Vulpes ferrilata NC027935 1	Mellivora capensis T370 1	0,21182057
Prionodon linsang ERR2391707 1	Mellivora capensis T370 1	0,21182209
Mellivora capensis T370 1	Diplogale hosei MH464790 1	0,21193735
Paradoxurus hermaphroditus NC03959	Mellivora capensis T370 1	0,21230215
Vulpes corsac NC023958 1	Mellivora capensis T370 1	0,21283016
Urocyon cinereoargenteus NC026723 2	Mellivora capensis T370 1	0,21290109
Urocyon littoralis catalinae KP129018	Mellivora capensis T370 1	0,21317038
Speothos venaticus C48 2	Mellivora capensis T370 1	0,21351776
Homotherium latidens MF871702 3	Phataginus tricuspis NC026780	0,21589616
Mellivora capensis T370 1	Cynogale bennetti KY117544 1	0,21593523
Mellivora capensis T370 1	Tapirus terrestris T358	0,21653745
Hemigalus derbyanus MH464791 1	Phataginus tricuspis NC026780	0,21846072
Canis adustus KT448271 1	Phataginus tricuspis NC026780	0,2208854
Phataginus tricuspis NC026780	Tapirus terrestris T358	0,22487813
Puma yagouaroundi NC028311 1	Phataginus tricuspis NC026780	0,22524206
Puma concolor NC016470 22	Phataginus tricuspis NC026780	0,22557169
Catopuma badia NC028300 1	Phataginus tricuspis NC026780	0,22578776
Arctictis binturong T605 2	Phataginus tricuspis NC026780	0,22595443
Leptailurus serval NC028316 1	Phataginus tricuspis NC026780	0,22631142
Prionailurus bengalensis NC028301 12	Phataginus tricuspis NC026780	0,2263739
Prionailurus bengalensis CKM45 20	Phataginus tricuspis NC026780	0,22657189
Viverricula indica KX891745 1	Phataginus tricuspis NC026780	0,22662733
Hydrurga leptonyx NC008425 1	Phataginus tricuspis NC026780	0,22670805
Viverricula indica KX891751 1	Phataginus tricuspis NC026780	0,22697055
Profelis aurata NC028299 1	Phataginus tricuspis NC026780	0,22705185
Prionailurus viverrinus NC028305 1	Phataginus tricuspis NC026780	0,22711936
Monachus monachus NC044972 5	Phataginus tricuspis NC026780	0,22713321
Genetta servalina NC024568 2	Phataginus tricuspis NC026780	0,2273033
Acinonyx jubatus NC005212 3	Phataginus tricuspis NC026780	0,22747581
Viverra zibetha T609 1	Phataginus tricuspis NC026780	0,22750384
Mungos mungo MMC7 1	Phataginus tricuspis NC026780	0,22771791
Leopardus tigrinus NC028317 1	Phataginus tricuspis NC026780	0,22773673
Prionailurus rubiginosus NC028304 2	Phataginus tricuspis NC026780	0,22784302
Paradoxurus jerdoni MH464793 1	Phataginus tricuspis NC026780	0,22804511
Viverricula indica NC025296 2	Phataginus tricuspis NC026780	0,22804868
Paradoxurus hermaphroditus NLNC 1	Phataginus tricuspis NC026780	0,22811289
Lobodon carcinophaga NC008423 1	Phataginus tricuspis NC026780	0,22812387
Caracal caracal NC028306 1	Phataginus tricuspis NC026780	0,2281992
Civettictis civetta GLC19 1	Phataginus tricuspis NC026780	0,22824191
Smilodon populator MF871700 1	Phataginus tricuspis NC026780	0,22826028
Catopuma temminckii NC027115 41	Phataginus tricuspis NC026780	0,22828029
Diplogale hosei MH464790 1	Phataginus tricuspis NC026780	0,22847393
Prionailurus planiceps KY682741 4	Phataginus tricuspis NC026780	0,22852986
Leopardus geoffroyi NC028320 1	Phataginus tricuspis NC026780	0,22862007
Paguma larvata PDDS11 2	Phataginus tricuspis NC026780	0,22872204
Civettictis civetta NC033378 1	Phataginus tricuspis NC026780	0,22883071
Erignathus barbatus NC008426 1	Phataginus tricuspis NC026780	0,22891891
Nandinia binotata NC024567 1	Phataginus tricuspis NC026780	0,22892016
Prionodon linsang ERR2391707 1	Phataginus tricuspis NC026780	0,22896336
Otocolobus manul NC028323 1	Phataginus tricuspis NC026780	0,22900976
Lynx lynx NC027083 4	Phataginus tricuspis NC026780	0,22927967
Prionodon pardicolor NC024569 2	Phataginus tricuspis NC026780	0,2293327
Cystophora cristata NC008427 1	Phataginus tricuspis NC026780	0,22951427
Felis chaus NC028307 1	Phataginus tricuspis NC026780	0,22955212
Felis margarita NC028308 1	Phataginus tricuspis NC026780	0,22969061
Lynx canadensis NC028313 1	Phataginus tricuspis NC026780	0,22969319
Conepatus chinga NC042596 1	Phataginus tricuspis NC026780	0,22972238
Leptonychotes weddellii NC008424 1	Phataginus tricuspis NC026780	0,22973184
Cynogale bennetti KY117544 1	Phataginus tricuspis NC026780	0,22973245
Leopardus pardalis NC028315 1	Phataginus tricuspis NC026780	0,22975038
Genetta genetta T297 1	Phataginus tricuspis NC026780	0,22979817
Crossarchus platycephalus C7R66 1	Phataginus tricuspis NC026780	0,22987133
Lynx pardinus NC028319 161	Phataginus tricuspis NC026780	0,22988592
Leopardus colocolo NC028314 1	Phataginus tricuspis NC026780	0,22998403
Prionailurus planiceps NC028312 6	Phataginus tricuspis NC026780	0,23001347
Felis silvestris lybica KP202275 4	Phataginus tricuspis NC026780	0,23009634
Felis nigripes NC028309 1	Phataginus tricuspis NC026780	0,23015328
Paradoxurus hermaphroditus NC03959	Phataginus tricuspis NC026780	0,23040506
Leopardus pardalis T262 1	Phataginus tricuspis NC026780	0,23049231
Urva javanica T413 1	Phataginus tricuspis NC026780	0,23052634
Phoca fasciata NC008428 1	Phataginus tricuspis NC026780	0,23060836
Monachus schauinslandi NC008421 1	Phataginus tricuspis NC026780	0,23070791
Pusa hispida NC 008433 1	Phataginus tricuspis NC026780	0,23073904
Chrotogale owstoni T607 1	Phataginus tricuspis NC026780	0,23086578
Felis catus NC001700 2	Phataginus tricuspis NC026780	0,23104022
Leopardus guigna NC028321 1	Phataginus tricuspis NC026780	0,23104829
Leopardus wiedii NC028318 1	Phataginus tricuspis NC026780	0,23116595
Cynictis penicillata T375 1	Phataginus tricuspis NC026780	0,23135829
Mirounga angustirostris SRR10331586	Phataginus tricuspis NC026780	0,23151457
Fossa fossana D350 1	Phataginus tricuspis NC026780	0,2316113
Arctonyx collaris NC020645 1	Phataginus tricuspis NC026780	0,23163065

Martes foina NC020643 1	Caracal caracal NC028306 1	0,18141642
Martes flavigula NC012141 3	Genetta abyssinica MG489822 1	0,18141803
Ursus spelaeus EU327344 13	Arctocephalus gazella BK010918 1	0,18141949
Ursus spelaeus EU327344 13	Mustela putorius NC020638 4	0,18142134
Leopardus wiedii NC028318 1	Chrysocyon brachyurus NC024172 1	0,18142685
Mustela itatsi NC034330 19	Canis lupus familiaris NC002008 1231	0,18142813
Ursus spelaeus EU327344 13	Martes foina NC020643 1	0,18142913
Vulpes lagopus NC026529 3	Civettictis civetta NC033378 1	0,18143375
Chrysocyon brachyurus NC024172 1	Arctictis binturong T605 2	0,18143468
Martes zibellina NC011579 39	Civettictis civetta NC033378 1	0,18143924
Prionailurus bengalensis NC028301 12	Gulo gulo NC009685 3	0,1814414
Martes flavigula NC012141 3	Leopardus pardalis T262 1	0,18144384
Nasua nasua NC020647 1	Mustela sibirica NC020637 6	0,18145093
Nasua nasua NC020647 1	Melogale moschata KP726273 1	0,18145235
Cuon alpinus NC013445 3	Catopuma badia NC028300 1	0,18145248
Phocarctos hookeri NC008418 1	Arctotherium sp NC030174 1	0,18145405
Salanoia concolor D378 1	Melogale moschata KP726273 1	0,18145777
Zalophus californianus NC008416 1	Canis adustus KT448271 1	0,18145883
Otocolobus manul NC028323 1	Enhydra lutris NC009692 1	0,18146309
Meles leucurus NC039173 4	Felis catus NC001700 2	0,18146446
Ursus thibetanus mupinensis NC008753 2	Martes melampus NC009678 1	0,181465
Meles meles T303 3	Felis chaus NC028307 1	0,18146569
Profelis aurata NC028299 1	Mustela frenata NC020640 1	0,18146624
Mustela kathiah NC023210 1	Lynx pardinus NC028319 161	0,18146639
Lynx pardinus NC028319 161	Canis aureus KT448274 1	0,1814664
Otaria byronia OTAB 1	Melogale moschata NC020644 1	0,18146695
Prionailurus planiceps NC028312 6	Mustela sibirica AP017394 11	0,18146826
Puma concolor NC016470 22	Mustela putorius NC020638 4	0,18146831
Neophoca cinerea NC008419 1	Martes foina NC020643 1	0,18146847
Puma concolor NC016470 22	Mustela itatsi NC034330 19	0,18146849
Paradoxurus hermaphroditus NLNC 1	Mustela itatsi NC034330 19	0,18146861
Neophoca cinerea NC008419 1	Mustela altaica NC021751 1	0,18146862
Paradoxurus hermaphroditus NC039591 1	Mustela kathiah NC023210 1	0,18146886
Smilodon populator MF871700 1	Canis lupus chanco NC010340 4	0,18146886
Vulpes lagopus NC026529 3	Viverricula indica NC025296 2	0,18146928
Prionailurus viverrinus NC028305 1	Mustela putorius NC020638 4	0,18146951
Meles anakuma NC009677 1	Arctodus simus NC011116 1	0,18147011
Mustela sibirica NC020637 6	Catopuma badia NC028300 1	0,18147044
Speothos venaticus C48 2	Mirounga leonina NC008422 1	0,18147096
Vulpes vulpes NC008434 5	Prionailurus bengalensis NC028301 12	0,18147099
Mustela nigripes NC024942 1	Leopardus guigna NC028321 1	0,18147114
Viverra tangalunga MH464792 1	Leopardus guigna NC028321 1	0,18147114
Mustela itatsi NC034330 19	Catopuma temminckii NC027115 41	0,18147127
Phoca vitulina NC001325 1	Nyctereutes procyonoides NC013700 3	0,18147226
Prionailurus bengalensis CKM45 20	Mustela eversmannii NC028013 1	0,18147287
Viverricula indica NC025296 2	Arctotherium sp NC030174 1	0,18147418
Ursus maritimus GU573488 Svalbard	Arctocephalus pusillus NC008417 1	0,18147425
Procyon lotor AB462046 3	Paguma larvata PDDS11 2	0,18147431
Pusa caspica NC008431 1	Prionodon pardicolor NC024569 2	0,18147437
Pusa caspica NC008431 1	Diplogale hosei MH464790 1	0,18147472
Procyon lotor AB462049 4	Prionailurus rubiginosus NC028304 2	0,18147555
Genetta genetta T297 1	Arctodus simus NC011116 1	0,18147792
Leopardus pardalis T262 1	Aonyx cinerea NC035814 2	0,1814782
Enhydra lutris NC009692 1	Acinonyx jubatus NC005212 3	0,18147932
Pusa sibirica NC008432 2	Canis mesomelas KT448280 1	0,18147965
Homotherium latidens MF871702 3	Canis mesomelas KT448280 1	0,18148139
Ommatophoca rossii AY377287etc 1	Canis aureus KT448274 1	0,18148184
Ursus thibetanus laniger MH281753 2	Arctocephalus forsteri KT693377 17	0,18148313
Leopardus guigna NC028321 1	Arctodus simus NC011116 1	0,18148386
Mungotictis decemlineata NC027828 1	Canis adustus KT448271 1	0,18148419
Zalophus wollebaeki SRR4431565 1	Ictonyx striatus T299 1	0,18148564
Vulpes vulpes NC008434 5	Acinonyx jubatus NC005212 3	0,18148582
Martes zibellina NC011579 39	Acinonyx jubatus NC005212 3	0,18148778
Meles anakuma NC009677 1	Eumetopias jubatus NC004030 10	0,1814907
Ursus thibetanus formosanus NC009331 1	Leopardus pardalis T262 1	0,18149248
Panthera tigris NC010642 35	Canis adustus KT448271 1	0,18149295
Ursus thibetanus thibetanus NC011118 4	Leopardus wiedii NC028318 1	0,18149441
Viverricula indica NC025296 2	Martes flavigula NC012141 3	0,18150273
Spilogale putorius NC010497 1	Martes americana NC020642 1	0,18150727
Zalophus wollebaeki SRR4431565 1	Ursus americanus JX196366 3	0,18151204
Parahyaena brunnea NC038159 15	Monachus monachus NC044972 5	0,18151303
Lynx rufus NC014456 3	Eumetopias jubatus NC004030 10	0,18151498
Mustela nivalis T306 5	Civettictis civetta NC033378 1	0,18151565
Martes flavigula NC012141 3	Canis aureus KT448274 1	0,18151763
Nandinia binotata NC024567 1	Arctocephalus townsendi NC008420 1	0,18152186
Salanoia concolor D378 1	Martes melampus NC009678 1	0,18152219
Tremarctos ornatus NC009969 2	Lutra lutra NC011358 9	0,18152243
Mustela eversmannii NC028013 1	Conepatus chinga NC042596 1	0,18152259
Tremarctos ornatus NC009969 2	Arctocephalus forsteri KT693377 17	0,18152502
Vulpes ferrilata NC027935 1	Cystophora cristata NC008427 1	0,18152513
Viverricula indica NC025296 2	Lutra lutra LC050126 1	0,18152633
Mustela nivalis T306 5	Callorhinus ursinus NC008415 1	0,18152907
Helarctos malayanus NC009968 2	Gulo gulo NC009685 3	0,18153112
Prionailurus rubiginosus NC028304 2	Meles anakuma NC009677 1	0,18153222
Ursus maritimus GU573488 Svalbard	Callorhinus ursinus NC008415 1	0,18153255
Meles leucurus NC039173 4	Caracal caracal NC028306 1	0,18153274
Viverra zibetha T609 1	Martes zibellina NC011579 39	0,18153282
Paradoxurus hermaphroditus NC039591 1	Cystophora cristata NC008427 1	0,18153323
Pusa hispida NC 008433 1	Prionodon linsang ERR2391707 1	0,1815334
Lynx rufus NC014456 3	Callorhinus ursinus NC008415 1	0,18153366
Martes zibellina NC011579 39	Civettictis civetta GLC19 1	0,18153366
Paradoxurus hermaphroditus NC039591 1	Meles meles T303 3	0,18153369
Vulpes ferrilata NC027935 1	Phoca fasciata NC008428 1	0,18153377
Vulpes lagopus NC026529 3	Civettictis civetta GLC19 1	0,18153416
Prionailurus planiceps NC028312 6	Martes melampus NC009678 1	0,1815342
Prionailurus bengalensis NC028301 12	Ailurus fulgens NC011124 1	0,18153459
Martes melampus NC009678 1	Leptailurus serval NC028316 1	0,1815349
Martes melampus NC009678 1	Leopardus jacobita NC028322 1	0,18153504
Tremarctos ornatus NC009969 2	Lynx lynx NC027083 4	0,18153533
Ursus maritimus NC003428 31	Melogale moschata KP726273 1	0,18153554
Prionailurus planiceps KY682741 4	Mustela itatsi NC034330 19	0,18153591
Tremarctos ornatus NC009969 2	Prionailurus rubiginosus NC028304 2	0,18153685
Vulpes vulpes NC008434 5	Leopardus pardalis NC028315 1	0,18153739

Neovison vison NC020641 3	Phataginus tricuspis NC026780	0,23169054
Phoca largha NC008430 1	Phataginus tricuspis NC026780	0,23169094
Leopardus jacobita NC028322 1	Phataginus tricuspis NC026780	0,2317052
Lynx rufus NC014456 3	Phataginus tricuspis NC026780	0,23178078
Mirounga leonina NC008422 1	Phataginus tricuspis NC026780	0,23182197
Urva javanica/auropunctata NC00683	Phataginus tricuspis NC026780	0,23185465
Pardofelis marmorata NLN3 2	Phataginus tricuspis NC026780	0,23191461
Urva brachyura KY117547 1	Phataginus tricuspis NC026780	0,23194511
Panthera leo NERO 19	Phataginus tricuspis NC026780	0,2321237
Phoca groenlandica NC008429 54	Phataginus tricuspis NC026780	0,23215741
Pusa caspica NC008431 1	Phataginus tricuspis NC026780	0,23254527
Mungos mungo/gambianus SRR77048	Phataginus tricuspis NC026780	0,23256481
Panthera pardus japonensis KJ866876	Phataginus tricuspis NC026780	0,23269585
Meles leucurus NC039173 4	Phataginus tricuspis NC026780	0,23282832
Genetta abyssinica MG489822 1	Phataginus tricuspis NC026780	0,23289323
Pusa sibirica NC008432 2	Phataginus tricuspis NC026780	0,23294963
Mephitis mephitis NC020648 1	Phataginus tricuspis NC026780	0,23297364
Panthera onca NC022842 1	Phataginus tricuspis NC026780	0,23312454
Mustela putorius NC020638 4	Phataginus tricuspis NC026780	0,23329078
Helogale parvula SRR7637809 1	Phataginus tricuspis NC026780	0,23329754
Arctocephalus pusillus NC008417 1	Phataginus tricuspis NC026780	0,23330015
Panthera onca KP202264 2	Phataginus tricuspis NC026780	0,23337896
Panthera leo spelaea KX258452 2	Phataginus tricuspis NC026780	0,23339835
Phoca vitulina NC001325 1	Phataginus tricuspis NC026780	0,23344192
Mustela sibirica AP017394 11	Phataginus tricuspis NC026780	0,23349933
Panthera uncia KP202269 1	Phataginus tricuspis NC026780	0,23352221
Meles meles T303 3	Phataginus tricuspis NC026780	0,23364335
Panthera pardus NC010641 5	Phataginus tricuspis NC026780	0,23369081
Meles anakuma NC009677 1	Phataginus tricuspis NC026780	0,23384666
Suricata suricatta SSM10 1	Phataginus tricuspis NC026780	0,23389666
Mustela nigripes NC024942 1	Phataginus tricuspis NC026780	0,23390527
Taxidea taxus NC020646 1	Phataginus tricuspis NC026780	0,23404311
Galerella sanguinea T378 1	Phataginus tricuspis NC026780	0,23419622
Urva semitorquata MH464789 1	Phataginus tricuspis NC026780	0,23425217
Callorhinus ursinus NC008415 1	Phataginus tricuspis NC026780	0,23430745
Mustela eversmannii NC028013 1	Phataginus tricuspis NC026780	0,23436806
Mustela kathiah NC023210 1	Phataginus tricuspis NC026780	0,23437041
Ailurus fulgens NC011124 1	Phataginus tricuspis NC026780	0,23438202
Panthera tigris amoyensis NC014770 2	Phataginus tricuspis NC026780	0,23446015
Mustela frenata NC020640 1	Phataginus tricuspis NC026780	0,23450702
Ailurus fulgens styani NC009691 1	Phataginus tricuspis NC026780	0,23451717
Panthera uncia NC010638 1	Phataginus tricuspis NC026780	0,23459272
Atilax paludinosus T606 1	Phataginus tricuspis NC026780	0,23459801
Mustela erminea T305 2	Phataginus tricuspis NC026780	0,23478611
Ichneumia albicauda T603 1	Phataginus tricuspis NC026780	0,23480691
Mustela sibirica NC020637 6	Phataginus tricuspis NC026780	0,23491561
Halichoerus grypus NC001602 2	Phataginus tricuspis NC026780	0,23497161
Poecilogale albinucha T602 1	Phataginus tricuspis NC026780	0,23498987
Canis latrans NC008093 7	Phataginus tricuspis NC026780	0,23532021
Spilogale putorius NC010497 1	Phataginus tricuspis NC026780	0,23535977
Viverra tangalunga MH464792 1	Phataginus tricuspis NC026780	0,23539075
Ommatophoca rossii AY377287etc 1	Phataginus tricuspis NC026780	0,23542574
Canis aureus KT448274 1	Phataginus tricuspis NC026780	0,23552668
Xenogale naso C07XAR110 1	Phataginus tricuspis NC026780	0,23566486
Panthera tigris NC010642 35	Phataginus tricuspis NC026780	0,23567367
Canis lupus familiaris NC002008 1231	Phataginus tricuspis NC026780	0,23570728
Bdeogale nigripes GLC15 1	Phataginus tricuspis NC026780	0,23585761
Chrysocyon brachyurus NC024172 1	Phataginus tricuspis NC026780	0,23595589
Zalophus californianus NC008416 1	Phataginus tricuspis NC026780	0,23609482
Lycaon pictus NC028427 2	Phataginus tricuspis NC026780	0,23616007
Mustela itatsi NC034330 19	Phataginus tricuspis NC026780	0,23633914
Canis lupus chanco NC010340 4	Phataginus tricuspis NC026780	0,23652382
Zalophus wollebaeki SRR4431565 1	Phataginus tricuspis NC026780	0,23695782
Salanoia concolor D378 1	Phataginus tricuspis NC026780	0,23703909
Arctocephalus townsendi NC008420 1	Phataginus tricuspis NC026780	0,23706768
Enhydra lutris NC009692 1	Phataginus tricuspis NC026780	0,23707823
Ailuropoda melanoleuca NC009492 5	Phataginus tricuspis NC026780	0,23714823
Galidictis fasciata DM333 1	Phataginus tricuspis NC026780	0,23758119
Cuon alpinus NC013445 3	Phataginus tricuspis NC026780	0,23760675
Canis anthus NC027956 2	Phataginus tricuspis NC026780	0,23775092
Ursus spelaeus NC011112 8	Phataginus tricuspis NC026780	0,23778547
Ursus spelaeus EU327344 13	Phataginus tricuspis NC026780	0,23784611
Hyaena hyaena NC020669 1	Phataginus tricuspis NC026780	0,23787299
Eumetopias jubatus NC004030 10	Phataginus tricuspis NC026780	0,23788528
Arctocephalus gazella BK010918 1	Phataginus tricuspis NC026780	0,23799318
Neofelis nebulosa NC008450 3	Phataginus tricuspis NC026780	0,23806116
Mungotictis decemlineata NC027828 1	Phataginus tricuspis NC026780	0,23811679
Mustela altaica NC021751 1	Phataginus tricuspis NC026780	0,23814917
Neophoca cinerea NC008419 1	Phataginus tricuspis NC026780	0,23826796
Lycalopex sechurae KT448284 1	Phataginus tricuspis NC026780	0,23833352
Bassariscus sumichrasti SRX1099089 1	Phataginus tricuspis NC026780	0,23836312
Nyctereutes procyonoides NC013700 3	Phataginus tricuspis NC026780	0,23840371
Arctocephalus forsteri NC004023 28	Phataginus tricuspis NC026780	0,23860008
Otaria byronia OTAB 1	Phataginus tricuspis NC026780	0,2386864
Arctodus simus NC011116 1	Phataginus tricuspis NC026780	0,23876552
Helarctos malayanus NC009968 2	Phataginus tricuspis NC026780	0,2387846
Arctocephalus australis MG023139 1	Phataginus tricuspis NC026780	0,23879835
Canis mesomelas KT448280 1	Phataginus tricuspis NC026780	0,23881981
Arctocephalus forsteri KT693377 17	Phataginus tricuspis NC026780	0,23886606
Ictonyx striatus T299 1	Phataginus tricuspis NC026780	0,23891455
Martes foina NC020643 1	Phataginus tricuspis NC026780	0,23891528
Proteles cristata T393 6	Phataginus tricuspis NC026780	0,23909795
Ursus thibetanus mupinensis NC00875	Phataginus tricuspis NC026780	0,23931569
Martes melampus NC009678 1	Phataginus tricuspis NC026780	0,23942886
Mustela nivalis T306 5	Phataginus tricuspis NC026780	0,23943609
Galictis vittata T412 1	Phataginus tricuspis NC026780	0,23952359
Eupleres goudotii D128 1	Phataginus tricuspis NC026780	0,23956028
Martes flavigula NC012141 3	Phataginus tricuspis NC026780	0,23970325
Ursus thibetanus laniger MH281753 2	Phataginus tricuspis NC026780	0,23973209
Parahyaena brunnea NC038159 15	Phataginus tricuspis NC026780	0,23976971
Lutra lutra LC050126 1	Phataginus tricuspis NC026780	0,23977236
Ursus thibetanus thibetanus NC01111	Phataginus tricuspis NC026780	0,23984703
Ursus maritimus GU573488 Svalbard	Phataginus tricuspis NC026780	0,23990734

Paguma larvata PDDS11 2	Canis latrans NC008093 7	0,18153793
Paradoxurus jerdoni MH464793 1	Canis anthus NC027956 2	0,18153864
Vulpes corsac NC023958 1	Monachus schauinslandi NC008421 1	0,18153967
Viverra zibetha T609 1	Mustela nivalis T306 5	0,18154421
Panthera uncia KP202269 1	Mirounga angustirostris SRR10331586 1	0,18154585
Ursus thibetanus laniger MH281753 2	Enhydra lutris NC009692 1	0,18154687
Poecilogale albinucha T602 1	Leopardus pardalis NC028315 1	0,18154688
Viverra zibetha T609 1	Ursus thibetanus mupinensis NC008753 2	0,18154718
Prionailurus viverrinus NC028305 1	Arctodus simus NC011116 1	0,18154784
Pusa sibirica NC008432 2	Arctictis binturong T605 2	0,18154882
Otocolobus manul NC028323 1	Martes foina NC020643 1	0,18155149
Felis silvestris lybica KP202275 4	Canis aureus KT448274 1	0,18155167
Prionailurus rubiginosus NC028304 2	Lycaon pictus NC028427 2	0,18155356
Martes pennanti NC020664 16	Arctocephalus forsteri NC004023 28	0,18155444
Ursus thibetanus formosanus NC009331 1	Civettictis civetta GLC19 1	0,18155453
Ursus thibetanus laniger MH281753 2	Ailurus fulgens NC011124 1	0,18155469
Ursus thibetanus laniger MH281753 2	Bassariscus sumichrasti SRX1099089 1	0,18155481
Ursus spelaeus EU327344 13	Mustela eversmannii NC028013 1	0,18155551
Ursus maritimus GU573488 Svalbard	Neovison vison NC020641 3	0,18155794
Viverricula indica NC025296 2	Helarctos malayanus NC009968 2	0,1815609
Zalophus californianus NC008416 1	Ictonyx striatus T299 1	0,18156688
Lycalopex sechurae KT448284 1	Catopuma temminckii NC027115 41	0,18156706
Smilodon populator MF871700 1	Martes foina NC020643 1	0,18156937
Felis margarita NC028308 1	Canis lupus familiaris NC002008 1231	0,18157198
Ursus americanus JX196366 3	Neophoca cinerea NC008419 1	0,18157323
Viverra tangalunga MH464792 1	Civettictis civetta NC033378 1	0,18157856
Galidictis fasciata DM333 1	Canis adustus KT448271 1	0,18157941
Ursus arctos isabellinus 18B5 2	Homotherium latidens MF871702 3	0,18157965
Civettictis civetta NC033378 1	Arctotherium sp NC030174 1	0,1815799
Martes flavigula NC012141 3	Leopardus guigna NC028321 1	0,18158133
Arctocephalus townsendi NC008420 1	Ailurus fulgens styani NC009691 1	0,18158709
Leopardus colocolo NC028314 1	Canis lupus familiaris NC002008 1231	0,1815889
Neophoca cinerea NC008419 1	Meles leucurus NC039173 4	0,18158907
Prionodon linsang ERR2391707 1	Mustela frenata NC020640 1	0,18158916
Phocarctos hookeri NC008418 1	Martes americana NC020642 1	0,18158965
Lutra lutra NC011358 9	Genetta servalina NC024568 2	0,18159127
Vulpes zerda KJ603240 1	Erignathus barbatus NC008426 1	0,18159403
Paradoxurus jerdoni MH464793 1	Canis mesomelas KT448280 1	0,18159406
Mustela eversmannii NC028013 1	Arctocephalus pusillus NC008417 1	0,18159422
Viverricula indica NC025296 2	Enhydra lutris NC009692 1	0,1815946
Taxidea taxus NC020646 1	Urva javanica T413 1	0,18159509
Fossa fossana D350 1	Arctocephalus pusillus NC008417 1	0,18159516
Martes flavigula NC012141 3	Canis lupus familiaris NC002008 1231	0,18159594
Prionailurus planiceps NC028312 6	Lutra lutra LC050126 1	0,18159825
Meles anakuma NC009677 1	Leopardus tigrinus NC028317 1	0,1815984
Leopardus pardalis T262 1	Arctonyx collaris NC020645 1	0,18159841
Paradoxurus hermaphroditus NC039591 1	Meles anakuma NC009677 1	0,18159923
Poecilogale albinucha T602 1	Arctocephalus forsteri KT693377 17	0,1815996
Meles meles T303 3	Caracal caracal NC028306 1	0,18160053
Meles anakuma NC009677 1	Arctotherium sp NC030174 1	0,18160143
Mustela itatsi NC034330 19	Lynx rufus NC014456 3	0,18160219
Paradoxurus hermaphroditus NLNC 1	Mustela sibirica AP017394 11	0,18160245
Mustela itatsi NC034330 19	Leopardus pardalis NC028315 1	0,18160246
Otocolobus manul NC028323 1	Mustela itatsi NC034330 19	0,18160431
Melogale moschata KP726273 1	Leopardus tigrinus NC028317 1	0,18160477
Paguma larvata PDDS11 2	Ailurus fulgens NC011124 1	0,18160494
Viverricula indica NC025296 2	Mustela nigripes NC024942 1	0,18160506
Otaria byronia OTAB 1	Martes foina NC020643 1	0,18160561
Melogale moschata NC020644 1	Genetta servalina NC024568 2	0,18160573
Vulpes zerda KJ603240 1	Leopardus pardalis T262 1	0,18160616
Prionodon linsang ERR2391707 1	Mirounga angustirostris SRR10331586 1	0,1816079
Vulpes lagopus NC026529 3	Prionailurus bengalensis CKM45 20	0,1816086
Viverricula indica NC025296 2	Mustela altaica NC021751 1	0,181609
Homotherium latidens MF871702 3	Canis anthus NC027956 2	0,18161042
Viverricula indica NC025296 2	Procyon lotor AB462046 3	0,18161045
Prionodon pardicolor NC024569 2	Phoca fasciata NC008428 1	0,18161121
Otocolobus manul NC028323 1	Neovison vison NC020641 3	0,18161678
Lynx pardinus NC028319 161	Ictonyx striatus T299 1	0,18161745
Otocolobus manul NC028323 1	Bassariscus sumichrasti SRX1099089 1	0,18161856
Lycalopex sechurae KT448284 1	Leptailurus serval NC028316 1	0,18162012
Homotherium latidens MF871702 3	Canis aureus KT448274 1	0,18162045
Meles meles T303 3	Canis lupus familiaris NC002008 1231	0,18162622
Zalophus californianus NC008416 1	Ursus maritimus GU573488 Svalbard	0,18162808
Procyon lotor AB462046 3	Mephitis mephitis NC020648 1	0,18162888
Martes martes T302 3	Leopardus colocolo NC028314 1	0,181629
Mustela nivalis T306 5	Acinonyx jubatus NC005212 3	0,18162923
Chrysocyon brachyurus NC024172 1	Catopuma badia NC028300 1	0,18163036
Helarctos malayanus NC009968 2	Canis adustus KT448271 1	0,1816328
Zalophus californianus NC008416 1	Poecilogale albinucha T602 1	0,18163313
Hemigalus derbyanus MH464791 1	Tapirus terrestris T358	0,18163393
Leopardus wiedii NC028318 1	Canis lupus familiaris NC002008 1231	0,18163769
Mustela sibirica AP017394 11	Civettictis civetta NC033378 1	0,18164165
Prionailurus bengalensis NC028301 12	Martes flavigula NC012141 3	0,18164369
Panthera tigris amoyensis NC014770 2	Monachus monachus NC044972 5	0,18164547
Smilodon populator MF871700 1	Helarctos malayanus NC009968 2	0,18164741
Monachus schauinslandi NC008421 1	Cuon alpinus NC013445 3	0,1816505
Nasua nasua NC020647 1	Lutra lutra NC011358 9	0,18165062
Diplogale hosei MH464790 1	Ailurus fulgens styani NC009691 1	0,18165265
Viverra tangalunga MH464792 1	Callorhinus ursinus NC008415 1	0,18165304
Melogale moschata KP726273 1	Arctocephalus pusillus NC008417 1	0,18165998
Vulpes corsac NC023958 1	Mustela frenata NC020640 1	0,18166138
Potos flavus T414 1	Mungos mungo MMC7 1	0,18166147
Ommatophoca rossii AY377287etc 1	Galidictis fasciata DM333 1	0,18166335
Salanoia concolor D378 1	Mustela erminea T305 2	0,18166386
Martes flavigula NC012141 3	Leopardus colocolo NC028314 1	0,18166433
Mungos mungo MMC7 1	Arctocephalus pusillus NC008417 1	0,18166478
Canis latrans NC008093 7	Arctonyx collaris NC020645 1	0,18166588
Melogale moschata NC020644 1	Arctocephalus pusillus NC008417 1	0,18166608
Vulpes vulpes NC008434 5	Phoca groenlandica NC008429 54	0,18166773
Procyon lotor AB462049 4	Ailurus fulgens NC011124 1	0,18166837
Urocyon cinereoargenteus NC026723 21	Phoca fasciata NC008428 1	0,18166895
Urocyon littoralis catalinae KP129018 15	Phoca fasciata NC008428 1	0,18166898
Leptailurus serval NC028316 1	Ailurus fulgens NC011124 1	0,18166989

Ursus arctos GU573486 5	Phataginus tricuspis NC026780	0,239921
Ursus thibetanus formosanus NC0093[illegible]	Phataginus tricuspis NC026780	0,24006689
Ursus arctos EU497665 29	Phataginus tricuspis NC026780	0,24010971
Ursus arctos GU573491 207	Phataginus tricuspis NC026780	0,24011619
Ursus arctos AP012576 6	Phataginus tricuspis NC026780	0,2401271
Ursus maritimus NC003428 31	Phataginus tricuspis NC026780	0,24017687
Galidia elegans D146 1	Phataginus tricuspis NC026780	0,2401855
Potos flavus T414 1	Phataginus tricuspis NC026780	0,2403632
Phocarctos hookeri NC008418 1	Phataginus tricuspis NC026780	0,24036369
Arctotherium sp NC030174 1	Phataginus tricuspis NC026780	0,24036835
Lontra canadensis SRR10409165 1	Phataginus tricuspis NC026780	0,24040549
Gulo gulo NC009685 3	Phataginus tricuspis NC026780	0,2404274
Procyon lotor AB462046 3	Phataginus tricuspis NC026780	0,24044034
Ursus arctos isabellinus 18B5 2	Phataginus tricuspis NC026780	0,24044681
Martes americana NC020642 1	Phataginus tricuspis NC026780	0,24051051
Ursus arctos pruinosus MG066703 3	Phataginus tricuspis NC026780	0,24052152
Ursus americanus JX196366 3	Phataginus tricuspis NC026780	0,24069238
Melogale moschata NC020644 1	Phataginus tricuspis NC026780	0,24094579
Lutra lutra NC011358 9	Phataginus tricuspis NC026780	0,24098483
Martes martes T302 3	Phataginus tricuspis NC026780	0,24106155
Odobenus rosmarus NC004029 29	Phataginus tricuspis NC026780	0,24111831
Procyon lotor AB462049 4	Phataginus tricuspis NC026780	0,24124756
Vulpes vulpes NC008434 5	Phataginus tricuspis NC026780	0,241355
Vulpes ferrilata NC027935 1	Phataginus tricuspis NC026780	0,24143265
Lutra sumatrana NC035810 1	Phataginus tricuspis NC026780	0,24153519
Melogale moschata V0735A 1	Phataginus tricuspis NC026780	0,24159856
Martes zibellina NC011579 39	Phataginus tricuspis NC026780	0,24179113
Melogale moschata KP726273 1	Phataginus tricuspis NC026780	0,24185903
Vulpes corsac NC023958 1	Phataginus tricuspis NC026780	0,24190119
Otocyon megalotis SAF1 2	Phataginus tricuspis NC026780	0,24253331
Crocuta crocuta NC020670 3	Phataginus tricuspis NC026780	0,24254262
Tremarctos ornatus NC009969 2	Phataginus tricuspis NC026780	0,24259415
Cryptoprocta ferox CFC13 1	Phataginus tricuspis NC026780	0,24269819
Martes pennanti NC020664 16	Phataginus tricuspis NC026780	0,24320097
Vulpes zerda KJ603240 1	Phataginus tricuspis NC026780	0,24339955
Speothos venaticus C48 2	Phataginus tricuspis NC026780	0,24389814
Urocyon littoralis catalinae KP129018 [illegible]	Phataginus tricuspis NC026780	0,24406604
Urocyon cinereoargenteus NC026723 [illegible]	Phataginus tricuspis NC026780	0,24420853
Vulpes lagopus NC026529 3	Phataginus tricuspis NC026780	0,24508271
Aonyx cinerea NC035814 2	Phataginus tricuspis NC026780	0,24578962
Bassaricyon neblina SRX1097850 1	Phataginus tricuspis NC026780	0,24601918
Melursus ursinus NC009970 2	Phataginus tricuspis NC026780	0,24627002
Nasua nasua NC020647 1	Phataginus tricuspis NC026780	0,24691479
Lutrogale perspicillata NC035811 1	Phataginus tricuspis NC026780	0,24853124
Mellivora capensis T370 1	Phataginus tricuspis NC026780	0,25034422

Potos flavus T414 1	Lynx rufus NC014456 3	0,18167089
Viverra zibetha T609 1	Ailurus fulgens NC011124 1	0,18167141
Viverricula indica KX891751 1	Ailurus fulgens NC011124 1	0,18167184
Puma yagouaroundi NC028311 1	Potos flavus T414 1	0,18167195
Smilodon populator MF871700 1	Arctotherium sp NC030174 1	0,18167207
Panthera leo spelaea KX258452 2	Mirounga leonina NC008422 1	0,18167216
Zalophus wollebaeki SRR4431565 1	Mustela kathiah NC023210 1	0,1816723
Mustela putorius NC020638 4	Catopuma badia NC028300 1	0,18167257
Mustela putorius NC020638 4	Leopardus guigna NC028321 1	0,18167298
Puma yagouaroundi NC028311 1	Martes americana NC020642 1	0,18167299
Martes americana NC020642 1	Leopardus pardalis NC028315 1	0,18167309
Mephitis mephitis NC020648 1	Lutra sumatrana NC035810 1	0,18167385
Felis silvestris lybica KP202275 4	Canis latrans NC008093 7	0,18167445
Viverricula indica KX891745 1	Procyon lotor AB462046 3	0,18167509
Martes martes T302 3	Genetta servalina NC024568 2	0,18167537
Martes martes T302 3	Arctotherium sp NC030174 1	0,18167581
Ursus arctos GU573491 207	Arctocephalus pusillus NC008417 1	0,18167633
Ursus maritimus NC003428 31	Mustela nigripes NC024942 1	0,18167727
Ursus arctos pruinosus MG066703 3	Nandinia binotata NC024567 1	0,18167742
Mustela altaica NC021751 1	Leopardus pardalis NC028315 1	0,18167841
Martes martes T302 3	Leopardus guigna NC028321 1	0,1816788
Ursus americanus JX196366 3	Homotherium latidens MF871702 3	0,181679
Prionailurus bengalensis CKM45 20	Canis aureus KT448274 1	0,18167996
Prionailurus planiceps NC028312 6	Martes martes T302 3	0,18168038
Ursus thibetanus mupinensis NC008753 2	Genetta servalina NC024568 2	0,18168187
Ursus spelaeus NC011112 8	Martes zibellina NC011579 39	0,18168312
Lynx lynx NC027083 4	Ictonyx striatus T299 1	0,18168385
Phoca groenlandica NC008429 54	Parahyaena brunnea NC038159 15	0,18168461
Prionailurus planiceps NC028312 6	Ictonyx striatus T299 1	0,18168561
Neovison vison NC020641 3	Lycaon pictus NC028427 2	0,18168733
Zalophus wollebaeki SRR4431565 1	Ursus arctos GU573486 5	0,1816874
Prionailurus bengalensis NC028301 12	Lycaon pictus NC028427 2	0,18168895
Puma yagouaroundi NC028311 1	Lycaon pictus NC028427 2	0,18168963
Ursus spelaeus NC011112 8	Arctonyx collaris NC020645 1	0,18168996
Phoca largha NC008430 1	Canis aureus KT448274 1	0,1816906
Ursus arctos pruinosus MG066703 3	Ommatophoca rossii AY377287etc 1	0,18169065
Ursus spelaeus EU327344 13	Melogale moschata NC020644 1	0,18169102
Phoca groenlandica NC008429 54	Canis mesomelas KT448280 1	0,18169108
Viverricula indica NC025296 2	Ursus thibetanus formosanus NC009331 1	0,18169133
Urva semitorquata MH464789 1	Canis lupus familiaris NC002008 1231	0,18169267
Nandinia binotata NC024567 1	Arctocephalus forsteri NC004023 28	0,18169308
Ursus spelaeus NC011112 8	Martes foina NC020643 1	0,18169832
Panthera pardus NC010641 5	Mirounga leonina NC008422 1	0,18170099
Mustela eversmannii NC028013 1	Canis lupus chanco NC010340 4	0,18170296
Lycaon pictus NC028427 2	Acinonyx jubatus NC005212 3	0,18170407
Melogale moschata NC020644 1	Civettictis civetta NC033378 1	0,18170927
Genetta servalina NC024568 2	Canis lupus chanco NC010340 4	0,18170966
Viverricula indica KX891745 1	Eumetopias jubatus NC004030 10	0,1817109
