# Supplementary material for: Evolutionary history of Carnivora (Mammalia, Laurasiatheria) inferred from mitochondrial genomes
Source: PLoS One. 2021 Feb 16;16(2):e0240770. doi: 10.1371/journal.pone.0240770 (PMC7886153; doi:10.1371/journal.pone.0240770)
Supplement: S5 Appendix — (PDF) [file pone.0240770.s005.pdf]

S5 Appendix. Bayesian tree reconstructed using the *mtDNA-Tv* dataset (220 taxa and 14,892 bp) and JC69+I+G model

Evolutionary history of Carnivora (Mammalia, Laurasiatheria) inferred from mitochondrial genomes

Alexandre Hassanin[1*], Géraldine Veron[1], Anne Ropiquet[2], Bettine Jansen van Vuuren[3], Alexis Lécu[4], Steven M. Goodman[5], Jibran Haider[1,6,7], Trung Thanh Nguyen[1]

[1] Institut de Systématique, Évolution, Biodiversité (ISYEB), Sorbonne Université, MNHN, CNRS, EPHE, UA, Paris.

[2] Department of Natural Sciences, Faculty of Science and Technology, Middlesex University, United Kingdom.

[3] Centre for Ecological Genomics and Wildlife Conservation, Department of Zoology, University of Johannesburg, South Africa.

[4] Parc zoologique de Paris, Muséum national d'Histoire naturelle, Paris.

[5] Field Museum of Natural History, Chicago, IL, USA.

[6] Department of Wildlife Management, Pir Mehr Ali Shah, Arid Agriculture University Rawalpindi, Pakistan.

[7] Forest Parks & Wildlife Department Gilgit-Baltistan, Pakistan.

* Correspondence: alexandre.hassanin@mnhn.fr

```
#NEXUS
[ID: 2871343750]
begin taxa;
   dimensions ntax=220;
   taxlabels
            Tapirus_terrestris_T358
            Phataginus_tricuspis_NC026780
            Acinonyx_jubatus_NC005212_3
            Ailuropoda_melanoleuca_NC009492_5
            Ailurus_fulgens_NC011124_1
            Ailurus_fulgens_styani_NC009691_1
            Aonyx_cinerea_NC035814_2
            Arctictis_binturong_T605_2
            Arctocephalus_australis_MG023139_1
            Arctocephalus_forsteri_KT693377_17
            Arctocephalus_forsteri_NC004023_28
            Arctocephalus_gazella_BK010918_1
            Arctocephalus_pusillus_NC008417_1
            Arctocephalus_townsendi_NC008420_1
            Arctodus_simus_NC011116_1
            Arctonyx_collaris_NC020645_1
            Arctotherium_sp_NC030174_1
            Atilax_paludinosus_T606_1
            Bassaricyon_neblina_SRX1097850_1
            Bassariscus_sumichrasti_SRX1099089_1
            Bdeogale_nigripes_GLC15_1
            Callorhinus_ursinus_NC008415_1
            Canis_adustus_KT448271_1
            Canis_anthus_NC027956_2
            Canis_aureus_KT448274_1
            Canis_latrans_NC008093_7
            Canis_lupus_chanco_NC010340_4
            Canis_lupus_familiaris_NC002008_1231
            Canis_mesomelas_KT448280_1
            Caracal_caracal_NC028306_1
            Catopuma_badia_NC028300_1
            Catopuma_temminckii_NC027115_41
            Chrotogale_owstoni_T607_1
            Chrysocyon_brachyurus_NC024172_1
            Civettictis_civetta_GLC19_1
            Civettictis_civetta_NC033378_1
            Conepatus_chinga_NC042596_1
            Crocuta_crocuta_NC020670_3
            Crossarchus_platycephalus_C7AR66_1
            Cryptoprocta_ferox_CFC13_1
            Cuon_alpinus_NC013445_3
            Cynictis_penicillata_T375_1
            Cynogale_bennetti_KY117544_1
            Cystophora_cristata_NC008427_1
            Diplogale_hosei_MH464790_1
            Enhydra_lutris_NC009692_1
            Erignathus_barbatus_NC008426_1
            Eumetopias_jubatus_NC004030_10
            Eupleres_goudotii_D128_1
            Felis_catus_NC001700_2
            Felis_chaus_NC028307_1
            Felis_margarita_NC028308_1
            Felis_nigripes_NC028309_1
            Felis_silvestris_lybica_KP202275_4
            Fossa_fossana_D350_1
```

Galerella_sanguinea_T378_1
Galictis_vittata_T412_1
Galidia_elegans_D146_1
Galidictis_fasciata_DM333_1
Genetta_abyssinica_MG489822_1
Genetta_genetta_T297_1
Genetta_servalina_NC024568_2
Gulo_gulo_NC009685_3
Halichoerus_grypus_NC001602_2
Helarctos_malayanus_NC009968_2
Helogale_parvula_SRR7637809_1
Hemigalus_derbyanus_MH464791_1
Herpestes_brachyurus_KY117547_1
Herpestes_javanicus_NC006835_1
Herpestes_javanicus_T413_1
Herpestes_naso_C07XAR110_1
Herpestes_semitorquatus_MH464789_1
Homotherium_latidens_MF871702_3
Hyaena_hyaena_NC020669_1
Hydrurga_leptonyx_NC008425_1
Ichneumia_albicauda_T603_1
Ictonyx_striatus_T299_1
Leopardus_colocolo_NC028314_1
Leopardus_geoffroyi_NC028320_1
Leopardus_guigna_NC028321_1
Leopardus_jacobita_NC028322_1
Leopardus_pardalis_NC028315_1
Leopardus_pardalis_T262_1
Leopardus_tigrinus_NC028317_1
Leopardus_wiedii_NC028318_1
Leptailurus_serval_NC028316_1
Leptonychotes_weddellii_NC008424_1
Lobodon_carcinophaga_NC008423_1
Lontra_canadensis_SRR10409165_1
Lutra_lutra_LC050126_1
Lutra_lutra_NC011358_9
Lutra_sumatrana_NC035810_1
Lutrogale_perspicillata_NC035811_1
Lycalopex_sechurae_KT448284_1
Lycaon_pictus_NC028427_2
Lynx_canadensis_NC028313_1
Lynx_lynx_NC027083_4
Lynx_pardinus_NC028319_161
Lynx_rufus_NC014456_3
Martes_americana_NC020642_1
Martes_flavigula_NC012141_3
Martes_foina_NC020643_1
Martes_martes_T302_3
Martes_melampus_NC009678_1
Martes_pennanti_NC020664_16
Martes_zibellina_NC011579_39
Meles_anakuma_NC009677_1
Meles_leucurus_NC039173_4
Meles_meles_T303_3
Mellivora_capensis_T370_1
Melogale_moschata_KP726273_1
Melogale_moschata_NC020644_1
Melogale_moschata_V0735A_1
Melursus_ursinus_NC009970_2
Mephitis_mephitis_NC020648_1

Mirounga_angustirostris_SRR10331586_1
Mirounga_leonina_NC008422_1
Monachus_monachus_NC044972_5
Monachus_schauinslandi_NC008421_1
Mungos_mungo_MMC7_1
Mungos_mungo_SRR7704821_1
Mungotictis_decemlineata_NC027828_1
Mustela_altaica_NC021751_1
Mustela_erminea_T305_2
Mustela_eversmannii_NC028013_1
Mustela_frenata_NC020640_1
Mustela_itatsi_NC034330_19
Mustela_kathiah_NC023210_1
Mustela_nigripes_NC024942_1
Mustela_nivalis_T306_5
Mustela_putorius_NC020638_4
Mustela_sibirica_AP017394_11
Mustela_sibirica_NC020637_6
Nandinia_binotata_NC024567_1
Nasua_nasua_NC020647_1
Neofelis_nebulosa_NC008450_3
Neophoca_cinerea_NC008419_1
Neovison_vison_NC020641_3
Nyctereutes_procyonoides_NC013700_3
Odobenus_rosmarus_NC004029_29
Ommatophoca_rossii_AY377287etc_1
Otaria_byronia_OTAB_1
Otocolobus_manul_NC028323_1
Otocyon_megalotis_SAF1_2
Paguma_larvata_PDDS11_2
Panthera_leo_NERO_19
Panthera_leo_spelaea_KX258452_2
Panthera_onca_KP202264_2
Panthera_onca_NC022842_1
Panthera_pardus_NC010641_5
Panthera_pardus_japonensis_KJ866876_8
Panthera_tigris_NC010642_35
Panthera_tigris_amoyensis_NC014770_2
Panthera_uncia_KP202269_1
Panthera_uncia_NC010638_1
Paradoxurus_hermaphroditus_NC039591_1
Paradoxurus_hermaphroditus_NLNC_1
Paradoxurus_jerdoni_MH464793_1
Parahyaena_brunnea_NC038159_15
Pardofelis_marmorata_NLN3_2
Phoca_fasciata_NC008428_1
Phoca_groenlandica_NC008429_54
Phoca_largha_NC008430_1
Phoca_vitulina_NC001325_1
Phocarctos_hookeri_NC008418_1
Poecilogale_albinucha_T602_1
Potos_flavus_T414_1
Prionailurus_bengalensis_CKM45_20
Prionailurus_bengalensis_NC028301_12
Prionailurus_planiceps_KY682741_4
Prionailurus_planiceps_NC028312_6
Prionailurus_rubiginosus_NC028304_2
Prionailurus_viverrinus_NC028305_1
Prionodon_linsang_ERR2391707_1
Prionodon_pardicolor_NC024569_2

```
		Procyon_lotor_AB462046_3
		Procyon_lotor_AB462049_4
		Profelis_aurata_NC028299_1
		Proteles_cristata_T393_6
		Puma_concolor_NC016470_22
		Puma_yagouaroundi_NC028311_1
		Pusa_caspica_NC008431_1
		Pusa_hispida_NC_008433_1
		Pusa_sibirica_NC008432_2
		Salanoia_concolor_D378_1
		Smilodon_populator_MF871700_1
		Speothos_venaticus_C48_2
		Spilogale_putorius_NC010497_1
		Suricata_suricatta_SSM10_1
		Taxidea_taxus_NC020646_1
		Tremarctos_ornatus_NC009969_2
		Urocyon_cinereoargenteus_NC026723_21
		Urocyon_littoralis_catalinae_KP129018_15
		Ursus_americanus_JX196366_3
		Ursus_arctos_AP012576_6
		Ursus_arctos_EU497665_29
		Ursus_arctos_GU573486_5
		Ursus_arctos_GU573491_207
		Ursus_arctos_isabellinus_18B5_2
		Ursus_arctos_pruinosus_MG066703_3
		Ursus_maritimus_GU573488_Svalbard
		Ursus_maritimus_NC003428_31
		Ursus_spelaeus_EU327344_13
		Ursus_spelaeus_NC011112_8
		Ursus_thibetanus_formosanus_NC009331_1
		Ursus_thibetanus_laniger_MH281753_2
		Ursus_thibetanus_mupinensis_NC008753_2
		Ursus_thibetanus_thibetanus_NC011118_4
		Viverra_tangalunga_MH464792_1
		Viverra_zibetha_T609_1
		Viverricula_indica_KX891745_1
		Viverricula_indica_KX891751_1
		Viverricula_indica_NC025296_2
		Vulpes_corsac_NC023958_1
		Vulpes_ferrilata_NC027935_1
		Vulpes_lagopus_NC026529_3
		Vulpes_vulpes_NC008434_5
		Vulpes_zerda_KJ603240_1
		Zalophus_californianus_NC008416_1
		Zalophus_wollebaeki_SRR4431565_1
		;
end;
begin trees;
	translate
		1	Tapirus_terrestris_T358,
		2	Phataginus_tricuspis_NC026780,
		3	Acinonyx_jubatus_NC005212_3,
		4	Ailuropoda_melanoleuca_NC009492_5,
		5	Ailurus_fulgens_NC011124_1,
		6	Ailurus_fulgens_styani_NC009691_1,
		7	Aonyx_cinerea_NC035814_2,
		8	Arctictis_binturong_T605_2,
		9	Arctocephalus_australis_MG023139_1,
		10	Arctocephalus_forsteri_KT693377_17,
		11	Arctocephalus_forsteri_NC004023_28,
```

12 Arctocephalus_gazella_BK010918_1,
13 Arctocephalus_pusillus_NC008417_1,
14 Arctocephalus_townsendi_NC008420_1,
15 Arctodus_simus_NC011116_1,
16 Arctonyx_collaris_NC020645_1,
17 Arctotherium_sp_NC030174_1,
18 Atilax_paludinosus_T606_1,
19 Bassaricyon_neblina_SRX1097850_1,
20 Bassariscus_sumichrasti_SRX1099089_1,
21 Bdeogale_nigripes_GLC15_1,
22 Callorhinus_ursinus_NC008415_1,
23 Canis_adustus_KT448271_1,
24 Canis_anthus_NC027956_2,
25 Canis_aureus_KT448274_1,
26 Canis_latrans_NC008093_7,
27 Canis_lupus_chanco_NC010340_4,
28 Canis_lupus_familiaris_NC002008_1231,
29 Canis_mesomelas_KT448280_1,
30 Caracal_caracal_NC028306_1,
31 Catopuma_badia_NC028300_1,
32 Catopuma_temminckii_NC027115_41,
33 Chrotogale_owstoni_T607_1,
34 Chrysocyon_brachyurus_NC024172_1,
35 Civettictis_civetta_GLC19_1,
36 Civettictis_civetta_NC033378_1,
37 Conepatus_chinga_NC042596_1,
38 Crocuta_crocuta_NC020670_3,
39 Crossarchus_platycephalus_C7AR66_1,
40 Cryptoprocta_ferox_CFC13_1,
41 Cuon_alpinus_NC013445_3,
42 Cynictis_penicillata_T375_1,
43 Cynogale_bennetti_KY117544_1,
44 Cystophora_cristata_NC008427_1,
45 Diplogale_hosei_MH464790_1,
46 Enhydra_lutris_NC009692_1,
47 Erignathus_barbatus_NC008426_1,
48 Eumetopias_jubatus_NC004030_10,
49 Eupleres_goudotii_D128_1,
50 Felis_catus_NC001700_2,
51 Felis_chaus_NC028307_1,
52 Felis_margarita_NC028308_1,
53 Felis_nigripes_NC028309_1,
54 Felis_silvestris_lybica_KP202275_4,
55 Fossa_fossana_D350_1,
56 Galerella_sanguinea_T378_1,
57 Galictis_vittata_T412_1,
58 Galidia_elegans_D146_1,
59 Galidictis_fasciata_DM333_1,
60 Genetta_abyssinica_MG489822_1,
61 Genetta_genetta_T297_1,
62 Genetta_servalina_NC024568_2,
63 Gulo_gulo_NC009685_3,
64 Halichoerus_grypus_NC001602_2,
65 Helarctos_malayanus_NC009968_2,
66 Helogale_parvula_SRR7637809_1,
67 Hemigalus_derbyanus_MH464791_1,
68 Herpestes_brachyurus_KY117547_1,
69 Herpestes_javanicus_NC006835_1,
70 Herpestes_javanicus_T413_1,
71 Herpestes_naso_C07XAR110_1,

72 Herpestes_semitorquatus_MH464789_1,
73 Homotherium_latidens_MF871702_3,
74 Hyaena_hyaena_NC020669_1,
75 Hydrurga_leptonyx_NC008425_1,
76 Ichneumia_albicauda_T603_1,
77 Ictonyx_striatus_T299_1,
78 Leopardus_colocolo_NC028314_1,
79 Leopardus_geoffroyi_NC028320_1,
80 Leopardus_guigna_NC028321_1,
81 Leopardus_jacobita_NC028322_1,
82 Leopardus_pardalis_NC028315_1,
83 Leopardus_pardalis_T262_1,
84 Leopardus_tigrinus_NC028317_1,
85 Leopardus_wiedii_NC028318_1,
86 Leptailurus_serval_NC028316_1,
87 Leptonychotes_weddellii_NC008424_1,
88 Lobodon_carcinophaga_NC008423_1,
89 Lontra_canadensis_SRR10409165_1,
90 Lutra_lutra_LC050126_1,
91 Lutra_lutra_NC011358_9,
92 Lutra_sumatrana_NC035810_1,
93 Lutrogale_perspicillata_NC035811_1,
94 Lycalopex_sechurae_KT448284_1,
95 Lycaon_pictus_NC028427_2,
96 Lynx_canadensis_NC028313_1,
97 Lynx_lynx_NC027083_4,
98 Lynx_pardinus_NC028319_161,
99 Lynx_rufus_NC014456_3,
100 Martes_americana_NC020642_1,
101 Martes_flavigula_NC012141_3,
102 Martes_foina_NC020643_1,
103 Martes_martes_T302_3,
104 Martes_melampus_NC009678_1,
105 Martes_pennanti_NC020664_16,
106 Martes_zibellina_NC011579_39,
107 Meles_anakuma_NC009677_1,
108 Meles_leucurus_NC039173_4,
109 Meles_meles_T303_3,
110 Mellivora_capensis_T370_1,
111 Melogale_moschata_KP726273_1,
112 Melogale_moschata_NC020644_1,
113 Melogale_moschata_V0735A_1,
114 Melursus_ursinus_NC009970_2,
115 Mephitis_mephitis_NC020648_1,
116 Mirounga_angustirostris_SRR10331586_1,
117 Mirounga_leonina_NC008422_1,
118 Monachus_monachus_NC044972_5,
119 Monachus_schauinslandi_NC008421_1,
120 Mungos_mungo_MMC7_1,
121 Mungos_mungo_SRR7704821_1,
122 Mungotictis_decemlineata_NC027828_1,
123 Mustela_altaica_NC021751_1,
124 Mustela_erminea_T305_2,
125 Mustela_eversmannii_NC028013_1,
126 Mustela_frenata_NC020640_1,
127 Mustela_itatsi_NC034330_19,
128 Mustela_kathiah_NC023210_1,
129 Mustela_nigripes_NC024942_1,
130 Mustela_nivalis_T306_5,
131 Mustela_putorius_NC020638_4,

132 Mustela_sibirica_AP017394_11,
133 Mustela_sibirica_NC020637_6,
134 Nandinia_binotata_NC024567_1,
135 Nasua_nasua_NC020647_1,
136 Neofelis_nebulosa_NC008450_3,
137 Neophoca_cinerea_NC008419_1,
138 Neovison_vison_NC020641_3,
139 Nyctereutes_procyonoides_NC013700_3,
140 Odobenus_rosmarus_NC004029_29,
141 Ommatophoca_rossii_AY377287etc_1,
142 Otaria_byronia_OTAB_1,
143 Otocolobus_manul_NC028323_1,
144 Otocyon_megalotis_SAF1_2,
145 Paguma_larvata_PDDS11_2,
146 Panthera_leo_NERO_19,
147 Panthera_leo_spelaea_KX258452_2,
148 Panthera_onca_KP202264_2,
149 Panthera_onca_NC022842_1,
150 Panthera_pardus_NC010641_5,
151 Panthera_pardus_japonensis_KJ866876_8,
152 Panthera_tigris_NC010642_35,
153 Panthera_tigris_amoyensis_NC014770_2,
154 Panthera_uncia_KP202269_1,
155 Panthera_uncia_NC010638_1,
156 Paradoxurus_hermaphroditus_NC039591_1,
157 Paradoxurus_hermaphroditus_NLNC_1,
158 Paradoxurus_jerdoni_MH464793_1,
159 Parahyaena_brunnea_NC038159_15,
160 Pardofelis_marmorata_NLN3_2,
161 Phoca_fasciata_NC008428_1,
162 Phoca_groenlandica_NC008429_54,
163 Phoca_largha_NC008430_1,
164 Phoca_vitulina_NC001325_1,
165 Phocarctos_hookeri_NC008418_1,
166 Poecilogale_albinucha_T602_1,
167 Potos_flavus_T414_1,
168 Prionailurus_bengalensis_CKM45_20,
169 Prionailurus_bengalensis_NC028301_12,
170 Prionailurus_planiceps_KY682741_4,
171 Prionailurus_planiceps_NC028312_6,
172 Prionailurus_rubiginosus_NC028304_2,
173 Prionailurus_viverrinus_NC028305_1,
174 Prionodon_linsang_ERR2391707_1,
175 Prionodon_pardicolor_NC024569_2,
176 Procyon_lotor_AB462046_3,
177 Procyon_lotor_AB462049_4,
178 Profelis_aurata_NC028299_1,
179 Proteles_cristata_T393_6,
180 Puma_concolor_NC016470_22,
181 Puma_yagouaroundi_NC028311_1,
182 Pusa_caspica_NC008431_1,
183 Pusa_hispida_NC_008433_1,
184 Pusa_sibirica_NC008432_2,
185 Salanoia_concolor_D378_1,
186 Smilodon_populator_MF871700_1,
187 Speothos_venaticus_C48_2,
188 Spilogale_putorius_NC010497_1,
189 Suricata_suricatta_SSM10_1,
190 Taxidea_taxus_NC020646_1,
191 Tremarctos_ornatus_NC009969_2,

192 Urocyon_cinereoargenteus_NC026723_21,
193 Urocyon_littoralis_catalinae_KP129018_15,
194 Ursus_americanus_JX196366_3,
195 Ursus_arctos_AP012576_6,
196 Ursus_arctos_EU497665_29,
197 Ursus_arctos_GU573486_5,
198 Ursus_arctos_GU573491_207,
199 Ursus_arctos_isabellinus_18B5_2,
200 Ursus_arctos_pruinosus_MG066703_3,
201 Ursus_maritimus_GU573488_Svalbard,
202 Ursus_maritimus_NC003428_31,
203 Ursus_spelaeus_EU327344_13,
204 Ursus_spelaeus_NC011112_8,
205 Ursus_thibetanus_formosanus_NC009331_1,
206 Ursus_thibetanus_laniger_MH281753_2,
207 Ursus_thibetanus_mupinensis_NC008753_2,
208 Ursus_thibetanus_thibetanus_NC011118_4,
209 Viverra_tangalunga_MH464792_1,
210 Viverra_zibetha_T609_1,
211 Viverricula_indica_KX891745_1,
212 Viverricula_indica_KX891751_1,
213 Viverricula_indica_NC025296_2,
214 Vulpes_corsac_NC023958_1,
215 Vulpes_ferrilata_NC027935_1,
216 Vulpes_lagopus_NC026529_3,
217 Vulpes_vulpes_NC008434_5,
218 Vulpes_zerda_KJ603240_1,
219 Zalophus_californianus_NC008416_1,
220 Zalophus_wollebaeki_SRR4431565_1
;
tree con_all_compat = [&U]
(1[&prob=1.00000000e+00,prob_stddev=0.00000000e+00,prob_range={1.00000000e+00,1.00000000e+00},p
rob(percent)="100",prob+-sd="100+-0"]:5.948905e-02[&length_mean=5.96305434e-
02,length_median=5.94890500e-02,length_95%HPD={4.90062900e-02,7.03460700e-
02}],2[&prob=1.00000000e+00,prob_stddev=0.00000000e+00,prob_range={1.00000000e+00,1.00000000e+0
0},prob(percent)="100",prob+-sd="100+-0"]:1.999766e-01[&length_mean=2.00505419e-
01,length_median=1.99976600e-01,length_95%HPD={1.80051800e-01,2.22492800e-
01}],((((((((((3[&prob=1.00000000e+00,prob_stddev=0.00000000e+00,prob_range={1.00000000e+00,1.0000
0000e+00},prob(percent)="100",prob+-sd="100+-0"]:4.253277e-03[&length_mean=4.27949119e-
03,length_median=4.25327700e-03,length_95%HPD={3.17555200e-03,5.39201100e-
03}],(180[&prob=1.00000000e+00,prob_stddev=0.00000000e+00,prob_range={1.00000000e+00,1.00000000
e+00},prob(percent)="100",prob+-sd="100+-0"]:2.437743e-03[&length_mean=2.46195774e-
03,length_median=2.43774300e-03,length_95%HPD={1.60018700e-03,3.34108200e-
03}],181[&prob=1.00000000e+00,prob_stddev=0.00000000e+00,prob_range={1.00000000e+00,1.00000000e
+00},prob(percent)="100",prob+-sd="100+-0"]:3.105919e-03[&length_mean=3.13059350e-
03,length_median=3.10591900e-03,length_95%HPD={2.21661800e-03,4.13694700e-
03}])[&prob=8.69617384e-01,prob_stddev=4.14780674e-03,prob_range={8.66684442e-01,8.72550327e-
01},prob(percent)="87",prob+-sd="87+-0"]:2.799851e-04[&length_mean=3.02671934e-
04,length_median=2.79985100e-04,length_95%HPD={2.06496400e-05,6.24492900e-
04}])[&prob=1.00000000e+00,prob_stddev=0.00000000e+00,prob_range={1.00000000e+00,1.00000000e+00
},prob(percent)="100",prob+-sd="100+-0"]:1.239764e-03[&length_mean=1.26940781e-
03,length_median=1.23976400e-03,length_95%HPD={6.86604300e-04,1.92431200e-
03}],(((31[&prob=1.00000000e+00,prob_stddev=0.00000000e+00,prob_range={1.00000000e+00,1.00000000
e+00},prob(percent)="100",prob+-sd="100+-0"]:2.120319e-03[&length_mean=2.15137487e-
03,length_median=2.12031900e-03,length_95%HPD={1.39485100e-03,2.98218600e-
03}],32[&prob=1.00000000e+00,prob_stddev=0.00000000e+00,prob_range={1.00000000e+00,1.00000000e+
00},prob(percent)="100",prob+-sd="100+-0"]:2.410740e-03[&length_mean=2.44088598e-
03,length_median=2.41074000e-03,length_95%HPD={1.63931100e-03,3.29680900e-
03}])[&prob=1.00000000e+00,prob_stddev=0.00000000e+00,prob_range={1.00000000e+00,1.00000000e+00
},prob(percent)="100",prob+-sd="100+-0"]:1.788525e-03[&length_mean=1.81247383e-

03,length_median=1.78852500e-03,length_95%HPD={1.14795200e-03,2.62484700e-
03}],160[&prob=1.00000000e+00,prob_stddev=0.00000000e+00,prob_range={1.00000000e+00,1.00000000e
+00},prob(percent)="100",prob+-sd="100+-0"]:3.060110e-03[&length_mean=3.07528776e-
03,length_median=3.06011000e-03,length_95%HPD={2.19788800e-03,4.05835500e-
03}])[&prob=9.47473670e-01,prob_stddev=9.80390684e-03,prob_range={9.40541261e-01,9.54406079e-
01},prob(percent)="95",prob+-sd="95+-1"]:2.451347e-04[&length_mean=2.69301000e-
04,length_median=2.45134700e-04,length_95%HPD={1.67921400e-05,5.72506900e-
04}],(((96[&prob=1.00000000e+00,prob_stddev=0.00000000e+00,prob_range={1.00000000e+00,1.00000000
e+00},prob(percent)="100",prob+-sd="100+-0"]:4.812089e-04[&length_mean=5.07976159e-
04,length_median=4.81208900e-04,length_95%HPD={1.59314300e-04,8.80030000e-
04}],98[&prob=1.00000000e+00,prob_stddev=0.00000000e+00,prob_range={1.00000000e+00,1.00000000e+
00},prob(percent)="100",prob+-sd="100+-0"]:1.974363e-04[&length_mean=2.17995697e-
04,length_median=1.97436300e-04,length_95%HPD={2.00542600e-05,4.57486500e-
04}])[&prob=9.60471937e-01,prob_stddev=9.42683350e-05,prob_range={9.60405279e-01,9.60538595e-
01},prob(percent)="96",prob+-sd="96+-0"]:1.228449e-04[&length_mean=1.46042889e-
04,length_median=1.22844900e-04,length_95%HPD={2.50929000e-07,3.46038600e-
04}],97[&prob=1.00000000e+00,prob_stddev=0.00000000e+00,prob_range={1.00000000e+00,1.00000000e+
00},prob(percent)="100",prob+-sd="100+-0"]:3.452784e-04[&length_mean=3.68818266e-
04,length_median=3.45278400e-04,length_95%HPD={9.21530100e-05,6.89149400e-
04}])[&prob=1.00000000e+00,prob_stddev=0.00000000e+00,prob_range={1.00000000e+00,1.00000000e+00
},prob(percent)="100",prob+-sd="100+-0"]:8.604681e-04[&length_mean=8.81795019e-
04,length_median=8.60468100e-04,length_95%HPD={4.21024800e-04,1.40360300e-
03}],99[&prob=1.00000000e+00,prob_stddev=0.00000000e+00,prob_range={1.00000000e+00,1.00000000e+
00},prob(percent)="100",prob+-sd="100+-0"]:1.959275e-03[&length_mean=1.98988042e-
03,length_median=1.95927500e-03,length_95%HPD={1.25901700e-03,2.74834700e-
03}])[&prob=1.00000000e+00,prob_stddev=0.00000000e+00,prob_range={1.00000000e+00,1.00000000e+00
},prob(percent)="100",prob+-sd="100+-0"]:8.548095e-04[&length_mean=8.80079746e-
04,length_median=8.54809500e-04,length_95%HPD={4.13228300e-04,1.37737600e-
03}])[&prob=5.82122384e-01,prob_stddev=7.82427181e-03,prob_range={5.76589788e-01,5.87654979e-
01},prob(percent)="58",prob+-sd="58+-1"]:9.799095e-05[&length_mean=1.22681091e-
04,length_median=9.79909500e-05,length_95%HPD={2.01561500e-08,3.18652300e-
04}])[&prob=1.00000000e+00,prob_stddev=0.00000000e+00,prob_range={1.00000000e+00,1.00000000e+00
},prob(percent)="100",prob+-sd="100+-0"]:1.001524e-03[&length_mean=1.02370421e-
03,length_median=1.00152400e-03,length_95%HPD={5.00952900e-04,1.57957400e-
03}],(((30[&prob=1.00000000e+00,prob_stddev=0.00000000e+00,prob_range={1.00000000e+00,1.00000000
e+00},prob(percent)="100",prob+-sd="100+-0"]:2.027437e-03[&length_mean=2.05702698e-
03,length_median=2.02743700e-03,length_95%HPD={1.27781300e-03,2.81841900e-
03}],178[&prob=1.00000000e+00,prob_stddev=0.00000000e+00,prob_range={1.00000000e+00,1.00000000e
+00},prob(percent)="100",prob+-sd="100+-0"]:1.361507e-03[&length_mean=1.39542418e-
03,length_median=1.36150700e-03,length_95%HPD={8.12347400e-04,2.05782900e-
03}])[&prob=1.00000000e+00,prob_stddev=0.00000000e+00,prob_range={1.00000000e+00,1.00000000e+00
},prob(percent)="100",prob+-sd="100+-0"]:9.258169e-04[&length_mean=9.54630802e-
04,length_median=9.25816900e-04,length_95%HPD={4.69690600e-04,1.50163100e-
03}],86[&prob=1.00000000e+00,prob_stddev=0.00000000e+00,prob_range={1.00000000e+00,1.00000000e+
00},prob(percent)="100",prob+-sd="100+-0"]:2.475825e-03[&length_mean=2.50161996e-
03,length_median=2.47582500e-03,length_95%HPD={1.67562100e-03,3.37881800e-
03}])[&prob=1.00000000e+00,prob_stddev=0.00000000e+00,prob_range={1.00000000e+00,1.00000000e+00
},prob(percent)="100",prob+-sd="100+-0"]:6.149051e-04[&length_mean=6.40673887e-
04,length_median=6.14905100e-04,length_95%HPD={2.46348200e-04,1.09100700e-
03}],((((((50[&prob=1.00000000e+00,prob_stddev=0.00000000e+00,prob_range={1.00000000e+00,1.000000
00e+00},prob(percent)="100",prob+-sd="100+-0"]:1.203853e-03[&length_mean=1.22624180e-
03,length_median=1.20385300e-03,length_95%HPD={6.71546000e-04,1.83703700e-
03}],54[&prob=1.00000000e+00,prob_stddev=0.00000000e+00,prob_range={1.00000000e+00,1.00000000e+
00},prob(percent)="100",prob+-sd="100+-0"]:1.282461e-04[&length_mean=1.52190340e-
04,length_median=1.28246100e-04,length_95%HPD={3.26526700e-06,3.63419300e-
04}])[&prob=8.23623517e-01,prob_stddev=1.82880570e-02,prob_range={8.10691908e-01,8.36555126e-
01},prob(percent)="82",prob+-sd="82+-2"]:1.830740e-04[&length_mean=2.09822833e-
04,length_median=1.83074000e-04,length_95%HPD={1.15009300e-05,4.65325600e-
04}],52[&prob=1.00000000e+00,prob_stddev=0.00000000e+00,prob_range={1.00000000e+00,1.00000000e+
00},prob(percent)="100",prob+-sd="100+-0"]:1.028820e-03[&length_mean=1.05395759e-

03,length_median=1.02882000e-03,length_95%HPD={5.27521100e-04,1.59912000e-
03}])[&prob=1.00000000e+00,prob_stddev=0.00000000e+00,prob_range={1.00000000e+00,1.00000000e+00
},prob(percent)="100",prob+-sd="100+-0"]:1.529367e-03[&length_mean=1.55060875e-
03,length_median=1.52936700e-03,length_95%HPD={9.38827600e-04,2.22666200e-
03}],53[&prob=1.00000000e+00,prob_stddev=0.00000000e+00,prob_range={1.00000000e+00,1.00000000e+
00},prob(percent)="100",prob+-sd="100+-0"]:2.537373e-03[&length_mean=2.56733920e-
03,length_median=2.53737300e-03,length_95%HPD={1.73591800e-03,3.47699900e-
03}])[&prob=3.33888815e-01,prob_stddev=7.25866180e-03,prob_range={3.28756166e-01,3.39021464e-
01},prob(percent)="33",prob+-sd="33+-1"]:4.867450e-05[&length_mean=7.32278071e-
05,length_median=4.86745000e-05,length_95%HPD={1.20965900e-08,2.22520500e-
04}],51[&prob=1.00000000e+00,prob_stddev=0.00000000e+00,prob_range={1.00000000e+00,1.00000000e+
00},prob(percent)="100",prob+-sd="100+-0"]:1.657572e-03[&length_mean=1.67383034e-
03,length_median=1.65757200e-03,length_95%HPD={1.00632500e-03,2.35479400e-
03}])[&prob=1.00000000e+00,prob_stddev=0.00000000e+00,prob_range={1.00000000e+00,1.00000000e+00
},prob(percent)="100",prob+-sd="100+-0"]:1.911948e-03[&length_mean=1.93018088e-
03,length_median=1.91194800e-03,length_95%HPD={1.19024600e-03,2.68817500e-
03}],143[&prob=1.00000000e+00,prob_stddev=0.00000000e+00,prob_range={1.00000000e+00,1.00000000e
+00},prob(percent)="100",prob+-sd="100+-0"]:5.036989e-03[&length_mean=5.06036592e-
03,length_median=5.03698900e-03,length_95%HPD={3.84761900e-03,6.25093100e-
03}])[&prob=9.20143981e-01,prob_stddev=3.20512339e-03,prob_range={9.17877616e-01,9.22410345e-
01},prob(percent)="92",prob+-sd="92+-0"]:2.431076e-04[&length_mean=2.67262388e-
04,length_median=2.43107600e-04,length_95%HPD={2.56300300e-05,5.70725100e-
04}],(((168[&prob=1.00000000e+00,prob_stddev=0.00000000e+00,prob_range={1.00000000e+00,1.0000000
0e+00},prob(percent)="100",prob+-sd="100+-0"]:2.100805e-04[&length_mean=2.35710271e-
04,length_median=2.10080500e-04,length_95%HPD={2.88036600e-05,5.06570900e-
04}],(169[&prob=1.00000000e+00,prob_stddev=0.00000000e+00,prob_range={1.00000000e+00,1.00000000
e+00},prob(percent)="100",prob+-sd="100+-0"]:6.096573e-04[&length_mean=6.37775217e-
04,length_median=6.09657300e-04,length_95%HPD={2.33266000e-04,1.06340200e-
03}],173[&prob=1.00000000e+00,prob_stddev=0.00000000e+00,prob_range={1.00000000e+00,1.00000000e
+00},prob(percent)="100",prob+-sd="100+-0"]:6.544207e-04[&length_mean=6.84372464e-
04,length_median=6.54420700e-04,length_95%HPD={2.80948400e-04,1.13342400e-
03}])[&prob=7.11105186e-01,prob_stddev=3.20512339e-03,prob_range={7.08838821e-01,7.13371550e-
01},prob(percent)="71",prob+-sd="71+-0"]:1.198661e-04[&length_mean=1.43554205e-
04,length_median=1.19866100e-04,length_95%HPD={6.40225700e-08,3.40947300e-
04}])[&prob=1.00000000e+00,prob_stddev=0.00000000e+00,prob_range={1.00000000e+00,1.00000000e+00
},prob(percent)="100",prob+-sd="100+-0"]:8.386947e-04[&length_mean=8.61342872e-
04,length_median=8.38694700e-04,length_95%HPD={3.96416100e-04,1.38267200e-
03}],(170[&prob=1.00000000e+00,prob_stddev=0.00000000e+00,prob_range={1.00000000e+00,1.00000000
e+00},prob(percent)="100",prob+-sd="100+-0"]:4.145035e-04[&length_mean=4.37894449e-
04,length_median=4.14503500e-04,length_95%HPD={1.21044300e-04,7.89598100e-
04}],171[&prob=1.00000000e+00,prob_stddev=0.00000000e+00,prob_range={1.00000000e+00,1.00000000e
+00},prob(percent)="100",prob+-sd="100+-0"]:1.205043e-04[&length_mean=1.44334432e-
04,length_median=1.20504300e-04,length_95%HPD={3.97461100e-07,3.51039400e-
04}])[&prob=1.00000000e+00,prob_stddev=0.00000000e+00,prob_range={1.00000000e+00,1.00000000e+00
},prob(percent)="100",prob+-sd="100+-0"]:1.224518e-03[&length_mean=1.25506040e-
03,length_median=1.22451800e-03,length_95%HPD={6.77485700e-04,1.85652400e-
03}])[&prob=6.41381149e-01,prob_stddev=9.61537017e-03,prob_range={6.34582056e-01,6.48180243e-
01},prob(percent)="64",prob+-sd="64+-1"]:1.050345e-04[&length_mean=1.30302341e-
04,length_median=1.05034500e-04,length_95%HPD={5.73971800e-08,3.30596100e-
04}],172[&prob=1.00000000e+00,prob_stddev=0.00000000e+00,prob_range={1.00000000e+00,1.00000000e
+00},prob(percent)="100",prob+-sd="100+-0"]:1.914947e-03[&length_mean=1.93691317e-
03,length_median=1.91494700e-03,length_95%HPD={1.21433600e-03,2.71060600e-
03}])[&prob=1.00000000e+00,prob_stddev=0.00000000e+00,prob_range={1.00000000e+00,1.00000000e+00
},prob(percent)="100",prob+-sd="100+-0"]:1.794562e-03[&length_mean=1.82337699e-
03,length_median=1.79456200e-03,length_95%HPD={1.11153000e-03,2.54468500e-
03}])[&prob=1.00000000e+00,prob_stddev=0.00000000e+00,prob_range={1.00000000e+00,1.00000000e+00
},prob(percent)="100",prob+-sd="100+-0"]:4.764821e-04[&length_mean=4.98428690e-
04,length_median=4.76482100e-04,length_95%HPD={1.53243900e-04,8.90499900e-
04}])[&prob=9.78736169e-01,prob_stddev=6.59878345e-04,prob_range={9.78269564e-01,9.79202773e-
01},prob(percent)="98",prob+-sd="98+-0"]:1.892316e-04[&length_mean=2.13968672e-

04,length_median=1.89231600e-04,length_95%HPD={1.36087700e-05,4.67066700e-04}])[&prob=1.00000000e+00,prob_stddev=0.00000000e+00,prob_range={1.00000000e+00,1.00000000e+00},prob(percent)="100",prob+-sd="100+-0"]:1.169201e-03[&length_mean=1.19261207e-03,length_median=1.16920100e-03,length_95%HPD={6.25158900e-04,1.80625100e-03}],((78[&prob=1.00000000e+00,prob_stddev=0.00000000e+00,prob_range={1.00000000e+00,1.00000000e+00},prob(percent)="100",prob+-sd="100+-0"]:2.264137e-03[&length_mean=2.28949588e-03,length_median=2.26413700e-03,length_95%HPD={1.51003600e-03,3.17359400e-03}],(81[&prob=1.00000000e+00,prob_stddev=0.00000000e+00,prob_range={1.00000000e+00,1.00000000e+00},prob(percent)="100",prob+-sd="100+-0"]:1.770174e-03[&length_mean=1.79400117e-03,length_median=1.77017400e-03,length_95%HPD={1.08339800e-03,2.55533200e-03}],((82[&prob=1.00000000e+00,prob_stddev=0.00000000e+00,prob_range={1.00000000e+00,1.00000000e+00},prob(percent)="100",prob+-sd="100+-0"]:6.259915e-04[&length_mean=6.53718825e-04,length_median=6.25991500e-04,length_95%HPD={2.68616700e-04,1.09886300e-03}],83[&prob=1.00000000e+00,prob_stddev=0.00000000e+00,prob_range={1.00000000e+00,1.00000000e+00},prob(percent)="100",prob+-sd="100+-0"]:7.036254e-04[&length_mean=7.30371194e-04,length_median=7.03625400e-04,length_95%HPD={3.09993400e-04,1.17453800e-03}])[&prob=9.97600320e-01,prob_stddev=1.69683003e-03,prob_range={9.96400480e-01,9.98800160e-01},prob(percent)="100",prob+-sd="100+-0"]:1.908273e-04[&length_mean=2.15262300e-04,length_median=1.90827300e-04,length_95%HPD={1.87932500e-05,4.62366200e-04}],85[&prob=1.00000000e+00,prob_stddev=0.00000000e+00,prob_range={1.00000000e+00,1.00000000e+00},prob(percent)="100",prob+-sd="100+-0"]:7.866077e-04[&length_mean=8.08419589e-04,length_median=7.86607700e-04,length_95%HPD={3.77841700e-04,1.30395000e-03}])[&prob=1.00000000e+00,prob_stddev=0.00000000e+00,prob_range={1.00000000e+00,1.00000000e+00},prob(percent)="100",prob+-sd="100+-0"]:3.629118e-04[&length_mean=3.88862378e-04,length_median=3.62911800e-04,length_95%HPD={9.65178300e-05,7.26330600e-04}])[&prob=5.29929343e-01,prob_stddev=6.78732012e-03,prob_range={5.25129983e-01,5.34728703e-01},prob(percent)="53",prob+-sd="53+-1"]:1.223724e-04[&length_mean=1.45033271e-04,length_median=1.22372400e-04,length_95%HPD={1.01388200e-06,3.49629200e-04}])[&prob=9.95267298e-01,prob_stddev=2.82805005e-04,prob_range={9.95067324e-01,9.95467271e-01},prob(percent)="100",prob+-sd="100+-0"]:2.088735e-04[&length_mean=2.32245405e-04,length_median=2.08873500e-04,length_95%HPD={1.50956900e-05,4.98461800e-04}],((79[&prob=1.00000000e+00,prob_stddev=0.00000000e+00,prob_range={1.00000000e+00,1.00000000e+00},prob(percent)="100",prob+-sd="100+-0"]:5.239690e-04[&length_mean=5.46306100e-04,length_median=5.23969000e-04,length_95%HPD={1.89273000e-04,9.29382700e-04}],80[&prob=1.00000000e+00,prob_stddev=0.00000000e+00,prob_range={1.00000000e+00,1.00000000e+00},prob(percent)="100",prob+-sd="100+-0"]:5.954780e-04[&length_mean=6.17601694e-04,length_median=5.95478000e-04,length_95%HPD={2.42971500e-04,1.06176300e-03}])[&prob=1.00000000e+00,prob_stddev=0.00000000e+00,prob_range={1.00000000e+00,1.00000000e+00},prob(percent)="100",prob+-sd="100+-0"]:5.522307e-04[&length_mean=5.78242499e-04,length_median=5.52230700e-04,length_95%HPD={2.04533700e-04,9.78749900e-04}],84[&prob=1.00000000e+00,prob_stddev=0.00000000e+00,prob_range={1.00000000e+00,1.00000000e+00},prob(percent)="100",prob+-sd="100+-0"]:1.334672e-03[&length_mean=1.35066987e-03,length_median=1.33467200e-03,length_95%HPD={7.56673400e-04,1.95874000e-03}])[&prob=1.00000000e+00,prob_stddev=0.00000000e+00,prob_range={1.00000000e+00,1.00000000e+00},prob(percent)="100",prob+-sd="100+-0"]:1.334503e-03[&length_mean=1.36159113e-03,length_median=1.33450300e-03,length_95%HPD={7.96829100e-04,2.02644600e-03}])[&prob=1.00000000e+00,prob_stddev=0.00000000e+00,prob_range={1.00000000e+00,1.00000000e+00},prob(percent)="100",prob+-sd="100+-0"]:2.219184e-03[&length_mean=2.24669799e-03,length_median=2.21918400e-03,length_95%HPD={1.48349800e-03,3.09553700e-03}])[&prob=1.00000000e+00,prob_stddev=0.00000000e+00,prob_range={1.00000000e+00,1.00000000e+00},prob(percent)="100",prob+-sd="100+-0"]:1.878123e-03[&length_mean=1.90259141e-03,length_median=1.87812300e-03,length_95%HPD={1.05648800e-03,2.72793600e-03}],(136[&prob=1.00000000e+00,prob_stddev=0.00000000e+00,prob_range={1.00000000e+00,1.00000000e+00},prob(percent)="100",prob+-sd="100+-0"]:4.745053e-03[&length_mean=4.77549919e-03,length_median=4.74505300e-03,length_95%HPD={3.58216700e-03,5.97486100e-03}],(((((146[&prob=1.00000000e+00,prob_stddev=0.00000000e+00,prob_range={1.00000000e+00,1.00000000e+00},prob(percent)="100",prob+-sd="100+-0"]:4.860256e-04[&length_mean=5.09511242e-04,length_median=4.86025600e-04,length_95%HPD={1.75633600e-04,8.95788600e-04}],147[&prob=1.00000000e+00,prob_stddev=0.00000000e+00,prob_range={1.00000000e+00,1.00000000e+00},prob(percent)="100",prob+-sd="100+-0"]:7.826375e-04[&length_mean=8.01678245e-

04,length_median=7.82637500e-04,length_95%HPD={3.67721100e-04,1.27453900e-
03}])[&prob=1.00000000e+00,prob_stddev=0.00000000e+00,prob_range={1.00000000e+00,1.00000000e+00
},prob(percent)="100",prob+-sd="100+-0"]:7.840609e-04[&length_mean=8.04900965e-
04,length_median=7.84060900e-04,length_95%HPD={3.80622100e-04,1.29715500e-
03}],(150[&prob=1.00000000e+00,prob_stddev=0.00000000e+00,prob_range={1.00000000e+00,1.00000000
e+00},prob(percent)="100",prob+-sd="100+-0"]:5.570181e-04[&length_mean=5.81148443e-
04,length_median=5.57018100e-04,length_95%HPD={2.19335600e-04,9.94578000e-
04}],151[&prob=1.00000000e+00,prob_stddev=0.00000000e+00,prob_range={1.00000000e+00,1.00000000e
+00},prob(percent)="100",prob+-sd="100+-0"]:4.124310e-04[&length_mean=4.35194290e-
04,length_median=4.12431000e-04,length_95%HPD={1.28282500e-04,7.87301300e-
04}])[&prob=1.00000000e+00,prob_stddev=0.00000000e+00,prob_range={1.00000000e+00,1.00000000e+00
},prob(percent)="100",prob+-sd="100+-0"]:7.891887e-04[&length_mean=8.09361206e-
04,length_median=7.89188700e-04,length_95%HPD={3.60211600e-04,1.29351700e-
03}])[&prob=9.99466738e-01,prob_stddev=5.65610010e-04,prob_range={9.99066791e-01,9.99866684e-
01},prob(percent)="100",prob+-sd="100+-0"]:2.701073e-04[&length_mean=2.95293291e-
04,length_median=2.70107300e-04,length_95%HPD={4.61558100e-05,5.88369000e-
04}],(154[&prob=1.00000000e+00,prob_stddev=0.00000000e+00,prob_range={1.00000000e+00,1.00000000
e+00},prob(percent)="100",prob+-sd="100+-0"]:3.256738e-04[&length_mean=3.48708306e-
04,length_median=3.25673800e-04,length_95%HPD={5.19802400e-05,6.76242100e-
04}],155[&prob=1.00000000e+00,prob_stddev=0.00000000e+00,prob_range={1.00000000e+00,1.00000000e
+00},prob(percent)="100",prob+-sd="100+-0"]:2.792111e-03[&length_mean=2.80495485e-
03,length_median=2.79211100e-03,length_95%HPD={1.92526800e-03,3.71746900e-
03}])[&prob=1.00000000e+00,prob_stddev=0.00000000e+00,prob_range={1.00000000e+00,1.00000000e+00
},prob(percent)="100",prob+-sd="100+-0"]:1.838428e-03[&length_mean=1.86161241e-
03,length_median=1.83842800e-03,length_95%HPD={1.14399900e-03,2.61832600e-
03}])[&prob=1.00000000e+00,prob_stddev=0.00000000e+00,prob_range={1.00000000e+00,1.00000000e+00
},prob(percent)="100",prob+-sd="100+-0"]:5.681230e-04[&length_mean=5.95123497e-
04,length_median=5.68123000e-04,length_95%HPD={2.08155400e-04,1.04519700e-
03}],(148[&prob=1.00000000e+00,prob_stddev=0.00000000e+00,prob_range={1.00000000e+00,1.00000000
e+00},prob(percent)="100",prob+-sd="100+-0"]:6.186478e-04[&length_mean=6.42509507e-
04,length_median=6.18647800e-04,length_95%HPD={2.29010400e-04,1.07809300e-
03}],149[&prob=1.00000000e+00,prob_stddev=0.00000000e+00,prob_range={1.00000000e+00,1.00000000e
+00},prob(percent)="100",prob+-sd="100+-0"]:4.991415e-04[&length_mean=5.20812761e-
04,length_median=4.99141500e-04,length_95%HPD={1.49307100e-04,8.95893500e-
04}])[&prob=1.00000000e+00,prob_stddev=0.00000000e+00,prob_range={1.00000000e+00,1.00000000e+00
},prob(percent)="100",prob+-sd="100+-0"]:9.119477e-04[&length_mean=9.40010046e-
04,length_median=9.11947700e-04,length_95%HPD={4.51670600e-04,1.45924400e-
03}])[&prob=1.00000000e+00,prob_stddev=0.00000000e+00,prob_range={1.00000000e+00,1.00000000e+00
},prob(percent)="100",prob+-sd="100+-0"]:8.565643e-04[&length_mean=8.82788164e-
04,length_median=8.56564300e-04,length_95%HPD={3.94660600e-04,1.42751400e-
03}],(152[&prob=1.00000000e+00,prob_stddev=0.00000000e+00,prob_range={1.00000000e+00,1.00000000
e+00},prob(percent)="100",prob+-sd="100+-0"]:9.242439e-04[&length_mean=9.47235593e-
04,length_median=9.24243900e-04,length_95%HPD={4.82499700e-04,1.48567700e-
03}],153[&prob=1.00000000e+00,prob_stddev=0.00000000e+00,prob_range={1.00000000e+00,1.00000000e
+00},prob(percent)="100",prob+-sd="100+-0"]:9.268317e-04[&length_mean=9.51625350e-
04,length_median=9.26831700e-04,length_95%HPD={4.52154600e-04,1.49031300e-
03}])[&prob=1.00000000e+00,prob_stddev=0.00000000e+00,prob_range={1.00000000e+00,1.00000000e+00
},prob(percent)="100",prob+-sd="100+-0"]:1.716002e-03[&length_mean=1.74227080e-
03,length_median=1.71600200e-03,length_95%HPD={1.03691800e-03,2.48821000e-
03}])[&prob=1.00000000e+00,prob_stddev=0.00000000e+00,prob_range={1.00000000e+00,1.00000000e+00
},prob(percent)="100",prob+-sd="100+-0"]:1.170323e-03[&length_mean=1.19891675e-
03,length_median=1.17032300e-03,length_95%HPD={5.71813300e-04,1.83908300e-
03}])[&prob=1.00000000e+00,prob_stddev=0.00000000e+00,prob_range={1.00000000e+00,1.00000000e+00
},prob(percent)="100",prob+-sd="100+-0"]:3.230891e-03[&length_mean=3.26165741e-
03,length_median=3.23089100e-03,length_95%HPD={2.21830900e-03,4.31566400e-
03}])[&prob=1.00000000e+00,prob_stddev=0.00000000e+00,prob_range={1.00000000e+00,1.00000000e+00
},prob(percent)="100",prob+-sd="100+-0"]:5.502887e-03[&length_mean=5.54644005e-
03,length_median=5.50288700e-03,length_95%HPD={3.93903800e-03,7.27625000e-
03}],(73[&prob=1.00000000e+00,prob_stddev=0.00000000e+00,prob_range={1.00000000e+00,1.00000000e
+00},prob(percent)="100",prob+-sd="100+-0"]:3.168409e-02[&length_mean=3.17811641e-

02,length_median=3.16840900e-02,length_95%HPD={2.74840500e-02,3.59438200e-02}],186[&prob=1.00000000e+00,prob_stddev=0.00000000e+00,prob_range={1.00000000e+00,1.00000000e+00},prob(percent)="100",prob+-sd="100+-0"]:1.175921e-02[&length_mean=1.17515171e-02,length_median=1.17592100e-02,length_95%HPD={9.14288400e-03,1.43580700e-02}])[&prob=9.91934409e-01,prob_stddev=4.24207508e-03,prob_range={9.88934809e-01,9.94934009e-01},prob(percent)="99",prob+-sd="99+-0"]:2.854856e-03[&length_mean=2.89049847e-03,length_median=2.85485600e-03,length_95%HPD={1.17494500e-03,4.68238100e-03}])[&prob=1.00000000e+00,prob_stddev=0.00000000e+00,prob_range={1.00000000e+00,1.00000000e+00},prob(percent)="100",prob+-sd="100+-0"]:1.398000e-02[&length_mean=1.40484769e-02,length_median=1.39800000e-02,length_95%HPD={1.12264200e-02,1.66958700e-02}],(174[&prob=1.00000000e+00,prob_stddev=0.00000000e+00,prob_range={1.00000000e+00,1.00000000e+00},prob(percent)="100",prob+-sd="100+-0"]:8.608344e-03[&length_mean=8.62126836e-03,length_median=8.60834400e-03,length_95%HPD={6.71823700e-03,1.06837500e-02}],175[&prob=1.00000000e+00,prob_stddev=0.00000000e+00,prob_range={1.00000000e+00,1.00000000e+00},prob(percent)="100",prob+-sd="100+-0"]:8.715117e-03[&length_mean=8.75000717e-03,length_median=8.71511700e-03,length_95%HPD={6.71905700e-03,1.08184100e-02}])[&prob=1.00000000e+00,prob_stddev=0.00000000e+00,prob_range={1.00000000e+00,1.00000000e+00},prob(percent)="100",prob+-sd="100+-0"]:2.856608e-02[&length_mean=2.86135942e-02,length_median=2.85660800e-02,length_95%HPD={2.47056800e-02,3.23474700e-02}])[&prob=9.97933609e-01,prob_stddev=9.42683350e-05,prob_range={9.97866951e-01,9.98000267e-01},prob(percent)="100",prob+-sd="100+-0"]:3.191706e-03[&length_mean=3.23332347e-03,length_median=3.19170600e-03,length_95%HPD={1.44930100e-03,5.07565600e-03}],((((8[&prob=1.00000000e+00,prob_stddev=0.00000000e+00,prob_range={1.00000000e+00,1.00000000e+00},prob(percent)="100",prob+-sd="100+-0"]:9.800841e-03[&length_mean=9.84493597e-03,length_median=9.80084100e-03,length_95%HPD={7.94252400e-03,1.17854500e-02}],(145[&prob=1.00000000e+00,prob_stddev=0.00000000e+00,prob_range={1.00000000e+00,1.00000000e+00},prob(percent)="100",prob+-sd="100+-0"]:2.928924e-03[&length_mean=2.96124717e-03,length_median=2.92892400e-03,length_95%HPD={1.96279300e-03,3.96237700e-03}],((156[&prob=1.00000000e+00,prob_stddev=0.00000000e+00,prob_range={1.00000000e+00,1.00000000e+00},prob(percent)="100",prob+-sd="100+-0"]:2.017068e-03[&length_mean=2.04050513e-03,length_median=2.01706800e-03,length_95%HPD={1.28701300e-03,2.85436700e-03}],157[&prob=1.00000000e+00,prob_stddev=0.00000000e+00,prob_range={1.00000000e+00,1.00000000e+00},prob(percent)="100",prob+-sd="100+-0"]:9.466500e-04[&length_mean=9.75981923e-04,length_median=9.46650000e-04,length_95%HPD={4.22471500e-04,1.55492900e-03}])[&prob=1.00000000e+00,prob_stddev=0.00000000e+00,prob_range={1.00000000e+00,1.00000000e+00},prob(percent)="100",prob+-sd="100+-0"]:1.071075e-03[&length_mean=1.09670924e-03,length_median=1.07107500e-03,length_95%HPD={5.30779900e-04,1.69543400e-03}],158[&prob=1.00000000e+00,prob_stddev=0.00000000e+00,prob_range={1.00000000e+00,1.00000000e+00},prob(percent)="100",prob+-sd="100+-0"]:4.350871e-03[&length_mean=4.37606825e-03,length_median=4.35087100e-03,length_95%HPD={3.20494200e-03,5.54861000e-03}])[&prob=1.00000000e+00,prob_stddev=0.00000000e+00,prob_range={1.00000000e+00,1.00000000e+00},prob(percent)="100",prob+-sd="100+-0"]:3.225086e-03[&length_mean=3.25240457e-03,length_median=3.22508600e-03,length_95%HPD={2.22628600e-03,4.34204600e-03}])[&prob=1.00000000e+00,prob_stddev=0.00000000e+00,prob_range={1.00000000e+00,1.00000000e+00},prob(percent)="100",prob+-sd="100+-0"]:5.686670e-03[&length_mean=5.71158232e-03,length_median=5.68667000e-03,length_95%HPD={4.22039500e-03,7.18944200e-03}])[&prob=1.00000000e+00,prob_stddev=0.00000000e+00,prob_range={1.00000000e+00,1.00000000e+00},prob(percent)="100",prob+-sd="100+-0"]:1.096540e-02[&length_mean=1.09979714e-02,length_median=1.09654000e-02,length_95%HPD={8.82421000e-03,1.31518700e-02}],(((33[&prob=1.00000000e+00,prob_stddev=0.00000000e+00,prob_range={1.00000000e+00,1.00000000e+00},prob(percent)="100",prob+-sd="100+-0"]:7.817863e-03[&length_mean=7.85808265e-03,length_median=7.81786300e-03,length_95%HPD={6.34560600e-03,9.65529200e-03}],67[&prob=1.00000000e+00,prob_stddev=0.00000000e+00,prob_range={1.00000000e+00,1.00000000e+00},prob(percent)="100",prob+-sd="100+-0"]:5.312653e-03[&length_mean=5.33131511e-03,length_median=5.31265300e-03,length_95%HPD={3.83167000e-03,6.83192100e-03}])[&prob=9.99933342e-01,prob_stddev=9.42683350e-05,prob_range={9.99866684e-01,1.00000000e+00},prob(percent)="100",prob+-sd="100+-0"]:1.543172e-03[&length_mean=1.57505004e-03,length_median=1.54317200e-03,length_95%HPD={6.95509900e-04,2.55857500e-03}],45[&prob=1.00000000e+00,prob_stddev=0.00000000e+00,prob_range={1.00000000e+00,1.00000000e+00},prob(percent)="100",prob+-sd="100+-0"]:1.072811e-02[&length_mean=1.07609330e-

02,length_median=1.07281100e-02,length_95%HPD={8.87159100e-03,1.27794000e-02}])[&prob=1.00000000e+00,prob_stddev=0.00000000e+00,prob_range={1.00000000e+00,1.00000000e+00},prob(percent)="100",prob+-sd="100+-0"]:4.794396e-03[&length_mean=4.83444724e-03,length_median=4.79439600e-03,length_95%HPD={3.27243700e-03,6.45268400e-03}],43[&prob=1.00000000e+00,prob_stddev=0.00000000e+00,prob_range={1.00000000e+00,1.00000000e+00},prob(percent)="100",prob+-sd="100+-0"]:1.885269e-02[&length_mean=1.88393771e-02,length_median=1.88526900e-02,length_95%HPD={1.59247800e-02,2.15810400e-02}])[&prob=1.00000000e+00,prob_stddev=0.00000000e+00,prob_range={1.00000000e+00,1.00000000e+00},prob(percent)="100",prob+-sd="100+-0"]:1.018243e-02[&length_mean=1.02082900e-02,length_median=1.01824300e-02,length_95%HPD={8.08570700e-03,1.23817200e-02}])[&prob=1.00000000e+00,prob_stddev=0.00000000e+00,prob_range={1.00000000e+00,1.00000000e+00},prob(percent)="100",prob+-sd="100+-0"]:3.122010e-03[&length_mean=3.17565433e-03,length_median=3.12201000e-03,length_95%HPD={1.86051300e-03,4.67158200e-03}],((((35[&prob=1.00000000e+00,prob_stddev=0.00000000e+00,prob_range={1.00000000e+00,1.00000000e+00},prob(percent)="100",prob+-sd="100+-0"]:2.447945e-04[&length_mean=2.70420582e-04,length_median=2.44794500e-04,length_95%HPD={3.63344100e-05,5.60157900e-04}],36[&prob=1.00000000e+00,prob_stddev=0.00000000e+00,prob_range={1.00000000e+00,1.00000000e+00},prob(percent)="100",prob+-sd="100+-0"]:2.102620e-04[&length_mean=2.35778915e-04,length_median=2.10262000e-04,length_95%HPD={2.62514900e-05,5.00883900e-04}])[&prob=1.00000000e+00,prob_stddev=0.00000000e+00,prob_range={1.00000000e+00,1.00000000e+00},prob(percent)="100",prob+-sd="100+-0"]:2.728701e-03[&length_mean=2.75472017e-03,length_median=2.72870100e-03,length_95%HPD={1.82889000e-03,3.69559000e-03}],210[&prob=1.00000000e+00,prob_stddev=0.00000000e+00,prob_range={1.00000000e+00,1.00000000e+00},prob(percent)="100",prob+-sd="100+-0"]:4.903488e-03[&length_mean=4.91252817e-03,length_median=4.90348800e-03,length_95%HPD={3.62191100e-03,6.08463600e-03}])[&prob=1.00000000e+00,prob_stddev=0.00000000e+00,prob_range={1.00000000e+00,1.00000000e+00},prob(percent)="100",prob+-sd="100+-0"]:1.808870e-03[&length_mean=1.84207663e-03,length_median=1.80887000e-03,length_95%HPD={1.05609600e-03,2.73139700e-03}],((211[&prob=1.00000000e+00,prob_stddev=0.00000000e+00,prob_range={1.00000000e+00,1.00000000e+00},prob(percent)="100",prob+-sd="100+-0"]:5.615349e-04[&length_mean=5.83438801e-04,length_median=5.61534900e-04,length_95%HPD={2.04740100e-04,9.84923700e-04}],212[&prob=1.00000000e+00,prob_stddev=0.00000000e+00,prob_range={1.00000000e+00,1.00000000e+00},prob(percent)="100",prob+-sd="100+-0"]:7.624744e-04[&length_mean=7.90850351e-04,length_median=7.62474400e-04,length_95%HPD={3.56047200e-04,1.29825900e-03}])[&prob=7.46300493e-01,prob_stddev=1.52714703e-02,prob_range={7.35501933e-01,7.57099053e-01},prob(percent)="75",prob+-sd="75+-2"]:1.814295e-04[&length_mean=2.05962886e-04,length_median=1.81429500e-04,length_95%HPD={3.56404900e-08,4.55758500e-04}],213[&prob=1.00000000e+00,prob_stddev=0.00000000e+00,prob_range={1.00000000e+00,1.00000000e+00},prob(percent)="100",prob+-sd="100+-0"]:1.354965e-03[&length_mean=1.37760192e-03,length_median=1.35496500e-03,length_95%HPD={7.80645300e-04,2.05284000e-03}])[&prob=1.00000000e+00,prob_stddev=0.00000000e+00,prob_range={1.00000000e+00,1.00000000e+00},prob(percent)="100",prob+-sd="100+-0"]:5.643051e-03[&length_mean=5.68052266e-03,length_median=5.64305100e-03,length_95%HPD={4.32157000e-03,7.17139800e-03}])[&prob=1.00000000e+00,prob_stddev=0.00000000e+00,prob_range={1.00000000e+00,1.00000000e+00},prob(percent)="100",prob+-sd="100+-0"]:7.187127e-03[&length_mean=7.23829202e-03,length_median=7.18712700e-03,length_95%HPD={5.60261200e-03,8.95832000e-03}],(60[&prob=1.00000000e+00,prob_stddev=0.00000000e+00,prob_range={1.00000000e+00,1.00000000e+00},prob(percent)="100",prob+-sd="100+-0"]:1.333509e-03[&length_mean=1.35985255e-03,length_median=1.33350900e-03,length_95%HPD={6.52777900e-04,2.04395800e-03}],(61[&prob=1.00000000e+00,prob_stddev=0.00000000e+00,prob_range={1.00000000e+00,1.00000000e+00},prob(percent)="100",prob+-sd="100+-0"]:1.239790e-03[&length_mean=1.26781005e-03,length_median=1.23979000e-03,length_95%HPD={6.88181000e-04,1.89625200e-03}],62[&prob=1.00000000e+00,prob_stddev=0.00000000e+00,prob_range={1.00000000e+00,1.00000000e+00},prob(percent)="100",prob+-sd="100+-0"]:6.044070e-04[&length_mean=6.27935890e-04,length_median=6.04407000e-04,length_95%HPD={2.53773800e-04,1.09688500e-03}])[&prob=1.00000000e+00,prob_stddev=0.00000000e+00,prob_range={1.00000000e+00,1.00000000e+00},prob(percent)="100",prob+-sd="100+-0"]:1.321918e-03[&length_mean=1.34697072e-03,length_median=1.32191800e-03,length_95%HPD={7.03893500e-04,2.02923800e-03}])[&prob=1.00000000e+00,prob_stddev=0.00000000e+00,prob_range={1.00000000e+00,1.00000000e+00},prob(percent)="100",prob+-sd="100+-0"]:1.331166e-02[&length_mean=1.33581721e-

02,length_median=1.33116600e-02,length_95%HPD={1.11588000e-02,1.56421800e-
02}])[&prob=9.99066791e-01,prob_stddev=5.65610010e-04,prob_range={9.98666844e-01,9.99466738e-
01},prob(percent)="100",prob+-sd="100+-0"]:1.674210e-03[&length_mean=1.71350806e-
03,length_median=1.67421000e-03,length_95%HPD={6.28417100e-04,2.86114700e-
03}])[&prob=1.00000000e+00,prob_stddev=0.00000000e+00,prob_range={1.00000000e+00,1.00000000e+00
},prob(percent)="100",prob+-sd="100+-0"]:1.014561e-02[&length_mean=1.01684900e-
02,length_median=1.01456100e-02,length_95%HPD={8.09425900e-03,1.24201500e-
02}],((((((18[&prob=1.00000000e+00,prob_stddev=0.00000000e+00,prob_range={1.00000000e+00,1.000000
00e+00},prob(percent)="100",prob+-sd="100+-0"]:1.714958e-03[&length_mean=1.73650436e-
03,length_median=1.71495800e-03,length_95%HPD={1.04981300e-03,2.46467500e-
03}],71[&prob=1.00000000e+00,prob_stddev=0.00000000e+00,prob_range={1.00000000e+00,1.00000000e+
00},prob(percent)="100",prob+-sd="100+-0"]:1.936652e-03[&length_mean=1.96339488e-
03,length_median=1.93665200e-03,length_95%HPD={1.24950200e-03,2.73916800e-
03}])[&prob=1.00000000e+00,prob_stddev=0.00000000e+00,prob_range={1.00000000e+00,1.00000000e+00
},prob(percent)="100",prob+-sd="100+-0"]:1.121005e-03[&length_mean=1.15036058e-
03,length_median=1.12100500e-03,length_95%HPD={6.19719900e-04,1.79202200e-
03}],((68[&prob=1.00000000e+00,prob_stddev=0.00000000e+00,prob_range={1.00000000e+00,1.00000000e
+00},prob(percent)="100",prob+-sd="100+-0"]:2.264310e-03[&length_mean=2.28494638e-
03,length_median=2.26431000e-03,length_95%HPD={1.44194400e-03,3.10301600e-
03}],(69[&prob=1.00000000e+00,prob_stddev=0.00000000e+00,prob_range={1.00000000e+00,1.00000000e
+00},prob(percent)="100",prob+-sd="100+-0"]:1.264107e-03[&length_mean=1.28673554e-
03,length_median=1.26410700e-03,length_95%HPD={6.98472700e-04,1.92888500e-
03}],70[&prob=1.00000000e+00,prob_stddev=0.00000000e+00,prob_range={1.00000000e+00,1.00000000e+
00},prob(percent)="100",prob+-sd="100+-0"]:1.700931e-03[&length_mean=1.72545075e-
03,length_median=1.70093100e-03,length_95%HPD={1.07241600e-03,2.48126500e-
03}])[&prob=1.00000000e+00,prob_stddev=0.00000000e+00,prob_range={1.00000000e+00,1.00000000e+00
},prob(percent)="100",prob+-sd="100+-0"]:1.578023e-03[&length_mean=1.61014664e-
03,length_median=1.57802300e-03,length_95%HPD={9.47601400e-04,2.32135400e-
03}])[&prob=1.00000000e+00,prob_stddev=0.00000000e+00,prob_range={1.00000000e+00,1.00000000e+00
},prob(percent)="100",prob+-sd="100+-0"]:2.925127e-03[&length_mean=2.93799776e-
03,length_median=2.92512700e-03,length_95%HPD={2.00916200e-03,3.90115400e-
03}],72[&prob=1.00000000e+00,prob_stddev=0.00000000e+00,prob_range={1.00000000e+00,1.00000000e+
00},prob(percent)="100",prob+-sd="100+-0"]:3.976558e-03[&length_mean=3.99627959e-
03,length_median=3.97655800e-03,length_95%HPD={2.94709500e-03,5.04981100e-
03}])[&prob=1.00000000e+00,prob_stddev=0.00000000e+00,prob_range={1.00000000e+00,1.00000000e+00
},prob(percent)="100",prob+-sd="100+-0"]:7.093765e-04[&length_mean=7.35334167e-
04,length_median=7.09376500e-04,length_95%HPD={2.93497500e-04,1.23951800e-
03}])[&prob=9.93334222e-01,prob_stddev=4.14780674e-03,prob_range={9.90401280e-01,9.96267164e-
01},prob(percent)="99",prob+-sd="99+-0"]:4.205141e-04[&length_mean=4.45719307e-
04,length_median=4.20514100e-04,length_95%HPD={7.71748300e-05,8.40674500e-
04}],(((21[&prob=1.00000000e+00,prob_stddev=0.00000000e+00,prob_range={1.00000000e+00,1.00000000
e+00},prob(percent)="100",prob+-sd="100+-0"]:2.333819e-03[&length_mean=2.36108590e-
03,length_median=2.33381900e-03,length_95%HPD={1.55695300e-03,3.24365400e-
03}],42[&prob=1.00000000e+00,prob_stddev=0.00000000e+00,prob_range={1.00000000e+00,1.00000000e+
00},prob(percent)="100",prob+-sd="100+-0"]:2.598436e-03[&length_mean=2.62255227e-
03,length_median=2.59843600e-03,length_95%HPD={1.77636700e-03,3.51407700e-
03}])[&prob=1.00000000e+00,prob_stddev=0.00000000e+00,prob_range={1.00000000e+00,1.00000000e+00
},prob(percent)="100",prob+-sd="100+-0"]:5.739461e-04[&length_mean=5.97858615e-
04,length_median=5.73946100e-04,length_95%HPD={1.98007200e-04,1.02589400e-
03}],76[&prob=1.00000000e+00,prob_stddev=0.00000000e+00,prob_range={1.00000000e+00,1.00000000e+
00},prob(percent)="100",prob+-sd="100+-0"]:3.244509e-03[&length_mean=3.25546181e-
03,length_median=3.24450900e-03,length_95%HPD={2.29205900e-03,4.21100000e-
03}])[&prob=1.00000000e+00,prob_stddev=0.00000000e+00,prob_range={1.00000000e+00,1.00000000e+00
},prob(percent)="100",prob+-sd="100+-0"]:5.126514e-04[&length_mean=5.39092991e-
04,length_median=5.12651400e-04,length_95%HPD={1.57137200e-04,9.54063100e-
04}],56[&prob=1.00000000e+00,prob_stddev=0.00000000e+00,prob_range={1.00000000e+00,1.00000000e+
00},prob(percent)="100",prob+-sd="100+-0"]:3.101158e-03[&length_mean=3.12329795e-
03,length_median=3.10115800e-03,length_95%HPD={2.15022000e-03,4.05130000e-
03}])[&prob=1.00000000e+00,prob_stddev=0.00000000e+00,prob_range={1.00000000e+00,1.00000000e+00
},prob(percent)="100",prob+-sd="100+-0"]:8.460873e-04[&length_mean=8.75163634e-

04,length_median=8.46087300e-04,length_95%HPD={3.85906400e-04,1.44155500e-
03}])[&prob=1.00000000e+00,prob_stddev=0.00000000e+00,prob_range={1.00000000e+00,1.00000000e+00
},prob(percent)="100",prob+-sd="100+-0"]:5.343750e-03[&length_mean=5.37876183e-
03,length_median=5.34375000e-03,length_95%HPD={3.89002300e-03,6.87965600e-
03}],(((39[&prob=1.00000000e+00,prob_stddev=0.00000000e+00,prob_range={1.00000000e+00,1.00000000
e+00},prob(percent)="100",prob+-sd="100+-0"]:3.401635e-03[&length_mean=3.42758244e-
03,length_median=3.40163500e-03,length_95%HPD={2.47724400e-03,4.47944800e-
03}],66[&prob=1.00000000e+00,prob_stddev=0.00000000e+00,prob_range={1.00000000e+00,1.00000000e+
00},prob(percent)="100",prob+-sd="100+-0"]:3.900251e-03[&length_mean=3.92376736e-
03,length_median=3.90025100e-03,length_95%HPD={2.84069400e-03,5.01721100e-
03}])[&prob=1.00000000e+00,prob_stddev=0.00000000e+00,prob_range={1.00000000e+00,1.00000000e+00
},prob(percent)="100",prob+-sd="100+-0"]:3.268058e-03[&length_mean=3.30149023e-
03,length_median=3.26805800e-03,length_95%HPD={2.23146000e-03,4.32939200e-
03}],(120[&prob=1.00000000e+00,prob_stddev=0.00000000e+00,prob_range={1.00000000e+00,1.00000000
e+00},prob(percent)="100",prob+-sd="100+-0"]:2.016068e-03[&length_mean=2.03563341e-
03,length_median=2.01606800e-03,length_95%HPD={1.30631100e-03,2.80811300e-
03}],121[&prob=1.00000000e+00,prob_stddev=0.00000000e+00,prob_range={1.00000000e+00,1.00000000e
+00},prob(percent)="100",prob+-sd="100+-0"]:1.180230e-03[&length_mean=1.19933955e-
03,length_median=1.18023000e-03,length_95%HPD={6.50953000e-04,1.82324300e-
03}])[&prob=1.00000000e+00,prob_stddev=0.00000000e+00,prob_range={1.00000000e+00,1.00000000e+00
},prob(percent)="100",prob+-sd="100+-0"]:4.776517e-03[&length_mean=4.80034482e-
03,length_median=4.77651700e-03,length_95%HPD={3.50091100e-03,6.00973000e-
03}])[&prob=1.00000000e+00,prob_stddev=0.00000000e+00,prob_range={1.00000000e+00,1.00000000e+00
},prob(percent)="100",prob+-sd="100+-0"]:2.371786e-03[&length_mean=2.39488106e-
03,length_median=2.37178600e-03,length_95%HPD={1.41257900e-03,3.39289600e-
03}],189[&prob=1.00000000e+00,prob_stddev=0.00000000e+00,prob_range={1.00000000e+00,1.00000000e
+00},prob(percent)="100",prob+-sd="100+-0"]:1.045989e-02[&length_mean=1.04997956e-
02,length_median=1.04598900e-02,length_95%HPD={8.58485000e-03,1.24612900e-
02}])[&prob=1.00000000e+00,prob_stddev=0.00000000e+00,prob_range={1.00000000e+00,1.00000000e+00
},prob(percent)="100",prob+-sd="100+-0"]:2.588529e-03[&length_mean=2.62354086e-
03,length_median=2.58852900e-03,length_95%HPD={1.49665800e-03,3.89588100e-
03}])[&prob=1.00000000e+00,prob_stddev=0.00000000e+00,prob_range={1.00000000e+00,1.00000000e+00
},prob(percent)="100",prob+-sd="100+-0"]:2.374756e-02[&length_mean=2.37813155e-
02,length_median=2.37475600e-02,length_95%HPD={2.05555600e-02,2.71505200e-
02}],((40[&prob=1.00000000e+00,prob_stddev=0.00000000e+00,prob_range={1.00000000e+00,1.00000000e
+00},prob(percent)="100",prob+-sd="100+-0"]:1.028522e-02[&length_mean=1.03144626e-
02,length_median=1.02852200e-02,length_95%HPD={8.35526600e-03,1.22044900e-
02}],(49[&prob=1.00000000e+00,prob_stddev=0.00000000e+00,prob_range={1.00000000e+00,1.00000000e
+00},prob(percent)="100",prob+-sd="100+-0"]:7.895455e-03[&length_mean=7.93411058e-
03,length_median=7.89545500e-03,length_95%HPD={6.29334400e-03,9.56795200e-
03}],55[&prob=1.00000000e+00,prob_stddev=0.00000000e+00,prob_range={1.00000000e+00,1.00000000e+
00},prob(percent)="100",prob+-sd="100+-0"]:7.192648e-03[&length_mean=7.22566973e-
03,length_median=7.19264800e-03,length_95%HPD={5.72908300e-03,8.85236000e-
03}])[&prob=1.00000000e+00,prob_stddev=0.00000000e+00,prob_range={1.00000000e+00,1.00000000e+00
},prob(percent)="100",prob+-sd="100+-0"]:5.181668e-03[&length_mean=5.22139852e-
03,length_median=5.18166800e-03,length_95%HPD={3.83936600e-03,6.65086400e-
03}])[&prob=9.88868151e-01,prob_stddev=8.48415015e-04,prob_range={9.88268231e-01,9.89468071e-
01},prob(percent)="99",prob+-sd="99+-0"]:1.013273e-03[&length_mean=1.05081682e-
03,length_median=1.01327300e-03,length_95%HPD={3.67107700e-04,1.85501400e-
03}],(58[&prob=1.00000000e+00,prob_stddev=0.00000000e+00,prob_range={1.00000000e+00,1.00000000e
+00},prob(percent)="100",prob+-sd="100+-0"]:5.153373e-03[&length_mean=5.16572128e-
03,length_median=5.15337300e-03,length_95%HPD={3.94389500e-03,6.50276000e-
03}],(59[&prob=1.00000000e+00,prob_stddev=0.00000000e+00,prob_range={1.00000000e+00,1.00000000e
+00},prob(percent)="100",prob+-sd="100+-0"]:1.775714e-03[&length_mean=1.80715594e-
03,length_median=1.77571400e-03,length_95%HPD={1.09535500e-03,2.56377500e-
03}],(122[&prob=1.00000000e+00,prob_stddev=0.00000000e+00,prob_range={1.00000000e+00,1.00000000
e+00},prob(percent)="100",prob+-sd="100+-0"]:7.617137e-04[&length_mean=7.90960751e-
04,length_median=7.61713700e-04,length_95%HPD={3.35054200e-04,1.28753900e-
03}],185[&prob=1.00000000e+00,prob_stddev=0.00000000e+00,prob_range={1.00000000e+00,1.00000000e
+00},prob(percent)="100",prob+-sd="100+-0"]:9.403409e-04[&length_mean=9.62538744e-

04,length_median=9.40340900e-04,length_95%HPD={4.41586800e-04,1.49705500e-
03}])[&prob=1.00000000e+00,prob_stddev=0.00000000e+00,prob_range={1.00000000e+00,1.00000000e+00
},prob(percent)="100",prob+-sd="100+-0"]:1.497142e-03[&length_mean=1.52593421e-
03,length_median=1.49714200e-03,length_95%HPD={8.86369600e-04,2.21036600e-
03}])[&prob=9.95000667e-01,prob_stddev=4.71341675e-04,prob_range={9.94667378e-01,9.95333955e-
01},prob(percent)="100",prob+-sd="100+-0"]:6.364383e-04[&length_mean=6.61939313e-
04,length_median=6.36438300e-04,length_95%HPD={1.38197800e-04,1.22774900e-
03}])[&prob=1.00000000e+00,prob_stddev=0.00000000e+00,prob_range={1.00000000e+00,1.00000000e+00
},prob(percent)="100",prob+-sd="100+-0"]:9.836613e-03[&length_mean=9.89162532e-
03,length_median=9.83661300e-03,length_95%HPD={8.06122400e-03,1.18742100e-
02}])[&prob=1.00000000e+00,prob_stddev=0.00000000e+00,prob_range={1.00000000e+00,1.00000000e+00
},prob(percent)="100",prob+-sd="100+-0"]:3.968757e-03[&length_mean=3.99608257e-
03,length_median=3.96875700e-03,length_95%HPD={2.52559700e-03,5.63877800e-
03}])[&prob=1.00000000e+00,prob_stddev=0.00000000e+00,prob_range={1.00000000e+00,1.00000000e+00
},prob(percent)="100",prob+-sd="100+-0"]:3.805322e-03[&length_mean=3.85556657e-
03,length_median=3.80532200e-03,length_95%HPD={2.13810300e-03,5.62466100e-
03}],((38[&prob=1.00000000e+00,prob_stddev=0.00000000e+00,prob_range={1.00000000e+00,1.00000000e
+00},prob(percent)="100",prob+-sd="100+-0"]:3.941174e-03[&length_mean=3.96479054e-
03,length_median=3.94117400e-03,length_95%HPD={2.84407900e-03,5.09497700e-
03}],(74[&prob=1.00000000e+00,prob_stddev=0.00000000e+00,prob_range={1.00000000e+00,1.00000000e
+00},prob(percent)="100",prob+-sd="100+-0"]:1.812028e-03[&length_mean=1.83430820e-
03,length_median=1.81202800e-03,length_95%HPD={1.12401200e-03,2.58384600e-
03}],159[&prob=1.00000000e+00,prob_stddev=0.00000000e+00,prob_range={1.00000000e+00,1.00000000e
+00},prob(percent)="100",prob+-sd="100+-0"]:1.352771e-03[&length_mean=1.38704233e-
03,length_median=1.35277100e-03,length_95%HPD={7.73954500e-04,2.02694000e-
03}])[&prob=1.00000000e+00,prob_stddev=0.00000000e+00,prob_range={1.00000000e+00,1.00000000e+00
},prob(percent)="100",prob+-sd="100+-0"]:1.775392e-03[&length_mean=1.80311453e-
03,length_median=1.77539200e-03,length_95%HPD={1.03989100e-03,2.56540700e-
03}])[&prob=9.99800027e-01,prob_stddev=2.82805005e-04,prob_range={9.99600053e-
01,1.00000000e+00},prob(percent)="100",prob+-sd="100+-0"]:2.126204e-03[&length_mean=2.16509107e-
03,length_median=2.12620400e-03,length_95%HPD={9.79968100e-04,3.41187900e-
03}],179[&prob=1.00000000e+00,prob_stddev=0.00000000e+00,prob_range={1.00000000e+00,1.00000000e
+00},prob(percent)="100",prob+-sd="100+-0"]:9.479823e-03[&length_mean=9.50264745e-
03,length_median=9.47982300e-03,length_95%HPD={7.48355500e-03,1.14744300e-
02}])[&prob=1.00000000e+00,prob_stddev=0.00000000e+00,prob_range={1.00000000e+00,1.00000000e+00
},prob(percent)="100",prob+-sd="100+-0"]:3.393801e-02[&length_mean=3.40603864e-
02,length_median=3.39380100e-02,length_95%HPD={2.99493100e-02,3.81032900e-
02}])[&prob=1.00000000e+00,prob_stddev=0.00000000e+00,prob_range={1.00000000e+00,1.00000000e+00
},prob(percent)="100",prob+-sd="100+-0"]:8.026865e-03[&length_mean=8.06839370e-
03,length_median=8.02686500e-03,length_95%HPD={5.89651300e-03,1.01996000e-
02}])[&prob=1.00000000e+00,prob_stddev=0.00000000e+00,prob_range={1.00000000e+00,1.00000000e+00
},prob(percent)="100",prob+-sd="100+-0"]:2.867234e-03[&length_mean=2.91029832e-
03,length_median=2.86723400e-03,length_95%HPD={1.34513800e-03,4.52356400e-
03}])[&prob=1.00000000e+00,prob_stddev=0.00000000e+00,prob_range={1.00000000e+00,1.00000000e+00
},prob(percent)="100",prob+-sd="100+-0"]:8.945512e-03[&length_mean=8.98894070e-
03,length_median=8.94551200e-03,length_95%HPD={6.20091100e-03,1.19157800e-
02}],134[&prob=1.00000000e+00,prob_stddev=0.00000000e+00,prob_range={1.00000000e+00,1.00000000e
+00},prob(percent)="100",prob+-sd="100+-0"]:2.566807e-02[&length_mean=2.56942805e-
02,length_median=2.56680700e-02,length_95%HPD={2.18902800e-02,2.97098100e-
02}])[&prob=1.00000000e+00,prob_stddev=0.00000000e+00,prob_range={1.00000000e+00,1.00000000e+00
},prob(percent)="100",prob+-sd="100+-0"]:2.266626e-02[&length_mean=2.27095415e-
02,length_median=2.26662600e-02,length_95%HPD={1.76629100e-02,2.78178900e-
02}],(((4[&prob=1.00000000e+00,prob_stddev=0.00000000e+00,prob_range={1.00000000e+00,1.00000000e
+00},prob(percent)="100",prob+-sd="100+-0"]:1.510290e-02[&length_mean=1.51513268e-
02,length_median=1.51029000e-02,length_95%HPD={1.26712800e-02,1.76955800e-
02}],((15[&prob=1.00000000e+00,prob_stddev=0.00000000e+00,prob_range={1.00000000e+00,1.00000000e
+00},prob(percent)="100",prob+-sd="100+-0"]:1.646298e-03[&length_mean=1.67452193e-
03,length_median=1.64629800e-03,length_95%HPD={1.03017600e-03,2.42146800e-
03}],(17[&prob=1.00000000e+00,prob_stddev=0.00000000e+00,prob_range={1.00000000e+00,1.00000000e
+00},prob(percent)="100",prob+-sd="100+-0"]:1.966225e-03[&length_mean=2.00008108e-

03,length_median=1.96622500e-03,length_95%HPD={1.28349500e-03,2.76871200e-03}],191[&prob=1.00000000e+00,prob_stddev=0.00000000e+00,prob_range={1.00000000e+00,1.00000000e+00},prob(percent)="100",prob+-sd="100+-0"]:2.014653e-03[&length_mean=2.04620214e-03,length_median=2.01465300e-03,length_95%HPD={1.28632900e-03,2.86280700e-03}])[&prob=9.99933342e-01,prob_stddev=9.42683350e-05,prob_range={9.99866684e-01,1.00000000e+00},prob(percent)="100",prob+-sd="100+-0"]:4.193316e-04[&length_mean=4.48147730e-04,length_median=4.19331600e-04,length_95%HPD={9.96383000e-05,8.40023200e-04}])[&prob=1.00000000e+00,prob_stddev=0.00000000e+00,prob_range={1.00000000e+00,1.00000000e+00},prob(percent)="100",prob+-sd="100+-0"]:2.820543e-03[&length_mean=2.84745934e-03,length_median=2.82054300e-03,length_95%HPD={1.81423500e-03,3.86305200e-03}],(((65[&prob=1.00000000e+00,prob_stddev=0.00000000e+00,prob_range={1.00000000e+00,1.00000000e+00},prob(percent)="100",prob+-sd="100+-0"]:2.244320e-03[&length_mean=2.26950514e-03,length_median=2.24432000e-03,length_95%HPD={1.51514500e-03,3.06142200e-03}],(194[&prob=1.00000000e+00,prob_stddev=0.00000000e+00,prob_range={1.00000000e+00,1.00000000e+00},prob(percent)="100",prob+-sd="100+-0"]:2.035248e-03[&length_mean=2.05349179e-03,length_median=2.03524800e-03,length_95%HPD={1.34444600e-03,2.85694000e-03}],(((205[&prob=1.00000000e+00,prob_stddev=0.00000000e+00,prob_range={1.00000000e+00,1.00000000e+00},prob(percent)="100",prob+-sd="100+-0"]:7.108922e-04[&length_mean=7.34700672e-04,length_median=7.10892200e-04,length_95%HPD={3.24048900e-04,1.19698800e-03}],208[&prob=1.00000000e+00,prob_stddev=0.00000000e+00,prob_range={1.00000000e+00,1.00000000e+00},prob(percent)="100",prob+-sd="100+-0"]:6.344834e-04[&length_mean=6.58591665e-04,length_median=6.34483400e-04,length_95%HPD={2.76613700e-04,1.08757700e-03}])[&prob=1.00000000e+00,prob_stddev=0.00000000e+00,prob_range={1.00000000e+00,1.00000000e+00},prob(percent)="100",prob+-sd="100+-0"]:2.693734e-04[&length_mean=2.92371679e-04,length_median=2.69373400e-04,length_95%HPD={4.93584300e-05,5.85837600e-04}],206[&prob=1.00000000e+00,prob_stddev=0.00000000e+00,prob_range={1.00000000e+00,1.00000000e+00},prob(percent)="100",prob+-sd="100+-0"]:4.140793e-04[&length_mean=4.38214147e-04,length_median=4.14079300e-04,length_95%HPD={1.29281000e-04,7.87530800e-04}])[&prob=1.00000000e+00,prob_stddev=0.00000000e+00,prob_range={1.00000000e+00,1.00000000e+00},prob(percent)="100",prob+-sd="100+-0"]:7.087991e-04[&length_mean=7.35672158e-04,length_median=7.08799100e-04,length_95%HPD={3.07121900e-04,1.18019100e-03}],207[&prob=1.00000000e+00,prob_stddev=0.00000000e+00,prob_range={1.00000000e+00,1.00000000e+00},prob(percent)="100",prob+-sd="100+-0"]:6.931399e-04[&length_mean=7.23579965e-04,length_median=6.93139900e-04,length_95%HPD={2.94162900e-04,1.18432100e-03}])[&prob=1.00000000e+00,prob_stddev=0.00000000e+00,prob_range={1.00000000e+00,1.00000000e+00},prob(percent)="100",prob+-sd="100+-0"]:4.272371e-04[&length_mean=4.52908289e-04,length_median=4.27237100e-04,length_95%HPD={1.20264300e-04,8.23377200e-04}])[&prob=7.93960805e-01,prob_stddev=1.60256170e-03,prob_range={7.92827623e-01,7.95093987e-01},prob(percent)="79",prob+-sd="79+-0"]:1.217207e-04[&length_mean=1.46284932e-04,length_median=1.21720700e-04,length_95%HPD={3.36762000e-08,3.59689000e-04}])[&prob=1.00000000e+00,prob_stddev=0.00000000e+00,prob_range={1.00000000e+00,1.00000000e+00},prob(percent)="100",prob+-sd="100+-0"]:2.852600e-04[&length_mean=3.09447325e-04,length_median=2.85260000e-04,length_95%HPD={4.72520400e-05,6.06204900e-04}],(((((195[&prob=1.00000000e+00,prob_stddev=0.00000000e+00,prob_range={1.00000000e+00,1.00000000e+00},prob(percent)="100",prob+-sd="100+-0"]:1.940015e-04[&length_mean=2.19877336e-04,length_median=1.94001500e-04,length_95%HPD={2.26450400e-05,4.78297700e-04}],198[&prob=1.00000000e+00,prob_stddev=0.00000000e+00,prob_range={1.00000000e+00,1.00000000e+00},prob(percent)="100",prob+-sd="100+-0"]:5.171294e-05[&length_mean=7.40357617e-05,length_median=5.17129400e-05,length_95%HPD={1.60649500e-08,2.21497000e-04}])[&prob=3.40354619e-01,prob_stddev=2.82805005e-03,prob_range={3.38354886e-01,3.42354353e-01},prob(percent)="34",prob+-sd="34+-0"]:4.829731e-05[&length_mean=7.09553241e-05,length_median=4.82973100e-05,length_95%HPD={1.30230600e-08,2.10194300e-04}],200[&prob=1.00000000e+00,prob_stddev=0.00000000e+00,prob_range={1.00000000e+00,1.00000000e+00},prob(percent)="100",prob+-sd="100+-0"]:4.177483e-04[&length_mean=4.38566324e-04,length_median=4.17748300e-04,length_95%HPD={1.20112100e-04,7.90014200e-04}])[&prob=1.00000000e+00,prob_stddev=0.00000000e+00,prob_range={1.00000000e+00,1.00000000e+00},prob(percent)="100",prob+-sd="100+-0"]:2.702329e-04[&length_mean=2.91524865e-04,length_median=2.70232900e-04,length_95%HPD={5.34588500e-05,5.70325800e-04}],199[&prob=1.00000000e+00,prob_stddev=0.00000000e+00,prob_range={1.00000000e+00,1.00000000e+00},prob(percent)="100",prob+-sd="100+-0"]:2.653519e-04[&length_mean=2.87648752e-

04,length_median=2.65351900e-04,length_95%HPD={5.21152500e-05,5.69621800e-
04}])[&prob=3.38688175e-01,prob_stddev=3.48792840e-03,prob_range={3.36221837e-01,3.41154513e-
01},prob(percent)="34",prob+-sd="34+-0"]:5.183948e-05[&length_mean=7.47719577e-
05,length_median=5.18394800e-05,length_95%HPD={3.66449700e-08,2.22027900e-
04}],(196[&prob=1.00000000e+00,prob_stddev=0.00000000e+00,prob_range={1.00000000e+00,1.00000000
e+00},prob(percent)="100",prob+-sd="100+-0"]:3.786816e-04[&length_mean=4.02477185e-
04,length_median=3.78681600e-04,length_95%HPD={9.52664100e-05,7.35987000e-
04}],(197[&prob=1.00000000e+00,prob_stddev=0.00000000e+00,prob_range={1.00000000e+00,1.00000000
e+00},prob(percent)="100",prob+-sd="100+-0"]:1.587841e-04[&length_mean=1.84114363e-
04,length_median=1.58784100e-04,length_95%HPD={7.73890300e-06,4.25255100e-
04}],(201[&prob=1.00000000e+00,prob_stddev=0.00000000e+00,prob_range={1.00000000e+00,1.00000000
e+00},prob(percent)="100",prob+-sd="100+-0"]:4.975104e-05[&length_mean=7.09461628e-
05,length_median=4.97510400e-05,length_95%HPD={2.04101500e-08,2.12959100e-
04}],202[&prob=1.00000000e+00,prob_stddev=0.00000000e+00,prob_range={1.00000000e+00,1.00000000e
+00},prob(percent)="100",prob+-sd="100+-0"]:5.002062e-05[&length_mean=7.17058093e-
05,length_median=5.00206200e-05,length_95%HPD={1.16142400e-08,2.09165100e-
04}])[&prob=4.85668578e-01,prob_stddev=6.22171011e-03,prob_range={4.81269164e-01,4.90067991e-
01},prob(percent)="49",prob+-sd="49+-1"]:8.327436e-05[&length_mean=1.10136039e-
04,length_median=8.32743600e-05,length_95%HPD={3.41518500e-08,3.09753800e-
04}])[&prob=6.84575390e-01,prob_stddev=3.01658672e-03,prob_range={6.82442341e-01,6.86708439e-
01},prob(percent)="68",prob+-sd="68+-0"]:1.223074e-04[&length_mean=1.47081197e-
04,length_median=1.22307400e-04,length_95%HPD={5.06038700e-06,3.54334700e-
04}])[&prob=9.96400480e-01,prob_stddev=2.07390337e-03,prob_range={9.94934009e-01,9.97866951e-
01},prob(percent)="100",prob+-sd="100+-0"]:1.770863e-04[&length_mean=2.00565622e-
04,length_median=1.77086300e-04,length_95%HPD={1.34151300e-05,4.41688800e-
04}])[&prob=1.00000000e+00,prob_stddev=0.00000000e+00,prob_range={1.00000000e+00,1.00000000e+00
},prob(percent)="100",prob+-sd="100+-0"]:7.775235e-04[&length_mean=8.07481381e-
04,length_median=7.77523500e-04,length_95%HPD={3.58738100e-04,1.29188400e-
03}],(203[&prob=1.00000000e+00,prob_stddev=0.00000000e+00,prob_range={1.00000000e+00,1.00000000
e+00},prob(percent)="100",prob+-sd="100+-0"]:1.931109e-04[&length_mean=2.17173346e-
04,length_median=1.93110900e-04,length_95%HPD={2.18781000e-05,4.64019100e-
04}],204[&prob=1.00000000e+00,prob_stddev=0.00000000e+00,prob_range={1.00000000e+00,1.00000000e
+00},prob(percent)="100",prob+-sd="100+-0"]:1.974072e-04[&length_mean=2.21474665e-
04,length_median=1.97407200e-04,length_95%HPD={1.67304700e-05,4.68617600e-
04}])[&prob=1.00000000e+00,prob_stddev=0.00000000e+00,prob_range={1.00000000e+00,1.00000000e+00
},prob(percent)="100",prob+-sd="100+-0"]:1.147701e-03[&length_mean=1.17252745e-
03,length_median=1.14770100e-03,length_95%HPD={6.17504800e-04,1.76033400e-
03}])[&prob=1.00000000e+00,prob_stddev=0.00000000e+00,prob_range={1.00000000e+00,1.00000000e+00
},prob(percent)="100",prob+-sd="100+-0"]:8.376324e-04[&length_mean=8.62585009e-
04,length_median=8.37632400e-04,length_95%HPD={4.07268400e-04,1.36955600e-
03}])[&prob=8.32822290e-01,prob_stddev=8.10707681e-03,prob_range={8.27089721e-01,8.38554859e-
01},prob(percent)="83",prob+-sd="83+-1"]:2.418888e-04[&length_mean=2.67585196e-
04,length_median=2.41888800e-04,length_95%HPD={1.23470000e-07,5.79008300e-
04}],114[&prob=1.00000000e+00,prob_stddev=0.00000000e+00,prob_range={1.00000000e+00,1.00000000e
+00},prob(percent)="100",prob+-sd="100+-0"]:2.620657e-03[&length_mean=2.63685087e-
03,length_median=2.62065700e-03,length_95%HPD={1.77880600e-03,3.51938000e-
03}])[&prob=1.00000000e+00,prob_stddev=0.00000000e+00,prob_range={1.00000000e+00,1.00000000e+00
},prob(percent)="100",prob+-sd="100+-0"]:4.082404e-03[&length_mean=4.10820474e-
03,length_median=4.08240400e-03,length_95%HPD={3.01499600e-03,5.37420400e-
03}])[&prob=1.00000000e+00,prob_stddev=0.00000000e+00,prob_range={1.00000000e+00,1.00000000e+00
},prob(percent)="100",prob+-sd="100+-0"]:3.816498e-03[&length_mean=3.85080675e-
03,length_median=3.81649800e-03,length_95%HPD={2.27622500e-03,5.45441600e-
03}])[&prob=1.00000000e+00,prob_stddev=0.00000000e+00,prob_range={1.00000000e+00,1.00000000e+00
},prob(percent)="100",prob+-sd="100+-0"]:2.324025e-02[&length_mean=2.32582307e-
02,length_median=2.32402500e-02,length_95%HPD={1.93913000e-02,2.68752800e-
02}],((((5[&prob=1.00000000e+00,prob_stddev=0.00000000e+00,prob_range={1.00000000e+00,1.00000000
e+00},prob(percent)="100",prob+-sd="100+-0"]:5.986634e-04[&length_mean=6.30069033e-
04,length_median=5.98663400e-04,length_95%HPD={2.14078300e-04,1.15024000e-
03}],6[&prob=1.00000000e+00,prob_stddev=0.00000000e+00,prob_range={1.00000000e+00,1.00000000e+0
0},prob(percent)="100",prob+-sd="100+-0"]:5.716420e-04[&length_mean=5.99189452e-

04,length_median=5.71642000e-04,length_95%HPD={1.77367600e-04,1.08022700e-
03}])[&prob=1.00000000e+00,prob_stddev=0.00000000e+00,prob_range={1.00000000e+00,1.00000000e+00
},prob(percent)="100",prob+-sd="100+-0"]:3.829463e-02[&length_mean=3.83758288e-
02,length_median=3.82946300e-02,length_95%HPD={3.32463600e-02,4.36926500e-
02}],(37[&prob=1.00000000e+00,prob_stddev=0.00000000e+00,prob_range={1.00000000e+00,1.00000000e
+00},prob(percent)="100",prob+-sd="100+-0"]:2.517163e-02[&length_mean=2.52683188e-
02,length_median=2.51716300e-02,length_95%HPD={2.13201800e-02,2.94401100e-
02}],(115[&prob=1.00000000e+00,prob_stddev=0.00000000e+00,prob_range={1.00000000e+00,1.00000000
e+00},prob(percent)="100",prob+-sd="100+-0"]:1.728433e-02[&length_mean=1.72882192e-
02,length_median=1.72843300e-02,length_95%HPD={1.43639000e-02,2.00625500e-
02}],188[&prob=1.00000000e+00,prob_stddev=0.00000000e+00,prob_range={1.00000000e+00,1.00000000e
+00},prob(percent)="100",prob+-sd="100+-0"]:1.451085e-02[&length_mean=1.45478434e-
02,length_median=1.45108500e-02,length_95%HPD={1.19348500e-02,1.73390500e-
02}])[&prob=1.00000000e+00,prob_stddev=0.00000000e+00,prob_range={1.00000000e+00,1.00000000e+00
},prob(percent)="100",prob+-sd="100+-0"]:1.059727e-02[&length_mean=1.06208719e-
02,length_median=1.05972700e-02,length_95%HPD={7.60505600e-03,1.35570300e-
02}])[&prob=1.00000000e+00,prob_stddev=0.00000000e+00,prob_range={1.00000000e+00,1.00000000e+00
},prob(percent)="100",prob+-sd="100+-0"]:4.021140e-02[&length_mean=4.03194715e-
02,length_median=4.02114000e-02,length_95%HPD={3.47247700e-02,4.62548300e-
02}])[&prob=7.86761765e-01,prob_stddev=6.88158846e-03,prob_range={7.81895747e-01,7.91627783e-
01},prob(percent)="79",prob+-sd="79+-1"]:3.887476e-03[&length_mean=3.95281714e-
03,length_median=3.88747600e-03,length_95%HPD={1.38772300e-03,6.71106600e-
03}],(((((((((((7[&prob=1.00000000e+00,prob_stddev=0.00000000e+00,prob_range={1.00000000e+00,1.000
00000e+00},prob(percent)="100",prob+-sd="100+-0"]:1.778665e-03[&length_mean=1.80348526e-
03,length_median=1.77866500e-03,length_95%HPD={1.09166300e-03,2.54661900e-
03}],93[&prob=1.00000000e+00,prob_stddev=0.00000000e+00,prob_range={1.00000000e+00,1.00000000e+
00},prob(percent)="100",prob+-sd="100+-0"]:1.998706e-03[&length_mean=2.02577416e-
03,length_median=1.99870600e-03,length_95%HPD={1.29110400e-03,2.82718600e-
03}])[&prob=1.00000000e+00,prob_stddev=0.00000000e+00,prob_range={1.00000000e+00,1.00000000e+00
},prob(percent)="100",prob+-sd="100+-0"]:3.787208e-03[&length_mean=3.81376694e-
03,length_median=3.78720800e-03,length_95%HPD={2.70510700e-03,4.89254300e-
03}],((90[&prob=1.00000000e+00,prob_stddev=0.00000000e+00,prob_range={1.00000000e+00,1.00000000e
+00},prob(percent)="100",prob+-sd="100+-0"]:7.463032e-04[&length_mean=7.75884399e-
04,length_median=7.46303200e-04,length_95%HPD={3.30551900e-04,1.27703700e-
03}],91[&prob=1.00000000e+00,prob_stddev=0.00000000e+00,prob_range={1.00000000e+00,1.00000000e+
00},prob(percent)="100",prob+-sd="100+-0"]:5.162855e-04[&length_mean=5.41568412e-
04,length_median=5.16285500e-04,length_95%HPD={1.76803000e-04,9.35846100e-
04}])[&prob=1.00000000e+00,prob_stddev=0.00000000e+00,prob_range={1.00000000e+00,1.00000000e+00
},prob(percent)="100",prob+-sd="100+-0"]:1.364914e-03[&length_mean=1.39339867e-
03,length_median=1.36491400e-03,length_95%HPD={8.09940800e-04,2.06593000e-
03}],92[&prob=1.00000000e+00,prob_stddev=0.00000000e+00,prob_range={1.00000000e+00,1.00000000e+
00},prob(percent)="100",prob+-sd="100+-0"]:2.148038e-03[&length_mean=2.17303239e-
03,length_median=2.14803800e-03,length_95%HPD={1.43078700e-03,2.96782100e-
03}])[&prob=1.00000000e+00,prob_stddev=0.00000000e+00,prob_range={1.00000000e+00,1.00000000e+00
},prob(percent)="100",prob+-sd="100+-0"]:2.765829e-03[&length_mean=2.79759381e-
03,length_median=2.76582900e-03,length_95%HPD={1.88673100e-03,3.79132000e-
03}])[&prob=1.00000000e+00,prob_stddev=0.00000000e+00,prob_range={1.00000000e+00,1.00000000e+00
},prob(percent)="100",prob+-sd="100+-0"]:2.928909e-03[&length_mean=2.96493427e-
03,length_median=2.92890900e-03,length_95%HPD={1.95676000e-03,4.02265300e-
03}],46[&prob=1.00000000e+00,prob_stddev=0.00000000e+00,prob_range={1.00000000e+00,1.00000000e+
00},prob(percent)="100",prob+-sd="100+-0"]:8.905854e-03[&length_mean=8.93027451e-
03,length_median=8.90585400e-03,length_95%HPD={7.25950600e-03,1.07260000e-
02}])[&prob=1.00000000e+00,prob_stddev=0.00000000e+00,prob_range={1.00000000e+00,1.00000000e+00
},prob(percent)="100",prob+-sd="100+-0"]:2.837834e-03[&length_mean=2.86386638e-
03,length_median=2.83783400e-03,length_95%HPD={1.85841800e-03,3.95820700e-
03}],89[&prob=1.00000000e+00,prob_stddev=0.00000000e+00,prob_range={1.00000000e+00,1.00000000e+
00},prob(percent)="100",prob+-sd="100+-0"]:1.254727e-02[&length_mean=1.25591111e-
02,length_median=1.25472700e-02,length_95%HPD={1.04778800e-02,1.47473400e-
02}])[&prob=1.00000000e+00,prob_stddev=0.00000000e+00,prob_range={1.00000000e+00,1.00000000e+00
},prob(percent)="100",prob+-sd="100+-0"]:2.352735e-03[&length_mean=2.37572358e-

03,length_median=2.35273500e-03,length_95%HPD={1.43025800e-03,3.39522500e-
03}],(57[&prob=1.00000000e+00,prob_stddev=0.00000000e+00,prob_range={1.00000000e+00,1.00000000e
+00},prob(percent)="100",prob+-sd="100+-0"]:1.933171e-02[&length_mean=1.93599228e-
02,length_median=1.93317100e-02,length_95%HPD={1.68375600e-02,2.22232900e-
02}],(77[&prob=1.00000000e+00,prob_stddev=0.00000000e+00,prob_range={1.00000000e+00,1.00000000e
+00},prob(percent)="100",prob+-sd="100+-0"]:7.574854e-03[&length_mean=7.58653966e-
03,length_median=7.57485400e-03,length_95%HPD={5.93934800e-03,9.26295800e-
03}],166[&prob=1.00000000e+00,prob_stddev=0.00000000e+00,prob_range={1.00000000e+00,1.00000000e
+00},prob(percent)="100",prob+-sd="100+-0"]:8.247012e-03[&length_mean=8.28112844e-
03,length_median=8.24701200e-03,length_95%HPD={6.50148600e-03,1.00266800e-
02}])[&prob=1.00000000e+00,prob_stddev=0.00000000e+00,prob_range={1.00000000e+00,1.00000000e+00
},prob(percent)="100",prob+-sd="100+-0"]:1.092475e-02[&length_mean=1.09551288e-
02,length_median=1.09247500e-02,length_95%HPD={8.88030900e-03,1.30771800e-
02}])[&prob=9.99200107e-01,prob_stddev=3.77073340e-04,prob_range={9.98933476e-01,9.99466738e-
01},prob(percent)="100",prob+-sd="100+-0"]:1.473054e-03[&length_mean=1.51119288e-
03,length_median=1.47305400e-03,length_95%HPD={5.44128400e-04,2.56428100e-
03}])[&prob=8.39888015e-01,prob_stddev=6.22171011e-03,prob_range={8.35488602e-01,8.44287428e-
01},prob(percent)="84",prob+-sd="84+-1"]:4.748062e-04[&length_mean=5.10765042e-
04,length_median=4.74806200e-04,length_95%HPD={8.70749700e-05,1.01024600e-
03}],((((((123[&prob=1.00000000e+00,prob_stddev=0.00000000e+00,prob_range={1.00000000e+00,1.00000
000e+00},prob(percent)="100",prob+-sd="100+-0"]:3.247297e-03[&length_mean=3.27021585e-
03,length_median=3.24729700e-03,length_95%HPD={2.28104800e-03,4.25968400e-
03}],130[&prob=1.00000000e+00,prob_stddev=0.00000000e+00,prob_range={1.00000000e+00,1.00000000e
+00},prob(percent)="100",prob+-sd="100+-0"]:4.816587e-03[&length_mean=4.84960830e-
03,length_median=4.81658700e-03,length_95%HPD={3.65200900e-03,6.06311700e-
03}])[&prob=1.00000000e+00,prob_stddev=0.00000000e+00,prob_range={1.00000000e+00,1.00000000e+00
},prob(percent)="100",prob+-sd="100+-0"]:2.665382e-03[&length_mean=2.69327294e-
03,length_median=2.66538200e-03,length_95%HPD={1.80175900e-03,3.69749400e-
03}],((((125[&prob=1.00000000e+00,prob_stddev=0.00000000e+00,prob_range={1.00000000e+00,1.000000
00e+00},prob(percent)="100",prob+-sd="100+-0"]:5.617955e-04[&length_mean=5.84993690e-
04,length_median=5.61795500e-04,length_95%HPD={2.23519700e-04,1.00908700e-
03}],131[&prob=1.00000000e+00,prob_stddev=0.00000000e+00,prob_range={1.00000000e+00,1.00000000e
+00},prob(percent)="100",prob+-sd="100+-0"]:4.843453e-04[&length_mean=5.08039889e-
04,length_median=4.84345300e-04,length_95%HPD={1.80084500e-04,9.10873200e-
04}])[&prob=1.00000000e+00,prob_stddev=0.00000000e+00,prob_range={1.00000000e+00,1.00000000e+00
},prob(percent)="100",prob+-sd="100+-0"]:2.646844e-04[&length_mean=2.89074842e-
04,length_median=2.64684400e-04,length_95%HPD={5.28809100e-05,5.87174300e-
04}],129[&prob=1.00000000e+00,prob_stddev=0.00000000e+00,prob_range={1.00000000e+00,1.00000000e
+00},prob(percent)="100",prob+-sd="100+-0"]:8.393297e-04[&length_mean=8.66203954e-
04,length_median=8.39329700e-04,length_95%HPD={4.06263200e-04,1.35847200e-
03}])[&prob=1.00000000e+00,prob_stddev=0.00000000e+00,prob_range={1.00000000e+00,1.00000000e+00
},prob(percent)="100",prob+-sd="100+-0"]:8.164444e-04[&length_mean=8.46123254e-
04,length_median=8.16444400e-04,length_95%HPD={3.96106600e-04,1.37828400e-
03}],(132[&prob=1.00000000e+00,prob_stddev=0.00000000e+00,prob_range={1.00000000e+00,1.00000000
e+00},prob(percent)="100",prob+-sd="100+-0"]:1.003468e-03[&length_mean=1.02682491e-
03,length_median=1.00346800e-03,length_95%HPD={5.11577700e-04,1.53047100e-
03}],133[&prob=1.00000000e+00,prob_stddev=0.00000000e+00,prob_range={1.00000000e+00,1.00000000e
+00},prob(percent)="100",prob+-sd="100+-0"]:4.101264e-04[&length_mean=4.33073133e-
04,length_median=4.10126400e-04,length_95%HPD={1.17731400e-04,7.69841300e-
04}])[&prob=1.00000000e+00,prob_stddev=0.00000000e+00,prob_range={1.00000000e+00,1.00000000e+00
},prob(percent)="100",prob+-sd="100+-0"]:1.180570e-03[&length_mean=1.20577274e-
03,length_median=1.18057000e-03,length_95%HPD={6.21644600e-04,1.80761800e-
03}])[&prob=1.00000000e+00,prob_stddev=0.00000000e+00,prob_range={1.00000000e+00,1.00000000e+00
},prob(percent)="100",prob+-sd="100+-0"]:1.275324e-03[&length_mean=1.30398054e-
03,length_median=1.27532400e-03,length_95%HPD={7.42189300e-04,2.01310900e-
03}],127[&prob=1.00000000e+00,prob_stddev=0.00000000e+00,prob_range={1.00000000e+00,1.00000000e
+00},prob(percent)="100",prob+-sd="100+-0"]:2.957961e-03[&length_mean=2.99867996e-
03,length_median=2.95796100e-03,length_95%HPD={2.03536300e-03,3.96408400e-
03}])[&prob=1.00000000e+00,prob_stddev=0.00000000e+00,prob_range={1.00000000e+00,1.00000000e+00
},prob(percent)="100",prob+-sd="100+-0"]:3.428885e-03[&length_mean=3.45658479e-

03,length_median=3.42888500e-03,length_95%HPD={2.47759400e-03,4.55782200e-
03}])[&prob=1.00000000e+00,prob_stddev=0.00000000e+00,prob_range={1.00000000e+00,1.00000000e+00
},prob(percent)="100",prob+-sd="100+-0"]:2.445534e-03[&length_mean=2.47663482e-
03,length_median=2.44553400e-03,length_95%HPD={1.55475900e-03,3.38173500e-
03}],124[&prob=1.00000000e+00,prob_stddev=0.00000000e+00,prob_range={1.00000000e+00,1.00000000e
+00},prob(percent)="100",prob+-sd="100+-0"]:4.618832e-03[&length_mean=4.63596533e-
03,length_median=4.61883200e-03,length_95%HPD={3.36880100e-03,5.87455900e-
03}])[&prob=1.00000000e+00,prob_stddev=0.00000000e+00,prob_range={1.00000000e+00,1.00000000e+00
},prob(percent)="100",prob+-sd="100+-0"]:2.695872e-03[&length_mean=2.72006646e-
03,length_median=2.69587200e-03,length_95%HPD={1.68866600e-03,3.73607500e-
03}],128[&prob=1.00000000e+00,prob_stddev=0.00000000e+00,prob_range={1.00000000e+00,1.00000000e
+00},prob(percent)="100",prob+-sd="100+-0"]:9.921786e-03[&length_mean=9.94474971e-
03,length_median=9.92178600e-03,length_95%HPD={8.03103400e-03,1.17662500e-
02}])[&prob=1.00000000e+00,prob_stddev=0.00000000e+00,prob_range={1.00000000e+00,1.00000000e+00
},prob(percent)="100",prob+-sd="100+-0"]:1.554092e-03[&length_mean=1.58836218e-
03,length_median=1.55409200e-03,length_95%HPD={8.22985100e-04,2.47071200e-
03}],209[&prob=1.00000000e+00,prob_stddev=0.00000000e+00,prob_range={1.00000000e+00,1.00000000e
+00},prob(percent)="100",prob+-sd="100+-0"]:1.163084e-02[&length_mean=1.16469186e-
02,length_median=1.16308400e-02,length_95%HPD={9.74921300e-03,1.36879200e-
02}])[&prob=1.00000000e+00,prob_stddev=0.00000000e+00,prob_range={1.00000000e+00,1.00000000e+00
},prob(percent)="100",prob+-sd="100+-0"]:3.237225e-03[&length_mean=3.27066288e-
03,length_median=3.23722500e-03,length_95%HPD={2.12031700e-03,4.48353900e-
03}],(126[&prob=1.00000000e+00,prob_stddev=0.00000000e+00,prob_range={1.00000000e+00,1.00000000
e+00},prob(percent)="100",prob+-sd="100+-0"]:4.644800e-03[&length_mean=4.68390174e-
03,length_median=4.64480000e-03,length_95%HPD={3.47306600e-03,6.03436400e-
03}],138[&prob=1.00000000e+00,prob_stddev=0.00000000e+00,prob_range={1.00000000e+00,1.00000000e
+00},prob(percent)="100",prob+-sd="100+-0"]:6.066759e-03[&length_mean=6.11457140e-
03,length_median=6.06675900e-03,length_95%HPD={4.73859800e-03,7.60563700e-
03}])[&prob=1.00000000e+00,prob_stddev=0.00000000e+00,prob_range={1.00000000e+00,1.00000000e+00
},prob(percent)="100",prob+-sd="100+-0"]:7.114219e-03[&length_mean=7.14038096e-
03,length_median=7.11421900e-03,length_95%HPD={5.54135600e-03,8.72194400e-
03}])[&prob=1.00000000e+00,prob_stddev=0.00000000e+00,prob_range={1.00000000e+00,1.00000000e+00
},prob(percent)="100",prob+-sd="100+-0"]:7.351893e-03[&length_mean=7.39407301e-
03,length_median=7.35189300e-03,length_95%HPD={5.81519700e-03,9.12970500e-
03}])[&prob=1.00000000e+00,prob_stddev=0.00000000e+00,prob_range={1.00000000e+00,1.00000000e+00
},prob(percent)="100",prob+-sd="100+-0"]:2.300785e-03[&length_mean=2.33214802e-
03,length_median=2.30078500e-03,length_95%HPD={1.36251000e-03,3.34160500e-
03}],((111[&prob=1.00000000e+00,prob_stddev=0.00000000e+00,prob_range={1.00000000e+00,1.00000000
e+00},prob(percent)="100",prob+-sd="100+-0"]:1.296500e-03[&length_mean=1.31698816e-
03,length_median=1.29650000e-03,length_95%HPD={7.62494400e-04,1.98098500e-
03}],113[&prob=1.00000000e+00,prob_stddev=0.00000000e+00,prob_range={1.00000000e+00,1.00000000e
+00},prob(percent)="100",prob+-sd="100+-0"]:5.986339e-04[&length_mean=6.21510782e-
04,length_median=5.98633900e-04,length_95%HPD={2.31811500e-04,1.05652000e-
03}])[&prob=5.09465405e-01,prob_stddev=6.59878345e-04,prob_range={5.08998800e-01,5.09932009e-
01},prob(percent)="51",prob+-sd="51+-0"]:1.706058e-04[&length_mean=1.93965143e-
04,length_median=1.70605800e-04,length_95%HPD={1.25331400e-07,4.43670300e-
04}],112[&prob=1.00000000e+00,prob_stddev=0.00000000e+00,prob_range={1.00000000e+00,1.00000000e
+00},prob(percent)="100",prob+-sd="100+-0"]:6.330168e-04[&length_mean=6.62996690e-
04,length_median=6.33016800e-04,length_95%HPD={2.64623300e-04,1.15902900e-
03}])[&prob=1.00000000e+00,prob_stddev=0.00000000e+00,prob_range={1.00000000e+00,1.00000000e+00
},prob(percent)="100",prob+-sd="100+-0"]:1.271292e-02[&length_mean=1.27673590e-
02,length_median=1.27129200e-02,length_95%HPD={1.07153800e-02,1.49698300e-
02}])[&prob=1.00000000e+00,prob_stddev=0.00000000e+00,prob_range={1.00000000e+00,1.00000000e+00
},prob(percent)="100",prob+-sd="100+-0"]:1.613139e-03[&length_mean=1.64186568e-
03,length_median=1.61313900e-03,length_95%HPD={7.64077100e-04,2.59778900e-
03}],110[&prob=1.00000000e+00,prob_stddev=0.00000000e+00,prob_range={1.00000000e+00,1.00000000e
+00},prob(percent)="100",prob+-sd="100+-0"]:3.080182e-02[&length_mean=3.08445064e-
02,length_median=3.08018200e-02,length_95%HPD={2.70969600e-02,3.46962700e-
02}])[&prob=5.19664045e-01,prob_stddev=1.50829336e-02,prob_range={5.08998800e-01,5.30329289e-
01},prob(percent)="52",prob+-sd="52+-2"]:6.573151e-04[&length_mean=6.95562627e-

04,length_median=6.57315100e-04,length_95%HPD={6.00309200e-06,1.35630200e-03}],((63[&prob=1.00000000e+00,prob_stddev=0.00000000e+00,prob_range={1.00000000e+00,1.00000000e+00},prob(percent)="100",prob+-sd="100+-0"]:6.660745e-03[&length_mean=6.67676074e-03,length_median=6.66074500e-03,length_95%HPD={5.22970500e-03,8.07221200e-03}],(((100[&prob=1.00000000e+00,prob_stddev=0.00000000e+00,prob_range={1.00000000e+00,1.00000000e+00},prob(percent)="100",prob+-sd="100+-0"]:1.491467e-03[&length_mean=1.52087426e-03,length_median=1.49146700e-03,length_95%HPD={8.69875800e-04,2.17224500e-03}],((103[&prob=1.00000000e+00,prob_stddev=0.00000000e+00,prob_range={1.00000000e+00,1.00000000e+00},prob(percent)="100",prob+-sd="100+-0"]:6.348076e-04[&length_mean=6.59880592e-04,length_median=6.34807600e-04,length_95%HPD={2.76392100e-04,1.10394300e-03}],106[&prob=1.00000000e+00,prob_stddev=0.00000000e+00,prob_range={1.00000000e+00,1.00000000e+00},prob(percent)="100",prob+-sd="100+-0"]:2.675476e-04[&length_mean=2.90672692e-04,length_median=2.67547600e-04,length_95%HPD={5.00867900e-05,5.71433300e-04}])[&prob=1.00000000e+00,prob_stddev=0.00000000e+00,prob_range={1.00000000e+00,1.00000000e+00},prob(percent)="100",prob+-sd="100+-0"]:4.844155e-04[&length_mean=5.08243661e-04,length_median=4.84415500e-04,length_95%HPD={1.79099600e-04,8.95199400e-04}],104[&prob=1.00000000e+00,prob_stddev=0.00000000e+00,prob_range={1.00000000e+00,1.00000000e+00},prob(percent)="100",prob+-sd="100+-0"]:1.224013e-03[&length_mean=1.24593902e-03,length_median=1.22401300e-03,length_95%HPD={6.85724300e-04,1.86760700e-03}])[&prob=8.83282229e-01,prob_stddev=4.05353841e-03,prob_range={8.80415945e-01,8.86148514e-01},prob(percent)="88",prob+-sd="88+-0"]:1.415473e-04[&length_mean=1.67463921e-04,length_median=1.41547300e-04,length_95%HPD={8.08002700e-08,4.03384900e-04}])[&prob=1.00000000e+00,prob_stddev=0.00000000e+00,prob_range={1.00000000e+00,1.00000000e+00},prob(percent)="100",prob+-sd="100+-0"]:9.607696e-04[&length_mean=9.83750467e-04,length_median=9.60769600e-04,length_95%HPD={4.72873200e-04,1.56918600e-03}],102[&prob=1.00000000e+00,prob_stddev=0.00000000e+00,prob_range={1.00000000e+00,1.00000000e+00},prob(percent)="100",prob+-sd="100+-0"]:3.431328e-03[&length_mean=3.45729826e-03,length_median=3.43132800e-03,length_95%HPD={2.52044800e-03,4.51936700e-03}])[&prob=1.00000000e+00,prob_stddev=0.00000000e+00,prob_range={1.00000000e+00,1.00000000e+00},prob(percent)="100",prob+-sd="100+-0"]:3.552138e-03[&length_mean=3.57954040e-03,length_median=3.55213800e-03,length_95%HPD={2.55957800e-03,4.68769400e-03}],101[&prob=1.00000000e+00,prob_stddev=0.00000000e+00,prob_range={1.00000000e+00,1.00000000e+00},prob(percent)="100",prob+-sd="100+-0"]:7.086125e-03[&length_mean=7.12232080e-03,length_median=7.08612500e-03,length_95%HPD={5.53812500e-03,8.58641100e-03}])[&prob=9.98533529e-01,prob_stddev=0.00000000e+00,prob_range={9.98533529e-01,9.98533529e-01},prob(percent)="100",prob+-sd="100+-0"]:8.637298e-04[&length_mean=8.92332273e-04,length_median=8.63729800e-04,length_95%HPD={2.79993500e-04,1.52322100e-03}])[&prob=1.00000000e+00,prob_stddev=0.00000000e+00,prob_range={1.00000000e+00,1.00000000e+00},prob(percent)="100",prob+-sd="100+-0"]:1.321287e-03[&length_mean=1.35550659e-03,length_median=1.32128700e-03,length_95%HPD={6.52809900e-04,2.11736800e-03}],105[&prob=1.00000000e+00,prob_stddev=0.00000000e+00,prob_range={1.00000000e+00,1.00000000e+00},prob(percent)="100",prob+-sd="100+-0"]:7.436461e-03[&length_mean=7.48362805e-03,length_median=7.43646100e-03,length_95%HPD={5.98427000e-03,9.08801000e-03}])[&prob=1.00000000e+00,prob_stddev=0.00000000e+00,prob_range={1.00000000e+00,1.00000000e+00},prob(percent)="100",prob+-sd="100+-0"]:4.406191e-03[&length_mean=4.44084815e-03,length_median=4.40619100e-03,length_95%HPD={3.16098200e-03,5.77818300e-03}])[&prob=6.65511265e-01,prob_stddev=1.92307403e-02,prob_range={6.51913078e-01,6.79109452e-01},prob(percent)="67",prob+-sd="67+-2"]:1.043452e-03[&length_mean=1.08195380e-03,length_median=1.04345200e-03,length_95%HPD={2.59604700e-04,1.95848000e-03}],(16[&prob=1.00000000e+00,prob_stddev=0.00000000e+00,prob_range={1.00000000e+00,1.00000000e+00},prob(percent)="100",prob+-sd="100+-0"]:5.102827e-03[&length_mean=5.13228977e-03,length_median=5.10282700e-03,length_95%HPD={3.75324400e-03,6.48039200e-03}],((107[&prob=1.00000000e+00,prob_stddev=0.00000000e+00,prob_range={1.00000000e+00,1.00000000e+00},prob(percent)="100",prob+-sd="100+-0"]:1.878153e-03[&length_mean=1.91247070e-03,length_median=1.87815300e-03,length_95%HPD={1.18375700e-03,2.68984800e-03}],108[&prob=1.00000000e+00,prob_stddev=0.00000000e+00,prob_range={1.00000000e+00,1.00000000e+00},prob(percent)="100",prob+-sd="100+-0"]:8.481843e-04[&length_mean=8.74124039e-04,length_median=8.48184300e-04,length_95%HPD={3.91061800e-04,1.38905400e-03}])[&prob=1.00000000e+00,prob_stddev=0.00000000e+00,prob_range={1.00000000e+00,1.00000000e+00},prob(percent)="100",prob+-sd="100+-0"]:2.316248e-03[&length_mean=2.34399291e-

03,length_median=2.31624800e-03,length_95%HPD={1.56581400e-03,3.23754600e-
03}],109[&prob=1.00000000e+00,prob_stddev=0.00000000e+00,prob_range={1.00000000e+00,1.00000000e
+00},prob(percent)="100",prob+-sd="100+-0"]:3.383210e-03[&length_mean=3.41647451e-
03,length_median=3.38321000e-03,length_95%HPD={2.43019900e-03,4.47984400e-
03}])[&prob=1.00000000e+00,prob_stddev=0.00000000e+00,prob_range={1.00000000e+00,1.00000000e+00
},prob(percent)="100",prob+-sd="100+-0"]:2.033328e-03[&length_mean=2.06132984e-
03,length_median=2.03332800e-03,length_95%HPD={1.15847900e-03,2.98238000e-
03}])[&prob=1.00000000e+00,prob_stddev=0.00000000e+00,prob_range={1.00000000e+00,1.00000000e+00
},prob(percent)="100",prob+-sd="100+-0"]:1.547040e-02[&length_mean=1.55273264e-
02,length_median=1.54704000e-02,length_95%HPD={1.31885400e-02,1.81061800e-
02}])[&prob=9.99000133e-01,prob_stddev=8.48415015e-04,prob_range={9.98400213e-01,9.99600053e-
01},prob(percent)="100",prob+-sd="100+-0"]:3.019414e-03[&length_mean=3.04508894e-
03,length_median=3.01941400e-03,length_95%HPD={1.31513100e-03,4.72301900e-
03}],190[&prob=1.00000000e+00,prob_stddev=0.00000000e+00,prob_range={1.00000000e+00,1.00000000e
+00},prob(percent)="100",prob+-sd="100+-0"]:2.404754e-02[&length_mean=2.41665748e-
02,length_median=2.40475400e-02,length_95%HPD={2.08787700e-02,2.77230200e-
02}])[&prob=1.00000000e+00,prob_stddev=0.00000000e+00,prob_range={1.00000000e+00,1.00000000e+00
},prob(percent)="100",prob+-sd="100+-0"]:2.279716e-02[&length_mean=2.28634727e-
02,length_median=2.27971600e-02,length_95%HPD={1.93911100e-02,2.65327100e-
02}],(((19[&prob=1.00000000e+00,prob_stddev=0.00000000e+00,prob_range={1.00000000e+00,1.00000000
e+00},prob(percent)="100",prob+-sd="100+-0"]:1.304993e-02[&length_mean=1.31132895e-
02,length_median=1.30499300e-02,length_95%HPD={1.06648800e-02,1.56345200e-
02}],135[&prob=1.00000000e+00,prob_stddev=0.00000000e+00,prob_range={1.00000000e+00,1.00000000e
+00},prob(percent)="100",prob+-sd="100+-0"]:1.923494e-02[&length_mean=1.92828963e-
02,length_median=1.92349400e-02,length_95%HPD={1.64162000e-02,2.24539900e-
02}])[&prob=1.00000000e+00,prob_stddev=0.00000000e+00,prob_range={1.00000000e+00,1.00000000e+00
},prob(percent)="100",prob+-sd="100+-0"]:1.692707e-02[&length_mean=1.70120692e-
02,length_median=1.69270700e-02,length_95%HPD={1.41373000e-02,2.01053400e-
02}],(20[&prob=1.00000000e+00,prob_stddev=0.00000000e+00,prob_range={1.00000000e+00,1.00000000e
+00},prob(percent)="100",prob+-sd="100+-0"]:7.307314e-03[&length_mean=7.36043214e-
03,length_median=7.30731400e-03,length_95%HPD={5.74228500e-03,9.29478900e-
03}],(176[&prob=1.00000000e+00,prob_stddev=0.00000000e+00,prob_range={1.00000000e+00,1.00000000
e+00},prob(percent)="100",prob+-sd="100+-0"]:5.344849e-04[&length_mean=5.65738569e-
04,length_median=5.34484900e-04,length_95%HPD={1.82584600e-04,9.97536300e-
04}],177[&prob=1.00000000e+00,prob_stddev=0.00000000e+00,prob_range={1.00000000e+00,1.00000000e
+00},prob(percent)="100",prob+-sd="100+-0"]:2.782472e-04[&length_mean=3.03390079e-
04,length_median=2.78247200e-04,length_95%HPD={2.37007300e-05,6.27076600e-
04}])[&prob=1.00000000e+00,prob_stddev=0.00000000e+00,prob_range={1.00000000e+00,1.00000000e+00
},prob(percent)="100",prob+-sd="100+-0"]:8.763971e-03[&length_mean=8.76713838e-
03,length_median=8.76397100e-03,length_95%HPD={6.98861200e-03,1.07453600e-
02}])[&prob=1.00000000e+00,prob_stddev=0.00000000e+00,prob_range={1.00000000e+00,1.00000000e+00
},prob(percent)="100",prob+-sd="100+-0"]:1.938484e-02[&length_mean=1.94432702e-
02,length_median=1.93848400e-02,length_95%HPD={1.62945900e-02,2.28052800e-
02}])[&prob=1.00000000e+00,prob_stddev=0.00000000e+00,prob_range={1.00000000e+00,1.00000000e+00
},prob(percent)="100",prob+-sd="100+-0"]:7.626890e-03[&length_mean=7.66930049e-
03,length_median=7.62689000e-03,length_95%HPD={5.32350200e-03,1.01514600e-
02}],167[&prob=1.00000000e+00,prob_stddev=0.00000000e+00,prob_range={1.00000000e+00,1.00000000e
+00},prob(percent)="100",prob+-sd="100+-0"]:2.264695e-02[&length_mean=2.27059052e-
02,length_median=2.26469500e-02,length_95%HPD={1.95446500e-02,2.61244600e-
02}])[&prob=1.00000000e+00,prob_stddev=0.00000000e+00,prob_range={1.00000000e+00,1.00000000e+00
},prob(percent)="100",prob+-sd="100+-0"]:6.217958e-03[&length_mean=6.26266127e-
03,length_median=6.21795800e-03,length_95%HPD={4.10873900e-03,8.36186800e-
03}])[&prob=1.00000000e+00,prob_stddev=0.00000000e+00,prob_range={1.00000000e+00,1.00000000e+00
},prob(percent)="100",prob+-sd="100+-0"]:5.941557e-03[&length_mean=5.96723090e-
03,length_median=5.94155700e-03,length_95%HPD={3.58919300e-03,8.33860600e-
03}])[&prob=1.00000000e+00,prob_stddev=0.00000000e+00,prob_range={1.00000000e+00,1.00000000e+00
},prob(percent)="100",prob+-sd="100+-0"]:9.368401e-03[&length_mean=9.42148452e-
03,length_median=9.36840100e-03,length_95%HPD={6.61123100e-03,1.21857700e-
02}],(((((((((9[&prob=1.00000000e+00,prob_stddev=0.00000000e+00,prob_range={1.00000000e+00,1.0000
0000e+00},prob(percent)="100",prob+-sd="100+-0"]:4.315069e-04[&length_mean=4.58539728e-

04,length_median=4.31506900e-04,length_95%HPD={1.31544900e-04,8.63100600e-04}],10[&prob=1.00000000e+00,prob_stddev=0.00000000e+00,prob_range={1.00000000e+00,1.00000000e+00},prob(percent)="100",prob+-sd="100+-0"]:3.847684e-04[&length_mean=4.09452982e-04,length_median=3.84768400e-04,length_95%HPD={8.40794700e-05,7.76934300e-04}])[&prob=9.95467271e-01,prob_stddev=7.54146680e-04,prob_range={9.94934009e-01,9.96000533e-01},prob(percent)="100",prob+-sd="100+-0"]:3.350828e-04[&length_mean=3.57390754e-04,length_median=3.35082800e-04,length_95%HPD={8.32615800e-05,6.75894500e-04}],11[&prob=1.00000000e+00,prob_stddev=0.00000000e+00,prob_range={1.00000000e+00,1.00000000e+00},prob(percent)="100",prob+-sd="100+-0"]:1.045741e-03[&length_mean=1.07321627e-03,length_median=1.04574100e-03,length_95%HPD={5.45507100e-04,1.64862000e-03}])[&prob=1.00000000e+00,prob_stddev=0.00000000e+00,prob_range={1.00000000e+00,1.00000000e+00},prob(percent)="100",prob+-sd="100+-0"]:7.103601e-04[&length_mean=7.36650498e-04,length_median=7.10360100e-04,length_95%HPD={2.73086200e-04,1.21010200e-03}],14[&prob=1.00000000e+00,prob_stddev=0.00000000e+00,prob_range={1.00000000e+00,1.00000000e+00},prob(percent)="100",prob+-sd="100+-0"]:1.593789e-03[&length_mean=1.61686925e-03,length_median=1.59378900e-03,length_95%HPD={9.71749500e-04,2.31555500e-03}])[&prob=5.72123717e-01,prob_stddev=1.06523219e-02,prob_range={5.64591388e-01,5.79656046e-01},prob(percent)="57",prob+-sd="57+-1"]:1.081766e-04[&length_mean=1.30864243e-04,length_median=1.08176600e-04,length_95%HPD={1.54589300e-08,3.31533400e-04}],12[&prob=1.00000000e+00,prob_stddev=0.00000000e+00,prob_range={1.00000000e+00,1.00000000e+00},prob(percent)="100",prob+-sd="100+-0"]:1.327935e-03[&length_mean=1.35363134e-03,length_median=1.32793500e-03,length_95%HPD={7.28129100e-04,1.96957100e-03}])[&prob=1.00000000e+00,prob_stddev=0.00000000e+00,prob_range={1.00000000e+00,1.00000000e+00},prob(percent)="100",prob+-sd="100+-0"]:4.922023e-04[&length_mean=5.16085736e-04,length_median=4.92202300e-04,length_95%HPD={1.75194400e-04,8.97885100e-04}],165[&prob=1.00000000e+00,prob_stddev=0.00000000e+00,prob_range={1.00000000e+00,1.00000000e+00},prob(percent)="100",prob+-sd="100+-0"]:2.349984e-03[&length_mean=2.36557336e-03,length_median=2.34998400e-03,length_95%HPD={1.55336700e-03,3.23324900e-03}])[&prob=4.43607519e-01,prob_stddev=5.75036844e-03,prob_range={4.39541394e-01,4.47673644e-01},prob(percent)="44",prob+-sd="44+-1"]:9.525386e-05[&length_mean=1.21101840e-04,length_median=9.52538600e-05,length_95%HPD={4.56624600e-08,3.22055900e-04}],((13[&prob=1.00000000e+00,prob_stddev=0.00000000e+00,prob_range={1.00000000e+00,1.00000000e+00},prob(percent)="100",prob+-sd="100+-0"]:2.687326e-03[&length_mean=2.70476547e-03,length_median=2.68732600e-03,length_95%HPD={1.85379300e-03,3.63412900e-03}],137[&prob=1.00000000e+00,prob_stddev=0.00000000e+00,prob_range={1.00000000e+00,1.00000000e+00},prob(percent)="100",prob+-sd="100+-0"]:2.238416e-03[&length_mean=2.26590774e-03,length_median=2.23841600e-03,length_95%HPD={1.49440700e-03,3.13043600e-03}])[&prob=9.60805226e-01,prob_stddev=8.86122349e-03,prob_range={9.54539395e-01,9.67071057e-01},prob(percent)="96",prob+-sd="96+-1"]:2.673300e-04[&length_mean=2.91648129e-04,length_median=2.67330000e-04,length_95%HPD={3.54525800e-05,6.05457600e-04}],142[&prob=1.00000000e+00,prob_stddev=0.00000000e+00,prob_range={1.00000000e+00,1.00000000e+00},prob(percent)="100",prob+-sd="100+-0"]:4.156530e-03[&length_mean=4.17333191e-03,length_median=4.15653000e-03,length_95%HPD={3.11629900e-03,5.35377300e-03}])[&prob=6.86508466e-01,prob_stddev=5.37329510e-03,prob_range={6.82708972e-01,6.90307959e-01},prob(percent)="69",prob+-sd="69+-1"]:1.244196e-04[&length_mean=1.48363894e-04,length_median=1.24419600e-04,length_95%HPD={1.39307600e-08,3.50038700e-04}])[&prob=1.00000000e+00,prob_stddev=0.00000000e+00,prob_range={1.00000000e+00,1.00000000e+00},prob(percent)="100",prob+-sd="100+-0"]:1.073873e-03[&length_mean=1.09622931e-03,length_median=1.07387300e-03,length_95%HPD={5.51858600e-04,1.69448700e-03}],(48[&prob=1.00000000e+00,prob_stddev=0.00000000e+00,prob_range={1.00000000e+00,1.00000000e+00},prob(percent)="100",prob+-sd="100+-0"]:3.231576e-03[&length_mean=3.24766067e-03,length_median=3.23157600e-03,length_95%HPD={2.29490300e-03,4.25212400e-03}],(219[&prob=1.00000000e+00,prob_stddev=0.00000000e+00,prob_range={1.00000000e+00,1.00000000e+00},prob(percent)="100",prob+-sd="100+-0"]:4.816491e-04[&length_mean=5.09661458e-04,length_median=4.81649100e-04,length_95%HPD={1.70229300e-04,8.93229800e-04}],220[&prob=1.00000000e+00,prob_stddev=0.00000000e+00,prob_range={1.00000000e+00,1.00000000e+00},prob(percent)="100",prob+-sd="100+-0"]:4.078905e-04[&length_mean=4.33815829e-04,length_median=4.07890500e-04,length_95%HPD={1.34895500e-04,7.96516600e-04}])[&prob=1.00000000e+00,prob_stddev=0.00000000e+00,prob_range={1.00000000e+00,1.00000000e+00},prob(percent)="100",prob+-sd="100+-0"]:2.270774e-03[&length_mean=2.29748069e-

03,length_median=2.27077400e-03,length_95%HPD={1.49397200e-03,3.19023000e-03}])[&prob=1.00000000e+00,prob_stddev=0.00000000e+00,prob_range={1.00000000e+00,1.00000000e+00},prob(percent)="100",prob+-sd="100+-0"]:9.248315e-04[&length_mean=9.41589219e-04,length_median=9.24831500e-04,length_95%HPD={4.19445400e-04,1.47574400e-03}])[&prob=1.00000000e+00,prob_stddev=0.00000000e+00,prob_range={1.00000000e+00,1.00000000e+00},prob(percent)="100",prob+-sd="100+-0"]:2.684545e-03[&length_mean=2.71480835e-03,length_median=2.68454500e-03,length_95%HPD={1.58737200e-03,3.80867100e-03}],22[&prob=1.00000000e+00,prob_stddev=0.00000000e+00,prob_range={1.00000000e+00,1.00000000e+00},prob(percent)="100",prob+-sd="100+-0"]:6.275662e-03[&length_mean=6.31479544e-03,length_median=6.27566200e-03,length_95%HPD={4.90827100e-03,7.84027200e-03}])[&prob=1.00000000e+00,prob_stddev=0.00000000e+00,prob_range={1.00000000e+00,1.00000000e+00},prob(percent)="100",prob+-sd="100+-0"]:1.346639e-02[&length_mean=1.35251908e-02,length_median=1.34663900e-02,length_95%HPD={1.10332800e-02,1.61121700e-02}],140[&prob=1.00000000e+00,prob_stddev=0.00000000e+00,prob_range={1.00000000e+00,1.00000000e+00},prob(percent)="100",prob+-sd="100+-0"]:2.144116e-02[&length_mean=2.15006696e-02,length_median=2.14411600e-02,length_95%HPD={1.85378500e-02,2.46279100e-02}])[&prob=1.00000000e+00,prob_stddev=0.00000000e+00,prob_range={1.00000000e+00,1.00000000e+00},prob(percent)="100",prob+-sd="100+-0"]:1.131533e-02[&length_mean=1.13414334e-02,length_median=1.13153300e-02,length_95%HPD={8.76921400e-03,1.38406300e-02}],(((44[&prob=1.00000000e+00,prob_stddev=0.00000000e+00,prob_range={1.00000000e+00,1.00000000e+00},prob(percent)="100",prob+-sd="100+-0"]:3.250221e-03[&length_mean=3.26600970e-03,length_median=3.25022100e-03,length_95%HPD={2.28179400e-03,4.21744100e-03}],((64[&prob=1.00000000e+00,prob_stddev=0.00000000e+00,prob_range={1.00000000e+00,1.00000000e+00},prob(percent)="100",prob+-sd="100+-0"]:1.283513e-03[&length_mean=1.31090140e-03,length_median=1.28351300e-03,length_95%HPD={7.32105500e-04,1.93539500e-03}],(((163[&prob=1.00000000e+00,prob_stddev=0.00000000e+00,prob_range={1.00000000e+00,1.00000000e+00},prob(percent)="100",prob+-sd="100+-0"]:5.534460e-04[&length_mean=5.75973029e-04,length_median=5.53446000e-04,length_95%HPD={1.96927400e-04,9.75657000e-04}],164[&prob=1.00000000e+00,prob_stddev=0.00000000e+00,prob_range={1.00000000e+00,1.00000000e+00},prob(percent)="100",prob+-sd="100+-0"]:8.480291e-04[&length_mean=8.73432857e-04,length_median=8.48029100e-04,length_95%HPD={4.20456600e-04,1.37165100e-03}])[&prob=1.00000000e+00,prob_stddev=0.00000000e+00,prob_range={1.00000000e+00,1.00000000e+00},prob(percent)="100",prob+-sd="100+-0"]:4.820825e-04[&length_mean=5.03618016e-04,length_median=4.82082500e-04,length_95%HPD={1.62305200e-04,8.69391900e-04}],182[&prob=1.00000000e+00,prob_stddev=0.00000000e+00,prob_range={1.00000000e+00,1.00000000e+00},prob(percent)="100",prob+-sd="100+-0"]:3.376518e-04[&length_mean=3.60477821e-04,length_median=3.37651800e-04,length_95%HPD={8.93577500e-05,6.74361300e-04}])[&prob=1.86108519e-01,prob_stddev=1.50829336e-03,prob_range={1.85041994e-01,1.87175043e-01},prob(percent)="19",prob+-sd="19+-0"]:4.992250e-05[&length_mean=7.20819864e-05,length_median=4.99225000e-05,length_95%HPD={3.10434800e-08,2.15207000e-04}],(183[&prob=1.00000000e+00,prob_stddev=0.00000000e+00,prob_range={1.00000000e+00,1.00000000e+00},prob(percent)="100",prob+-sd="100+-0"]:7.932685e-04[&length_mean=8.18707267e-04,length_median=7.93268500e-04,length_95%HPD={3.79090600e-04,1.33380100e-03}],184[&prob=1.00000000e+00,prob_stddev=0.00000000e+00,prob_range={1.00000000e+00,1.00000000e+00},prob(percent)="100",prob+-sd="100+-0"]:7.130114e-04[&length_mean=7.40361763e-04,length_median=7.13011400e-04,length_95%HPD={3.10403400e-04,1.20030100e-03}])[&prob=7.49966671e-01,prob_stddev=1.02752485e-02,prob_range={7.42700973e-01,7.57232369e-01},prob(percent)="75",prob+-sd="75+-1"]:1.175457e-04[&length_mean=1.41875148e-04,length_median=1.17545700e-04,length_95%HPD={5.93630600e-07,3.41664800e-04}])[&prob=1.88108252e-01,prob_stddev=1.31975669e-03,prob_range={1.87175043e-01,1.89041461e-01},prob(percent)="19",prob+-sd="19+-0"]:4.984844e-05[&length_mean=7.39697154e-05,length_median=4.98484400e-05,length_95%HPD={7.16840300e-08,2.19623000e-04}])[&prob=1.00000000e+00,prob_stddev=0.00000000e+00,prob_range={1.00000000e+00,1.00000000e+00},prob(percent)="100",prob+-sd="100+-0"]:2.133115e-03[&length_mean=2.15030080e-03,length_median=2.13311500e-03,length_95%HPD={1.41296800e-03,2.95187200e-03}],(161[&prob=1.00000000e+00,prob_stddev=0.00000000e+00,prob_range={1.00000000e+00,1.00000000e+00},prob(percent)="100",prob+-sd="100+-0"]:1.583721e-03[&length_mean=1.60785503e-03,length_median=1.58372100e-03,length_95%HPD={9.14534100e-04,2.28087700e-03}],162[&prob=1.00000000e+00,prob_stddev=0.00000000e+00,prob_range={1.00000000e+00,1.00000000e+00},prob(percent)="100",prob+-sd="100+-0"]:2.129097e-03[&length_mean=2.15344129e-

03,length_median=2.12909700e-03,length_95%HPD={1.40248300e-03,2.98188800e-03}])[&prob=1.00000000e+00,prob_stddev=0.00000000e+00,prob_range={1.00000000e+00,1.00000000e+00},prob(percent)="100",prob+-sd="100+-0"]:9.031748e-04[&length_mean=9.22032781e-04,length_median=9.03174800e-04,length_95%HPD={4.25248000e-04,1.46261600e-03}])[&prob=9.89734702e-01,prob_stddev=1.31975669e-03,prob_range={9.88801493e-01,9.90667911e-01},prob(percent)="99",prob+-sd="99+-0"]:3.429643e-04[&length_mean=3.69831269e-04,length_median=3.42964300e-04,length_95%HPD={5.95531400e-05,7.45698000e-04}])[&prob=1.00000000e+00,prob_stddev=0.00000000e+00,prob_range={1.00000000e+00,1.00000000e+00},prob(percent)="100",prob+-sd="100+-0"]:2.899917e-03[&length_mean=2.92116293e-03,length_median=2.89991700e-03,length_95%HPD={1.90500500e-03,3.93287400e-03}],47[&prob=1.00000000e+00,prob_stddev=0.00000000e+00,prob_range={1.00000000e+00,1.00000000e+00},prob(percent)="100",prob+-sd="100+-0"]:6.384375e-03[&length_mean=6.39713549e-03,length_median=6.38437500e-03,length_95%HPD={4.90783200e-03,7.83131100e-03}])[&prob=1.00000000e+00,prob_stddev=0.00000000e+00,prob_range={1.00000000e+00,1.00000000e+00},prob(percent)="100",prob+-sd="100+-0"]:2.012637e-03[&length_mean=2.03421719e-03,length_median=2.01263700e-03,length_95%HPD={1.05851700e-03,2.97763900e-03}],(((((75[&prob=1.00000000e+00,prob_stddev=0.00000000e+00,prob_range={1.00000000e+00,1.00000000e+00},prob(percent)="100",prob+-sd="100+-0"]:2.146767e-03[&length_mean=2.17392944e-03,length_median=2.14676700e-03,length_95%HPD={1.41747400e-03,2.97613600e-03}],87[&prob=1.00000000e+00,prob_stddev=0.00000000e+00,prob_range={1.00000000e+00,1.00000000e+00},prob(percent)="100",prob+-sd="100+-0"]:1.635099e-03[&length_mean=1.65498601e-03,length_median=1.63509900e-03,length_95%HPD={9.92331400e-04,2.37230400e-03}])[&prob=1.00000000e+00,prob_stddev=0.00000000e+00,prob_range={1.00000000e+00,1.00000000e+00},prob(percent)="100",prob+-sd="100+-0"]:1.662122e-03[&length_mean=1.68819663e-03,length_median=1.66212200e-03,length_95%HPD={1.04626700e-03,2.41564400e-03}],88[&prob=1.00000000e+00,prob_stddev=0.00000000e+00,prob_range={1.00000000e+00,1.00000000e+00},prob(percent)="100",prob+-sd="100+-0"]:2.466079e-03[&length_mean=2.49059418e-03,length_median=2.46607900e-03,length_95%HPD={1.66502500e-03,3.37064500e-03}])[&prob=8.34822024e-01,prob_stddev=2.45097671e-03,prob_range={8.33088921e-01,8.36555126e-01},prob(percent)="83",prob+-sd="83+-0"]:1.834508e-04[&length_mean=2.11172066e-04,length_median=1.83450800e-04,length_95%HPD={3.84145100e-07,4.83428700e-04}],141[&prob=1.00000000e+00,prob_stddev=0.00000000e+00,prob_range={1.00000000e+00,1.00000000e+00},prob(percent)="100",prob+-sd="100+-0"]:4.671839e-03[&length_mean=4.71920065e-03,length_median=4.67183900e-03,length_95%HPD={3.60199300e-03,5.89637600e-03}])[&prob=1.00000000e+00,prob_stddev=0.00000000e+00,prob_range={1.00000000e+00,1.00000000e+00},prob(percent)="100",prob+-sd="100+-0"]:1.348142e-03[&length_mean=1.37290484e-03,length_median=1.34814200e-03,length_95%HPD={7.82944500e-04,2.05379500e-03}],(116[&prob=1.00000000e+00,prob_stddev=0.00000000e+00,prob_range={1.00000000e+00,1.00000000e+00},prob(percent)="100",prob+-sd="100+-0"]:1.458020e-03[&length_mean=1.48453396e-03,length_median=1.45802000e-03,length_95%HPD={8.70097500e-04,2.15284400e-03}],117[&prob=1.00000000e+00,prob_stddev=0.00000000e+00,prob_range={1.00000000e+00,1.00000000e+00},prob(percent)="100",prob+-sd="100+-0"]:7.439017e-04[&length_mean=7.67595689e-04,length_median=7.43901700e-04,length_95%HPD={3.26894100e-04,1.24050500e-03}])[&prob=1.00000000e+00,prob_stddev=0.00000000e+00,prob_range={1.00000000e+00,1.00000000e+00},prob(percent)="100",prob+-sd="100+-0"]:4.173134e-03[&length_mean=4.19994205e-03,length_median=4.17313400e-03,length_95%HPD={3.12066500e-03,5.40154900e-03}])[&prob=1.00000000e+00,prob_stddev=0.00000000e+00,prob_range={1.00000000e+00,1.00000000e+00},prob(percent)="100",prob+-sd="100+-0"]:6.533469e-04[&length_mean=6.84334108e-04,length_median=6.53346900e-04,length_95%HPD={2.04426500e-04,1.17634900e-03}],(118[&prob=1.00000000e+00,prob_stddev=0.00000000e+00,prob_range={1.00000000e+00,1.00000000e+00},prob(percent)="100",prob+-sd="100+-0"]:5.045028e-03[&length_mean=5.06713432e-03,length_median=5.04502800e-03,length_95%HPD={3.85918900e-03,6.33414300e-03}],119[&prob=1.00000000e+00,prob_stddev=0.00000000e+00,prob_range={1.00000000e+00,1.00000000e+00},prob(percent)="100",prob+-sd="100+-0"]:4.502638e-03[&length_mean=4.53840868e-03,length_median=4.50263800e-03,length_95%HPD={3.39948400e-03,5.72007200e-03}])[&prob=9.99533396e-01,prob_stddev=2.82805005e-04,prob_range={9.99333422e-01,9.99733369e-01},prob(percent)="100",prob+-sd="100+-0"]:6.173325e-04[&length_mean=6.42887358e-04,length_median=6.17332500e-04,length_95%HPD={2.00018200e-04,1.14415900e-03}])[&prob=1.00000000e+00,prob_stddev=0.00000000e+00,prob_range={1.00000000e+00,1.00000000e+00},prob(percent)="100",prob+-sd="100+-0"]:4.701090e-03[&length_mean=4.73715801e-

03,length_median=4.70109000e-03,length_95%HPD={3.39743200e-03,6.06100100e-
03}])[&prob=1.00000000e+00,prob_stddev=0.00000000e+00,prob_range={1.00000000e+00,1.00000000e+00
},prob(percent)="100",prob+-sd="100+-0"]:9.775461e-03[&length_mean=9.79980518e-
03,length_median=9.77546100e-03,length_95%HPD={7.52838000e-03,1.21691700e-
02}])[&prob=1.00000000e+00,prob_stddev=0.00000000e+00,prob_range={1.00000000e+00,1.00000000e+00
},prob(percent)="100",prob+-sd="100+-0"]:2.111916e-02[&length_mean=2.11518196e-
02,length_median=2.11191600e-02,length_95%HPD={1.77209600e-02,2.48386600e-
02}])[&prob=9.97200373e-01,prob_stddev=1.88536670e-04,prob_range={9.97067058e-01,9.97333689e-
01},prob(percent)="100",prob+-sd="100+-0"]:3.363344e-03[&length_mean=3.44098062e-
03,length_median=3.36334400e-03,length_95%HPD={1.35798300e-03,5.77808200e-
03}])[&prob=1.00000000e+00,prob_stddev=0.00000000e+00,prob_range={1.00000000e+00,1.00000000e+00
},prob(percent)="100",prob+-sd="100+-0"]:1.236673e-02[&length_mean=1.24239344e-
02,length_median=1.23667300e-02,length_95%HPD={8.53391400e-03,1.65571400e-
02}],(((((((23[&prob=1.00000000e+00,prob_stddev=0.00000000e+00,prob_range={1.00000000e+00,1.00000
000e+00},prob(percent)="100",prob+-sd="100+-0"]:2.947596e-03[&length_mean=2.98906658e-
03,length_median=2.94759600e-03,length_95%HPD={1.91437700e-03,4.12902800e-
03}],(((((24[&prob=1.00000000e+00,prob_stddev=0.00000000e+00,prob_range={1.00000000e+00,1.0000000
0e+00},prob(percent)="100",prob+-sd="100+-0"]:1.376621e-03[&length_mean=1.39732196e-
03,length_median=1.37662100e-03,length_95%HPD={7.68078300e-04,2.02045300e-
03}],(27[&prob=1.00000000e+00,prob_stddev=0.00000000e+00,prob_range={1.00000000e+00,1.00000000e
+00},prob(percent)="100",prob+-sd="100+-0"]:7.007935e-04[&length_mean=7.25529358e-
04,length_median=7.00793500e-04,length_95%HPD={3.28593400e-04,1.22052300e-
03}],28[&prob=1.00000000e+00,prob_stddev=0.00000000e+00,prob_range={1.00000000e+00,1.00000000e+
00},prob(percent)="100",prob+-sd="100+-0"]:8.532934e-04[&length_mean=8.74654799e-
04,length_median=8.53293400e-04,length_95%HPD={4.02280000e-04,1.35884400e-
03}])[&prob=3.18157579e-01,prob_stddev=2.82805005e-04,prob_range={3.17957606e-01,3.18357552e-
01},prob(percent)="32",prob+-sd="32+-0"]:5.079391e-05[&length_mean=7.41456134e-
05,length_median=5.07939100e-05,length_95%HPD={6.10533800e-08,2.24668200e-
04}])[&prob=8.68217571e-01,prob_stddev=9.52110184e-03,prob_range={8.61485135e-01,8.74950007e-
01},prob(percent)="87",prob+-sd="87+-1"]:1.216222e-04[&length_mean=1.44317537e-
04,length_median=1.21622200e-04,length_95%HPD={1.24729300e-07,3.51227900e-
04}],25[&prob=1.00000000e+00,prob_stddev=0.00000000e+00,prob_range={1.00000000e+00,1.00000000e+
00},prob(percent)="100",prob+-sd="100+-0"]:7.215817e-04[&length_mean=7.46577952e-
04,length_median=7.21581700e-04,length_95%HPD={3.35101300e-04,1.23525500e-
03}])[&prob=1.00000000e+00,prob_stddev=0.00000000e+00,prob_range={1.00000000e+00,1.00000000e+00
},prob(percent)="100",prob+-sd="100+-0"]:4.879663e-04[&length_mean=5.12652964e-
04,length_median=4.87966300e-04,length_95%HPD={1.81713100e-04,9.19752100e-
04}],26[&prob=1.00000000e+00,prob_stddev=0.00000000e+00,prob_range={1.00000000e+00,1.00000000e+
00},prob(percent)="100",prob+-sd="100+-0"]:1.532055e-03[&length_mean=1.55699215e-
03,length_median=1.53205500e-03,length_95%HPD={9.24360800e-04,2.27135800e-
03}])[&prob=1.00000000e+00,prob_stddev=0.00000000e+00,prob_range={1.00000000e+00,1.00000000e+00
},prob(percent)="100",prob+-sd="100+-0"]:1.706978e-03[&length_mean=1.72990963e-
03,length_median=1.70697800e-03,length_95%HPD={1.02923600e-03,2.46006600e-
03}],29[&prob=1.00000000e+00,prob_stddev=0.00000000e+00,prob_range={1.00000000e+00,1.00000000e+
00},prob(percent)="100",prob+-sd="100+-0"]:2.330987e-03[&length_mean=2.35239869e-
03,length_median=2.33098700e-03,length_95%HPD={1.52723700e-03,3.18704900e-
03}])[&prob=1.00000000e+00,prob_stddev=0.00000000e+00,prob_range={1.00000000e+00,1.00000000e+00
},prob(percent)="100",prob+-sd="100+-0"]:5.614376e-04[&length_mean=5.84524088e-
04,length_median=5.61437600e-04,length_95%HPD={2.03957200e-04,1.03551500e-
03}],41[&prob=1.00000000e+00,prob_stddev=0.00000000e+00,prob_range={1.00000000e+00,1.00000000e+
00},prob(percent)="100",prob+-sd="100+-0"]:4.114474e-03[&length_mean=4.13264725e-
03,length_median=4.11447400e-03,length_95%HPD={3.07803100e-03,5.23577400e-
03}])[&prob=1.00000000e+00,prob_stddev=0.00000000e+00,prob_range={1.00000000e+00,1.00000000e+00
},prob(percent)="100",prob+-sd="100+-0"]:8.423322e-04[&length_mean=8.69387094e-
04,length_median=8.42332200e-04,length_95%HPD={3.74352600e-04,1.43414500e-
03}])[&prob=9.33275563e-01,prob_stddev=9.42683350e-05,prob_range={9.33208905e-01,9.33342221e-
01},prob(percent)="93",prob+-sd="93+-0"]:4.005432e-04[&length_mean=4.31295821e-
04,length_median=4.00543200e-04,length_95%HPD={6.84064000e-05,8.55374900e-
04}],95[&prob=1.00000000e+00,prob_stddev=0.00000000e+00,prob_range={1.00000000e+00,1.00000000e+
00},prob(percent)="100",prob+-sd="100+-0"]:6.176186e-03[&length_mean=6.20440840e-

03,length_median=6.17618600e-03,length_95%HPD={4.80994800e-03,7.64667000e-
03}])[&prob=1.00000000e+00,prob_stddev=0.00000000e+00,prob_range={1.00000000e+00,1.00000000e+00
},prob(percent)="100",prob+-sd="100+-0"]:1.429958e-03[&length_mean=1.46300718e-
03,length_median=1.42995800e-03,length_95%HPD={8.00704200e-04,2.17280400e-
03}],187[&prob=1.00000000e+00,prob_stddev=0.00000000e+00,prob_range={1.00000000e+00,1.00000000e
+00},prob(percent)="100",prob+-sd="100+-0"]:8.678302e-03[&length_mean=8.69424149e-
03,length_median=8.67830200e-03,length_95%HPD={7.00178500e-03,1.03467400e-
02}])[&prob=9.04679376e-01,prob_stddev=3.58219673e-03,prob_range={9.02146380e-01,9.07212372e-
01},prob(percent)="90",prob+-sd="90+-0"]:3.881205e-04[&length_mean=4.25123225e-
04,length_median=3.88120500e-04,length_95%HPD={2.44811300e-05,8.71424600e-
04}],(34[&prob=1.00000000e+00,prob_stddev=0.00000000e+00,prob_range={1.00000000e+00,1.00000000e
+00},prob(percent)="100",prob+-sd="100+-0"]:6.353257e-03[&length_mean=6.38209214e-
03,length_median=6.35325700e-03,length_95%HPD={5.03653300e-03,7.85858100e-
03}],94[&prob=1.00000000e+00,prob_stddev=0.00000000e+00,prob_range={1.00000000e+00,1.00000000e+
00},prob(percent)="100",prob+-sd="100+-0"]:6.113691e-03[&length_mean=6.12325734e-
03,length_median=6.11369100e-03,length_95%HPD={4.78001600e-03,7.50759400e-
03}])[&prob=9.98666844e-01,prob_stddev=3.77073340e-04,prob_range={9.98400213e-01,9.98933476e-
01},prob(percent)="100",prob+-sd="100+-0"]:9.875095e-04[&length_mean=1.01420038e-
03,length_median=9.87509500e-04,length_95%HPD={4.26056400e-04,1.69438200e-
03}])[&prob=1.00000000e+00,prob_stddev=0.00000000e+00,prob_range={1.00000000e+00,1.00000000e+00
},prob(percent)="100",prob+-sd="100+-0"]:1.355587e-02[&length_mean=1.35672903e-
02,length_median=1.35558700e-02,length_95%HPD={1.11042900e-02,1.58331500e-
02}],(192[&prob=1.00000000e+00,prob_stddev=0.00000000e+00,prob_range={1.00000000e+00,1.00000000
e+00},prob(percent)="100",prob+-sd="100+-0"]:1.192990e-04[&length_mean=1.45261806e-
04,length_median=1.19299000e-04,length_95%HPD={4.31224100e-07,3.59549400e-
04}],193[&prob=1.00000000e+00,prob_stddev=0.00000000e+00,prob_range={1.00000000e+00,1.00000000e
+00},prob(percent)="100",prob+-sd="100+-0"]:1.979562e-04[&length_mean=2.20959400e-
04,length_median=1.97956200e-04,length_95%HPD={8.22944400e-06,4.76723600e-
04}])[&prob=1.00000000e+00,prob_stddev=0.00000000e+00,prob_range={1.00000000e+00,1.00000000e+00
},prob(percent)="100",prob+-sd="100+-0"]:1.895467e-02[&length_mean=1.89700569e-
02,length_median=1.89546700e-02,length_95%HPD={1.55285600e-02,2.23357100e-
02}])[&prob=4.03746167e-01,prob_stddev=2.53581821e-02,prob_range={3.85815225e-01,4.21677110e-
01},prob(percent)="40",prob+-sd="40+-3"]:7.265459e-04[&length_mean=7.92450639e-
04,length_median=7.26545900e-04,length_95%HPD={6.02201300e-08,1.65618200e-
03}],(139[&prob=1.00000000e+00,prob_stddev=0.00000000e+00,prob_range={1.00000000e+00,1.00000000
e+00},prob(percent)="100",prob+-sd="100+-0"]:2.149787e-02[&length_mean=2.15440999e-
02,length_median=2.14978700e-02,length_95%HPD={1.86790100e-02,2.46480200e-
02}],((((214[&prob=1.00000000e+00,prob_stddev=0.00000000e+00,prob_range={1.00000000e+00,1.000000
00e+00},prob(percent)="100",prob+-sd="100+-0"]:1.149754e-03[&length_mean=1.18079721e-
03,length_median=1.14975400e-03,length_95%HPD={6.11908000e-04,1.80112200e-
03}],215[&prob=1.00000000e+00,prob_stddev=0.00000000e+00,prob_range={1.00000000e+00,1.00000000e
+00},prob(percent)="100",prob+-sd="100+-0"]:1.277423e-03[&length_mean=1.30455277e-
03,length_median=1.27742300e-03,length_95%HPD={7.11233500e-04,1.91236800e-
03}])[&prob=1.00000000e+00,prob_stddev=0.00000000e+00,prob_range={1.00000000e+00,1.00000000e+00
},prob(percent)="100",prob+-sd="100+-0"]:2.710060e-03[&length_mean=2.72417496e-
03,length_median=2.71006000e-03,length_95%HPD={1.79846300e-03,3.59544400e-
03}],217[&prob=1.00000000e+00,prob_stddev=0.00000000e+00,prob_range={1.00000000e+00,1.00000000e
+00},prob(percent)="100",prob+-sd="100+-0"]:2.059465e-03[&length_mean=2.08056781e-
03,length_median=2.05946500e-03,length_95%HPD={1.34565600e-03,2.90290800e-
03}])[&prob=1.00000000e+00,prob_stddev=0.00000000e+00,prob_range={1.00000000e+00,1.00000000e+00
},prob(percent)="100",prob+-sd="100+-0"]:1.198404e-03[&length_mean=1.22816270e-
03,length_median=1.19840400e-03,length_95%HPD={5.87342600e-04,1.88507500e-
03}],216[&prob=1.00000000e+00,prob_stddev=0.00000000e+00,prob_range={1.00000000e+00,1.00000000e
+00},prob(percent)="100",prob+-sd="100+-0"]:4.381816e-03[&length_mean=4.41544862e-
03,length_median=4.38181600e-03,length_95%HPD={3.28457700e-03,5.67457000e-
03}])[&prob=1.00000000e+00,prob_stddev=0.00000000e+00,prob_range={1.00000000e+00,1.00000000e+00
},prob(percent)="100",prob+-sd="100+-0"]:2.412662e-03[&length_mean=2.44862990e-
03,length_median=2.41266200e-03,length_95%HPD={1.46177600e-03,3.52136800e-
03}],218[&prob=1.00000000e+00,prob_stddev=0.00000000e+00,prob_range={1.00000000e+00,1.00000000e
+00},prob(percent)="100",prob+-sd="100+-0"]:6.127062e-03[&length_mean=6.16111865e-

03,length_median=6.12706200e-03,length_95%HPD={4.71510000e-03,7.73459300e-03}])[&prob=1.00000000e+00,prob_stddev=0.00000000e+00,prob_range={1.00000000e+00,1.00000000e+00},prob(percent)="100",prob+-sd="100+-0"]:1.295179e-02[&length_mean=1.30038556e-02,length_median=1.29517900e-02,length_95%HPD={1.06804300e-02,1.54069300e-02}])[&prob=9.98200240e-01,prob_stddev=1.79109837e-03,prob_range={9.96933742e-01,9.99466738e-01},prob(percent)="100",prob+-sd="100+-0"]:2.178575e-03[&length_mean=2.23145068e-03,length_median=2.17857500e-03,length_95%HPD={1.07587600e-03,3.48974400e-03}])[&prob=4.82602320e-01,prob_stddev=3.37480639e-02,prob_range={4.58738835e-01,5.06465805e-01},prob(percent)="48",prob+-sd="48+-3"]:1.718053e-03[&length_mean=1.79531560e-03,length_median=1.71805300e-03,length_95%HPD={1.43509800e-04,3.46890900e-03}],144[&prob=1.00000000e+00,prob_stddev=0.00000000e+00,prob_range={1.00000000e+00,1.00000000e+00},prob(percent)="100",prob+-sd="100+-0"]:1.325668e-02[&length_mean=1.32337491e-02,length_median=1.32566800e-02,length_95%HPD={1.00817400e-02,1.59819600e-02}])[&prob=1.00000000e+00,prob_stddev=0.00000000e+00,prob_range={1.00000000e+00,1.00000000e+00},prob(percent)="100",prob+-sd="100+-0"]:5.858885e-02[&length_mean=5.87188373e-02,length_median=5.85888500e-02,length_95%HPD={5.19559900e-02,6.51480400e-02}])[&prob=1.00000000e+00,prob_stddev=0.00000000e+00,prob_range={1.00000000e+00,1.00000000e+00},prob(percent)="100",prob+-sd="100+-0"]:1.216383e-02[&length_mean=1.22994295e-02,length_median=1.21638300e-02,length_95%HPD={7.59731500e-03,1.70670300e-02}])[&prob=1.00000000e+00,prob_stddev=0.00000000e+00,prob_range={1.00000000e+00,1.00000000e+00},prob(percent)="100",prob+-sd="100+-0"]:3.142149e-02[&length_mean=3.15315280e-02,length_median=3.14214900e-02,length_95%HPD={2.25854200e-02,4.04206400e-02}]);
end;
