# Supplementary material for: Evolutionary history of Carnivora (Mammalia, Laurasiatheria) inferred from mitochondrial genomes
Source: PLoS One. 2021 Feb 16;16(2):e0240770. doi: 10.1371/journal.pone.0240770 (PMC7886153; doi:10.1371/journal.pone.0240770)
Supplement: S6 Appendix — (PDF) [file pone.0240770.s006.pdf]

S6 Appendix. Bootstrap 50% majority-rule consensus tree reconstructed from the MRP matrix of the SuperTRI analysis

Evolutionary history of Carnivora (Mammalia, Laurasiatheria) inferred from mitochondrial genomes

Alexandre Hassanin[1*], Géraldine Veron[1], Anne Ropiquet[2], Bettine Jansen van Vuuren[3], Alexis Lécu[4], Steven M. Goodman[5], Jibran Haider[1,6,7], Trung Thanh Nguyen[1]

[1] Institut de Systématique, Évolution, Biodiversité (ISYEB), Sorbonne Université, MNHN, CNRS, EPHE, UA, Paris.

[2] Department of Natural Sciences, Faculty of Science and Technology, Middlesex University, United Kingdom.

[3] Centre for Ecological Genomics and Wildlife Conservation, Department of Zoology, University of Johannesburg, South Africa.

[4] Parc zoologique de Paris, Muséum national d'Histoire naturelle, Paris.

[5] Field Museum of Natural History, Chicago, IL, USA.

[6] Department of Wildlife Management, Pir Mehr Ali Shah, Arid Agriculture University Rawalpindi, Pakistan.

[7] Forest Parks & Wildlife Department Gilgit-Baltistan, Pakistan.

* Correspondence: alexandre.hassanin@mnhn.fr

Bootstrap method with heuristic search:
  Number of bootstrap replicates = 1000
  Starting seed = generated automatically
  Characters sampled proportionally to character weights
  Number of characters resampled in each replicate = 216618
  Optimality criterion = parsimony
    Character-status summary:
      Of 3398 total characters:
        All characters are of type 'unord'
        Character weights in effect:
          175 characters have weight 1
          3223 characters have weights other than 1
        All characters are parsimony-informative
  Starting tree(s) obtained via stepwise addition
    Addition sequence: simple (reference taxon = Tapirus terrestris T358)
    Number of trees held at each step = 1
  Branch-swapping algorithm: tree-bisection-reconnection (TBR) with reconnection limit = 8
    Steepest descent option not in effect
  Initial 'Maxtrees' setting = 100
  Branches collapsed (creating polytomies) if maximum branch length is zero
  'MulTrees' option in effect
  No topological constraints in effect
  Trees are unrooted

  1000 bootstrap replicates completed
  Time used = 57.62 sec (CPU time = 58.15 sec)

Bootstrap 50% majority-rule consensus tree
   (plus other groups compatible with this tree)

```
/------------------------------------------------------------- Tapirus terrestris T358(1)
|
|  /---------------------------------------------------------- Phataginus tricuspis NC026780(2)
|  |
|  |                                                    /----- Acinonyx jubatus NC005212 3(3)
|  |                                                    |
|  |                                                 100+ /--- Puma concolor NC016470 22(180)
|  |                                                 |  100
|  |                                                 |    \--- Puma yagouaroundi NC028311 1(181)
|  |                                              /90+
|  |                                              |  |    /--- Catopuma badia NC028300 1(31)
|  |                                              |  |  100
|  |                                              |  |  | \--- Catopuma temminckii NC027115 41(32)
|  |                                              |  \90+
|  |                                              |     \----- Pardofelis marmorata NLN3 2(160)
|  |                                    /---100---+
|  |                                    |         |       /--- Lynx canadensis NC028313 1(96)
|  |                                    |         |     58+
|  |                                    |         |     | \--- Lynx pardinus NC028319 161(98)
|  |                                    |         |  100+
|  |                                    |         100+  \----- Lynx lynx NC027083 4(97)
|  |                                    |            |
|  |                                    |            \-------- Lynx rufus NC014456 3(99)
|  |                                    |
|  |                                    |                 /--- Caracal caracal NC028306 1(30)
|  |                                 100+               100
|  |                                 |  |               | \--- Profelis aurata NC028299 1(178)
|  |                                 |  | /-----100-----+
|  |                                 |  | |             \----- Leptailurus serval NC028316 1(86)
|  |                                 |  | |
|  |                                 |  | |               /--- Felis catus NC001700 2(50)
|  |                                 |  | |             100
|  |                                 |  | |             | \--- Felis silvestris lybica KP202275(54)
|  |                                 |  | |          100+
|  |                                 |  | |       100+  \----- Felis margarita NC028308 1(52)
|  |                                 |  100       |  |
|  |                                 |    |     100  \-------- Felis nigripes NC028309 1(53)
|  |                                 |    |     | |
|  |                                 |    |  100+ \----------- Felis chaus NC028307 1(51)
|  |                                 |    |  |  |
|  |                                 |    |  |  \------------- Otocolobus manul NC028323 1(143)
|  |                                 |    |  |
|  |                                 |    |  |            /--- Prionailurus bengalensis CKM45 2(168)
|  |                              100+    |  |          100
|  |                              |  |    100+          | \--- Prionailurus viverrinus NC028305(173)
|  |                              |  |       |       100+
```

```
|  |                              |  |       |       |  \----- Prionailurus bengalensis NC02830(169)
|  |                              |  |       |    100+
|  |                              |  |       |    |  |    /--- Prionailurus planiceps KY682741 (170)
|  |                              |  |       \100-+  \100-+
|  |                              |  |            |       \--- Prionailurus planiceps NC028312 (171)
|  |                              |  |            |
|  |                              |  |            \----------- Prionailurus rubiginosus NC02830(172)
|  |                              |  |
|  |                              |  |                    /--- Leopardus colocolo NC028314 1(78)
|  |                              |  |               /100-+
|  |                              |  |               |    \--- Leopardus jacobita NC028322 1(81)
|  |                              |  |               |
|  |                              |  |            100+    /--- Leopardus pardalis NC028315 1(82)
|  |                              |  |            |  |  100
|  |                              |  |            |  |  | \--- Leopardus pardalis T262 1(83)
|  |                            100  |            |  100+
|  |                            | |  \----100-----+     \----- Leopardus wiedii NC028318 1(85)
|  |                            | |               |
|  |                            | |               |       /--- Leopardus geoffroyi NC028320 1(79)
|  |                            | |               |     100
|  |                            | |               |     | \--- Leopardus guigna NC028321 1(80)
|  |                            | |               \-100-+
|  |                            | |                     \----- Leopardus tigrinus NC028317 1(84)
|  |                            | |
|  |                            | |          /---------------- Neofelis nebulosa NC008450 3(136)
|  |                            | |          |
|  |                            | |          |            /--- Panthera leo NERO 19(146)
|  |                            | |          |          100
|  |                            | |          |          | \--- Panthera leo spelaea KX258452 2(147)
|  |                            | |          |       100+
|  |                            | |          |       |  | /--- Panthera pardus NC010641 5(150)
|  |                         100+ |          |       |  100
|  |                         |  | \---100----+    100+    \--- Panthera pardus japonensis KJ866(151)
|  |                         |  |            |    |  |
|  |                         |  |            |    |  |    /--- Panthera uncia KP202269 1(154)
|  |                         |  |            |    |  \100-+
|  |                         |  |            |  100       \--- Panthera uncia NC010638 1(155)
|  |                         |  |            |  | |
|  |                         |  |            |  | |       /--- Panthera onca KP202264 2(148)
|  |                         |  |            100+ \--100--+
|  |                         |  |               |         \--- Panthera onca NC022842 1(149)
|  |                       100  |               |
|  |                       | |  |               |         /--- Panthera tigris NC010642 35(152)
|  |                       | |  |               \---100---+
|  |                       | |  |                         \--- Panthera tigris amoyensis NC0147(153)
|  |                       | |  |
|  |                       | |  |                         /--- Homotherium latidens MF871702 3(73)
|  |                       | |  \-----------100-----------+
|  |                       | |                            \--- Smilodon populator MF871700 1(186)
|  |                       | |
|  |                       | |                            /--- Prionodon linsang ERR2391707 1(174)
|  |                       | \------------100-------------+
|  |                       |                              \--- Prionodon pardicolor NC024569 2(175)
|  |                       |
|  |                       |                      /----------- Arctictis binturong T605 2(8)
|  |                       |                      |
|  |                       |                    100  /-------- Paguma larvata PDDS11 2(145)
|  |                       |                    | |  |
|  |                       |                    | 100+    /--- Paradoxurus hermaphroditus NC039(156)
|  |                       |                    |    |  100
|  |                       |                    |    |  | \--- Paradoxurus hermaphroditus NLNC (157)
|  |                       |                    |    100+
|  |                       |                 100+       \----- Paradoxurus jerdoni MH464793 1(158)
|  |                       |                 |  |
|  |                       |                 |  |         /--- Chrotogale owstoni T607 1(33)
|  |                       |                 |  |       100
|  |                       |                 |  |       | \--- Hemigalus derbyanus MH464791 1(67)
|  |                       |                 |  |    100+
|  |                       |                 |  \100-+  \----- Diplogale hosei MH464790 1(45)
|  |                       |                 |       |
|  |                    100+                 |       \-------- Cynogale bennetti KY117544 1(43)
|  |                    | |                 |
|  |                    | |         /--100--+           /--- Civettictis civetta GLC19 1(35)
|  |                    | |         |       |         100
|  |                    | |         |       |         | \--- Civettictis civetta NC033378 1(36)
|  |                    | |         |       |      100+
|  |                    | |         |       |       |  \----- Viverra zibetha T609 1(210)
|  |                    | |         |       |       |
|  |                    | |         |       |    100+    /--- Viverricula indica KX891745 1(211)
\--+                    | |         |       |    |  |  100
   |                    | |         |       |    |  |  | \--- Viverricula indica KX891751 1(212)
   |                    | |         |       |    |  100+
   |                    | |         |       \100-+     \----- Viverricula indica NC025296 2(213)
   |                    | |         |            |
   |                    | |         |            |     /----- Genetta abyssinica MG489822 1(60)
   |                    | |         |            |     |
   |                    | |         |            \-100-+ /--- Genetta genetta T297 1(61)
   |                    | |         |                  100
   |                    | |         |                    \--- Genetta servalina NC024568 2(62)
   |                    | |         |
```

```
|                    |  |         |                    /--- Atilax paludinosus T606 1(18)
|                    |  |         |            /--100--+
|                    |  |         |            |       \--- Herpestes naso C07XAR110 1(71)
|                    |  |         |            |
|                    |  |         |          100     /----- Herpestes brachyurus KY117547 1(68)
|                    |  |         |          | |     |
|                    |  |         |          | |  100+ /--- Herpestes javanicus NC006835 1(69)
|                    |  |         |          | |  |  100
|                    |  |         |          | 100+    \--- Herpestes javanicus T413 1(70)
|                    |  \---100---+          |    |
|                    |            |       100+    \-------- Herpestes semitorquatus MH464789(72)
|                    |            |       |  |
|                    |            |       |  |         /--- Bdeogale nigripes GLC15 1(21)
|                    |            |       |  |       100
|                    |            |       |  |       | \--- Cynictis penicillata T375 1(42)
|                    |            |       |  |    100+
| /-------100--------+            |       |  \100-+  \----- Ichneumia albicauda T603 1(76)
| |                  |            |       |       |
| |                  |            |    100+       \-------- Galerella sanguinea T378 1(56)
| |                  |            |    |  |
| |                  |            |    |  |            /--- Crossarchus platycephalus C7AR66(39)
| |                  |            |    |  |          100
| |                  |            |    |  |          | \--- Helogale parvula SRR7637809 1(66)
| |                  |            |    |  |       100+
| |                  |            |    |  |       |  | /--- Mungos mungo MMC7 1(120)
| |                  |            |    |  |       |  100
| |                  |            |  100  \--100--+    \--- Mungos mungo SRR7704821 1(121)
| |                  |            |  | |          |
| |                  |            |  | |          \-------- Suricata suricatta SSM10 1(189)
| |                  |            |  | |
| |                  |            |  | |             /----- Cryptoprocta ferox CFC13 1(40)
| |                  |            |  | |             |
| |                  |            |  | |       /-100-+ /--- Eupleres goudotii D128 1(49)
| |                  |            |  | |       |     100
| |                  |            |  | |       |       \--- Fossa fossana D350 1(55)
| |                  |            |  | \--100--+
| |                  |            |  |         |  /-------- Galidia elegans D146 1(58)
| |                  |            |  |         |  |
| |                  |            100+         100+  /----- Galidictis fasciata DM333 1(59)
| |                  |               |            |  |
| |                  |               |            100+ /--- Mungotictis decemlineata NC02782(122)
| |                  |               |               100
| |                  |               |                 \--- Salanoia concolor D378 1(185)
| |                  |               |
| |                  |               |               /----- Crocuta crocuta NC020670 3(38)
| |                  |               |               |
| |                  |               |            100+ /--- Hyaena hyaena NC020669 1(74)
| |                  |               |            |  100
| |                  |               \----100-----+    \--- Parahyaena brunnea NC038159 15(159)
| |                  |                            |
| |                  |                            \-------- Proteles cristata T393 6(179)
| |                  |
| |                  \------------------------------------- Nandinia binotata NC024567 1(134)
| |
| |                            /--------------------------- Ailuropoda melanoleuca NC009492 (4)
| |                            |
| |                            |                     /----- Arctodus simus NC011116 1(15)
| |                            |                     |
| |                            |  /-------100--------+ /--- Arctotherium sp NC030174 1(17)
| |                            |  |                  100
| |                            |  |                    \--- Tremarctos ornatus NC009969 2(191)
| |                            |  |
| |                            |  |          /------------- Helarctos malayanus NC009968 2(65)
| |     /---------100----------+  |          |
| |     |                      |  |          | /----------- Ursus americanus JX196366 3(194)
| |     |                      |  |    /-100-+ |
| |     |                      |  |    |     | |       /--- Ursus thibetanus formosanus NC00(205)
| |     |                      |  |    |     80+     100
| |     |                      |  |    |       |     | \--- Ursus thibetanus thibetanus NC01(208)
| |     |                      |  |    |       |  /96+
| |     |                      |  |    |       100+  \----- Ursus thibetanus mupinensis NC00(207)
| |     |                      100+    |          |
| |     |                         |    |          \-------- Ursus thibetanus laniger MH28175(206)
| |     |                         |    |
| |     |                         |    |               /--- Ursus arctos AP012576 6(195)
| |     |                         |    |             100
| |     |                         |  100             | \--- Ursus arctos GU573491 207(198)
| |     |                         |  | |       /-100-+
| |     |                         |  | |       |     \----- Ursus arctos pruinosus MG066703 (200)
| |     |                         |  | |     100
| |     |                         |  | |     | |  /-------- Ursus arctos EU497665 29(196)
| |     |                         |  | |     | |  |
| |     |                         |  | |     | 100+  /----- Ursus arctos GU573486 5(197)
| |     |                         |  | |     |    |  |
| |     |                         |  | |  100+    100+ /--- Ursus maritimus GU573488 Svalbar(201)
| |     |                         100+ |  |  |       100
| |     |                            | |  |  |         \--- Ursus maritimus NC003428 31(202)
| |     |                            | 100+  |
| |     |                            |    |  \------------- Ursus arctos isabellinus 18B5 2(199)
| |     |                            |    |
```

```
| |     |                            |    |            /--- Ursus spelaeus EU327344 13(203)
| |     |                            |    \----100-----+
| |     |                            |                 \--- Ursus spelaeus NC011112 8(204)
| |     |                            |
| |     |                            \---------------------- Melursus ursinus NC009970 2(114)
| |     |
| |     |                                              /--- Ailurus fulgens NC011124 1(5)
| |     |                                         /100-+
| |     |                                         |    \--- Ailurus fulgens styani NC009691 (6)
| |     |    /----------------100-----------------+
| |     |    |                                    |  /----- Conepatus chinga NC042596 1(37)
| |     |    |                                    |  |
| |     |    |                                    100+ /--- Mephitis mephitis NC020648 1(115)
| |     |    |                                       100
| |     |    |                                         \--- Spilogale putorius NC010497 1(188)
| |     |    |
| |     |    |                                         /--- Aonyx cinerea NC035814 2(7)
| |     |    |                                    /100-+
| |     |    |                                    |    \--- Lutrogale perspicillata NC035811(93)
| |     |    |                                    |
| |     |    |                                 100+    /--- Lutra lutra LC050126 1(90)
| |     |    |                                 |  |  100
100     |    |                                 |  |  | \--- Lutra lutra NC011358 9(91)
  |     |    |                               100  100+
  |     |    |                               | |     \----- Lutra sumatrana NC035810 1(92)
  |     |    |                            100+ |
  |     |    |                            |  | \----------- Enhydra lutris NC009692 1(46)
  |     |    |                            |  |
  |     |    |                 /---100----+  \------------- Lontra canadensis SRR10409165 1(89)
  |     |    |                 |          |
  |     |    |                 |          |          /----- Galictis vittata T412 1(57)
  |     |    |                 |          |          |
  |     |    |                 |          \---100----+ /--- Ictonyx striatus T299 1(77)
  |     |    |                 |                     100
  |     |    |                 |                       \--- Poecilogale albinucha T602 1(166)
  |  100+    |                 |
  |  |  |    |                 |                       /--- Mustela altaica NC021751 1(123)
  |  |  |    |                 |             /---100---+
  |  |  |    |                 |             |         \--- Mustela nivalis T306 5(130)
  |  |  |    |                 |             |
  |  |  |    |                 |             |         /--- Mustela eversmannii NC028013 1(125)
  |  |  |    |                 |             |       100
  |  |  |    |               100          100+       | \--- Mustela putorius NC020638 4(131)
  |  |  |    |               | |          |  |    100+
  |  |  |    |               | |          |  |    |  \----- Mustela nigripes NC024942 1(129)
  |  |  |    |               | |          |  | 100+
  |  |  |    |               | |          |  | |  |    /--- Mustela sibirica AP017394 11(132)
  |  |  |    |               | |       100+  100  \100-+
  |  |  |    |               | |       |  |    |       \--- Mustela sibirica NC020637 6(133)
  |  |  |    |               | |       |  |    |
  |  |  |    |               | |       |  |    \----------- Mustela itatsi NC034330 19(127)
  |  |  |    |               | |     100  |
  |  |  |  100+               | |     | |  \---------------- Mustela erminea T305 2(124)
  |  |  | |  |            100+ |  100+ |
  |  |  | |  |            |  | |  |  | \------------------- Mustela kathiah NC023210 1(128)
  |  |  | |  |            |  | |  |  |
  |  |  | |  |            |  | 100+  \--------------------- Viverra tangalunga MH464792 1(209)
  |  |  | |  |            |  |    |
  |  |  | |  |            |  |    |                    /--- Mustela frenata NC020640 1(126)
  |  |  | |  |            |  |    \--------100---------+
  |  |  | |  |            |  |                         \--- Neovison vison NC020641 3(138)
  |  |  | |  |            |  |
  |  |  | |  |            |  |                       /----- Melogale moschata KP726273 1(111)
  |  |  | |  |            |  |                       |
  |  |  | |  |            |  \----------100----------+ /--- Melogale moschata NC020644 1(112)
  |  |  | |  |          77+                          100
  |  |  | |  |          | |                            \--- Melogale moschata V0735A 1(113)
  |  |  | |  |          | |
  |  |  | |  |          | |               /---------------- Gulo gulo NC009685 3(63)
  |  |  | |  |          | |               |
  |  |  | |  |          | |               |       /-------- Martes americana NC020642 1(100)
  |  |  | |  |          | |               |       |
  |  |  | |  |          | |               |    100+    /--- Martes martes T302 3(103)
  |  |  | |  |          | |            100+    |  |  100
  |  |  | |  |          | |            |  |    |  |  | \--- Martes zibellina NC011579 39(106)
  |  |  | |  |       100+ |            |  |  100  100+
  |  |  | |  |       |  | |            |  |  | |     \----- Martes melampus NC009678 1(104)
  |  |  | |  |       |  | \----100-----+  |  | |
  |  |  | |  |       |  |              |  100+ \----------- Martes foina NC020643 1(102)
  |  |  | |  |       |  |              |     |
  |  |  | |  |       |  |              |     \------------- Martes flavigula NC012141 3(101)
  |  |  | |  |    100+  |              |
  |  |  | |  |    |  |  |              \------------------- Martes pennanti NC020664 16(105)
  |  |  | |  |    |  |  |
  |  |  | |  |    |  |  \---------------------------------- Mellivora capensis T370 1(110)
  |  |  | |  |    |  |
  |  |  | |  |    |  |                            /-------- Arctonyx collaris NC020645 1(16)
  |  |  | |  |    |  |                            |
  |  |  | |  |  100  \------------100-------------+    /--- Meles anakuma NC009677 1(107)
  |  |  | |  |  | |                               |  100
```

```
|  |  | |  |  | |                               |  | \--- Meles leucurus NC039173 4(108)
|  |  | |  |  | |                               100+
|  |  | |  |  | |                                  \----- Meles meles T303 3(109)
|  |  | |  |  | |
|  |  | |  |  | \---------------------------------------- Taxidea taxus NC020646 1(190)
|  |  | |  100+
|  |  | |     |                                      /--- Bassaricyon neblina SRX1097850 1(19)
|  |  | |     |                                 /100-+
|  |  100     |                                 |    \--- Nasua nasua NC020647 1(135)
|  |    |     |                              100+
|  |    |     |                              |  |  /----- Bassariscus sumichrasti SRX10990(20)
|  |    |     |                              |  |  |
|  |    |     |                              |  100+ /--- Procyon lotor AB462046 3(176)
|  |    |     \-------------100--------------+     100
|  |    |                                    |       \--- Procyon lotor AB462049 4(177)
|  |    |                                    |
|  |    |                                    \----------- Potos flavus T414 1(167)
|  |    |
|  |    |                                            /--- Arctocephalus australis MG023139(9)
|  |    |                                          100
|  |    |                                          | \--- Arctocephalus forsteri KT693377 (10)
|  |    |                                       100+
|  |    |                                    100+  \----- Arctocephalus forsteri NC004023 (11)
|  |    |                                    |  |
|  |    |                                  100  \-------- Arctocephalus gazella BK010918 1(12)
|  |    |                                  | |
|  |    |                                  | \----------- Arctocephalus townsendi NC008420(14)
100+    |                               100+
   |    |                               |  |         /--- Neophoca cinerea NC008419 1(137)
   |    |                            100+  \---88----+
   |    |                            |  |            \--- Phocarctos hookeri NC008418 1(165)
   |    |                            |  |
   |    |                          100  \---------------- Otaria byronia OTAB 1(142)
   |    |                          | |
   |    |                          | \------------------- Arctocephalus pusillus NC008417 (13)
   |    |                       100+
   |    |                       |  |               /----- Eumetopias jubatus NC004030 10(48)
   |    |                       |  |               |
   |    |                    100+  \------100------+ /--- Zalophus californianus NC008416 (219)
   |    |                    |  |                  100
   |    |                    |  |                    \--- Zalophus wollebaeki SRR4431565 1(220)
   |    |                  100  |
   |    |                  | |  \------------------------ Callorhinus ursinus NC008415 1(22)
   |    |                  | |
   |    |                  | \--------------------------- Odobenus rosmarus NC004029 29(140)
   |    |                  |
   |    |                  |            /---------------- Cystophora cristata NC008427 1(44)
   |    |                  |            |
   |    |                  |            |            /--- Halichoerus grypus NC001602 2(64)
   |    |                  |            |          100
   |    |                  |            |          | \--- Pusa caspica NC008431 1(182)
   |    |                  |            |       /88+
   |    |                  |         100+    100+  \----- Pusa sibirica NC008432 2(184)
   |    |                  |         |  |    |  |
   |    \-------100--------+         |  |    |  \-------- Pusa hispida NC 008433 1(183)
   |                       |         |  |  100
   |                       |         |  |  | |       /--- Phoca largha NC008430 1(163)
   |                       |         |  |  | \--100--+
   |                       |       100  100+         \--- Phoca vitulina NC001325 1(164)
   |                       |       | |     |
   |                       |       | |     |         /--- Phoca fasciata NC008428 1(161)
   |                       |       | |     \---100---+
   |                       |       | |               \--- Phoca groenlandica NC008429 54(162)
   |                       |       | |
   |                       |       | \------------------- Erignathus barbatus NC008426 1(47)
   |                       |       |
   |                       |       |                 /--- Hydrurga leptonyx NC008425 1(75)
   |                       \--100--+               100
   |                               |               | \--- Leptonychotes weddellii NC008424(87)
   |                               |            /51+
   |                               |         100+  \----- Lobodon carcinophaga NC008423 1(88)
   |                               |         |  |
   |                               |         |  \-------- Ommatophoca rossii AY377287etc 1(141)
   |                               |       100
   |                               |       | |       /--- Mirounga angustirostris SRR10331(116)
   |                               |       | \--100--+
   |                               \--100--+         \--- Mirounga leonina NC008422 1(117)
   |                                       |
   |                                       |         /--- Monachus monachus NC044972 5(118)
   |                                       \---100---+
   |                                                 \--- Monachus schauinslandi NC008421 (119)
   |
   |                                 /------------------- Canis adustus KT448271 1(23)
   |                                 |
   |                                 |             /----- Canis anthus NC027956 2(24)
   |                                 |             |
   |                                 |          100+ /--- Canis lupus chanco NC010340 4(27)
   |                                 |          |  100
   |                               100       100+    \--- Canis lupus familiaris NC002008 (28)
   |                               | |       |  |
```

```
        |                               | |     100  \-------- Canis aureus KT448274 1(25)
        |                               | |     | |
        |                               | |  100+ \----------- Canis latrans NC008093 7(26)
        |                            100+ |  |  |
        |                            |  | 100+  \------------- Canis mesomelas KT448280 1(29)
        |                            |  |    |
        |                         100+  |    \---------------- Cuon alpinus NC013445 3(41)
        |                         |  |  |
        |                         |  |  \--------------------- Lycaon pictus NC028427 2(95)
        |                         |  |
        |                       100  \------------------------ Speothos venaticus C48 2(187)
        |                       | |
        |                       | |                       /--- Chrysocyon brachyurus NC024172 1(34)
        |                       | \----------100----------+
        |                       |                         \--- Lycalopex sechurae KT448284 1(94)
        |                       |
        |                       |               /------------- Nyctereutes procyonoides NC01370(139)
        |                       |               |
        |                       |               |         /--- Vulpes corsac NC023958 1(214)
        \----------100----------+               |       100
                                |            100+       | \--- Vulpes ferrilata NC027935 1(215)
                                |            |  |    100+
                                |            |  | 100+  \----- Vulpes vulpes NC008434 5(217)
                                |            |  | |  |
                                |         100+  100  \-------- Vulpes lagopus NC026529 3(216)
                                |         |  |    |
                                |         |  |    \----------- Vulpes zerda KJ603240 1(218)
                                |         |  |
                                \---100---+  \---------------- Otocyon megalotis SAF1 2(144)
                                          |
                                          |               /--- Urocyon cinereoargenteus NC02672(192)
                                          \------100------+
                                                          \--- Urocyon littoralis catalinae KP1(193)
```

Bipartitions found in one or more trees and frequency of occurrence (bootstrap support values):

```
                                                                                                   1
         1         2         3         4         5         6         7         8         9         0
1234567890123456789012345678901234567890123456789012345678901234567890123456789012345678901234567890
----------------------------------------------------------------------------------------------------
....................................................................................................
....................................................................................................
....................................................................................................
....................................................................................................
....................................................................................................
....................................................................................................
....................................................................................................
....................................................................................................
.................................*...........................................................*......
..........................**........................................................................
.......................*..**........................................................................
.......................**.**........................................................................
.......................*****........................................................................
.......................******.......................................................................
.......................******...........*...........................................................
......................*******...........*...........................................................
......................*******...........*.....................................................*.....
......................*******...........*.....................................................*.....
......................*******....*......*....................................................**.....
......................*******....*......*....................................................**.....
....................................................................................................
....................................................................................................
..........................................................................*...........*.............
..........................................................................*...........**............
..........................................................................*...........**............
..........................................................................*...........**............
....................................................................................................
....................................................................................................
...............................................................*....................................
...............................................................*....................................
...............................................................*....................................
...........................................*...................*....................................
...........................................*..*................*....................................
...........................................*..*................*..........*...........**............
....................................................................................................
...............................................*....................................................
........**..........................................................................................
........***.........................................................................................
........****........................................................................................
........****.*......................................................................................
........****.*......................................................................................
........****.*......................................................................................
........******......................................................................................
........******.................................*....................................................
........******.......*.........................*....................................................
........******.......*.........................*....................................................
........******.......*.....................*..**...............*..........*...........**............
....................................................................................................
...................*................................................................................
..................*.................................................................................
```

..................**..
..................**..
....
....
...............*..
....
....
....*
....*
....*
....*....................................*
....*....................................*
....
....
....
....
....
....
....
....
....
....
....
....
....
....
....*.......................
....*...................*.......................
....**.........
....***........
......*...*.......
......*..****.......
......*......................................*...****.......
......*......................................*..*****.......
......*......................................*..........*...................*...........*****.......
......*......................................*..........*...................*...........*****.......
......*......................................*..........*...................*...........*****.......
......*......................................*..........*.....*.............*...........*****......*
......*........*.............................*..........*.....*.............*...........*****......*
......*........*.............................*..........*.....*.............*...........*****......*
......*........*..**.........................*..........*.....*.............*...........*****......*
....
....................................*...
....**..
....**..............................*...
....***........*..**................*........*..........*.....*.............*...........*****......*
....***.******.*..**.*..............*......*.***........*.....**..........*.*.........*******......*
....
....
....
....
....
....
....
....
....
....
....
....*...................................
....*...................................
....*...................................
................*...
..............*.*...
..............*.*...*...................................
...*..........*.*...*...................................
...****.*********.**.*..............*......*.***........*.....***.........*.*.........*******......*
...****.*********.**.********....*..*...*..*.***........*.....***.........*.*.........*********....*
....*..........................
.....................................*...................................*..........................
.....................................*...................................*..........................
....
....*...
....**...
....*.....*...
....*........*.....*...
....*........*.....*..**...
....
......................................*..........................*..................................
......................................*..........................*..................................
......................................*..........................*..................................
....................*....................*..
....................*....................*.................................*........................
....................*....................*.............*...................*........................
....**..............................
....***..............................
....***.*............................
.................*..*.............................
.................*...*****............................
.................*..*....................*.............*...........*****...*........................
.................*..*.................*..*.............*.........*.*****...*........................

```
.................*..*.................**.*......*.....**.**......*.*****...*........................
.................*..*................***.*......*.....**.**......*.*****.*.*........................
............................................................**......................................
...........................................................***......................................
....................................................................................................
....................................................................................................
..................................**................................................................
..................................**................................................................
..................................**................................................................
..................................**.......................***......................................
................................*.................................*.................................
................................*...........*.....................*.................................
................................*.........*.*.....................*.................................
....................................................................................................
....................................................................................................
....................................................................................................
.......*............................................................................................
.......*........................*.........*.*.....................*.................................
.......*........................*.**......*.*..............***....*.................................
.......*.........*..*...........*.**.***.**.*...*.....**.*****...*******.*.*........................
....................................................................................................
........................................................................*...........................
....................................................................................................
....................................................................................................
....................................................................................................
....................................................................................................
....................................................................................................
....................................................................................................
....................................................................................................
....................................................................................................
....................................................................................................
....................................................................................................
..............................................................................**....................
..............................................................................**...*................
.................................................................................**.................
.................................................................................**.*...............
.............................................................................*..*...................
.............................................................................*..***.*...............
.............................................................................********...............
....................................................................................................
....................................................................................................
....................................................................................................
....................................................................................................
....................................................................................................
.................................................*...*..............................................
.................................................*.*.*..............................................
.................................................*.***..............................................
.................................................*****..............................................
.................................................*****..............................................
.................................................*****..............................................
.............................*......................................................................
.............................*.......................................................*..............
.............................*...................*****...............................*..............
...............................................................................................***..
...............................................................................................****.
..............................**....................................................................
....................................................................................................
..*.................................................................................................
..*...........................**...............................................................****.
..*..........................***.................*****...............................*.........****.
..*..........................***.................*****.......................*********.........****.
..*..........................***.................*****.......................*********.........****.
..*..........................***.................*****..................*....*********.........****.
..*..........................***.................*****..................*....*********.........****.
..*....*.........*..*........****.**.***.**.*...*********.*****..*********.*.*********.........****.
..*....*.........*..*........****.**.***.**.*...*********.*****..*********.*.*********.........****.
..**************************************************************************************************
...............................................................*....................................
....................................................................................................
..*...........................**....................................................................
..............................**....................................................................
...............................................................*....................................
....................................................................................................
....................................................................................................
......*......................................*..........*.....*.............*...........*****......*
...............................................................................................*.*..
..........................................................................*...........**............
..........................................................................*...........*.............
...............................................................................................**...
......*......................................*..........*...................*...........*****.......
................................................................*...................................
....................................................................................................
........****.*......................................................................................
..*...........................**....................................................................
...............................................................................................****.
....................................................................................................

1        1         1         1         1         1         1         1         1         1         2
0        1         2         3         4         5         6         7         8         9         0
1234567890123456789012345678901234567890123456789012345678901234567890123456789012345678901234567890
```

```
--------------------------------------------------
...........................................................................................**.......
....................................................................................................
....................................................................................................
....................................................................................................
....................................................................................................
......................................*.............................................................
......................................*....*........................................................
......................................*....*...............................................**.......
....................................................................................................
....................................................................................................
....................................................................................................
....................................................................................................
....................................................................................................
....................................................................................................
....................................................................................................
....................................................................................................
....................................................................................................
......................................................................................*.............
......................................................................................*.............
......................................*....*..........................................*....**.......
.................**.................................................................................
...............**...................................................................................
....................................................................................................
........................................*...........................................................
...............**.......................*...........................................................
...............****.....................*...........................................................
............................................................**......................................
..............................................................**....................................
.................................................................................*..................
..............................................................**.................***................
............................................................****.................***................
............................................................****.................***................
............................................................****.................***................
...............****.....................*...................****.................***................
....................................................................................................
....................................................................................................
....................................................................................................
....................................................................................................
....................................................................................................
....................................................................................................
....................................*...........................*...................................
....................................*....*......................*...................................
....................................*....*......................*...................................
....................................*....*......................*...................................
....................................*....*......................*...................................
....................................*..*.*......................*...................................
...............****.................*..***..................*****................***................
...........................................................................**.......................
...........................................................................**.......................
..................................*.................................................................
..................................*........................................**.......................
..................................*...............................*........**.......................
......**............................................................................................
......***...........................................................................................
......***...........................................................................................
..*..*..............................................................................................
..**.*..............................................................................................
..**.*..............................................................................................
.***.*..............................................................................................
****.*..............................................................................................
****.*..............................................................................................
******..............................................................................................
...........**.......................................................................................
..........***.......................................................................................
.........................*...........*..............................................................
...............................**...................................................................
........................*.....*.....................................................................
........................*...*.*.....................................................................
........................*...*.***...................................................................
........................*.*.*.***...................................................................
......................*......*......................................................................
......................*.*.*.*****...................................................................
......................***.*.*****...................................................................
......................***.*******...................................................................
......................***.*******...................................................................
......................***********....*..............................................................
.................................................................*..................................
.................................................................*..................................
....................................................................................................
....................................................................................................
....................................................................................................
....................................................................................................
....................................................................................................
....................................................................................................
.................................................................*..................................
......................***********....*...........................*..................................
..........***.........***********....*...........................*..................................
******...****.........***********....*...........................*..................................
*************.........***********....*...........................*..................................
```

```
*************.........***********....*...........................*.......................*..........
*************.........***********.*..*...........................**........**............*..........
..............*........................................................................*............
..............*........................................................................*............
....................................................................................................
..............*........................................................................*............
*************.*.......***********.*..*...........................**........**..........*.*..........
*************.*****...***********.*.**.***..................*******........**....***...*.*..........
....................................................................................................
....................................................................................................
................................................................................................*...
...............................................................................................**...
..............................................................................................*..*..
..............................................................................................*..*.*
..............................................................................................****.*
..............................................................................................******
..............................................................................................******
....................................................................................................
....................................................................................................
.............................................................................................*......
.............................................................................................*******
.............*...............................................................................*******
..........................................................................................*.........
..........................................................................................*.........
.............*............................................................................*..*******
.............*............................................................................*..*******
*******************...***********.*.**.***..................*******........**....***...*.**..*******
*******************...***********.*.******.*................*******........**....***..**.***********
..........................................................*.........................................
..........................................................*.........................................
..........................................................*...................*.....................
.....................*..............................................................*...............
.....................*..............................................................*...............
.....................*..............................................................*...............
....................................................................................................
....................................................................................................
.....................*..............................................................*...............
...................**...............................................................................
....................................................................................................
...................**...............................................................................
...................**...................................................................*...........
....................................................................................................
....................................................................................................
....................................................................................................
....................................................................................................
....................................................................................................
....................................................................................................
....................................................................................................
....................................................................................................
....................................................................................................
...................**...................................................................*...........
...................***..............................................................*...*...........
...................***....................................*...................*.....*...*...........
....................................................................................................
....................................................................................................
....................................................................................................
....................................................................................................
....................................................................................................
....................................................................................................
....................................................................................................
....................................................................................................
....................................................................................................
....................................................................................................
....................................................................................................
.......................................................**...........................................
.......................................................***..........................................
............................................*..........***..........................................
............................................*..........***..........................................
............................................*..........***..........................................
............................................*..........***..........................................
...................***......................*..........****...................*.....*...*...........
.........................................................................**.........................
.....................................................................................*..............
...................................................**...............................................
...............................................**...................................................
.....................................................**.............................................
.................................................**.................................................
.............................................**.....................................................
.............................................**..**.................................................
.............................................**..**..**.............................................
.............................................******..**.............................................
.............................................**********.............................................
...................................*.........**********.............................................
....................................................................................................
....................................................................................................
....................................................................................................
....................................................................................................
....................................................................................................
....................................................................................................
....................................................................................................
```

```
.....................................................................**.............................
...................................................................*....*...........................
...................................................................**...*...........................
...................................................................****.*...........................
...................................................................******...........................
....................................................................................................
....................................................................................................
....................................................................................................
....................................................................................................
..........................................*.........................................................
..........................................*........................******...........................
.............................................................................*......................
.............................................................................*......................
..........................................*........................******....*......................
....................................................................................................
....................................................................................................
....................................................................................................
...............................................................................**...................
...............................................................................**...................
...........................................................*...................**...................
..........................................*................*.......******....*.**...................
..........................................*................*.......******....*.**...................
...................................*......*..**********....*.......******....*.**...................
...................................*......*..**********....*.......******....*.**....*..............
...................................*......*..**********....*.......********..*.**....*..............
...................***.............*......*.****************.......********..****...**..*...........
...................***...........*.*......*.****************.......********..****...**..*...........
****************************************************************************************************
.................................................................................***................
....................................................................................................
...........................................................*...................**...................
...........................................................*........................................
.................................................................................*.*................
....................................*...........................*...................................
.............................................................................................*......
******....***.........***********....*...........................*..................................
....................................................................................................
....................................................................................................
........................................*...........................................................
....................................................................................................
.........****.........***********....*...........................*..................................
.............................................................................................*......
..................................................................................**................
................................................................*...................................
...............................................................................**...................
...........................................................*........................................
....................................................................................................
```

```
2        2         2
0        1         2
12345678901234567890           Freq      %
-------------------------------------------
....................       1000.00  100.00%
.............**.....       1000.00  100.00%
.............**.*...       1000.00  100.00%
.............****...       1000.00  100.00%
.............*****..       1000.00  100.00%
.............*****..       1000.00  100.00%
.............*****..       1000.00  100.00%
.............*****..       1000.00  100.00%
....................       1000.00  100.00%
....................       1000.00  100.00%
....................       1000.00  100.00%
....................       1000.00  100.00%
....................       1000.00  100.00%
....................       1000.00  100.00%
....................       1000.00  100.00%
....................       1000.00  100.00%
....................       1000.00  100.00%
....................       1000.00  100.00%
....................       1000.00  100.00%
.............*****..       1000.00  100.00%
....................       1000.00  100.00%
....................       1000.00  100.00%
....................       1000.00  100.00%
....................       1000.00  100.00%
....................       1000.00  100.00%
....................       1000.00  100.00%
....................       1000.00  100.00%
....................       1000.00  100.00%
....................       1000.00  100.00%
....................       1000.00  100.00%
....................       1000.00  100.00%
....................       1000.00  100.00%
....................       1000.00  100.00%
....................       1000.00  100.00%
..................**       1000.00  100.00%
..................**       1000.00  100.00%
....................       1000.00  100.00%
....................       1000.00  100.00%
```

```
....................      1000.00  100.00%
....................      1000.00  100.00%
....................      1000.00  100.00%
....................      1000.00  100.00%
....................      1000.00  100.00%
..................**      1000.00  100.00%
..................**      1000.00  100.00%
..................**      1000.00  100.00%
..................**      1000.00  100.00%
....................      1000.00  100.00%
....................      1000.00  100.00%
....................      1000.00  100.00%
....................      1000.00  100.00%
....................      1000.00  100.00%
....................      1000.00  100.00%
....................      1000.00  100.00%
....................      1000.00  100.00%
....................      1000.00  100.00%
....................      1000.00  100.00%
....................      1000.00  100.00%
....................      1000.00  100.00%
....................      1000.00  100.00%
....................      1000.00  100.00%
....................      1000.00  100.00%
....................      1000.00  100.00%
....................      1000.00  100.00%
....................      1000.00  100.00%
....................      1000.00  100.00%
....................      1000.00  100.00%
....................      1000.00  100.00%
....................      1000.00  100.00%
....................      1000.00  100.00%
....................      1000.00  100.00%
....................      1000.00  100.00%
....................      1000.00  100.00%
....................      1000.00  100.00%
........*...........      1000.00  100.00%
........*...........      1000.00  100.00%
....................      1000.00  100.00%
....................      1000.00  100.00%
....................      1000.00  100.00%
....................      1000.00  100.00%
....................      1000.00  100.00%
....................      1000.00  100.00%
....................      1000.00  100.00%
....................      1000.00  100.00%
....................      1000.00  100.00%
........*...........      1000.00  100.00%
........*...........      1000.00  100.00%
........*...........      1000.00  100.00%
........*...........      1000.00  100.00%
........*...........      1000.00  100.00%
........*...........      1000.00  100.00%
....................      1000.00  100.00%
....................      1000.00  100.00%
....................      1000.00  100.00%
....................      1000.00  100.00%
........*...........      1000.00  100.00%
........*.........**      1000.00  100.00%
..**................      1000.00  100.00%
**..................      1000.00  100.00%
**..................      1000.00  100.00%
**..................      1000.00  100.00%
....................      1000.00  100.00%
....................      1000.00  100.00%
**..................      1000.00  100.00%
**..................      1000.00  100.00%
****................      1000.00  100.00%
....*..*............      1000.00  100.00%
....****............      1000.00  100.00%
....****............      1000.00  100.00%
********............      1000.00  100.00%
********............      1000.00  100.00%
....................      1000.00  100.00%
....................      1000.00  100.00%
********............      1000.00  100.00%
********............      1000.00  100.00%
*********.........**      1000.00  100.00%
*********...********      1000.00  100.00%
....................      1000.00  100.00%
....................      1000.00  100.00%
....................      1000.00  100.00%
....................      1000.00  100.00%
....................      1000.00  100.00%
....................      1000.00  100.00%
....................      1000.00  100.00%
....................      1000.00  100.00%
....................      1000.00  100.00%
....................      1000.00  100.00%
....................      1000.00  100.00%
```

```
....................      1000.00  100.00%
....................      1000.00  100.00%
....................      1000.00  100.00%
....................      1000.00  100.00%
....................      1000.00  100.00%
....................      1000.00  100.00%
....................      1000.00  100.00%
....................      1000.00  100.00%
....................      1000.00  100.00%
....................      1000.00  100.00%
....................      1000.00  100.00%
....................      1000.00  100.00%
....................      1000.00  100.00%
....................      1000.00  100.00%
....................      1000.00  100.00%
....................      1000.00  100.00%
..........**........      1000.00  100.00%
..........***.......      1000.00  100.00%
....................      1000.00  100.00%
.........*..........      1000.00  100.00%
.........****.......      1000.00  100.00%
.........****.......      1000.00  100.00%
....................      1000.00  100.00%
....................      1000.00  100.00%
....................      1000.00  100.00%
....................      1000.00  100.00%
....................      1000.00  100.00%
....................      1000.00  100.00%
....................      1000.00  100.00%
....................      1000.00  100.00%
.........****.......      1000.00  100.00%
.........****.......      1000.00  100.00%
....................      1000.00  100.00%
....................      1000.00  100.00%
....................      1000.00  100.00%
....................      1000.00  100.00%
....................      1000.00  100.00%
....................      1000.00  100.00%
....................      1000.00  100.00%
....................      1000.00  100.00%
....................      1000.00  100.00%
....................      1000.00  100.00%
....................      1000.00  100.00%
....................      1000.00  100.00%
....................      1000.00  100.00%
....................      1000.00  100.00%
....................      1000.00  100.00%
....................      1000.00  100.00%
....................      1000.00  100.00%
....................      1000.00  100.00%
....................      1000.00  100.00%
....................      1000.00  100.00%
....................      1000.00  100.00%
....................      1000.00  100.00%
....................      1000.00  100.00%
....................      1000.00  100.00%
....................      1000.00  100.00%
....................      1000.00  100.00%
....................      1000.00  100.00%
....................      1000.00  100.00%
....................      1000.00  100.00%
....................      1000.00  100.00%
....................      1000.00  100.00%
....................      1000.00  100.00%
....................      1000.00  100.00%
....................      1000.00  100.00%
....................      1000.00  100.00%
....................      1000.00  100.00%
....................      1000.00  100.00%
....................      1000.00  100.00%
....................      1000.00  100.00%
....................      1000.00  100.00%
....................      1000.00  100.00%
....................      1000.00  100.00%
....................      1000.00  100.00%
....................      1000.00  100.00%
.........****.......      1000.00  100.00%
.........****.......      1000.00  100.00%
********************      1000.00  100.00%
....................       997.00   99.70%
....*.**............       960.50   96.05%
....................       901.50   90.15%
....................       901.00   90.10%
....................       877.00   87.70%
....................       877.00   87.70%
....****............       802.50   80.25%
........*...........       766.50   76.65%
....................       585.00   58.50%
....................       506.50   50.65%
....................       493.50   49.35%
```

```
....................        415.00   41.50%
........*...........        233.50   23.35%
....................        197.50   19.75%
....................        123.00   12.30%
....................        123.00   12.30%
....................         99.00    9.90%
....................         97.50    9.75%
....**.*............         39.50    3.95%

2 groups at (relative) frequency less than 1% not shown
```
