# Supplementary material for: Evolutionary history of Carnivora (Mammalia, Laurasiatheria) inferred from mitochondrial genomes
Source: PLoS One. 2021 Feb 16;16(2):e0240770. doi: 10.1371/journal.pone.0240770 (PMC7886153; doi:10.1371/journal.pone.0240770)
Supplement: S7 Appendix — (PDF) [file pone.0240770.s007.pdf]

S7 Appendix. Cytochrome b phylogeny of the genus *Melogale*

Evolutionary history of Carnivora (Mammalia, Laurasiatheria) inferred from mitochondrial genomes

Alexandre Hassanin[1*], Géraldine Veron[1], Anne Ropiquet[2], Bettine Jansen van Vuuren[3], Alexis Lécu[4], Steven M. Goodman[5], Jibran Haider[1,6,7], Trung Thanh Nguyen[1]

[1] Institut de Systématique, Évolution, Biodiversité (ISYEB), Sorbonne Université, MNHN, CNRS, EPHE, UA, Paris.

[2] Department of Natural Sciences, Faculty of Science and Technology, Middlesex University, United Kingdom.

[3] Centre for Ecological Genomics and Wildlife Conservation, Department of Zoology, University of Johannesburg, South Africa.

[4] Parc zoologique de Paris, Muséum national d'Histoire naturelle, Paris.

[5] Field Museum of Natural History, Chicago, IL, USA.

[6] Department of Wildlife Management, Pir Mehr Ali Shah, Arid Agriculture University Rawalpindi, Pakistan.

[7] Forest Parks & Wildlife Department Gilgit-Baltistan, Pakistan.

* Correspondence: alexandre.hassanin@mnhn.fr

Nexus CYB data set: DIMENSIONS NTAX=31 NCHAR=553
Method: neighbor-joining search
Number of bootstrap replicates = 1000

Bootstrap 50% majority-rule consensus tree

```
/------------------------------ Mustelafrenata NC 020640(1)
|
|                          /--- AF498158 Mmoschata(2)
|                      /60-+
|                      |   \--- MH986731 Mmoschata IPEERASAVA13 169(23)
|                   /81+
|                   |  \------- HM106328 Mmoschata YP6128(3)
|                /62+
|                |  \---------- MH986733 Mmoschata IPEERASSP1(24)
|      /---72----+
|      |         |         /--- Melogale moschata V0735A(6)
|      |         \---60----+
|      |                   \--- MH986732 Mmoschata IPEERASM10(25)
|      |
|      |                   /--- EF987756 Mpersonata(7)
|      |               /69-+
|      |               |   \--- MH986737 Mpersonata IPEE(19)
|      |         /-55--+
|      |         |     \------- MH986739 Mpersonata IPEE(18)
|      |         |
|      |         |         /--- MH986736 Mpersonata IPEE(15)
|      |     /72-+     /26-+
|      |     |   |     |   \--- MH986744 Mpersonata ZMMU S 161135(17)
|  /52-+     |   |  /52+
|  |   |     |   |  |  \------- MH986738 Mpersonata IPEE(16)
|  |   |     |   \66+
|  |   |     |      |      /--- MH986745 Mpersonata ZMMU S 72616(20)
|  |   |     |      \--76--+
|  |   |     |             \--- MH986746 Mpersonata ZMMU S 77190(21)
|  |   |     |
|  |   |  /73+             /--- MH986734 Mpersonata IPEE(8)
|  |   |  |  |         /18-+
|  |   |  |  |         |   \--- MH986741 Mpersonata IPEE(9)
|  |   |  |  |      /10+
|  |   |  |  |      |  \------- MH986742 Mpersonata IPEE(10)
100+   |  |  |   /64+
|  |   |  |  |   |  |      /--- MH986743 Mpersonata ZMMU S 137607(11)
|  |   100+  |   |  \--19--+
|  |      |  \82-+         \--- MH986748 Mpersonata IPEE(12)
|  |      |     |
|  |      |     |         /--- MH986735 Mpersonata IPEE(13)
|  |      |     \---54----+
|  |      |               \--- MH986740 Mpersonata IPEE(14)
|  |      |
|  |      \-------------------- MH986747 Mpersonata IPEE(22)
|  |
|  |                       /--- KP726273 Mmoschata subaurantiaca TESRIS3911(4)
|  \----------100----------+
|                          \--- MN400429 Mmoschata(5)
|
|                          /--- MH986726 Mcucphuongensis IPEERAS 2803131(26)
|                   /--68--+
|                   |      \--- MH986728 Mcucphuongensis IPEE(27)
|                   |
|                /91+      /--- MH986729 Mcucphuongensis IPEE(28)
|                |  |  /36-+
|                |  |  |   \--- MH986730 Mcucphuongensis IPEE(29)
\------100-------+  \63+
                 |     \------- MK167045 Mcucphuongensis 2018000079(31)
                 |
                 \------------- MH986727 Mcucphuongensis IPEE(30)
```
