# Supplementary material for: Evolutionary history of Carnivora (Mammalia, Laurasiatheria) inferred from mitochondrial genomes
Source: PLoS One. 2021 Feb 16;16(2):e0240770. doi: 10.1371/journal.pone.0240770 (PMC7886153; doi:10.1371/journal.pone.0240770)
Supplement: S8 Appendix — (PDF) [file pone.0240770.s008.pdf]

S8 Appendix. D-loop phylogeny of the species *Leopardus pardalis*

Evolutionary history of Carnivora (Mammalia, Laurasiatheria) inferred from mitochondrial genomes

Alexandre Hassanin[1*], Géraldine Veron[1], Anne Ropiquet[2], Bettine Jansen van Vuuren[3], Alexis Lécu[4], Steven M. Goodman[5], Jibran Haider[1,6,7], Trung Thanh Nguyen[1]

[1] Institut de Systématique, Évolution, Biodiversité (ISYEB), Sorbonne Université, MNHN, CNRS, EPHE, UA, Paris.

[2] Department of Natural Sciences, Faculty of Science and Technology, Middlesex University, United Kingdom.

[3] Centre for Ecological Genomics and Wildlife Conservation, Department of Zoology, University of Johannesburg, South Africa.

[4] Parc zoologique de Paris, Muséum national d'Histoire naturelle, Paris.

[5] Field Museum of Natural History, Chicago, IL, USA.

[6] Department of Wildlife Management, Pir Mehr Ali Shah, Arid Agriculture University Rawalpindi, Pakistan.

[7] Forest Parks & Wildlife Department Gilgit-Baltistan, Pakistan.

* Correspondence: alexandre.hassanin@mnhn.fr

Nexus Dloop data set: DIMENSIONS NTAX=53 NCHAR=403
Method: neighbor-joining search
Number of bootstrap replicates = 1000

Bootstrap 50% majority-rule consensus tree

```
/--------------------------------------------------------- Leopardus wiedii(1)
|
|                                /------------------------ AH010257.2(2)
|                                |
|                                |               /-------- AH010259.2(8)
|                                |               |
|                                |               +-------- AH010300.2(9)
|                                |               |
|                       /---57---+               +-------- AH010301.2(10)
|                       |        |       /--94---+
|                       |        |       |       +-------- AH010302.2(11)
|                       |        |       |       |
|                       |        |       |       +-------- AH010303.2(12)
|                       |        \--80---+       |
|                       |                |       \-------- AH010262.2(13)
|                       |                |
|                       |                \---------------- AH010260.2(38)
|                       |
|                       |                        /-------- AH010258.2(4)
|                       |                        |
|                       |                        +-------- AH010297.2(5)
|               /--62---+-----------84-----------+
|               |       |                        +-------- AH010298.2(6)
|               |       |                        |
|               |       |                        \-------- AH010299.2(7)
|               |       |
|               |       +--------------------------------- AH010261.2(19)
|               |       |
|               |       +--------------------------------- AH010263.2(20)
|               |       |
|               |       |                        /-------- AH010264.2(21)
|               |       |        /------96-------+
|               |       |        |               \-------- AH010304.2(22)
|               |       |        |
|               |       \---83---+               /-------- AH010265.2(23)
|               |                |       /--80---+
+------96-------+                |       |       \-------- AH010305.2(24)
|               |                \--58---+
|               |                        \---------------- AH010266.2(25)
|               |
|               |                                /-------- NC 028315(3)
|               |                /------99-------+
|               |                |               \-------- AH010269.2(14)
|               |                |
|               |                |               /-------- AH010271.2(15)
|               |                |               |
|               |                |               +-------- AH010306.2(16)
|               |                |       /--92---+
|               |                |       |       +-------- AH010307.2(17)
|               |                |       |       |
|               \-------65-------+--54---+       \-------- AH010308.2(18)
|                                |       |
|                                |       \---------------- AH010270.2(27)
|                                |
|                                +------------------------ AH010272.2(26)
|                                |
|                                +------------------------ AH010267.2(31)
|                                |
|                                \------------------------ AH010268.2(36)
|
|               /----------------------------------------- AH010275.2(28)
|               |
|               |                                /-------- AH010276.2(29)
```

```
|               |                        /--73---+
|               |                        |       \-------- AH010311.2(30)
|       /--63---+                        |
|       |       |                /--52---+---------------- AH010277.2(32)
|       |       |                |       |
|       |       |        /---65---+       \---------------- AH010279.2(33)
|       |       |        |        |
|       |       \--94---+        \------------------------ AH010278.2(34)
|       |               |
+--60---+               \--------------------------------- AH010280.2(35)
|       |
|       +------------------------------------------------- AH010273.2(46)
|       |
|       |                                        /-------- AH010274.2(49)
|       |                                        |
|       |                                        +-------- AH010309.2(51)
|       \------------------100-------------------+
|                                                +-------- AH010310.2(52)
|                                                |
|                                                \-------- pardalT262b(53)
|
+--------------------------------------------------------- AH010338.2(37)
|
|                                        /---------------- AH010283.2(39)
|                                        |
|                                        |       /-------- AH010281.2(40)
|                                        +--55---+
|                                        |       \-------- AH010282.2(41)
|                                /--96---+
|                                |       |       /-------- AH010286.2(42)
|                                |       +--82---+
|                                |       |       \-------- AH010312.2(43)
+---------------60---------------+       |
|                                |       \---------------- AH010284.2(44)
|                                |
|                                \------------------------ AH010285.2(45)
|
+--------------------------------------------------------- AH010287.2(47)
|
|                                                /-------- AH010296.2(48)
\-----------------------94-----------------------+
                                                 \-------- AH010295.2(50)
```
